# Additive CHARMM force field for naturally occurring modified ribonucleotides

**DOI:** 10.1002/jcc.24307

**Published:** 2016-02-03

**Authors:** You Xu, Kenno Vanommeslaeghe, Alexey Aleksandrov, Alexander D. MacKerell, Lennart Nilsson

**Affiliations:** ^1^ Department of Biosciences and NutritionKarolinska InstitutetHUDDINGESE‐141 83Sweden; ^2^ Department of Pharmaceutical SciencesSchool of Pharmacy, University of Maryland20 Penn StreetBaltimoreMaryland21201; ^3^ Department of Analytical Chemistry and Pharmaceutical Technology (FABI)Center for Pharmaceutical Research (CePhaR), Vrije Universiteit Brussel (VUB)Laarbeeklaan 103BrusselsB‐1090Belgium; ^4^ Department of BiologyEcole Polytechnique, Laboratoire De Biochimie (CNRS UMR7654)PalaiseauF‐91128France

**Keywords:** nucleic acids, RNA, transfer RNA, empirical force field, oligonucleotide, molecular dynamics

## Abstract

More than 100 naturally occurring modified nucleotides have been found in RNA molecules, in particular in tRNAs. We have determined molecular mechanics force field parameters compatible with the CHARMM36 all‐atom additive force field for all these modifications using the CHARMM force field parametrization strategy. Emphasis was placed on fine tuning of the partial atomic charges and torsion angle parameters. Quantum mechanics calculations on model compounds provided the initial set of target data, and extensive molecular dynamics simulations of nucleotides and oligonucleotides in aqueous solutions were used for further refinement against experimental data. The presented parameters will allow for computational studies of a wide range of RNAs containing modified nucleotides, including the ribosome and transfer RNAs. © 2016 The Authors. Journal of Computational Chemistry Published by Wiley Periodicals, Inc.

## Introduction

Post‐transcriptionally modified nucleotides are very common in ribonucleic acids (RNA), with approximately 25% of the nucleotides in eukaryotic tRNAs being modified. Of the 112 naturally occurring modified nucleotides that have been described,^1^, ^2^ more than 90% are found in transfer RNA (tRNA)^3^, with marked differences in the types of modifications occurring in archaea, prokaryotes and eukaryotes.^2^ These modifications, which are introduced in RNA by a variety of enzymes, come with a significant energetic and genetic cost to the organism—in bacteria four times as much genetic information is required for tRNA modifying enzymes as for the tRNAs themselves.^4^

RNA modifications have been implicated in the development of a number of human diseases, mostly related to energy metabolism (e.g. obesity, diabetes type 2, mitochondrial diseases) but also in various tumors and neurodegenerative diseases.^2^ The biological effects of these RNA modifications and the mechanisms by which they contribute to human disease are in general not known, except in a handful of cases such as the mitochondrial pathogeneses MELAS and MERRF,^5^ where disruption of the modification of uridine in the anticodon wobble position (first anticodon nucleotide) of tRNA^Lys^ and tRNA^Leu^ leads to reduced translation of the corresponding codons, and hence insufficient mitochondrial protein production.

Modifications add to the RNA structural repertoire by allowing specific interactions contributing to well‐defined three‐dimensional (3D) structures. Such structures often have special functions such as UV sensing and are involved in a range of biological phenomena ranging from regulation of cellular processes while sensing the cell's metabolic state^1^, ^2^ to the numerous interactions between tRNA and other partners in the translational machinery, such as synthetases, ribosomes, messenger RNA (mRNA), initiation and elongation factors.^1^, ^5^, ^6^, ^7^, ^8^ Some modifications are crucial to codon reading. For example, in tRNA a modified uridine in the wobble position 34 can match multiple bases, thus allowing one tRNA to read more than one codon, and the hypermodified purine commonly found in position 37, immediately 3′ to the anticodon, stabilizes the anticodon‐codon mini‐helix and helps maintain the reading frame.^1^, ^5^, ^7^, ^9^ Other modifications that are highly conserved in tRNA, like the dihydrouridine(s) (D) in positions 16 to 20, the 7‐methylguanosine (m^7^G) in position 46, and the 5‐methyluridine and pseudouridine (*Ψ*) in positions 54 and 55, are responsible for local structural folding and flexibility for an individual tRNA.^6^, ^10^, ^11^ The overall 3D structure of tRNA is as important as the anticodon triplet for ribosomal tRNA identification, as mutations far from the anticodon in tRNA^Trp^ cause stop‐codon read‐through.^12^, ^13^

Molecular simulation and modeling methods^2^, ^14^, ^15^ have been applied to yield a detailed view of the structural and energetic effects of modified nucleotides. These include studies of the anticodon stem loop (ASL) and codon‐anticodon interactions,^16^, ^17^, ^18^ and on the thermodynamic stability of tRNA structures.^19^ In order to make such studies more widely accessible, reliable force field parameters that describe the conformational energetics and interactions with the environment of the modified nucleotides are required. In classical molecular simulations an empirical force field is used to evaluate energies and forces in the system, and the reliability of the simulation results depends critically on the quality of the force field. Force fields have been developed for all major components of biomolecules: proteins,^20^, ^21^, ^22^, ^23^, ^24^, ^25^ nucleic acids,^21^, ^25^, ^26^, ^27^, ^28^ carbohydrates,^25^, ^29^, ^30^, ^31^ lipids,^25^, ^32^, ^33^, ^34^, ^35^ and small organic molecules.^25^, ^36^, ^37^, ^38^ Typically these force fields contain descriptions of the standard building blocks, and the RNA modifications in general have not been included. Several years ago, an AMBER‐compatible force field for 107 modified nucleotides was released.^39^ This first systematic parametrization effort for RNA modifications focused on the atomic partial charges, and relied on analogy with similar functional groups in the AMBER force field for all other parameters.

In the present work we develop CHARMM compatible force field parameters of similar quality as those for the standard nucleotides, including partial atomic charges and bonded parameters, with special attention to the glycosidic torsions, for 112 naturally occurring modified nucleotides. The parametrization approach uses the same methodology as for the additive CHARMM36 Nucleic Acid Force Field (NA36),^26^, ^40^ and the CHARMM General Force Field (CGenFF).^36^, ^41^ For several of the modifications we have also developed parameters for different tautomers and protonation states. In the parameter optimization we initially targeted *ab initio* quantum mechanical (QM) data, and then compared results from MD simulations using the initial parameters to experimental data for nucleosides, and oligonucleotides for further refinement and validation; it should be noted that in comparison to the common nucleotides (A, C, G, U), there is much less experimental data available for validation of the large number of RNA modifications.

## Methods

### CHARMM potential energy function

The potential energy function used in the CHARMM force field is based on the following equation:

$$U=\sum_{nonbond}\frac{q_{i}q_{j}}{4\pi {\varepsilon \varepsilon }_{0}r_{ij}}+\sum_{nonbond}\varepsilon_{ij}\left[{\left(\frac{R_{ij}^{min}}{r_{ij}}\right)}^{12}-2{\left(\frac{R_{ij}^{min}}{r_{ij}}\right)}^{6}\right]+\sum_{bonds}{K_{b}\left(b-b_{0}\right)}^{2}+\sum_{angle}K_{\theta }{\left(\theta -\theta_{0}\right)}^{2}+\sum_{UB}K_{UB}{\left(r_{1,3}-r_{1,3;0}\right)}^{2}+\sum_{improper}K_{\varphi }{\left(\varphi -\varphi_{0}\right)}^{2}+\sum_{dihedrals}K_{\emptyset }\left(1+cos\left(n\emptyset -\delta \right)\right)$$

The first two sums, the so‐called non‐bonded terms, represent electrostatic and van der Waals (vdW) interactions. Here *q_i_* and *q_j_* are partial charges of two particles separated by distance *r_ij_*, *ɛ_0_* is the vacuum permittivity, and *ε* is the relative permittivity, generally assigned a value of 1 for explicit solvent simulations. The vdW energy is described by a Lennard‐Jones potential (LJ term) where $R_{ij}^{min}$ is the distance between the two particles at which the potential reaches its minimum, and *ɛ_ij_* is the depth of the LJ potential well. The remaining sums are the bonded terms that represent bonds, valence angles and dihedral or torsion angles. For proteins, an extra 2D dihedral energy correction map^23^ is also included for the backbone phi, psi torsion angles. The bonds, angles, Urey‐Bradley interactions (UB) and improper dihedrals are described by harmonic expressions, where *K_b_*, *K_θ_*, *K*_UB_ and *K_φ_* are force constants and *b*_0_, *θ*_0_, *r*_1,3;0_, and *φ*_0_ are equilibrium values. The dihedral angles are described by cosine functions, where *K_Φ_*, *n* and *δ* are the amplitude, periodicity and phase angle, respectively. In principle, *n* can be any integer and *δ* any value between 0° and 360°, but in the current CHARMM force field, *n* is only taken to be 1, 2, 3, 4 and 6, and *δ* is either 0° or 180°. In addition, a given torsion angle may be treated as a Fourier series over a sum over 2 or more periodicities.

### Parametrization Scheme

**Figure 1:**
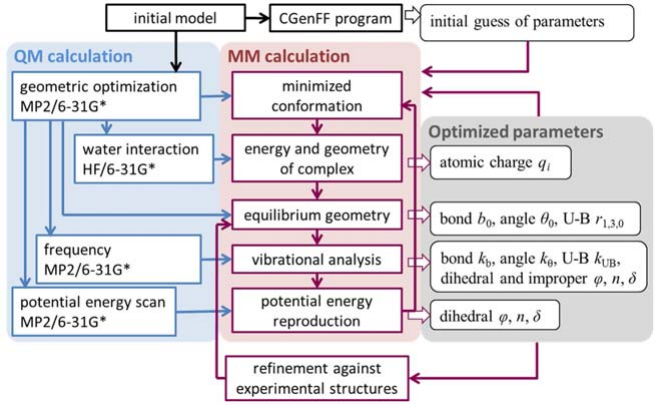
Flow diagram of the parametrization procedure.

The atom types were mainly taken from CGenFF^36^ for the base atoms and from NA36^26^, ^40^ for the ribose and phosphate. Additionally, atom types from the CHARMM carbohydrate force field (Carb36)^29^ were used for hexoses. Since most modifications are on the base, the parametrization strategy closely followed that of CGenFF, in which the use of L‐J parameters transferred from the remainder of the additive force field has been verified,^36^ such that they were not optimized in the present study. The full parametrization protocol is shown in a flow chart in Figure 1.

In brief, initial guesses for the parameters of representative model compounds (see “Model compounds” below) were generated using the CGenFF program,^42^, ^43^ via the ParamChem online server (https://cgenff.paramchem.org) that performs atom typing and assigns parameters and charges to new compounds based on analogy.^42^, ^43^ The resulting CGenFF atomic charges, equilibrium geometries, harmonic force constants and dihedral terms were subsequently optimized. For each novel model compound QM target data were first generated and then the relevant force field parameters were modified iteratively until convergence was reached between QM and the molecular mechanics (MM) data. When experimental data were available, they were used as additional target data to optimize the parameters as needed to get more accurate simulation results with respect to condensed phase properties.

#### Charge optimization

Partial atomic charges were optimized targeting interactions between the model compound and individual water molecules, as well as the dipole moment of the model compound. The peripheral atoms in the QM minimized model compound were probed by individual water molecules in idealized linear orientations. Only monohydrates were studied and we used several different rotations of the water molecule around the interaction axis: for polar atoms, the complex was calculated every 60° of water probe rotation, and for nonpolar atoms, every 90° or 180°. The model compound‐water interaction distances were then optimized and the interaction energy calculated. Before comparing with the MM calculations the QM HF/6‐31G(d) interaction energy was multiplied by 1.16 for neutral polar compounds and the minimum interaction distance was offset by −0.2 Å for all polar interactions,^44^, ^45^ consistent with the optimization of the remainder of the CHARMM additive force field.^20^, ^26^, ^29^, ^36^ In the case of sulfur atoms, the thiocarbonyl compound interactions were calculated at the MP2/6‐31G* level without applying energy scaling. The partial atomic charge optimization targeted the root mean square deviation (RMSD) of the MM from the (scaled) QM interaction energies over all water probes, and the deviation of the magnitude and direction of MM dipole moment from QM HF/6‐31G(d) values. All other details were identical to the CGenFF parametrization protocol.^36^

The charge optimization was performed with a C++ program based on the Powell and Amoeba minimization algorithms from Numerical Recipes.^46^ The following weighted terms were included in the target function: the RMS deviation between empirical and *ab initio* minimum interaction energies, the RMS deviation between *ab initio* and empirical minimum interaction distances, the absolute difference between the norms of the empirical and *ab initio* dipole moments, the angle between the empirical and *ab initio* dipole moments, and a term associated with restraints on the charges. The latter term was specifically introduced to prevent large deviations from the starting guess for charges. The dipole moments were calculated at the HF/6‐31G(d) level, and were not scaled in the charge optimization. Charges of symmetrical atoms were constrained during optimization to have identical values. Charges of aliphatic groups were not optimized, in accord with the standard CHARMM method. For example, all methyl groups have a charge of 0.09 |e| on protons. We also reiterate that the LJ parameters were not subjected to optimization.

The molecular dipole moment, which is determined by the charge distribution, was used to provide additional target data for optimization of the atomic charges. Only the neutral compounds were considered here; for charged molecules, the dipole moment is ill‐defined, and the net charge, which is the lowest non‐zero electric moment, is the leading contribution to electrostatic interaction instead of the dipole moment. The dipole moment refers to the permanent moment in vacuum at the HF/6‐31G(d) level of the QM MP2 optimized conformation. Since in additive force fields the molecular polarizability is not explicitly taken into account, to reproduce the electronic distribution in aqueous solution the MM estimated dipole moment is typically overestimated by 20% to 50% with respect to the QM values for small polar compounds.^36^ However, in this study the HF/6‐31G(d) values were directly targeted as this level of theory typically overestimates the experimental gas phase dipoles and the restrained optimization of the charges was dominated by the interactions with water.

#### Determination of bonded harmonic energy terms

Parameters for the bonded terms described by harmonic potentials (i.e. bonds, angles, Urey‐Bradley, and improper dihedrals), as well as for the nonrotatable dihedrals in aromatic rings (e.g. stiff dihedrals, see below), were determined as follows. The equilibrium values of the MM parameters were adjusted until the RMSD between the MM and the optimized QM geometries could not be reduced significantly. The force constants were determined by calculating the molecular vibrational frequencies; the contributions of the internal coordinates to the vibrations of the model compound were defined by potential energy distribution (PED) analysis using the internal valence coordinate system.^47^ The QM frequencies were scaled by 0.943^48^ before comparison with the MM calculated PED using MOLVIB^49^ in CHARMM. After the force constants were optimized, the molecular geometry was re‐evaluated using the new parameters, and the equilibrium parameters fine‐tuned once again to assure that the MM geometries accurately reproduce the QM results.

#### Flexible dihedral optimization

Dihedrals can be considered in two classes. Stiff dihedrals, such as torsions about aromatic or conjugated bonds, were optimized based on vibrational analysis. The other class includes low‐energy barrier rotatable dihedrals that may undergo large fluctuations during simulations. The treatment of this class of dihedral is crucial to the quality of a force field in reproducing conformational properties. Potential energy surface (PES) scans were performed on these torsions, in which acyclic torsion angles were scanned over 0°– 360° in 5° increments, and ring torsions involving sp^3^ carbons were scanned over an interval of ±(60°–90°) around the energy minimum in 3° increments. Each conformation for the MM calculations was extracted from the QM scan and minimized with a harmonic restraint force constant of 10^4^ kcal/mol/radian^2^ on the target torsion. The MM dihedral parameters were optimized to achieve a minimum deviation between the QM and MM surfaces in the lower energy regions (<12 kcal/mol above the minimum energy). This dihedral optimization is sensitive to all the other parameters, thus when any atomic charge or bonded term was modified during subsequent testing, the related dihedral parameters were reevaluated.

**Figure 2:**
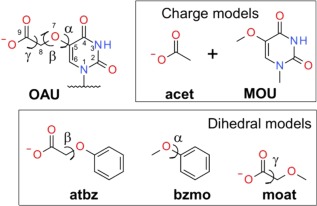
The model compounds for base OAU (5‐carboxymethoxy uracil). The charge models include acetate (acet), which was available in CGenFF, and 5‐methoxyuracil (MOU) that had been optimized prior to OAU as part of the present study. The parameters for three dihedral angles were determined from three simpler model compounds, atbz, bzmo and moat. The bond, angle and improper torsion parameters were mostly taken from acet and MOU, except parameters for the linkage angles, which were taken from moat.

Since the PES contains contributions from all the energy terms, rather than just the dihedral term itself, the resulting dihedral parameters play a role in accounting for limitations in the overall form of the energy function with respect to the change in energy as a function of conformation. Thus, it is common that optimization is performed on low‐penalty dihedral parameters obtained from the CGenFF program. For example, the ideal parameters for the same dihedral between different ring systems will typically differ due to different contributions of the nonbond interactions to the PES. However, given that the same dihedral parameters, based on identical atom types defining the dihedral, cannot be optimized specifically for each model compound in the present study, it is necessary to compromise, which is often done by selecting a model compound for that parameter that is representative of all the compounds that contain that term. Typically, a simplified model compound is designed that contains only the necessary functional groups related to the target dihedral being optimized by substituting specific functional groups on an individual nucleotide by a methyl group or hydrogens. This approach was applied to the 5‐substituted uracils, N4‐substituted cytosines, 7‐substituted deazaguanines, and N6‐substituted adenines, where each group has diverse side chains but identical base ring systems. For example, in the case of the C5‐O7 bond in 5‐substituted uracils, the functional groups in the side chain often interact with O4 in the base (Fig. 2), impacting the PES. To account for this, the bases with multiple functional groups were simplified to benzene (or other simple heterocycles).

To optimize the C5‐O7 dihedral in 5‐carboxymethoxy uracil (OAU and others), methoxylbenzene (bzmo, Supporting Information [supporting data document SDD], 3.17) was the model compound, and the optimized C6‐C5‐O7‐C8 dihedral parameters were transferred to OAU. While the atom types of C6‐C5‐O7‐C8 in bzmo are slightly different from those in OAU, this allows the generalization of one set of parameters to a family of analogs. Whenever a conformational correction is required for an individual molecule, only the dihedral term unique for that molecule (e.g. C4‐C5‐O7‐C8 for OAU) is updated and the universal dihedral term (C6‐C5‐O7‐C8 here, shared by a group of analogs) is kept unchanged.

### Molecular Structures and Nomenclature

#### Model compounds

In the charge optimization, the model compounds for the modified bases were built with a methyl group replacing the ribose. The net charge of this methyl was set to zero to maintain an integer charge for the base, consistent with the CHARMM strategy of using modular building blocks. Modified bases with extra rings or flexible side chains were further broken down into several parts (e.g. side chains and base heterocycle) to avoid confounding intramolecular interactions that may interfere with the transferability of the parameters (see an example in Fig. 2). The cleavage sites were chosen as nonconjugated and nonpolar bonds (e.g. an aliphatic C‐C bond) with the new termini capped with a methyl group with zero net charge or a +0.09 charged hydrogen atom compensated by a −0.09 change in the charge on the atom to which it was attached. As special cases, the models for base‐conjugated hypermodification, such as the side chains of carbamoyladenosines (HNA, 26A, 66A, t6A, 12A, and 6GA) had benzene substituting the adenine.

For bonded terms the model compounds were built to maintain simplicity while including all atoms necessary for the parameters being targeted. Thus, some model compounds were the same as those used in the charge optimization, and some were fragments from modified bases, whose charges were either well predicted by the CGenFF program or already optimized in the previous step (Fig. 2). The parametrized small model compounds were then assembled into the full molecule. In cases where both the base and ribose were involved in the target dihedral, the model compound contained sugar ring parameters transferred from NA36 with the base treated by CGenFF. In total, 53 target model compounds for the charges and 95 for the bonded and dihedral terms were optimized (see Supporting Information [SDD]).

3D structures of modified nucleosides were taken from Aduri et al.^39^ or created using Maestro9.3 (Schrödinger, LLC, New York, NY, 2012) based on the information in the RNA Modification Database (http://mods.rna.albany.edu). If the modification had multiple protonation states and/or tautomers, the physiologically dominant state was treated as the default species. For example, the primary and secondary aliphatic amines had the protonated state as the main species, and the carboxylate was in the ionized, negatively charged state; other accessible states were included in the force field as shown in the Supporting Information SDD.

#### Nomenclature

**Figure 3:**
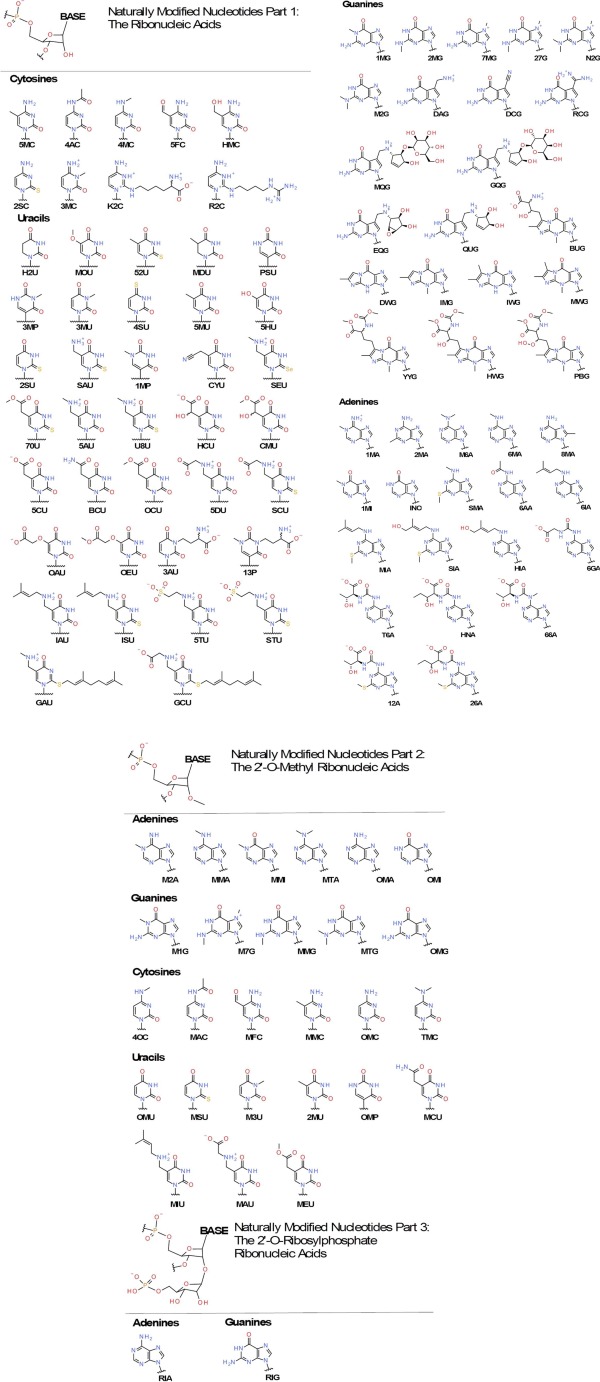
2D structures of modified nucleotides covered in this force field. The bases are sorted by attached ribose types, i.e. canonical ribose, 2′*‐O‐*methylribose and 2′*‐O‐*ribosylphosphate ribose. [Color figure can be viewed in the online issue, which is available at wileyonlinelibrary.com.]

We use three types of names for the modified nucleosides: the IUPAC common name, the symbol used in the text, and the three‐letter code used in the force field toppar files. The symbol designations are consistent with the RNA Modification Database, which have been widely accepted in the literature. In most cases, the three‐letter codes adopted the AMBER convention^39^ to avoid confusion for users, except for some nucleotides for which the names used in the Protein Data Bank (PDB) were used instead of the AMBER names. This was done to avoid problems where AMBER names were already taken by compounds previously defined in CGenFF, and to keep the users from having to edit frequently occurring modified nucleotides in PDB files, such as 2′*‐O‐*methylnucleotides and dihydrouridine. The modified nucleotides including their designation, and tautomer names as well as the 2D structures are in Table S1 in the Supporting Information and Figure 3, respectively. The alternative three‐letter codes that differ between CHARMM, AMBER and PDB are also provided as comment lines in the topology file Supporting Information. As a part of the list, Table 1 shows the compounds that are presented in detail in this work. In this study, the nucleotides and their bases are named using capital three‐letter uppercase codes, and the fragment compounds are named using four‐letter lowercase codes.

**Table 1:** Modified nucleosides that are presented in detail in this work; for a complete list of the modified nucleosides in the force field see Supporting Information.

Symbol^a^	Code^b^	Common name
Am	OMA	2′*‐O‐*methyladenosine
m^6^A	6MA	N6‐methyladenosine
Cm	OMC	2′*‐O‐*methylcytidine
ac^4^C	4AC	N4‐acetylcytidine
ac^4^Cm	MAC	N4‐acetyl‐2′*‐O‐*methylcytidine
m^4^C	4MC	N4‐methylcytidine
k^2^C	K2C	Lysidine
Gm	OMG	2′*‐O‐*methylguanosine
m^2^G	2MG	N2‐methylguanosine
m^7^G	7MG	7‐Methylguanosine
Um	OMU	2′*‐O‐*methyluridine
*Ψ*	PSU	Pseudouridine
D	H2U	Dihydrouridine

a Symbols conventionally used in the literature.

b The three‐letter code used in the force field files, and figure legends.

Atom names from the PDB were used when possible, but significant differences are present in a number of nucleotides. In these cases, the PDB nonunique atom names were changed and we used the following numbering scheme: For the bases, atoms in the substituent side chain were numbered starting from #7 in pyrimidines and from #10 in purines, with the number increasing along the chain of nonhydrogen atoms; carbonyl or hydroxyl oxygen and all hydrogens were given the same number as the heavy atom to which they are attached. All backbone ribose atom names end with a prime as is done in NA36. The 2′*‐O‐*methyl carbon was named CM2 as generally occurs in the PDB, and the 2′*‐O‐*ribosylation atom names end with “A” for ribosyl atoms and “X” for phosphate atoms. Since there is currently no standard nomenclature for modified nucleotides, users are advised to check and make sure the nucleotide and atom names are consistent with the topology file.

### Computational Details

All QM calculations were performed with Gaussian09 (Gaussian, Inc, Wallingford CT, 2009), with an RMS force convergence criterion of 10^−5^ Hartree/Bohr (Opt = tight) for structural optimizations. Geometry optimizations, frequency and potential energy calculations were performed with the MP2 method, while for water‐compound interactions (except for thio‐compounds) the HF method was used. The basis set 6‐31G(d) was applied for neutral and cationic compounds, and 6‐31+G(d) for anions. Dipole moments were the HF/6‐31G(d) values based on the MP2/6‐31G(d) geometries. Empirical force field calculations were performed with CHARMM.^14^ Energy minimizations used an infinite cutoff for non‐bonded interactions. Depending on system size, the minimization included 100 to 300 steps of conjugate gradient followed by 50 to 100 steps using the adopted basis Newton Raphson (ABNR) method, with an RMS force convergence criterion of 10^−5^ kcal/mol/Å. In PES a harmonic restraint with a 10^4^ kcal/mol/radian^2^ force constant was applied on the target dihedral. The PED of vibrational analysis was carried out using MOLVIB^49^ in CHARMM.

Coordinates for the nucleotides used in MD simulations were initially generated in *anti*, *north* conformations. Oligonucleotides were built using Maestro9.3 in an A‐RNA conformation, and modifications were generated in an energy minimum conformation using CHARMM while keeping the sugar pucker and glycosidic torsion unchanged. In all cases the 5′ and 3′ termini of oligonucleotides were terminated with a hydroxyl group. Simulations were performed in rhombic dodecahedral solution boxes using the CHARMM‐modified TIP3P water model^44^ and with periodic boundary conditions applied. The distance from solute nonhydrogen atoms to the edge of the box was at least 12 Å. Na^+^ ions were added to achieve charge neutrality by randomly substituting the appropriate number of water molecules. After the solvent box setup, a harmonic restraint with a force constant of 80 kcal/mol/Å^2^ was applied to the solute nonhydrogen atoms, and 100 to 300 steps of ABNR minimization was performed.

In production simulations, all harmonic restraints on solute atoms were released. The SHAKE algorithm^50^ was used to constrain the lengths of covalent bonds involving hydrogen to their equilibrium values, allowing a 2 fs time step to be used in the integration of Newton's equation. A lookup table^51^ was applied for nonbonded interactions, and the fshift and vfswitch methods^52^ with a 12 Å cutoff, were employed to treat the electrostatic and vdW interactions, respectively. The systems were first heated from 48 to 298 K in 20 ps and then equilibrated for an additional 10 ps at 298 K in the NPT ensemble. The production simulations were performed in the NVE ensemble for 100 ns, where the temperature was allowed to deviate ±5 from 298 K.

### Conformational Definitions and Analyses

The dihedral (*φ*) about a rotatable bond is always defined using nonhydrogen atoms unless the terminal atom is hydrogen. When the first atom eclipses the fourth atom (*φ* = 0°) the dihedral is *cis*, and when *φ* = 180° the dihedral is *trans*. The glycosidic torsion (*χ*), which is about the base‐ribose linkage, is defined by the dihedral O4′‐C1′‐N1‐C2 (pyrimidine), O4′‐C1′‐N9‐C4 (purine) or O4′‐C1′‐C5‐C4 (pseudouridine), and its conformation is denoted as *anti* for 170° < *χ* < 300° and as *syn* for 30° < *χ* < 90°.^53^

The pucker of a five‐membered ring is defined by the pseudorotation phase angle^54^ (*P*) which is a combination of five ring torsions. Thus the ribose pucker is denoted by the pseudorotation quadrants, which are *north* (315° < *P* ≤ 45°), *west* (45° < *P* ≤ 135°), *south* (135° < *P* ≤ 225°), and *east* (225° < *P* ≤ 315°). Since in unmodified RNA the puckers are mainly found in the *north* (*P* ≈ 18°) and *south* (*P* ≈ 162°) quadrants,^55^ the bisectional notation of pseudorotation is also adopted in experiments: *C3'endo* (270° < *P* ≤ 90°) which includes *north*, and *C2'endo* (90° < *P* ≤ 270°) which includes *south*. In QM PES, the furanose was kept in *C3'endo* by restricting C4′‐O4′‐C1′‐C2′ = 0.0°, and in *C2'endo* by restricting C3′‐C4′‐O4′‐C1′ = 0.0° as previously described.^56^

Base stacking was described using three geometric terms: the distance (*R*) between two glycosidic nitrogen atoms, the pseudo dihedral (*Φ*) formed by two base‐axis atoms, i.e. N1‐C4 of pyrimidine (Y) or N9‐C6 of purine (R), and the angle (*Θ)* between normal vectors of the two bases.^16^, ^57^, ^58^ The bases were considered to be stacked when *R* ≤ 6 Å, (*Φ*_0_ – 40°) ≤ *Φ* ≤ (*Φ*_0_ + 40°), and *Θ* ≤ 40° or *Θ* ≥ 140°, where *Φ*_0_ is the value when two bases are stacked in the ideal A‐RNA geometry (*Φ*_0_ ≈ 20° for 5′‐R‐R‐3′ or 5′‐Y‐Y‐3′ stacking, *Φ*_0_ ≈ 40° for 5′‐R‐Y‐3′ and *Φ*_0_ ≈ 0° for 5′‐Y‐R‐3′ stacking).

## Results and Discussion

The 112 modified nucleotides consist of 26 adenosines, 15 cytidines, 27 guanosines, and 44 uridines, and include combinations of more than 70 base modifications with two ribose modifications. Several tautomers and protonation variants have also been included. This section gives an overall description of the parametrization process, exemplified using the molecule 4MC. Results for all the molecules are in the Supporting Information SDD. The validation is also discussed in detail for several modifications in nucleosides or oligonucleotides for which experimental data are available.

For the parameter optimization the nucleotides were split into smaller model compounds, based on two structural classes. The first class includes bases with a simple modification (e.g. methylation, hydroxylation, or thiolation) and aromatic or conjugated molecules without a long aliphatic side chain. Examples in this class are the bases of inosine, 4‐methylcytidine, 7‐methylguanosine, 2‐thiouridine, pseudouridine, and wyosine, as well as the scaffold of a group of complicated modified bases (e.g. 7‐deazaguanine and 2‐aminocytosine). These molecules were subjected to optimization of the charges and bonded energy terms and, in general, did not require any dihedral PES scans. The second class contains flexible chains that have been separated from the bases (e.g. dimethylammonium, N1, N1, N2‐trimethylurea, and zwitterionic alanine) and any nonaromatic bases and ribose (e.g. dihydrouracil and 2′*‐O‐*methyl ribose). These molecules were subjected to charge, bonded, and dihedral parameter optimization.

Atoms in modified bases were represented using the atom types from CGenFF. For the ribose moiety, which is unmodified in most cases, the parameters were taken directly from NA36. For the ribose modifications, the 2′*‐O‐*methyl group used CGenFF atom types and the 2′*‐O‐*ribosylmonophosphate used Carb36, while the ribose ring and phosphate maintained the NA36 atom types. Thus, optimization was performed separately targeting the novel parameters in the base and ribose moieties, after which the fragments were assembled into nucleosides and nucleotides. This modular approach means that the base modifications are also transferable to deoxyribonucleotides.

### Charge optimization

#### Model compound‐water interaction

The atomic charges were optimized targeting water‐model compound minimum interaction energies and distances along with dipole moment magnitudes and orientations. We illustrate the charge optimization protocol with N1, N4‐dimethylcytosine (4MC, the model compound for the N4‐methylcytidine base) (Fig. 17 in Supporting Information SDD 1.17). For 4MC three water probes were used on H4 (each time the water rotated 60° around the O_w_···H_4_ axis), six on O_2_ and N_3_ (water rotated 60° around the H_w_···O_2_/N_3_ axis), two on H5 and H6 (water rotated 90° around the O_w_···H_5_/H_6_ axis), and one on H41 and H43. Interactions with H42 were excluded because the interacting waters were very close to N3, resulting in an unfavorable interaction. Thus, a total of 21 water‐model compound complexes were used in the charge optimization of 4MC (Table 51 in Supporting Information SDD 1.17).

In each H_2_O‐4MC complex, the minimum interaction energy and distance between interacting atoms (O_w_···H_4MC_ or H_w_···O_4MC_) were calculated as $\Delta E=\left(E_{complex}{-E}_{4MC}-E_{water}\right)$ and $R=\left|r_{4\text{MC}}-r_{\text{water}}\right|$, where **r**_x_ represents the coordinates of the two directly interacting atoms. As previously described,^59^ the QM interaction energy of the neutral compounds was multiplied by 1.16^44^, ^45^ since 4MC is a neutral polar compound, and an offset −0.2 was applied to the QM polar interaction (hydrogen bond) distances (i.e., the O_w_···H_5_/H_6_/H_41,3_ interactions were excluded).

The starting atomic charges were generated by the CGenFF program using the ParamChem website. The atomic charges were then adjusted iteratively until the interaction energy and geometry differences (ΔΔ*E =* 1.16Δ*E*_QM_ – Δ*E*_MM_ and Δ*R = R*_QM_ *+R*_offset_ *– R*_MM_) between MM and QM calculations were fully minimized, yielding an RMS difference of 0.20 kcal/mol for energies and 0.10 Å for distances. For comparison, the results calculated using initial charges gave RMS differences of 0.54 kcal/mol and 0.10 Å for the interaction energies and distances, respectively. Consistent with previous CHARMM parametrization efforts^26^, ^36^, ^60^ reproducing the QM results with an energy RMSD <0.5 kcal/mol and distance RMSD <0.2 Å was considered acceptable.

**Figure 4:**
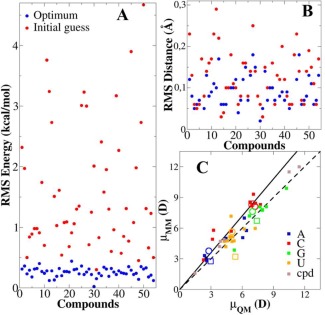
RMS difference between QM and MM calculated a) interaction energies and b) distances for 53 independent compounds, and c) the dipole moments of 40 neutral compounds. Each dot in a) and b) is the RMS calculated from all water probes with one model compound. The solid squares in c) are 40 compounds for modified bases (a, c, g and u) and fragments (cpd); open symbols are the four canonical bases (circles: H‐N1/N9; squares: Me‐N1/N9). The dashed line represents **μ**_MM_ = **μ**_QM_ and the solid line represents **μ**_MM_ = 1.2 **μ**_QM_. [Color figure can be viewed in the online issue, which is available at wileyonlinelibrary.com.]

The charges of the 53 model compounds were determined in the same way, resulting in substantial improvements, in particular for the RMS differences between QM and MM calculated interaction energies (Fig. 4a). Similar to previous observations for the LJ parameters and the minimum interaction distances,^61^ the distances were not very sensitive to different charge assignments, such that significant improvements did not occur during the optimizations (Fig. 4b). However, the interaction energies showed significant improvement in the agreement with QM energies after the optimization in a number of cases. From inspection of the results the poorest initial predictions mainly belonged to the protonated amines, positively charged heterocycles, and wyosines (hypermodified guanosines), as analogs of these species are not yet available in CGenFF. For the remaining compounds the initial energy RMS difference was typically less than 1.5 kcal/mol and in some cases less than 0.5 kcal/mol. Thus, the CGenFF program assigned reasonable charges in a number of cases, though for cases in which compounds similar to those being presently studied were not included in the CGenFF training set, significant disagreement with the QM data was obtained. We note that for these compounds the CGenFF penalties for the charges were typically quite large (around 150), indicating the need for further optimization, as previously described.^42^, ^43^

Finally we emphasize that the charges were subjected to a restraint during the optimization. This was performed to prevent large deviations from the starting “CHARMM‐like” charges that could be obtained from over fitting. However, while only very small changes to the initial CGenFF charges occurred, good agreement between QM and MM interaction energies and dipole moments (see following section) was obtained.

#### Dipole moments

The resulting dipole moments for the cytosine and fragment compounds showed the expected overestimation of their magnitudes, while for a number of other compounds there is an underestimation (Fig. 4c). This discrepancy is due to the methyl group added to the bases whose charge was set to zero to keep an integer net charge on the base. Considering the canonical bases (parameters from NA36) as the control group, the QM calculated dipole moments (**μ**_QM_) of A, G, and U is directed from N9/N1 to H9/H1, while in C it is in the opposite direction, from H1 to N1. When the H9/H1 is replaced by a methyl group, the methyl group had a small positive charge in the QM calculation, so the ǁ**μ**_QM_ǁ remained largely unchanged for the four bases; slightly increasing for A, U, and G and slightly decreasing for C (see open circles *vs*. open squares in Fig. 4c). Furthermore, using NA36, the methylation decreased ǁ**μ**_MM_ǁ significantly for A, G, and U, and increased it slightly for C, which was opposite to the QM results. Correspondingly, the methylated analogs of the modified bases showed the same trend as their parent bases. Importantly, the orientations of the dipole moments were reproduced correctly, with an RMSD of the angles between **μ**_MM_ and **μ**_QM_, of 17.1° for bases and 5.1° for fragment compounds (Table 2). Overall, the optimized charges satisfactorily reproduce the QM dipole moments while also reproducing the interactions with water, indicating their suitability for modeling and simulation studies in aqueous environments.

**Table 2:** Dipole moment deviations between QM and MM.

	Magnitude difference (%)			Angle difference (°)		
	All	Base	Model	All	Base	Model
AD^a^	10.2	10.0	11.1	12.3	13.8	3.8
AAD^b^	15.4	16.2	11.1	12.3	13.8	3.8
RMSD^c^	22.6	23.9	12.2	15.9	17.1	5.1

a AD: average difference.

b AAD: average of absolute deviation.

c RMSD: root mean square deviation.

### Bonded geometry optimization

The geometric terms were divided into two categories. The first includes bond, angle, Urey‐Bradley, stiff dihedral terms, and improper dihedral terms. With these stiff degrees of freedom small deformations yield energy changes of several tens to hundreds of kcal/mol, which is out of the sampling range of typical MD simulations. The second category contains the flexible dihedrals whose energy changes are often less than 10 kcal/mol, a range that is sampled in simulations, hence accurate treatment of these dihedral PES is important for proper conformational sampling in MD simulations.

#### Bond, angle, and improper dihedral optimization

The equilibrium geometry parameters were optimized by fitting the MM geometry to the MP2 optimized geometry, and force constants were optimized by reproducing the MP2 vibrational modes. Since any vibrational mode contains contributions from multiple internal degrees of freedom, the degree of freedom that dominates the vibration was first taken into account, and then the less significant terms were considered, with degrees of freedom that contribute less than 15% ignored. When the PED results were ambiguous, the force constant was determined by three‐point potential energy scans.^62^ With the optimized equilibrium terms and force constants, the empirical model reproduced the QM results quite well (Table 186 in Supporting Information SDD 2.9). Generally the deviations between QM and MM were less than 0.03 Å and 3° for bonds and valence angles, respectively, while the vibrational frequencies were within 5% of the target QM values.

A general consideration is the limited number of atom types in the force field such that the same bonded parameter occurs in multiple molecules, thereby limiting the ability to optimally reproduce the target data for all the molecules. In the present study parameters previously available in CGenFF were not subject to additional optimization. All other parameters assigned by the CGenFF program have an associated penalty score, which is low when there is a closely analogous parameter based on similar atom types present in CGenFF. Such parameters with low penalties (<5 for bonds and <10 for angles) were transferred directly from CGenFF to the modified nucleotides. Thus, there were only a limited number of bonded parameters for optimization for each model compound. For this reason there are a few angles with differences >3° in the 4MC case (Table 185 in Supporting Information SDD 2.9). Table 3 summarizes the overall statistics of MM geometries relative to the QM values using the optimized parameters.

**Table 3:** Statistics for internal geometries and the related vibrational frequencies of all rigid bonded terms.

	Number of data points	AD^a^	AAD^a^	RMSD^a^
Bond length^b^ (Å)	68	0.001	0.011	0.015
Valance angle (°)	201	0.38	1.10	1.46
Stiff dihedral^c^ (°)	108	0.13	1.40	2.13
Improper (°)	22	0.35	0.64	0.99
Vibrational frequencies	148	1.3%	4.8%	7.0%

Geometric data based on the final parameter set.

a AD: average deviation; AAD: absolute deviation; RMSD: root mean square deviation.

b Including 1,3‐distance in UB terms.

c Dihedrals about one bond were counted only once.

#### Dihedral optimization

The parameters of flexible dihedrals were determined using PES. Here we mainly focus on the determination of glycosidic torsions and the reproduction of nucleoside conformations. The fitted energy surfaces of all torsions are in the Supporting Information, and additional technical details concerning determination of torsion parameters can be found in the previous CGenFF publications.^36^, ^41^

The glycosidic dihedrals (*χ*) represent an interesting situation as they contain atom types from two different sets of initial parameters; NA36 for the sugar and CGenFF for the bases. In NA36, there are unique *χ* parameters for each nucleoside, while in CGenFF there are only two sets (one for A/G and one for C/U). The *χ* torsion parameters in NA36 were adjusted accurately for the *anti* and then the *syn* conformations, as required to treat the conformational properties of oligonucleotides in condensed phase simulations.^26^, ^53^, ^63^, ^64^ To check the feasibility of transferring *χ* parameters from CGenFF we first calculated the PES using both NA36 and CGenFF for a number of model compounds (Supporting Information Fig. S1), showing the CGenFF parameters to be consistent with NA36, especially for the minima and barriers. Furthermore, when both sets of parameters were used in nucleoside simulations in solution, a significant difference only occurred with uridine (Supporting Information Fig. S2), with the population of the *C3'endo* sugar pucker of uridine being larger than that obtained in NMR experiments.^65^, ^66^, ^67^ This enhancement corresponds to about a 0.4 kcal/mol free energy difference (according to the Boltzmann distribution), which does cause an excess of north pucker in monomer simulations. However, this discrepancy does not occur for unmodified uridine, which uses NA36 parameters, while most modified uridines, especially in the anticodon, are known to stabilize the north (*C3'endo*) conformation.^68^, ^69^ The one exception, dihydrouridine (discussed below), does not use this set of *χ* parameters. Accordingly, the *χ* parameters of CGenFF were adopted for the modified bases.

Most modifications in the base do not explicitly change the atom types about the glycosidic bond so the *χ* parameters of these nucleosides are identical to the canonical ones and no optimization was required. However, four groups of nucleosides, *viz*. pseudouridines, dihydrouridines, 7‐methylguanosines, and 2‐aminocytidines (lysidine and agmatidine), have new *χ* parameters and optimization was necessary. The model compounds for *χ* parametrization included the whole base (except for lysidine or agmatidine whose long side chain was excluded), whereas the ribose was represented by tetrahydrofuran (with the same atom numbering and atom typing as ribose), the simplest model to mimic the ribose pucker. The *χ* torsions were scanned with the furanose restricted in both *C2'endo* and *C3'endo* puckers (Supporting Information SDD 3.58–3.62). The parameter determination combined with the conformational analysis is discussed in the next section.

In addition to the *χ* torsions, dihedrals of ribose 2′*‐O‐*modifications also involve atom types from both NA36 and CGenFF. The ribose ring atoms use NA36 atom types and the atoms in the substituent, including O2′ and CM2, use CGenFF atom types. The 2′*‐O‐*ribosylation is bulky and restricts the conformational diversity of the ribose; optimization of the 2′*‐O‐*ribosyl linkage is not discussed here, but shown in the SI. The dihedral about the C2′‐O2′ bond of 2′*‐O‐*Me was determined with both the *C2′endo* and *C3′endo* furanose puckers; the conformational details of the four nucleosides Am, Cm, Gm, and Um are discussed below.

### Conformation of nucleosides and oligonucleotides

The glycosidic torsion is very important for structural properties of nucleotides, and contributes to base pairing and stacking. At the nucleoside or nucleotide level, the *χ* torsion is often coupled with the sugar pucker (*P*), mediated by the O5′ group.^53^, ^56^, ^64^, ^70^ Therefore, structural correlations observed in nucleoside simulations were used to fine tune the parameter fits targeting the QM PES for the four groups of modified nucleosides with new *χ* parameters. In nucleosides that adopt the canonical *χ* parameters small modifications of atomic charges in the base were made to correct deviations from experimental conformations, and one example illustrated below is ac^4^C. The conformational effect of 2′*O*‐methylation will also be discussed since it is a very frequent modification in nucleic acids. Finally an example of *N*‐methylated bases will also be described to show that in many cases fitting gas‐phase QM PES is adequate to yield good agreement with experimental conformational properties. These nucleoside 2D‐structures are shown in Figure 3.

#### Dihydrouridine (D)

**Figure 5:**
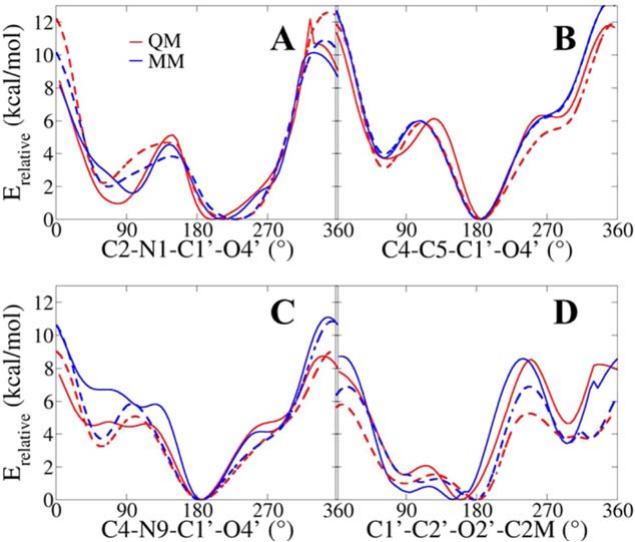
Potential energy surface scans of the *χ* torsion for a) dihydrouridine, b) pseudouridine, and c) 7‐methylguanosine, and 2′‐OMe torsions for d) 2′*‐O‐*methylribose in vacuum. The solid lines represent the energy scanned with a *C3'endo* furanose pucker and the dashed lines with a *C2'endo* pucker. [Color figure can be viewed in the online issue, which is available at wileyonlinelibrary.com.]

Dihydrouridine is a common nucleotide in tRNA, especially in the D‐arm, as well as in other RNA molecules.^6^ It has a C5‐C6 saturated bond, so the base loses the aromatic planarity and is unable to stack.^67^, ^71^ Its *χ* torsion has been reported as *anti*,^71^, ^72^, ^73^ and it intrinsically prefers the *C2'endo* pucker, and also imparts flexibility to the local RNA structure, thereby destabilizing the A‐form helix.^67^, ^71^, ^73^ In PES analysis, the energy of the *χ* torsion had similar barrier heights and positions of the minima for both the *C3'endo* and *C2'endo* puckers (Fig. 5a). The lowest energy minimum, which corresponds to *anti*, was around 210° to 225°, and the second, related to *syn,* was around 75°. In particular, there were two differences from canonical U in the PES. First, the energy difference between *anti* and *syn* is smaller than for U, only 1 to 2 kcal/mol in favor of *anti*. Second, the *anti* minimum is broader and located at higher values of *χ*.

**Figure 6:**
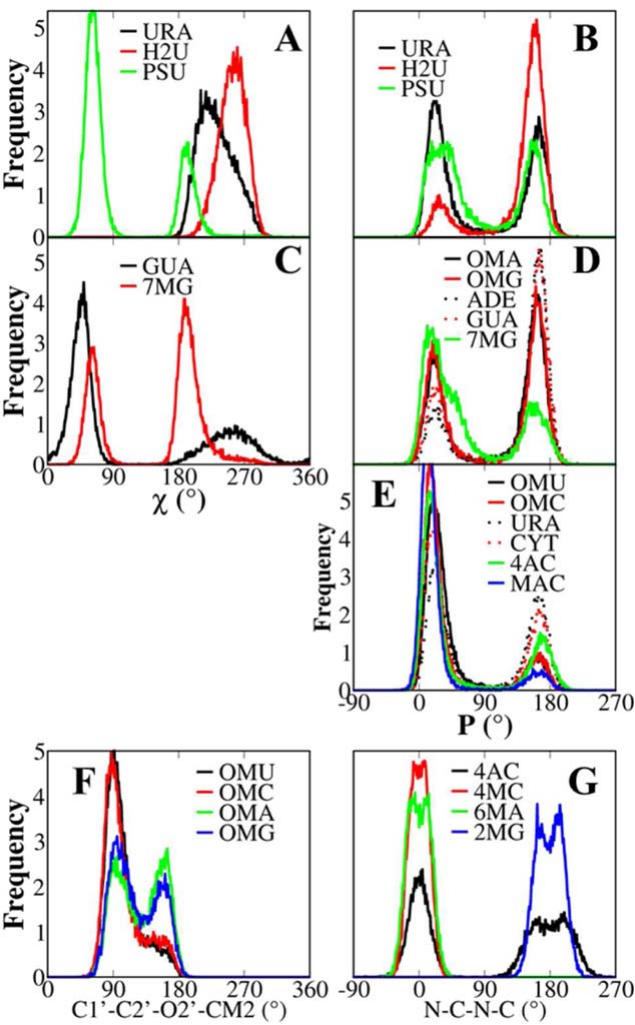
Conformational distributions of different torsions for nucleosides from the MD simulations. Each distribution was sampled from 25,000 snapshots over 100 ns. a) The glycosidic *χ* torsions and b) the pucker P pseudorotations of dihydrouridine and pseudouridine compared with uridine; c) the *χ* torsion of 7‐methylguanosine and guanosine, and the pucker P of d) modified purines and e) modified pyrimidines for the canonical nucleosides; f) the 2′‐OMe torsions of four nucleosides with canonical bases; g) torsions of N‐substituted nucleosides where the dihedral notation corresponds to N3‐C4‐N4‐C7 in 4AC, N3‐C4‐N4‐CM4 in 4MC, N1‐C6‐N6‐CM6 in 6MA, and N1‐C2‐N2‐CM2 in 2MG. [Color figure can be viewed in the online issue, which is available at wileyonlinelibrary.com.]

In simulations of the dihydrouridine nucleosides the *χ* torsion remained in its starting conformation (Fig. 6a), indicating that the barrier between *anti* and *syn* of a nucleoside is increased in aqueous solution compared with the gas phase PES (Fig. 5a). The sugar pucker always preferred *C2'endo* even though the starting structures were *C3'endo* (Fig. 6b). However, the pucker was interconverting, with an energy difference between the two conformations of 1.0 to 1.3 kcal/mol. In longer RNAs, the conformation of dihydrouridine will be dependent on the environment, but one could expect *C2'endo* to dominate. Recent assessments of AMBER force fields^74^, ^75^ have identified *anti* and *C2'endo* as the conformational benchmark for dihydrouridine, but here we will consider additional information. First the *C2'end*o pucker phase was ∼10° lower compared with canonical U, whose *C2'endo* minimum was located around 165°. This is related to the *C2'endo‐C1'exo* conformations reported in crystal structures,^71^, ^73^ but slightly different from the *C2'endo‐C3'exo* obtained using NMR vicinal coupling data.^72^ Second, consistent with crystal structures, where *χ* is in the range 228 to 253°,^71^, ^73^ the *χ* torsion was 30° to 40° higher than *anti* in U, which distributes around 225°. This shift in conformation comes from the unsaturated base, which has different carbon and hydrogen orientations of C5‐C6, causing deformation of the ribose ring. It has been suggested that a π* orbital interaction in canonical uracil stabilizes the interaction between conjugated C5=C6 and O4′ and can affect the sugar pucker.^68^, ^69^ In response to *C2'endo‐C1'exo*, the dihedral C4′‐O4′‐C1′‐N1 decreases from the canonical ∼228° to ∼208° (206.1°, 212.0°, 205.8°, and 212.0° in the crystal structures), shifts the base outward from the ribose ring around the O4′‐C1′ bond, and shifts the *χ* torsion to higher values.

#### Pseudouridine (Ψ)

Pseudouridine was the first identified modified base and it is a ubiquitous nucleotide in RNA molecules.^6^, ^76^ In tRNA it is mainly present in the anticodon stem and TΨC‐loop. In contrast to dihydrouridine, *Ψ* induces more *C3'endo* in its neighbors and enhances the rigidity of the local structure, and thus stabilizes the A‐form RNA architecture.^76^, ^77^, ^78^, ^79^ *Ψ* is like a uracil flipped 180° around the N3‐C6 axis, and C5 becomes the linkage atom (Fig. 3). The linkage torsion O4′‐C1′‐C5‐C4 is still called *χ*, and its PES was very similar for the *C3'end*o and *C2'endo* puckers (Fig. 5b). The lowest energy minimum corresponding to *anti* was almost 180°, and the valley was narrower and shifted toward lower values compared with thymine or uridine in NA36. This is in agreement with the “low *anti*” seen in experiments for an RNA strand where the *χ* torsion of *Ψ* is rigid.^78^ The second minimum, corresponding to *syn*, was around 60° and 3.5 kcal/mol higher than *anti*. This property is consistent with experimental observations of RNA oligomers where *Ψ* always favors *anti*.^76^, ^77^, ^78^, ^79^, ^80^, ^81^, ^82^, ^83^

**Figure 7:**
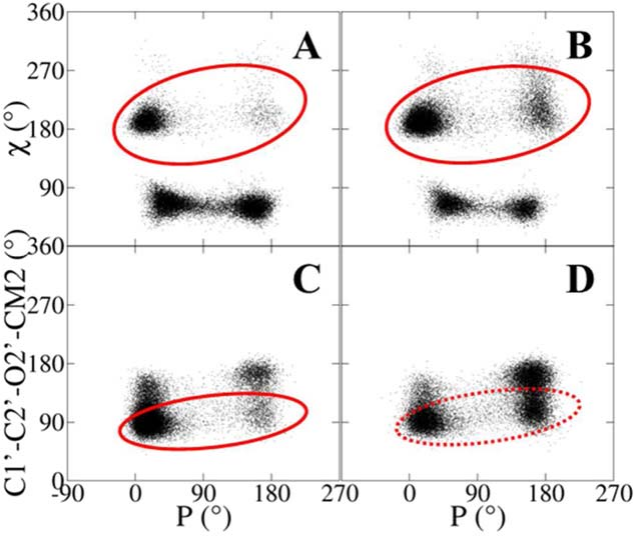
Correlation between torsions and sugar pucker for nucleosides from the MD simulations, including the *χ*/P correlation of a) pseudouridine and b) 7‐methylguanosine, and the 2'OMe/P correlation of c) 2′*‐O‐*methylcytidine and d) 2′*‐O‐*methylguanosine. The solid red circles in a) and b) show the dominance of *north* when *χ* is *anti*, and similarly dominant *north* occurs in c) 2′*O*‐methylpyrimidine when 2'OMe torsion is *Base* in c), but this correlation is weaker in d) 2′*O*‐methylpurine. [Color figure can be viewed in the online issue, which is available at wileyonlinelibrary.com.]

Interestingly in nucleoside simulations *Ψ* mainly sampled the *syn* conformation (Fig. 6a), which agrees with experiments on nucleoside *Ψ* or its base‐alkyl derivatives, where Ψs were found in *syn* and their U counterparts in *anti*.^84^, ^85^, ^86^ No significant pucker preference of Ψs was seen in these experiments, nor in our simulations (Fig. 6b). A similar conformation was also obtained with AMBER and *Ψ* was considered to be flexible.^74^ This seemingly conflicts with the depiction that *Ψ* is rigid in *anti*/*C3'endo*. But our analysis indicates that *anti* is highly correlated with *C3'endo* for *Ψ* (Fig. 7a), which is not the case for canonical nucleotides^64^ (Supporting Information Figs. S3a–S3d). In oligonucleotides, the structure is ordered by WC base pairing and stacking, so *Ψ* will be more restricted to *anti* and thus the *C3'endo* pucker becomes dominant. An additional observation based on experimental structures indicated that a water molecule bridges between N1‐H1 and the 5′‐phosphate O1P, reducing base motion and improving the structural stability of RNA strands.^76^, ^87^, ^88^ However the stabilization of *Ψ* still exists in the absence of H1, as 1‐methyl *Ψ* displayed a similar stacking enhancement as *Ψ* does in oligonucleotides.^80^ We therefore suggest that the rigidity of *Ψ* is an intrinsic property, and it mainly populates the *anti*/*north* conformation in an RNA oligomer context. Conformational comparison of dihydrouridine and *Ψ* in oligonucleotides is further discussed below.

#### 7‐Methylguanosine (m^7^G)

7‐methylguanosine is a common nucleoside, especially in tRNA, and it is also the 5′‐cap of eukaryotic mRNA.^89^ In tRNA it is mainly located in position 46 in the variable loop. The methylation on N7 results in a positively charged purine ring and makes N1 more acidic. Although m^7^G has been found to have both protonated (keto) and deprotonated (enol) zwitterionic states, its biochemical functions are mostly related to the protonated state.^90^, ^91^, ^92^ Furthermore, a positively charged m^7^G keeps the same configuration as guanine, in contrast to the N1 deprotonated state, so that hydrogen bonding and stacking patterns in tRNA are not disturbed.^93^, ^94^ Therefore we only considered the protonated state for m^7^G and its base‐alkyl derivatives.

The PES along the torsion O4′‐C1′‐N9‐C4 had the same minimum positions (*anti*) around 180° and barrier heights in both furanose puckers (Fig. 5c). Compared with the PES of canonical G (Supporting Information Fig. S1), the range of *anti* was much narrower in m^7^G and the *syn* minimum was 3 to 4.5 kcal/mol higher than *anti*; in *C3'endo* the *syn* minimum almost disappeared. It is clear from the QM energy scan that the 7‐methylation enhances the stability of *anti* and makes m^7^G more rigid than G.

In crystal structures protonated m^7^G is reported to be in an *anti*‐*C3'endo* conformation^95^ and NMR experiments indicate that it is a mixture of *syn* and *anti* in solution with *anti* highly related to *C3'endo*.^91^ The trend in the simulations was consistent with the PES prediction and experimental data. The *χ* torsion preferred *anti* and the sugar was more in *C3'endo* compared with G (Fig. 6c). The *syn* was still significantly sampled due to the attractive interaction between O5′ and the N2 amino group, which stabilized *syn* even though this interaction was weaker in the absence of the phosphate. Also the *χ* torsion distribution was more narrow than in G and shifted to ∼190°, and the pucker was *north*/*west* of *C3'endo* (Fig. 6d), which coincides with the *C3'endo‐C4'exo* conformation (*χ* = 181° and *P* = 40°) in the crystal structure.^95^ Here the *west* pucker (*P* ≈ 50°) was related to the *syn*‐*C3'endo* conformation, while it was still *north* (*P* ≈ 18°) in *anti*. Similar to the pseudouridylation on U, the 7‐methylation on G induces a strong correlation between *anti* and *C3'endo* (Fig. 7b), hence combined with evidence that the added methyl and polarization of the base effectively increase the base stacking, hydrogen bond and backbone stability,^58^, ^90^, ^92^ one can speculate the effect of m^7^G is to explicitly stabilize the A‐form structure and base interaction.

#### 2‐Aminocytidines

Lysidine (k^2^C) and agmatidine (C+) are hypermodified cytidines with the 2‐carbonyl replaced by lysine and arginine, respectively. They are mainly found in the wobble position in the antcodon of tRNA^Ile^ where they are paired with A instead of G, and thus tRNA^Ile^ can read the Isoleucine codon AUA rather than the initiator codon AUG.^96^ The 2‐aminocytidine has a positively charged base and it is the fourth group that required new *χ* torsion parameters. To avoid confounding interactions between the amino acid chain at the 2 position and the rest of the molecule, the side chain was replaced by a methyl group on N2 (Supporting Information SDD 3.61) for the PES scan of the torsion O4′‐C1′‐N1‐C2. However, the bulky methyl substitution (with the methyl group toward the furanose) still contaminated the potential energy along the *χ* torsion. Rotating the 2‐methylamino group 180° introduced an interaction between the amino hydrogen and furanose oxygen, so this did not alleviate the problem. Considering that in practice the k^2^C and C+ conformations will be very restricted, and that electrostatic attraction and steric repulsion of the 2‐position side chain will dominate the *χ* conformational sampling rather than the *χ* torsion, *χ* parameters were optimized directly targeting the PES of the 2‐aminomethyl analog.

#### Conformational effects of D and Ψ

The dihydrouridine base is not aromatic and the ribose has a *C2'endo* pucker, thus causing destabilization of A‐form RNA, while the features of *Ψ* are the opposite. To evaluate these effects it is important to use oligonucleotides. The simple motif ADA naturally occurs in *E. coli* 23S rRNA and the impact of D on this trinucleotide has been studied by NMR.^67^ This NMR experiment observed a reduction of *C3'endo* pucker in the first two nucleotides, and base stacking in ADA compared to AUA. As a comparison, *Ψ* was also studied in another NMR experiment using both AΨA and AAΨA sequences and, as expected, both *C3'endo* pucker and stacking were enhanced in the 5′‐neighbors of *Ψ*, and at low temperature also for the 3′ A.^77^ We have simulated the three trinucleotides AUA, ADA and AΨA under identical conditions.

In ADA the *C3'endo* pucker was destabilized compared with AUA, with a decreased fraction of *north* pucker in 5′ A and D, whereas A‐3′ was less affected (Table 4), which is the same as the trend observed in NMR. However, the pucker populations were not quantitatively equivalent to NMR values, i.e. the fraction *north* of the first two nucleotides was overestimated whereas the last one was underestimated. This is likely because the CHARMM nucleic acid force field was calibrated to give good performance for structurally ordered oligonucleotides. Thus, in the presence of base stacking and an inter‐ribosyl hydrogen bond between O2′ and O4′, the fraction *north* puckers of 5′ A and U were 0.98 and 0.85 in the simulation, compared with 0.45 and 0.46 in NMR, while for A‐3′ it was 0.13 *versus* 0.47, due to the absence of stabilization from an adjacent 3′‐nucleotide. This overestimation of the *C2'endo* pucker for the 3′‐nucleotide was observed in all our trinucleotide simulations. The destabilizing effect of D in the trinucleotide was also seen in the loss of base stacking of D with both adenines, which even allowed the two adenines to stack when the D flipped out of the stacked orientation (Table 4, Supporting Information Fig. S4).

**Table 4:** Population analysis of nucleoside conformations and base stacking in trinucleotides 5′‐ApDpA‐3′, 5′‐ApUpA‐3′ and 5′‐ApΨpA‐3′.

	NMR (%)		Simulation (%)		
Sequence	Nt north		North	Anti	Stacking
5′‐A‐D‐A‐3′	1	42^a^	67.2	60.2	13.0^b^
	2	9	33.4	99.8	10.5^c^
	3	45	10.2	86.2	22.1^d^
5′‐A‐U‐A‐3′	1	45^a^	97.8	42.1	58.5
	2	46	85.3	99.9	21.8
	3	47	12.8	95.0	7.3
5′‐A‐*Ψ*‐A‐3′	1	55^5^	99.6	34.2	89.7
	2	65	99.8	99.9	43.6
	3	55	24.1	88.3	0.0

a From Ref. 57.

b Stacking of first and second bases.

c Stacking of second and third bases.

d Stacking of first and third bases.

From Ref. 66.

The opposite effects happened with AΨA. Here the *north* pucker of the first two nucleotides was increased, and to some extent in the 3′ A (Table 4). This is qualitatively consistent with NMR data for AΨA.^77^ However, the relative populations of *north* pucker of A1, A2, Ψ3, and A4 in the same experiment were 0.64, 0.75, 1.0, and 0.55, respectively, which shows a substantial enhancement of *north* pucker in the 5′‐nucleotides, to which our data were much closer. Considering that the force field is mainly intended for modeling of RNA polymers, the tetranucleotide data are more relevant. Furthermore, the *syn* conformation was not observed for *Ψ* in the oligonucleotide. Combining this with the correlation of *anti*/*C3'endo* discussed above, *Ψ* was kept in a very restricted conformation in an RNA strand. Along with the pucker stabilization, *Ψ* also enhanced the base stacking, as in double‐strand RNA a *Ψ*:A base pair increases the stacking for 5′‐bases but the effect is less pronounced for 3′‐bases.^82^ Here, however, the simulation showed that 3′‐stacking also improved (Table 4, Supporting Information Fig. S4). We think this is because the novel arrangement of *Ψ* base atoms might not only increase the base interactions, but its conformational rigidity also helped order the conformation of the 3′‐nucleotide. The simulation results support the notion that *Ψ* is rigid in long RNA and plays an important role to stabilize base stacking and A‐form RNA.^76^, ^77^, ^78^, ^79^, ^80^, ^81^, ^82^, ^83^

Admittedly the effect of *Ψ* and D can be sequence‐dependent, but it is beyond the scope of this study to analyze all possible combinations of base‐base interactions. Since the influence of the modification was as expected, these modifications are likely to have a similar local role in complicated systems as they have in small model systems.

#### 2′‐O‐Methyl (2′‐OMe) Nucleosides

The 2′‐hydroxyl group, which is the major chemical difference between DNA and RNA, increases the *C3'endo* pucker and stabilizes the A‐form helix of RNA. A previous study on the impact of the 2′‐OH orientation on the stability of RNA in our laboratories lead to the NA36 force field.^42^ Three possible orientations of the 2′‐OH torsion exist, i.e. *Base* (30°–98°), *O3*′ (190°–280°) and *O4′* (305°–360°), with *Base* dominating in solution.^97^, ^98^, ^99^ Once the 2′‐OH is methylated, the hydrogen bond capacity is lost and steric repulsion becomes the dominating factor for 2′‐OMe rotation. The 2′*‐O‐*methylation is a very common modification, and it occurs in dozens of different nucleotides. This modification is often considered to increase the *north* pucker.^100^ It has been found at the wobble position of tRNA, where the Cm has better binding efficiency than C when pairing a G,^101^ but the definite mechanism is still unclear.

To determine the parameters of the 2′‐OMe torsion, a similar model as for the *χ* torsion was applied. We again adopt the tetrahydrofuran scaffold but add the 3′‐OH and 2′‐OMe, and simplify the base to an imidazole (Supporting Information SDD 3.59). The torsion C1′‐C2′‐O2′‐CM2 of the methoxy group was scanned with the sugar in both *C2'endo* and *C3'endo* conformations. In the QM PES the energy patterns along the 2′‐OMe torsion are similar in both puckers (Fig. 5d), with the main minimum around 170°, corresponding to *O3′* (CM2 directed towards O3′); the second minimum was around 85° (*Base*) and ∼1.5 kcal/mol higher, and the third minimum, which is 4 to 5 kcal/mol higher in energy, was around 300° (*O4′*). The first two conformations can also be denoted *g+* and *g‐* (defining the torsion as H2′‐C2′‐O2′‐CM2), respectively. The barrier across O4′ was ∼3 kcal/mol higher in *C3'endo* than in *C2'endo*.

In simulations of the four canonical 2'O‐Me nucleosides Am, Cm, Gm, and Um, the 2′‐OMe torsion mostly sampled the *Base* orientation in pyrimidines, and alternatively as *Base* and *O3′* in purines (Fig. 6f). This differs slightly from the gas‐phase PES, where *O3′* was the minimum, due to the solvent effect decreasing the charge repulsion between O2′ and O3′ when the 2′‐OMe torsion is in *Base*. Importantly, *Base* was highly correlated to the *C3'endo* pucker, and *O3′* was related to *C2'endo*, with pyrimidines having stronger correlation than purines (Figs. 7c and 7d and Supporting Information Figs. S3e and S3f), which were not seen in 2′‐OH nucleosides. Also the *C3'endo* population was higher in pyrimidines than in purines. This agrees with experiments in which 2′‐OMe pyrimidines increased the *north* pucker^102^, ^103^ and stabilized the base stacking and A‐form RNA architectures.^103^, ^104^, ^105^ Early NMR data demonstrated that Am preferred *C2'endo* puckering and 2′‐OMe caused reduced stacking of adenosine.^105^, ^106^, ^107^ In the present work, although *C3'endo* sampling in the 2′‐OMe purine also increased, *C2'endo* was still dominant; but the *C3'endo* enhancement in 2′‐OMe pyrimidines, especially uridine, was more prominent (Figs. 6d and 6e). The difference between pyrimidine and purine is that O2 in pyrimidines is closer to the 2′*‐O‐*methyl compared with the N3 in purines, and *Base* together with *C2'endo* would cause larger steric repulsion.

#### Effects of 2′‐O‐methylation in trinucleotides

Methylation effects on dinucleotides have been reported in several NMR studies,^103^, ^104^, ^105^, ^106^, ^107^ and analysis of base stacking using dinucleotides was also previously performed using simulations, primarily using umbrella sampling.^108^, ^109^ In MD simulations of 100 ns we found that the dinucleotides were less ordered due to thermal motion, so the effects of the modification were not evident (data not shown). Simulations were then undertaken on trinucleotide models, with the target 2′‐OMe nucleotide located in the central position between two adenosines with standard ribose sugars. Thus, eight trinucleotides (the four canonical bases with either 2′‐OH or 2′‐OMe riboses) were simulated and the distributions of sugar pucker, glycosidic torsion, and base stacking were computed. Consistent with the base stacking stability in dinucleotides,^109^ a central purine contributed more to base stacking than pyrimidine in the eight trinucleotides. Furthermore, guanine stacked better than adenine and cytosine better than uracil (Table 5). As discussed above, *C3'endo* of the first two nucleotides increased significantly while the third nucleotide was primarily *C2'endo*.

**Table 5:** Comparison of the 2′*‐O‐*methyl and 2′‐OH nucleotide sugar pucker and stacking populations in trinucleotides 5′‐ApXpA‐3′.

Sequence^a^	*North* (%)^a^		Stacking (%)^a^
5'‐A‐Am‐A‐3' (5'‐A‐A‐A‐3')	1	97.3 (92.6)	62.7^b^ (50.9)
	2	97.5 (72.2)	50.9^c^ (32.8)
	3	7.5 (13.7)	
5'‐A‐Cm‐A‐3' (5'‐A‐C‐A‐3')	1	87.2 (98.9)	75.7 (77.9)
	2	99.9 (94.8)	35.3 (33.0)
	3	18.3 (14.0)	
5'‐A‐Gm‐A‐3' (5'‐A‐G‐A‐3')	1	95.3 (85.5)	79.3 (73.3)
	2	100 (99.4)	80.0 (72.0)
	3	10.5 (23.5)	
5'‐A‐Um‐A‐3' (5'‐A‐U‐A‐3')	1	92.8 (97.8)	51.1 (58.5)
	2	96.3 (85.3)	25.1 (21.8)
	3	16.1 (12.8)	

a The data shown in parenthesis are for canonical trinucleotide sequences.

b Stacking of first and second base.

c Stacking of second and third base.

The four central 2′‐OMe nucleotides increased their *C3'endo* puckers (close to 100%), compared with the corresponding 2′‐OH nucleotides, but the effects on their neighbors were less significant, and also different between purines and pyrimidines. For the 3′ A following a 2′‐OMe pyrimidine *C3'endo* increased while after a purine some amount of *C3'endo* was lost. In contrast, the 5′ A preceding a 2′‐OMe purine increased *C3'endo*, while the 5'A preceding a pyrimidine decreased *C3'endo* puckering. Furthermore, stacking on both sides was increased with a central 2′‐OMe purine, but for pyrimidine only stacking with the 3′ A was increased and stacking with the 5′ A even decreased. Stabilization of the 3′ neighbor is expected since the *C3'endo* enhancement for the 3′ nucleotide was reported in dinucleotides containing Cm,^104^, ^105^ Um,^103^, ^105^ and Gm,^105^ although the destabilization by Am^106^, ^107^ was not observed. The slight destabilization of the 5′ A pucker and stacking with a 2′‐OMe pyrimidine has not been previously reported, and there is no experimental evidence that 2′‐OMe affects the 5′ neighbor. Considering that the stabilizing and destabilizing effects brought by 2′‐OMe were quite limited, the influence of 2′‐OMe should be much less than that of D and *Ψ*. Thus, in short and single strands, thermal motion might mask its effect. Also structural stability is sequence dependent. For example, the 3′ A following Am and Gm had less *C3'endo* but better stacking, which indicates that the base had a bigger influence than the ribose. We have not been able to further elucidate a detailed mechanism based on the current models.

The 2′‐OMe torsion in oligonucleotides is only compatible with the *Base* conformation, because of the steric repulsion of the bulky methyl group with the phosphodiester backbone. As occurs in nucleosides, *Base* was correlated with *C3'endo* pucker, hence a 2′‐OMe nucleotide prefers *north* pucker in oligonucleotides more than a ribose nucleotide, whose 2′‐OH torsion is able to rotate to induce both A‐form (*Base*) and noncanonical (*O3′*) structures in oligonucleotides.^110^ However, QM calculations reported that hydrogen bonding of a 2′‐OH to water stabilized the *Base* orientation as well as the *C3'endo* conformation of the sugar, analogous to what occurs with a 2′‐OMe.^111^ This agrees with the experimental^100^, ^112^ and simulation^113^ observations of RNA duplexes indicating that the 2′‐OMe does not change the conformation of the oligonucleotide, but stabilizes the overall structure of the RNA. This was suggested to be due to stabilization of the hydration pattern in the minor groove (pyrimidine is more affected than purine). More recently, the stabilizing effect of 2′‐OMe was indicated to be due to the inability of the 2′‐OMe to hydrogen bond with the O3′ phosphate moiety due to steric restrictions, leading to intrinsic stabilization of the A conformation of the phosphodiester backbone, thereby contributing to overall stabilization of the RNA.^110^

#### N4‐acetylcytidine (ac^4^C)

N4‐acetylcytidine is found in the wobble position of the *E. coli* elongator ${\text{tRNA}}_{m}^{\text{met}}$ anticodon, which matches only one codon (AUG) and prevents misreading of AUA, an isoleucine codon, in contrast to unmodified cytidine which reads both AUG and AUA.^114^ Interestingly, this is one of only two cases where a purine in the first codon position is specific for one amino acid; the other one is the Hirsh suppressor.^12^ In ac^4^C the substituted acetyl group shares the delocalized electrons with the C4‐N4 bond and is conjugated to the pyrimidine ring, so the acetyl group is coplanar with the ring. The crystal structure reported the 4‐acetyl group to be in a *trans* orientation to N3^115^ (or “proximal to C5”). This is reasonable because in this conformation ac^4^C can form a WC base pair with a G. Our PES of the torsion N3‐C4‐N4‐C7 in vacuum (Supporting Information SDD 2.28) and a previous QM calculation using density functional theory^116^ agree with this observation. The energy difference in PES between *cis* and *trans* was ∼10 kcal/mol. However, this difference had to be increased to >14 kcal/mol in order to obtain *trans* in solution simulation, indicative of a significant solvation effect. Conformations where O7 and N3 would not come close in vacuum were stabilized in aqueous solution by a water bridge. At the same time, the reduced electrostatic attraction between O7 and H5 further counteracts the *trans* preference. In short, the energy barrier for interconversion between *trans* and *cis* is lowered in solution. This effect is similar to the 2′‐OH orientation in NA36,^40^ and *Base*/*O3′* energy minimum in 2′‐OMe. We have not found any published data that would allow an accurate determination of the force constant of this torsion. As a general strategy, we made the sampling of both conformations almost equal (Fig. 6g) which will not significantly disturb the ac^4^C:G base pair in oligonucleotide simulations, but for a nucleoside there would be more freedom to respond to the environment.

Another important structural feature of ac4C is the *C3'endo* pucker stabilization. According to NMR data, the N4‐acetyl group withdraws electrons from C5 and C6, thereby deshielding the H5 and H6 protons.^102^ This effect enhances the interaction between O4' and H6 and is believed to induce a low‐*anti χ* torsion which correlates with *C3'endo* puckering, similar to the correlation found in *Ψ*. To reproduce the *C3'endo* enhancement, the optimized atomic charges were manually adjusted by ±0.03 to 0.04 electrons to emphasize the electron‐withdrawing influence. The adjustment worsened the water‐interaction results somewhat, but obviously a compromise between charge and conformation is required. Furthermore, since this *C3'endo* enhancement was caused by the base substitution using a different mechanism than the enhancement by 2'‐OMe, an additive *C3'endo* improvement should be true for O2'‐methyl‐N4‐acetylcytidine (ac^4^Cm, MAC). This is observed in NMR experiment^102^ and also reproduced in this study (Fig. 6e).

#### N‐methyl cytidines, adenosines, and guanosines

In contrast to ac^4^C, the C4‐N4 torsion in N4‐methylcytidine (m^4^C) showed a 20:1 preference of being *cis* to N3 in solution NMR (corresponding to an energy difference of ∼2 kcal/mol), and the activation enthalpies of converting from *cis* to *trans* were 11 to 18 kcal/mol.^117^, ^118^ These features were also observed in the vacuum PES of torsion N3‐C4‐N4‐C7 in m^4^C where the energy barrier is higher than 12 kcal/mol and the difference between the two minima is 1.5 kcal/mol (Fig. 68 in Supporting Information SDD 2.9). Note that although the rotation of C4‐N4 bond in the two directions is equivalent, the barriers in the figure are different, as in the QM scan the sp^2^ N4 distorts during rotation out‐of‐plane and assumes a pyramidal configuration; thus the orientation of H4 influences the energy, which is lower when H4 is directed toward N3 and higher when H4 points toward C5. This artifact was also found in similar exocyclic *N*‐methylations, for example N2‐methylguanine (m^2^G, Supporting Information SDD 2.26) and N6‐methyladenine (m^6^A, Supporting Information SDD 2.32). The conformational distribution from the m^4^C nucleoside simulation was consistent with the PES prediction (Fig. 6g). This conformation was mainly sampled in *cis*, and although m^4^C may thus potentially lose WC base pairing, in oligonucleotides its influence is actually small. In solution NMR, m^4^C destabilized the m^4^C:G base pair by 1.0 to 1.8 kcal/mol in a RNA dimer.^118^ A decreased m^4^C:G pairing was also found in a DNA hexamer at 19°C,^119^ but in a crystal structure of Z‐DNA, m^4^C base paired with G just as C does.^120^ Furthermore, in an RNA duplex it was even found to stabilize the structure where m^4^C was in *trans* and paired with G.^121^ Obviously the effect of the 4‐methyl group depends on the environment. In a nucleoside or short single strand, the intrinsic torsion energy dominates the conformation of C4‐N4, thereby disturbing the formation of a WC base pair, whereas in a double strand, the hydrogen bond is stabilized by base pairing and stacking, and the contribution of the conjugated N4‐methyl group to stacking even surpasses the torsion disturbance.

This N‐C torsion disturbance occurs in other exocyclic mono *N*‐methyl bases, and the order of influence is: m^6^A > m^4^C > m^2^G. This is easily understood from their structures, and the PES of these torsions also show this order (Supporting Information SDD 2.9, 2.32, and 2.26). For m^2^G the minimum was *trans* to N1 which is correct for WC base pairing, and though m^6^A had a similar minimum as m^4^C (Fig. 6g), the A:U/T base pair is weaker than G:C, so that the higher base opening propensity of A:U/T is further enhanced by the presence of the methyl group in m^6^A.

## Conclusions

The present study involved a comprehensive development of empirical force field parameters for the 112 known modified ribonucleotides. The parametrization was performed to be consistent with the philosophy behind the development of the CHARMM additive force field, such that the quality of the parameters will be compatible with the other components in the CHARMM force field. The parameters were optimized targeting QM data and further refined against experimental data when possible. Notable was the optimization of the partial atomic charges based on the reproduction of water‐model compound minimum interaction energies. Starting from the partial atomic charges obtained from the CGenFF program, additional optimization was performed using a least squares fitting approach that always reduced the RMS difference for each model compound. We note that quality of the optimized charges is mainly determined by the initial guess. In this work we started from the CGenFF charges, which were already optimized targeting similar compounds. To further ensure that the optimized charges do not deviate strongly from the CGenFF charges restraints were applied on the charges during the charge optimization. Given the overall improvements obtained in the interactions with water and in the dipole moments, this approach is recommended for fine tuning of charge distributions when extending CGenFF to new species.

Concerning the intramolecular parameters, the dihedral terms in particular were carefully optimized to reproduce reasonable conformational properties based on an extensive set of PES scans. Notable is the quality of the new glycosidic and 2′‐OMe torsions and selected sugar torsions that were fine‐tuned based on MD simulations to achieve good agreement between simulations and experiments. Given the care taken in the force field optimization, in addition to extending the CHARMM force field to modified nucleotides, the parameters developed in this study will be included in the training set of the CGenFF program, significantly expanding the coverage of the force field.

Emphasis was placed on the quality of some of the more common modified nucleotides, including dihydrouridines, pseudouridines, 7‐methylguanosine, and 2′‐OMe nucleotides, whose conformations were shown to be consistent with experimental data. Important properties include that the A form of RNA can be structurally stabilized by *Ψ*, m^7^G and 2'OMe, and destabilized by D. In addition to the previously characterized *χ*/P correlation for canonical nucleic acids,^53^, ^63^, ^64^ new correlations of *anti*/*C3'endo* and *Base*/*C3'endo* were found for *Ψ* and m^7^G and 2'OMe nucleotides, respectively, thereby illustrating how they stabilize RNA structures.

Initial parameters were obtained from NA36, Carb36, and CGenFF, with parameters not previously in the force field assigned values by analogy using the CGenFF program. Subsequently, only parameters that were not previously in the force field were subjected to optimization, with new parameters with low CGenFF penalties not subjected to additional optimization with the exception of selected dihedral parameters. For example, the *χ* or 2'OMe torsion parameters were fine‐tuned in a number of cases. In cases where the parameters of the ribose were identical to canonical nucleic acids, the *χ* torsion and sugar pucker could not be optimized, which indeed provided a challenge. Although we assumed that base modifications would not have a large effect on backbone conformational properties, there are known relationships between base modifications and sugar pucker.^68^, ^69^ For example, electron‐withdrawing 5‐substitutions increase *C3'endo* for uridines whereas electron‐donating 5‐substitutions increase *C2'endo*. In this force field all 5‐substituted uridines use the same *χ* and pucker parameters as U. In this case to improve the sugar pucker small changes in the base charges were made. However, this is risky as base charges were optimized targeting water interaction and changing them significantly would adversely affect the behavior in solution. Thus, compromises were required that were very carefully assessed based on available experimental evidence. An example is ac^4^C whose *C3'endo* population was improved by adjusting the base charges, leading to better agreement with NMR data.^102^ However, experiments on sugar conformation are lacking for many modified nucleic acids, and further refinement of the associated parameters, beyond targeting the QM data, is limited.

The parametrization of base torsions was relatively straight forward due to these terms often being chemically specific for a few molecules. Nevertheless the difficulty here is the elusive solvent effect on torsions involving polar atoms. This led to discrepancies between vacuum QM energies and solution MD simulation conformational sampling in ac^4^C, where without the revision based on simulation, the conformation from the force field would have been opposite to the experimental observation. Therefore if experimental data are available, it is always worth calibrating the associated parameters based on explicit solvent simulations.

## Supporting information

Supporting Information

Supporting Information

Supporting Information
