# Supplementary material for: Additive CHARMM force field for naturally occurring modified ribonucleotides
Source: J Comput Chem. 2016 Feb 3;37(10):896–912. doi: 10.1002/jcc.24307 (PMC4801715; doi:10.1002/jcc.24307)
Supplement: Supplementary file 1 — Supporting Information [file JCC-37-896-s001.pdf]

Supporting information

For

Additive CHARMM force field for naturally occurring modified ribonucleotides

---

You Xu, Kenno Vanommeslaeghe, Alexey Aleksandrov,

Alexander D. MacKerell Jr., Lennart Nilsson

Table S1 Comparison of different nomenclatures of natural nucleic acids, including the CHARMM and AMBER three-letter RESI names and CHARMM PRES name in topology files.

	Symbol	Common name	Three-letter code			CHARMM patch residue
			CHARMM	AMBER	PDB	
	**Adenosines and inosines**					
1	A	Adenosine	ADE			ADEP, ADEI
2	m^{1}A	1-methyladenosine	1MA			1MAN
3	m^{2}A	2-methyladenosine	2MA			
4	m^{6}A	N6-methyladenosine	6MA		6MZ	
5	Am	2'-O-methyladenosine	OMA	MRA	A2M	ADEP, ADEI
6	ms^{2}m^{6}A	2-methylthio-N6-methyladenosine	SMA			
7	i^{6}A	N6-isopentenyladenosine	6IA			
8	ms^{2}i^{6}A	2-methylthio-N6-isopentenyladenosine	MIA	SPA		
9	io^{6}A	N6-(cis-hydroxyisopentenyl)adenosine	HIA			
10	ms^{2}io^{6}A	2-methylthio-N6-(cis-hydroxyisopentenyl) adenosine	SIA			
11	g^{6}A	N6-glycinylcarbamoyladenosine	6GA			6AH
12	t^{6}A	N6-threonylcarbamoyladenosine	T6A	6TA		6AH
13	ms^{2}t^{6}A	2-methylthio-N6-threonyl carbamoyladenosine	12A	STA		6AH
14	m^{6}t^{6}A	N6-methyl-N6-threonylcarbamoyladenosine	66A		AET	6AH
15	hn^{6}A	N6-hydroxynorvalylcarbamoyladenosine	HNA			
16	ms^{2}hn^{6}A	2-methylthio-N6-hydroxynorvalyl carbamoyladenosine	26A			6AH
17	Ar(p)	2'-O-ribosyladenosine(phosphate)	RIA	2RA		
18	m$^{6}_{2}$A	N6,N6-dimethyladenosine	M6A	DMA	MA6	
19	m^{6}Am	N6,2'-O-dimethyladenosine	MMA			
20	m$^{6}_{2}$Am	N6,N6,2'-O-trimethyladenosine	MTA			
21	m1Am	1,2'-O-dimethyladenosine	M2A			1MAN
22	ac^{6}A	N6-acetyladenosine	6AA			
23	m^{8}A	8-methyladenosine	8MA			
24	I	inosine	INO			
25	m^{1}I	1-methylinosine	1MI			
26	m^{1}Im	1,2'-O-dimethylinosine	MMI			
27	Im	2'-O-methylinosine	OMI	MRI		
	**Cytidines**					
28	C	cytidine	CYT			CYTP
29	m^{3}C	3-methylcytidine	3MC			3MCN
30	m^{5}C	5-methylcytidine	5MC			
31	Cm	2'-O-methylcytidine	OMC	MRC		CYTP
32	s^{2}C	2-thiocytidine	2SC			
33	ac^{4}C	N4-acetylcytidine	4AC			
34	f^{5}C	5-formylcytidine	5FC		RSQ	
35	m^{5}Cm	5,2'-O-dimethylcytidine	MMC			
36	ac^{4}Cm	N4-acetyl-2'-O-methylcytidine	MAC			

37	k^2C	lysidine	K2C			5UHA, K2CN, 34HC
38	m^4C	N4-methylcytidine	4MC			
39	m^4Cm	N4,2'-O-dimethylcytidine	4OC	M4C		
40	hm^5C	5-hydroxymethylcytidine	HMC		5HM	
41	f^5Cm	5-formyl-2'-O-methylcytidine	MFC			
42	$m^4{}_2Cm$	N4,N4,2'-O-trimethylcytidine	TMC			
43	C^+	agmatidine	R2C		AG9	K2CN, 34HC
	**Guanosines and 7-deazaguanosines**					
44	G	guanosine	GUA			
45	m^1G	1-methylguanosine	1MG			
46	m^2G	N2-methylguanosine	2MG			
47	m^7G	7-methylguanosine	7MG			
48	Gm	2'-O-methylguanosine	OMG	MRG		
49	$m^2{}_2G$	N2,N2-dimethylguanosine	M2G	DMG		
50	m^2Gm	N2,2'-O-dimethylguanosine	MMG			
51	$m^2{}_2Gm$	N2,N2,2'-O-trimethylguanosine	MTG			
52	Gr(p)	2'-O-ribosylguanosine(phosphate)	RIG	2RG		
53	yW	wybutosine	YYG	WBG	YG	
54	o_2yW	peroxywybutosine	PBG			
55	OHyW	hydroxywybutosine	HWG			
56	OHyW*	undermodified hydroxywybutosine	BUG			
57	imG	wyosine	IMG			
58	mimG	methylwyosine	MWG			
59	$m^{2,7}G$	N2,7-dimethylguanosine	27G			
60	$m^{2,2,7}G$	N2,N2,7-trimethylguanosine	N2G			
61	m^1Gm	1,2'-O-dimethylguanosine	M1G			
62	imG-14	4-demethylwyosine	DWG			
63	imG2	isowyosine	IWG			
64	$m^{2,7}Gm$	N2,7,2'-O-trimethylguanosine	M7G			
65	Q	queuosine	QUG		QUO	7GNM
66	oQ	epoxyqueuosine	EQG			7GNM
67	galQ	β-galactosyl-queuosine	GQG			7GNM
68	manQ	β-mannosyl-queuosine	MQG			7GNM
69	preQ0	7-cyano-7-deazaguanosine	DCG			
70	preQ1	7-aminomethyl-7-deazaguanosine	DAG		PQ1	7GNA
71	G^+	archaeosine	RCG			
	**Uridines and pseudouridines**					
72	U	uridine	URA			
73	Ψ	pseudouridine	PSU			
74	D	dihydrouridine	H2U	DHU		
75	m^5U	5-methyluridine	5MU		38T	
76	Um	2'-O-methyluridine	OMU	MRU		
77	m^5Um	5,2'-O-dimethyluridine	2MU	MMU		

78	$m^{1}\Psi$	1-methylpseudouridine	1MP			
79	Ψm	2'-O-methylpseudouridine	OMP	MRP		
80	$s^{2}U$	2-thiouridine	2SU			
81	$s^{4}U$	4-thiouridine	4SU			
82	$m^{5}s^{2}U$	5-methyl-2-thiouridine	52U			
83	$s^{2}Um$	2-thio-2'-O-methyluridine	MSU			
84	$acp^{3}U$	3-(3-amino-3-carboxypropyl)uridine	3AU			5UHA
85	$ho^{5}U$	5-hydroxyuridine	5HU			
86	$mo^{5}U$	5-methoxyuridine	MOU			
87	$cmo^{5}U$	uridine 5-oxyacetic acid	OAU		CM0	5UHC, ENOU
88	$mcmo^{5}U$	uridine 5-oxyacetic acid methyl ester	OEU			ENOU
89	$chm^{5}U$	5-(carboxyhydroxymethyl)uridine	HCU			5UHC
90	$mchm^{5}U$	5-(carboxyhydroxymethyl)uridine methyl ester	CMU			
91	$mcm^{5}U$	5-methoxycarbonylmethyluridine	OCU			
92	$mcm^{5}Um$	5-methoxycarbonylmethyl-2'-O-methyluridine	MEU			
93	$mcm^{5}s^{2}U$	5-methoxycarbonylmethyl-2-thiouridine	70U	SMU		
94	$nm^{5}s^{2}U$	5-aminomethyl-2-thiouridine	SAU			5UNA
95	$mnm^{5}U$	5-methylaminomethyluridine	5AU			
96	$mnm^{5}s^{2}U$	5-methylaminomethyl-2-thiouridine	U8U	ESU		
97	$mnm^{5}se^{2}U$	5-methylaminomethyl-2-selenouridine	SEU			
98	$ncm^{5}U$	5-carbamoylmethyluridine	BCU			
99	$ncm^{5}Um$	5-carbamoylmethyl-2'-O-methyluridine	MCU			
100	$cmnm^{5}U$	5-carboxymethylaminomethyluridine	5DU			5UHG
101	$cmnm^{5}Um$	5-carboxymethylaminomethyl-2'-O-methyluridine	MAU			5UHG
102	$cmnm^{5}s^{2}U$	5-carboxymethylaminomethyl-2-thiouridine	SCU			5UHG
103	$m^{3}U$	3-methyluridine	3MU		UR3	
104	$m^{1}acp^{3}\Psi$	1-methyl-3-(3-amino-3-carboxypropyl)pseudouridine	13P			5UHA
105	$cm^{5}U$	5-carboxymethyluridine	5CU			5UHC
106	$m^{3}Um$	3,2'-O-dimethyluridine	M3U			
107	$m^{5}D$	5-methyldihydrouridine	MDU	DMU		
108	$m^{3}\Psi$	3-methylpseudouridine	3MP			
109	$tm^{5}U$	5-taurinomethyluridine	5TU		TM2	
110	$tm^{5}s^{2}U$	5-taurinomethyl-2-thiouridine	STU			
111	$inm^{5}U$	5-(isopentenylaminomethyl)uridine	IAU			5UNI
112	$inm^{5}s^{2}U$	5-(isopentenylaminomethyl)-2-thiouridine	ISU			5UNI
113	$inm^{5}Um$	5-(isopentenylaminomethyl)-2'-O-methyluridine	MIU			5UNI
114	$gmnm^{5}s^{2}U$	geranylated 5-methylaminomethyl-2-thiouridine	GAU			
115	$gcmnm^{5}s^{2}U$	geranylated 5-carboxymethylaminomethyl-2-thiouridine	GCU			5UHG
116	$cnm^{5}U$	5-cyanomethyl-uridine	CYU			

Figures of supporting information

Figure Captions

Figure S1. The potential energy surface scans of χ torsion for four canonical nucleosides

Figure S2. Conformational distributions of canonical **ribo-nucleosides**. The simulations using CGenFF (CG36) were compared with the same molecule using NA36.

Figure S3. Conformational distributions of canonical **deoxyribo-nucleosides**. The simulations using CGenFF (CG36) were compared with the same molecule using NA36.

Figure S4. Correlations between χ or 2'OMe torsions and sugar pucker for nucleosides in MD simulations.

Figure S5. Structural destabilization and stabilization **related to the geometry of base stacking,** caused by H2U (D) and PSU (Ψ) in trinucleotides 5'-ApDpA-3' and 5'-ApΨpA-3' compared with 5'-ApUpA-3'.

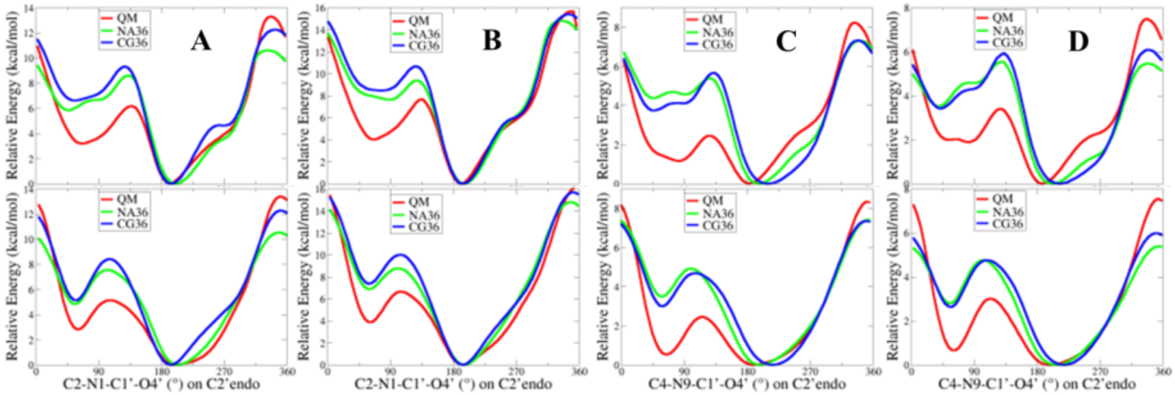

Figure S1. The potential energy surface scans of χ torsion for four canonical nucleosides: (A) Uridine, (B) Cytidine, (C) Adenosine and (D) Guanosine, with the furanose restricted to the *C3’endo* (top) and *C2’endo* (bottom) conformations. Empirical is presented for the CHARMM36 RNA force field (NA36) and CGenFF (CG36).

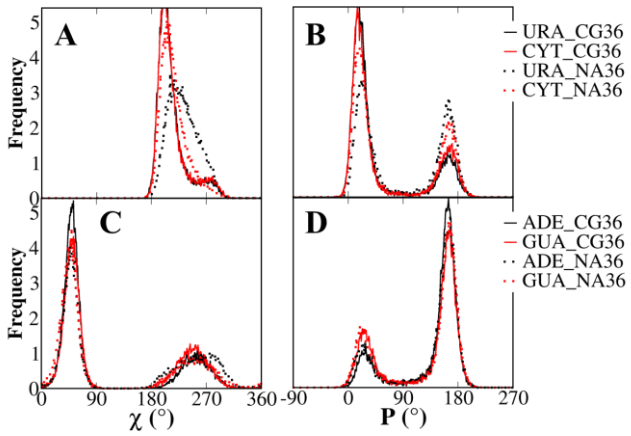

Figure S2. χ torsion (A,C) and sugar pucker phase (B,D) distributions of canonical pyrimidine (A,B) and purine (C,D) ribonucleosides from simulations using CG36 and NA36.

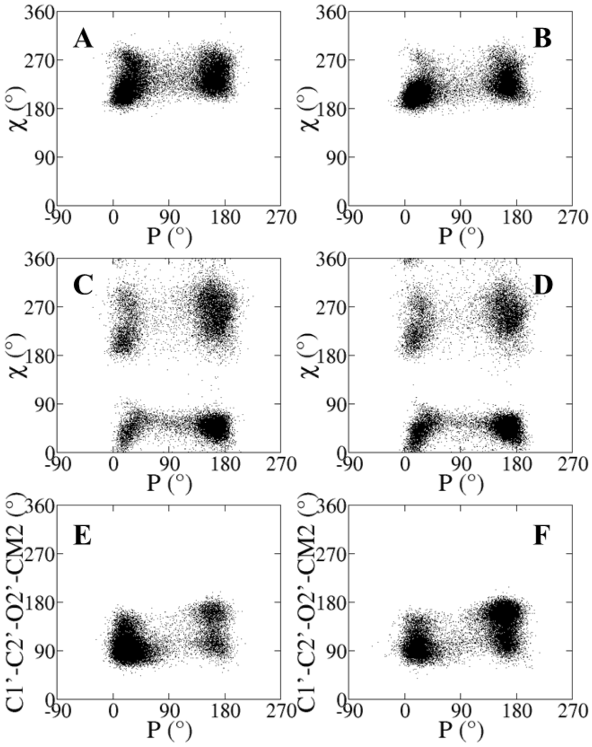

Figure S3. Correlations between χ or 2'OMe torsions and sugar pucker for nucleosides in MD simulations, including the χ/P correlation of (A) uridine, (B) cytidine, (C) adenosine and (D) guanosine; and the 2'OMe/P correlation of (E) 2'-O-methyluridine and (F) 2'-O-methyladenosine. No correlation was observed between χ=*anti* and P=*north* for 2'OH nucleosides, weak correlation was present for 2'OMe adenosine, and strong correlation was present for 2'OMe uridine.

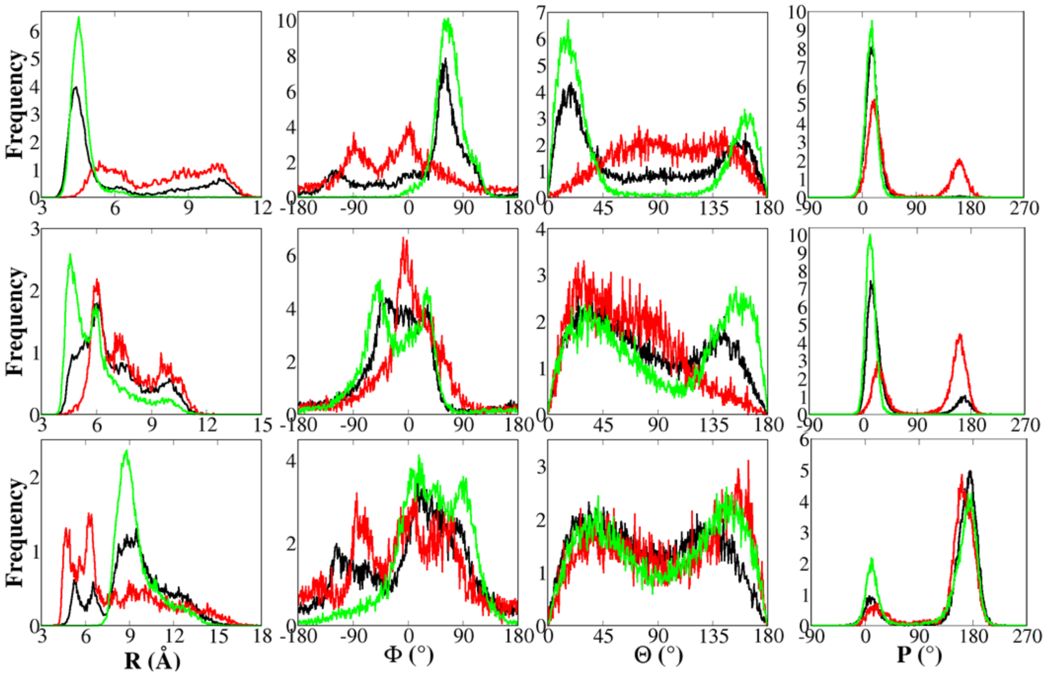

Figure S4. Structural destabilization and stabilization related to the geometry of base stacking, caused by H2U (D) and PSU (Ψ) in trinucleotides 5'-ApDpA-3' and 5'-ApΨpA-3' compared with 5'-ApUpA-3'. From left to right the first three columns are geometric descriptors for base stacking: i.e. 1) distance between N1/N9; 2) pseudo torsion of the in-plane axes of two bases; 3) angle between normal vectors of two bases (see 'Conformational definitions and analyses' in main text), and the fourth column is pseudorotation of sugar pucker. From top to bottom the three rows of 1st – 3rd columns are base-base geometries between 1) 5' base and middle base, 2) middle base and 3' base and 3) 5' base and 3' base, and of 4th column are puckers of 5' nucleoside, middle nucleoside and 3' nucleoside. Color scheme is: ApUpA in black, ApDpA in red and ApΨpA in green.
