# Supplementary material for: Additive CHARMM force field for naturally occurring modified ribonucleotides
Source: J Comput Chem. 2016 Feb 3;37(10):896–912. doi: 10.1002/jcc.24307 (PMC4801715; doi:10.1002/jcc.24307)

The Supporting Data Document of the manuscript
"Additive CHARMM force field for naturally occurring modified ribonucleotides"

You Xu, Kenno Vanommeslaeghe, Alexey Aleksandrov,
Alexander D. MacKerell Jr. and Lennart Nilsson

July 2015

Contents

1 The Detail of Water-Compound Complexes Which Involved in Charge Optimization **7**

1.1 1-methyladenine (1MAn) . . . 7
1.2 1-methyladenine protonated (1MA) . . . 9
1.3 1-methylguanine (1MG) . . . 11
1.4 1-methylinosine (1MI) . . . 13
1.5 1-methylpseudouracil (1MP) . . . 15
1.6 N2,7-dimethylguanine (27G) . . . 17
1.7 2-methyladenine (2MA) . . . 19
1.8 N2-methylguanine (2MG) . . . 21
1.9 2-thiocytosine (2SC) . . . 23
1.10 2-thiouracil (2SU) . . . 25
1.11 2-methylamino-4-amino-pyrimidine, protonated (34hc, for K2C, R2C) . . . 27
1.12 3-methylcytosine (3MCn) . . . 29
1.13 3-methylcytosine protonated (3MC) . . . 31
1.14 3-methylpseudouracil (3MP) . . . 33
1.15 3-methyluracil (3MU) . . . 35
1.16 N4-acetylcytosine (4AC) . . . 37
1.17 N4-methylcytosine (4MC) . . . 39
1.18 4-thiouracil (4SU) . . . 41
1.19 5-formylcytosine (5FC) . . . 43
1.20 5-hydroxyuracil (5HU) . . . 45
1.21 5-methylcytosine (5MC) . . . 47
1.22 N6-acetyladenine (6AA) . . . 49
1.23 N6-methyladenine (6MA) . . . 51
1.24 7-methylguanine (7MG) . . . 53
1.25 zwitterionic alanine (alai, for 3AU, 13P) . . . 55
1.26 3-ammonio-cyclopentene (cpe, for QUG, MQ, GQG) . . . 57
1.27 3-ammonio-epoxycyclopentane (cpo, for EQG) . . . 59
1.28 7-cyano-7-deazaguanine (DCG) . . . 61
1.29 dihydrouracil (H2U) . . . 63
1.30 dimethylammonium (dmam, for 5AU, U8U, IAU, ISU etc.) . . . 65
1.31 N1,N2-dimethyl-N1-phenylurea (dmpu, for 66A) . . . 67
1.32 7-deazaguanine (7dng, for RCG, DAG, QUG etc.) . . . 69
1.33 4-demethylwyosine (DWG) . . . 71
1.34 5-methoxyuracil, enol form (enou) . . . 73
1.35 wyosine (IMG) . . . 75
1.36 inosine (INO) . . . 77
1.37 N2,N2-dimethylguanine (M2G) . . . 79
1.38 N6,N6-dimethyladenine (M6A) . . . 81
1.39 methyl hydrogen peroxide (mhpo, for PBG) . . . 83
1.40 7-amidinio-7-deazapurine (mip, for RCG) . . . 85
1.41 5-methoxyuracil (MOU) . . . 87

1.42 N2-methyl-N1-phenylurea (mpu, for HNA, 26A, T6A, 12A, 6GA) 89
1.43 2-methylthiouracil (2msu, for GAU, GCU) 91
1.44 N2,N2,7-trimethylguanine (N2G) 93
1.45 2-methylamino-4-amino-pyrimidine (ncy, for K2Cn) 95
1.46 2-methylamino-4-amino-pyrimidine, protonated (ncyp, for K2C) 97
1.47 carboxymethyl-methyl-ammonium (nmg, for 5DU, MAU, SCU) 99
1.48 1H-pseudoisocytosine (1PC) 101
1.49 3H-pseudoisocytosine (3PC) 103
1.50 Pseudouracil (PSU) 105
1.51 Adenine protonated (ADEp) 107
1.52 Cytosine protonated (CYTp) 109
1.53 2-methylthio-N6-methyladenine (SMA) 111
1.54 N4,N4,2'-O-trimethylcytosine (TMC) 113
1.55 1,3,5-trimethyl-pseudouracil (mdmp, for 13P) 115
1.56 N1,N1,N2-trimethylurea (tmu, for 66A) 117

2 The Geometric Information of Modified Bases 119

2.1 1-methyl guanine (1MG) 120
2.2 1-methyl inosine (1MI) 122
2.3 1H-pseudoisocytosine (1PC) 124
2.4 2-methyladenine (2MA) 126
2.5 2-thiouracil (2SU) 128
2.6 3-methylcytosine (3MCn) 130
2.7 3-methylcytosine protonated (3MC) 132
2.8 3H-pseudoisocytosine (3PC) 134
2.9 N4-methylcytosine (4MC) 136
2.10 4-thiouracil (4SU) 138
2.11 5-methyl-2-thiouracil (52U) 140
2.12 5-formylcytosine (5FC) 142
2.13 5-hydroxyuracil (5HU) 144
2.14 7-methylguanine (7MG) 146
2.15 8-methyladenine (8MA) 148
2.16 7-cyano-7-deaza guanine (DCG) 150
2.17 dihydrouracil (H2U) 152
2.18 N6,N6-dimethyl adenine (M6A) 155
2.19 4-demethylwyosine (DWG) 158
2.20 5-hydroxymethyl cytosine (HMC) 161
2.21 methylwyosine (MWG) 163
2.22 5-methyoxyuracil (MOU) 167
2.23 adenine, protonated (ADEp) 169
2.24 cytosine, protonated (CYTp) 171
2.25 5-aminomethyl-2-thiouracil (SAU) 173
2.26 N2-methylguanine (2MG) 175
2.27 2-thiocytosine (2SC) 176
2.28 N4-acetylcytosine (4AC) 177

2.29 5-methylaminomethyl uracil (5AU) 178
2.30 5-carboxymethyl uracil (5CU) 179
2.31 5-methylcytosine (5MC) 180
2.32 6-methyladenine (6MA) 181
2.33 N2,N2-dimethyl guanine (M2G) 182
2.34 5-methyldihydro uracil (MDU) 183
2.35 N4,N4-dimethylcytosine (TMC) 184

3 The Geometric Information of Base Scaffolds and Substitutent Side Chains 185
3.1 2-methylthio-adenine (2msa, for SMA, MIA, SIA, etc.) 186
3.2 7-deazapurin-7-yl-methyl ammonium (ampu, for DAG) 189
3.3 N-(6-purinyl)-N-methyl acetamide (m6pa, for 66A) 192
3.4 7-deazapurin-7-yl-methyl amidinium (7mip, for RCG) 196
3.5 2-methylamino-4-imino pyrimidine, protonated (ncyp, for K2C & R2C) 199
3.6 3-methylbuten-1-yl methyl ammonium (nmba, for IAU, ISU & MIU) 201
3.7 1,5-hexadiene (15he, for GAU, GCU) 204
3.8 N3-propyluracil (3pru, for 3AU) 205
3.9 2-carboxamide-3-methoxy butyraldehyde (amba, for PBG) 206
3.10 2-hydroxyl-3-amino butane (aboh, for BUG) 207
3.11 carbomoylbenzene (acbz, for BCU) 208
3.12 phenylacetate (bzac, for 5CU) 209
3.13 phenylammonium (bzam, for SAU) 210
3.14 phenylacetic acid (bzaa, for 5CU) 211
3.15 2-phenyl-2-hydroxylacetate (bzha, for HCU) 212
3.16 1-hydroxylethyl benzene (bzhe, for CMU) 213
3.17 methoxybenzene (bzmo, for MOU) 214
3.18 N1-(3-pyridinyl)-N1,N2-dimethylurea (pmmu, for 66A) 215
3.19 1-(2-pyridinyl)amino-3-methyl butene (bepa, for 6IA, HIA, MIA & SIA) 216
3.20 1-(3-pyrrolyl)-N-(3-cyclopentenyl)-methyl amine (cena, for QUG, MQG & GQG) 218
3.21 carboxyhydroxymethyl benzene (cmbz, for CMU) 220
3.22 2-oxo-5-formylpyrimidine (5fop, for 5FC) 221
3.23 3-methylamino-1,2-epoxycyclopentene (cona, for EQG) 222
3.24 5-hydroxyl-cyclopenten-3-yl ammonium (cpea, for QUG, MQG & GQG) 223
3.25 epoxycyclopenten-3-yl ammonium (cpoa, for EQG) 224
3.26 cyanomethylbenzene (cybz, for CYU) 225
3.27 N-methyl phenylethylamine (ambz, for 5AU, U8U, 5DU, SCU, etc.) 226
3.28 3-methylamino-propene (penm, for IAU, ISU, etc) 227
3.29 5-methoxyuracil, enol form (enou, for 5-suburidines) 228
3.30 1-(3-cyclopentenoxy) tetrahydropyran (pepr, for QUG, MQG & GQG) 229
3.31 2-hydroxyl propionate methylester (hpme, for CMU) 231
3.32 2-hydroxyl propanoate (hpat, for HCU) 232
3.33 1-hydroxyl-2-methyl butene (hmbt, for HIA & SIA) 233
3.34 3-aminomethyl indole (idam, for DAG) 234
3.35 N1-(3-pyridinyl)-N2-methylurea (mpyu, for HNA, 26A, T6A, 12A & 6GA) . . . 235
3.36 phenylacetate methylester (mebz, for OCU & MEU) 236

3.37 2-methylamino ethyl sulfate (maes, for 5TU & STU) 237
3.38 N1,N2-dimethylurea (12mu, for HNA, 26A, T6A, 12A & 6GA) 239
3.39 methoxy acetate (moat, for OAU) 240
3.40 methoxyacetate methyl ester (moae, for OEU) 241
3.41 N1-(4-pyrimidinyl)-N2-methylurea (pymu, for HNA, 26A, T6A, 12A & 6GA) . . 242
3.42 2-methylamino-4-imino-pyrimidine (ncy, for K2C & R2C) 243
3.43 carboxymethyl methyl ammonium (nmgi, for 5DU, SCU & MAU) 244
3.44 methylamino acetic acid (nmgn, for 5DU, SCU & MAU) 245
3.45 phenoxyacetate (atbz, for OAU) 246
3.46 methoxyacetic acid (moac, for OAU) 247
3.47 phenoxyacetate methylester (oebz, for OEU) 248
3.48 1-(3-pyrrolyl)prop-2-yl hydrogen peroxide (ppox, for PBG) 249
3.49 2-(propene-3-yl)thiouracil (pesu, for GAU, GCU) 250
3.50 3-(2-hydroxylpropyl)indolizine (hpiz, for HWG) 251
3.51 benzyl methyl ammonium (bzma, for 5AU, U8U, 5DU, SCU, IAU, ISU, etc.) . . 253
3.52 2-propylamino-4-imino-pyrimidine (prnc, for K2C & R2C) 255
3.53 2-propylamino-4-imino-pyrimidine, protonated (pncp, for K2C & R2C) 257
3.54 3-methylamino epoxypentene (ponm, for EQG) 259
3.55 1-(3-pyrrolyl)-N-(3-cyclopentenyl) methylammonium (pnpa, for QUG, MQG & GQG) 260
3.56 tetrahydrofuran-1-oxy-cyclopentane (rbrb, for RIA & RIG) 262
3.57 N1,N1,N2-trimethylurea (mmmu, for HNA, 26A, T6A, 12A & 6GA) 264
3.58 1-(tetrahydrofuran-2-yl) dihydrouracil (dfu, for chi of H2U) 265
3.59 1-(1-imidazolyl)-2-methoxyl-3-hydroxyl tetrahydrofuran (ifu, for phi of 2'-OMe) 267
3.60 1-(tetrahydrofuran-2-yl)-7-methylguanine (mgf, for chi of 7MG) 268
3.61 1-(tetrahydrofuran-2-yl)-2-methylthialcytosine (pcf, for chi of K2C, R2C) 270
3.62 1-(tetrahydrofuran-2-yl) pseudouracil (pfu, for chi of PSU) 272

Abbreviations of assignment mode used in the tables of vibrational analysis

Bond and angle

s: bond stretching, with ss for methylene/methyl/ammonia symmetrical stretching and sa for asymmetrical stretching;
d: angle deformation, with sd for methyl/ammonia symmetrical deformation and ad for asymmetrical deformation;
c: methylene/amino scissoring;
r: methyl/methylene/amino/imino rocking,
i: methylene twisting;
w: methylene/amino/imino wagging.
tXXXX: torsion of X atoms.

Ring correlation

t5r, t5r': torsion of 5-membered ring;
d5r, d5r': deformation of 5-membered ring;
at6r, at6r': asymmetrical torsion of 6-membered ring;
pk6r: pucker of 6-membered ring;
td6r: trigonal deformation of 6-membered ring;
ad6r, ad6r': asymmetrical deformation of 6-membered ring;
btfl: butterfly (junctions of two fused rings)
The number after heavy atoms, e.g. N2, C2, C10, is the atom numbering, while the number after hydrogen, e.g. NH2, CH3, is the number of hydrogen attached to this group.

Good to know for the plots of potential energy surface (PES)

One may notice the abrupt peaks happen in some plots of PES scan in section 2 and 3. It is the issue with relaxed dihedral scans, i.e. during the scan in both QM and MM only the target dihedral is constrained. Thus when the target torsion rotated to some conformations, some other torsion suddenly flips to release the high energy of the strained conformation. However this does not really affect the result of parameters. This phenomenon can be eliminated by carefully constraining additional torsions in some cases, but this is not really feasible in a project of this magnitude.

1 The Detail of Water-Compound Complexes Which Involved in Charge Optimization

1.1 1-methyladenine (1MAn)

Figure 1: The molecule used for water complex calculations corresponding to 1-methyladenine (1MAn), with possible interacting water positions. NOTE, only one water molecule was included in each calculation

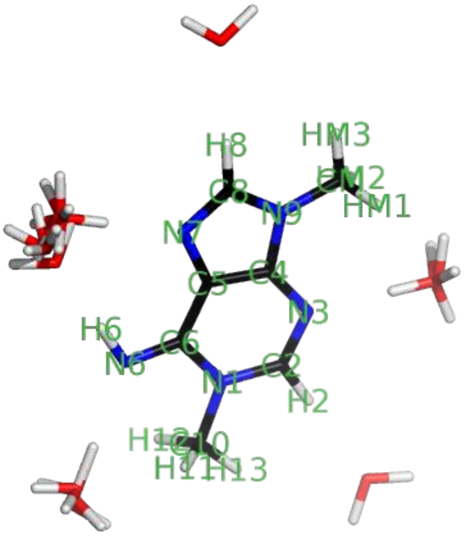

Table 1: Statistics of calculated water interaction and dipole moment for 1-methyladenine (1MAn). QM is in HF level except for thio-compounds.

	RMS/Max Deviation from QM		Dipole moment	Dipole angle
	Energy (kcal/mol)	Distance (Å)	QM/MM (debye)	difference (°)
Original	2.32/4.38	0.23/0.48	4.44/2.55	50.62
Optimized	0.31/0.65	0.11/0.30	4.44/5.01	0.34

Table 2: The comparison list of optimized atomic charges and their initial guess for 1-methyladenine (1MAn), referring to the penalties of initial guess

	Charges		
Atom	Optima	Initial	Penalty
N9	0.002	-0.047	0.000
C8	0.419	0.339	0.000
H8	0.059	0.123	0.000
N7	-0.789	-0.734	9.911
C5	0.278	0.253	19.806
C6	0.271	0.316	136.981
N6	-0.827	-0.541	135.123
H6	0.374	0.378	20.206
N1	-0.381	-0.358	29.152
C2	0.558	0.540	17.968
H2	0.093	0.103	13.393
N3	-0.826	-0.813	5.825
C4	0.534	0.445	8.403
C10	-0.035	-0.274	5.752
H11	0.090	0.090	0.035
H12	0.090	0.090	0.035
H13	0.090	0.090	0.035
CM	-0.270	-0.270	0.000
HM1	0.090	0.090	0.000
HM2	0.090	0.090	0.000
HM3	0.090	0.090	0.000

Table 3: Interaction energies and geometries between probe water and selected 1-methyladenine (1MAn) site calculated using the optimized and initial charges

			QM / Optima / Initial	
N	Probe site	Angle (°)	Energy (kcal/mol)	Distance (Å)
1	H6N6	0	-1.14/-0.56/-4.75	1.94/1.93/1.83
2	H6N6	60	1.22/0.86/-3.17	2.34/2.04/1.89
3	H6N6	120	1.22/0.86/-3.17	2.34/2.04/1.89
4	H2C2	0	-3.67/-3.70/-2.81	2.39/2.38/2.39
5	H8C8	0	-3.09/-3.20/-3.98	2.40/2.26/2.21
6	N6C6	0	-7.18/-7.07/-2.87	2.01/2.20/2.50
7	N6C6	60	-6.43/-6.41/-2.89	2.09/2.21/2.49
8	N6C6	120	-4.34/-4.32/-2.84	2.26/2.27/2.49
9	N6C6	180	-2.87/-2.78/-2.89	2.40/2.35/2.46
10	N6C6	240	-4.34/-4.31/-2.84	2.26/2.27/2.49
11	N6C6	300	-6.43/-6.42/-2.89	2.09/2.21/2.49
12	N3C4	0	-6.29/-5.70/-6.25	1.94/1.96/1.96
13	N3C4	120	-5.67/-5.44/-6.12	1.95/1.96/1.95
14	N3C4	180	-4.29/-4.94/-5.77	2.02/1.97/1.96
15	N3C4	240	-5.67/-5.43/-6.12	1.95/1.96/1.95
16	N7C5	0	-5.94/-6.29/-6.61	1.92/1.89/1.88
17	N7C5	60	-6.92/-6.68/-6.58	1.88/1.88/1.88
18	N7C5	120	-7.05/-6.96/-5.53	1.89/1.89/1.90
19	N7C5	180	-5.94/-6.49/-3.98	1.98/1.92/1.95
20	N7C5	240	-7.05/-6.96/-5.53	1.89/1.89/1.90
21	N7C5	300	-6.92/-6.69/-6.58	1.88/1.88/1.88

1.2 1-methyladenine protonated (1MA)

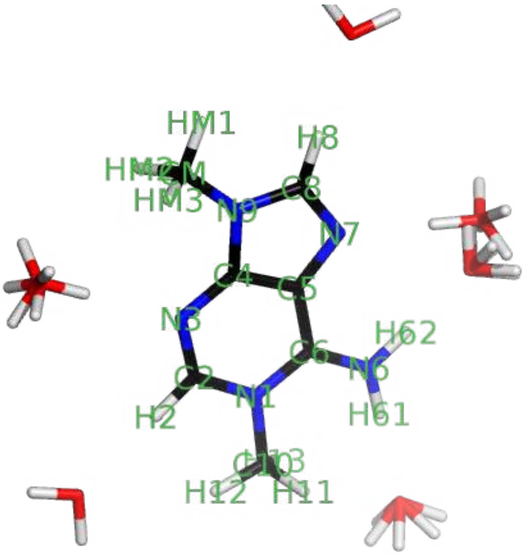

Figure 2: The molecule used for water complex calculations corresponding to 1-methyladenine protonated (1MA), with possible interacting water positions. NOTE, only one water molecule was included in each calculation

Table 4: Statistics of calculated water interaction and dipole moment for 1-methyladenine protonated (1MA). QM is in HF level except for thio-compounds.

	RMS/Max Deviation from QM		Dipole moment	Dipole angle
	Energy (kcal/mol)	Distance (Å)	QM/MM (debye)	difference (°)
Original	1.97/3.00	0.13/0.23	3.92/8.02	26.64
Optimized	0.35/0.66	0.08/0.13	3.92/5.55	8.76

Table 5: The comparison list of optimized atomic charges and their initial guess for 1-methyladenine protonated (1MA), referring to the penalties of initial guess

	Charges		
Atom	Optima	Initial	Penalty
N9	0.010	-0.047	0.000
C8	0.347	0.339	0.000
H8	0.137	0.123	0.000
N7	-0.668	-0.734	13.119
C5	0.097	0.258	27.678
C6	0.615	1.277	201.291
N6	-0.808	-1.127	201.247
H61	0.407	0.460	20.716
H62	0.401	0.460	20.716
N1	-0.139	-0.436	79.504!
C2	0.283	0.468	24.904
H2	0.169	0.101	16.693
N3	-0.607	-0.813	9.018
C4	0.545	0.445	11.182
C10	-0.059	-0.044	75.230
H11	0.090	0.090	3.767
H12	0.090	0.090	3.767
H13	0.090	0.090	3.767
CM	-0.270	-0.270	0.000
HM1	0.090	0.090	0.000
HM2	0.090	0.090	0.000
HM3	0.090	0.090	0.000

Table 6: Interaction energies and geometries between probe water and selected 1-methyladenine protonated (1MA) site calculated using the optimized and initial charges

			QM / Optima / Initial	
N	Probe site	Angle (°)	Energy (kcal/mol)	Distance (Å)
1	H61N6	0	-11.32/-11.36/-12.74	1.97/2.02/1.97
2	H61N6	60	-12.01/-11.99/-13.35	1.96/1.99/1.96
3	H61N6	120	-12.01/-11.99/-13.35	1.96/1.99/1.96
4	H62N6	0	-11.09/-10.44/-13.40	1.75/1.81/1.77
5	H62N6	60	-9.33/-9.55/-12.33	1.81/1.83/1.79
6	H62N6	120	-9.33/-9.55/-12.33	1.81/1.83/1.79
7	H2C2	0	-8.91/-8.69/-7.40	2.18/2.30/2.34
8	H8C8	0	-7.46/-7.65/-6.96	2.18/2.16/2.18
9	N3C4	0	-1.25/-0.96/-3.26	2.19/2.09/1.99
10	N3C4	60	-1.48/-1.10/-3.47	2.16/2.08/1.98
11	N3C4	120	-0.45/-0.77/-3.37	2.21/2.08/1.98
12	N3C4	240	-0.41/-0.77/-3.37	2.21/2.08/1.98
13	N3C4	300	-1.48/-1.10/-3.47	2.16/2.08/1.98
14	N7C5	60	-4.43/-4.84/-4.73	1.96/1.89/1.87
15	N7C5	120	-1.94/-1.42/-0.31	2.02/1.93/1.93
16	N7C5	240	-1.94/-1.41/-0.31	2.02/1.93/1.93
17	N7C5	300	-4.43/-4.84/-4.73	1.96/1.88/1.87

1.3 1-methylguanine (1MG)

Figure 3: The molecule used for water complex calculations corresponding to 1-methylguanine (1MG), with possible interacting water positions. NOTE, only one water molecule was included in each calculation

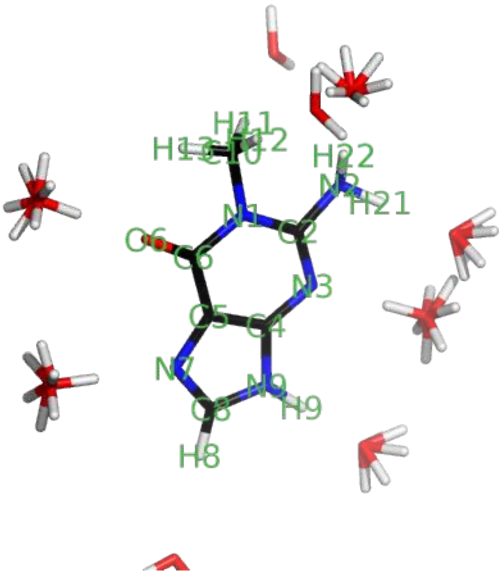

Table 7: Statistics of calculated water interaction and dipole moment for 1-methylguanine (1MG). QM is in HF level except for thio-compounds.

	RMS/Max Deviation from QM		Dipole moment	Dipole angle
	Energy (kcal/mol)	Distance (Å)	QM/MM (debye)	difference (°)
Original	0.50/1.02	0.06/0.14	6.69/6.73	2.84
Optimized	0.27/0.70	0.05/0.13	6.69/7.19	0.72

Table 8: The comparison list of optimized atomic charges and their initial guess for 1-methylguanine (1MG), referring to the penalties of initial guess

	Charges		
Atom	Optima	Initial	Penalty
N9	-0.290	-0.155	0.000
C8	0.250	0.254	0.000
H8	0.130	0.123	0.000
N7	-0.620	-0.598	0.000
C5	-0.010	0.004	8.209
C6	0.620	0.634	8.050
O6	-0.500	-0.466	0.000
N1	-0.210	-0.219	15.755
C2	0.730	0.726	18.063
N2	-0.640	-0.651	17.333
H21	0.320	0.337	0.000
H22	0.320	0.337	0.000
N3	-0.770	-0.735	5.821
C4	0.290	0.126	0.000
C10	-0.230	-0.274	5.525
H11	0.090	0.090	0.025
H12	0.090	0.090	0.025
H13	0.090	0.090	0.025
H9	0.340	0.287	0.000

Table 9: Interaction energies and geometries between probe water and selected 1-methylguanine (1MG) site calculated using the optimized and initial charges

			QM / Optima / Initial	
N	Probe site	Angle (°)	Energy (kcal/mol)	Distance (Å)
1	H9N9	0	-7.49/-7.72/-6.84	1.79/1.82/1.84
2	H9N9	60	-7.54/-7.62/-6.76	1.79/1.82/1.85
3	H9N9	120	-7.54/-7.64/-6.78	1.79/1.82/1.85
4	H21N2	0	-5.27/-5.24/-5.70	1.85/1.88/1.86
5	H21N2	60	-4.74/-4.94/-5.38	1.89/1.89/1.88
6	H21N2	120	-4.78/-4.91/-5.35	1.89/1.90/1.88
7	H22N2	0	-5.72/-6.02/-6.07	2.14/2.13/2.11
8	H22N2	60	-6.29/-6.17/-6.20	2.10/2.11/2.09
9	H22N2	120	-5.80/-5.99/-6.09	2.12/2.13/2.10
10	H8C8	0	-3.05/-2.89/-3.06	2.38/2.25/2.24
11	H11C10	0	-1.72/-1.62/-1.32	2.55/2.65/2.68
12	H12C10	0	-3.05/-2.44/-2.17	2.52/2.61/2.63
13	N7C5	0	-5.14/-5.14/-4.78	2.03/1.97/1.98
14	N7C5	60	-5.67/-5.82/-5.44	2.00/1.96/1.96
15	N7C5	120	-7.27/-7.49/-7.01	1.97/1.93/1.94
16	N7C5	180	-8.65/-8.74/-8.17	1.93/1.91/1.92
17	N7C5	240	-7.27/-7.55/-7.09	1.96/1.93/1.94
18	N7C5	300	-5.72/-5.88/-5.52	2.00/1.96/1.96
19	O6C6	0	-7.05/-7.20/-6.47	1.83/1.76/1.78
20	O6C6	60	-6.60/-6.61/-5.95	1.83/1.77/1.79
21	O6C6	120	-5.67/-5.60/-5.11	1.85/1.78/1.81
22	O6C6	180	-5.09/-5.15/-4.76	1.87/1.79/1.81
23	O6C6	240	-5.63/-5.68/-5.21	1.86/1.78/1.81
24	O6C6	300	-6.60/-6.69/-6.04	1.83/1.77/1.79
25	N3C4	0	-4.29/-4.37/-4.17	1.97/1.95/1.97
26	N3C4	60	-5.67/-5.14/-5.09	1.88/1.93/1.93
27	N3C4	120	-6.07/-5.77/-5.95	1.86/1.91/1.91
28	N3C4	180	-4.65/-5.35/-5.67	1.94/1.93/1.92
29	N3C4	240	-6.29/-6.22/-6.38	1.87/1.91/1.91
30	N3C4	300	-6.38/-5.72/-5.80	1.87/1.93/1.92

1.4 1-methylinosine (1MI)

Figure 4: The molecule used for water complex calculations corresponding to 1-methylinosine (1MI), with possible interacting water positions. NOTE, only one water molecule was included in each calculation

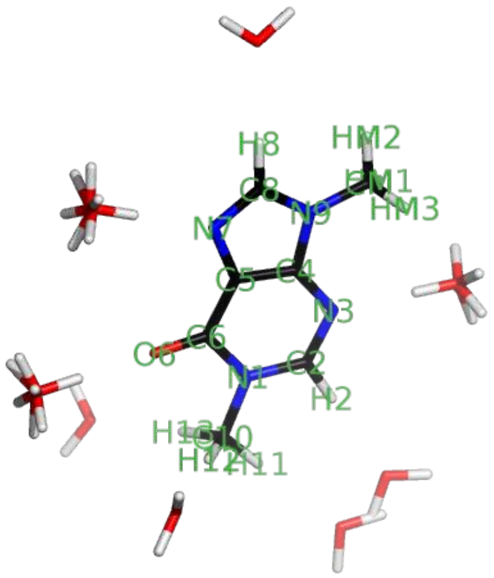

Table 10: Statistics of calculated water interaction and dipole moment for 1-methylinosine (1MI). QM is in HF level except for thio-compounds.

	RMS/Max Deviation from QM		Dipole moment	Dipole angle
	Energy (kcal/mol)	Distance (Å)	QM/MM (debye)	difference (°)
Original	0.84/1.36	0.05/0.14	6.05/4.59	30.34
Optimized	0.30/0.62	0.07/0.15	6.05/5.07	14.61

Table 11: The comparison list of optimized atomic charges and their initial guess for 1-methylinosine (1MI), referring to the penalties of initial guess

	Charges		
Atom	Optima	Initial	Penalty
N9	-0.000	-0.000	0.000
C8	0.365	0.250	0.000
H8	0.093	0.122	0.000
N7	-0.701	-0.600	0.000
C5	0.073	0.014	8.209
C6	0.652	0.626	9.862
O6	-0.530	-0.467	0.000
N1	-0.356	-0.120	14.434
C2	0.676	0.645	15.108
H2	0.062	0.102	13.228
N3	-0.807	-0.822	5.821
C4	0.347	0.255	0.000
C10	-0.144	-0.275	5.326
H11	0.090	0.090	0.025
H12	0.090	0.090	0.025
H13	0.090	0.090	0.025
CM	-0.270	-0.270	0.000
HM1	0.090	0.090	0.000
HM2	0.090	0.090	0.000
HM3	0.090	0.090	0.000

Table 12: Interaction energies and geometries between probe water and selected 1-methylinosine (1MI) site calculated using the optimized and initial charges

			QM / Optima / Initial	
N	Probe site	Angle (°)	Energy (kcal/mol)	Distance (Å)
1	H2C2	0	-4.16/-4.12/-5.07	2.36/2.37/2.33
2	H8C8	0	-3.23/-3.17/-2.88	2.39/2.24/2.24
3	H11C10	0	-3.36/-3.36/-3.56	2.59/2.57/2.58
4	H12C10	0	-1.27/-1.04/-1.23	2.54/2.63/2.64
5	H13C10	0	-1.27/-1.04/-1.23	2.54/2.63/2.64
6	O6C6	0	-7.14/-7.38/-6.04	1.82/1.75/1.79
7	O6C6	60	-6.65/-6.84/-5.54	1.84/1.76/1.80
8	O6C6	120	-5.80/-5.84/-4.63	1.85/1.77/1.81
9	O6C6	180	-5.36/-5.33/-4.20	1.86/1.78/1.82
10	O6C6	240	-5.80/-5.84/-4.63	1.85/1.77/1.81
11	O6C6	300	-6.65/-6.84/-5.54	1.84/1.76/1.80
12	N3C4	0	-5.80/-5.18/-5.24	1.96/1.98/1.98
13	N3C4	60	-6.07/-5.53/-5.84	1.94/1.97/1.96
14	N3C4	120	-5.14/-5.73/-6.50	1.97/1.96/1.95
15	N3C4	240	-5.14/-5.73/-6.50	1.97/1.96/1.95
16	N3C4	300	-6.07/-5.53/-5.84	1.94/1.97/1.96
17	N7C5	0	-5.00/-5.02/-4.78	2.02/1.96/1.97
18	N7C5	60	-5.58/-5.69/-5.30	2.00/1.95/1.96
19	N7C5	120	-7.18/-7.34/-6.48	1.97/1.92/1.95
20	N7C5	180	-8.56/-8.55/-7.37	1.93/1.90/1.93
21	N7C5	240	-7.18/-7.35/-6.48	1.97/1.92/1.95
22	N7C5	300	-5.58/-5.70/-5.30	2.00/1.95/1.96

1.5 1-methylpseudouracil (1MP)

Figure 5: The molecule used for water complex calculations corresponding to 1-methylpseudouracil (1MP), with possible interacting water positions. NOTE, only one water molecule was included in each calculation

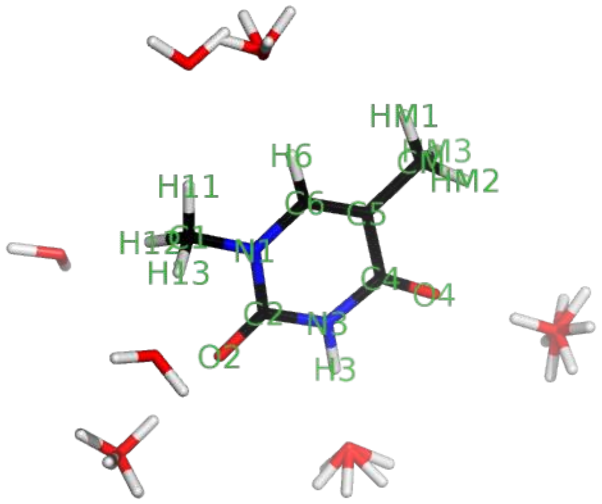

Table 13: Statistics of calculated water interaction and dipole moment for 1-methylpseudouracil (1MP). QM is in HF level except for thio-compounds.

	RMS/Max Deviation from QM		Dipole moment	Dipole angle
	Energy (kcal/mol)	Distance (Å)	QM/MM (debye)	difference (°)
Original	0.89/1.90	0.06/0.16	5.10/4.10	41.42
Optimized	0.18/0.42	0.07/0.08	5.10/5.53	8.11

Table 14: The comparison list of optimized atomic charges and their initial guess for 1-methylpseudouracil (1MP), referring to the penalties of initial guess

	Charges		
Atom	Optima	Initial	Penalty
C5	-0.197	-0.145	0.000
C4	0.467	0.499	0.000
O4	-0.455	-0.450	0.000
N3	-0.486	-0.461	0.000
H3	0.351	0.362	0.000
C2	0.520	0.509	0.000
O2	-0.470	-0.410	0.000
N1	-0.271	-0.337	0.000
C6	0.174	0.167	0.000
H6	0.163	0.170	0.000
C1	-0.066	-0.273	0.000
H11	0.090	0.090	0.000
H12	0.090	0.090	0.000
H13	0.090	0.090	0.000
CM	-0.270	-0.171	0.000
HM1	0.090	0.090	0.000
HM2	0.090	0.090	0.000
HM3	0.090	0.090	0.000

Table 15: Interaction energies and geometries between probe water and selected 1-methylpseudouracil (1MP) site calculated using the optimized and initial charges

			QM / Optima / Initial	
N	Probe site	Angle (°)	Energy (kcal/mol)	Distance (Å)
1	H3N3	0	-6.69/-6.35/-7.55	1.75/1.82/1.80
2	H3N3	60	-5.58/-5.81/-7.04	1.78/1.83/1.81
3	H3N3	120	-5.58/-5.81/-7.04	1.78/1.83/1.81
4	H6C6	0	-4.52/-4.93/-4.50	2.38/2.33/2.33
5	H6C6	90	-5.36/-5.15/-4.62	2.30/2.32/2.33
6	H11C1	0	-3.49/-3.54/-1.59	2.59/2.57/2.75
7	H12C1	0	-1.63/-1.46/na	2.55/2.61/na
8	H13C1	0	-1.63/-1.46/na	2.55/2.61/na
9	O2C2	0	-5.54/-5.64/-4.54	1.86/1.78/1.82
10	O2C2	60	-5.45/-5.53/-4.72	1.86/1.78/1.82
11	O2C2	120	-5.14/-5.13/-4.99	1.87/1.79/1.81
12	O2C2	180	-4.87/-4.83/-5.09	1.87/1.79/1.81
13	O2C2	240	-5.14/-5.13/-4.99	1.87/1.79/1.81
14	O2C2	300	-5.45/-5.53/-4.72	1.86/1.78/1.82
15	O4C4	0	-5.54/-5.48/-4.90	1.86/1.79/1.80
16	O4C4	60	-5.58/-5.59/-4.93	1.86/1.79/1.80
17	O4C4	120	-5.54/-5.70/-4.82	1.86/1.79/1.80
18	O4C4	180	-5.49/-5.71/-4.69	1.86/1.79/1.80
19	O4C4	240	-5.54/-5.70/-4.82	1.86/1.79/1.80
20	O4C4	300	-5.58/-5.59/-4.93	1.86/1.79/1.80

1.6 N2,7-dimethylguanine (27G)

Figure 6: The molecule used for water complex calculations corresponding to N2,7-dimethylguanine (27G), with possible interacting water positions. NOTE, only one water molecule was included in each calculation

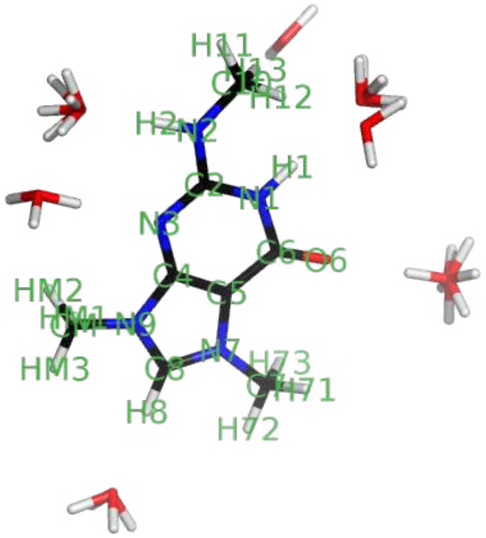

Table 16: Statistics of calculated water interaction and dipole moment for N2,7-dimethylguanine (27G). QM is in HF level except for thio-compounds.

	RMS/Max Deviation from QM		Dipole moment	Dipole angle
	Energy (kcal/mol)	Distance (Å)	QM/MM (debye)	difference (°)
Original	0.98/2.21	0.17/0.26	4.71/2.23	99.23
Optimized	0.28/0.51	0.17/0.23	4.71/3.22	83.06

Table 17: The comparison list of optimized atomic charges and their initial guess for N2,7-dimethylguanine (27G), referring to the penalties of initial guess

	Charges		
Atom	Optima	Initial	Penalty
N9	-0.103	-0.211	112.765
C8	0.405	0.358	100.119
H8	0.149	0.180	19.849
N7	-0.406	-0.395	114.033
C5	0.132	0.122	79.191
C6	0.589	0.562	29.115
O6	-0.459	-0.481	14.853
N1	-0.280	-0.323	49.157
H1	0.242	0.287	2.500
C2	0.734	0.856	60.165
N2	-0.518	-0.490	55.455
H2	0.361	0.391	3.771
N3	-0.567	-0.515	41.536
C4	0.140	0.094	78.160
C7	0.146	0.192	109.300
H71	0.090	0.090	3.537
H72	0.090	0.090	3.537
H73	0.090	0.090	3.537
C10	-0.105	-0.167	9.838
H11	0.090	0.090	0.600
H12	0.090	0.090	0.600
H13	0.090	0.090	0.600
CM	-0.270	-0.270	109.300
HM1	0.090	0.090	3.537
HM2	0.090	0.090	3.537
HM3	0.090	0.090	3.537

Table 18: Interaction energies and geometries between probe water and selected N2,7-dimethylguanine (27G) site calculated using the optimized and initial charges

			QM / Optima / Initial	
N	Probe site	Angle (°)	Energy (kcal/mol)	Distance (Å)
1	H1N1	0	-8.53/-8.48/-9.26	2.12/2.30/2.23
2	H1N1	60	-8.30/-8.55/-9.31	2.19/2.29/2.23
3	H1N1	120	-8.30/-8.55/-9.31	2.19/2.29/2.23
4	H2N2	0	-8.87/-9.01/-11.08	1.86/1.99/1.96
5	H2N2	60	-9.03/-8.97/-10.94	1.86/2.00/1.96
6	H2N2	120	-9.03/-9.08/-10.94	1.86/1.99/1.96
7	H12C10	0	-5.81/-5.30/-5.57	2.35/2.55/2.54
8	H13C10	0	-5.81/-5.29/-5.57	2.35/2.55/2.54
9	H8C8	0	-10.67/-10.94/-10.92	2.08/1.99/1.99
10	H8C8	90	-11.74/-11.40/-11.24	2.04/1.99/1.98
11	O6C6	0	-2.21/-2.62/-3.03	2.05/1.82/1.80
12	O6C6	60	-2.13/-2.36/-2.80	2.05/1.82/1.80
13	O6C6	120	-1.52/-1.36/-1.81	2.07/1.84/1.82
14	O6C6	180	-0.98/-0.56/-0.99	2.09/1.86/1.84
15	O6C6	240	-1.52/-1.36/-1.81	2.07/1.84/1.82
16	O6C6	300	-2.13/-2.36/-2.80	2.05/1.82/1.80
17	N3C4	120	-1.67/-1.71/-1.29	2.09/2.08/2.10
18	N3C4	240	-1.67/-1.71/-1.29	2.09/2.08/2.10

1.7 2-methyladenine (2MA)

Figure 7: The molecule used for water complex calculations corresponding to 2-methyladenine (2MA), with possible interacting water positions. NOTE, only one water molecule was included in each calculation

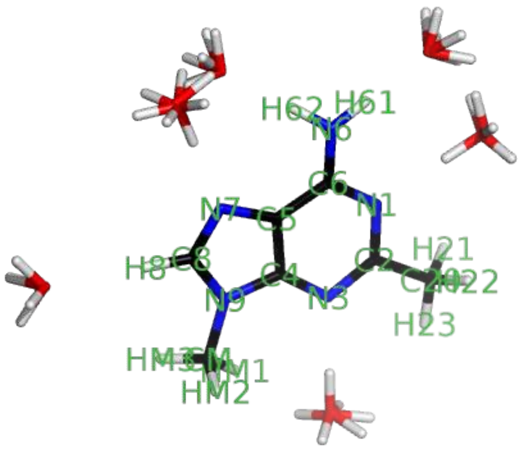

Table 19: Statistics of calculated water interaction and dipole moment for 2-methyladenine (2MA). QM is in HF level except for thio-compounds.

	RMS/Max Deviation from QM		Dipole moment	Dipole angle
	Energy (kcal/mol)	Distance (Å)	QM/MM (debye)	difference (°)
Original	0.98/1.77	0.08/0.18	2.59/2.94	16.44
Optimized	0.35/0.92	0.05/0.14	2.59/2.80	13.49

Table 20: The comparison list of optimized atomic charges and their initial guess for 2-methyladenine (2MA), referring to the penalties of initial guess

	Charges		
Atom	Optima	Initial	Penalty
N9	0.040	0.005	0.000
C8	0.430	0.335	0.000
H8	0.060	0.119	0.000
N7	-0.840	-0.713	0.000
C5	0.290	0.277	0.000
C6	0.570	0.384	2.500
N6	-0.760	-0.773	0.000
H61	0.360	0.375	0.000
H62	0.360	0.375	0.000
N1	-0.780	-0.647	27.954
C2	0.380	0.427	40.889
N3	-0.830	-0.696	27.954
C4	0.540	0.431	2.500
C20	-0.090	-0.169	13.338
H21	0.090	0.090	3.536
H22	0.090	0.090	3.536
H23	0.090	0.090	3.536
CM	-0.270	-0.270	0.000
HM1	0.090	0.090	0.000
HM2	0.090	0.090	0.000
HM3	0.090	0.090	0.000

Table 21: Interaction energies and geometries between probe water and selected 2-methyladenine (2MA) site calculated using the optimized and initial charges

			QM / Optima / Initial	
N	Probe site	Angle (°)	Energy (kcal/mol)	Distance (Å)
1	H61N6	0	-4.38/-4.24/-4.28	1.88/1.89/1.88
2	H61N6	60	-3.94/-3.91/-4.02	1.91/1.90/1.89
3	H61N6	120	-4.07/-4.01/-4.10	1.90/1.89/1.89
4	H62N6	0	-4.60/-4.69/-4.53	1.82/1.85/1.85
5	H62N6	60	-3.98/-4.31/-4.21	1.85/1.86/1.87
6	H62N6	120	-2.65/-3.51/-3.58	1.92/1.89/1.89
7	H8C8	0	-3.23/-3.33/-4.06	2.39/2.25/2.21
8	H8C8	90	-3.41/-3.30/-4.04	2.36/2.25/2.21
9	N1C2	0	-5.32/-5.79/-4.78	1.96/1.95/1.99
10	N1C2	60	-7.49/-7.36/-6.31	1.86/1.91/1.95
11	N1C2	120	-7.80/-7.77/-6.66	1.86/1.91/1.94
12	N1C2	180	-6.96/-7.17/-6.17	1.90/1.92/1.95
13	N1C2	240	-7.45/-7.15/-6.02	1.86/1.91/1.95
14	N1C2	300	-6.38/-6.31/-5.27	1.89/1.93/1.97
15	N3C4	0	-6.74/-7.03/-5.73	1.93/1.97/2.02
16	N3C4	60	-7.40/-7.31/-5.95	1.89/1.96/2.01
17	N3C4	120	-6.83/-6.59/-5.41	1.91/1.97/2.01
18	N3C4	240	-5.67/-6.20/-5.01	1.96/1.98/2.03
19	N3C4	300	-7.49/-7.06/-5.72	1.90/1.96/2.01
20	N7C5	0	-6.96/-7.88/-6.60	1.88/1.86/1.89
21	N7C5	60	-7.98/-8.16/-6.99	1.85/1.86/1.89
22	N7C5	120	-7.76/-7.37/-6.42	1.85/1.86/1.89
23	N7C5	180	-4.87/-5.13/-4.30	1.97/1.91/1.95
24	N7C5	240	-6.20/-6.03/-5.07	1.89/1.88/1.91
25	N7C5	300	-7.67/-7.46/-6.28	1.85/1.86/1.89

1.8 N2-methylguanine (2MG)

Figure 8: The molecule used for water complex calculations corresponding to N2-methylguanine (2MG), with possible interacting water positions. NOTE, only one water molecule was included in each calculation

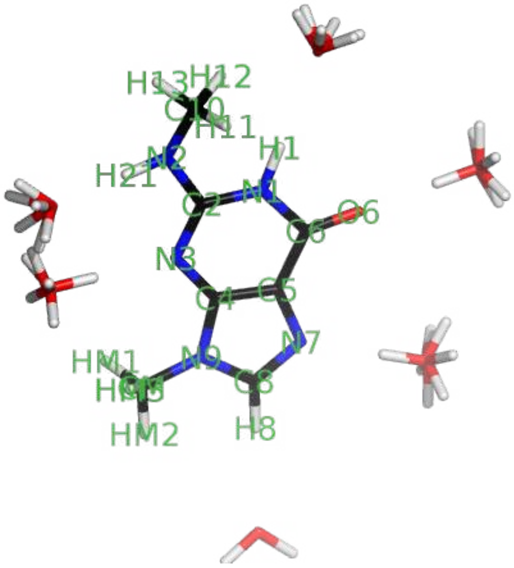

Table 22: Statistics of calculated water interaction and dipole moment for N2-methylguanine (2MG). QM is in HF level except for thio-compounds.

	RMS/Max Deviation from QM		Dipole moment	Dipole angle
	Energy (kcal/mol)	Distance (Å)	QM/MM (debye)	difference (°)
Original	0.91/2.28	0.14/0.35	7.71/6.90	14.49
Optimized	0.40/0.90	0.13/0.33	7.71/8.08	24.86

Table 23: The comparison list of optimized atomic charges and their initial guess for N2-methylguanine (2MG), referring to the penalties of initial guess

	Charges		
Atom	Optima	Initial	Penalty
N9	-0.010	-0.010	0.000
C8	0.274	0.249	0.000
H8	0.127	0.123	0.000
N7	-0.630	-0.599	0.000
C5	0.036	0.005	0.000
C6	0.514	0.535	2.500
O6	-0.504	-0.508	0.000
N1	-0.436	-0.350	47.475
H1	0.312	0.260	2.500
C2	0.677	0.850	58.598
N2	-0.464	-0.517	55.455
H21	0.323	0.364	3.771
N3	-0.773	-0.740	30.744
C4	0.302	0.262	2.500
C10	-0.018	-0.194	9.838
H11	0.090	0.090	0.600
H12	0.090	0.090	0.600
H13	0.090	0.090	0.600
CM	-0.270	-0.270	0.000
HM1	0.090	0.090	0.000
HM2	0.090	0.090	0.000
HM3	0.090	0.090	0.000

Table 24: Interaction energies and geometries between probe water and selected N2-methylguanine (2MG) site calculated using the optimized and initial charges

			QM / Optima / Initial	
N	Probe site	Angle (°)	Energy (kcal/mol)	Distance (Å)
1	H1N1	0	-4.16/-4.23/-3.65	2.31/2.62/2.61
2	H1N1	60	-4.52/-4.33/-3.59	2.26/2.60/2.61
3	H1N1	120	-4.29/-4.51/-3.64	2.33/2.58/2.61
4	H21N2	0	-5.27/-5.17/-6.17	1.85/2.03/2.00
5	H21N2	60	-4.92/-4.82/-5.82	1.88/2.04/2.02
6	H21N2	120	-4.92/-4.87/-5.90	1.88/2.04/2.01
7	H8C8	0	-2.83/-2.86/-2.75	2.41/2.24/2.25
8	O6C6	0	-5.49/-5.12/-5.00	1.86/1.79/1.79
9	O6C6	60	-5.89/-5.71/-5.53	1.85/1.78/1.78
10	O6C6	120	-6.74/-6.99/-6.66	1.84/1.76/1.77
11	O6C6	180	-7.23/-7.66/-7.27	1.83/1.75/1.76
12	O6C6	240	-6.78/-7.07/-6.71	1.84/1.76/1.77
13	O6C6	300	-5.94/-5.81/-5.59	1.85/1.78/1.78
14	N3C4	0	-4.74/-3.92/-2.60	1.97/2.01/2.06
15	N3C4	60	-6.56/-5.72/-4.28	1.89/1.96/1.99
16	N3C4	120	-6.25/-7.15/-5.76	1.91/1.93/1.96
17	N3C4	240	-5.98/-6.74/-5.46	1.91/1.94/1.96
18	N3C4	300	-5.85/-5.22/-3.82	1.91/1.96/2.00
19	N7C8	0	-8.74/-8.72/-8.20	1.93/1.91/1.92
20	N7C8	60	-7.36/-7.45/-7.02	1.96/1.93/1.94
21	N7C8	120	-5.76/-5.79/-5.47	1.99/1.95/1.96
22	N7C8	180	-5.23/-5.13/-4.83	2.01/1.97/1.98
23	N7C8	240	-5.80/-5.85/-5.51	1.99/1.95/1.96
24	N7C8	300	-7.40/-7.54/-7.08	1.96/1.93/1.94

1.9 2-thiocytosine (2SC)

Figure 9: The molecule used for water complex calculations corresponding to 2-thiocytosine (2SC), with possible interacting water positions. NOTE, only one water molecule was included in each calculation

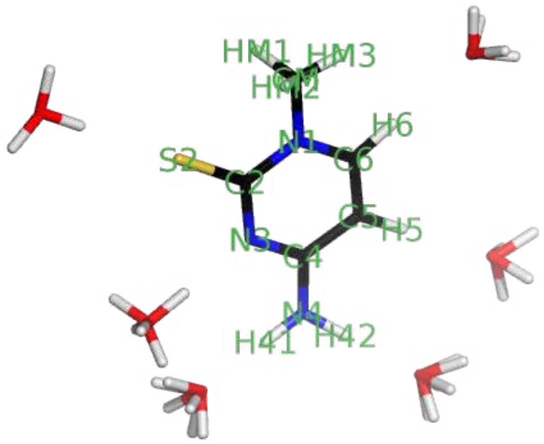

Table 25: Statistics of calculated water interaction and dipole moment for 2-thiocytosine (2SC). QM is in HF level except for thio-compounds.

	RMS/Max Deviation from QM		Dipole moment	Dipole angle
	Energy (kcal/mol)	Distance (Å)	QM/MM (debye)	difference (°)
Original	1.74/2.74	0.16/0.34	7.28/10.00	5.30
Optimized	0.26/0.70	0.08/0.13	7.28/8.43	3.74

Table 26: The comparison list of optimized atomic charges and their initial guess for 2-thiocytosine (2SC), referring to the penalties of initial guess

	Charges		
Atom	Optima	Initial	Penalty
N1	-0.210	0.102	51.485
C2	0.310	-0.011	113.511
S2	-0.180	-0.255	111.636
N3	-0.770	-0.517	46.173
C4	0.480	0.440	9.122
N4	-0.690	-0.754	5.664
H41	0.360	0.350	0.656
H42	0.360	0.350	0.656
C5	-0.130	-0.200	15.903
H5	0.140	0.198	7.395
C6	0.110	-0.004	23.774
H6	0.220	0.301	13.189
CM	-0.270	-0.270	4.625
HM1	0.090	0.090	0.075
HM2	0.090	0.090	0.075
HM3	0.090	0.090	0.075

Table 27: Interaction energies and geometries between probe water and selected 2-thiocytosine (2SC) site calculated using the optimized and initial charges

			QM / Optima / Initial	
	Probe	Angle	Energy	Distance
N	site	(°)	(kcal/mol)	(Å)
1	H42N4	0	-6.92/-7.16/-5.94	1.82/1.87/1.91
2	H42N4	60	-7.63/-7.60/-6.46	1.79/1.86/1.89
3	H42N4	120	-7.00/-7.28/-6.08	1.81/1.86/1.90
4	H5C5	0	-3.89/-4.12/-4.78	2.31/2.39/2.35
5	H5C5	90	-4.60/-4.46/-5.12	2.24/2.37/2.34
6	H6C6	0	-4.78/-5.48/-7.53	2.25/2.30/2.25
7	H6C6	90	-5.63/-5.60/-7.69	2.20/2.30/2.25
8	S2C2	0	-2.92/-3.00/-4.09	2.49/2.43/2.36
9	S2C2	180	-2.29/-2.34/-3.19	2.53/2.47/2.39
10	N3C2	60	-6.56/-6.32/-4.59	1.85/1.93/2.02
11	N3C2	120	-9.49/-9.38/-7.76	1.79/1.89/1.95
12	N3C2	240	-9.67/-9.96/-8.33	1.80/1.89/1.95
13	N3C2	300	-7.40/-7.25/-5.48	1.83/1.92/2.00

1.10 2-thiouracil (2SU)

Figure 10: The molecule used for water complex calculations corresponding to 2-thiouracil (2SU), with possible interacting water positions. NOTE, only one water molecule was included in each calculation

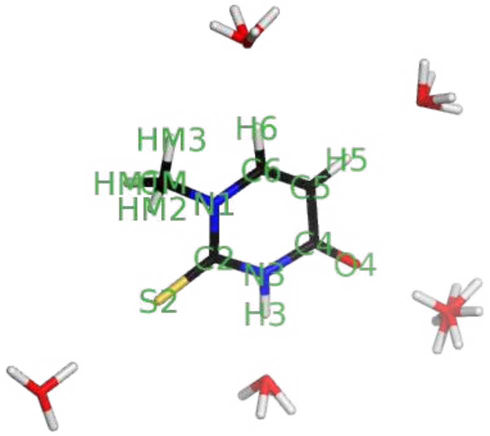

Table 28: Statistics of calculated water interaction and dipole moment for 2-thiouracil (2SU). QM is in HF level except for thio-compounds.

	RMS/Max Deviation from QM		Dipole moment	Dipole angle
	Energy (kcal/mol)	Distance (Å)	QM/MM (debye)	difference (°)
Original	0.70/1.46	0.17/0.41	5.02/4.54	33.76
Optimized	0.25/0.61	0.09/0.15	5.02/4.68	11.82

Table 29: The comparison list of optimized atomic charges and their initial guess for 2-thiouracil (2SU), referring to the penalties of initial guess

	Charges		
Atom	Optima	Initial	Penalty
N1	-0.253	-0.162	46.932
C2	0.309	-0.003	113.413
S2	-0.220	-0.255	111.938
N3	-0.563	-0.281	46.932
H3	0.404	0.362	2.500
C4	0.401	0.492	2.500
O4	-0.434	-0.474	0.000
C5	-0.247	-0.146	0.000
H5	0.198	0.116	0.000
C6	0.202	0.210	2.500
H6	0.203	0.141	0.000
CM	-0.270	-0.270	2.500
HM1	0.090	0.090	0.000
HM2	0.090	0.090	0.000
HM3	0.090	0.090	0.000

Table 30: Interaction energies and geometries between probe water and selected 2-thiouracil (2SU) site calculated using the optimized and initial charges

			QM / Optima / Initial	
N	Probe site	Angle (°)	Energy (kcal/mol)	Distance (Å)
1	H3N3	60	-6.96/-6.84/-7.79	1.68/1.81/1.80
2	H3N3	120	-6.96/-6.84/-7.79	1.68/1.81/1.80
3	H5C5	0	-3.63/-3.60/-3.11	2.23/2.35/2.39
4	H5C5	90	-3.54/-3.55/-3.04	2.23/2.35/2.39
5	H6C6	0	-5.18/-5.71/-4.83	2.24/2.30/2.33
6	H6C6	90	-6.12/-5.81/-4.89	2.18/2.29/2.33
7	S2C2	0	-1.81/-2.04/-2.67	2.59/2.47/2.42
8	S2C2	180	-1.45/-2.06/-2.91	2.61/2.46/2.40
9	O4C4	0	-5.14/-5.26/-4.79	1.83/1.80/1.80
10	O4C4	60	-5.14/-5.30/-4.86	1.83/1.80/1.80
11	O4C4	120	-5.14/-5.20/-4.86	1.83/1.80/1.80
12	O4C4	180	-5.09/-5.09/-4.79	1.83/1.80/1.80
13	O4C4	240	-5.14/-5.21/-4.86	1.83/1.80/1.80
14	O4C4	300	-5.14/-5.30/-4.86	1.83/1.80/1.80

1.11 2-methylamino-4-amino-pyrimidine, protonated (34hc, for K2C, R2C)

Figure 11: The molecule used for water complex calculations corresponding to 2-methylamino-4-amino-pyrimidine, protonated (34hc, for K2C, R2C), with possible interacting water positions. NOTE, only one water molecule was included in each calculation

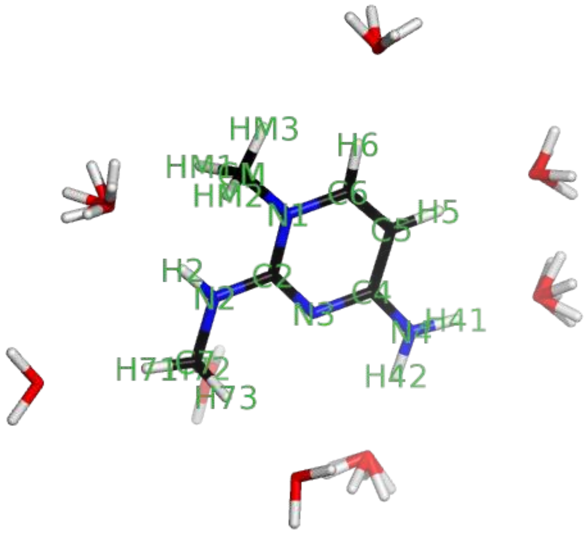

Table 31: Statistics of calculated water interaction and dipole moment for 2-methylamino-4-amino-pyrimidine, protonated (34hc, for K2C, R2C). QM is in HF level except for thio-compounds.

	RMS/Max Deviation from QM		Dipole moment	Dipole angle
	Energy (kcal/mol)	Distance (Å)	QM/MM (debye)	difference (°)
Original	3.76/5.33	0.23/0.49	2.29/10.71	73.45
Optimized	0.36/0.81	0.11/0.29	2.29/2.88	53.37

Table 32: The comparison list of optimized atomic charges and their initial guess for 2-methylamino-4-amino-pyrimidine, protonated (34hc, for K2C, R2C), referring to the penalties of initial guess

	Charges		
Atom	Optima	Initial	Penalty
N1	-0.430	-0.416	52.284
C2	0.810	0.459	61.622
N2	-0.570	-0.517	55.455
H2	0.460	0.364	3.771
N3	-0.620	-0.660	37.127
C4	0.610	1.649	199.747
N4	-0.800	-1.180	200.294
H41	0.380	0.460	20.716
H42	0.380	0.460	20.716
C5	0.080	-0.164	22.794
H5	0.070	0.169	6.869
C6	0.180	-0.005	25.439
H6	0.200	0.305	13.355
C7	-0.020	-0.194	9.838
H71	0.090	0.090	0.600
H72	0.090	0.090	0.600
H73	0.090	0.090	0.600
CM	-0.270	-0.270	5.949
HM1	0.090	0.090	0.079
HM2	0.090	0.090	0.079
HM3	0.090	0.090	0.079

Table 33: Interaction energies and geometries between probe water and selected 2-methylamino-4-amino-pyrimidine, protonated (34hc, for K2C, R2C) site calculated using the optimized and initial charges

			QM / Optima / Initial	
N	Probe site	Angle (°)	Energy (kcal/mol)	Distance (Å)
1	H2N2	0	-8.89/-9.18/-5.17	2.30/2.33/2.54
2	H2N2	60	-9.39/-9.27/-5.22	2.33/2.33/2.54
3	H2N2	120	-9.39/-9.27/-5.22	2.33/2.33/2.54
4	H41N4	0	-10.69/-11.50/-16.03	1.88/1.84/1.78
5	H41N4	60	-11.65/-11.97/-16.69	1.85/1.83/1.77
6	H41N4	120	-11.65/-11.97/-16.69	1.85/1.82/1.77
7	H42N4	0	-9.43/-9.22/-14.31	1.84/1.85/1.76
8	H42N4	60	-9.66/-9.45/-14.19	1.85/1.85/1.77
9	H42N4	120	-9.66/-9.52/-14.19	1.85/1.85/1.77
10	H5C5	0	-7.21/-7.57/-10.57	2.29/2.50/2.43
11	H5C5	90	-8.32/-7.91/-11.00	2.20/2.49/2.41
12	H6C6	0	-8.89/-9.34/-10.47	2.19/2.27/2.24
13	H6C6	90	-10.16/-9.51/-10.64	2.13/2.26/2.23
14	H71C7	0	-5.37/-5.09/-2.72	2.41/2.55/2.68
15	H72C7	0	-4.14/-4.08/-2.55	2.52/2.62/3.02
16	H73C7	0	-4.14/-4.12/-2.55	2.52/2.61/3.02

1.12 3-methylcytosine (3MCn)

Figure 12: The molecule used for water complex calculations corresponding to 3-methylcytosine (3MCn), with possible interacting water positions. NOTE, only one water molecule was included in each calculation

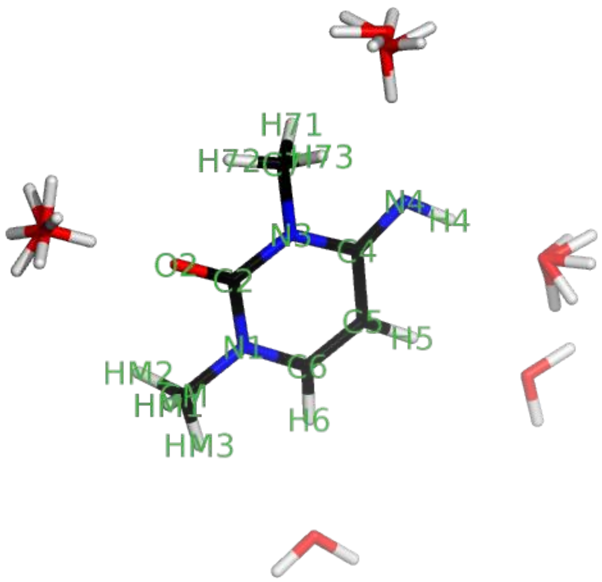

Table 34: Statistics of calculated water interaction and dipole moment for 3-methylcytosine (3MCn). QM is in HF level except for thio-compounds.

	RMS/Max Deviation from QM		Dipole moment	Dipole angle
	Energy (kcal/mol)	Distance (Å)	QM/MM (debye)	difference (°)
Original	3.24/6.69	0.29/0.61	4.95/8.03	54.14
Optimized	0.12/0.23	0.10/0.19	4.95/5.71	8.31

Table 35: The comparison list of optimized atomic charges and their initial guess for 3-methylcytosine (3MCn), referring to the penalties of initial guess

	Charges		
Atom	Optima	Initial	Penalty
N1	-0.080	-0.278	0.261
C2	0.413	0.245	9.436
O2	-0.441	-0.367	0.000
N3	-0.338	-0.196	25.738
C4	0.491	0.575	136.321
N4	-0.861	-0.561	135.009
H4	0.320	0.378	20.206
C5	0.036	-0.023	15.605
H5	0.019	0.019	3.113
C6	0.131	0.041	8.748
H6	0.102	0.170	0.000
C7	-0.062	-0.273	2.299
H71	0.090	0.090	0.025
H72	0.090	0.090	0.025
H73	0.090	0.090	0.025
CM	-0.270	-0.270	0.000
HM1	0.090	0.090	0.000
HM2	0.090	0.090	0.000
HM3	0.090	0.090	0.000

Table 36: Interaction energies and geometries between probe water and selected 3-methylcytosine (3MCn) site calculated using the optimized and initial charges

			QM / Optima / Initial	
N	Probe site	Angle (°)	Energy (kcal/mol)	Distance (Å)
1	H4N4	0	-2.58/-2.81/-9.28	2.21/2.02/1.82
2	H4N4	60	-3.25/-3.10/-9.52	2.13/2.00/1.81
3	H4N4	120	-3.25/-3.10/-9.52	2.13/2.00/1.81
4	H5C5	0	-2.76/-2.85/-3.58	2.52/2.45/2.45
5	H6C6	0	-4.27/-4.35/-3.89	2.38/2.36/2.35
6	O2C2	0	-5.83/-5.67/-5.92	1.85/1.79/1.80
7	O2C2	60	-5.83/-5.69/-5.96	1.84/1.79/1.80
8	O2C2	120	-5.56/-5.57/-5.99	1.84/1.79/1.80
9	O2C2	180	-5.29/-5.46/-5.99	1.85/1.79/1.80
10	O2C2	240	-5.56/-5.57/-5.99	1.84/1.79/1.80
11	O2C2	300	-5.83/-5.69/-5.96	1.84/1.79/1.80
12	N4C4	0	-5.07/-4.91/-0.72	2.16/2.35/2.77
13	N4C4	60	-4.63/-4.61/-1.01	2.26/2.36/2.75
14	N4C4	120	-3.65/-3.58/-1.72	2.43/2.43/2.72
15	N4C4	180	-2.94/-2.89/-2.28	2.55/2.49/2.67
16	N4C4	240	-3.65/-3.58/-1.72	2.43/2.43/2.72
17	N4C4	300	-4.63/-4.61/-1.01	2.26/2.36/2.75

1.13 3-methylcytosine protonated (3MC)

Figure 13: The molecule used for water complex calculations corresponding to 3-methylcytosine protonated (3MC), with possible interacting water positions. NOTE, only one water molecule was included in each calculation

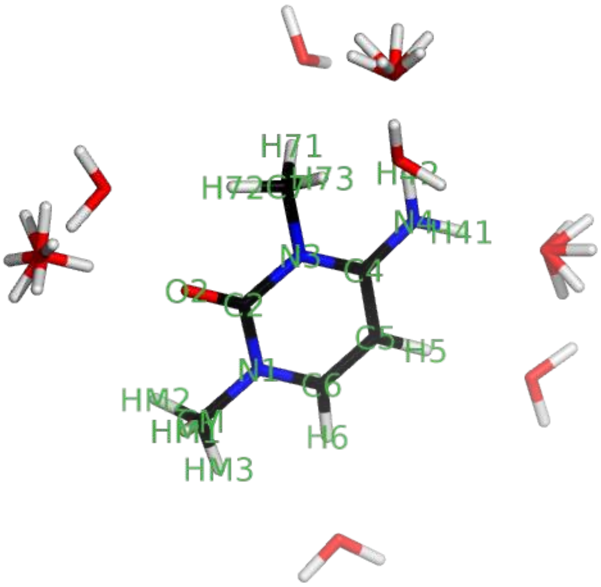

Table 37: Statistics of calculated water interaction and dipole moment for 3-methylcytosine protonated (3MC). QM is in HF level except for thio-compounds.

	RMS/Max Deviation from QM		Dipole moment	Dipole angle
	Energy (kcal/mol)	Distance (Å)	QM/MM (debye)	difference (°)
Original	2.73/4.94	0.22/0.35	5.93/13.80	18.90
Optimized	0.41/0.81	0.18/0.25	5.93/7.41	11.73

Table 38: The comparison list of optimized atomic charges and their initial guess for 3-methylcytosine protonated (3MC), referring to the penalties of initial guess

	Charges		
Atom	Optima	Initial	Penalty
N1	-0.110	-0.278	9.406
C2	0.550	0.166	19.137
O2	-0.330	-0.367	9.326
N3	-0.350	-0.278	78.066!
C4	0.680	1.536	200.580
N4	-0.760	-1.147	201.141
H41	0.390	0.460	20.716
H42	0.390	0.460	20.716
C5	-0.180	-0.018	22.447
H5	0.090	0.019	3.275
C6	0.160	0.041	11.887
H6	0.200	0.170	0.000
C7	0.000	-0.034	73.714
H71	0.090	0.090	3.767
H72	0.090	0.090	3.767
H73	0.090	0.090	3.767
CM	-0.270	-0.270	0.000
HM1	0.090	0.090	0.000
HM2	0.090	0.090	0.000
HM3	0.090	0.090	0.000

Table 39: Interaction energies and geometries between probe water and selected 3-methylcytosine protonated (3MC) site calculated using the optimized and initial charges

			QM / Optima / Initial	
N	Probe site	Angle (°)	Energy (kcal/mol)	Distance (Å)
1	H41N4	0	-12.91/-13.72/-17.86	1.83/1.80/1.75
2	H41N4	60	-14.06/-14.12/-18.26	1.80/1.79/1.74
3	H41N4	120	-14.06/-14.13/-18.26	1.80/1.79/1.74
4	H42N4	0	-11.15/-10.45/-13.21	2.05/2.27/2.17
5	H42N4	60	-11.50/-10.94/-13.63	2.06/2.24/2.14
6	H42N4	120	-11.50/-10.94/-13.63	2.06/2.24/2.14
7	H5C5	0	-8.09/-7.88/-9.71	2.27/2.36/2.32
8	H6C6	0	-9.70/-9.61/-7.99	2.14/2.26/2.30
9	H71C7	0	-7.44/-7.05/-8.17	2.30/2.51/2.49
10	H72C7	0	-3.53/-3.89/-2.78	2.58/2.77/2.78
11	H73C7	0	-7.44/-7.04/-8.17	2.30/2.51/2.49
12	O2C2	0	-0.27/0.05/-1.98	2.18/1.95/1.85
13	O2C2	60	-0.58/-0.38/-2.71	2.15/1.93/1.83
14	O2C2	120	-0.77/-1.01/-3.93	2.14/1.91/1.81
15	O2C2	180	-0.66/-1.23/-4.45	2.15/1.91/1.80
16	O2C2	240	-0.77/-1.01/-3.93	2.14/1.91/1.81
17	O2C2	300	-0.58/-0.38/-2.71	2.15/1.93/1.83

1.14 3-methylpseudouracil (3MP)

Figure 14: The molecule used for water complex calculations corresponding to 3-methylpseudouracil (3MP), with possible interacting water positions. NOTE, only one water molecule was included in each calculation

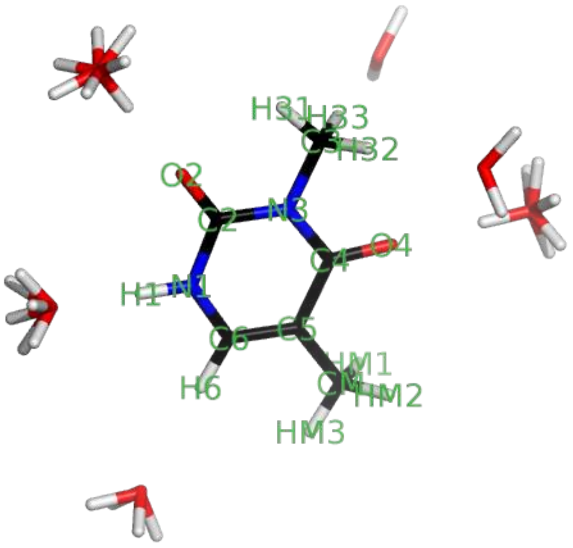

Table 40: Statistics of calculated water interaction and dipole moment for 3-methylpseudouracil (3MP). QM is in HF level except for thio-compounds.

	RMS/Max Deviation from QM		Dipole moment	Dipole angle
	Energy (kcal/mol)	Distance (Å)	QM/MM (debye)	difference (°)
Original	1.51/3.03	0.03/0.05	4.28/7.64	5.63
Optimized	0.14/0.28	0.06/0.08	4.28/4.69	5.99

Table 41: The comparison list of optimized atomic charges and their initial guess for 3-methylpseudouracil (3MP), referring to the penalties of initial guess

	Charges		
Atom	Optima	Initial	Penalty
C5	-0.193	-0.185	6.841
C4	0.625	0.595	6.708
O4	-0.475	-0.399	0.000
N3	-0.398	-0.480	4.743
C2	0.525	0.518	0.000
O2	-0.473	-0.401	0.000
N1	-0.320	-0.290	0.000
H1	0.305	0.357	0.000
C6	0.030	0.080	0.000
H6	0.194	0.199	0.000
C3	-0.090	-0.264	0.750
H31	0.090	0.090	0.000
H32	0.090	0.090	0.000
H33	0.090	0.090	0.000
CM	-0.270	-0.270	0.000
HM1	0.090	0.090	0.000
HM2	0.090	0.090	0.000
HM3	0.090	0.090	0.000

Table 42: Interaction energies and geometries between probe water and selected 3-methylpseudouracil (3MP) site calculated using the optimized and initial charges

			QM / Optima / Initial	
N	Probe site	Angle (°)	Energy (kcal/mol)	Distance (Å)
1	H1N1	0	-7.78/-7.70/-10.63	1.78/1.83/1.78
2	H1N1	60	-7.47/-7.53/-10.50	1.78/1.84/1.78
3	H1N1	120	-7.47/-7.54/-10.50	1.78/1.84/1.78
4	H6C6	0	-4.49/-4.75/-5.79	2.34/2.32/2.30
5	H6C6	90	-5.03/-4.88/-5.97	2.29/2.32/2.30
6	H32C3	0	-0.45/-0.23/na	2.70/2.70/na
7	H33C3	0	-0.45/-0.23/na	2.70/2.69/na
8	O2C2	0	-5.29/-5.57/-5.30	1.87/1.78/1.81
9	O2C2	60	-5.38/-5.50/-4.87	1.86/1.78/1.82
10	O2C2	120	-5.25/-5.16/-3.90	1.86/1.79/1.84
11	O2C2	180	-5.07/-4.90/-3.35	1.87/1.79/1.85
12	O2C2	240	-5.25/-5.16/-3.90	1.86/1.79/1.84
13	O2C2	300	-5.38/-5.50/-4.87	1.86/1.78/1.82
14	O4C4	0	-5.52/-5.48/-5.44	1.86/1.79/1.81
15	O4C4	60	-5.56/-5.55/-5.20	1.86/1.79/1.82
16	O4C4	120	-5.60/-5.61/-4.74	1.85/1.79/1.83
17	O4C4	180	-5.56/-5.61/-4.52	1.85/1.79/1.83
18	O4C4	240	-5.60/-5.62/-4.74	1.85/1.79/1.83
19	O4C4	300	-5.56/-5.55/-5.20	1.86/1.79/1.82

1.15 3-methyluracil (3MU)

Figure 15: The molecule used for water complex calculations corresponding to 3-methyluracil (3MU), with possible interacting water positions. NOTE, only one water molecule was included in each calculation

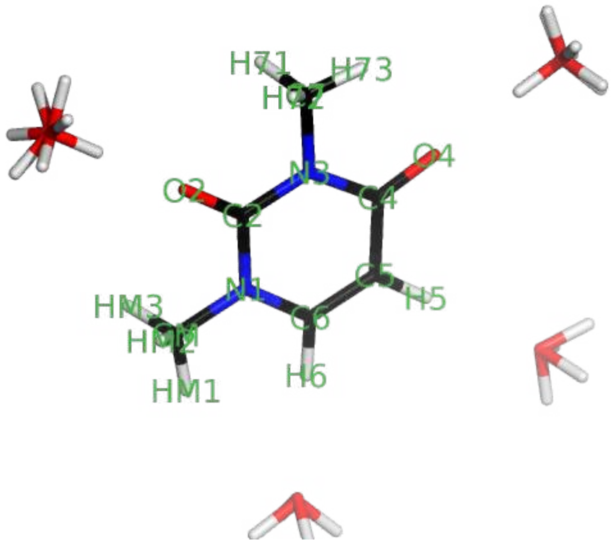

Table 43: Statistics of calculated water interaction and dipole moment for 3-methyluracil (3MU). QM is in HF level except for thio-compounds.

	RMS/Max Deviation from QM		Dipole moment	Dipole angle
	Energy (kcal/mol)	Distance (Å)	QM/MM (debye)	difference (°)
Original	1.03/1.70	0.04/0.07	4.80/6.06	29.63
Optimized	0.20/0.48	0.06/0.08	4.80/5.17	20.23

Table 44: The comparison list of optimized atomic charges and their initial guess for 3-methyluracil (3MU), referring to the penalties of initial guess

	Charges		
Atom	Optima	Initial	Penalty
N1	-0.205	-0.264	0.000
C2	0.525	0.581	0.000
O2	-0.444	-0.368	0.000
N3	-0.319	-0.415	4.743
C4	0.499	0.584	6.708
O4	-0.479	-0.432	0.000
C5	-0.217	-0.142	6.841
H5	0.086	0.116	0.000
C6	0.317	0.202	0.000
H6	0.115	0.141	0.000
C7	-0.148	-0.273	0.750
H71	0.090	0.090	0.000
H72	0.090	0.090	0.000
H73	0.090	0.090	0.000
CM	-0.270	-0.270	0.000
HM1	0.090	0.090	0.000
HM2	0.090	0.090	0.000
HM3	0.090	0.090	0.000

Table 45: Interaction energies and geometries between probe water and selected 3-methyluracil (3MU) site calculated using the optimized and initial charges

			QM / Optima / Initial	
N	Probe site	Angle (°)	Energy (kcal/mol)	Distance (Å)
1	H5C5	0	-2.21/-2.16/-3.43	2.44/2.44/2.38
2	H5C5	90	-2.03/-2.06/-3.35	2.45/2.45/2.38
3	H6C6	0	-4.65/-5.12/-5.01	2.35/2.33/2.33
4	H6C6	90	-5.36/-5.17/-5.08	2.29/2.32/2.32
5	O2C2	0	-4.96/-5.16/-3.98	1.86/1.80/1.85
6	O2C2	60	-5.18/-5.26/-4.16	1.86/1.80/1.85
7	O2C2	120	-5.49/-5.38/-4.52	1.85/1.80/1.84
8	O2C2	180	-5.54/-5.42/-4.71	1.85/1.80/1.84
9	O2C2	240	-5.49/-5.39/-4.52	1.85/1.80/1.84
10	O2C2	300	-5.18/-5.26/-4.16	1.86/1.80/1.85
11	O4C4	0	-5.76/-5.52/-4.06	1.85/1.78/1.83
12	O4C4	60	-5.76/-5.63/-4.36	1.85/1.78/1.82
13	O4C4	120	-5.58/-5.76/-4.92	1.85/1.78/1.81
14	O4C4	180	-5.40/-5.76/-5.17	1.86/1.78/1.81
15	O4C4	240	-5.58/-5.75/-4.92	1.85/1.78/1.81
16	O4C4	300	-5.76/-5.63/-4.36	1.85/1.78/1.82

1.16 N4-acetylcytosine (4AC)

Figure 16: The molecule used for water complex calculations corresponding to N4-acetylcytosine (4AC), with possible interacting water positions. NOTE, only one water molecule was included in each calculation

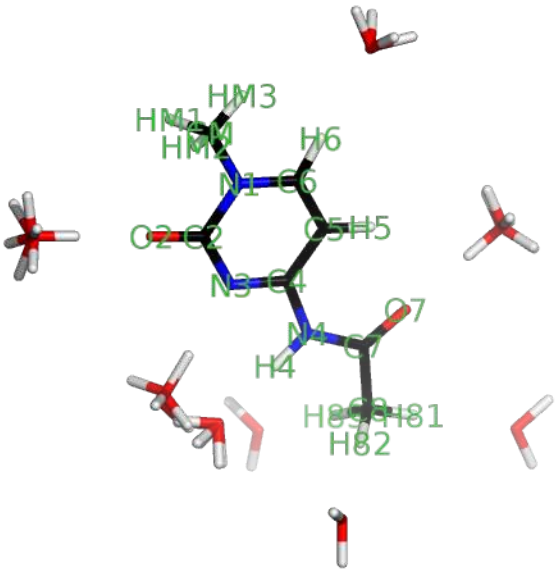

Table 46: Statistics of calculated water interaction and dipole moment for N4-acetylcytosine (4AC). QM is in HF level except for thio-compounds.

	RMS/Max Deviation from QM		Dipole moment	Dipole angle
	Energy (kcal/mol)	Distance (Å)	QM/MM (debye)	difference (°)
Original	1.61/3.36	0.10/0.20	3.25/3.49	8.53
Optimized	0.33/0.61	0.06/0.13	3.25/5.80	11.69

Table 47: The comparison list of optimized atomic charges and their initial guess for N4-acetylcytosine (4AC), referring to the penalties of initial guess

	Charges		
Atom	Optima	Initial	Penalty
N1	-0.090	-0.136	4.542
C2	0.540	0.511	5.350
O2	-0.470	-0.480	0.000
N3	-0.800	-0.666	4.988
C4	0.640	0.884	4.363
N4	-0.640	-0.617	4.607
H4	0.370	0.319	0.425
C5	-0.090	-0.066	2.439
H5	0.090	0.025	0.300
C6	0.130	0.057	2.191
H6	0.160	0.170	0.000
C7	0.590	0.551	0.477
O7	-0.490	-0.550	0.000
C8	-0.210	-0.272	0.000
H81	0.090	0.090	0.000
H82	0.090	0.090	0.000
H83	0.090	0.090	0.000
CM	-0.270	-0.270	3.605
HM1	0.090	0.090	0.000
HM2	0.090	0.090	0.000
HM3	0.090	0.090	0.000

Table 48: Interaction energies and geometries between probe water and selected N4-acetylcytosine (4AC) site calculated using the optimized and initial charges

			QM / Optima / Initial	
N	Probe site	Angle (°)	Energy (kcal/mol)	Distance (Å)
1	H4N4	0	-7.27/-6.71/-7.01	1.78/1.85/1.87
2	H4N4	60	-6.12/-6.11/-6.47	1.82/1.86/1.88
3	H4N4	120	-6.12/-6.13/-6.47	1.82/1.86/1.88
4	H6C6	0	-4.56/-5.08/-4.82	2.34/2.31/2.32
5	H6C6	90	-5.09/-5.10/-4.80	2.29/2.31/2.32
6	H81C8	0	-1.89/-1.54/-0.71	2.48/2.61/2.67
7	H82C8	0	-3.18/-2.78/-2.16	2.47/2.56/2.62
8	H83C8	0	-3.18/-2.78/-2.16	2.47/2.56/2.62
9	O7C7	0	-3.85/-3.63/-5.02	1.88/1.80/1.76
10	O7C7	60	-4.83/-4.63/-5.93	1.84/1.78/1.75
11	O7C7	120	-5.58/-5.76/-7.13	1.83/1.77/1.74
12	O7C7	180	-5.49/-6.11/-7.53	1.85/1.77/1.74
13	O7C7	240	-5.58/-5.78/-7.13	1.83/1.77/1.74
14	O7C7	300	-4.83/-4.62/-5.93	1.84/1.78/1.75
15	N3C4	60	-8.65/-9.04/-5.63	1.84/1.89/1.97
16	N3C4	120	-5.36/-4.82/-2.00	1.91/1.95/2.06
17	N3C4	180	-2.34/-1.89/0.26	2.08/2.04/2.22
18	N3C4	240	-5.36/-4.82/-2.00	1.91/1.95/2.06
19	N3C4	300	-8.65/-9.04/-5.63	1.84/1.89/1.97
20	O2C2	0	-5.98/-6.20/-6.25	1.83/1.78/1.77
21	O2C2	60	-6.38/-6.46/-6.43	1.83/1.77/1.77
22	O2C2	120	-7.05/-7.05/-6.77	1.81/1.77/1.77
23	O2C2	180	-7.36/-7.32/-6.94	1.80/1.76/1.76
24	O2C2	240	-7.05/-7.04/-6.77	1.81/1.77/1.77
25	O2C2	300	-6.38/-6.46/-6.43	1.83/1.78/1.77

1.17 N4-methylcytosine (4MC)

Figure 17: The molecule used for water complex calculations corresponding to N4-methylcytosine (4MC), with possible interacting water positions. NOTE, only one water molecule was included in each calculation

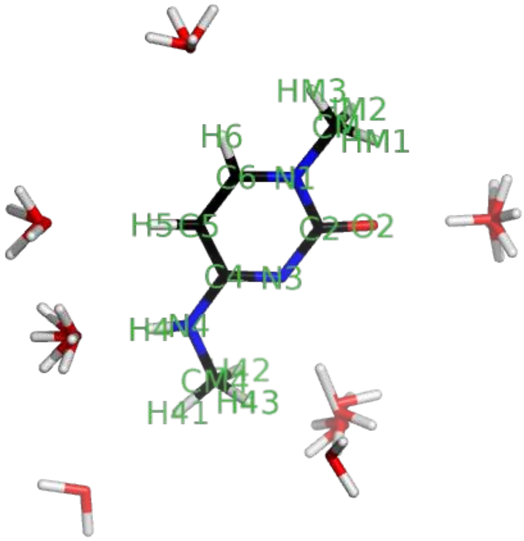

Table 49: Statistics of calculated water interaction and dipole moment for N4-methylcytosine (4MC). QM is in HF level except for thio-compounds.

	RMS/Max Deviation from QM		Dipole moment	Dipole angle
	Energy (kcal/mol)	Distance (Å)	QM/MM (debye)	difference (°)
Original	0.54/1.48	0.10/0.33	6.78/8.95	6.14
Optimized	0.19/0.39	0.10/0.31	6.78/8.50	7.13

Table 50: The comparison list of optimized atomic charges and their initial guess for N4-methylcytosine (4MC), referring to the penalties of initial guess

	Charges		
Atom	Optima	Initial	Penalty
N1	-0.107	-0.136	4.542
C2	0.520	0.512	5.336
O2	-0.490	-0.480	0.000
N3	-0.681	-0.662	31.009
C4	0.634	0.640	55.633
N4	-0.519	-0.523	49.656
H4	0.318	0.364	3.209
C5	-0.053	-0.049	33.263
H5	0.023	0.025	1.800
C6	0.061	0.056	13.482
H6	0.158	0.170	0.000
CM4	-0.134	-0.187	16.708
H41	0.090	0.090	0.600
H42	0.090	0.090	0.600
H43	0.090	0.090	0.600
CM	-0.270	-0.270	3.605
HM1	0.090	0.090	0.000
HM2	0.090	0.090	0.000
HM3	0.090	0.090	0.000

Table 51: Interaction energies and geometries between probe water and selected N4-methylcytosine (4MC) site calculated using the optimized and initial charges

			QM / Optima / Initial	
N	Probe site	Angle (°)	Energy (kcal/mol)	Distance (Å)
1	H4N4	0	-5.56/-5.86/-7.04	1.95/2.05/2.02
2	H4N4	60	-5.92/-5.98/-7.20	1.93/2.05/2.01
3	H4N4	120	-6.45/-6.13/-7.29	1.91/2.04/2.01
4	H5C5	0	-2.81/-3.06/-3.37	2.58/2.48/2.47
5	H5C5	90	-3.56/-3.33/-3.68	2.44/2.45/2.44
6	H6C6	0	-4.18/-4.51/-4.68	2.38/2.34/2.33
7	H6C6	90	-4.80/-4.58/-4.75	2.32/2.33/2.33
8	H41CM4	0	-1.61/-1.21/-1.03	2.72/2.71/2.75
9	H43CM4	0	-0.36/-0.23/-0.03	2.78/2.73/2.78
10	N3C4	0	-4.80/-4.66/-4.54	2.47/2.78/2.80
11	N3C4	60	-3.61/-3.67/-3.63	2.67/2.83/2.85
12	N3C4	120	-2.01/-1.85/-2.06	2.86/2.95/2.95
13	N3C4	180	-1.03/-1.00/-1.40	3.03/3.06/3.01
14	N3C4	240	-1.92/-2.00/-2.20	2.92/2.95/2.96
15	N3C4	300	-3.83/-3.82/-3.76	2.67/2.82/2.85
16	O2C2	0	-6.40/-6.60/-6.51	1.82/1.77/1.77
17	O2C2	60	-6.80/-6.89/-6.77	1.81/1.76/1.77
18	O2C2	120	-7.52/-7.46/-7.34	1.80/1.76/1.76
19	O2C2	180	-7.91/-7.77/-7.65	1.79/1.75/1.76
20	O2C2	240	-7.56/-7.50/-7.38	1.80/1.76/1.76
21	O2C2	300	-6.80/-6.91/-6.81	1.81/1.76/1.77

1.18 4-thiouracil (4SU)

Figure 18: The molecule used for water complex calculations corresponding to 4-thiouracil (4SU), with possible interacting water positions. NOTE, only one water molecule was included in each calculation

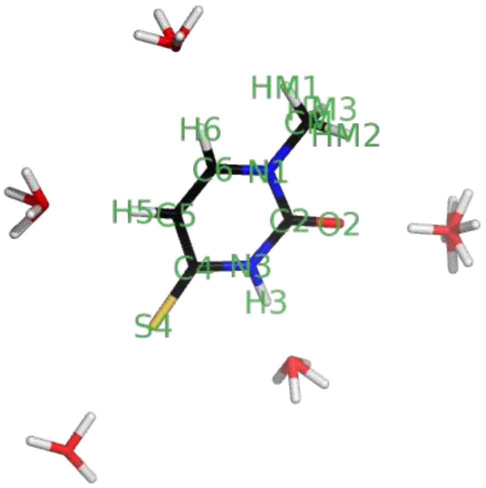

Table 52: Statistics of calculated water interaction and dipole moment for 4-thiouracil (4SU). QM is in HF level except for thio-compounds.

	RMS/Max Deviation from QM		Dipole moment	Dipole angle
	Energy (kcal/mol)	Distance (Å)	QM/MM (debye)	difference (°)
Original	1.08/1.69	0.18/0.37	5.23/2.61	38.53
Optimized	0.28/0.54	0.10/0.16	5.23/4.75	46.52

Table 53: The comparison list of optimized atomic charges and their initial guess for 4-thiouracil (4SU), referring to the penalties of initial guess

	Charges		
Atom	Optima	Initial	Penalty
N1	-0.186	-0.337	0.000
C2	0.433	0.504	2.500
O2	-0.396	-0.410	0.000
N3	-0.647	-0.281	46.932
C4	0.326	0.091	2.500
S4	-0.252	-0.255	107.824
C5	-0.195	-0.096	106.802
C6	0.131	0.204	26.696
H3	0.421	0.362	2.500
H5	0.155	0.077	2.500
H6	0.210	0.141	0.000
CM	-0.270	-0.270	0.000
HM1	0.090	0.090	0.000
HM2	0.090	0.090	0.000
HM3	0.090	0.090	0.000

Table 54: Interaction energies and geometries between probe water and selected 4-thiouracil (4SU) site calculated using the optimized and initial charges

			QM / Optima / Initial	
	Probe	Angle	Energy	Distance
N	site	(°)	(kcal/mol)	(Å)
1	H5C5	0	-3.49/-3.46/-2.02	2.21/2.37/2.45
2	H5C5	90	-3.23/-3.45/-1.96	2.22/2.37/2.46
3	H6C6	0	-5.00/-5.55/-4.13	2.24/2.30/2.34
4	H6C6	90	-5.85/-5.63/-4.16	2.18/2.29/2.34
5	H3N3	60	-7.09/-6.92/-8.78	1.68/1.80/1.79
6	H3N3	120	-7.09/-6.92/-8.78	1.68/1.80/1.78
7	O2C2	0	-4.60/-5.07/-4.84	1.83/1.81/1.81
8	O2C2	60	-4.83/-5.09/-4.70	1.83/1.81/1.82
9	O2C2	120	-5.14/-5.02/-4.32	1.82/1.81/1.83
10	O2C2	180	-5.23/-4.92/-4.08	1.82/1.82/1.83
11	O2C2	240	-5.14/-5.02/-4.32	1.82/1.81/1.83
12	O2C2	300	-4.83/-5.09/-4.70	1.83/1.81/1.82
13	S4C4	0	-1.72/-1.92/-2.33	2.60/2.46/2.43
14	S4C4	180	-1.76/-2.16/-2.14	2.60/2.45/2.44

1.19 5-formylcytosine (5FC)

Figure 19: The molecule used for water complex calculations corresponding to 5-formylcytosine (5FC), with possible interacting water positions. NOTE, only one water molecule was included in each calculation

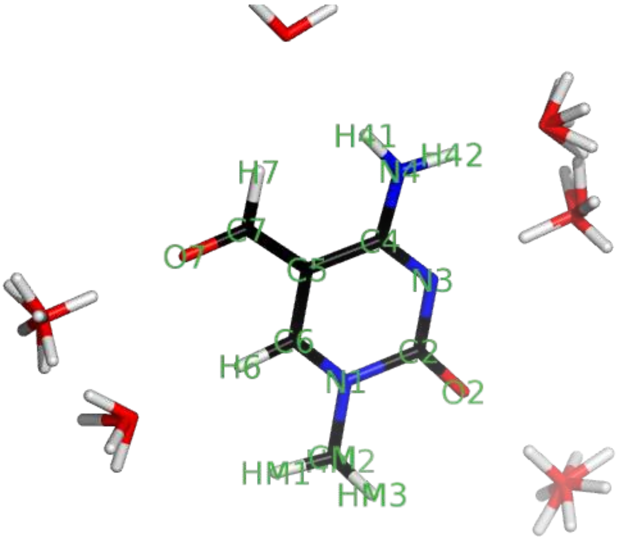

Table 55: Statistics of calculated water interaction and dipole moment for 5-formylcytosine (5FC). QM is in HF level except for thio-compounds.

	RMS/Max Deviation from QM		Dipole moment	Dipole angle
	Energy (kcal/mol)	Distance (Å)	QM/MM (debye)	difference (°)
Original	1.09/1.80	0.11/0.39	3.14/6.87	12.22
Optimized	0.28/0.77	0.10/0.34	3.14/4.91	8.10

Table 56: The comparison list of optimized atomic charges and their initial guess for 5-formylcytosine (5FC), referring to the penalties of initial guess

	Charges		
Atom	Optima	Initial	Penalty
N1	-0.080	-0.100	4.799
C2	0.620	0.505	4.714
O2	-0.470	-0.485	0.000
N3	-0.830	-0.673	17.453
C4	0.610	0.614	17.736
N4	-0.750	-0.770	23.762
H41	0.370	0.345	0.000
H42	0.370	0.345	0.000
C5	0.040	0.100	25.628
C6	0.150	0.053	2.815
H6	0.170	0.135	2.787
C7	0.170	0.266	21.126
H7	0.080	0.080	0.549
O7	-0.450	-0.415	0.552
CM	-0.270	-0.270	3.605
HM1	0.090	0.090	0.000
HM2	0.090	0.090	0.000
HM3	0.090	0.090	0.000

Table 57: Interaction energies and geometries between probe water and selected 5-formylcytosine (5FC) site calculated using the optimized and initial charges

			QM / Optima / Initial	
N	Probe site	Angle (°)	Energy (kcal/mol)	Distance (Å)
1	H42N4	0	-6.47/-5.96/-5.59	1.80/1.85/1.87
2	H42N4	60	-5.67/-5.41/-5.09	1.82/1.86/1.88
3	H42N4	120	-5.67/-5.41/-5.09	1.82/1.86/1.88
4	H6C6	0	-4.60/-4.41/-3.36	2.18/2.29/2.34
5	H6C6	90	-3.18/-3.66/-2.77	2.31/2.32/2.37
6	H7C7	0	-4.69/-5.46/-6.49	2.62/2.27/2.22
7	O7C7	0	-3.89/-3.90/-3.25	1.91/1.81/1.84
8	O7C7	60	-4.38/-4.45/-3.54	1.89/1.79/1.83
9	O7C7	120	-5.05/-5.19/-3.90	1.87/1.78/1.82
10	O7C7	180	-5.23/-5.47/-4.02	1.87/1.78/1.82
11	O7C7	240	-5.05/-5.19/-3.90	1.87/1.78/1.82
12	O7C7	300	-4.38/-4.45/-3.54	1.89/1.80/1.83
13	N3C4	60	-8.29/-8.55/-7.09	1.86/1.90/1.95
14	N3C4	120	-5.54/-5.19/-3.74	1.92/1.95/2.01
15	N3C4	180	-3.01/-2.82/-1.59	2.06/2.01/2.09
16	N3C4	240	-5.54/-5.18/-3.74	1.92/1.95/2.01
17	N3C4	300	-8.29/-8.56/-7.09	1.86/1.90/1.95
18	O2C2	0	-5.40/-5.55/-6.50	1.85/1.79/1.77
19	O2C2	60	-5.85/-5.88/-6.73	1.84/1.79/1.77
20	O2C2	120	-6.60/-6.54/-7.19	1.83/1.78/1.76
21	O2C2	180	-6.96/-6.88/-7.42	1.82/1.77/1.76
22	O2C2	240	-6.60/-6.54/-7.19	1.83/1.78/1.76
23	O2C2	300	-5.85/-5.88/-6.73	1.84/1.79/1.77

1.20 5-hydroxyuracil (5HU)

Figure 20: The molecule used for water complex calculations corresponding to 5-hydroxyuracil (5HU), with possible interacting water positions. NOTE, only one water molecule was included in each calculation

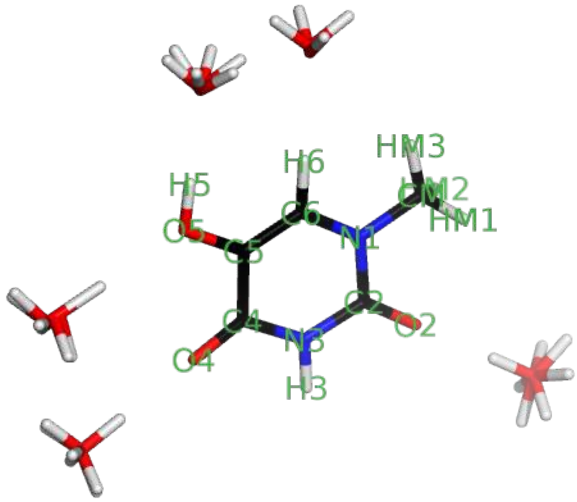

Table 58: Statistics of calculated water interaction and dipole moment for 5-hydroxyuracil (5HU). QM is in HF level except for thio-compounds.

	RMS/Max Deviation from QM		Dipole moment	Dipole angle
	Energy (kcal/mol)	Distance (Å)	QM/MM (debye)	difference (°)
Original	0.68/1.77	0.10/0.19	6.21/4.77	21.07
Optimized	0.27/0.82	0.12/0.23	6.21/5.91	15.37

Table 59: The comparison list of optimized atomic charges and their initial guess for 5-hydroxyuracil (5HU), referring to the penalties of initial guess

	Charges		
Atom	Optima	Initial	Penalty
N1	-0.274	-0.354	15.091
C2	0.545	0.509	0.000
O2	-0.453	-0.410	0.000
N3	-0.492	-0.461	20.934
H3	0.253	0.362	0.000
C4	0.594	0.480	31.270
O4	-0.473	-0.450	22.794
C5	0.132	0.087	24.572
C6	0.242	0.202	15.769
H6	0.132	0.144	3.798
O5	-0.599	-0.529	6.134
H5	0.393	0.420	0.532
CM	-0.270	-0.270	0.000
HM1	0.090	0.090	0.000
HM2	0.090	0.090	0.000
HM3	0.090	0.090	0.000

Table 60: Interaction energies and geometries between probe water and selected 5-hydroxyuracil (5HU) site calculated using the optimized and initial charges

			QM / Optima / Initial	
N	Probe site	Angle (°)	Energy (kcal/mol)	Distance (Å)
1	H5O5	0	-8.25/-8.79/-10.02	1.99/1.83/1.80
2	H5O5	60	-9.63/-9.45/-10.64	1.96/1.82/1.78
3	H5O5	120	-9.63/-9.45/-10.64	1.96/1.82/1.78
4	H6C6	0	-5.27/-6.09/-5.55	2.38/2.31/2.32
5	H6C6	90	-6.60/-6.32/-5.83	2.27/2.30/2.31
6	O2C2	0	-4.83/-5.16/-4.90	1.87/1.80/1.81
7	O2C2	60	-5.09/-5.22/-4.83	1.87/1.80/1.81
8	O2C2	120	-5.45/-5.32/-4.63	1.86/1.80/1.82
9	O2C2	180	-5.54/-5.34/-4.48	1.86/1.80/1.83
10	O2C2	240	-5.45/-5.33/-4.63	1.86/1.80/1.82
11	O2C2	300	-5.09/-5.23/-4.83	1.87/1.80/1.81
12	O4C4	0	-5.67/-5.69/-5.71	1.87/1.79/1.79
13	O4C4	60	-5.45/-5.48/-5.48	1.88/1.80/1.80
14	O4C4	120	-5.14/-5.16/-5.06	1.90/1.81/1.81
15	O4C4	180	-5.00/-5.05/-4.83	1.90/1.81/1.81
16	O4C4	240	-5.14/-5.16/-5.06	1.90/1.81/1.81
17	O4C4	300	-5.45/-5.48/-5.48	1.88/1.80/1.80
18	O5C5	0	-2.38/-1.90/-1.37	2.30/2.11/2.21
19	O5C5	60	-2.78/-2.65/-2.16	2.30/2.07/2.15
20	O5C5	120	-4.87/-5.00/-4.57	2.13/1.98/2.02
21	O5C5	180	-7.18/-7.33/-6.87	2.00/1.90/1.92
22	O5C5	240	-4.87/-5.00/-4.57	2.13/1.98/2.02
23	O5C5	300	-2.78/-2.65/-2.16	2.30/2.07/2.15

1.21 5-methylcytosine (5MC)

Figure 21: The molecule used for water complex calculations corresponding to 5-methylcytosine (5MC), with possible interacting water positions. NOTE, only one water molecule was included in each calculation

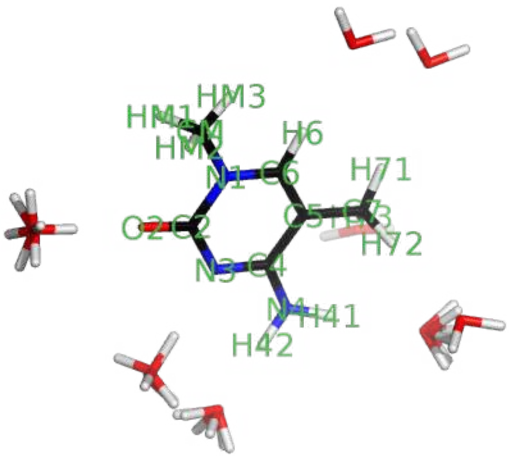

Table 61: Statistics of calculated water interaction and dipole moment for 5-methylcytosine (5MC). QM is in HF level except for thio-compounds.

	RMS/Max Deviation from QM		Dipole moment	Dipole angle
	Energy (kcal/mol)	Distance (Å)	QM/MM (debye)	difference (°)
Original	0.91/1.98	0.06/0.12	7.08/9.46	13.82
Optimized	0.43/0.81	0.04/0.09	7.08/9.31	10.59

Table 62: The comparison list of optimized atomic charges and their initial guess for 5-methylcytosine (5MC), referring to the penalties of initial guess

	Charges		
Atom	Optima	Initial	Penalty
N1	-0.120	-0.139	4.542
C2	0.500	0.511	4.714
O2	-0.451	-0.480	0.000
N3	-0.792	-0.666	12.529
C4	0.620	0.640	19.504
N4	-0.643	-0.755	15.051
H41	0.316	0.350	0.000
H42	0.316	0.350	0.000
C5	0.052	-0.127	13.291
C6	0.020	0.018	0.000
H6	0.190	0.199	0.000
C7	-0.218	-0.171	2.119
H71	0.070	0.090	0.025
H72	0.070	0.090	0.025
H73	0.070	0.090	0.025
CM	-0.270	-0.270	3.605
HM1	0.090	0.090	0.000
HM2	0.090	0.090	0.000
HM3	0.090	0.090	0.000

Table 63: Interaction energies and geometries between probe water and selected 5-methylcytosine (5MC) site calculated using the optimized and initial charges

			QM / Optima / Initial	
N	Probe site	Angle (°)	Energy (kcal/mol)	Distance (Å)
1	H41N4	0	-3.96/-4.70/-4.83	2.31/2.30/2.32
2	H41N4	60	-4.49/-5.02/-5.29	2.28/2.26/2.26
3	H41N4	120	-4.54/-4.87/-4.98	2.23/2.28/2.29
4	H42N4	0	-5.29/-4.81/-5.34	1.83/1.88/1.87
5	H42N4	60	-4.49/-4.28/-4.88	1.87/1.89/1.88
6	H42N4	120	-4.23/-4.08/-4.72	1.87/1.90/1.88
7	H6C6	0	-4.23/-5.03/-4.72	2.39/2.32/2.33
8	H71C7	0	-2.36/-2.87/-3.12	2.72/2.64/2.61
9	H72C7	0	-2.85/-3.14/-3.42	2.59/2.59/2.57
10	H73C7	0	-1.96/-2.45/-2.61	2.58/2.60/2.58
11	O2C2	0	-7.91/-7.53/-7.70	1.79/1.76/1.75
12	O2C2	60	-7.52/-7.16/-7.41	1.80/1.77/1.76
13	O2C2	120	-6.76/-6.56/-6.93	1.81/1.78/1.77
14	O2C2	180	-6.40/-6.28/-6.73	1.82/1.78/1.77
15	O2C2	240	-6.80/-6.62/-7.02	1.81/1.78/1.77
16	O2C2	300	-7.60/-7.27/-7.51	1.80/1.77/1.76
17	N3C4	60	-8.89/-9.12/-7.69	1.86/1.90/1.94
18	N3C4	120	-6.58/-5.94/-4.60	1.90/1.95/1.99
19	N3C4	180	-3.83/-3.39/-2.07	2.04/2.01/2.08
20	N3C4	240	-5.65/-5.10/-3.71	1.93/1.96/2.02
21	N3C4	300	-8.58/-8.60/-7.12	1.86/1.91/1.95

1.22 N6-acetyladenine (6AA)

Figure 22: The molecule used for water complex calculations corresponding to N6-acetyladenine (6AA), with possible interacting water positions. NOTE, only one water molecule was included in each calculation

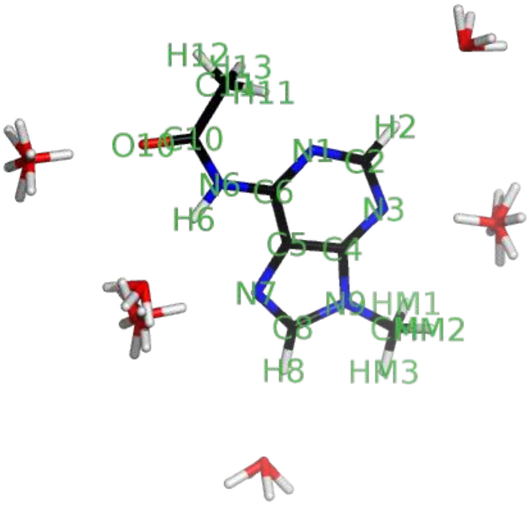

Table 64: Statistics of calculated water interaction and dipole moment for N6-acetyladenine (6AA). QM is in HF level except for thio-compounds.

	RMS/Max Deviation from QM		Dipole moment	Dipole angle
	Energy (kcal/mol)	Distance (Å)	QM/MM (debye)	difference (°)
Original	1.06/1.53	0.08/0.14	6.20/6.88	6.31
Optimized	0.30/0.78	0.07/0.14	6.20/6.99	9.35

Table 65: The comparison list of optimized atomic charges and their initial guess for N6-acetyladenine (6AA), referring to the penalties of initial guess

	Charges		
Atom	Optima	Initial	Penalty
N9	-0.010	-0.010	0.000
C8	0.398	0.330	0.000
H8	0.090	0.121	0.000
N7	-0.816	-0.711	3.308
C5	0.347	0.284	5.017
C6	0.699	0.716	6.186
N6	-0.681	-0.591	5.878
H6	0.326	0.317	0.685
N1	-0.684	-0.749	3.983
C2	0.387	0.498	3.927
H2	0.146	0.125	0.000
N3	-0.820	-0.750	0.000
C4	0.590	0.427	2.840
C10	0.464	0.549	0.768
O10	-0.442	-0.552	0.000
C11	-0.264	-0.274	0.000
H11	0.090	0.090	0.000
H12	0.090	0.090	0.000
H13	0.090	0.090	0.000
CM	-0.270	-0.270	0.000
HM1	0.090	0.090	0.000
HM2	0.090	0.090	0.000
HM3	0.090	0.090	0.000

Table 66: Interaction energies and geometries between probe water and selected N6-acetyladenine (6AA) site calculated using the optimized and initial charges

			QM / Optima / Initial	
N	Probe site	Angle (°)	Energy (kcal/mol)	Distance (Å)
1	H6N6	60	-0.34/-0.17/-1.02	1.96/2.10/2.06
2	H6N6	120	-0.34/-0.17/-1.02	1.96/2.10/2.06
3	H2C2	0	-2.07/-2.05/-2.40	2.46/2.41/2.40
4	H2C2	90	-1.98/-1.92/-2.26	2.48/2.42/2.41
5	H8C8	0	-4.07/-4.45/-4.68	2.34/2.21/2.20
6	H8C8	90	-4.43/-4.41/-4.66	2.30/2.22/2.20
7	O10C10	0	-5.54/-5.54/-6.80	1.87/1.80/1.75
8	O10C10	60	-5.58/-5.64/-6.95	1.87/1.80/1.75
9	O10C10	120	-5.67/-5.78/-7.17	1.86/1.79/1.75
10	O10C10	180	-5.72/-5.82/-7.25	1.86/1.79/1.75
11	O10C10	240	-5.67/-5.78/-7.17	1.86/1.79/1.75
12	O10C10	300	-5.58/-5.64/-6.95	1.87/1.79/1.75
13	N3C4	0	-6.60/-6.50/-5.60	1.94/1.95/1.98
14	N3C4	60	-6.69/-6.28/-5.56	1.92/1.94/1.97
15	N3C4	120	-5.36/-5.26/-4.95	1.95/1.96/1.98
16	N3C4	180	-3.85/-4.50/-4.42	2.03/1.97/1.98
17	N3C4	240	-5.36/-5.26/-4.95	1.95/1.96/1.98
18	N3C4	300	-6.69/-6.28/-5.56	1.92/1.94/1.97
19	N7C5	0	-3.98/-4.77/-3.73	1.90/1.87/1.89
20	N7C5	60	-5.32/-5.05/-4.00	1.86/1.87/1.90
21	N7C5	120	-5.63/-5.22/-4.13	1.90/1.90/1.93
22	N7C5	180	-4.78/-4.93/-3.91	2.06/1.96/2.01
23	N7C5	240	-5.63/-5.23/-4.13	1.90/1.90/1.93
24	N7C5	300	-5.32/-5.06/-4.00	1.86/1.87/1.90

1.23 N6-methyladenine (6MA)

Figure 23: The molecule used for water complex calculations corresponding to N6-methyladenine (6MA), with possible interacting water positions. NOTE, only one water molecule was included in each calculation

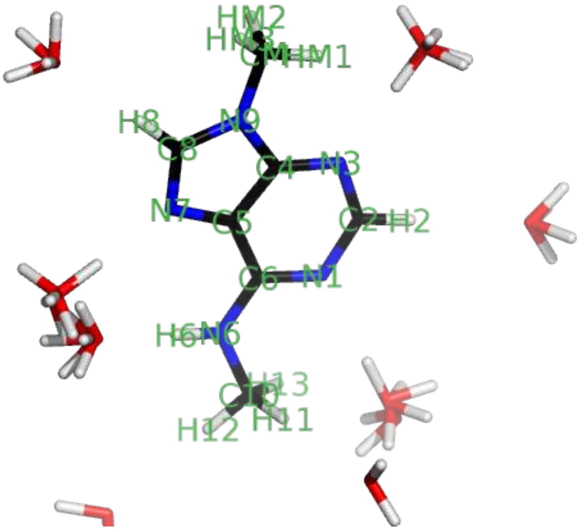

Table 67: Statistics of calculated water interaction and dipole moment for N6-methyladenine (6MA). QM is in HF level except for thio-compounds.

	RMS/Max Deviation from QM		Dipole moment	Dipole angle
	Energy (kcal/mol)	Distance (Å)	QM/MM (debye)	difference (°)
Original	1.30/2.41	0.16/0.39	2.54/4.43	5.86
Optimized	0.30/0.74	0.12/0.31	2.54/3.06	27.23

Table 68: The comparison list of optimized atomic charges and their initial guess for N6-methyladenine (6MA), referring to the penalties of initial guess

	Charges		
Atom	Optima	Initial	Penalty
N9	-0.000	-0.000	0.000
C8	0.431	0.340	0.000
H8	0.078	0.119	0.000
N7	-0.863	-0.708	2.500
C5	0.319	0.282	21.933
C6	0.453	0.567	46.368
N6	-0.452	-0.584	40.152
H6	0.334	0.360	3.233
N1	-0.782	-0.763	30.744
C2	0.487	0.510	2.500
H2	0.140	0.123	0.000
N3	-0.860	-0.752	0.000
C4	0.528	0.427	2.500
C10	-0.083	-0.191	17.069
H11	0.090	0.090	0.600
H12	0.090	0.090	0.600
H13	0.090	0.090	0.600
CM	-0.270	-0.270	0.000
HM1	0.090	0.090	0.000
HM2	0.090	0.090	0.000
HM3	0.090	0.090	0.000

Table 69: Interaction energies and geometries between probe water and selected N6-methyladenine (6MA) site calculated using the optimized and initial charges

			QM / Optima / Initial	
N	Probe site	Angle (°)	Energy (kcal/mol)	Distance (Å)
1	H6N6	0	-4.38/-3.64/-3.23	1.81/2.02/2.04
2	H6N6	60	-2.87/-2.90/-2.72	1.88/2.05/2.07
3	H6N6	120	-1.76/-2.38/-2.32	1.96/2.07/2.09
4	H2C2	0	-1.14/-1.14/-1.51	2.54/2.43/2.42
5	H2C2	90	-1.01/-1.01/-1.36	2.57/2.45/2.43
6	H8C8	0	-3.23/-3.55/-4.40	2.38/2.24/2.21
7	H8C8	90	-3.45/-3.51/-4.37	2.35/2.24/2.21
8	H11C10	0	-0.56/-0.54/na	2.81/2.71/na
9	H12C10	0	-1.54/-1.44/-0.29	2.69/2.66/2.82
10	N1C2	0	-1.94/-1.91/-3.18	2.70/2.66/2.62
11	N1C2	60	-2.65/-2.83/-3.33	2.57/2.59/2.61
12	N1C2	120	-3.89/-3.83/-3.36	2.33/2.53/2.60
13	N1C2	180	-3.98/-3.97/-3.19	2.23/2.53/2.62
14	N1C2	240	-3.67/-3.68/-3.26	2.32/2.54/2.60
15	N1C2	300	-3.09/-2.66/-3.26	2.44/2.58/2.60
16	N3C4	0	-7.05/-7.14/-5.78	1.91/1.96/2.00
17	N3C4	60	-7.27/-7.09/-5.75	1.90/1.95/2.00
18	N3C4	120	-6.52/-6.26/-5.03	1.92/1.96/2.00
19	N3C4	240	-5.49/-5.91/-4.70	1.97/1.97/2.02
20	N3C4	300	-7.45/-6.92/-5.59	1.90/1.96/2.00
21	N7C5	60	-8.03/-8.28/-5.84	1.84/1.85/1.89
22	N7C5	120	-7.49/-6.96/-5.16	1.85/1.86/1.91
23	N7C5	180	-4.60/-4.69/-3.45	1.98/1.91/1.97
24	N7C5	240	-6.47/-6.24/-4.47	1.88/1.87/1.92
25	N7C5	300	-7.89/-7.95/-5.48	1.84/1.85/1.90

1.24 7-methylguanine (7MG)

Figure 24: The molecule used for water complex calculations corresponding to 7-methylguanine (7MG), with possible interacting water positions. NOTE, only one water molecule was included in each calculation

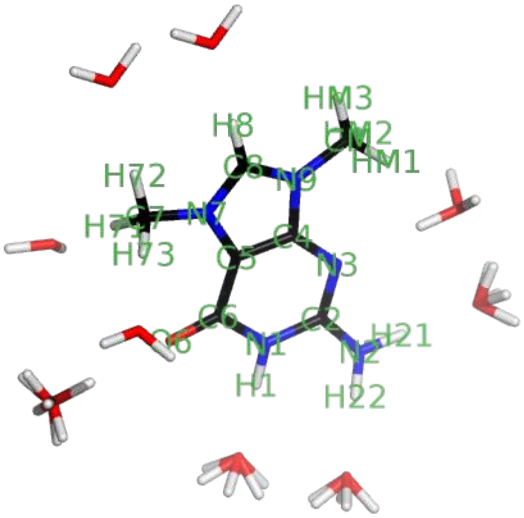

Table 70: Statistics of calculated water interaction and dipole moment for 7-methylguanine (7MG). QM is in HF level except for thio-compounds.

	RMS/Max Deviation from QM		Dipole moment	Dipole angle
	Energy (kcal/mol)	Distance (Å)	QM/MM (debye)	difference (°)
Original	1.35/2.44	0.16/0.27	4.35/2.33	159.06
Optimized	0.23/0.47	0.15/0.24	4.35/1.77	117.01

Table 71: The comparison list of optimized atomic charges and their initial guess for 7-methylguanine (7MG), referring to the penalties of initial guess

	Charges		
Atom	Optima	Initial	Penalty
N9	-0.103	-0.211	112.765
C8	0.398	0.358	100.119
H8	0.148	0.180	19.849
N7	-0.399	-0.395	114.033
C5	0.151	0.122	79.191
C6	0.602	0.566	29.008
O6	-0.453	-0.481	14.853
N1	-0.294	-0.314	12.750
H1	0.245	0.290	0.000
C2	0.742	0.757	13.641
N2	-0.689	-0.650	0.000
H21	0.334	0.364	0.000
H22	0.338	0.364	0.000
N3	-0.548	-0.510	27.930
C4	0.156	0.098	78.120
C7	0.102	0.192	109.300
H71	0.090	0.090	3.537
H72	0.090	0.090	3.537
H73	0.090	0.090	3.537
CM	-0.270	-0.270	109.300
HM1	0.090	0.090	3.537
HM2	0.090	0.090	3.537
HM3	0.090	0.090	3.537

Table 72: Interaction energies and geometries between probe water and selected 7-methylguanine (7MG) site calculated using the optimized and initial charges

			QM / Optima / Initial	
N	Probe site	Angle (°)	Energy (kcal/mol)	Distance (Å)
1	H1N1	0	-11.97/-12.32/-13.73	1.82/1.83/1.80
2	H1N1	60	-12.40/-12.44/-13.94	1.80/1.83/1.80
3	H1N1	120	-12.93/-12.64/-14.17	1.79/1.82/1.79
4	H21N2	0	-9.33/-9.38/-11.62	1.84/1.84/1.80
5	H21N2	60	-9.49/-9.25/-11.43	1.85/1.85/1.80
6	H21N2	120	-9.37/-9.27/-11.45	1.85/1.85/1.80
7	H22N2	0	-11.44/-11.73/-13.87	1.88/1.84/1.80
8	H22N2	60	-11.94/-12.01/-14.18	1.86/1.83/1.80
9	H22N2	120	-12.74/-12.27/-14.49	1.83/1.83/1.79
10	H8C8	0	-10.90/-10.98/-10.85	2.07/1.99/1.99
11	H71C7	0	-5.23/-5.59/-5.69	2.29/2.53/2.51
12	H72C7	0	-6.88/-7.12/-7.54	2.38/2.51/2.49
13	H73C7	0	-5.27/-5.63/-5.74	2.29/2.52/2.51
14	O6C6	0	-0.75/-0.48/-1.03	2.11/1.86/1.83
15	O6C6	60	-1.33/-1.22/-1.86	2.08/1.85/1.82
16	O6C6	120	-2.02/-2.10/-2.79	2.06/1.83/1.80
17	O6C6	180	-2.09/-2.31/-2.96	2.06/1.83/1.80
18	O6C6	240	-1.98/-2.06/-2.74	2.06/1.83/1.80
19	O6C6	300	-1.33/-1.18/-1.82	2.08/1.85/1.82
20	N3C4	120	-1.40/-1.67/-2.08	2.10/2.09/2.08
21	N3C4	240	-1.29/-1.43/-1.84	2.10/2.09/2.09

1.25 zwitterionic alanine (alai, for 3AU, 13P)

Figure 25: The molecule used for water complex calculations corresponding to zwitterionic alanine (alai, for 3AU, 13P), with possible interacting water positions. NOTE, only one water molecule was included in each calculation

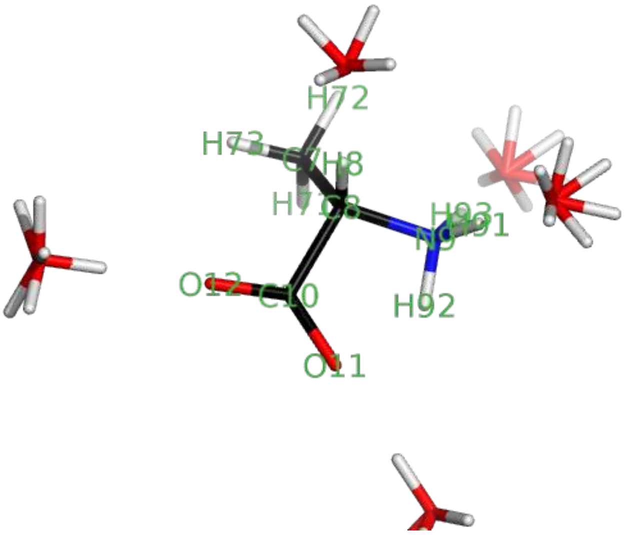

Table 73: Statistics of calculated water interaction and dipole moment for zwitterionic alanine (alai, for 3AU, 13P). QM is in HF level except for thio-compounds.

	RMS/Max Deviation from QM		Dipole moment	Dipole angle
	Energy (kcal/mol)	Distance (Å)	QM/MM (debye)	difference (°)
Original	3.01/4.24	0.08/0.16	10.50/14.62	0.76
Optimized	0.33/0.63	0.07/0.19	10.50/11.49	2.11

Table 74: The comparison list of optimized atomic charges and their initial guess for zwitterionic alanine (alai, for 3AU, 13P), referring to the penalties of initial guess

	Charges		
Atom	Optima	Initial	Penalty
C7	-0.270	-0.270	31.192
H71	0.090	0.090	2.536
H72	0.090	0.090	2.536
H73	0.090	0.090	2.536
C8	0.159	0.343	104.975
H8	0.122	0.089	4.013
N9	-0.347	-0.342	36.591
H91	0.298	0.331	2.536
H92	0.298	0.331	2.536
H93	0.298	0.331	2.536
C10	0.306	0.435	102.378
O11	-0.567	-0.759	8.442
O12	-0.567	-0.759	8.442

Table 75: Interaction energies and geometries between probe water and selected zwitterionic alanine (alai, for 3AU, 13P) site calculated using the optimized and initial charges

			QM / Optima / Initial	
	Probe	Angle	Energy	Distance
N	site	(°)	(kcal/mol)	(Å)
1	H91N9	0	-11.29/-11.24/-14.02	1.74/1.79/1.75
2	H91N9	60	-11.62/-11.48/-14.32	1.73/1.78/1.74
3	H91N9	120	-11.54/-11.34/-14.14	1.73/1.79/1.75
4	H93N9	0	-11.00/-10.96/-13.85	1.76/1.81/1.76
5	H93N9	60	-11.05/-11.01/-13.97	1.76/1.81/1.76
6	H93N9	120	-11.00/-11.10/-14.02	1.76/1.80/1.76
7	H8C8	0	-3.81/-3.39/-4.22	2.37/2.55/2.52
8	H8C8	90	-3.16/-3.14/-3.88	2.44/2.57/2.54
9	O11C10	120	-7.25/-6.62/-9.34	1.74/1.73/1.66
10	O11C10	180	-9.29/-9.43/-13.04	1.72/1.70/1.64
11	O11C10	240	-9.54/-9.95/-13.66	1.72/1.69/1.63
12	O11C10	300	-8.11/-7.82/-10.86	1.73/1.71/1.65
13	O12C10	0	-7.52/-7.88/-10.70	1.78/1.73/1.67
14	O12C10	120	-9.25/-9.87/-13.49	1.75/1.71/1.65
15	O12C10	180	-9.23/-9.75/-13.39	1.76/1.71/1.65
16	O12C10	240	-8.40/-8.75/-11.97	1.77/1.72/1.66
17	O12C10	300	-7.60/-7.78/-10.61	1.78/1.73/1.67

1.26 3-ammonio-cyclopentene (cpe, for QUG, MQ, GQG)

Figure 26: The molecule used for water complex calculations corresponding to 3-ammonio-cyclopentene (cpe, for QUG, MQ, GQG), with possible interacting water positions. NOTE, only one water molecule was included in each calculation

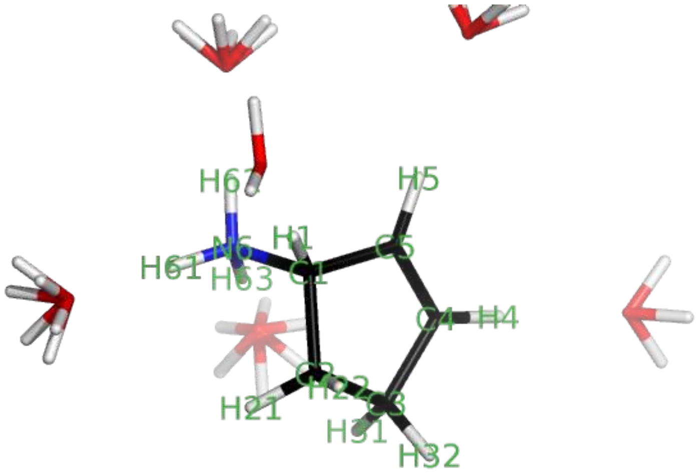

Table 76: Statistics of calculated water interaction and dipole moment for 3-ammonio-cyclopentene (cpe, for QUG, MQ, GQG). QM is in HF level except for thio-compounds.

	RMS/Max Deviation from QM		Dipole moment	Dipole angle
	Energy (kcal/mol)	Distance (Å)	QM/MM (debye)	difference (°)
Original	3.23/4.25	0.14/0.28	5.61/10.33	9.21
Optimized	0.21/0.69	0.13/0.26	5.61/6.59	11.37

Table 77: The comparison list of optimized atomic charges and their initial guess for 3-ammonio-cyclopentene (cpe, for QUG, MQ, GQG), referring to the penalties of initial guess

	Charges		
Atom	Optima	Initial	Penalty
C1	0.336	0.089	57.537
H1	0.090	0.090	0.854
C2	-0.180	-0.180	46.652
H21	0.090	0.090	2.500
H22	0.090	0.090	2.500
C3	-0.180	-0.180	15.887
H31	0.090	0.090	0.000
H32	0.090	0.090	0.000
C4	-0.203	-0.437	3.218
H4	0.265	0.293	0.228
C5	-0.239	-0.244	44.960
H5	0.176	0.277	3.715
N6	-0.319	-0.004	18.236
H61	0.298	0.312	10.007
H62	0.298	0.312	10.007
H63	0.298	0.312	10.007

Table 78: Interaction energies and geometries between probe water and selected 3-ammonio-cyclopentene (cpe, for QUG, MQ, GQG) site calculated using the optimized and initial charges

			QM / Optima / Initial	
N	Probe site	Angle (°)	Energy (kcal/mol)	Distance (Å)
1	H61N6	0	-17.14/-17.17/-21.26	1.73/1.75/1.70
2	H61N6	60	-17.21/-17.25/-21.27	1.73/1.75/1.70
3	H61N6	120	-17.00/-17.21/-21.25	1.74/1.75/1.70
4	H62N6	0	-16.94/-16.95/-21.09	1.73/1.76/1.71
5	H62N6	60	-17.06/-17.02/-21.14	1.73/1.75/1.71
6	H62N6	120	-17.19/-17.11/-21.34	1.73/1.75/1.71
7	H63N6	0	-15.35/-15.57/-19.43	1.77/1.78/1.72
8	H63N6	60	-15.53/-15.52/-18.82	1.78/1.78/1.73
9	H63N6	120	-16.35/-15.66/-19.12	1.76/1.78/1.72
10	H1C1	0	-8.79/-8.68/-8.21	2.23/2.49/2.51
11	H4C4	0	-6.47/-6.42/-5.06	2.27/2.48/2.53
12	H4C4	90	-6.60/-6.44/-5.11	2.26/2.48/2.52
13	H5C5	0	-6.85/-6.88/-7.91	2.32/2.52/2.47
14	H5C5	90	-7.14/-7.02/-8.04	2.29/2.51/2.46

1.27 3-ammonio-epoxycyclopentane (cpo, for EQG)

Figure 27: The molecule used for water complex calculations corresponding to 3-ammonio-epoxycyclopentane (cpo, for EQG), with possible interacting water positions. NOTE, only one water molecule was included in each calculation

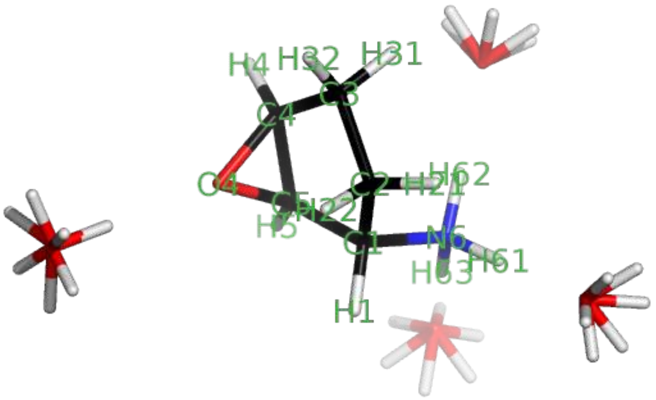

Table 79: Statistics of calculated water interaction and dipole moment for 3-ammonio-epoxycyclopentane (cpo, for EQG). QM is in HF level except for thio-compounds.

	RMS/Max Deviation from QM		Dipole moment	Dipole angle
	Energy (kcal/mol)	Distance (Å)	QM/MM (debye)	difference (°)
Original	3.00/3.99	0.25/0.42	8.80/12.15	5.09
Optimized	0.25/0.48	0.18/0.29	8.80/9.39	3.78

Table 80: The comparison list of optimized atomic charges and their initial guess for 3-ammonio-epoxycyclopentane (cpo, for EQG), referring to the penalties of initial guess

	Charges		
Atom	Optima	Initial	Penalty
C1	0.323	0.014	45.287
H1	0.090	0.090	3.663
C2	-0.180	-0.180	25.051
H21	0.090	0.090	2.500
H22	0.090	0.090	2.500
C3	-0.180	-0.180	21.225
H31	0.090	0.090	1.675
H32	0.090	0.090	1.675
C4	0.156	0.124	99.388
H4	0.090	0.090	3.925
O4	-0.250	-0.396	133.532
C5	-0.098	0.156	104.098
H5	0.090	0.090	4.649
N6	-0.316	-0.287	15.991
H61	0.305	0.373	7.984
H62	0.305	0.373	7.984
H63	0.305	0.373	7.984

Table 81: Interaction energies and geometries between probe water and selected 3-ammonio-epoxycyclopentane (cpo, for EQG) site calculated using the optimized and initial charges

			QM / Optima / Initial	
	Probe	Angle	Energy	Distance
N	site	(°)	(kcal/mol)	(Å)
1	H61N6	0	-18.37/-18.23/-21.39	1.71/1.74/1.70
2	H61N6	60	-18.49/-18.30/-21.35	1.71/1.74/1.70
3	H61N6	120	-18.22/-18.23/-21.29	1.71/1.74/1.70
4	H62N6	0	-15.71/-16.19/-19.70	1.81/1.81/1.75
5	H62N6	60	-16.13/-16.54/-19.98	1.81/1.81/1.75
6	H62N6	120	-16.63/-16.66/-20.00	1.80/1.80/1.74
7	H63N6	0	-18.43/-18.28/-21.98	1.72/1.74/1.70
8	H63N6	60	-18.29/-18.24/-21.84	1.72/1.74/1.70
9	H63N6	120	-18.68/-18.30/-22.03	1.71/1.74/1.70
10	O4C4	0	-0.22/0.06/-2.03	2.17/1.89/1.77
11	O4C4	60	0.20/0.53/-1.90	2.19/1.91/1.77
12	O4C4	120	-0.18/-0.18/-2.71	2.17/1.88/1.76
13	O4C4	180	-0.89/-1.17/-3.52	2.14/1.85/1.75
14	O4C4	240	-1.31/-1.54/-3.61	2.11/1.84/1.75
15	O4C4	300	-1.02/-1.01/-2.92	2.12/1.86/1.76

1.28 7-cyano-7-deazaguanine (DCG)

Figure 28: The molecule used for water complex calculations corresponding to 7-cyano-7-deazaguanine (DCG), with possible interacting water positions. NOTE, only one water molecule was included in each calculation

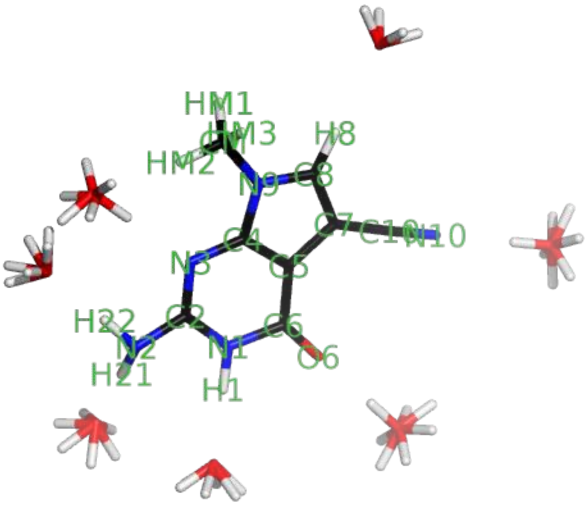

Table 82: Statistics of calculated water interaction and dipole moment for 7-cyano-7-deazaguanine (DCG). QM is in HF level except for thio-compounds.

	RMS/Max Deviation from QM		Dipole moment	Dipole angle
	Energy (kcal/mol)	Distance (Å)	QM/MM (debye)	difference (°)
Original	0.71/1.89	0.14/0.32	10.25/7.62	3.32
Optimized	0.36/0.95	0.14/0.34	10.25/9.89	12.62

Table 83: The comparison list of optimized atomic charges and their initial guess for 7-cyano-7-deazaguanine (DCG), referring to the penalties of initial guess

	Charges		
Atom	Optima	Initial	Penalty
N9	0.180	0.032	25.313
C8	-0.090	-0.074	40.172
H8	0.190	0.189	2.788
C7	-0.100	0.106	77.505
C5	-0.110	-0.106	44.594
C6	0.570	0.480	22.948
O6	-0.510	-0.521	12.532
N1	-0.350	-0.354	10.757
H1	0.260	0.250	0.000
C2	0.770	0.738	0.000
N2	-0.600	-0.690	0.000
H21	0.290	0.324	0.000
H22	0.290	0.324	0.000
N3	-0.730	-0.753	14.276
C4	0.140	0.223	30.901
C10	0.240	0.309	73.236
N10	-0.440	-0.477	4.221
CM	-0.270	-0.270	10.090
HM1	0.090	0.090	0.150
HM2	0.090	0.090	0.150
HM3	0.090	0.090	0.150

Table 84: Interaction energies and geometries between probe water and selected 7-cyano-7-deazaguanine (DCG) site calculated using the optimized and initial charges

			QM / Optima / Initial	
N	Probe site	Angle (°)	Energy (kcal/mol)	Distance (Å)
1	H1N1	0	-8.03/-7.84/-6.43	1.78/1.85/1.88
2	H1N1	60	-7.45/-7.53/-6.13	1.79/1.86/1.89
3	H1N1	120	-8.38/-7.93/-6.49	1.77/1.85/1.88
4	H21N2	0	-8.34/-8.48/-8.12	1.86/1.86/1.86
5	H21N2	60	-7.94/-8.36/-8.01	1.87/1.86/1.86
6	H21N2	120	-8.65/-8.63/-8.23	1.83/1.86/1.85
7	H22N2	0	-5.85/-5.58/-5.31	1.85/1.88/1.88
8	H22N2	60	-5.72/-5.27/-4.95	1.87/1.89/1.89
9	H22N2	120	-5.54/-5.30/-4.99	1.87/1.89/1.89
10	H8C8	0	-4.03/-4.31/-5.08	2.31/2.20/2.18
11	H8C8	90	-4.03/-4.14/-4.91	2.29/2.20/2.18
12	N10C10	0	-5.09/-5.37/-5.01	2.03/1.93/1.93
13	N10C10	60	-5.32/-5.57/-5.25	2.02/1.93/1.92
14	N10C10	120	-5.67/-5.94/-5.74	2.01/1.92/1.92
15	N10C10	180	-5.89/-6.12/-5.99	2.01/1.92/1.91
16	N10C10	240	-5.63/-5.88/-5.68	2.01/1.92/1.92
17	N10C10	300	-5.27/-5.50/-5.20	2.02/1.93/1.92
18	O6C6	0	-7.00/-7.59/-7.53	1.85/1.77/1.76
19	O6C6	60	-6.07/-6.43/-6.64	1.87/1.78/1.77
20	O6C6	120	-4.78/-4.69/-5.35	1.89/1.80/1.78
21	O6C6	180	-4.29/-3.92/-4.78	1.90/1.81/1.79
22	O6C6	240	-4.74/-4.65/-5.30	1.89/1.80/1.78
23	O6C6	300	-6.03/-6.42/-6.61	1.87/1.78/1.77
24	N3C4	0	-1.72/-1.72/-2.25	2.14/2.44/2.41
25	N3C4	60	-3.67/-2.73/-3.29	2.03/2.37/2.34
26	N3C4	120	-3.32/-3.32/-3.85	2.10/2.34/2.32
27	N3C4	240	-3.01/-2.97/-3.45	2.11/2.35/2.34
28	N3C4	300	-3.14/-2.25/-2.74	2.05/2.39/2.37

1.29 dihydrouracil (H2U)

Figure 29: The molecule used for water complex calculations corresponding to dihydrouracil (H2U), with possible interacting water positions. NOTE, only one water molecule was included in each calculation

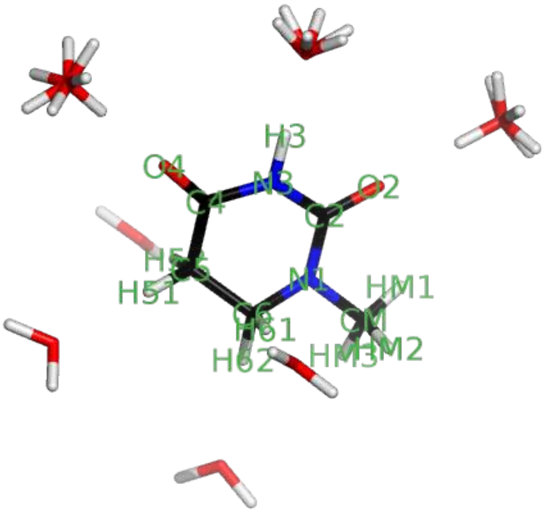

Table 85: Statistics of calculated water interaction and dipole moment for dihydrouracil (H2U). QM is in HF level except for thio-compounds.

	RMS/Max Deviation from QM		Dipole moment	Dipole angle
	Energy (kcal/mol)	Distance (Å)	QM/MM (debye)	difference (°)
Original	1.25/3.16	0.08/0.14	4.60/3.68	44.98
Optimized	0.22/0.47	0.08/0.12	4.60/4.77	25.26

Table 86: The comparison list of optimized atomic charges and their initial guess for dihydrouracil (H2U), referring to the penalties of initial guess

	Charges		
Atom	Optima	Initial	Penalty
N1	-0.184	-0.241	29.116
C2	0.321	0.131	27.532
O2	-0.417	-0.367	5.144
N3	-0.405	-0.186	29.154
H3	0.318	0.347	0.600
C4	0.536	0.540	21.113
O4	-0.490	-0.503	11.180
C5	-0.136	-0.168	13.719
H51	0.090	0.090	0.350
H52	0.090	0.090	0.350
C6	0.097	0.087	18.367
H61	0.090	0.090	0.675
H62	0.090	0.090	0.675
CM	-0.270	-0.270	15.881
HM1	0.090	0.090	0.181
HM2	0.090	0.090	0.181
HM3	0.090	0.090	0.181

Table 87: Interaction energies and geometries between probe water and selected dihydrouracil (H2U) site calculated using the optimized and initial charges

			QM / Optima / Initial	
N	Probe site	Angle (°)	Energy (kcal/mol)	Distance (Å)
1	H3N3	0	-6.43/-5.95/-9.06	1.76/1.83/1.78
2	H3N3	60	-5.27/-5.38/-8.43	1.79/1.85/1.79
3	H3N3	120	-5.32/-5.42/-8.48	1.79/1.85/1.79
4	H51C5	0	-2.38/-2.44/-2.24	2.47/2.59/2.60
5	H52C5	0	-2.83/-2.60/-2.32	2.45/2.57/2.59
6	H61C6	0	-2.78/-3.19/-2.85	2.54/2.55/2.57
7	H62C6	0	-3.27/-3.15/-2.56	2.52/2.57/2.60
8	O2C2	0	-5.58/-5.28/-4.63	1.86/1.80/1.82
9	O2C2	60	-5.49/-5.37/-4.89	1.86/1.80/1.82
10	O2C2	120	-5.32/-5.50/-5.38	1.86/1.79/1.80
11	O2C2	180	-5.14/-5.57/-5.62	1.86/1.79/1.80
12	O2C2	240	-5.36/-5.58/-5.48	1.86/1.80/1.80
13	O2C2	300	-5.58/-5.46/-4.99	1.85/1.80/1.81
14	O4C4	0	-5.09/-5.22/-4.72	1.88/1.79/1.80
15	O4C4	60	-5.09/-5.25/-4.89	1.89/1.79/1.79
16	O4C4	120	-5.05/-5.09/-4.99	1.88/1.79/1.79
17	O4C4	180	-5.00/-4.89/-4.91	1.88/1.79/1.79
18	O4C4	240	-5.00/-4.93/-4.84	1.89/1.79/1.79
19	O4C4	300	-5.05/-5.11/-4.75	1.89/1.79/1.79

1.30 dimethylammonium (dmam, for 5AU, U8U, IAU, ISU etc.)

Figure 30: The molecule used for water complex calculations corresponding to dimethylammonium (dmam, for 5AU, U8U, IAU, ISU etc.), with possible interacting water positions. NOTE, only one water molecule was included in each calculation

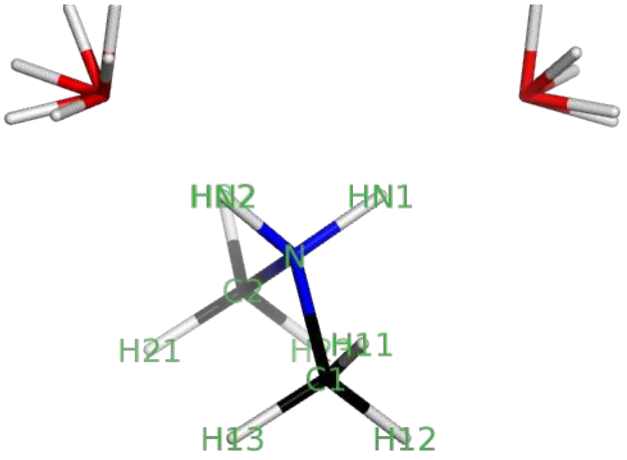

Table 88: Statistics of calculated water interaction and dipole moment for dimethylammonium (dmam, for 5AU, U8U, IAU, ISU etc.). QM is in HF level except for thio-compounds.

	RMS/Max Deviation from QM		Dipole moment	Dipole angle
	Energy (kcal/mol)	Distance (Å)	QM/MM (debye)	difference (°)
Original	2.01/2.02	0.06/0.06	1.56/0.69	0.00
Optimized	0.02/0.02	0.02/0.02	1.56/1.44	0.02

Table 89: The comparison list of optimized atomic charges and their initial guess for dimethylammonium (dmam, for 5AU, U8U, IAU, ISU etc.), referring to the penalties of initial guess

	Charges		
Atom	Optima	Initial	Penalty
N	-0.538	-0.522	10.109
HN1	0.389	0.330	1.461
HN2	0.389	0.330	1.461
C1	0.110	0.161	7.153
H11	0.090	0.090	0.574
H12	0.090	0.090	0.574
H13	0.090	0.090	0.574
C2	0.110	0.161	7.153
H21	0.090	0.090	0.574
H22	0.090	0.090	0.574
H23	0.090	0.090	0.574

Table 90: Interaction energies and geometries between probe water and selected dimethylammonium (dmam, for 5AU, U8U, IAU, ISU etc.) site calculated using the optimized and initial charges

			QM / Optima / Initial	
N	Probe site	Angle (°)	Energy (kcal/mol)	Distance (Å)
1	HN1N	0	-17.82/-17.83/-15.79	1.72/1.75/1.78
2	HN1N	60	-17.87/-17.89/-15.88	1.72/1.75/1.78
3	HN1N	120	-17.82/-17.84/-15.81	1.72/1.75/1.78
4	HN2N	0	-17.82/-17.83/-15.79	1.72/1.75/1.78
5	HN2N	60	-17.87/-17.89/-15.88	1.72/1.75/1.78
6	HN2N	120	-17.82/-17.84/-15.81	1.72/1.75/1.78

1.31 N1,N2-dimethyl-N1-phenylurea (dmpu, for 66A)

Figure 31: The molecule used for water complex calculations corresponding to N1,N2-dimethyl-N1-phenylurea (dmpu, for 66A), with possible interacting water positions. NOTE, only one water molecule was included in each calculation

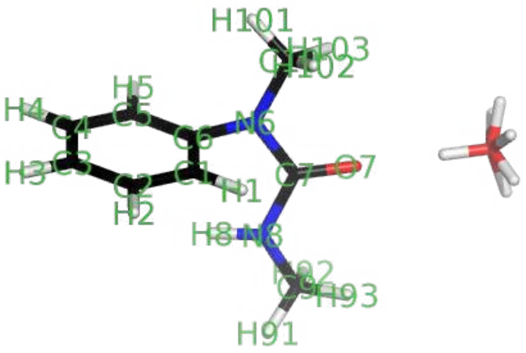

Table 91: Statistics of calculated water interaction and dipole moment for N1,N2-dimethyl-N1-phenylurea (dmpu, for 66A). QM is in HF level except for thio-compounds.

	RMS/Max Deviation from QM		Dipole moment	Dipole angle
	Energy (kcal/mol)	Distance (Å)	QM/MM (debye)	difference (°)
Original	0.30/0.37	0.03/0.03	4.26/4.14	5.20
Optimized	0.15/0.24	0.05/0.05	4.26/4.85	7.29

Table 92: The comparison list of optimized atomic charges and their initial guess for N1,N2-dimethyl-N1-phenylurea (dmpu, for 66A), referring to the penalties of initial guess

	Charges		
Atom	Optima	Initial	Penalty
C1	-0.117	-0.123	5.740
C2	-0.103	-0.109	0.437
C3	-0.112	-0.115	0.000
C4	-0.103	-0.109	0.437
C5	-0.119	-0.123	5.740
C6	0.229	0.079	34.959
H1	0.115	0.115	0.050
H2	0.115	0.115	0.000
H3	0.115	0.115	0.000
H4	0.115	0.115	0.000
H5	0.115	0.115	0.050
N6	-0.494	-0.096	40.905
C7	0.482	0.087	22.861
O7	-0.478	-0.395	20.377
N8	-0.446	-0.384	20.468
H8	0.294	0.307	0.600
C9	-0.069	-0.041	12.017
H91	0.090	0.090	0.000
H92	0.090	0.090	0.000
H93	0.090	0.090	0.000
C10	-0.079	-0.093	24.856
H101	0.090	0.090	1.510
H102	0.090	0.090	1.510
H103	0.090	0.090	1.510

Table 93: Interaction energies and geometries between probe water and selected N1,N2-dimethyl-N1-phenylurea (dmpu, for 66A) site calculated using the optimized and initial charges

			QM / Optima / Initial	
N	Probe site	Angle (°)	Energy (kcal/mol)	Distance (Å)
1	O7C7	0	-6.69/-6.94/-6.38	1.81/1.76/1.78
2	O7C7	60	-6.83/-6.96/-6.56	1.81/1.76/1.77
3	O7C7	120	-6.83/-6.86/-6.63	1.81/1.76/1.77
4	O7C7	180	-6.78/-6.69/-6.47	1.81/1.76/1.77
5	O7C7	240	-6.87/-6.83/-6.50	1.80/1.76/1.77
6	O7C7	300	-6.78/-6.98/-6.48	1.81/1.76/1.77

1.32 7-deazaguanine (7dng, for RCG, DAG, QUG etc.)

Figure 32: The molecule used for water complex calculations corresponding to 7-deazaguanine (7dng, for RCG, DAG, QUG etc.), with possible interacting water positions. NOTE, only one water molecule was included in each calculation

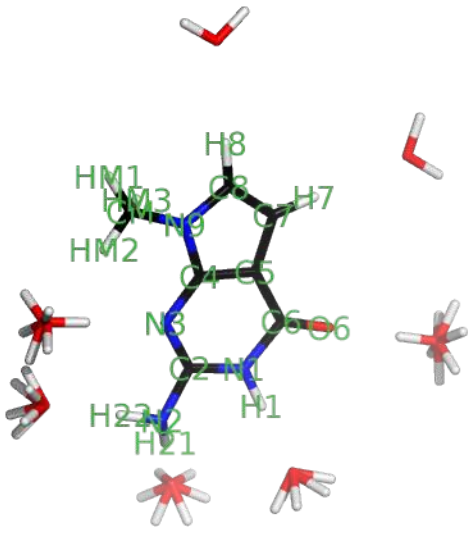

Table 94: Statistics of calculated water interaction and dipole moment for 7-deazaguanine (7dng, for RCG, DAG, QUG etc.). QM is in HF level except for thio-compounds.

	RMS/Max Deviation from QM		Dipole moment	Dipole angle
	Energy (kcal/mol)	Distance (Å)	QM/MM (debye)	difference (°)
Original	0.83/1.75	0.15/0.32	5.24/3.78	48.41
Optimized	0.35/0.79	0.14/0.30	5.24/3.84	13.17

Table 95: The comparison list of optimized atomic charges and their initial guess for 7-deazaguanine (7dng, for RCG, DAG, QUG etc.), referring to the penalties of initial guess

	Charges		
Atom	Optima	Initial	Penalty
N9	0.040	-0.152	15.470
C8	0.060	-0.014	19.215
H8	0.080	0.180	1.500
C7	-0.270	-0.254	27.002
H7	0.130	0.172	1.241
C5	-0.060	-0.079	27.531
C6	0.420	0.498	16.675
O6	-0.520	-0.508	12.532
N1	-0.380	-0.341	10.757
H1	0.300	0.263	0.000
C2	0.650	0.751	0.000
N2	-0.600	-0.677	0.000
H21	0.300	0.337	0.000
H22	0.300	0.337	0.000
N3	-0.770	-0.740	14.276
C4	0.320	0.227	22.652
CM	-0.270	-0.270	10.090
HM1	0.090	0.090	0.150
HM2	0.090	0.090	0.150
HM3	0.090	0.090	0.150

Table 96: Interaction energies and geometries between probe water and selected 7-deazaguanine (7dng, for RCG, DAG, QUG etc.) site calculated using the optimized and initial charges

			QM / Optima / Initial	
N	Probe site	Angle (°)	Energy (kcal/mol)	Distance (Å)
1	H1N1	0	-6.92/-6.83/-6.98	1.80/1.85/1.86
2	H1N1	60	-6.25/-6.51/-6.71	1.82/1.86/1.87
3	H1N1	120	-7.14/-6.86/-7.10	1.79/1.85/1.86
4	H21N2	0	-7.40/-7.60/-8.91	1.88/1.86/1.84
5	H21N2	60	-7.00/-7.42/-8.75	1.90/1.87/1.84
6	H21N2	120	-7.63/-7.67/-9.03	1.86/1.86/1.83
7	H22N2	0	-5.14/-4.69/-6.19	1.86/1.89/1.85
8	H22N2	60	-4.87/-4.31/-5.67	1.89/1.90/1.87
9	H22N2	120	-4.74/-4.34/-5.73	1.90/1.90/1.86
10	H8C8	0	-2.16/-2.08/-2.78	2.53/2.33/2.25
11	H7C7	0	-1.14/-0.35/-0.94	2.48/2.71/2.64
12	O6C6	0	-6.78/-7.06/-6.35	1.82/1.75/1.77
13	O6C6	60	-6.74/-6.94/-6.25	1.82/1.75/1.77
14	O6C6	120	-6.38/-6.49/-5.79	1.83/1.76/1.77
15	O6C6	180	-6.12/-6.15/-5.42	1.83/1.77/1.78
16	O6C6	240	-6.34/-6.42/-5.69	1.83/1.76/1.77
17	O6C6	300	-6.65/-6.88/-6.16	1.82/1.75/1.77
18	N3C4	0	-2.29/-2.63/-1.97	2.12/2.37/2.42
19	N3C4	60	-4.29/-3.68/-3.42	2.01/2.31/2.33
20	N3C4	120	-4.07/-4.26/-4.91	2.08/2.29/2.28
21	N3C4	240	-3.85/-3.96/-4.52	2.08/2.29/2.29
22	N3C4	300	-3.67/-3.17/-2.87	2.03/2.33/2.35

1.33 4-demethylwyosine (DWG)

Figure 33: The molecule used for water complex calculations corresponding to 4-demethylwyosine (DWG), with possible interacting water positions. NOTE, only one water molecule was included in each calculation

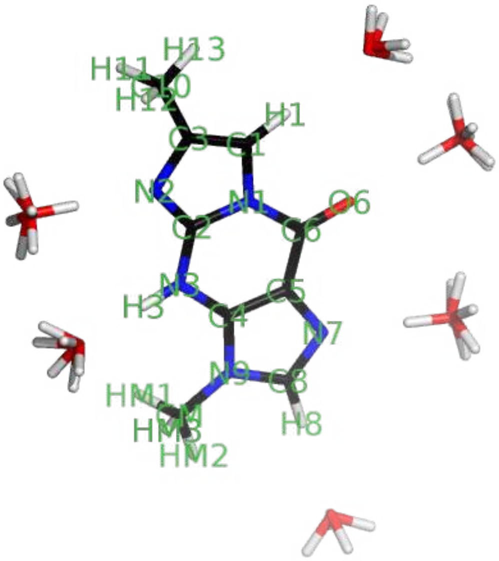

Table 97: Statistics of calculated water interaction and dipole moment for 4-demethylwyosine (DWG). QM is in HF level except for thio-compounds.

	RMS/Max Deviation from QM		Dipole moment	Dipole angle
	Energy (kcal/mol)	Distance (Å)	QM/MM (debye)	difference (°)
Original	2.41/4.56	0.10/0.22	7.95/3.16	151.95
Optimized	0.24/0.71	0.07/0.14	7.95/7.79	13.91

Table 98: The comparison list of optimized atomic charges and their initial guess for 4-demethylwyosine (DWG), referring to the penalties of initial guess

	Charges		
Atom	Optima	Initial	Penalty
N9	0.039	-0.031	23.566
C8	0.377	0.258	17.076
H8	0.090	0.129	0.000
N7	-0.671	-0.593	15.446
C5	0.002	0.059	37.804
C6	0.681	0.981	41.871
O6	-0.500	-0.490	4.912
N1	-0.112	-0.082	52.348
C2	0.443	-0.083	66.856
N2	-0.691	-0.339	36.754
N3	-0.420	-0.623	67.503
H3	0.344	0.204	5.661
C4	0.292	0.119	46.490
C1	-0.443	-0.061	29.451
H1	0.207	0.157	1.241
C3	0.392	0.425	21.409
C10	-0.300	-0.300	6.551
H11	0.090	0.090	0.450
H12	0.090	0.090	0.450
H13	0.090	0.090	0.450
CM	-0.270	-0.270	1.350
HM1	0.090	0.090	0.000
HM2	0.090	0.090	0.000
HM3	0.090	0.090	0.000

Table 99: Interaction energies and geometries between probe water and selected 4-demethylwyosine (DWG) site calculated using the optimized and initial charges

			QM / Optima / Initial	
N	Probe site	Angle (°)	Energy (kcal/mol)	Distance (Å)
1	H1C1	0	-1.85/-1.29/-5.20	2.30/2.26/2.17
2	H1C1	90	-0.78/-0.73/-4.79	2.41/2.29/2.19
3	H3N3	0	-8.38/-8.62/na	1.79/1.82/na
4	H3N3	60	-8.60/-8.45/na	1.78/1.82/na
5	H3N3	120	-8.56/-8.60/na	1.78/1.82/na
6	H8C8	0	-3.81/-4.15/-3.10	2.36/2.22/2.24
7	H8C8	90	-4.21/-4.14/-3.08	2.31/2.22/2.24
8	O6C6	0	-5.05/-5.24/-2.09	1.89/1.80/1.88
9	O6C6	60	-5.32/-5.44/-2.70	1.89/1.79/1.86
10	O6C6	120	-5.85/-5.90/-3.87	1.87/1.79/1.83
11	O6C6	180	-6.16/-6.21/-4.47	1.86/1.78/1.82
12	O6C6	240	-5.89/-5.96/-3.94	1.87/1.79/1.83
13	O6C6	300	-5.36/-5.50/-2.78	1.88/1.79/1.86
14	N2C2	0	-7.76/-8.47/-3.20	1.90/1.88/2.08
15	N2C2	60	-7.94/-8.08/-3.96	1.88/1.89/2.05
16	N2C2	120	-6.74/-6.43/-5.29	1.89/1.90/2.01
17	N2C2	180	-4.92/-4.77/-5.69	1.95/1.93/2.00
18	N2C2	240	-6.12/-5.87/-4.93	1.91/1.91/2.02
19	N2C2	300	-7.67/-7.71/-3.73	1.88/1.89/2.05
20	N7C8	0	-7.98/-7.99/-5.58	1.95/1.91/1.96
21	N7C8	60	-6.52/-6.63/-5.23	1.99/1.93/1.96
22	N7C8	120	-4.78/-4.76/-4.87	2.04/1.96/1.96
23	N7C8	180	-4.16/-3.97/-4.67	2.06/1.98/1.97
24	N7C8	240	-4.83/-4.79/-4.92	2.04/1.96/1.96
25	N7C8	300	-6.52/-6.66/-5.28	1.99/1.93/1.96

1.34 5-methoxyuracil, enol form (enou)

Figure 34: The molecule used for water complex calculations corresponding to 5-methoxyuracil, enol form (enou), with possible interacting water positions. NOTE, only one water molecule was included in each calculation

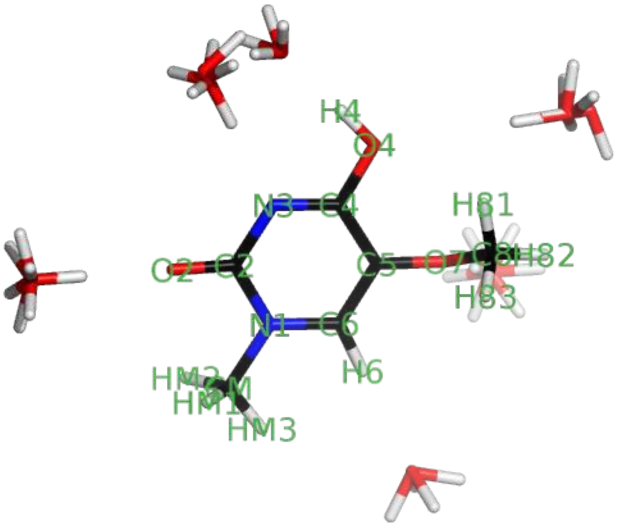

Table 100: Statistics of calculated water interaction and dipole moment for 5-methoxyuracil, enol form (enou). QM is in HF level except for thio-compounds.

	RMS/Max Deviation from QM		Dipole moment	Dipole angle
	Energy (kcal/mol)	Distance (Å)	QM/MM (debye)	difference (°)
Original	1.58/3.09	0.96/4.87	4.93/8.26	19.38
Optimized	0.31/0.72	0.11/0.31	4.93/7.17	17.19

Table 101: The comparison list of optimized atomic charges and their initial guess for 5-methoxyuracil, enol form (enou), referring to the penalties of initial guess

	Charges		
Atom	Optima	Initial	Penalty
N1	-0.149	-0.171	22.987
C2	0.426	0.295	4.941
O2	-0.419	-0.480	0.949
N3	-0.684	-0.478	21.677
C4	0.336	0.246	23.936
O4	-0.464	-0.529	17.050
H4	0.447	0.420	2.525
C5	0.392	0.636	22.397
C6	0.201	0.184	22.046
H6	0.119	0.098	0.550
O7	-0.484	-0.391	5.372
C8	0.009	-0.100	0.392
H81	0.090	0.090	0.025
H82	0.090	0.090	0.025
H83	0.090	0.090	0.025
CM	-0.270	-0.270	4.046
HM1	0.090	0.090	0.000
HM2	0.090	0.090	0.000
HM3	0.090	0.090	0.000

Table 102: Interaction energies and geometries between probe water and selected 5-methoxyuracil, enol form (enou) site calculated using the optimized and initial charges

			QM / Optima / Initial	
N	Probe site	Angle (°)	Energy (kcal/mol)	Distance (Å)
1	H4O4	60	-7.54/-7.39/-6.80	1.67/1.78/1.81
2	H4O4	120	-7.49/-7.36/-6.78	1.67/1.78/1.81
3	H6C6	0	-4.47/-4.65/-5.09	2.31/2.33/2.32
4	H6C6	90	-4.78/-4.57/-5.01	2.28/2.33/2.33
5	O2C2	0	-5.72/-5.78/-7.33	1.84/1.80/1.75
6	O2C2	60	-6.07/-5.98/-7.50	1.84/1.79/1.75
7	O2C2	120	-6.78/-6.52/-7.91	1.82/1.78/1.75
8	O2C2	180	-7.14/-6.84/-8.16	1.81/1.78/1.74
9	O2C2	240	-6.83/-6.64/-8.01	1.82/1.78/1.75
10	O2C2	300	-6.12/-6.11/-7.59	1.84/1.79/1.75
11	O4C4	0	-1.45/-1.54/-1.74	2.84/2.93/2.91
12	O4C4	60	-0.96/-0.42/-0.81	3.01/3.07/3.02
13	O4C4	180	-0.25/-0.26/-0.19	3.32/3.01/3.02
14	O4C4	240	-1.98/-2.09/-1.63	2.84/2.79/2.84
15	O4C4	300	-1.89/-2.17/-2.04	2.80/2.85/2.86
16	O7C5	0	-4.16/-4.51/-2.51	1.89/1.72/1.79
17	O7C5	60	-3.89/-3.76/-1.55	1.88/1.73/1.82
18	O7C5	120	-3.67/-3.21/-0.68	1.89/1.75/1.87
19	O7C5	180	-4.16/-3.81/-1.07	1.89/1.75/1.87
20	O7C5	240	-5.00/-5.03/-2.42	1.86/1.72/1.81
21	O7C5	300	-4.83/-5.21/-3.03	1.86/1.71/1.78
22	N3C4	60	-6.87/-7.14/-5.55	1.88/1.92/1.98
23	N3C4	120	-4.16/-3.66/-2.02	1.95/1.97/2.09
24	N3C4	180	-1.94/-1.22/0.04	2.13/2.09/7.00
25	N3C4	240	-4.25/-3.87/-2.15	1.96/1.97/2.09
26	N3C4	300	-7.00/-7.41/-5.69	1.88/1.91/1.98

1.35 wyosine (IMG)

Figure 35: The molecule used for water complex calculations corresponding to wyosine (IMG), with possible interacting water positions. NOTE, only one water molecule was included in each calculation

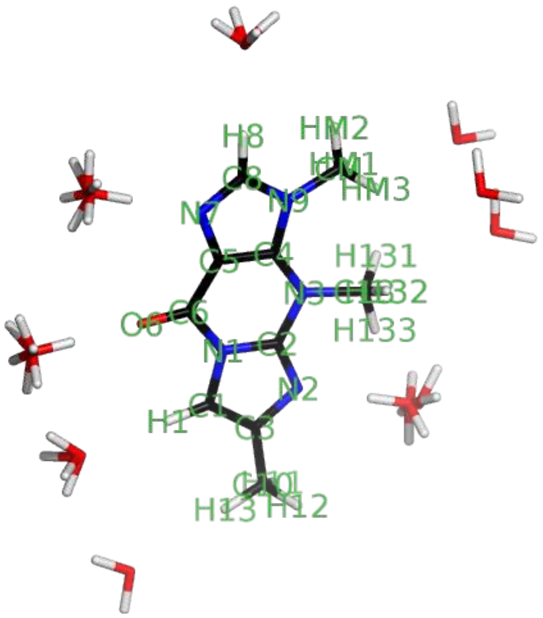

Table 103: Statistics of calculated water interaction and dipole moment for wyosine (IMG). QM is in HF level except for thio-compounds.

	RMS/Max Deviation from QM		Dipole moment	Dipole angle
	Energy (kcal/mol)	Distance (Å)	QM/MM (debye)	difference (°)
Original	1.95/3.29	0.16/0.33	8.32/2.59	106.48
Optimized	0.33/0.94	0.10/0.17	8.32/8.09	13.14

Table 104: The comparison list of optimized atomic charges and their initial guess for wyosine (IMG), referring to the penalties of initial guess

	Charges		
Atom	Optima	Initial	Penalty
N9	0.046	-0.048	23.566
C8	0.382	0.243	17.076
H8	0.085	0.114	0.000
N7	-0.681	-0.608	15.446
C5	0.004	0.044	37.804
C6	0.681	0.966	41.871
O6	-0.512	-0.505	4.912
N1	-0.104	-0.097	52.348
C2	0.450	0.003	66.878
N2	-0.691	-0.354	36.754
N3	-0.332	-0.639	67.525
C13	-0.040	-0.040	5.675
H131	0.090	0.090	0.212
H132	0.090	0.090	0.212
H133	0.090	0.090	0.212
C4	0.303	0.205	46.522
C1	-0.436	-0.076	29.451
H1	0.203	0.142	1.241
C3	0.402	0.410	21.409
C10	-0.300	-0.300	6.551
H11	0.090	0.090	0.450
H12	0.090	0.090	0.450
H13	0.090	0.090	0.450
CM	-0.270	-0.270	1.350
HM1	0.090	0.090	0.000
HM2	0.090	0.090	0.000
HM3	0.090	0.090	0.000

Table 105: Interaction energies and geometries between probe water and selected wyosine (IMG) site calculated using the optimized and initial charges

			QM / Optima / Initial	
N	Probe site	Angle (°)	Energy (kcal/mol)	Distance (Å)
1	H1C1	0	-1.81/-1.25/-4.45	2.30/2.26/2.19
2	H1C1	90	-0.70/-0.66/-3.99	2.41/2.30/2.21
3	H8C8	0	-3.72/-4.04/-2.53	2.38/2.23/2.26
4	H8C8	90	-4.21/-4.05/-2.52	2.32/2.23/2.26
5	H13C10	0	-1.27/-0.66/-2.29	2.74/2.74/2.64
6	H131C3	0	-2.87/-2.45/-0.59	3.38/3.34/3.60
7	H132C3	0	-2.69/-2.53/-0.72	3.45/3.41/3.61
8	H133C3	0	-2.07/-2.30/-0.70	3.72/3.62/3.74
9	O6C6	0	-5.23/-5.45/-2.65	1.88/1.79/1.86
10	O6C6	60	-5.54/-5.69/-3.25	1.88/1.79/1.84
11	O6C6	120	-6.07/-6.19/-4.39	1.86/1.78/1.82
12	O6C6	180	-6.43/-6.51/-5.00	1.85/1.77/1.81
13	O6C6	240	-6.12/-6.24/-4.46	1.86/1.78/1.82
14	O6C6	300	-5.54/-5.73/-3.31	1.88/1.79/1.84
15	N2C2	0	-6.03/-6.97/-3.08	1.95/2.09/2.28
16	N2C2	60	-6.56/-6.53/-3.52	1.93/2.10/2.25
17	N2C2	120	-5.27/-4.76/-3.83	1.98/2.13/2.24
18	N2C2	180	-2.52/-2.92/-3.33	2.21/2.20/2.28
19	N2C2	240	-4.69/-4.23/-3.33	2.01/2.15/2.26
20	N2C2	300	-6.47/-6.24/-3.22	1.93/2.10/2.27
21	N7C8	0	-8.29/-8.33/-6.21	1.95/1.91/1.94
22	N7C8	60	-6.69/-6.84/-5.72	1.99/1.93/1.95
23	N7C8	120	-4.87/-4.90/-5.22	2.04/1.96/1.96
24	N7C8	180	-4.25/-4.09/-4.99	2.06/1.97/1.96
25	N7C8	240	-4.92/-4.94/-5.26	2.04/1.96/1.96
26	N7C8	300	-6.69/-6.87/-5.76	1.99/1.93/1.95

1.36 inosine (INO)

Figure 36: The molecule used for water complex calculations corresponding to inosine (INO), with possible interacting water positions. NOTE, only one water molecule was included in each calculation

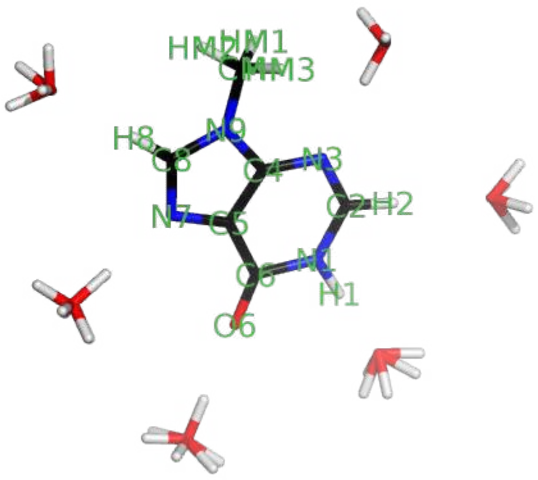

Table 106: Statistics of calculated water interaction and dipole moment for inosine (INO). QM is in HF level except for thio-compounds.

	RMS/Max Deviation from QM		Dipole moment	Dipole angle
	Energy (kcal/mol)	Distance (Å)	QM/MM (debye)	difference (°)
Original	1.30/3.41	0.06/0.13	6.40/6.14	45.10
Optimized	0.21/0.41	0.07/0.16	6.40/5.77	23.43

Table 107: The comparison list of optimized atomic charges and their initial guess for inosine (INO), referring to the penalties of initial guess

	Charges		
Atom	Optima	Initial	Penalty
N9	0.000	-0.013	0.000
C8	0.256	0.252	0.000
H8	0.145	0.123	0.000
N7	-0.616	-0.599	0.000
C5	0.011	0.005	0.000
C6	0.548	0.532	5.697
O6	-0.514	-0.508	0.000
N1	-0.315	-0.248	12.091
H1	0.250	0.277	7.606
C2	0.511	0.618	13.911
H2	0.104	0.123	13.105
N3	-0.646	-0.818	0.000
C4	0.266	0.256	0.000
CM	-0.270	-0.270	0.000
HM1	0.090	0.090	0.000
HM2	0.090	0.090	0.000
HM3	0.090	0.090	0.000

Table 108: Interaction energies and geometries between probe water and selected inosine (INO) site calculated using the optimized and initial charges

			QM / Optima / Initial	
N	Probe site	Angle (°)	Energy (kcal/mol)	Distance (Å)
1	H1N1	0	-7.58/-7.47/-9.87	1.78/1.85/1.80
2	H1N1	60	-7.18/-7.24/-9.66	1.78/1.85/1.81
3	H1N1	120	-7.18/-7.24/-9.66	1.78/1.85/1.81
4	H2C2	0	-4.38/-4.72/-5.80	2.31/2.34/2.31
5	H2C2	90	-4.56/-4.72/-5.80	2.29/2.34/2.31
6	H8C8	0	-3.32/-3.46/-2.64	2.38/2.22/2.25
7	H8C8	90	-3.63/-3.43/-2.60	2.34/2.22/2.25
8	O6C6	0	-6.87/-7.28/-7.14	1.84/1.76/1.76
9	O6C6	60	-6.43/-6.70/-6.47	1.85/1.77/1.77
10	O6C6	120	-5.72/-5.60/-5.16	1.86/1.78/1.79
11	O6C6	180	-5.36/-5.05/-4.49	1.87/1.79/1.80
12	O6C6	240	-5.72/-5.60/-5.16	1.86/1.78/1.79
13	O6C6	300	-6.43/-6.70/-6.47	1.85/1.77/1.77
14	N3C4	120	-4.92/-4.62/-6.70	1.98/2.01/1.95
15	N3C4	240	-4.92/-4.62/-6.70	1.98/2.01/1.94
16	N7C5	0	-4.83/-4.73/-5.01	2.03/1.98/1.97
17	N7C5	60	-5.40/-5.46/-5.61	2.01/1.96/1.96
18	N7C5	120	-7.00/-7.15/-7.01	1.97/1.93/1.94
19	N7C5	180	-8.34/-8.34/-8.07	1.94/1.92/1.92
20	N7C5	240	-7.00/-7.13/-7.01	1.97/1.93/1.94
21	N7C5	300	-5.40/-5.46/-5.61	2.01/1.96/1.96

1.37 N2,N2-dimethylguanine (M2G)

Figure 37: The molecule used for water complex calculations corresponding to N2,N2-dimethylguanine (M2G), with possible interacting water positions. NOTE, only one water molecule was included in each calculation

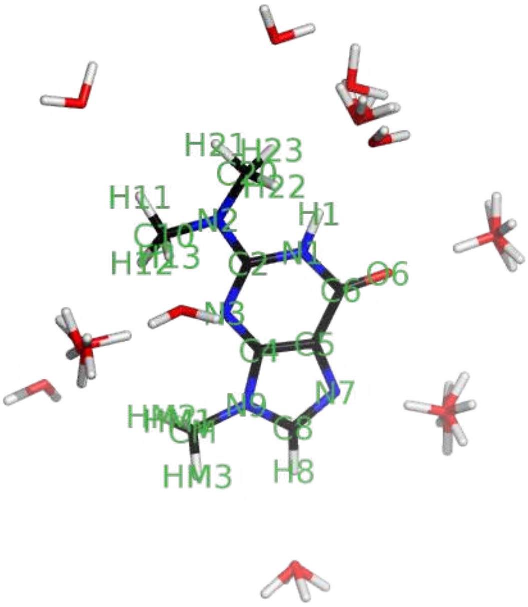

Table 109: Statistics of calculated water interaction and dipole moment for N2,N2-dimethylguanine (M2G). QM is in HF level except for thio-compounds.

	RMS/Max Deviation from QM		Dipole moment	Dipole angle
	Energy (kcal/mol)	Distance (Å)	QM/MM (debye)	difference (°)
Original	0.60/1.34	0.13/0.32	7.92/5.39	14.43
Optimized	0.35/0.90	0.13/0.33	7.92/7.75	10.70

Table 110: The comparison list of optimized atomic charges and their initial guess for N2,N2-dimethylguanine (M2G), referring to the penalties of initial guess

	Charges		
Atom	Optima	Initial	Penalty
N9	-0.005	-0.005	0.000
C8	0.319	0.255	0.000
H8	0.096	0.122	0.000
N7	-0.647	-0.600	0.000
C5	0.004	0.004	0.000
C6	0.481	0.534	2.500
O6	-0.504	-0.509	0.000
N1	-0.468	-0.351	47.475
H1	0.331	0.259	2.500
C2	0.715	1.057	63.006
N2	-0.343	-0.524	58.361
N3	-0.598	-0.741	30.744
C4	0.319	0.261	2.500
C10	-0.120	-0.151	16.865
H11	0.090	0.090	2.657
H12	0.090	0.090	2.657
H13	0.090	0.090	2.657
C20	-0.120	-0.151	16.865
H21	0.090	0.090	2.657
H22	0.090	0.090	2.657
H23	0.090	0.090	2.657
CM	-0.270	-0.270	0.000
HM1	0.090	0.090	0.000
HM2	0.090	0.090	0.000
HM3	0.090	0.090	0.000

Table 111: Interaction energies and geometries between probe water and selected N2,N2-dimethylguanine (M2G) site calculated using the optimized and initial charges

			QM / Optima / Initial	
N	Probe site	Angle (°)	Energy (kcal/mol)	Distance (Å)
1	H1N1	0	-3.85/-3.71/-3.89	2.44/2.75/2.75
2	H1N1	60	-4.03/-3.70/-3.80	2.42/2.75/2.75
3	H1N1	120	-3.76/-3.75/-3.84	2.49/2.75/2.75
4	H8C8	0	-2.78/-2.67/-2.92	2.41/2.26/2.24
5	H8C8	90	-3.05/-2.64/-2.88	2.37/2.26/2.25
6	H11C10	0	-2.43/-1.87/-1.09	2.64/2.66/2.74
7	H12C10	0	-1.14/-0.61/-0.29	4.77/4.63/5.07
8	H13C10	0	-1.58/-1.68/-1.11	2.76/2.66/2.73
9	H21C10	0	-2.47/-2.47/-1.86	2.91/2.89/2.97
10	H22C10	0	-3.01/-3.47/-3.25	2.68/2.62/2.64
11	H23C10	0	-4.43/-4.22/-3.86	2.47/2.52/2.55
12	O6C6	0	-5.58/-5.34/-4.79	1.86/1.78/1.80
13	O6C6	60	-5.98/-5.94/-5.33	1.85/1.78/1.79
14	O6C6	120	-6.83/-7.15/-6.48	1.83/1.76/1.77
15	O6C6	180	-7.32/-7.80/-7.09	1.82/1.75/1.76
16	O6C6	240	-6.87/-7.20/-6.52	1.83/1.76/1.77
17	O6C6	300	-6.03/-5.99/-5.39	1.85/1.78/1.79
18	N3C4	0	-1.10/-0.83/-1.74	2.76/2.82/2.76
19	N3C4	60	-1.72/-1.47/-2.18	2.67/2.77/2.72
20	N3C4	120	-2.21/-2.14/-2.61	2.52/2.71/2.68
21	N3C4	180	-1.23/-2.13/-2.54	2.59/2.71/2.69
22	N3C4	240	-1.63/-1.94/-2.39	2.59/2.72/2.69
23	N3C4	300	-2.03/-1.30/-2.01	2.57/2.76/2.72
24	N7C8	0	-8.78/-8.98/-7.96	1.93/1.90/1.92
25	N7C8	60	-7.40/-7.68/-6.84	1.96/1.92/1.94
26	N7C8	120	-5.80/-5.89/-5.36	1.99/1.95/1.96
27	N7C8	180	-5.23/-5.13/-4.74	2.01/1.97/1.98
28	N7C8	240	-5.85/-5.92/-5.39	1.99/1.95/1.96
29	N7C8	300	-7.40/-7.72/-6.88	1.96/1.92/1.94

1.38 N6,N6-dimethyladenine (M6A)

Figure 38: The molecule used for water complex calculations corresponding to N6,N6-dimethyladenine (M6A), with possible interacting water positions. NOTE, only one water molecule was included in each calculation

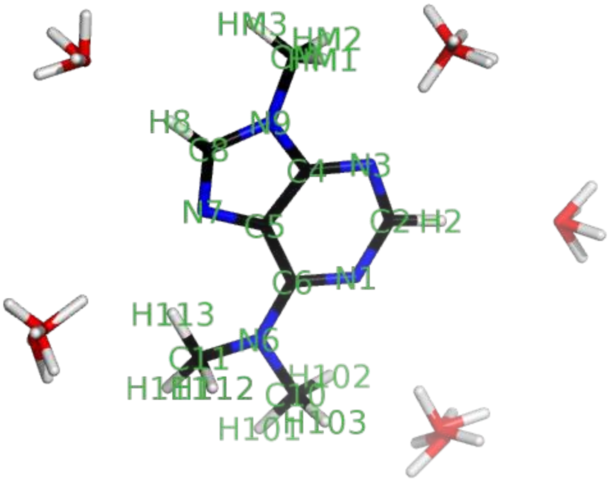

Table 112: Statistics of calculated water interaction and dipole moment for N6,N6-dimethyladenine (M6A). QM is in HF level except for thio-compounds.

	RMS/Max Deviation from QM		Dipole moment	Dipole angle
	Energy (kcal/mol)	Distance (Å)	QM/MM (debye)	difference (°)
Original	0.96/1.98	0.12/0.29	2.53/3.93	17.57
Optimized	0.25/0.71	0.08/0.20	2.53/3.01	19.61

Table 113: The comparison list of optimized atomic charges and their initial guess for N6,N6-dimethyladenine (M6A), referring to the penalties of initial guess

	Charges		
Atom	Optima	Initial	Penalty
N9	-0.010	-0.010	0.000
C8	0.310	0.332	0.000
H8	0.088	0.120	0.000
N7	-0.670	-0.707	2.500
C5	0.415	0.285	24.896
C6	0.516	0.765	52.207
N6	-0.539	-0.587	42.774
N1	-0.682	-0.762	30.744
C2	0.574	0.511	2.500
H2	0.082	0.124	0.000
N3	-0.868	-0.751	0.000
C4	0.434	0.428	2.500
C10	-0.095	-0.144	23.670
H101	0.090	0.090	2.657
H102	0.090	0.090	2.657
H103	0.090	0.090	2.657
C11	-0.095	-0.144	23.670
H111	0.090	0.090	2.657
H112	0.090	0.090	2.657
H113	0.090	0.090	2.657
CM	-0.270	-0.270	0.000
HM1	0.090	0.090	0.000
HM2	0.090	0.090	0.000
HM3	0.090	0.090	0.000

Table 114: Interaction energies and geometries between probe water and selected N6,N6-dimethyladenine (M6A) site calculated using the optimized and initial charges

			QM / Optima / Initial	
N	Probe site	Angle (°)	Energy (kcal/mol)	Distance (Å)
1	H2C2	0	-1.18/-1.13/-1.92	2.55/2.46/2.41
2	H2C2	90	-1.05/-0.99/-1.78	2.57/2.48/2.42
3	H8C8	0	-3.32/-3.50/-4.30	2.38/2.24/2.21
4	H8C8	90	-3.58/-3.48/-4.27	2.34/2.24/2.21
5	N1C2	0	-0.87/-1.14/-1.86	3.20/3.11/3.05
6	N1C2	60	-1.23/-1.51/-1.93	3.12/3.07/3.05
7	N1C2	120	-1.72/-1.85/-1.86	2.98/3.03/3.04
8	N1C2	180	-1.63/-1.88/-1.73	2.96/3.03/3.05
9	N1C2	240	-1.58/-1.77/-1.80	3.00/3.03/3.03
10	N3C4	0	-7.23/-7.11/-5.57	1.91/1.96/2.00
11	N3C4	60	-7.45/-6.98/-5.47	1.91/1.95/2.00
12	N3C4	120	-5.45/-6.16/-4.69	1.98/1.97/2.01
13	N3C4	240	-6.43/-6.42/-4.94	1.93/1.96/2.00
14	N3C4	300	-7.27/-7.05/-5.55	1.91/1.95/2.00
15	N7C5	0	-1.32/-1.54/-1.47	3.07/2.99/3.02
16	N7C5	60	-1.58/-1.71/-1.78	3.04/2.98/2.99
17	N7C5	120	-1.50/-1.54/-2.06	3.06/3.00/2.97
18	N7C5	180	-0.78/-0.96/-1.85	3.30/3.09/3.00
19	N7C5	240	-1.14/-1.03/-1.64	3.15/3.06/3.02
20	N7C5	300	-1.54/-1.36/-1.49	3.03/3.01/3.01

1.39 methyl hydrogen peroxide (mhpo, for PBG)

Figure 39: The molecule used for water complex calculations corresponding to methyl hydrogen peroxide (mhpo, for PBG), with possible interacting water positions. NOTE, only one water molecule was included in each calculation

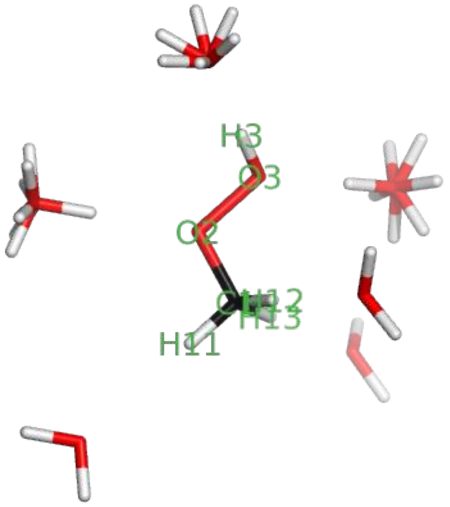

Table 115: Statistics of calculated water interaction and dipole moment for methyl hydrogen peroxide (mhpo, for PBG). QM is in HF level except for thio-compounds.

	RMS/Max Deviation from QM		Dipole moment	Dipole angle
	Energy (kcal/mol)	Distance (Å)	QM/MM (debye)	difference (°)
Original	3.17/4.56	0.20/0.29	1.46/2.55	68.57
Optimized	0.22/0.54	0.09/0.16	1.46/1.58	0.84

Table 116: The comparison list of optimized atomic charges and their initial guess for methyl hydrogen peroxide (mhpo, for PBG), referring to the penalties of initial guess

	Charges		
Atom	Optima	Initial	Penalty
C1	-0.015	-0.100	5.148
H11	0.090	0.090	2.250
H12	0.090	0.090	2.250
H13	0.090	0.090	2.250
O2	-0.248	-0.459	331.256!
O3	-0.431	-0.211	331.917!
H3	0.424	0.500	27.274

Table 117: Interaction energies and geometries between probe water and selected methyl hydrogen peroxide (mhpo, for PBG) site calculated using the optimized and initial charges

			QM / Optima / Initial	
	Probe	Angle	Energy	Distance
N	site	(°)	(kcal/mol)	(Å)
1	H3O3	0	-7.20/-7.09/-11.76	1.72/1.78/1.70
2	H3O3	60	-7.03/-6.97/-11.53	1.73/1.78/1.70
3	H3O3	120	-7.12/-7.02/-11.56	1.72/1.78/1.70
4	H11C1	0	-1.63/-1.09/na	2.61/2.67/na
5	H12C1	0	-1.12/-1.04/na	2.76/2.69/na
6	H13C1	0	-1.01/-0.54/na	2.66/2.70/na
7	O2C1	0	-3.49/-3.53/-5.45	1.96/1.80/1.71
8	O2C1	60	-4.18/-4.23/-6.84	1.92/1.78/1.68
9	O2C1	120	-3.89/-4.04/-7.57	1.93/1.79/1.67
10	O2C1	180	-3.32/-3.47/-7.30	1.97/1.81/1.68
11	O2C1	240	-3.34/-3.19/-6.49	1.95/1.81/1.69
12	O2C1	300	-3.23/-3.08/-5.38	1.96/1.82/1.71
13	O3O2	0	-3.67/-3.69/-0.66	1.97/1.95/2.21
14	O3O2	60	-3.69/-3.50/-0.85	1.94/1.95/2.18
15	O3O2	120	-3.45/-3.50/-1.54	1.96/1.95/2.11
16	O3O2	180	-3.49/-3.92/-2.19	1.98/1.94/2.07
17	O3O2	240	-4.29/-4.47/-2.17	1.92/1.92/2.08
18	O3O2	300	-4.18/-4.20/-1.30	1.93/1.94/2.15

1.40 7-amidinio-7-deazapurine (mip, for RCG)

Figure 40: The molecule used for water complex calculations corresponding to 7-amidinio-7-deazapurine (mip, for RCG), with possible interacting water positions. NOTE, only one water molecule was included in each calculation

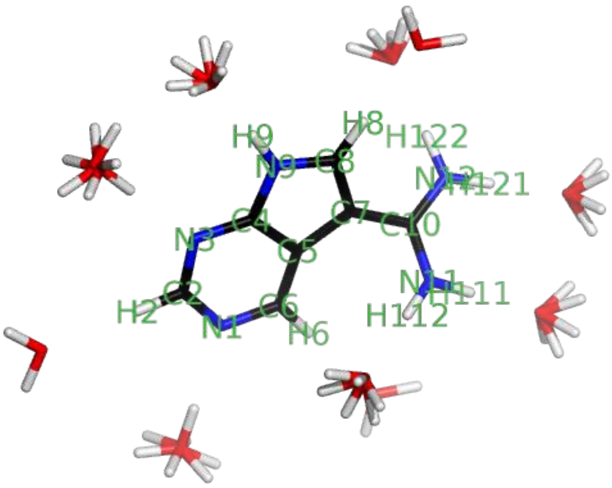

Table 118: Statistics of calculated water interaction and dipole moment for 7-amidinio-7-deazapurine (mip, for RCG). QM is in HF level except for thio-compounds.

	RMS/Max Deviation from QM		Dipole moment	Dipole angle
	Energy (kcal/mol)	Distance (Å)	QM/MM (debye)	difference (°)
Original	2.27/4.10	0.12/0.29	10.74/15.47	5.28
Optimized	0.26/0.56	0.07/0.16	10.74/11.49	3.59

Table 119: The comparison list of optimized atomic charges and their initial guess for 7-amidinio-7-deazapurine (mip, for RCG), referring to the penalties of initial guess

	Charges		
Atom	Optima	Initial	Penalty
N9	-0.397	-0.396	3.037
C8	-0.213	-0.106	34.294
H8	0.244	0.213	2.500
C7	-0.002	0.200	83.942
C5	-0.199	0.011	41.013
C6	0.242	0.317	12.081
H6	0.075	0.104	0.580
N1	-0.600	-0.756	12.075
C2	0.577	0.497	0.000
H2	0.116	0.145	0.000
N3	-0.575	-0.777	14.276
C4	0.410	0.398	22.789
C10	0.592	0.683	78.738
N11	-0.431	-0.584	8.107
H111	0.294	0.320	4.838
H112	0.294	0.320	4.838
N12	-0.431	-0.584	8.107
H121	0.294	0.320	4.838
H122	0.288	0.320	4.838
H9	0.422	0.355	0.000

Table 120: Interaction energies and geometries between probe water and selected 7-amidinio-7-deazapurine (mip, for RCG) site calculated using the optimized and initial charges

			QM / Optima / Initial	
N	Probe site	Angle (°)	Energy (kcal/mol)	Distance (Å)
1	H111N11	0	-13.43/-13.74/-14.09	1.83/1.82/1.81
2	H111N11	60	-14.50/-14.16/-14.51	1.80/1.81/1.81
3	H111N11	120	-14.85/-14.29/-14.64	1.79/1.81/1.80
4	H112N11	0	-12.32/-12.63/-16.01	1.89/1.90/1.88
5	H112N11	60	-12.36/-12.88/-16.46	1.94/1.92/1.90
6	H112N11	120	-12.32/-12.44/-15.88	1.90/1.90/1.87
7	H121N12	0	-13.39/-13.79/-14.07	1.83/1.82/1.81
8	H121N12	60	-14.43/-14.22/-14.49	1.80/1.81/1.81
9	H121N12	120	-14.81/-14.35/-14.63	1.79/1.81/1.80
10	H122N12	0	-13.12/-13.48/-15.31	1.84/1.83/1.82
11	H122N12	60	-12.47/-12.89/-14.92	1.85/1.85/1.83
12	H122N12	120	-13.89/-13.61/-15.68	1.83/1.83/1.82
13	H9N9	0	-12.47/-12.64/-10.71	1.77/1.76/1.80
14	H9N9	60	-12.55/-12.62/-10.56	1.77/1.76/1.80
15	H9N9	120	-12.59/-12.65/-10.57	1.76/1.76/1.80
16	H8C8	0	-9.14/-9.20/-10.55	2.17/2.17/2.15
17	H6C6	0	-5.54/-5.24/-6.76	2.44/2.54/2.41
18	H2C2	0	-4.93/-4.92/-3.38	2.26/2.35/2.39
19	N1C6	0	-2.59/-2.40/-5.07	2.14/2.05/1.96
20	N1C6	60	-2.71/-2.49/-4.81	2.11/2.04/1.95
21	N1C6	120	-2.02/-1.98/-3.48	2.12/2.03/1.97
22	N1C6	180	-1.17/-1.29/-2.35	2.17/2.05/1.99
23	N1C6	240	-1.75/-1.68/-3.21	2.13/2.04/1.97
24	N1C6	300	-2.51/-2.27/-4.60	2.12/2.04/1.96
25	N3C4	0	-2.82/-2.96/-6.47	2.13/2.03/1.92
26	N3C4	60	-2.74/-2.59/-5.94	2.09/2.02/1.92
27	N3C4	120	-1.14/-0.99/-4.00	2.14/2.05/1.94
28	N3C4	180	0.47/0.46/-2.38	2.26/2.10/1.97
29	N3C4	240	-1.14/-1.05/-4.05	2.14/2.04/1.94
30	N3C4	300	-2.74/-2.66/-6.00	2.09/2.02/1.92

1.41 5-methoxyuracil (MOU)

Figure 41: The molecule used for water complex calculations corresponding to 5-methoxyuracil (MOU), with possible interacting water positions. NOTE, only one water molecule was included in each calculation

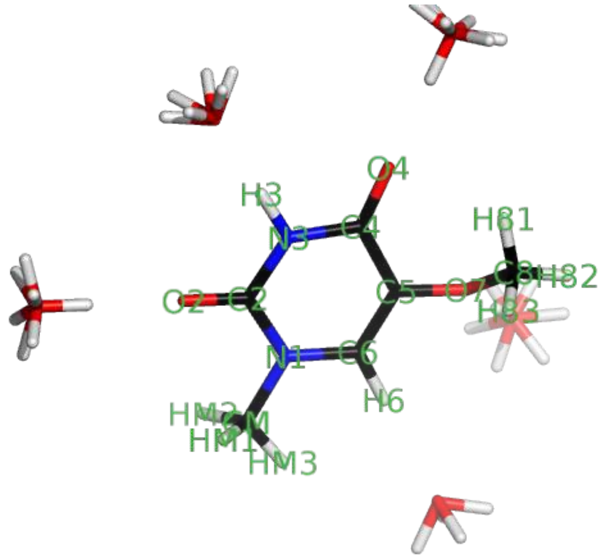

Table 121: Statistics of calculated water interaction and dipole moment for 5-methoxyuracil (MOU). QM is in HF level except for thio-compounds.

	RMS/Max Deviation from QM		Dipole moment	Dipole angle
	Energy (kcal/mol)	Distance (Å)	QM/MM (debye)	difference (°)
Original	0.57/1.14	0.08/0.14	4.43/2.61	34.16
Optimized	0.26/0.55	0.09/0.15	4.43/4.17	35.24

Table 122: The comparison list of optimized atomic charges and their initial guess for 5-methoxyuracil (MOU), referring to the penalties of initial guess

	Charges		
Atom	Optima	Initial	Penalty
N1	-0.289	-0.340	17.431
C2	0.518	0.509	0.000
O2	-0.445	-0.410	0.000
N3	-0.479	-0.461	20.934
H3	0.334	0.362	0.000
C4	0.470	0.479	31.162
O4	-0.471	-0.450	22.794
C5	0.184	0.204	26.758
C6	0.188	0.187	14.677
H6	0.161	0.141	0.550
O7	-0.433	-0.391	5.623
C8	-0.008	-0.100	0.488
H81	0.090	0.090	0.025
H82	0.090	0.090	0.025
H83	0.090	0.090	0.025
CM	-0.270	-0.270	0.000
HM1	0.090	0.090	0.000
HM2	0.090	0.090	0.000
HM3	0.090	0.090	0.000

Table 123: Interaction energies and geometries between probe water and selected 5-methoxyuracil (MOU) site calculated using the optimized and initial charges

			QM / Optima / Initial	
N	Probe site	Angle (°)	Energy (kcal/mol)	Distance (Å)
1	H6C6	0	-4.29/-4.52/-3.95	2.32/2.32/2.34
2	H6C6	90	-4.65/-4.40/-3.82	2.29/2.32/2.34
3	H3N3	0	-7.00/-6.53/na	1.74/1.82/na
4	H3N3	60	-5.89/-5.98/na	1.77/1.83/na
5	H3N3	120	-5.94/-5.98/na	1.77/1.83/na
6	O2C2	0	-4.78/-5.34/-4.86	1.88/1.80/1.81
7	O2C2	60	-5.00/-5.25/-4.71	1.87/1.80/1.82
8	O2C2	120	-5.32/-5.11/-4.42	1.87/1.80/1.82
9	O2C2	180	-5.40/-5.04/-4.26	1.87/1.81/1.83
10	O2C2	240	-5.36/-5.21/-4.49	1.86/1.80/1.82
11	O2C2	300	-5.05/-5.35/-4.78	1.87/1.80/1.82
12	O4C4	0	-5.00/-4.97/-4.76	1.87/1.79/1.80
13	O4C4	60	-5.49/-5.59/-5.09	1.86/1.78/1.80
14	O4C4	120	-5.40/-5.64/-4.89	1.87/1.78/1.80
15	O4C4	180	-5.18/-5.34/-4.54	1.87/1.79/1.81
16	O4C4	240	-5.05/-4.98/-4.39	1.87/1.79/1.81
17	O4C4	300	-4.74/-4.65/-4.39	1.88/1.80/1.81
18	O7C5	0	-4.56/-4.85/-4.75	1.88/1.73/1.74
19	O7C5	60	-4.07/-3.94/-3.87	1.87/1.74/1.75
20	O7C5	120	-3.58/-3.19/-3.00	1.89/1.76/1.78
21	O7C5	180	-4.07/-3.83/-3.37	1.89/1.75/1.77
22	O7C5	240	-5.27/-5.24/-4.64	1.85/1.72/1.74
23	O7C5	300	-5.32/-5.61/-5.23	1.85/1.71/1.73

1.42 N2-methyl-N1-phenylurea (mpu, for HNA, 26A, T6A, 12A, 6GA)

Figure 42: The molecule used for water complex calculations corresponding to N2-methyl-N1-phenylurea (mpu, for HNA, 26A, T6A, 12A, 6GA), with possible interacting water positions. NOTE, only one water molecule was included in each calculation

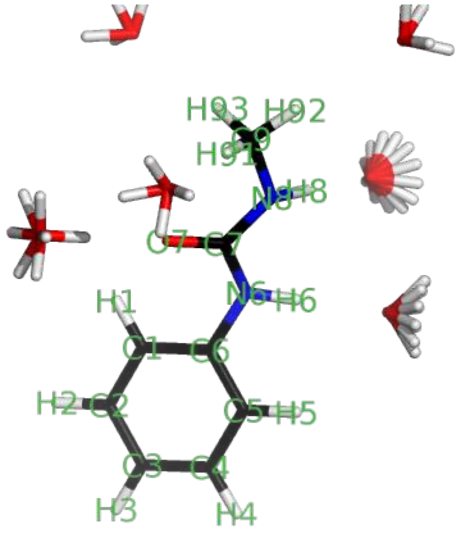

Table 124: Statistics of calculated water interaction and dipole moment for N2-methyl-N1-phenylurea (mpu, for HNA, 26A, T6A, 12A, 6GA). QM is in HF level except for thio-compounds.

	RMS/Max Deviation from QM		Dipole moment	Dipole angle
	Energy (kcal/mol)	Distance (Å)	QM/MM (debye)	difference (°)
Original	0.85/1.57	0.06/0.11	3.82/4.81	8.77
Optimized	0.24/0.48	0.05/0.11	3.82/4.20	9.76

Table 125: The comparison list of optimized atomic charges and their initial guess for N2-methyl-N1-phenylurea (mpu, for HNA, 26A, T6A, 12A, 6GA), referring to the penalties of initial guess

	Charges		
Atom	Optima	Initial	Penalty
C1	-0.118	-0.120	1.329
C2	-0.123	-0.109	0.000
C3	-0.138	-0.115	0.000
C4	-0.124	-0.109	0.000
C5	-0.132	-0.120	1.329
C6	0.131	0.131	17.176
H1	0.115	0.115	0.000
H2	0.115	0.115	0.000
H3	0.115	0.115	0.000
H4	0.115	0.115	0.000
H5	0.115	0.115	0.000
N6	-0.468	-0.303	21.014
H6	0.296	0.332	0.550
C7	0.601	0.082	16.077
O7	-0.481	-0.397	15.652
N8	-0.486	-0.383	13.740
H8	0.286	0.307	0.550
C9	-0.089	-0.041	12.017
H91	0.090	0.090	0.000
H92	0.090	0.090	0.000
H93	0.090	0.090	0.000

Table 126: Interaction energies and geometries between probe water and selected N2-methyl-N1-phenylurea (mpu, for HNA, 26A, T6A, 12A, 6GA) site calculated using the optimized and initial charges

			QM / Optima / Initial	
N	Probe site	Angle (°)	Energy (kcal/mol)	Distance (Å)
1	H8N8	0	-6.32/-6.25/-6.52	1.91/1.91/1.89
2	H8N8	30	-6.72/-6.43/-6.81	1.89/1.90/1.89
3	H8N8	60	-6.58/-6.38/-6.81	1.89/1.90/1.88
4	H8N8	90	-6.09/-6.16/-6.52	1.92/1.91/1.89
5	H8N8	120	-5.69/-5.97/-6.22	1.94/1.91/1.90
6	H8N8	150	-5.78/-6.01/-6.21	1.94/1.91/1.90
7	H6N6	0	-5.78/-6.20/-7.27	1.96/1.93/1.89
8	H6N6	30	-6.63/-6.52/-7.65	1.91/1.91/1.87
9	H6N6	60	-7.34/-6.85/-8.05	1.89/1.90/1.86
10	H6N6	90	-7.25/-6.85/-8.07	1.90/1.90/1.86
11	H6N6	120	-6.45/-6.54/-7.72	1.93/1.91/1.87
12	H6N6	150	-5.74/-6.20/-7.31	1.96/1.93/1.89
13	H91C9	0	-0.32/-0.25/na	2.63/2.68/na
14	H91C9	90	-0.50/-0.26/na	2.58/2.68/na
15	H92C9	0	-1.69/-1.57/na	2.71/2.66/na
16	H92C9	90	-1.83/-1.64/na	2.68/2.66/na
17	H93C9	0	-0.81/-0.98/na	2.77/2.66/na
18	H93C9	90	-1.07/-1.07/na	2.69/2.65/na
19	O7C7	0	-6.23/-6.50/-7.09	1.83/1.77/1.77
20	O7C7	60	-6.67/-6.70/-7.39	1.82/1.77/1.76
21	O7C7	120	-6.27/-6.28/-7.07	1.83/1.77/1.77
22	O7C7	180	-6.00/-5.83/-6.63	1.83/1.78/1.77
23	O7C7	240	-5.92/-5.78/-6.61	1.83/1.78/1.77
24	O7C7	300	-5.74/-6.00/-6.72	1.84/1.78/1.77

1.43 2-methylthiouracil (2msu, for GAU, GCU)

Figure 43: The molecule used for water complex calculations corresponding to 2-methylthiouracil (2msu, for GAU, GCU), with possible interacting water positions. NOTE, only one water molecule was included in each calculation

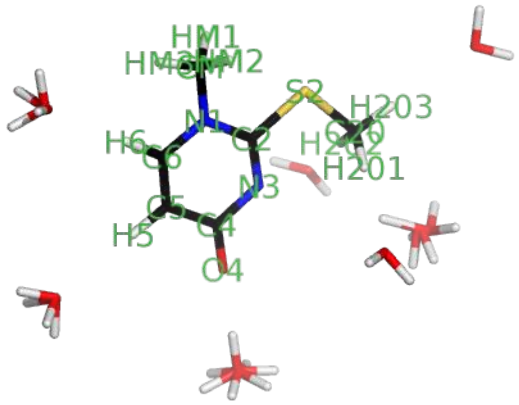

Table 127: Statistics of calculated water interaction and dipole moment for 2-methylthiouracil (2msu, for GAU, GCU). QM is in HF level except for thio-compounds.

	RMS/Max Deviation from QM		Dipole moment	Dipole angle
	Energy (kcal/mol)	Distance (Å)	QM/MM (debye)	difference (°)
Original	1.31/2.13	0.06/0.13	7.25/4.53	60.74
Optimized	0.26/0.51	0.06/0.08	7.25/5.98	4.32

Table 128: The comparison list of optimized atomic charges and their initial guess for 2-methylthiouracil (2msu, for GAU, GCU), referring to the penalties of initial guess

	Charges		
Atom	Optima	Initial	Penalty
N1	-0.232	-0.360	34.647
C2	0.418	0.468	51.734
S2	-0.120	-0.273	50.593
N3	-0.562	-0.694	33.811
C4	0.519	0.929	4.392
O4	-0.523	-0.544	0.000
C5	-0.175	-0.193	5.110
H5	0.088	0.122	2.982
C6	0.242	0.225	2.669
H6	0.145	0.141	0.269
C20	-0.070	-0.091	21.807
H201	0.090	0.090	1.625
H202	0.090	0.090	1.625
H203	0.090	0.090	1.625
CM	-0.270	-0.270	5.766
HM1	0.090	0.090	0.025
HM2	0.090	0.090	0.025
HM3	0.090	0.090	0.025

Table 129: Interaction energies and geometries between probe water and selected 2-methylthiouracil (2msu, for GAU, GCU) site calculated using the optimized and initial charges

			QM / Optima / Initial	
N	Probe site	Angle (°)	Energy (kcal/mol)	Distance (Å)
1	H201C20	0	-0.56/-0.53/na	2.55/2.62/na
2	H202C20	0	-0.56/-0.53/na	2.55/2.62/na
3	H203C20	0	-2.12/-1.65/-0.70	2.55/2.62/2.69
4	H5C5	0	-1.76/-1.63/-3.67	2.47/2.46/2.36
5	H5C5	90	-1.58/-1.51/-3.57	2.49/2.46/2.37
6	H6C6	0	-4.38/-4.89/-4.65	2.40/2.33/2.34
7	H6C6	90	-5.18/-4.96/-4.71	2.32/2.32/2.33
8	N3C2	0	-1.98/-2.43/-1.75	3.51/3.48/3.54
9	N3C2	60	-1.36/-1.60/-1.32	3.61/3.56/3.60
10	N3C2	120	-0.21/-0.01/-0.48	3.86/3.91/3.79
11	N3C2	240	-0.21/-0.01/-0.48	3.86/3.91/3.79
12	N3C2	300	-1.36/-1.60/-1.32	3.61/3.56/3.60
13	O4C4	0	-7.32/-7.61/-6.07	1.81/1.75/1.77
14	O4C4	60	-7.18/-7.43/-5.73	1.82/1.75/1.78
15	O4C4	120	-6.92/-7.07/-5.05	1.82/1.75/1.79
16	O4C4	180	-6.83/-6.84/-4.70	1.82/1.76/1.80
17	O4C4	240	-6.92/-7.07/-5.05	1.82/1.75/1.79
18	O4C4	300	-7.18/-7.45/-5.73	1.82/1.75/1.78

1.44 N2,N2,7-trimethylguanine (N2G)

Figure 44: The molecule used for water complex calculations corresponding to N2,N2,7-trimethylguanine (N2G), with possible interacting water positions. NOTE, only one water molecule was included in each calculation

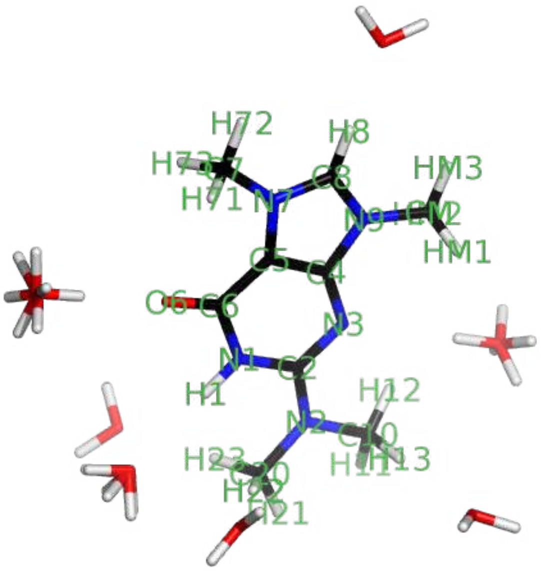

Table 130: Statistics of calculated water interaction and dipole moment for N2,N2,7-trimethylguanine (N2G). QM is in HF level except for thio-compounds.

	RMS/Max Deviation from QM		Dipole moment	Dipole angle
	Energy (kcal/mol)	Distance (Å)	QM/MM (debye)	difference (°)
Original	0.77/1.62	0.18/0.24	5.04/6.13	83.44
Optimized	0.32/0.61	0.18/0.25	5.04/3.93	63.19

Table 131: The comparison list of optimized atomic charges and their initial guess for N2,N2,7-trimethylguanine (N2G), referring to the penalties of initial guess

	Charges		
Atom	Optima	Initial	Penalty
N9	-0.082	-0.209	112.765
C8	0.412	0.362	100.119
H8	0.124	0.184	19.849
N7	-0.399	-0.391	114.033
C5	0.129	0.126	79.191
C6	0.575	0.566	29.115
O6	-0.458	-0.477	14.853
N1	-0.308	-0.319	49.157
H1	0.230	0.291	2.500
C2	0.736	1.068	64.466
N2	-0.296	-0.492	58.361
N3	-0.515	-0.511	41.536
C4	0.204	0.098	78.160
C7	0.092	0.196	109.300
H71	0.090	0.090	3.537
H72	0.090	0.090	3.537
H73	0.090	0.090	3.537
C10	-0.127	-0.151	16.865
H11	0.090	0.090	2.657
H12	0.090	0.090	2.657
H13	0.090	0.090	2.657
C20	-0.127	-0.151	16.865
H21	0.090	0.090	2.657
H22	0.090	0.090	2.657
H23	0.090	0.090	2.657
CM	-0.270	-0.270	109.300
HM1	0.090	0.090	3.537
HM2	0.090	0.090	3.537
HM3	0.090	0.090	3.537

Table 132: Interaction energies and geometries between probe water and selected N2,N2,7-trimethylguanine (N2G) site calculated using the optimized and initial charges

			QM / Optima / Initial	
N	Probe site	Angle (°)	Energy (kcal/mol)	Distance (Å)
1	H1N1	0	-7.68/-7.66/-9.10	2.28/2.53/2.42
2	H1N1	60	-7.61/-7.75/-9.10	2.35/2.52/2.42
3	H1N1	120	-7.46/-7.74/-9.07	2.39/2.52/2.43
4	H8C8	0	-10.60/-10.53/-11.38	2.08/2.01/1.98
5	H13C10	0	-4.16/-3.66/-3.21	2.52/2.62/2.66
6	H22C20	0	-5.46/-5.34/-5.54	2.37/2.54/2.54
7	H23C20	0	-6.15/-5.63/-5.90	2.32/2.53/2.53
8	O6C6	0	-2.32/-2.64/-2.69	2.04/1.82/1.81
9	O6C6	60	-2.25/-2.41/-2.42	2.04/1.82/1.81
10	O6C6	120	-1.63/-1.54/-1.40	2.06/1.84/1.83
11	O6C6	180	-1.10/-0.85/-0.56	2.09/1.85/1.85
12	O6C6	240	-1.63/-1.56/-1.42	2.06/1.84/1.83
13	O6C6	300	-2.25/-2.43/-2.44	2.04/1.82/1.81
14	N3C4	60	0.59/0.80/0.62	2.84/2.93/2.97
15	N3C4	120	0.86/0.48/0.26	2.74/2.85/2.87
16	N3C4	240	1.09/0.47/0.26	2.78/2.84/2.86
17	N3C4	300	0.24/0.76/0.60	2.71/2.90/2.94

1.45 2-methylamino-4-amino-pyrimidine (ncy, for K2Cn)

Figure 45: The molecule used for water complex calculations corresponding to 2-methylamino-4-amino-pyrimidine (ncy, for K2Cn), with possible interacting water positions. NOTE, only one water molecule was included in each calculation

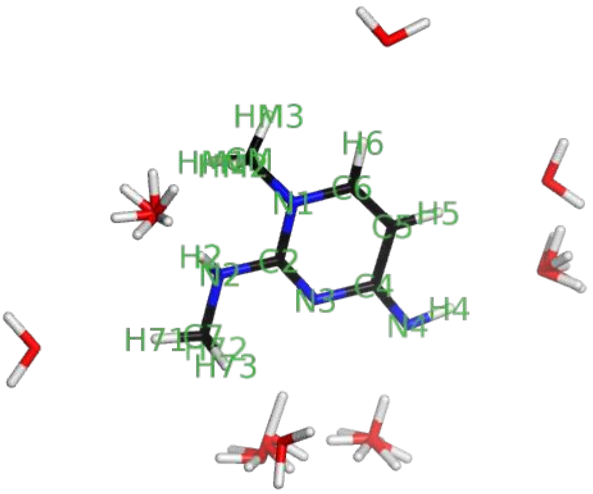

Table 133: Statistics of calculated water interaction and dipole moment for 2-methylamino-4-amino-pyrimidine (ncy, for K2Cn). QM is in HF level except for thio-compounds.

	RMS/Max Deviation from QM		Dipole moment	Dipole angle
	Energy (kcal/mol)	Distance (Å)	QM/MM (debye)	difference (°)
Original	3.90/6.52	0.23/0.52	7.75/7.14	63.66
Optimized	0.29/0.55	0.11/0.27	7.75/8.30	4.93

Table 134: The comparison list of optimized atomic charges and their initial guess for 2-methylamino-4-amino-pyrimidine (ncy, for K2Cn), referring to the penalties of initial guess

	Charges		
Atom	Optima	Initial	Penalty
N1	-0.293	-0.416	52.284
C2	0.852	0.459	60.771
N2	-0.587	-0.517	55.455
H2	0.370	0.364	3.771
N3	-0.730	-0.660	34.454
C4	0.632	0.625	134.037
N4	-0.902	-0.614	132.335
H4	0.316	0.378	20.206
C5	-0.211	-0.164	18.977
H5	0.130	0.169	6.810
C6	0.105	-0.005	24.420
H6	0.155	0.305	13.355
C7	-0.107	-0.194	9.838
H71	0.090	0.090	0.600
H72	0.090	0.090	0.600
H73	0.090	0.090	0.600
CM	-0.270	-0.270	5.949
HM1	0.090	0.090	0.079
HM2	0.090	0.090	0.079
HM3	0.090	0.090	0.079

Table 135: Interaction energies and geometries between probe water and selected 2-methylamino-4-amino-pyrimidine (ncy, for K2Cn) site calculated using the optimized and initial charges

			QM / Optima / Initial	
N	Probe site	Angle (°)	Energy (kcal/mol)	Distance (Å)
1	H2N2	0	-4.43/-4.77/-2.02	2.36/2.35/2.49
2	H2N2	60	-4.43/-4.80/-2.03	2.33/2.35/2.50
3	H2N2	120	-5.27/-5.01/-2.18	2.27/2.33/2.48
4	H4N4	0	-1.14/-1.21/-7.26	2.40/2.14/1.88
5	H4N4	60	-1.72/-1.59/-7.90	2.29/2.10/1.86
6	H4N4	120	-1.81/-1.61/-7.94	2.27/2.10/1.86
7	H5C5	0	-2.03/-2.39/-4.49	2.58/2.60/2.52
8	H6C6	0	-3.63/-4.16/-5.90	2.44/2.36/2.28
9	H71C7	0	-1.41/-1.33/0.05	2.76/2.69/2.88
10	H73C7	0	-0.03/-0.19/na	2.78/2.68/na
11	N3C2	0	-6.25/-6.24/-4.55	2.35/2.63/2.71
12	N3C2	60	-4.56/-4.55/-4.03	2.57/2.70/2.74
13	N3C2	120	-2.47/-1.96/-3.42	2.79/2.88/2.79
14	N3C2	180	-1.54/-0.98/-3.41	2.92/3.01/2.79
15	N3C2	240	-2.56/-2.44/-3.87	2.82/2.85/2.77
16	N3C2	300	-4.83/-5.03/-4.42	2.56/2.68/2.72
17	N4C4	0	-9.76/-9.46/-3.23	1.88/1.89/2.08
18	N4C4	60	-9.89/-9.70/-3.91	1.87/1.88/2.05
19	N4C4	120	-10.20/-10.29/-5.69	1.86/1.86/2.00
20	N4C4	180	-10.38/-10.76/-6.99	1.85/1.85/1.96
21	N4C4	240	-10.11/-10.40/-5.86	1.86/1.86/2.00
22	N4C4	300	-9.89/-9.80/-4.03	1.87/1.88/2.05

1.46 2-methylamino-4-amino-pyrimidine, protonated (ncyp, for K2C)

Figure 46: The molecule used for water complex calculations corresponding to 2-methylamino-4-amino-pyrimidine, protonated (ncyp, for K2C), with possible interacting water positions. NOTE, only one water molecule was included in each calculation

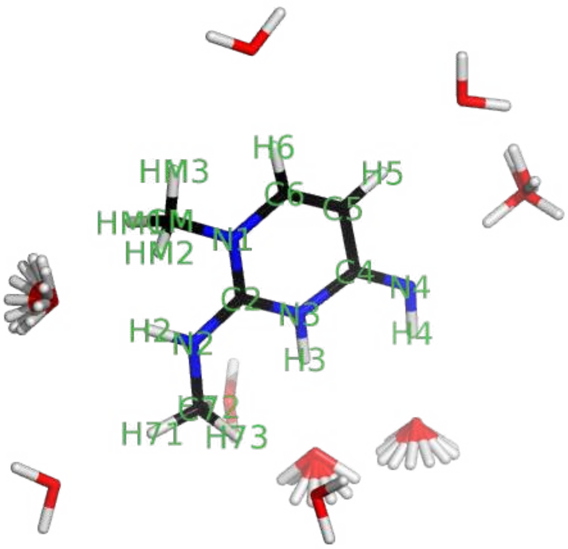

Table 136: Statistics of calculated water interaction and dipole moment for 2-methylamino-4-amino-pyrimidine, protonated (ncyp, for K2C). QM is in HF level except for thio-compounds.

	RMS/Max Deviation from QM		Dipole moment	Dipole angle
	Energy (kcal/mol)	Distance (Å)	QM/MM (debye)	difference (°)
Original	1.08/2.61	0.14/0.26	6.48/5.04	56.36
Optimized	0.21/0.43	0.20/0.46	6.48/6.57	16.33

Table 137: The comparison list of optimized atomic charges and their initial guess for 2-methylamino-4-amino-pyrimidine, protonated (ncyp, for K2C), referring to the penalties of initial guess

	Charges		
Atom	Optima	Initial	Penalty
N1	-0.233	-1.070	86.432!
C2	0.771	0.942	188.600
N2	-0.601	-0.717	186.303
H2	0.489	0.631	20.716
N3	-0.818	-0.442	60.494
H3	0.449	0.389	13.497
C4	0.562	0.329	128.700
N4	-0.675	-0.508	125.549
H4	0.343	0.264	20.206
C5	-0.246	0.086	17.737
H5	0.219	0.163	15.133
C6	0.130	0.239	17.119
H6	0.197	0.271	3.976
C7	0.143	0.153	20.270
H71	0.090	0.090	1.650
H72	0.090	0.090	1.650
H73	0.090	0.090	1.650
CM	-0.270	-0.270	73.000
HM1	0.090	0.090	3.767
HM2	0.090	0.090	3.767
HM3	0.090	0.090	3.767

Table 138: Interaction energies and geometries between probe water and selected 2-methylamino-4-amino-pyrimidine, protonated (ncyp, for K2C) site calculated using the optimized and initial charges

			QM / Optima / Initial	
N	Probe site	Angle (°)	Energy (kcal/mol)	Distance (Å)
1	H2N2	0	-10.10/-10.33/-10.26	2.27/2.46/2.38
2	H2N2	30	-10.25/-10.36/-10.19	2.28/2.46/2.38
3	H2N2	60	-10.56/-10.43/-10.10	2.30/2.46/2.39
4	H2N2	90	-10.75/-10.48/-10.06	2.31/2.45/2.39
5	H2N2	120	-10.56/-10.43/-10.10	2.30/2.46/2.39
6	H2N2	150	-10.25/-10.36/-10.19	2.28/2.46/2.38
7	H3N3	30	-10.44/-10.36/-11.40	2.10/2.56/2.52
8	H3N3	60	-10.94/-10.96/-11.97	2.27/2.52/2.48
9	H3N3	90	-11.17/-11.19/-12.25	2.27/2.50/2.46
10	H3N3	120	-10.94/-10.95/-11.97	2.27/2.52/2.48
11	H3N3	150	-10.48/-10.36/-11.40	2.27/2.56/2.52
12	H4N4	0	-7.30/-7.59/-7.70	2.10/1.93/1.98
13	H4N4	30	-7.84/-8.02/-8.14	2.07/1.92/1.97
14	H4N4	60	-8.99/-8.84/-8.98	2.01/1.90/1.94
15	H4N4	90	-9.52/-9.22/-9.38	1.99/1.89/1.93
16	H4N4	120	-8.99/-8.84/-8.98	2.01/1.90/1.94
17	H4N4	150	-7.84/-8.02/-8.14	2.07/1.92/1.97
18	H5C5	0	-6.15/-5.72/-7.17	2.20/2.49/2.46
19	H6C6	0	-8.30/-8.30/-8.64	2.23/2.29/2.26
20	H71C7	0	-7.42/-7.51/-7.70	2.28/2.49/2.48
21	H72C7	0	-8.03/-8.24/-8.68	2.28/2.47/2.46
22	H73C7	0	-8.03/-8.25/-8.68	2.28/2.47/2.46
23	N4C4	60	-3.40/-3.74/-1.84	2.06/1.95/2.02
24	N4C4	120	-2.02/-1.81/0.36	2.10/1.99/2.08
25	N4C4	180	-0.60/-0.24/2.01	2.20/2.04/2.19
26	N4C4	240	-2.02/-1.81/0.36	2.10/1.99/2.08
27	N4C4	300	-3.40/-3.74/-1.84	2.06/1.95/2.02

1.47 carboxymethyl-methyl-ammonium (nmg, for 5DU, MAU, SCU)

Figure 47: The molecule used for water complex calculations corresponding to carboxymethyl-methyl-ammonium (nmg, for 5DU, MAU, SCU), with possible interacting water positions. NOTE, only one water molecule was included in each calculation

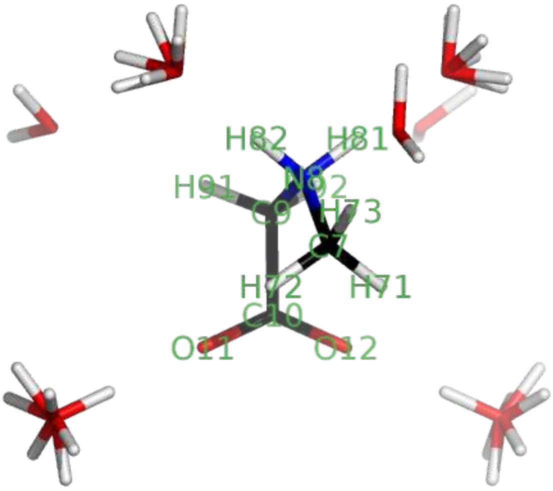

Table 139: Statistics of calculated water interaction and dipole moment for carboxymethyl-methyl-ammonium (nmg, for 5DU, MAU, SCU). QM is in HF level except for thio-compounds.

	RMS/Max Deviation from QM		Dipole moment	Dipole angle
	Energy (kcal/mol)	Distance (Å)	QM/MM (debye)	difference (°)
Original	1.98/3.22	0.07/0.08	11.48/14.27	1.98
Optimized	0.23/0.67	0.06/0.12	11.48/11.99	1.96

Table 140: The comparison list of optimized atomic charges and their initial guess for carboxymethyl-methyl-ammonium (nmg, for 5DU, MAU, SCU), referring to the penalties of initial guess

	Charges		
Atom	Optima	Initial	Penalty
C7	-0.045	0.151	17.283
H71	0.090	0.090	0.520
H72	0.090	0.090	0.520
H73	0.090	0.090	0.520
N8	-0.344	-0.491	28.149
H81	0.348	0.330	2.032
H82	0.348	0.330	2.032
C9	-0.027	0.311	101.288
H91	0.090	0.090	3.822
H92	0.090	0.090	3.822
C10	0.576	0.437	100.540
O11	-0.653	-0.759	8.442
O12	-0.653	-0.759	8.442

Table 141: Interaction energies and geometries between probe water and selected carboxymethyl-methyl-ammonium (nmg, for 5DU, MAU, SCU) site calculated using the optimized and initial charges

			QM / Optima / Initial	
N	Probe site	Angle (°)	Energy (kcal/mol)	Distance (Å)
1	H81N8	0	-12.16/-12.26/-12.26	1.72/1.78/1.79
2	H81N8	60	-12.14/-12.23/-12.23	1.72/1.78/1.79
3	H81N8	120	-12.58/-12.51/-12.68	1.71/1.77/1.78
4	H82N8	0	-12.16/-12.26/-12.26	1.72/1.78/1.79
5	H82N8	60	-12.58/-12.51/-12.68	1.71/1.77/1.79
6	H82N8	120	-12.14/-12.22/-12.23	1.72/1.78/1.79
7	H73C7	0	-4.47/-3.80/-4.82	2.45/2.57/2.52
8	H91C9	0	-2.34/-2.13/-3.21	2.51/2.62/2.55
9	H92C9	0	-2.34/-2.14/-3.21	2.51/2.62/2.55
10	O11C10	0	-8.00/-7.85/-9.76	1.74/1.71/1.67
11	O11C10	60	-9.36/-9.32/-11.69	1.73/1.70/1.66
12	O11C10	120	-10.54/-10.81/-13.66	1.72/1.69/1.64
13	O11C10	180	-10.69/-10.99/-13.91	1.71/1.68/1.64
14	O11C10	240	-9.27/-9.56/-12.00	1.73/1.69/1.65
15	O11C10	300	-7.76/-7.87/-9.81	1.75/1.71/1.67
16	O12C10	0	-8.00/-7.84/-9.76	1.74/1.71/1.67
17	O12C10	60	-7.76/-7.88/-9.80	1.75/1.71/1.67
18	O12C10	120	-9.27/-9.55/-11.99	1.73/1.70/1.65
19	O12C10	180	-10.69/-10.98/-13.91	1.71/1.68/1.64
20	O12C10	240	-10.54/-10.80/-13.66	1.72/1.69/1.64
21	O12C10	300	-9.36/-9.35/-11.69	1.73/1.70/1.66

1.48 1H-pseudoisocytosine (1PC)

Figure 48: The molecule used for water complex calculations corresponding to 1H-pseudoisocytosine (1PC), with possible interacting water positions. NOTE, only one water molecule was included in each calculation

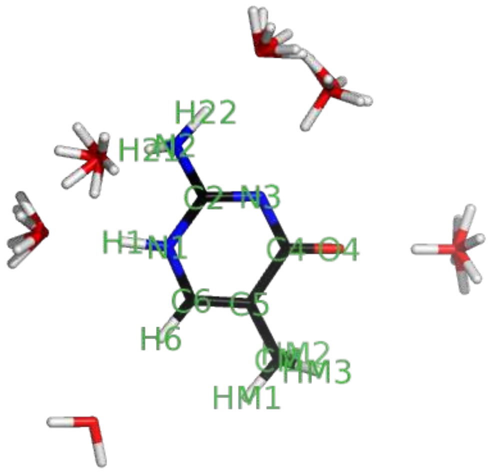

Table 142: Statistics of calculated water interaction and dipole moment for 1H-pseudoisocytosine (1PC). QM is in HF level except for thio-compounds.

	RMS/Max Deviation from QM		Dipole moment	Dipole angle
	Energy (kcal/mol)	Distance (Å)	QM/MM (debye)	difference (°)
Original	2.48/5.73	0.10/0.25	8.13/5.38	7.42
Optimized	0.30/0.72	0.04/0.06	8.13/9.51	4.69

Table 143: The comparison list of optimized atomic charges and their initial guess for 1H-pseudoisocytosine (1PC), referring to the penalties of initial guess

	Charges		
Atom	Optima	Initial	Penalty
N1	-0.360	-0.536	2.215
H1	0.290	0.219	0.000
C2	0.650	0.456	3.063
N2	-0.660	-0.667	0.827
H21	0.310	0.345	0.000
H22	0.310	0.345	0.000
N3	-0.770	-0.666	10.435
C4	0.530	0.941	10.392
O4	-0.470	-0.512	0.000
C5	-0.130	-0.229	7.844
C6	0.170	0.106	1.229
H6	0.130	0.198	0.269
CM	-0.270	-0.270	1.050
HM1	0.090	0.090	0.000
HM2	0.090	0.090	0.000
HM3	0.090	0.090	0.000

Table 144: Interaction energies and geometries between probe water and selected 1H-pseudoisocytosine (1PC) site calculated using the optimized and initial charges

N	Probe site	Angle (°)	QM / Optima / Initial Energy (kcal/mol)	QM / Optima / Initial Distance (Å)
1	H1N1	0	-8.47/-8.42/-3.07	1.81/1.85/2.05
2	H1N1	60	-9.11/-8.71/-3.38	1.79/1.84/2.03
3	H1N1	120	-8.20/-8.32/-3.02	1.82/1.85/2.06
4	H21N2	0	-6.76/-6.88/-6.13	1.91/1.89/1.87
5	H21N2	60	-7.12/-7.15/-6.19	1.89/1.88/1.87
6	H21N2	120	-6.32/-6.69/-5.95	1.93/1.90/1.88
7	H6C6	0	-3.81/-4.43/-4.04	2.40/2.35/2.34
8	O4C4	0	-7.58/-7.67/-6.02	1.80/1.76/1.78
9	O4C4	60	-7.29/-7.32/-5.75	1.81/1.76/1.79
10	O4C4	120	-6.85/-6.71/-5.31	1.82/1.77/1.80
11	O4C4	180	-6.69/-6.44/-5.13	1.82/1.78/1.80
12	O4C4	240	-6.89/-6.77/-5.38	1.82/1.77/1.80
13	O4C4	300	-7.34/-7.37/-5.82	1.81/1.76/1.79
14	N3C2	0	-9.38/-10.11/-6.97	1.87/1.90/1.95
15	N3C2	60	-8.71/-8.74/-6.16	1.87/1.91/1.97
16	N3C2	120	-5.96/-5.57/-3.97	1.92/1.96/2.01
17	N3C2	180	-3.47/-3.27/-2.06	2.04/2.02/2.08
18	N3C2	240	-5.40/-4.97/-3.36	1.93/1.97/2.02
19	N3C2	300	-8.27/-8.26/-5.68	1.87/1.92/1.97

1.49 3H-pseudoisocytosine (3PC)

Figure 49: The molecule used for water complex calculations corresponding to 3H-pseudoisocytosine (3PC), with possible interacting water positions. NOTE, only one water molecule was included in each calculation

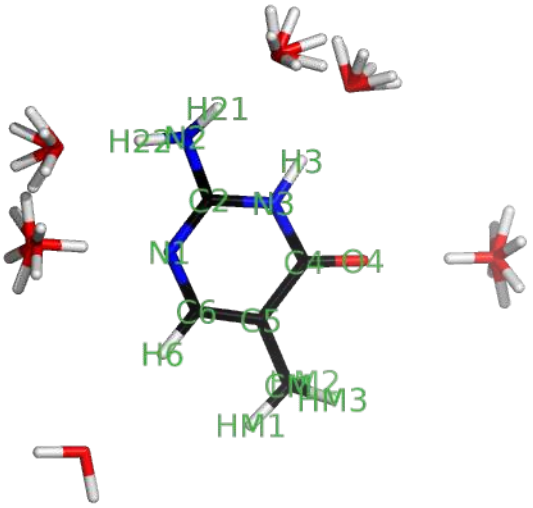

Table 145: Statistics of calculated water interaction and dipole moment for 3H-pseudoisocytosine (3PC). QM is in HF level except for thio-compounds.

	RMS/Max Deviation from QM		Dipole moment	Dipole angle
	Energy (kcal/mol)	Distance (Å)	QM/MM (debye)	difference (°)
Original	1.92/3.24	0.09/0.16	3.93/6.68	12.71
Optimized	0.32/0.65	0.05/0.12	3.93/4.11	6.29

Table 146: The comparison list of optimized atomic charges and their initial guess for 3H-pseudoisocytosine (3PC), referring to the penalties of initial guess

	Charges		
Atom	Optima	Initial	Penalty
N1	-0.780	-0.721	9.985
C2	0.630	0.846	4.681
N2	-0.560	-0.667	0.760
H21	0.310	0.345	0.000
H22	0.310	0.345	0.000
N3	-0.510	-0.388	2.670
H3	0.350	0.233	0.000
C4	0.460	0.192	4.190
O4	-0.480	-0.441	0.000
C5	-0.160	-0.192	7.069
C6	0.280	0.265	10.263
H6	0.150	0.183	3.723
CM	-0.270	-0.270	3.479
HM1	0.090	0.090	0.000
HM2	0.090	0.090	0.000
HM3	0.090	0.090	0.000

Table 147: Interaction energies and geometries between probe water and selected 3H-pseudoisocytosine (3PC) site calculated using the optimized and initial charges

			QM / Optima / Initial	
N	Probe site	Angle (°)	Energy (kcal/mol)	Distance (Å)
1	H21N2	0	-7.56/-8.00/-9.50	1.88/1.86/1.82
2	H21N2	60	-7.20/-7.86/-9.33	1.89/1.86/1.82
3	H21N2	120	-7.91/-8.19/-9.46	1.85/1.85/1.82
4	H22N2	0	-5.72/-5.25/-7.21	1.83/1.88/1.84
5	H22N2	60	-5.03/-4.82/-6.88	1.87/1.89/1.85
6	H22N2	120	-5.05/-4.88/-6.91	1.87/1.89/1.85
7	H3N3	0	-7.60/-7.38/-4.40	1.78/1.83/1.93
8	H3N3	60	-6.94/-7.11/-4.17	1.80/1.84/1.94
9	H3N3	120	-7.71/-7.40/-4.48	1.78/1.83/1.93
10	H6C6	0	-2.03/-2.43/-3.29	2.51/2.39/2.34
11	N1C2	0	-6.38/-6.86/-5.15	1.90/1.91/1.95
12	N1C2	60	-6.78/-6.47/-4.64	1.86/1.91/1.95
13	N1C2	120	-5.47/-5.03/-2.93	1.90/1.94/1.99
14	N1C2	180	-4.01/-3.93/-1.74	1.99/1.97/2.04
15	N1C2	240	-6.18/-5.67/-3.65	1.88/1.93/1.97
16	N1C2	300	-7.09/-6.87/-5.11	1.86/1.91/1.94
17	O4C4	0	-5.78/-5.62/-6.41	1.84/1.78/1.77
18	O4C4	60	-5.96/-5.86/-6.64	1.84/1.77/1.77
19	O4C4	120	-6.27/-6.34/-7.10	1.83/1.77/1.76
20	O4C4	180	-6.43/-6.47/-7.32	1.83/1.77/1.76
21	O4C4	240	-6.36/-6.38/-7.19	1.83/1.77/1.76
22	O4C4	300	-6.05/-5.96/-6.75	1.84/1.77/1.77

1.50 Pseudouracil (PSU)

Figure 50: The molecule used for water complex calculations corresponding to Pseudouracil (PSU), with possible interacting water positions. NOTE, only one water molecule was included in each calculation

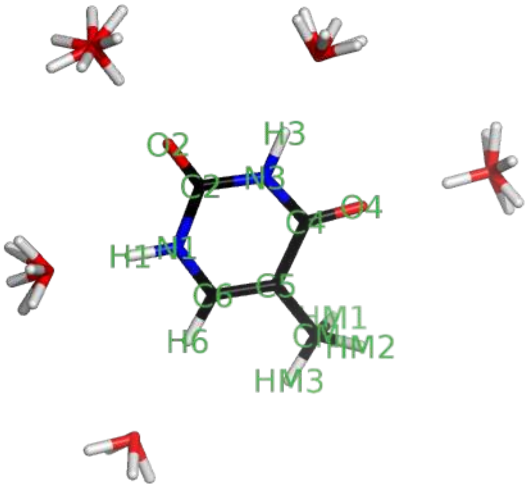

Table 148: Statistics of calculated water interaction and dipole moment for Pseudouracil (PSU). QM is in HF level except for thio-compounds.

	RMS/Max Deviation from QM		Dipole moment	Dipole angle
	Energy (kcal/mol)	Distance (Å)	QM/MM (debye)	difference (°)
Original	0.45/1.06	0.06/0.07	4.86/5.20	11.39
Optimized	0.14/0.40	0.07/0.08	4.86/5.08	5.80

Table 149: The comparison list of optimized atomic charges and their initial guess for Pseudouracil (PSU), referring to the penalties of initial guess

	Charges		
Atom	Optima	Initial	Penalty
C5	-0.162	-0.189	0.000
C4	0.532	0.508	0.000
O4	-0.460	-0.441	0.000
N3	-0.517	-0.525	0.000
H3	0.363	0.371	0.000
C2	0.455	0.446	0.000
O2	-0.459	-0.443	0.000
N1	-0.363	-0.363	0.000
H1	0.328	0.357	0.000
C6	0.101	0.080	0.000
H6	0.182	0.199	0.000
CM	-0.270	-0.270	0.000
HM1	0.090	0.090	0.000
HM2	0.090	0.090	0.000
HM3	0.090	0.090	0.000

Table 150: Interaction energies and geometries between probe water and selected Pseudouracil (PSU) site calculated using the optimized and initial charges

			QM / Optima / Initial	
N	Probe site	Angle (°)	Energy (kcal/mol)	Distance (Å)
1	H1N1	0	-8.07/-8.00/-8.99	1.77/1.82/1.80
2	H1N1	60	-7.80/-7.87/-8.87	1.77/1.83/1.80
3	H1N1	120	-7.80/-7.85/-8.87	1.77/1.83/1.80
4	H3N3	0	-6.87/-6.47/-6.53	1.75/1.81/1.81
5	H3N3	60	-5.80/-5.92/-6.02	1.78/1.82/1.82
6	H3N3	120	-5.80/-5.92/-6.02	1.78/1.82/1.82
7	H6C6	0	-4.65/-4.97/-5.12	2.34/2.32/2.31
8	H6C6	90	-5.18/-5.10/-5.27	2.28/2.32/2.31
9	O2C2	0	-5.32/-5.46/-5.23	1.87/1.79/1.80
10	O2C2	60	-5.32/-5.38/-5.13	1.87/1.79/1.80
11	O2C2	120	-5.05/-5.06/-4.76	1.87/1.79/1.80
12	O2C2	180	-4.87/-4.81/-4.48	1.88/1.80/1.81
13	O2C2	240	-5.05/-5.06/-4.76	1.87/1.79/1.80
14	O2C2	300	-5.32/-5.38/-5.13	1.87/1.79/1.80
15	O4C4	0	-5.36/-5.29/-5.12	1.87/1.79/1.81
16	O4C4	60	-5.40/-5.35/-5.23	1.87/1.80/1.80
17	O4C4	120	-5.32/-5.42/-5.33	1.87/1.79/1.80
18	O4C4	180	-5.27/-5.40/-5.34	1.87/1.79/1.80
19	O4C4	240	-5.32/-5.42/-5.33	1.87/1.79/1.80
20	O4C4	300	-5.40/-5.38/-5.23	1.87/1.79/1.80

1.51 Adenine protonated (ADEp)

Figure 51: The molecule used for water complex calculations corresponding to Adenine protonated (ADEp), with possible interacting water positions. NOTE, only one water molecule was included in each calculation

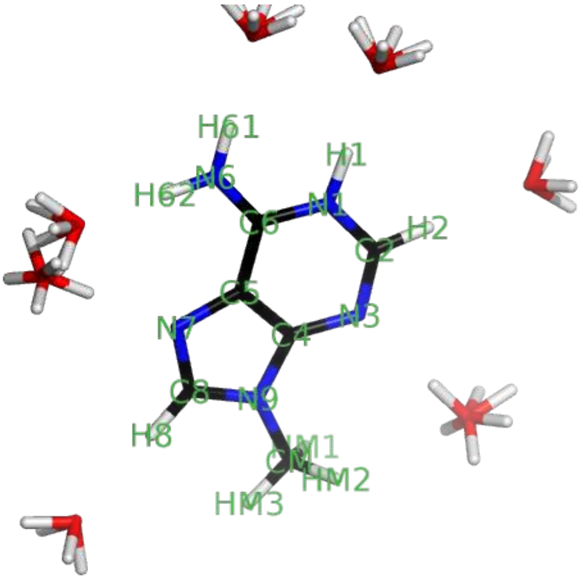

Table 151: Statistics of calculated water interaction and dipole moment for Adenine protonated (ADEp). QM is in HF level except for thio-compounds.

	RMS/Max Deviation from QM		Dipole moment	Dipole angle
	Energy (kcal/mol)	Distance (Å)	QM/MM (debye)	difference (°)
Original	2.73/5.68	0.14/0.25	4.63/9.86	20.73
Optimized	0.34/0.57	0.08/0.16	4.63/5.13	8.51

Table 152: The comparison list of optimized atomic charges and their initial guess for Adenine protonated (ADEp), referring to the penalties of initial guess

	Charges		
Atom	Optima	Initial	Penalty
N9	0.069	-0.047	0.000
C8	0.387	0.339	0.000
H8	0.120	0.123	0.000
N7	-0.721	-0.734	13.119
C5	0.254	0.243	24.638
C6	0.697	1.317	201.211
N6	-0.779	-1.132	201.722
H61	0.405	0.460	20.716
H62	0.397	0.460	20.716
N1	-0.768	-0.745	28.162!
H1	0.447	0.493	15.609
C2	0.314	0.486	19.163
H2	0.254	0.102	13.982
N3	-0.598	-0.810	1.258
C4	0.522	0.445	11.182
CM	-0.270	-0.270	0.000
HM1	0.090	0.090	0.000
HM2	0.090	0.090	0.000
HM3	0.090	0.090	0.000

Table 153: Interaction energies and geometries between probe water and selected Adenine protonated (ADEp) site calculated using the optimized and initial charges

			QM / Optima / Initial	
N	Probe site	Angle (°)	Energy (kcal/mol)	Distance (Å)
1	H61N6	0	-12.80/-13.29/-15.62	1.85/1.80/1.78
2	H61N6	60	-14.18/-13.95/-16.44	1.80/1.79/1.77
3	H61N6	120	-14.18/-13.96/-16.44	1.80/1.79/1.77
4	H62N6	0	-11.92/-11.35/-13.98	1.74/1.79/1.75
5	H62N6	60	-10.39/-10.56/-12.98	1.78/1.81/1.77
6	H62N6	120	-10.39/-10.56/-12.98	1.78/1.81/1.77
7	H1N1	0	-14.90/-15.46/-20.58	1.80/1.77/1.71
8	H1N1	60	-16.51/-16.25/-21.31	1.76/1.76/1.70
9	H1N1	120	-16.51/-16.25/-21.31	1.76/1.76/1.70
10	H8C8	0	-7.67/-8.01/-6.87	2.17/2.16/2.18
11	H8C8	90	-8.20/-8.02/-6.87	2.14/2.16/2.18
12	H2C2	0	-9.27/-9.42/-7.48	2.11/2.26/2.33
13	H2C2	90	-9.77/-9.57/-7.64	2.09/2.25/2.32
14	N7C8	60	-1.77/-1.24/-0.46	2.02/1.93/1.93
15	N7C8	120	-4.26/-4.59/-4.89	1.97/1.88/1.87
16	N7C8	240	-4.26/-4.59/-4.89	1.97/1.88/1.87
17	N7C8	300	-1.77/-1.24/-0.46	2.02/1.93/1.93
18	N3C4	0	-1.12/-0.91/-3.25	2.20/2.09/1.99
19	N3C4	60	-1.31/-0.99/-3.49	2.17/2.08/1.98
20	N3C4	120	-0.20/-0.44/-3.44	2.23/2.09/1.98
21	N3C4	240	-0.20/-0.44/-3.44	2.23/2.09/1.98
22	N3C4	300	-1.31/-0.99/-3.49	2.17/2.08/1.98

1.52 Cytosine protonated (CYTp)

Figure 52: The molecule used for water complex calculations corresponding to Cytosine protonated (CYTp), with possible interacting water positions. NOTE, only one water molecule was included in each calculation

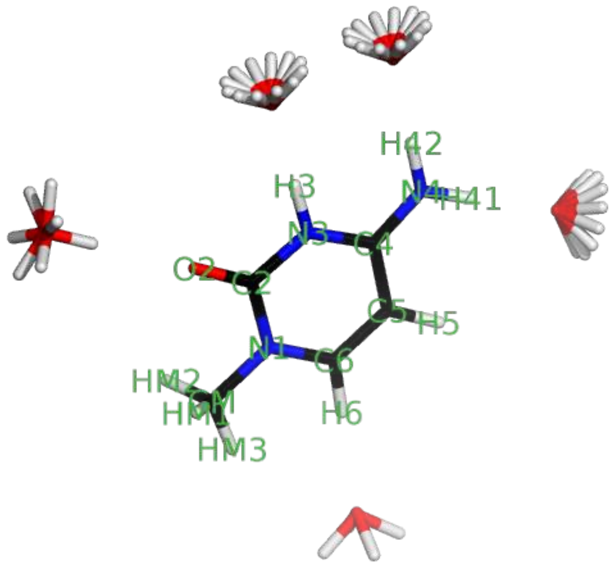

Table 154: Statistics of calculated water interaction and dipole moment for Cytosine protonated (CYTp). QM is in HF level except for thio-compounds.

	RMS/Max Deviation from QM		Dipole moment	Dipole angle
	Energy (kcal/mol)	Distance (Å)	QM/MM (debye)	difference (°)
Original	4.67/7.18	0.19/0.39	6.20/14.75	22.26
Optimized	0.30/0.53	0.13/0.28	6.20/7.00	9.17

Table 155: The comparison list of optimized atomic charges and their initial guess for Cytosine protonated (CYTp), referring to the penalties of initial guess

	Charges		
Atom	Optima	Initial	Penalty
N1	-0.097	-0.351	1.851
C2	0.780	0.252	12.754
O2	-0.390	-0.409	1.588
N3	-0.707	-0.543	25.065!
H3	0.396	0.496	13.882
C4	0.743	1.584	200.584
N4	-0.785	-1.152	201.616
H41	0.402	0.460	20.716
H42	0.413	0.460	20.716
C5	-0.152	-0.027	19.624
H5	0.059	0.019	3.275
C6	0.088	0.041	11.887
H6	0.250	0.170	0.000
CM	-0.270	-0.270	0.000
HM1	0.090	0.090	0.000
HM2	0.090	0.090	0.000
HM3	0.090	0.090	0.000

Table 156: Interaction energies and geometries between probe water and selected Cytosine protonated (CYTp) site calculated using the optimized and initial charges

			QM / Optima / Initial	
N	Probe site	Angle (°)	Energy (kcal/mol)	Distance (Å)
1	H3N3	0	-14.43/-14.47/-21.61	1.77/1.78/1.69
2	H3N3	30	-14.73/-14.83/-21.73	1.76/1.77/1.69
3	H3N3	60	-15.33/-15.26/-21.94	1.75/1.77/1.68
4	H3N3	90	-15.61/-15.39/-22.01	1.74/1.77/1.68
5	H3N3	120	-15.33/-15.29/-21.94	1.75/1.77/1.68
6	H3N3	150	-14.73/-14.81/-21.73	1.76/1.78/1.69
7	H41N4	0	-13.39/-13.92/-18.08	1.81/1.79/1.75
8	H41N4	30	-13.77/-14.05/-18.21	1.80/1.79/1.75
9	H41N4	60	-14.50/-14.22/-18.46	1.78/1.78/1.74
10	H41N4	90	-14.87/-14.42/-18.60	1.77/1.78/1.74
11	H41N4	120	-14.50/-14.22/-18.46	1.78/1.78/1.74
12	H41N4	150	-13.77/-14.05/-18.21	1.80/1.79/1.75
13	H42N4	0	-14.12/-14.56/-18.83	1.82/1.79/1.76
14	H42N4	30	-14.58/-14.85/-19.08	1.80/1.78/1.75
15	H42N4	60	-15.50/-15.22/-19.61	1.78/1.78/1.74
16	H42N4	90	-15.96/-15.49/-19.86	1.76/1.77/1.74
17	H42N4	120	-15.50/-15.28/-19.61	1.78/1.78/1.74
18	H42N4	150	-14.58/-14.85/-19.08	1.80/1.78/1.75
19	H6C6	0	-10.00/-10.42/-7.63	2.13/2.24/2.30
20	H6C6	90	-11.09/-10.59/-7.70	2.09/2.23/2.30
21	O2C2	0	-0.48/-0.19/-1.90	2.18/1.93/1.84
22	O2C2	60	-0.62/-0.46/-2.72	2.17/1.92/1.82
23	O2C2	120	-0.64/-0.84/-4.17	2.17/1.91/1.80
24	O2C2	180	-0.52/-0.95/-4.80	2.18/1.91/1.79
25	O2C2	240	-0.64/-0.83/-4.17	2.17/1.92/1.80
26	O2C2	300	-0.62/-0.47/-2.72	2.17/1.92/1.82

1.53 2-methylthio-N6-methyladenine (SMA)

Figure 53: The molecule used for water complex calculations corresponding to 2-methylthio-N6-methyladenine (SMA), with possible interacting water positions. NOTE, only one water molecule was included in each calculation

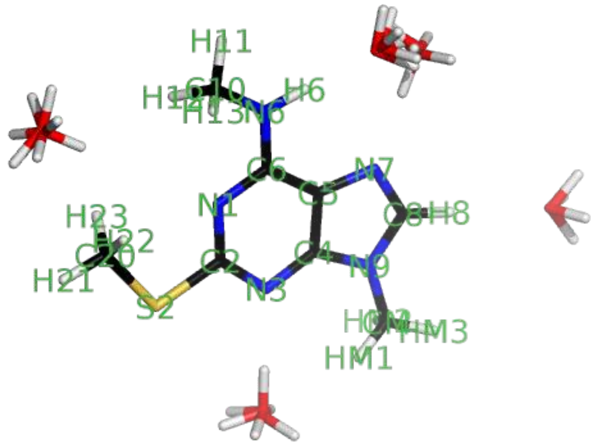

Table 157: Statistics of calculated water interaction and dipole moment for 2-methylthio-N6-methyladenine (SMA). QM is in HF level except for thio-compounds.

	RMS/Max Deviation from QM		Dipole moment	Dipole angle
	Energy (kcal/mol)	Distance (Å)	QM/MM (debye)	difference (°)
Original	1.31/2.53	0.15/0.29	2.38/4.16	17.58
Optimized	0.33/0.78	0.11/0.21	2.38/3.31	28.46

Table 158: The comparison list of optimized atomic charges and their initial guess for 2-methylthio-N6-methyladenine (SMA), referring to the penalties of initial guess

	Charges		
Atom	Optima	Initial	Penalty
N9	-0.010	-0.010	0.000
C8	0.376	0.341	0.000
H8	0.098	0.120	0.000
N7	-0.837	-0.707	2.500
C5	0.283	0.283	21.933
C6	0.382	0.499	46.435
N6	-0.483	-0.583	40.152
H6	0.356	0.361	3.233
C10	-0.096	-0.190	17.069
H11	0.090	0.090	0.600
H12	0.090	0.090	0.600
H13	0.090	0.090	0.600
N1	-0.584	-0.663	45.097
C2	0.631	0.639	50.724
N3	-0.764	-0.694	32.993
C4	0.477	0.434	3.536
S2	-0.272	-0.276	50.095
C20	-0.097	-0.094	21.807
H21	0.090	0.090	1.625
H22	0.090	0.090	1.625
H23	0.090	0.090	1.625
CM	-0.270	-0.270	0.000
HM1	0.090	0.090	0.000
HM2	0.090	0.090	0.000
HM3	0.090	0.090	0.000

Table 159: Interaction energies and geometries between probe water and selected 2-methylthio-N6-methyladenine (SMA) site calculated using the optimized and initial charges

			QM / Optima / Initial	
N	Probe site	Angle (°)	Energy (kcal/mol)	Distance (Å)
1	H6N6	0	-4.83/-4.05/-3.37	1.80/2.01/2.04
2	H6N6	60	-3.23/-3.24/-2.84	1.87/2.04/2.07
3	H6N6	120	-2.16/-2.74/-2.46	1.93/2.06/2.09
4	H8C8	0	-3.23/-3.44/-4.48	2.38/2.24/2.20
5	H8C8	90	-3.49/-3.40/-4.45	2.35/2.24/2.21
6	N1C2	0	-0.03/-0.24/-1.37	3.77/3.60/3.48
7	N1C2	60	-0.30/-0.50/-1.29	3.71/3.56/3.50
8	N1C2	120	-0.56/-0.51/-0.83	3.71/3.60/3.59
9	N1C2	180	-0.47/-0.31/-0.47	3.79/3.67/3.68
10	N1C2	240	-0.47/-0.41/-0.76	3.73/3.61/3.59
11	N1C2	300	-0.30/-0.36/-1.20	3.69/3.57/3.50
12	N3C4	0	-7.40/-7.85/-7.10	1.94/1.99/2.01
13	N3C4	60	-7.14/-7.03/-6.18	1.93/2.00/2.03
14	N3C4	120	-5.72/-5.29/-4.36	1.96/2.03/2.07
15	N3C4	240	-4.65/-4.97/-4.06	2.03/2.05/2.09
16	N3C4	300	-7.32/-6.85/-6.04	1.93/2.00/2.04
17	N7C5	60	-8.11/-8.51/-5.83	1.84/1.84/1.90
18	N7C5	120	-7.40/-6.97/-5.09	1.85/1.86/1.91
19	N7C5	180	-4.47/-4.57/-3.38	1.98/1.91/1.97
20	N7C5	240	-6.52/-6.30/-4.45	1.87/1.87/1.92
21	N7C5	300	-8.03/-8.17/-5.50	1.84/1.85/1.90

1.54 N4,N4,2'-O-trimethylcytosine (TMC)

Figure 54: The molecule used for water complex calculations corresponding to N4,N4,2'-O-trimethylcytosine (TMC), with possible interacting water positions. NOTE, only one water molecule was included in each calculation

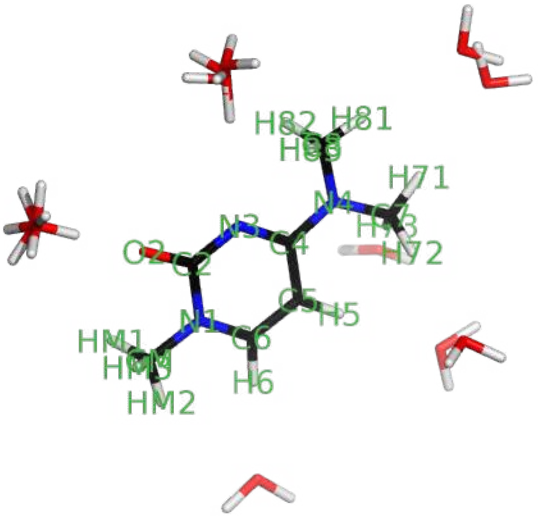

Table 160: Statistics of calculated water interaction and dipole moment for N4,N4,2'-O-trimethylcytosine (TMC). QM is in HF level except for thio-compounds.

	RMS/Max Deviation from QM		Dipole moment	Dipole angle
	Energy (kcal/mol)	Distance (Å)	QM/MM (debye)	difference (°)
Original	0.66/1.41	0.11/0.27	6.91/8.02	4.18
Optimized	0.18/0.41	0.06/0.18	6.91/8.18	6.99

Table 161: The comparison list of optimized atomic charges and their initial guess for N4,N4,2'-O-trimethylcytosine (TMC), referring to the penalties of initial guess

	Charges		
Atom	Optima	Initial	Penalty
N1	-0.031	-0.136	4.542
C2	0.565	0.512	5.336
O2	-0.513	-0.480	0.000
N3	-0.741	-0.662	31.009
C4	0.594	0.837	60.874
N4	-0.369	-0.527	52.158
C5	-0.177	-0.047	35.560
H5	0.040	0.025	1.830
C6	0.107	0.056	13.710
H6	0.135	0.170	0.000
C7	-0.075	-0.144	23.362
H71	0.090	0.090	2.657
H72	0.090	0.090	2.657
H73	0.090	0.090	2.657
C8	-0.075	-0.144	23.362
H81	0.090	0.090	2.657
H82	0.090	0.090	2.657
H83	0.090	0.090	2.657
CM	-0.270	-0.270	3.605
HM1	0.090	0.090	0.000
HM2	0.090	0.090	0.000
HM3	0.090	0.090	0.000

Table 162: Interaction energies and geometries between probe water and selected N4,N4,2'-O-trimethylcytosine (TMC) site calculated using the optimized and initial charges

			QM / Optima / Initial	
N	Probe site	Angle (°)	Energy (kcal/mol)	Distance (Å)
1	H5C5	0	-2.65/-2.54/-3.25	2.68/2.61/2.52
2	H6C6	0	-4.07/-4.13/-4.92	2.39/2.35/2.33
3	H71C7	0	-2.61/-2.20/-1.19	2.62/2.64/2.72
4	H72C7	0	-2.12/-2.28/-2.10	2.61/2.61/2.66
5	H73C7	0	-2.12/-2.28/-2.10	2.61/2.61/2.66
6	H81C8	0	-1.94/-1.67/-0.69	2.75/2.69/2.80
7	N3C4	0	-4.47/-4.71/-3.71	2.65/2.84/2.93
8	N3C4	60	-3.49/-3.68/-2.96	2.81/2.89/2.98
9	N3C4	120	-1.85/-1.62/-1.62	3.00/3.04/3.10
10	N3C4	240	-1.85/-1.62/-1.62	3.00/3.04/3.10
11	N3C4	300	-3.49/-3.68/-2.96	2.81/2.89/2.98
12	O2C2	0	-6.60/-6.73/-6.33	1.81/1.76/1.77
13	O2C2	60	-7.00/-7.07/-6.60	1.81/1.76/1.77
14	O2C2	120	-7.76/-7.73/-7.15	1.79/1.75/1.76
15	O2C2	180	-8.07/-8.03/-7.43	1.78/1.74/1.76
16	O2C2	240	-7.76/-7.72/-7.15	1.79/1.75/1.76
17	O2C2	300	-7.00/-7.07/-6.60	1.81/1.76/1.77

1.55 1,3,5-trimethyl-pseudouracil (mdmp, for 13P)

Figure 55: The molecule used for water complex calculations corresponding to 1,3,5-trimethyl-pseudouracil (mdmp, for 13P), with possible interacting water positions. NOTE, only one water molecule was included in each calculation

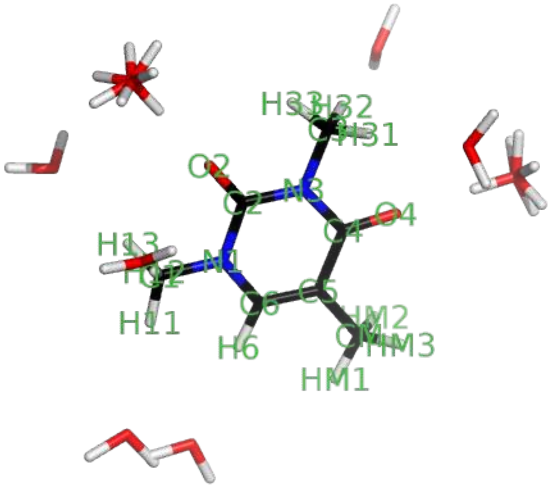

Table 163: Statistics of calculated water interaction and dipole moment for 1,3,5-trimethyl-pseudouracil (mdmp, for 13P). QM is in HF level except for thio-compounds.

	RMS/Max Deviation from QM		Dipole moment	Dipole angle
	Energy (kcal/mol)	Distance (Å)	QM/MM (debye)	difference (°)
Original	1.23/1.72	0.06/0.15	4.53/5.41	19.55
Optimized	0.20/0.51	0.07/0.08	4.53/4.74	0.62

Table 164: The comparison list of optimized atomic charges and their initial guess for 1,3,5-trimethyl-pseudouracil (mdmp, for 13P), referring to the penalties of initial guess

	Charges		
Atom	Optima	Initial	Penalty
C5	-0.214	-0.132	6.841
C4	0.733	0.595	6.708
O4	-0.512	-0.399	0.000
N3	-0.442	-0.407	4.743
C2	0.858	0.590	0.000
O2	-0.530	-0.359	0.000
N1	-0.330	-0.255	0.000
C6	0.025	0.176	0.000
H6	0.195	0.179	0.000
C1	-0.136	-0.264	0.000
H11	0.090	0.090	0.000
H12	0.090	0.090	0.000
H13	0.090	0.090	0.000
C3	-0.187	-0.264	0.750
H31	0.090	0.090	0.000
H32	0.090	0.090	0.000
H33	0.090	0.090	0.000
CM	-0.270	-0.270	0.000
HM1	0.090	0.090	0.000
HM2	0.090	0.090	0.000
HM3	0.090	0.090	0.000

Table 165: Interaction energies and geometries between probe water and selected 1,3,5-trimethyl-pseudouracil (mdmp, for 13P) site calculated using the optimized and initial charges

			QM / Optima / Initial	
N	Probe site	Angle (°)	Energy (kcal/mol)	Distance (Å)
1	H6C6	0	-4.38/-4.47/-5.32	2.39/2.34/2.31
2	H11C1	0	-3.36/-2.85/-2.46	2.60/2.61/2.67
3	H12C1	0	-1.41/-1.22/-0.63	2.57/2.64/2.72
4	H13C1	0	-1.41/-1.22/-0.63	2.57/2.64/2.72
5	H31C3	0	-0.34/0.01/na	2.71/2.75/na
6	H32C3	0	-0.34/0.01/na	2.71/2.75/na
7	O2C2	0	-5.45/-5.69/-4.31	1.86/1.78/1.85
8	O2C2	60	-5.49/-5.58/-4.16	1.85/1.78/1.85
9	O2C2	120	-5.27/-5.23/-3.84	1.85/1.78/1.86
10	O2C2	180	-5.05/-4.95/-3.66	1.86/1.78/1.86
11	O2C2	240	-5.27/-5.24/-3.84	1.85/1.78/1.86
12	O2C2	300	-5.49/-5.57/-4.16	1.85/1.78/1.85
13	O4C4	0	-5.67/-5.85/-4.86	1.85/1.78/1.82
14	O4C4	60	-5.76/-5.84/-4.66	1.85/1.78/1.83
15	O4C4	120	-5.76/-5.78/-4.25	1.85/1.78/1.83
16	O4C4	180	-5.76/-5.74/-4.04	1.85/1.78/1.84
17	O4C4	240	-5.76/-5.79/-4.25	1.85/1.77/1.83
18	O4C4	300	-5.76/-5.84/-4.66	1.85/1.78/1.83

1.56 N1,N1,N2-trimethylurea (tmu, for 66A)

Figure 56: The molecule used for water complex calculations corresponding to N1,N1,N2-trimethylurea (tmu, for 66A), with possible interacting water positions. NOTE, only one water molecule was included in each calculation

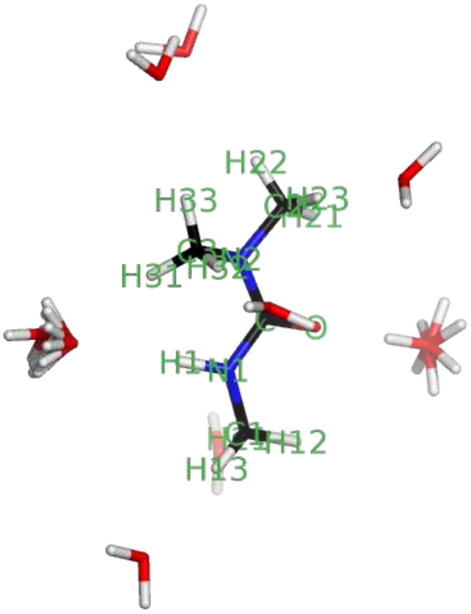

Table 166: Statistics of calculated water interaction and dipole moment for N1,N1,N2-trimethylurea (tmu, for 66A). QM is in HF level except for thio-compounds.

	RMS/Max Deviation from QM		Dipole moment	Dipole angle
	Energy (kcal/mol)	Distance (Å)	QM/MM (debye)	difference (°)
Original	0.50/1.05	0.17/0.30	3.90/4.45	4.24
Optimized	0.12/0.23	0.13/0.23	3.90/4.70	0.76

Table 167: The comparison list of optimized atomic charges and their initial guess for N1,N1,N2-trimethylurea (tmu, for 66A), referring to the penalties of initial guess

	Charges		
Atom	Optima	Initial	Penalty
N1	-0.445	-0.385	25.359
H1	0.373	0.307	0.600
C1	-0.106	-0.041	12.017
H11	0.090	0.090	0.000
H12	0.090	0.090	0.000
H13	0.090	0.090	0.000
C	0.264	0.084	8.956
O	-0.426	-0.388	4.495
N2	-0.298	-0.199	28.507
C2	-0.086	-0.094	15.662
H21	0.090	0.090	0.175
H22	0.090	0.090	0.175
H23	0.090	0.090	0.175
C3	-0.086	-0.094	15.662
H31	0.090	0.090	0.175
H32	0.090	0.090	0.175
H33	0.090	0.090	0.175

Table 168: Interaction energies and geometries between probe water and selected N1,N1,N2-trimethylurea (tmu, for 66A) site calculated using the optimized and initial charges

			QM / Optima / Initial	
N	Probe site	Angle (°)	Energy (kcal/mol)	Distance (Å)
1	H1N1	0	-4.18/-4.22/-3.37	2.35/2.55/2.63
2	H1N1	30	-4.58/-4.39/-3.55	2.31/2.53/2.61
3	H1N1	60	-4.60/-4.38/-3.55	2.30/2.53/2.61
4	H1N1	90	-4.27/-4.19/-3.38	2.34/2.55/2.63
5	H1N1	120	-3.85/-4.00/-3.19	2.39/2.57/2.65
6	H1N1	150	-3.81/-4.01/-3.19	2.40/2.57/2.65
7	H11C1	0	-0.58/-0.37/-0.57	2.79/2.70/2.66
8	H13C1	0	-1.36/-1.31/-1.59	2.76/2.69/2.65
9	H22C2	0	-1.36/-1.25/-1.31	2.75/2.68/2.67
10	H23C2	0	-0.74/-0.63/-0.58	2.83/2.70/2.70
11	H31C3	0	-3.45/-3.53/-3.00	2.57/2.55/2.57
12	H32C3	0	-1.41/-1.43/-1.27	2.68/2.62/2.63
13	H33C3	0	-2.05/-1.89/-1.92	2.66/2.64/2.64
14	OC	0	-6.63/-6.67/-6.46	1.81/1.77/1.78
15	OC	60	-6.72/-6.69/-6.56	1.81/1.77/1.77
16	OC	120	-6.65/-6.67/-6.64	1.81/1.77/1.77
17	OC	180	-6.54/-6.54/-6.56	1.82/1.77/1.78
18	OC	240	-6.69/-6.70/-6.68	1.81/1.77/1.77
19	OC	300	-6.72/-6.77/-6.66	1.81/1.77/1.77

2 The Geometric Information of Modified Bases

2.1 1-methyl guanine (1MG)

Figure 57: The energy-minimized structure of 1MG.

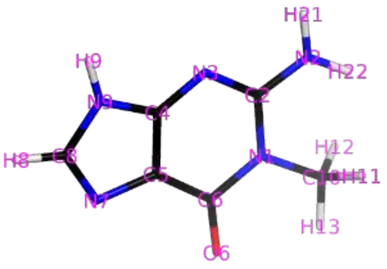

Table 169: The calculated geometric terms of 1MG.

Terms	QM	MM	diff
N9-C8	1.375	1.370	-0.005
N9-C4	1.371	1.348	-0.023
N9-H9	1.013	1.001	-0.012
C8-H8	1.083	1.093	0.010
C8-N7	1.323	1.321	-0.003
N7-C5	1.379	1.392	0.012
C5-C6	1.437	1.416	-0.021
C5-C4	1.390	1.398	0.008
C6-O6	1.228	1.237	0.010
C6-N1	1.446	1.418	-0.028
N1-C2	1.378	1.406	0.028
N1-C10	1.463	1.489	0.025
C2-N2	1.388	1.332	-0.056
C2-N3	1.315	1.339	0.024
N2-H21	1.016	0.994	-0.021
N2-H22	1.014	0.992	-0.022
N3-C4	1.359	1.324	-0.036
C10-H11	1.091	1.114	0.023
C10-H12	1.095	1.114	0.019
C10-H13	1.088	1.114	0.026
C8-N9-C4	106.80	107.37	0.56
C8-N9-H9	127.73	130.09	2.36
C4-N9-H9	125.47	122.55	-2.92
N9-C8-H8	121.86	123.12	1.26
N9-C8-N7	113.00	112.46	-0.54
H8-C8-N7	125.13	124.42	-0.71
C8-N7-C5	103.71	104.53	0.82
N7-C5-C6	128.99	131.51	2.52
N7-C5-C4	111.61	109.52	-2.09
C6-C5-C4	119.40	118.97	-0.43
C5-C6-O6	129.08	126.96	-2.12
C5-C6-N1	110.27	113.49	3.23
O6-C6-N1	120.65	119.55	-1.11
C6-N1-C2	124.16	122.37	-1.79
C6-N1-C10	116.09	118.39	2.30
C2-N1-C10	119.75	119.24	-0.51
N1-C2-N2	116.48	118.64	2.16
N1-C2-N3	125.37	122.91	-2.46
N2-C2-N3	118.01	118.45	0.44
C2-N2-H21	109.95	116.63	6.68
C2-N2-H22	115.89	121.89	6.00
H21-N2-H22	112.13	121.48	9.35
C2-N3-C4	111.92	114.86	2.94
N9-C4-C5	104.88	106.13	1.25
N9-C4-N3	126.29	126.47	0.18
C5-C4-N3	128.81	127.40	-1.41
N1-C10-H11	109.62	111.42	1.80
N1-C10-H12	111.12	111.42	0.30
N1-C10-H13	106.63	113.50	6.87
H11-C10-H12	110.03	108.27	-1.76
H11-C10-H13	110.76	105.94	-4.82
H12-C10-H13	108.64	105.94	-2.70
C4-N9-C8-H8	179.75	-180.00	0.25
C4-N9-C8-N7	-0.22	-0.00	0.22
H9-N9-C8-H8	0.11	-0.00	-0.11
H9-N9-C8-N7	-179.86	180.00	0.14
C8-N9-C4-C5	0.25	0.00	-0.25
C8-N9-C4-N3	178.68	180.00	1.32
H9-N9-C4-C5	179.89	-180.00	0.11
H9-N9-C4-N3	-1.68	0.00	1.68
N9-C8-N7-C5	0.09	0.00	-0.09
H8-C8-N7-C5	-179.88	180.00	0.12
C8-N7-C5-C6	179.85	180.00	0.15
C8-N7-C5-C4	0.07	0.00	-0.07
N7-C5-C6-O6	0.39	0.00	-0.39
N7-C5-C6-N1	-178.84	-180.00	-1.16
C4-C5-C6-O6	-179.85	-180.00	-0.15
C4-C5-C6-N1	0.92	0.00	-0.92
N7-C5-C4-N9	-0.21	0.00	0.21
N7-C5-C4-N3	-178.58	-180.00	-1.42
C6-C5-C4-N9	179.99	-180.00	0.01
C6-C5-C4-N3	1.62	0.00	-1.62
C5-C6-N1-C2	-2.74	-0.00	2.74
C5-C6-N1-C10	176.19	-180.00	3.81
O6-C6-N1-C2	177.96	180.00	2.04
O6-C6-N1-C10	-3.11	0.00	3.11
C6-N1-C2-N2	177.85	-180.00	2.15
C6-N1-C2-N3	2.33	0.00	-2.33
C10-N1-C2-N2	-1.05	0.00	1.05
C10-N1-C2-N3	-176.57	180.00	3.43
N1-C2-N2-H21	172.24	-180.00	7.76
N1-C2-N2-H22	43.80	0.00	-43.80
N3-C2-N2-H21	-11.90	0.00	11.90
N3-C2-N2-H22	-140.33	-180.00	-39.67
N1-C2-N3-C4	0.25	0.00	-0.25
N2-C2-N3-C4	-175.21	180.00	4.79
C2-N3-C4-N9	179.70	-180.00	0.30
C2-N3-C4-C5	-2.25	0.00	2.25

Table 170: The statistics of vibrational frequencies of 1MG. Only the terms with the occupancies greater than 15% were shown. Refer to p.6 for the meanings of assig.

QM (MP2, scaled by 0.943)							MM						
Freq	Assig.	%	Assig.	%	Assig.	%	Freq	Assig.	%	Assig.	%	Assig.	%
96.3	at6r'	79.	pk6r	25.			67.1	tHCNC	90.				
126.1	at6r	87.					132.3	at6r'	64.	btfl	27.		
151.2	btfl	45.	pk6r	30.	wC10-N	17.	168.7	at6r	48.				
203.5	tHCNC	79.					274.2	pk6r	35.	t5r	23.		
264.5	wC10-N	79.					328.0	sC-N.ar	36.	rN2-C	16.		
303.2	sC-N.ar	20.					356.5	btfl	37.	at6r'	26.		
340.2	t5r'	21.	btfl	18.	at6r'	17.	369.7	rN2-C	35.	sC-N.ar	18.	rC10-N	16.
360.9	rN2-C	44.	rC10-N	21.			375.3	wC10-N	45.	tHNCN	36.		
373.5	rC=O	26.	tHNCN	24.	rC10-N	16.	427.5	tHNCN	46.	wC10-N	17.		
410.2	tHNCN	52.	rC10-N	25.			436.4	rC10-N	46.	rC=O	31.	sC-N.ar	17.
448.9	ad6r'	61.					451.4	sC-N.ar	36.	ad6r'	22.		
489.5	ad6r	47.					491.0	wN-H	63.	t5r'	31.		
496.0	wN-H	72.					513.5	ad6r	29.	ad6r'	25.		
587.4	t5r	42.	t5r'	28.			553.6	wNH2	58.				
605.6	sC-N.ar	36.	td6r	20.	sC-C.ar	16.	563.1	sC-N.ar	33.	ad6r	19.	td6r	16.
624.8	wN2-C	41.	pk6r	22.			660.0	t5r'	34.	t5r	28.	wN-H	21.
642.0	wC=O	21.	t5r'	18.	t5r	18.	680.4	sC-N.ar	35.	rN2-C	16.		
642.5	t5r	21.	t5r'	16.			718.2	t5r	43.	pk6r	24.	at6r	16.
660.9	wC=O	72.					739.1	sC-N.ar	32.	d5r	16.		
736.6	wNH2	61.	wN2-C	18.			741.0	wN2-C	47.	wNH2	27.	pk6r	17.
761.3	wC-H	105.					844.9	d5r	33.	d5r'	25.	sC-N.ar	16.
773.8	td6r	23.	d5r'	16.			879.2	wC=O	101.				
903.8	d5r	44.	d5r'	28.			898.0	sC-N.ar	37.				
940.4	sC-N.ar	27.					904.2	wC-H	101.				
993.7	sC-N.ar	55.					998.3	rNH2	60.	sC-N.ar	23.		
1080.0	sC-N.ar	49.	rN-H	35.			1009.0	sC-N.ar	45.	rCH3'	18.		
1129.2	rNH2	47.	sC-N.ar	26.			1019.8	rCH3	56.	rCH3'	19.		
1142.5	rCH3	49.	rCH3'	43.			1044.5	sC-N.ar	52.	rN-H	19.		
1149.9	sC-N.ar	38.	rC-H	17.			1114.5	sC-N.ar	26.	rCH3'	15.		
1216.8	sC-N	24.					1151.2	sC-N.ar	31.	rCH3'	20.		
1233.8	sC-N.ar	28.	rCH3'	22.	sC-N	18.	1200.4	sC-N.ar	29.	rC-H	27.		
1286.2	sC-N.ar	51.	rC-H	33.			1257.9	sC-N.ar	31.	rN-H	17.		
1358.0	sC-N.ar	56.	sC-C.ar	16.			1371.6	sC-N.ar	32.				
1382.2	sC-N.ar	52.	rN-H	27.			1414.0	adCH3'	39.				
1406.6	sdCH3	52.	sC-N.ar	17.			1414.5	adCH3	65.	adCH3'	22.		
1449.0	sC-N.ar	48.					1439.0	sC-N.ar	41.				
1465.6	sC-N.ar	38.	sdCH3	24.			1477.5	sC-N.ar	32.				
1510.3	adCH3'	82.					1544.6	sC-N.ar	24.	sdCH3	21.	sC-C.ar	20.
1518.4	adCH3	27.	sC-N.ar	25.	sC-C.ar	15.	1553.5	sdCH3	37.	sC-N.ar	29.		
1532.2	adCH3	61.					1579.8	sC-N.ar	30.	rC-H	26.	sC-C.ar	17.
1592.4	sC-N.ar	38.	sC-C.ar	22.			1623.2	sC-N.ar	29.	cNH2	21.		
1603.4	sC-N.ar	58.	sC-C.ar	15.			1648.9	cNH2	51.	sC-N.ar	17.		
1655.5	cNH2	70.	sC-N.ar	15.			1696.6	sC-N.ar	34.	ad6r	19.		
1777.1	sC=O	66.	sC-C.ar	19.			1840.2	sC=O	38.	sC-C.ar	19.		
3014.3	ssCH3	91.					2909.7	ssCH3	99.				
3104.2	asCH3	73.	asCH3'	24.			2952.7	sC-H	99.				
3155.6	asCH3'	66.	asCH3	27.			2957.1	asCH3'	90.				
3205.1	sC-H	99.					2959.2	asCH3	90.				
3463.8	ssNH	100.					3443.6	ssNH	99.				
3546.7	sN-H	100.					3501.6	sN-H	100.				
3575.6	asNH2	100.					3563.8	asNH2	100.				

2.2 1-methyl inosine (1MI)

Figure 58: The energy-minimized structure of 1MI.

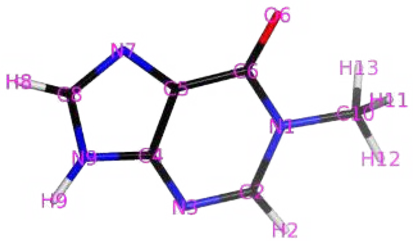

Table 171: The calculated geometric terms of 1MI.

Terms	QM	MM	diff
N9-C8	1.372	1.369	-0.003
N9-C4	1.372	1.346	-0.026
N9-H9	1.014	1.000	-0.013
C8-H8	1.083	1.093	0.010
C8-N7	1.326	1.321	-0.005
N7-C5	1.376	1.396	0.020
C5-C6	1.441	1.419	-0.022
C5-C4	1.394	1.399	0.005
C6-O6	1.228	1.237	0.008
C6-N1	1.434	1.414	-0.020
N1-C2	1.370	1.400	0.030
N1-C10	1.464	1.480	0.016
C2-H2	1.088	1.091	0.003
C2-N3	1.310	1.357	0.047
N3-C4	1.362	1.326	-0.036
C10-H11	1.091	1.116	0.025
C10-H12	1.090	1.114	0.024
C10-H13	1.091	1.116	0.025
C8-N9-C4	106.76	107.53	0.78
C8-N9-H9	127.76	130.04	2.28
C4-N9-H9	125.48	122.42	-3.06
N9-C8-H8	121.90	122.95	1.04
N9-C8-N7	113.12	112.53	-0.59
H8-C8-N7	124.97	124.52	-0.45
C8-N7-C5	103.76	104.41	0.65
N7-C5-C6	129.10	132.01	2.91
N7-C5-C4	111.48	109.40	-2.08
C6-C5-C4	119.42	118.60	-0.83
C5-C6-O6	130.06	128.20	-1.86
C5-C6-N1	109.84	113.24	3.41
O6-C6-N1	120.10	118.56	-1.54
C6-N1-C2	124.76	123.93	-0.83
C6-N1-C10	114.95	117.59	2.63
C2-N1-C10	120.28	118.48	-1.80
N1-C2-H2	115.49	117.14	1.65
N1-C2-N3	125.93	121.48	-4.45
H2-C2-N3	118.57	121.38	2.81
C2-N3-C4	111.43	114.95	3.52
N9-C4-C5	104.88	106.13	1.25
N9-C4-N3	126.50	126.06	-0.44
C5-C4-N3	128.62	127.80	-0.81
N1-C10-H11	109.58	110.93	1.36
N1-C10-H12	108.86	113.84	4.98
N1-C10-H13	109.58	110.93	1.36
H11-C10-H12	110.21	107.08	-3.13
H11-C10-H13	108.41	106.62	-1.79
H12-C10-H13	110.21	107.08	-3.13
C4-N9-C8-H8	-180.00	180.00	0.00
C4-N9-C8-N7	0.00	0.00	-0.00
H9-N9-C8-H8	-0.01	0.00	0.01
H9-N9-C8-N7	179.99	180.00	0.01
C8-N9-C4-C5	-0.00	0.00	0.00
C8-N9-C4-N3	180.00	180.00	0.00
H9-N9-C4-C5	180.01	-180.00	-0.01
H9-N9-C4-N3	0.01	0.00	-0.01
N9-C8-N7-C5	-0.00	-0.00	0.00
H8-C8-N7-C5	180.00	-180.00	-0.00
C8-N7-C5-C6	180.00	180.00	0.00
C8-N7-C5-C4	-0.00	0.00	0.00
N7-C5-C6-O6	0.00	0.00	-0.00
N7-C5-C6-N1	-180.00	-180.00	0.00
C4-C5-C6-O6	180.00	180.00	0.00
C4-C5-C6-N1	-0.00	0.00	0.00
N7-C5-C4-N9	0.00	0.00	-0.00
N7-C5-C4-N3	180.00	-180.00	0.00
C6-C5-C4-N9	180.00	180.00	-0.00
C6-C5-C4-N3	0.00	0.00	-0.00
C5-C6-N1-C2	0.00	0.00	-0.00
C5-C6-N1-C10	180.00	180.00	-0.00
O6-C6-N1-C2	180.00	-180.00	-0.00
O6-C6-N1-C10	-0.00	0.00	0.00
C6-N1-C2-H2	-180.00	180.00	-0.00
C6-N1-C2-N3	-0.00	0.00	0.00
C10-N1-C2-H2	0.00	0.00	-0.00
C10-N1-C2-N3	-180.00	-180.00	-0.00
N1-C2-N3-C4	-0.00	0.00	0.00
H2-C2-N3-C4	180.00	-180.00	0.00
C2-N3-C4-N9	180.00	-180.00	0.00
C2-N3-C4-C5	0.00	0.00	-0.00

Table 172: The statistics of vibrational frequencies of 1MI. Only the terms with the occupancies greater than 15% were shown. Refer to p.6 for the meanings of assig.

QM (MP2, scaled by 0.943)							MM						
Freq	Assig.	%	Assig.	%	Assig.	%	Freq	Assig.	%	Assig.	%	Assig.	%
102.1	at6r'	57.	pk6r	34.			52.4	tN1CH3	98.				
117.5	tN1CH3	76.	wN1C	17.			137.1	at6r'	66.	btf	26.		
154.7	at6r	34.	pk6r	19.	btf	16.	237.6	at6r	73.	t5r	15.		
245.2	btf	59.	at6r	18.			317.8	btf	43.	pk6r	27.	at6r'	15.
279.9	wN1C	74.	at6r'	16.			327.1	rN1C	46.	sN-C.ar	22.	sC-C.ar	16.
306.7	rN1C	22.	sN-C.ar	16.			371.1	pk6r	31.	at6r'	21.		
334.4	rN1C	47.	rC=O	33.			394.6	rC=O	46.	rN1C	22.		
447.4	ad6r'	64.					454.0	wN1C	49.	pk6r	42.		
490.5	wN-H	50.	t5r'	31.	at6r	20.	461.9	ad6r'	39.	sN-C.ar	35.		
525.2	ad6r	47.					490.0	wN-H	59.	t5r'	35.		
546.4	wN-H	40.	at6r	25.	pk6r	18.	544.9	ad6r	46.	sN-C.ar	30.		
611.0	pk6r	41.	t5r	35.			592.6	sN-C.ar	43.				
613.1	sN-C.ar	30.	rN1C	17.			642.1	sN-C.ar	50.	ad6r'	16.		
644.7	wC=O	34.	t5r	32.	t5r'	28.	651.9	t5r'	39.	wN-H	26.	t5r	16.
656.3	wC=O	63.	t5r'	21.			700.6	t5r	54.	at6r	21.		
681.8	sN-C.ar	58.	d5r	16.			779.3	td6r	26.	sN-C.ar	20.		
776.6	wC-H	105.					834.4	wC2H	56.	wC=O	33.		
795.3	td6r	39.	sN1-C10	17.			853.8	d5r	46.	sN-C.ar	19.	d5r'	15.
872.8	wC2H	109.					900.9	wC-H	44.	wC=O	35.	wC2H	19.
908.0	d5r	45.	d5r'	33.			905.8	wC-H	62.	wC=O	34.		
974.7	sN-C.ar	43.	rC=O	16.			985.4	sN-C.ar	61.				
1058.5	rCH3'	35.	sN-C.ar	32.			1010.4	rCH3	54.	rCH3'	18.		
1085.3	sN-C.ar	48.	rN-H	36.			1019.4	rCH3'	46.	sN-C.ar	18.	rCH3	15.
1141.4	rCH3	68.	rCH3'	22.			1044.7	sN-C.ar	50.	rN-H	15.		
1142.1	sN-C.ar	37.					1109.1	sN-C.ar	48.	rN-H	21.		
1220.1	sN1-C10	32.	sN-C.ar	24.	td6r	15.	1125.7	sN-C.ar	43.				
1274.1	sN-C.ar	52.	rC-H	26.			1223.6	rC-H	34.	sN-C.ar	24.		
1331.9	sN-C.ar	55.					1268.9	sN-C.ar	56.	rN-H	18.		
1363.5	sN-C.ar	45.	rC2H	22.			1316.0	rC2H	31.	sN-C.ar	27.		
1378.9	sN-C.ar	60.	sC-C.ar	21.			1366.3	sN-C.ar	28.	sdCH3	19.		
1403.3	rC2H	41.	sN-C.ar	29.	rN-H	20.	1405.0	adCH3'	47.	adCH3	16.		
1452.9	sN-C.ar	60.	rC-H	19.			1405.7	adCH3	65.	adCH3'	20.		
1456.4	sdCH3	90.					1461.0	sN-C.ar	38.	sdCH3	18.		
1482.8	adCH3	70.	adCH3'	24.			1518.5	sdCH3	51.	sN-C.ar	18.	sN1-C10	16.
1502.0	sN-C.ar	35.	sC-C.ar	34.			1550.8	sN-C.ar	33.	sC-C.ar	28.		
1519.6	adCH3'	62.	adCH3	21.			1580.2	sN-C.ar	32.	rC-H	30.		
1572.5	sN-C.ar	48.	sC-C.ar	27.			1634.6	sN-C.ar	35.	sC-C.ar	18.		
1598.8	sN-C.ar	67.					1687.8	sN-C.ar	37.	rC2H	17.		
1771.2	sC=O	69.	sC-C.ar	19.			1838.8	sC=O	39.	sC-C.ar	18.		
3036.1	ssCH3	100.					2910.8	ssCH3	100.				
3129.8	asCH3	75.	asCH3'	25.			2952.8	sC-H	99.				
3136.3	sC-H	74.	asCH3'	19.			2958.6	asCH3	75.	asCH3'	25.		
3144.6	asCH3'	56.	sC-H	25.	asCH3	19.	2961.0	asCH3'	75.	asCH3	25.		
3205.7	sC-H	99.					3118.3	sC-H	99.				
3543.5	sN-H	100.					3501.5	sN-H	100.				

2.3 1H-pseudoisocytosine (1PC)

Figure 59: The energy-minimized structure of 1PC.

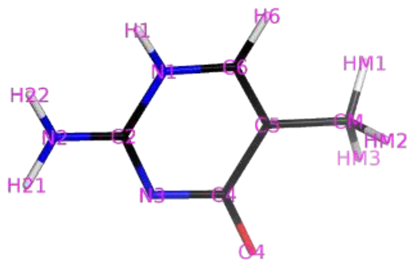

Table 173: The calculated geometric terms of 1PC.

Terms	QM	MM	diff
N1-H1	1.013	1.001	-0.012
N1-C2	1.371	1.375	0.003
N1-C6	1.384	1.358	-0.026
C2-N2	1.391	1.316	-0.075
C2-N3	1.300	1.315	0.014
N2-H21	1.016	0.996	-0.020
N2-H22	1.016	0.990	-0.026
N3-C4	1.408	1.361	-0.047
C4-O4	1.234	1.231	-0.002
C4-C5	1.475	1.464	-0.011
C5-C6	1.354	1.366	0.012
C5-CM	1.495	1.509	0.013
C6-H6	1.086	1.094	0.007
H1-N1-C2	119.19	116.50	-2.69
H1-N1-C6	120.02	124.25	4.23
C2-N1-C6	119.17	119.25	0.08
N1-C2-N2	114.85	117.03	2.18
N1-C2-N3	124.66	122.66	-2.00
N2-C2-N3	120.45	120.31	-0.14
C2-N2-H21	114.67	115.19	0.53
C2-N2-H22	109.70	124.36	14.67
H21-N2-H22	111.47	120.44	8.97
C2-N3-C4	119.10	120.29	1.19
N3-C4-O4	121.00	119.99	-1.01
N3-C4-C5	117.79	119.06	1.27
O4-C4-C5	121.20	120.94	-0.26
C4-C5-C6	118.95	116.91	-2.04
C4-C5-CM	117.72	121.60	3.88
C6-C5-CM	123.31	120.73	-2.58
N1-C6-C5	120.20	121.37	1.17
N1-C6-H6	115.88	118.34	2.47
C5-C6-H6	123.91	120.29	-3.62
H1-N1-C2-N2	10.85	0.94	-9.92
H1-N1-C2-N3	-166.65	-178.80	-12.15
C6-N1-C2-N2	176.34	-178.97	4.69
C6-N1-C2-N3	-1.17	1.29	2.46
H1-N1-C6-C5	166.49	-178.30	15.21
H1-N1-C6-H6	-14.86	1.71	16.57
C2-N1-C6-C5	1.13	1.60	0.47
C2-N1-C6-H6	179.78	-178.39	1.83
N1-C2-N2-H21	47.88	-179.76	132.36
N1-C2-N2-H22	174.19	0.62	-173.57
N3-C2-N2-H21	-134.50	-0.01	134.49
N3-C2-N2-H22	-8.19	-179.64	-171.45
N1-C2-N3-C4	2.68	0.97	-1.71
N2-C2-N3-C4	-174.69	-178.76	-4.07
C2-N3-C4-O4	177.21	175.07	-2.13
C2-N3-C4-C5	-3.96	-5.71	-1.75
N3-C4-C5-C6	3.97	8.19	4.22
N3-C4-C5-CM	-177.50	178.26	4.25
O4-C4-C5-C6	-177.20	-172.60	4.60
O4-C4-C5-CM	1.34	-2.53	-3.87
C4-C5-C6-N1	-2.57	-6.13	-3.55
C4-C5-C6-H6	178.88	173.86	-5.02
CM-C5-C6-N1	178.98	-176.29	4.74
CM-C5-C6-H6	0.44	3.70	3.27

Table 174: The statistics of vibrational frequencies of 1PC. Only the terms with the occupancies greater than 15% were shown. Refer to p.6 for the meanings of assig.

QM (MP2, scaled by 0.943)							MM						
Freq	Assig.	%	Assig.	%	Assig.	%	Freq	Assig.	%	Assig.	%	Assig.	%
84.5	at6r	89.					129.3	at6r	56.	at6r'	44.	wC-C	-20.
155.6	tC5CM	81.					155.5	at6r'	23.	pk6r	21.	wC-N	17.
178.5	at6r'	31.	pk6r	28.			163.7	tC5CM	92.				
276.5	rC-C	70.					283.1	tC2N2	41.	wC-C	19.	wN-H	15.
296.0	wC-C	73.					285.9	rC-C	69.				
326.6	tC2N2	67.					318.1	pk6r	36.	tC2N2	26.	wC-C	19.
347.2	rC-N	35.	tC2N2	26.			368.1	rC-N	41.	sN-C.r	21.	rC=O	20.
368.4	at6r'	48.	pk6r	20.	wN-H	16.	428.9	wC-C	56.	at6r'	21.		
458.2	ad6r	26.	ad6r'	24.	wN-H	23.	463.8	wN-H	59.	tC2N2	26.		
474.1	wN-H	48.					480.3	ad6r'	31.	sN-C.r	20.	sC-N	17.
535.6	ad6r	29.	ad6r'	26.	rC=O	15.	540.5	wN-H	25.	wHH2	19.	wC-N	16.
567.8	rC=O	28.	rC-N	21.			574.8	rC=O	20.	rC-N	19.	sC-C.r	17.
624.0	wC-N	53.	pk6r	17.			603.2	ad6r	44.				
692.6	wC=O	68.	pk6r	17.			660.5	wHH2	50.	wC-N	20.	wC=O	19.
733.5	sC-C.r	39.	sN-C.r	27.			713.8	td6r	20.	sC-N	17.	sC-C	16.
758.9	wHH2	18.	td6r	16.			738.9	wC=O	51.	pk6r	18.		
791.5	wHH2	37.	td6r	24.			763.3	sC-C.r	25.	ad6r'	22.	sN-C.r	20.
820.8	wC-H	93.					814.0	wC-H	98.				
963.6	sN-C.r	39.	rCH3	20.			907.7	sN-C.r	41.	rNH2	19.		
1013.9	rCH3	38.	sN-C.r	19.	td6r	18.	991.7	rCH3	36.	rCH3'	32.		
1053.0	rCH3'	89.					1009.2	sN-C.r	39.	rNH2	34.		
1098.8	sN-C.r	30.	rNH2	24.	rCH3	17.	1014.6	rCH3'	48.	rCH3	34.		
1172.3	rNH2	39.	sN-C.r	15.			1036.1	sN-C.r	43.	rNH2	24.		
1210.5	sN-C.r	51.	sC-C	20.			1196.4	sN-C.r	24.	sC-C	15.		
1277.0	rC-H	32.	sN-C.r	19.			1296.1	rC-H	33.	sC-C.r	17.		
1348.6	rN-H	33.	sN-C.r	18.	sC-N	17.	1348.7	rN-H	20.				
1387.2	rC-H	32.	sC-C.r	29.			1408.7	adCH3	73.				
1426.4	sdCH3	94.					1413.1	adCH3'	70.	sdCH3	20.		
1482.5	adCH3'	91.					1435.2	sdCH3	61.	adCH3'	17.	adCH3	16.
1504.2	adCH3	87.					1518.7	sN-C.r	29.	sC-C.r	29.		
1541.9	sN-C.r	52.	rN-H	22.			1554.8	sN-C.r	37.	rN-H	19.		
1607.7	sN-C.r	32.	rNH2	31.			1601.8	sN-C.r	30.	rC-H	20.		
1649.8	rNH2	35.	sC-C.r	21.			1638.7	rNH2	66.				
1693.1	sN-C.r	32.	sC-C.r	30.			1689.2	sN-C.r	19.	ad6r'	16.		
1707.0	sC=O	72.					1810.2	sC=O	45.	ad6r	18.		
3010.9	ssCH3	99.					2904.4	ssCH3	100.				
3097.5	asCH3'	95.					2957.8	asCH3'	89.				
3102.2	asCH3	94.					2959.3	asCH3	89.				
3150.7	sC-H	99.					2996.2	sC-H	99.				
3454.1	ssNH2	100.					3438.7	ssNH2	78.	sN-H	20.		
3532.4	sN-H	100.					3454.1	sN-H	79.	ssNH2	21.		
3564.4	asNH2	100.					3560.1	asNH2	99.				

2.4 2-methyladenine (2MA)

Figure 60: The energy-minimized structure of 2MA.

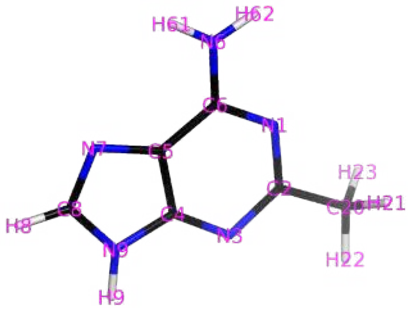

Table 175: The calculated geometric terms of 2MA.

Terms	QM	MM	diff
N9-C8	1.372	1.370	-0.002
N9-C4	1.378	1.348	-0.030
N9-H9	1.013	0.999	-0.014
C8-H8	1.083	1.093	0.010
C8-N7	1.326	1.324	-0.002
N7-C5	1.381	1.409	0.027
C5-C6	1.409	1.410	0.002
C5-C4	1.397	1.392	-0.005
C6-N6	1.366	1.347	-0.019
C6-N1	1.338	1.363	0.025
N6-H61	1.012	0.996	-0.017
N6-H62	1.012	0.995	-0.017
N1-C2	1.359	1.356	-0.004
C2-N3	1.342	1.353	0.011
C2-C20	1.501	1.498	-0.003
N3-C4	1.343	1.326	-0.016
C20-H21	1.094	1.110	0.016
C20-H22	1.094	1.110	0.017
C20-H23	1.091	1.110	0.019
C8-N9-C4	106.90	107.65	0.75
C8-N9-H9	127.44	130.86	3.42
C4-N9-H9	125.66	121.49	-4.17
N9-C8-H8	121.73	122.49	0.76
N9-C8-N7	113.52	113.03	-0.50
H8-C8-N7	124.75	124.48	-0.26
C8-N7-C5	103.21	103.24	0.03
N7-C5-C6	132.23	134.32	2.08
N7-C5-C4	112.08	110.26	-1.82
C6-C5-C4	115.66	115.42	-0.24
C5-C6-N6	121.74	124.28	2.54
C5-C6-N1	119.05	118.64	-0.41
N6-C6-N1	119.13	117.09	-2.04
C6-N6-H61	115.46	121.02	5.55
C6-N6-H62	116.56	117.38	0.82
H61-N6-H62	116.70	121.60	4.90
C6-N1-C2	119.16	119.64	0.48
N1-C2-N3	127.33	125.59	-1.74
N1-C2-C20	115.36	116.67	1.31
N3-C2-C20	117.32	117.74	0.43
C2-N3-C4	111.58	112.97	1.39
N9-C4-C5	104.28	105.82	1.54
N9-C4-N3	128.49	126.43	-2.06
C5-C4-N3	127.22	127.75	0.53
C2-C20-H21	110.22	109.90	-0.32
C2-C20-H22	110.23	111.56	1.33
C2-C20-H23	109.91	109.90	-0.01
H21-C20-H22	107.31	108.42	1.11
H21-C20-H23	109.54	108.56	-0.98
H22-C20-H23	109.60	108.42	-1.17
C4-N9-C8-H8	-179.87	-180.00	-0.13
C4-N9-C8-N7	0.12	0.00	-0.12
H9-N9-C8-H8	0.04	-0.00	-0.04
H9-N9-C8-N7	-179.97	180.00	0.03
C8-N9-C4-C5	-0.36	0.00	0.36
C8-N9-C4-N3	179.36	180.00	0.64
H9-N9-C4-C5	179.72	-180.00	0.28
H9-N9-C4-N3	-0.56	0.00	0.56
N9-C8-N7-C5	0.19	0.00	-0.19
H8-C8-N7-C5	-179.83	180.00	0.17
C8-N7-C5-C6	-178.53	-180.00	-1.47
C8-N7-C5-C4	-0.43	0.00	0.43
N7-C5-C6-N6	-4.92	0.00	4.92
N7-C5-C6-N1	178.36	180.00	1.64
C4-C5-C6-N6	177.03	-180.00	2.97
C4-C5-C6-N1	0.31	0.00	-0.31
N7-C5-C4-N9	0.50	0.00	-0.50
N7-C5-C4-N3	-179.22	-180.00	-0.78
C6-C5-C4-N9	178.95	180.00	1.05
C6-C5-C4-N3	-0.78	0.00	0.78
C5-C6-N6-H61	164.18	0.00	-164.18
C5-C6-N6-H62	21.57	180.00	158.43
N1-C6-N6-H61	-19.09	-180.00	-160.91
N1-C6-N6-H62	-161.70	0.00	161.70
C5-C6-N1-C2	0.17	0.00	-0.17
N6-C6-N1-C2	-176.64	-180.00	-3.36
C6-N1-C2-N3	-0.31	-0.00	0.31
C6-N1-C2-C20	179.78	-180.00	0.22
N1-C2-N3-C4	-0.08	0.00	0.08
C20-C2-N3-C4	179.83	180.00	0.17
N1-C2-C20-H21	-60.00	59.71	119.70
N1-C2-C20-H22	58.27	-180.00	121.72
N1-C2-C20-H23	179.18	-59.71	121.11
N3-C2-C20-H21	120.09	-120.29	119.62
N3-C2-C20-H22	-121.64	0.00	121.64
N3-C2-C20-H23	-0.74	120.29	121.03
C2-N3-C4-N9	-179.01	180.00	0.99
C2-N3-C4-C5	0.65	-0.00	-0.65

Table 176: The statistics of vibrational frequencies of 2MA. Only the terms with the occupancies greater than 15% were shown. Refer to p.6 for the meanings of assig.

QM (MP2, scaled by 0.943)							MM						
Freq	Assig.	%	Assig.	%	Assig.	%	Freq	Assig.	%	Assig.	%	Assig.	%
61.2	tC2C	94.					48.3	tC2C	99.				
129.8	at6r	81.					149.9	at6r	50.	wC4N4	22.	at6r’	15.
168.9	btf	47.	pk6r	23.			156.5	wC4N4	52.	btf	25.	wC2C20	21.
225.9	at6r’	62.	btf	16.			233.6	at6r’	39.	at6r	20.	wC2C20	17.
262.3	rC4N4	55.					289.9	rC2C20	43.	rC4N4	32.	sN-C.ar	19.
296.3	rC2C20	67.					313.3	rC4N4	31.	sN-C.ar	20.	rC2C20	15.
336.7	t5r’	24.	at6r’	22.	wC2C20	17.	331.0	wC2C20	86.				
456.4	wNH2	55.	tC4N	35.			370.8	tC4N	70.				
471.4	tC4N	49.	wNH2	22.			393.1	at6r’	26.	btf	22.		
490.5	ad6r	32.	ad6r’	25.			431.7	pk6r	67.	wC2C20	-17.		
500.6	wN-H	69.					465.6	sN-C.ar	35.	ad6r	24.	ad6r’	15.
518.6	ad6r’	33.	ad6r	22.	rC4N4	18.	485.1	wN-H	64.	t5r’	30.		
587.5	wC4N4	36.	wC2C20	19.	t5r’	16.	529.4	sC6-N6	28.	sN-C.ar	22.	td6r	18.
598.3	td6r	23.	sN-C.ar	17.			563.3	ad6r’	28.	sN-C.ar	26.	ad6r	23.
609.8	wC4N4	37.	t5r	18.			602.0	wNH2	79.				
628.4	d5r’	23.	sC-C.ar	23.	sN-C.ar	15.	637.7	sN-C.ar	21.	d5r’	16.		
645.9	t5r	52.	t5r’	40.			654.5	t5r	55.	t5r’	22.		
671.2	pk6r	64.					704.7	pk6r	29.	t5r	26.	t5r’	18.
788.0	wC-H	105.					751.6	sN-C.ar	34.	d5r	18.	sC2-C20	17.
806.5	sN-C.ar	28.	td6r	21.	d5r	20.	852.8	d5r	39.	sN-C.ar	23.	sC-C.ar	17.
914.4	d5r	43.	d5r’	32.			904.9	wC-H	108.				
957.5	sN-C.ar	44.					929.0	sN-C.ar	58.				
1014.4	rCH3’	42.	sN-C.ar	19.			990.6	rNH2	52.	sN-C.ar	23.		
1057.6	rCH3	63.	rCH3’	19.			992.0	rCH3	66.	rCH3’	22.		
1088.8	sN-C.ar	53.	rN-H	36.			995.9	rCH3’	33.	sN-C.ar	23.		
1113.5	rNH2	28.	sN-C.ar	21.			1031.9	sN-C.ar	44.	rN-H	18.		
1142.3	sN-C.ar	37.					1050.4	sN-C.ar	28.	rNH2	15.		
1241.5	sN-C.ar	36.	rC-H	31.			1171.7	sN-C.ar	38.	rC-H	33.		
1292.1	sN-C.ar	65.					1191.5	sN-C.ar	55.	rN-H	18.		
1367.4	sN-C.ar	51.	rN-H	30.			1321.1	sN-C.ar	42.	rN-H	21.		
1377.6	sN-C.ar	59.					1362.7	sdCH3	32.	sN-C.ar	23.	td6r	18.
1392.0	sN-C.ar	52.	sdCH3	26.			1407.1	adCH3’	47.	adCH3	16.		
1421.8	sdCH3	40.	sN-C.ar	28.			1417.4	adCH3	67.	adCH3’	22.		
1446.7	sN-C.ar	39.					1419.1	sN-C.ar	28.	sC6-N6	18.		
1476.0	sN-C.ar	45.	adCH3’	17.			1443.2	sdCH3	44.	sN-C.ar	17.		
1481.6	sN-C.ar	41.	rC-H	15.			1463.8	sN-C.ar	44.				
1490.7	adCH3	72.	adCH3’	24.			1545.7	sN-C.ar	68.	sC-C.ar	17.		
1510.0	adCH3’	47.	sN-C.ar	17.			1582.4	sN-C.ar	45.	rC-H	39.		
1595.4	cNH2	53.	sC-C.ar	25.			1624.3	cNH2	73.				
1627.7	sN-C.ar	55.	sC-C.ar	19.			1672.4	sN-C.ar	40.	sC-C.ar	22.	ad6r	16.
1657.5	sC-C.ar	32.	cNH2	30.	sC6-N6	19.	1711.5	sC-C.ar	36.	ad6r’	17.		
3017.7	ssCH3	99.					2904.0	ssCH3	100.				
3095.4	asCH3	76.	asCH3’	24.			2953.2	sC-H	99.				
3129.2	asCH3’	75.	asCH3	24.			2959.8	asCH3	75.	asCH3’	25.		
3201.1	sC-H	99.					2961.8	asCH3’	75.	asCH3	25.		
3489.3	ssNH2	100.					3444.6	ssNH2	99.				
3548.7	sN-H	100.					3503.2	sN-H	100.				
3616.3	asNH2	100.					3563.8	asNH2	100.				

2.5 2-thiouracil (2SU)

Figure 61: The energy-minimized structure of 2SU.

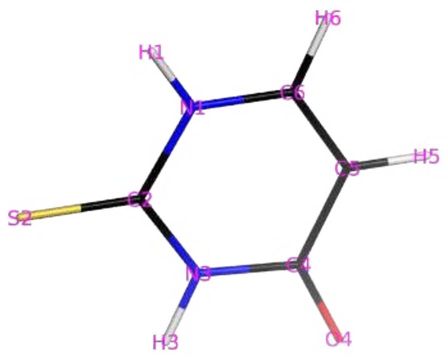

Table 177: The calculated geometric terms of 2SU.

Terms	QM	MM	diff
N1-C2	1.377	1.394	0.017
N1-C6	1.375	1.373	-0.002
N1-H1	1.015	1.010	-0.005
C2-S2	1.650	1.644	-0.007
C2-N3	1.372	1.385	0.012
N3-H3	1.018	1.003	-0.015
N3-C4	1.413	1.382	-0.031
C4-O4	1.227	1.228	0.001
C4-C5	1.454	1.428	-0.026
C5-H5	1.082	1.086	0.004
C5-C6	1.353	1.371	0.018
C6-H6	1.085	1.094	0.008
C2-N1-C6	124.24	122.76	-1.48
C2-N1-H1	115.08	114.81	-0.27
C6-N1-H1	120.68	122.43	1.75
N1-C2-S2	123.03	123.17	0.14
N1-C2-N3	112.54	113.43	0.89
S2-C2-N3	124.43	123.40	-1.03
C2-N3-H3	115.88	118.27	2.39
C2-N3-C4	128.95	127.43	-1.52
H3-N3-C4	115.17	114.30	-0.87
N3-C4-O4	120.22	118.80	-1.42
N3-C4-C5	113.07	115.71	2.64
O4-C4-C5	126.71	125.49	-1.22
C4-C5-H5	118.61	119.77	1.17
C4-C5-C6	119.65	118.90	-0.75
H5-C5-C6	121.74	121.32	-0.42
N1-C6-C5	121.55	121.76	0.21
N1-C6-H6	115.34	117.80	2.47
C5-C6-H6	123.11	120.43	-2.68
C6-N1-C2-S2	-180.00	-180.00	0.00
C6-N1-C2-N3	0.00	0.00	-0.00
H1-N1-C2-S2	0.00	0.00	0.00
H1-N1-C2-N3	180.00	180.00	0.00
C2-N1-C6-C5	0.00	0.00	0.00
C2-N1-C6-H6	180.00	-180.00	0.00
H1-N1-C6-C5	180.00	-180.00	0.00
H1-N1-C6-H6	0.00	0.00	0.00
N1-C2-N3-H3	180.00	-180.00	0.00
N1-C2-N3-C4	0.00	0.00	0.00
S2-C2-N3-H3	0.00	0.00	-0.00
S2-C2-N3-C4	180.00	180.00	-0.00
C2-N3-C4-O4	180.00	180.00	-0.00
C2-N3-C4-C5	-0.00	0.00	0.00
H3-N3-C4-O4	0.00	0.00	-0.00
H3-N3-C4-C5	180.00	180.00	0.00
N3-C4-C5-H5	180.00	-180.00	-0.00
N3-C4-C5-C6	0.00	0.00	-0.00
O4-C4-C5-H5	-0.00	0.00	0.00
O4-C4-C5-C6	-180.00	-180.00	0.00
C4-C5-C6-N1	-0.00	0.00	0.00
C4-C5-C6-H6	180.00	180.00	0.00
H5-C5-C6-N1	180.00	180.00	0.00
H5-C5-C6-H6	-0.00	0.00	0.00

Table 178: The statistics of vibrational frequencies of 2SU. Only the terms with the occupancies greater than 15% were shown. Refer to p.6 for the meanings of assig.

QM (MP2, scaled by 0.943)							MM						
Freq	Assig.	%	Assig.	%	Assig.	%	Freq	Assig.	%	Assig.	%	Assig.	%
118.3	pk6r	55.	at6r’	48.			171.1	at6r’	44.	wC=S	31.	pk6r	19.
146.9	at6r	100.					184.4	at6r	72.	pk6r	19.		
259.1	rC=S	69.					260.2	rC=S	66.	sN-C	23.		
360.0	at6r’	60.	pk6r	23.			376.9	at6r’	58.	pk6r	33.	at6r	23.
437.0	ad6r’	39.	sC=S	26.	sN-C	18.	408.4	sC=S	30.	sN-C	26.	ad6r’	21.
464.8	rC=O	62.	rC=S	16.			553.4	rC=O	44.	sC-C	15.		
505.9	ad6r	73.					574.4	wN1-H1	71.				
569.1	wN1-H1	69.	wC=S	46.			587.0	ad6r	56.	sN-C	16.		
586.2	wC=S	49.	wN1-H1	17.			590.9	wC=S	46.	wC=O	19.		
655.1	wC=O	48.	pk6r	43.			642.5	wC-H	49.	wN1-H1	25.		
687.6	ad6r’	28.	sC-C	22.	sN-C	16.	716.4	wN3-H3	108.				
702.6	wN3-H3	100.					738.0	ad6r’	32.	sC-C	28.	sN-C	25.
744.4	wC-H	85.	wC=O	22.			779.0	wC-H	50.	wC=O	27.	pk6r	26.
868.1	wC-H	113.					824.4	sN-C	53.				
885.0	sN-C	37.	sC-C	28.			909.5	sN-C	54.	td6r	26.		
954.1	td6r	53.	sN-C	36.			1001.1	td6r	32.	sN-C	20.	rC-H	17.
1043.3	sN-C	38.	rC-H	31.	sC=C	17.	1023.2	wC-H	94.	wC=O	29.		
1141.6	sC=S	36.	sN-C	21.	rN-H	18.	1151.6	rC-H	48.	sC=C	25.		
1175.6	rC-H	37.	sN-C	37.	rN-H	22.	1235.9	rN-H	37.	sC=S	33.		
1204.2	sN-C	35.	rC-H	34.			1318.0	sN-C	42.	rN-H	37.		
1337.3	rN-H	68.					1385.0	rC-H	52.	rC=O	16.		
1355.5	sN-C	44.	rC-H	41.			1466.9	rN-H	48.	sN-C	30.		
1402.4	sN-C	35.	rC-H	17.	rN-H	16.	1489.0	sN-C	21.	sC-C	18.	rN-H	16.
1518.7	rN-H	50.	sN-C	34.			1593.9	rC-H	30.	sC=C	25.	rN-H	20.
1602.4	sC=C	59.					1614.6	rC-H	23.	sN-C	22.		
1718.9	sC=O	70.					1803.0	sC=O	47.				
3080.3	sC-H	100.					2993.5	sC-H	99.				
3116.7	sC-H	99.					2996.3	sC-H	98.				
3389.5	sN-H	100.					3453.2	sN-H	99.				
3428.0	sN-H	100.					3460.0	sN-H	99.				

2.6 3-methylcytosine (3MCn)

Figure 62: The energy-minimized structure of 3MCn.

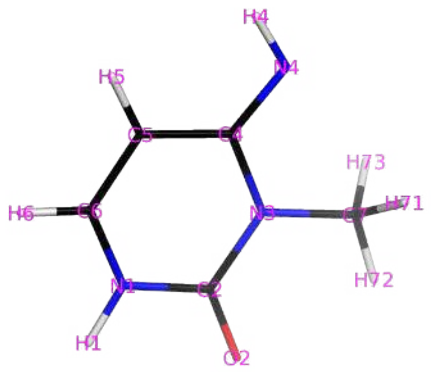

Table 179: The calculated geometric terms of 3MCn.

Terms	QM	MM	diff
N1-C2	1.391	1.386	-0.004
N1-C6	1.375	1.371	-0.004
N1-H1	1.012	1.007	-0.006
C2-O2	1.229	1.228	-0.001
C2-N3	1.386	1.399	0.013
N3-C4	1.410	1.410	-0.000
N3-C7	1.463	1.480	0.018
C4-N4	1.294	1.299	0.005
C4-C5	1.455	1.442	-0.013
N4-H4	1.024	0.998	-0.026
C5-H5	1.084	1.089	0.005
C5-C6	1.348	1.371	0.023
C6-H6	1.085	1.092	0.007
C7-H71	1.091	1.115	0.024
C7-H72	1.087	1.112	0.025
C7-H73	1.091	1.115	0.024
C2-N1-C6	124.22	121.84	-2.38
C2-N1-H1	114.15	113.22	-0.93
C6-N1-H1	121.63	124.94	3.31
N1-C2-O2	120.91	119.85	-1.06
N1-C2-N3	114.30	117.00	2.69
O2-C2-N3	124.79	123.16	-1.63
C2-N3-C4	125.48	122.98	-2.50
C2-N3-C7	117.64	117.93	0.29
C4-N3-C7	116.89	119.09	2.21
N3-C4-N4	116.88	117.28	0.39
N3-C4-C5	115.28	117.28	1.99
N4-C4-C5	127.83	125.45	-2.38
C4-N4-H4	108.82	109.15	0.33
C4-C5-H5	119.13	121.26	2.13
C4-C5-C6	120.15	118.64	-1.51
H5-C5-C6	120.72	120.10	-0.62
N1-C6-C5	120.56	122.26	1.70
N1-C6-H6	116.01	117.38	1.36
C5-C6-H6	123.42	120.36	-3.06
N3-C7-H71	109.39	110.94	1.55
N3-C7-H72	107.53	113.86	6.33
N3-C7-H73	109.39	110.94	1.55
H71-C7-H72	111.13	106.81	-4.32
H71-C7-H73	108.24	107.13	-1.11
H72-C7-H73	111.13	106.81	-4.32
C6-N1-C2-O2	180.00	-180.00	-0.00
C6-N1-C2-N3	0.01	0.00	-0.01
H1-N1-C2-O2	-0.01	0.00	0.01
H1-N1-C2-N3	180.00	-180.00	0.00
C2-N1-C6-C5	-0.01	0.00	0.01
C2-N1-C6-H6	179.99	180.00	0.01
H1-N1-C6-C5	-179.99	180.00	0.01
H1-N1-C6-H6	0.01	0.00	-0.01
N1-C2-N3-C4	-0.00	0.00	0.00
N1-C2-N3-C7	180.00	-180.00	0.00
O2-C2-N3-C4	180.00	180.00	-0.00
O2-C2-N3-C7	0.00	0.00	-0.00
C2-N3-C4-N4	180.00	180.00	0.00
C2-N3-C4-C5	-0.00	0.00	0.00
C7-N3-C4-N4	-0.00	0.00	0.00
C7-N3-C4-C5	180.00	180.00	0.00
N3-C4-N4-H4	-180.00	-180.00	0.00
C5-C4-N4-H4	0.00	0.00	-0.00
N3-C4-C5-H5	180.00	180.00	-0.00
N3-C4-C5-C6	0.00	0.00	-0.00
N4-C4-C5-H5	0.00	-0.00	-0.00
N4-C4-C5-C6	-180.00	-180.00	-0.00
C4-C5-C6-N1	0.00	0.00	-0.00
C4-C5-C6-H6	180.00	180.00	0.00
H5-C5-C6-N1	180.00	-180.00	-0.00
H5-C5-C6-H6	-0.00	0.00	0.00

Figure 63: The PES scan for flexible dihedral corresponding to 3MCn.

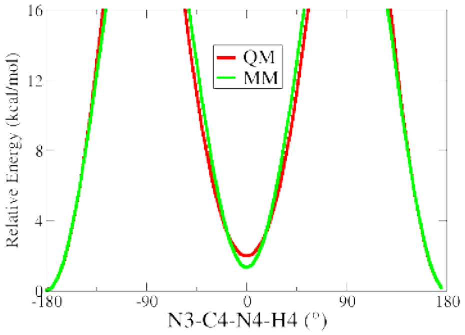

Table 180: The statistics of vibrational frequencies of 3MCn. Only the terms with the occupancies greater than 15% were shown. Refer to p.6 for the meanings of assig.

QM (MP2, scaled by 0.943)							MM						
Freq	Assig.	%	Assig.	%	Assig.	%	Freq	Assig.	%	Assig.	%	Assig.	%
83.0	pk6r	58.	at6r	38.	wN3-C7	-37.	80.0	tCNCH3	95.				
130.5	at6r	74.	at6r’	27.			157.9	at6r	65.	wN3-C7	19.		
152.4	tCNCH3	73.	wN3-C7	17.			285.1	at6r’	58.	pk6r	35.	at6r	24.
205.3	wN3-C7	94.					344.9	wN3-C7	49.	wC-N4	30.		
359.5	at6r’	43.	pk6r	30.	wN3-C7	18.	418.2	rN3-C7	54.	rC-N4	28.		
366.6	rN3-C7	70.					424.1	sN-C.r	29.	rN3-C7	22.	rC-N4	22.
391.7	rC=O	29.	rC-N4	28.			497.1	pk6r	45.	at6r’	35.	wN3-C7	19.
498.8	ad6r	34.	ad6r’	21.	sN-C.r	17.	528.0	ad6r’	44.	sN-C.r	16.		
511.0	wN-H	76.					549.3	ad6r	39.	sN-C.r	21.		
528.9	ad6r’	29.	ad6r	23.			552.5	wN-H	98.				
559.6	rC=O	25.	rC-N4	18.			633.9	ad6r	31.	rC=O	18.	sN-C.r	16.
609.0	wC-N4	74.	pk6r	19.			670.5	sN-C.r	45.	sC-C.r	23.		
685.1	wC=O	75.	wC5-H5	19.			709.1	wC5-H5	57.	wC6-H6	34.		
695.9	sN-C.r	56.	sN3-C7	15.			806.2	wC=O	34.	wC-N4	27.	tC4N4	20.
709.5	wC5-H5	51.	wC=O	28.			871.4	td6r	44.	sN-C.r	20.		
821.5	tC4N4	95.					875.3	wC=O	49.				
844.3	td6r	57.	sN3-C7	17.			929.1	wC6-H6	48.	tC4N4	20.	wC5-H5	15.
865.7	wC6-H6	91.	wC5-H5	19.			940.1	sN-C.r	32.	sC-C.r	16.		
980.5	sN-C.r	32.	rCH3’	19.	sC-C.r	16.	978.0	tC4N4	43.	wC5-H5	26.	wC6-H6	17.
1069.2	sN-C.r	27.	sC-C.r	24.			993.2	sN-C.r	31.				
1112.5	sN-C.r	24.	dCNH	16.	rC-H	16.	1023.4	rCH3	49.	rCH3’	16.		
1149.1	rCH3	69.	rCH3’	22.			1127.3	sC-C.r	23.	dCNH	16.	rC-H	15.
1161.7	rC-H	41.	sN3-C7	22.	sN-C.r	20.	1148.8	rCH3’	26.				
1203.4	dCNH	28.	sN3-C7	26.			1166.6	rC-H	36.	dCNH	20.	sC-C.r	19.
1243.5	sN-C.r	23.	rC-H	22.	rN-H	19.	1271.4	sN3-C7	30.	dCNH	20.		
1339.1	sN-C.r	48.					1389.4	rN-H	50.				
1391.4	rC-H	54.					1404.3	adCH3’	51.	adCH3	17.		
1424.2	sdCH3	57.					1415.4	adCH3	64.	adCH3’	19.		
1449.3	sdCH3	29.	rN-H	20.	sN-C.r	17.	1475.9	rC-H	33.				
1499.8	adCH3	70.	adCH3’	26.			1511.5	sdCH3	58.	sN-C.r	16.		
1500.8	sN-C.r	36.					1537.8	sN-C.r	42.	sC-C.r	17.		
1516.5	adCH3’	56.	adCH3	19.			1572.6	rC-H	42.	sC4-N4	19.	sC-C.r	16.
1613.7	sC4-N4	59.	sC-C.r	21.			1613.6	sN-C.r	25.	sN3-C7	18.		
1701.8	sC-C.r	54.					1734.1	sC-C.r	26.	sC4-N4	20.	ad6r	15.
1772.8	sC=O	72.					1787.8	sC=O	50.	sN-C.r	21.		
3041.7	ssCH3	98.					2910.2	ssCH3	100.				
3123.8	asCH3	75.	asCH3’	25.			2958.9	asCH3	75.	asCH3’	25.		
3160.8	sC-H	100.					2959.9	asCH3’	75.	asCH3	25.		
3171.9	asCH3’	73.	asCH3	24.			2992.8	sC-H	99.				
3179.8	sC-H	99.					2996.6	sC-H	99.				
3393.6	sNH	100.					3379.0	sNH	100.				
3550.7	sN-H	100.					3459.6	sN-H	99.				

2.7 3-methylcytosine protonated (3MC)

Figure 64: The energy-minimized structure of 3MC.

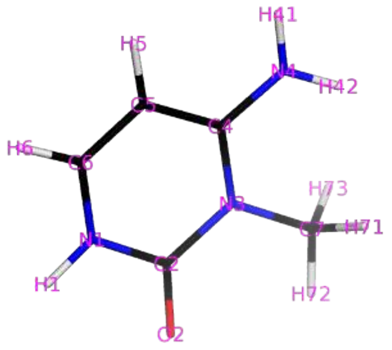

Table 181: The calculated geometric terms of 3MC.

Terms	QM	MM	diff
N1-C2	1.388	1.391	0.003
N1-C6	1.357	1.365	0.008
N1-H1	1.019	1.011	-0.008
C2-O2	1.213	1.227	0.014
C2-N3	1.427	1.429	0.002
N3-C4	1.356	1.373	0.018
N3-C7	1.474	1.484	0.010
C4-N4	1.333	1.347	0.014
C4-C5	1.422	1.415	-0.007
N4-H41	1.014	0.998	-0.016
N4-H42	1.012	0.996	-0.016
C5-H5	1.083	1.089	0.006
C5-C6	1.359	1.367	0.008
C6-H6	1.085	1.099	0.014
C7-H71	1.092	1.114	0.022
C7-H72	1.087	1.116	0.028
C7-H73	1.092	1.114	0.022
C2-N1-C6	124.85	122.31	-2.54
C2-N1-H1	114.22	113.70	-0.52
C6-N1-H1	120.93	123.99	3.06
N1-C2-O2	123.67	117.74	-5.93
N1-C2-N3	113.29	116.75	3.47
O2-C2-N3	123.05	125.50	2.46
C2-N3-C4	123.34	120.66	-2.67
C2-N3-C7	116.05	117.19	1.14
C4-N3-C7	120.62	122.15	1.53
N3-C4-N4	119.44	119.08	-0.36
N3-C4-C5	119.71	120.33	0.61
N4-C4-C5	120.85	120.60	-0.26
C4-N4-H41	119.82	121.44	1.62
C4-N4-H42	123.28	120.53	-2.75
H41-N4-H42	116.89	118.02	1.13
C4-C5-H5	120.69	121.01	0.32
C4-C5-C6	118.09	118.76	0.67
H5-C5-C6	121.22	120.22	-0.99
N1-C6-C5	120.72	121.18	0.46
N1-C6-H6	116.44	117.97	1.53
C5-C6-H6	122.84	120.85	-1.99
N3-C7-H71	109.69	111.45	1.76
N3-C7-H72	107.24	113.00	5.76
N3-C7-H73	109.69	111.45	1.76
H71-C7-H72	109.41	106.11	-3.30
H71-C7-H73	111.31	108.41	-2.91
H72-C7-H73	109.41	106.11	-3.30
C6-N1-C2-O2	-180.00	180.00	0.00
C6-N1-C2-N3	0.00	0.00	-0.00
H1-N1-C2-O2	0.00	0.00	-0.00
H1-N1-C2-N3	180.00	180.00	-0.00
C2-N1-C6-C5	-0.00	0.00	0.00
C2-N1-C6-H6	180.00	-180.00	0.00
H1-N1-C6-C5	-180.00	-180.00	0.00
H1-N1-C6-H6	0.00	0.00	-0.00
N1-C2-N3-C4	-0.00	0.00	0.00
N1-C2-N3-C7	-180.00	180.00	-0.00
O2-C2-N3-C4	180.00	180.00	0.00
O2-C2-N3-C7	-0.00	0.00	0.00
C2-N3-C4-N4	180.00	-180.00	0.00
C2-N3-C4-C5	-0.00	0.00	0.00
C7-N3-C4-N4	-0.00	0.00	0.00
C7-N3-C4-C5	180.00	-180.00	0.00
N3-C4-N4-H41	-180.00	180.00	0.00
N3-C4-N4-H42	-0.00	0.00	0.00
C5-C4-N4-H41	0.00	0.00	-0.00
C5-C4-N4-H42	180.00	-180.00	0.00
N3-C4-C5-H5	-180.00	180.00	0.00
N3-C4-C5-C6	0.00	0.00	-0.00
N4-C4-C5-H5	0.00	0.00	-0.00
N4-C4-C5-C6	180.00	180.00	-0.00
C4-C5-C6-N1	0.00	0.00	-0.00
C4-C5-C6-H6	180.00	-180.00	0.00
H5-C5-C6-N1	180.00	180.00	0.00
H5-C5-C6-H6	-0.00	0.00	0.00

Figure 65: The PES scan for flexible dihedral corresponding to 3MC.

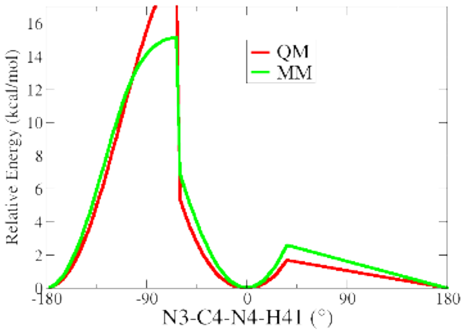

Table 182: The statistics of vibrational frequencies of 3MC. Only the terms with the occupancies greater than 15% were shown. Refer to p.6 for the meanings of assig.

QM (MP2, scaled by 0.943)							MM						
Freq	Assig.	%	Assig.	%	Assig.	%	Freq	Assig.	%	Assig.	%	Assig.	%
98.7	at6r'	58.	pk6r	52.	wN3-C7	-17.	75.7	tCNCH3	64.	pk6r	16.		
157.4	at6r	75.	at6r'	21.			127.4	tCNCH3	33.	wN3-C7	16.	at6r	16.
205.8	tCNCH3	63.	wN3-C7	31.	at6r	17.	173.0	at6r	53.	at6r'	39.		
231.2	wN3-C7	56.					240.3	wN3-C7	78.				
367.4	rC-N4	45.	rN3-C7	29.			361.4	wN-H	100.				
382.2	rN3-C7	46.	rC=O	24.			366.4	rC-N4	65.				
397.7	wNH2	71.	pk6r	22.	at6r'	16.	398.5	rN3-C7	59.	rC=O	20.		
414.6	wNH2	38.	at6r'	16.			399.4	wNH2	27.	at6r'	26.	at6r	22.
492.1	ad6r	29.	ad6r'	28.			496.6	wNH2	48.	pk6r	27.	at6r'	19.
521.4	tC4N4	101.					499.3	ad6r'	41.	sN3-C7	16.		
527.5	rC=O	27.	ad6r	22.	rC-N4	17.	530.7	sN-C.r	28.	rC=O	25.		
551.8	ad6r'	25.	rC=O	22.	ad6r	21.	589.3	tC4N4	38.	wC-N4	25.		
622.8	wC-N4	77.	wN-H	18.			604.5	ad6r	43.				
661.5	wC=O	34.	wN-H	31.	pk6r	23.	638.9	wC5-H5	59.				
693.2	sC2-N3	24.	sN3-C7	19.	sN-C.r	18.	659.8	sN-C.r	21.	sC2-N3	16.	sN3-C7	16.
705.6	wC=O	66.	wN-H	51.			696.9	wC=O	77.				
734.7	wC5-H5	84.					829.6	wC-N4	34.	tC4N4	24.	wNH2	21.
837.5	td6r	53.					843.4	td6r	32.				
921.5	wC6-H6	100.					917.9	wC6-H6	88.	wC5-H5	26.		
965.5	sC-C.r	20.	rNH2	18.			935.2	sN-C.r	26.	sC-C.r	20.		
1039.8	rNH2	35.					970.5	rCH3	66.	rCH3'	22.		
1111.5	sN-C.r	27.					982.2	rCH3'	22.	sN-C.r	19.		
1140.1	rCH3	69.	rCH3'	23.			1039.7	rNH2	45.				
1153.7	rC-H	32.	sN-C.r	32.			1111.7						
1182.5	sN3-C7	41.					1160.3	rC-H	44.	sC-C.r	29.		
1249.5	rC-H	41.	sN-C.r	28.	rN-H	22.	1168.4	sN3-C7	16.				
1262.4	rCH3'	27.	sC2-N3	22.			1314.7	sC4-N4	22.	rC-H	21.	rNH2	16.
1383.6	rC-H	59.					1402.0	adCH3'	47.	adCH3	16.		
1434.0	sdCH3	89.					1408.4	adCH3	67.	adCH3'	20.		
1474.0	rN-H	46.					1408.7	rN-H	40.				
1506.0	adCH3'	56.	adCH3	19.			1491.6	sdCH3	33.				
1516.0	adCH3	71.	adCH3'	23.			1535.4	cNH2	28.	rC-H	28.	sdCH3	20.
1563.1	cNH2	24.	sC4-N4	24.	sN3-C4	16.	1544.3	sN3-C4	18.	cNH2	16.	rC-H	15.
1588.7	sC-C.r	26.	sN3-C4	19.	sN-C.r	16.	1591.1	rC-H	24.	sdCH3	16.		
1658.7	cNH2	56.	sC-C.r	24.			1612.0	sN3-C4	18.	cNH2	15.		
1698.4	sC-C.r	32.	sC4-N4	18.	cNH2	17.	1685.8	sC-C.r	26.	rC-H	20.	sC4-N4	17.
1818.0	sC=O	76.					1809.0	sC=O	44.	sC2-N3	19.		
3029.3	ssCH3	95.					2854.5	ssCH3	100.				
3122.9	asCH3	75.	asCH3'	25.			2909.2	asCH3'	75.	asCH3	25.		
3170.4	asCH3'	71.	asCH3	24.			2911.0	asCH3	75.	asCH3'	25.		
3178.9	sC-H	99.					2994.0	sC-H	99.				
3196.7	sC-H	99.					2997.7	sC-H	99.				
3483.7	sN-H	96.					3330.7	ssNH2	99.				
3498.4	ssNH2	96.					3435.5	asNH2	100.				
3614.1	asNH2	99.					3459.5	sN-H	99.				

2.8 3H-pseudoisocytosine (3PC)

Figure 66: The energy-minimized structure of 3PC.

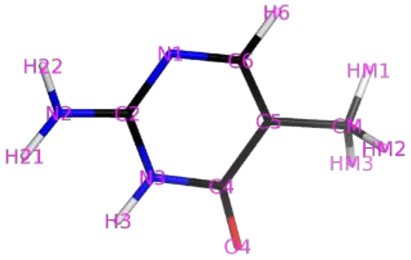

Table 183: The calculated geometric terms of 3PC.

Terms	QM	MM	diff
N1-C2	1.308	1.338	0.031
N1-C6	1.377	1.376	-0.001
C2-N2	1.386	1.321	-0.064
C2-N3	1.366	1.370	0.003
N2-H21	1.015	0.992	-0.023
N2-H22	1.015	0.994	-0.021
N3-H3	1.018	0.997	-0.021
N3-C4	1.408	1.373	-0.035
C4-O4	1.233	1.229	-0.005
C4-C5	1.450	1.445	-0.005
C5-C6	1.368	1.381	0.013
C5-CM	1.496	1.500	0.003
C6-H6	1.089	1.092	0.003
C2-N1-C6	115.56	114.92	-0.64
N1-C2-N2	120.36	120.39	0.03
N1-C2-N3	123.07	122.43	-0.63
N2-C2-N3	116.47	117.17	0.70
C2-N2-H21	115.43	123.66	8.22
C2-N2-H22	110.54	114.92	4.38
H21-N2-H22	112.10	121.42	9.32
C2-N3-H3	120.84	118.63	-2.21
C2-N3-C4	124.23	124.17	-0.06
H3-N3-C4	114.80	117.19	2.40
N3-C4-O4	120.25	118.29	-1.96
N3-C4-C5	112.67	114.99	2.33
O4-C4-C5	127.08	126.71	-0.37
C4-C5-C6	118.58	117.01	-1.57
C4-C5-CM	117.31	119.46	2.15
C6-C5-CM	124.10	123.04	-1.06
N1-C6-C5	125.86	126.24	0.38
N1-C6-H6	114.34	115.04	0.70
C5-C6-H6	119.79	118.70	-1.09
C6-N1-C2-N2	175.66	-179.80	4.55
C6-N1-C2-N3	-0.53	0.77	1.30
C2-N1-C6-C5	1.81	2.93	1.12
C2-N1-C6-H6	-179.08	-178.55	0.53
N1-C2-N2-H21	140.25	179.94	39.69
N1-C2-N2-H22	11.71	0.21	-11.50
N3-C2-N2-H21	-43.32	-0.59	42.73
N3-C2-N2-H22	-171.87	179.67	8.46
N1-C2-N3-H3	174.75	178.05	3.30
N1-C2-N3-C4	-0.86	-1.25	-0.39
N2-C2-N3-H3	-1.57	-1.40	0.17
N2-C2-N3-C4	-177.18	179.30	3.52
C2-N3-C4-O4	-179.75	179.22	1.02
C2-N3-C4-C5	1.00	-1.66	-2.66
H3-N3-C4-O4	4.39	-0.09	-4.48
H3-N3-C4-C5	-174.85	179.03	6.12
N3-C4-C5-C6	0.17	4.79	4.62
N3-C4-C5-CM	179.27	177.02	-2.24
O4-C4-C5-C6	-179.01	-176.18	2.83
O4-C4-C5-CM	0.08	-3.95	-4.03
C4-C5-C6-N1	-1.63	-5.82	-4.19
C4-C5-C6-H6	179.30	175.72	-3.59
CM-C5-C6-N1	179.35	-177.74	2.91
CM-C5-C6-H6	0.28	3.79	3.51

Table 184: The statistics of vibrational frequencies of 3PC. Only the terms with the occupancies greater than 15% were shown. Refer to p.6 for the meanings of assig.

QM (MP2, scaled by 0.943)							MM						
Freq	Assig.	%	Assig.	%	Assig.	%	Freq	Assig.	%	Assig.	%	Assig.	%
120.3	at6r	40.	at6r'	38.			126.7	at6r'	47.	at6r	43.		
140.1	C5CM	74.	at6r	22.			146.5	C5CM	96.				
160.5	at6r'	36.	pk6r	33.			262.8	wC-N	38.	at6r	30.	pk6r	29.
267.1	rC-C	68.					281.7	rC-C	72.				
297.2	wC-C	63.	tC2N2	18.			340.1	tC2N2	45.	wC-C	29.		
329.7	tC2N2	68.					359.0	rC-N	38.	sN-C.r	20.	rC=O	19.
336.1	rC-N	47.	rC=O	15.			372.5	wC-C	56.	tC2N2	29.		
403.4	at6r'	35.	pk6r	29.	at6r	22.	427.6	at6r'	54.	at6r	29.		
454.4	ad6r	39.	ad6r'	29.			471.2	ad6r'	27.	ad6r	23.	sC-N	17.
540.2	rC=O	25.	ad6r'	23.	sN-C.r	19.	511.6	pk6r	34.	wHH2	30.		
563.6	ad6r	34.					558.7	ad6r	36.	sN-C.r	19.		
611.9	wC-N	37.	wN-H	37.			579.8	sC-C.r	27.	ad6r	16.		
664.9	wN-H	37.	pk6r	34.	wHH2	16.	643.5	wN-H	87.	wHH2	17.		
696.1	wC=O	81.					670.7	td6r	42.				
729.3	wHH2	24.	wC-N	22.			681.5	wHH2	36.	wC-N	31.		
743.2	sC-C.r	42.	sC-C	15.			755.2	wC=O	22.	sC-C.r	21.	ad6r'	19.
778.1	td6r	41.	wHH2	18.			765.7	wC=O	49.				
898.2	wC-H	104.					887.2	sN-C.r	43.	wC-H	24.		
980.6	rCH3	30.	sN-C.r	29.			890.6	wC-H	69.				
1027.1	rCH3	35.	sN-C.r	21.	td6r	18.	967.2	rCH3	37.	rNH2	23.		
1053.7	rCH3'	88.					987.5	rNH2	54.	rCH3	16.		
1101.9	rNH2	57.	sN-C.r	15.			1017.3	rCH3'	57.	rCH3	21.		
1158.4	sN-C.r	48.	sC-C	20.			1147.9	sC-C	29.	sN-C.r	22.	td6r	15.
1245.2	sN1-C6	34.	sC-C	19.			1233.4	sN1-C6	38.	sC-N	21.		
1297.4	rC-H	22.	rN-H	21.	sC-N	19.	1289.8	sC-C.r	28.	rC-H	27.		
1318.3	rN-H	26.	rC-H	22.	sN1-C6	19.	1344.0	rC-H	30.	rN-H	26.		
1403.1	sC-C.r	31.	rC-H	30.			1406.2	adCH3	67.	sdCH3	24.		
1426.4	sdCH3	93.					1411.2	adCH3'	68.	sdCH3	18.		
1485.7	adCH3'	92.					1431.6	sdCH3	53.	adCH3'	22.	adCH3	19.
1504.2	adCH3	73.					1491.1	sC-C.r	23.	sN-C.r	19.	rC=O	17.
1516.0	sN-C.r	32.					1568.4	sN-C.r	56.				
1608.0	sN-C.r	28.	rNH2	25.			1583.2	rN-H	25.	rNH2	22.	sN-C.r	18.
1624.6	sC-C.r	42.					1649.0	rNH2	58.				
1664.0	rNH2	39.	sN-C.r	26.			1682.9	sC-C.r	21.	sN1-C6	16.		
1752.6	sC=O	66.					1819.9	sC=O	38.				
3011.1	ssCH3	100.					2904.0	ssCH3	100.				
3091.1	asCH3'	100.					2958.2	asCH3'	91.				
3106.8	asCH3	99.					2959.1	asCH3	91.				
3117.6	sC-H	98.					3077.3	sC-H	99.				
3462.6	ssNH2	99.					3440.9	ssNH2	91.				
3481.4	sN-H	99.					3456.7	sN-H	91.				
3574.5	asNH2	100.					3562.5	asNH2	99.				

2.9 N4-methylcytosine (4MC)

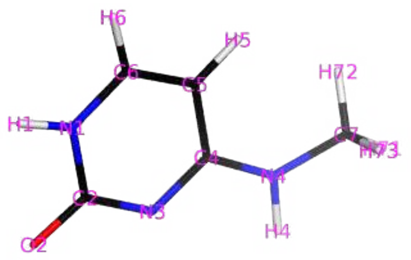

Figure 67: The energy-minimized structure of 4MC.

Table 185: The calculated geometric terms of 4MC.

Terms	QM	MM	diff
N1-C2	1.421	1.387	-0.034
N1-C6	1.356	1.364	0.008
N1-H1	1.014	1.006	-0.009
C2-O2	1.228	1.224	-0.004
C2-N3	1.376	1.351	-0.025
N3-C4	1.325	1.351	0.026
C4-N4	1.361	1.364	0.003
C4-C5	1.437	1.438	0.001
N4-H4	1.014	1.018	0.004
N4-C7	1.449	1.481	0.032
C5-H5	1.081	1.089	0.008
C5-C6	1.361	1.366	0.006
C6-H6	1.086	1.093	0.007
C7-H71	1.091	1.113	0.023
C7-H72	1.094	1.112	0.018
C7-H73	1.097	1.113	0.016
C2-N1-C6	123.88	121.39	-2.50
C2-N1-H1	114.69	112.43	-2.26
C6-N1-H1	121.43	126.18	4.76
N1-C2-O2	118.52	117.25	-1.28
N1-C2-N3	115.88	119.26	3.38
O2-C2-N3	125.60	123.49	-2.10
C2-N3-C4	120.21	120.88	0.67
N3-C4-N4	115.78	117.21	1.44
N3-C4-C5	124.26	120.54	-3.72
N4-C4-C5	119.95	122.24	2.29
C4-N4-H4	113.05	115.28	2.22
C4-N4-C7	124.89	127.46	2.57
H4-N4-C7	119.07	114.66	-4.41
C4-C5-H5	123.23	122.17	-1.05
C4-C5-C6	115.88	117.62	1.74
H5-C5-C6	120.90	120.20	-0.69
N1-C6-C5	119.88	120.31	0.42
N1-C6-H6	116.83	118.36	1.54
C5-C6-H6	123.29	121.33	-1.96

Terms	QM	MM	diff
N4-C7-H71	108.03	110.40	2.38
N4-C7-H72	110.95	111.76	0.81
N4-C7-H73	112.63	110.78	-1.84
H71-C7-H72	107.75	107.48	-0.27
H71-C7-H73	108.48	107.93	-0.54
H72-C7-H73	108.86	108.33	-0.53
C6-N1-C2-O2	179.81	179.87	0.06
C6-N1-C2-N3	0.08	-0.06	-0.14
H1-N1-C2-O2	0.11	-0.15	-0.25
H1-N1-C2-N3	-179.62	179.92	0.46
C2-N1-C6-C5	0.22	0.00	-0.22
C2-N1-C6-H6	-179.87	-179.99	-0.12
H1-N1-C6-C5	179.91	-179.98	0.12
H1-N1-C6-H6	-0.19	0.03	0.22
N1-C2-N3-C4	-0.44	-0.08	0.36
O2-C2-N3-C4	179.85	179.99	0.14
C2-N3-C4-N4	-178.16	179.45	2.39
C2-N3-C4-C5	0.53	0.28	-0.25
N3-C4-N4-H4	-7.78	-5.29	2.49
N3-C4-N4-C7	-167.93	-165.89	2.04
C5-C4-N4-H4	173.47	173.87	0.39
C5-C4-N4-C7	13.32	13.27	-0.06
N3-C4-C5-H5	179.80	179.98	0.18
N3-C4-C5-C6	-0.22	-0.33	-0.11
N4-C4-C5-H5	-1.56	0.85	2.42
N4-C4-C5-C6	178.42	-179.46	2.12
C4-C5-C6-N1	-0.16	0.19	0.35
C4-C5-C6-H6	179.94	-179.82	0.24
H5-C5-C6-N1	179.83	179.88	0.05
H5-C5-C6-H6	-0.07	-0.13	-0.05

Figure 68: The PES scan for flexible dihedral corresponding to 4MC.

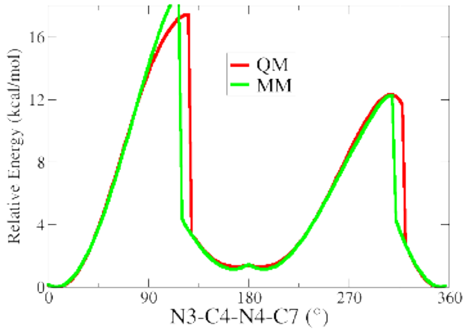

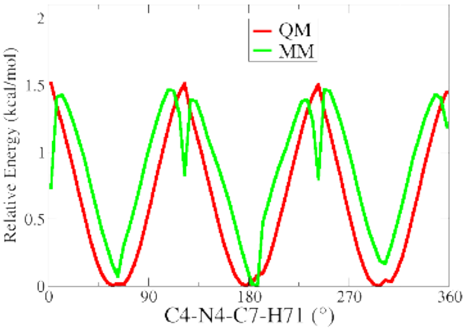

Table 186: The statistics of vibrational frequencies of 4MC. Only the terms with the occupancies greater than 15% were shown. Refer to p.6 for the meanings of assig.

QM (MP2, scaled by 0.943)							MM						
Freq	Assig.	%	Assig.	%	Assig.	%	Freq	Assig.	%	Assig.	%	Assig.	%
65.0	wN4H	70.	tC4N4	35.			93.0	tC4N4	42.	wNH	26.		
119.3	at6r	60.	at6r'	27.	CNCH	17.	157.2	at6r	51.	at6r'	31.		
150.4	CNCH	46.	pk6r	23.	at6r'	22.	195.7	CNCH	20.	rC-N4	18.	tC4N4	18.
208.5	at6r	33.	CNCH	32.	pk6r	25.	229.5	rC-N4	37.	dNH	18.		
220.0	rC-N4	42.	dN4H	35.			284.6	CNCH	55.	at6r	25.		
358.4	at6r'	43.	pk6r	21.			373.2	dNH	25.	sN-C.r	23.	sC4-N4	15.
400.9	rC=O	25.	dN4H	20.	sN-C.r	19.	446.6	at6r'	56.	pk6r	37.		
485.6	tC4N4	33.	ad6r	31.			539.8	sN-C.r	31.	ad6r'	21.	rC=O	19.
509.6	rC=O	31.	ad6r	19.			550.7	wN-H	100.				
549.6	ad6r'	61.	ad6r	16.			582.0	ad6r	34.	sC4-N4	19.	rC=O	18.
602.9	dN4H	22.	ad6r'	17.	rC=O	16.	629.5	ad6r'	21.				
612.8	wNH	66.	wC-N4	28.			670.6	wC5-H5	45.	wC6-H6	20.		
664.8	wC-N4	50.	pk6r	30.			690.6	wC=O	30.	wC-N4	26.	wC5-H5	17.
703.1	wC5-H5	53.	wC=O	23.			734.2	sC-C.r	26.	ad6r	22.	sN-C.r	22.
712.9	wC=O	74.	wC5-H5	16.			768.9	wNH	32.	wC=O	32.		
755.0	sN-C.r	27.	sC-C.r	26.			830.5	wNH	22.	wC-N4	22.	wC=O	18.
882.5	wC6-H6	97.					869.2	sN-C.r	59.				
908.8	sN-C.r	51.	td6r	15.			926.4	wC6-H6	75.	wC5-H5	30.		
973.1	td6r	47.	sC-C.r	25.			949.0	sN-C.r	44.	td6r	23.	sC-C.r	17.
1070.3	sN4-C7	37.	rCH3	26.			1030.3	td6r	37.	sN-C.r	22.		
1117.6	sN-C.r	31.	rC-H	30.	sC-C.r	20.	1052.7	sN4-C7	46.	rCH3	18.		
1145.8	rCH3'	91.					1084.9	rCH3'	42.	rCH3	40.		
1176.8	rCH3	41.	sN-C.r	18.	sN4-C7	17.	1125.7	rCH3'	30.	sN4-C7	25.	rCH3	19.
1213.2	rC-H	50.	rNH	26.	sN-C.r	16.	1159.1	rC-H	43.	sC-C.r	30.		
1252.1	sN-C.r	43.					1322.1	rC-H	25.	sC4-N4	20.	rN-H	15.
1344.5	rC-H	42.					1404.7	rN-H	39.				
1419.5	sN-C.r	46.					1420.4	sN-C.r	36.				
1428.7	rNH	23.	rN4H	22.			1431.8	adCH3	58.	adCH3'	17.		
1474.8	sdCH3	71.					1438.7	adCH3'	40.	sdCH3	25.	adCH3	25.
1502.7	adCH3'	94.					1462.1	sdCH3	65.	adCH3'	29.		
1531.3	adCH3	80.					1529.1	sN-C.r	40.	rC-H	28.	rC=O	18.
1549.2	sC4-N4	25.	sC-C.r	18.			1558.4	rC-H	45.	sN-C.r	24.		
1577.2	sN-C.r	26.	sC-C.r	26.	rN4H	21.	1618.3	rNH	42.				
1681.7	sC-C.r	41.	sN-C.r	24.			1696.5	sC-C.r	24.	rC-H	21.	sC4-N4	16.
1774.6	sC=O	72.					1768.2	sC=O	54.	sN-C.r	19.		
2995.2	ssCH3	90.					2974.3	ssCH3	99.				
3071.4	asCH3'	94.					2994.0	sC5-H5	50.	sC6-H6	49.		
3121.9	asCH3	94.					2997.2	sC6-H6	50.	sC5-H5	48.		
3153.7	sC6-H6	96.					3019.6	asCH3	92.				
3199.5	sC5-H5	96.					3022.7	asCH3'	92.				
3521.5	sN-H	84.	sNH	15.			3363.5	sNH	100.				
3523.9	sNH	84.	sN-H	15.			3459.7	sN-H	99.				

2.10 4-thiouracil (4SU)

Figure 69: The energy-minimized structure of 4SU.

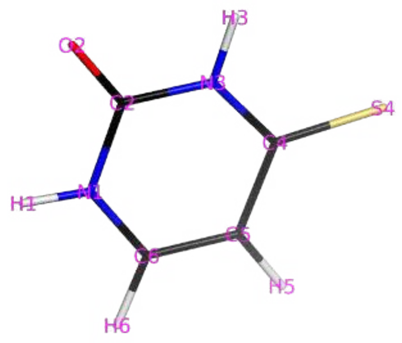

Table 187: The calculated geometric terms of 4SU.

Terms	QM	MM	diff
N1-C2	1.387	1.374	-0.013
N1-C6	1.376	1.368	-0.007
N1-H1	1.014	1.005	-0.008
C2-O2	1.224	1.224	0.000
C2-N3	1.390	1.362	-0.027
N3-C4	1.389	1.385	-0.004
N3-H3	1.018	1.002	-0.017
C4-S4	1.647	1.648	0.001
C4-C5	1.444	1.436	-0.008
C5-C6	1.355	1.373	0.018
C5-H5	1.082	1.088	0.005
C6-H6	1.085	1.095	0.009
C2-N1-C6	123.57	121.64	-1.93
C2-N1-H1	115.00	113.23	-1.78
C6-N1-H1	121.42	125.13	3.71
N1-C2-O2	123.65	122.02	-1.63
N1-C2-N3	112.56	116.09	3.52
O2-C2-N3	123.78	121.89	-1.89
C2-N3-C4	128.79	126.53	-2.27
C2-N3-H3	114.43	115.02	0.60
C4-N3-H3	116.78	118.45	1.67
N3-C4-S4	121.40	121.22	-0.18
N3-C4-C5	113.43	114.86	1.44
S4-C4-C5	125.18	123.92	-1.26
C4-C5-C6	120.45	119.35	-1.11
C4-C5-H5	118.60	120.27	1.67
C6-C5-H5	120.94	120.38	-0.57
N1-C6-C5	121.19	121.53	0.34
N1-C6-H6	115.77	117.97	2.20
C5-C6-H6	123.05	120.50	-2.55
C6-N1-C2-O2	180.00	180.00	0.00
C6-N1-C2-N3	-0.00	0.00	0.00
H1-N1-C2-O2	-0.00	0.00	0.00
H1-N1-C2-N3	180.00	-180.00	0.00
C2-N1-C6-C5	0.00	0.00	-0.00
C2-N1-C6-H6	180.00	-180.00	0.00
H1-N1-C6-C5	180.00	180.00	-0.00
H1-N1-C6-H6	0.00	0.00	0.00
N1-C2-N3-C4	0.00	0.00	-0.00
N1-C2-N3-H3	-180.00	-180.00	-0.00
O2-C2-N3-C4	180.00	-180.00	-0.00
O2-C2-N3-H3	0.00	0.00	-0.00
C2-N3-C4-S4	-180.00	180.00	0.00
C2-N3-C4-C5	0.00	0.00	0.00
H3-N3-C4-S4	0.00	0.00	-0.00
H3-N3-C4-C5	180.00	180.00	0.00
N3-C4-C5-C6	-0.00	0.00	0.00
N3-C4-C5-H5	180.00	-180.00	0.00
S4-C4-C5-C6	-180.00	-180.00	0.00
S4-C4-C5-H5	-0.00	0.00	0.00
C4-C5-C6-N1	0.00	0.00	-0.00
C4-C5-C6-H6	180.00	-180.00	-0.00
H5-C5-C6-N1	180.00	180.00	-0.00
H5-C5-C6-H6	0.00	0.00	-0.00

Table 188: The statistics of vibrational frequencies of 4SU. Only the terms with the occupancies greater than 15% were shown. Refer to p.6 for the meanings of assig.

QM (MP2, scaled by 0.943)							MM						
Freq	Assig.	%	Assig.	%	Assig.	%	Freq	Assig.	%	Assig.	%	Assig.	%
118.3	pk6r	47.	at6r	43.			157.3	at6r	35.	pk6r	27.	wC=S	20.
151.3	at6r	53.	at6r'	42.	pk6r	15.	191.9	at6r	51.	at6r'	29.	wC=O	20.
267.4	rC=S	72.					242.3	rC=S	74.	sN-C	16.		
359.9	at6r'	59.	pk6r	16.	wC=S	16.	387.2	at6r'	59.	pk6r	33.		
432.4	ad6r	57.	sC=S	22.			422.8	sC=S	36.	ad6r	24.		
479.1	rC=O	63.	rC=S	16.			517.5	wC=S	45.	pk6r	19.		
538.6	wN1-H1	72.	wC=S	30.			525.3	wN1-H1	88.				
548.6	ad6r'	73.					543.6	sN-C	32.	rC=O	30.	ad6r'	16.
611.8	wC=S	44.	pk6r	42.	wN1-H1	22.	570.1	ad6r	26.	ad6r'	22.	sN-C	18.
682.7	wC=O	70.	wN3-H3	16.			631.2	wC6-H6	48.	wC5-H5	25.	wC=O	18.
708.1	sN-C	23.	ad6r	18.	sC-C	18.	702.1	wN3-H3	82.	wC=O	25.		
714.8	wN3-H3	89.	wC=O	34.	pk6r	-16.	733.9	sC-C	27.	sN-C	23.	ad6r'	22.
760.3	wC5-H5	67.	wC6-H6	28.			736.6	pk6r	39.	wN3-H3	20.	wC=O	18.
881.9	wC6-H6	78.	wC5-H5	36.			817.1	sN-C	67.				
928.4	sN-C	64.					879.6	wC5-H5	65.	wC6-H6	50.		
980.3	td6r	60.	sC-C	18.			929.6	sN-C	41.	td6r	32.		
1087.6	sN-C	43.	rC-H	26.	sC=C	17.	1002.0	sN-C	27.	td6r	24.		
1175.8	sC=S	39.	rN-H	16.			1146.8	rC-H	38.	sC=C	31.		
1205.3	rC-H	47.	sN-C	32.			1213.0	rC-H	34.	sC=S	20.	sC-C	18.
1259.3	sN-C	44.	rC-H	24.	rN-H	18.	1302.1	rN-H	39.	sN-C	19.		
1358.2	sN-C	41.	rC-H	30.			1398.8	rN-H	69.				
1393.1	rN-H	43.	sN-C	18.			1483.8	rN-H	23.	sN-C	19.		
1466.8	rN-H	49.	sN-C	18.			1496.5	sN-C	34.	rC=O	23.	rC-H	22.
1491.7	sN-C	29.	rC-H	22.	rN-H	21.	1561.4	sN-C	33.	rC-H	31.	rN-H	16.
1661.7	sC=C	59.	rC-H	15.			1648.8	rC-H	29.	sC=C	29.		
1804.2	sC=O	71.					1784.2	sC=O	49.	sN-C	21.		
3165.8	sC-H	100.					2993.3	sC-H	99.				
3201.7	sC-H	99.					2996.8	sC-H	98.				
3478.9	sN-H	100.					3453.3	sN-H	99.				
3537.4	sN-H	100.					3459.9	sN-H	99.				

2.11 5-methyl-2-thiouracil (52U)

Figure 70: The energy-minimized structure of 52U.

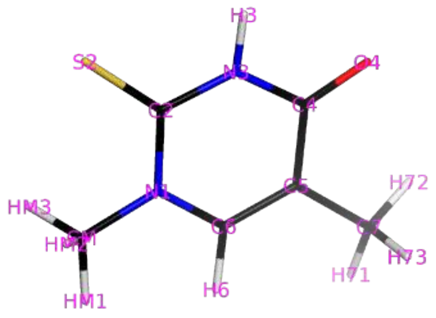

Table 189: The calculated geometric terms of 52U.

Terms	QM	MM	diff
N1-C2	1.378	1.417	0.039
N1-C6	1.384	1.374	-0.010
N1-CM	1.465	1.471	0.006
C2-S2	1.656	1.661	0.005
C2-N3	1.380	1.394	0.013
N3-H3	1.020	1.005	-0.015
N3-C4	1.400	1.384	-0.016
C4-O4	1.236	1.230	-0.006
C4-C5	1.455	1.443	-0.012
C5-C6	1.360	1.374	0.015
C5-C7	1.497	1.500	0.003
C6-H6	1.087	1.089	0.002
C7-H71	1.095	1.112	0.018
C7-H72	1.094	1.114	0.020
C7-H73	1.094	1.113	0.019
C2-N1-C6	121.63	121.45	-0.18
C2-N1-CM	118.63	117.98	-0.65
C6-N1-CM	119.60	120.57	0.97
N1-C2-S2	124.31	125.71	1.40
N1-C2-N3	113.50	113.44	-0.07
S2-C2-N3	122.18	120.85	-1.33
C2-N3-H3	115.21	118.14	2.93
C2-N3-C4	128.93	127.70	-1.22
H3-N3-C4	115.49	114.11	-1.37
N3-C4-O4	120.70	118.48	-2.22
N3-C4-C5	113.77	115.86	2.09
O4-C4-C5	125.52	125.65	0.14
C4-C5-C6	117.88	117.77	-0.11
C4-C5-C7	118.43	119.26	0.83
C6-C5-C7	123.64	122.39	-1.25
N1-C6-C5	124.00	123.58	-0.41
N1-C6-H6	114.73	115.91	1.18
C5-C6-H6	121.26	120.51	-0.75
C5-C7-H71	110.41	111.70	1.29
C5-C7-H72	110.45	111.04	0.58
C5-C7-H73	110.92	110.76	-0.17
H71-C7-H72	107.00	107.43	0.43
H71-C7-H73	108.96	108.24	-0.72
H72-C7-H73	108.98	107.50	-1.48
C6-N1-C2-S2	-177.07	179.66	3.28
C6-N1-C2-N3	4.25	-0.85	-5.10
CM-N1-C2-S2	-1.33	-0.42	0.91
CM-N1-C2-N3	179.98	179.07	-0.91
C2-N1-C6-C5	-2.70	-1.33	1.37
C2-N1-C6-H6	178.70	177.91	-0.79
CM-N1-C6-C5	-178.39	178.75	2.86
CM-N1-C6-H6	3.01	-2.01	-5.02
N1-C2-N3-H3	-178.87	-177.88	0.99
N1-C2-N3-C4	-6.28	-0.31	5.98
S2-C2-N3-H3	2.42	1.64	-0.77
S2-C2-N3-C4	175.00	179.21	4.21
C2-N3-C4-O4	-175.38	-176.93	-1.55
C2-N3-C4-C5	5.81	3.32	-2.49
H3-N3-C4-O4	-2.81	0.72	3.53
H3-N3-C4-C5	178.38	-179.02	2.60
N3-C4-C5-C6	-3.22	-5.15	-1.93
N3-C4-C5-C7	179.14	-176.67	4.20
O4-C4-C5-C6	178.04	175.13	-2.91
O4-C4-C5-C7	0.40	3.61	3.22
C4-C5-C6-N1	2.09	4.44	2.35
C4-C5-C6-H6	-179.40	-174.77	4.64
C7-C5-C6-N1	179.60	175.67	-3.92
C7-C5-C6-H6	-1.89	-3.54	-1.64

Table 190: The statistics of vibrational frequencies of 52U. Only the terms with the occupancies greater than 15% were shown. Refer to p.6 for the meanings of assig.

QM (MP2, scaled by 0.943)							MM						
Freq	Assig.	%	Assig.	%	Assig.	%	Freq	Assig.	%	Assig.	%	Assig.	%
57.1	at6r'	62.	at6r	22.	tCCCHm	13.	107.7	at6r	89.	at6r'	25.	wN-CM	-15.
76.1	at6r	90.	wN-CM	29.			150.8	tCCCH	93.				
115.8	tCCCHm	77.	at6r'	13.			173.5	wC-C7	20.	at6r'	17.	at6r	17.
128.7	tCCCH	89.					211.1	tCCCHm	94.				
172.6	wN-CM	69.	pk6r	39.			245.8	rC=S	41.	rC-C7	31.	sN-C.ar	20.
224.5	wC-C7	105.	at6r'	-10.			277.7	pk6r	64.	wC-C7	37.	wN-CM	-19.
244.4	rC-C7	51.	rC=S	31.	sN-C.ar	12.	293.7	rC-C7	40.	rC=S	27.		
267.7	rC=S	40.	rC-C7	20.	rC=O	13.	344.0	rN-CM	26.	sN-C.ar	21.	sC=S	18.
327.8	at6r'	65.	pk6r	47.	wC-C7	-20.	391.3	wN-CM	48.				
334.9	rN-CM	46.	rC=O	21.	sN-C.ar	14.	400.5	wN-CM	31.				
388.3	ad6r'	33.	ad6r	30.	sC=S	11.	447.2	at6r'	56.	wC-C7	23.		
476.1	ad6r	38.	sN-C.ar	18.	ad6r'	10.	507.1	ad6r	45.	sN-C.ar	21.		
487.3	wC=S	90.	wC=O	14.	pk6r	-11.	617.1	sC-C.ar	24.	rN-CM	21.	td6r	16.
563.4	rC=O	29.	rN-CM	22.	rC=S	17.	641.3	rC=O	21.	wC=S	19.		
604.2	wC=O	60.	wN-H	15.	pk6r	10.	645.1	wC=S	38.	wN-CM	21.		
640.3	wN-H	37.	td6r	21.	wC=O	18.	713.7	wN-H	102.				
653.0	wN-H	45.	td6r	21.			737.6	ad6r'	35.	sN-C.ar	24.	sC-C.ar	24.
685.5	ad6r'	29.	sC-C.ar	25.	sN-C.ar	23.	771.9	pk6r	29.	wC=O	28.	wC-H	22.
802.8	wC-H	101.					774.4	sN-C.ar	27.				
810.0	sN-C.ar	22.	sC-C.ar	21.	sN-C.al	19.	791.3	wC-H	52.	wC=O	22.		
987.6	rCH3'	44.	rCH3	22.			961.5	rCH3	39.	sN-C.ar	27.		
1006.2	rCH3	61.	rCH3'	28.			998.4	rCH3	51.	rCH3'	24.		
1045.9	rCH3'	28.	sN-C.ar	26.			1012.0	rCH3'	63.	rCH3	20.		
1083.5	sN-C.ar	25.	rCH3'	21.	sC=S	20.	1022.5	rCH3'	71.				
1097.9	rCH3	76.	rCH3'	17.			1076.8	rCH3	42.	sN-C.ar	17.		
1153.8	sN-C.ar	26.	rN-H	16.	sC=S	15.	1178.8	sN-C.ar	21.	td6r	15.		
1158.8	sN-C.ar	29.	sN-C.al	28.			1212.6	sN-C.ar	41.				
1219.5	sN-C.ar	47.	rC-H	18.	sC-C.ar	11.	1316.1	rC-H	43.	sC=C	22.		
1287.2	sN-C.ar	32.	rN-H	18.			1336.6	rN-H	20.				
1344.1	rC-H	36.	sN-C.ar	22.	sC=C	11.	1400.3	adCH3	63.	sN-C.ar	17.		
1379.7	sdCH3	83.					1406.7	adCH3	77.				
1396.8	sN-C.ar	22.	sdCH3	16.	rN-H	13.	1412.2	adCH3'	68.	sdCH3	21.		
1413.6	sdCH3	60.					1420.1	adCH3'	89.				
1416.7	adCH3	71.	adCH3'	22.			1435.6	sdCH3	58.	adCH3	17.		
1422.2	adCH3	71.	adCH3'	23.			1443.2	sN-C.ar	33.	adCH3	22.	sdCH3	16.
1440.7	sdCH3	25.	adCH3'	22.	rN-H	17.	1475.5	sdCH3	37.				
1454.6	adCH3'	62.	adCH3	20.			1537.4	rN-H	22.	sN-C.ar	17.	sdCH3	16.
1481.7	adCH3'	38.	sN-C.ar	17.	adCH3	12.	1585.9	sN-C.ar	22.	sC-C.ar	15.		
1603.4	sC=C	51.	sC=O	14.	rC-H	11.	1663.1	rC-H	29.	sC=C	16.	sC=O	15.
1658.4	sC=O	62.					1809.7	sC=O	41.	ad6r	16.		
2921.1	ssCH3	100.					2904.6	ssCH3	100.				
2941.6	ssCH3	99.					2908.1	ssCH3	100.				
3000.0	asCH3	76.	asCH3'	24.			2958.0	asCH3'	90.				
3011.4	asCH3'	76.	asCH3	24.			2958.9	asCH3	88.				
3029.4	asCH3	86.	asCH3'	14.			2959.1	asCH3'	96.				
3046.3	asCH3'	71.	sC-H	18.	asCH3	10.	2959.2	asCH3	98.				
3058.0	sC-H	81.	asCH3'	15.			2996.5	sC-H	98.				
3368.0	sN-H	100.					3460.0	sN-H	99.				

2.12 5-formylcytosine (5FC)

Figure 71: The energy-minimized structure of 5FC.

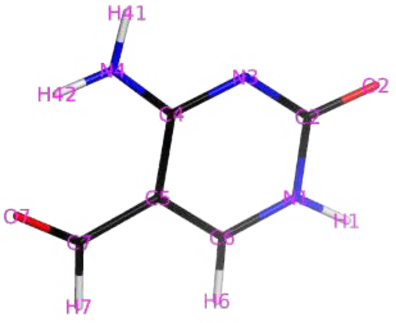

Table 191: The calculated geometric terms of 5FC.

Terms	QM	MM	diff
N1-C2	1.439	1.387	-0.052
N1-C6	1.340	1.365	0.024
N1-H1	1.016	1.007	-0.009
C2-O2	1.224	1.223	-0.001
C2-N3	1.370	1.350	-0.020
N3-C4	1.323	1.344	0.020
C4-N4	1.343	1.339	-0.005
C4-C5	1.456	1.444	-0.012
N4-H41	1.011	0.996	-0.016
N4-H42	1.014	0.999	-0.015
C5-C6	1.371	1.369	-0.002
C5-C7	1.454	1.465	0.011
C6-H6	1.089	1.094	0.005
C7-H7	1.109	1.111	0.001
C7-O7	1.236	1.215	-0.021
C2-N1-C6	123.67	121.37	-2.30
C2-N1-H1	114.62	112.16	-2.46
C6-N1-H1	121.72	126.47	4.76
N1-C2-O2	117.39	117.24	-0.15
N1-C2-N3	116.22	119.17	2.96
O2-C2-N3	126.39	123.59	-2.80
C2-N3-C4	120.46	121.08	0.62
N3-C4-N4	118.02	117.39	-0.63
N3-C4-C5	123.77	120.85	-2.92
N4-C4-C5	118.21	121.76	3.55
C4-N4-H41	117.76	117.09	-0.67
C4-N4-H42	119.94	121.55	1.60
H41-N4-H42	122.29	121.36	-0.93
C4-C5-C6	115.71	117.00	1.29
C4-C5-C7	125.12	123.71	-1.41
C6-C5-C7	119.17	119.29	0.12
N1-C6-C5	120.18	120.53	0.35
N1-C6-H6	117.44	117.85	0.41
C5-C6-H6	122.38	121.62	-0.76
C5-C7-H7	114.78	115.01	0.22
C5-C7-O7	125.53	127.09	1.56
H7-C7-O7	119.68	117.90	-1.78
C6-N1-C2-O2	180.00	180.00	0.00
C6-N1-C2-N3	0.00	0.00	0.00
H1-N1-C2-O2	0.00	0.00	0.00
H1-N1-C2-N3	180.00	180.00	0.00
C2-N1-C6-C5	0.00	0.00	-0.00
C2-N1-C6-H6	180.00	-180.00	-0.00
H1-N1-C6-C5	180.00	-180.00	-0.00
H1-N1-C6-H6	0.00	0.00	0.00
N1-C2-N3-C4	0.00	0.00	0.00
O2-C2-N3-C4	180.00	-180.00	0.00
C2-N3-C4-N4	180.00	-180.00	-0.00
C2-N3-C4-C5	0.00	0.00	0.00
N3-C4-N4-H41	-0.00	0.00	0.00
N3-C4-N4-H42	180.00	180.00	0.00
C5-C4-N4-H41	-180.00	-180.00	0.00
C5-C4-N4-H42	-0.00	0.00	0.00
N3-C4-C5-C6	0.00	0.00	0.00
N3-C4-C5-C7	180.00	180.00	0.00
N4-C4-C5-C6	180.00	180.00	0.00
N4-C4-C5-C7	-0.00	0.00	0.00
C4-C5-C6-N1	-0.00	0.00	0.00
C4-C5-C6-H6	180.00	-180.00	0.00
C7-C5-C6-N1	180.00	180.00	0.00
C7-C5-C6-H6	0.00	0.00	0.00
C4-C5-C7-H7	180.00	-180.00	-0.00
C4-C5-C7-O7	-0.00	0.00	0.00
C6-C5-C7-H7	0.00	0.00	-0.00
C6-C5-C7-O7	180.00	-180.00	0.00

Figure 72: The PES scan for flexible dihedral corresponding to 5FC.

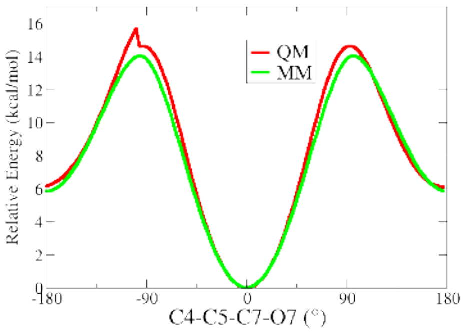

Table 192: The statistics of vibrational frequencies of 5FC. Only the terms with the occupancies greater than 15% were shown. Refer to p.6 for the meanings of assig.

QM (MP2, scaled by 0.943)							MM						
Freq	Assig.	%	Assig.	%	Assig.	%	Freq	Assig.	%	Assig.	%	Assig.	%
62.2	at6r	41.	wC7-C5	30.	at6r'	27.	89.7	at6r'	46.	at6r	26.	wC7-C5	18.
152.2	tCCCO	26.	wC7-C5	20.	at6r'	19.	170.5	tCCCO	48.	pk6r	22.		
177.9	at6r	37.	pk6r	25.			199.4	at6r	45.	wC-N4	27.	pk6r	20.
246.3	rC7-C5	74.	dCCO	18.			256.3	rC7-C5	71.				
274.0	wC7-C5	53.	tCCCO	40.			303.6	wC7-C5	39.	tCCCO	32.	pk6r	26.
334.7	wHH2	83.	pk6r	30.			370.0	rC-N4	50.	sN-C.r	21.		
363.5	rC-N4	52.	rC=O	19.			387.3	dCCO	23.	sC-C7	19.	ad6r'	18.
388.6	at6r'	38.	at6r	20.	pk6r	18.	454.8	at6r'	38.	at6r	18.	wC7-C5	17.
396.3	ad6r	25.	ad6r'	19.	sC-C7	15.	523.5	tC4N4	90.				
509.9	rC=O	36.	ad6r	27.	rC-N4	19.	557.9	sC-N4	20.	sN-C.r	17.		
554.6	ad6r'	32.	ad6r	19.	sN-C.r	16.	559.5	wN-H	104.				
587.2	wC-N4	65.					591.5	rC=O	27.	ad6r	25.	sN-C.r	15.
637.0	dCCO	29.					607.6	dCCO	21.	ad6r	17.		
664.4	tC4N4	34.	wC=O	23.	pk6r	18.	643.5	wHH2	63.				
673.6	wN-H	62.	tC4N4	32.			731.4	sC-C.r	38.	ad6r'	24.		
720.9	wC=O	70.	tC4N4	20.			738.4	wC=O	65.	wHH2	16.		
726.5	sN-C.r	38.	sC-C.r	37.			812.6	sN-C.r	19.				
795.0	td6r	30.	sN-C.r	18.	dCCO	16.	833.6	wC-H	63.	wC-N4	16.		
885.4	wC-H	102.					881.1	wC-H	37.	pk6r	29.	wC-N4	20.
906.0	td6r	33.	sN-C.r	31.			899.3	sN-C.r	42.	td6r	25.		
969.6	wCHO	92.					982.3	wCHO	86.				
1075.0	cNH2	34.	sN-C.r	32.			1021.8	sN-C.r	49.				
1191.1	rN-H	33.	rC-H	21.	sN-C.r	21.	1037.5	sN-C.r	32.	cNH2	31.		
1251.5	sN-C.r	46.	sC-C7	16.			1240.1	sC-C.r	22.				
1292.3	sN-C.r	27.	sC-C7	16.			1264.6	rC-H	22.	sC-C7	21.	rCHO	17.
1323.7	rC-H	37.	sC-N4	16.			1339.1	rCHO	45.	sC=O	34.		
1412.9	rCHO	74.					1406.3	rN-H	62.				
1440.0	rN-H	34.	sN-C.r	28.			1470.8	sN-C.r	23.	rC-H	17.		
1476.2	rNH2	20.	sN-C.r	20.	sC-N4	16.	1532.2	sN-C.r	52.				
1556.0	sN-C.r	37.	sC-C.r	31.			1594.6	sN-C.r	22.	sC-C.r	17.		
1616.7	rNH2	46.	sC-N4	21.			1622.5	rNH2	35.	rC-H	19.		
1679.7	sC=O	53.					1675.7	rNH2	29.				
1712.8	sC=O	26.	sC-C.r	22.	sN-C.r	15.	1760.5	sC=O	58.	sN-C.r	15.		
1785.7	sC=O	73.					1831.7	sC=O	31.	sC-C7	22.		
2897.7	sC7-H	100.					2907.8	sC7-H	99.				
3131.6	sC6-H	99.					2995.4	sC6-H	99.				
3466.7	ssNH2	97.					3442.3	ssNH2	99.				
3508.2	sN-H	100.					3460.6	sN-H	99.				
3618.3	asNH2	97.					3562.4	asNH2	99.				

2.13 5-hydroxyuracil (5HU)

Figure 73: The energy-minimized structure of 5HU.

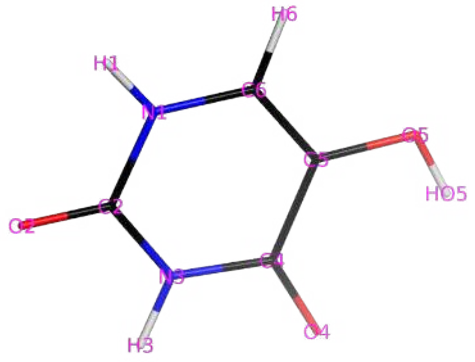

Table 193: The calculated geometric terms of 5HU.

Terms	QM	MM	diff
N1-C2	1.378	1.376	-0.002
N1-C6	1.390	1.375	-0.015
N1-H1	1.015	1.007	-0.007
C2-O2	1.230	1.223	-0.007
C2-N3	1.397	1.363	-0.033
N3-H3	1.019	1.002	-0.017
N3-C4	1.387	1.384	-0.003
C4-O4	1.240	1.233	-0.007
C4-C5	1.458	1.463	0.005
C5-C6	1.355	1.374	0.020
C5-O5	1.362	1.372	0.010
C6-H6	1.085	1.093	0.008
O5-HO5	0.983	0.967	-0.016
C2-N1-C6	124.63	121.70	-2.93
C2-N1-H1	114.83	113.52	-1.31
C6-N1-H1	120.01	124.78	4.77
N1-C2-O2	124.20	121.54	-2.67
N1-C2-N3	112.82	116.46	3.64
O2-C2-N3	122.96	122.01	-0.95
C2-N3-H3	115.33	116.98	1.65
C2-N3-C4	127.45	125.70	-1.75
H3-N3-C4	116.87	117.32	0.45
N3-C4-O4	123.14	117.91	-5.23
N3-C4-C5	114.60	116.42	1.82
O4-C4-C5	122.24	125.67	3.43
C4-C5-C6	120.05	117.16	-2.88
C4-C5-O5	116.92	121.66	4.73
C6-C5-O5	122.95	121.18	-1.77
N1-C6-C5	120.04	122.55	2.51
N1-C6-H6	116.93	118.34	1.42
C5-C6-H6	122.98	119.10	-3.88
C5-O5-HO5	105.74	108.61	2.86
C6-N1-C2-O2	175.62	-180.00	4.38
C6-N1-C2-N3	-5.95	-0.00	5.95
H1-N1-C2-O2	3.98	0.00	-3.98
H1-N1-C2-N3	-177.58	-180.00	-2.42
C2-N1-C6-C5	5.05	0.00	-5.05
C2-N1-C6-H6	-177.46	-180.00	-2.54
H1-N1-C6-C5	176.29	180.00	3.71
H1-N1-C6-H6	-6.23	0.00	6.23
N1-C2-N3-H3	179.40	180.00	0.60
N1-C2-N3-C4	6.49	0.00	-6.49
O2-C2-N3-H3	-2.14	0.00	2.14
O2-C2-N3-C4	-175.05	-180.00	-4.95
C2-N3-C4-O4	175.91	180.00	4.09
C2-N3-C4-C5	-5.55	-0.00	5.55
H3-N3-C4-O4	3.10	0.00	-3.10
H3-N3-C4-C5	-178.36	-180.00	-1.64
N3-C4-C5-C6	3.78	0.00	-3.78
N3-C4-C5-O5	-179.39	-180.00	-0.61
O4-C4-C5-C6	-177.68	180.00	2.32
O4-C4-C5-O5	-0.84	-0.00	0.84
C4-C5-C6-N1	-3.67	0.00	3.67
C4-C5-C6-H6	179.00	180.00	1.00
O5-C5-C6-N1	179.69	-180.00	0.31
O5-C5-C6-H6	2.37	-0.00	-2.37
C4-C5-O5-HO5	1.50	-0.00	-1.50
C6-C5-O5-HO5	178.24	180.00	1.76

Figure 74: The PES scan for flexible dihedral corresponding to 5HU.

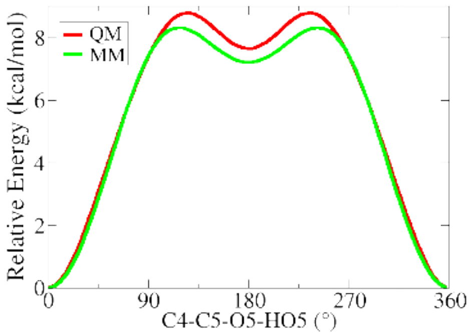

Table 194: The statistics of vibrational frequencies of 5HU. Only the terms with the occupancies greater than 15% were shown. Refer to p.6 for the meanings of assig.

QM (MP2, scaled by 0.943)							MM						
Freq	Assig.	%	Assig.	%	Assig.	%	Freq	Assig.	%	Assig.	%	Assig.	%
96.5	at6r'	67.	at6r	25.			124.6	at6r	44.	at6r'	38.		
130.2	pk6r	69.	at6r	67.	wC-O	-17.	168.2	pk6r	33.	at6r	26.	at6r'	19.
286.0	at6r'	50.	pk6r	39.	at6r	20.	273.9	wC-O	50.	pk6r	26.		
295.5	rC-O	60.	rC=O	22.			319.2	rC-O	72.				
325.4	wC-O	99.					396.8	tCCOH	78.				
378.1	rC=O	63.	sN-C	24.			402.2	rC=O	52.	sN-C	40.		
434.1	tCCOH	85.					406.0	at6r'	39.	tCCOH	19.	wC-O	17.
450.9	ad6r	47.	ad6r'	27.			472.1	ad6r'	35.	sN-C	25.	sC-O	16.
515.7	wN-H	73.					550.3	wN-H	97.				
525.7	ad6r'	28.	ad6r	18.			590.8	ad6r	47.				
592.8	wC=O4	69.	wC=O2	21.			627.9	rC=O	36.	sN-C	22.		
613.4	rC=O	47.	rC-O	22.			639.7	wC=O2	46.	wC=O4	25.		
643.6	wN-H	106.					676.4	wN-H	106.	pk6r	-15.		
681.4	wC=O2	84.	wC=O4	21.			747.6	sC-C	27.	ad6r'	27.	sN-C	23.
739.5	sC-C	38.	sN-C	30.			768.0	td6r	27.	rC=O	15.		
785.9	wC-H	100.					772.8	wC-H	89.				
799.3	td6r	55.	sC-O	18.			780.6	pk6r	46.	wC=O4	39.		
958.1	sN-C	51.					876.8	sN-C	49.	td6r	19.		
1132.6	sN-C	41.	rN-H	18.			1036.3	sN-C	57.	rN-H	17.		
1212.1	sN-C	38.	rC-H	35.			1206.6	sN-C	19.	td6r	18.	sC-O	16.
1245.6	dCOH	41.	sN-C	27.			1295.7	rC-H	34.	sC=C	22.		
1263.8	sC-O	44.	sN-C	37.			1388.1	rN-H	62.	sC=O	21.		
1368.5	rN-H	63.	sC=O	18.			1412.2	rN-H	65.				
1392.7	sN-C	29.	rC-H	25.	dCOH	22.	1479.2	sN-C	35.				
1418.3	rN-H	55.					1542.0	sN-C	40.	rC=O	24.		
1501.5	sN-C	32.	sC-C	18.			1621.5	dCOH	60.				
1684.1	sC=C	43.	sC=O	27.			1670.0	rC-H	23.	sC=C	18.		
1695.0	sC=O	49.	sC=C	18.			1759.5	sC=O	56.				
1759.0	sC=O	66.					1836.2	sC=O	37.				
3175.3	sC-H	99.					2998.7	sC-H	99.				
3478.7	sN-H	100.					3457.3	sN-H	99.				
3504.4	sO-H	100.					3461.9	sN-H	99.				
3528.5	sN-H	100.					3749.5	sO-H	100.				

2.14 7-methylguanine (7MG)

Figure 75: The energy-minimized structure of 7MG.

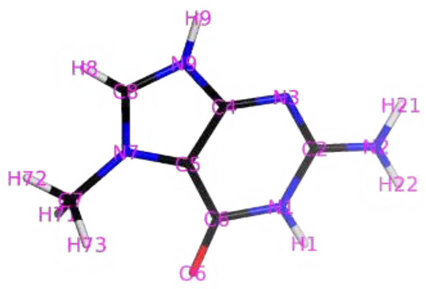

Table 195: The calculated geometric terms of 7MG.

Terms	QM	MM	diff
N9-C8	1.350	1.334	-0.016
N9-C4	1.380	1.365	-0.014
N9-H9	1.018	0.998	-0.020
C8-H8	1.081	1.078	-0.003
C8-N7	1.340	1.333	-0.006
N7-C5	1.376	1.399	0.023
N7-C7	1.472	1.481	0.008
C5-C6	1.442	1.415	-0.028
C5-C4	1.387	1.390	0.004
C6-O6	1.223	1.230	0.007
C6-N1	1.415	1.386	-0.029
N1-H1	1.020	1.001	-0.019
N1-C2	1.381	1.377	-0.004
C2-N2	1.346	1.326	-0.020
C2-N3	1.327	1.346	0.020
N2-H21	1.013	0.997	-0.016
N2-H22	1.011	0.994	-0.017
N3-C4	1.344	1.333	-0.011
C7-H71	1.089	1.113	0.024
C7-H72	1.089	1.113	0.024
C7-H73	1.089	1.113	0.024
C8-N9-C4	109.41	109.70	0.29
C8-N9-H9	125.63	127.54	1.91
C4-N9-H9	124.96	122.76	-2.21
N9-C8-H8	125.82	125.34	-0.48
N9-C8-N7	108.72	109.33	0.61
H8-C8-N7	125.46	125.33	-0.13
C8-N7-C5	108.14	107.96	-0.18
C8-N7-C7	126.29	126.52	0.23
C5-N7-C7	125.57	125.52	-0.05
N7-C5-C6	131.07	133.56	2.49
N7-C5-C4	108.25	106.55	-1.70
C6-C5-C4	120.68	119.89	-0.79
C5-C6-O6	128.57	129.05	0.48
C5-C6-N1	108.43	112.37	3.94
O6-C6-N1	122.99	118.58	-4.42
C6-N1-H1	113.50	116.42	2.92
C6-N1-C2	126.71	125.16	-1.56
H1-N1-C2	119.76	118.42	-1.34
N1-C2-N2	117.34	116.45	-0.88
N1-C2-N3	123.60	121.45	-2.15
N2-C2-N3	119.03	122.09	3.06
C2-N2-H21	116.82	117.52	0.70
C2-N2-H22	122.39	123.63	1.24
H21-N2-H22	117.53	118.85	1.32
C2-N3-C4	112.27	115.39	3.12
N9-C4-C5	105.48	106.45	0.98
N9-C4-N3	126.21	127.80	1.59
C5-C4-N3	128.31	125.74	-2.56
N7-C7-H71	108.72	109.08	0.36
N7-C7-H72	108.16	111.12	2.96
N7-C7-H73	108.74	109.08	0.34
H71-C7-H72	110.70	109.25	-1.45
H71-C7-H73	109.77	109.02	-0.76
H72-C7-H73	110.69	109.25	-1.43
C4-N9-C8-H8	-179.95	180.00	0.05
C4-N9-C8-N7	0.07	-0.00	-0.07
H9-N9-C8-H8	-0.13	0.00	0.13
H9-N9-C8-N7	179.89	-180.00	0.11
C8-N9-C4-C5	-0.15	0.00	0.15
C8-N9-C4-N3	-179.72	-180.00	-0.28
H9-N9-C4-C5	-179.97	180.00	0.03
H9-N9-C4-N3	0.46	0.00	-0.46
N9-C8-N7-C5	0.04	0.00	-0.04
N9-C8-N7-C7	179.98	180.00	0.02
H8-C8-N7-C5	-179.94	-180.00	-0.06
H8-C8-N7-C7	-0.00	0.00	0.00
C8-N7-C5-C6	-179.50	180.00	0.50
C8-N7-C5-C4	-0.14	0.00	0.14
C7-N7-C5-C6	0.56	0.00	-0.56
C7-N7-C5-C4	179.93	-180.00	0.07
N7-C5-C6-O6	-0.16	0.00	0.16
N7-C5-C6-N1	179.54	-180.00	0.46
C4-C5-C6-O6	-179.46	180.00	0.54
C4-C5-C6-N1	0.24	0.00	-0.24
N7-C5-C4-N9	0.17	0.00	-0.17
N7-C5-C4-N3	179.73	180.00	0.27
C6-C5-C4-N9	179.62	-180.00	0.38
C6-C5-C4-N3	-0.82	0.00	0.82
C5-C6-N1-H1	-177.92	-180.00	-2.08
C5-C6-N1-C2	0.22	0.00	-0.22
O6-C6-N1-H1	1.79	0.00	-1.79
O6-C6-N1-C2	179.94	-180.00	0.06
C6-N1-C2-N2	-178.08	180.00	1.92
C6-N1-C2-N3	-0.21	0.00	0.21
H1-N1-C2-N2	-0.04	0.00	0.04
H1-N1-C2-N3	177.83	180.00	2.17
N1-C2-N2-H21	-174.88	-180.00	-5.12
N1-C2-N2-H22	-15.79	0.00	15.79
N3-C2-N2-H21	7.15	0.00	-7.15
N3-C2-N2-H22	166.23	-180.00	13.77
N1-C2-N3-C4	-0.28	0.00	0.28
N2-C2-N3-C4	177.56	-180.00	2.44
C2-N3-C4-N9	-179.72	180.00	0.28
C2-N3-C4-C5	0.80	0.00	-0.80

Table 196: The statistics of vibrational frequencies of 7MG. Only the terms with the occupancies greater than 15% were shown. Refer to p.6 for the meanings of assig.

QM (MP2, scaled by 0.943)							MM						
Freq	Assig.	%	Assig.	%	Assig.	%	Freq	Assig.	%	Assig.	%	Assig.	%
66.3	tN7C7	60.	at6r	18.	wN7C7	17.	105.0	at6r	36.	btfl	19.		
98.6	at6r	51.	tN7C7	25.			118.1	tN7C7	89.				
136.5	pk6r	45.	btfl	45.			191.5	t5r	39.	btfl	20.		
175.5	at6r’	79.					244.8	rN7C7	39.				
210.6	rN7C7	35.	ad6r	17.			258.8	wN2-C	30.	at6r’	19.	btfl	19.
213.0	wN7C7	41.	at6r	20.			301.1	at6r’	46.	wN7C7	24.		
226.8	wNH2	61.	tC2N2	21.			335.1	rN2-C	40.	sC-N.ar	27.		
332.3	rN2-C	45.	rC=O	27.	sC-N.ar	17.	353.4	tC2N2	63.	wN7C7	15.		
357.4	t5r’	27.	wN7C7	19.			368.7	rC=O	25.	sC-N.ar	16.		
373.5	rN7C7	35.					395.0	at6r’	26.	t5r’	23.	tC2N2	20.
460.4	tC2N2	51.	wNH2	24.			488.1						
471.4	ad6r	33.	ad6r’	22.			532.2	wN1-H	28.	wNH2	25.	pk6r	21.
508.8	ad6r’	47.					563.0	wN9H	37.	t5r’	35.		
574.9	t5r	48.					563.1	ad6r	20.				
586.9	t5r’	46.	t5r	28.			573.2	ad6r’	44.	sC-N.ar	16.		
611.2	sC-N.ar	27.	td6r	19.			610.4	wN1-H	35.	t5r	23.	at6r	16.
614.3	wN1-H	32.	wC=O	27.			641.7						
636.5	wN2-C	27.	wC=O	26.			652.0	wN1-H	38.	pk6r	21.	t5r	18.
644.0	wN2-C	29.					691.4	rC=O	20.	sC-N.ar	17.		
653.9	wN9H	81.	t5r’	15.			698.0	wNH2	47.	wN2-C	29.		
681.3	wC=O	19.	wN2-C	16.	wN1-H	15.	718.9	wN9H	44.	pk6r	18.	wC-H	18.
687.2	wN1-H	20.	wC=O	16.			762.1	wC=O	72.	wC-H	34.		
738.5	wC-H	102.					769.5	d5r	37.	sC-N.ar	16.		
825.1	d5r	43.	td6r	27.			791.4	wC=O	26.	wC-H	25.	t5r’	16.
1012.4	sC-N.ar	44.	rNH2	20.			922.8	sC-N.ar	54.				
1054.4	sC-N.ar	21.	rCH3’	16.			969.3	rCH3	62.	rCH3’	21.		
1067.2	rNH2	37.	sC-N.ar	31.			989.1	rCH3’	55.	rCH3	18.		
1118.9	sC-N.ar	27.	rCH3’	27.			1014.3	rNH2	74.				
1129.5	rC-H	33.	sC-N.ar	26.			1047.2	sC-N.ar	25.	sC-C.ar	18.		
1142.8	rCH3	66.	rCH3’	23.			1064.2	sN7-C7	22.	sC-N.ar	19.		
1152.5	sC-N.ar	33.					1159.5	sC-N.ar	53.	rN9-H	22.		
1238.4	sC-N.ar	25.	sN7-C7	18.			1235.4	sC-N.ar	20.				
1309.2	rC-H	29.	sC-N.ar	22.			1276.7	sC-N.ar	23.	rN9-H	17.	rC-H	17.
1348.6	rN1-H	55.	sC2-N2	18.			1339.9	rN1-H	29.	sC-N.ar	15.		
1405.4	sN7-C5	38.	sC-N.ar	23.			1351.3	sC-N.ar	42.	rC=O	16.		
1416.6	sC-N.ar	43.	rN9-H	31.			1413.4	adCH3’	60.	adCH3	20.		
1456.6	sdCH3	67.					1414.4	adCH3	69.	adCH3’	23.		
1464.1	sC-N.ar	27.	sdCH3	22.			1452.1	sdCH3	57.				
1480.9	adCH3	67.	adCH3’	23.			1507.2	sC-C.ar	20.	sC-N.ar	20.		
1501.7	adCH3’	53.	adCH3	18.			1538.8	sC-N.ar	31.	rN9-H	20.	sN9-C4	15.
1561.6							1583.3	sC-N.ar	26.	rN1-H	26.	sC-C.ar	17.
1569.3	sC-N.ar	60.					1596.4	sC-N.ar	39.				
1599.1	cNH2	40.	sC-N.ar	25.			1622.9	sC-N.ar	25.	d5r’	15.		
1638.0	sC-N.ar	38.	sC-C.ar	29.			1638.0	cNH2	43.	sC-N.ar	15.		
1674.6	cNH2	36.	sC2-N2	30.	sC-N.ar	20.	1677.9	cNH2	31.				
1794.9	sC=O	69.	sC-C.ar	15.			1824.3	sC=O	40.				
3053.2	ssCH3	100.					2848.1	ssCH3	100.				
3164.6	asCH3	60.	asCH3’	40.			2914.0	asCH3	75.	asCH3’	25.		
3166.0	asCH3’	60.	asCH3	40.			2915.4	asCH3’	75.	asCH3	25.		
3234.5	sC-H	99.					3057.8	sC-H	100.				
3460.3	sN-H	99.					3415.2	sN-H	99.				
3498.5	sN-H	96.					3443.8	ssNH	89.				
3506.4	ssNH	95.					3458.7	sN-H	88.				
3629.0	asNH2	100.					3558.3	asNH2	99.				

2.15 8-methyladenine (8MA)

Figure 76: The energy-minimized structure of 8MA.

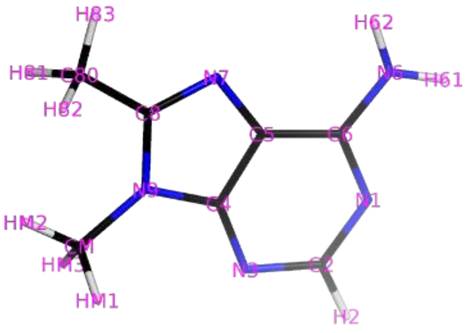

Table 197: The calculated geometric terms of 8MA.

Terms	QM	MM	diff
N9-C8	1.379	1.388	0.009
N9-C4	1.378	1.381	0.003
N9-CM	1.453	1.474	0.021
C8-N7	1.333	1.322	-0.011
C8-C80	1.489	1.490	0.001
N7-C5	1.377	1.397	0.020
C5-C6	1.407	1.410	0.003
C5-C4	1.399	1.399	0.000
C6-N6	1.367	1.345	-0.022
C6-N1	1.342	1.359	0.017
N6-H61	1.013	0.995	-0.017
N6-H62	1.012	0.996	-0.016
N1-C2	1.352	1.362	0.010
C2-H2	1.088	1.094	0.006
C2-N3	1.340	1.358	0.018
N3-C4	1.343	1.344	0.001
C80-H81	1.095	1.109	0.014
C80-H82	1.091	1.109	0.019
C80-H83	1.095	1.111	0.016
C8-N9-C4	106.60	105.62	-0.98
C8-N9-CM	128.29	128.76	0.47
C4-N9-CM	125.12	125.62	0.51
N9-C8-N7	113.10	114.18	1.07
N9-C8-C80	122.03	122.51	0.48
N7-C8-C80	124.87	123.32	-1.55
C8-N7-C5	103.71	103.46	-0.25
N7-C5-C6	132.36	132.58	0.21
N7-C5-C4	111.69	110.97	-0.73
C6-C5-C4	115.92	116.46	0.54
C5-C6-N6	121.90	124.62	2.72
C5-C6-N1	119.06	118.71	-0.35
N6-C6-N1	118.96	116.67	-2.28
C6-N6-H61	115.25	117.18	1.93
C6-N6-H62	116.24	121.41	5.17
H61-N6-H62	116.44	121.42	4.97
C6-N1-C2	118.29	119.95	1.66
N1-C2-H2	115.20	117.45	2.24
N1-C2-N3	128.75	125.08	-3.67
H2-C2-N3	116.05	117.48	1.43
C2-N3-C4	110.88	113.87	2.99
N9-C4-C5	104.90	105.78	0.88
N9-C4-N3	128.00	128.28	0.28
C5-C4-N3	127.10	125.94	-1.16
C8-C80-H81	111.62	109.84	-1.79
C8-C80-H82	108.12	109.84	1.72
C8-C80-H83	111.57	109.88	-1.69
H81-C80-H82	108.84	109.64	0.81
H81-C80-H83	107.85	108.81	0.96
H82-C80-H83	108.78	108.81	0.03
C4-N9-C8-N7	0.12	0.00	-0.12
C4-N9-C8-C80	-179.92	180.00	0.08
CM-N9-C8-N7	-179.92	-180.00	-0.08
CM-N9-C8-C80	0.04	-0.00	-0.04
C8-N9-C4-C5	-0.38	-0.00	0.38
C8-N9-C4-N3	179.35	180.00	0.65
CM-N9-C4-C5	179.66	180.00	0.34
CM-N9-C4-N3	-0.61	0.00	0.61
N9-C8-N7-C5	0.19	0.00	-0.19
C80-C8-N7-C5	-179.76	-180.00	-0.24
C8-N7-C5-C6	-178.51	-180.00	-1.49
C8-N7-C5-C4	-0.44	0.00	0.44
N7-C5-C6-N6	-5.02	0.00	5.02
N7-C5-C6-N1	178.38	180.00	1.62
C4-C5-C6-N6	176.99	180.00	3.01
C4-C5-C6-N1	0.38	0.00	-0.38
N7-C5-C4-N9	0.52	0.00	-0.52
N7-C5-C4-N3	-179.21	-180.00	-0.79
C6-C5-C4-N9	178.93	-180.00	1.07
C6-C5-C4-N3	-0.80	0.00	0.80
C5-C6-N6-H61	163.55	-180.00	16.45
C5-C6-N6-H62	22.16	-0.00	-22.16
N1-C6-N6-H61	-19.84	0.00	19.84
N1-C6-N6-H62	-161.23	180.00	18.77
C5-C6-N1-C2	0.07	0.00	-0.07
N6-C6-N1-C2	-176.63	-180.00	-3.37
C6-N1-C2-H2	179.95	-180.00	0.05
C6-N1-C2-N3	-0.24	0.00	0.24
N1-C2-N3-C4	-0.09	-0.00	0.09
H2-C2-N3-C4	179.71	180.00	0.29
C2-N3-C4-N9	-179.04	180.00	0.96
C2-N3-C4-C5	0.64	0.00	-0.64

Table 198: The statistics of vibrational frequencies of 8MA. Only the terms with the occupancies greater than 15% were shown. Refer to p.6 for the meanings of assig.

QM (MP2, scaled by 0.943)							MM						
Freq	Assig.	%	Assig.	%	Assig.	%	Freq	Assig.	%	Assig.	%	Assig.	%
85.1	tCMH3	53.	wN-C	37.			70.2	tC8H3	81.				
107.6	at6r	29.					113.1	tCMH3	92.				
120.3	wC-C80	30.	tC8H3	17.	btf	17.	124.7	wC-N	43.	at6r	29.	t5r'	22.
142.5	tC8H3	64.	btf	26.			144.4	btf	38.				
199.0	wN-C	41.	at6r	21.			220.9	at6r'	36.	t5r'	34.	wC-N	20.
207.6	rC-N	23.	ad6r	17.			243.2	rC-C80	29.	sN-C.ar	24.	rC-N	20.
252.9	rN-C	53.	rC-C80	34.			300.6	at6r	43.	wC-N	21.	wC-C80	20.
275.4	at6r'	53.	wN-C	16.			316.7	rN-C	51.	rC-C80	26.	sN-C.ar	16.
315.1	btf	30.	wC-C80	26.			346.9	rC-N	40.	rC-C80	18.	sN-C.ar	16.
337.3	rC-N	37.	rC-C80	23.			366.1	tC6N	71.	pk6r	15.		
458.9	tC6N	65.	wNH2	17.			420.2	tC6N	25.	pk6r	21.		
476.1	wNH2	32.	tC6N	27.			441.2	sN-C.ar	30.	d5r	20.		
500.5	wNH2	43.	ad6r'	31.			464.3	wN-C	35.	pk6r	32.		
530.1	at6r	35.	t5r'	26.	wC-N	25.	494.0	t5r'	30.	wC-C80	28.	btf	20.
560.6	ad6r	21.	d5r	18.			513.6	sN-C.ar	22.	sC-N	20.		
586.4	rC-N	16.					600.0	ad6r'	27.	d5r	25.		
602.6	wC-N	37.	t5r'	20.			604.2	wNH2	81.				
605.9	d5r	28.	sC-C80	17.			669.2	sN-C.ar	38.				
638.9	t5r	57.	t5r'	28.			693.8	ad6r	35.	sN-C.ar	28.		
660.8	pk6r	82.					697.0	t5r	58.				
726.8	sN-C.ar	28.	sN-C	20.	ad6r	18.	765.6	sN-C.ar	25.	sC-C80	21.	rCH3'	17.
845.1	sN-C.ar	37.	sC-C80	24.			787.1	rCH3	27.				
891.9	td6r	49.	sN-C.ar	19.			853.0	td6r	32.	sN-C.ar	23.	sC-C.ar	16.
907.5	wC-H2	110.					933.8	rCH3'	37.				
1008.9	rCH3'	44.	sN-C.ar	23.	rCH3	15.	950.2	wC-H2	36.	wN-C	28.	rCH3	28.
1017.0	sN-C.ar	38.	rNH2	24.			971.2	rNH2	53.	sN-C.ar	30.		
1052.8	rCH3	65.	rCH3'	20.			1006.8	wC-H2	61.	wN-C	15.		
1076.4	sN-C.ar	32.	rNH2	20.			1036.2	sN-C.ar	35.	rCH3'	30.		
1140.2	rCH3	69.	rCH3'	23.			1048.9	sN-C.ar	22.	rCH3'	18.	d5r'	16.
1168.7	rCH3'	38.					1091.8	rCH3	60.	rCH3'	20.		
1230.5	sN-C.ar	50.					1121.7	sN-C.ar	50.				
1266.1	rNH2	29.	sN-C.ar	28.			1164.1	rC-H	46.	sN-C.ar	28.		
1337.9	sN-C.ar	69.					1167.7	sN-C	18.	sdCH3	17.	sN-C.ar	15.
1355.8	sN-C.ar	51.	rC-H	34.			1235.6	sN-C.ar	67.	td6r	19.		
1401.0	sdCH3	63.	sN-C.ar	21.			1354.9	sN-C.ar	27.	sdCH3	26.		
1409.0	sN-C.ar	51.	rC-H	25.			1394.0	sN-C.ar	33.	adCH3'	17.		
1415.8	sN-C.ar	36.	sdCH3	33.			1397.0	adCH3	71.	adCH3'	23.		
1445.1	sdCH3	69.	sN-C.ar	15.			1403.4	adCH3'	52.	adCH3	17.		
1481.1	adCH3'	39.	sN-C.ar	23.			1430.1	sdCH3	36.	sN-C.ar	20.		
1487.6	sN-C.ar	36.	rC-H	18.			1438.0	adCH3	64.	adCH3'	21.		
1489.9	adCH3	74.	adCH3'	23.			1441.6	adCH3'	44.				
1496.5	sN-C.ar	38.	sdCH3	16.	sC-C80	15.	1488.9	sN-C.ar	45.	sdCH3	18.		
1506.6	adCH3	70.	adCH3'	25.			1561.8	sdCH3	50.	sN-C.ar	19.		
1518.8	adCH3'	56.	adCH3	19.			1574.9	sN-C.ar	44.	rC-H	20.	sC-C.ar	19.
1526.4	adCH3'	28.	sN-C.ar	25.			1591.5	sN-C.ar	36.				
1594.5	cNH2	43.	sN-C.ar	26.	sC-C.ar	21.	1636.5	cNH2	54.	sN-C.ar	17.		
1614.9	sN-C.ar	46.	sC-C.ar	18.	cNH2	16.	1680.1	sN-C.ar	31.	sC-C.ar	20.		
1658.3	sC-C.ar	34.	cNH2	28.	sC-N	18.	1724.5	sC-C.ar	35.	ad6r'	18.		
3006.9	ssCH3	94.					2849.0	ssCH3	100.				
3020.0	ssCH3	98.					2853.9	ssCH3	100.				
3078.4	asCH3	73.	asCH3'	25.			2911.8	asCH3	75.	asCH3'	25.		
3106.1	asCH3	75.	asCH3'	25.			2913.1	asCH3'	75.	asCH3	25.		
3129.0	asCH3'	72.	asCH3	24.			2914.5	asCH3	75.	asCH3'	25.		
3134.3	sC-H	97.					2915.1	asCH3'	75.	asCH3	25.		
3135.0	asCH3'	72.	asCH3	24.			3119.5	sC-H	99.				
3487.1	ssNH2	100.					3445.1	ssNH2	99.				
3613.5	asNH2	100.					3563.8	asNH2	100.				

2.16 7-cyano-7-deaza guanine (DCG)

Figure 77: The energy-minimized structure of DCG.

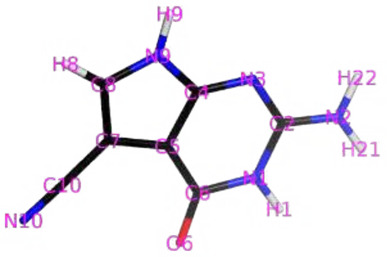

Table 199: The calculated geometric terms of DCG.

Terms	QM	MM	diff
N9-C8	1.373	1.387	0.014
N9-C4	1.368	1.350	-0.018
N9-H9	1.014	1.009	-0.005
C8-H8	1.081	1.083	0.002
C8-C7	1.389	1.370	-0.019
C7-C5	1.427	1.437	0.010
C7-C10	1.421	1.427	0.006
C5-C6	1.440	1.404	-0.036
C5-C4	1.395	1.402	0.006
C6-O6	1.227	1.229	0.002
C6-N1	1.423	1.381	-0.042
N1-H1	1.018	0.997	-0.020
N1-C2	1.374	1.376	0.002
C2-N2	1.384	1.328	-0.056
C2-N3	1.310	1.343	0.034
N2-H21	1.015	0.992	-0.022
N2-H22	1.015	0.995	-0.020
N3-C4	1.366	1.332	-0.035
C10-N10	1.184	1.180	-0.004
C8-N9-C4	110.00	111.08	1.08
C8-N9-H9	126.17	128.44	2.26
C4-N9-H9	123.83	120.49	-3.34
N9-C8-H8	121.93	124.20	2.27
N9-C8-C7	108.23	107.80	-0.43
H8-C8-C7	129.84	128.00	-1.84
C8-C7-C5	106.73	106.76	0.03
C8-C7-C10	125.49	126.08	0.59
C5-C7-C10	127.78	127.16	-0.62
C7-C5-C6	133.70	133.00	-0.70
C7-C5-C4	107.44	107.60	0.16
C6-C5-C4	118.83	119.40	0.58
C5-C6-O6	129.61	129.16	-0.45
C5-C6-N1	109.91	112.72	2.81
O6-C6-N1	120.48	118.12	-2.36
C6-N1-H1	113.86	116.11	2.26
C6-N1-C2	126.30	125.25	-1.05
H1-N1-C2	119.61	118.64	-0.98
N1-C2-N2	115.94	116.75	0.82
N1-C2-N3	123.96	121.67	-2.28
N2-C2-N3	120.01	121.57	1.56
C2-N2-H21	115.56	123.38	7.82
C2-N2-H22	111.18	116.10	4.93
H21-N2-H22	112.24	120.52	8.28
C2-N3-C4	112.38	114.89	2.51
N9-C4-C5	107.60	106.77	-0.83
N9-C4-N3	123.81	127.17	3.37
C5-C4-N3	128.58	126.06	-2.52
C4-N9-C8-H8	-179.83	180.00	0.17
C4-N9-C8-C7	0.10	0.00	-0.10
H9-N9-C8-H8	-0.17	0.00	0.17
H9-N9-C8-C7	179.75	-180.00	0.25
C8-N9-C4-C5	-0.41	0.00	0.41
C8-N9-C4-N3	-179.07	180.00	0.93
H9-N9-C4-C5	179.93	180.00	0.07
H9-N9-C4-N3	1.26	0.00	-1.26
N9-C8-C7-C5	0.25	-0.00	-0.25
N9-C8-C7-C10	179.79	180.00	0.21
H8-C8-C7-C5	-179.84	-180.00	-0.16
H8-C8-C7-C10	-0.30	0.00	0.30
C8-C7-C5-C6	-178.44	-180.00	-1.56
C8-C7-C5-C4	-0.49	-0.00	0.49
C10-C7-C5-C6	2.03	0.00	-2.03
C10-C7-C5-C4	179.98	180.00	0.02
C7-C5-C6-O6	-0.50	0.00	0.50
C7-C5-C6-N1	178.93	-180.00	1.07
C4-C5-C6-O6	-178.26	180.00	1.74
C4-C5-C6-N1	1.16	0.00	-1.16
C7-C5-C4-N9	0.55	-0.00	-0.55
C7-C5-C4-N3	179.13	-180.00	0.87
C6-C5-C4-N9	178.86	180.00	1.14
C6-C5-C4-N3	-2.56	0.00	2.56
C5-C6-N1-H1	-174.42	180.00	5.58
C5-C6-N1-C2	0.06	-0.00	-0.06
O6-C6-N1-H1	5.07	0.00	-5.07
O6-C6-N1-C2	179.55	180.00	0.45
C6-N1-C2-N2	-176.67	-180.00	-3.33
C6-N1-C2-N3	-0.27	0.00	0.27
H1-N1-C2-N2	-2.48	0.00	2.48
H1-N1-C2-N3	173.92	-180.00	6.08
N1-C2-N2-H21	-42.13	0.00	42.13
N1-C2-N2-H22	-171.56	-180.00	-8.44
N3-C2-N2-H21	141.32	180.00	38.68
N3-C2-N2-H22	11.89	0.00	-11.89
N1-C2-N3-C4	-0.76	0.00	0.76
N2-C2-N3-C4	175.50	-180.00	4.50
C2-N3-C4-N9	-179.38	-180.00	-0.62
C2-N3-C4-C5	2.24	0.00	-2.24

Table 200: The statistics of vibrational frequencies of DCG. Only the terms with the occupancies greater than 15% were shown. Refer to p.6 for the meanings of assig.

QM (MP2, scaled by 0.943)							MM						
Freq	Assig.	%	Assig.	%	Assig.	%	Freq	Assig.	%	Assig.	%	Assig.	%
66.6	at6r	47.	wC7C10	41.			70.9	wC7C10	43.	t5r	21.	at6r	21.
110.8	rC7C10	50.	dCCN	30.			142.4	rC7C10	58.	dCCN	23.		
135.5	btfl	57.	pk6r	45.			161.9	at6r	23.				
162.2	at6r'	58.	wC7C10	21.			255.7	t5r	23.	at6r'	19.	btfl	19.
185.8	at6r	42.	at6r'	20.	wC7C10	17.	273.4	wN2-C	26.	at6r'	24.	pk6r	20.
288.6	dCCN	23.	rC=O	18.			324.1	rN2-C	42.	sC-N.ar	35.		
316.5	rN2-C	28.					326.9	t5r'	33.	tC2N2	31.	at6r'	26.
327.3	tC2N2	81.					348.6	rC=O	42.	sC-C.ar	21.		
331.8	rN2-C	23.					368.8	tC2N2	53.	t5r'	26.		
407.3	ad6r	26.	d5r'	18.			414.3	sC-C.ar	26.	ad6r	23.	dCCN	19.
439.7	tC7C10	54.	t5r	16.			427.4	btfl	27.	tC7C10	20.	t5r'	15.
498.8	ad6r'	67.					490.7	sC-N.ar	32.				
509.0	wNH	39.	t5r	25.	tC7C10	19.	513.1	wNH	30.	wNH2	26.	pk6r	20.
525.1	t5r	47.	wNH	31.			547.7	wNH	30.	at6r	21.	tC7C10	18.
534.1	dCCN	27.	rC7C10	26.			573.1	sC-C.ar	18.	sC-N.ar	17.		
582.9	wNH	56.	wN2-C	28.			582.1	ad6r'	27.	dCCN	18.		
593.3	sC-N.ar	19.	t5r'	15.			608.0	wNH	74.				
603.0	t5r'	55.	wNH	16.			613.8	tC7C10	37.	wNH	20.	wC7C10	16.
616.5	sC-C.ar	23.	ad6r	18.			638.2	ad6r	29.				
628.0	pk6r	33.	wC=O	30.			655.6	pk6r	19.	wNH	19.		
657.7	wC=O	35.	sC-C.ar	15.			696.0	rN2-C	19.	rC=O	18.	sC-N.ar	17.
672.4	wC-H	54.					697.9	wNH2	33.	wN2-C	32.	pk6r	26.
685.9	wC-H	47.	wC=O	22.	wNH	18.	712.8	wC-H	78.	wNH	23.		
715.4	wNH2	58.	wN2-C	24.			786.5	d5r	42.				
816.3	d5r	44.	td6r	25.			791.9	wC=O	101.				
1025.9	sC-N.ar	46.	td6r	18.			916.8	sC-N.ar	59.				
1047.7	sC-N.ar	34.					998.1	rNH2	73.	sC-N.ar	19.		
1073.5	sC-N.ar	32.	rNH2	16.			1050.0	sC-N.ar	24.	sC-C.ar	16.		
1107.3	sC-N.ar	36.	rC-H	26.	rN-H	17.	1070.0	sC-C.ar	21.	sC-N.ar	16.		
1148.8	rNH2	36.	sC-N.ar	35.			1111.2	rC-H	33.	sC-N.ar	16.	d5r'	15.
1245.6	rC-H	24.	sC-N.ar	22.	rN-H	17.	1208.6	rN-H	31.	sC-N.ar	21.	rC-H	20.
1265.6	sC-C.ar	39.	sC-N.ar	27.			1248.3	sC-N.ar	38.	rN-H	22.		
1312.7	rN-H	47.	sC2-N2	17.			1330.3	rN-H	44.				
1370.6	sC-N.ar	56.	rN-H	22.			1344.0	sC-N.ar	57.				
1442.3	sC-C.ar	53.	sC-N.ar	15.			1502.2	sC-N.ar	29.	sC-C.ar	27.		
1471.2	sC-N.ar	38.	sC-C.ar	26.			1524.5	sC-C.ar	40.	sC-N.ar	20.		
1519.4	sC-C.ar	35.	sC-N.ar	27.			1567.6	sC-N.ar	29.	rN-H	16.	sC-C.ar	15.
1546.4	sC-C.ar	27.	sC-N.ar	21.			1600.5	sC-N.ar	47.				
1585.3	sC-C.ar	41.	sC-N.ar	31.			1608.9	sC-C.ar	33.	rN-H	17.		
1621.6	cNH2	49.	sC-N.ar	35.			1649.0	cNH2	57.				
1669.5	cNH2	44.	sC-N.ar	32.			1693.0	sC-C.ar	29.	sC-N.ar	19.		
1782.2	sC=O	69.	sC-C.ar	17.			1842.8	sC=O	37.	sC-C.ar	18.		
2140.0	sC#N	83.	sC7-C10	17.			2103.4	sC#N	80.	sC7-C10	18.		
3215.6	sC-H	99.					3188.6	sC-H	99.				
3466.1	ssNH	99.					3441.8	ssNH	90.				
3485.8	sN-H	99.					3457.3	sN-H	90.				
3544.4	sN-H	100.					3506.3	sN-H	100.				
3577.9	asNH2	100.					3560.9	asNH2	99.				

2.17 dihydrouracil (H2U)

Figure 78: The energy-minimized structure of H2U.

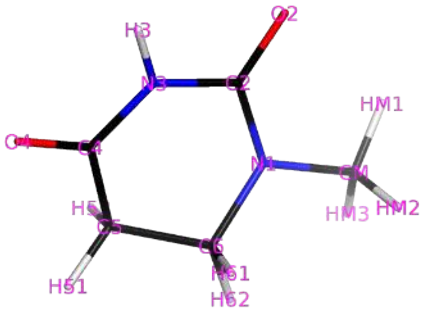

Table 201: The calculated geometric terms of H2U.

Terms	QM	MM	diff
N1-C2	1.374	1.375	0.001
N1-C6	1.458	1.455	-0.003
N1-CM	1.456	1.452	-0.004
C2-O2	1.227	1.224	-0.003
C2-N3	1.408	1.360	-0.048
N3-H3	1.017	0.989	-0.028
N3-C4	1.387	1.324	-0.063
C4-O4	1.225	1.222	-0.002
C4-C5	1.510	1.482	-0.028
C5-H51	1.093	1.111	0.018
C5-H52	1.097	1.112	0.015
C5-C6	1.520	1.524	0.004
C6-H61	1.101	1.114	0.012
C6-H62	1.094	1.114	0.020
C2-N1-C6	118.70	119.34	0.64
C2-N1-CM	116.94	119.90	2.96
C6-N1-CM	117.36	118.40	1.04
N1-C2-O2	125.30	125.29	-0.01
N1-C2-N3	114.30	114.99	0.69
O2-C2-N3	120.39	119.71	-0.68
C2-N3-H3	114.27	116.39	2.12
C2-N3-C4	128.42	127.11	-1.31
H3-N3-C4	116.59	116.51	-0.08
N3-C4-O4	121.84	120.82	-1.03
N3-C4-C5	113.29	118.29	5.00
O4-C4-C5	124.85	120.89	-3.96
C4-C5-H51	108.14	109.86	1.72
C4-C5-H52	107.96	108.88	0.92
C4-C5-C6	110.85	106.87	-3.98
H51-C5-H52	108.04	109.23	1.19
H51-C5-C6	112.03	111.54	-0.50
H52-C5-C6	109.68	110.41	0.73
N1-C6-C5	109.62	110.00	0.38
N1-C6-H61	111.01	109.42	-1.60
N1-C6-H62	107.76	109.11	1.36

Terms	QM	MM	diff
C5-C6-H61	110.56	111.98	1.42
C5-C6-H62	110.31	108.82	-1.49
H61-C6-H62	107.52	107.43	-0.09
C6-N1-C2-O2	161.68	166.29	4.61
C6-N1-C2-N3	-19.17	-14.60	4.57
CM-N1-C2-O2	11.69	4.02	-7.67
CM-N1-C2-N3	-169.16	-176.88	-7.72
C2-N1-C6-C5	51.48	46.72	-4.76
C2-N1-C6-H61	-70.96	-76.70	-5.74
C2-N1-C6-H62	171.55	166.03	-5.53
CM-N1-C6-C5	-158.66	-150.74	7.92
CM-N1-C6-H61	78.90	85.84	6.94
CM-N1-C6-H62	-38.58	-31.43	7.15
N1-C2-N3-H3	179.41	170.90	-8.51
N1-C2-N3-C4	-10.77	-9.25	1.52
O2-C2-N3-H3	-1.39	-9.94	-8.55
O2-C2-N3-C4	168.43	169.91	1.48
C2-N3-C4-O4	-174.62	177.07	8.30
C2-N3-C4-C5	4.25	-3.00	-7.24
H3-N3-C4-O4	-5.01	-3.07	1.93
H3-N3-C4-C5	173.86	176.86	2.99
N3-C4-C5-H51	152.01	155.49	3.48
N3-C4-C5-H52	-91.33	-84.94	6.39
N3-C4-C5-C6	28.83	34.33	5.49
O4-C4-C5-H51	-29.16	-24.59	4.58
O4-C4-C5-H52	87.50	94.99	7.49
O4-C4-C5-C6	-152.34	-145.75	6.59
C4-C5-C6-N1	-54.54	-53.29	1.25
C4-C5-C6-H61	68.17	68.61	0.44
C4-C5-C6-H62	-173.04	-172.78	0.26
H51-C5-C6-N1	-175.44	-173.38	2.06
H51-C5-C6-H61	-52.73	-51.48	1.25
H51-C5-C6-H62	66.06	67.13	1.07
H52-C5-C6-N1	64.59	64.97	0.39
H52-C5-C6-H61	-172.71	-173.12	-0.42
H52-C5-C6-H62	-53.92	-54.52	-0.60

Figure 79: The PES scan for flexible dihedral corresponding to H2U.

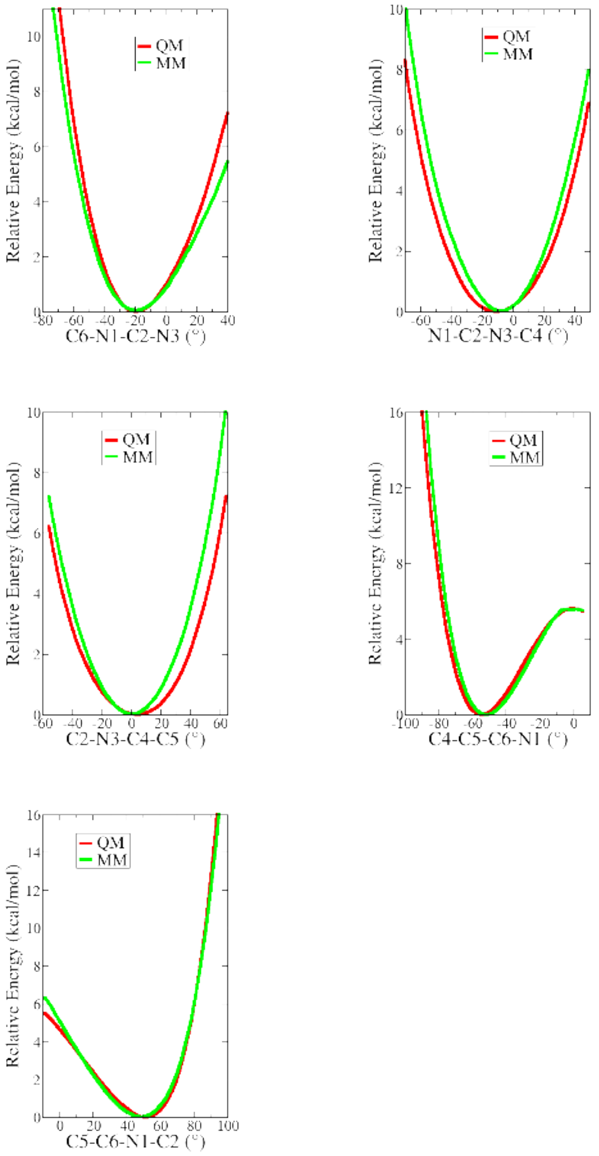

Table 202: The statistics of vibrational frequencies of H2U. Only the terms with the occupancies greater than 15% were shown. Refer to p.6 for the meanings of assig.

QM (MP2, scaled by 0.943)							MM						
Freq	Assig.	%	Assig.	%	Assig.	%	Freq	Assig.	%	Assig.	%	Assig.	%
79.9	at6r	98.					71.0	at6r	108.				
130.9	at6r'	50.	pk6r	27.	tCNCH	22.	118.8	wN-CM	33.	tCNCH	33.	at6r'	27.
150.4	tCNCH	66.	at6r'	25.			156.1	pk6r	59.				
176.9	wN-CM	63.	pk6r	38.			193.8	at6r'	36.	tCNCH	34.	pk6r	19.
256.3	at6r'	31.	wN-CM	23.	pk6r	18.	295.0	wN-CM	42.	rN-CM	18.		
337.9	rN-CM	63.					347.8	rN-CM	47.	rC=O2	24.		
370.7	rC=O2	27.	rC=O4	26.	ad6r	19.	363.3	rC=O2	26.	rC=O4	20.	sN-C2	15.
424.2	ad6r	52.					404.5	ad6r	57.				
467.1	ad6r'	38.	sN-C2	16.			461.2	ad6r'	71.				
542.6	rC=O2	23.	rC=O4	23.			536.3	rC=O4	39.	rC=O2	20.		
592.5	wC=O4	26.					604.3	td6r	26.	rC5H2	17.		
642.2	wN-H	90.					647.7	wN-H	35.				
701.7	sN-C2	21.	td6r	17.			688.2	wC=O2	44.				
720.2	wC=O2	81.					716.2	wC=O2	22.	td6r	16.	sN-CM	16.
750.5	wC=O2	18.	td6r	16.			770.0	wC=O2	25.	wN-H	20.	rC5H2	19.
830.3	sC-C.ar	26.					845.4	wC=O4	31.	wN-H	23.		
944.3	sN-C2	24.	rC5H2	19.			869.3	rC6H2	29.	sN-C2	17.		
985.3	sC-C.al	21.					948.9	rC6H2	22.	rC5H2	21.		
1051.5	rC5H2	23.	rC6H2	20.	rCH3	19.	1021.8	sC-C.al	27.	rCH3'	24.		
1076.1	sC-C.al	27.	rC6H2	19.			1038.0	rCH3'	58.				
1138.8	rCH3'	77.					1040.6	rCH3	35.	sN-C6	18.		
1169.9	sN-CM	33.	iC6H2	18.			1086.7	sN-CM	34.	sN-C2	18.		
1197.8	iC5H2	58.					1156.9	iC5H2	78.				
1236.2	sN-C2	34.	iC6H2	30.			1208.5	rCH3	29.	wC6H2	21.	sN-C6	15.
1269.9	sN-C6	27.	sN-CM	18.			1228.9	iC6H2	72.				
1298.3	wC5H2	25.	sN-C2	15.			1259.1	wC5H2	70.				
1342.5	wC5H2	32.	iC6H2	24.			1325.1	rN-H	61.				
1367.0	rN-H	33.	wC6H2	20.			1378.7	cC6H2	40.	wC6H2	28.		
1397.2	sN-C2	28.	rN-H	27.			1408.4	sN-C2	30.	sC-C.ar	22.		
1431.1	sdCH3	52.	sN-C2	17.			1436.3	asCH3	48.	asCH3'	32.		
1453.9	cC5H2	43.	sdCH3	26.			1438.6	cC5H2	81.				
1459.5	cC5H2	53.					1446.5	asCH3'	45.	sdCH3	17.		
1492.4	asCH3'	63.	asCH3	16.			1496.9	sdCH3	38.	asCH3	20.		
1502.6	asCH3	40.	cC6H2	31.	asCH3'	21.	1538.0	cC6H2	37.	wC6H2	27.	sN-C6	17.
1522.3	cC6H2	59.	asCH3	18.			1596.8	sN-C2	46.	sC=O	22.		
1725.6	sC=O	83.					1704.3	sC=O	50.	sN-C2	27.		
1733.1	sC=O	69.					1818.7	sC=O	42.	sN-C2	35.		
2955.1	ssC-H	73.	saC-H	27.			2842.8	ssCH3	100.				
2987.0	ssCH3	81.					2858.8	ssC-H	98.				
3011.4	ssC-H	89.					2892.0	saC-H	97.				
3059.4	saC-H	72.	ssC-H	28.			2916.6	saCH3'	71.	saCH3	28.		
3079.4	saCH3'	85.					2918.2	saCH3	71.	saCH3'	28.		
3084.7	saC-H	90.					2919.7	ssC-H	98.				
3141.7	saCH3	91.					2952.2	saC-H	100.				
3482.5	sN-H	100.					3335.3	sN-H	99.				

2.18 N6,N6-dimethyl adenine (M6A)

Figure 80: The energy-minimized structure of M6A.

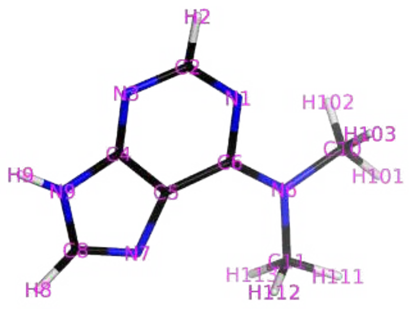

Table 203: The calculated geometric terms of M6A.

Terms	QM	MM	diff
N9-C8	1.367	1.369	0.002
N9-C4	1.377	1.349	-0.028
N9-H9	1.013	1.002	-0.011
C8-H8	1.083	1.093	0.010
C8-N7	1.325	1.323	-0.002
N7-C5	1.386	1.406	0.019
C5-C6	1.424	1.419	-0.005
C5-C4	1.405	1.400	-0.005
C6-N6	1.363	1.387	0.024
C6-N1	1.350	1.384	0.034
N6-C10	1.457	1.462	0.006
N6-C11	1.458	1.460	0.002
N1-C2	1.347	1.358	0.011
C2-H2	1.089	1.093	0.004
C2-N3	1.337	1.357	0.020
N3-C4	1.343	1.325	-0.018
C10-H101	1.093	1.112	0.019
C10-H102	1.088	1.114	0.026
C10-H103	1.098	1.114	0.016
C11-H111	1.093	1.113	0.019
C11-H112	1.099	1.114	0.015
C11-H113	1.087	1.114	0.027
C8-N9-C4	107.04	107.36	0.32
C8-N9-H9	127.79	130.82	3.02
C4-N9-H9	125.16	121.82	-3.34
N9-C8-H8	122.10	122.57	0.47
N9-C8-N7	113.26	112.90	-0.36
H8-C8-N7	124.64	124.53	-0.11
C8-N7-C5	104.12	104.08	-0.04
N7-C5-C6	133.85	134.51	0.67
N7-C5-C4	110.71	109.28	-1.43
C6-C5-C4	115.44	116.21	0.77
C5-C6-N6	125.07	126.33	1.25
C5-C6-N1	117.50	116.81	-0.69
N6-C6-N1	117.37	116.77	-0.59
C6-N6-C10	118.39	120.62	2.23
C6-N6-C11	121.43	121.31	-0.12
C10-N6-C11	115.24	117.83	2.59
C6-N1-C2	119.82	120.86	1.04
N1-C2-H2	115.18	117.45	2.27
N1-C2-N3	128.73	124.95	-3.78
H2-C2-N3	116.09	117.59	1.51
C2-N3-C4	110.23	113.36	3.13
N9-C4-C5	104.87	106.38	1.51
N9-C4-N3	126.84	125.85	-0.99
C5-C4-N3	128.28	127.77	-0.51
N6-C10-H101	107.78	111.49	3.71
N6-C10-H102	109.71	111.45	1.74
N6-C10-H103	111.79	111.11	-0.67
H101-C10-H102	110.01	107.08	-2.93
H101-C10-H103	109.03	107.58	-1.44
H102-C10-H103	108.51	107.92	-0.60
N6-C11-H111	108.56	111.49	2.94
N6-C11-H112	110.97	111.68	0.72
N6-C11-H113	110.19	111.07	0.88
H111-C11-H112	108.45	107.08	-1.37
H111-C11-H113	109.41	107.44	-1.97
H112-C11-H113	109.23	107.86	-1.37
C4-N9-C8-H8	179.99	-179.91	0.10
C4-N9-C8-N7	0.02	0.06	0.04
H9-N9-C8-H8	-0.30	-0.49	-0.19
H9-N9-C8-N7	179.73	179.48	-0.25
C8-N9-C4-C5	-0.05	-0.08	-0.03
C8-N9-C4-N3	179.08	179.32	0.24
H9-N9-C4-C5	-179.77	-179.56	0.20
H9-N9-C4-N3	-0.64	-0.16	0.48
N9-C8-N7-C5	0.02	-0.01	-0.03
H8-C8-N7-C5	-179.95	179.96	0.09
C8-N7-C5-C6	-179.43	-179.87	-0.44
C8-N7-C5-C4	-0.05	-0.04	0.01
N7-C5-C6-N6	2.23	1.57	-0.66
N7-C5-C6-N1	179.20	177.91	-1.29
C4-C5-C6-N6	-177.13	-178.25	-1.12
C4-C5-C6-N1	-0.15	-1.91	-1.76
N7-C5-C4-N9	0.06	0.07	0.02
N7-C5-C4-N3	-179.05	-179.31	-0.26
C6-C5-C4-N9	179.56	179.94	0.38
C6-C5-C4-N3	0.45	0.55	0.10
C5-C6-N6-C10	-175.96	167.96	16.08
C5-C6-N6-C11	-22.04	-17.81	4.23
N1-C6-N6-C10	7.06	-8.38	-15.44
N1-C6-N6-C11	160.98	165.85	4.87
C5-C6-N1-C2	-0.35	2.31	2.66
N6-C6-N1-C2	176.86	179.01	2.15
C6-N1-C2-H2	-179.76	179.28	0.96
C6-N1-C2-N3	0.72	-1.30	-2.02
N1-C2-N3-C4	-0.43	-0.15	0.28
H2-C2-N3-C4	-179.95	179.26	0.79
C2-N3-C4-N9	-179.10	-178.76	0.34
C2-N3-C4-C5	-0.18	0.51	0.69

Figure 81: The PES scan for flexible dihedral corresponding to M6A.

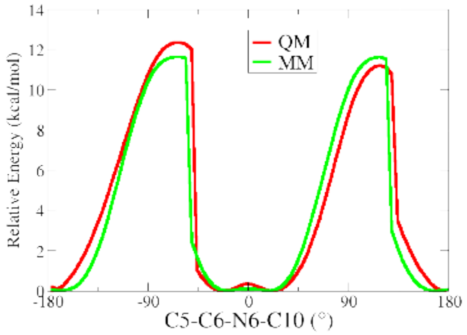

Table 204: The statistics of vibrational frequencies of M6A. Only the terms with the occupancies greater than 15% were shown. Refer to p.6 for the meanings of assig.

QM (MP2, scaled by 0.943)							MM						
Freq	Assig.	%	Assig.	%	Assig.	%	Freq	Assig.	%	Assig.	%	Assig.	%
69.3	tC6N	80.					52.9	tC6N	90.				
94.0	at6r	24.	wNCH3	21.	atN6C	20.	82.6	wNCH3	79.				
127.9	stN6C	46.	atN6C	29.			145.0	atN6C	64.				
152.2	atN6C	39.	stN6C	32.	at6r	20.	149.0	stN6C	93.				
208.6	btf	38.	wNCH3	33.			203.8	at6r	37.	atN6C	20.		
230.6	wNCH3	24.	btf	20.	at6r’	15.	225.0	btf	33.	rC4N4	20.		
237.6	rC4N4	34.	stN6C	17.			241.8	rC4N4	33.				
291.9	at6r’	45.	btf	17.			311.2	at6r’	43.				
366.5	rNCH3	50.					352.7	cNCH3	42.	sC6-N6	18.		
379.0	cNCH3	48.					376.1	rNCH3	49.				
487.4	wN-H	57.	t5r’	19.	at6r	16.	429.6	pk6r	63.	at6r	16.		
509.0	ad6r’	54.					494.7	sN-C.ar	45.	ad6r’	21.		
518.4	ad6r	34.					498.0	wN-H	53.	t5r’	28.		
540.5	wN-H	28.	wC4N4	24.	at6r	20.	535.0	ad6r	24.	cNCH3	22.	sN-C.ar	18.
611.9	wC4N4	41.	t5r’	17.	t5r	17.	547.3	wC4N4	42.	t5r	25.		
620.1	d5r’	18.	sN-C.ar	18.			583.7	sN-C.ar	42.				
644.0	t5r	60.	t5r’	33.			658.8	sN-C.ar	28.	ad6r’	19.	d5r’	16.
656.4	pk6r	74.					678.2	t5r’	35.	wN-H	31.	btf	21.
680.1	sN-C.ar	39.	d5r	16.			757.5	t5r	54.	wC4N4	15.		
783.6	wC-H	106.					852.7	td6r	29.	ad6r	24.	sN-C.ar	21.
853.6	td6r	25.	sN-C.ar	19.	ad6r	15.	859.2	d5r	41.	sN-C.ar	22.	sC-C.ar	16.
908.7	d5r’	34.	d5r	18.	td6r	16.	905.4	wC-H	107.				
916.5	wCH	110.					935.2	ssNCH3	39.	sN-C.ar	19.		
939.6	ssNCH3	37.	d5r	24.			985.8	wCH	97.				
1078.9	rCH3	30.	asNCH3	23.	rCH3’	18.	1028.9	sN-C.ar	71.				
1084.7	sN-C.ar	35.					1038.8	sN-C.ar	23.	rCH3	20.		
1101.4	sN-C.ar	38.	rN-H	29.			1048.2	rCH3’	39.	rCH3’	39.		
1121.1	rCH3’	34.	rCH3’	21.	rCH3	20.	1060.4	rCH3	46.	rCH3	28.		
1145.7	sN-C.ar	68.					1081.9	sN-C.ar	56.	rN-H	15.		
1164.8	rCH3	26.	rCH3’	25.	rCH3’	22.	1098.8	rCH3’	37.	rCH3’	36.		
1213.5	sN-C.ar	21.	rC-H	20.	td6r	17.	1106.1	sN-C.ar	41.	rCH	26.		
1260.0	sN-C.ar	48.	rC-H	23.			1186.5	sN-C.ar	32.	rC-H	24.	rCH	18.
1280.0	asNCH3	46.					1224.8	sN-C.ar	42.	td6r	28.		
1325.4	sN-C.ar	69.					1252.1	sN-C.ar	31.				
1371.3	sN-C.ar	47.					1321.2	sN-C.ar	42.				
1390.7	sN-C.ar	75.					1389.9	asNCH3	34.	adCH3	19.		
1412.9	sN-C.ar	39.	rCH	24.	rN-H	21.	1418.3	adCH3	38.	adCH3	22.		
1440.6	sdCH3	78.					1424.2	adCH3	32.	adCH3	19.		
1443.6	sN-C.ar	34.	sC-C.ar	23.			1428.6	adCH3’	56.				
1463.4	sdCH3	79.					1438.7	adCH3’	61.	adCH3’	18.		
1477.2	sN-C.ar	57.	rC-H	25.			1472.9	sdCH3	50.	sdCH3	38.		
1495.1	adCH3’	82.					1480.0	sN-C.ar	29.	asNCH3	17.		
1503.3	adCH3	36.	adCH3’	18.			1497.1	sdCH3	40.	sdCH3	39.		
1515.3	adCH3	55.					1529.6	sC6-N6	25.	sN-C.ar	18.		
1518.3	adCH3’	53.	adCH3	21.	adCH3	17.	1565.6	sN-C.ar	48.	rC-H	17.	sC-C.ar	16.
1545.9	sN-C.ar	17.					1594.9	sN-C.ar	48.	rC-H	21.	rCH	16.
1602.7	sN-C.ar	54.	sC-C.ar	18.			1659.6	sN-C.ar	41.	sC-C.ar	22.		
1625.7	sC-C.ar	32.	sC6-N6	22.	sN-C.ar	20.	1716.1	sC-C.ar	37.	ad6r’	19.		
2979.8	ssCH3	77.	asCH3’	19.			2953.6	sC-H	99.				
2991.3	ssCH3	79.	asCH3’	17.			2977.1	ssCH3	71.	ssCH3	28.		
3068.5	asCH3	65.	asCH3’	22.			2977.4	ssCH3	71.	ssCH3	28.		
3080.6	asCH3	58.	asCH3’	29.			3019.2	asCH3	50.	asCH3	49.		
3129.2	sC-H	100.					3021.8	asCH3’	69.				
3151.5	asCH3’	53.	asCH3	40.			3023.0	asCH3	33.	asCH3	31.	asCH3’	28.
3156.5	asCH3’	59.	asCH3	33.			3023.0	asCH3’	81.				
3202.2	sC-H	99.					3118.6	sC-H	99.				
3548.4	sN-H	100.					3505.7	sN-H	100.				

2.19 4-demethylwyosine (DWG)

Figure 82: The energy-minimized structure of DWG.

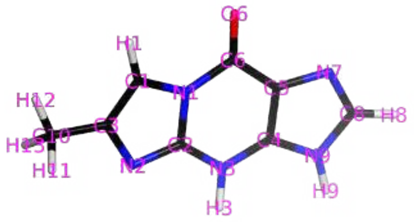

Table 205: The calculated geometric terms of DWG.

Terms	QM	MM	diff
N9-C8	1.385	1.369	-0.017
N9-C4	1.367	1.361	-0.006
N9-H9	1.013	1.003	-0.010
C8-H8	1.082	1.092	0.010
C8-N7	1.316	1.318	0.002
N7-C5	1.383	1.394	0.011
C5-C6	1.447	1.425	-0.023
C5-C4	1.392	1.394	0.003
C6-O6	1.222	1.239	0.018
C6-N1	1.436	1.433	-0.003
N1-C2	1.383	1.403	0.020
N1-C1	1.389	1.381	-0.008
C2-N2	1.314	1.339	0.025
C2-N3	1.387	1.370	-0.017
N2-C3	1.393	1.385	-0.008
N3-H3	1.014	1.005	-0.009
N3-C4	1.368	1.376	0.007
C1-H1	1.079	1.083	0.004
C1-C3	1.375	1.362	-0.013
C3-C10	1.492	1.481	-0.011
C10-H11	1.094	1.109	0.015
C10-H12	1.093	1.109	0.016
C10-H13	1.094	1.109	0.015
C8-N9-C4	106.34	106.87	0.53
C8-N9-H9	126.56	126.71	0.15
C4-N9-H9	127.10	126.40	-0.70
N9-C8-H8	121.42	123.05	1.63
N9-C8-N7	112.69	112.35	-0.34
H8-C8-N7	125.89	124.60	-1.29
C8-N7-C5	104.64	105.41	0.76
N7-C5-C6	127.64	131.09	3.45
N7-C5-C4	110.50	108.74	-1.75
C6-C5-C4	121.84	120.16	-1.68
C5-C6-O6	129.71	125.61	-4.10
C5-C6-N1	109.40	112.80	3.40
O6-C6-N1	120.88	121.57	0.69
C6-N1-C2	127.89	125.27	-2.63
C6-N1-C1	126.55	129.43	2.88
C2-N1-C1	105.43	105.22	-0.21
N1-C2-N2	113.56	111.88	-1.68
N1-C2-N3	119.40	119.46	0.07
N2-C2-N3	127.01	128.64	1.62
C2-N2-C3	104.02	104.44	0.43
C2-N3-H3	116.54	115.19	-1.35
C2-N3-C4	115.67	116.77	1.09
H3-N3-C4	123.26	124.86	1.60
N9-C4-C5	105.81	106.64	0.82
N9-C4-N3	128.69	128.17	-0.52
C5-C4-N3	125.25	125.18	-0.07
N1-C1-H1	120.66	123.35	2.69
N1-C1-C3	105.90	107.23	1.33
H1-C1-C3	133.44	129.42	-4.02
N2-C3-C1	111.09	111.22	0.13
N2-C3-C10	120.35	118.44	-1.91
C1-C3-C10	128.56	130.34	1.78
C3-C10-H11	110.73	109.42	-1.31
C3-C10-H12	110.60	110.29	-0.31
C3-C10-H13	110.76	109.40	-1.36
H11-C10-H12	108.52	109.33	0.80
H11-C10-H13	107.62	109.07	1.45
H12-C10-H13	108.52	109.31	0.79
C4-N9-C8-H8	178.78	-179.91	1.31
C4-N9-C8-N7	-0.77	-0.06	0.71
H9-N9-C8-H8	-1.05	-1.51	-0.47
H9-N9-C8-N7	179.41	178.34	-1.07
C8-N9-C4-C5	1.20	0.13	-1.08
C8-N9-C4-N3	175.66	178.67	3.01
H9-N9-C4-C5	-178.98	-178.28	0.70
H9-N9-C4-N3	-4.52	0.27	4.79
N9-C8-N7-C5	-0.03	-0.03	-0.01
H8-C8-N7-C5	-179.55	179.81	0.64
C8-N7-C5-C6	179.14	179.48	0.34
C8-N7-C5-C4	0.82	0.11	-0.70
N7-C5-C6-O6	0.10	1.69	1.59
N7-C5-C6-N1	179.78	-176.76	3.46
C4-C5-C6-O6	178.25	-179.00	2.74
C4-C5-C6-N1	-2.06	2.55	4.61
N7-C5-C4-N9	-1.28	-0.15	1.14
N7-C5-C4-N3	-175.99	-178.75	-2.76
C6-C5-C4-N9	-179.72	-179.60	0.12
C6-C5-C4-N3	5.57	1.80	-3.77
C5-C6-N1-C2	2.34	-2.38	-4.71
C5-C6-N1-C1	177.71	173.82	-3.89
O6-C6-N1-C2	-177.95	179.10	2.95
O6-C6-N1-C1	-2.58	-4.70	-2.12
C6-N1-C2-N2	176.34	176.41	0.07
C6-N1-C2-N3	-5.56	-2.19	3.37
C1-N1-C2-N2	0.20	-0.55	-0.74
C1-N1-C2-N3	178.30	-179.15	2.56
C6-N1-C1-H1	3.53	3.34	-0.19
C6-N1-C1-C3	-176.34	-176.59	-0.26
C2-N1-C1-H1	179.74	-179.88	0.38
C2-N1-C1-C3	-0.13	0.19	0.31
N1-C2-N2-C3	-0.17	0.66	0.83
N3-C2-N2-C3	-178.10	179.10	2.80
N1-C2-N3-H3	164.84	167.22	2.38
N1-C2-N3-C4	7.87	6.37	-1.49

Terms	QM	MM	diff
N2-C2-N3-H3	-17.35	-11.12	6.23
N2-C2-N3-C4	-174.31	-171.96	2.35
C2-N2-C3-C1	0.09	-0.54	-0.62
C2-N2-C3-C10	-179.93	179.83	0.24
C2-N3-C4-N9	178.25	175.26	-2.99
C2-N3-C4-C5	-8.28	-6.45	1.83
H3-N3-C4-N9	22.99	16.47	-6.52
H3-N3-C4-C5	-163.54	-165.23	-1.69
N1-C1-C3-N2	0.03	0.21	0.19
N1-C1-C3-C10	-179.95	179.79	0.26
H1-C1-C3-N2	-179.82	-179.72	0.10
H1-C1-C3-C10	0.21	-0.14	-0.34

Table 206: The statistics of vibrational frequencies of DWG. Only the terms with the occupancies greater than 15% were shown. Refer to p.6 for the meanings of assig.

QM (MP2, scaled by 0.943)							MM						
Freq	Assig.	%	Assig.	%	Assig.	%	Freq	Assig.	%	Assig.	%	Assig.	%
67.9	at6r'	35.	btfl2	29.	at6r	24.	40.7	at6r'	51.	btfl2	31.	at6r	19.
113.0	tCCH3	77.					129.8	tCCH3	71.				
134.3	pk6r	40.	at6r'	24.	tCCH3	17.	168.1	at6r'	23.	tCCH3	22.	wC3C10	21.
162.0	at6r	54.	btfl1	22.	at6r'	15.	186.1	at6r	42.				
198.3	wC3C10	32.	btfl1	29.			201.6	rC3C10	34.	sC-N.r	20.		
205.7	rC3C10	39.					252.3	pk6r	33.	t5r	24.		
263.7	btfl1	30.	wN3H	28.	wC3C10	16.	265.2	wN3H	54.	wC3C10	20.		
303.1	rC=O	32.					299.2	btfl1	54.	pk6r	16.		
330.9	btfl2	32.	pk6r	30.			332.9	sC-N.r	22.	ad6r'	19.	rC=O	16.
340.6	wN3H	60.					371.4	sC-C.r	26.	rC=O	24.		
373.8	rC3C10	31.	sC6-N1	20.	dr3	15.	373.7	btfl2	28.	wN3H	28.		
404.1	ad6r'	26.	sC-N.r	24.			402.7	rC3C10	33.				
412.1	wN9H	87.					455.7	wN9H	69.	t5r'	19.		
484.3	ad6r	35.	ad6r'	15.			530.4	ad6r	53.	sC-N.r	24.		
565.3	t5r	30.	tr3'	19.	t5r'	19.	540.9	tr3'	33.	ad6r'	18.		
577.8							550.5	tr3'	31.	ad6r'	20.		
586.2	tr3'	49.	t5r'	16.			585.0	sC3-C10	27.	sC-N.r	24.	dr3'	16.
614.5	tr3	52.					597.0	tr3	67.	wC1H	25.		
634.0	t5r	52.	t5r'	37.			624.5	t5r'	42.	wN9H	16.		
653.7	wC=O	74.	wC1H	16.			675.1	wC=O	48.				
675.8	wC1H	78.					678.2	sC-N.r	16.	td6r	15.		
689.4	dr3'	28.	sC3-C10	24.			702.5	t5r	61.	pk6r	22.		
741.3	wC-H	100.					709.0	sC-N.r	40.	td6r	19.	dr3	18.
744.5	dr3	23.	d5r'	22.			727.0	wC1H	62.				
789.2	td6r	38.	sC-N.r	23.			835.1	d5r	29.	d5r'	24.	sC-N.r	21.
909.0	d5r	50.	d5r'	30.			877.4	dr3'	32.	sC-N.r	32.		
955.7	sC-N.r	44.	rC=O	19.			898.0	sC-N.r	49.	rC=O	18.		
977.7	sC-N.r	40.	dr3'	25.			901.7	wC-H	107.				
1005.4	rCH3'	47.	CH3	16.			1020.9	sC-N.r	43.				
1058.5	CH3	65.	rCH3'	21.			1041.6	sC-N.r	53.				
1080.6	sC-N.r	57.	rN-H	29.			1052.8	rCH3'	24.	rC-H	23.	sC-N.r	19.
1131.2	rC-H	28.	sC-N.r	23.			1080.9	sC-N.r	35.	rCH3'	19.		
1160.5	sC-N.r	44.	rC-H	21.			1085.4	CH3	63.	rCH3'	19.		
1199.8	sC-N.r	37.	rC-H	26.			1130.5	rN-H	29.	sC-N.r	28.		
1251.3	rN-H	44.	sC-N.r	30.			1143.0	rN-H	28.	rC-H	23.	sC-N.r	17.
1295.1	sC-N.r	43.	rC-H	26.	rN-H	15.	1214.6	sC-N.r	37.				
1306.8	sC-N.r	40.					1222.4	sC-N.r	34.	rC-H	23.	rN-H	17.
1365.9	sC-N.r	65.	sC-C.r	15.			1326.9	sC-N.r	60.				
1372.2	sC-N.r	46.					1369.0	rN-H	43.	sC-N.r	22.		
1387.5	rN-H	42.	sC-N.r	37.			1428.6	adCH3'	62.	adCH3	20.		
1424.3	sdCH3	91.					1438.1	sC-N.r	27.	sC-C.r	18.	rN-H	17.
1442.2	sC-N.r	57.	rN-H	19.			1440.1	adCH3	65.	adCH3'	20.		
1475.1	sC-N.r	70.	rC-H	20.			1479.7	sC-N.r	37.	sC-C.r	19.	sdCH3	18.
1490.0	adCH3	70.	adCH3'	26.			1499.7	sdCH3	40.	sC-N.r	25.		
1506.6	adCH3'	62.	adCH3	22.			1536.2	sC-C.r	34.	sC-N.r	22.		
1545.7	sC-N.r	40.	sC-C.r	29.			1573.5	sC-N.r	32.				
1579.7	sC-C.r	52.	sC3-C10	16.			1580.1	sC-N.r	37.	rC-H	33.		
1590.7	sC-N.r	40.	sC-C.r	28.			1619.0	sC-C.r	27.	sC-N.r	23.		
1648.9	sC-N.r	39.	sN3-C4	30.			1699.2	sC-N.r	36.				
1772.8	sC=O	77.					1806.3	sC=O	45.	sC-C.r	21.		
3011.3	ssCH3	100.					2848.4	ssCH3	100.				
3091.3	asCH3	75.	asCH3'	25.			2915.1	asCH3	73.	asCH3'	27.		
3105.0	asCH3'	75.	asCH3	25.			2915.4	asCH3'	73.	asCH3	26.		
3211.3	sC-H	99.					2952.6	sC-H	99.				
3239.9	sC-H	99.					3141.0	sC-H	99.				
3523.8	sN-H	100.					3455.9	sN-H	100.				
3544.0	sN-H	100.					3496.2	sN-H	100.				

2.20 5-hydroxymethyl cytosine (HMC)

Figure 83: The energy-minimized structure of HMC.

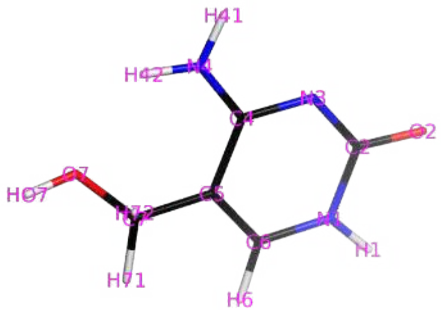

Table 207: The calculated geometric terms of HMC.

Terms	QM	MM	diff
N1-C2	1.419	1.385	-0.034
N1-C6	1.357	1.364	0.007
N1-H1	1.015	1.005	-0.009
C2-O2	1.226	1.224	-0.002
C2-N3	1.381	1.349	-0.032
N3-C4	1.318	1.343	0.025
C4-N4	1.370	1.336	-0.034
C4-C5	1.445	1.447	0.002
N4-H41	1.015	0.995	-0.020
N4-H42	1.015	0.996	-0.019
C5-C6	1.361	1.371	0.009
C5-C7	1.491	1.498	0.006
C6-H6	1.087	1.093	0.006
C7-H71	1.099	1.115	0.016
C7-H72	1.100	1.115	0.015
C7-O7	1.440	1.425	-0.015
O7-HO7	0.974	0.964	-0.010
C2-N1-C6	124.00	121.43	-2.57
C2-N1-H1	114.68	111.92	-2.76
C6-N1-H1	121.28	126.65	5.37
N1-C2-O2	118.82	117.11	-1.71
N1-C2-N3	115.81	119.32	3.50
O2-C2-N3	125.36	123.57	-1.78
C2-N3-C4	120.01	120.76	0.76
N3-C4-N4	117.79	117.79	-0.00
N3-C4-C5	124.70	121.30	-3.40
N4-C4-C5	117.46	120.91	3.45
C4-N4-H41	113.33	116.59	3.26
C4-N4-H42	115.14	120.49	5.34
H41-N4-H42	116.18	121.52	5.34
C4-C5-C6	115.33	116.44	1.11
C4-C5-C7	121.96	121.35	-0.61
C6-C5-C7	122.65	122.18	-0.47
N1-C6-C5	120.00	120.70	0.71
N1-C6-H6	116.91	118.06	1.15
C5-C6-H6	123.09	121.23	-1.86
C5-C7-H71	109.58	108.77	-0.81
C5-C7-H72	109.88	108.51	-1.37
C5-C7-O7	108.46	110.84	2.38
H71-C7-H72	108.08	108.66	0.58
H71-C7-O7	110.87	109.27	-1.60
H72-C7-O7	109.95	110.73	0.78
C7-O7-HO7	107.38	108.31	0.93
C6-N1-C2-O2	177.13	178.61	1.48
C6-N1-C2-N3	-4.02	-1.28	2.74
H1-N1-C2-O2	-0.62	-0.73	-0.11
H1-N1-C2-N3	178.24	179.38	1.15
C2-N1-C6-C5	1.54	0.56	-0.98
C2-N1-C6-H6	-177.70	-178.87	-1.17
H1-N1-C6-C5	179.15	179.80	0.65
H1-N1-C6-H6	-0.09	0.36	0.46
N1-C2-N3-C4	2.82	0.01	-2.82
O2-C2-N3-C4	-178.41	-179.87	-1.46
C2-N3-C4-N4	177.88	-177.52	4.59
C2-N3-C4-C5	0.60	1.91	1.31
N3-C4-N4-H41	12.35	0.46	-11.89
N3-C4-N4-H42	149.51	167.16	17.65
C5-C4-N4-H41	-170.16	-178.97	-8.81
C5-C4-N4-H42	-33.01	-12.27	20.74
N3-C4-C5-C6	-3.10	-2.52	0.58
N3-C4-C5-C7	174.29	175.50	1.22
N4-C4-C5-C6	179.61	176.89	-2.72
N4-C4-C5-C7	-3.01	-5.09	-2.08
C4-C5-C6-N1	1.91	1.26	-0.66
C4-C5-C6-H6	-178.89	-179.32	-0.43
C7-C5-C6-N1	-175.45	-176.75	-1.30
C7-C5-C6-H6	3.74	2.67	-1.07
C4-C5-C7-O7	60.63	65.00	4.37
C6-C5-C7-O7	-122.18	-117.09	5.09
C5-C7-O7-HO7	-173.88	174.31	11.81

Figure 84: The PES scan for flexible dihedral corresponding to HMC.

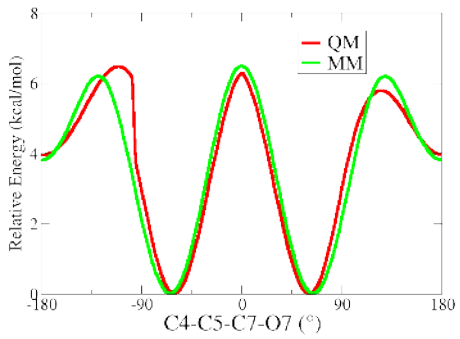

Table 208: The statistics of vibrational frequencies of HMC. Only the terms with the occupancies greater than 15% were shown. Refer to p.6 for the meanings of assig.

QM (MP2, scaled by 0.943)							MM						
Freq	Assig.	%	Assig.	%	Assig.	%	Freq	Assig.	%	Assig.	%	Assig.	%
76.8	at6r'	50.	at6r	46.			82.7	at6r'	30.	wC7-C5	30.	tCCCO	22.
124.4	tCCCO	80.					121.1	tCCCO	62.	at6r	20.	at6r'	19.
191.3	pk6r	38.	wC7-C5	23.			195.7	pk6r	37.	at6r	28.	wC-N4	24.
207.8	at6r	34.	pk6r	15.			223.9	wC7-C5	36.	rC7-C5	16.		
267.8	rC7-C5	55.					307.0	rC7-C5	55.				
345.3	tCCOH	79.					350.2	cCCO	21.				
363.9	rC-N4	24.	rC=O	17.			364.5	rC-N4	44.	sN-C.r	20.		
372.3	at6r'	38.	pk6r	33.			405.9	tCCOH	86.				
391.9	cCCO	17.					443.8	at6r'	35.				
512.9	ad6r	38.	rC=O	23.			492.8	tC4N4	88.				
544.2	ad6r'	39.	tC4N4	20.			548.6	ad6r'	20.	cCCO	18.	sN-C.r	17.
567.3	rC=O	26.					551.9	wN-H	103.				
580.5	tC4N4	68.					570.6	ad6r	20.	sC-N4	18.		
611.8	wN-H	51.	wC-N4	30.	pk6r	-19.	599.8	rC=O	29.	ad6r	18.	sN-C.r	17.
650.9	wN-H	34.	wHH2	33.	pk6r	19.	647.5	wHH2	55.				
692.9	wHH2	36.	pk6r	22.	wC-N4	16.	730.1	sC-C.r	36.	ad6r'	23.		
717.0	wC=O	81.					741.8	wC=O	67.				
732.2	sC-C.r	37.	sN-C.r	23.			760.3	td6r	17.				
784.1	td6r	40.	sN-C.r	18.			873.4	sN-C.r	25.	wC-N4	18.		
861.9	wC-H	103.					891.4	wC-N4	21.	rCH2	16.	pk6r	16.
918.8	sN-C.r	42.	td6r	17.			903.2	wC-H	65.				
1000.7	rCH2	67.					919.9	rCH2	53.	wC-H	29.		
1027.0	sC-O	89.					1010.2	sN-C.r	34.	rNH2	24.		
1117.2	sN-C.r	33.	rNH2	28.			1021.4	rNH2	34.	sN-C.r	23.		
1183.7	rNH2	27.	dCOH	18.			1045.6	sC-O	30.	sN-C.r	21.	wCH2	17.
1208.2	sN-C.r	35.	dCOH	28.	rN-H	20.	1175.9	td6r	24.	iCH2	16.		
1239.9	sN-C.r	28.	dCOH	22.			1214.2	iCH2	29.	sC-C.r	20.	rC-H	18.
1242.7	iCH2	67.					1270.2	iCH2	46.				
1279.0	sN-C.r	23.	sC-N4	20.	wCH2	15.	1284.3	dCOH	63.				
1342.1	rC-H	43.					1401.0	rN-H	63.				
1429.6	rN-H	43.	sN-C.r	23.			1455.9	sN-C.r	20.				
1440.6	wCH2	56.	dCOH	19.			1477.2	cCH2	66.				
1486.0	sN-C.r	24.	sC-N4	21.			1521.5	wCH2	38.	sC-O	27.	cCH2	20.
1524.7	cCH2	101.					1531.9	sN-C.r	52.	sC-C.r	15.		
1570.9	sN-C.r	42.	sC-C.r	29.			1595.5	sN-C.r	27.	sC-C.r	17.		
1629.6	cNH2	79.					1632.7	cNH2	60.				
1705.7	sC-C.r	41.	sN-C.r	22.			1699.1	sC-C.r	21.				
1775.3	sC=O	73.					1773.9	sC=O	48.	sN-C.r	20.		
2967.0	ssCH2	100.					2802.8	ssCH2	99.				
3016.5	asCH2	99.					2842.3	asCH2	99.				
3141.4	sC-H	99.					2995.1	sC-H	99.				
3457.1	ssNH2	99.					3444.0	ssNH2	99.				
3520.0	sN-H	100.					3460.0	sN-H	99.				
3582.1	asNH2	100.					3563.7	asNH2	100.				
3637.2	sO-H	100.					3682.9	sO-H	100.				

2.21 methylwyosine (MWG)

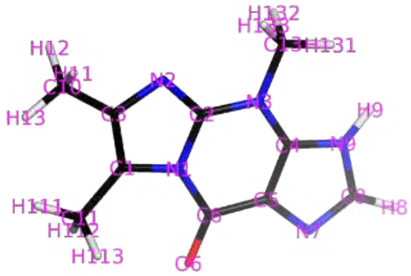

Figure 85: The energy-minimized structure of MWG.

Table 209: The calculated geometric terms of MWG.

Terms	QM	MM	diff
N9-C8	1.386	1.366	-0.020
N9-C4	1.369	1.364	-0.004
N9-H9	1.013	1.002	-0.011
C8-H8	1.082	1.092	0.010
C8-N7	1.316	1.316	-0.001
N7-C5	1.383	1.396	0.013
C5-C6	1.444	1.421	-0.023
C5-C4	1.391	1.393	0.002
C6-O6	1.224	1.240	0.016
C6-N1	1.439	1.430	-0.009
N1-C2	1.389	1.408	0.018
N1-C1	1.404	1.383	-0.021
C2-N2	1.313	1.347	0.033
C2-N3	1.387	1.392	0.006
N2-C3	1.390	1.383	-0.007
N3-C4	1.361	1.384	0.024
N3-C13	1.454	1.472	0.018
C1-C3	1.380	1.374	-0.006
C1-C11	1.492	1.515	0.023
C3-C10	1.493	1.488	-0.005
C10-H11	1.094	1.109	0.015
C10-H12	1.094	1.109	0.015
C10-H13	1.093	1.108	0.016
C11-H111	1.093	1.111	0.018
C11-H112	1.092	1.112	0.020
C11-H113	1.092	1.112	0.020
C13-H131	1.094	1.112	0.018
C13-H132	1.095	1.114	0.019
C13-H133	1.089	1.116	0.027
C8-N9-C4	106.23	107.20	0.97
C8-N9-H9	126.67	127.00	0.34
C4-N9-H9	127.08	125.79	-1.29
N9-C8-H8	121.41	123.12	1.72
N9-C8-N7	112.74	112.22	-0.52
H8-C8-N7	125.85	124.66	-1.19
C8-N7-C5	104.66	105.48	0.82
N7-C5-C6	127.54	130.85	3.31
N7-C5-C4	110.48	108.85	-1.63
C6-C5-C4	121.96	120.29	-1.67
C5-C6-O6	128.48	122.27	-6.22
C5-C6-N1	109.70	113.15	3.45
O6-C6-N1	121.82	124.58	2.76
C6-N1-C2	126.74	125.17	-1.56
C6-N1-C1	127.68	128.96	1.28
C2-N1-C1	105.55	105.86	0.31
N1-C2-N2	113.45	111.49	-1.96
N1-C2-N3	120.49	119.89	-0.60
N2-C2-N3	126.03	128.62	2.59
C2-N2-C3	104.22	104.52	0.31
C2-N3-C4	115.14	115.40	0.26
C2-N3-C13	120.26	119.59	-0.67
C4-N3-C13	123.88	124.92	1.04
N9-C4-C5	105.88	106.25	0.36
N9-C4-N3	128.28	127.75	-0.53
C5-C4-N3	125.78	126.00	0.21
N1-C1-C3	105.04	106.54	1.50
N1-C1-C11	123.59	125.03	1.44
C3-C1-C11	131.37	128.44	-2.94
N2-C3-C1	111.74	111.59	-0.15
N2-C3-C10	119.17	116.75	-2.42
C1-C3-C10	129.09	131.66	2.57
C3-C10-H11	110.53	109.40	-1.13
C3-C10-H12	110.55	109.40	-1.16
C3-C10-H13	111.46	110.65	-0.81
H11-C10-H12	107.53	109.02	1.49
H11-C10-H13	108.33	109.17	0.85
H12-C10-H13	108.31	109.17	0.86
C1-C11-H111	108.56	111.46	2.91
C1-C11-H112	111.89	110.06	-1.83
C1-C11-H113	111.83	110.05	-1.78
H111-C11-H112	108.67	108.49	-0.18
H111-C11-H113	108.64	108.49	-0.15
H112-C11-H113	107.16	108.20	1.05
N3-C13-H131	109.78	112.41	2.63
N3-C13-H132	111.12	110.96	-0.16
N3-C13-H133	107.02	111.44	4.42
H131-C13-H132	110.00	108.21	-1.79
H131-C13-H133	109.46	106.65	-2.81
H132-C13-H133	109.39	106.91	-2.49
C4-N9-C8-H8	179.46	179.97	0.52
C4-N9-C8-N7	-0.43	0.04	0.47
H9-N9-C8-H8	1.15	0.63	-0.53
H9-N9-C8-N7	-178.73	-179.30	-0.57
C8-N9-C4-C5	0.64	-0.07	-0.72
C8-N9-C4-N3	178.10	179.56	1.46
H9-N9-C4-C5	178.94	179.28	0.34
H9-N9-C4-N3	-3.60	-1.08	2.52
N9-C8-N7-C5	0.01	0.01	-0.00
H8-C8-N7-C5	-179.87	-179.92	-0.05
C8-N7-C5-C6	179.10	179.49	0.38
C8-N7-C5-C4	0.41	-0.06	-0.47
N7-C5-C6-O6	0.66	1.21	0.55
N7-C5-C6-N1	-179.56	-178.13	1.43
C4-C5-C6-O6	179.22	-179.29	1.49
C4-C5-C6-N1	-1.00	1.37	2.37
N7-C5-C4-N9	-0.67	0.08	0.75
N7-C5-C4-N3	-178.22	-179.56	-1.35

Terms	QM	MM	diff
C6-C5-C4-N9	-179.45	-179.52	-0.07
C6-C5-C4-N3	3.01	0.84	-2.17
C5-C6-N1-C2	1.35	-1.24	-2.59
C5-C6-N1-C1	179.11	177.50	-1.60
O6-C6-N1-C2	-178.85	179.43	1.72
O6-C6-N1-C1	-1.10	-1.82	-0.72
C6-N1-C2-N2	178.29	178.98	0.69
C6-N1-C2-N3	-3.43	-1.09	2.34
C1-N1-C2-N2	0.14	-0.01	-0.15
C1-N1-C2-N3	178.41	179.92	1.51
C6-N1-C1-C3	-178.31	-178.97	-0.66
C6-N1-C1-C11	1.77	0.97	-0.80
C2-N1-C1-C3	-0.18	-0.04	0.14
C2-N1-C1-C11	179.90	179.91	0.01
N1-C2-N2-C3	-0.04	0.05	0.09
N3-C2-N2-C3	-178.20	-179.87	-1.68
N1-C2-N3-C4	4.74	3.14	-1.60
N1-C2-N3-C13	175.38	179.94	4.56
N2-C2-N3-C4	-177.22	-176.94	0.28
N2-C2-N3-C13	-6.58	-0.14	6.43
C2-N2-C3-C1	-0.08	-0.07	0.01
C2-N2-C3-C10	-179.81	180.00	0.19
C2-N3-C4-N9	178.27	177.28	-1.00
C2-N3-C4-C5	-4.74	-3.15	1.58
C13-N3-C4-N9	8.01	0.67	-7.34
C13-N3-C4-C5	-175.00	-179.76	-4.76
N1-C1-C3-N2	0.17	0.07	-0.10
N1-C1-C3-C10	179.86	179.99	0.12
C11-C1-C3-N2	-179.92	-179.87	0.05
C11-C1-C3-C10	-0.23	0.04	0.27

Table 210: The statistics of vibrational frequencies of MWG. Only the terms with the occupancies greater than 15% were shown. Refer to p.6 for the meanings of assig.

QM (MP2, scaled by 0.943)							MM						
Freq	Assig.	%	Assig.	%	Assig.	%	Freq	Assig.	%	Assig.	%	Assig.	%
54.1	wN-C13	36.	tCCH3	27.	at6r'	22.	30.9	at6r'	46.	at6r	19.	wN-C13	18.
59.2	wN-C13	45.	at6r'	32.			34.7	tCCH3	83.				
64.8	tCCH3	82.					64.2	wN-C13	51.	at6r'	17.	btfl2	15.
82.3	pk6r	24.	wC-C11	23.	at6r	19.	91.5	wC-C11	23.	tr3'	18.		
107.2	tCCH3	62.					119.3	tCCH3	94.				
149.8	at6r	39.	pk6r	21.	btfl1	21.	140.1	tCCH3	92.				
178.8	tCCH3	88.					180.3	wC-C	30.	at6r	25.		
191.2	wC-C	33.	btfl1	28.			207.3	sC-N.r	26.	rC-C	23.		
208.0	sC-N.r	24.	rC-C	23.			244.8	wC-C11	37.	at6r'	19.	pk6r	17.
231.8	wC-C11	47.					252.4	t5r	23.	at6r	18.		
261.0	rN-C13	43.					266.4	rN-C13	62.				
279.2	btfl1	37.	pk6r	31.	at6r'	21.	299.5	btfl1	57.				
292.4	rC-C11	44.	rC-C	32.			304.6	rC-C11	35.	rC-C	22.	sC-N.r	16.
323.8	rC=O	31.					344.0	ad6r'	20.	sC-N.r	19.		
326.1	btfl2	32.	wC-C	19.			371.5	btfl2	32.	wC-C	23.		
386.0	wNH	84.					386.1	sC-N.r	20.	rC=O	17.	ad6r	16.
388.9	sC-N.r	24.	dr3	22.			401.9	rC=O	26.	sC-N.r	25.	dr3	17.
394.9	ad6r'	40.	sC-N.r	21.	ad6r	17.	447.5	wNH	72.	t5r'	19.		
506.8	sC-N.r	29.	ad6r'	20.			473.8	sC-N.r	27.	ad6r'	22.	sC-C.r	16.
525.9	ad6r	32.					559.5	ad6r	30.	sC-N.r	20.		
534.6	t5r	41.	t5r'	27.			561.5	tr3'	42.	tr3	21.	wC=O	20.
566.5	tr3'	50.	tr3	22.			578.3	sC-C	40.				
608.1	tr3	55.	tr3'	23.			598.3	sC-N.r	31.	sC-C	19.	td6r	17.
622.5	sC-C	22.	td6r	20.	dr3'	16.	625.3	t5r'	40.				
631.2	t5r	50.	t5r'	30.	wC=O	21.	654.8	tr3	39.				
647.3	wC=O	71.					674.5	sC-N.r	25.	td6r	22.		
691.8	dr3'	24.	td6r	22.	sC-N.r	22.	696.7	wC=O	31.	t5r	21.		
735.6	wCH	106.					712.9	t5r	39.	pk6r	22.	wC=O	17.
748.1	sC-C	28.	sC-N.r	15.			766.5	sC-N.r	24.				
790.5	d5r'	19.	rC=O	18.			844.0	d5r	34.	d5r'	25.	sC-N.r	19.
908.4	d5r	52.	d5r'	27.			900.9	wCH	107.				
951.8	sC-N.r	32.	rCH3	26.			916.5	sC-N.r	44.	rC=O	18.		
994.7	rCH3'	37.	sC-N.r	25.			939.5	sC-N.r	43.				
1017.0	sC-N.r	24.	sN-C	18.			985.0	rCH3'	66.	rCH3	22.		
1051.5	rCH3'	45.	rCH3	42.			1004.8	sC-N.r	35.	rCH3	15.		
1056.2	sC-N.r	30.	rCH3	17.			1020.0	sC-N.r	30.	rCH3	25.	rCH3'	18.
1057.7	rCH3'	66.	rCH3	22.			1064.5	sC-N.r	38.	rCH3	21.		
1090.8	sC-N.r	55.	rNH	23.			1073.3	rCH3'	66.	rCH3	16.		
1124.4	sC-N.r	35.	rCH3	23.			1080.2	rCH3	29.	sC-N.r	23.	rCH3'	17.
1140.9	rCH3	56.	rCH3'	37.			1084.7	rCH3	45.	rCH3'	34.		
1188.9	rCH3'	28.	sC-N.r	22.	rCH3	18.	1101.3	rCH3	34.	sC-N.r	21.		
1212.8	sC-N.r	28.	rCH	19.			1122.0	sC-N.r	40.				
1250.9	sC-N.r	48.	sN-C	16.			1171.9	sC-N.r	28.	rCH3	20.		
1279.9	sC-N.r	59.					1200.1	sC-N.r	36.	rCH	31.		
1312.7	sC-N.r	46.	rCH	20.			1302.7	sC-N.r	40.				
1355.2	sC-N.r	41.	sC-C.r	18.			1327.2	sC-N.r	42.	rNH	18.		
1372.0	sC-N.r	45.	rNH	30.			1366.8	sC-N.r	34.	sdCH3	24.		
1381.7	sC-N.r	45.	sdCH3	23.			1396.7	adCH3	61.	adCH3'	21.		
1409.4	sC-N.r	54.					1417.7	adCH3'	59.	adCH3	21.		
1429.8	sdCH3	71.					1427.2	adCH3	61.	adCH3'	15.		
1434.8	sdCH3	85.					1435.0	adCH3'	84.	rCH3'	16.		
1444.5	sdCH3	81.					1438.0	sC-N.r	34.				
1475.4	sC-N.r	63.	rCH	19.			1440.1	adCH3	70.				
1487.5	adCH3'	63.	adCH3	30.			1440.4	adCH3'	48.	adCH3	28.		
1492.5	adCH3'	53.	adCH3	40.			1475.0	sdCH3	39.	sC-N.r	27.	sC-C.r	16.
1499.6	adCH3	86.					1505.2	sdCH3	52.	sC-N.r	17.		
1506.5	adCH3'	67.	adCH3	24.			1507.6	sdCH3	48.				
1507.0	adCH3'	64.	adCH3	26.			1529.7	sdCH3	51.	sC-N.r	16.		

QM (MP2, scaled by 0.943)								MM							
1512.0	adCH3	63.	adCH3'	21.				1575.4	sC-N.r	23.	rCH	20.	sC-C.r	16.
1547.7	sC-N.r	39.	sC-C.r	27.				1582.1	sC-N.r	29.	sdCH3	19.		
1593.1	sC-N.r	61.						1627.0	sC-N.r	34.				
1607.9	sC-C.r	57.	sC-C	20.				1647.9	sC-C.r	27.	sC-N.r	16.		
1655.1	sN-H	41.	sC-N.r	22.	sC-C.r	16.		1694.5	sC-N.r	41.				
1764.8	sC=O	75.	sC-C.r	15.				1810.1	sC=O	43.	sC-C.r	20.		
3002.2	ssCH3	94.						2848.5	ssCH3	100.				
3010.7	ssCH3	99.						2851.4	ssCH3	100.				
3023.3	ssCH3	100.						2907.8	ssCH3	100.				
3081.1	asCH3	71.	asCH3'	28.				2912.5	asCH3'	100.				
3086.2	asCH3	74.	asCH3'	26.				2914.2	asCH3	66.	asCH3'	33.		
3106.9	asCH3'	56.	asCH3	44.				2915.0	asCH3	75.	asCH3'	25.		
3107.6	asCH3'	100.						2918.8	asCH3	58.	asCH3'	42.		
3114.2	asCH3	81.	asCH3'	18.				2952.5	sC-H	99.				
3142.0	asCH3'	66.	asCH3	28.				2956.8	asCH3'	79.	asCH3	21.		
3209.7	sC-H	99.						2958.3	asCH3	79.	asCH3'	21.		
3548.9	sN-H	100.						3496.8	sN-H	100.				

2.22 5-methyoxyuracil (MOU)

Figure 86: The energy-minimized structure of MOU.

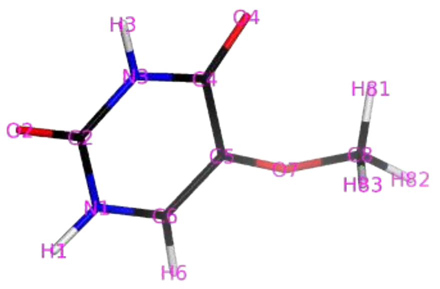

Table 211: The calculated geometric terms of MOU.

Terms	QM	MM	diff
N1-C2	1.383	1.376	-0.007
N1-C6	1.382	1.373	-0.009
N1-H1	1.015	1.007	-0.008
C2-O2	1.230	1.223	-0.006
C2-N3	1.390	1.365	-0.025
N3-H3	1.020	1.003	-0.017
N3-C4	1.403	1.385	-0.018
C4-O4	1.233	1.232	-0.001
C4-C5	1.467	1.466	-0.001
C5-C6	1.358	1.374	0.015
C5-O7	1.368	1.367	-0.000
C6-H6	1.085	1.092	0.007
O7-C8	1.446	1.428	-0.018
C8-H81	1.091	1.113	0.022
C8-H82	1.089	1.112	0.022
C8-H83	1.096	1.113	0.018
C2-N1-C6	123.85	121.41	-2.44
C2-N1-H1	115.22	113.81	-1.41
C6-N1-H1	120.62	124.77	4.15
N1-C2-O2	123.66	121.42	-2.24
N1-C2-N3	112.65	116.52	3.87
O2-C2-N3	123.67	122.07	-1.61
C2-N3-H3	115.06	116.95	1.89
C2-N3-C4	128.58	125.82	-2.76
H3-N3-C4	115.58	117.19	1.60
N3-C4-O4	121.00	117.21	-3.79
N3-C4-C5	113.17	116.15	2.99
O4-C4-C5	125.82	126.64	0.82
C4-C5-C6	119.54	117.00	-2.53
C4-C5-O7	120.21	124.94	4.74
C6-C5-O7	119.78	117.69	-2.09
N1-C6-C5	121.64	122.99	1.35
N1-C6-H6	116.93	118.14	1.21
C5-C6-H6	121.43	118.87	-2.56
C5-O7-C8	113.81	110.31	-3.50
O7-C8-H81	110.55	110.96	0.41
O7-C8-H82	105.19	108.67	3.48
O7-C8-H83	109.72	112.15	2.43
H81-C8-H82	110.38	107.48	-2.90
H81-C8-H83	110.82	109.45	-1.37
H82-C8-H83	110.04	107.97	-2.06
C6-N1-C2-O2	176.91	-179.63	3.46
C6-N1-C2-N3	-4.60	0.27	4.86
H1-N1-C2-O2	3.27	1.36	-1.90
H1-N1-C2-N3	-178.24	-178.74	-0.50
C2-N1-C6-C5	2.45	1.30	-1.16
C2-N1-C6-H6	-177.80	-178.11	-0.31
H1-N1-C6-C5	175.77	-179.81	4.42
H1-N1-C6-H6	-4.49	0.79	5.27
N1-C2-N3-H3	177.80	177.98	0.18
N1-C2-N3-C4	8.41	0.56	-7.85
O2-C2-N3-H3	-3.71	-2.12	1.59
O2-C2-N3-C4	-173.09	-179.54	-6.45
C2-N3-C4-O4	172.65	177.53	4.87
C2-N3-C4-C5	-8.91	-2.65	6.25
H3-N3-C4-O4	3.32	0.11	-3.20
H3-N3-C4-C5	-178.24	179.93	1.83
N3-C4-C5-C6	5.48	3.89	-1.59
N3-C4-C5-O7	177.55	176.81	-0.74
O4-C4-C5-C6	-176.17	-176.31	-0.14
O4-C4-C5-O7	-4.10	-3.39	0.71
C4-C5-C6-N1	-2.86	-3.39	-0.53
C4-C5-C6-H6	177.41	176.01	-1.40
O7-C5-C6-N1	-174.96	-176.84	-1.87
O7-C5-C6-H6	5.30	2.56	-2.74
C4-C5-O7-C8	65.94	58.78	-7.16
C6-C5-O7-C8	-122.00	-128.34	-6.34

Figure 87: The PES scan for flexible dihedral corresponding to MOU.

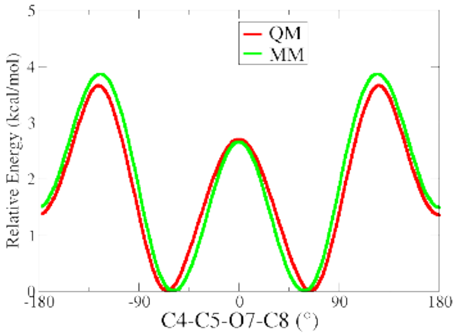

Table 212: The statistics of vibrational frequencies of MOU. Only the terms with the occupancies greater than 15% were shown. Refer to p.6 for the meanings of assig.

QM (MP2, scaled by 0.943)							MM						
Freq	Assig.	%	Assig.	%	Assig.	%	Freq	Assig.	%	Assig.	%	Assig.	%
77.3	at6r'	51.	at6r	35.	pk6r	-17.	60.7	tCCOC	59.	wC-O	26.		
99.3	tCCOC	66.	at6r	22.	tCOCH	17.	105.0	at6r	31.	at6r'	25.	tCCOC	22.
126.9	pk6r	77.	at6r	29.			157.1	at6r	31.	pk6r	21.	tCOCH	17.
161.1	tCOCH	65.					189.0	tCOCH	34.	pk6r	18.	at6r'	17.
206.3	dCOC	26.	wC-O	24.	rC-O	22.	259.8	tCOCH	27.	tCCOC	20.	at6r	16.
316.9	rC-O	28.	rC=O	21.			332.3	pk6r	29.	dCOC	26.	at6r'	24.
329.6	at6r'	48.	pk6r	34.			389.4	rC-O	24.	sN-C	22.		
376.0	rC=O	49.	sN-C	22.			411.5	rC=O	46.	sN-C	21.		
409.4	ad6r	24.	wC-O	19.			431.0	sN-C	18.	wC-O	17.	at6r'	17.
474.8	ad6r	24.	dCOC	23.			538.0	wN-H	63.	dCOC	16.		
524.3	wN-H	76.					559.9	wN-H	37.	dCOC	24.		
528.7	ad6r'	40.	sN-C	18.			593.9	ad6r	47.				
608.0	rC=O	47.	rC-O	22.			637.6	rC=O	16.				
639.2	wC2=O	32.	pk6r	23.	wC4=O	20.	649.6	wC2=O	37.				
662.9	wN-H	96.	pk6r	-29.	wC2=O	25.	680.3	wN-H	107.				
697.9	wC2=O	47.	wC4=O	37.			737.0	ad6r'	23.	sN-C	21.	sC-C	16.
721.7	sC-C	33.	sN-C	19.			787.7	pk6r	39.	wC4=O	29.		
783.2	td6r	45.					796.0	sN-C	16.	wC6-H	16.		
827.9	wC6-H	104.					824.4	wC6-H	69.	wC-O	16.		
960.9	sN-C	51.	td6r	17.			885.2	sN-C	41.	td6r	22.		
1005.5	sO-C8	82.					975.2	rCH3'	26.	sO-C8	25.	sN-C	16.
1137.2	sN-C	40.					1025.3	rCH3	69.				
1155.7	rCH3	68.	rCH3'	27.			1062.2	rCH3'	39.	sN-C	22.		
1185.1	sN-C	31.	rCH3'	25.	rC-H	18.	1070.6	sO-C8	48.	rCH3'	17.		
1200.2	sN-C	36.	rCH3'	29.			1235.0	sC5-O	25.	sN-C	22.	td6r	15.
1266.9	sC5-O	46.	sN-C	25.			1341.3	rC-H	26.	rN-H	23.	sC=O	19.
1334.9	rC-H	44.	sN-C	22.			1396.0	rN-H	55.				
1372.5	rN-H	70.	sC=O	16.			1405.2	adCH3'	53.	adCH3	38.		
1403.6	rN-H	29.	sN-C	29.	rC=O	16.	1426.6	rN-H	56.				
1456.4	sdCH3	77.					1445.6	adCH3	49.	adCH3'	37.		
1479.4	sN-C	25.	rN-H	22.	sdCH3	19.	1511.7	sN-C	50.	rC=O	16.		
1488.0	adCH3	95.					1523.9	sdCH3	75.				
1512.6	adCH3'	94.					1567.3	sN-C	21.				
1653.2	sC=C	58.					1669.5	rC-H	23.				
1709.7	sC=O	72.					1763.1	sC=O	57.				
1757.6	sC=O	67.					1849.8	sC=O	38.	ad6r	15.		
3002.3	ssCH3	93.					2853.4	ssCH3	100.				
3104.1	asCH3	62.	asCH3'	34.			2911.8	asCH3'	86.				
3142.9	asCH3'	58.	asCH3	39.			2917.3	asCH3	87.				
3168.7	sC-H	99.					2999.4	sC-H	99.				
3475.1	sN-H	100.					3457.5	sN-H	99.				
3527.6	sN-H	100.					3461.4	sN-H	99.				

2.23 adenine, protonated (ADEp)

Figure 88: The energy-minimized structure of ADEp.

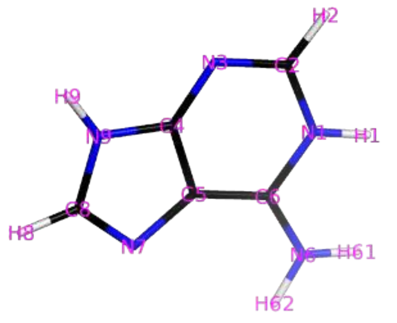

Table 213: The calculated geometric terms of ADEp.

Terms	QM	MM	diff
N9-C8	1.374	1.376	0.002
N9-C4	1.363	1.360	-0.003
N9-H9	1.018	1.004	-0.013
C8-H8	1.083	1.096	0.013
C8-N7	1.329	1.327	-0.002
N7-C5	1.366	1.393	0.027
C5-C6	1.407	1.387	-0.019
C5-C4	1.400	1.397	-0.003
C6-N6	1.329	1.345	0.016
C6-N1	1.364	1.394	0.030
N6-H61	1.013	0.994	-0.019
N6-H62	1.016	1.001	-0.015
N1-H1	1.020	0.996	-0.024
N1-C2	1.388	1.417	0.029
C2-H2	1.085	1.105	0.019
C2-N3	1.305	1.362	0.056
N3-C4	1.355	1.344	-0.011
C8-N9-C4	107.10	107.48	0.38
C8-N9-H9	126.99	128.89	1.90
C4-N9-H9	125.91	123.64	-2.27
N9-C8-H8	122.00	122.91	0.91
N9-C8-N7	113.18	112.59	-0.59
H8-C8-N7	124.82	124.50	-0.32
C8-N7-C5	103.10	103.95	0.85
N7-C5-C6	129.39	133.86	4.46
N7-C5-C4	112.44	110.63	-1.81
C6-C5-C4	118.17	115.51	-2.66
C5-C6-N6	123.50	127.83	4.33
C5-C6-N1	113.66	119.03	5.37
N6-C6-N1	122.84	113.14	-9.70
C6-N6-H61	123.97	122.72	-1.25
C6-N6-H62	117.92	118.96	1.04
H61-N6-H62	118.11	118.32	0.22
C6-N1-H1	119.25	117.63	-1.62
C6-N1-C2	123.95	121.49	-2.46

Terms	QM	MM	diff
H1-N1-C2	116.80	120.89	4.09
N1-C2-H2	115.21	119.58	4.36
N1-C2-N3	124.32	119.86	-4.46
H2-C2-N3	120.47	120.56	0.10
C2-N3-C4	112.81	116.44	3.63
N9-C4-C5	104.18	105.35	1.17
N9-C4-N3	128.73	126.98	-1.75
C5-C4-N3	127.09	127.67	0.58
C4-N9-C8-H8	180.00	-180.00	0.00
C4-N9-C8-N7	-0.00	0.00	0.00
H9-N9-C8-H8	-0.00	0.00	0.00
H9-N9-C8-N7	180.00	180.00	0.00
C8-N9-C4-C5	0.00	0.00	-0.00
C8-N9-C4-N3	-180.00	180.00	0.00
H9-N9-C4-C5	180.00	-180.00	-0.00
H9-N9-C4-N3	0.00	0.00	0.00
N9-C8-N7-C5	-0.00	0.00	0.00
H8-C8-N7-C5	180.00	180.00	0.00
C8-N7-C5-C6	180.00	180.00	0.00
C8-N7-C5-C4	0.00	0.00	-0.00
N7-C5-C6-N6	0.00	0.00	-0.00
N7-C5-C6-N1	180.00	-180.00	-0.00
C4-C5-C6-N6	180.00	180.00	-0.00
C4-C5-C6-N1	-0.00	0.00	0.00
N7-C5-C4-N9	-0.00	0.00	0.00
N7-C5-C4-N3	180.00	-180.00	0.00
C6-C5-C4-N9	-180.00	-180.00	-0.00
C6-C5-C4-N3	0.00	0.00	-0.00
C5-C6-N6-H61	180.00	180.00	-0.00
C5-C6-N6-H62	-0.00	0.00	0.00
N1-C6-N6-H61	0.00	0.00	-0.00
N1-C6-N6-H62	180.00	180.00	0.00
C5-C6-N1-H1	-180.00	-180.00	-0.00
C5-C6-N1-C2	0.00	0.00	-0.00
N6-C6-N1-H1	-0.00	0.00	0.00
N6-C6-N1-C2	180.00	-180.00	0.00
C6-N1-C2-H2	-180.00	-180.00	-0.00
C6-N1-C2-N3	-0.00	0.00	0.00
H1-N1-C2-H2	0.00	0.00	-0.00
H1-N1-C2-N3	180.00	180.00	0.00
N1-C2-N3-C4	0.00	0.00	-0.00
H2-C2-N3-C4	180.00	180.00	0.00
C2-N3-C4-N9	180.00	180.00	0.00
C2-N3-C4-C5	-0.00	0.00	0.00

Table 214: The statistics of vibrational frequencies of ADEp. Only the terms with the occupancies greater than 15% were shown. Refer to p.6 for the meanings of assig.

QM (MP2, scaled by 0.943)							MM						
Freq	Assig.	%	Assig.	%	Assig.	%	Freq	Assig.	%	Assig.	%	Assig.	%
151.8	at6r	55.	pk6r	36.			170.6	at6r	46.	wC6N6	21.	at6r'	17.
194.4	btf	83.	at6r'	20.			211.8	btf	76.				
265.6	at6r'	64.					296.0	at6r'	39.	at6r	26.	t5r'	15.
266.4	rC6N6	54.					323.2	rC6N6	65.				
300.4	wNH2	77.					381.4	tC6N	54.	wNH2	29.		
495.0	ad6r'	63.	sN-C.ar	18.			415.0	pk6r	65.	at6r	22.		
509.7	tC6N	55.	t5r'	20.			485.7	sN-C.ar	49.	ad6r'	30.		
512.8	ad6r	43.					498.3	wN-H	57.	t5r'	32.		
526.8	tC6N	21.	t5r'	21.	at6r	21.	546.0	ad6r	44.	sN-C.ar	29.		
566.0	wC6N6	66.	pk6r	16.			550.8	t5r	30.	wN1H1	22.	wC6N6	16.
600.2	d5r'	32.	sC-C.ar	21.			585.4	sN-C.ar	34.	sC-C.ar	24.	d5r'	21.
605.3	pk6r	37.	wN-H	33.	t5r'	17.	671.1	t5r'	37.	wN-H	36.		
617.3	t5r	65.	wN1H1	16.			672.1	sN-C.ar	31.	ad6r'	27.		
656.0	wN-H	57.	t5r'	30.			686.5	tC6N	31.	wNH2	30.		
686.2	wN1H1	84.					705.4	t5r	41.	wN1H1	40.		
706.6	sN-C.ar	55.	sC-C.ar	18.			844.6	td6r	33.	ad6r	22.	sN-C.ar	21.
838.9	wC-H	105.					857.0	wC2H2	65.				
864.5	wC2H2	109.					857.7	d5r	27.	sN-C.ar	25.	sC-C.ar	17.
875.2	td6r	55.	ad6r	17.			911.7	wC-H	105.				
914.8	d5r	38.	d5r'	33.			930.5	wC2H2	30.	wC6N6	25.	pk6r	21.
988.9	rNH2	49.	sN-C.ar	42.			993.9	rNH2	59.	sN-C.ar	30.		
1088.0	sN-C.ar	53.	rN-H	32.			1034.2	sN-C.ar	70.				
1116.5	sN-C.ar	65.					1063.9	sN-C.ar	41.				
1159.6	sN-C.ar	34.	rNH2	21.			1096.9	sN-C.ar	56.	rN-H	18.		
1236.4	rC-H	44.	sN-C.ar	37.			1172.6	sN-C.ar	43.	rC-H	28.		
1331.6	sN-C.ar	66.					1204.9	sN-C.ar	34.	rC-H	28.		
1347.1	sN-C.ar	53.	rC-H	33.			1251.4	sN-C.ar	52.	rC-H	16.		
1400.9	sN-C.ar	34.	rN-H	30.	rC-H	28.	1341.4	sN-C.ar	32.	rN-H	26.		
1416.9	rN-H	46.	sN-C.ar	16.			1386.2	cNH2	23.	rN-H	20.	sN-C.ar	18.
1435.1	sN-C.ar	55.	rC-H	23.			1464.8	sN-C.ar	47.	rN-H	25.		
1478.6	sN-C.ar	45.	sC-C.ar	30.			1560.0	rC-H	36.	sN-C.ar	27.		
1508.9	sN-C.ar	55.	sC-C.ar	17.			1582.1	cNH2	45.	rC-H	17.	sN-C.ar	16.
1569.4	sN-C.ar	48.	rN-H	18.	sC-C.ar	16.	1609.2	sN-C.ar	31.	sC-C.ar	30.	rN-H	16.
1609.6	cNH2	42.	sN-C.ar	32.			1654.5	sN-C.ar	35.	rC-H	30.		
1627.1	cNH2	35.	sN-C.ar	31.	sC-C.ar	15.	1678.0	sN-C.ar	40.	sC-C.ar	22.		
1724.9	sC6-N6	33.	sC-C.ar	29.			1752.4	sN-C.ar	28.	sC-C.ar	17.		
3178.8	sC-H	99.					2954.6	sC-H	99.				
3217.7	sC-H	99.					3124.9	sC-H	99.				
3459.8	sN-H	91.					3327.8	ssNH2	97.				
3470.2	ssNH2	89.					3385.8	sN-H	95.				
3501.9	sN-H	99.					3435.5	asNH2	98.				
3594.4	asNH2	98.					3502.4	sN-H	100.				

2.24 cytosine, protonated (CYTp)

Figure 89: The energy-minimized structure of CYPp.

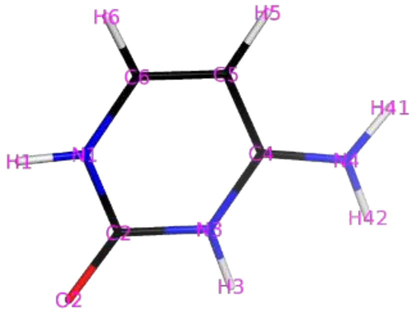

Table 215: The calculated geometric terms of CYPp.

Terms	QM	MM	diff
N1-C2	1.392	1.389	-0.003
N1-C6	1.358	1.374	0.016
N1-H1	1.019	1.009	-0.009
C2-O2	1.210	1.221	0.011
C2-N3	1.415	1.400	-0.015
N3-H3	1.021	0.989	-0.031
N3-C4	1.354	1.347	-0.007
C4-N4	1.329	1.335	0.006
C4-C5	1.420	1.415	-0.005
N4-H41	1.014	0.997	-0.017
N4-H42	1.015	0.995	-0.020
C5-H5	1.082	1.089	0.007
C5-C6	1.364	1.372	0.008
C6-H6	1.085	1.102	0.016
C2-N1-C6	124.23	121.95	-2.28
C2-N1-H1	114.90	113.01	-1.89
C6-N1-H1	120.87	125.03	4.17
N1-C2-O2	125.50	119.18	-6.33
N1-C2-N3	111.89	116.83	4.94
O2-C2-N3	122.60	123.99	1.39
C2-N3-H3	112.58	114.40	1.83
C2-N3-C4	126.39	121.82	-4.56
H3-N3-C4	121.04	123.78	2.74
N3-C4-N4	119.48	115.07	-4.41
N3-C4-C5	117.82	120.66	2.85
N4-C4-C5	122.71	124.27	1.56
C4-N4-H41	120.37	120.78	0.41
C4-N4-H42	122.87	120.95	-1.91
H41-N4-H42	116.76	118.27	1.51
C4-C5-H5	120.99	121.35	0.36
C4-C5-C6	117.93	118.14	0.20
H5-C5-C6	121.08	120.52	-0.56
N1-C6-C5	121.74	120.59	-1.15
N1-C6-H6	116.11	118.61	2.50
C5-C6-H6	122.15	120.80	-1.35

Terms	QM	MM	diff
C6-N1-C2-O2	-180.00	180.00	0.00
C6-N1-C2-N3	0.00	0.00	-0.00
H1-N1-C2-O2	0.00	0.00	0.00
H1-N1-C2-N3	180.00	-180.00	0.00
C2-N1-C6-C5	-0.00	0.00	0.00
C2-N1-C6-H6	180.00	180.00	0.00
H1-N1-C6-C5	-180.00	-180.00	-0.00
H1-N1-C6-H6	0.00	0.00	-0.00
N1-C2-N3-H3	180.00	-180.00	-0.00
N1-C2-N3-C4	-0.00	0.00	0.00
O2-C2-N3-H3	0.00	0.00	-0.00
O2-C2-N3-C4	180.00	180.00	0.00
C2-N3-C4-N4	180.00	180.00	-0.00
C2-N3-C4-C5	0.00	0.00	-0.00
H3-N3-C4-N4	0.00	0.00	-0.00
H3-N3-C4-C5	180.00	-180.00	0.00
N3-C4-N4-H41	180.00	-180.00	-0.00
N3-C4-N4-H42	-0.00	0.00	0.00
C5-C4-N4-H41	0.00	0.00	-0.00
C5-C4-N4-H42	180.00	-180.00	0.00
N3-C4-C5-H5	180.00	180.00	0.00
N3-C4-C5-C6	0.00	-0.00	0.00
N4-C4-C5-H5	-0.00	0.00	0.00
N4-C4-C5-C6	-180.00	-180.00	0.00
C4-C5-C6-N1	0.00	0.00	0.00
C4-C5-C6-H6	180.00	-180.00	0.00
H5-C5-C6-N1	180.00	180.00	0.00
H5-C5-C6-H6	0.00	0.00	0.00

Table 216: The statistics of vibrational frequencies of CYPp. Only the terms with the occupancies greater than 15% were shown. Refer to p.6 for the meanings of assig.

QM (MP2, scaled by 0.943)							MM						
Freq	Assig.	%	Assig.	%	Assig.	%	Freq	Assig.	%	Assig.	%	Assig.	%
149.1	at6r'	62.	pk6r	40.			163.5	pk6r	36.	at6r	29.	wC4N4	20.
174.1	at6r	82.	pk6r	17.			179.3	at6r	50.	at6r'	38.		
341.5	rC4N4	53.	rC=O	22.			339.3	rC4N4	63.	sN-C.r	17.	rC=O	15.
385.3	at6r'	50.	pk6r	23.	at6r	19.	340.1	wN-H	94.				
424.9	wHH2	88.					389.5	at6r'	27.	at6r	23.	wC4N4	16.
482.8	tC4N4	73.					454.3	tC4N4	43.	at6r'	24.	wHH2	18.
493.8	rC=O	41.	ad6r	28.	rC4N4	23.	518.6	rC=O	41.	sN-C.r	28.	rC4N4	20.
520.9	ad6r	52.	sN-C.r	21.			554.9	ad6r'	51.	sN-C.r	22.		
552.0	ad6r'	73.	sN-C.r	16.			561.5	pk6r	37.	wC4N4	24.		
622.6	wC4N4	79.					590.9	ad6r	55.	sN-C.r	21.		
656.2	wC=O	31.	pk6r	30.	wN3-H3	16.	626.5	wC-H	33.	wC=O	28.		
675.3	wN3-H3	55.	wN-H	45.			656.0	wC-H	48.	wC=O	19.		
712.0	wC=O	64.	wN-H	27.	wN3-H3	23.	721.5	wN3-H3	47.	wC=O	34.	tC4N4	16.
737.6	wC-H	91.					751.7	sN-C.r	40.	sC-C.r	20.	ad6r'	18.
768.1	sN-C.r	48.	sC-C.r	17.			850.0	wHH2	34.	wC4N4	32.	wN3-H3	25.
931.9	wC-H	111.					886.4	sN-C.r	46.	sC-C.r	16.		
943.2	sN-C.r	43.	td6r	32.			922.7	wC-H	115.				
994.0	td6r	37.	sC-C.r	36.			946.6	sN-C.r	42.	sC-C.r	23.	td6r	18.
1064.0	rNH2	51.	sN-C.r	30.			999.9	rNH2	43.	sN-C.r	27.		
1123.7	rC-H	33.	sN-C.r	28.	sC-C.r	20.	1047.5	rNH2	31.	td6r	19.		
1200.9	sN-C.r	72.	rC-H	17.			1159.7	rC-H	47.	sC-C.r	31.		
1244.1	rC-H	42.	sN-C.r	32.	rN-H	18.	1281.4	sN-C.r	35.	cNH2	17.	rN3-H3	15.
1375.7	rN3-H3	41.	sN-C.r	28.			1376.7	rN-H	33.	rN3-H3	26.		
1390.5	rC-H	51.	rN3-H3	22.			1405.3	rN-H	27.				
1471.4	rN-H	45.	sN-C.r	25.			1496.9	sN-C.r	43.				
1575.0	sN-C.r	43.	sC-C.r	19.			1549.0	rC-H	30.	sN-C.r	24.	sC-C.r	20.
1599.0	sN-C.r	25.	cNH2	23.	sC-C.r	22.	1565.1	cNH2	40.	sN-C.r	26.		
1657.3	cNH2	48.	sC-C.r	27.			1604.3	rC-H	27.	sN-C.r	26.	sC-C.r	20.
1708.0	sN-C.r	39.	sC-C.r	22.	cNH2	18.	1684.4	sC-C.r	25.	sN-C.r	24.	rC-H	20.
1844.8	sC=O	75.					1820.8	sC=O	39.	sN-C.r	31.		
3177.7	sC-H	99.					2994.4	sC-H	99.				
3199.8	sC-H	99.					2999.5	sC-H	99.				
3454.0	sN-H	98.					3327.2	sNH	98.				
3480.0	sNH	58.	sN-H	42.			3385.1	sN-H	97.				
3485.7	sN-H	60.	sNH	39.			3434.1	sN3-H3	99.				
3597.2	sN3-H3	100.					3459.1	sN-H	99.				

2.25 5-aminomethyl-2-thiouracil (SAU)

Figure 90: The energy-minimized structure of SAU.

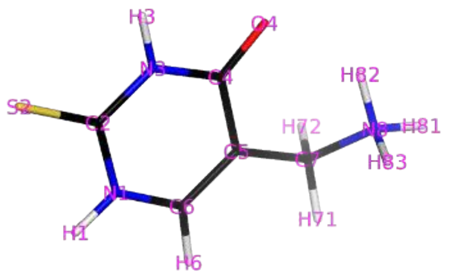

Table 217: The calculated geometric terms of SAU.

Terms	QM	MM	diff
N1-C2	1.395	1.392	-0.003
N1-C6	1.357	1.371	0.014
N1-H1	1.018	1.014	-0.004
C2-S2	1.627	1.642	0.014
C2-N3	1.392	1.382	-0.010
N3-H3	1.021	1.008	-0.014
N3-C4	1.376	1.381	0.005
C4-O4	1.247	1.231	-0.016
C4-C5	1.454	1.443	-0.011
C5-C6	1.362	1.379	0.016
C5-C7	1.492	1.493	0.001
C6-H6	1.088	1.095	0.008
C7-H71	1.092	1.101	0.009
C7-H72	1.094	1.102	0.008
C7-N8	1.515	1.498	-0.017
N8-H81	1.028	1.039	0.012
N8-H82	1.051	1.052	0.001
N8-H83	1.027	1.039	0.012
C2-N1-C6	125.12	123.06	-2.06
C2-N1-H1	114.70	113.98	-0.73
C6-N1-H1	120.16	122.94	2.78
N1-C2-S2	123.69	123.12	-0.57
N1-C2-N3	111.71	113.66	1.95
S2-C2-N3	124.60	123.22	-1.38
C2-N3-H3	115.80	116.85	1.04
C2-N3-C4	128.01	127.10	-0.91
H3-N3-C4	116.18	116.04	-0.14
N3-C4-O4	121.57	119.06	-2.51
N3-C4-C5	115.32	116.41	1.08
O4-C4-C5	123.09	124.46	1.37
C4-C5-C6	118.71	117.63	-1.08
C4-C5-C7	116.94	116.16	-0.77
C6-C5-C7	124.03	125.87	1.84
N1-C6-C5	121.10	122.08	0.98
N1-C6-H6	115.69	116.69	1.00
C5-C6-H6	123.20	121.21	-2.00
C5-C7-H71	113.02	109.78	-3.25
C5-C7-H72	111.64	108.07	-3.57
C5-C7-N8	108.75	112.07	3.32
H71-C7-H72	109.16	109.84	0.67
H71-C7-N8	108.82	108.89	0.07
H72-C7-N8	105.11	108.17	3.06
C7-N8-H81	112.68	111.20	-1.48
C7-N8-H82	104.70	106.18	1.49
C7-N8-H83	111.88	111.57	-0.31
H81-N8-H82	110.29	108.98	-1.30
H81-N8-H83	108.48	110.14	1.65
H82-N8-H83	108.71	108.63	-0.08
C6-N1-C2-S2	178.80	179.82	1.02
C6-N1-C2-N3	-1.74	-0.12	1.61
H1-N1-C2-S2	0.45	1.66	1.21
H1-N1-C2-N3	179.91	-178.29	1.80
C2-N1-C6-C5	1.50	1.95	0.45
C2-N1-C6-H6	-177.87	-176.63	1.24
H1-N1-C6-C5	179.76	179.95	0.18
H1-N1-C6-H6	0.40	1.37	0.97
N1-C2-N3-H3	-179.06	178.39	2.54
N1-C2-N3-C4	0.70	-0.44	-1.14
S2-C2-N3-H3	0.39	-1.56	-1.95
S2-C2-N3-C4	-179.85	179.62	0.54
C2-N3-C4-O4	-178.08	-177.74	0.34
C2-N3-C4-C5	0.52	-0.72	-1.24
H3-N3-C4-O4	1.68	3.42	1.74
H3-N3-C4-C5	-179.71	-179.56	0.16
N3-C4-C5-C6	-0.86	2.40	3.27
N3-C4-C5-C7	172.90	176.11	3.22
O4-C4-C5-C6	177.71	179.25	1.53
O4-C4-C5-C7	-8.53	-7.04	1.48
C4-C5-C6-N1	-0.05	-3.06	-3.01
C4-C5-C6-H6	179.26	175.46	-3.81
C7-C5-C6-N1	-173.34	-176.09	-2.75
C7-C5-C6-H6	5.98	2.42	-3.55
C4-C5-C7-N8	50.33	51.85	1.53
C6-C5-C7-N8	-136.28	-135.02	1.25

Figure 91: The PES scan for flexible dihedral corresponding to SAU.

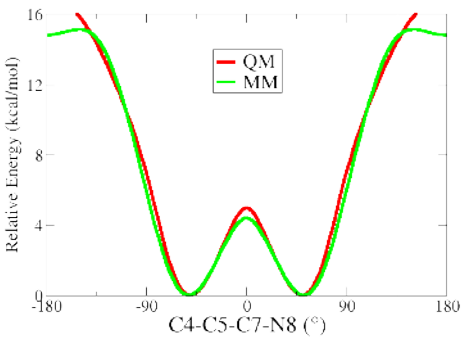

Table 218: The statistics of vibrational frequencies of SAU. Only the terms with the occupancies greater than 15% were shown. Refer to p.6 for the meanings of assig.

QM (MP2, scaled by 0.943)							MM						
Freq	Assig.	%	Assig.	%	Assig.	%	Freq	Assig.	%	Assig.	%	Assig.	%
69.6	at6r'	63.	at6r	24.			81.3	at6r'	39.	wC7-C5	22.	tCCCN	17.
131.4	tCCCN	78.	at6r	15.			133.9	tCCCN	70.	at6r	25.		
153.5	pk6r	66.	at6r	41.			184.1	pk6r	38.	at6r	28.	wC=O	17.
219.6	rC=S	28.	rC7-C5	22.	wC7-C5	17.	215.5	rC=S	35.	rC7-C5	29.	sN-C.ar	17.
250.0	wC7-C5	39.	rC7-C5	19.			243.8	tCCNH	68.				
323.2	rC7-C5	35.	rC=S	33.			270.6	wC7-C5	26.	tCCNH	17.		
349.3	tCCNH	22.	ad6r'	22.			332.0	rC7-C5	26.	sN-C.ar	15.		
364.7	tCCNH	68.					340.7	rC7-C5	27.				
382.7	at6r'	54.	pk6r	25.			390.5	at6r'	26.	wC7-C5	17.		
471.0	cCCN	34.	ad6r'	19.	rC=O	18.	489.3	cCCN	40.				
508.1	rC=O	26.	sN-C.ar	20.			552.4	rC=O	27.				
567.2	ad6r	42.					588.2	wC=S	38.				
588.9	wC=S	90.	pk6r	15.			593.5	ad6r	39.	sC-C.ar	22.		
651.1	wN-H	83.	wC=O	17.			626.7	wN-H	91.	wC=S	18.		
692.0	wC=O	31.	wN-H	23.	pk6r	21.	684.7	sC5-C7	19.	td6r	15.	sN-C.ar	15.
710.6	ad6r'	21.	sC-C.ar	20.	sN-C.ar	17.	731.8	wN-H	95.	wC=O	16.		
731.2	wN-H	87.	wC=O	18.			745.0	ad6r'	29.	sC-C.ar	27.	sN-C.ar	21.
738.0	sC5-C7	19.	wC=O	18.	sC-C.ar	18.	778.3	wC=O	40.	pk6r	39.		
866.0	rCH2	57.	rNH3'	27.			843.4	rCH2	27.	sN-C.ar	22.	wC-H	19.
906.1	wC-H	94.					877.6	wC-H	29.	sN-C.ar	19.		
928.7	sC-N	73.					904.7	wC-H	42.	rNH3'	25.	rCH2	19.
951.7	sN-C.ar	35.	td6r	30.			969.3	sC-N	37.	rNH3	30.	sN-C.ar	20.
1075.1	rNH3	37.					1019.7	rNH3	29.	rNH3'	20.		
1108.3	iCH2	25.	rNH3'	24.	rNH3	21.	1029.4	sC-N	41.	sN-C.ar	28.		
1174.7	sC=S	25.	sN-C.ar	23.			1050.0	rNH3'	33.	rCH2	25.	rNH3	19.
1199.9	sN-C.ar	42.	rC-H	18.	sC=S	16.	1179.5	sN-C.ar	21.	sC5-C7	16.		
1239.1	sN-C.ar	40.	rN-H	26.			1242.6	rN-H	27.	sC=S	25.		
1260.6	sN-C.ar	50.	sC5-C7	15.			1311.1	rC-H	28.	sC-C.ar	22.		
1320.2	iCH2	32.	rNH3'	18.			1321.9	sN-C.ar	45.	rN-H	31.		
1360.6	rC-H	30.	iCH2	26.	sN-C.ar	18.	1358.8	iCH2	76.				
1391.7	rN-H	55.	sN-C.ar	18.			1466.7	sdNH3	69.	cCH2	24.		
1401.9	wCH2	61.					1472.4	rN-H	38.	sN-C.ar	20.		
1450.0	sN-C.ar	20.	sC-C.ar	19.			1478.2	cCH2	34.	rN-H	16.		
1482.1	sdNH3	76.					1501.5	wCH2	37.	cCH2	26.		
1498.2	cCH2	100.					1522.7	wCH2	18.	sC-C.ar	16.	rC-H	16.
1571.8	rN-H	44.	sN-C.ar	32.			1596.9	rN-H	36.	sN-C.ar	22.		
1630.2	adNH3'	59.					1621.6	adNH3'	77.				
1664.1	sC-C.ar	35.	adNH3'	17.			1636.1	adNH3	81.	adNH3'	16.		
1686.2	adNH3	81.					1675.5	sC-C.ar	24.	rC-H	21.	sC=O	16.
1726.1	sC=O	37.	sC-C.ar	17.			1819.5	sC=O	39.				
3006.0	ssNH3	45.	asNH3'	40.			2773.9	ssCH2	99.				
3043.7	ssCH2	94.					2807.8	asCH2	100.				
3115.4	asCH2	95.					2993.8	sC-H	99.				
3148.0	sC-H	99.					3151.7	ssNH3	99.				
3362.2	ssNH3	54.	asNH3'	33.			3251.9	asNH3'	81.	asNH3	18.		
3436.4	asNH3	74.	asNH3'	26.			3259.0	asNH3	81.	asNH3'	18.		
3451.5	sN-H	100.					3455.1	sN-H	99.				
3484.9	sN-H	100.					3461.2	sN-H	99.				

2.26 N2-methylguanine (2MG)

Figure 92: The energy-minimized structure of 2MG.

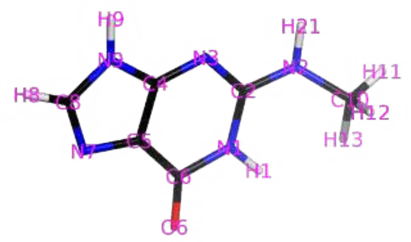

Table 219: The calculated geometric terms of 2MG.

Terms	QM	MM	diff
N9-C8	1.375	1.373	-0.003
N9-C4	1.371	1.347	-0.024
N9-H9	1.013	1.001	-0.013
C8-H8	1.082	1.093	0.011
C8-N7	1.323	1.322	-0.002
N7-C5	1.378	1.390	0.011
C5-C6	1.440	1.417	-0.023
C5-C4	1.395	1.403	0.008
C6-O6	1.226	1.232	0.006
C6-N1	1.432	1.383	-0.049
N1-H1	1.016	0.997	-0.020
N1-C2	1.372	1.380	0.008
C2-N2	1.376	1.352	-0.025
C2-N3	1.316	1.345	0.029
N2-H21	1.014	1.015	0.001
N2-C10	1.456	1.480	0.024
N3-C4	1.363	1.333	-0.029
C10-H11	1.090	1.113	0.023
C10-H12	1.097	1.113	0.016
C10-H13	1.094	1.111	0.017
C8-N9-C4	107.02	107.26	0.24
C8-N9-H9	127.70	130.18	2.47
C4-N9-H9	125.27	122.57	-2.71
N9-C8-H8	121.89	123.06	1.17
N9-C8-N7	112.93	112.69	-0.24
H8-C8-N7	125.18	124.25	-0.93
C8-N7-C5	103.79	104.35	0.55
N7-C5-C6	129.61	131.82	2.21
N7-C5-C4	111.62	109.65	-1.97
C6-C5-C4	118.77	118.53	-0.24
C5-C6-O6	131.25	130.71	-0.55
C5-C6-N1	109.13	112.35	3.22
O6-C6-N1	119.62	116.94	-2.67
C6-N1-H1	112.80	114.91	2.11
C6-N1-C2	127.03	126.45	-0.58
H1-N1-C2	120.12	118.62	-1.50
N1-C2-N2	117.34	121.58	4.24
N1-C2-N3	123.73	120.64	-3.09
N2-C2-N3	118.89	117.78	-1.11
C2-N2-H21	110.21	113.58	3.37
C2-N2-C10	121.78	125.91	4.13
H21-N2-C10	115.98	114.17	-1.80
C2-N3-C4	111.64	115.06	3.42
N9-C4-C5	104.64	106.06	1.42
N9-C4-N3	125.66	126.97	1.31
C5-C4-N3	129.69	126.98	-2.72
N2-C10-H11	107.90	110.36	2.46
N2-C10-H12	113.11	110.80	-2.31
N2-C10-H13	110.65	111.99	1.34
H11-C10-H12	108.82	107.90	-0.92
H11-C10-H13	107.09	107.36	0.27
H12-C10-H13	109.08	108.28	-0.79

Figure 93: The PES scan for flexible dihedral corresponding to 2MG.

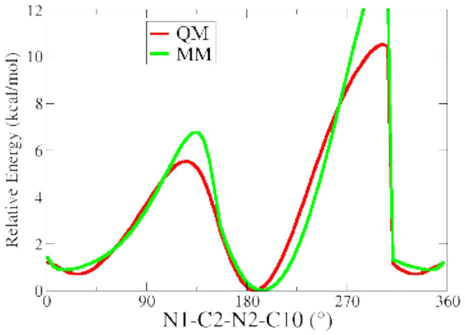

2.27 2-thiocytosine (2SC)

Figure 94: The energy-minimized structure of 2SC.

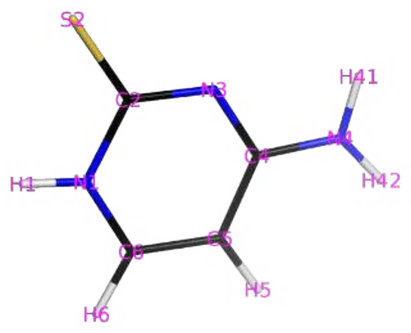

Table 220: The calculated geometric terms of 2SC.

Terms	QM	MM	diff
N1-C2	1.401	1.401	0.000
N1-C6	1.356	1.402	0.046
N1-H1	1.016	1.007	-0.010
C2-S2	1.658	1.650	-0.008
C2-N3	1.367	1.357	-0.010
N3-C4	1.325	1.336	0.012
C4-N4	1.365	1.325	-0.040
C4-C5	1.432	1.383	-0.049
N4-H41	1.014	0.995	-0.018
N4-H42	1.011	0.991	-0.019
C5-H5	1.084	1.073	-0.010
C5-C6	1.362	1.403	0.041
C6-H6	1.086	1.083	-0.003
C2-N1-C6	124.07	121.38	-2.69
C2-N1-H1	115.03	112.96	-2.07
C6-N1-H1	120.90	125.66	4.76
N1-C2-S2	119.01	119.74	0.73
N1-C2-N3	116.24	117.93	1.69
S2-C2-N3	124.75	122.33	-2.42
C2-N3-C4	120.31	120.95	0.64
N3-C4-N4	116.44	115.56	-0.88
N3-C4-C5	123.95	124.12	0.17
N4-C4-C5	119.56	120.32	0.77
C4-N4-H41	114.87	112.16	-2.71
C4-N4-H42	119.14	125.28	6.14
H41-N4-H42	116.50	122.57	6.07
C4-C5-H5	122.70	121.06	-1.64
C4-C5-C6	115.79	116.69	0.91
H5-C5-C6	121.51	122.24	0.73
N1-C6-C5	119.64	118.93	-0.71
N1-C6-H6	116.64	118.88	2.25
C5-C6-H6	123.72	122.19	-1.53

2.28 N4-acetylcytosine (4AC)

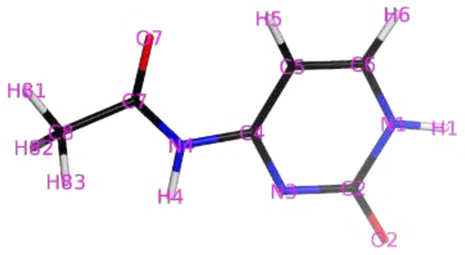

Figure 95: The energy-minimized structure of 4AC.

Table 221: The calculated geometric terms of 4AC.

Terms	QM	MM	diff
N1-C2	1.413	1.384	-0.029
N1-C6	1.357	1.365	0.007
N1-H1	1.017	1.005	-0.013
C2-O2	1.231	1.224	-0.007
C2-N3	1.383	1.349	-0.035
N3-C4	1.321	1.351	0.030
C4-N4	1.395	1.365	-0.030
C4-C5	1.427	1.416	-0.011
N4-H4	1.019	0.988	-0.031
N4-C7	1.388	1.323	-0.066
C5-H5	1.080	1.087	0.007
C5-C6	1.366	1.366	-0.001
C6-H6	1.086	1.095	0.009
C7-O7	1.232	1.222	-0.010
C7-C8	1.511	1.480	-0.031
C8-H81	1.090	1.111	0.021
C8-H82	1.094	1.110	0.016
C8-H83	1.095	1.110	0.015
C2-N1-C6	123.86	121.23	-2.63
C2-N1-H1	114.98	112.04	-2.95
C6-N1-H1	121.15	126.73	5.58
N1-C2-O2	119.56	117.43	-2.13
N1-C2-N3	115.64	118.99	3.35
O2-C2-N3	124.80	123.58	-1.22
C2-N3-C4	120.05	120.88	0.84
N3-C4-N4	112.91	113.05	0.15
N3-C4-C5	125.03	120.89	-4.14
N4-C4-C5	122.06	126.06	4.00
C4-N4-H4	112.01	105.34	-6.67
C4-N4-C7	129.44	129.41	-0.03
H4-N4-C7	118.23	125.25	7.02
C4-C5-H5	122.49	122.37	-0.12
C4-C5-C6	115.15	117.74	2.59
H5-C5-C6	122.36	119.90	-2.46
N1-C6-C5	120.26	120.26	0.00
N1-C6-H6	116.69	118.75	2.06
C5-C6-H6	123.04	120.98	-2.06
N4-C7-O7	123.52	123.86	0.33
N4-C7-C8	113.46	115.65	2.19
O7-C7-C8	123.01	120.49	-2.52
C7-C8-H81	108.33	109.41	1.08
C7-C8-H82	111.63	110.04	-1.58
C7-C8-H83	109.88	110.04	0.17
H81-C8-H82	109.72	108.97	-0.75
H81-C8-H83	108.96	108.97	0.01
H82-C8-H83	108.30	109.39	1.08

Figure 96: The PES scan for flexible dihedral corresponding to 4AC.

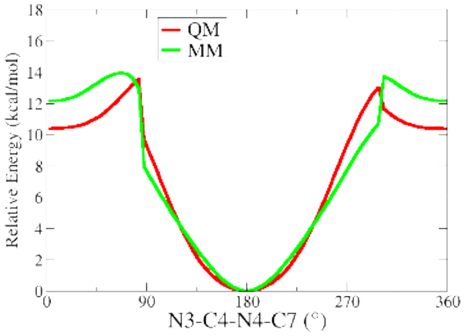

2.29 5-methylaminomethyl uracil (5AU)

Figure 97: The energy-minimized structure of 5AU.

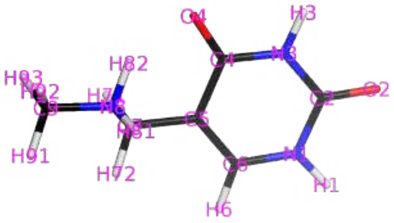

Table 222: The calculated geometric terms of 5AU.

Terms	QM	MM	diff
N1-C2	1.375	1.377	0.001
N1-C6	1.421	1.365	-0.056
N1-H1	1.015	1.011	-0.004
C2-O2	1.236	1.221	-0.016
C2-N3	1.390	1.360	-0.030
N3-H3	1.016	1.003	-0.013
N3-C4	1.397	1.373	-0.024
C4-O4	1.291	1.230	-0.061
C4-C5	1.395	1.450	0.055
C5-C6	1.431	1.378	-0.052
C5-C7	1.483	1.481	-0.002
C6-H6	1.089	1.096	0.008
C7-H71	1.099	1.105	0.006
C7-H72	1.095	1.104	0.009
C7-N8	1.517	1.499	-0.018
N8-H81	1.026	1.010	-0.015
N8-H82	1.086	1.020	-0.066
N8-C9	1.480	1.502	0.022
C9-H91	1.092	1.110	0.018
C9-H92	1.091	1.111	0.020
C9-H93	1.090	1.111	0.020
C2-N1-C6	124.66	122.30	-2.36
C2-N1-H1	112.60	112.83	0.23
C6-N1-H1	118.71	124.81	6.10
N1-C2-O2	123.52	121.94	-1.57
N1-C2-N3	114.25	116.09	1.83
O2-C2-N3	122.19	121.97	-0.23
C2-N3-H3	116.43	115.80	-0.64
C2-N3-C4	125.20	125.57	0.37
H3-N3-C4	117.69	118.63	0.94
N3-C4-O4	117.89	118.90	1.01
N3-C4-C5	117.20	116.88	-0.32
O4-C4-C5	124.89	124.14	-0.75
C4-C5-C6	121.00	117.39	-3.61
C4-C5-C7	115.74	117.81	2.08
C6-C5-C7	122.76	124.68	1.92
N1-C6-C5	114.75	121.74	6.99
N1-C6-H6	113.65	116.87	3.22
C5-C6-H6	120.75	121.38	0.63
C5-C7-H71	113.78	110.55	-3.23
C5-C7-H72	112.92	111.78	-1.15
C5-C7-N8	107.43	108.24	0.81
H71-C7-H72	109.17	112.28	3.11
H71-C7-N8	104.31	106.76	2.45
H72-C7-N8	108.75	106.97	-1.79
C7-N8-H81	107.88	109.76	1.88
C7-N8-H82	99.63	106.42	6.79
C7-N8-C9	116.20	115.50	-0.70
H81-N8-H82	107.17	104.59	-2.58
H81-N8-C9	111.94	110.38	-1.56
H82-N8-C9	113.04	109.56	-3.48
N8-C9-H91	109.71	107.38	-2.33
N8-C9-H92	109.36	107.15	-2.22
N8-C9-H93	108.20	107.23	-0.96
H91-C9-H92	110.13	111.61	1.47
H91-C9-H93	109.89	111.77	1.89
H92-C9-H93	109.52	111.40	1.88

Figure 98: The PES scan for flexible dihedral corresponding to 5AU.

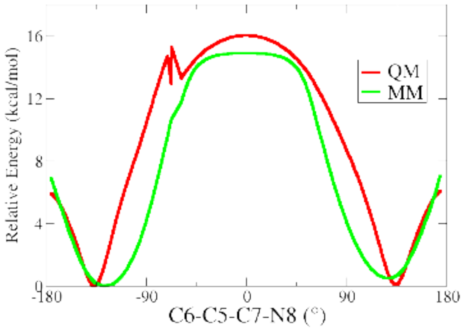

2.30 5-carboxymethyl uracil (5CU)

Figure 99: The energy-minimized structure of 5CU.

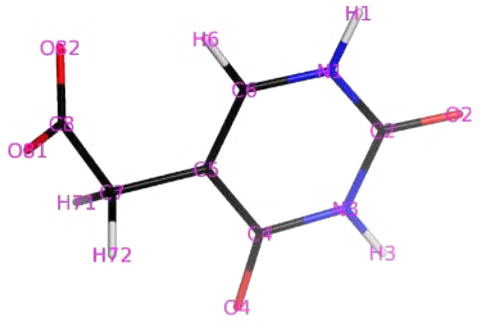

Table 223: The calculated geometric terms of 5CU.

Terms	QM	MM	diff
N1-C2	1.373	1.374	0.000
N1-C6	1.388	1.376	-0.012
N1-H1	1.014	1.005	-0.009
C2-O2	1.240	1.226	-0.015
C2-N3	1.381	1.359	-0.022
N3-H3	1.018	0.997	-0.021
N3-C4	1.414	1.379	-0.035
C4-O4	1.239	1.232	-0.007
C4-C5	1.457	1.452	-0.005
C5-C6	1.361	1.379	0.019
C5-C7	1.507	1.517	0.011
C6-H6	1.088	1.096	0.008
C7-H71	1.100	1.110	0.010
C7-H72	1.094	1.110	0.016
C7-C8	1.556	1.528	-0.029
C8-O81	1.263	1.255	-0.008
C8-O82	1.277	1.258	-0.019
C2-N1-C6	124.25	121.66	-2.59
C2-N1-H1	115.49	115.31	-0.18
C6-N1-H1	119.97	123.03	3.06
N1-C2-O2	124.07	122.01	-2.07
N1-C2-N3	112.30	116.15	3.86
O2-C2-N3	123.62	121.84	-1.78
C2-N3-H3	115.64	117.25	1.61
C2-N3-C4	128.25	126.19	-2.05
H3-N3-C4	115.65	116.56	0.91
N3-C4-O4	118.55	117.18	-1.37
N3-C4-C5	114.71	116.46	1.75
O4-C4-C5	126.73	126.36	-0.37
C4-C5-C6	117.68	117.13	-0.56
C4-C5-C7	119.14	119.01	-0.13
C6-C5-C7	123.11	123.86	0.75
N1-C6-C5	122.42	122.41	-0.02
N1-C6-H6	117.49	118.11	0.62
C5-C6-H6	120.08	119.48	-0.61
C5-C7-H71	108.66	107.81	-0.85
C5-C7-H72	108.83	108.27	-0.55
C5-C7-C8	113.85	115.72	1.86
H71-C7-H72	107.26	108.61	1.36
H71-C7-C8	108.59	107.50	-1.08
H72-C7-C8	109.45	108.75	-0.70
C7-C8-O81	116.12	115.76	-0.36
C7-C8-O82	115.13	116.09	0.96
O81-C8-O82	128.75	128.15	-0.60

Figure 100: The PES scan for flexible dihedral corresponding to 5CU.

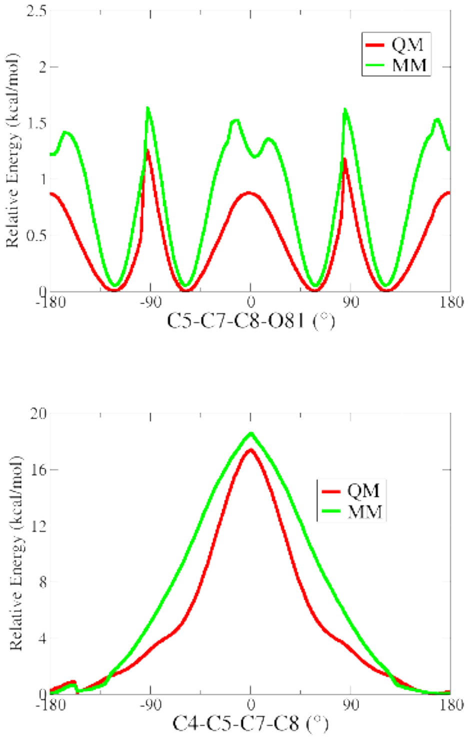

2.31 5-methylcytosine (5MC)

Figure 101: The energy-minimized structure of 5MC.

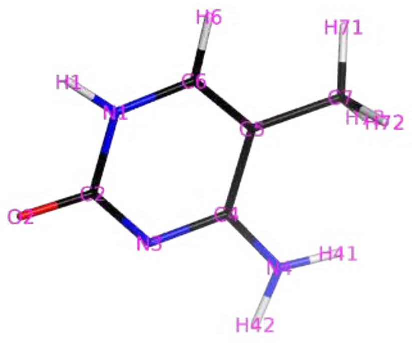

Table 224: The calculated geometric terms of 5MC.

Terms	QM	MM	diff
N1-C2	1.414	1.384	-0.030
N1-C6	1.360	1.364	0.004
N1-H1	1.015	1.004	-0.011
C2-O2	1.227	1.225	-0.003
C2-N3	1.381	1.349	-0.033
N3-C4	1.318	1.337	0.019
C4-N4	1.374	1.329	-0.044
C4-C5	1.443	1.449	0.006
N4-H41	1.011	0.991	-0.020
N4-H42	1.014	0.995	-0.019
C5-C6	1.362	1.370	0.008
C5-C7	1.500	1.504	0.004
C6-H6	1.087	1.093	0.005
C7-H71	1.093	1.113	0.019
C7-H72	1.097	1.113	0.016
C7-H73	1.095	1.113	0.017
C2-N1-C6	124.12	121.27	-2.85
C2-N1-H1	114.74	111.94	-2.80
C6-N1-H1	121.14	126.79	5.65
N1-C2-O2	119.20	117.13	-2.07
N1-C2-N3	115.57	119.33	3.76
O2-C2-N3	125.23	123.54	-1.69
C2-N3-C4	120.05	120.83	0.78
N3-C4-N4	116.40	117.03	0.63
N3-C4-C5	125.25	121.47	-3.78
N4-C4-C5	118.26	121.49	3.23
C4-N4-H41	118.34	123.64	5.30
C4-N4-H42	113.53	114.43	0.90
H41-N4-H42	115.35	121.93	6.58
C4-C5-C6	114.60	116.13	1.53
C4-C5-C7	122.44	121.89	-0.55
C6-C5-C7	122.96	121.56	-1.39
N1-C6-C5	120.42	120.90	0.48
N1-C6-H6	116.66	118.09	1.43
C5-C6-H6	122.92	121.02	-1.90
C5-C7-H71	110.48	111.25	0.78
C5-C7-H72	111.94	110.70	-1.24
C5-C7-H73	111.19	111.55	0.36
H71-C7-H72	107.29	107.82	0.53
H71-C7-H73	107.96	107.07	-0.89
H72-C7-H73	107.80	108.28	0.48

2.32 6-methyladenine (6MA)

Figure 102: The energy-minimized structure of 6MA.

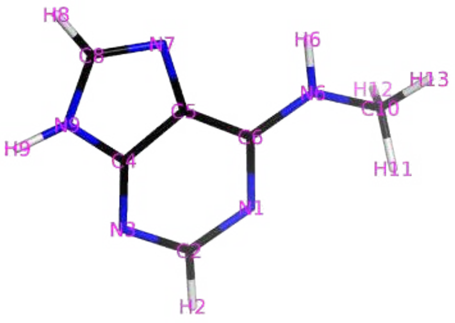

Table 225: The calculated geometric terms of 6MA.

Terms	QM	MM	diff
N9-C8	1.371	1.370	-0.001
N9-C4	1.378	1.346	-0.032
N9-H9	1.013	0.999	-0.014
C8-H8	1.083	1.093	0.010
C8-N7	1.327	1.325	-0.002
N7-C5	1.380	1.404	0.024
C5-C6	1.413	1.404	-0.009
C5-C4	1.397	1.394	-0.003
C6-N6	1.355	1.374	0.019
C6-N1	1.344	1.377	0.033
N6-H6	1.013	1.019	0.006
N6-C10	1.452	1.479	0.027
C10-H11	1.091	1.113	0.022
C10-H12	1.090	1.112	0.022
C10-H13	1.096	1.112	0.016
N1-C2	1.354	1.363	0.010
C2-H2	1.088	1.095	0.007
C2-N3	1.338	1.359	0.021
N3-C4	1.345	1.326	-0.019
C8-N9-C4	106.92	107.58	0.66
C8-N9-H9	127.42	130.98	3.56
C4-N9-H9	125.66	121.44	-4.22
N9-C8-H8	121.77	122.48	0.71
N9-C8-N7	113.47	113.07	-0.40
H8-C8-N7	124.77	124.46	-0.31
C8-N7-C5	103.25	103.22	-0.03
N7-C5-C6	131.80	133.56	1.76
N7-C5-C4	112.08	110.35	-1.73
C6-C5-C4	116.11	116.09	-0.02
C5-C6-N6	121.43	121.27	-0.16
C5-C6-N1	118.77	117.89	-0.88
N6-C6-N1	119.77	120.83	1.06
C6-N6-H6	115.48	117.70	2.22
C6-N6-C10	121.71	125.34	3.63
H6-N6-C10	119.13	114.31	-4.82
N6-C10-H11	108.24	111.24	3.00
N6-C10-H12	109.33	110.70	1.37
N6-C10-H13	112.25	110.74	-1.51
H11-C10-H12	109.36	107.92	-1.45
H11-C10-H13	109.39	107.67	-1.72
H12-C10-H13	108.23	108.45	0.22
C6-N1-C2	118.20	120.19	1.99
N1-C2-H2	115.00	117.45	2.45
N1-C2-N3	129.13	124.85	-4.28
H2-C2-N3	115.87	117.71	1.84
C2-N3-C4	110.67	113.14	2.46
N9-C4-C5	104.28	105.78	1.50
N9-C4-N3	128.61	126.38	-2.23
C5-C4-N3	127.11	127.84	0.73

Figure 103: The PES scan for flexible dihedral corresponding to 6MA.

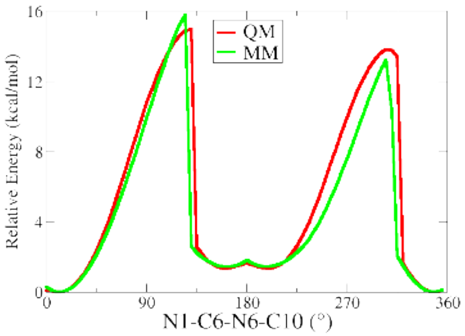

2.33 N2,N2-dimethyl guanine (M2G)

Figure 104: The energy-minimized structure of M2G.

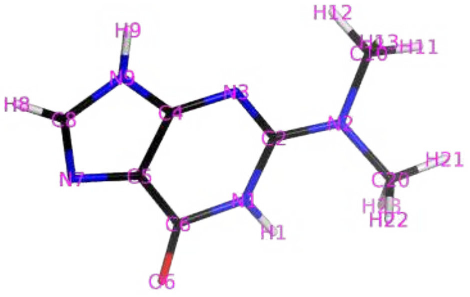

Table 226: The calculated geometric terms of M2G.

Terms	QM	MM	diff
N9-C8	1.375	1.374	-0.001
N9-C4	1.372	1.370	-0.001
N9-H9	1.013	1.010	-0.003
C8-H8	1.082	1.091	0.009
C8-N7	1.324	1.325	0.001
N7-C5	1.378	1.391	0.012
C5-C6	1.439	1.417	-0.022
C5-C4	1.394	1.408	0.014
C6-O6	1.226	1.230	0.004
C6-N1	1.430	1.379	-0.051
N1-H1	1.015	0.998	-0.017
N1-C2	1.378	1.383	0.005
C2-N2	1.378	1.375	-0.003
C2-N3	1.318	1.358	0.040
N2-C10	1.460	1.462	0.003
N2-C20	1.457	1.463	0.005
N3-C4	1.362	1.343	-0.019
C10-H11	1.092	1.113	0.021
C10-H12	1.088	1.114	0.026
C10-H13	1.098	1.114	0.016
C20-H21	1.090	1.114	0.024
C20-H22	1.099	1.114	0.015
C20-H23	1.094	1.114	0.020
C8-N9-C4	107.03	106.54	-0.49
C8-N9-H9	127.70	127.23	-0.47
C4-N9-H9	125.27	126.23	0.96
N9-C8-H8	121.91	122.06	0.16
N9-C8-N7	112.92	113.34	0.42
H8-C8-N7	125.17	124.59	-0.58
C8-N7-C5	103.76	104.40	0.64
N7-C5-C6	129.84	131.20	1.35
N7-C5-C4	111.70	109.89	-1.81
C6-C5-C4	118.46	118.91	0.45
C5-C6-O6	131.20	130.60	-0.59
C5-C6-N1	109.29	112.39	3.10
O6-C6-N1	119.51	117.01	-2.50
C6-N1-H1	112.06	115.06	2.99
C6-N1-C2	127.42	127.21	-0.21
H1-N1-C2	120.49	117.73	-2.75
N1-C2-N2	117.40	117.86	0.46
N1-C2-N3	122.85	119.64	-3.21
N2-C2-N3	119.72	122.34	2.63
C2-N2-C10	115.97	119.73	3.76
C2-N2-C20	119.27	120.31	1.04
C10-N2-C20	116.19	116.93	0.74
C2-N3-C4	112.03	115.64	3.62
N9-C4-C5	104.59	105.83	1.24
N9-C4-N3	125.47	128.00	2.54
C5-C4-N3	129.93	126.16	-3.77
N2-C10-H11	108.08	111.24	3.16
N2-C10-H12	108.98	111.61	2.63
N2-C10-H13	112.24	111.18	-1.06
H11-C10-H12	109.55	106.99	-2.56
H11-C10-H13	109.46	107.72	-1.74
H12-C10-H13	108.50	107.90	-0.61
N2-C20-H21	107.93	110.98	3.05
N2-C20-H22	112.85	112.00	-0.85
N2-C20-H23	111.14	111.28	0.13
H21-C20-H22	108.59	106.79	-1.80
H21-C20-H23	106.84	107.01	0.17
H22-C20-H23	109.27	108.55	-0.72

Figure 105: The PES scan for flexible dihedral corresponding to M2G.

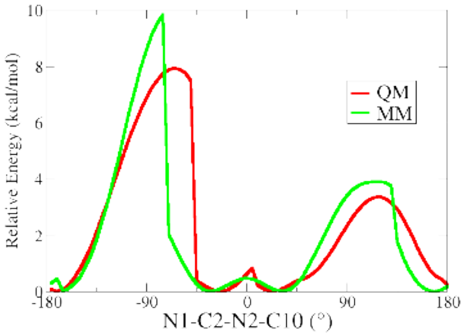

2.34 5-methyldihydro uracil (MDU)

Figure 106: The energy-minimized structure of MDU.

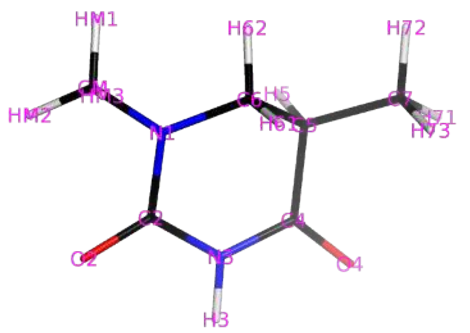

Table 227: The calculated geometric terms of MDU.

Terms	QM	MM	diff
N1-C2	1.373	1.372	-0.001
N1-C6	1.457	1.454	-0.003
N1-CM	1.456	1.449	-0.007
C2-O2	1.228	1.225	-0.003
C2-N3	1.408	1.364	-0.044
N3-H3	1.017	0.991	-0.026
N3-C4	1.386	1.331	-0.055
C4-O4	1.226	1.226	0.000
C4-C5	1.516	1.501	-0.015
C5-H5	1.100	1.111	0.011
C5-C6	1.523	1.537	0.014
C5-C7	1.523	1.552	0.029
C6-H61	1.103	1.113	0.010
C6-H62	1.095	1.113	0.018
C7-H71	1.091	1.112	0.022
C7-H72	1.094	1.111	0.017
C7-H73	1.094	1.111	0.017
C2-N1-C6	118.66	118.74	0.08
C2-N1-CM	117.12	118.00	0.89
C6-N1-CM	117.57	119.17	1.59
N1-C2-O2	125.44	125.22	-0.23
N1-C2-N3	114.08	114.48	0.40
O2-C2-N3	120.47	120.28	-0.18
C2-N3-H3	114.34	116.24	1.91
C2-N3-C4	128.50	128.17	-0.33
H3-N3-C4	116.48	115.51	-0.98
N3-C4-O4	121.46	120.29	-1.17
N3-C4-C5	113.97	118.42	4.46
O4-C4-C5	124.57	121.28	-3.29
C4-C5-H5	106.17	106.86	0.69
C4-C5-C6	109.47	106.74	-2.73
C4-C5-C7	111.03	112.12	1.09
H5-C5-C6	107.46	108.72	1.26
H5-C5-C7	109.69	109.08	-0.61
C6-C5-C7	112.74	113.08	0.33
N1-C6-C5	110.54	111.13	0.59
N1-C6-H61	111.16	109.44	-1.73
N1-C6-H62	107.79	109.00	1.21
C5-C6-H61	110.08	111.19	1.11
C5-C6-H62	109.75	108.59	-1.17
H61-C6-H62	107.44	107.40	-0.04
C5-C7-H71	110.18	110.64	0.47
C5-C7-H72	110.50	110.46	-0.04
C5-C7-H73	110.61	111.04	0.42
H71-C7-H72	109.02	108.02	-0.99
H71-C7-H73	107.87	108.13	0.26
H72-C7-H73	108.60	108.45	-0.15

Figure 107: The PES scan for flexible dihedral corresponding to MDU.

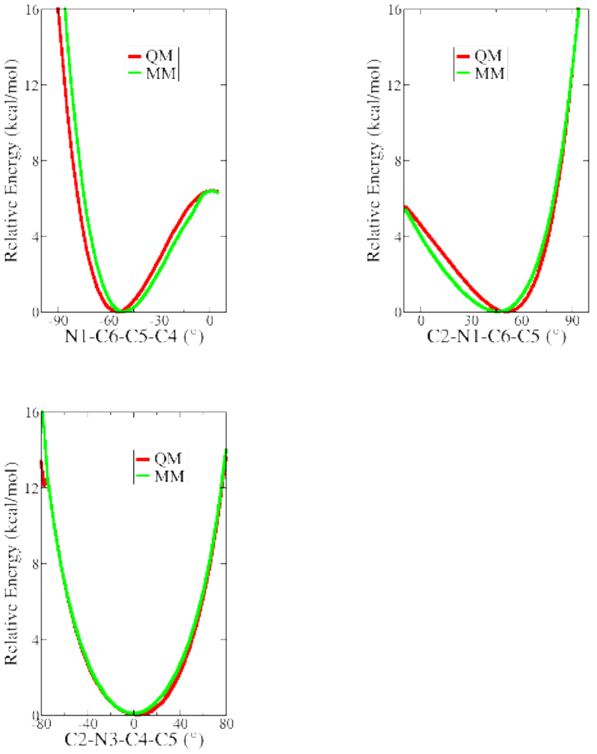

2.35 N4,N4-dimethylcytosine (TMC)

Figure 108: The energy-minimized structure of TMC.

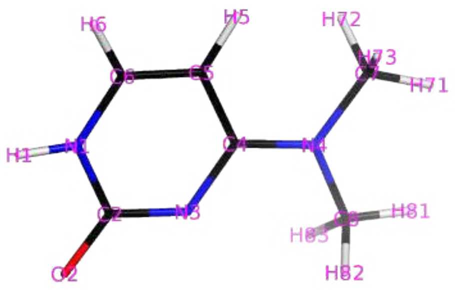

Table 228: The calculated geometric terms of TMC.

Terms	QM	MM	diff
N1-C2	1.418	1.379	-0.039
N1-C6	1.357	1.357	0.001
N1-H1	1.014	1.003	-0.012
C2-O2	1.228	1.224	-0.004
C2-N3	1.376	1.348	-0.029
N3-C4	1.327	1.360	0.034
C4-N4	1.368	1.377	0.009
C4-C5	1.442	1.441	-0.002
N4-C7	1.455	1.460	0.006
N4-C8	1.459	1.463	0.003
C5-H5	1.080	1.089	0.008
C5-C6	1.360	1.365	0.005
C6-H6	1.086	1.093	0.007
C7-H71	1.098	1.113	0.015
C7-H72	1.091	1.114	0.023
C7-H73	1.092	1.114	0.022
C8-H81	1.097	1.111	0.014
C8-H82	1.088	1.114	0.027
C8-H83	1.092	1.114	0.022
C2-N1-C6	123.52	121.38	-2.14
C2-N1-H1	114.94	111.71	-3.22
C6-N1-H1	121.54	126.91	5.37
N1-C2-O2	118.68	116.99	-1.70
N1-C2-N3	116.01	119.36	3.35
O2-C2-N3	125.31	123.66	-1.65
C2-N3-C4	120.86	121.56	0.70
N3-C4-N4	116.84	119.23	2.39
N3-C4-C5	123.18	119.02	-4.16
N4-C4-C5	119.97	121.75	1.78
C4-N4-C7	120.75	120.94	0.19
C4-N4-C8	118.17	119.86	1.70
C7-N4-C8	117.18	119.20	2.02
C4-C5-H5	123.79	122.24	-1.55
C4-C5-C6	116.28	118.26	1.98
H5-C5-C6	119.92	119.50	-0.42
N1-C6-C5	120.12	120.42	0.31
N1-C6-H6	116.80	118.22	1.42
C5-C6-H6	123.09	121.36	-1.73
N4-C7-H71	112.66	111.32	-1.34
N4-C7-H72	108.10	111.27	3.18
N4-C7-H73	110.93	111.27	0.34
H71-C7-H72	108.42	107.25	-1.17
H71-C7-H73	109.08	107.25	-1.83
H72-C7-H73	107.49	108.28	0.79
N4-C8-H81	111.78	111.77	-0.01
N4-C8-H82	109.03	111.12	2.09
N4-C8-H83	108.31	111.12	2.81
H81-C8-H82	108.24	107.53	-0.71
H81-C8-H83	109.36	107.53	-1.82
H82-C8-H83	110.12	107.55	-2.57

Figure 109: The PES scan for flexible dihedral corresponding to TMC.

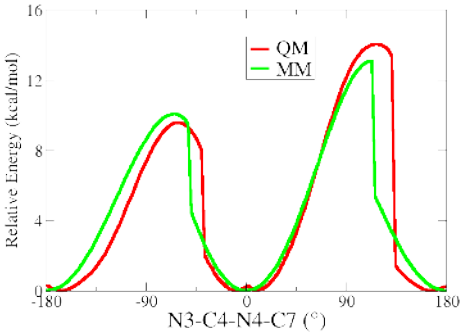

3 The Geometric Information of Base Scaffolds and Substitutent Side Chains

3.1 2-methylthio-adenine (2msa, for SMA, MIA, SIA, etc.)

Figure 110: The energy-minimized structure of TMC.

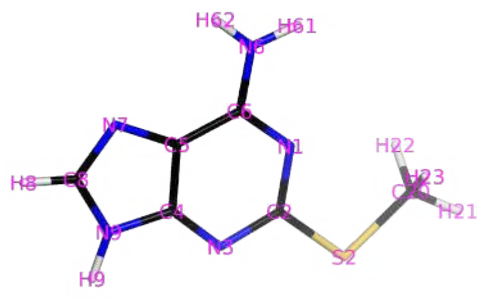

Table 229: The calculated geometric terms of TMC.

Terms	QM	MM	diff
N9-C8	1.374	1.372	-0.002
N9-C4	1.377	1.349	-0.028
N9-H9	1.013	1.000	-0.013
C8-H8	1.083	1.094	0.011
C8-N7	1.325	1.324	-0.001
N7-C5	1.381	1.399	0.018
C5-C6	1.407	1.408	0.002
C5-C4	1.399	1.396	-0.003
C6-N6	1.364	1.353	-0.010
C6-N1	1.341	1.367	0.025
N6-H61	1.012	0.996	-0.017
N6-H62	1.012	0.999	-0.014
N1-C2	1.352	1.367	0.016
C2-N3	1.341	1.358	0.016
C2-S2	1.768	1.751	-0.017
N3-C4	1.339	1.325	-0.014
S2-C20	1.807	1.830	0.023
C20-H21	1.093	1.111	0.019
C20-H22	1.089	1.112	0.023
C20-H23	1.090	1.112	0.022
C8-N9-C4	106.92	107.47	0.55
C8-N9-H9	127.48	130.47	3.00
C4-N9-H9	125.60	122.06	-3.55
N9-C8-H8	121.69	122.65	0.97
N9-C8-N7	113.54	112.89	-0.64
H8-C8-N7	124.78	124.45	-0.32
C8-N7-C5	103.22	103.61	0.39
N7-C5-C6	132.14	133.34	1.21
N7-C5-C4	112.07	110.24	-1.83
C6-C5-C4	115.77	116.42	0.65
C5-C6-N6	121.98	123.16	1.18
C5-C6-N1	119.04	118.03	-1.01
N6-C6-N1	118.90	118.81	-0.09

Terms	QM	MM	diff
C6-N6-H61	116.62	119.52	2.91
C6-N6-H62	115.90	120.16	4.26
H61-N6-H62	116.86	120.31	3.46
C6-N1-C2	118.49	119.65	1.16
N1-C2-N3	128.48	125.56	-2.91
N1-C2-S2	118.03	120.06	2.02
N3-C2-S2	113.49	114.38	0.89
C2-N3-C4	110.93	112.97	2.04
N9-C4-C5	104.25	105.79	1.54
N9-C4-N3	128.47	126.85	-1.62
C5-C4-N3	127.28	127.36	0.09
C2-S2-C20	100.83	99.06	-1.77
S2-C20-H21	105.98	110.36	4.37
S2-C20-H22	111.16	111.73	0.56
S2-C20-H23	111.07	111.73	0.65
H21-C20-H22	109.63	107.22	-2.41
H21-C20-H23	109.65	107.22	-2.44
H22-C20-H23	109.29	108.39	-0.90
C4-N9-C8-H8	179.86	-180.00	0.14
C4-N9-C8-N7	-0.12	0.00	0.12
H9-N9-C8-H8	-0.02	0.00	0.02
H9-N9-C8-N7	180.00	-180.00	-0.00
C8-N9-C4-C5	0.38	0.00	-0.38
C8-N9-C4-N3	-179.43	-180.00	-0.57
H9-N9-C4-C5	-179.74	180.00	0.26
H9-N9-C4-N3	0.45	0.00	-0.45
N9-C8-N7-C5	-0.20	0.00	0.20
H8-C8-N7-C5	179.82	180.00	0.18
C8-N7-C5-C6	178.47	180.00	1.53
C8-N7-C5-C4	0.46	0.00	-0.46
N7-C5-C6-N6	4.87	-0.00	-4.87
N7-C5-C6-N1	-178.38	-180.00	-1.62
C4-C5-C6-N6	-177.18	-180.00	-2.82
C4-C5-C6-N1	-0.43	0.00	0.43
N7-C5-C4-N9	-0.53	0.00	0.53
N7-C5-C4-N3	179.28	180.00	0.72
C6-C5-C4-N9	-178.90	180.00	1.10
C6-C5-C4-N3	0.92	0.00	-0.92
C5-C6-N6-H61	-20.50	-180.00	-159.50
C5-C6-N6-H62	-164.12	-0.00	164.12
N1-C6-N6-H61	162.74	0.00	-162.74
N1-C6-N6-H62	19.12	180.00	160.88
C5-C6-N1-C2	-0.12	0.00	0.12
N6-C6-N1-C2	176.74	-180.00	3.26
C6-N1-C2-N3	0.33	0.00	-0.33
C6-N1-C2-S2	-179.94	180.00	0.06
N1-C2-N3-C4	0.06	0.00	-0.06
S2-C2-N3-C4	-179.68	-180.00	-0.32
N1-C2-S2-C20	1.17	0.00	-1.17
N3-C2-S2-C20	-179.06	180.00	0.94
C2-N3-C4-N9	179.05	-180.00	0.95
C2-N3-C4-C5	-0.72	0.00	0.72
C2-S2-C20-H21	179.29	-180.00	0.71
C2-S2-C20-H22	-61.66	-60.81	0.85
C2-S2-C20-H23	60.26	60.81	0.55

Figure 111: The PES scan for flexible dihedral corresponding to TMC.

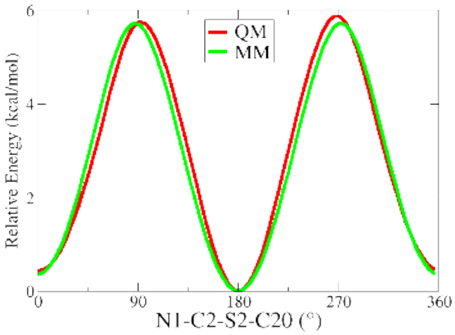

Table 230: The statistics of vibrational frequencies of TMC. Only the terms with the occupancies greater than 15% were shown. Refer to p.6 for the meanings of assig.

QM (MP2, scaled by 0.943)							MM						
Freq	Assig.	%	Assig.	%	Assig.	%	Freq	Assig.	%	Assig.	%	Assig.	%
53.4	tNCSC	97.					68.6	tNCSC	78.				
104.9	at6r	68.	wC2S2	22.			129.0	wC4N4	35.	wNH2	21.	btf	20.
158.8	pk6r	25.	tCSCH	23.	btf	18.	154.5	at6r	37.	wC4N4	32.		
165.5	rC2S2	59.	dCSC	27.			176.1	rC2S2	74.				
171.4	tCSCH	69.	btf	22.			218.4	wNH2	42.	at6r'	17.		
226.0	at6r'	64.	btf	16.			243.8	btf	23.	wNH2	21.	tCSCH	16.
262.5	rC4N4	46.					264.5	tCSCH	69.				
295.7	dCSC	48.					275.3	dCSC	39.	sN-C.ar	19.	sC2-S2	15.
319.2	t5r'	25.	at6r'	21.	btf	20.	314.6	rC4N4	44.	dCSC	29.		
396.3	sC2-S2	37.	ad6r	35.			363.0	wC2S2	56.	t5r'	21.	btf	18.
450.2	wNH2	89.					375.0	tC4N	90.				
467.8	tC4N	86.					398.8	sC2-S2	29.	ad6r	22.	dCSC	19.
500.5	wN-H	80.					432.4	wC2S2	34.	at6r'	19.	wC4N4	16.
522.8	ad6r'	69.					481.3	pk6r	30.	t5r'	26.	wN-H	21.
560.6	td6r	23.	ad6r	19.			495.7	pk6r	42.	wN-H	38.		
590.2	wC4N4	80.					522.7	sC6-N6	25.	ad6r'	23.		
603.8	t5r'	33.	t5r	32.			548.2	sN-C.ar	43.	ad6r	17.	ad6r'	17.
632.6	d5r'	30.	sC-C.ar	20.			626.8	sN-C.ar	24.	d5r'	17.	rC4N4	16.
643.6	t5r	57.	t5r'	36.			657.0	t5r	51.	t5r'	24.	btf	15.
671.0	pk6r	53.	wC2S2	39.			704.5	pk6r	29.	t5r	29.	t5r'	17.
741.0	sS2-C20	84.					704.8	sN-C.ar	39.	d5r	17.		
776.2	sN-C.ar	32.	d5r	19.			744.4	sS2-C20	83.				
787.1	wC-H	106.					853.4	d5r	39.	sN-C.ar	23.	sC-C.ar	17.
913.8	d5r	45.	d5r'	31.			906.3	wC-H	108.				
920.6	td6r	33.	sN-C.ar	30.			908.8	sN-C.ar	46.	td6r	18.		
1003.0	rCH3	81.					968.9	rCH3'	90.				
1004.5	rCH3'	94.					970.1	rCH3	84.				
1065.1	rNH2	38.	sN-C.ar	28.			995.6	rNH2	32.	sN-C.ar	29.		
1086.6	sN-C.ar	52.	rN-H	34.			1025.7	sN-C.ar	40.	rNH2	19.		
1134.9	sN-C.ar	43.					1048.2	sN-C.ar	43.	rNH2	17.		
1238.6	sN-C.ar	36.	rNH2	23.	rC-H	19.	1166.4	sN-C.ar	46.	rC-H	26.		
1258.7	sN-C.ar	53.	rC-H	22.			1184.9	sN-C.ar	49.				
1339.0	sN-C.ar	70.					1313.9	sN-C.ar	44.	rN-H	16.		
1364.1	sN-C.ar	63.	rN-H	19.			1337.2	sN-C.ar	40.	td6r	34.		
1382.5	sN-C.ar	72.					1416.6	sN-C.ar	24.	adCH3	21.		
1394.4	sdCH3	90.					1419.5	adCH3	73.				
1436.0	sN-C.ar	48.	sC-C.ar	25.			1432.5	adCH3'	91.				
1470.3	sN-C.ar	57.					1450.0	sN-C.ar	54.	rN-H	19.		
1473.1	adCH3'	95.					1483.3	sdCH3	93.				
1481.9	sN-C.ar	44.	rC-H	18.			1527.1	sN-C.ar	75.				
1493.4	adCH3	86.					1581.3	sN-C.ar	43.	rC-H	41.		
1592.9	cNH2	52.	sC-C.ar	28.			1627.7	cNH2	72.				
1630.0	sN-C.ar	56.	sC-C.ar	18.			1669.6	sN-C.ar	40.	sC-C.ar	22.	ad6r	15.
1656.9	sC-C.ar	32.	cNH2	32.	sC6-N6	20.	1709.8	sC-C.ar	36.	ad6r'	16.		
3032.3	ssCH3	98.					2851.2	ssCH3	100.				
3130.5	asCH3	98.					2908.0	asCH3	100.				
3146.2	asCH3'	100.					2909.8	asCH3'	100.				
3201.9	sC-H	99.					2953.6	sC-H	99.				
3490.4	ssNH2	100.					3446.7	ssNH2	100.				
3547.5	sN-H	100.					3502.5	sN-H	100.				
3617.4	asNH2	100.					3562.1	asNH2	100.				

3.2 7-deazapurin-7-yl-methyl ammonium (ampu, for DAG)

Figure 112: The energy-minimized structure of ampu.

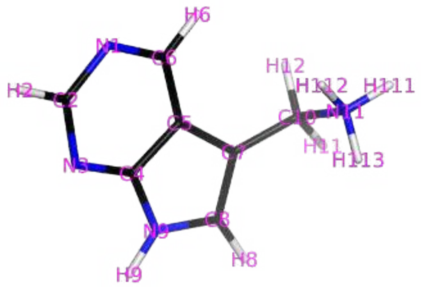

Table 231: The calculated geometric terms of ampu.

Terms	QM	MM	diff
N9-C8	1.363	1.386	0.023
N9-C4	1.376	1.353	-0.024
N9-H9	1.016	1.010	-0.006
C8-H8	1.084	1.084	-0.000
C8-C7	1.390	1.374	-0.016
C7-C5	1.433	1.438	0.005
C7-C10	1.475	1.487	0.013
C5-C6	1.405	1.395	-0.010
C5-C4	1.414	1.388	-0.027
C6-H6	1.092	1.084	-0.008
C6-N1	1.333	1.335	0.001
N1-C2	1.357	1.376	0.019
C2-H2	1.086	1.093	0.006
C2-N3	1.338	1.366	0.028
N3-C4	1.334	1.322	-0.012
C10-H11	1.092	1.102	0.010
C10-H12	1.093	1.101	0.008
C10-N11	1.539	1.493	-0.046
N11-H111	1.030	1.040	0.009
N11-H112	1.029	1.040	0.011
N11-H113	1.028	1.040	0.012
C8-N9-C4	109.44	111.42	1.97
C8-N9-H9	126.43	127.95	1.52
C4-N9-H9	124.09	120.58	-3.52
N9-C8-H8	120.92	122.53	1.61
N9-C8-C7	109.46	107.65	-1.82
H8-C8-C7	129.59	129.83	0.23
C8-C7-C5	106.57	105.98	-0.60
C8-C7-C10	126.29	127.34	1.05
C5-C7-C10	127.11	126.61	-0.50
C7-C5-C6	138.48	134.89	-3.59
C7-C5-C4	106.73	108.79	2.07
C6-C5-C4	114.78	116.31	1.53
C5-C6-H6	123.14	122.11	-1.03
C5-C6-N1	120.76	121.35	0.59
H6-C6-N1	116.09	116.54	0.45
C6-N1-C2	118.04	116.89	-1.15
N1-C2-H2	115.99	117.04	1.05
N1-C2-N3	127.41	126.06	-1.35
H2-C2-N3	116.60	116.90	0.30
C2-N3-C4	112.74	113.54	0.80
N9-C4-C5	107.80	106.16	-1.64
N9-C4-N3	125.94	128.02	2.08
C5-C4-N3	126.26	125.82	-0.44
C7-C10-H11	112.99	110.83	-2.16
C7-C10-H12	113.43	110.20	-3.22
C7-C10-N11	109.24	109.22	-0.02
H11-C10-H12	109.10	109.33	0.23
H11-C10-N11	106.13	108.38	2.25
H12-C10-N11	105.43	108.83	3.40
C10-N11-H111	113.12	110.49	-2.64
C10-N11-H112	109.95	109.31	-0.64
C10-N11-H113	109.95	109.06	-0.90
H111-N11-H112	108.22	109.75	1.52
H111-N11-H113	108.48	109.63	1.15
H112-N11-H113	106.90	108.58	1.68
C4-N9-C8-H8	-178.40	-179.01	-0.60
C4-N9-C8-C7	-0.17	0.67	0.84
H9-N9-C8-H8	-0.50	-1.70	-1.20
H9-N9-C8-C7	177.74	177.98	0.24
C8-N9-C4-C5	0.14	0.04	-0.10
C8-N9-C4-N3	179.88	-179.75	0.37
H9-N9-C4-C5	-177.83	-177.50	0.33
H9-N9-C4-N3	1.91	2.72	0.81
N9-C8-C7-C5	0.14	-1.06	-1.19
N9-C8-C7-C10	178.46	176.04	-2.42
H8-C8-C7-C5	178.17	178.59	0.42
H8-C8-C7-C10	-3.50	-4.31	-0.81
C8-C7-C5-C6	-178.54	-177.78	0.75
C8-C7-C5-C4	-0.05	1.11	1.16
C10-C7-C5-C6	3.15	5.09	1.94
C10-C7-C5-C4	-178.36	-176.02	2.34
C8-C7-C10-N11	-103.33	-99.28	4.06
C5-C7-C10-N11	74.66	77.25	2.59
C7-C5-C6-H6	0.34	0.18	-0.16
C7-C5-C6-N1	179.43	-179.00	1.57
C4-C5-C6-H6	-178.06	-178.66	-0.59
C4-C5-C6-N1	1.02	2.16	1.14
C7-C5-C4-N9	-0.05	-0.71	-0.66
C7-C5-C4-N3	-179.79	179.09	1.12
C6-C5-C4-N9	178.84	178.42	-0.43
C6-C5-C4-N3	-0.90	-1.79	-0.89
C5-C6-N1-C2	-0.48	-1.95	-1.47
H6-C6-N1-C2	178.67	178.82	0.16
C6-N1-C2-H2	179.94	-179.63	0.43
C6-N1-C2-N3	-0.35	1.33	1.69
N1-C2-N3-C4	0.50	-0.88	-1.38
H2-C2-N3-C4	-179.79	-179.92	-0.13
C2-N3-C4-N9	-179.52	-179.12	0.40
C2-N3-C4-C5	0.17	1.13	0.96

Figure 113: The PES scan for flexible dihedral corresponding to ampu.

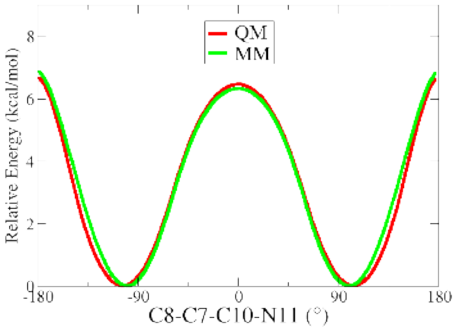

Table 232: The statistics of vibrational frequencies of ampu. Only the terms with the occupancies greater than 15% were shown. Refer to p.6 for the meanings of assig.

QM (MP2, scaled by 0.943)							MM						
Freq	Assig.	%	Assig.	%	Assig.	%	Freq	Assig.	%	Assig.	%	Assig.	%
67.6	tC7C10	81.					83.8	tC7C10	89.				
95.3	wC7C10	73.					99.4	wC7C10	57.	t5r	26.		
177.5	tC10N11	35.	rC7C10	33.			138.7	tC10N11	92.				
208.5	at6r	24.	btfl	22.	at6r'	17.	197.1	btfl	43.	at6r'	28.	t5r	22.
218.8	btfl	42.	tC10N11	17.			231.0	at6r	37.	t5r'	17.		
260.0	tC10N11	43.	rC7C10	20.			263.3	rC7C10	43.				
382.3	at6r	28.	at6r'	27.	cCCN	17.	340.1	t5r'	52.	cCCN	23.	t5r	15.
406.2	at6r'	40.	cCCN	18.			398.6	at6r'	34.	btfl	21.	at6r	20.
470.8	ad6r	22.	rC7C10	21.			445.5	at6r	31.	at6r'	31.	t5r'	19.
530.5	wNH	32.	t5r'	21.			489.7	sC-N.ar	35.	ad6r'	16.		
550.5	ad6r'	38.	sC-C.ar	16.			501.9	pk6r	23.	sC-N.ar	22.	ad6r'	18.
556.0	ad6r'	20.	t5r	17.	d5r'	17.	546.7	pk6r	20.	d5r'	19.		
587.7	t5r	29.	cCCN	17.			561.3	sC-C.ar	39.	rC7C10	15.		
612.9	wNH	55.	t5r'	36.			614.0	wNH	71.				
691.3	pk6r	92.					659.2	t5r	18.				
724.3	d5r	34.	ad6r	20.	sC7-C10	16.	687.2	sC-C.ar	16.	ad6r	15.		
763.0	sC-C.ar	34.	sC-N.ar	23.			707.7	ad6r'	30.	sC-N.ar	20.	sC-C.ar	20.
772.1	wC8-H	105.					774.1	wC8-H	99.				
833.8	sC10-N	80.					827.7	td6r	25.	ad6r	20.	d5r	19.
845.4	rCH2	44.	rCH3'	37.			885.3	rCH3'	54.	rCH2	34.		
855.7	wC-H	107.					937.3	wC-H	100.				
887.1	td6r	40.	sC-N.ar	19.			959.4	rCH3	43.	sC10-N	38.		
937.5	wC-H	107.					1004.6	wC-H	44.	sC10-N	32.	rCH3	20.
1016.4	rCH3	20.					1013.4	sC-N.ar	75.				
1067.1	rCH3	29.	rCH3'	17.	tCH2	15.	1017.6	wC-H	63.	sC10-N	19.		
1083.1	sC-N.ar	43.	rCH3	19.			1043.3	rCH2	24.	rCH3'	22.		
1117.7	rC-H	30.	rN-H	27.	sC-N.ar	25.	1073.1	sC7-C10	18.	sC-N.ar	15.		
1139.4	sC-N.ar	39.	td6r	29.			1081.5	rC-H	43.	sC-N.ar	31.		
1250.6	sC-N.ar	34.	rC-H	23.	sC-C.ar	20.	1112.9	sC-N.ar	39.	rC-H	26.	rN-H	17.
1260.3	sC-N.ar	21.	rN-H	19.	rC-H	18.	1166.5	sC-C.ar	34.	d5r'	16.		
1312.2	tCH2	37.	sC-N.ar	20.			1227.5	rC-H	28.	rN-H	26.	sC-N.ar	17.
1338.4	rC-H	35.	sC-N.ar	23.	tCH2	15.	1286.3	sC-N.ar	35.	rC-H	31.		
1351.5	sC-N.ar	79.					1316.0	sC-N.ar	43.	rC-H	37.		
1370.5	rC-H	37.	sC-C.ar	25.	wCH2	17.	1345.9	tCH2	79.				
1388.4	wCH2	53.	rC-H	17.			1429.7	sC-N.ar	37.	rC-H	27.		
1427.8	sC-N.ar	50.	rC-H	17.			1454.9	sdNH3	64.	cCH2	19.		
1440.8	sC-C.ar	26.	rC-H	26.	sC-N.ar	22.	1480.6	cCH2	45.	sdNH3	32.	wCH2	19.
1474.5	sC-N.ar	49.	rC-H	27.			1514.2	cCH2	21.	wCH2	21.	sC-C.ar	20.
1497.7	cCH2	98.					1523.8	sC-N.ar	32.				
1498.7	sdNH3	98.					1558.2	sC-N.ar	42.	sC-C.ar	33.		
1556.4	sC-C.ar	42.					1613.3	sC-N.ar	34.	rC-H	21.		
1576.4	sC-C.ar	59.	sC-N.ar	20.			1617.3	adNH3'	94.				
1621.1	sC-N.ar	47.	sC-C.ar	27.			1623.9	adNH3	90.				
1653.0	adNH3'	94.					1658.8	sC-C.ar	38.	sC-N.ar	22.		
1666.0	adNH3	94.					1701.6	sC-C.ar	47.				
3048.9	ssCH2	99.					2773.9	ssCH2	99.				
3084.5	sC-H	100.					2808.0	asCH2	100.				
3117.9	asCH2	99.					3033.6	sC-H	99.				
3166.5	sC-H	100.					3117.8	sC-H	99.				
3189.5	sC-H	99.					3153.5	ssNH3	100.				
3297.3	ssNH3	99.					3187.8	sC-H	99.				
3410.7	asNH3	98.					3256.4	asNH3'	99.				
3417.9	asNH3'	99.					3257.8	asNH3	99.				
3517.4	sN-H	100.					3505.2	sN-H	100.				

3.3 N-(6-purinyl)-N-methyl acetamide (m6pa, for 66A)

Figure 114: The energy-minimized structure of m6pa.

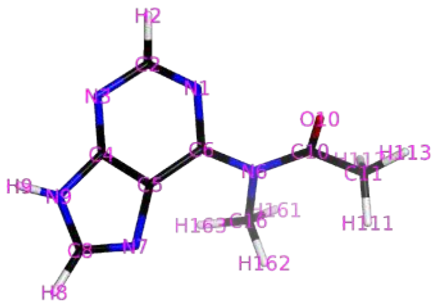

Table 233: The calculated geometric terms of m6pa.

Terms	QM	MM	diff
N9-C8	1.371	1.368	-0.003
N9-C4	1.377	1.350	-0.027
N9-H9	1.014	1.001	-0.012
C8-H8	1.083	1.094	0.010
C8-N7	1.325	1.323	-0.002
N7-C5	1.383	1.411	0.027
C5-C6	1.405	1.413	0.008
C5-C4	1.409	1.396	-0.013
C6-N1	1.337	1.387	0.051
C6-N6	1.405	1.419	0.014
N1-C2	1.351	1.356	0.006
C2-H2	1.087	1.095	0.008
C2-N3	1.343	1.354	0.011
N3-C4	1.336	1.324	-0.012
N6-C16	1.466	1.469	0.003
N6-C10	1.399	1.370	-0.029
C16-H161	1.091	1.113	0.022
C16-H162	1.093	1.113	0.020
C16-H163	1.090	1.113	0.023
C10-O10	1.226	1.226	-0.001
C10-C11	1.514	1.488	-0.027
C11-H111	1.094	1.109	0.016
C11-H112	1.094	1.109	0.016
C11-H113	1.090	1.112	0.022
C8-N9-C4	106.72	107.59	0.87
C8-N9-H9	127.59	130.51	2.92
C4-N9-H9	125.69	121.90	-3.79
N9-C8-H8	121.63	122.61	0.98
N9-C8-N7	113.89	112.86	-1.03
H8-C8-N7	124.49	124.54	0.05
C8-N7-C5	103.47	103.97	0.49
N7-C5-C6	133.13	133.90	0.77
N7-C5-C4	111.32	109.34	-1.98
C6-C5-C4	115.54	116.73	1.19
C5-C6-N1	119.33	115.81	-3.51
C5-C6-N6	122.09	125.43	3.34
N1-C6-N6	118.58	118.53	-0.05
C6-N1-C2	118.60	121.70	3.10
N1-C2-H2	115.57	117.44	1.87
N1-C2-N3	128.33	124.49	-3.84
H2-C2-N3	116.10	118.03	1.93
C2-N3-C4	111.19	113.24	2.04
N9-C4-C5	104.60	106.25	1.65
N9-C4-N3	128.37	125.86	-2.51
C5-C4-N3	126.99	127.89	0.90
C6-N6-C16	117.78	118.85	1.07
C6-N6-C10	118.28	120.24	1.96
C16-N6-C10	122.19	120.48	-1.71
N6-C16-H161	109.54	109.79	0.25
N6-C16-H162	112.45	108.26	-4.19
N6-C16-H163	108.23	110.22	1.99
H161-C16-H162	110.06	111.40	1.33
H161-C16-H163	108.17	107.46	-0.71
H162-C16-H163	108.27	109.71	1.44
N6-C10-O10	122.23	124.93	2.70
N6-C10-C11	115.59	117.66	2.07
O10-C10-C11	122.18	117.33	-4.85
C10-C11-H111	111.74	110.75	-0.99
C10-C11-H112	111.32	110.43	-0.89
C10-C11-H113	107.07	108.82	1.75
H111-C11-H112	108.11	110.22	2.11
H111-C11-H113	109.23	108.29	-0.94
H112-C11-H113	109.35	108.26	-1.09
C4-N9-C8-H8	-179.98	179.82	0.20
C4-N9-C8-N7	0.11	-0.24	-0.35
H9-N9-C8-H8	0.82	0.36	-0.46
H9-N9-C8-N7	-179.10	-179.70	-0.61
C8-N9-C4-C5	-0.19	0.29	0.48
C8-N9-C4-N3	-178.09	179.63	2.29
H9-N9-C4-C5	179.03	179.81	0.78
H9-N9-C4-N3	1.14	-0.86	-2.00
N9-C8-N7-C5	0.03	0.08	0.05
H8-C8-N7-C5	-179.89	-179.99	-0.10
C8-N7-C5-C6	179.00	178.28	-0.72
C8-N7-C5-C4	-0.16	0.11	0.26
N7-C5-C6-N1	-179.14	178.41	2.45
N7-C5-C6-N6	1.77	4.01	2.24
C4-C5-C6-N1	-0.01	-3.52	-3.51
C4-C5-C6-N6	-179.10	-177.92	1.18
N7-C5-C4-N9	0.22	-0.25	-0.47
N7-C5-C4-N3	178.15	-179.57	2.28
C6-C5-C4-N9	-179.10	-178.78	0.32
C6-C5-C4-N3	-1.17	1.91	3.07
C5-C6-N1-C2	1.11	4.34	3.23
N6-C6-N1-C2	-179.77	179.15	1.08
C5-C6-N6-C16	40.55	30.80	-9.75
C5-C6-N6-C10	-124.72	-141.71	-16.99
N1-C6-N6-C16	-138.55	-143.47	-4.92
N1-C6-N6-C10	56.19	44.02	-12.16
C6-N1-C2-H2	179.32	178.79	-0.53
C6-N1-C2-N3	-1.35	-3.38	-2.04
N1-C2-N3-C4	0.28	1.34	1.05
H2-C2-N3-C4	179.61	179.15	-0.46
C2-N3-C4-N9	178.47	-179.86	1.67
C2-N3-C4-C5	1.02	-0.67	-1.69

Terms	QM	MM	diff
C6-N6-C10-O10	1.03	6.57	5.55
C6-N6-C10-C11	-179.11	-176.85	2.25
C16-N6-C10-O10	-163.55	-165.81	-2.26
C16-N6-C10-C11	16.31	10.76	-5.55

Figure 115: The PES scan for flexible dihedral corresponding to m6pa.

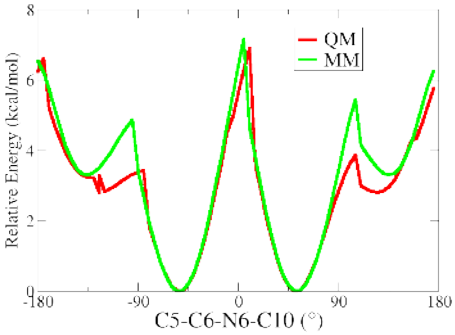

Table 234: The statistics of vibrational frequencies of m6pa. Only the terms with the occupancies greater than 15% were shown. Refer to p.6 for the meanings of assig.

QM (MP2, scaled by 0.943)							MM						
Freq	Assig.	%	Assig.	%	Assig.	%	Freq	Assig.	%	Assig.	%	Assig.	%
37.4	tC6N	83.					52.1	tC6N	85.				
76.5	tCNCO	69.					81.3	tCNCO	71.				
87.5	wC6N6	23.	at6r	19.	rCNC	16.	116.5	at6r	23.	at6r'	20.		
142.7	tCNCH	35.	wCNC	33.			158.1	tCNCH	45.	rC6N6	16.		
152.7	tCNCH	50.	wCNC	20.			201.0	tOCCH	41.				
196.4	at6r	37.	rCNC	22.			222.5	btf	39.	tOCCH	23.	at6r'	19.
213.0	tOCCH	87.					242.0	at6r	23.				
215.9	btf	73.	at6r'	16.			249.6	tOCCH	21.	at6r	19.		
265.3	cCNC	23.	rC6N6	17.			301.9	wCNC	19.	cCNC	15.		
306.2	at6r'	49.					337.7	at6r'	24.	rCNC	19.		
348.0	rC=O	23.					359.3						
366.1	rC=O	27.	cCNC	26.	rCNC	15.	433.6	pk6r	52.				
487.0	wN-H	53.					436.2	rC=O	21.				
497.0	ad6r'	20.	wN-H	18.			488.4	wN-H	61.	t5r'	30.		
529.2	wC6N6	16.					499.9	sN-C.ar	57.	ad6r'	17.		
543.0	rC=O	19.	ad6r	19.			525.2	dNCC	33.				
564.6	wC=O	30.					551.8	sN-C.ar	29.	ad6r	17.		
574.0	wC=O	21.	at6r	17.			576.1	rC=O	22.	cCNC	18.		
610.2							609.7	wC=O	19.	sN-C.ar	16.		
639.9	t5r'	51.					629.7	wC=O	48.	t5r	16.		
648.5	t5r	50.					653.6	sN-C.ar	24.	d5r'	16.		
673.8	sN-C.ar	33.					678.7	t5r'	33.	wN-H	28.	btf	17.
694.4	pk6r	60.	wC6N6	18.			777.7	t5r	19.	sC-C	16.		
807.1	sC-C	26.	sN-C.ar	17.	sN6-C10	15.	793.5	t5r	20.	wC6N6	19.		
811.2	wC-H	105.					857.8	d5r	44.	sN-C.ar	21.		
878.1	td6r	44.	sN-C.ar	15.			879.2	td6r	38.	ad6r	19.	sN-C.ar	19.
908.9	wC-H	56.	d5r	19.	d5r'	16.	906.3	wC-H	107.				
911.6	wC-H	52.	d5r	23.	d5r'	15.	978.5	rCH3'	41.				
1000.5	rCH3'	46.					994.1	wC-H	90.				
1045.5	rCH3	53.	rCH3'	19.	wC=O	18.	1017.9	rCH3'	21.	sN-C.ar	16.		
1061.7	rC16H3	21.	sN-C.ar	20.			1026.3	sN-C.ar	70.				
1090.5	sN-C.ar	49.	rN-H	41.			1045.1	rC16H3'	24.	rCH3	20.	rC16H3	16.
1133.5	rC16H3'	40.	sN-C.ar	29.			1057.0	rCH3	45.				
1137.9	rC16H3	33.	rC16H3'	25.	sN-C.ar	20.	1064.3	sN-C.ar	39.	rC16H3	16.		
1171.5	sN-C.ar	31.					1099.1	sN-C.ar	50.	rC-H	23.	rN-H	16.
1184.7	sN6-C16	25.	sN-C.ar	22.	td6r	16.	1163.7	rC16H3	36.	rC16H3'	20.		
1248.2	sN-C.ar	43.	rC-H	37.			1176.5	sN6-C16	26.	sN-C.ar	19.		
1318.8	sN6-C10	21.	sN-C.ar	20.			1197.0	rC-H	38.	sN-C.ar	24.		
1321.0	sN-C.ar	72.					1235.3	sN-C.ar	62.				
1349.3	sN-C.ar	28.					1304.4	sN-C.ar	27.	rN-H	21.	td6r	17.
1375.1	sN-C.ar	71.					1385.2	sdCH3	40.	sN-C.ar	21.		
1397.5	sN-C.ar	30.	rN-H	22.	sdCH3	17.	1399.7	sdCH3	57.				
1405.3	sdCH3	63.					1416.2	adC16H3	50.	adC16H3'	27.		
1427.3	sN-C.ar	42.	sC-C.ar	16.			1423.1	adCH3'	73.				
1457.2	sdC16H3	42.	sN-C.ar	22.	rC-H	22.	1437.7	adCH3	70.				
1473.0	sN-C.ar	34.	sdC16H3	29.	rC-H	20.	1451.8	sN-C.ar	48.	rN-H	17.		
1483.5	sN-C.ar	31.	adCH3'	20.	rC-H	19.	1475.3	adC16H3'	30.	adC16H3	21.	sdC16H3	17.
1488.8	adCH3'	44.	sdC16H3	18.			1517.5	sN6-C10	20.	sC-C	17.		
1497.1	adCH3	62.	adC16H3	16.			1558.5	sN-C.ar	36.	rC-H	28.		
1510.3	adC16H3'	38.	adC16H3	33.	adCH3	17.	1572.9	sdC16H3	39.				
1518.8	adC16H3	36.	adC16H3'	34.			1594.5	sN-C.ar	48.	rC-H	35.		
1594.4	sC-C.ar	57.	sN-C.ar	21.			1645.3	sN-C.ar	42.	sC-C.ar	17.		
1616.7	sN-C.ar	62.	sC-C.ar	17.			1693.5	sC-C.ar	38.	ad6r'	18.	sN-C.ar	16.
1739.2	sC=O	82.					1705.7	sC=O	47.	sN6-C10	23.		
3019.3	ssCH3	90.					2847.3	ssC16H3	100.				
3023.6	ssC16H3	89.					2914.7	asC16H3	51.	asC16H3'	33.	ssCH3	15.
3101.1	asCH3	76.	asCH3'	24.			2916.9	ssCH3	84.				
3111.7	asC16H3'	55.	asC16H3	41.			2920.3	asC16H3'	57.	asC16H3	42.		

QM (MP2, scaled by 0.943)					MM				
3134.8	asC16H3	56.	asC16H3'	40.	2953.8	sC-H	99.		
3141.9	asCH3'	71.	asCH3	23.	2973.2	asCH3'	72.	asCH3	28.
3146.4	sC-H	100.			2975.0	asCH3	72.	asCH3'	28.
3200.1	sC-H	99.			3120.8	sC-H	99.		
3545.9	sN-H	100.			3503.8	sN-H	100.		

3.4 7-deazapurin-7-yl-methyl amidinium (7mip, for RCG)

Figure 116: The energy-minimized structure of 7mip.

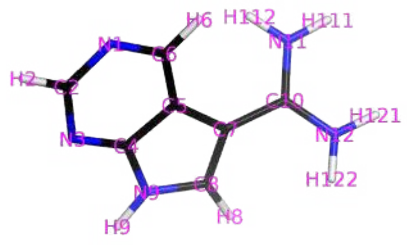

Table 235: The calculated geometric terms of 7mip.

Terms	QM	MM	diff
N9-C8	1.354	1.391	0.037
N9-C4	1.382	1.347	-0.035
N9-H9	1.018	1.011	-0.006
C8-H8	1.084	1.088	0.004
C8-C7	1.398	1.383	-0.015
C7-C5	1.436	1.446	0.010
C7-C10	1.437	1.454	0.017
C5-C6	1.406	1.394	-0.013
C5-C4	1.412	1.386	-0.025
C6-H6	1.090	1.085	-0.005
C6-N1	1.333	1.332	-0.001
N1-C2	1.357	1.374	0.017
C2-H2	1.086	1.093	0.007
C2-N3	1.339	1.364	0.026
N3-C4	1.333	1.318	-0.015
C10-N11	1.330	1.325	-0.006
C10-N12	1.332	1.326	-0.006
N11-H111	1.015	0.998	-0.017
N11-H112	1.014	1.000	-0.014
N12-H121	1.015	0.999	-0.016
N12-H122	1.013	0.999	-0.014
C8-N9-C4	109.81	111.83	2.02
C8-N9-H9	126.25	130.37	4.12
C4-N9-H9	123.90	117.80	-6.10
N9-C8-H8	121.13	122.25	1.12
N9-C8-C7	109.04	107.64	-1.39
H8-C8-C7	129.74	130.08	0.35
C8-C7-C5	106.94	105.13	-1.81
C8-C7-C10	125.23	127.17	1.94
C5-C7-C10	127.77	127.69	-0.08
C7-C5-C6	138.91	134.71	-4.20
C7-C5-C4	106.24	109.39	3.15
C6-C5-C4	114.74	115.88	1.14
C5-C6-H6	123.19	122.07	-1.12
C5-C6-N1	120.42	121.31	0.89
H6-C6-N1	116.38	116.62	0.24
C6-N1-C2	118.38	117.09	-1.29
N1-C2-H2	116.10	116.93	0.83
N1-C2-N3	127.23	126.08	-1.15
H2-C2-N3	116.67	116.99	0.33
C2-N3-C4	112.60	113.14	0.54
N9-C4-C5	107.98	106.01	-1.96
N9-C4-N3	125.44	127.51	2.07
C5-C4-N3	126.57	126.47	-0.10
C7-C10-N11	119.41	118.90	-0.51
C7-C10-N12	120.15	120.03	-0.12
N11-C10-N12	120.45	121.07	0.62
C10-N11-H111	122.72	120.75	-1.97
C10-N11-H112	119.68	119.41	-0.27
H111-N11-H112	117.31	119.81	2.49
C10-N12-H121	122.57	120.49	-2.08
C10-N12-H122	119.77	119.65	-0.12
H121-N12-H122	117.23	119.77	2.54
C4-N9-C8-H8	-176.40	-178.52	-2.12
C4-N9-C8-C7	0.24	-0.27	-0.52
H9-N9-C8-H8	1.25	1.14	-0.11
H9-N9-C8-C7	177.90	179.38	1.48
C8-N9-C4-C5	0.00	0.06	0.06
C8-N9-C4-N3	-179.08	-179.93	-0.85
H9-N9-C4-C5	-177.71	-179.64	-1.93
H9-N9-C4-N3	3.20	0.37	-2.83
N9-C8-C7-C5	-0.39	0.35	0.74
N9-C8-C7-C10	-177.63	-178.33	-0.70
H8-C8-C7-C5	175.88	178.41	2.54
H8-C8-C7-C10	-1.37	-0.27	1.10
C8-C7-C5-C6	176.07	177.54	1.47
C8-C7-C5-C4	0.38	-0.33	-0.71
C10-C7-C5-C6	-6.78	-3.79	2.99
C10-C7-C5-C4	177.53	178.34	0.81
C8-C7-C10-N11	146.36	141.65	-4.71
C8-C7-C10-N12	-33.47	-38.51	-5.04
C5-C7-C10-N11	-30.31	-36.75	-6.44
C5-C7-C10-N12	149.87	143.10	-6.77
C7-C5-C6-H6	1.49	0.83	-0.66
C7-C5-C6-N1	-177.76	-179.62	-1.86
C4-C5-C6-H6	176.94	178.60	1.66
C4-C5-C6-N1	-2.32	-1.86	0.46
C7-C5-C4-N9	-0.24	0.17	0.41
C7-C5-C4-N3	178.83	-179.85	1.32
C6-C5-C4-N9	-177.12	-178.15	-1.03
C6-C5-C4-N3	1.95	1.84	-0.11
C5-C6-N1-C2	1.05	1.07	0.02
H6-C6-N1-C2	-178.25	-179.36	-1.11
C6-N1-C2-H2	-179.66	179.91	0.43
C6-N1-C2-N3	0.99	-0.07	-1.06
N1-C2-N3-C4	-1.40	-0.04	1.36
H2-C2-N3-C4	179.25	179.97	0.72
C2-N3-C4-N9	178.73	179.08	0.35
C2-N3-C4-C5	-0.18	-0.90	-0.72

Figure 117: The PES scan for flexible dihedral corresponding to 7mip.

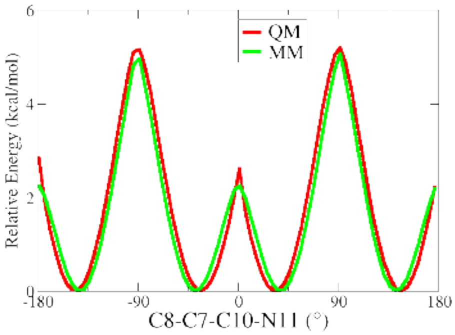

Table 236: The statistics of vibrational frequencies of 7mip. Only the terms with the occupancies greater than 15% were shown. Refer to p.6 for the meanings of assig.

QM (MP2, scaled by 0.943)							MM						
Freq	Assig.	%	Assig.	%	Assig.	%	Freq	Assig.	%	Assig.	%	Assig.	%
82.1	tC7C10	82.					83.6	tC7C10	87.				
102.1	wC7C10	65.					104.5	wC7C10	42.	t5r	36.		
137.2	rC7C10	65.					154.9	rC7C10	82.				
216.9	btfl	60.	at6r'	22.			198.8	btfl	52.	at6r'	26.		
237.3	at6r	48.	wC7C10	19.			252.7	at6r	41.	t5r'	37.		
341.1	cC(NH2)2	25.	sC-C.ar	16.	rC(NH2)2	16.	348.8	cC(NH2)2	29.	sC7-C10	26.		
383.6	cC(NH2)2	22.	d5r'	20.	rC(NH2)2	19.	356.1	t5r'	40.	rC(NH2)2	27.		
393.8	at6r'	63.	btfl	16.	at6r	15.	395.1	at6r'	34.	btfl	19.		
446.7	wNH2	97.					440.0	at6r'	26.	t5r'	24.	at6r	21.
480.9	stCCNH	69.	wNH2	21.			455.6	pk6r	24.	atCCNH	17.		
489.9	wNH2	19.	stCCNH	16.			493.1	sC-N.ar	53.	ad6r'	31.		
497.1	wNH2	64.					536.5	pk6r	47.				
539.1	ad6r'	33.	t5r	27.			557.1	stCCNH	78.	wNH2	20.		
559.5	t5r'	42.	at6r	19.			565.5	sC-C.ar	34.	d5r'	18.		
576.8	d5r'	24.	cC(NH2)2	18.			594.8	wNH2	38.	atCCNH	24.	wC(NH2)2	20.
596.9	ad6r	17.					611.6	cC(NH2)2	25.	atCCNH	24.	ad6r	20.
634.3	wNH	75.					618.6	wNH	38.				
642.2	atCCNH	86.					651.9	wNH	38.	t5r	20.		
674.8	wC(NH2)2	86.					657.8	wNH2	75.	stCCNH	19.		
694.0	pk6r	91.					692.9	sC-C.ar	18.	ad6r	18.	cC(NH2)2	17.
713.7	d5r	31.	ad6r	27.			707.1	ad6r'	30.	sC-N.ar	21.	sC-C.ar	18.
776.1	sC-C.ar	32.	sC-N.ar	24.			783.6	wC8-H	79.				
787.6	wC8-H	104.					802.2	wC(NH2)2	42.	wNH2	33.	wC8-H	21.
869.2	wC-H	108.					838.4	td6r	26.	d5r	24.	ad6r	17.
885.5	td6r	40.	sC-N.ar	20.			937.7	wC-H	100.				
944.8	wC-H	106.					975.3	ssC(NH2)	39.				
1030.0	sC-N.ar	19.					1009.0	wC-H	104.				
1047.3	rNH2	83.					1015.3	sC-N.ar	73.				
1089.4	rNH2	43.	ssC(NH2)	38.			1032.5	rNH2	90.				
1114.8	sC-N.ar	41.	rC-H	17.	rN-H	16.	1052.2	rNH2	51.	ssC(NH2)	15.		
1137.6	sC-N.ar	40.	rC-H	27.	td6r	18.	1091.6	sC-N.ar	50.	rC-H	45.		
1229.9	sC-N.ar	25.	rN-H	18.			1118.0	rC-H	36.	sC-N.ar	28.	rN-H	20.
1244.3	rC-H	30.	sC-N.ar	30.	sC-C.ar	28.	1164.1	sC-C.ar	37.				
1321.0	sC-N.ar	45.	rC-H	29.			1205.4	rC-H	27.	sC-N.ar	17.		
1343.7	sC-N.ar	53.	sC-C.ar	20.	rC-H	18.	1271.8	sC-N.ar	30.	rC-H	24.	rN-H	18.
1358.7	rC-H	34.	sC-N.ar	32.	sC-C.ar	24.	1315.1	rC-H	48.	sC-N.ar	36.		
1416.6	sC-N.ar	34.	rC-H	30.	sC-C.ar	24.	1382.9	sC-N.ar	38.	rC-H	19.		
1435.3	sC-N.ar	33.	rC-H	26.	rN-H	25.	1415.7	cNH2	51.	asC(NH2)	25.		
1465.3	sC-N.ar	41.	rC-H	30.			1458.1	sC-N.ar	27.	rC-H	17.		
1507.3	sC-N.ar	28.	sC-C.ar	23.	rC-H	18.	1505.6	sC-C.ar	29.	sC-N.ar	27.	rN-H	20.
1550.4	cNH2	48.	asC(NH2)	27.			1551.4	sC-N.ar	44.	sC-C.ar	33.		
1569.9	sC-C.ar	46.	cNH2	16.			1582.0	cNH2	53.	sC-N.ar	15.		
1589.2	sC-N.ar	25.	sC7-C10	21.	sC-C.ar	20.	1623.1	sC-N.ar	28.	rC-H	16.	sC-C.ar	16.
1623.1	sC-N.ar	38.	sC-C.ar	25.			1646.9	sC-N.ar	24.	sC-C.ar	23.		
1664.6	cNH2	76.					1677.1	asC(NH2)	46.	cNH2	28.		
1695.3	asC(NH2)	55.	cNH2	37.			1709.0	sC-C.ar	41.				
3113.8	sC-H	100.					3034.1	sC-H	99.				
3169.6	sC-H	100.					3117.9	sC-H	99.				
3192.8	sC-H	99.					3187.6	sC-H	99.				
3475.4	ssNH2	99.					3322.8	ssNH2	99.				
3486.4	ssNH2	97.					3332.0	ssNH2	99.				
3503.3	sN-H	98.					3435.8	asNH2	100.				
3598.9	asNH2	99.					3442.0	asNH2	99.				
3605.0	asNH2	98.					3509.0	sN-H	100.				

3.5 2-methylamino-4-imino pyrimidine, protonated (ncyp, for K2C & R2C)

Figure 118: The energy-minimized structure of ncyp.

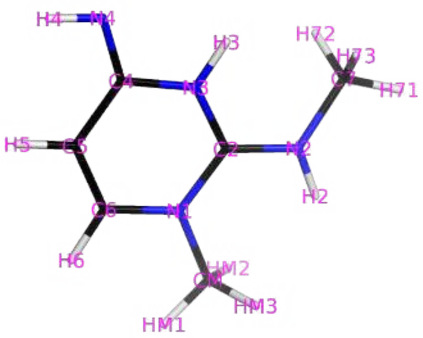

Table 237: The calculated geometric terms of ncyp.

Terms	QM	MM	diff
N1-C2	1.355	1.383	0.029
N1-C6	1.402	1.405	0.002
N1-CM	1.469	1.482	0.013
C2-N2	1.336	1.341	0.005
C2-N3	1.340	1.366	0.026
N2-H2	1.011	0.997	-0.015
N2-C7	1.465	1.474	0.009
N3-H3	1.019	0.988	-0.031
N3-C4	1.427	1.399	-0.027
C4-N4	1.278	1.293	0.015
C4-C5	1.452	1.430	-0.022
N4-H4	1.023	0.998	-0.025
C5-H5	1.084	1.077	-0.007
C5-C6	1.347	1.388	0.040
C6-H6	1.084	1.084	0.001
C7-H71	1.088	1.115	0.026
C7-H72	1.093	1.116	0.023
C7-H73	1.093	1.115	0.022
C2-N1-C6	119.20	120.66	1.46
C2-N1-CM	120.50	120.89	0.40
C6-N1-CM	120.31	118.45	-1.86
N1-C2-N2	121.12	122.43	1.31
N1-C2-N3	118.72	117.65	-1.07
N2-C2-N3	120.16	119.92	-0.24
C2-N2-H2	118.45	113.82	-4.63
C2-N2-C7	123.55	123.11	-0.44
H2-N2-C7	118.00	121.67	3.68
C2-N3-H3	120.26	121.73	1.47
C2-N3-C4	126.56	125.66	-0.89
H3-N3-C4	113.19	112.56	-0.62
N3-C4-N4	114.61	116.81	2.20
N3-C4-C5	112.43	115.10	2.67
N4-C4-C5	132.96	128.09	-4.86
C4-N4-H4	111.62	111.04	-0.58
C4-C5-H5	119.70	118.28	-1.43
C4-C5-C6	120.39	120.71	0.32
H5-C5-C6	119.91	121.02	1.11
N1-C6-C5	122.71	120.22	-2.48
N1-C6-H6	114.60	116.31	1.71
C5-C6-H6	122.69	123.47	0.78
N2-C7-H71	107.61	110.90	3.30
N2-C7-H72	110.80	112.12	1.32
N2-C7-H73	110.80	111.65	0.85
H71-C7-H72	108.51	106.97	-1.55
H71-C7-H73	108.51	106.85	-1.66
H72-C7-H73	110.50	108.08	-2.42
C6-N1-C2-N2	180.00	179.64	-0.36
C6-N1-C2-N3	-0.00	-0.02	-0.02
CM-N1-C2-N2	0.00	-0.24	-0.24
CM-N1-C2-N3	180.00	-179.91	0.09
C2-N1-C6-C5	0.00	0.01	0.01
C2-N1-C6-H6	180.00	-179.90	0.10
CM-N1-C6-C5	180.00	179.90	-0.10
CM-N1-C6-H6	-0.00	-0.01	-0.01
N1-C2-N2-H2	0.01	-0.47	-0.48
N1-C2-N2-C7	180.00	166.22	-13.78
N3-C2-N2-H2	180.01	179.19	-0.82
N3-C2-N2-C7	-0.00	-14.13	-14.12
N1-C2-N3-H3	-180.00	177.47	2.53
N1-C2-N3-C4	0.00	0.07	0.07
N2-C2-N3-H3	0.00	-2.20	-2.20
N2-C2-N3-C4	180.00	-179.60	0.39
C2-N3-C4-N4	-180.00	179.81	0.19
C2-N3-C4-C5	-0.00	-0.10	-0.09
H3-N3-C4-N4	-0.00	2.20	2.20
H3-N3-C4-C5	180.00	-177.71	2.30
N3-C4-N4-H4	180.00	-179.92	0.08
C5-C4-N4-H4	0.00	-0.03	-0.03
N3-C4-C5-H5	180.00	179.96	-0.04
N3-C4-C5-C6	0.00	0.08	0.08
N4-C4-C5-H5	0.00	0.07	0.07
N4-C4-C5-C6	180.00	-179.82	0.18
C4-C5-C6-N1	-0.00	-0.04	-0.04
C4-C5-C6-H6	180.00	179.87	-0.13
H5-C5-C6-N1	180.00	-179.92	0.08
H5-C5-C6-H6	0.00	-0.01	-0.02

Table 238: The statistics of vibrational frequencies of ncyp. Only the terms with the occupancies greater than 15% were shown. Refer to p.6 for the meanings of assig.

QM (MP2, scaled by 0.943)							MM						
Freq	Assig.	%	Assig.	%	Assig.	%	Freq	Assig.	%	Assig.	%	Assig.	%
67.1	at6r	77.	wNCM	20.			33.7	tC2N2	102.				
104.4	tC2N2	51.					80.6	at6r	57.	wNCM	22.		
138.0	adCH3'	33.	pk6r	30.	rCH3'	25.	119.9	tN1CH3	60.	wNCM	18.		
150.4	tN2CH3	40.	tC2N2	31.			132.3	tN2CH3	72.	tN1CH3	18.		
172.4	wNCM	67.	pk6r	17.			168.2	at6r'	23.	tN2CH3	21.	wC=N2	17.
202.9	rC=N2	45.	dN2H	38.			208.6	rC=N2	51.	dN2H	22.		
221.8	tN2CH3	47.	at6r'	20.			249.2	wNCM	34.	at6r	31.		
332.2	rNCM	46.	C-N	19.			329.4	rNCM	56.				
347.6	wN2H	68.					361.3	at6r'	63.	pk6r	26.		
358.9	at6r'	47.	pk6r	18.	wC-N4	16.	413.0	dN2H	29.	rC-N4	28.		
406.9	rC-N4	44.	dN2H	24.			501.4	ad6r	45.	C-N	19.		
458.3	ad6r	76.					525.4	rC-N4	22.	wN2H	17.	rNCM	17.
549.1	rC=N2	24.	rC-N4	22.	C-N	17.	546.3	wC=N2	17.				
572.2	ad6r'	50.	sN-C.r	22.			589.8	wN3H	48.	pk6r	36.		
591.1	wC-N4	50.	pk6r	50.			637.2	ad6r'	36.				
606.9	wC=N2	86.	wN3H	29.	pk6r	-19.	644.8	wC6H	44.	wC5H	41.		
642.9	wN3H	63.	wC-N4	20.			654.0	wN2H	55.	wC-N4	19.		
733.6	wC5H	62.	wC6H	20.			728.1	td6r	33.				
737.6	sC-C.r	25.	td6r	19.	sN-C.r	18.	767.8	wN3H	45.	wC-N4	23.	wC=N2	20.
765.4	C-N	23.	td6r	22.			804.7	sN-C.r	22.	ad6r'	20.		
848.2	tC4N4	95.					838.8	wC6H	54.	wC5H	53.		
873.2	wC6H	58.	wC5H	40.			914.2	C-N	31.	rCH3	26.		
955.3	sC-C.r	25.	sN-C.r	21.			951.0	sC-C.r	31.	td6r	20.	sN-C.r	17.
1024.0	sN1-C6	21.	td6r	18.			961.2	tC4N4	84.				
1067.5	C-N	32.	dN4H	27.			969.2	rCH3	49.	rCH3'	19.		
1101.5	dN4H	25.	sdCH3	19.	C-N	16.	974.0	rCH3'	84.				
1135.1	adCH3'	66.	rCH3'	37.			976.2	rCH3'	70.				
1143.9	rCH3'	94.					1014.6	rCH3	52.	C-N	19.		
1176.3	rCH3	38.					1118.5	sC-C.r	27.				
1182.4	rCH3	22.	rC5H	19.			1135.3	dN4H	18.				
1221.6	C-N	32.	sN-C.r	16.			1166.7	C-N	25.				
1313.0	rN2H	16.	rC6H	15.			1197.3	dN4H	27.	rC6H	18.	rC5H	16.
1323.4	rC5H	23.	dN4H	20.			1319.2	rN3H	52.				
1352.3	rN3H	38.					1392.9	adCH3	72.				
1406.4	rC6H	21.	adCH3	17.			1397.5	adCH3	90.				
1441.9	rN2H	18.	sN-C.r	15.			1407.8	adCH3'	93.				
1472.7	sdCH3	88.					1409.8	adCH3'	94.				
1477.8	adCH3	141.	rCH3	-45.	sdCH3	-31.	1419.6	rC5H	42.				
1499.4	rCH3	86.	tN1CH3	84.	adCH3'	-58.	1445.0	sdCH3	27.	rN2H	25.		
1501.4	adCH3'	96.					1458.5	rC6H	35.	sdCH3	30.	sC-C.r	16.
1521.1	adCH3	49.	rCH3	24.	sdCH3	20.	1485.1	sdCH3	38.	sN-C.r	19.		
1532.5	rCH3	39.	sdCH3	32.			1511.3	sdCH3	27.	sN-C.r	26.		
1584.1	sN-C.r	36.	sC-C.r	28.	rN2H	17.	1535.8	sdCH3	38.				
1645.9	sC4=N4	35.	sC-C.r	21.	sN-C.r	17.	1592.5	sN-C.r	35.	sC4=N4	23.		
1680.9	sC-N.r	42.	sN-C.r	21.			1684.5	sC-N.r	26.	sN-C.r	23.	rN2H	20.
1725.8	sC-C.r	32.	sC4=N4	30.			1748.1	sN-C.r	45.	rN3H	21.		
3020.6	ssCH3	44.	asCH3	35.	adCH3'	20.	2853.8	ssCH3	100.				
3022.9	ssCH3	95.					2854.1	ssCH3	100.				
3108.9	asCH3'	91.					2908.8	asCH3	99.				
3109.9	asCH3'	100.					2910.0	asCH3	97.				
3154.6	rCH3	48.	tN1CH3	48.	ssCH3	17.	2911.3	asCH3'	100.				
3154.9	asCH3	95.					2911.6	asCH3'	97.				
3176.5	sC-H	84.					3008.9	sC-H	99.				
3189.9	sC-H	73.	rC6H	19.			3054.2	sC-H	100.				
3426.3	sN-H	100.					3379.2	sN-H	100.				
3471.9	sN-H	100.					3386.5	sN-H	99.				
3560.7	sN-H	100.					3392.8	sN-H	99.				

3.6 3-methylbuten-1-yl methyl ammonium (nmba, for IAU, ISU & MIU)

Figure 119: The energy-minimized structure of nmba.

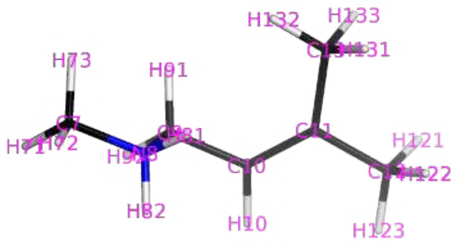

Table 239: The calculated geometric terms of nmba.

Terms	QM	MM	diff
C7-H71	1.090	1.111	0.021
C7-H72	1.090	1.111	0.021
C7-H73	1.089	1.111	0.021
C7-N8	1.500	1.504	0.004
N8-H81	1.029	1.012	-0.017
N8-H82	1.029	1.011	-0.018
N8-C9	1.527	1.512	-0.014
C9-H91	1.093	1.105	0.011
C9-H92	1.091	1.105	0.013
C9-C10	1.491	1.499	0.008
C10-H10	1.089	1.101	0.012
C10-C11	1.354	1.348	-0.006
C11-C12	1.502	1.504	0.002
C11-C13	1.504	1.507	0.002
C12-H121	1.096	1.112	0.017
C12-H122	1.093	1.112	0.019
C12-H123	1.096	1.113	0.017
C13-H131	1.096	1.112	0.017
C13-H132	1.093	1.109	0.016
C13-H133	1.096	1.113	0.017
H71-C7-H72	110.39	111.46	1.07
H71-C7-H73	110.38	111.56	1.18
H71-C7-N8	109.10	107.40	-1.70
H72-C7-H73	110.24	111.43	1.19
H72-C7-N8	108.34	107.34	-1.00
H73-C7-N8	108.34	107.38	-0.96
C7-N8-H81	110.02	110.51	0.49
C7-N8-H82	110.30	110.59	0.28
C7-N8-C9	115.03	115.75	0.72
H81-N8-H82	105.18	104.44	-0.74
H81-N8-C9	108.13	106.91	-1.22
H82-N8-C9	107.69	107.98	0.29
N8-C9-H91	105.74	105.86	0.12
N8-C9-H92	105.93	106.02	0.10
N8-C9-C10	109.84	109.94	0.10
H91-C9-H92	108.57	109.63	1.06
H91-C9-C10	112.24	113.58	1.34
H92-C9-C10	114.02	111.40	-2.62
C9-C10-H10	116.03	115.92	-0.11
C9-C10-C11	125.24	127.03	1.79
H10-C10-C11	118.71	116.92	-1.80
C10-C11-C12	120.16	120.73	0.57
C10-C11-C13	125.06	125.38	0.32
C12-C11-C13	114.77	113.89	-0.88
C11-C12-H121	110.53	110.59	0.07
C11-C12-H122	112.07	110.90	-1.17
C11-C12-H123	110.00	113.72	3.71
H121-C12-H122	108.72	107.01	-1.71
H121-C12-H123	106.87	107.05	0.18
H122-C12-H123	108.49	107.26	-1.23
C11-C13-H131	109.71	110.37	0.65
C11-C13-H132	114.46	115.35	0.89
C11-C13-H133	109.95	110.07	0.13
H131-C13-H132	107.72	107.14	-0.59
H131-C13-H133	106.49	106.53	0.04
H132-C13-H133	108.18	106.96	-1.22
C7-N8-C9-C10	-178.30	179.17	2.53
N8-C9-C10-C11	-94.92	-95.87	-0.95
C9-C10-C11-C12	-178.64	-178.94	-0.30
C9-C10-C11-C13	0.20	1.64	1.44

Figure 120: The PES scan for flexible dihedral corresponding to nmba.

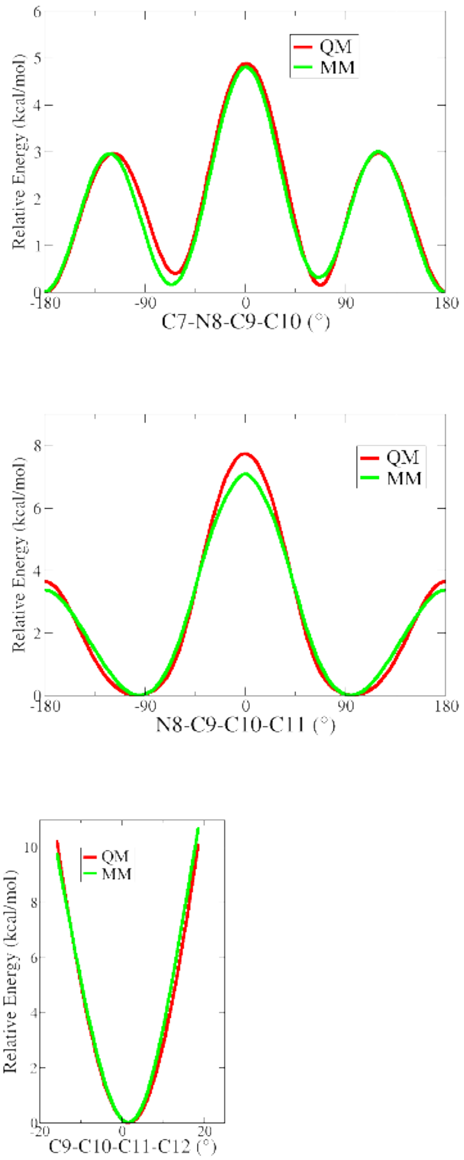

Table 240: The statistics of vibrational frequencies of nmba. Only the terms with the occupancies greater than 15% were shown. Refer to p.6 for the meanings of assig.

QM (MP2, scaled by 0.943)							MM						
Freq	Assig.	%	Assig.	%	Assig.	%	Freq	Assig.	%	Assig.	%	Assig.	%
37.4	tNCCC	88.					55.0	tNCCC	79.				
77.6	tCNCC	85.					90.3	tCNCC	82.				
104.4	stCCCH	31.	tCCCC	24.	cCNC	16.	127.7	tCCCC	38.	cCNC	24.		
107.5	stCCCH	63.	tCCCC	16.			182.2	tCCCH'	80.				
174.9	atCCCH	97.					199.9	tHCNC	94.				
222.9	tHCNC	93.					205.7	tCCCH	95.				
302.3	dCCC	31.	cNCC	29.	r=CCC	17.	310.5	cNCC	61.				
312.9	cNCC	36.	r=CCC	33.			326.5	r=CCC	28.	w=CCC	17.	cCNC	17.
356.3	sd=CCC	31.	cCNC	20.	w=CCC	16.	339.8	dCCC	36.	w=CCC	21.	r=CCC	20.
409.5	sd=CCC	44.	w=CCC	30.			376.4	tCCCC	42.	wCH	28.	w=CCC	19.
452.5	w=CCC	47.					416.7	sd=CCC	68.				
556.6	dCCC	24.	r=CCC	24.			534.4	dCCC	24.	r=CCC	23.		
759.5	ssC-C	36.	rCH2	19.	rNH2	19.	606.3	wCH	41.	cCNC	17.		
785.4	wCH	45.	sC-N	16.	tCCCC	15.	762.4	ssC-C	36.	sC-C	26.		
802.9	rNH2	42.	ssC-C	29.			821.2	rNH2	78.				
881.0	sC-N	52.	wCH	17.			911.8	rCH3'	51.	rCH3'	40.		
952.0	rCH2	36.	rCH3'	16.			957.1	sC-N	49.	rCH3'	17.		
958.6	rCH3'	55.	rCH3'	19.	asC-C	18.	964.5	rCH3'	41.	rCH2	19.		
983.0	rCH3'	59.	rCH3'	22.			980.5	rCH3'	30.	rCH2	20.	rCH3'	16.
996.6	sC-N	97.					995.6	rCH3'	50.	rCH3'	29.		
1026.8	rCH3'	27.	sC-C	26.			1006.5	rCH3'	45.	rCH3'	22.		
1084.4	rCH3'	59.	rCH3'	19.			1068.1	sC-N	89.				
1104.4	rCH3'	23.	tCH2	19.			1100.0	sC-C	47.				
1132.8	sC-C	27.	rCH3'	21.	rCH3'	19.	1163.5	rCH3'	47.	rCH3'	18.		
1155.8	rCH3'	37.	sC-C	15.			1183.6	rCH3'	46.	rCH3'	18.		
1242.8	asC-C	36.					1253.2	asC-C	28.	sdCH3	27.		
1293.4	tCH2	37.	rCH3'	19.			1290.4	tCH3	91.				
1339.6	wCH2	51.	wNH2	16.			1331.8	tCH2	73.				
1367.7	tCH3	55.	tCH2	23.			1372.4	sdCH3	29.	rCH	18.		
1377.3	rCH	43.					1403.7	adCH3'	48.	adCH3	44.		
1405.2	wNH2	60.	wCH2	30.			1411.2	adCH3'	78.				
1417.7	sdCH3	77.					1413.5	adCH3	65.	adCH3'	20.		
1426.6	sdCH3	88.					1416.1	sdCH3	53.	wNH2	34.		
1455.7	sdCH3	98.					1427.6	adCH3	36.	adCH3'	26.		
1461.0	adCH3	69.	adCH3'	24.			1438.8	wCH2	32.	sdCH3	28.		
1480.0	adCH3'	73.	cCH2	19.			1447.6	wNH2	28.	sdCH3	17.	cCH2	16.
1482.6	adCH3	91.					1466.9	adCH3'	53.				
1490.7	adCH3'	44.	adCH3	32.			1467.7	adCH3	64.	adCH3'	17.		
1494.1	adCH3'	74.					1504.2	cCH2	43.	sdCH3	22.		
1495.4	adCH3	61.	adCH3'	20.			1546.4	sdCH3	40.	wCH2	17.		
1510.8	cCH2	62.	adCH3'	27.			1608.4	sdCH3	50.	asC-C	33.		
1647.2	cNH2	100.					1726.9	sC-C	46.	rCH	25.		
1676.3	sC-C	71.					1739.3	cNH2	96.				
2998.2	ssCH3	101.					2769.5	ssCH2	99.				
3001.1	ssCH3	103.					2805.0	asCH2	99.				
3047.7	ssCH3	97.					2855.9	ssCH3	100.				
3050.3	ssCH2	93.					2857.1	ssCH3	100.				
3072.2	asCH3	86.	asCH3'	15.			2907.5	asCH3	95.				
3074.9	asCH3	84.	asCH3'	16.			2909.0	asCH3	84.	asCH3'	16.		
3093.5	asCH3'	95.					2910.5	asCH3'	85.				
3103.0	asCH3'	66.	asCH3	24.			2913.4	asCH3'	95.				
3108.2	sCH	71.	asCH2	17.			2919.7	ssCH3	100.				
3123.4	asCH2	75.	sCH	20.			2988.5	asCH3'	78.	asCH3	22.		
3158.9	asCH3	99.					2989.0	asCH3	78.	asCH3'	22.		
3162.5	asCH3'	99.					3034.0	sCH	99.				
3325.9	ssNH2	100.					3469.1	ssNH2	100.				
3392.3	asNH2	100.					3519.0	asNH2	100.				

3.7 1,5-hexadiene (15he, for GAU, GCU)

Figure 121: The energy-minimized structure of 15he.

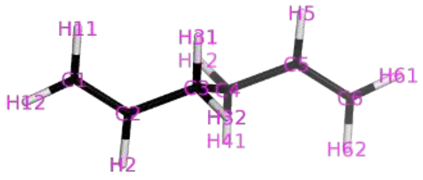

Table 241: The calculated geometric terms of 15he.

Terms	QM	MM	diff
C1-H11	1.087	1.101	0.014
C1-H12	1.085	1.101	0.016
C1-C2	1.339	1.344	0.005
C2-H2	1.091	1.103	0.012
C2-C3	1.499	1.507	0.007
C3-H31	1.097	1.114	0.017
C3-H32	1.098	1.113	0.016
C3-C4	1.538	1.547	0.008
C4-H41	1.097	1.114	0.017
C4-H42	1.098	1.113	0.016
C4-C5	1.499	1.507	0.007
C5-H5	1.091	1.103	0.012
C5-C6	1.339	1.344	0.005
C6-H61	1.085	1.101	0.016
C6-H62	1.087	1.101	0.014
H11-C1-H12	116.75	118.41	1.66
H11-C1-C2	121.44	120.43	-1.01
H12-C1-C2	121.81	121.16	-0.65
C1-C2-H2	119.13	118.57	-0.56
C1-C2-C3	124.56	126.02	1.46
H2-C2-C3	116.29	115.41	-0.88
C2-C3-H31	109.57	110.82	1.25
C2-C3-H32	110.15	109.33	-0.82
C2-C3-C4	112.01	111.97	-0.04
H31-C3-H32	106.90	106.79	-0.12
H31-C3-C4	109.62	109.00	-0.63
H32-C3-C4	108.46	108.79	0.33
C3-C4-H41	109.62	109.00	-0.63
C3-C4-H42	108.46	108.79	0.33
C3-C4-C5	112.01	111.97	-0.04
H41-C4-H42	106.90	106.79	-0.11
H41-C4-C5	109.56	110.82	1.25
H42-C4-C5	110.15	109.33	-0.82
C4-C5-H5	116.29	115.41	-0.88
C4-C5-C6	124.56	126.02	1.46
H5-C5-C6	119.13	118.57	-0.56
C5-C6-H61	121.81	121.16	-0.65
C5-C6-H62	121.44	120.43	-1.01
H61-C6-H62	116.75	118.41	1.66

Figure 122: The PES scan for flexible dihedral corresponding to 15he.

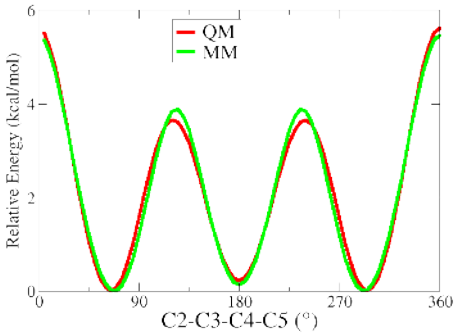

3.8 N3-propyluracil (3pru, for 3AU)

Figure 123: The energy-minimized structure of 3pru.

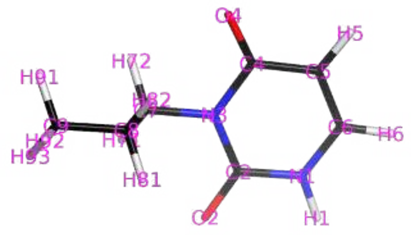

Table 242: The calculated geometric terms of 3pru.

Terms	QM	MM	diff
N1-C2	1.389	1.378	-0.012
N1-C6	1.373	1.357	-0.016
N1-H1	1.013	1.006	-0.007
C2-O2	1.228	1.227	-0.001
C2-N3	1.390	1.391	0.001
N3-C4	1.415	1.405	-0.010
N3-C7	1.469	1.474	0.006
C4-O4	1.231	1.231	0.000
C4-C5	1.455	1.432	-0.023
C5-H5	1.082	1.088	0.006
C5-C6	1.350	1.364	0.014
C6-H6	1.085	1.094	0.009
C7-H71	1.091	1.114	0.023
C7-H72	1.091	1.114	0.023
C7-C8	1.523	1.535	0.012
C8-H81	1.095	1.114	0.019
C8-H82	1.095	1.115	0.020
C8-C9	1.526	1.533	0.007
C9-H91	1.094	1.111	0.017
C9-H92	1.093	1.111	0.018
C9-H93	1.094	1.111	0.017
C2-N1-C6	124.16	121.96	-2.20
C2-N1-H1	114.34	113.45	-0.90
C6-N1-H1	121.44	124.59	3.15
N1-C2-O2	121.77	119.79	-1.98
N1-C2-N3	114.05	117.21	3.15
O2-C2-N3	124.18	123.01	-1.17
C2-N3-C4	125.76	122.83	-2.92
C2-N3-C7	116.64	118.31	1.67
C4-N3-C7	117.40	118.76	1.36
N3-C4-O4	120.59	120.08	-0.51
N3-C4-C5	114.82	116.77	1.95
O4-C4-C5	124.59	123.15	-1.44
C4-C5-H5	117.87	120.36	2.49
C4-C5-C6	120.18	119.31	-0.87
H5-C5-C6	121.94	120.32	-1.61
N1-C6-C5	120.86	121.88	1.02
N1-C6-H6	115.81	117.68	1.87
C5-C6-H6	123.33	120.43	-2.90
N3-C7-H71	107.08	108.85	1.78
N3-C7-H72	106.57	108.92	2.35
N3-C7-C8	111.74	112.27	0.53
H71-C7-H72	109.64	106.69	-2.95
H71-C7-C8	110.90	109.93	-0.96
H72-C7-C8	110.75	110.01	-0.74
C7-C8-H81	108.60	108.91	0.31
C7-C8-H82	108.49	108.81	0.33
C7-C8-C9	111.39	113.53	2.15
H81-C8-H82	107.88	107.59	-0.29
H81-C8-C9	110.18	108.92	-1.27
H82-C8-C9	110.21	108.90	-1.30
C8-C9-H91	110.91	110.43	-0.48
C8-C9-H92	111.04	110.56	-0.47
C8-C9-H93	110.95	110.47	-0.48
H91-C9-H92	107.87	108.41	0.54
H91-C9-H93	108.07	108.46	0.39
H92-C9-H93	107.87	108.43	0.57

Figure 124: The PES scan for flexible dihedral corresponding to 3pru.

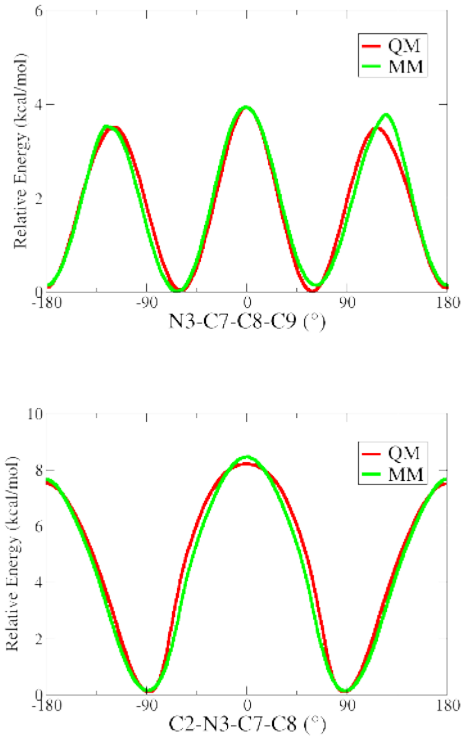

3.9 2-carboxamide-3-methoxy butyraldehyde (amba, for PBG)

Figure 125: The energy-minimized structure of amba.

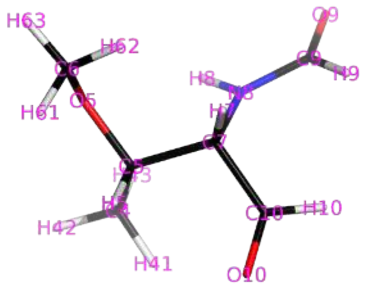

Table 243: The calculated geometric terms of amba.

Terms	QM	MM	diff
C4-H41	1.093	1.110	0.017
C4-H42	1.094	1.109	0.015
C4-H43	1.092	1.111	0.018
C4-C5	1.521	1.545	0.024
C5-H5	1.100	1.115	0.015
C5-O5	1.432	1.424	-0.007
C5-C7	1.528	1.521	-0.006
O5-C6	1.425	1.423	-0.002
C6-H61	1.095	1.111	0.016
C6-H62	1.098	1.112	0.014
C6-H63	1.091	1.112	0.021
C7-H7	1.101	1.114	0.014
C7-N8	1.452	1.444	-0.007
C7-C10	1.532	1.507	-0.025
N8-H8	1.015	0.996	-0.018
N8-C9	1.356	1.332	-0.023
C9-O9	1.233	1.224	-0.009
C9-H9	1.103	1.096	-0.007
C10-O10	1.223	1.216	-0.006
C10-H10	1.103	1.110	0.007
H41-C4-H42	109.19	107.96	-1.22
H41-C4-H43	108.48	108.44	-0.04
H41-C4-C5	109.77	110.57	0.80
H42-C4-H43	107.45	108.18	0.73
H42-C4-C5	110.71	110.98	0.27
H43-C4-C5	111.17	110.61	-0.56
C4-C5-H5	110.62	109.04	-1.57
C4-C5-O5	111.95	112.17	0.22
C4-C5-C7	111.82	109.77	-2.05
H5-C5-O5	109.54	109.18	-0.37
H5-C5-C7	107.20	107.68	0.48
O5-C5-C7	105.47	108.89	3.42
C5-O5-C6	113.27	112.90	-0.37
O5-C6-H61	112.04	111.08	-0.96
O5-C6-H62	111.01	110.84	-0.16
O5-C6-H63	106.25	108.94	2.69
H61-C6-H62	109.03	109.55	0.52
H61-C6-H63	108.93	107.97	-0.96
H62-C6-H63	109.52	108.38	-1.15
C5-C7-H7	109.81	109.37	-0.44
C5-C7-N8	108.38	112.62	4.24
C5-C7-C10	109.38	108.14	-1.24
H7-C7-N8	110.38	109.78	-0.60
H7-C7-C10	109.13	108.34	-0.79
N8-C7-C10	109.75	108.48	-1.26
C7-N8-H8	116.09	117.33	1.25
C7-N8-C9	119.09	124.15	5.06
H8-N8-C9	121.14	118.30	-2.84
N8-C9-O9	123.09	120.88	-2.21
N8-C9-H9	113.86	113.35	-0.51
O9-C9-H9	123.03	125.77	2.74
C7-C10-O10	121.26	125.57	4.32
C7-C10-H10	116.89	115.89	-1.01
O10-C10-H10	121.78	118.54	-3.24

Figure 126: The PES scan for flexible dihedral corresponding to amba.

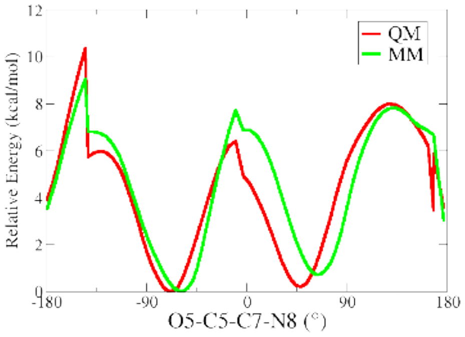

3.10 2-hydroxyl-3-amino butane (aboh, for BUG)

Figure 127: The energy-minimized structure of aboh.

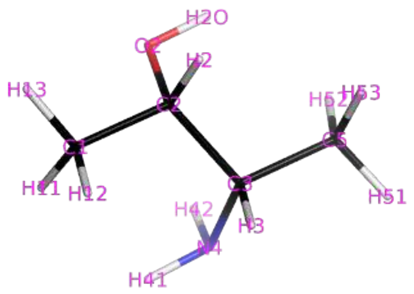

Table 244: The calculated geometric terms of aboh.

Terms	QM	MM	diff
C1-H11	1.093	1.110	0.017
C1-H12	1.094	1.109	0.015
C1-H13	1.092	1.111	0.019
C1-C2	1.516	1.538	0.022
C2-H2	1.102	1.115	0.012
C2-O2	1.437	1.426	-0.011
C2-C3	1.539	1.547	0.008
O2-H2O	0.972	0.960	-0.012
C3-H3	1.099	1.119	0.020
C3-N4	1.466	1.481	0.015
C3-C5	1.526	1.535	0.009
N4-H41	1.019	1.014	-0.006
N4-H42	1.022	1.016	-0.006
C5-H51	1.091	1.112	0.020
C5-H52	1.095	1.109	0.014
C5-H53	1.095	1.109	0.014
H11-C1-H12	109.01	108.93	-0.08
H11-C1-H13	107.98	107.98	0.00
H11-C1-C2	110.09	110.57	0.48
H12-C1-H13	108.89	108.68	-0.21
H12-C1-C2	110.78	110.65	-0.13
H13-C1-C2	110.03	109.97	-0.06
C1-C2-H2	108.92	108.54	-0.38
C1-C2-O2	105.79	107.50	1.71
C1-C2-C3	113.45	110.51	-2.94
H2-C2-O2	109.80	108.66	-1.14
H2-C2-C3	108.46	108.39	-0.07
O2-C2-C3	110.36	113.14	2.77
C2-O2-H2O	107.43	105.88	-1.55
C2-C3-H3	107.54	107.52	-0.02
C2-C3-N4	113.82	114.28	0.47
C2-C3-C5	111.24	109.59	-1.65
H3-C3-N4	107.56	108.85	1.29
H3-C3-C5	108.32	107.99	-0.33
N4-C3-C5	108.18	108.44	0.25
C3-N4-H41	109.90	111.53	1.62
C3-N4-H42	107.25	109.28	2.03
H41-N4-H42	105.92	103.36	-2.56
C3-C5-H51	109.82	109.64	-0.18
C3-C5-H52	110.46	111.02	0.56
C3-C5-H53	111.58	110.79	-0.79
H51-C5-H52	107.24	107.86	0.62
H51-C5-H53	108.97	108.33	-0.64
H52-C5-H53	108.65	109.11	0.46

Figure 128: The PES scan for flexible dihedral corresponding to aboh.

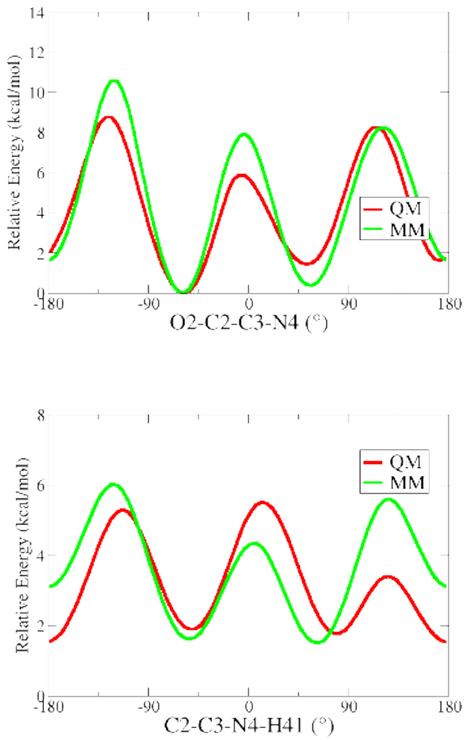

3.11 carbomoylbenzene (acbz, for BCU)

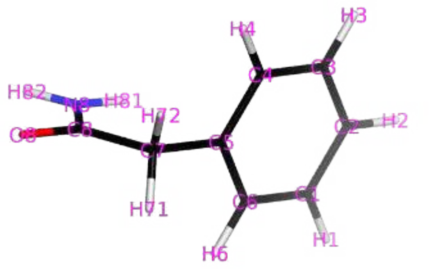

Figure 129: The energy-minimized structure of acbz.

Table 245: The calculated geometric terms of acbz.

Terms	QM	MM	diff
C1-H1	1.088	1.081	-0.007
C1-C2	1.400	1.401	0.001
C1-C6	1.398	1.401	0.003
C2-H2	1.088	1.081	-0.007
C2-C3	1.399	1.401	0.002
C3-H3	1.088	1.081	-0.008
C3-C4	1.399	1.401	0.002
C4-H4	1.090	1.080	-0.010
C4-C5	1.404	1.405	0.001
C5-C6	1.404	1.405	0.000
C5-C7	1.505	1.516	0.011
C6-H6	1.090	1.080	-0.010
C7-H71	1.096	1.112	0.017
C7-H72	1.097	1.112	0.015
C7-C8	1.529	1.503	-0.026
C8-N8	1.366	1.354	-0.012
C8-O8	1.235	1.228	-0.007
N8-H81	1.012	0.996	-0.016
N8-H82	1.014	0.995	-0.019
H1-C1-C2	120.08	120.01	-0.07
H1-C1-C6	119.83	120.04	0.21
C2-C1-C6	120.09	119.95	-0.14
C1-C2-H2	120.15	119.98	-0.18
C1-C2-C3	119.68	120.05	0.37
H2-C2-C3	120.17	119.98	-0.19
C2-C3-H3	120.10	120.01	-0.09
C2-C3-C4	120.07	119.95	-0.11
H3-C3-C4	119.83	120.04	0.21
C3-C4-H4	119.82	119.92	0.10
C3-C4-C5	120.74	120.31	-0.43
H4-C4-C5	119.44	119.77	0.33
C4-C5-C6	118.69	119.42	0.74
C4-C5-C7	120.88	120.28	-0.60
C6-C5-C7	120.40	120.28	-0.11
C1-C6-C5	120.73	120.31	-0.42
C1-C6-H6	119.83	119.92	0.09
C5-C6-H6	119.44	119.77	0.33
C5-C7-H71	111.09	108.38	-2.71
C5-C7-H72	110.61	108.38	-2.23
C5-C7-C8	115.87	116.69	0.82
H71-C7-H72	106.38	107.12	0.74
H71-C7-C8	106.05	107.95	1.90
H72-C7-C8	106.29	107.95	1.66
C7-C8-N8	116.48	120.54	4.06
C7-C8-O8	120.87	119.12	-1.75
N8-C8-O8	122.56	120.33	-2.23
C8-N8-H81	121.17	119.23	-1.93
C8-N8-H82	117.04	116.69	-0.35
H81-N8-H82	118.31	124.08	5.77

Figure 130: The PES scan for flexible dihedral corresponding to acbz.

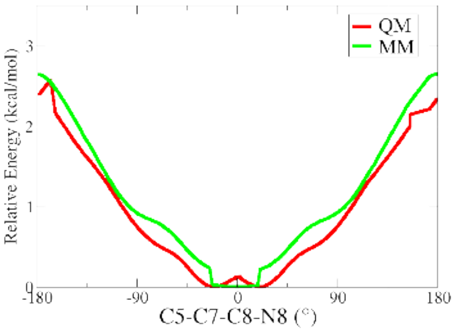

3.12 phenylacetate (bzac, for 5CU)

Figure 131: The energy-minimized structure of bzac.

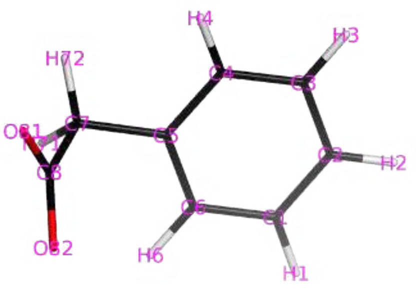

Table 246: The calculated geometric terms of bzac.

Terms	QM	MM	diff
C1-H1	1.090	1.079	-0.011
C1-C2	1.399	1.402	0.003
C1-C6	1.400	1.401	0.001
C2-H2	1.090	1.079	-0.010
C2-C3	1.401	1.402	0.000
C3-H3	1.090	1.079	-0.011
C3-C4	1.398	1.402	0.004
C4-H4	1.088	1.079	-0.008
C4-C5	1.407	1.404	-0.003
C5-C6	1.405	1.404	-0.001
C5-C7	1.502	1.509	0.007
C6-H6	1.090	1.081	-0.009
C7-H71	1.095	1.109	0.013
C7-H72	1.099	1.109	0.011
C7-C8	1.569	1.521	-0.048
C8-O81	1.269	1.258	-0.011
C8-O82	1.266	1.258	-0.008
H1-C1-C2	120.09	120.42	0.33
H1-C1-C6	119.83	119.71	-0.12
C2-C1-C6	120.08	119.87	-0.22
C1-C2-H2	120.39	119.95	-0.44
C1-C2-C3	119.16	120.00	0.84
H2-C2-C3	120.44	120.04	-0.40
C2-C3-H3	119.83	120.25	0.42
C2-C3-C4	120.59	119.89	-0.69
H3-C3-C4	119.58	119.85	0.27
C3-C4-H4	121.04	120.53	-0.52
C3-C4-C5	120.83	120.49	-0.33
H4-C4-C5	118.04	118.97	0.93
C4-C5-C6	118.00	119.19	1.18
C4-C5-C7	120.83	120.26	-0.57
C6-C5-C7	121.17	120.53	-0.64
C1-C6-C5	121.33	120.56	-0.77
C1-C6-H6	119.74	120.85	1.11
C5-C6-H6	118.91	118.56	-0.35
C5-C7-H71	109.98	108.61	-1.37
C5-C7-H72	109.55	109.71	0.16
C5-C7-C8	111.93	112.63	0.70
H71-C7-H72	108.77	108.88	0.11
H71-C7-C8	109.29	107.93	-1.36
H72-C7-C8	107.23	109.01	1.78
C7-C8-O81	114.48	115.26	0.79
C7-C8-O82	115.79	116.60	0.82
O81-C8-O82	129.74	128.12	-1.62

Figure 132: The PES scan for flexible dihedral corresponding to bzac.

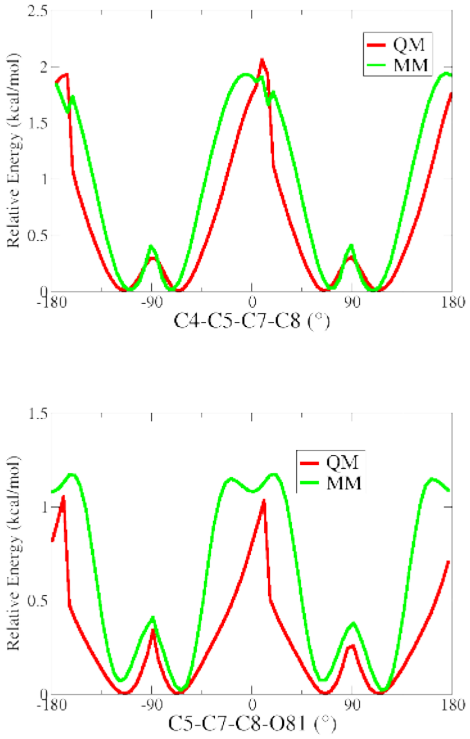

3.13 phenylammonium (bzam, for SAU)

Figure 133: The energy-minimized structure of bzam.

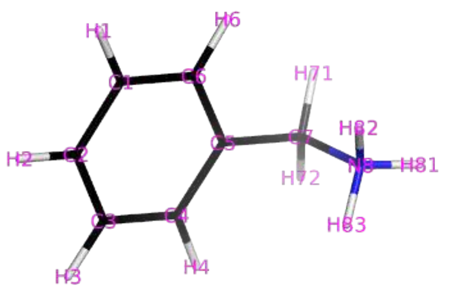

Table 247: The calculated geometric terms of bzam.

Terms	QM	MM	diff
C1-H1	1.086	1.082	-0.004
C1-C2	1.398	1.400	0.002
C1-C6	1.395	1.400	0.005
C2-H2	1.087	1.082	-0.004
C2-C3	1.398	1.400	0.002
C3-H3	1.086	1.082	-0.004
C3-C4	1.395	1.400	0.005
C4-H4	1.090	1.081	-0.009
C4-C5	1.402	1.410	0.007
C5-C6	1.402	1.410	0.007
C5-C7	1.493	1.492	-0.001
C6-H6	1.090	1.081	-0.009
C7-H71	1.092	1.102	0.009
C7-H72	1.092	1.102	0.009
C7-N8	1.531	1.483	-0.048
N8-H81	1.030	1.042	0.012
N8-H82	1.029	1.042	0.013
N8-H83	1.029	1.042	0.013
H1-C1-C2	120.13	119.67	-0.46
H1-C1-C6	119.88	120.31	0.43
C2-C1-C6	119.99	120.01	0.02
C1-C2-H2	119.85	119.93	0.08
C1-C2-C3	120.30	120.14	-0.16
H2-C2-C3	119.85	119.93	0.08
C2-C3-H3	120.13	119.67	-0.46
C2-C3-C4	119.99	120.01	0.02
H3-C3-C4	119.88	120.31	0.43
C3-C4-H4	119.79	118.98	-0.81
C3-C4-C5	119.77	120.38	0.61
H4-C4-C5	120.39	120.64	0.25
C4-C5-C6	120.18	119.07	-1.11
C4-C5-C7	119.86	120.46	0.60
C6-C5-C7	119.86	120.46	0.60
C1-C6-C5	119.77	120.38	0.61
C1-C6-H6	119.79	118.98	-0.81
C5-C6-H6	120.39	120.64	0.25
C5-C7-H71	113.21	110.92	-2.29
C5-C7-H72	113.21	110.92	-2.29
C5-C7-N8	108.39	101.29	-7.10
H71-C7-H72	109.40	111.57	2.17
H71-C7-N8	106.06	110.85	4.79
H72-C7-N8	106.06	110.85	4.79
C7-N8-H81	113.20	110.80	-2.40
C7-N8-H82	109.89	110.08	0.18
C7-N8-H83	109.89	110.08	0.18
H81-N8-H82	108.38	108.86	0.49
H81-N8-H83	108.38	108.86	0.49
H82-N8-H83	106.89	108.11	1.21

Figure 134: The PES scan for flexible dihedral corresponding to bzam.

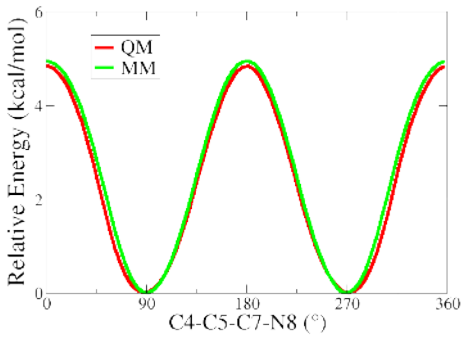

3.14 phenylacetic acid (bzaa, for 5CU)

Figure 135: The energy-minimized structure of bzaa.

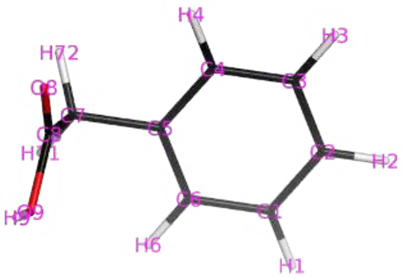

Table 248: The calculated geometric terms of bzaa.

Terms	QM	MM	diff
C1-H1	1.087	1.081	-0.007
C1-C2	1.396	1.401	0.005
C1-C6	1.397	1.401	0.005
C2-H2	1.087	1.081	-0.007
C2-C3	1.397	1.401	0.004
C3-H3	1.087	1.081	-0.007
C3-C4	1.395	1.401	0.007
C4-H4	1.088	1.080	-0.008
C4-C5	1.401	1.404	0.002
C5-C6	1.399	1.404	0.005
C5-C7	1.515	1.503	-0.012
C6-H6	1.088	1.080	-0.008
C7-H71	1.093	1.110	0.017
C7-H72	1.094	1.110	0.016
C7-C8	1.507	1.514	0.007
C8-O8	1.219	1.217	-0.003
C8-O9	1.359	1.378	0.019
O9-H9	0.980	0.956	-0.024
H1-C1-C2	120.10	120.03	-0.08
H1-C1-C6	119.76	120.02	0.26
C2-C1-C6	120.13	119.95	-0.18
C1-C2-H2	120.14	120.00	-0.14
C1-C2-C3	119.71	120.03	0.31
H2-C2-C3	120.15	119.98	-0.17
C2-C3-H3	120.07	120.05	-0.02
C2-C3-C4	120.20	119.95	-0.25
H3-C3-C4	119.73	120.00	0.27
C3-C4-H4	120.33	119.96	-0.37
C3-C4-C5	120.26	120.27	0.02
H4-C4-C5	119.41	119.77	0.36
C4-C5-C6	119.35	119.53	0.18
C4-C5-C7	119.70	120.03	0.32
C6-C5-C7	120.94	120.43	-0.51

Terms	QM	MM	diff
C1-C6-C5	120.34	120.26	-0.08
C1-C6-H6	119.98	119.94	-0.04
C5-C6-H6	119.68	119.80	0.12
C5-C7-H71	110.54	109.91	-0.63
C5-C7-H72	110.79	109.08	-1.71
C5-C7-C8	108.95	109.41	0.46
H71-C7-H72	109.09	109.06	-0.03
H71-C7-C8	109.80	110.57	0.76
H72-C7-C8	107.62	108.78	1.17
C7-C8-O8	125.39	126.22	0.83
C7-C8-O9	111.57	113.90	2.33
O8-C8-O9	122.98	119.86	-3.13
C8-O9-H9	105.53	107.23	1.70

Figure 136: The PES scan for flexible dihedral corresponding to bzaa.

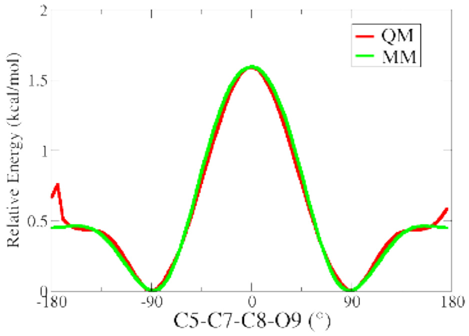

3.15 2-phenyl-2-hydroxylacetate (bzha, for HCU)

Figure 137: The energy-minimized structure of bzha.

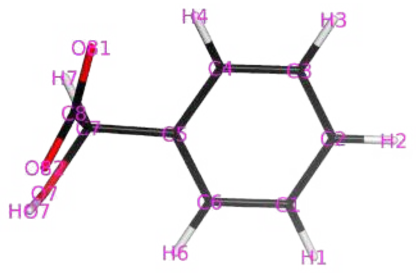

Table 249: The calculated geometric terms of bzha.

Terms	QM	MM	diff
C1-H1	1.090	1.079	-0.011
C1-C2	1.399	1.400	0.001
C1-C6	1.400	1.401	0.000
C2-H2	1.090	1.080	-0.010
C2-C3	1.401	1.400	-0.001
C3-H3	1.090	1.080	-0.010
C3-C4	1.398	1.400	0.002
C4-H4	1.087	1.081	-0.006
C4-C5	1.406	1.409	0.003
C5-C6	1.402	1.412	0.010
C5-C7	1.512	1.535	0.022
C6-H6	1.087	1.080	-0.007
C7-H7	1.100	1.110	0.010
C7-O7	1.426	1.443	0.017
C7-C8	1.572	1.566	-0.006
O7-HO7	0.997	0.978	-0.019
C8-O81	1.258	1.260	0.002
C8-O82	1.274	1.265	-0.009
H1-C1-C2	119.92	120.25	0.32
H1-C1-C6	119.68	119.75	0.08
C2-C1-C6	120.40	120.00	-0.40
C1-C2-H2	120.42	120.08	-0.34
C1-C2-C3	119.19	119.90	0.72
H2-C2-C3	120.38	120.01	-0.37
C2-C3-H3	119.92	120.37	0.45
C2-C3-C4	120.48	119.97	-0.51
H3-C3-C4	119.60	119.66	0.06
C3-C4-H4	120.74	120.00	-0.75
C3-C4-C5	120.52	121.05	0.53
H4-C4-C5	118.66	118.94	0.27
C4-C5-C6	118.77	118.17	-0.60
C4-C5-C7	120.39	119.00	-1.39
C6-C5-C7	120.84	122.82	1.98
C1-C6-C5	120.64	120.92	0.28
C1-C6-H6	121.10	119.74	-1.36
C5-C6-H6	118.26	119.34	1.08
C5-C7-H7	108.79	109.28	0.49
C5-C7-O7	111.46	114.38	2.92
C5-C7-C8	111.22	110.78	-0.44
H7-C7-O7	108.96	106.33	-2.63
H7-C7-C8	107.08	106.36	-0.73
O7-C7-C8	109.20	109.32	0.12
C7-O7-HO7	99.91	97.26	-2.65
C7-C8-O81	116.26	116.58	0.33
C7-C8-O82	113.44	116.22	2.78
O81-C8-O82	130.30	127.18	-3.11

Figure 138: The PES scan for flexible dihedral corresponding to bzha.

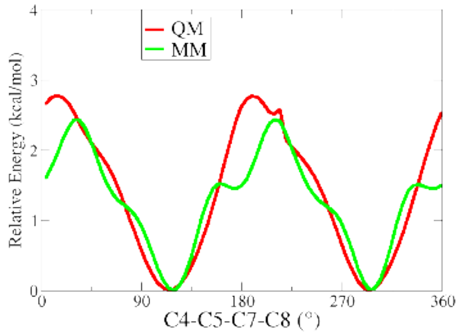

3.16 1-hydroxylethyl benzene (bzhe, for CMU)

Figure 139: The energy-minimized structure of bzhe.

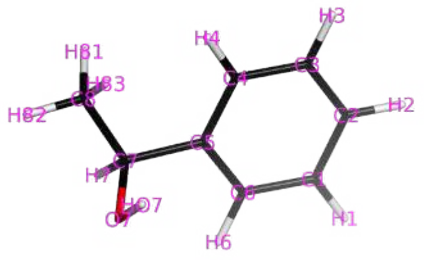

Table 250: The calculated geometric terms of bzhe.

Terms	QM	MM	diff
C1-H1	1.088	1.081	-0.007
C1-C2	1.396	1.399	0.003
C1-C6	1.395	1.399	0.004
C2-H2	1.087	1.081	-0.007
C2-C3	1.396	1.399	0.004
C3-H3	1.088	1.081	-0.007
C3-C4	1.397	1.402	0.005
C4-H4	1.087	1.078	-0.008
C4-C5	1.402	1.413	0.011
C5-C6	1.403	1.409	0.006
C5-C7	1.521	1.523	0.002
C6-H6	1.086	1.081	-0.005
C7-H7	1.097	1.117	0.020
C7-O7	1.435	1.418	-0.017
C7-C8	1.524	1.539	0.015
O7-HO7	0.975	0.960	-0.015
C8-H81	1.093	1.110	0.017
C8-H82	1.093	1.112	0.019
C8-H83	1.095	1.110	0.015
H1-C1-C2	120.06	120.11	0.04
H1-C1-C6	119.70	119.98	0.29
C2-C1-C6	120.24	119.91	-0.33
C1-C2-H2	120.32	120.00	-0.32
C1-C2-C3	119.45	119.95	0.50
H2-C2-C3	120.24	120.05	-0.18
C2-C3-H3	120.05	119.88	-0.17
C2-C3-C4	120.31	120.19	-0.12
H3-C3-C4	119.64	119.93	0.29
C3-C4-H4	118.87	117.93	-0.94
C3-C4-C5	120.66	120.52	-0.14
H4-C4-C5	120.46	121.55	1.08
C4-C5-C6	118.58	118.43	-0.15
C4-C5-C7	122.33	123.89	1.56
C6-C5-C7	119.06	117.68	-1.38
C1-C6-C5	120.76	121.01	0.25
C1-C6-H6	120.34	119.83	-0.51
C5-C6-H6	118.90	119.16	0.26
C5-C7-H7	108.81	108.74	-0.07
C5-C7-O7	111.04	112.68	1.64
C5-C7-C8	114.21	113.53	-0.68
H7-C7-O7	103.44	107.47	4.03
H7-C7-C8	108.86	107.33	-1.53
O7-C7-C8	109.89	106.79	-3.10
C7-O7-HO7	106.24	105.19	-1.05
C7-C8-H81	112.24	110.61	-1.63
C7-C8-H82	108.74	109.75	1.01
C7-C8-H83	110.40	111.26	0.86
H81-C8-H82	108.33	107.96	-0.37
H81-C8-H83	108.29	109.09	0.80
H82-C8-H83	108.77	108.08	-0.69

Figure 140: The PES scan for flexible dihedral corresponding to bzhe.

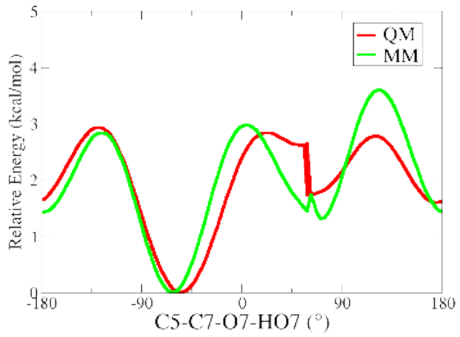

3.17 methoxybenzene (bzmo, for MOU)

Figure 141: The energy-minimized structure of bzmo.

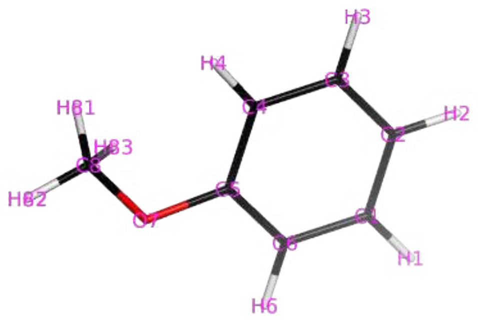

Table 251: The calculated geometric terms of bzmo.

Terms	QM	MM	diff
C1-H1	1.088	1.081	-0.007
C1-C2	1.402	1.400	-0.002
C1-C6	1.393	1.401	0.008
C2-H2	1.087	1.081	-0.007
C2-C3	1.395	1.400	0.005
C3-H3	1.088	1.081	-0.007
C3-C4	1.402	1.404	0.001
C4-H4	1.085	1.077	-0.009
C4-C5	1.401	1.413	0.012
C5-C6	1.403	1.401	-0.002
C5-O7	1.375	1.413	0.038
C6-H6	1.087	1.080	-0.008
O7-C8	1.428	1.426	-0.002
C8-H81	1.096	1.112	0.017
C8-H82	1.090	1.112	0.022
C8-H83	1.096	1.112	0.017
H1-C1-C2	120.14	120.16	0.02
H1-C1-C6	119.39	120.08	0.69
C2-C1-C6	120.47	119.77	-0.71
C1-C2-H2	120.40	119.96	-0.44
C1-C2-C3	119.26	120.04	0.78
H2-C2-C3	120.35	120.00	-0.34
C2-C3-H3	120.07	119.91	-0.16
C2-C3-C4	120.97	120.11	-0.86
H3-C3-C4	118.96	119.98	1.02
C3-C4-H4	119.25	117.91	-1.34
C3-C4-C5	119.20	120.22	1.02
H4-C4-C5	121.55	121.87	0.32
C4-C5-C6	120.18	118.89	-1.29
C4-C5-O7	124.62	123.19	-1.43
C6-C5-O7	115.20	117.93	2.73
C1-C6-C5	119.91	120.98	1.06
C1-C6-H6	121.52	120.25	-1.27
C5-C6-H6	118.56	118.77	0.20
C5-O7-C8	117.12	117.59	0.48
O7-C8-H81	111.07	110.92	-0.16
O7-C8-H82	105.27	108.74	3.47
O7-C8-H83	111.07	110.92	-0.16
H81-C8-H82	109.73	108.06	-1.67
H81-C8-H83	109.87	110.05	0.18
H82-C8-H83	109.73	108.06	-1.67

Figure 142: The PES scan for flexible dihedral corresponding to bzmo.

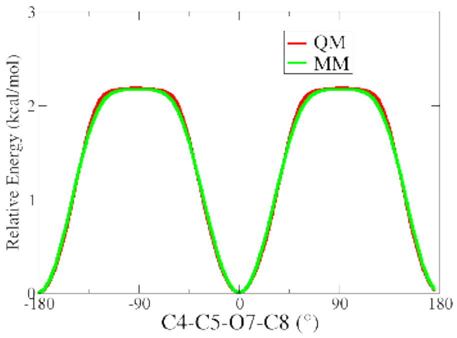

3.18 N1-(3-pyridinyl)-N1,N2-dimethylurea (pmmu, for 66A)

Figure 143: The energy-minimized structure of pmmu.

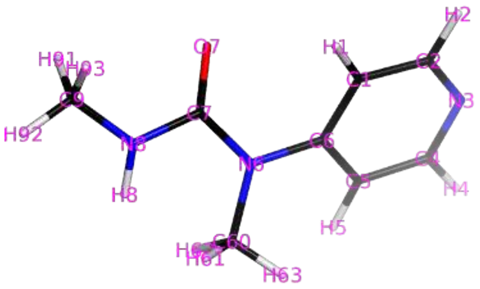

Table 252: The calculated geometric terms of pmmu.

Terms	QM	MM	diff
C1-H1	1.081	1.077	-0.005
C1-C2	1.393	1.401	0.007
C1-C6	1.402	1.408	0.006
C2-H2	1.089	1.083	-0.005
C2-N3	1.346	1.330	-0.016
N3-C4	1.344	1.329	-0.015
C4-H4	1.089	1.083	-0.006
C4-C5	1.393	1.402	0.009
C5-H5	1.086	1.076	-0.010
C5-C6	1.401	1.409	0.007
C6-N6	1.415	1.442	0.027
N6-C60	1.459	1.467	0.007
N6-C7	1.399	1.385	-0.014
C60-H61	1.091	1.113	0.022
C60-H62	1.099	1.112	0.013
C60-H63	1.091	1.112	0.022
C7-O7	1.231	1.226	-0.006
C7-N8	1.385	1.367	-0.018
N8-H8	1.012	0.990	-0.022
N8-C9	1.459	1.443	-0.016
C9-H91	1.095	1.114	0.019
C9-H92	1.091	1.112	0.021
C9-H93	1.089	1.114	0.025
H1-C1-C2	120.78	119.07	-1.72
H1-C1-C6	120.80	121.56	0.76
C2-C1-C6	118.41	119.35	0.94
C1-C2-H2	119.47	120.27	0.81
C1-C2-N3	124.88	121.88	-3.00
H2-C2-N3	115.65	117.84	2.19
C2-N3-C4	115.79	120.27	4.48
N3-C4-H4	115.93	117.64	1.71
N3-C4-C5	124.22	121.96	-2.26
H4-C4-C5	119.85	120.40	0.56
C4-C5-H5	119.51	118.77	-0.74
C4-C5-C6	119.11	119.21	0.11
H5-C5-C6	121.37	121.96	0.59
C1-C6-C5	117.59	117.32	-0.27
C1-C6-N6	122.56	122.91	0.34
C5-C6-N6	119.81	119.74	-0.07
C6-N6-C60	118.24	119.34	1.10
C6-N6-C7	120.31	120.72	0.41
C60-N6-C7	120.93	119.72	-1.20
N6-C60-H61	108.88	109.57	0.69
N6-C60-H62	113.32	108.34	-4.98
N6-C60-H63	108.42	109.55	1.13
H61-C60-H62	109.25	112.25	3.00
H61-C60-H63	108.42	106.75	-1.67
H62-C60-H63	108.46	110.37	1.91
N6-C7-O7	123.76	127.30	3.54
N6-C7-N8	114.32	113.05	-1.27
O7-C7-N8	121.86	119.60	-2.26
C7-N8-H8	116.78	117.98	1.20
C7-N8-C9	116.39	121.26	4.87
H8-N8-C9	115.05	118.98	3.93
N8-C9-H91	112.61	110.58	-2.04
N8-C9-H92	108.11	110.71	2.60
N8-C9-H93	108.83	110.48	1.65
H91-C9-H92	109.72	108.55	-1.17
H91-C9-H93	108.23	108.04	-0.20
H92-C9-H93	109.30	108.40	-0.90

Figure 144: The PES scan for flexible dihedral corresponding to pmmu.

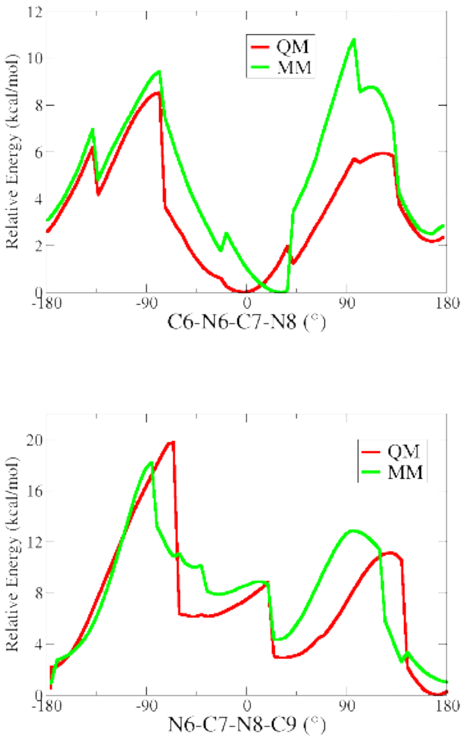

3.19 1-(2-pyridinyl)amino-3-methyl butene (bepa, for 6IA, HIA, MIA & SIA)

Figure 145: The energy-minimized structure of bepa.

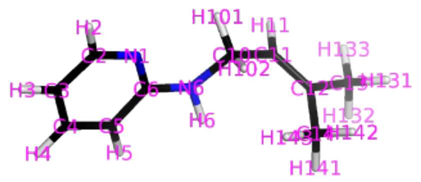

Table 253: The calculated geometric terms of bepa.

Terms	QM	MM	diff
N1-C2	1.348	1.330	-0.018
N1-C6	1.344	1.325	-0.019
C2-H2	1.089	1.083	-0.006
C2-C3	1.389	1.403	0.014
C3-H3	1.086	1.077	-0.008
C3-C4	1.400	1.403	0.003
C4-H4	1.088	1.080	-0.008
C4-C5	1.385	1.409	0.023
C5-H5	1.088	1.077	-0.011
C5-C6	1.411	1.400	-0.011
C6-N6	1.387	1.371	-0.016
N6-H6	1.015	1.014	-0.001
N6-C10	1.464	1.486	0.022
C10-H101	1.094	1.114	0.020
C10-H102	1.098	1.114	0.016
C10-C11	1.497	1.508	0.010
C11-H11	1.091	1.100	0.009
C11-C12	1.347	1.348	0.001
C12-C13	1.506	1.505	-0.001
C12-C14	1.505	1.506	0.001
C13-H131	1.097	1.111	0.015
C13-H132	1.097	1.111	0.014
C13-H133	1.093	1.112	0.019
C14-H141	1.096	1.111	0.015
C14-H142	1.096	1.111	0.015
C14-H143	1.091	1.111	0.021
C2-N1-C6	117.42	121.38	3.96
N1-C2-H2	115.21	117.83	2.62
N1-C2-C3	124.11	121.53	-2.57
H2-C2-C3	120.68	120.64	-0.05
C2-C3-H3	120.63	120.66	0.03
C2-C3-C4	117.89	117.97	0.07
H3-C3-C4	121.48	121.37	-0.10
C3-C4-H4	120.71	120.31	-0.40
C3-C4-C5	119.23	119.48	0.26
H4-C4-C5	120.06	120.20	0.14
C4-C5-H5	121.10	120.69	-0.41
C4-C5-C6	118.68	117.91	-0.76
H5-C5-C6	120.22	121.40	1.18
N1-C6-C5	122.68	121.72	-0.95
N1-C6-N6	116.98	118.35	1.36
C5-C6-N6	120.28	119.92	-0.36
C6-N6-H6	114.23	114.32	0.09
C6-N6-C10	119.15	120.77	1.62
H6-N6-C10	113.23	115.74	2.51
N6-C10-H101	107.30	109.89	2.59
N6-C10-H102	111.71	109.47	-2.25
N6-C10-C11	108.54	108.77	0.23
H101-C10-H102	106.14	106.58	0.44
H101-C10-C11	110.35	109.88	-0.47
H102-C10-C11	112.65	112.22	-0.43
C10-C11-H11	114.42	114.17	-0.25
C10-C11-C12	127.56	127.36	-0.20
H11-C11-C12	118.02	118.23	0.22
C11-C12-C13	120.41	121.28	0.87
C11-C12-C14	125.25	124.25	-1.00
C13-C12-C14	114.34	114.47	0.13
C12-C13-H131	110.75	110.52	-0.22
C12-C13-H132	110.83	110.66	-0.17
C12-C13-H133	111.60	113.05	1.45
H131-C13-H132	106.83	107.53	0.70
H131-C13-H133	108.27	107.38	-0.89
H132-C13-H133	108.39	107.47	-0.92
C12-C14-H141	110.08	110.59	0.51
C12-C14-H142	110.29	110.38	0.09
C12-C14-H143	113.29	113.57	0.28
H141-C14-H142	106.73	107.43	0.70
H141-C14-H143	108.18	107.38	-0.79
H142-C14-H143	108.04	107.22	-0.81

Figure 146: The PES scan for flexible dihedral corresponding to bepa.

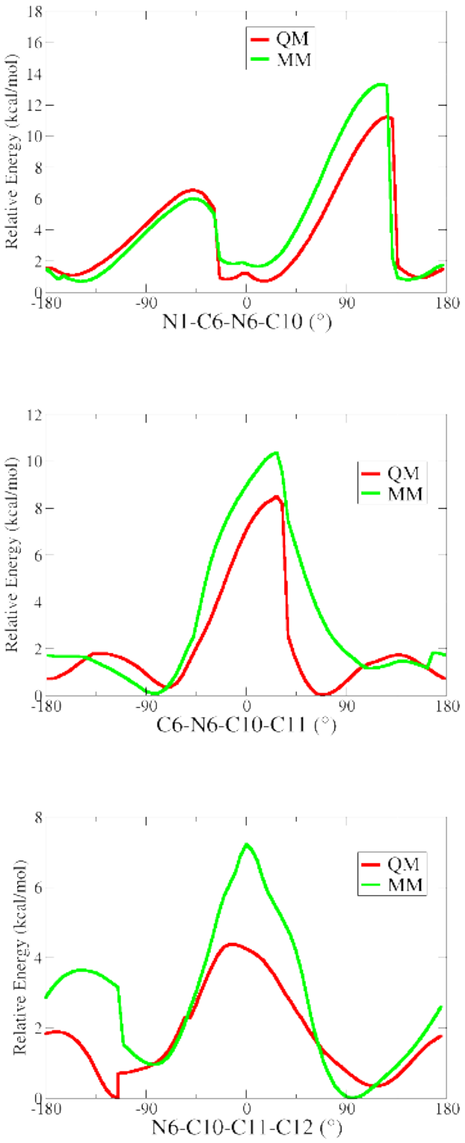

3.20 1-(3-pyrrolyl)-N-(3-cyclopentenyl)-methyl amine (cena, for QUG, MQG & GQG)

Figure 147: The energy-minimized structure of cena.

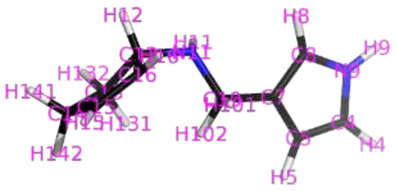

Table 254: The calculated geometric terms of cena.

Terms	QM	MM	diff
N9-H9	1.011	1.011	-0.000
N9-C8	1.373	1.378	0.005
N9-C4	1.373	1.374	0.001
C8-H8	1.081	1.083	0.002
C8-C7	1.386	1.364	-0.022
C7-C5	1.421	1.367	-0.054
C7-C10	1.498	1.517	0.019
C5-H5	1.083	1.080	-0.003
C5-C4	1.383	1.358	-0.025
C4-H4	1.081	1.084	0.002
C10-H101	1.097	1.112	0.014
C10-H102	1.104	1.109	0.005
C10-N11	1.465	1.479	0.014
N11-H11	1.021	1.020	-0.002
N11-C12	1.465	1.455	-0.010
C12-H12	1.101	1.103	0.002
C12-C13	1.551	1.515	-0.036
C12-C16	1.508	1.514	0.006
C13-H131	1.094	1.100	0.005
C13-H132	1.095	1.100	0.005
C13-C14	1.542	1.527	-0.015
C14-H141	1.099	1.102	0.004
C14-H142	1.096	1.101	0.005
C14-C15	1.509	1.514	0.005
C15-H15	1.087	1.082	-0.005
C15-C16	1.341	1.372	0.031
C16-H16	1.087	1.079	-0.008
H9-N9-C8	124.85	125.40	0.55
H9-N9-C4	124.90	125.54	0.64
C8-N9-C4	110.23	109.06	-1.18
N9-C8-H8	122.13	125.51	3.38
N9-C8-C7	107.59	107.25	-0.35
H8-C8-C7	130.28	127.25	-3.03
C8-C7-C5	107.08	107.57	0.49
C8-C7-C10	125.44	126.58	1.14
C5-C7-C10	127.47	125.81	-1.65
C7-C5-H5	126.52	124.89	-1.63
C7-C5-C4	107.85	109.88	2.04
H5-C5-C4	125.64	125.23	-0.40
N9-C4-C5	107.25	106.24	-1.01
N9-C4-H4	121.35	124.42	3.07
C5-C4-H4	131.40	129.34	-2.06
C7-C10-H101	111.08	107.72	-3.37
C7-C10-H102	108.72	108.12	-0.60
C7-C10-N11	109.54	111.76	2.22
H101-C10-H102	105.95	107.30	1.35
H101-C10-N11	107.23	110.61	3.38
H102-C10-N11	114.28	111.16	-3.12
C10-N11-H11	108.48	109.41	0.93
C10-N11-C12	115.57	115.48	-0.09
H11-N11-C12	109.72	110.61	0.89
N11-C12-H12	105.39	107.60	2.21
N11-C12-C13	118.27	115.88	-2.38
N11-C12-C16	112.28	108.86	-3.42
H12-C12-C13	109.37	111.97	2.61
H12-C12-C16	108.94	109.59	0.65
C13-C12-C16	102.42	102.74	0.32
C12-C13-H131	109.03	112.56	3.53
C12-C13-H132	112.27	111.44	-0.83
C12-C13-C14	105.50	105.97	0.46
H131-C13-H132	107.57	105.15	-2.42
H131-C13-C14	109.24	110.23	1.00
H132-C13-C14	113.16	111.62	-1.55
C13-C14-H141	111.26	110.71	-0.55
C13-C14-H142	112.92	111.67	-1.25
C13-C14-C15	102.73	103.27	0.54
H141-C14-H142	106.61	106.35	-0.26
H141-C14-C15	110.29	111.38	1.09
H142-C14-C15	113.11	113.56	0.45
C14-C15-H15	123.04	124.17	1.14
C14-C15-C16	111.73	110.02	-1.72
H15-C15-C16	125.14	125.63	0.49
C12-C16-C15	111.91	110.81	-1.10
C12-C16-H16	121.90	121.40	-0.50
C15-C16-H16	126.09	125.17	-0.92

Figure 148: The PES scan for flexible dihedral corresponding to cena.

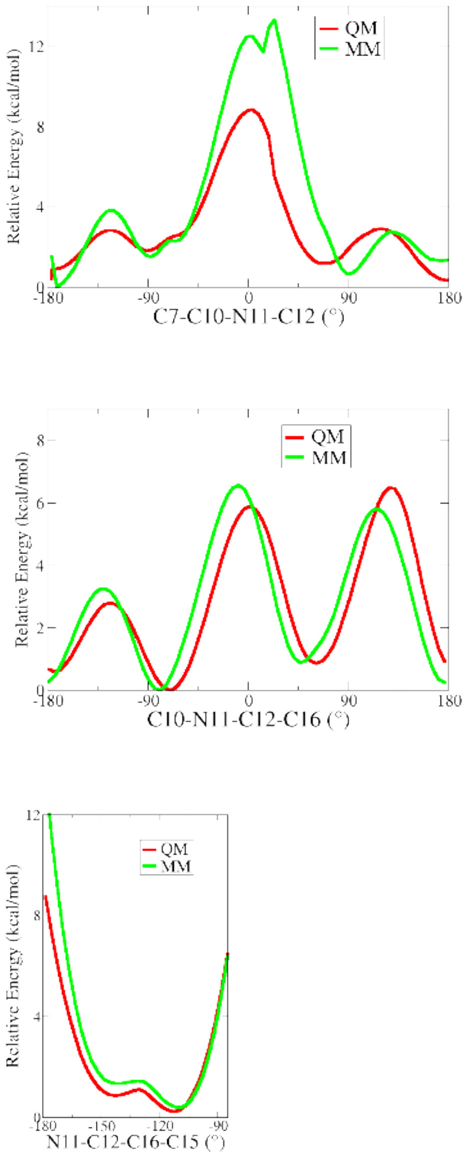

3.21 carboxyhydroxymethyl benzene (cmbz, for CMU)

Figure 149: The energy-minimized structure of cmbz.

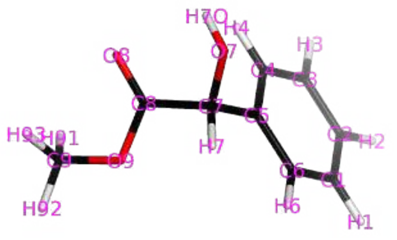

Table 255: The calculated geometric terms of cmbz.

Terms	QM	MM	diff
C1-H1	1.088	1.081	-0.007
C1-C2	1.397	1.400	0.003
C1-C6	1.395	1.401	0.006
C2-H2	1.087	1.081	-0.007
C2-C3	1.396	1.400	0.004
C3-H3	1.088	1.081	-0.007
C3-C4	1.397	1.400	0.004
C4-H4	1.085	1.081	-0.005
C4-C5	1.397	1.409	0.012
C5-C6	1.400	1.407	0.006
C5-C7	1.521	1.515	-0.006
C6-H6	1.088	1.080	-0.007
C7-H7	1.098	1.112	0.014
C7-O7	1.414	1.444	0.031
C7-C8	1.523	1.565	0.043
O7-H7O	0.979	0.966	-0.013
O8-C8	1.224	1.221	-0.003
C8-O9	1.341	1.341	-0.000
O9-C9	1.447	1.438	-0.009
C9-H91	1.091	1.114	0.023
C9-H92	1.088	1.112	0.025
C9-H93	1.090	1.114	0.023
H1-C1-C2	120.07	119.98	-0.08
H1-C1-C6	119.75	119.97	0.21
C2-C1-C6	120.18	120.05	-0.13
C1-C2-H2	120.13	120.02	-0.11
C1-C2-C3	119.66	119.97	0.30
H2-C2-C3	120.20	120.01	-0.19
C2-C3-H3	120.01	120.01	-0.01
C2-C3-C4	120.34	120.04	-0.30
H3-C3-C4	119.65	119.95	0.31
C3-C4-H4	120.77	119.56	-1.22
C3-C4-C5	119.98	120.45	0.47
H4-C4-C5	119.22	120.00	0.77
C4-C5-C6	119.74	119.03	-0.71
C4-C5-C7	119.99	120.84	0.85
C6-C5-C7	120.27	120.11	-0.16
C1-C6-C5	120.10	120.47	0.37
C1-C6-H6	120.04	119.52	-0.52
C5-C6-H6	119.86	120.01	0.15
C5-C7-H7	109.48	111.36	1.88
C5-C7-O7	112.88	112.55	-0.33
C5-C7-C8	108.59	107.87	-0.72
H7-C7-O7	108.68	107.02	-1.66
H7-C7-C8	108.58	106.96	-1.62
O7-C7-C8	108.54	110.97	2.43
C7-O7-H7O	105.32	107.32	2.00
C7-C8-O8	122.60	124.79	2.20
C7-C8-O9	112.61	110.66	-1.95
O8-C8-O9	124.78	124.55	-0.24
C8-O9-C9	114.32	112.38	-1.94
O9-C9-H91	110.03	110.85	0.82
O9-C9-H92	104.91	109.84	4.92
O9-C9-H93	110.12	110.88	0.76
H91-C9-H92	111.01	108.20	-2.81
H91-C9-H93	109.63	108.78	-0.85
H92-C9-H93	111.06	108.21	-2.84

Figure 150: The PES scan for flexible dihedral corresponding to cmbz.

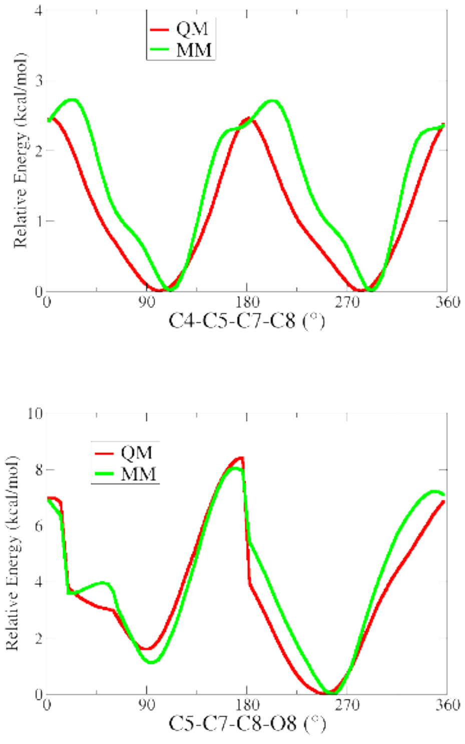

3.22 2-oxo-5-formylpyrimidine (5fop, for 5FC)

Figure 151: The energy-minimized structure of 5fop.

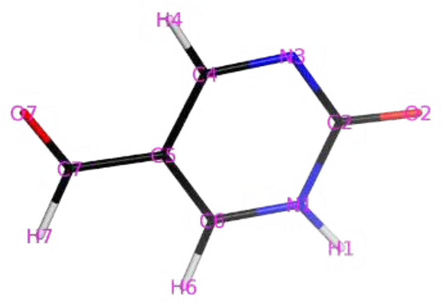

Table 256: The calculated geometric terms of 5fop.

Terms	QM	MM	diff
N1-C2	1.421	1.385	-0.037
N1-C6	1.350	1.360	0.010
N1-H1	1.019	1.006	-0.012
C2-O2	1.225	1.222	-0.003
C2-N3	1.399	1.361	-0.039
N3-C4	1.312	1.367	0.055
C4-H4	1.090	1.090	0.000
C4-C5	1.434	1.370	-0.064
C5-C6	1.372	1.368	-0.004
C5-C7	1.467	1.457	-0.010
C6-H6	1.089	1.091	0.003
C7-H7	1.110	1.111	0.001
C7-O7	1.230	1.213	-0.018
C2-N1-C6	124.30	120.70	-3.60
C2-N1-H1	114.61	112.64	-1.97
C6-N1-H1	121.09	126.66	5.57
N1-C2-O2	119.16	116.76	-2.41
N1-C2-N3	116.21	119.78	3.58
O2-C2-N3	124.63	123.46	-1.17
C2-N3-C4	118.97	118.06	-0.90
N3-C4-H4	116.55	115.75	-0.80
N3-C4-C5	125.13	123.39	-1.74
H4-C4-C5	118.31	120.85	2.54
C4-C5-C6	116.44	117.54	1.10
C4-C5-C7	122.60	119.84	-2.76
C6-C5-C7	120.96	122.62	1.66
N1-C6-C5	118.95	120.51	1.56
N1-C6-H6	117.24	117.62	0.38
C5-C6-H6	123.81	121.87	-1.94
C5-C7-H7	115.67	116.58	0.91
C5-C7-O7	123.92	125.04	1.11
H7-C7-O7	120.41	118.39	-2.02

Figure 152: The PES scan for flexible dihedral corresponding to 5fop.

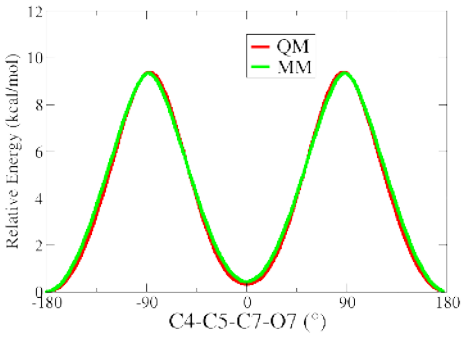

3.23 3-methylamino-1,2-epoxycyclopentene (cona, for EQG)

Figure 153: The energy-minimized structure of cona.

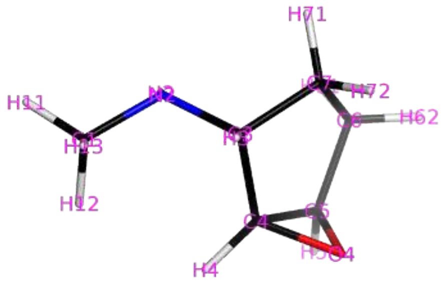

Table 257: The calculated geometric terms of cona.

Terms	QM	MM	diff
C1-H11	1.093	1.114	0.021
C1-H12	1.101	1.111	0.010
C1-H13	1.094	1.114	0.020
C1-N2	1.462	1.470	0.007
N2-H2	1.021	1.021	0.000
N2-C3	1.465	1.453	-0.012
C3-H3	1.097	1.101	0.004
C3-C4	1.519	1.531	0.012
C3-C7	1.541	1.544	0.002
C4-H4	1.089	1.099	0.010
C4-O4	1.449	1.437	-0.012
C4-C5	1.468	1.475	0.007
O4-C5	1.449	1.440	-0.009
C5-H5	1.089	1.100	0.012
C5-C6	1.509	1.509	0.000
C6-H61	1.098	1.101	0.003
C6-H62	1.095	1.099	0.004
C6-C7	1.542	1.547	0.005
C7-H71	1.093	1.098	0.006
C7-H72	1.092	1.102	0.010
H11-C1-H12	108.11	107.74	-0.37
H11-C1-H13	107.86	107.33	-0.53
H11-C1-N2	108.76	110.60	1.84
H12-C1-H13	107.89	108.10	0.21
H12-C1-N2	115.49	112.00	-3.49
H13-C1-N2	108.49	110.89	2.40
C1-N2-H2	109.31	106.98	-2.33
C1-N2-C3	113.81	113.41	-0.40
H2-N2-C3	108.81	111.29	2.48
N2-C3-H3	107.27	107.77	0.50
N2-C3-C4	112.68	112.02	-0.66
N2-C3-C7	111.05	111.19	0.14
H3-C3-C4	111.95	111.90	-0.05
H3-C3-C7	111.06	111.66	0.60
C4-C3-C7	102.89	102.32	-0.57
C3-C4-H4	121.60	121.96	0.35
C3-C4-O4	113.86	116.16	2.30
C3-C4-C5	108.89	108.72	-0.18
H4-C4-O4	114.94	115.16	0.23
H4-C4-C5	122.50	119.72	-2.78
C4-C5-H5	122.55	120.98	-1.57
C4-C5-C6	109.43	110.18	0.75
O4-C5-H5	114.87	115.11	0.24
O4-C5-C6	112.79	112.42	-0.37
H5-C5-C6	121.80	122.54	0.74
C5-C6-H61	109.32	109.97	0.65
C5-C6-H62	112.45	112.26	-0.18
C5-C6-C7	103.26	103.18	-0.08
H61-C6-H62	107.47	107.74	0.27
H61-C6-C7	111.88	111.26	-0.62
H62-C6-C7	112.49	112.44	-0.05
C3-C7-C6	105.98	107.36	1.38
C3-C7-H71	110.32	112.64	2.32
C3-C7-H72	109.40	108.68	-0.72
C6-C7-H71	113.27	112.40	-0.87
C6-C7-H72	109.32	109.75	0.43
H71-C7-H72	108.50	105.96	-2.54

Figure 154: The PES scan for flexible dihedral corresponding to cona.

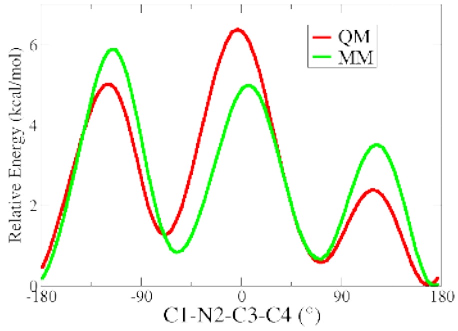

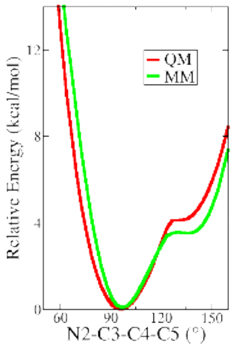

3.24 5-hydroxyl-cyclopenten-3-yl ammonium (cpea, for QUG, MQG & GQG)

Figure 155: The energy-minimized structure of cpea.

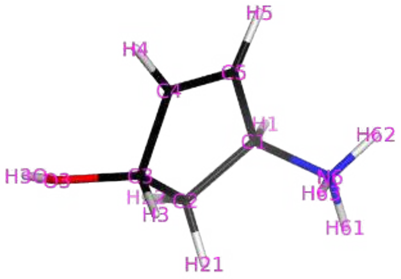

Table 258: The calculated geometric terms of cpea.

Terms	QM	MM	diff
C1-H1	1.094	1.085	-0.008
C1-C2	1.530	1.518	-0.011
C1-C5	1.500	1.495	-0.006
C1-N6	1.540	1.484	-0.056
C2-H21	1.095	1.100	0.004
C2-H22	1.094	1.108	0.014
C2-C3	1.539	1.516	-0.023
C3-H3	1.101	1.105	0.004
C3-O3	1.417	1.413	-0.004
C3-C4	1.512	1.512	0.000
O3-H3O	0.974	0.963	-0.011
C4-H4	1.087	1.085	-0.002
C4-C5	1.343	1.373	0.030
C5-H5	1.086	1.084	-0.003
N6-H61	1.029	1.039	0.009
N6-H62	1.029	1.039	0.010
N6-H63	1.029	1.039	0.011
H1-C1-C2	114.34	115.20	0.86
H1-C1-C5	114.99	113.28	-1.71
H1-C1-N6	104.29	106.70	2.42
C2-C1-C5	104.51	104.68	0.16
C2-C1-N6	110.01	109.78	-0.23
C5-C1-N6	108.64	106.93	-1.71
C1-C2-H21	114.41	114.21	-0.20
C1-C2-H22	108.35	107.85	-0.49
C1-C2-C3	106.06	107.47	1.41
H21-C2-H22	107.35	104.48	-2.87
H21-C2-C3	114.05	111.86	-2.19
H22-C2-C3	106.22	110.91	4.70
C2-C3-H3	112.23	111.89	-0.34
C2-C3-O3	106.74	108.48	1.74
C2-C3-C4	102.75	103.37	0.63
H3-C3-O3	110.65	109.73	-0.92
H3-C3-C4	110.78	110.17	-0.61
O3-C3-C4	113.43	113.08	-0.35
C3-O3-H3O	109.26	111.54	2.28
C3-C4-H4	122.51	121.92	-0.58
C3-C4-C5	112.68	111.32	-1.36
H4-C4-C5	124.81	126.12	1.31
C1-C5-C4	110.52	110.51	-0.01
C1-C5-H5	123.15	123.56	0.41
C4-C5-H5	126.33	124.89	-1.44
C1-N6-H61	111.98	110.06	-1.92
C1-N6-H62	111.41	109.61	-1.80
C1-N6-H63	110.10	107.88	-2.21
H61-N6-H62	107.73	110.14	2.41
H61-N6-H63	108.20	109.84	1.63
H62-N6-H63	107.24	109.27	2.03

Figure 156: The PES scan for flexible dihedral corresponding to cpea.

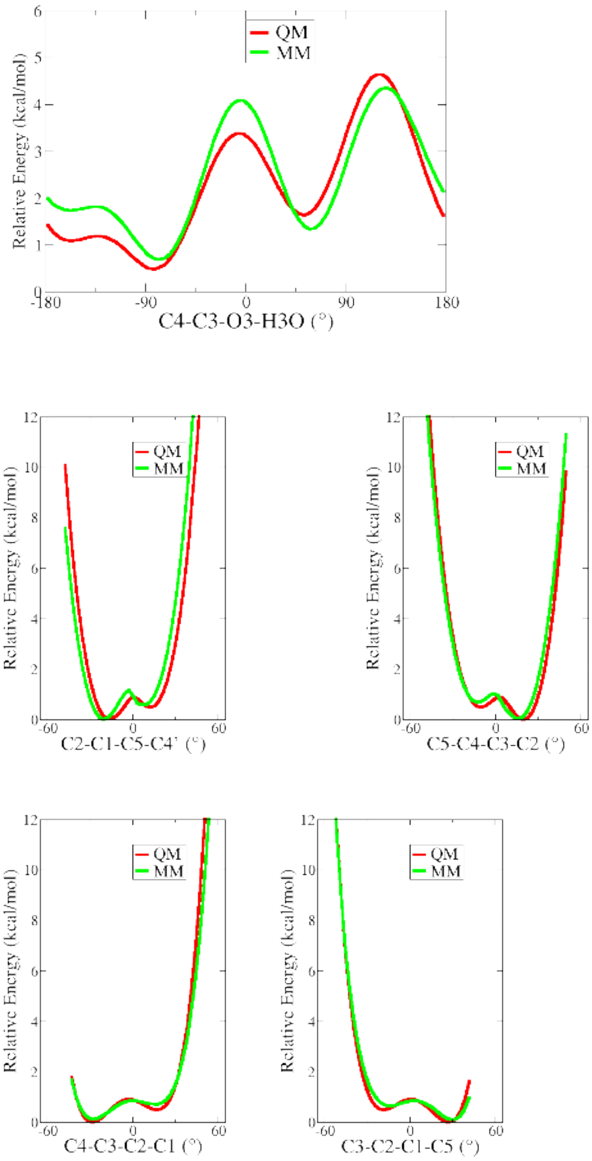

3.25 epoxycyclopenten-3-yl ammonium (cpoa, for EQG)

Figure 157: The energy-minimized structure of cpoa.

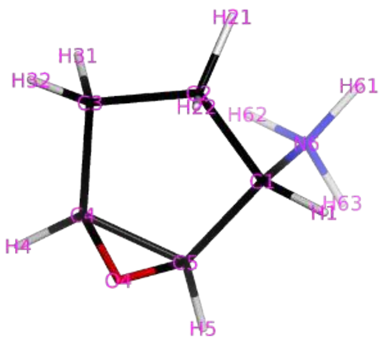

Table 259: The calculated geometric terms of cpoa.

Terms	QM	MM	diff
C1-H1	1.093	1.087	-0.007
C1-C2	1.534	1.553	0.019
C1-C5	1.513	1.502	-0.011
C1-N6	1.526	1.486	-0.040
C2-H21	1.095	1.098	0.002
C2-H22	1.091	1.106	0.015
C2-C3	1.543	1.537	-0.007
C3-H31	1.098	1.101	0.003
C3-H32	1.093	1.102	0.009
C3-C4	1.510	1.507	-0.002
C4-H4	1.087	1.103	0.016
C4-O4	1.446	1.436	-0.010
C4-C5	1.475	1.478	0.003
O4-C5	1.430	1.435	0.005
C5-H5	1.090	1.076	-0.014
N6-H61	1.029	1.039	0.010
N6-H62	1.029	1.039	0.010
N6-H63	1.030	1.039	0.010
H1-C1-C2	114.03	116.95	2.92
H1-C1-C5	114.45	110.65	-3.80
H1-C1-N6	105.97	108.42	2.44
C2-C1-C5	104.60	104.68	0.09
C2-C1-N6	110.03	108.14	-1.89
C5-C1-N6	107.63	107.61	-0.02
C1-C2-H21	113.06	114.45	1.39
C1-C2-H22	107.16	107.33	0.17
C1-C2-C3	105.77	106.73	0.96
H21-C2-H22	107.06	105.54	-1.52
H21-C2-C3	113.68	113.62	-0.06
H22-C2-C3	109.94	108.92	-1.02
C2-C3-H31	112.73	112.83	0.10
C2-C3-H32	111.52	112.26	0.75
C2-C3-C4	103.35	102.53	-0.81

Terms	QM	MM	diff
H31-C3-H32	107.05	107.04	-0.01
H31-C3-C4	110.04	110.65	0.61
H32-C3-C4	112.23	111.59	-0.65
C3-C4-H4	122.46	121.75	-0.72
C3-C4-O4	112.09	113.34	1.26
C3-C4-C5	109.75	111.30	1.55
H4-C4-O4	115.16	114.28	-0.88
H4-C4-C5	121.96	121.08	-0.88
C1-C5-C4	108.02	107.55	-0.47
C1-C5-O4	110.27	110.58	0.31
C1-C5-H5	121.71	123.19	1.48
C4-C5-H5	124.81	123.61	-1.20
O4-C5-H5	116.24	115.69	-0.55
C1-N6-H61	111.32	109.69	-1.63
C1-N6-H62	110.66	108.62	-2.04
C1-N6-H63	112.07	109.81	-2.25
H61-N6-H62	107.64	109.40	1.77
H61-N6-H63	107.46	109.73	2.27
H62-N6-H63	107.49	109.56	2.07

Figure 158: The PES scan for flexible dihedral corresponding to cpoa.

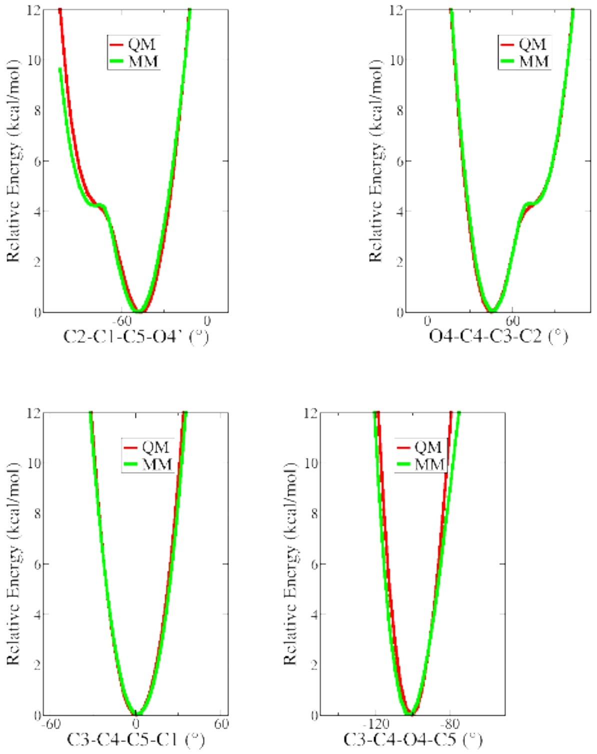

3.26 cyanomethylbenzene (cybz, for CYU)

Figure 159: The energy-minimized structure of cybz.

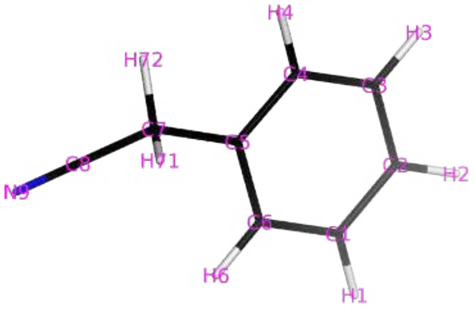

Table 260: The calculated geometric terms of cybz.

Terms	QM	MM	diff
C1-H1	1.087	1.081	-0.007
C1-C2	1.395	1.400	0.005
C1-C6	1.397	1.401	0.003
C2-H2	1.087	1.081	-0.006
C2-C3	1.397	1.400	0.003
C3-H3	1.087	1.081	-0.007
C3-C4	1.394	1.401	0.007
C4-H4	1.089	1.080	-0.009
C4-C5	1.401	1.406	0.006
C5-C6	1.397	1.408	0.011
C5-C7	1.520	1.511	-0.009
C6-H6	1.087	1.080	-0.007
C7-H71	1.097	1.110	0.013
C7-H72	1.097	1.110	0.014
C7-C8	1.464	1.471	0.006
C8-N9	1.202	1.179	-0.023
H1-C1-C2	120.09	120.01	-0.08
H1-C1-C6	119.49	120.01	0.52
C2-C1-C6	120.41	119.98	-0.44
C1-C2-H2	120.21	119.97	-0.23
C1-C2-C3	119.62	120.02	0.40
H2-C2-C3	120.18	120.01	-0.17
C2-C3-H3	120.17	119.96	-0.21
C2-C3-C4	120.05	119.98	-0.08
H3-C3-C4	119.77	120.06	0.29
C3-C4-H4	119.73	119.47	-0.26
C3-C4-C5	120.46	120.53	0.07
H4-C4-C5	119.81	120.00	0.19
C4-C5-C6	119.36	118.98	-0.38
C4-C5-C7	118.07	119.80	1.73
C6-C5-C7	122.56	121.18	-1.39
C1-C6-C5	120.09	120.50	0.41
C1-C6-H6	119.82	119.48	-0.34
C5-C6-H6	120.09	120.02	-0.07
C5-C7-H71	110.12	107.48	-2.64
C5-C7-H72	110.07	109.39	-0.69
C5-C7-C8	113.70	115.25	1.55
H71-C7-H72	106.45	108.70	2.25
H71-C7-C8	108.11	108.27	0.15
H72-C7-C8	108.10	107.61	-0.49

Figure 160: The PES scan for flexible dihedral corresponding to cybz.

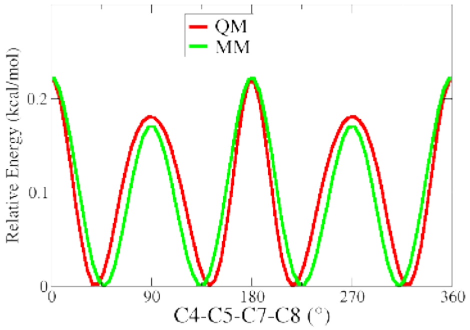

3.27 N-methyl phenylethylamine (ambz, for 5AU, U8U, 5DU, SCU, etc.)

Figure 161: The energy-minimized structure of ambz.

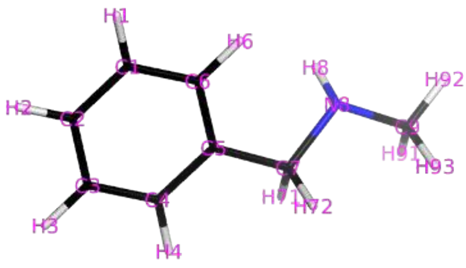

Table 261: The calculated geometric terms of ambz.

Terms	QM	MM	diff
C1-H1	1.088	1.081	-0.007
C1-C2	1.398	1.400	0.002
C1-C6	1.395	1.401	0.006
C2-H2	1.087	1.080	-0.007
C2-C3	1.396	1.400	0.004
C3-H3	1.088	1.081	-0.007
C3-C4	1.397	1.401	0.004
C4-H4	1.089	1.080	-0.009
C4-C5	1.399	1.407	0.008
C5-C6	1.402	1.411	0.010
C5-C7	1.508	1.519	0.011
C6-H6	1.087	1.080	-0.007
C7-H71	1.107	1.113	0.007
C7-H72	1.099	1.113	0.014
C7-N8	1.461	1.481	0.020
N8-H8	1.020	1.024	0.004
N8-C9	1.460	1.467	0.007
C9-H91	1.103	1.113	0.010
C9-H92	1.093	1.114	0.021
C9-H93	1.094	1.114	0.020
H1-C1-C2	119.95	120.06	0.11
H1-C1-C6	119.79	119.94	0.15
C2-C1-C6	120.26	120.00	-0.26
C1-C2-H2	120.17	119.98	-0.19
C1-C2-C3	119.67	119.99	0.31
H2-C2-C3	120.15	120.03	-0.12
C2-C3-H3	120.11	120.00	-0.11
C2-C3-C4	120.01	119.95	-0.06
H3-C3-C4	119.88	120.05	0.16
C3-C4-H4	119.81	119.57	-0.24
C3-C4-C5	120.64	120.83	0.20
H4-C4-C5	119.55	119.59	0.04
C4-C5-C6	119.05	118.54	-0.51
C4-C5-C7	121.04	117.84	-3.20
C6-C5-C7	119.91	123.59	3.68
C1-C6-C5	120.37	120.69	0.31
C1-C6-H6	120.76	119.38	-1.38
C5-C6-H6	118.86	119.93	1.07
C5-C7-H71	109.39	108.10	-1.30
C5-C7-H72	109.99	106.98	-3.01
C5-C7-N8	110.81	113.69	2.88
H71-C7-H72	106.27	108.58	2.31
H71-C7-N8	113.07	109.73	-3.34
H72-C7-N8	107.17	109.61	2.45
C7-N8-H8	108.06	111.67	3.61
C7-N8-C9	111.93	111.10	-0.84
H8-N8-C9	109.30	106.70	-2.60
N8-C9-H91	114.05	111.49	-2.56
N8-C9-H92	109.28	110.87	1.59
N8-C9-H93	108.87	110.78	1.91
H91-C9-H92	108.59	108.06	-0.52
H91-C9-H93	107.86	108.00	0.15
H92-C9-H93	108.03	107.47	-0.56

Figure 162: The PES scan for flexible dihedral corresponding to ambz.

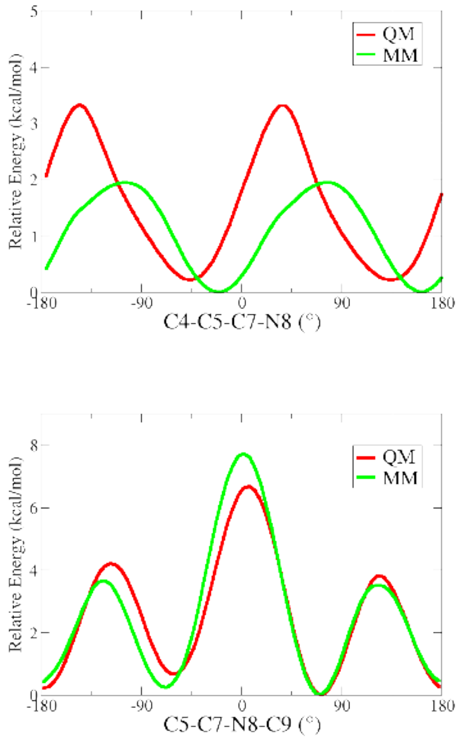

3.28 3-methylamino-propene (penm, for IAU, ISU, etc)

Figure 163: The energy-minimized structure of penm.

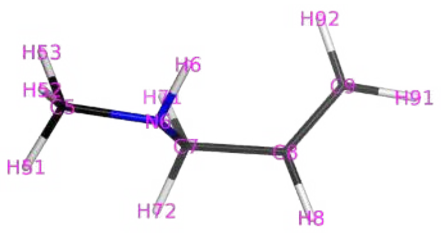

Table 262: The calculated geometric terms of penm.

Terms	QM	MM	diff
C5-H51	1.094	1.114	0.020
C5-H52	1.093	1.114	0.021
C5-H53	1.103	1.113	0.010
C5-N6	1.460	1.467	0.008
N6-H6	1.020	1.023	0.003
N6-C7	1.462	1.472	0.009
C7-H71	1.106	1.114	0.008
C7-H72	1.098	1.115	0.016
C7-C8	1.498	1.504	0.006
C8-H8	1.089	1.102	0.013
C8-C9	1.339	1.349	0.010
C9-H91	1.085	1.101	0.016
C9-H92	1.087	1.101	0.014
H51-C5-H52	107.98	107.50	-0.48
H51-C5-H53	107.86	108.02	0.16
H51-C5-N6	108.89	110.79	1.91
H52-C5-H53	108.55	108.07	-0.48
H52-C5-N6	109.30	110.84	1.54
H53-C5-N6	114.09	111.47	-2.62
C5-N6-H6	109.05	107.18	-1.87
C5-N6-C7	111.98	111.82	-0.16
H6-N6-C7	107.88	111.36	3.48
N6-C7-H71	113.32	110.36	-2.96
N6-C7-H72	107.15	109.36	2.21
N6-C7-C8	109.94	108.59	-1.35
H71-C7-H72	106.67	107.30	0.63
H71-C7-C8	109.75	110.92	1.17
H72-C7-C8	109.89	110.30	0.40
C7-C8-H8	115.78	114.27	-1.51
C7-C8-C9	123.91	127.30	3.38
H8-C8-C9	120.30	118.26	-2.04
C8-C9-H91	121.81	121.03	-0.78
C8-C9-H92	121.46	120.60	-0.85
H91-C9-H92	116.73	118.36	1.64

Figure 164: The PES scan for flexible dihedral corresponding to penm.

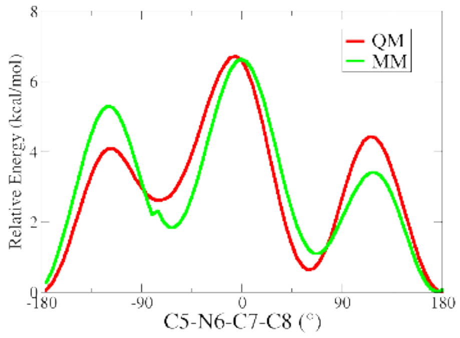

3.29 5-methoxyuracil, enol form (enou, for 5-suburidines)

Figure 165: The energy-minimized structure of enou.

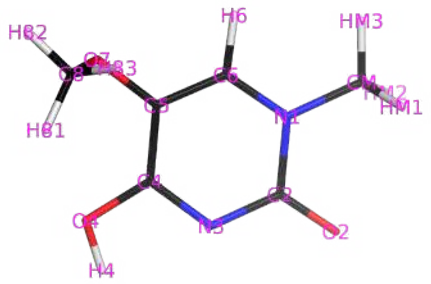

Table 263: The calculated geometric terms of enou.

Terms	QM	MM	diff
N1-C2	1.417	1.418	0.001
N1-C6	1.362	1.363	0.001
N1-CM	1.462	1.475	0.013
C2-O2	1.229	1.230	0.001
C2-N3	1.386	1.363	-0.022
N3-C4	1.306	1.389	0.083
C4-O4	1.351	1.347	-0.004
N3-O4	2.285	2.267	-0.018
C4-H4	1.862	1.826	-0.036
C4-C5	1.429	1.393	-0.035
O4-H4	0.981	0.968	-0.013
C5-C6	1.366	1.363	-0.003
C5-O7	1.371	1.372	0.001
C6-H6	1.086	1.088	0.002
O7-C8	1.440	1.431	-0.009
C8-H81	1.092	1.114	0.022
C8-H82	1.090	1.113	0.023
C8-H83	1.095	1.114	0.019
C2-N1-C6	122.36	119.67	-2.69
C2-N1-CM	116.00	118.89	2.89
C6-N1-CM	121.63	121.43	-0.20
N1-C2-O2	119.30	119.04	-0.26
N1-C2-N3	116.22	118.90	2.68
O2-C2-N3	124.48	122.06	-2.42
C2-N3-C4	120.60	119.43	-1.17
N3-C4-O4	118.58	111.95	-6.64
N3-C4-C5	124.52	122.36	-2.16
O4-C4-C5	116.90	125.69	8.80
C4-O4-H4	104.81	102.96	-1.85
C4-C5-C6	115.44	116.77	1.33
C4-C5-O7	123.50	123.53	0.03
C6-C5-O7	120.93	119.64	-1.30
N1-C6-C5	120.85	122.83	1.98
N1-C6-H6	117.85	117.18	-0.66
C5-C6-H6	121.29	119.98	-1.31
C5-O7-C8	112.96	109.92	-3.04
O7-C8-H81	110.88	111.54	0.66
O7-C8-H82	105.57	108.64	3.07
O7-C8-H83	110.29	111.97	1.67
H81-C8-H82	109.98	107.37	-2.62
H81-C8-H83	110.12	109.37	-0.75
H82-C8-H83	109.90	107.78	-2.12
C6-N1-C2-O2	-178.85	-178.76	0.09
C6-N1-C2-N3	1.41	1.32	-0.09
CM-N1-C2-O2	0.68	0.27	-0.41
CM-N1-C2-N3	-179.06	-179.64	-0.59
C2-N1-C6-C5	-1.17	-0.45	0.72
C2-N1-C6-H6	-179.78	-179.35	0.43
CM-N1-C6-C5	179.33	-179.46	1.21
CM-N1-C6-H6	0.71	1.64	0.92
N1-C2-N3-C4	-0.47	-0.22	0.25
O2-C2-N3-C4	179.81	179.87	0.06
C2-N3-C4-O4	179.21	177.71	-1.50
C2-N3-C4-C5	-0.71	-1.80	-1.08
N3-C4-O4-H4	-0.62	-3.17	-2.55
C5-C4-O4-H4	179.31	176.32	-2.99
N3-C4-C5-C6	0.98	2.61	1.64
N3-C4-C5-O7	176.85	179.67	2.82
O4-C4-C5-C6	-178.95	-176.82	2.12
O4-C4-C5-O7	-3.07	0.23	3.31
C4-C5-C6-N1	-0.01	-1.46	-1.45
C4-C5-C6-H6	178.56	177.41	-1.14
O7-C5-C6-N1	-176.00	-178.64	-2.64
O7-C5-C6-H6	2.57	0.23	-2.33
C4-C5-O7-C8	72.26	72.31	0.06
C6-C5-O7-C8	-112.09	-110.71	1.38

Figure 166: The PES scan for flexible dihedral corresponding to enou.

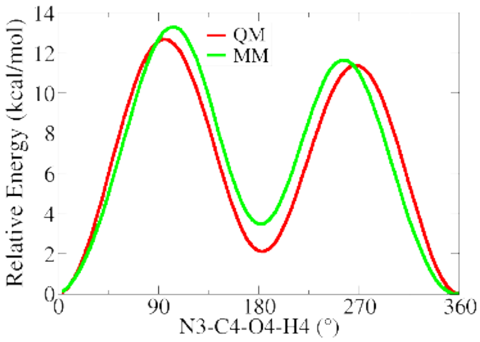

3.30 1-(3-cyclopentenoxy) tetrahydropyran (pepr, for QUG, MQG & GQG)

Figure 167: The energy-minimized structure of pepr.

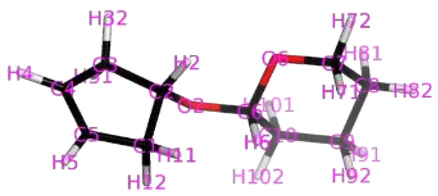

Table 264: The calculated geometric terms of pepr.

Terms	QM	MM	diff
C1-H11	1.095	1.100	0.005
C1-H12	1.099	1.102	0.004
C1-C5	1.506	1.517	0.011
C1-C2	1.535	1.521	-0.014
C3-H31	1.098	1.102	0.005
C3-H32	1.093	1.101	0.008
C3-C4	1.507	1.517	0.011
C3-C2	1.540	1.518	-0.022
C4-H4	1.087	1.083	-0.004
C4-C5	1.343	1.369	0.026
C5-H5	1.086	1.083	-0.003
C2-H2	1.101	1.104	0.004
C2-O2	1.432	1.426	-0.006
O2-C6	1.392	1.418	0.026
C6-H6	1.111	1.114	0.003
C6-O6	1.427	1.424	-0.003
C6-C10	1.515	1.540	0.024
O6-C7	1.431	1.423	-0.008
C7-H71	1.103	1.112	0.009
C7-H72	1.093	1.113	0.020
C7-C8	1.522	1.537	0.015
C8-H81	1.096	1.114	0.018
C8-H82	1.097	1.111	0.014
C8-C9	1.529	1.530	0.002
C9-H91	1.095	1.112	0.017
C9-H92	1.098	1.114	0.015
C9-C10	1.530	1.533	0.002
C10-H101	1.095	1.113	0.017
C10-H102	1.095	1.110	0.015
H11-C1-H12	107.32	106.29	-1.04
H11-C1-C5	114.28	112.89	-1.39
H11-C1-C2	112.42	111.03	-1.40
H12-C1-C5	111.52	111.65	0.14
H12-C1-C2	108.90	110.81	1.91
C5-C1-C2	102.34	104.28	1.94
H31-C3-H32	107.11	105.99	-1.12
H31-C3-C4	111.84	111.84	0.00
H31-C3-C2	108.92	110.85	1.93
H32-C3-C4	114.53	113.14	-1.38
H32-C3-C2	112.29	110.79	-1.50
C4-C3-C2	102.09	104.34	2.25
C3-C4-H4	123.44	123.55	0.11
C3-C4-C5	111.53	110.04	-1.50
H4-C4-C5	125.00	126.11	1.11
C1-C5-C4	111.26	110.02	-1.25
C1-C5-H5	123.43	123.76	0.33
C4-C5-H5	125.25	125.91	0.66
C1-C2-C3	105.14	104.83	-0.31
C1-C2-H2	109.57	111.20	1.64
C1-C2-O2	109.04	109.11	0.08
C3-C2-H2	109.27	111.17	1.90
C3-C2-O2	114.62	108.26	-6.36
H2-C2-O2	109.08	111.98	2.90
C2-O2-C6	114.10	110.90	-3.20
O2-C6-H6	110.34	109.85	-0.50
O2-C6-O6	108.56	111.14	2.58
O2-C6-C10	108.45	108.15	-0.30
H6-C6-O6	107.88	109.73	1.85
H6-C6-C10	110.81	108.70	-2.11
O6-C6-C10	110.77	109.22	-1.55
C6-O6-C7	111.07	112.44	1.37
O6-C7-H71	109.86	109.56	-0.30
O6-C7-H72	105.65	108.75	3.10
O6-C7-C8	110.71	111.46	0.75
H71-C7-H72	108.19	107.76	-0.43
H71-C7-C8	110.24	109.81	-0.44
H72-C7-C8	112.06	109.42	-2.64
C7-C8-H81	108.45	107.76	-0.69
C7-C8-H82	109.98	110.36	0.38
C7-C8-C9	109.76	111.25	1.50
H81-C8-H82	107.64	108.13	0.50
H81-C8-C9	109.88	108.59	-1.30
H82-C8-C9	111.07	110.62	-0.45
C8-C9-H91	110.88	110.67	-0.21
C8-C9-H92	109.21	109.27	0.06
C8-C9-C10	110.22	108.96	-1.26
H91-C9-H92	106.67	108.18	1.51
H91-C9-C10	110.42	110.59	0.17
H92-C9-C10	109.36	109.15	-0.21
C6-C10-C9	110.06	111.83	1.78
C6-C10-H101	106.81	107.65	0.85
C6-C10-H102	109.32	109.33	0.01
C9-C10-H101	110.75	109.32	-1.42
C9-C10-H102	111.76	110.62	-1.14
H101-C10-H102	108.01	107.96	-0.05

Figure 168: The PES scan for flexible dihedral corresponding to pepr.

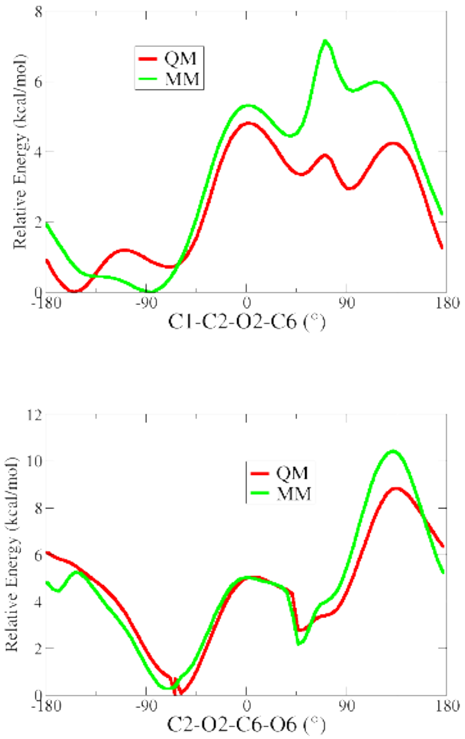

3.31 2-hydroxyl propionate methylester (hpme, for CMU)

Figure 169: The energy-minimized structure of hpme.

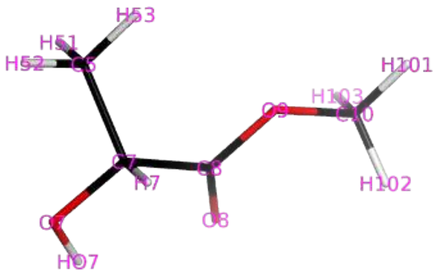

Table 265: The calculated geometric terms of hpme.

Terms	QM	MM	diff
C5-H51	1.094	1.111	0.017
C5-H52	1.093	1.111	0.018
C5-H53	1.093	1.110	0.017
C5-C7	1.522	1.545	0.023
C7-H7	1.100	1.111	0.011
C7-O7	1.422	1.439	0.017
C7-C8	1.518	1.572	0.054
O7-HO7	0.979	0.968	-0.011
C8-O8	1.227	1.220	-0.006
C8-O9	1.345	1.338	-0.007
O9-C10	1.450	1.439	-0.011
C10-H101	1.088	1.112	0.024
C10-H102	1.091	1.114	0.023
C10-H103	1.091	1.114	0.023
H51-C5-H52	108.38	108.28	-0.10
H51-C5-H53	109.53	108.82	-0.71
H51-C5-C7	109.96	110.41	0.45
H52-C5-H53	109.27	108.62	-0.64
H52-C5-C7	108.66	110.02	1.36
H53-C5-C7	110.99	110.63	-0.36
C5-C7-H7	109.62	109.33	-0.30
C5-C7-O7	109.36	107.47	-1.89
C5-C7-C8	112.08	112.83	0.76
H7-C7-O7	109.30	108.72	-0.58
H7-C7-C8	107.01	107.42	0.40
O7-C7-C8	109.42	111.02	1.60
C7-O7-HO7	107.08	107.43	0.35
C7-C8-O8	123.52	124.68	1.16
C7-C8-O9	112.12	110.48	-1.64
O8-C8-O9	124.35	124.82	0.47
C8-O9-C10	114.85	112.44	-2.42
O9-C10-H101	104.77	109.85	5.08
O9-C10-H102	109.95	110.90	0.96
O9-C10-H103	109.94	110.88	0.94
H101-C10-H102	111.15	108.20	-2.95
H101-C10-H103	111.14	108.18	-2.96
H102-C10-H103	109.79	108.75	-1.05

Figure 170: The PES scan for flexible dihedral corresponding to hpme.

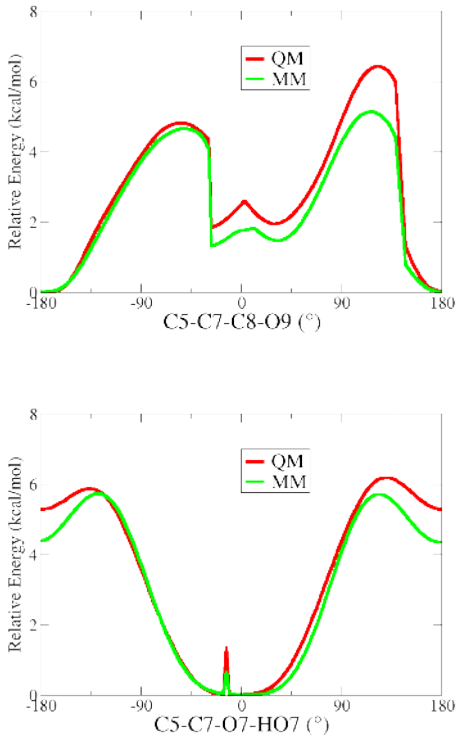

3.32 2-hydroxyl propanoate (hpat, for HCU)

Figure 171: The energy-minimized structure of hpat.

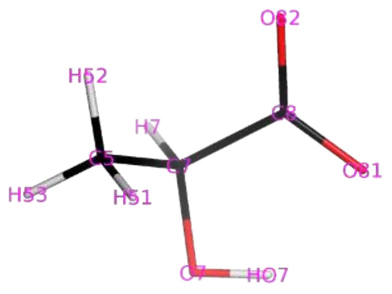

Table 266: The calculated geometric terms of hpat.

Terms	QM	MM	diff
C5-H51	1.096	1.110	0.014
C5-H52	1.094	1.110	0.016
C5-H53	1.097	1.109	0.012
C5-C7	1.522	1.543	0.022
C7-H7	1.100	1.110	0.009
C7-O7	1.435	1.433	-0.002
C7-C8	1.556	1.566	0.010
O7-HO7	0.994	0.977	-0.017
C8-O81	1.278	1.264	-0.014
C8-O82	1.262	1.259	-0.003
H51-C5-H52	108.97	108.62	-0.35
H51-C5-H53	108.39	109.06	0.67
H51-C5-C7	109.34	109.83	0.48
H52-C5-H53	109.68	109.35	-0.33
H52-C5-C7	110.06	109.72	-0.34
H53-C5-C7	110.36	110.23	-0.13
C5-C7-H7	109.49	109.79	0.31
C5-C7-O7	109.83	107.78	-2.04
C5-C7-C8	111.31	112.05	0.74
H7-C7-O7	108.63	109.09	0.46
H7-C7-C8	107.83	107.12	-0.71
O7-C7-C8	109.70	110.97	1.27
C7-O7-HO7	100.05	97.39	-2.66
C7-C8-O81	114.30	116.50	2.19
C7-C8-O82	116.30	115.94	-0.35
O81-C8-O82	129.40	127.56	-1.84

Figure 172: The PES scan for flexible dihedral corresponding to hpat.

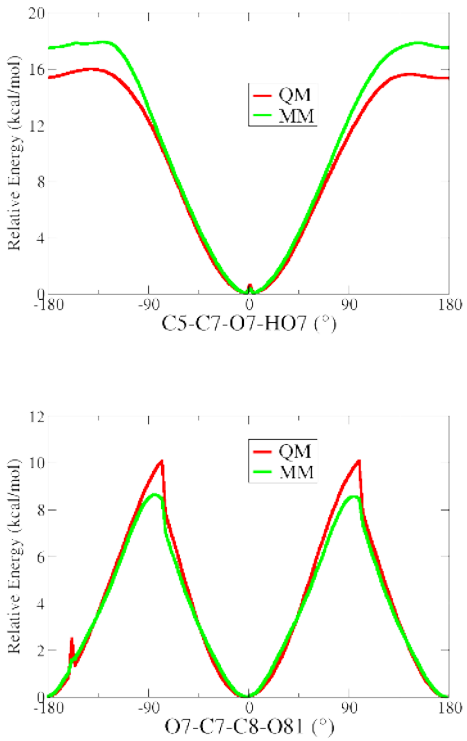

3.33 1-hydroxyl-2-methyl butene (hmbt, for HIA & SIA)

Figure 173: The energy-minimized structure of hmbt.

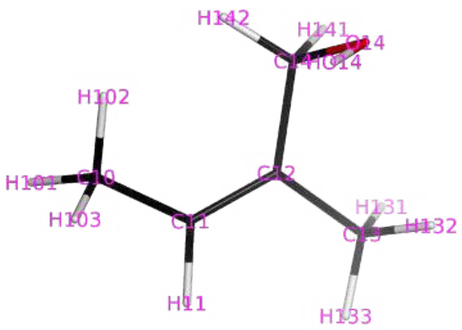

Table 267: The calculated geometric terms of hmbt.

Terms	QM	MM	diff
C10-H101	1.096	1.112	0.016
C10-H102	1.091	1.110	0.019
C10-H103	1.095	1.112	0.017
C10-C11	1.501	1.512	0.012
C11-H11	1.091	1.101	0.010
C11-C12	1.346	1.352	0.005
C12-C13	1.505	1.510	0.005
C12-C14	1.501	1.513	0.012
C13-H131	1.097	1.111	0.014
C13-H132	1.093	1.112	0.019
C13-H133	1.093	1.112	0.019
C14-H141	1.102	1.113	0.011
C14-H142	1.096	1.112	0.016
C14-O14	1.435	1.426	-0.009
O14-HO14	0.972	0.962	-0.010
H101-C10-H102	107.87	107.32	-0.55
H101-C10-H103	106.88	107.23	0.35
H101-C10-C11	110.88	110.76	-0.12
H102-C10-H103	107.72	106.76	-0.96
H102-C10-C11	113.14	113.91	0.77
H103-C10-C11	110.10	110.55	0.45
C10-C11-H11	114.48	113.99	-0.49
C10-C11-C12	128.76	128.33	-0.43
H11-C11-C12	116.76	117.68	0.92
C11-C12-C13	121.42	118.73	-2.68
C11-C12-C14	124.26	124.02	-0.25
C13-C12-C14	114.32	117.25	2.93
C12-C13-H131	110.80	110.60	-0.20
C12-C13-H132	110.44	110.24	-0.21
C12-C13-H133	111.17	113.77	2.60
H131-C13-H132	107.09	107.17	0.07
H131-C13-H133	108.33	107.44	-0.88
H132-C13-H133	108.89	107.35	-1.53
C12-C14-H141	108.80	109.36	0.56
C12-C14-H142	111.87	114.09	2.22
C12-C14-O14	107.56	110.06	2.50
H141-C14-H142	107.53	106.30	-1.23
H141-C14-O14	110.23	107.94	-2.30
H142-C14-O14	110.84	108.88	-1.97
C14-O14-HO14	107.24	106.57	-0.67

Figure 174: The PES scan for flexible dihedral corresponding to hmbt.

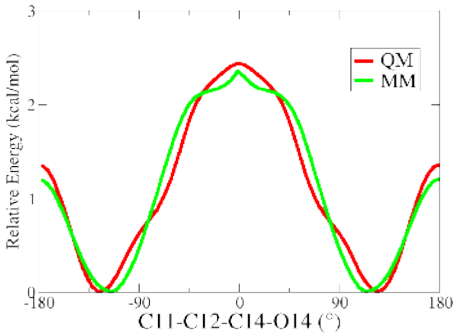

3.34 3-aminomethyl indole (idam, for DAG)

Figure 175: The energy-minimized structure of idam.

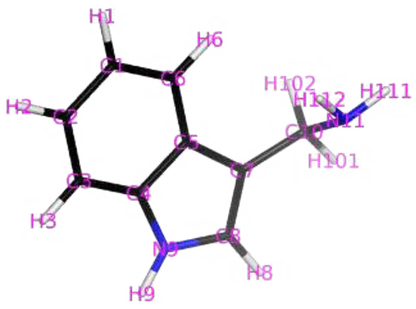

Table 268: The calculated geometric terms of idam.

Terms	QM	MM	diff
N9-C8	1.379	1.388	0.009
N9-C4	1.377	1.371	-0.006
N9-H9	1.011	1.010	-0.002
C8-H8	1.083	1.085	0.002
C8-C7	1.378	1.371	-0.007
C7-C5	1.434	1.437	0.003
C7-C10	1.493	1.507	0.014
C5-C6	1.408	1.393	-0.015
C5-C4	1.422	1.403	-0.020
C6-H6	1.089	1.079	-0.010
C6-C1	1.388	1.403	0.014
C1-H1	1.087	1.083	-0.005
C1-C2	1.412	1.401	-0.011
C2-H2	1.087	1.083	-0.004
C2-C3	1.389	1.402	0.013
C3-H3	1.088	1.079	-0.010
C3-C4	1.401	1.384	-0.017
C10-H101	1.096	1.115	0.019
C10-H102	1.103	1.113	0.010
C10-N11	1.474	1.483	0.008
N11-H111	1.021	1.015	-0.006
N11-H112	1.020	1.015	-0.004
C8-N9-C4	109.51	110.89	1.38
C8-N9-H9	125.16	126.28	1.12
C4-N9-H9	125.33	122.82	-2.51
N9-C8-H8	120.78	124.61	3.84
N9-C8-C7	109.72	107.81	-1.91
H8-C8-C7	129.51	127.56	-1.95
C8-C7-C5	106.39	107.16	0.78
C8-C7-C10	127.19	128.30	1.11
C5-C7-C10	126.27	124.54	-1.73
C7-C5-C6	133.79	132.34	-1.45
C7-C5-C4	107.46	107.85	0.39
C6-C5-C4	118.73	119.81	1.08
C5-C6-H6	120.67	119.56	-1.11
C5-C6-C1	118.92	119.56	0.63
H6-C6-C1	120.40	120.89	0.48
C6-C1-H1	119.64	120.03	0.39
C6-C1-C2	121.25	120.19	-1.06
H1-C1-C2	119.11	119.78	0.67
C1-C2-H2	119.34	119.85	0.51
C1-C2-C3	121.29	120.08	-1.22
H2-C2-C3	119.36	120.07	0.71
C2-C3-H3	121.16	120.98	-0.18
C2-C3-C4	117.22	119.35	2.13
H3-C3-C4	121.62	119.67	-1.95
N9-C4-C5	106.92	106.29	-0.63
N9-C4-C3	130.50	132.68	2.18
C5-C4-C3	122.58	121.03	-1.55
C7-C10-H101	109.56	109.46	-0.10
C7-C10-H102	108.65	109.33	0.68
C7-C10-N11	110.32	109.46	-0.86
H101-C10-H102	106.71	107.78	1.07
H101-C10-N11	107.81	109.78	1.97
H102-C10-N11	113.67	111.01	-2.67
C10-N11-H111	109.29	114.21	4.92
C10-N11-H112	108.37	113.19	4.83
H111-N11-H112	106.20	106.62	0.42

Figure 176: The PES scan for flexible dihedral corresponding to idam.

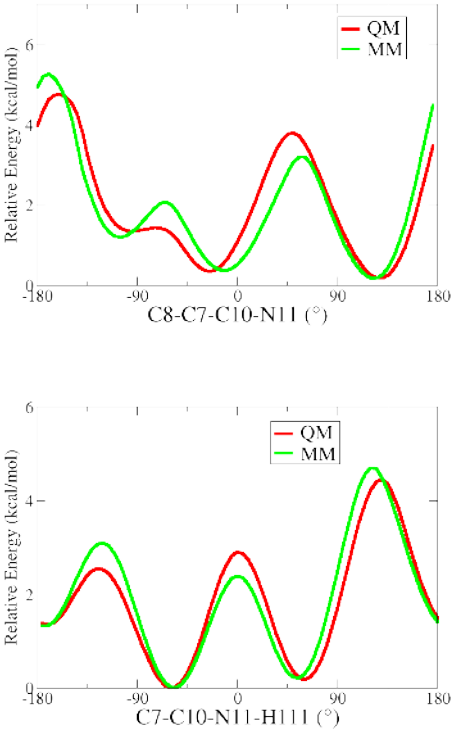

3.35 N1-(3-pyridinyl)-N2-methylurea (mpyu, for HNA, 26A, T6A, 12A & 6GA)

Figure 177: The energy-minimized structure of mpyu.

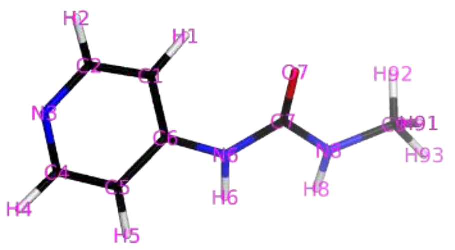

Table 269: The calculated geometric terms of mpyu.

Terms	QM	MM	diff
C1-H1	1.081	1.076	-0.005
C1-C2	1.394	1.402	0.008
C1-C6	1.401	1.402	0.001
C2-H2	1.089	1.083	-0.006
C2-N3	1.345	1.331	-0.014
N3-C4	1.345	1.330	-0.015
C4-H4	1.089	1.083	-0.005
C4-C5	1.391	1.402	0.011
C5-H5	1.089	1.076	-0.012
C5-C6	1.401	1.404	0.003
C6-N6	1.402	1.419	0.017
N6-H6	1.014	0.987	-0.027
N6-C7	1.395	1.365	-0.030
C7-O7	1.228	1.223	-0.005
C7-N8	1.383	1.363	-0.020
N8-H8	1.014	0.991	-0.022
N8-C9	1.457	1.444	-0.014
C9-H91	1.089	1.114	0.025
C9-H92	1.095	1.114	0.019
C9-H93	1.091	1.112	0.021
H1-C1-C2	120.97	119.56	-1.41
H1-C1-C6	121.01	121.89	0.88
C2-C1-C6	118.00	118.55	0.54
C1-C2-H2	119.21	120.35	1.14
C1-C2-N3	125.19	121.78	-3.40
H2-C2-N3	115.60	117.87	2.26
C2-N3-C4	115.72	120.67	4.95
N3-C4-H4	116.02	117.74	1.72
N3-C4-C5	124.07	121.68	-2.39
H4-C4-C5	119.91	120.58	0.67
C4-C5-H5	119.96	120.35	0.40
C4-C5-C6	119.22	118.64	-0.58
H5-C5-C6	120.82	121.01	0.19
C1-C6-C5	117.79	118.68	0.89
C1-C6-N6	123.99	124.68	0.69
C5-C6-N6	118.16	116.62	-1.54
C6-N6-H6	114.68	114.90	0.23
C6-N6-C7	126.26	127.69	1.44
H6-N6-C7	115.59	117.31	1.72
N6-C7-O7	124.65	126.79	2.14
N6-C7-N8	112.10	110.77	-1.33
O7-C7-N8	123.24	122.44	-0.80
C7-N8-H8	115.87	119.79	3.92
C7-N8-C9	117.33	121.37	4.04
H8-N8-C9	115.47	118.73	3.27
N8-C9-H91	108.57	110.57	2.01
N8-C9-H92	112.67	110.59	-2.07
N8-C9-H93	108.36	110.71	2.35
H91-C9-H92	108.37	108.07	-0.30
H91-C9-H93	109.23	108.39	-0.84
H92-C9-H93	109.60	108.42	-1.17

Figure 178: The PES scan for flexible dihedral corresponding to mpyu.

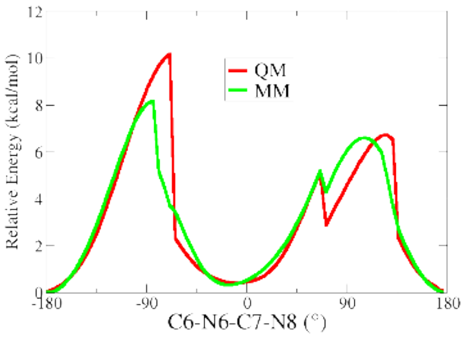

3.36 phenylacetate methylester (mebz, for OCU & MEU)

Figure 179: The energy-minimized structure of mebz.

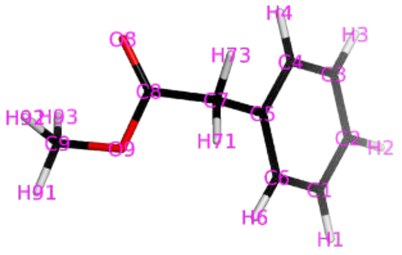

Table 270: The calculated geometric terms of mebz.

Terms	QM	MM	diff
C1-H1	1.087	1.080	-0.007
C1-C2	1.396	1.401	0.005
C1-C6	1.397	1.401	0.005
C2-H2	1.087	1.080	-0.007
C2-C3	1.397	1.401	0.004
C3-H3	1.087	1.081	-0.007
C3-C4	1.395	1.401	0.007
C4-H4	1.088	1.080	-0.008
C4-C5	1.401	1.403	0.002
C5-C6	1.399	1.403	0.005
C5-C7	1.514	1.501	-0.013
C6-H6	1.089	1.080	-0.008
C7-H71	1.093	1.109	0.016
C7-H73	1.094	1.110	0.016
C7-C8	1.509	1.514	0.004
C8-O8	1.221	1.217	-0.004
C8-O9	1.354	1.336	-0.018
O9-C9	1.443	1.438	-0.004
C9-H91	1.088	1.112	0.024
C9-H92	1.091	1.114	0.023
C9-H93	1.091	1.114	0.023
H1-C1-C2	120.10	120.03	-0.07
H1-C1-C6	119.77	120.02	0.25
C2-C1-C6	120.13	119.95	-0.19
C1-C2-H2	120.15	119.99	-0.15
C1-C2-C3	119.70	120.03	0.33
H2-C2-C3	120.16	119.98	-0.18
C2-C3-H3	120.06	120.04	-0.01
C2-C3-C4	120.21	119.95	-0.26
H3-C3-C4	119.73	120.01	0.27
C3-C4-H4	120.35	119.92	-0.43
C3-C4-C5	120.28	120.25	-0.03
H4-C4-C5	119.36	119.83	0.47
C4-C5-C6	119.29	119.57	0.28
C4-C5-C7	119.73	120.05	0.32
C6-C5-C7	120.98	120.36	-0.61
C1-C6-C5	120.38	120.25	-0.13
C1-C6-H6	119.97	119.95	-0.03
C5-C6-H6	119.64	119.80	0.16
C5-C7-H71	110.52	109.90	-0.61
C5-C7-H73	110.81	109.45	-1.36
C5-C7-C8	108.86	109.37	0.52
H71-C7-H73	109.09	109.43	0.34
H71-C7-C8	109.82	109.91	0.08
H73-C7-C8	107.70	108.75	1.05
C7-C8-O8	125.04	124.67	-0.36
C7-C8-O9	111.03	110.16	-0.87
O8-C8-O9	123.87	125.14	1.28
C8-O9-C9	113.97	112.36	-1.61
O9-C9-H91	105.05	109.83	4.79
O9-C9-H92	110.38	110.88	0.49
O9-C9-H93	110.25	110.80	0.56
H91-C9-H92	110.91	108.27	-2.65
H91-C9-H93	110.91	108.21	-2.70
H92-C9-H93	109.29	108.77	-0.52

Figure 180: The PES scan for flexible dihedral corresponding to mebz.

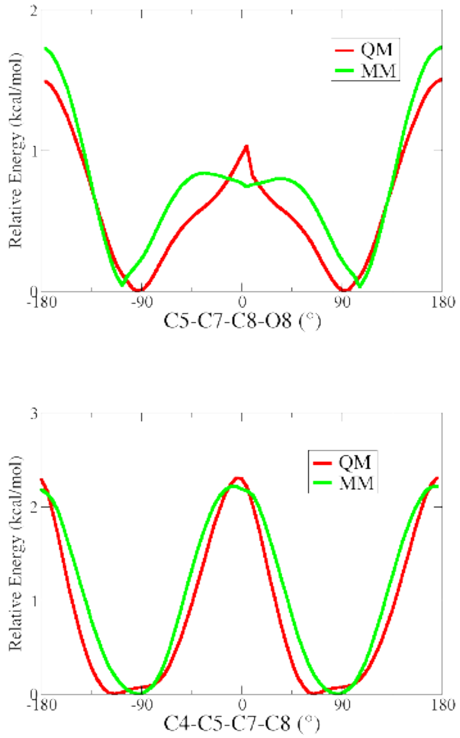

3.37 2-methylamino ethyl sulfate (maes, for 5TU & STU)

Figure 181: The energy-minimized structure of maes.

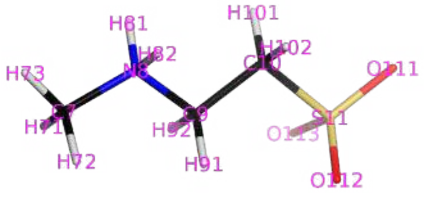

Table 271: The calculated geometric terms of maes.

Terms	QM	MM	diff
C7-H71	1.091	1.111	0.020
C7-H72	1.091	1.111	0.020
C7-H73	1.091	1.110	0.019
C7-N8	1.490	1.497	0.007
N8-H81	1.025	1.010	-0.015
N8-H82	1.025	1.010	-0.015
N8-C9	1.516	1.502	-0.014
C9-H91	1.091	1.106	0.015
C9-H92	1.094	1.106	0.012
C9-C10	1.522	1.517	-0.006
C10-H101	1.094	1.107	0.013
C10-H102	1.094	1.107	0.013
C10-S11	1.822	1.772	-0.050
S11-O111	1.469	1.441	-0.028
S11-O112	1.521	1.444	-0.077
S11-O113	1.491	1.444	-0.047
C7-H71	1.091	1.111	0.020
C7-H72	1.091	1.111	0.020
C7-H73	1.091	1.110	0.019
C7-N8	1.490	1.497	0.007
N8-H81	1.025	1.010	-0.015
N8-H82	1.025	1.010	-0.015
N8-C9	1.516	1.502	-0.014
C9-H91	1.091	1.106	0.015
C9-H92	1.094	1.106	0.012
C9-C10	1.522	1.517	-0.006
C10-H101	1.094	1.107	0.013
C10-H102	1.094	1.107	0.013
C10-S11	1.822	1.772	-0.050
S11-O111	1.469	1.441	-0.028
S11-O112	1.521	1.444	-0.077
S11-O113	1.491	1.444	-0.047
H71-C7-H72	111.07	111.51	0.44
H71-C7-H73	110.46	111.80	1.34
H71-C7-N8	109.50	107.06	-2.44
H72-C7-H73	110.24	111.80	1.56
H72-C7-N8	106.73	107.06	0.33
H73-C7-N8	108.73	107.27	-1.46
C7-N8-H81	110.48	111.68	1.20
C7-N8-H82	108.83	111.68	2.85
C7-N8-C9	113.66	114.76	1.11
H81-N8-H82	106.84	105.27	-1.57
H81-N8-C9	109.87	106.41	-3.47
H82-N8-C9	106.87	106.41	-0.46
N8-C9-H91	106.39	105.07	-1.32
N8-C9-H92	107.98	105.07	-2.91
N8-C9-C10	111.03	106.18	-4.85
H91-C9-H92	109.53	110.01	0.48
H91-C9-C10	109.20	114.74	5.55
H92-C9-C10	112.53	114.74	2.21
C9-C10-H101	108.89	114.02	5.13
C9-C10-H102	113.33	114.02	0.69
C9-C10-S11	108.90	103.41	-5.49
H101-C10-H102	109.16	110.75	1.59
H101-C10-S11	108.38	106.94	-1.44
H102-C10-S11	108.07	106.94	-1.14
C10-S11-O111	107.43	102.67	-4.76
C10-S11-O112	100.43	101.43	1.00
C10-S11-O113	102.30	101.43	-0.87
O111-S11-O112	114.89	116.15	1.26
O111-S11-O113	118.08	116.15	-1.93
O112-S11-O113	111.18	115.40	4.22
H71-C7-H72	111.07	111.51	0.44
H71-C7-H73	110.46	111.80	1.34
H71-C7-N8	109.50	107.06	-2.44
H72-C7-H73	110.24	111.80	1.56
H72-C7-N8	106.73	107.06	0.33
H73-C7-N8	108.73	107.27	-1.46
C7-N8-H81	110.48	111.68	1.20
C7-N8-H82	108.83	111.68	2.85
C7-N8-C9	113.66	114.76	1.11
H81-N8-H82	106.84	105.27	-1.57
H81-N8-C9	109.87	106.41	-3.47
H82-N8-C9	106.87	106.41	-0.46
N8-C9-H91	106.39	105.07	-1.32
N8-C9-H92	107.98	105.07	-2.91
N8-C9-C10	111.03	106.18	-4.85
H91-C9-H92	109.53	110.01	0.48
H91-C9-C10	109.20	114.74	5.55
H92-C9-C10	112.53	114.74	2.21
C9-C10-H101	108.89	114.02	5.13
C9-C10-H102	113.33	114.02	0.69
C9-C10-S11	108.90	103.41	-5.49
H101-C10-H102	109.16	110.75	1.59
H101-C10-S11	108.38	106.94	-1.44
H102-C10-S11	108.07	106.94	-1.14
C10-S11-O111	107.43	102.67	-4.76
C10-S11-O112	100.43	101.43	1.00
C10-S11-O113	102.30	101.43	-0.87
O111-S11-O112	114.89	116.15	1.26
O111-S11-O113	118.08	116.15	-1.93
O112-S11-O113	111.18	115.40	4.22

Figure 182: The PES scan for flexible dihedral corresponding to maes.

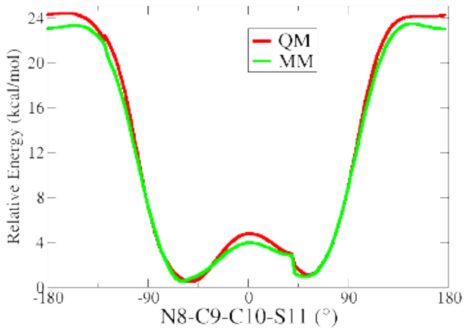

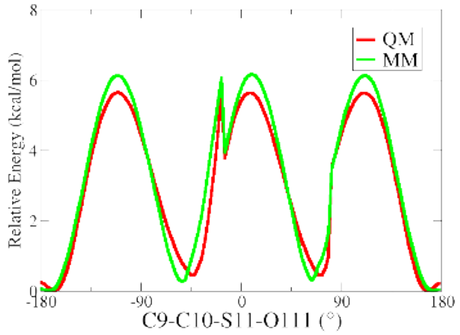

3.38 N1,N2-dimethylurea (12mu, for HNA, 26A, T6A, 12A & 6GA)

Figure 183: The energy-minimized structure of 12mu.

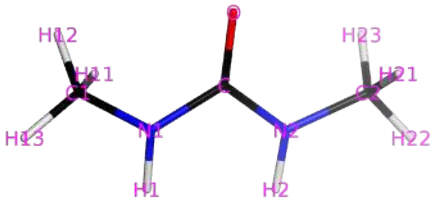

Table 272: The calculated geometric terms of 12mu.

Terms	QM	MM	diff
N1-H1	1.014	0.993	-0.021
N1-C1	1.456	1.440	-0.017
N1-C	1.388	1.362	-0.026
C1-H11	1.096	1.113	0.017
C1-H12	1.089	1.113	0.024
C1-H13	1.091	1.111	0.020
C-O	1.231	1.223	-0.007
C-N2	1.388	1.362	-0.026
N2-H2	1.456	0.993	-0.464
N2-H23	1.014	2.104	1.090
H2-C2	1.089	2.108	1.019
H2-H21	1.091	2.843	1.752
H2-H22	1.096	2.321	1.225
H1-N1-C1	115.13	119.04	3.92
H1-N1-C	114.58	120.66	6.08
C1-N1-C	117.18	120.30	3.11
N1-C1-H11	112.89	110.33	-2.56
N1-C1-H12	108.45	110.33	1.89
N1-C1-H13	108.55	110.71	2.16
H11-C1-H12	108.20	108.13	-0.08
H11-C1-H13	109.46	108.64	-0.82
H12-C1-H13	109.24	108.64	-0.60
N1-C-O	123.33	123.68	0.35
N1-C-N2	113.34	112.64	-0.69
O-C-N2	123.33	123.68	0.35
C-N2-H2	117.18	120.66	3.48
C-N2-H23	114.58	102.82	-11.76
H2-N2-H23	115.13	129.81	14.69
N2-H2-C2	108.45	36.65	-71.79
N2-H2-H21	108.55	34.63	-73.92
N2-H2-H22	112.89	65.20	-47.69
C2-H2-H21	109.24	19.66	-89.57
C2-H2-H22	108.20	28.55	-79.65
H21-H2-H22	109.46	39.35	-70.11

Figure 184: The PES scan for flexible dihedral corresponding to 12mu.

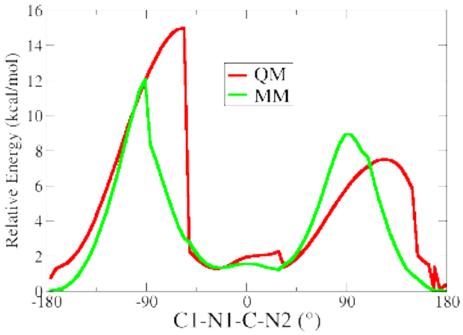

3.39 methoxy acetate (moat, for OAU)

Figure 185: The energy-minimized structure of moat.

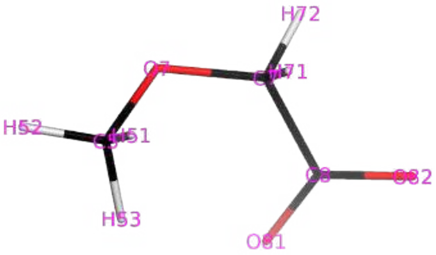

Table 273: The calculated geometric terms of moat.

Terms	QM	MM	diff
C5-H51	1.104	1.110	0.006
C5-H52	1.095	1.110	0.015
C5-H53	1.093	1.112	0.019
C5-O7	1.420	1.422	0.002
O7-C7	1.434	1.443	0.009
C7-H71	1.103	1.109	0.006
C7-H72	1.094	1.109	0.015
C7-C8	1.557	1.547	-0.010
C8-O81	1.265	1.257	-0.008
C8-O82	1.270	1.263	-0.007
H51-C5-H52	108.87	109.35	0.47
H51-C5-H53	109.79	109.08	-0.71
H51-C5-O7	110.54	110.47	-0.07
H52-C5-H53	110.34	108.44	-1.91
H52-C5-O7	106.54	109.34	2.80
H53-C5-O7	110.69	110.14	-0.55
C5-O7-C7	111.72	111.72	0.01
O7-C7-H71	109.38	109.03	-0.34
O7-C7-H72	105.20	107.79	2.59
O7-C7-C8	117.83	119.48	1.65
H71-C7-H72	107.84	107.71	-0.13
H71-C7-C8	108.09	106.63	-1.46
H72-C7-C8	108.09	105.66	-2.43
C7-C8-O81	118.25	119.72	1.47
C7-C8-O82	111.97	113.25	1.27
O81-C8-O82	129.77	127.03	-2.75

Figure 186: The PES scan for flexible dihedral corresponding to moat.

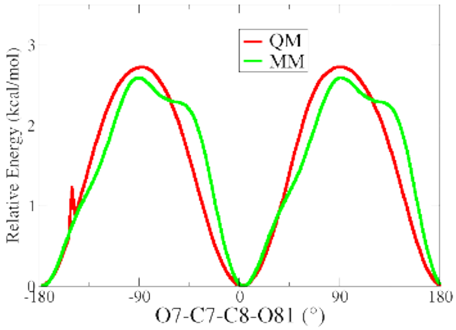

3.40 methoxyacetate methyl ester (moae, for OEU)

Terms	QM	MM	diff
O9-C10-H103	110.07	1.00	-109.08
H101-C10-H102	111.07	1.00	-110.07
H101-C10-H103	109.63	1.11	-108.52
H102-C10-H103	111.04	1.00	-110.04

Figure 187: The energy-minimized structure of moae.

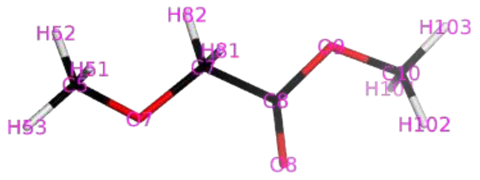

Figure 188: The PES scan for flexible dihedral corresponding to moae.

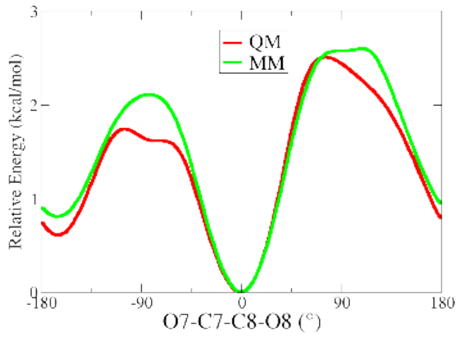

Table 274: The calculated geometric terms of moae.

Terms	QM	MM	diff
C5-H51	1.090	1.000	-0.090
C5-H52	1.094	1.000	-0.094
C5-H53	1.099	1.111	0.012
C5-O7	1.430	1.000	-0.430
O7-C7	1.406	1.000	-0.406
C7-H81	1.102	1.111	0.009
C7-H82	1.094	1.000	-0.094
C7-C8	1.522	1.000	-0.522
C8-O8	1.220	1.111	-0.109
C8-O9	1.357	1.000	-0.357
O9-C10	1.447	1.000	-0.447
C10-H101	1.091	1.415	0.324
C10-H102	1.088	1.000	-0.088
C10-H103	1.091	1.000	-0.091
H51-C5-H52	109.67	1.42	-108.26
H51-C5-H53	109.43	1.00	-108.43
H51-C5-O7	105.97	1.00	-104.97
H52-C5-H53	110.06	1.11	-108.95
H52-C5-O7	111.27	1.00	-110.27
H53-C5-O7	110.34	1.00	-109.34
C5-O7-C7	113.03	1.11	-111.92
O7-C7-H81	111.85	1.00	-110.85
O7-C7-H82	107.02	1.00	-106.03
O7-C7-C8	113.42	1.52	-111.90
H81-C7-H82	107.70	1.00	-106.70
H81-C7-C8	108.39	1.00	-107.39
H82-C7-C8	108.24	1.22	-107.02
C7-C8-O8	126.41	1.00	-125.41
C7-C8-O9	109.30	1.00	-108.30
O8-C8-O9	124.29	1.33	-122.95
C8-O9-C10	114.74	1.00	-113.74
O9-C10-H101	110.05	1.00	-109.05
O9-C10-H102	104.89	1.43	-103.46

3.41 N1-(4-pyrimidinyl)-N2-methylurea (pymu, for HNA, 26A, T6A, 12A & 6GA)

Figure 189: The energy-minimized structure of pymu.

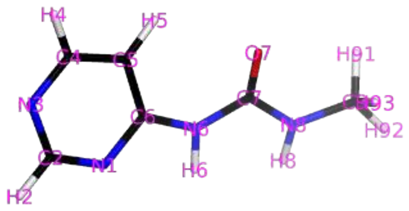

Table 275: The calculated geometric terms of pymu.

Terms	QM	MM	diff
N1-C2	1.340	1.361	0.021
N1-C6	1.340	1.361	0.021
C2-H2	1.088	1.093	0.005
C2-N3	1.341	1.368	0.027
N3-C4	1.346	1.334	-0.012
C4-H4	1.089	1.086	-0.002
C4-C5	1.388	1.408	0.020
C5-H5	1.087	1.074	-0.013
C5-C6	1.400	1.395	-0.006
C6-N6	1.396	1.396	0.001
N6-H6	1.015	0.986	-0.029
N6-C7	1.418	1.355	-0.063
C7-O7	1.221	1.222	0.001
C7-N8	1.380	1.362	-0.018
N8-H8	1.014	0.992	-0.021
N8-C9	1.457	1.444	-0.013
C9-H91	1.089	1.114	0.025
C9-H92	1.095	1.112	0.017
C9-H93	1.091	1.114	0.023
C2-N1-C6	115.82	116.65	0.83
N1-C2-H2	115.83	117.58	1.76
N1-C2-N3	127.77	125.09	-2.68
H2-C2-N3	116.40	117.27	0.87
C2-N3-C4	114.95	116.12	1.17
N3-C4-H4	116.34	116.92	0.58
N3-C4-C5	122.80	123.69	0.89
H4-C4-C5	120.86	119.38	-1.47
C4-C5-H5	121.30	121.38	0.08
C4-C5-C6	116.75	115.89	-0.86
H5-C5-C6	121.95	122.68	0.73
N1-C6-C5	121.88	122.11	0.23
N1-C6-N6	117.13	112.12	-5.01
C5-C6-N6	120.90	124.96	4.06
C6-N6-H6	115.49	109.83	-5.65
C6-N6-C7	122.15	126.32	4.16
H6-N6-C7	114.49	121.11	6.62
N6-C7-O7	124.75	125.73	0.97
N6-C7-N8	110.69	111.06	0.37
O7-C7-N8	124.49	123.18	-1.30
C7-N8-H8	115.10	119.55	4.45
C7-N8-C9	117.59	121.58	3.99
H8-N8-C9	116.14	118.87	2.73
N8-C9-H91	108.56	110.62	2.06
N8-C9-H92	112.51	110.65	-1.86
N8-C9-H93	108.52	110.61	2.09
H91-C9-H92	108.31	108.38	0.06
H91-C9-H93	109.26	108.12	-1.13
H92-C9-H93	109.63	108.36	-1.26

Figure 190: The PES scan for flexible dihedral corresponding to pymu.

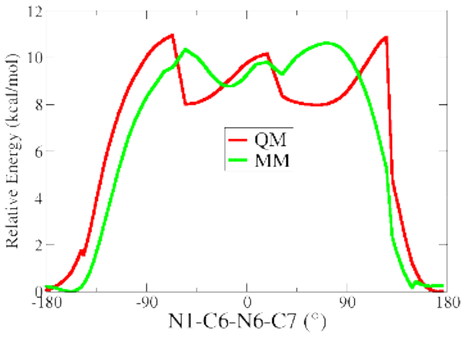

3.42 2-methylamino-4-imino-pyrimidine (ncy, for K2C & R2C)

Figure 191: The energy-minimized structure of nmcy.

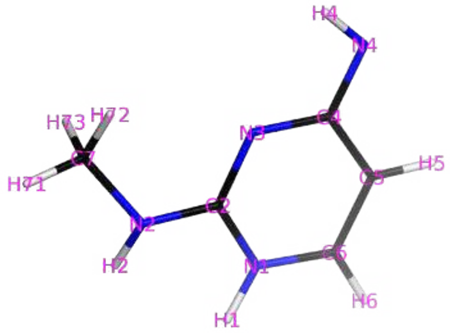

Table 276: The calculated geometric terms of nmcy.

Terms	QM	MM	diff
N1-C2	1.386	1.389	0.003
N1-C6	1.393	1.395	0.002
N1-H1	1.013	1.002	-0.012
C2-N2	1.383	1.355	-0.028
C2-N3	1.298	1.335	0.037
N2-H2	1.016	1.010	-0.005
N2-C7	1.460	1.485	0.025
N3-C4	1.414	1.383	-0.032
C4-N4	1.292	1.325	0.032
C4-C5	1.461	1.426	-0.035
N4-H4	1.028	0.997	-0.030
C5-H5	1.084	1.076	-0.008
C5-C6	1.348	1.391	0.043
C6-H6	1.085	1.085	-0.000
C7-H71	1.091	1.112	0.021
C7-H72	1.089	1.114	0.025
C7-H73	1.095	1.113	0.017
C2-N1-C6	118.13	120.30	2.17
C2-N1-H1	117.53	114.63	-2.90
C6-N1-H1	118.62	124.72	6.10
N1-C2-N2	114.03	116.14	2.11
N1-C2-N3	124.97	122.18	-2.78
N2-C2-N3	121.00	121.66	0.66
C2-N2-H2	113.20	110.26	-2.94
C2-N2-C7	117.19	120.62	3.43
H2-N2-C7	113.81	109.87	-3.94
C2-N3-C4	118.88	119.01	0.13
N3-C4-N4	122.60	120.12	-2.48
N3-C4-C5	117.71	121.11	3.40
N4-C4-C5	119.70	118.77	-0.92
C4-N4-H4	107.63	105.78	-1.85
C4-C5-H5	118.58	119.21	0.62
C4-C5-C6	119.99	118.53	-1.46
H5-C5-C6	121.42	122.26	0.84
N1-C6-C5	120.08	118.86	-1.22
N1-C6-H6	115.71	119.40	3.68
C5-C6-H6	124.16	121.74	-2.41
N2-C7-H71	107.87	110.73	2.87
N2-C7-H72	108.76	111.15	2.38
N2-C7-H73	112.72	111.38	-1.34
H71-C7-H72	109.38	107.30	-2.09
H71-C7-H73	109.95	108.28	-1.68
H72-C7-H73	108.11	107.85	-0.27

3.43 carboxymethyl methyl ammonium (nmgi, for 5DU, SCU & MAU)

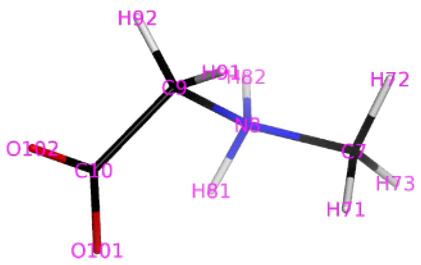

Figure 192: The energy-minimized structure of nmgi.

Table 277: The calculated geometric terms of nmgi.

Terms	QM	MM	diff
C7-H71	1.090	1.112	0.021
C7-H72	1.090	1.110	0.020
C7-H73	1.091	1.110	0.019
C7-N8	1.486	1.497	0.011
N8-H81	1.030	1.025	-0.005
N8-H82	1.023	1.005	-0.018
N8-C9	1.503	1.530	0.027
C9-H91	1.091	1.095	0.005
C9-H92	1.092	1.096	0.004
C9-C10	1.571	1.534	-0.037
C10-O101	1.280	1.261	-0.019
C10-O102	1.241	1.251	0.010
C7-H71	1.090	1.112	0.021
C7-H72	1.090	1.110	0.020
C7-H73	1.091	1.110	0.019
C7-N8	1.486	1.497	0.011
N8-H81	1.030	1.025	-0.005
N8-H82	1.023	1.005	-0.018
N8-C9	1.503	1.530	0.027
C9-H91	1.091	1.095	0.005
C9-H92	1.092	1.096	0.004
C9-C10	1.571	1.534	-0.037
C10-O101	1.280	1.261	-0.019
C10-O102	1.241	1.251	0.010
H71-C7-H72	110.47	111.85	1.38
H71-C7-H73	109.77	111.60	1.84
H71-C7-N8	107.24	106.62	-0.62
H72-C7-H73	110.53	112.13	1.60
H72-C7-N8	109.56	107.15	-2.41
H73-C7-N8	109.21	107.11	-2.10
C7-N8-H81	108.75	109.08	0.33
C7-N8-H82	111.05	113.44	2.39
C7-N8-C9	114.28	115.62	1.34
H81-N8-H82	111.17	105.71	-5.47
H81-N8-C9	98.08	101.44	3.35
H82-N8-C9	112.76	110.39	-2.37
N8-C9-H91	110.18	106.97	-3.21
N8-C9-H92	109.31	106.54	-2.77
N8-C9-C10	105.13	109.83	4.71
H91-C9-H92	109.73	112.35	2.62
H91-C9-C10	112.19	110.71	-1.48
H92-C9-C10	110.19	110.28	0.09
C9-C10-O101	112.10	116.52	4.41
C9-C10-O102	115.37	114.75	-0.62
O101-C10-O102	132.53	128.74	-3.79
H71-C7-H72	110.47	111.85	1.38
H71-C7-H73	109.77	111.60	1.84
H71-C7-N8	107.24	106.62	-0.62
H72-C7-H73	110.53	112.13	1.60
H72-C7-N8	109.56	107.15	-2.41
H73-C7-N8	109.21	107.11	-2.10
C7-N8-H81	108.75	109.08	0.33
C7-N8-H82	111.05	113.44	2.39
C7-N8-C9	114.28	115.62	1.34
H81-N8-H82	111.17	105.71	-5.47
H81-N8-C9	98.08	101.44	3.35
H82-N8-C9	112.76	110.39	-2.37
N8-C9-H91	110.18	106.97	-3.21
N8-C9-H92	109.31	106.54	-2.77
N8-C9-C10	105.13	109.83	4.71
H91-C9-H92	109.73	112.35	2.62
H91-C9-C10	112.19	110.71	-1.48
H92-C9-C10	110.19	110.28	0.09
C9-C10-O101	112.10	116.52	4.41
C9-C10-O102	115.37	114.75	-0.62
O101-C10-O102	132.53	128.74	-3.79

Figure 193: The PES scan for flexible dihedral corresponding to nmgi.

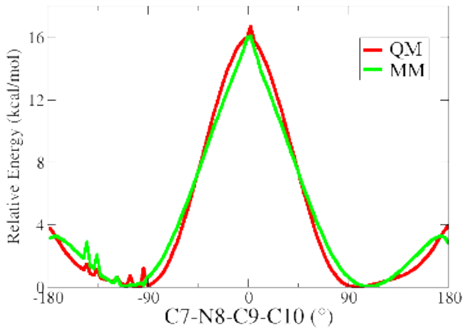

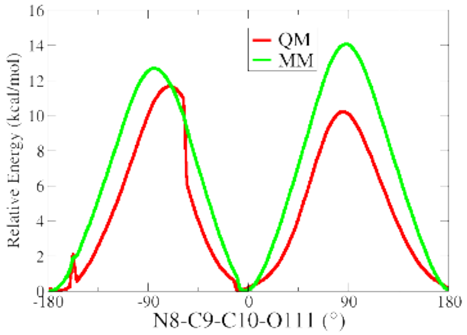

3.44 methylamino acetic acid (nmgn, for 5DU, SCU & MAU)

Figure 194: The energy-minimized structure of nmgn.

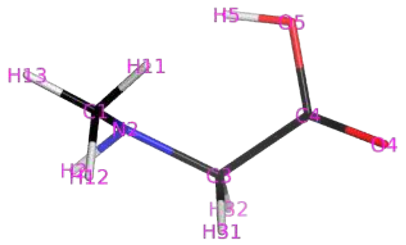

Table 278: The calculated geometric terms of nmgn.

Terms	QM	MM	diff
C1-H11	1.093	1.114	0.021
C1-H12	1.099	1.113	0.015
C1-H13	1.092	1.114	0.022
C1-N2	1.466	1.468	0.002
N2-H2	1.018	1.028	0.010
N2-C3	1.462	1.508	0.046
C3-H31	1.098	1.109	0.010
C3-H32	1.096	1.112	0.017
C3-C4	1.531	1.560	0.029
C4-O4	1.215	1.214	-0.001
C4-O5	1.349	1.376	0.027
O5-H5	0.992	0.961	-0.031
H11-C1-H12	108.35	107.77	-0.58
H11-C1-H13	108.17	107.38	-0.79
H11-C1-N2	108.84	111.30	2.46
H12-C1-H13	109.08	107.74	-1.34
H12-C1-N2	113.10	111.56	-1.54
H13-C1-N2	109.19	110.90	1.71
C1-N2-H2	109.84	104.96	-4.88
C1-N2-C3	112.39	110.64	-1.75
H2-N2-C3	110.24	112.64	2.40
N2-C3-H31	114.41	109.84	-4.57
N2-C3-H32	109.30	108.23	-1.06
N2-C3-C4	110.05	119.40	9.35
H31-C3-H32	107.70	107.20	-0.51
H31-C3-C4	108.04	106.66	-1.37
H32-C3-C4	107.07	104.84	-2.22
C3-C4-O4	123.31	125.45	2.14
C3-C4-O5	113.20	112.28	-0.92
O4-C4-O5	123.46	122.26	-1.20
C4-O5-H5	104.35	107.58	3.22

Figure 195: The PES scan for flexible dihedral corresponding to nmgn.

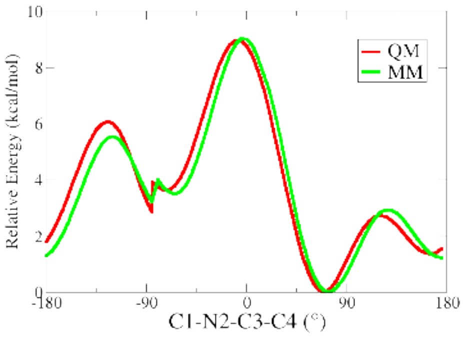

3.45 phenoxyacetate (atbz, for OAU)

Figure 196: The energy-minimized structure of atbz.

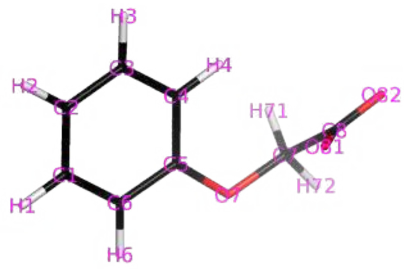

Table 279: The calculated geometric terms of atbz.

Terms	QM	MM	diff
C1-H1	1.090	1.080	-0.010
C1-C2	1.401	1.400	-0.001
C1-C6	1.395	1.401	0.007
C2-H2	1.089	1.080	-0.009
C2-C3	1.398	1.400	0.002
C3-H3	1.089	1.080	-0.009
C3-C4	1.400	1.403	0.003
C4-H4	1.085	1.078	-0.007
C4-C5	1.405	1.411	0.006
C5-C6	1.407	1.402	-0.005
C5-O7	1.359	1.413	0.055
C6-H6	1.088	1.079	-0.009
O7-C7	1.444	1.448	0.004
C7-H71	1.098	1.109	0.011
C7-H72	1.093	1.110	0.017
C7-C8	1.555	1.547	-0.008
C8-O81	1.260	1.256	-0.004
C8-O82	1.270	1.262	-0.008
H1-C1-C2	120.18	120.26	0.08
H1-C1-C6	119.42	120.00	0.58
C2-C1-C6	120.39	119.73	-0.66
C1-C2-H2	120.57	120.10	-0.47
C1-C2-C3	118.87	119.97	1.11
H2-C2-C3	120.54	119.92	-0.62
C2-C3-H3	119.91	120.36	0.44
C2-C3-C4	121.42	120.08	-1.33
H3-C3-C4	118.67	119.56	0.89
C3-C4-H4	119.60	118.47	-1.13
C3-C4-C5	119.38	120.41	1.02
H4-C4-C5	120.92	121.09	0.18
C4-C5-C6	119.37	118.73	-0.65
C4-C5-O7	124.98	123.42	-1.56
C6-C5-O7	115.60	117.83	2.22
C1-C6-C5	120.55	121.07	0.52
C1-C6-H6	121.32	120.56	-0.76
C5-C6-H6	118.11	118.37	0.26
C5-O7-C7	116.97	118.51	1.54
O7-C7-H71	109.38	110.25	0.87
O7-C7-H72	103.86	107.04	3.18
O7-C7-C8	116.10	119.56	3.46
H71-C7-H72	108.37	106.86	-1.52
H71-C7-C8	109.94	107.32	-2.62
H72-C7-C8	108.82	105.06	-3.75
C7-C8-O81	117.31	119.17	1.86
C7-C8-O82	111.82	113.52	1.70
O81-C8-O82	130.86	127.31	-3.55

Figure 197: The PES scan for flexible dihedral corresponding to atbz.

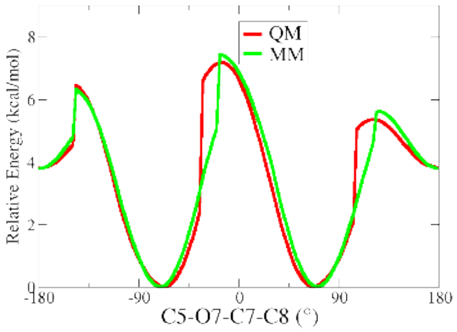

3.46 methoxyacetic acid (moac, for OAU)

Figure 198: The energy-minimized structure of moac.

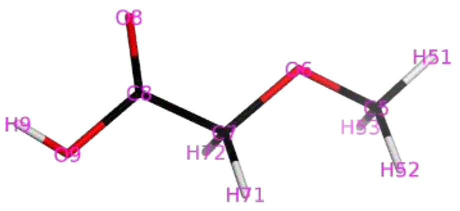

Table 280: The calculated geometric terms of moac.

Terms	QM	MM	diff
C5-H51	1.090	1.112	0.022
C5-H52	1.099	1.112	0.013
C5-H53	1.099	1.112	0.013
C5-O6	1.420	1.420	-0.000
O6-C7	1.404	1.428	0.025
C7-H71	1.102	1.110	0.008
C7-H72	1.102	1.110	0.008
C7-C8	1.512	1.535	0.024
C8-O8	1.213	1.218	0.005
C8-O9	1.364	1.384	0.021
O9-H9	0.979	0.957	-0.022
H51-C5-H52	109.41	108.62	-0.79
H51-C5-H53	109.41	108.62	-0.79
H51-C5-O6	106.40	109.13	2.73
H52-C5-H53	108.77	109.52	0.76
H52-C5-O6	111.40	110.45	-0.95
H53-C5-O6	111.40	110.45	-0.95
C5-O6-C7	110.96	111.34	0.38
O6-C7-H71	112.17	109.46	-2.71
O6-C7-H72	112.17	109.46	-2.71
O6-C7-C8	108.77	112.70	3.94
H71-C7-H72	106.89	109.24	2.35
H71-C7-C8	108.36	107.95	-0.40
H72-C7-C8	108.36	107.95	-0.40
C7-C8-O8	127.02	128.32	1.30
C7-C8-O9	109.18	112.73	3.56
O8-C8-O9	123.80	118.94	-4.86
C8-O9-H9	105.57	107.07	1.50

Figure 199: The PES scan for flexible dihedral corresponding to moac.

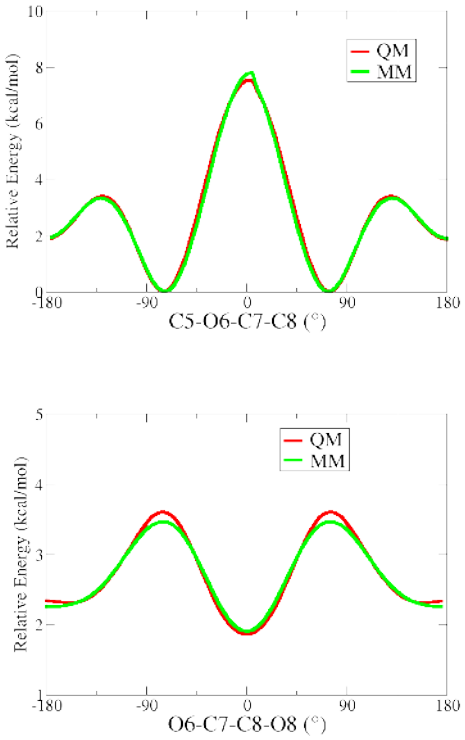

3.47 phenoxyacetate methylester (oebz, for OEU)

Figure 200: The energy-minimized structure of oebz.

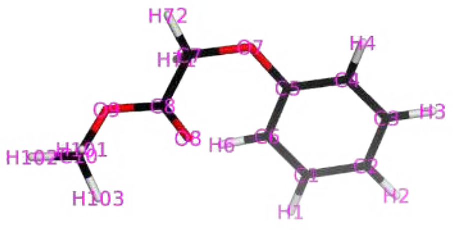

Table 281: The calculated geometric terms of oebz.

Terms	QM	MM	diff
C1-H1	1.088	1.081	-0.007
C1-C2	1.395	1.400	0.005
C1-C6	1.402	1.403	0.002
C2-H2	1.087	1.081	-0.007
C2-C3	1.401	1.400	-0.001
C3-H3	1.088	1.081	-0.007
C3-C4	1.394	1.401	0.007
C4-H4	1.087	1.080	-0.008
C4-C5	1.400	1.401	0.000
C5-C6	1.400	1.412	0.012
C5-O7	1.382	1.413	0.031
C6-H6	1.086	1.076	-0.010
O7-C7	1.414	1.440	0.026
C7-H71	1.097	1.109	0.013
C7-H72	1.094	1.110	0.016
C7-C8	1.522	1.544	0.022
C8-O8	1.218	1.218	0.000
C8-O9	1.356	1.342	-0.014
O9-C10	1.449	1.439	-0.010
C10-H101	1.091	1.114	0.023
C10-H102	1.088	1.112	0.024
C10-H103	1.091	1.114	0.023
H1-C1-C2	120.12	119.90	-0.22
H1-C1-C6	118.97	120.00	1.03
C2-C1-C6	120.91	120.10	-0.81
C1-C2-H2	120.29	120.01	-0.28
C1-C2-C3	119.34	120.02	0.69
H2-C2-C3	120.37	119.96	-0.41
C2-C3-H3	120.17	120.16	-0.01
C2-C3-C4	120.43	119.77	-0.66
H3-C3-C4	119.40	120.07	0.67
C3-C4-H4	121.58	120.21	-1.36
C3-C4-C5	119.79	120.96	1.17
H4-C4-C5	118.64	118.83	0.19
C4-C5-C6	120.45	118.92	-1.54
C4-C5-O7	114.94	117.91	2.97
C6-C5-O7	124.61	123.17	-1.43
C1-C6-C5	119.08	120.23	1.15
C1-C6-H6	118.92	117.93	-0.99
C5-C6-H6	122.00	121.84	-0.16
C5-O7-C7	116.65	118.25	1.60
O7-C7-H71	112.31	111.10	-1.21
O7-C7-H72	105.49	107.26	1.77
O7-C7-C8	111.83	115.79	3.96
H71-C7-H72	108.17	107.16	-1.00
H71-C7-C8	110.22	108.96	-1.26
H72-C7-C8	108.59	106.11	-2.48
C7-C8-O8	125.52	127.27	1.75
C7-C8-O9	109.64	108.40	-1.24
O8-C8-O9	124.82	124.33	-0.49
C8-O9-C10	114.47	112.19	-2.28
O9-C10-H101	110.02	110.86	0.84
O9-C10-H102	104.87	109.89	5.01
O9-C10-H103	109.89	110.90	1.01
H101-C10-H102	111.13	108.19	-2.93
H101-C10-H103	109.69	108.68	-1.00
H102-C10-H103	111.15	108.23	-2.92

Figure 201: The PES scan for flexible dihedral corresponding to oebz.

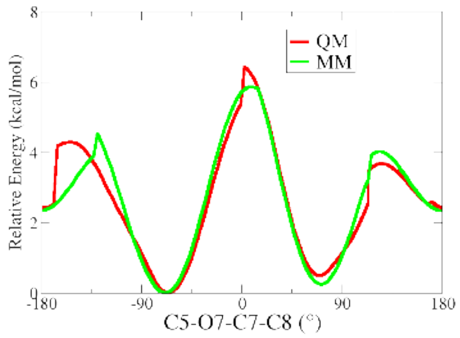

3.48 1-(3-pyrrolyl)prop-2-yl hydrogen peroxide (ppox, for PBG)

Figure 202: The energy-minimized structure of ppox.

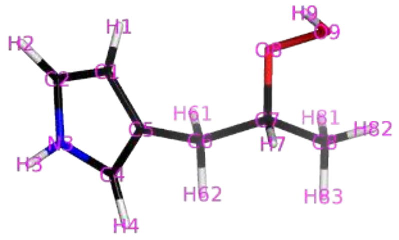

Table 282: The calculated geometric terms of ppox.

Terms	QM	MM	diff
C1-H1	1.081	1.081	-0.001
C1-C2	1.382	1.357	-0.025
C1-C5	1.423	1.363	-0.061
C2-H2	1.081	1.084	0.003
C2-N3	1.373	1.374	0.001
N3-H3	1.011	1.011	0.001
N3-C4	1.372	1.379	0.006
C4-H4	1.082	1.082	-0.000
C4-C5	1.387	1.362	-0.025
C5-C6	1.498	1.512	0.014
C6-H61	1.098	1.112	0.014
C6-H62	1.097	1.111	0.013
C6-C7	1.528	1.548	0.020
C7-H7	1.099	1.116	0.017
C7-C8	1.518	1.540	0.022
C7-O8	1.437	1.427	-0.010
C8-H81	1.094	1.110	0.016
C8-H82	1.092	1.110	0.018
C8-H83	1.095	1.109	0.014
O8-O9	1.470	1.470	-0.000
O9-H9	0.978	0.962	-0.016
H1-C1-C2	126.07	125.50	-0.57
H1-C1-C5	126.09	124.60	-1.49
C2-C1-C5	107.84	109.89	2.06
C1-C2-H2	131.33	129.33	-2.00
C1-C2-N3	107.48	106.18	-1.30
H2-C2-N3	121.19	124.49	3.30
C2-N3-H3	125.04	125.15	0.11
C2-N3-C4	109.93	109.02	-0.91
H3-N3-C4	125.03	125.83	0.81
N3-C4-H4	121.37	124.57	3.20
N3-C4-C5	107.92	107.07	-0.85
H4-C4-C5	130.71	128.36	-2.35

Terms	QM	MM	diff
C1-C5-C4	106.83	107.83	1.00
C1-C5-C6	127.51	125.71	-1.81
C4-C5-C6	125.65	126.46	0.82
C5-C6-H61	111.15	108.91	-2.24
C5-C6-H62	109.68	108.85	-0.83
C5-C6-C7	113.53	115.24	1.72
H61-C6-H62	106.56	106.99	0.43
H61-C6-C7	107.82	108.15	0.33
H62-C6-C7	107.82	108.40	0.58
C6-C7-H7	109.43	108.28	-1.15
C6-C7-C8	113.43	110.93	-2.50
C6-C7-O8	103.78	109.09	5.31
H7-C7-C8	110.82	108.68	-2.14
H7-C7-O8	108.42	109.82	1.41
C8-C7-O8	110.65	110.00	-0.64
C7-C8-H81	109.76	109.71	-0.05
C7-C8-H82	110.14	110.52	0.38
C7-C8-H83	110.59	110.53	-0.05
H81-C8-H82	108.73	108.36	-0.38
H81-C8-H83	108.88	108.96	0.08
H82-C8-H83	108.70	108.72	0.02
C7-O8-O9	106.67	108.37	1.70
O8-O9-H9	98.27	98.59	0.32

Figure 203: The PES scan for flexible dihedral corresponding to ppox.

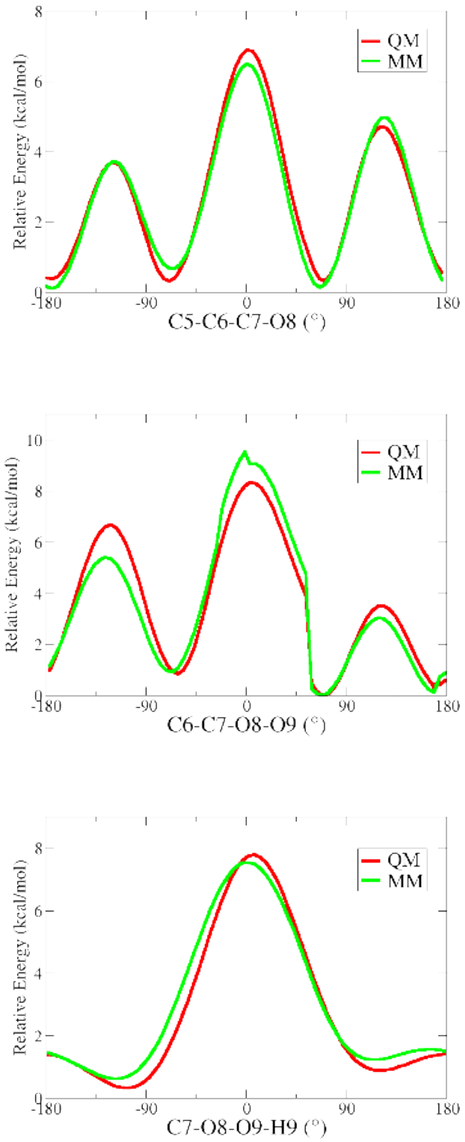

3.49 2-(propene-3-yl)thiouracil (pesu, for GAU, GCU)

Figure 204: The energy-minimized structure of pesu.

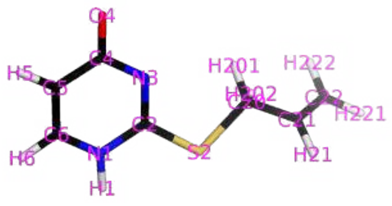

Table 283: The calculated geometric terms of pesu.

Terms	QM	MM	diff
N1-C2	1.374	1.394	0.020
N1-C6	1.378	1.359	-0.019
N1-H1	1.014	1.008	-0.006
C2-S2	1.772	1.769	-0.003
C2-N3	1.296	1.349	0.053
S2-C20	1.825	1.828	0.003
N3-C4	1.421	1.363	-0.058
C4-O4	1.229	1.229	-0.000
C4-C5	1.464	1.439	-0.025
C5-H5	1.084	1.088	0.004
C5-C6	1.353	1.366	0.013
C6-H6	1.085	1.092	0.007
C20-H201	1.092	1.113	0.021
C20-H202	1.093	1.112	0.020
C20-C21	1.496	1.507	0.011
C21-H21	1.089	1.103	0.015
C21-C22	1.339	1.347	0.008
C22-H221	1.085	1.102	0.017
C22-H222	1.086	1.102	0.015
C2-N1-C6	119.27	118.61	-0.66
C2-N1-H1	120.23	117.74	-2.50
C6-N1-H1	120.49	123.65	3.16
N1-C2-S2	113.74	115.56	1.82
N1-C2-N3	124.33	121.66	-2.67
S2-C2-N3	121.94	122.78	0.84
C2-S2-C20	99.82	100.89	1.07
C2-N3-C4	119.92	120.45	0.53
N3-C4-O4	120.53	120.58	0.04
N3-C4-C5	116.25	119.00	2.75
O4-C4-C5	123.22	120.42	-2.80
C4-C5-H5	118.53	120.92	2.38
C4-C5-C6	120.48	118.53	-1.94
H5-C5-C6	120.99	120.55	-0.44
N1-C6-C5	119.75	121.74	1.99
N1-C6-H6	116.05	117.58	1.53
C5-C6-H6	124.20	120.68	-3.52
S2-C20-H201	109.20	110.34	1.14
S2-C20-H202	106.71	110.44	3.73
S2-C20-C21	108.39	108.20	-0.19
H201-C20-H202	108.24	106.84	-1.40
H201-C20-C21	111.58	111.17	-0.41
H202-C20-C21	112.56	109.85	-2.71
C20-C21-H21	116.45	115.04	-1.42
C20-C21-C22	123.37	126.76	3.38
H21-C21-C22	120.17	118.21	-1.96
C21-C22-H221	121.52	121.18	-0.34
C21-C22-H222	121.55	120.89	-0.66
H221-C22-H222	116.93	117.93	1.00

Figure 205: The PES scan for flexible dihedral corresponding to pesu.

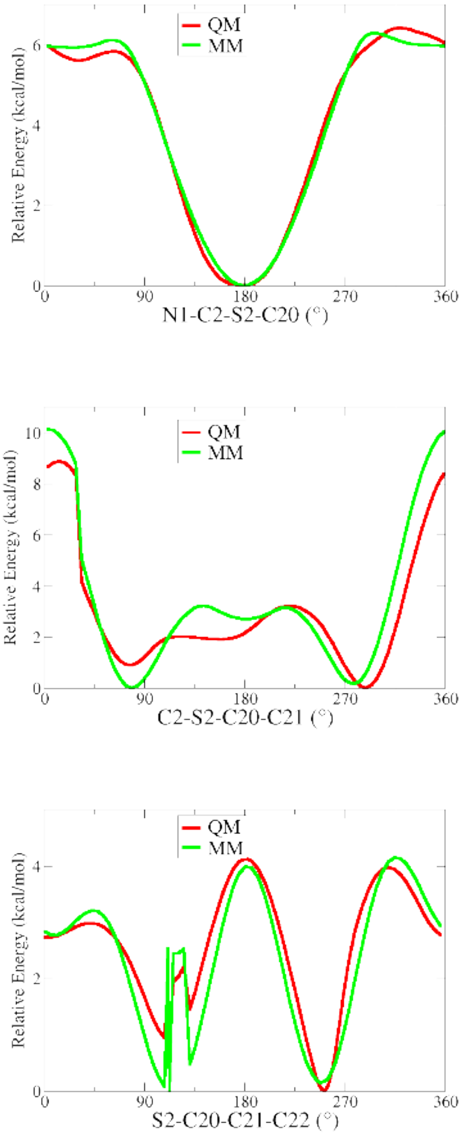

3.50 3-(2-hydroxylpropyl)indolizine (hpiz, for HWG)

Figure 206: The energy-minimized structure of hpiz.

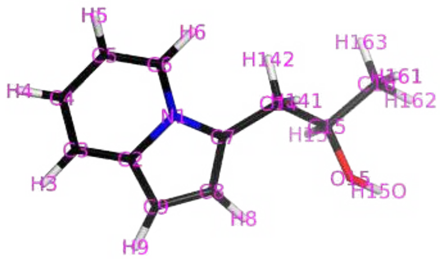

Table 284: The calculated geometric terms of hpiz.

Terms	QM	MM	diff
C5-H5	1.086	1.080	-0.006
C5-C6	1.370	1.401	0.031
C5-C4	1.420	1.401	-0.019
C6-H6	1.084	1.079	-0.005
C6-N1	1.379	1.402	0.022
N1-C7	1.376	1.373	-0.003
N1-C2	1.421	1.425	0.004
C7-C8	1.398	1.372	-0.026
C7-C14	1.494	1.513	0.019
C8-H8	1.082	1.079	-0.003
C8-C9	1.401	1.370	-0.031
C9-H9	1.083	1.079	-0.003
C9-C2	1.399	1.422	0.023
C2-C3	1.408	1.387	-0.021
C3-H3	1.088	1.079	-0.009
C3-C4	1.379	1.403	0.024
C4-H4	1.086	1.080	-0.006
C14-H141	1.101	1.111	0.010
C14-H142	1.100	1.110	0.010
C14-C15	1.522	1.551	0.029
C15-H15	1.101	1.116	0.015
C15-O15	1.433	1.418	-0.015
C15-C16	1.522	1.531	0.009
O15-H15O	0.974	0.960	-0.014
C16-H161	1.093	1.112	0.018
C16-H162	1.095	1.110	0.015
C16-H163	1.094	1.110	0.016
H5-C5-C6	118.47	119.87	1.39
H5-C5-C4	120.76	119.98	-0.78
C6-C5-C4	120.77	120.16	-0.61
C5-C6-H6	123.61	120.51	-3.09
C5-C6-N1	119.83	120.88	1.05
H6-C6-N1	116.56	118.61	2.05
C6-N1-C7	129.12	133.82	4.70
C6-N1-C2	121.15	118.29	-2.87
C7-N1-C2	109.72	107.90	-1.83
N1-C7-C8	106.82	108.15	1.33
N1-C7-C14	121.11	123.59	2.48
C8-C7-C14	132.04	128.26	-3.78
C7-C8-H8	123.79	123.84	0.05
C7-C8-C9	109.30	110.66	1.36
H8-C8-C9	126.88	125.46	-1.42
C8-C9-H9	127.19	126.97	-0.22
C8-C9-C2	107.67	106.59	-1.07
H9-C9-C2	125.15	126.43	1.28
N1-C2-C9	106.49	106.69	0.20
N1-C2-C3	118.10	120.71	2.61
C9-C2-C3	135.41	132.60	-2.82
C2-C3-H3	118.27	118.85	0.58
C2-C3-C4	120.70	120.39	-0.31
H3-C3-C4	121.03	120.76	-0.27
C5-C4-C3	119.45	119.58	0.13
C5-C4-H4	120.10	120.18	0.08
C3-C4-H4	120.44	120.23	-0.21
C7-C14-H141	111.71	107.82	-3.89
C7-C14-H142	109.71	107.55	-2.16
C7-C14-C15	113.35	117.92	4.57
H141-C14-H142	106.69	107.41	0.72
H141-C14-C15	107.52	108.47	0.95
H142-C14-C15	107.55	107.24	-0.31
C14-C15-H15	108.46	109.06	0.60
C14-C15-O15	106.35	110.61	4.26
C14-C15-C16	111.78	111.75	-0.03
H15-C15-O15	110.03	109.59	-0.43
H15-C15-C16	109.58	109.18	-0.40
O15-C15-C16	110.58	106.61	-3.97
C15-O15-H15O	107.24	106.33	-0.91
C15-C16-H161	109.73	110.21	0.48
C15-C16-H162	110.61	111.02	0.41
C15-C16-H163	111.27	110.26	-1.01
H161-C16-H162	108.28	108.55	0.27
H161-C16-H163	109.08	108.38	-0.70
H162-C16-H163	107.79	108.34	0.55

Figure 207: The PES scan for flexible dihedral corresponding to hpiz.

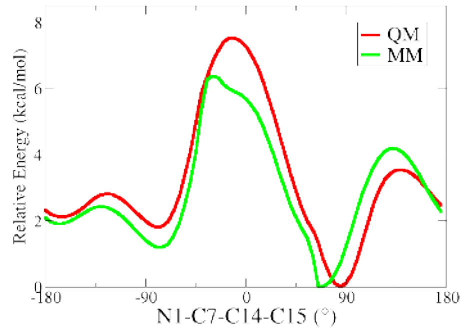

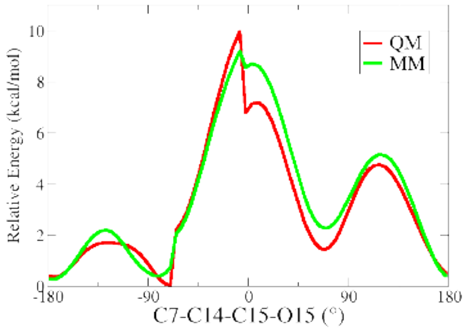

3.51 benzyl methyl ammonium (bzma, for 5AU, U8U, 5DU, SCU, IAU, ISU, etc.)

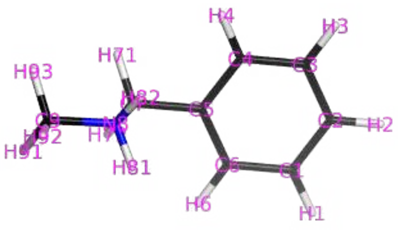

Figure 208: The energy-minimized structure of bzma.

Table 285: The calculated geometric terms of bzma.

Terms	QM	MM	diff
C1-H1	1.087	1.082	-0.005
C1-C2	1.399	1.400	0.000
C1-C6	1.397	1.401	0.003
C2-H2	1.087	1.082	-0.005
C2-C3	1.399	1.400	0.000
C3-H3	1.087	1.082	-0.005
C3-C4	1.397	1.401	0.003
C4-H4	1.091	1.081	-0.010
C4-C5	1.404	1.407	0.003
C5-C6	1.404	1.407	0.003
C5-C7	1.496	1.499	0.004
C6-H6	1.091	1.081	-0.010
C7-H71	1.094	1.103	0.010
C7-H72	1.094	1.103	0.010
C7-N8	1.523	1.510	-0.013
N8-H81	1.030	1.012	-0.018
N8-H82	1.030	1.012	-0.018
N8-C9	1.500	1.502	0.002
C9-H91	1.090	1.111	0.021
C9-H92	1.090	1.111	0.021
C9-H93	1.090	1.111	0.021
C1-H1	1.087	1.082	-0.005
C1-C2	1.399	1.400	0.000
C1-C6	1.397	1.401	0.003
C2-H2	1.087	1.082	-0.005
C2-C3	1.399	1.400	0.000
C3-H3	1.087	1.082	-0.005
C3-C4	1.397	1.401	0.003
C4-H4	1.091	1.081	-0.010
C4-C5	1.404	1.407	0.003
C5-C6	1.404	1.407	0.003
C5-C7	1.496	1.499	0.004
C6-H6	1.091	1.081	-0.010
C7-H71	1.094	1.103	0.010
C7-H72	1.094	1.103	0.010
C7-N8	1.523	1.510	-0.013
N8-H81	1.030	1.012	-0.018
N8-H82	1.030	1.012	-0.018
N8-C9	1.500	1.502	0.002
C9-H91	1.090	1.111	0.021
C9-H92	1.090	1.111	0.021
C9-H93	1.090	1.111	0.021
H1-C1-C2	120.14	119.69	-0.45
H1-C1-C6	119.87	120.27	0.40
C2-C1-C6	119.98	120.03	0.05
C1-C2-H2	119.87	119.96	0.08
C1-C2-C3	120.24	120.09	-0.16
H2-C2-C3	119.87	119.96	0.08
C2-C3-H3	120.14	119.69	-0.45
C2-C3-C4	119.98	120.03	0.05
H3-C3-C4	119.87	120.27	0.40
C3-C4-H4	119.69	118.97	-0.72
C3-C4-C5	119.90	120.25	0.35
H4-C4-C5	120.37	120.78	0.40
C4-C5-C6	119.99	119.35	-0.64
C4-C5-C7	119.97	120.32	0.35
C6-C5-C7	119.97	120.32	0.35
C1-C6-C5	119.90	120.25	0.35
C1-C6-H6	119.69	118.97	-0.72
C5-C6-H6	120.37	120.78	0.40
C5-C7-H71	112.89	111.06	-1.83
C5-C7-H72	112.89	111.06	-1.83
C5-C7-N8	108.91	109.57	0.67
H71-C7-H72	109.21	111.56	2.35
H71-C7-N8	106.27	106.69	0.43
H72-C7-N8	106.27	106.69	0.43
C7-N8-H81	107.82	107.79	-0.03
C7-N8-H82	107.82	107.79	-0.03
C7-N8-C9	115.13	115.34	0.21
H81-N8-H82	105.10	104.45	-0.65
H81-N8-C9	110.23	110.43	0.20
H82-N8-C9	110.23	110.43	0.20
N8-C9-H91	108.37	107.28	-1.09
N8-C9-H92	108.97	107.22	-1.76
N8-C9-H93	108.37	107.28	-1.09
H91-C9-H92	110.39	111.55	1.16
H91-C9-H93	110.28	111.67	1.39
H92-C9-H93	110.39	111.55	1.16
H1-C1-C2	120.14	119.69	-0.45
H1-C1-C6	119.87	120.27	0.40
C2-C1-C6	119.98	120.03	0.05
C1-C2-H2	119.87	119.96	0.08
C1-C2-C3	120.24	120.09	-0.16
H2-C2-C3	119.87	119.96	0.08
C2-C3-H3	120.14	119.69	-0.45
C2-C3-C4	119.98	120.03	0.05
H3-C3-C4	119.87	120.27	0.40
C3-C4-H4	119.69	118.97	-0.72
C3-C4-C5	119.90	120.25	0.35
H4-C4-C5	120.37	120.78	0.40
C4-C5-C6	119.99	119.35	-0.64
C4-C5-C7	119.97	120.32	0.35
C6-C5-C7	119.97	120.32	0.35
C1-C6-C5	119.90	120.25	0.35

Terms	QM	MM	diff
C1-C6-H6	119.69	118.97	-0.72
C5-C6-H6	120.37	120.78	0.40
C5-C7-H71	112.89	111.06	-1.83
C5-C7-H72	112.89	111.06	-1.83
C5-C7-N8	108.91	109.57	0.67
H71-C7-H72	109.21	111.56	2.35
H71-C7-N8	106.27	106.69	0.43
H72-C7-N8	106.27	106.69	0.43
C7-N8-H81	107.82	107.79	-0.03
C7-N8-H82	107.82	107.79	-0.03
C7-N8-C9	115.13	115.34	0.21
H81-N8-H82	105.10	104.45	-0.65
H81-N8-C9	110.23	110.43	0.20
H82-N8-C9	110.23	110.43	0.20
N8-C9-H91	108.37	107.28	-1.09
N8-C9-H92	108.97	107.22	-1.76
N8-C9-H93	108.37	107.28	-1.09
H91-C9-H92	110.39	111.55	1.16
H91-C9-H93	110.28	111.67	1.39
H92-C9-H93	110.39	111.55	1.16

Figure 209: The PES scan for flexible dihedral corresponding to bzma.

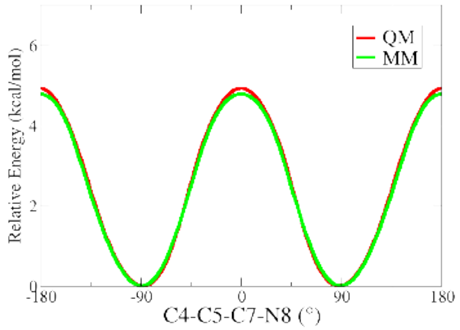

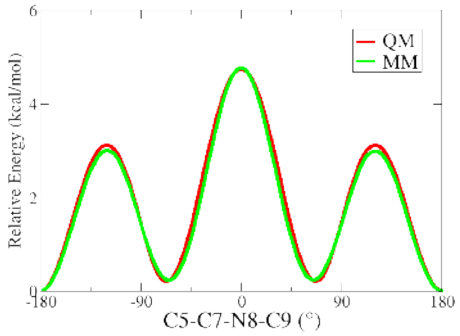

3.52 2-propylamino-4-imino-pyrimidine (prnc, for K2C & R2C)

Figure 210: The energy-minimized structure of prnc.

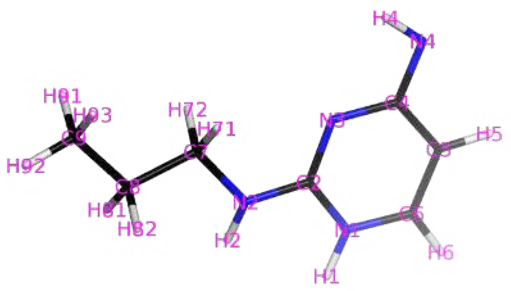

Table 286: The calculated geometric terms of prnc.

Terms	QM	MM	diff
N1-C2	1.386	1.393	0.007
N1-C6	1.393	1.366	-0.027
N1-H1	1.013	1.002	-0.011
C2-N2	1.383	1.349	-0.034
C2-N3	1.298	1.336	0.038
N2-H2	1.017	1.007	-0.010
N2-C7	1.462	1.484	0.022
N3-C4	1.414	1.382	-0.032
C4-N4	1.293	1.324	0.031
C4-C5	1.461	1.427	-0.034
N4-H4	1.028	0.998	-0.030
C5-H5	1.084	1.074	-0.009
C5-C6	1.348	1.392	0.044
C6-H6	1.085	1.085	0.000
C7-H71	1.092	1.115	0.023
C7-H72	1.099	1.114	0.015
C7-C8	1.522	1.535	0.014
C8-H81	1.098	1.114	0.015
C8-H82	1.095	1.115	0.019
C8-C9	1.526	1.535	0.009
C9-H91	1.094	1.112	0.018
C9-H92	1.093	1.112	0.018
C9-H93	1.094	1.112	0.018
C2-N1-C6	118.20	120.24	2.04
C2-N1-H1	117.50	116.55	-0.96
C6-N1-H1	118.66	123.09	4.43
N1-C2-N2	113.96	116.42	2.46
N1-C2-N3	124.89	122.31	-2.58
N2-C2-N3	121.14	121.25	0.11
C2-N2-H2	113.18	112.07	-1.12
C2-N2-C7	117.75	120.38	2.63
H2-N2-C7	113.15	118.53	5.39
C2-N3-C4	118.89	118.28	-0.61
N3-C4-N4	122.57	120.62	-1.95
N3-C4-C5	117.74	121.65	3.91
N4-C4-C5	119.69	117.73	-1.96
C4-N4-H4	107.60	103.81	-3.79
C4-C5-H5	118.59	119.15	0.56
C4-C5-C6	119.97	117.52	-2.46
H5-C5-C6	121.43	123.33	1.90
N1-C6-C5	120.07	120.00	-0.07
N1-C6-H6	115.73	118.42	2.69
C5-C6-H6	124.16	121.57	-2.59
N2-C7-H71	107.37	110.21	2.84
N2-C7-H72	111.35	110.50	-0.85
N2-C7-C8	109.94	110.39	0.45
H71-C7-H72	106.61	107.73	1.12
H71-C7-C8	110.70	108.76	-1.94
H72-C7-C8	110.78	109.19	-1.59
C7-C8-H81	109.56	109.17	-0.38
C7-C8-H82	108.85	108.75	-0.10
C7-C8-C9	111.73	113.84	2.11
H81-C8-H82	106.84	107.64	0.80
H81-C8-C9	109.43	108.62	-0.81
H82-C8-C9	110.31	108.64	-1.67
C8-C9-H91	111.02	110.70	-0.31
C8-C9-H92	111.14	110.63	-0.51
C8-C9-H93	110.80	110.60	-0.20
H91-C9-H92	107.93	108.30	0.37
H91-C9-H93	107.89	108.28	0.39
H92-C9-H93	107.93	108.24	0.31

Figure 211: The PES scan for flexible dihedral corresponding to prnc.

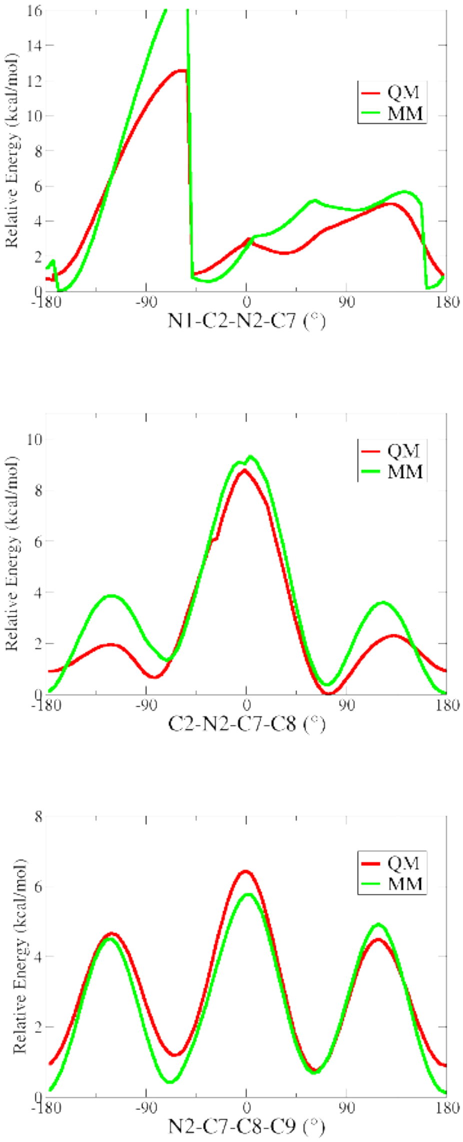

3.53 2-propylamino-4-imino-pyrimidine, protonated (pncp, for K2C & R2C)

Figure 212: The energy-minimized structure of pncp.

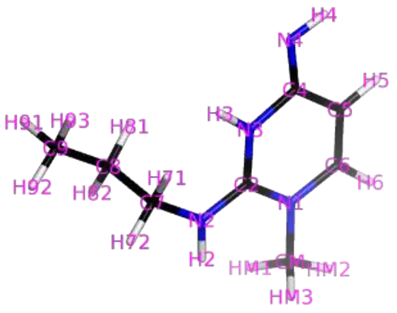

Table 287: The calculated geometric terms of pncp.

Terms	QM	MM	diff
N1-C2	1.356	1.384	0.028
N1-C6	1.402	1.403	0.002
N1-CM	1.468	1.482	0.014
C2-N2	1.336	1.341	0.005
C2-N3	1.341	1.369	0.028
N2-H2	1.013	0.994	-0.018
N2-C7	1.473	1.467	-0.006
N3-H3	1.018	0.988	-0.030
N3-C4	1.425	1.401	-0.024
C4-N4	1.279	1.294	0.015
C4-C5	1.452	1.425	-0.027
N4-H4	1.023	0.998	-0.025
C5-H5	1.084	1.077	-0.007
C5-C6	1.347	1.387	0.039
C6-H6	1.084	1.085	0.001
C7-H71	1.096	1.106	0.010
C7-H72	1.093	1.106	0.013
C7-C8	1.527	1.538	0.012
C8-H81	1.096	1.113	0.017
C8-H82	1.095	1.114	0.019
C8-C9	1.527	1.537	0.010
C9-H91	1.092	1.113	0.021
C9-H92	1.093	1.112	0.018
C9-H93	1.094	1.112	0.018
C2-N1-C6	119.33	120.76	1.42
C2-N1-CM	120.46	120.92	0.46
C6-N1-CM	120.19	118.32	-1.87
N1-C2-N2	120.87	121.65	0.78
N1-C2-N3	118.40	118.09	-0.32
N2-C2-N3	120.71	120.26	-0.45
C2-N2-H2	117.69	115.67	-2.03
C2-N2-C7	124.67	123.88	-0.79
H2-N2-C7	117.05	120.32	3.26
C2-N3-H3	120.07	122.57	2.51
C2-N3-C4	126.73	124.30	-2.43
H3-N3-C4	113.20	113.12	-0.08
N3-C4-N4	114.65	114.63	-0.02
N3-C4-C5	112.48	116.36	3.88
N4-C4-C5	132.87	129.01	-3.86
C4-N4-H4	111.53	108.98	-2.55
C4-C5-H5	119.76	118.54	-1.22
C4-C5-C6	120.30	120.27	-0.03
H5-C5-C6	119.95	121.19	1.24
N1-C6-C5	122.74	120.23	-2.52
N1-C6-H6	114.60	116.28	1.68
C5-C6-H6	122.65	123.49	0.84
N2-C7-H71	108.49	112.36	3.86
N2-C7-H72	105.87	109.48	3.61
N2-C7-C8	113.43	109.62	-3.82
H71-C7-H72	106.04	105.73	-0.31
H71-C7-C8	111.99	110.71	-1.28
H72-C7-C8	110.57	108.82	-1.75
C7-C8-H81	110.15	110.69	0.54
C7-C8-H82	108.66	109.85	1.19
C7-C8-C9	111.02	110.96	-0.06
H81-C8-H82	106.59	107.64	1.05
H81-C8-C9	110.07	108.75	-1.32
H82-C8-C9	110.24	108.87	-1.37
C8-C9-H91	110.42	110.36	-0.06
C8-C9-H92	111.11	111.18	0.07
C8-C9-H93	111.36	111.26	-0.10
H91-C9-H92	107.83	107.73	-0.11
H91-C9-H93	107.81	107.75	-0.06
H92-C9-H93	108.17	108.42	0.26

Figure 213: The PES scan for flexible dihedral corresponding to pncp.

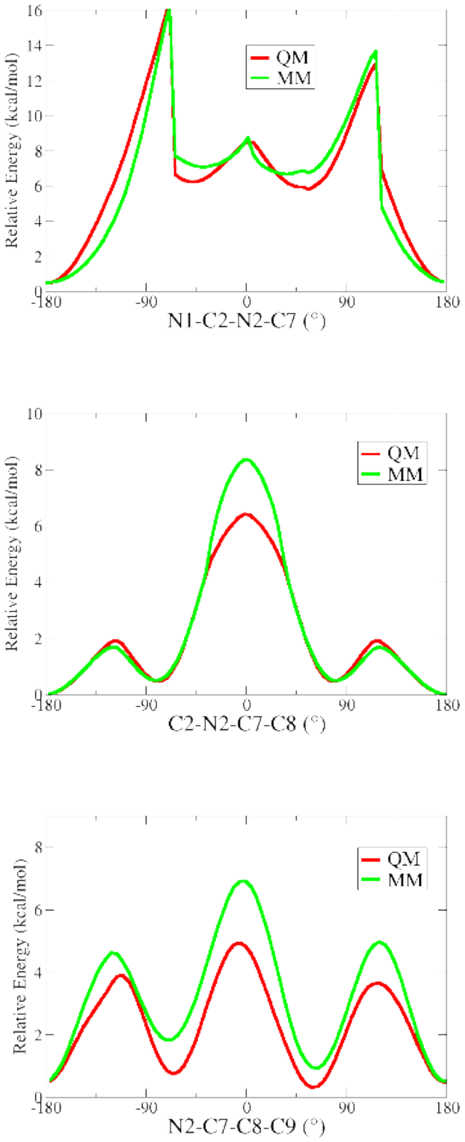

3.54 3-methylamino epoxypentene (ponm, for EQG)

Figure 214: The energy-minimized structure of ponm.

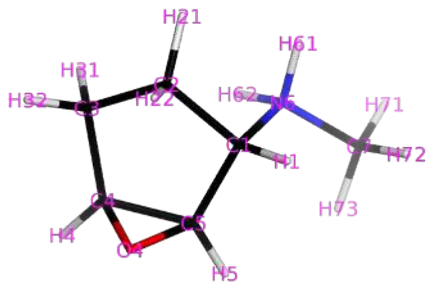

Table 288: The calculated geometric terms of ponm.

Terms	QM	MM	diff
C1-H1	1.094	1.086	-0.008
C1-C2	1.536	1.554	0.017
C1-C5	1.515	1.505	-0.009
C1-N6	1.521	1.495	-0.026
C2-H21	1.096	1.097	0.001
C2-H22	1.091	1.106	0.015
C2-C3	1.543	1.536	-0.006
C3-H31	1.098	1.101	0.004
C3-H32	1.093	1.102	0.009
C3-C4	1.509	1.507	-0.002
C4-H4	1.087	1.103	0.016
C4-O4	1.445	1.436	-0.009
C4-C5	1.476	1.478	0.003
O4-C5	1.433	1.436	0.003
C5-H5	1.088	1.075	-0.013
N6-H61	1.029	1.009	-0.020
N6-H62	1.030	1.008	-0.022
N6-C7	1.503	1.503	0.001
C7-H71	1.090	1.111	0.021
C7-H72	1.089	1.110	0.021
C7-H73	1.087	1.110	0.022
H1-C1-C2	113.50	116.58	3.08
H1-C1-C5	114.19	110.64	-3.55
H1-C1-N6	105.93	108.35	2.42
C2-C1-C5	104.18	104.49	0.31
C2-C1-N6	110.08	107.60	-2.49
C5-C1-N6	108.93	108.94	0.01
C1-C2-H21	112.94	114.40	1.46
C1-C2-H22	107.35	107.33	-0.02
C1-C2-C3	105.94	106.71	0.77
H21-C2-H22	107.03	105.55	-1.47
H21-C2-C3	113.53	113.60	0.08
H22-C2-C3	109.91	109.00	-0.91
C2-C3-H31	112.69	112.76	0.07
C2-C3-H32	111.60	112.32	0.72
C2-C3-C4	103.18	102.40	-0.78
H31-C3-H32	107.15	107.12	-0.03
H31-C3-C4	110.08	110.62	0.54
H32-C3-C4	112.21	111.67	-0.54
C3-C4-H4	122.43	121.72	-0.71
C3-C4-O4	112.00	113.24	1.24
C3-C4-C5	109.78	111.43	1.65
H4-C4-O4	115.19	114.30	-0.89
H4-C4-C5	121.97	121.03	-0.94
C1-C5-C4	108.14	107.39	-0.75
C1-C5-O4	110.32	110.71	0.39
C1-C5-H5	122.39	123.47	1.08
C4-C5-H5	124.31	123.48	-0.83
O4-C5-H5	115.44	115.40	-0.04
C1-N6-H61	108.18	106.08	-2.10
C1-N6-H62	108.45	105.91	-2.54
C1-N6-C7	115.53	117.53	2.00
H61-N6-H62	105.81	105.51	-0.30
H61-N6-C7	108.82	110.21	1.39
H62-N6-C7	109.60	110.82	1.22
N6-C7-H71	108.65	107.02	-1.63
N6-C7-H72	108.12	107.34	-0.78
N6-C7-H73	109.03	107.65	-1.37
H71-C7-H72	110.40	111.43	1.03
H71-C7-H73	110.13	111.32	1.18
H72-C7-H73	110.46	111.81	1.34

Figure 215: The PES scan for flexible dihedral corresponding to ponm.

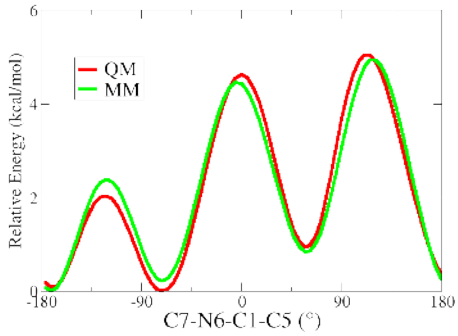

3.55 1-(3-pyrrolyl)-N-(3-cyclopentenyl) methylammonium (pnpa, for QUG, MQG & GQG)

Figure 216: The energy-minimized structure of pnpa.

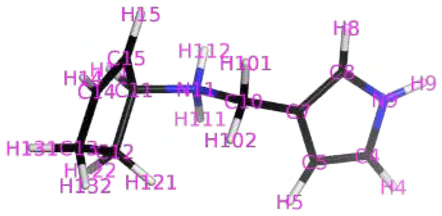

Table 289: The calculated geometric terms of pnpa.

Terms	QM	MM	diff
N9-H9	1.014	1.015	0.001
N9-C8	1.364	1.377	0.014
N9-C4	1.372	1.375	0.003
C8-H8	1.082	1.084	0.002
C8-C7	1.392	1.365	-0.027
C7-C5	1.424	1.366	-0.058
C7-C10	1.480	1.486	0.006
C5-H5	1.084	1.081	-0.002
C5-C4	1.381	1.358	-0.023
C4-H4	1.081	1.085	0.005
C10-H101	1.091	1.102	0.011
C10-H102	1.092	1.101	0.009
C10-N11	1.524	1.516	-0.009
N11-H111	1.030	1.008	-0.022
N11-H112	1.030	1.005	-0.025
N11-C11	1.531	1.499	-0.032
C11-H11	1.096	1.084	-0.012
C11-C12	1.538	1.527	-0.010
C11-C15	1.499	1.503	0.004
C12-H121	1.095	1.102	0.006
C12-H122	1.093	1.102	0.009
C12-C13	1.543	1.523	-0.021
C13-H131	1.094	1.105	0.011
C13-H132	1.097	1.104	0.007
C13-C14	1.504	1.514	0.010
C14-H14	1.087	1.089	0.002
C14-C15	1.344	1.372	0.028
C15-H15	1.087	1.082	-0.004
H9-N9-C8	124.68	125.96	1.29
H9-N9-C4	124.59	124.64	0.05
C8-N9-C4	110.70	109.34	-1.36
N9-C8-H8	121.71	123.33	1.62
N9-C8-C7	107.03	106.70	-0.33
H8-C8-C7	131.21	129.95	-1.26
C8-C7-C5	107.55	108.16	0.61
C8-C7-C10	125.70	126.59	0.89
C5-C7-C10	126.64	125.25	-1.39
C7-C5-H5	127.34	125.99	-1.35
C7-C5-C4	107.15	109.52	2.37
H5-C5-C4	125.44	124.46	-0.98
N9-C4-C5	107.57	106.28	-1.29
N9-C4-H4	121.33	124.15	2.82
C5-C4-H4	131.09	129.55	-1.54
C7-C10-H101	113.24	111.93	-1.30
C7-C10-H102	112.72	111.33	-1.39
C7-C10-N11	108.77	109.00	0.23
H101-C10-H102	109.03	110.90	1.88
H101-C10-N11	106.27	106.43	0.16
H102-C10-N11	106.38	106.98	0.59
C10-N11-H111	107.38	106.81	-0.57
C10-N11-H112	106.65	108.14	1.50
C10-N11-C11	117.80	120.67	2.87
H111-N11-H112	105.03	105.49	0.46
H111-N11-C11	109.64	106.69	-2.95
H112-N11-C11	109.57	108.07	-1.50
N11-C11-H11	103.94	106.53	2.59
N11-C11-C12	111.79	109.89	-1.90
N11-C11-C15	110.24	110.23	-0.00
H11-C11-C12	112.83	113.99	1.16
H11-C11-C15	113.87	111.63	-2.24
C12-C11-C15	104.37	104.60	0.23
C11-C12-H121	113.33	113.62	0.29
C11-C12-H122	107.43	111.06	3.63
C11-C12-C13	105.84	105.64	-0.20
H121-C12-H122	106.65	105.44	-1.21
H121-C12-C13	113.87	109.59	-4.28
H122-C12-C13	109.54	111.61	2.07
C12-C13-H131	111.59	110.92	-0.67
C12-C13-H132	113.04	111.72	-1.32
C12-C13-C14	103.08	104.28	1.21
H131-C13-H132	106.32	105.38	-0.94
H131-C13-C14	112.03	111.42	-0.62
H132-C13-C14	110.92	113.26	2.34
C13-C14-H14	122.89	123.17	0.28
C13-C14-C15	112.71	110.18	-2.53
H14-C14-C15	124.40	126.26	1.86
C11-C15-C14	110.87	110.85	-0.02
C11-C15-H15	123.22	123.86	0.65
C14-C15-H15	125.91	125.24	-0.68

Figure 217: The PES scan for flexible dihedral corresponding to pnpa.

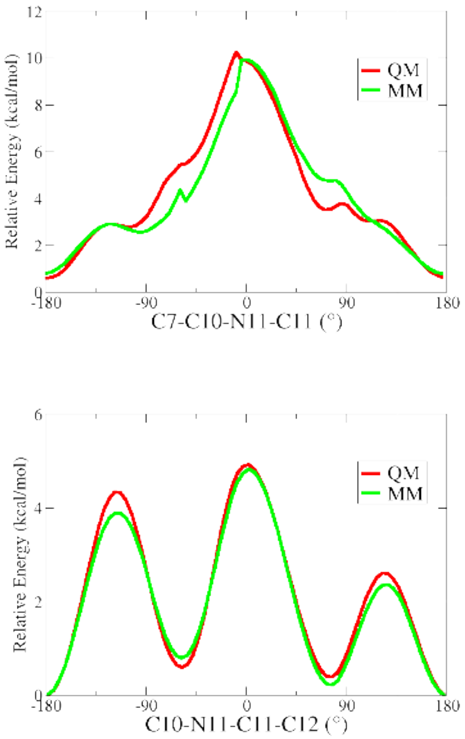

3.56 tetrahydrofuran-1-oxy-cyclopentane (rbrb, for RIA & RIG)

Figure 218: The energy-minimized structure of rbrb.

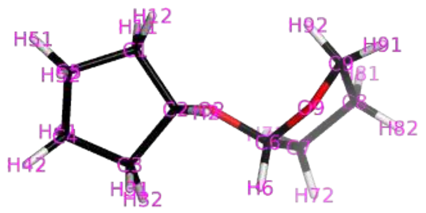

Table 290: The calculated geometric terms of rbrb.

Terms	QM	MM	diff
C1-H11	1.091	1.102	0.011
C1-H12	1.098	1.098	-0.000
C1-C2	1.526	1.537	0.011
C1-C5	1.536	1.538	0.001
C2-H2	1.100	1.102	0.002
C2-O2	1.440	1.424	-0.015
C2-C3	1.523	1.539	0.017
O2-C6	1.411	1.419	0.007
C3-H31	1.096	1.101	0.004
C3-H32	1.093	1.098	0.005
C3-C4	1.544	1.538	-0.005
C4-H41	1.093	1.103	0.010
C4-H42	1.094	1.098	0.004
C4-C5	1.552	1.530	-0.022
C5-H51	1.094	1.098	0.005
C5-H52	1.094	1.103	0.008
C6-H6	1.098	1.100	0.002
C6-C7	1.517	1.531	0.014
C6-O9	1.417	1.427	0.010
C7-H71	1.092	1.100	0.009
C7-H72	1.095	1.098	0.003
C7-C8	1.531	1.540	0.010
C8-H81	1.092	1.098	0.005
C8-H82	1.093	1.102	0.009
C8-C9	1.537	1.523	-0.014
C9-H91	1.095	1.099	0.004
C9-H92	1.095	1.101	0.006
C9-O9	1.446	1.425	-0.022
H11-C1-H12	108.28	106.22	-2.05
H11-C1-C2	112.23	109.90	-2.33
H11-C1-C5	114.23	109.89	-4.34
H12-C1-C2	108.50	112.17	3.67
H12-C1-C5	110.55	112.47	1.92
C2-C1-C5	102.89	106.22	3.33
C1-C2-H2	113.66	111.86	-1.80
C1-C2-O2	111.07	108.06	-3.01
C1-C2-C3	102.78	106.23	3.45
H2-C2-O2	109.36	111.37	2.01
H2-C2-C3	113.62	111.33	-2.29
O2-C2-C3	106.00	107.75	1.75
C2-O2-C6	114.79	110.99	-3.79
C2-C3-H31	108.80	109.42	0.62
C2-C3-H32	111.58	112.79	1.21
C2-C3-C4	104.05	106.26	2.21
H31-C3-H32	108.02	106.43	-1.60
H31-C3-C4	110.80	110.24	-0.56
H32-C3-C4	113.51	111.72	-1.78
C3-C4-H41	110.38	109.57	-0.82
C3-C4-H42	111.30	113.30	2.00
C3-C4-C5	105.71	104.06	-1.65
H41-C4-H42	107.03	106.78	-0.25
H41-C4-C5	110.55	109.60	-0.95
H42-C4-C5	111.91	113.50	1.59
C1-C5-C4	105.37	103.80	-1.58
C1-C5-H51	109.60	113.09	3.49
C1-C5-H52	112.15	109.96	-2.19
C4-C5-H51	110.06	113.31	3.25
C4-C5-H52	112.44	109.91	-2.53
H51-C5-H52	107.23	106.79	-0.44
O2-C6-H6	110.24	111.27	1.02
O2-C6-C7	106.80	107.61	0.81
O2-C6-O9	113.44	111.48	-1.96
H6-C6-C7	115.95	113.55	-2.40
H6-C6-O9	105.93	108.37	2.44
C7-C6-O9	104.54	104.39	-0.15
C6-C7-H71	112.34	110.72	-1.62
C6-C7-H72	109.05	111.77	2.73
C6-C7-C8	100.76	104.82	4.06
H71-C7-H72	109.32	106.67	-2.65
H71-C7-C8	113.94	111.11	-2.84
H72-C7-C8	111.14	111.84	0.70
C7-C8-H81	109.69	113.34	3.64
C7-C8-H82	113.19	110.11	-3.08
C7-C8-C9	102.54	103.22	0.68
H81-C8-H82	108.42	107.11	-1.32
H81-C8-C9	110.70	113.14	2.45
H82-C8-C9	112.22	109.92	-2.30
C8-C9-H91	112.55	114.87	2.32
C8-C9-H92	112.47	112.79	0.33
C8-C9-O9	106.72	102.38	-4.34
H91-C9-H92	108.59	109.11	0.52
H91-C9-O9	106.87	109.53	2.66
H92-C9-O9	109.45	107.73	-1.72
C6-O9-C9	108.13	108.11	-0.02

Figure 219: The PES scan for flexible dihedral corresponding to rbrb.

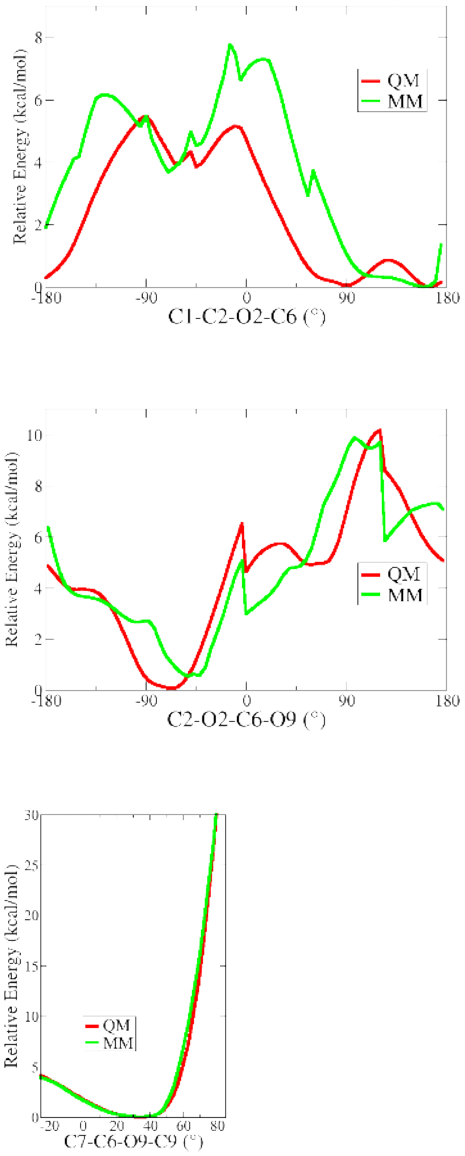

3.57 N1,N1,N2-trimethylurea (mmmu, for HNA, 26A, T6A, 12A & 6GA)

Figure 220: The energy-minimized structure of mmmu.

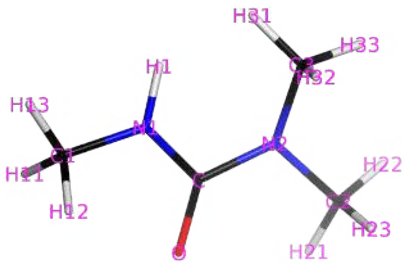

Table 291: The calculated geometric terms of mmmu.

Terms	QM	MM	diff
N1-H1	1.012	0.991	-0.021
N1-C1	1.457	1.442	-0.015
N1-C	1.386	1.365	-0.021
C1-H11	1.096	1.114	0.018
C1-H12	1.089	1.114	0.025
C1-H13	1.092	1.112	0.020
C-O	1.234	1.226	-0.008
C-N2	1.390	1.376	-0.014
N2-C2	1.458	1.451	-0.007
N2-C3	1.457	1.451	-0.006
C2-H21	1.088	1.113	0.026
C2-H22	1.093	1.110	0.017
C2-H23	1.098	1.111	0.013
C3-H31	1.093	1.111	0.017
C3-H32	1.097	1.112	0.014
C3-H33	1.091	1.111	0.020
H1-N1-C1	115.20	119.32	4.12
H1-N1-C	116.33	118.25	1.92
C1-N1-C	116.81	120.93	4.12
N1-C1-H11	112.81	110.41	-2.41
N1-C1-H12	108.68	110.29	1.61
N1-C1-H13	108.32	110.85	2.53
H11-C1-H12	108.15	108.02	-0.14
H11-C1-H13	109.53	108.68	-0.85
H12-C1-H13	109.30	108.51	-0.79
N1-C-O	121.85	121.69	-0.16
N1-C-N2	115.07	113.84	-1.22
O-C-N2	123.08	124.47	1.39
C-N2-C2	115.21	115.42	0.21
C-N2-C3	120.11	121.18	1.07
C2-N2-C3	115.35	118.69	3.34
N2-C2-H21	108.57	110.36	1.78
N2-C2-H22	108.80	107.37	-1.43
N2-C2-H23	112.12	107.51	-4.61
H21-C2-H22	109.74	109.47	-0.28
H21-C2-H23	108.52	111.04	2.51
H22-C2-H23	109.06	111.01	1.96
N2-C3-H31	111.63	110.21	-1.42
N2-C3-H32	112.68	107.81	-4.87
N2-C3-H33	107.96	106.92	-1.04
H31-C3-H32	108.69	112.50	3.81
H31-C3-H33	106.85	108.91	2.06
H32-C3-H33	108.82	110.32	1.50

Figure 221: The PES scan for flexible dihedral corresponding to mmmu.

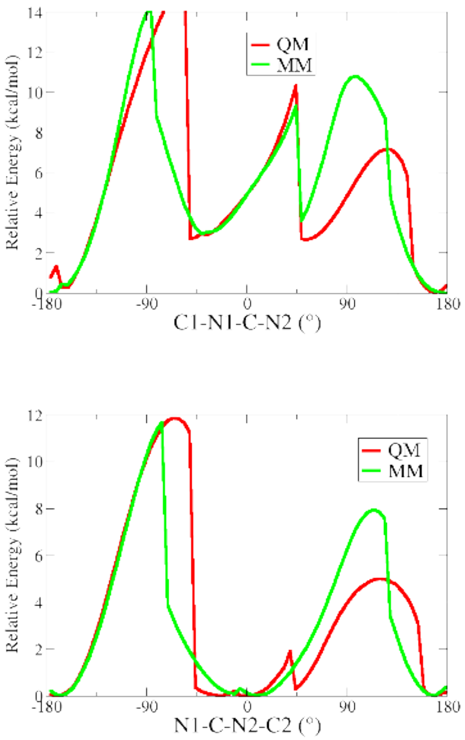

3.58 1-(tetrahydrofuran-2-yl) dihydrouracil (dfu, for chi of H2U)

Figure 222: The energy-minimized structure of dfu.

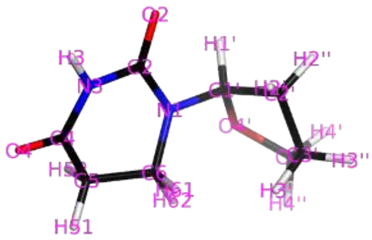

Table 292: The calculated geometric terms of dfu.

Terms	QM	MM	diff
N1-C2	1.370	1.368	-0.002
N1-C6	1.458	1.461	0.003
N1-C1’	1.443	1.449	0.006
C2-O2	1.228	1.224	-0.005
C2-N3	1.408	1.359	-0.049
N3-H3	1.017	0.989	-0.028
N3-C4	1.390	1.322	-0.068
C4-O4	1.224	1.222	-0.002
C4-C5	1.510	1.480	-0.030
C5-H51	1.093	1.110	0.017
C5-H52	1.097	1.112	0.016
C5-C6	1.520	1.527	0.007
C6-H61	1.099	1.113	0.014
C6-H62	1.091	1.114	0.022
C1’-H1’	1.095	1.118	0.023
C1’-C2’	1.525	1.498	-0.027
C1’-O4’	1.433	1.414	-0.019
C2’-H2’	1.096	1.110	0.015
C2’-H2”	1.093	1.114	0.021
C2’-C3’	1.525	1.500	-0.025
C3’-H3’	1.093	1.110	0.017
C3’-H3”	1.095	1.111	0.016
C3’-C4’	1.534	1.520	-0.014
C4’-H4’	1.095	1.112	0.018
C4’-H4”	1.096	1.112	0.015
C4’-O4’	1.442	1.448	0.005
C2-N1-C6	120.55	119.66	-0.88
C2-N1-C1’	119.07	115.15	-3.92
C6-N1-C1’	119.78	123.66	3.88
N1-C2-O2	125.58	124.95	-0.64
N1-C2-N3	113.60	115.03	1.43
O2-C2-N3	120.81	120.02	-0.79
C2-N3-H3	114.23	116.31	2.08
C2-N3-C4	127.49	127.64	0.14
H3-N3-C4	116.34	116.06	-0.28
N3-C4-O4	121.67	120.94	-0.73
N3-C4-C5	113.26	117.89	4.63
O4-C4-C5	125.07	121.17	-3.90
C4-C5-H51	108.12	110.00	1.88
C4-C5-H52	108.33	108.94	0.61
C4-C5-C6	110.44	107.04	-3.39
H51-C5-H52	108.20	109.19	1.00
H51-C5-C6	112.18	111.50	-0.68
H52-C5-C6	109.47	110.12	0.65
N1-C6-C5	109.13	110.30	1.17
N1-C6-H61	110.90	109.44	-1.47
N1-C6-H62	107.15	110.39	3.24
C5-C6-H61	110.29	111.61	1.32
C5-C6-H62	110.53	107.57	-2.96
H61-C6-H62	108.80	107.48	-1.31
N1-C1’-H1’	106.40	111.89	5.49
N1-C1’-C2’	115.89	115.18	-0.70
N1-C1’-O4’	107.56	109.43	1.87
H1’-C1’-C2’	111.17	108.88	-2.29
H1’-C1’-O4’	110.93	104.31	-6.62
C2’-C1’-O4’	104.88	106.45	1.57
C1’-C2’-H2’	109.70	112.21	2.51
C1’-C2’-H2”	111.82	109.67	-2.16
C1’-C2’-C3’	101.17	101.59	0.42
H2’-C2’-H2”	108.67	109.41	0.74
H2’-C2’-C3’	110.91	113.17	2.26
H2”-C2’-C3’	114.38	110.58	-3.80
C2’-C3’-H3’	113.75	112.46	-1.28
C2’-C3’-H3”	110.03	112.75	2.73
C2’-C3’-C4’	102.08	101.43	-0.64
H3’-C3’-H3”	107.91	110.28	2.36
H3’-C3’-C4’	112.57	108.95	-3.62
H3”-C3’-C4’	110.45	110.61	0.16
C3’-C4’-H4’	112.92	110.18	-2.74
C3’-C4’-H4”	111.75	112.27	0.52
C3’-C4’-O4’	106.65	103.01	-3.64
H4’-C4’-H4”	108.46	111.60	3.15
H4’-C4’-O4’	108.85	108.31	-0.54
H4”-C4’-O4’	108.06	111.08	3.02
C1’-O4’-C4’	109.20	109.85	0.66

Figure 223: The PES scan for flexible dihedral corresponding to dfu.

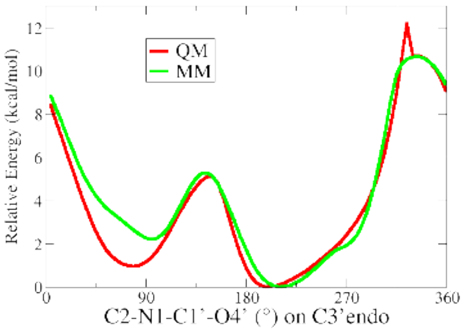

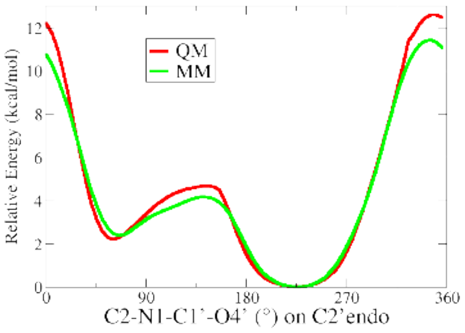

3.59 1-(1-imidazolyl)-2-methoxyl-3-hydroxyl tetrahydrofuran (ifu, for phi of 2'-OMe)

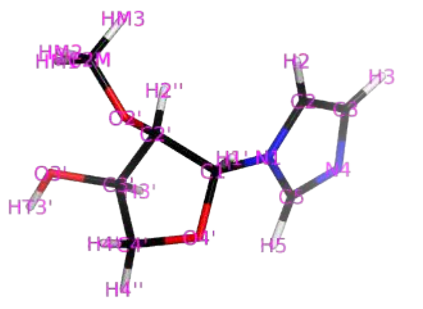

Figure 224: The energy-minimized structure of ifu.

Table 293: The calculated geometric terms of ifu.

Terms	QM	MM	diff
C1'-H1'	1.096	1.115	0.019
C1'-C2'	1.535	1.516	-0.019
C1'-O4'	1.421	1.426	0.005
C1'-N1	1.458	1.478	0.020
C2'-H2"	1.099	1.109	0.010
C2'-O2'	1.416	1.431	0.015
C2'-C3'	1.528	1.521	-0.007
O2'-C2M	1.431	1.423	-0.008
C3'-H3'	1.100	1.113	0.013
C3'-O3'	1.418	1.431	0.013
C3'-C4'	1.525	1.524	-0.000
O3'-HT3'	0.973	0.961	-0.011
C4'-H4'	1.095	1.113	0.017
C4'-H4"	1.093	1.110	0.017
C4'-O4'	1.440	1.447	0.007
N1-C2	1.378	1.379	0.001
N1-C5	1.371	1.372	0.002
C2-H2	1.081	1.081	-0.001
C2-C3	1.379	1.362	-0.017
C3-H3	1.082	1.084	0.002
C3-N4	1.375	1.384	0.010
N4-C5	1.328	1.321	-0.006
C5-H5	1.080	1.092	0.012
H1'-C1'-C2'	110.38	109.07	-1.31
H1'-C1'-O4'	110.51	106.07	-4.45
H1'-C1'-N1	108.81	109.16	0.35
C2'-C1'-O4'	107.03	107.56	0.53
C2'-C1'-N1	112.06	111.75	-0.31
O4'-C1'-N1	108.02	113.02	4.99
C1'-C2'-H2"	112.14	113.94	1.80
C1'-C2'-O2'	105.96	107.12	1.15
C1'-C2'-C3'	101.03	100.61	-0.42
H2"-C2'-O2'	111.55	110.93	-0.62
H2"-C2'-C3'	113.61	111.33	-2.28
O2'-C2'-C3'	111.84	112.49	0.65
C2'-O2'-C2M	113.71	110.93	-2.78
C2'-C3'-H3'	108.60	110.48	1.88
C2'-C3'-O3'	111.26	114.69	3.43
C2'-C3'-C4'	100.05	101.05	1.00
H3'-C3'-O3'	111.60	110.99	-0.61
H3'-C3'-C4'	109.83	108.37	-1.47
O3'-C3'-C4'	114.81	110.71	-4.10
C3'-O3'-HT3'	107.58	110.37	2.79
C3'-C4'-H4'	109.47	109.54	0.07
C3'-C4'-H4"	114.69	112.07	-2.62
C3'-C4'-O4'	104.03	103.51	-0.52
H4'-C4'-H4"	110.10	111.73	1.64
H4'-C4'-O4'	111.10	108.44	-2.66
H4"-C4'-O4'	107.29	111.19	3.91
C1'-O4'-C4'	108.92	108.77	-0.15
C1'-N1-C2	125.45	126.00	0.55
C1'-N1-C5	127.31	127.98	0.67
C2-N1-C5	107.03	106.02	-1.01
N1-C2-H2	121.98	123.73	1.74
N1-C2-C3	105.30	106.30	1.00
H2-C2-C3	132.71	129.97	-2.73
C2-C3-H3	127.71	129.60	1.89
C2-C3-N4	110.94	110.72	-0.21
H3-C3-N4	121.35	119.68	-1.67
C3-N4-C5	104.94	104.53	-0.41
N1-C5-N4	111.79	112.43	0.64
N1-C5-H5	121.99	121.88	-0.12
N4-C5-H5	126.14	125.66	-0.48

Figure 225: The PES scan for flexible dihedral corresponding to ifu.

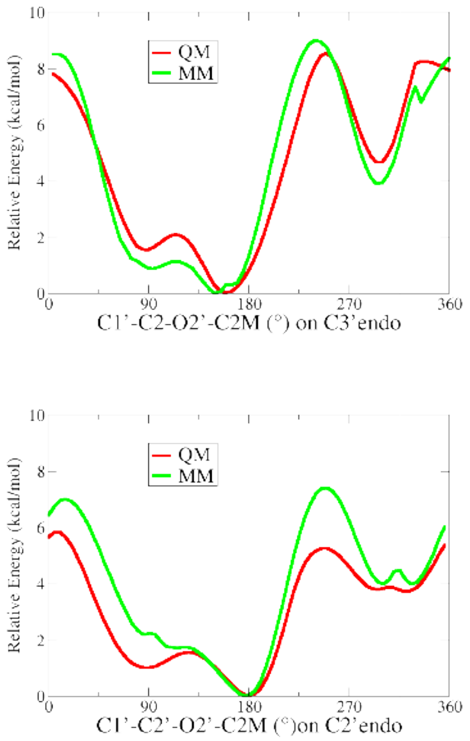

3.60 1-(tetrahydrofuran-2-yl)-7-methylguanine (mgf, for chi of 7MG)

Figure 226: The energy-minimized structure of mgf.

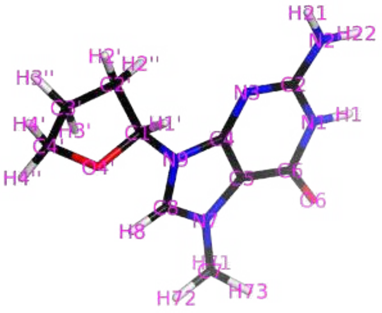

Table 294: The calculated geometric terms of mgf.

Terms	QM	MM	diff
N9-C8	1.348	1.329	-0.019
N9-C4	1.380	1.373	-0.007
N9-C1’	1.497	1.489	-0.008
C8-H8	1.080	1.073	-0.006
C8-N7	1.344	1.336	-0.008
N7-C5	1.374	1.408	0.034
N7-C7	1.470	1.481	0.010
C5-C6	1.441	1.416	-0.025
C5-C4	1.386	1.401	0.015
C6-O6	1.223	1.230	0.007
C6-N1	1.416	1.389	-0.027
N1-H1	1.020	1.003	-0.017
N1-C2	1.381	1.383	0.002
C2-N2	1.353	1.332	-0.021
C2-N3	1.324	1.349	0.026
N2-H21	1.013	0.998	-0.015
N2-H22	1.012	0.996	-0.016
N3-C4	1.351	1.344	-0.008
C7-H71	1.089	1.113	0.024
C7-H72	1.089	1.114	0.024
C7-H73	1.089	1.113	0.024
C1’-H1’	1.095	1.114	0.020
C1’-C2’	1.527	1.498	-0.029
C1’-O4’	1.402	1.424	0.022
C2’-H2”	1.091	1.110	0.018
C2’-H2’	1.095	1.115	0.020
C2’-C3’	1.529	1.502	-0.027
C3’-H3’	1.095	1.111	0.016
C3’-H3”	1.092	1.112	0.019
C3’-C4’	1.519	1.521	0.002
C4’-H4’	1.095	1.113	0.018
C4’-H4”	1.091	1.112	0.021
C4’-O4’	1.460	1.450	-0.010

Terms	QM	MM	diff
C8-N9-C4	108.54	112.30	3.75
C8-N9-C1’	125.20	122.88	-2.33
C4-N9-C1’	126.20	124.75	-1.46
N9-C8-H8	124.65	124.26	-0.39
N9-C8-N7	109.32	108.20	-1.12
H8-C8-N7	126.01	127.54	1.53
C8-N7-C5	107.93	107.90	-0.03
C8-N7-C7	126.27	126.06	-0.21
C5-N7-C7	125.79	126.03	0.24
N7-C5-C6	131.23	132.56	1.33
N7-C5-C4	107.92	107.65	-0.27
C6-C5-C4	120.85	119.78	-1.07
C5-C6-O6	128.74	128.75	0.01
C5-C6-N1	108.43	112.62	4.19
O6-C6-N1	122.82	118.63	-4.19
C6-N1-H1	113.56	116.53	2.97
C6-N1-C2	126.74	125.45	-1.28
H1-N1-C2	119.66	118.02	-1.64
N1-C2-N2	116.97	116.88	-0.09
N1-C2-N3	123.69	121.02	-2.67
N2-C2-N3	119.27	122.10	2.82
C2-N2-H21	115.77	117.91	2.15
C2-N2-H22	121.00	123.73	2.74
H21-N2-H22	116.34	118.35	2.01
C2-N3-C4	112.30	115.82	3.52
N9-C4-C5	106.28	103.95	-2.34
N9-C4-N3	125.73	130.73	5.00
C5-C4-N3	127.97	125.31	-2.67
N7-C7-H71	108.98	109.21	0.22
N7-C7-H72	108.05	111.11	3.06
N7-C7-H73	108.91	109.20	0.29
H71-C7-H72	110.62	109.18	-1.44
H71-C7-H73	109.68	108.97	-0.71
H72-C7-H73	110.55	109.14	-1.41
N9-C1’-H1’	105.83	108.02	2.19
N9-C1’-C2’	110.68	110.38	-0.30
N9-C1’-O4’	106.01	109.75	3.73
H1’-C1’-C2’	114.81	112.73	-2.08
H1’-C1’-O4’	111.19	109.45	-1.74
C2’-C1’-O4’	107.99	106.50	-1.49
C1’-C2’-H2”	112.63	114.44	1.81
C1’-C2’-H2’	108.22	109.48	1.26
C1’-C2’-C3’	102.01	101.19	-0.81
H2”-C2’-H2’	107.96	108.74	0.79
H2”-C2’-C3’	115.42	112.51	-2.91
H2’-C2’-C3’	110.36	110.28	-0.08
C2’-C3’-H3’	111.25	112.49	1.24
C2’-C3’-H3”	112.83	112.72	-0.11
C2’-C3’-C4’	100.99	101.57	0.57
H3’-C3’-H3”	108.24	110.23	1.99
H3’-C3’-C4’	110.71	109.06	-1.65
H3”-C3’-C4’	112.74	110.42	-2.32
C3’-C4’-H4’	112.16	110.11	-2.05
C3’-C4’-H4”	115.20	112.34	-2.85
C3’-C4’-O4’	104.47	102.75	-1.72
H4’-C4’-H4”	109.28	111.33	2.05
H4’-C4’-O4’	108.47	108.47	0.00
H4”-C4’-O4’	106.83	111.46	4.64
C1’-O4’-C4’	109.08	109.57	0.49

Figure 227: The PES scan for flexible dihedral corresponding to mgf.

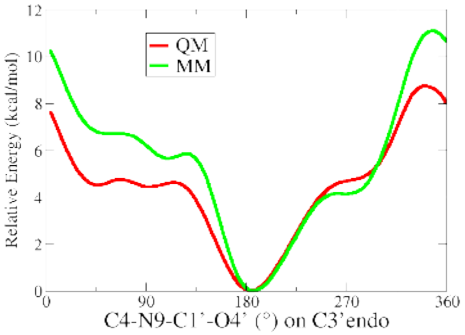

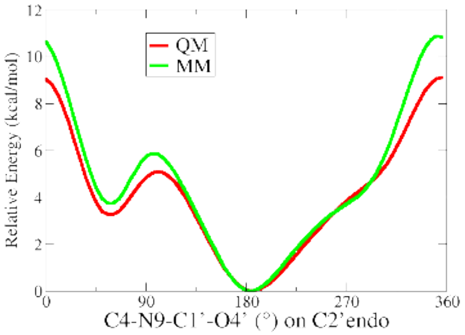

3.61 1-(tetrahydrofuran-2-yl)-2-methylthialcytosine (pcf, for chi of K2C, R2C)

Figure 228: The energy-minimized structure of pcf.

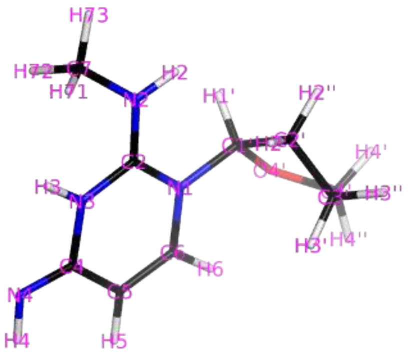

Table 295: The calculated geometric terms of pcf.

Terms	QM	MM	diff
N1-C2	1.353	1.377	0.024
N1-C6	1.401	1.412	0.010
N1-C1’	1.511	1.490	-0.021
C2-N2	1.341	1.347	0.006
C2-N3	1.341	1.371	0.030
N2-H2	1.010	0.996	-0.014
N2-C7	1.464	1.476	0.011
N3-H3	1.019	0.988	-0.031
N3-C4	1.426	1.401	-0.025
C4-N4	1.279	1.294	0.015
C4-C5	1.450	1.422	-0.028
N4-H4	1.023	0.998	-0.025
C5-H5	1.084	1.077	-0.007
C5-C6	1.349	1.385	0.036
C6-H6	1.081	1.086	0.005
C7-H71	1.093	1.115	0.022
C7-H72	1.093	1.116	0.023
C7-H73	1.089	1.115	0.026
C1’-H1’	1.098	1.116	0.018
C1’-C2’	1.535	1.499	-0.035
C1’-O4’	1.393	1.412	0.019
C2’-H2’	1.093	1.110	0.018
C2’-H2”	1.096	1.115	0.019
C2’-C3’	1.529	1.501	-0.029
C3’-H3’	1.094	1.111	0.017
C3’-H3”	1.092	1.112	0.019
C3’-C4’	1.518	1.518	0.000
C4’-H4’	1.095	1.113	0.018
C4’-H4”	1.091	1.113	0.022
C4’-O4’	1.460	1.450	-0.010
C2-N1-C6	119.28	120.22	0.95
C2-N1-C1’	120.14	118.50	-1.64
C6-N1-C1’	120.51	121.26	0.74
N1-C2-N2	121.69	123.75	2.07
N1-C2-N3	118.80	118.31	-0.49
N2-C2-N3	119.51	117.94	-1.57
C2-N2-H2	118.72	116.70	-2.02
C2-N2-C7	123.48	122.62	-0.86
H2-N2-C7	117.80	119.73	1.93
C2-N3-H3	120.35	122.83	2.48
C2-N3-C4	126.51	124.43	-2.07
H3-N3-C4	113.14	112.74	-0.40
N3-C4-N4	114.57	114.58	0.00
N3-C4-C5	112.27	116.28	4.01
N4-C4-C5	133.15	129.14	-4.02
C4-N4-H4	111.40	108.91	-2.49
C4-C5-H5	119.55	118.75	-0.80
C4-C5-C6	120.69	120.12	-0.57
H5-C5-C6	119.75	121.12	1.38
N1-C6-C5	122.42	120.58	-1.83
N1-C6-H6	113.92	116.12	2.21
C5-C6-H6	123.64	123.30	-0.35
N2-C7-H71	111.00	111.75	0.76
N2-C7-H72	110.79	112.16	1.37
N2-C7-H73	107.58	110.91	3.33
H71-C7-H72	110.41	108.11	-2.30
H71-C7-H73	108.47	106.79	-1.69
H72-C7-H73	108.47	106.83	-1.64
N1-C1’-H1’	106.02	109.53	3.52
N1-C1’-C2’	111.08	113.64	2.56
N1-C1’-O4’	107.82	111.78	3.96
H1’-C1’-C2’	115.01	111.28	-3.73
H1’-C1’-O4’	109.10	104.52	-4.58
C2’-C1’-O4’	107.61	105.66	-1.94
C1’-C2’-H2’	113.39	114.49	1.10
C1’-C2’-H2”	109.01	110.12	1.11
C1’-C2’-C3’	102.01	101.06	-0.95
H2’-C2’-H2”	108.48	109.38	0.90
H2’-C2’-C3’	113.71	111.56	-2.16
H2”-C2’-C3’	110.06	109.97	-0.09
C2’-C3’-H3’	111.01	112.51	1.50
C2’-C3’-H3”	112.71	113.00	0.29
C2’-C3’-C4’	101.03	101.27	0.24
H3’-C3’-H3”	108.35	110.26	1.90
H3’-C3’-C4’	110.76	108.96	-1.80
H3”-C3’-C4’	112.88	110.46	-2.42
C3’-C4’-H4’	112.38	110.33	-2.06
C3’-C4’-H4”	115.11	112.26	-2.86
C3’-C4’-O4’	104.32	102.61	-1.71
H4’-C4’-H4”	109.36	111.30	1.94
H4’-C4’-O4’	108.43	108.61	0.18
H4”-C4’-O4’	106.77	111.36	4.59
C1’-O4’-C4’	109.71	110.30	0.59

Figure 229: The PES scan for flexible dihedral corresponding to pcf.

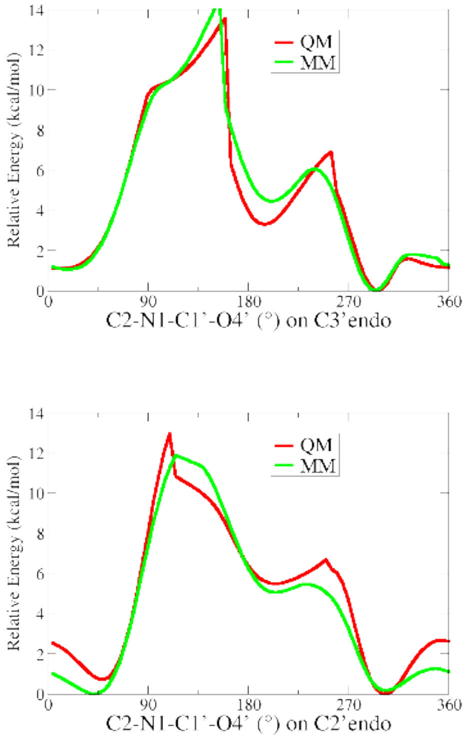

3.62 1-(tetrahydrofuran-2-yl) pseudouracil (pfu, for chi of PSU)

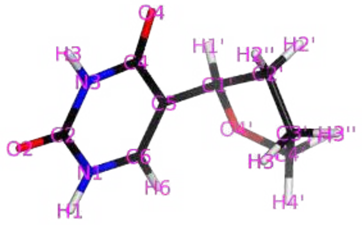

Figure 230: The energy-minimized structure of pfu.

Table 296: The calculated geometric terms of pfu.

Terms	QM	MM	diff
C5-C6	1.354	1.381	0.027
C5-C4	1.457	1.445	-0.013
C5-C1'	1.496	1.503	0.007
C6-H6	1.084	1.094	0.010
C6-N1	1.378	1.369	-0.008
N1-H1	1.013	1.006	-0.008
N1-C2	1.386	1.377	-0.008
C2-O2	1.225	1.223	-0.002
C2-N3	1.388	1.362	-0.026
N3-H3	1.017	1.000	-0.017
N3-C4	1.401	1.376	-0.025
C4-O4	1.232	1.229	-0.004
C1'-H1'	1.102	1.115	0.013
C1'-C2'	1.527	1.498	-0.029
C1'-O4'	1.435	1.422	-0.012
C2'-H2'	1.096	1.114	0.018
C2'-H2"	1.092	1.111	0.019
C2'-C3'	1.526	1.499	-0.028
C3'-H3'	1.093	1.112	0.018
C3'-H3"	1.095	1.111	0.016
C3'-C4'	1.531	1.520	-0.011
C4'-H4'	1.096	1.111	0.015
C4'-O4'	1.444	1.449	0.005
C4'-H4"	1.095	1.112	0.017
C6-C5-C4	119.38	117.78	-1.61
C6-C5-C1'	122.85	122.91	0.06
C4-C5-C1'	117.77	119.26	1.49
C5-C6-H6	121.61	119.76	-1.86
C5-C6-N1	121.63	122.25	0.62
H6-C6-N1	116.75	117.99	1.24
C6-N1-H1	120.87	124.92	4.05
C6-N1-C2	124.23	121.23	-3.00
H1-N1-C2	114.89	113.85	-1.04
N1-C2-O2	123.64	121.73	-1.91
N1-C2-N3	112.31	116.56	4.25
O2-C2-N3	124.05	121.71	-2.34
C2-N3-H3	115.40	117.28	1.89
C2-N3-C4	128.53	125.92	-2.61
H3-N3-C4	116.07	116.77	0.70
C5-C4-N3	113.92	116.22	2.30
C5-C4-O4	125.26	126.02	0.76
N3-C4-O4	120.83	117.76	-3.06
C5-C1'-H1'	108.95	110.26	1.31
C5-C1'-C2'	114.80	112.69	-2.11
C5-C1'-O4'	108.99	111.39	2.39
H1'-C1'-C2'	109.23	110.07	0.84
H1'-C1'-O4'	109.87	106.94	-2.93
C2'-C1'-O4'	104.90	105.25	0.35
C1'-C2'-H2'	109.03	110.00	0.97
C1'-C2'-H2"	112.08	113.49	1.41
C1'-C2'-C3'	100.95	100.66	-0.28
H2'-C2'-H2"	108.97	109.03	0.07
H2'-C2'-C3'	110.93	110.98	0.05
H2"-C2'-C3'	114.61	112.48	-2.13
C2'-C3'-H3'	113.56	112.00	-1.56
C2'-C3'-H3"	109.98	112.95	2.97
C2'-C3'-C4'	102.24	101.09	-1.15
H3'-C3'-H3"	107.96	110.56	2.60
H3'-C3'-C4'	112.50	109.02	-3.48
H3"-C3'-C4'	110.55	110.82	0.28
C3'-C4'-H4'	111.79	112.12	0.33
C3'-C4'-O4'	106.63	102.98	-3.65
C3'-C4'-H4"	113.08	110.17	-2.91
H4'-C4'-O4'	107.90	111.18	3.27
H4'-C4'-H4"	108.49	111.66	3.17
O4'-C4'-H4"	108.79	108.34	-0.44
C1'-O4'-C4'	109.12	109.81	0.68

Figure 231: The PES scan for flexible dihedral corresponding to pfu.

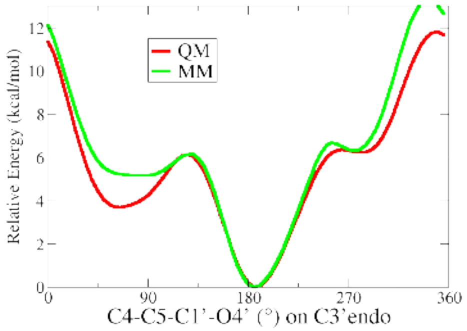

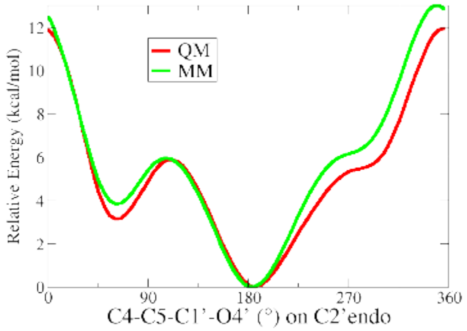

Supplement: Supplementary file 2 — Supporting Information [file JCC-37-896-s002.pdf]
