# Supplementary material for: Additive CHARMM force field for naturally occurring modified ribonucleotides
Source: J Comput Chem. 2016 Feb 3;37(10):896–912. doi: 10.1002/jcc.24307 (PMC4801715; doi:10.1002/jcc.24307)
Supplement: Supplementary file 3 — Supporting Information [file JCC-37-896-s003.pdf]

```
* Additive CHARMM Force Field for modified ribonucleic acids, including
* those founds in tRNA. You Xu, Karolinska Institutet, 2015.
* |
* WARNING: PARAMETERS AND ATOM TYPE ASSIGNMENTS IN THIS FILE ARE SPECIFIC
* FOR THESE COMPOUNDS AND SHOULD UNDER NO CIRCUMSTANCES BE APPLIED TO
* OTHER ORGANIC MOLECULES!!!
*

! Xu, Y., Vanommeslaeghe, K., Aleksandrov, A., MacKerell, A.D.,
! Jr. Nilsson, L., “Additive CHARMM force field for naturally
! occurring modified ribonucleotides,” Journal of Computational
!  Chemistry, In Press, 2015

! Should be used with parent files CGenFF: top_all36_cgenff.inp and
par_all36_cgenff.inp
!                                    NA36: top_all36_na.inp and par_all36_na.inp
!                              and Carb36: top_all36_carb.inp and par_all36_carb.inp
!

read rtf card append
* Topologies of modified nucleotides
*
36 1

MASS   413 SEGD1   78.96000  ! selenocarbonyl Se

DECL  +P
DECL  +O1P
DECL  +O2P
DECL  +O5'
DECL  -O3'

DEFA FIRS none LAST none
AUTOGENERATE ANGLES DIHEDRALS PATCH

!!!!!!!! ############################### !!!!!!!!
!!!!!!!! ######## Nucleic Acids ######## !!!!!!!!
!!!!!!!! ############################### !!!!!!!!

!!***Uridines***
RESI OMU           -1.00 ! 2'-O-methyluridine, MRU
GROUP
ATOM N1     NN2B   -0.34 !                  O4
ATOM C2     CN1T    0.55 !                  ||
ATOM O2     ON1    -0.45 !                  C4    H3
ATOM N3     NN2U   -0.46 !                 /  \  /
ATOM H3     HN2     0.36 !             H5-C5   N3
ATOM C4     CN1     0.53 !                ||   |
ATOM O4     ON1    -0.48 !             H6-C6   C2
ATOM C5     CN3    -0.15 !                 \  / \\
ATOM H5     HN3     0.10 !                  N1   O2
ATOM C6     CN3     0.20 !                   \
ATOM H6     HN3     0.14 !                    \
GROUP                    !                     \
ATOM P      P       1.50 ! O1P    H5' H4'  O4'  \
ATOM O1P    ON3    -0.78 !  |      |    \ /   \  \
ATOM O2P    ON3    -0.78 ! -P-O5'-C5'---C4'    C1'
ATOM O5'    ON2    -0.57 !  |      |     \     / \
ATOM C5'    CN8B   -0.08 ! O2P    H5''   C3'--C2' H1'
ATOM H5'    HN8     0.09 !               / \   / \
ATOM H5''   HN8     0.09 !            O3' H3' O2' H2''
GROUP                    !             |      |
ATOM C4'    CN7     0.16 !                    CM2
```

```
ATOM H4'    HN7      0.09 !                   /  |  \
ATOM O4'    ON6B   -0.50 !                 HM1 HM2 HM3
ATOM C1'    CN7B    0.16
ATOM H1'    HN7      0.09
GROUP
ATOM C2'    CN7B    0.08
ATOM H2''   HN7      0.09
ATOM O2'    OG301  -0.34
ATOM CM2    CG331  -0.10
ATOM HM1    HGA3    0.09
ATOM HM2    HGA3    0.09
ATOM HM3    HGA3    0.09
GROUP
ATOM C3'    CN7      0.01
ATOM H3'    HN7      0.09
ATOM O3'    ON2    -0.57
BOND N1   C2      N1   C6      C2   O2      C2   N3
BOND N3   C4      N3   H3      C4   O4      C4   C5
BOND C5   C6      C5   H5      C6   H6
BOND P    O1P     P    O2P     P    O5'     O5'  C5'     C5'  H5''
BOND C5'  C4'     C4'  O4'     C4'  C3'     O4'  C1'
BOND C1'  N1      C1'  C2'     C2'  C3'     C3'  O3'     O3'  +P
BOND C2'  O2'     CM2  O2'     CM2  HM1     HM2  CM2     HM3  CM2
BOND C1'  H1'     C2'  H2''    C3'  H3'     C4'  H4'     C5'  H5'
IMPR C2     N1     N3     O2      C4     N3     C5     O4
!2OM-ribose
IC -O3' P    O5'  C5'    1.6001  101.45  -39.25  119.00  1.4401
IC -O3' O5'  *P   O1P    1.6001  101.45 -115.82  109.74  1.4802
IC -O3' O5'  *P   O2P    1.6001  101.45  115.90  109.80  1.4801
IC  P   O5'  C5'  C4'    1.5996  119.00 -151.39  110.04  1.5160
IC O5'  C5'  C4'  C3'    1.4401  108.83 -179.85  116.10  1.5284
IC C5'  C4'  C3'  O3'    1.5160  116.10   76.70  115.12  1.4212
IC C4'  C3'  O3'  +P     1.5284  111.92  159.13  119.05  1.6001
IC C3'  O3'  +P   +O5'   1.4212  119.05  -98.86  101.45  1.5996
IC O4'  C3'  *C4' C5'    1.4572  104.06 -120.04  116.10  1.5160
IC C2'  C4'  *C3' O3'    1.5284  100.16 -124.08  115.12  1.4212
IC C4'  C3'  C2'  C1'    1.5284  100.16   39.58  102.04  1.5251
IC C1'  C3'  *C2' O2'    1.5284  102.04 -114.67  110.81  1.4212
IC H2'  O2'  C2'  C3'    0.9600  114.97  148.63  111.92  1.5284
IC C1'  C2'  O2'  CM2    1.5393  107.13   90.00  107.00  1.4150
IC C2'  O2'  CM2  HM2    1.4206  107.00  180.00    0.0   0.0
IC HM2  O2'  *CM2 HM3    0.0       0.0   120.00    0.0   0.0
IC HM2  O2'  *CM2 HM1    0.0       0.0  -120.00    0.0   0.0
IC O4'  C2'  *C1' H1'    0.0       0.0  -115.0     0.0   0.0
IC C1'  C3'  *C2' H2''   0.0       0.0   115.0     0.0   0.0
IC C2'  C4'  *C3' H3'    0.0       0.0   115.0     0.0   0.0
IC C3'  O4'  *C4' H4'    0.0       0.0  -115.0     0.0   0.0
IC C4'  O5'  *C5' H5'    0.0       0.0  -115.0     0.0   0.0
IC C4'  O5'  *C5' H5''   0.0       0.0   115.0     0.0   0.0
IC C3'  C2'  C1'  N1     1.5284  101.97  144.39  113.71  1.4896
IC O4'  C1'  N1   C2     1.5251  113.71  -96.0   117.06  1.3746
IC C1'  C2   *N1  C6     1.3966  121.57  179.97  122.49  1.4896
IC C2   C6   *N1  C1'       1.3966  121.57  179.97  122.49  1.4896
IC C2   N1   C6   C5        1.3746  121.57    0.06  122.01  1.3437
IC C5   N1   *C6  H6        1.3437  122.01  179.93  117.26  1.0925
IC C6   N1   C2   N3        1.3855  121.57   -0.06  114.90  1.3903
IC N3   N1   *C2  O2        1.3903  114.90 -179.93  123.17  1.2297
IC N1   C2   N3   C4        1.3966  114.90    0.06  126.93  1.4029
IC C4   C2   *N3  H3        1.4029  126.93  179.94  115.02  1.0026
IC C5   N3   *C4  O4        1.4519  113.66  179.98  120.79  1.2333
IC C4   C6   *C5  H5        1.4519  120.92 -179.99  118.61  1.0863
DONO H3   N3
ACCE O2   C2
```

```
ACCE O4   C4
ACCE O1P  P
ACCE O2P  P
ACCE O2'
ACCE O3'
ACCE O4'
ACCE O5'

RESI 2SU           -1.00 ! 2-thiouridine
GROUP
ATOM N1     NG2R61 -0.26 !                  O4
ATOM C2     CG2R63  0.31 !                  ||
ATOM S2     SG2D1  -0.22 !                  C4    H3
ATOM N3     NG2R61 -0.56 !                 /  \  /
ATOM H3     HGP1    0.40 !             H5-C5   N3
ATOM C4     CG2R63  0.41 !                ||    |
ATOM O4     OG2D4  -0.43 !             H6-C6   C2
ATOM C5     CG2R62 -0.25 !                 \  / \\
ATOM H5     HGR62   0.20 !                  N1   S2
ATOM C6     CG2R62  0.20 !                   \
ATOM H6     HGR62   0.20 !                    \
GROUP                    !                     \
ATOM P      P       1.50 ! O1P    H5' H4'  O4'  \
ATOM O1P    ON3    -0.78 !  |      |    \ /   \  \
ATOM O2P    ON3    -0.78 ! -P-O5'-C5'---C4'    C1'
ATOM O5'    ON2    -0.57 !  |      |     \     / \
ATOM C5'    CN8B   -0.08 ! O2P    H5''   C3'--C2' H1'
ATOM H5'    HN8     0.09 !              / \   / \
ATOM H5''   HN8     0.09 !           O3' H3' O2' H2''
GROUP                    !            |       |
ATOM C4'    CN7     0.16 !                   H2'
ATOM H4'    HN7     0.09
ATOM O4'    ON6B   -0.50
ATOM C1'    CN7B    0.16
ATOM H1'    HN7     0.09
GROUP
ATOM C2'    CN7B    0.14
ATOM H2''   HN7     0.09
ATOM O2'    ON5    -0.66
ATOM H2'    HN5     0.43
GROUP
ATOM C3'    CN7     0.01
ATOM H3'    HN7     0.09
ATOM O3'    ON2    -0.57
BOND N1   C2      N1   C6      C2   S2      C2   N3
BOND N3   C4      N3   H3      C4   O4      C4   C5
BOND C5   C6      C5   H5      C6   H6
BOND P    O1P     P    O2P     P    O5'     O5'  C5'     C5'  H5''
BOND C5'  C4'     C4'  O4'     C4'  C3'     O4'  C1'
BOND C1'  N1      C1'  C2'     C2'  C3'     C3'  O3'     O3'  +P
BOND C2'  O2'     O2'  H2'
BOND C1'  H1'     C2'  H2''    C3'  H3'     C4'  H4'     C5'  H5'
IMPR C2     N1     N3     S2     C4     C5     N3     O4
!ribose
IC -O3' P    O5'   C5'    1.6001  101.45  -39.25  119.00  1.4401
IC -O3' O5'  *P    O1P    1.6001  101.45 -115.82  109.74  1.4802
IC -O3' O5'  *P    O2P    1.6001  101.45  115.90  109.80  1.4801
IC  P   O5'  C5'   C4'    1.5996  119.00 -151.39  110.04  1.5160
IC O5'  C5'  C4'   C3'    1.4401  108.83 -179.85  116.10  1.5284
IC C5'  C4'  C3'   O3'    1.5160  116.10   76.70  115.12  1.4212
IC C4'  C3'  O3'   +P     1.5284  111.92  159.13  119.05  1.6001
IC C3'  O3'  +P    +O5'   1.4212  119.05  -98.86  101.45  1.5996
IC O4'  C3'  *C4'  C5'    1.4572  104.06 -120.04  116.10  1.5160
```

```
IC C2'  C4'  *C3'  O3'    1.5284  100.16 -124.08  115.12  1.4212
IC C4'  C3'  C2'   C1'    1.5284  100.16   39.58  102.04  1.5251
IC C1'  C3'  *C2'  O2'    1.5284  102.04 -114.67  110.81  1.4212
IC H2'  O2'  C2'   C3'    0.9600  114.97  148.63  111.92  1.5284
IC O4'  C2'  *C1'  H1'    0.0       0.0  -115.0     0.0   0.0
IC C1'  C3'  *C2'  H2''   0.0       0.0   115.0     0.0   0.0
IC C2'  C4'  *C3'  H3'    0.0       0.0   115.0     0.0   0.0
IC C3'  O4'  *C4'  H4'    0.0       0.0  -115.0     0.0   0.0
IC C4'  O5'  *C5'  H5'    0.0       0.0  -115.0     0.0   0.0
IC C4'  O5'  *C5'  H5''   0.0       0.0   115.0     0.0   0.0
IC C2   C6   *N1   C1'       1.3966  121.57  179.97  122.49  1.4896
IC C3'  C2'  C1'   N1     1.5284  101.97  144.39  113.71  1.4896
IC O4'  C1'  N1    C2     1.5251  113.71  -96.0   117.06  1.3746
IC C1'  C2   *N1   C6     1.3966  121.57  179.97  122.49  1.4896
IC C2   N1   C6    C5        1.3935  122.75    0.00  121.77  1.3714
IC C6   N1   C2    N3        1.3735  122.75    0.00  113.44  1.3851
IC N1   N3   *C2   S2        1.3935  113.44  180.00  123.40  1.6441
IC N1   C2   N3    C4        1.3935  113.44    0.00  127.42  1.3818
IC C5   N3   *C4   O4        1.4272  115.74  180.00  118.81  1.2288
IC C2   C4   *N3   H3        1.3851  127.42  180.00  114.32  1.0030
IC C6   C4   *C5   H5        1.3714  118.88  180.00  119.84  1.0857
IC N1   C5   *C6   H6        1.3735  121.77  180.00  120.43  1.0936
DONO H2'  O2'
DONO H3   N3
ACCE O4   C4
ACCE S2   C2
ACCE O1P  P
ACCE O2P  P
ACCE O2'
ACCE O3'
ACCE O4'
ACCE O5'

RESI MSU           -1.00 ! 2-thio-2'-O-methyluridine
GROUP
ATOM N1     NG2R61 -0.26 !                     O4
ATOM C2     CG2R63  0.31 !                     ||
ATOM S2     SG2D1  -0.22 !                     C4    H3
ATOM N3     NG2R61 -0.56 !                    /  \  /
ATOM H3     HGP1    0.40 !                H5-C5   N3
ATOM C4     CG2R63  0.41 !                   ||   |
ATOM O4     OG2D4  -0.43 !                H6-C6   C2
ATOM C5     CG2R62 -0.25 !                    \  / \\
ATOM H5     HGR62   0.20 !                     N1   S2
ATOM C6     CG2R62  0.20 !                      \
ATOM H6     HGR62   0.20 !                       \
GROUP                    !                        \
ATOM P      P       1.50 !   O1P    H5' H4'  O4'  \
ATOM O1P    ON3    -0.78 !    |      |    \ /   \  \
ATOM O2P    ON3    -0.78 !   -P-O5'-C5'---C4'    C1'
ATOM O5'    ON2    -0.57 !    |      |     \     / \
ATOM C5'    CN8B   -0.08 !   O2P    H5''   C3'--C2' H1'
ATOM H5'    HN8     0.09 !                 / \   / \
ATOM H5''   HN8     0.09 !              O3' H3' O2' H2''
GROUP                    !               |       |
ATOM C4'    CN7     0.16 !                      CM2
ATOM H4'    HN7     0.09 !                     /  |  \
ATOM O4'    ON6B   -0.50 !                  HM1 HM2 HM3
ATOM C1'    CN7B    0.16
ATOM H1'    HN7     0.09
GROUP
ATOM C2'    CN7B    0.08
ATOM H2''   HN7     0.09
```

```
ATOM O2'    OG301  -0.34
ATOM CM2    CG331  -0.10
ATOM HM1    HGA3    0.09
ATOM HM2    HGA3    0.09
ATOM HM3    HGA3    0.09
GROUP
ATOM C3'    CN7     0.01
ATOM H3'    HN7     0.09
ATOM O3'    ON2    -0.57
BOND N1   C2      N1   C6      C2   S2      C2   N3
BOND N3   C4      N3   H3      C4   O4      C4   C5
BOND C5   C6      C5   H5      C6   H6
BOND P    O1P     P    O2P     P    O5'     O5'  C5'     C5'  H5''
BOND C5'  C4'     C4'  O4'     C4'  C3'     O4'  C1'
BOND C1'  N1      C1'  C2'     C2'  C3'     C3'  O3'     O3'  +P
BOND C2'  O2'     CM2  O2'     CM2  HM1     HM2  CM2     HM3  CM2
BOND C1'  H1'     C2'  H2''    C3'  H3'     C4'  H4'     C5'  H5'
IMPR C2     N1     N3     S2     C4     C5     N3     O4
!2OM-ribose
IC -O3' P    O5'   C5'    1.6001  101.45  -39.25  119.00  1.4401
IC -O3' O5'  *P    O1P    1.6001  101.45 -115.82  109.74  1.4802
IC -O3' O5'  *P    O2P    1.6001  101.45  115.90  109.80  1.4801
IC  P   O5'  C5'   C4'    1.5996  119.00 -151.39  110.04  1.5160
IC O5'  C5'  C4'   C3'    1.4401  108.83 -179.85  116.10  1.5284
IC C5'  C4'  C3'   O3'    1.5160  116.10   76.70  115.12  1.4212
IC C4'  C3'  O3'   +P     1.5284  111.92  159.13  119.05  1.6001
IC C3'  O3'  +P    +O5'   1.4212  119.05  -98.86  101.45  1.5996
IC O4'  C3'  *C4'  C5'    1.4572  104.06 -120.04  116.10  1.5160
IC C2'  C4'  *C3'  O3'    1.5284  100.16 -124.08  115.12  1.4212
IC C4'  C3'  C2'   C1'    1.5284  100.16   39.58  102.04  1.5251
IC C3'  C2'  C1'   N1     1.5284  101.97  144.39  113.71  1.4896
IC O4'  C1'  N1    C2     1.5251  113.71  -96.0   117.06  1.3746
IC C3'  C1'  *C2'  O2'    1.5312  102.03  117.61  107.13  1.4206
IC C1'  C2'  O2'   CM2    1.5393  107.13   90.00  107.00  1.4150
IC C2'  O2'  CM2   HM2    1.4206  107.00  180.00    0.0   0.0
IC HM2  O2'  *CM2  HM3    0.0       0.0   120.00    0.0   0.0
IC HM2  O2'  *CM2  HM1    0.0       0.0  -120.00    0.0   0.0
IC O4'  C2'  *C1'  H1'    0.0       0.0  -115.0     0.0   0.0
IC C1'  C3'  *C2'  H2''   0.0       0.0   115.0     0.0   0.0
IC C2'  C4'  *C3'  H3'    0.0       0.0   115.0     0.0   0.0
IC C3'  O4'  *C4'  H4'    0.0       0.0  -115.0     0.0   0.0
IC C4'  O5'  *C5'  H5'    0.0       0.0  -115.0     0.0   0.0
IC C4'  O5'  *C5'  H5''   0.0       0.0   115.0     0.0   0.0
IC C2   C6   *N1   C1'       1.3966  121.57  179.97  122.49  1.4896
IC C3'  C2'  C1'   N1     1.5284  101.97  144.39  113.71  1.4896
IC O4'  C1'  N1    C2     1.5251  113.71  -96.0   117.06  1.3746
IC C1'  C2   *N1   C6     1.3966  121.57  179.97  122.49  1.4896
IC C2   N1   C6    C5        1.3935  122.75    0.00  121.77  1.3714
IC C6   N1   C2    N3        1.3735  122.75    0.00  113.44  1.3851
IC N1   N3   *C2   S2        1.3935  113.44  180.00  123.40  1.6441
IC N1   C2   N3    C4        1.3935  113.44    0.00  127.42  1.3818
IC C5   N3   *C4   O4        1.4272  115.74  180.00  118.81  1.2288
IC C2   C4   *N3   H3        1.3851  127.42  180.00  114.32  1.0030
IC C6   C4   *C5   H5        1.3714  118.88  180.00  119.84  1.0857
IC N1   C5   *C6   H6        1.3735  121.77  180.00  120.43  1.0936
DONO H3   N3
ACCE O4   C4
ACCE S2   C2
ACCE O1P  P
ACCE O2P  P
ACCE O2'
ACCE O3'
ACCE O4'
```

```
ACCE O5'

RESI 4SU            -1.00 ! 4-thiouridine
GROUP
ATOM N1      NG2R61 -0.20 !                  S4
ATOM C2      CG2R63  0.44 !                  ||
ATOM O2      OG2D4  -0.39 !                  C4    H3
ATOM N3      NG2R61 -0.64 !                 /  \  /
ATOM H3      HGP1    0.42 !             H5-C5   N3
ATOM C4      CG2R63  0.33 !                ||   |
ATOM S4      SG2D1  -0.25 !             H6-C6   C2
ATOM C5      CG2R62 -0.20 !                 \  / \\
ATOM H5      HGR62   0.13 !                  N1   O2
ATOM C6      CG2R62  0.15 !                   \
ATOM H6      HGR62   0.21 !                    \
GROUP                     !                     \
ATOM P       P       1.50 !  O1P    H5' H4'  O4'  \
ATOM O1P     ON3    -0.78 !   |      |    \ /   \  \
ATOM O2P     ON3    -0.78 !  -P-O5'-C5'---C4'    C1'
ATOM O5'     ON2    -0.57 !   |      |     \     / \
ATOM C5'     CN8B   -0.08 !  O2P    H5''   C3'--C2' H1'
ATOM H5'     HN8     0.09 !               / \   / \
ATOM H5''    HN8     0.09 !             O3' H3' O2' H2''
GROUP                     !              |       |
ATOM C4'     CN7     0.16 !                     H2'
ATOM H4'     HN7     0.09
ATOM O4'     ON6B   -0.50
ATOM C1'     CN7B    0.16
ATOM H1'     HN7     0.09
GROUP
ATOM C2'     CN7B    0.14
ATOM H2''    HN7     0.09
ATOM O2'     ON5    -0.66
ATOM H2'     HN5     0.43
GROUP
ATOM C3'     CN7     0.01
ATOM H3'     HN7     0.09
ATOM O3'     ON2    -0.57
BOND N1   C2      N1   C6      C2   O2      C2   N3
BOND N3   C4      N3   H3      C4   S4      C4   C5
BOND C5   C6      C5   H5      C6   H6
BOND P    O1P     P    O2P     P    O5'     O5'  C5'     C5'  H5''
BOND C5'  C4'     C4'  O4'     C4'  C3'     O4'  C1'
BOND C1'  N1      C1'  C2'     C2'  C3'     C3'  O3'     O3'  +P
BOND C2'  O2'     O2'  H2'
BOND C1'  H1'     C2'  H2''    C3'  H3'     C4'  H4'     C5'  H5'
IMPR C2      N1      N3      O2          C4      C5      N3      S4
!ribose
IC -O3' P    O5'   C5'    1.6001  101.45  -39.25  119.00  1.4401
IC -O3' O5'  *P    O1P    1.6001  101.45 -115.82  109.74  1.4802
IC -O3' O5'  *P    O2P    1.6001  101.45  115.90  109.80  1.4801
IC  P   O5'  C5'   C4'    1.5996  119.00 -151.39  110.04  1.5160
IC O5'  C5'  C4'   C3'    1.4401  108.83 -179.85  116.10  1.5284
IC C5'  C4'  C3'   O3'    1.5160  116.10   76.70  115.12  1.4212
IC C4'  C3'  O3'   +P     1.5284  111.92  159.13  119.05  1.6001
IC C3'  O3'  +P    +O5'   1.4212  119.05  -98.86  101.45  1.5996
IC O4'  C3'  *C4'  C5'    1.4572  104.06 -120.04  116.10  1.5160
IC C2'  C4'  *C3'  O3'    1.5284  100.16 -124.08  115.12  1.4212
IC C4'  C3'  C2'   C1'    1.5284  100.16   39.58  102.04  1.5251
IC C3'  C2'  C1'   N1     1.5284  101.97  144.39  113.71  1.4896
IC O4'  C1'  N1    C2     1.5251  113.71  -96.0   117.06  1.3746
IC C1'  C3'  *C2'  O2'    1.5284  102.04 -114.67  110.81  1.4212
IC H2'  O2'  C2'   C3'    0.9600  114.97  148.63  111.92  1.5284
```

```
IC O4'  C2'  *C1'  H1'    0.0       0.0  -115.0     0.0   0.0
IC C1'  C3'  *C2'  H2''   0.0       0.0   115.0     0.0   0.0
IC C2'  C4'  *C3'  H3'    0.0       0.0   115.0     0.0   0.0
IC C3'  O4'  *C4'  H4'    0.0       0.0  -115.0     0.0   0.0
IC C4'  O5'  *C5'  H5'    0.0       0.0  -115.0     0.0   0.0
IC C4'  O5'  *C5'  H5''   0.0       0.0   115.0     0.0   0.0
IC C3'  C2'  C1'   N1     1.5284  101.97  144.39  113.71  1.4896
IC O4'  C1'  N1    C2     1.5251  113.71  -96.0   117.06  1.3746
IC C1'  C2   *N1   C6     1.3966  121.57  179.97  122.49  1.4896
IC C2   C6   *N1   C1'       1.3966  121.57  179.97  122.49  1.4896
IC C6   N1   C2    N3        1.3687  121.40   -0.04  116.20  1.3702
IC N3   N1   *C2   O2        1.3702  116.20 -179.97  121.82  1.2241
IC N1   C2   N3    C4        1.3765  116.20    0.07  126.36  1.3930
IC C4   C2   *N3   H3        1.3930  126.36  179.94  114.82  1.0033
IC C2   N3   C4    S4        1.3702  126.36 -179.98  121.19  1.6451
IC S4   N3   *C4   C5        1.6451  121.19  179.90  114.76  1.4391
IC C6   C4   *C5   H5        1.3754  119.25  179.98  120.55  1.0880
IC C5   N1   *C6   H6        1.3754  122.03 -180.00  117.68  1.0936
DONO H3   N3
DONO H2'  O2'
ACCE O2   C2
ACCE S4   C4
ACCE O1P  P
ACCE O2P  P
ACCE O2'
ACCE O3'
ACCE O4'
ACCE O5'

RESI 52U           -1.00 ! 5-methyl-2-thiouridine
GROUP
ATOM N1     NG2R61 -0.26 !            H51   O4
ATOM C2     CG2R63  0.29 !             |    ||
ATOM S2     SG2D1  -0.22 !         H52-C5M  C4    H3
ATOM N3     NG2R61 -0.56 !            /  \ /  \  /
ATOM H3     HGP1    0.40 !          H53   C5   N3
ATOM C4     CG2R63  0.39 !                ||   |
ATOM O4     OG2D4  -0.41 !             H6-C6   C2
ATOM C5     CG2R62 -0.13 !                  \  / \\
ATOM C6     CG2R62  0.18 !                   N1   S2
ATOM H6     HGR62   0.22 !                    \
ATOM C5M    CG331  -0.17 !                     \
ATOM H51    HGA3    0.09 !                      \
ATOM H52    HGA3    0.09 ! O1P    H5' H4'  O4'  \
ATOM H53    HGA3    0.09 !  |      |    \ /   \  \
GROUP                    ! -P-O5'-C5'---C4'    C1'
ATOM P      P       1.50 !  |      |     \     / \
ATOM O1P    ON3    -0.78 ! O2P    H5''   C3'--C2' H1'
ATOM O2P    ON3    -0.78 !              / \   / \
ATOM O5'    ON2    -0.57 !            O3' H3' O2' H2''
ATOM C5'    CN8B   -0.08 !             |       |
ATOM H5'    HN8     0.09 !                    H2'
ATOM H5''   HN8     0.09
GROUP
ATOM C4'    CN7     0.16
ATOM H4'    HN7     0.09
ATOM O4'    ON6B   -0.50
ATOM C1'    CN7B    0.16
ATOM H1'    HN7     0.09
GROUP
ATOM C2'    CN7B    0.14
ATOM H2''   HN7     0.09
ATOM O2'    ON5    -0.66
```

```
ATOM H2'    HN5      0.43
GROUP
ATOM C3'    CN7      0.01
ATOM H3'    HN7      0.09
ATOM O3'    ON2     -0.57
BOND N1   C2   N1   C6   C2   S2   C2   N3
BOND N3   C4   N3   H3   C4   O4   C4   C5
BOND C5   C6   C5   C5M  C6   H6   C5M  H51
BOND C5M  H52  C5M  H53
BOND P    O1P     P    O2P     P    O5'     O5'  C5'     C5'  H5''
BOND C5'  C4'     C4'  O4'     C4'  C3'     O4'  C1'
BOND C1'  N1      C1'  C2'     C2'  C3'     C3'  O3'     O3'  +P
BOND C2'  O2'     O2'  H2'
BOND C1'  H1'     C2'  H2''    C3'  H3'     C4'  H4'     C5'  H5'
IMPR C2     N1     N3     S2     C4     C5     N3     O4
!ribose
IC -O3' P    O5'   C5'    1.6001  101.45  -39.25  119.00  1.4401
IC -O3' O5'  *P    O1P    1.6001  101.45 -115.82  109.74  1.4802
IC -O3' O5'  *P    O2P    1.6001  101.45  115.90  109.80  1.4801
IC  P   O5'  C5'   C4'    1.5996  119.00 -151.39  110.04  1.5160
IC O5'  C5'  C4'   C3'    1.4401  108.83 -179.85  116.10  1.5284
IC C5'  C4'  C3'   O3'    1.5160  116.10   76.70  115.12  1.4212
IC C4'  C3'  O3'   +P     1.5284  111.92  159.13  119.05  1.6001
IC C3'  O3'  +P    +O5'   1.4212  119.05  -98.86  101.45  1.5996
IC O4'  C3'  *C4'  C5'    1.4572  104.06 -120.04  116.10  1.5160
IC C2'  C4'  *C3'  O3'    1.5284  100.16 -124.08  115.12  1.4212
IC C4'  C3'  C2'   C1'    1.5284  100.16   39.58  102.04  1.5251
IC C3'  C2'  C1'   N1     1.5284  101.97  144.39  113.71  1.4896
IC O4'  C1'  N1    C2     1.5251  113.71  -96.0   117.06  1.3746
IC C1'  C3'  *C2'  O2'    1.5284  102.04 -114.67  110.81  1.4212
IC H2'  O2'  C2'   C3'    0.9600  114.97  148.63  111.92  1.5284
IC O4'  C2'  *C1'  H1'    0.0       0.0  -115.0     0.0   0.0
IC C1'  C3'  *C2'  H2''   0.0       0.0   115.0     0.0   0.0
IC C2'  C4'  *C3'  H3'    0.0       0.0   115.0     0.0   0.0
IC C3'  O4'  *C4'  H4'    0.0       0.0  -115.0     0.0   0.0
IC C4'  O5'  *C5'  H5'    0.0       0.0  -115.0     0.0   0.0
IC C4'  O5'  *C5'  H5''   0.0       0.0   115.0     0.0   0.0
IC C2   C6   *N1   C1'       1.3966  121.57  179.97  122.49  1.4896
IC C3'  C2'  C1'   N1     1.5284  101.97  144.39  113.71  1.4896
IC O4'  C1'  N1    C2     1.5251  113.71  -96.0   117.06  1.3746
IC C1'  C2   *N1   C6     1.3966  121.57  179.97  122.49  1.4896
IC C6   N1   C2    N3        1.3739  121.45   -0.83  113.48  1.3934
IC N3   N1   *C2   S2        1.3934  113.48 -179.53  125.70  1.6609
IC N1   C2   N3    C4        1.4163  113.48   -0.27  127.68  1.3841
IC C4   C2   *N3   H3        1.3841  127.68 -177.67  118.21  1.0051
IC C2   N3   C4    C5        1.3934  127.68    3.27  115.90  1.4431
IC C5   N3   *C4   O4        1.4431  115.90  179.82  118.51  1.2307
IC C5   N1   *C6   H6        1.3751  123.54  179.26  115.91  1.0889
IC C6   C4   *C5   C5M       1.3751  117.76 -171.50  119.31  1.4999
IC C4   C5   C5M   H51       1.4431  119.31 -155.47  111.71  1.1119
IC H51  C5   *C5M  H52       1.1119  111.71  119.96  110.99  1.1136
IC H51  C5   *C5M  H53       1.1119  111.71 -120.72  110.71  1.1137
DONO H2'  O2'
DONO H3   N3
ACCE O4   C4
ACCE S2   C2
ACCE O1P  P
ACCE O2P  P
ACCE O2'
ACCE O3'
ACCE O4'
ACCE O5'
```

```
RESI 5MU           -1.00 ! 5-Methyluridine, 38T
GROUP
ATOM N1     NN2B   -0.34 !              H51    O4
ATOM C2     CN1T    0.51 !               |     ||
ATOM O2     ON1    -0.41 !          H52-C5M   C4    H3
ATOM N3     NN2U   -0.46 !               |  \ /  \  /
ATOM H3     HN2     0.36 !              H53  C5   N3
ATOM C4     CN1     0.50 !                   ||   |
ATOM O4     ON1    -0.45 !                H6-C6   C2
ATOM C5     CN3T   -0.15 !                    \  / \\
ATOM C6     CN3     0.17 !                     N1   O2
ATOM H6     HN3     0.17 !                      \
ATOM C5M    CN9    -0.11 !                       \
ATOM H51    HN9     0.07 !                        \
ATOM H52    HN9     0.07 !    O1P    H5' H4'  O4'  \
ATOM H53    HN9     0.07 !     |      |    \ /   \  \
GROUP                    !    -P-O5'-C5'---C4'    C1'
ATOM P      P       1.50 !     |      |     \     / \
ATOM O1P    ON3    -0.78 !    O2P    H5''   C3'--C2' H1'
ATOM O2P    ON3    -0.78 !                 / \   / \
ATOM O5'    ON2    -0.57 !               O3' H3' O2' H2''
ATOM C5'    CN8B   -0.08 !                |       |
ATOM H5'    HN8     0.09 !                       H2'
ATOM H5''   HN8     0.09
GROUP
ATOM C4'    CN7     0.16
ATOM H4'    HN7     0.09
ATOM O4'    ON6B   -0.50
ATOM C1'    CN7B    0.16
ATOM H1'    HN7     0.09
GROUP
ATOM C2'    CN7B    0.14
ATOM H2''   HN7     0.09
ATOM O2'    ON5    -0.66
ATOM H2'    HN5     0.43
GROUP
ATOM C3'    CN7     0.01
ATOM H3'    HN7     0.09
ATOM O3'    ON2    -0.57
BOND N1   C2      N1   C6      C6   H6      C2   O2      C4   O4
BOND C2   N3      N3   H3      N3   C4      C4   C5      C5   C6
BOND C5   C5M     C5M  H51     C5M  H52     C5M  H53
BOND P    O1P     P    O2P     P    O5'     O5'  C5'     C5'  H5''
BOND C5'  C4'     C4'  O4'     C4'  C3'     O4'  C1'
BOND C1'  N1      C1'  C2'     C2'  C3'     C3'  O3'     O3'  +P
BOND C2'  O2'     O2'  H2'
BOND C1'  H1'     C2'  H2''    C3'  H3'     C4'  H4'     C5'  H5'
IMPR C2   N1   N3   O2        C4   C5   N3   O4        C5   C4   C6   C5M
!ribose
IC -O3' P    O5'   C5'    1.6001  101.45  -39.25  119.00  1.4401
IC -O3' O5'  *P    O1P    1.6001  101.45 -115.82  109.74  1.4802
IC -O3' O5'  *P    O2P    1.6001  101.45  115.90  109.80  1.4801
IC  P   O5'  C5'   C4'    1.5996  119.00 -151.39  110.04  1.5160
IC O5'  C5'  C4'   C3'    1.4401  108.83 -179.85  116.10  1.5284
IC C5'  C4'  C3'   O3'    1.5160  116.10   76.70  115.12  1.4212
IC C4'  C3'  O3'   +P     1.5284  111.92  159.13  119.05  1.6001
IC C3'  O3'  +P    +O5'   1.4212  119.05  -98.86  101.45  1.5996
IC O4'  C3'  *C4'  C5'    1.4572  104.06 -120.04  116.10  1.5160
IC C2'  C4'  *C3'  O3'    1.5284  100.16 -124.08  115.12  1.4212
IC C4'  C3'  C2'   C1'    1.5284  100.16   39.58  102.04  1.5251
IC C3'  C2'  C1'   N1     1.5284  101.97  144.39  113.71  1.4896
IC O4'  C1'  N1    C2     1.5251  113.71  -96.0   117.06  1.3746
IC C1'  C3'  *C2'  O2'    1.5284  102.04 -114.67  110.81  1.4212
```

```
IC H2'  O2'  C2'   C3'    0.9600  114.97  148.63  111.92  1.5284
IC O4'  C2'  *C1'  H1'    0.0       0.0  -115.0     0.0   0.0
IC C1'  C3'  *C2'  H2''   0.0       0.0   115.0     0.0   0.0
IC C2'  C4'  *C3'  H3'    0.0       0.0   115.0     0.0   0.0
IC C3'  O4'  *C4'  H4'    0.0       0.0  -115.0     0.0   0.0
IC C4'  O5'  *C5'  H5'    0.0       0.0  -115.0     0.0   0.0
IC C4'  O5'  *C5'  H5''   0.0       0.0   115.0     0.0   0.0
IC C2   C6   *N1   C1'       1.3966  121.57  179.97  122.49  1.4896
IC C3'  C2'  C1'   N1     1.5284  101.97  144.39  113.71  1.4896
IC O4'  C1'  N1    C2     1.5251  113.71  -96.0   117.06  1.3746
IC C1'  C2   *N1   C6     1.3966  121.57  179.97  122.49  1.4896
IC C2   N1   C6    C5        1.3870  121.43    2.52  123.43  1.3391
IC C5   N1   *C6   H6        1.3391  123.43  179.04  114.27  1.0910
IC C6   N1   C2    N3        1.3729  121.43   -0.25  114.35  1.3718
IC N3   N1   *C2   O2        1.3718  114.35 -179.99  123.11  1.2262
IC N1   C2   N3    C4        1.3870  114.35    0.14  127.12  1.3808
IC C4   C2   *N3   H3        1.3808  127.12  178.62  115.85  0.9993
IC C5   N3   *C4   O4        1.4439  115.35 -179.96  119.56  1.2290
IC C4   C6   *C5   C5M       1.4439  118.18 -171.70  122.58  1.4978
IC C6   C5   C5M   H51       1.3391  122.58   88.86  110.71  1.1125
IC H51  C5   *C5M  H52       1.1125  110.71  119.72  111.24  1.1134
IC H51  C5   *C5M  H53       1.1125  110.71 -120.42  111.48  1.1131
DONO H3   N3
DONO H2'  O2'
ACCE O2   C2
ACCE O4   C4
ACCE O1P  P
ACCE O2P  P
ACCE O2'
ACCE O3'
ACCE O4'
ACCE O5'

RESI 2MU           -1.00 ! 5,2'-O-dimethyluridine, MMU
GROUP
ATOM N1     NN2B   -0.34 !             H51    O4
ATOM C2     CN1T    0.51 !              |     ||
ATOM O2     ON1    -0.41 !          H52-C5M   C4    H3
ATOM N3     NN2U   -0.46 !              |  \ /  \  /
ATOM H3     HN2     0.36 !             H53  C5   N3
ATOM C4     CN1     0.50 !                  ||   |
ATOM O4     ON1    -0.45 !               H6-C6   C2
ATOM C5     CN3T   -0.15 !                    \  / \\
ATOM C6     CN3     0.17 !                    N1   O2
ATOM H6     HN3     0.17 !                      \
ATOM C5M    CN9    -0.11 !                       \
ATOM H51    HN9     0.07 !                        \
ATOM H52    HN9     0.07 !   O1P    H5' H4'  O4'   \
ATOM H53    HN9     0.07 !    |      |    \ /    \  \
GROUP                    !   -P-O5'-C5'---C4'    C1'
ATOM P      P       1.50 !    |      |     \      / \
ATOM O1P    ON3    -0.78 !   O2P    H5''   C3'--C2' H1'
ATOM O2P    ON3    -0.78 !                 / \   / \
ATOM O5'    ON2    -0.57 !              O3' H3' O2' H2''
ATOM C5'    CN8B   -0.08 !                |      |
ATOM H5'    HN8     0.09 !                     CM2
ATOM H5''   HN8     0.09 !                    /  |  \
GROUP                    !                 HM1 HM2 HM3
ATOM C4'    CN7     0.16
ATOM H4'    HN7     0.09
ATOM O4'    ON6B   -0.50
ATOM C1'    CN7B    0.16
ATOM H1'    HN7     0.09
```

```
GROUP
ATOM C2'    CN7B    0.08
ATOM H2''   HN7     0.09
ATOM O2'    OG301  -0.34
ATOM CM2    CG331  -0.10
ATOM HM1    HGA3    0.09
ATOM HM2    HGA3    0.09
ATOM HM3    HGA3    0.09
GROUP
ATOM C3'    CN7     0.01
ATOM H3'    HN7     0.09
ATOM O3'    ON2    -0.57
BOND N1   C2      N1   C6      C6   H6      C2   O2      C4   O4
BOND C2   N3      N3   H3      N3   C4      C4   C5      C5   C6
BOND C5   C5M     C5M  H51     C5M  H52     C5M  H53
BOND P    O1P     P    O2P     P    O5'     O5'  C5'     C5'  H5''
BOND C5'  C4'     C4'  O4'     C4'  C3'     O4'  C1'
BOND C1'  N1      C1'  C2'     C2'  C3'     C3'  O3'     O3'  +P
BOND C2'  O2'     CM2  O2'     CM2  HM1     HM2  CM2     HM3  CM2
BOND C1'  H1'     C2'  H2''    C3'  H3'     C4'  H4'     C5'  H5'
IMPR C2   N1   N3   O2        C4   C5   N3   O4        C5   C4   C6   C5M
!2OM-ribose
IC -O3' P    O5'   C5'    1.6001  101.45  -39.25  119.00  1.4401
IC -O3' O5'  *P    O1P    1.6001  101.45 -115.82  109.74  1.4802
IC -O3' O5'  *P    O2P    1.6001  101.45  115.90  109.80  1.4801
IC  P   O5'  C5'   C4'    1.5996  119.00 -151.39  110.04  1.5160
IC O5'  C5'  C4'   C3'    1.4401  108.83 -179.85  116.10  1.5284
IC C5'  C4'  C3'   O3'    1.5160  116.10   76.70  115.12  1.4212
IC C4'  C3'  O3'   +P     1.5284  111.92  159.13  119.05  1.6001
IC C3'  O3'  +P    +O5'   1.4212  119.05  -98.86  101.45  1.5996
IC O4'  C3'  *C4'  C5'    1.4572  104.06 -120.04  116.10  1.5160
IC C2'  C4'  *C3'  O3'    1.5284  100.16 -124.08  115.12  1.4212
IC C4'  C3'  C2'   C1'    1.5284  100.16   39.58  102.04  1.5251
IC C3'  C2'  C1'   N1     1.5284  101.97  144.39  113.71  1.4896
IC O4'  C1'  N1    C2     1.5251  113.71  -96.0   117.06  1.3746
IC C3'  C1'  *C2'  O2'    1.5312  102.03  117.61  107.13  1.4206
IC C1'  C2'  O2'   CM2    1.5393  107.13   90.00  107.00  1.4150
IC C2'  O2'  CM2   HM2    1.4206  107.00  180.00    0.0   0.0
IC HM2  O2'  *CM2  HM3    0.0       0.0   120.00    0.0   0.0
IC HM2  O2'  *CM2  HM1    0.0       0.0  -120.00    0.0   0.0
IC O4'  C2'  *C1'  H1'    0.0       0.0  -115.0     0.0   0.0
IC C1'  C3'  *C2'  H2''   0.0       0.0   115.0     0.0   0.0
IC C2'  C4'  *C3'  H3'    0.0       0.0   115.0     0.0   0.0
IC C3'  O4'  *C4'  H4'    0.0       0.0  -115.0     0.0   0.0
IC C4'  O5'  *C5'  H5'    0.0       0.0  -115.0     0.0   0.0
IC C4'  O5'  *C5'  H5''   0.0       0.0   115.0     0.0   0.0
IC C2   C6   *N1   C1'       1.3966  121.57  179.97  122.49  1.4896
IC C3'  C2'  C1'   N1     1.5284  101.97  144.39  113.71  1.4896
IC O4'  C1'  N1    C2     1.5251  113.71  -96.0   117.06  1.3746
IC C1'  C2   *N1   C6     1.3966  121.57  179.97  122.49  1.4896
IC C2   N1   C6    C5        1.3870  121.43    2.52  123.43  1.3391
IC C5   N1   *C6   H6        1.3391  123.43  179.04  114.27  1.0910
IC C6   N1   C2    N3        1.3729  121.43   -0.25  114.35  1.3718
IC N3   N1   *C2   O2        1.3718  114.35 -179.99  123.11  1.2262
IC N1   C2   N3    C4        1.3870  114.35    0.14  127.12  1.3808
IC C4   C2   *N3   H3        1.3808  127.12  178.62  115.85  0.9993
IC C5   N3   *C4   O4        1.4439  115.35 -179.96  119.56  1.2290
IC C4   C6   *C5   C5M       1.4439  118.18 -171.70  122.58  1.4978
IC C6   C5   C5M   H51       1.3391  122.58   88.86  110.71  1.1125
IC H51  C5   *C5M  H52       1.1125  110.71  119.72  111.24  1.1134
IC H51  C5   *C5M  H53       1.1125  110.71 -120.42  111.48  1.1131
DONO H3   N3
ACCE O2   C2
```

```
ACCE O4   C4
ACCE O1P  P
ACCE O2P  P
ACCE O2'
ACCE O3'
ACCE O4'
ACCE O5'

RESI H2U           -1.00 ! Dihydrouridine, DHU, D
GROUP
ATOM N1     NG2S0  -0.19 !                     O4
ATOM C2     CG2O6   0.32 !                     ||
ATOM O2     OG2D1  -0.42 !                H51  C4    H3
ATOM N3     NG2S1  -0.40 !                  \ /  \  /
ATOM H3     HGP1    0.32 !               H52-C5   N3
ATOM C4     CG2O1   0.55 !                   |    |
ATOM O4     OG2D1  -0.49 !               H61-C6   C2
ATOM C5     CG321  -0.14 !                  / \  / \\
ATOM H51    HGA2    0.09 !                H62  N1   O2
ATOM H52    HGA2    0.09 !                       \
ATOM C6     CG321   0.09 !                        \
ATOM H61    HGA2    0.09 !                         \
ATOM H62    HGA2    0.09 !    O1P    H5' H4'  O4'  \
GROUP                    !     |      |    \ /   \  \
ATOM P      P       1.50 !    -P-O5'-C5'---C4'    C1'
ATOM O1P    ON3    -0.78 !     |      |     \     / \
ATOM O2P    ON3    -0.78 !    O2P    H5''   C3'--C2' H1'
ATOM O5'    ON2    -0.57 !                  / \   / \
ATOM C5'    CN8B   -0.08 !                O3' H3' O2' H2''
ATOM H5'    HN8     0.09 !                 |       |
ATOM H5''   HN8     0.09 !                        H2'
GROUP
ATOM C4'    CN7     0.16
ATOM H4'    HN7     0.09
ATOM O4'    ON6B   -0.50
ATOM C1'    CN7B    0.16
ATOM H1'    HN7     0.09
GROUP
ATOM C2'    CN7B    0.14
ATOM H2''   HN7     0.09
ATOM O2'    ON5    -0.66
ATOM H2'    HN5     0.43
GROUP
ATOM C3'    CN7     0.01
ATOM H3'    HN7     0.09
ATOM O3'    ON2    -0.57
BOND N1   C2      N1   C6      C2   O2      C2   N3
BOND N3   C4      N3   H3      C4   O4      C4   C5
BOND C5   C6      C5   H51     C5   H52     C6   H61     C6   H62
BOND P    O1P     P    O2P     P    O5'     O5'  C5'     C5'  H5''
BOND C5'  C4'     C4'  O4'     C4'  C3'     O4'  C1'
BOND C1'  N1      C1'  C2'     C2'  C3'     C3'  O3'     O3'  +P
BOND C2'  O2'     O2'  H2'
BOND C1'  H1'     C2'  H2''    C3'  H3'     C4'  H4'     C5'  H5'
IMPR C2     N1     N3     O2      C4     C5     N3     O4
!ribose
IC -O3' P    O5'   C5'    1.6001  101.45  -39.25  119.00  1.4401
IC -O3' O5'  *P    O1P    1.6001  101.45 -115.82  109.74  1.4802
IC -O3' O5'  *P    O2P    1.6001  101.45  115.90  109.80  1.4801
IC  P   O5'  C5'   C4'    1.5996  119.00 -151.39  110.04  1.5160
IC O5'  C5'  C4'   C3'    1.4401  108.83 -179.85  116.10  1.5284
IC C5'  C4'  C3'   O3'    1.5160  116.10   76.70  115.12  1.4212
IC C4'  C3'  O3'   +P     1.5284  111.92  159.13  119.05  1.6001
```

```
IC C3'  O3'  +P    +O5'   1.4212  119.05  -98.86  101.45  1.5996
IC O4'  C3'  *C4'  C5'    1.4572  104.06 -120.04  116.10  1.5160
IC C2'  C4'  *C3'  O3'    1.5284  100.16 -124.08  115.12  1.4212
IC C4'  C3'  C2'   C1'    1.5284  100.16   39.58  102.04  1.5251
IC C3'  C2'  C1'   N1     1.5284  101.97  144.39  113.71  1.4711
IC C6   N1   C1'   C2'    1.4585  122.65  -92.08  112.86  1.5297
IC C1'  C3'  *C2'  O2'    1.5284  102.04 -114.67  110.81  1.4212
IC H2'  O2'  C2'   C3'    0.9600  114.97  148.63  111.92  1.5284
IC O4'  C2'  *C1'  H1'    0.0       0.0  -115.0     0.0   0.0
IC C1'  C3'  *C2'  H2''   0.0       0.0   115.0     0.0   0.0
IC C2'  C4'  *C3'  H3'    0.0       0.0   115.0     0.0   0.0
IC C3'  O4'  *C4'  H4'    0.0       0.0  -115.0     0.0   0.0
IC C4'  O5'  *C5'  H5'    0.0       0.0  -115.0     0.0   0.0
IC C4'  O5'  *C5'  H5''   0.0       0.0   115.0     0.0   0.0
IC C2   C6   *N1   C1'       1.3742  118.68  149.90  117.36  1.4557
IC C6   N1   C2    N3        1.4585  118.68  -19.15  114.34  1.4086
IC N3   N1   *C2   O2        1.4086  114.34 -179.13  125.27  1.2276
IC N1   C2   N3    C4        1.3659  114.34  -10.76  128.37  1.3869
IC C4   C2   *N3   H3        1.3869  128.37 -179.79  114.28  1.0167
IC C2   N3   C4    O4        1.4086  128.37 -174.61  121.81  1.2248
IC O4   N3   *C4   C5        1.2248  121.81  178.84  113.35  1.5100
IC C6   C4   *C5   H51       1.5203  110.81  123.16  108.13  1.0922
IC C6   C4   *C5   H52       1.5203  110.81 -120.07  107.95  1.0960
IC C5   N1   *C6   H61       1.5203  109.67 -122.40  111.00  1.1014
IC C5   N1   *C6   H62       1.5203  109.67  120.10  107.79  1.0939
DONO H2'  O2'
DONO H3   N3
ACCE O2   C2
ACCE O4   C4
ACCE O1P  P
ACCE O2P  P
ACCE O2'
ACCE O3'
ACCE O4'
ACCE O5'

RESI MDU           -1.00 ! 5-methyldihydrouridine, DMU
GROUP
ATOM N1     NG2S0  -0.19 !        H52 H53  O4
ATOM C2     CG2O6   0.32 !           \ |   ||
ATOM O2     OG2D1  -0.42 !        H51-C5M  C4    H3
ATOM N3     NG2S1  -0.40 !              \ /  \  /
ATOM H3     HGP1    0.32 !            H5-C5   N3
ATOM C4     CG2O1   0.55 !               |     |
ATOM O4     OG2D1  -0.49 !           H61-C6   C2
ATOM C5     CG311  -0.05 !              / \  / \\
ATOM H5     HGA1    0.09 !            H62  N1   O2
ATOM C6     CG321   0.09 !                  \
ATOM H61    HGA2    0.09 !                   \
ATOM H62    HGA2    0.09 !                    \
GROUP                    !                     \
ATOM C5M    CG331  -0.27 ! O1P    H5' H4'  O4'  \
ATOM H51    HGA3    0.09 !  |       |    \ /   \  \
ATOM H52    HGA3    0.09 ! -P-O5'-C5'---C4'    C1'
ATOM H53    HGA3    0.09 !  |       |     \     / \
GROUP                    ! O2P    H5''   C3'--C2' H1'
ATOM P      P       1.50 !              / \   / \
ATOM O1P    ON3    -0.78 !            O3' H3' O2' H2''
ATOM O2P    ON3    -0.78 !             |       |
ATOM O5'    ON2    -0.57 !                    H2'
ATOM C5'    CN8B   -0.08
ATOM H5'    HN8     0.09
ATOM H5''   HN8     0.09
```

```
GROUP
ATOM C4'    CN7     0.16
ATOM H4'    HN7     0.09
ATOM O4'    ON6B   -0.50
ATOM C1'    CN7B    0.16
ATOM H1'    HN7     0.09
GROUP
ATOM C2'    CN7B    0.14
ATOM H2''   HN7     0.09
ATOM O2'    ON5    -0.66
ATOM H2'    HN5     0.43
GROUP
ATOM C3'    CN7     0.01
ATOM H3'    HN7     0.09
ATOM O3'    ON2    -0.57
BOND N1   C2   N1   C6   C2   O2   C2   N3
BOND N3   C4   N3   H3   C4   O4   C4   C5
BOND C5   C6   C5   H5   C5   C5M  C6   H61
BOND C6   H62  C5M  H51  C5M  H52  C5M  H53
BOND P    O1P     P    O2P     P    O5'     O5'  C5'     C5'  H5''
BOND C5'  C4'     C4'  O4'     C4'  C3'     O4'  C1'
BOND C1'  N1      C1'  C2'     C2'  C3'     C3'  O3'     O3'  +P
BOND C2'  O2'     O2'  H2'
BOND C1'  H1'     C2'  H2''    C3'  H3'     C4'  H4'     C5'  H5'
IMPR C2     N1     N3     O2     C4     C5     N3     O4
!ribose
IC -O3' P    O5'   C5'    1.6001  101.45  -39.25  119.00  1.4401
IC -O3' O5'  *P    O1P    1.6001  101.45 -115.82  109.74  1.4802
IC -O3' O5'  *P    O2P    1.6001  101.45  115.90  109.80  1.4801
IC  P   O5'  C5'   C4'    1.5996  119.00 -151.39  110.04  1.5160
IC O5'  C5'  C4'   C3'    1.4401  108.83 -179.85  116.10  1.5284
IC C5'  C4'  C3'   O3'    1.5160  116.10   76.70  115.12  1.4212
IC C4'  C3'  O3'   +P     1.5284  111.92  159.13  119.05  1.6001
IC C3'  O3'  +P    +O5'   1.4212  119.05  -98.86  101.45  1.5996
IC O4'  C3'  *C4'  C5'    1.4572  104.06 -120.04  116.10  1.5160
IC C2'  C4'  *C3'  O3'    1.5284  100.16 -124.08  115.12  1.4212
IC C4'  C3'  C2'   C1'    1.5284  100.16   39.58  102.04  1.5251
IC C3'  C2'  C1'   N1     1.5284  101.97  144.39  113.71  1.4711
IC C6   N1   C1'   C2'    1.4585  122.65  -92.08  112.86  1.5297
IC C1'  C3'  *C2'  O2'    1.5284  102.04 -114.67  110.81  1.4212
IC H2'  O2'  C2'   C3'    0.9600  114.97  148.63  111.92  1.5284
IC O4'  C2'  *C1'  H1'    0.0       0.0  -115.0     0.0   0.0
IC C1'  C3'  *C2'  H2''   0.0       0.0   115.0     0.0   0.0
IC C2'  C4'  *C3'  H3'    0.0       0.0   115.0     0.0   0.0
IC C3'  O4'  *C4'  H4'    0.0       0.0  -115.0     0.0   0.0
IC C4'  O5'  *C5'  H5'    0.0       0.0  -115.0     0.0   0.0
IC C4'  O5'  *C5'  H5''   0.0       0.0   115.0     0.0   0.0
IC C2   C6   *N1   C1'       1.3742  118.68  149.90  117.36  1.4557
IC C6   N1   C2    N3        1.4585  118.68  -19.15  114.34  1.4086
IC N3   N1   *C2   O2        1.4086  114.34 -179.13  125.27  1.2276
IC N1   C2   N3    C4        1.3659  114.34  -10.76  128.37  1.3869
IC C4   C2   *N3   H3        1.3746  128.31 -172.71  114.62  0.9979
IC C2   N3   C4    C5        1.3894  128.31    1.31  114.48  1.5143
IC C5   N3   *C4   O4        1.5143  114.48 -178.52  121.13  1.1925
IC C6   C4   *C5   C5M       1.5253  109.83  125.75  111.56  1.5268
IC C6   C4   *C5   H5        1.5253  109.83 -115.69  105.40  1.0885
IC C5   N1   *C6   H61       1.5253  111.03 -123.02  110.99  1.0898
IC C5   N1   *C6   H62       1.5253  111.03  119.81  107.83  1.0823
IC C4   C5   C5M   H51       1.5143  111.56   55.84  110.51  1.0809
IC H51  C5   *C5M  H52       1.0809  110.51  120.13  110.28  1.0856
IC H51  C5   *C5M  H53       1.0809  110.51 -119.75  111.15  1.0839
DONO H2'  O2'
DONO H3   N3
```

```
ACCE O2   C2
ACCE O4   C4
ACCE O1P  P
ACCE O2P  P
ACCE O2'
ACCE O3'
ACCE O4'
ACCE O5'

RESI 5HU           -1.00 ! 5-hydroxyuridine     ! adjusted
GROUP
ATOM N1     NG2R61 -0.25 !             HO5  O4
ATOM C2     CG2R63  0.52 !              |   ||
ATOM O2     OG2D4  -0.43 !              O5  C4    H3
ATOM N3     NG2R61 -0.48 !               \ /  \  /
ATOM H3     HGP1    0.30 !                C5   N3
ATOM C4     CG2R63  0.55 !                ||   |
ATOM O4     OG2D4  -0.46 !             H6-C6   C2
ATOM C5     CG2R62  0.14 !                  \  / \\
ATOM O5     OG311  -0.60 !                   N1   O2
ATOM HO5    HGP1    0.39 !                    \
ATOM C6     CG2R62  0.17 !                     \
ATOM H6     HGR62   0.15 !                      \
GROUP                    !  O1P    H5' H4'  O4'  \
ATOM P      P       1.50 !   |      |    \ /   \  \
ATOM O1P    ON3    -0.78 !  -P-O5'-C5'---C4'    C1'
ATOM O2P    ON3    -0.78 !   |      |     \     / \
ATOM O5'    ON2    -0.57 !  O2P    H5''   C3'--C2' H1'
ATOM C5'    CN8B   -0.08 !               / \   / \
ATOM H5'    HN8     0.09 !             O3' H3' O2' H2''
ATOM H5''   HN8     0.09 !              |       |
GROUP                    !                     H2'
ATOM C4'    CN7     0.16
ATOM H4'    HN7     0.09
ATOM O4'    ON6B   -0.50
ATOM C1'    CN7B    0.16
ATOM H1'    HN7     0.09
GROUP
ATOM C2'    CN7B    0.14
ATOM H2''   HN7     0.09
ATOM O2'    ON5    -0.66
ATOM H2'    HN5     0.43
GROUP
ATOM C3'    CN7     0.01
ATOM H3'    HN7     0.09
ATOM O3'    ON2    -0.57
BOND N1   C2   N1   C6   C2   O2   C2   N3
BOND N3   C4   N3   H3   C4   O4   C4   C5
BOND C5   C6   C5   O5   O5   HO5  C6   H6
BOND P    O1P     P    O2P     P    O5'     O5'  C5'     C5'  H5''
BOND C5'  C4'     C4'  O4'     C4'  C3'     O4'  C1'
BOND C1'  N1      C1'  C2'     C2'  C3'     C3'  O3'     O3'  +P
BOND C2'  O2'     O2'  H2'
BOND C1'  H1'     C2'  H2''    C3'  H3'     C4'  H4'     C5'  H5'
IMPR C2     N1     N3     O2        C4     C5     N3     O4
!ribose
IC -O3' P    O5'   C5'    1.6001  101.45  -39.25  119.00  1.4401
IC -O3' O5'  *P    O1P    1.6001  101.45 -115.82  109.74  1.4802
IC -O3' O5'  *P    O2P    1.6001  101.45  115.90  109.80  1.4801
IC  P   O5'  C5'   C4'    1.5996  119.00 -151.39  110.04  1.5160
IC O5'  C5'  C4'   C3'    1.4401  108.83 -179.85  116.10  1.5284
IC C5'  C4'  C3'   O3'    1.5160  116.10   76.70  115.12  1.4212
IC C4'  C3'  O3'   +P     1.5284  111.92  159.13  119.05  1.6001
```

```
IC C3'  O3'  +P    +O5'   1.4212  119.05  -98.86  101.45  1.5996
IC O4'  C3'  *C4'  C5'    1.4572  104.06 -120.04  116.10  1.5160
IC C2'  C4'  *C3'  O3'    1.5284  100.16 -124.08  115.12  1.4212
IC C4'  C3'  C2'   C1'    1.5284  100.16   39.58  102.04  1.5251
IC C3'  C2'  C1'   N1     1.5284  101.97  144.39  113.71  1.4896
IC O4'  C1'  N1    C2     1.5251  113.71  -96.0   117.06  1.3746
IC C1'  C3'  *C2'  O2'    1.5284  102.04 -114.67  110.81  1.4212
IC H2'  O2'  C2'   C3'    0.9600  114.97  148.63  111.92  1.5284
IC O4'  C2'  *C1'  H1'    0.0       0.0  -115.0     0.0   0.0
IC C1'  C3'  *C2'  H2''   0.0       0.0   115.0     0.0   0.0
IC C2'  C4'  *C3'  H3'    0.0       0.0   115.0     0.0   0.0
IC C3'  O4'  *C4'  H4'    0.0       0.0  -115.0     0.0   0.0
IC C4'  O5'  *C5'  H5'    0.0       0.0  -115.0     0.0   0.0
IC C4'  O5'  *C5'  H5''   0.0       0.0   115.0     0.0   0.0
IC C2   C6   *N1   C1'       1.3966  121.57  179.97  122.49  1.4896
IC C3'  C2'  C1'   N1     1.5284  101.97  144.39  113.71  1.4896
IC O4'  C1'  N1    C2     1.5251  113.71  -96.0   117.06  1.3746
IC C1'  C2   *N1   C6     1.3966  121.57  179.97  122.49  1.4896
IC C6   N1   C2    N3        1.3716  121.77   -0.07  116.47  1.3604
IC N3   N1   *C2   O2        1.3604  116.47 -179.98  121.39  1.2212
IC N1   C2   N3    C4        1.3726  116.47    0.05  125.62  1.3824
IC C4   C2   *N3   H3        1.3824  125.62  179.93  116.97  1.0026
IC C2   N3   C4    C5        1.3604  125.62   -0.03  116.68  1.4650
IC C5   N3   *C4   O4        1.4650  116.68 -179.96  117.80  1.2334
IC C5   N1   *C6   H6        1.3743  122.84  179.95  117.93  1.0927
IC C6   C4   *C5   O5        1.3743  116.63 -179.98  118.48  1.3743
IC C4   C5   O5    HO5       1.4650  118.48   -0.03  106.43  0.9674
DONO H2'  O2'
DONO H3   N3
DONO HO5  O5
ACCE O5   C5
ACCE O2   C2
ACCE O4   C4
ACCE O1P  P
ACCE O2P  P
ACCE O2'
ACCE O3'
ACCE O4'
ACCE O5'

RESI MOU           -1.00 ! 5-methoxyuridine     ! adjusted
GROUP
ATOM N1     NG2R61 -0.29 !        H81 H82 H83
ATOM C2     CG2R63  0.53 !           \ | /
ATOM O2     OG2D4  -0.44 !            C8   O4
ATOM N3     NG2R61 -0.47 !             |   ||
ATOM H3     HGP1    0.33 !            O7   C4    H3
ATOM C4     CG2R63  0.46 !              \ /  \  /
ATOM O4     OG2D4  -0.46 !               C5   N3
ATOM C5     CG2R62  0.19 !               ||   |
ATOM C6     CG2R62  0.17 !            H6-C6   C2
ATOM H6     HGR62   0.16 !                \  / \\
ATOM O7     OG301  -0.43 !                 N1   O2
ATOM C8     CG331  -0.02 !                  \
ATOM H81    HGA3    0.09 !                   \
ATOM H82    HGA3    0.09 !                    \
ATOM H83    HGA3    0.09 ! O1P    H5' H4'  O4'  \
GROUP                    !  |      |    \ /   \  \
ATOM P      P       1.50 ! -P-O5'-C5'---C4'    C1'
ATOM O1P    ON3    -0.78 !  |      |     \     / \
ATOM O2P    ON3    -0.78 ! O2P    H5''   C3'--C2' H1'
ATOM O5'    ON2    -0.57 !              / \   / \
ATOM C5'    CN8B   -0.08 !            O3' H3' O2' H2''
```

```
ATOM H5'    HN8      0.09 !              |       |
ATOM H5''   HN8      0.09 !                     H2'
GROUP
ATOM C4'    CN7      0.16
ATOM H4'    HN7      0.09
ATOM O4'    ON6B    -0.50
ATOM C1'    CN7B     0.16
ATOM H1'    HN7      0.09
GROUP
ATOM C2'    CN7B     0.14
ATOM H2''   HN7      0.09
ATOM O2'    ON5     -0.66
ATOM H2'    HN5      0.43
GROUP
ATOM C3'    CN7      0.01
ATOM H3'    HN7      0.09
ATOM O3'    ON2     -0.57
BOND N1   C2   N1   C6   C2   O2   C2   N3
BOND N3   C4   N3   H3   C4   O4   C4   C5
BOND C5   C6   C5   O7   C6   H6   O7   C8
BOND C8   H81  C8   H82  C8   H83
BOND P    O1P     P    O2P     P    O5'     O5'  C5'     C5'  H5''
BOND C5'  C4'     C4'  O4'     C4'  C3'     O4'  C1'
BOND C1'  N1      C1'  C2'     C2'  C3'     C3'  O3'     O3'  +P
BOND C2'  O2'     O2'  H2'
BOND C1'  H1'     C2'  H2''    C3'  H3'     C4'  H4'     C5'  H5'
IMPR C2     N1     N3     O2     C4     C5     N3     O4
!ribose
IC -O3' P    O5'   C5'    1.6001  101.45  -39.25  119.00  1.4401
IC -O3' O5'  *P    O1P    1.6001  101.45 -115.82  109.74  1.4802
IC -O3' O5'  *P    O2P    1.6001  101.45  115.90  109.80  1.4801
IC  P   O5'  C5'   C4'    1.5996  119.00 -151.39  110.04  1.5160
IC O5'  C5'  C4'   C3'    1.4401  108.83 -179.85  116.10  1.5284
IC C5'  C4'  C3'   O3'    1.5160  116.10   76.70  115.12  1.4212
IC C4'  C3'  O3'   +P     1.5284  111.92  159.13  119.05  1.6001
IC C3'  O3'  +P    +O5'   1.4212  119.05  -98.86  101.45  1.5996
IC O4'  C3'  *C4'  C5'    1.4572  104.06 -120.04  116.10  1.5160
IC C2'  C4'  *C3'  O3'    1.5284  100.16 -124.08  115.12  1.4212
IC C4'  C3'  C2'   C1'    1.5284  100.16   39.58  102.04  1.5251
IC C3'  C2'  C1'   N1     1.5284  101.97  144.39  113.71  1.4896
IC O4'  C1'  N1    C2     1.5251  113.71  -96.0   117.06  1.3746
IC C1'  C3'  *C2'  O2'    1.5284  102.04 -114.67  110.81  1.4212
IC H2'  O2'  C2'   C3'    0.9600  114.97  148.63  111.92  1.5284
IC O4'  C2'  *C1'  H1'    0.0       0.0  -115.0     0.0   0.0
IC C1'  C3'  *C2'  H2''   0.0       0.0   115.0     0.0   0.0
IC C2'  C4'  *C3'  H3'    0.0       0.0   115.0     0.0   0.0
IC C3'  O4'  *C4'  H4'    0.0       0.0  -115.0     0.0   0.0
IC C4'  O5'  *C5'  H5'    0.0       0.0  -115.0     0.0   0.0
IC C4'  O5'  *C5'  H5''   0.0       0.0   115.0     0.0   0.0
IC C2   C6   *N1   C1'       1.3966  121.57  179.97  122.49  1.4896
IC C3'  C2'  C1'   N1     1.5284  101.97  144.39  113.71  1.4896
IC O4'  C1'  N1    C2     1.5251  113.71  -96.0   117.06  1.3746
IC C1'  C2   *N1   C6     1.3966  121.57  179.97  122.49  1.4896
IC C6   N1   C2    N3        1.3881  122.08    6.31  113.57  1.3863
IC N3   N1   *C2   O2        1.3863  113.57  178.37  123.87  1.2422
IC N1   C2   N3    C4        1.3745  113.57   -9.83  129.06  1.4100
IC C4   C2   *N3   H3        1.4100  129.06 -179.32  114.78  1.0184
IC C2   N3   C4    C5        1.3863  129.06    8.65  112.36  1.4599
IC C5   N3   *C4   O4        1.4599  112.36  178.60  119.90  1.2352
IC C5   N1   *C6   H6        1.3637  122.82 -178.63  118.66  1.0908
IC C6   C4   *C5   O7        1.3637  119.46  179.91  117.75  1.3607
IC C4   C5   O7    C8        1.4599  117.75  116.30  115.35  1.4567
IC C5   O7   C8    H83       1.3607  115.35   77.36  109.53  1.0701
```

```
IC H83  O7   *C8   H82       1.0701  109.53  119.97  109.49  1.0703
IC H83  O7   *C8   H81       1.0701  109.53 -120.00  109.51  1.0700
DONO H2'  O2'
DONO H3   N3
ACCE O2   C2
ACCE O4   C4
ACCE O7
ACCE O1P  P
ACCE O2P  P
ACCE O2'
ACCE O3'
ACCE O4'
ACCE O5'

RESI 3MU           -1.00 ! 3-methyluridine, UR3
GROUP
ATOM N1     NG2R61 -0.20 !                     O4       H31
ATOM C2     CG2R63  0.52 !                     ||      /
ATOM O2     OG2D4  -0.44 !                     C4    C3U-H32
ATOM N3     NG2R61 -0.32 !                    /  \  / \
ATOM C4     CG2R63  0.50 !                H5-C5   N3   H33
ATOM O4     OG2D4  -0.48 !                   ||    |
ATOM C5     CG2R62 -0.22 !                H6-C6   C2
ATOM H5     HGR62   0.09 !                    \  / \\
ATOM C6     CG2R62  0.31 !                     N1   O2
ATOM H6     HGR62   0.11 !                      \
ATOM C3U    CG331  -0.14 !                       \
ATOM H31    HGA3    0.09 !                        \
ATOM H32    HGA3    0.09 !   O1P    H5' H4'  O4'  \
ATOM H33    HGA3    0.09 !    |      |    \ /   \  \
GROUP                    !   -P-O5'-C5'---C4'    C1'
ATOM P      P       1.50 !    |      |     \     / \
ATOM O1P    ON3    -0.78 !   O2P    H5''   C3'--C2' H1'
ATOM O2P    ON3    -0.78 !                /  \   / \
ATOM O5'    ON2    -0.57 !              O3' H3' O2' H2''
ATOM C5'    CN8B   -0.08 !               |       |
ATOM H5'    HN8     0.09 !                      H2'
ATOM H5''   HN8     0.09
GROUP
ATOM C4'    CN7     0.16
ATOM H4'    HN7     0.09
ATOM O4'    ON6B   -0.50
ATOM C1'    CN7B    0.16
ATOM H1'    HN7     0.09
GROUP
ATOM C2'    CN7B    0.14
ATOM H2''   HN7     0.09
ATOM O2'    ON5    -0.66
ATOM H2'    HN5     0.43
GROUP
ATOM C3'    CN7     0.01
ATOM H3'    HN7     0.09
ATOM O3'    ON2    -0.57
BOND N1   C2      N1   C6      C2   O2      C2   N3
BOND N3   C4      N3   C3U     C4   O4      C4   C5
BOND C5   C6      C5   H5      C6   H6      C3U  H31
BOND C3U  H32     C3U  H33
BOND P    O1P     P    O2P     P    O5'     O5'  C5'     C5'  H5''
BOND C5'  C4'     C4'  O4'     C4'  C3'     O4'  C1'
BOND C1'  N1      C1'  C2'     C2'  C3'     C3'  O3'     O3'  +P
BOND C2'  O2'     O2'  H2'
BOND C1'  H1'     C2'  H2''    C3'  H3'     C4'  H4'     C5'  H5'
IMPR C2     N1     N3     O2      C4     C5     N3     O4
```

```
!ribose
IC -O3' P    O5'   C5'    1.6001  101.45  -39.25  119.00  1.4401
IC -O3' O5'  *P    O1P    1.6001  101.45 -115.82  109.74  1.4802
IC -O3' O5'  *P    O2P    1.6001  101.45  115.90  109.80  1.4801
IC  P   O5'  C5'   C4'    1.5996  119.00 -151.39  110.04  1.5160
IC O5'  C5'  C4'   C3'    1.4401  108.83 -179.85  116.10  1.5284
IC C5'  C4'  C3'   O3'    1.5160  116.10   76.70  115.12  1.4212
IC C4'  C3'  O3'   +P     1.5284  111.92  159.13  119.05  1.6001
IC C3'  O3'  +P    +O5'   1.4212  119.05  -98.86  101.45  1.5996
IC O4'  C3'  *C4'  C5'    1.4572  104.06 -120.04  116.10  1.5160
IC C2'  C4'  *C3'  O3'    1.5284  100.16 -124.08  115.12  1.4212
IC C4'  C3'  C2'   C1'    1.5284  100.16   39.58  102.04  1.5251
IC C3'  C2'  C1'   N1     1.5284  101.97  144.39  113.71  1.4896
IC O4'  C1'  N1    C2     1.5251  113.71  -96.0   117.06  1.3746
IC C1'  C3'  *C2'  O2'    1.5284  102.04 -114.67  110.81  1.4212
IC H2'  O2'  C2'   C3'    0.9600  114.97  148.63  111.92  1.5284
IC O4'  C2'  *C1'  H1'    0.0       0.0  -115.0     0.0   0.0
IC C1'  C3'  *C2'  H2''   0.0       0.0   115.0     0.0   0.0
IC C2'  C4'  *C3'  H3'    0.0       0.0   115.0     0.0   0.0
IC C3'  O4'  *C4'  H4'    0.0       0.0  -115.0     0.0   0.0
IC C4'  O5'  *C5'  H5'    0.0       0.0  -115.0     0.0   0.0
IC C4'  O5'  *C5'  H5''   0.0       0.0   115.0     0.0   0.0
IC C2   C6   *N1   C1'       1.3966  121.57  179.97  122.49  1.4896
IC C3'  C2'  C1'   N1     1.5284  101.97  144.39  113.71  1.4896
IC O4'  C1'  N1    C2     1.5251  113.71  -96.0   117.06  1.3746
IC C1'  C2   *N1   C6     1.3966  121.57  179.97  122.49  1.4896
IC C6   N1   C2    N3        1.3618  119.90   -1.45  117.07  1.3999
IC N3   N1   *C2   O2        1.3999  117.07  179.83  121.38  1.2329
IC N1   C2   N3    C4        1.4086  117.07    3.31  123.57  1.4040
IC C4   C2   *N3   C3U       1.4040  123.57  175.75  118.18  1.4799
IC C2   N3   C4    O4        1.3999  123.57  176.21  120.08  1.2299
IC O4   N3   *C4   C5        1.2299  120.08 -179.93  116.52  1.4263
IC C6   C4   *C5   H5        1.3662  119.41  178.31  120.24  1.0881
IC C5   N1   *C6   H6        1.3662  123.45 -179.85  116.15  1.0904
IC C2   N3   C3U   H31       1.3999  118.18  -90.44  110.86  1.1144
IC H31  N3   *C3U  H32       1.1144  110.86  120.27  112.50  1.1137
IC H31  N3   *C3U  H33       1.1144  110.86 -119.94  112.16  1.1142
DONO H2'  O2'
ACCE O2   C2
ACCE O4   C4
ACCE O1P  P
ACCE O2P  P
ACCE O2'
ACCE O3'
ACCE O4'
ACCE O5'

RESI M3U           -1.00 ! 3,2'-O-dimethyluridine
GROUP
ATOM N1     NG2R61 -0.20 !                    O4      H31
ATOM C2     CG2R63  0.52 !                    ||      /
ATOM O2     OG2D4  -0.44 !                    C4    C3U-H32
ATOM N3     NG2R61 -0.32 !                   /  \  / \
ATOM C4     CG2R63  0.50 !               H5-C5   N3   H33
ATOM O4     OG2D4  -0.48 !                  ||   |
ATOM C5     CG2R62 -0.22 !               H6-C6   C2
ATOM H5     HGR62   0.09 !                   \  / \\
ATOM C6     CG2R62  0.31 !                    N1   O2
ATOM H6     HGR62   0.11 !                     \
ATOM C3U    CG331  -0.14 !                      \
ATOM H31    HGA3    0.09 !                       \
ATOM H32    HGA3    0.09 !   O1P    H5' H4'  O4'  \
ATOM H33    HGA3    0.09 !    |       |    \ /   \  \
```

```
GROUP                     !   -P-O5'-C5'---C4'    C1'
ATOM P      P       1.50 !    |      |      \     / \
ATOM O1P    ON3    -0.78 !   O2P    H5''   C3'--C2' H1'
ATOM O2P    ON3    -0.78 !                 / \   / \
ATOM O5'    ON2    -0.57 !              O3' H3' O2' H2''
ATOM C5'    CN8B   -0.08 !               |       |
ATOM H5'    HN8     0.09 !                      CM2
ATOM H5''   HN8     0.09 !                    /  |  \
GROUP                     !                  HM1 HM2 HM3
ATOM C4'    CN7     0.16
ATOM H4'    HN7     0.09
ATOM O4'    ON6B   -0.50
ATOM C1'    CN7B    0.16
ATOM H1'    HN7     0.09
GROUP
ATOM C2'    CN7B    0.08
ATOM H2''   HN7     0.09
ATOM O2'    OG301  -0.34
ATOM CM2    CG331  -0.10
ATOM HM1    HGA3    0.09
ATOM HM2    HGA3    0.09
ATOM HM3    HGA3    0.09
GROUP
ATOM C3'    CN7     0.01
ATOM H3'    HN7     0.09
ATOM O3'    ON2    -0.57
BOND N1   C2      N1   C6      C2   O2      C2   N3
BOND N3   C4      N3   C3U     C4   O4      C4   C5
BOND C5   C6      C5   H5      C6   H6      C3U  H31
BOND C3U  H32     C3U  H33
BOND P    O1P     P    O2P     P    O5'     O5'  C5'     C5'  H5''
BOND C5'  C4'     C4'  O4'     C4'  C3'     O4'  C1'
BOND C1'  N1      C1'  C2'     C2'  C3'     C3'  O3'     O3'  +P
BOND C2'  O2'     CM2  O2'     CM2  HM1     HM2  CM2     HM3  CM2
BOND C1'  H1'     C2'  H2''    C3'  H3'     C4'  H4'     C5'  H5'
IMPR C2     N1     N3     O2     C4     C5     N3     O4
!2OM-ribose
IC -O3' P    O5'  C5'    1.6001  101.45  -39.25  119.00  1.4401
IC -O3' O5'  *P   O1P    1.6001  101.45 -115.82  109.74  1.4802
IC -O3' O5'  *P   O2P    1.6001  101.45  115.90  109.80  1.4801
IC  P   O5'  C5'  C4'    1.5996  119.00 -151.39  110.04  1.5160
IC O5'  C5'  C4'  C3'    1.4401  108.83 -179.85  116.10  1.5284
IC C5'  C4'  C3'  O3'    1.5160  116.10   76.70  115.12  1.4212
IC C4'  C3'  O3'  +P     1.5284  111.92  159.13  119.05  1.6001
IC C3'  O3'  +P   +O5'   1.4212  119.05  -98.86  101.45  1.5996
IC O4'  C3'  *C4' C5'    1.4572  104.06 -120.04  116.10  1.5160
IC C2'  C4'  *C3' O3'    1.5284  100.16 -124.08  115.12  1.4212
IC C4'  C3'  C2'  C1'    1.5284  100.16   39.58  102.04  1.5251
IC C3'  C2'  C1'  N1     1.5284  101.97  144.39  113.71  1.4896
IC O4'  C1'  N1   C2     1.5251  113.71  -96.0   117.06  1.3746
IC C3'  C1'  *C2' O2'    1.5312  102.03  117.61  107.13  1.4206
IC C1'  C2'  O2'  CM2    1.5393  107.13   90.00  107.00  1.4150
IC C2'  O2'  CM2  HM2    1.4206  107.00  180.00    0.0   0.0
IC HM2  O2'  *CM2 HM3    0.0       0.0   120.00    0.0   0.0
IC HM2  O2'  *CM2 HM1    0.0       0.0  -120.00    0.0   0.0
IC O4'  C2'  *C1' H1'    0.0       0.0  -115.0     0.0   0.0
IC C1'  C3'  *C2' H2''   0.0       0.0   115.0     0.0   0.0
IC C2'  C4'  *C3' H3'    0.0       0.0   115.0     0.0   0.0
IC C3'  O4'  *C4' H4'    0.0       0.0  -115.0     0.0   0.0
IC C4'  O5'  *C5' H5'    0.0       0.0  -115.0     0.0   0.0
IC C4'  O5'  *C5' H5''   0.0       0.0   115.0     0.0   0.0
IC C2   C6   *N1  C1'       1.3966  121.57  179.97  122.49  1.4896
IC C3'  C2'  C1'  N1     1.5284  101.97  144.39  113.71  1.4896
```

```
IC O4'  C1'  N1    C2      1.5251  113.71  -96.0   117.06  1.3746
IC C1'  C2   *N1   C6      1.3966  121.57  179.97  122.49  1.4896
IC C6   N1   C2    N3        1.3618  119.90   -1.45  117.07  1.3999
IC N3   N1   *C2   O2        1.3999  117.07  179.83  121.38  1.2329
IC N1   C2   N3    C4        1.4086  117.07    3.31  123.57  1.4040
IC C4   C2   *N3   C3U       1.4040  123.57  175.75  118.18  1.4799
IC C2   N3   C4    O4        1.3999  123.57  176.21  120.08  1.2299
IC O4   N3   *C4   C5        1.2299  120.08 -179.93  116.52  1.4263
IC C6   C4   *C5   H5        1.3662  119.41  178.31  120.24  1.0881
IC C5   N1   *C6   H6        1.3662  123.45 -179.85  116.15  1.0904
IC C2   N3   C3U   H31       1.3999  118.18  -90.44  110.86  1.1144
IC H31  N3   *C3U  H32       1.1144  110.86  120.27  112.50  1.1137
IC H31  N3   *C3U  H33       1.1144  110.86 -119.94  112.16  1.1142
ACCE O2   C2
ACCE O4   C4
ACCE O1P  P
ACCE O2P  P
ACCE O2'
ACCE O3'
ACCE O4'
ACCE O5'

RESI CYU           -1.00 ! 5-cyanomethyl-uridine
GROUP
ATOM N1     NG2R61 -0.34 !              H71    O4
ATOM C2     CG2R63  0.51 !          _    |      ||
ATOM O2     OG2D4  -0.41 !        N9= C8--C7\   C4    H3
ATOM N3     NG2R61 -0.46 !                |  \ /  \  /
ATOM H3     HGP1    0.36 !              H73  C5   N3
ATOM C4     CG2R63  0.50 !                   ||   |
ATOM O4     OG2D4  -0.45 !                H6-C6   C2
ATOM C5     CG2R62 -0.05 !                    \  / \\
ATOM C6     CG2R62  0.17 !                     N1   O2
ATOM H6     HGR62   0.17 !                      \
GROUP                    !                       \
ATOM C7     CG321  -0.08 !                        \
ATOM H71    HGA2    0.09 !    O1P    H5' H4'  O4'  \
ATOM H72    HGA2    0.09 !     |      |    \ /   \  \
ATOM C8     CG1N1   0.36 !    -P-O5'-C5'---C4'    C1'
ATOM N9     NG1T1  -0.46 !     |      |     \     / \
GROUP                    !    O2P    H5''   C3'--C2' H1'
ATOM P      P       1.50 !                 / \   / \
ATOM O1P    ON3    -0.78 !               O3' H3' O2' H2''
ATOM O2P    ON3    -0.78 !                |       |
ATOM O5'    ON2    -0.57 !                       H2'
ATOM C5'    CN8B   -0.08
ATOM H5'    HN8     0.09
ATOM H5''   HN8     0.09
GROUP
ATOM C4'    CN7     0.16
ATOM H4'    HN7     0.09
ATOM O4'    ON6B   -0.50
ATOM C1'    CN7B    0.16
ATOM H1'    HN7     0.09
GROUP
ATOM C2'    CN7B    0.14
ATOM H2''   HN7     0.09
ATOM O2'    ON5    -0.66
ATOM H2'    HN5     0.43
GROUP
ATOM C3'    CN7     0.01
ATOM H3'    HN7     0.09
ATOM O3'    ON2    -0.57
```

```
BOND N1   C2      N1   C6      C2   O2      C2   N3
BOND N3   H3      N3   C4      C4   O4      C4   C5
BOND C5   C6      C5   C7      C6   H6      C7   C8
BOND C7   H71     C7   H72     C8   N9
BOND P    O1P     P    O2P     P    O5'     O5'  C5'     C5'  H5''
BOND C5'  C4'     C4'  O4'     C4'  C3'     O4'  C1'
BOND C1'  N1      C1'  C2'     C2'  C3'     C3'  O3'     O3'  +P
BOND C2'  O2'     O2'  H2'
BOND C1'  H1'     C2'  H2''    C3'  H3'     C4'  H4'     C5'  H5'
IMPR C2   N1   N3   O2        C4   C5   N3   O4
!ribose
IC -O3' P    O5'  C5'    1.6001  101.45  -39.25  119.00  1.4401
IC -O3' O5'  *P   O1P    1.6001  101.45 -115.82  109.74  1.4802
IC -O3' O5'  *P   O2P    1.6001  101.45  115.90  109.80  1.4801
IC  P   O5'  C5'  C4'    1.5996  119.00 -151.39  110.04  1.5160
IC O5'  C5'  C4'  C3'    1.4401  108.83 -179.85  116.10  1.5284
IC C5'  C4'  C3'  O3'    1.5160  116.10   76.70  115.12  1.4212
IC C4'  C3'  O3'  +P     1.5284  111.92  159.13  119.05  1.6001
IC C3'  O3'  +P   +O5'   1.4212  119.05  -98.86  101.45  1.5996
IC O4'  C3'  *C4' C5'    1.4572  104.06 -120.04  116.10  1.5160
IC C2'  C4'  *C3' O3'    1.5284  100.16 -124.08  115.12  1.4212
IC C4'  C3'  C2'  C1'    1.5284  100.16   39.58  102.04  1.5251
IC C3'  C2'  C1'  N1     1.5284  101.97  144.39  113.71  1.4896
IC O4'  C1'  N1   C2     1.5251  113.71  -96.0   117.06  1.3746
IC C1'  C3'  *C2' O2'    1.5284  102.04 -114.67  110.81  1.4212
IC H2'  O2'  C2'  C3'    0.9600  114.97  148.63  111.92  1.5284
IC O4'  C2'  *C1' H1'    0.0       0.0  -115.0     0.0   0.0
IC C1'  C3'  *C2' H2''   0.0       0.0   115.0     0.0   0.0
IC C2'  C4'  *C3' H3'    0.0       0.0   115.0     0.0   0.0
IC C3'  O4'  *C4' H4'    0.0       0.0  -115.0     0.0   0.0
IC C4'  O5'  *C5' H5'    0.0       0.0  -115.0     0.0   0.0
IC C4'  O5'  *C5' H5''   0.0       0.0   115.0     0.0   0.0
IC C2   C6   *N1  C1'      1.3830  122.00  180.00  120.50  1.4560
IC C3'  C2'  C1'  N1     1.5284  101.97  144.39  113.71  1.4896
IC O4'  C1'  N1   C2     1.5251  113.71  -96.0   117.06  1.3746
IC C1'  C2   *N1  C6     1.3966  121.57  179.97  122.49  1.4896
IC C6   N1   C2   N3       1.3430  122.00  180.00  114.00  1.3830
IC N3   N1   *C2  O2       1.3830  114.00  180.00  119.40  1.2340
IC N1   C2   N3   C4       1.3830  114.00  180.00  130.20  1.3830
IC C4   C2   *N3  H3       1.3830  130.20  180.00  115.40  1.0100
IC C2   N3   C4   C5       1.3830  130.20  180.00  113.50  1.4030
IC C5   N3   *C4  O4       1.4030  113.50  180.00  119.40  1.2340
IC C5   N1   *C6  H6       1.3500  122.90  180.00  115.00  1.0900
IC C6   C4   *C5  C7       1.3500  116.70  180.00  119.10  1.4800
IC C4   C5   C7   C8       1.4030  119.10  180.00  112.00  1.4700
IC C8   C5   *C7  H71      1.4700  112.00  120.00  107.50  1.1110
IC C8   C5   *C7  H72      1.4700  112.00 -120.00  107.50  1.1110
IC C5   C7   C8   N9       1.4800  112.00  180.00  180.00  1.1800
DONO H3   N3
DONO H2'  O2'
ACCE O2   C2
ACCE O4   C4
ACCE N9   C8
ACCE O1P  P
ACCE O2P  P
ACCE O2'
ACCE O3'
ACCE O4'
ACCE O5'

RESI PSU           -1.00 ! Pseudouridine
GROUP
ATOM C5     CG2R62 -0.17 !                 O2
```

```
ATOM C4      CG2R63  0.53 !                 ||
ATOM O4      OG2D4  -0.46 !                 C2
ATOM N3      NG2R61 -0.51 !                /  \
ATOM H3      HGP1    0.37 !           H1-N1   N3-H3
ATOM C2      CG2R63  0.45 !               |     |
ATOM O2      OG2D4  -0.46 !           H6-C6   C4
ATOM N1      NG2R61 -0.36 !                \\ / \\
ATOM H1      HGP1    0.33 !                 C5   O4
ATOM C6      CG2R62  0.10 !                   \
ATOM H6      HGR62   0.18 !                    \
GROUP                     !                     \
ATOM P       P       1.50 ! O1P    H5' H4'  O4'  \
ATOM O1P     ON3    -0.78 !  |       |    \ /   \  \
ATOM O2P     ON3    -0.78 ! -P-O5'-C5'---C4'     C1'
ATOM O5'     ON2    -0.57 !  |       |      \     / \
ATOM C5'     CN8B   -0.08 ! O2P    H5''   C3'--C2' H1'
ATOM H5'     HN8     0.09 !              / \   / \
ATOM H5''    HN8     0.09 !           O3' H3' O2' H2''
GROUP                     !             |       |
ATOM C1'     CN7B    0.12 !                    H2'
ATOM H1'     HN7     0.09
ATOM C4'     CN7     0.16
ATOM O4'     ON6B   -0.46
ATOM H4'     HN7     0.09
GROUP
ATOM C2'     CN7B    0.14
ATOM H2''    HN7     0.09
ATOM O2'     ON5    -0.66
ATOM H2'     HN5     0.43
GROUP
ATOM C3'     CN7     0.01
ATOM H3'     HN7     0.09
ATOM O3'     ON2    -0.57
BOND C5   C4      C5   C6      C4   O4      C4   N3
BOND N3   C2      N3   H3      C2   O2      C2   N1
BOND N1   C6      N1   H1      C6   H6
BOND P    O1P     P    O2P     P    O5'     O5'  C5'     C5'  H5''
BOND C5'  C4'     C4'  O4'     C4'  C3'     O4'  C1'
BOND C1'  C5      C1'  C2'     C2'  C3'     C3'  O3'     O3'  +P
BOND C2'  O2'     O2'  H2'
BOND C1'  H1'     C2'  H2''    C3'  H3'     C4'  H4'     C5'  H5'
IMPR C2   N1   N3   O2        C4   C5   N3   O4
!ribose
IC -O3' P    O5'   C5'    1.6001  101.45  -39.25  119.00  1.4401
IC -O3' O5'  *P    O1P    1.6001  101.45 -115.82  109.74  1.4802
IC -O3' O5'  *P    O2P    1.6001  101.45  115.90  109.80  1.4801
IC  P   O5'  C5'   C4'    1.5996  119.00 -151.39  110.04  1.5160
IC O5'  C5'  C4'   C3'    1.4401  108.83 -179.85  116.10  1.5284
IC C5'  C4'  C3'   O3'    1.5160  116.10   76.70  115.12  1.4212
IC C4'  C3'  O3'   +P     1.5284  111.92  159.13  119.05  1.6001
IC C3'  O3'  +P    +O5'   1.4212  119.05  -98.86  101.45  1.5996
IC O4'  C3'  *C4'  C5'    1.4572  104.06 -120.04  116.10  1.5160
IC C2'  C4'  *C3'  O3'    1.5284  100.16 -124.08  115.12  1.4212
IC C4'  C3'  C2'   C1'    1.5284  100.16   39.58  102.04  1.5251
IC C5   C1'  O4'   C4'    1.4022  102.36 -123.09  109.06  1.4572
IC C6   C5   C1'   C2'    1.3750  121.43  -77.12  118.79  1.5251
IC C1'  C3'  *C2'  O2'    1.5284  102.04 -114.67  110.81  1.4212
IC H2'  O2'  C2'   C3'    0.9600  114.97  148.63  111.92  1.5284
IC O4'  C2'  *C1'  H1'    0.0       0.0  -115.0     0.0   0.0
IC C1'  C3'  *C2'  H2''   0.0       0.0   115.0     0.0   0.0
IC C2'  C4'  *C3'  H3'    0.0       0.0   115.0     0.0   0.0
IC C3'  O4'  *C4'  H4'    0.0       0.0  -115.0     0.0   0.0
IC C4'  O5'  *C5'  H5'    0.0       0.0  -115.0     0.0   0.0
```

```
IC C4'  O5'  *C5'  H5''   0.0       0.0   115.0     0.0   0.0
IC C4   C1'  *C5   C6     1.4540  120.46 -172.70  121.43  1.3750
IC C6   C5   C4    N3        1.3750  117.92    5.54  115.92  1.3763
IC N3   C5   *C4   O4        1.3763  115.92  179.71  126.09  1.2290
IC C5   C4   N3    C2        1.4540  115.92   -3.52  125.84  1.3628
IC C2   C4   *N3   H3        1.3628  125.84 -177.07  116.70  1.0003
IC C4   N3   C2    O2        1.3763  125.84 -179.25  121.71  1.2234
IC O2   N3   *C2   N1        1.2234  121.71  179.89  116.64  1.3784
IC C6   C2   *N1   H1        1.3699  121.33 -179.28  113.56  1.0057
IC N1   C5   *C6   H6        1.3699  122.13 -179.99  119.95  1.0937
DONO H3   N3
DONO H1   N1
DONO H2'  O2'
ACCE O2   C2
ACCE O4   C4
ACCE O1P  P
ACCE O2P  P
ACCE O2'
ACCE O3'
ACCE O4'
ACCE O5'

RESI OMP           -1.00 ! 2'-O-methylpseudouridine, MRP
GROUP
ATOM C5     CG2R62 -0.17 !                  O2
ATOM C4     CG2R63  0.53 !                  ||
ATOM O4     OG2D4  -0.46 !                  C2
ATOM N3     NG2R61 -0.51 !                 /  \
ATOM H3     HGP1    0.37 !             H1-N1   N3-H3
ATOM C2     CG2R63  0.45 !                |    |
ATOM O2     OG2D4  -0.46 !             H6-C6   C4
ATOM N1     NG2R61 -0.36 !                 \\ / \\
ATOM H1     HGP1    0.33 !                  C5   O4
ATOM C6     CG2R62  0.10 !                    \
ATOM H6     HGR62   0.18 !                     \
GROUP                    !                      \
ATOM P      P       1.50 !  O1P    H5' H4'  O4'  \
ATOM O1P    ON3    -0.78 !   |      |    \ /   \  \
ATOM O2P    ON3    -0.78 !  -P-O5'-C5'---C4'    C1'
ATOM O5'    ON2    -0.57 !   |      |     \     / \
ATOM C5'    CN8B   -0.08 !  O2P    H5''   C3'--C2' H1'
ATOM H5'    HN8     0.09 !               / \   / \
ATOM H5''   HN8     0.09 !             O3' H3' O2' H2''
GROUP                    !              |      |
ATOM C1'    CN7B    0.12 !                    CM2
ATOM H1'    HN7     0.09 !                  /  |  \
ATOM C4'    CN7     0.16 !                HM1 HM2 HM3
ATOM O4'    ON6B   -0.46
ATOM H4'    HN7     0.09
GROUP
ATOM C2'    CN7B    0.08
ATOM H2''   HN7     0.09
ATOM O2'    OG301  -0.34
ATOM CM2    CG331  -0.10
ATOM HM1    HGA3    0.09
ATOM HM2    HGA3    0.09
ATOM HM3    HGA3    0.09
GROUP
ATOM C3'    CN7     0.01
ATOM H3'    HN7     0.09
ATOM O3'    ON2    -0.57
BOND C5   C4      C5   C6      C4   O4      C4   N3
BOND N3   C2      N3   H3      C2   O2      C2   N1
```

```
BOND N1   C6       N1   H1       C6   H6
BOND P    O1P      P    O2P      P    O5'      O5'  C5'      C5'  H5''
BOND C5'  C4'      C4'  O4'      C4'  C3'      O4'  C1'
BOND C1'  C5       C1'  C2'      C2'  C3'      C3'  O3'      O3'  +P
BOND C2'  O2'      CM2  O2'      CM2  HM1      HM2  CM2      HM3  CM2
BOND C1'  H1'      C2'  H2''     C3'  H3'      C4'  H4'      C5'  H5'
IMPR C2   N1   N3   O2         C4   C5   N3   O4
!2OM-ribose
IC -O3' P    O5'   C5'    1.6001  101.45  -39.25  119.00  1.4401
IC -O3' O5'  *P    O1P    1.6001  101.45 -115.82  109.74  1.4802
IC -O3' O5'  *P    O2P    1.6001  101.45  115.90  109.80  1.4801
IC  P   O5'  C5'   C4'    1.5996  119.00 -151.39  110.04  1.5160
IC O5'  C5'  C4'   C3'    1.4401  108.83 -179.85  116.10  1.5284
IC C5'  C4'  C3'   O3'    1.5160  116.10   76.70  115.12  1.4212
IC C4'  C3'  O3'   +P     1.5284  111.92  159.13  119.05  1.6001
IC C3'  O3'  +P    +O5'   1.4212  119.05  -98.86  101.45  1.5996
IC O4'  C3'  *C4'  C5'    1.4572  104.06 -120.04  116.10  1.5160
IC C2'  C4'  *C3'  O3'    1.5284  100.16 -124.08  115.12  1.4212
IC C4'  C3'  C2'   C1'    1.5284  100.16   39.58  102.04  1.5251
IC C5   C1'  O4'   C4'    1.4022  102.36 -123.09  109.06  1.4572
IC C6   C5   C1'   C2'    1.3750  121.43  -77.12  118.79  1.5251
IC C3'  C1'  *C2'  O2'    1.5312  102.03  117.61  107.13  1.4206
IC C1'  C2'  O2'   CM2    1.5393  107.13   90.00  107.00  1.4150
IC C2'  O2'  CM2   HM2    1.4206  107.00  180.00    0.0   0.0
IC HM2  O2'  *CM2  HM3    0.0       0.0   120.00    0.0   0.0
IC HM2  O2'  *CM2  HM1    0.0       0.0  -120.00    0.0   0.0
IC O4'  C2'  *C1'  H1'    0.0       0.0  -115.0     0.0   0.0
IC C1'  C3'  *C2'  H2''   0.0       0.0   115.0     0.0   0.0
IC C2'  C4'  *C3'  H3'    0.0       0.0   115.0     0.0   0.0
IC C3'  O4'  *C4'  H4'    0.0       0.0  -115.0     0.0   0.0
IC C4'  O5'  *C5'  H5'    0.0       0.0  -115.0     0.0   0.0
IC C4'  O5'  *C5'  H5''   0.0       0.0   115.0     0.0   0.0
IC C4   C1'  *C5   C6     1.4540  120.46 -172.70  121.43  1.3750
IC C6   C5   C4    N3        1.3750  117.92    5.54  115.92  1.3763
IC N3   C5   *C4   O4        1.3763  115.92  179.71  126.09  1.2290
IC C5   C4   N3    C2        1.4540  115.92   -3.52  125.84  1.3628
IC C2   C4   *N3   H3        1.3628  125.84 -177.07  116.70  1.0003
IC C4   N3   C2    O2        1.3763  125.84 -179.25  121.71  1.2234
IC O2   N3   *C2   N1        1.2234  121.71  179.89  116.64  1.3784
IC C6   C2   *N1   H1        1.3699  121.33 -179.28  113.56  1.0057
IC N1   C5   *C6   H6        1.3699  122.13 -179.99  119.95  1.0937
DONO H3   N3
DONO H1   N1
ACCE O2   C2
ACCE O4   C4
ACCE O1P  P
ACCE O2P  P
ACCE O2'
ACCE O3'
ACCE O4'
ACCE O5'

RESI 1MP           -1.00 ! 1-methylpseudouridine
GROUP
ATOM C5     CG2R62 -0.19 !           H11      O2
ATOM C4     CG2R63  0.48 !              \     ||
ATOM O4     OG2D4  -0.46 !          H12-C1    C2     H3
ATOM N3     NG2R61 -0.49 !              / \ /  \  /
ATOM H3     HGP1    0.35 !           H13   N1   N3
ATOM C2     CG2R63  0.52 !                 |     |
ATOM O2     OG2D4  -0.47 !              H6-C6    C4
ATOM N1     NG2R61 -0.27 !                   \\ / \\
ATOM C6     CG2R62  0.17 !                    C5   O4
```

```
ATOM H6     HGR62   0.16 !                      \
ATOM C1     CG331  -0.07 !                       \
ATOM H11    HGA3    0.09 !                        \
ATOM H12    HGA3    0.09 !  O1P    H5' H4'  O4'  \
ATOM H13    HGA3    0.09 !   |      |    \ /   \  \
GROUP                      !  -P-O5'-C5'---C4'    C1'
ATOM P      P       1.50 !   |      |     \     / \
ATOM O1P    ON3    -0.78 !  O2P    H5''   C3'--C2' H1'
ATOM O2P    ON3    -0.78 !               / \   / \
ATOM O5'    ON2    -0.57 !             O3' H3' O2' H2''
ATOM C5'    CN8B   -0.08 !              |       |
ATOM H5'    HN8     0.09 !                     H2'
ATOM H5''   HN8     0.09
GROUP
ATOM C1'    CN7B    0.12
ATOM H1'    HN7     0.09
ATOM C4'    CN7     0.16
ATOM O4'    ON6B   -0.46
ATOM H4'    HN7     0.09
GROUP
ATOM C2'    CN7B    0.14
ATOM H2''   HN7     0.09
ATOM O2'    ON5    -0.66
ATOM H2'    HN5     0.43
GROUP
ATOM C3'    CN7     0.01
ATOM H3'    HN7     0.09
ATOM O3'    ON2    -0.57
BOND C5   C4   C5   C6   C4   O4   C4   N3
BOND N3   C2   N3   H3   C2   O2   C2   N1
BOND N1   C6   N1   C1   C6   H6   C1   H11
BOND C1   H12  C1   H13
BOND P    O1P     P    O2P     P    O5'     O5'  C5'     C5'  H5''
BOND C5'  C4'     C4'  O4'     C4'  C3'     O4'  C1'
BOND C1'  C5      C1'  C2'     C2'  C3'     C3'  O3'     O3'  +P
BOND C2'  O2'     O2'  H2'
BOND C1'  H1'     C2'  H2''    C3'  H3'     C4'  H4'     C5'  H5'
IMPR C4     C5     N3     O4     C2     N3     N1     O2
!ribose
IC -O3' P    O5'   C5'    1.6001  101.45  -39.25  119.00  1.4401
IC -O3' O5'  *P    O1P    1.6001  101.45 -115.82  109.74  1.4802
IC -O3' O5'  *P    O2P    1.6001  101.45  115.90  109.80  1.4801
IC  P   O5'  C5'   C4'    1.5996  119.00 -151.39  110.04  1.5160
IC O5'  C5'  C4'   C3'    1.4401  108.83 -179.85  116.10  1.5284
IC C5'  C4'  C3'   O3'    1.5160  116.10   76.70  115.12  1.4212
IC C4'  C3'  O3'   +P     1.5284  111.92  159.13  119.05  1.6001
IC C3'  O3'  +P    +O5'   1.4212  119.05  -98.86  101.45  1.5996
IC O4'  C3'  *C4'  C5'    1.4572  104.06 -120.04  116.10  1.5160
IC C2'  C4'  *C3'  O3'    1.5284  100.16 -124.08  115.12  1.4212
IC C4'  C3'  C2'   C1'    1.5284  100.16   39.58  102.04  1.5251
IC C5   C1'  O4'   C4'    1.4022  102.36 -123.09  109.06  1.4572
IC C6   C5   C1'   C2'    1.3750  121.43  -77.12  118.79  1.5251
IC C1'  C3'  *C2'  O2'    1.5284  102.04 -114.67  110.81  1.4212
IC H2'  O2'  C2'   C3'    0.9600  114.97  148.63  111.92  1.5284
IC O4'  C2'  *C1'  H1'    0.0       0.0  -115.0     0.0   0.0
IC C1'  C3'  *C2'  H2''   0.0       0.0   115.0     0.0   0.0
IC C2'  C4'  *C3'  H3'    0.0       0.0   115.0     0.0   0.0
IC C3'  O4'  *C4'  H4'    0.0       0.0  -115.0     0.0   0.0
IC C4'  O5'  *C5'  H5'    0.0       0.0  -115.0     0.0   0.0
IC C4'  O5'  *C5'  H5''   0.0       0.0   115.0     0.0   0.0
IC C4   C1'  *C5   C6     1.4540  120.46 -172.70  121.43  1.3750
IC C6   C5   C4    N3       1.3748  118.04   -5.52  115.97  1.3740
IC N3   C5   *C4   O4       1.3740  115.97 -179.63  126.00  1.2280
```

```
IC C5   C4   N3    C2        1.4483  115.97    4.05  126.12  1.3653
IC C2   C4   *N3   H3        1.3653  126.12  176.43  116.74  1.0004
IC C4   N3   C2    N1        1.3740  126.12   -1.11  116.65  1.4029
IC N1   N3   *C2   O2        1.4029  116.65  179.53  120.76  1.2278
IC N1   C5   *C6   H6        1.3704  123.08 -179.36  119.87  1.0913
IC C6   C2   *N1   C1        1.3704  119.91  179.62  117.53  1.4719
IC C2   N1   C1    H11       1.4029  117.53  179.84  112.54  1.1131
IC H11  N1   *C1   H12       1.1131  112.54  120.48  110.95  1.1154
IC H11  N1   *C1   H13       1.1131  112.54 -120.49  111.02  1.1148
DONO H2'  O2'
DONO H3   N3
ACCE O2   C2
ACCE O4   C4
ACCE O1P  P
ACCE O2P  P
ACCE O2'
ACCE O3'
ACCE O4'
ACCE O5'

RESI 3MP           -1.00 ! 3-methylpseudouridine
GROUP
ATOM C5     CG2R62 -0.20 !                  O2    H31
ATOM C4     CG2R63  0.63 !                  ||     |
ATOM O4     OG2D4  -0.47 !                  C2    C3 -H32
ATOM N3     NG2R61 -0.40 !                 /  \  /  \
ATOM C2     CG2R63  0.53 !             H1-N1   N3   H33
ATOM O2     OG2D4  -0.47 !                 |    |
ATOM N1     NG2R61 -0.32 !             H6-C6   C4
ATOM H1     HGP1    0.31 !                 \\ / \\
ATOM C6     CG2R62  0.03 !                  C5   O4
ATOM H6     HGR62   0.19 !                    \
ATOM C3     CG331  -0.10 !                     \
ATOM H31    HGA3    0.09 !                      \
ATOM H32    HGA3    0.09 !  O1P    H5' H4'  O4'  \
ATOM H33    HGA3    0.09 !   |      |    \ /   \  \
GROUP                    !  -P-O5'-C5'---C4'    C1'
ATOM P      P       1.50 !   |      |     \     / \
ATOM O1P    ON3    -0.78 !  O2P    H5''   C3'--C2' H1'
ATOM O2P    ON3    -0.78 !               / \   / \
ATOM O5'    ON2    -0.57 !             O3' H3' O2' H2''
ATOM C5'    CN8B   -0.08 !              |       |
ATOM H5'    HN8     0.09 !                     H2'
ATOM H5''   HN8     0.09
GROUP
ATOM C1'    CN7B    0.12
ATOM H1'    HN7     0.09
ATOM C4'    CN7     0.16
ATOM O4'    ON6B   -0.46
ATOM H4'    HN7     0.09

ATOM C2'    CN7B    0.14
ATOM H2''   HN7     0.09
ATOM O2'    ON5    -0.66
ATOM H2'    HN5     0.43
GROUP
ATOM C3'    CN7     0.01
ATOM H3'    HN7     0.09
ATOM O3'    ON2    -0.57
BOND C5   C4      C5   C6      C4   O4      C4   N3
BOND N3   C2      N3   C3      C2   O2      C2   N1
BOND N1   C6      N1   H1      C6   H6      C3   H31
BOND C3   H32     C3   H33
```

```
BOND P    O1P     P    O2P     P    O5'     O5'  C5'     C5'  H5''
BOND C5'  C4'     C4'  O4'     C4'  C3'     O4'  C1'
BOND C1'  C5      C1'  C2'     C2'  C3'     C3'  O3'     O3'  +P
BOND C2'  O2'     O2'  H2'
BOND C1'  H1'     C2'  H2''    C3'  H3'     C4'  H4'     C5'  H5'
IMPR C4     C5     N3     O4       C2     N3     N1     O2
!ribose
IC -O3' P    O5'  C5'    1.6001  101.45  -39.25  119.00  1.4401
IC -O3' O5'  *P   O1P    1.6001  101.45 -115.82  109.74  1.4802
IC -O3' O5'  *P   O2P    1.6001  101.45  115.90  109.80  1.4801
IC  P   O5'  C5'  C4'    1.5996  119.00 -151.39  110.04  1.5160
IC O5'  C5'  C4'  C3'    1.4401  108.83 -179.85  116.10  1.5284
IC C5'  C4'  C3'  O3'    1.5160  116.10   76.70  115.12  1.4212
IC C4'  C3'  O3'  +P     1.5284  111.92  159.13  119.05  1.6001
IC C3'  O3'  +P   +O5'   1.4212  119.05  -98.86  101.45  1.5996
IC O4'  C3'  *C4' C5'    1.4572  104.06 -120.04  116.10  1.5160
IC C2'  C4'  *C3' O3'    1.5284  100.16 -124.08  115.12  1.4212
IC C4'  C3'  C2'  C1'    1.5284  100.16   39.58  102.04  1.5251
IC C5   C1'  O4'  C4'    1.4022  102.36 -123.09  109.06  1.4572
IC C6   C5   C1'  C2'    1.3750  121.43  -77.12  118.79  1.5251
IC C1'  C3'  *C2' O2'    1.5284  102.04 -114.67  110.81  1.4212
IC H2'  O2'  C2'  C3'    0.9600  114.97  148.63  111.92  1.5284
IC O4'  C2'  *C1' H1'    0.0       0.0  -115.0     0.0   0.0
IC C1'  C3'  *C2' H2''   0.0       0.0   115.0     0.0   0.0
IC C2'  C4'  *C3' H3'    0.0       0.0   115.0     0.0   0.0
IC C3'  O4'  *C4' H4'    0.0       0.0  -115.0     0.0   0.0
IC C4'  O5'  *C5' H5'    0.0       0.0  -115.0     0.0   0.0
IC C4'  O5'  *C5' H5''   0.0       0.0   115.0     0.0   0.0
IC C4   C1'  *C5  C6     1.4540  120.46 -172.70  121.43  1.3750
IC C6   C5   C4   N3        1.3500  116.70  180.00  113.50  1.3830
IC N3   C5   *C4  O4        1.3830  113.50  180.00  124.60  1.2340
IC C5   C4   N3   C2        1.4030  113.50  180.00  130.20  1.3830
IC C2   C4   *N3  C3        1.3830  130.20  180.00  115.40  1.4560
IC C4   N3   C2   O2        1.3830  130.20  180.00  119.40  1.2340
IC O2   N3   *C2  N1        1.2340  119.40  180.00  114.00  1.3830
IC C6   C2   *N1  H1        1.3430  122.00  180.00  115.40  1.0100
IC N1   C5   *C6  H6        1.3430  122.90  180.00  119.00  1.0900
IC C4   N3   C3   H31       1.3830  115.40  180.00  110.10  1.1110
IC H31  N3   *C3  H32       1.1110  110.10  120.00  110.10  1.1110
IC H31  N3   *C3  H33       1.1110  110.10 -120.00  110.10  1.1110
DONO H2'  O2'
DONO H1   N1
ACCE O2   C2
ACCE O4   C4
ACCE O1P  P
ACCE O2P  P
ACCE O2'
ACCE O3'
ACCE O4'
ACCE O5'

RESI SAU            0.00 ! 5-aminomethyl-2-thiouridine
GROUP
ATOM N1     NG2R61 -0.26 !        H81  H71  O4
ATOM C2     CG2R63  0.29 !     (+) |    |   ||
ATOM S2     SG2D1  -0.22 !    H82-N8 --C7   C4    H3
ATOM N3     NG2R61 -0.56 !         |   / \ /  \  /
ATOM H3     HGP1    0.40 !       H83 H72  C5   N3
ATOM C4     CG2R63  0.39 !                ||   |
ATOM O4     OG2D4  -0.41 !            H6-C6   C2
ATOM C5     CG2R62 -0.03 !                \  / \\
ATOM C6     CG2R62  0.18 !                 N1   S2
ATOM H6     HGR62   0.22 !                  \
```

```
GROUP                    !                     \
ATOM C7     CG324   0.21 !                      \
ATOM H71    HGA2    0.05 !  O1P    H5' H4'  O4'  \
ATOM H72    HGA2    0.05 !   |      |    \ /   \  \
ATOM N8     NG3P3  -0.30 !  -P-O5'-C5'---C4'    C1'
ATOM H81    HGP2    0.33 !   |      |     \     / \
ATOM H82    HGP2    0.33 !  O2P    H5''   C3'--C2' H1'
ATOM H83    HGP2    0.33 !               / \   / \
GROUP                    !             O3' H3' O2' H2''
ATOM P      P       1.50 !              |       |
ATOM O1P    ON3    -0.78 !                     H2'
ATOM O2P    ON3    -0.78
ATOM O5'    ON2    -0.57 !  !!!! PATCH 5UNA for the neutral amino form
ATOM C5'    CN8B   -0.08
ATOM H5'    HN8     0.09
ATOM H5''   HN8     0.09
GROUP
ATOM C4'    CN7     0.16
ATOM H4'    HN7     0.09
ATOM O4'    ON6B   -0.50
ATOM C1'    CN7B    0.16
ATOM H1'    HN7     0.09
GROUP
ATOM C2'    CN7B    0.14
ATOM H2''   HN7     0.09
ATOM O2'    ON5    -0.66
ATOM H2'    HN5     0.43
GROUP
ATOM C3'    CN7     0.01
ATOM H3'    HN7     0.09
ATOM O3'    ON2    -0.57
BOND N1   C2   N1   C6   C2   S2   C2   N3
BOND N3   C4   N3   H3   C4   O4   C4   C5
BOND C5   C6   C5   C7   C6   H6   C7   N8
BOND C7   H71  C7   H72  N8   H81  N8   H82
BOND N8   H83
BOND P    O1P     P    O2P     P    O5'     O5'  C5'     C5'  H5''
BOND C5'  C4'     C4'  O4'     C4'  C3'     O4'  C1'
BOND C1'  N1      C1'  C2'     C2'  C3'     C3'  O3'     O3'  +P
BOND C2'  O2'     O2'  H2'
BOND C1'  H1'     C2'  H2''    C3'  H3'     C4'  H4'     C5'  H5'
IMPR C2     N1     N3     S2     C4     C5     N3     O4
!ribose
IC -O3' P    O5'   C5'    1.6001  101.45  -39.25  119.00  1.4401
IC -O3' O5'  *P    O1P    1.6001  101.45 -115.82  109.74  1.4802
IC -O3' O5'  *P    O2P    1.6001  101.45  115.90  109.80  1.4801
IC  P   O5'  C5'   C4'    1.5996  119.00 -151.39  110.04  1.5160
IC O5'  C5'  C4'   C3'    1.4401  108.83 -179.85  116.10  1.5284
IC C5'  C4'  C3'   O3'    1.5160  116.10   76.70  115.12  1.4212
IC C4'  C3'  O3'   +P     1.5284  111.92  159.13  119.05  1.6001
IC C3'  O3'  +P    +O5'   1.4212  119.05  -98.86  101.45  1.5996
IC O4'  C3'  *C4'  C5'    1.4572  104.06 -120.04  116.10  1.5160
IC C2'  C4'  *C3'  O3'    1.5284  100.16 -124.08  115.12  1.4212
IC C4'  C3'  C2'   C1'    1.5284  100.16   39.58  102.04  1.5251
IC C3'  C2'  C1'   N1     1.5284  101.97  144.39  113.71  1.4896
IC O4'  C1'  N1    C2     1.5251  113.71  -96.0   117.06  1.3746
IC C1'  C3'  *C2'  O2'    1.5284  102.04 -114.67  110.81  1.4212
IC H2'  O2'  C2'   C3'    0.9600  114.97  148.63  111.92  1.5284
IC O4'  C2'  *C1'  H1'    0.0       0.0  -115.0     0.0   0.0
IC C1'  C3'  *C2'  H2''   0.0       0.0   115.0     0.0   0.0
IC C2'  C4'  *C3'  H3'    0.0       0.0   115.0     0.0   0.0
IC C3'  O4'  *C4'  H4'    0.0       0.0  -115.0     0.0   0.0
IC C4'  O5'  *C5'  H5'    0.0       0.0  -115.0     0.0   0.0
```

```
IC C4'  O5'  *C5'  H5''   0.0       0.0   115.0     0.0   0.0
IC C2   C6   *N1   C1'       1.3966  121.57  179.97  122.49  1.4896
IC C3'  C2'  C1'   N1     1.5284  101.97  144.39  113.71  1.4896
IC O4'  C1'  N1    C2     1.5251  113.71  -96.0   117.06  1.3746
IC C1'  C2   *N1   C6     1.3966  121.57  179.97  122.49  1.4896
IC C6   N1   C2    N3        1.3573  125.04   -1.76  111.76  1.3911
IC N3   N1   *C2   S2        1.3911  111.76 -179.44  123.63  1.6271
IC N1   C2   N3    C4        1.3961  111.76    0.70  128.01  1.3770
IC C4   C2   *N3   H3        1.3770  128.01 -179.75  115.84  1.0214
IC C2   N3   C4    C5        1.3911  128.01    0.54  115.30  1.4536
IC C5   N3   *C4   O4        1.4536  115.30 -178.63  121.57  1.2470
IC C5   N1   *C6   H6        1.3622  121.12 -179.33  115.66  1.0881
IC C6   C4   *C5   C7        1.3622  118.74  173.77  116.93  1.4929
IC C4   C5   C7    N8        1.4536  116.93   50.30  108.75  1.5144
IC N8   C5   *C7   H71       1.5144  108.75  121.03  113.01  1.0917
IC N8   C5   *C7   H72       1.5144  108.75 -115.50  111.64  1.0941
IC C5   C7   N8    H81       1.4929  108.75 -171.02  112.69  1.0270
IC H81  C7   *N8   H82       1.0270  112.69  119.89  104.72  1.0513
IC H81  C7   *N8   H83       1.0270  112.69 -122.57  111.85  1.0273
DONO H2'  O2'
DONO H3   N3
DONO H81  N8
DONO H82  N8
DONO H83  N8
ACCE O4   C4
ACCE S2   C2
ACCE O1P  P
ACCE O2P  P
ACCE O2'
ACCE O3'
ACCE O4'
ACCE O5'

RESI 5AU            0.00 ! 5-methylaminomethyluridine
GROUP
ATOM N1     NG2R61 -0.34 !     HA3 HN1  HC2  O4
ATOM C2     CG2R63  0.51 !       |   |(+) |    ||
ATOM O2     OG2D4  -0.41 !  HA2-CA -N --C     C4    H3
ATOM N3     NG2R61 -0.46 !       |   |   / \ /  \  /
ATOM H3     HGP1    0.36 !     HA1 HN2 HC1 C5   N3
ATOM C4     CG2R63  0.50 !                  ||   |
ATOM O4     OG2D4  -0.45 !               H6-C6   C2
ATOM C5     CG2R62 -0.05 !                   \  / \\
ATOM C6     CG2R62  0.17 !                    N1   O2
ATOM H6     HGR62   0.17 !                     \
GROUP                    !                      \
ATOM C      CG324   0.20 !                       \
ATOM HC1    HGA2    0.09 !  O1P    H5' H4'  O4'  \
ATOM HC2    HGA2    0.09 !   |      |    \ /   \  \
ATOM N      NG3P2  -0.52 !  -P-O5'-C5'---C4'    C1'
ATOM HN1    HGP2    0.38 !   |      |     \     / \
ATOM HN2    HGP2    0.38 !  O2P    H5''   C3'--C2' H1'
ATOM CA     CG334   0.11 !               / \   / \
ATOM HA1    HGA3    0.09 !             O3' H3' O2' H2''
ATOM HA2    HGA3    0.09 !              |       |
ATOM HA3    HGA3    0.09 !                     H2'
GROUP
ATOM P      P       1.50
ATOM O1P    ON3    -0.78
ATOM O2P    ON3    -0.78
ATOM O5'    ON2    -0.57
ATOM C5'    CN8B   -0.08
ATOM H5'    HN8     0.09
```

```
ATOM H5''   HN8      0.09
GROUP
ATOM C4'    CN7      0.16
ATOM H4'    HN7      0.09
ATOM O4'    ON6B    -0.50
ATOM C1'    CN7B     0.16
ATOM H1'    HN7      0.09
GROUP
ATOM C2'    CN7B     0.14
ATOM H2''   HN7      0.09
ATOM O2'    ON5     -0.66
ATOM H2'    HN5      0.43
GROUP
ATOM C3'    CN7      0.01
ATOM H3'    HN7      0.09
ATOM O3'    ON2     -0.57
BOND N1   C2    N1   C6     C2   O2     C2   N3
BOND N3   C4    N3   H3     C4   O4     C4   C5
BOND C5   C6    C5   C      C6   H6     C    N
BOND C    HC1   C    HC2    N    CA     N    HN2
BOND N    HN1   CA   HA1    CA   HA2    CA   HA3
BOND P    O1P     P    O2P     P    O5'     O5'  C5'     C5'  H5''
BOND C5'  C4'     C4'  O4'     C4'  C3'     O4'  C1'
BOND C1'  N1      C1'  C2'     C2'  C3'     C3'  O3'     O3'  +P
BOND C2'  O2'     O2'  H2'
BOND C1'  H1'     C2'  H2''    C3'  H3'     C4'  H4'     C5'  H5'
IMPR C2     N1     N3     O2      C4     C5     N3     O4
!ribose
IC -O3' P    O5'   C5'    1.6001  101.45  -39.25  119.00  1.4401
IC -O3' O5'  *P    O1P    1.6001  101.45 -115.82  109.74  1.4802
IC -O3' O5'  *P    O2P    1.6001  101.45  115.90  109.80  1.4801
IC  P   O5'  C5'   C4'    1.5996  119.00 -151.39  110.04  1.5160
IC O5'  C5'  C4'   C3'    1.4401  108.83 -179.85  116.10  1.5284
IC C5'  C4'  C3'   O3'    1.5160  116.10   76.70  115.12  1.4212
IC C4'  C3'  O3'   +P     1.5284  111.92  159.13  119.05  1.6001
IC C3'  O3'  +P    +O5'   1.4212  119.05  -98.86  101.45  1.5996
IC O4'  C3'  *C4'  C5'    1.4572  104.06 -120.04  116.10  1.5160
IC C2'  C4'  *C3'  O3'    1.5284  100.16 -124.08  115.12  1.4212
IC C4'  C3'  C2'   C1'    1.5284  100.16   39.58  102.04  1.5251
IC C3'  C2'  C1'   N1     1.5284  101.97  144.39  113.71  1.4896
IC O4'  C1'  N1    C2     1.5251  113.71  -96.0   117.06  1.3746
IC C1'  C3'  *C2'  O2'    1.5284  102.04 -114.67  110.81  1.4212
IC H2'  O2'  C2'   C3'    0.9600  114.97  148.63  111.92  1.5284
IC O4'  C2'  *C1'  H1'    0.0       0.0  -115.0     0.0   0.0
IC C1'  C3'  *C2'  H2''   0.0       0.0   115.0     0.0   0.0
IC C2'  C4'  *C3'  H3'    0.0       0.0   115.0     0.0   0.0
IC C3'  O4'  *C4'  H4'    0.0       0.0  -115.0     0.0   0.0
IC C4'  O5'  *C5'  H5'    0.0       0.0  -115.0     0.0   0.0
IC C4'  O5'  *C5'  H5''   0.0       0.0   115.0     0.0   0.0
IC C2   C6   *N1   C1'       1.3966  121.57  179.97  122.49  1.4896
IC C3'  C2'  C1'   N1     1.5284  101.97  144.39  113.71  1.4896
IC O4'  C1'  N1    C2     1.5251  113.71  -96.0   117.06  1.3746
IC C1'  C2   *N1   C6     1.3966  121.57  179.97  122.49  1.4896
IC C6   N1   C2    N3        1.4208  124.69  -16.11  114.20  1.3903
IC N3   N1   *C2   O2        1.3903  114.20 -177.82  123.55  1.2364
IC N1   C2   N3    C4        1.3756  114.20    7.49  125.23  1.3971
IC C4   C2   *N3   H3        1.3971  125.23  170.29  116.40  1.0156
IC C2   N3   C4    C5        1.3903  125.23   -4.21  117.19  1.3955
IC C5   N3   *C4   O4        1.3955  117.19  178.46  117.91  1.2912
IC C5   N1   *C6   H6        1.4306  114.74  179.71  113.61  1.0884
IC C6   C4   *C5   C         1.4306  121.01  172.12  115.75  1.4823
IC C4   C5   C     N         1.3955  115.75  -47.39  107.42  1.5168
IC N    C5   *C    HC1       1.5168  107.42  114.91  113.80  1.0991
```

```
IC N    C5   *C    HC2       1.5168  107.42 -119.88  112.95  1.0952
IC C5   C    N     CA        1.4823  107.42  170.25  116.20  1.4795
IC CA   C    *N    HN2       1.4795  116.20 -121.77   99.66  1.0863
IC CA   C    *N    HN1       1.4795  116.20  126.56  107.84  1.0264
IC C    N    CA    HA1       1.5168  116.20   58.74  109.77  1.0917
IC HA1  N    *CA   HA2       1.0917  109.77  120.95  109.39  1.0915
IC HA1  N    *CA   HA3       1.0917  109.77 -119.89  108.19  1.0903
DONO H2'  O2'
DONO H3   N3
DONO HN1  N
DONO HN2  N
ACCE O2   C2
ACCE O4   C4
ACCE O1P  P
ACCE O2P  P
ACCE O2'
ACCE O3'
ACCE O4'
ACCE O5'

RESI U8U            0.00 ! 5-methylaminomethyl-2-thiouridine, ESU
GROUP
ATOM N1     NG2R61 -0.26 !      HA3 HN1  HC2  O4
ATOM C2     CG2R63  0.29 !        |   |(+) |   ||
ATOM S2     SG2D1  -0.22 !   HA2-CA -N --C    C4    H3
ATOM N3     NG2R61 -0.56 !        |   |   / \ /  \  /
ATOM H3     HGP1    0.40 !      HA1 HN2 HC1 C5   N3
ATOM C4     CG2R63  0.39 !                  ||   |
ATOM O4     OG2D4  -0.41 !               H6-C6   C2
ATOM C5     CG2R62 -0.03 !                   \  / \\
ATOM C6     CG2R62  0.18 !                    N1   S2
ATOM H6     HGR62   0.22 !                     \
GROUP                    !                      \
ATOM C      CG324   0.20 !                       \
ATOM HC1    HGA2    0.09 !   O1P    H5' H4'  O4'  \
ATOM HC2    HGA2    0.09 !    |      |    \ /   \  \
ATOM N      NG3P2  -0.52 !   -P-O5'-C5'---C4'    C1'
ATOM HN1    HGP2    0.38 !    |      |     \     / \
ATOM HN2    HGP2    0.38 !   O2P    H5''   C3'--C2' H1'
ATOM CA     CG334   0.11 !                / \   / \
ATOM HA1    HGA3    0.09 !             O3' H3' O2' H2''
ATOM HA2    HGA3    0.09 !              |       |
ATOM HA3    HGA3    0.09 !                     H2'
GROUP
ATOM P      P       1.50
ATOM O1P    ON3    -0.78
ATOM O2P    ON3    -0.78
ATOM O5'    ON2    -0.57
ATOM C5'    CN8B   -0.08
ATOM H5'    HN8     0.09
ATOM H5''   HN8     0.09
GROUP
ATOM C4'    CN7     0.16
ATOM H4'    HN7     0.09
ATOM O4'    ON6B   -0.50
ATOM C1'    CN7B    0.16
ATOM H1'    HN7     0.09
GROUP
ATOM C2'    CN7B    0.14
ATOM H2''   HN7     0.09
ATOM O2'    ON5    -0.66
ATOM H2'    HN5     0.43
GROUP
```

```
ATOM C3'    CN7     0.01
ATOM H3'    HN7     0.09
ATOM O3'    ON2    -0.57
BOND N1   C2     N1   C6     C2   S2     C2   N3
BOND N3   C4     N3   H3     C4   O4     C4   C5
BOND C5   C6     C5   C      C6   H6     C    N
BOND C    HC1    C    HC2    N    CA     N    HN2
BOND N    HN1    CA   HA1    CA   HA2    CA   HA3
BOND P    O1P     P    O2P     P    O5'     O5'  C5'     C5'  H5''
BOND C5'  C4'     C4'  O4'     C4'  C3'     O4'  C1'
BOND C1'  N1      C1'  C2'     C2'  C3'     C3'  O3'     O3'  +P
BOND C2'  O2'     O2'  H2'
BOND C1'  H1'     C2'  H2''    C3'  H3'     C4'  H4'     C5'  H5'
IMPR C2     N1     N3     S2      C4     C5     N3     O4
!ribose
IC -O3' P    O5'  C5'    1.6001  101.45  -39.25  119.00  1.4401
IC -O3' O5'  *P   O1P    1.6001  101.45 -115.82  109.74  1.4802
IC -O3' O5'  *P   O2P    1.6001  101.45  115.90  109.80  1.4801
IC  P   O5'  C5'  C4'    1.5996  119.00 -151.39  110.04  1.5160
IC O5'  C5'  C4'  C3'    1.4401  108.83 -179.85  116.10  1.5284
IC C5'  C4'  C3'  O3'    1.5160  116.10   76.70  115.12  1.4212
IC C4'  C3'  O3'  +P     1.5284  111.92  159.13  119.05  1.6001
IC C3'  O3'  +P   +O5'   1.4212  119.05  -98.86  101.45  1.5996
IC O4'  C3'  *C4' C5'    1.4572  104.06 -120.04  116.10  1.5160
IC C2'  C4'  *C3' O3'    1.5284  100.16 -124.08  115.12  1.4212
IC C4'  C3'  C2'  C1'    1.5284  100.16   39.58  102.04  1.5251
IC C3'  C2'  C1'  N1     1.5284  101.97  144.39  113.71  1.4896
IC O4'  C1'  N1   C2     1.5251  113.71  -96.0   117.06  1.3746
IC C1'  C3'  *C2' O2'    1.5284  102.04 -114.67  110.81  1.4212
IC H2'  O2'  C2'  C3'    0.9600  114.97  148.63  111.92  1.5284
IC O4'  C2'  *C1' H1'    0.0       0.0  -115.0     0.0   0.0
IC C1'  C3'  *C2' H2''   0.0       0.0   115.0     0.0   0.0
IC C2'  C4'  *C3' H3'    0.0       0.0   115.0     0.0   0.0
IC C3'  O4'  *C4' H4'    0.0       0.0  -115.0     0.0   0.0
IC C4'  O5'  *C5' H5'    0.0       0.0  -115.0     0.0   0.0
IC C4'  O5'  *C5' H5''   0.0       0.0   115.0     0.0   0.0
IC C2   C6   *N1  C1'       1.3966  121.57  179.97  122.49  1.4896
IC C3'  C2'  C1'  N1     1.5284  101.97  144.39  113.71  1.4896
IC O4'  C1'  N1   C2     1.5251  113.71  -96.0   117.06  1.3746
IC C1'  C2   *N1  C6     1.3966  121.57  179.97  122.49  1.4896
IC C6   N1   C2   N3        1.3692  121.64   -0.01  113.60  1.3883
IC N3   N1   *C2  S2        1.3883  113.60  179.99  125.69  1.6493
IC N1   C2   N3   C4        1.4164  113.60    0.01  127.46  1.3796
IC C4   C2   *N3  H3        1.3796  127.46 -179.97  117.38  1.0079
IC C2   N3   C4   C5        1.3883  127.46   -0.04  116.59  1.4397
IC C5   N3   *C4  O4        1.4397  116.59 -179.92  118.72  1.2271
IC C5   N1   *C6  H6        1.3841  123.38  179.98  113.81  1.0876
IC C6   C4   *C5  C         1.3841  117.33  179.92  110.96  1.4814
IC C4   C5   C    N         1.4397  110.96 -179.99  111.92  1.4901
IC N    C5   *C   HC1       1.4901  111.92  118.45  110.27  1.1068
IC N    C5   *C   HC2       1.4901  111.92 -118.34  110.19  1.1079
IC C5   C    N    CA        1.4814  111.92 -179.99  113.58  1.5065
IC CA   C    *N   HN2       1.5065  113.58  121.86  109.64  1.0123
IC CA   C    *N   HN1       1.5065  113.58 -121.81  109.59  1.0125
IC C    N    CA   HA1       1.4901  113.58  179.98  107.28  1.1113
IC HA1  N    *CA  HA2       1.1113  107.28  120.02  107.33  1.1102
IC HA1  N    *CA  HA3       1.1113  107.28 -119.89  107.33  1.1110
DONO H2'  O2'
DONO H3   N3
DONO HN1  N
DONO HN2  N
ACCE O4   C4
ACCE S2   C2
```

```
ACCE O1P  P
ACCE O2P  P
ACCE O2'
ACCE O3'
ACCE O4'
ACCE O5'

RESI SEU             0.00 ! 5-methylaminomethyl-2-selenouridine
GROUP
ATOM N1      NG2R61 -0.26 !      HA3 HN1  HC2  O4
ATOM C2      CG2R63  0.25 !        |   |(+) |   ||
ATOM SE2     SEGD1  -0.16 !   HA2-CA -N --C    C4    H3
ATOM N3      NG2R61 -0.56 !        |   |   / \ /  \  /
ATOM H3      HGP1    0.40 !      HA1 HN2 HC1 C5   N3
ATOM C4      CG2R63  0.39 !                  ||   |
ATOM O4      OG2D4  -0.41 !               H6-C6   C2
ATOM C5      CG2R62 -0.05 !                    \  / \\
ATOM C6      CG2R62  0.18 !                     N1   SE2
ATOM H6      HGR62   0.22 !                      \
GROUP                     !                       \
ATOM C       CG324   0.20 !                        \
ATOM HC1     HGA2    0.09 !   O1P    H5' H4'  O4'  \
ATOM HC2     HGA2    0.09 !    |      |    \ /   \  \
ATOM N       NG3P2  -0.52 !   -P-O5'-C5'---C4'    C1'
ATOM HN1     HGP2    0.38 !    |      |     \     / \
ATOM HN2     HGP2    0.38 !   O2P    H5''   C3'--C2' H1'
ATOM CA      CG334   0.11 !                / \   / \
ATOM HA1     HGA3    0.09 !              O3' H3' O2' H2''
ATOM HA2     HGA3    0.09 !               |       |
ATOM HA3     HGA3    0.09 !                      H2'
GROUP
ATOM P       P       1.50
ATOM O1P     ON3    -0.78
ATOM O2P     ON3    -0.78
ATOM O5'     ON2    -0.57
ATOM C5'     CN8B   -0.08
ATOM H5'     HN8     0.09
ATOM H5''    HN8     0.09
GROUP
ATOM C4'     CN7     0.16
ATOM H4'     HN7     0.09
ATOM O4'     ON6B   -0.50
ATOM C1'     CN7B    0.16
ATOM H1'     HN7     0.09
GROUP
ATOM C2'     CN7B    0.14
ATOM H2''    HN7     0.09
ATOM O2'     ON5    -0.66
ATOM H2'     HN5     0.43
GROUP
ATOM C3'     CN7     0.01
ATOM H3'     HN7     0.09
ATOM O3'     ON2    -0.57
BOND N1   C2     N1   C6     C2   SE2    C2   N3
BOND N3   C4     N3   H3     C4   O4     C4   C5
BOND C5   C6     C5   C      C6   H6     C    N
BOND C    HC1    C    HC2    N    CA     N    HN2
BOND N    HN1    CA   HA1    CA   HA2    CA   HA3
BOND P    O1P     P    O2P     P    O5'     O5'  C5'     C5'  H5''
BOND C5'  C4'     C4'  O4'     C4'  C3'     O4'  C1'
BOND C1'  N1      C1'  C2'     C2'  C3'     C3'  O3'     O3'  +P
BOND C2'  O2'     O2'  H2'
BOND C1'  H1'     C2'  H2''    C3'  H3'     C4'  H4'     C5'  H5'
```

```
IMPR C2      N1      N3      SE2      C4      C5      N3      O4
!ribose
IC -O3' P    O5'   C5'    1.6001  101.45  -39.25  119.00  1.4401
IC -O3' O5'  *P    O1P    1.6001  101.45 -115.82  109.74  1.4802
IC -O3' O5'  *P    O2P    1.6001  101.45  115.90  109.80  1.4801
IC  P   O5'  C5'   C4'    1.5996  119.00 -151.39  110.04  1.5160
IC O5'  C5'  C4'   C3'    1.4401  108.83 -179.85  116.10  1.5284
IC C5'  C4'  C3'   O3'    1.5160  116.10   76.70  115.12  1.4212
IC C4'  C3'  O3'   +P     1.5284  111.92  159.13  119.05  1.6001
IC C3'  O3'  +P    +O5'   1.4212  119.05  -98.86  101.45  1.5996
IC O4'  C3'  *C4'  C5'    1.4572  104.06 -120.04  116.10  1.5160
IC C2'  C4'  *C3'  O3'    1.5284  100.16 -124.08  115.12  1.4212
IC C4'  C3'  C2'   C1'    1.5284  100.16   39.58  102.04  1.5251
IC C3'  C2'  C1'   N1     1.5284  101.97  144.39  113.71  1.4896
IC O4'  C1'  N1    C2     1.5251  113.71  -96.0   117.06  1.3746
IC C1'  C3'  *C2'  O2'    1.5284  102.04 -114.67  110.81  1.4212
IC H2'  O2'  C2'   C3'    0.9600  114.97  148.63  111.92  1.5284
IC O4'  C2'  *C1'  H1'    0.0       0.0  -115.0     0.0   0.0
IC C1'  C3'  *C2'  H2''   0.0       0.0   115.0     0.0   0.0
IC C2'  C4'  *C3'  H3'    0.0       0.0   115.0     0.0   0.0
IC C3'  O4'  *C4'  H4'    0.0       0.0  -115.0     0.0   0.0
IC C4'  O5'  *C5'  H5'    0.0       0.0  -115.0     0.0   0.0
IC C4'  O5'  *C5'  H5''   0.0       0.0   115.0     0.0   0.0
IC C2   C6   *N1   C1'       1.3966  121.57  179.97  122.49  1.4896
IC C3'  C2'  C1'   N1     1.5284  101.97  144.39  113.71  1.4896
IC O4'  C1'  N1    C2     1.5251  113.71  -96.0   117.06  1.3746
IC C1'  C2   *N1   C6     1.3966  121.57  179.97  122.49  1.4896
IC C6   N1   C2    N3        1.3692  121.64   -0.01  113.60  1.3883
IC N3   N1   *C2   SE2       1.3883  113.60  179.99  125.69  1.8793
IC N1   C2   N3    C4        1.4164  113.60    0.01  127.46  1.3796
IC C4   C2   *N3   H3        1.3796  127.46 -179.97  117.38  1.0079
IC C2   N3   C4    C5        1.3883  127.46   -0.04  116.59  1.4397
IC C5   N3   *C4   O4        1.4397  116.59 -179.92  118.72  1.2271
IC C5   N1   *C6   H6        1.3841  123.38  179.98  113.81  1.0876
IC C6   C4   *C5   C         1.3841  117.33  179.92  110.96  1.4814
IC C4   C5   C     N         1.4397  110.96 -179.99  111.92  1.4901
IC N    C5   *C    HC1       1.4901  111.92  118.45  110.27  1.1068
IC N    C5   *C    HC2       1.4901  111.92 -118.34  110.19  1.1079
IC C5   C    N     CA        1.4814  111.92 -179.99  113.58  1.5065
IC CA   C    *N    HN2       1.5065  113.58  121.86  109.64  1.0123
IC CA   C    *N    HN1       1.5065  113.58 -121.81  109.59  1.0125
IC C    N    CA    HA1       1.4901  113.58  179.98  107.28  1.1113
IC HA1  N    *CA   HA2       1.1113  107.28  120.02  107.33  1.1102
IC HA1  N    *CA   HA3       1.1113  107.28 -119.89  107.33  1.1110
DONO H2'  O2'
DONO H3   N3
DONO HN1  N
DONO HN2  N
ACCE O4   C4
ACCE O1P  P
ACCE O2P  P
ACCE O2'
ACCE O3'
ACCE O4'
ACCE O5'

RESI 5DU           -1.00 ! 5-carboxymethylaminomethyluridine
GROUP
ATOM N1     NG2R61 -0.34 !   O11  O12 (-)
ATOM C2     CG2R63  0.51 !     \\ /
ATOM O2     OG2D4  -0.41 !      C10
ATOM N3     NG2R61 -0.46 !       |   H81 H72  O4
ATOM H3     HGP1    0.36 !       |   |(+) |   ||
```

```
ATOM C4     CG2R63  0.50 !  H92-C9 -N8 -C7   C4    H3
ATOM O4     OG2D4  -0.45 !       |   |   / \ /  \  /
ATOM C5     CG2R62 -0.05 !      H91 H82 H71 C5   N3
ATOM C6     CG2R62  0.17 !                  ||   |
ATOM H6     HGR62   0.17 !               H6-C6   C2
GROUP                    !                   \  / \\
ATOM C7     CG324   0.04 !                    N1   O2
ATOM H71    HGA2    0.09 !                     \
ATOM H72    HGA2    0.09 !                      \
ATOM N8     NG3P2  -0.34 !                       \
ATOM H81    HGP2    0.34 !  O1P    H5' H4'  O4'  \
ATOM H82    HGP2    0.34 !   |      |    \ /   \  \
ATOM C9     CG324  -0.01 !  -P-O5'-C5'---C4'    C1'
ATOM H91    HGA2    0.09 !   |      |     \     / \
ATOM H92    HGA2    0.09 !  O2P    H5''   C3'--C2'  H1'
ATOM C10    CG2O3   0.59 !               / \   / \
ATOM O11    OG2D2  -0.66 !             O3' H3' O2' H2''
ATOM O12    OG2D2  -0.66 !              |       |
GROUP                    !                     H2'
ATOM P      P       1.50
ATOM O1P    ON3    -0.78 ! !! PATCH 5UHG for the non-ionic tautomer
ATOM O2P    ON3    -0.78
ATOM O5'    ON2    -0.57
ATOM C5'    CN8B   -0.08
ATOM H5'    HN8     0.09
ATOM H5''   HN8     0.09
GROUP
ATOM C4'    CN7     0.16
ATOM H4'    HN7     0.09
ATOM O4'    ON6B   -0.50
ATOM C1'    CN7B    0.16
ATOM H1'    HN7     0.09
GROUP
ATOM C2'    CN7B    0.14
ATOM H2''   HN7     0.09
ATOM O2'    ON5    -0.66
ATOM H2'    HN5     0.43
GROUP
ATOM C3'    CN7     0.01
ATOM H3'    HN7     0.09
ATOM O3'    ON2    -0.57
BOND N1   C2    N1   C6     C2   O2     C2   N3
BOND N3   C4    N3   H3     C4   O4     C4   C5
BOND C5   C6    C5   C7     C6   H6     C7   H71
BOND C7   H72   C7   N8     N8   H82    N8   C9
BOND N8   H81   C9   H91    C9   H92    C9   C10
BOND C10  O11   C10  O12
BOND P    O1P     P    O2P     P    O5'     O5'  C5'     C5'  H5''
BOND C5'  C4'     C4'  O4'     C4'  C3'     O4'  C1'
BOND C1'  N1      C1'  C2'     C2'  C3'     C3'  O3'     O3'  +P
BOND C2'  O2'     O2'  H2'
BOND C1'  H1'     C2'  H2''    C3'  H3'     C4'  H4'     C5'  H5'
IMPR C2     N1     N3     O2     C4     C5     N3     O4     C10    O11    O12    C9
!ribose
IC -O3' P    O5'  C5'    1.6001  101.45  -39.25  119.00  1.4401
IC -O3' O5'  *P   O1P    1.6001  101.45 -115.82  109.74  1.4802
IC -O3' O5'  *P   O2P    1.6001  101.45  115.90  109.80  1.4801
IC  P   O5'  C5'  C4'    1.5996  119.00 -151.39  110.04  1.5160
IC O5'  C5'  C4'  C3'    1.4401  108.83 -179.85  116.10  1.5284
IC C5'  C4'  C3'  O3'    1.5160  116.10   76.70  115.12  1.4212
IC C4'  C3'  O3'  +P     1.5284  111.92  159.13  119.05  1.6001
IC C3'  O3'  +P   +O5'   1.4212  119.05  -98.86  101.45  1.5996
IC O4'  C3'  *C4' C5'    1.4572  104.06 -120.04  116.10  1.5160
```

```
IC C2'  C4'  *C3'  O3'    1.5284  100.16 -124.08  115.12  1.4212
IC C4'  C3'  C2'   C1'    1.5284  100.16   39.58  102.04  1.5251
IC C3'  C2'  C1'   N1     1.5284  101.97  144.39  113.71  1.4896
IC O4'  C1'  N1    C2     1.5251  113.71  -96.0   117.06  1.3746
IC C1'  C3'  *C2'  O2'    1.5284  102.04 -114.67  110.81  1.4212
IC H2'  O2'  C2'   C3'    0.9600  114.97  148.63  111.92  1.5284
IC O4'  C2'  *C1'  H1'    0.0       0.0  -115.0     0.0   0.0
IC C1'  C3'  *C2'  H2''   0.0       0.0   115.0     0.0   0.0
IC C2'  C4'  *C3'  H3'    0.0       0.0   115.0     0.0   0.0
IC C3'  O4'  *C4'  H4'    0.0       0.0  -115.0     0.0   0.0
IC C4'  O5'  *C5'  H5'    0.0       0.0  -115.0     0.0   0.0
IC C4'  O5'  *C5'  H5''   0.0       0.0   115.0     0.0   0.0
IC C2   C6   *N1   C1'       1.3966  121.57  179.97  122.49  1.4896
IC C3'  C2'  C1'   N1     1.5284  101.97  144.39  113.71  1.4896
IC O4'  C1'  N1    C2     1.5251  113.71  -96.0   117.06  1.3746
IC C1'  C2   *N1   C6     1.3966  121.57  179.97  122.49  1.4896
IC C6   N1   C2    N3        1.3690  120.32   -0.64  116.29  1.3675
IC N3   N1   *C2   O2        1.3675  116.29 -179.80  123.20  1.2280
IC N1   C2   N3    C4        1.4086  116.29   -0.41  125.89  1.3741
IC C4   C2   *N3   H3        1.3741  125.89 -178.68  116.23  1.0014
IC C2   N3   C4    C5        1.3675  125.89    1.81  116.64  1.4450
IC C5   N3   *C4   O4        1.4450  116.64 -177.87  118.52  1.2288
IC C5   N1   *C6   H6        1.3774  122.95  179.71  116.21  1.0944
IC C6   C4   *C5   C7        1.3774  117.88 -179.98  117.96  1.4771
IC C4   C5   C7    N8        1.4450  117.96   67.18  106.85  1.4910
IC N8   C5   *C7   H71       1.4910  106.85 -117.44  112.03  1.1037
IC H71  C5   *C7   H72       1.1037  112.03 -126.80  111.28  1.1063
IC C5   C7   N8    C9        1.4771  106.85  165.57  114.72  1.5267
IC C9   C7   *N8   H82       1.5267  114.72 -114.75  107.77  1.0182
IC H82  C7   *N8   H81       1.0182  107.77 -117.08  111.26  1.0087
IC C7   N8   C9    C10       1.4910  114.72  -83.88  110.30  1.5371
IC C10  N8   *C9   H91       1.5371  110.30  121.01  106.94  1.0951
IC H91  N8   *C9   H92       1.0951  106.94  120.09  106.41  1.0965
IC N8   C9   C10   O11       1.5267  110.30  176.78  114.55  1.2509
IC O11  C9   *C10  O12       1.2509  114.55  179.88  116.86  1.2620
DONO H2'  O2'
DONO H3   N3
DONO H81  N8
DONO H82  N8
ACCE O2   C2
ACCE O4   C4
ACCE O11  C10
ACCE O12  C10
ACCE O1P  P
ACCE O2P  P
ACCE O2'
ACCE O3'
ACCE O4'
ACCE O5'

RESI MAU           -1.00 ! 5-carboxymethylaminomethyl-2'-O-methyluridine
GROUP
ATOM N1     NG2R61 -0.34 !    O11  O12 (-)
ATOM C2     CG2R63  0.51 !      \\ /
ATOM O2     OG2D4  -0.41 !       C10
ATOM N3     NG2R61 -0.46 !       |   H81 H72  O4
ATOM H3     HGP1    0.36 !       |   |(+) |   ||
ATOM C4     CG2R63  0.50 !   H92-C9 -N8 -C7   C4    H3
ATOM O4     OG2D4  -0.45 !       |   |   / \ /  \  /
ATOM C5     CG2R62 -0.05 !      H91 H82 H71 C5   N3
ATOM C6     CG2R62  0.17 !                  ||   |
ATOM H6     HGR62   0.17 !              H6-C6   C2
GROUP                    !                    \  / \\
```

```
ATOM C7     CG324   0.04 !                    N1   O2
ATOM H71    HGA2    0.09 !                     \
ATOM H72    HGA2    0.09 !                      \
ATOM N8     NG3P2  -0.34 !                       \
ATOM H81    HGP2    0.34 !   O1P    H5' H4'  O4'  \
ATOM H82    HGP2    0.34 !    |      |    \ /   \  \
ATOM C9     CG324  -0.01 !   -P-O5'-C5'---C4'    C1'
ATOM H91    HGA2    0.09 !    |      |     \     / \
ATOM H92    HGA2    0.09 !   O2P    H5''   C3'--C2' H1'
ATOM C10    CG2O3   0.59 !                / \   / \
ATOM O11    OG2D2  -0.66 !              O3' H3' O2' H2''
ATOM O12    OG2D2  -0.66 !               |      |
GROUP                    !                     CM2
ATOM P      P       1.50 !                   /  |  \
ATOM O1P    ON3    -0.78 !                 HM1 HM2 HM3
ATOM O2P    ON3    -0.78
ATOM O5'    ON2    -0.57 !!! PATCH 5UHG for the non-ionic tautomer
ATOM C5'    CN8B   -0.08
ATOM H5'    HN8     0.09
ATOM H5''   HN8     0.09
GROUP
ATOM C4'    CN7     0.16
ATOM H4'    HN7     0.09
ATOM O4'    ON6B   -0.50
ATOM C1'    CN7B    0.16
ATOM H1'    HN7     0.09
GROUP
ATOM C2'    CN7B    0.08
ATOM H2''   HN7     0.09
ATOM O2'    OG301  -0.34
ATOM CM2    CG331  -0.10
ATOM HM1    HGA3    0.09
ATOM HM2    HGA3    0.09
ATOM HM3    HGA3    0.09
GROUP
ATOM C3'    CN7     0.01
ATOM H3'    HN7     0.09
ATOM O3'    ON2    -0.57
BOND N1   C2    N1   C6     C2   O2     C2   N3
BOND N3   C4    N3   H3     C4   O4     C4   C5
BOND C5   C6    C5   C7     C6   H6     C7   H71
BOND C7   H72   C7   N8     N8   H82    N8   C9
BOND N8   H81   C9   H91    C9   H92    C9   C10
BOND C10  O11   C10  O12
BOND P    O1P     P    O2P     P    O5'     O5'  C5'     C5'  H5''
BOND C5'  C4'     C4'  O4'     C4'  C3'     O4'  C1'
BOND C1'  N1      C1'  C2'     C2'  C3'     C3'  O3'     O3'  +P
BOND C2'  O2'     CM2  O2'     CM2  HM1     HM2  CM2     HM3  CM2
BOND C1'  H1'     C2'  H2''    C3'  H3'     C4'  H4'     C5'  H5'
IMPR C2     N1     N3     O2     C4     C5     N3     O4     C10    O11    O12    C9
!2OM-ribose
IC -O3' P    O5'   C5'    1.6001  101.45  -39.25  119.00  1.4401
IC -O3' O5'  *P    O1P    1.6001  101.45 -115.82  109.74  1.4802
IC -O3' O5'  *P    O2P    1.6001  101.45  115.90  109.80  1.4801
IC  P   O5'  C5'   C4'    1.5996  119.00 -151.39  110.04  1.5160
IC O5'  C5'  C4'   C3'    1.4401  108.83 -179.85  116.10  1.5284
IC C5'  C4'  C3'   O3'    1.5160  116.10   76.70  115.12  1.4212
IC C4'  C3'  O3'   +P     1.5284  111.92  159.13  119.05  1.6001
IC C3'  O3'  +P    +O5'   1.4212  119.05  -98.86  101.45  1.5996
IC O4'  C3'  *C4'  C5'    1.4572  104.06 -120.04  116.10  1.5160
IC C2'  C4'  *C3'  O3'    1.5284  100.16 -124.08  115.12  1.4212
IC C4'  C3'  C2'   C1'    1.5284  100.16   39.58  102.04  1.5251
IC C3'  C2'  C1'   N1     1.5284  101.97  144.39  113.71  1.4896
```

```
IC O4'  C1'  N1    C2      1.5251  113.71  -96.0   117.06  1.3746
IC C3'  C1'  *C2'  O2'     1.5312  102.03  117.61  107.13  1.4206
IC C1'  C2'  O2'   CM2     1.5393  107.13   90.00  107.00  1.4150
IC C2'  O2'  CM2   HM2     1.4206  107.00  180.00    0.0   0.0
IC HM2  O2'  *CM2  HM3     0.0       0.0   120.00    0.0   0.0
IC HM2  O2'  *CM2  HM1     0.0       0.0  -120.00    0.0   0.0
IC O4'  C2'  *C1'  H1'     0.0       0.0  -115.0     0.0   0.0
IC C1'  C3'  *C2'  H2''    0.0       0.0   115.0     0.0   0.0
IC C2'  C4'  *C3'  H3'     0.0       0.0   115.0     0.0   0.0
IC C3'  O4'  *C4'  H4'     0.0       0.0  -115.0     0.0   0.0
IC C4'  O5'  *C5'  H5'     0.0       0.0  -115.0     0.0   0.0
IC C4'  O5'  *C5'  H5''    0.0       0.0   115.0     0.0   0.0
IC C2   C6   *N1   C1'        1.3966  121.57  179.97  122.49  1.4896
IC C3'  C2'  C1'   N1      1.5284  101.97  144.39  113.71  1.4896
IC O4'  C1'  N1    C2      1.5251  113.71  -96.0   117.06  1.3746
IC C1'  C2   *N1   C6      1.3966  121.57  179.97  122.49  1.4896
IC C6   N1   C2    N3         1.3690  120.32   -0.64  116.29  1.3675
IC N3   N1   *C2   O2         1.3675  116.29 -179.80  123.20  1.2280
IC N1   C2   N3    C4         1.4086  116.29   -0.41  125.89  1.3741
IC C4   C2   *N3   H3         1.3741  125.89 -178.68  116.23  1.0014
IC C2   N3   C4    C5         1.3675  125.89    1.81  116.64  1.4450
IC C5   N3   *C4   O4         1.4450  116.64 -177.87  118.52  1.2288
IC C5   N1   *C6   H6         1.3774  122.95  179.71  116.21  1.0944
IC C6   C4   *C5   C7         1.3774  117.88 -179.98  117.96  1.4771
IC C4   C5   C7    N8         1.4450  117.96   67.18  106.85  1.4910
IC N8   C5   *C7   H71        1.4910  106.85 -117.44  112.03  1.1037
IC H71  C5   *C7   H72        1.1037  112.03 -126.80  111.28  1.1063
IC C5   C7   N8    C9         1.4771  106.85  165.57  114.72  1.5267
IC C9   C7   *N8   H82        1.5267  114.72 -114.75  107.77  1.0182
IC H82  C7   *N8   H81        1.0182  107.77 -117.08  111.26  1.0087
IC C7   N8   C9    C10        1.4910  114.72  -83.88  110.30  1.5371
IC C10  N8   *C9   H91        1.5371  110.30  121.01  106.94  1.0951
IC H91  N8   *C9   H92        1.0951  106.94  120.09  106.41  1.0965
IC N8   C9   C10   O11        1.5267  110.30  176.78  114.55  1.2509
IC O11  C9   *C10  O12        1.2509  114.55  179.88  116.86  1.2620
DONO H3   N3
DONO H81  N8
DONO H82  N8
ACCE O2   C2
ACCE O4   C4
ACCE O11  C10
ACCE O12  C10
ACCE O1P  P
ACCE O2P  P
ACCE O2'
ACCE O3'
ACCE O4'
ACCE O5'

RESI SCU           -1.00 ! 5-carboxymethylaminomethyl-2-thiouridine
GROUP
ATOM N1     NG2R61 -0.26 !   O11  O12 (-)
ATOM C2     CG2R63  0.29 !      \\ /
ATOM S2     SG2D1  -0.22 !       C10
ATOM N3     NG2R61 -0.56 !       |   H82 H72  O4
ATOM H3     HGP1    0.40 !       |   |(+) |    ||
ATOM C4     CG2R63  0.39 !  H92-C9 -N8 -C7    C4    H3
ATOM O4     OG2D4  -0.41 !       |   |   / \ /  \  /
ATOM C5     CG2R62 -0.03 !     H91 H81 H71 C5   N3
ATOM C6     CG2R62  0.18 !                 ||   |
ATOM H6     HGR62   0.22 !              H6-C6   C2
GROUP                    !                  \  / \\
ATOM C7     CG324   0.04 !                   N1   S2
```

```
ATOM H71    HGA2    0.09 !                     \
ATOM H72    HGA2    0.09 !                      \
ATOM N8     NG3P2  -0.34 !                       \
ATOM H81    HGP2    0.34 !  O1P    H5' H4'  O4'  \
ATOM H82    HGP2    0.34 !   |      |    \ /   \  \
ATOM C9     CG324  -0.01 !  -P-O5'-C5'---C4'    C1'
ATOM H91    HGA2    0.09 !   |      |     \     / \
ATOM H92    HGA2    0.09 !  O2P    H5''   C3'--C2' H1'
ATOM C10    CG2O3   0.59 !               / \   / \
ATOM O11    OG2D2  -0.66 !             O3' H3' O2' H2''
ATOM O12    OG2D2  -0.66 !              |       |
GROUP                    !                      H2'
ATOM P      P       1.50
ATOM O1P    ON3    -0.78 !!! PATCH 5UHG for the non-ionic tautomer
ATOM O2P    ON3    -0.78
ATOM O5'    ON2    -0.57
ATOM C5'    CN8B   -0.08
ATOM H5'    HN8     0.09
ATOM H5''   HN8     0.09
GROUP
ATOM C4'    CN7     0.16
ATOM H4'    HN7     0.09
ATOM O4'    ON6B   -0.50
ATOM C1'    CN7B    0.16
ATOM H1'    HN7     0.09
GROUP
ATOM C2'    CN7B    0.14
ATOM H2''   HN7     0.09
ATOM O2'    ON5    -0.66
ATOM H2'    HN5     0.43
GROUP
ATOM C3'    CN7     0.01
ATOM H3'    HN7     0.09
ATOM O3'    ON2    -0.57
BOND N1   C2     N1   C6      C2   S2      C2   N3
BOND N3   C4     N3   H3      C4   O4      C4   C5
BOND C5   C6     C5   C7      C6   H6      C7   H71
BOND C7   H72    C7   N8      N8   H82     N8   C9
BOND N8   H81    C9   H91     C9   H92     C9   C10
BOND C10  O11    C10  O12
BOND P    O1P     P    O2P     P    O5'     O5'  C5'     C5'  H5''
BOND C5'  C4'     C4'  O4'     C4'  C3'     O4'  C1'
BOND C1'  N1      C1'  C2'     C2'  C3'     C3'  O3'     O3'  +P
BOND C2'  O2'     O2'  H2'
BOND C1'  H1'     C2'  H2''    C3'  H3'     C4'  H4'     C5'  H5'
IMPR C2     N1     N3     S2      C4     C5     N3     O4     C10    O11    O12    C9
!ribose
IC -O3' P    O5'  C5'    1.6001  101.45  -39.25  119.00  1.4401
IC -O3' O5'  *P   O1P    1.6001  101.45 -115.82  109.74  1.4802
IC -O3' O5'  *P   O2P    1.6001  101.45  115.90  109.80  1.4801
IC  P   O5'  C5'  C4'    1.5996  119.00 -151.39  110.04  1.5160
IC O5'  C5'  C4'  C3'    1.4401  108.83 -179.85  116.10  1.5284
IC C5'  C4'  C3'  O3'    1.5160  116.10   76.70  115.12  1.4212
IC C4'  C3'  O3'  +P     1.5284  111.92  159.13  119.05  1.6001
IC C3'  O3'  +P   +O5'   1.4212  119.05  -98.86  101.45  1.5996
IC O4'  C3'  *C4' C5'    1.4572  104.06 -120.04  116.10  1.5160
IC C2'  C4'  *C3' O3'    1.5284  100.16 -124.08  115.12  1.4212
IC C4'  C3'  C2'  C1'    1.5284  100.16   39.58  102.04  1.5251
IC C3'  C2'  C1'  N1     1.5284  101.97  144.39  113.71  1.4896
IC O4'  C1'  N1   C2     1.5251  113.71  -96.0   117.06  1.3746
IC C1'  C3'  *C2' O2'    1.5284  102.04 -114.67  110.81  1.4212
IC H2'  O2'  C2'  C3'    0.9600  114.97  148.63  111.92  1.5284
IC O4'  C2'  *C1' H1'    0.0       0.0  -115.0     0.0   0.0
```

```
IC C1'  C3'  *C2'  H2''   0.0       0.0   115.0     0.0   0.0
IC C2'  C4'  *C3'  H3'    0.0       0.0   115.0     0.0   0.0
IC C3'  O4'  *C4'  H4'    0.0       0.0  -115.0     0.0   0.0
IC C4'  O5'  *C5'  H5'    0.0       0.0  -115.0     0.0   0.0
IC C4'  O5'  *C5'  H5''   0.0       0.0   115.0     0.0   0.0
IC C2   C6   *N1   C1'       1.3966  121.57  179.97  122.49  1.4896
IC C3'  C2'  C1'   N1     1.5284  101.97  144.39  113.71  1.4896
IC O4'  C1'  N1    C2     1.5251  113.71  -96.0   117.06  1.3746
IC C1'  C2   *N1   C6     1.3966  121.57  179.97  122.49  1.4896
IC C6   N1   C2    N3        1.3733  121.46   -2.03  113.69  1.3910
IC N3   N1   *C2   S2        1.3910  113.69 -179.36  125.75  1.6502
IC N1   C2   N3    C4        1.4174  113.69   -0.23  127.36  1.3809
IC C4   C2   *N3   H3        1.3809  127.36 -177.30  117.42  1.0065
IC C2   N3   C4    C5        1.3910  127.36    4.30  116.16  1.4387
IC C5   N3   *C4   O4        1.4387  116.16 -178.34  118.68  1.2302
IC C5   N1   *C6   H6        1.3770  123.16  175.65  116.13  1.0953
IC C6   C4   *C5   C7        1.3770  117.90 -173.50  117.03  1.4771
IC C4   C5   C7    N8        1.4387  117.03   77.83  105.54  1.4957
IC N8   C5   *C7   H71       1.4957  105.54 -117.69  112.73  1.1030
IC H71  C5   *C7   H72       1.1030  112.73 -126.83  110.51  1.1028
IC C5   C7   N8    C9        1.4771  105.54  135.65  114.81  1.5290
IC C9   C7   *N8   H82       1.5290  114.81 -115.59  107.22  1.0157
IC H82  C7   *N8   H81       1.0157  107.22 -115.58  111.17  1.0066
IC C7   N8   C9    C10       1.4957  114.81  -73.24  111.27  1.5390
IC C10  N8   *C9   H91       1.5390  111.27  121.18  106.68  1.0956
IC H91  N8   *C9   H92       1.0956  106.68  119.54  106.23  1.0972
IC N8   C9   C10   O11       1.5290  111.27  178.06  114.32  1.2501
IC O11  C9   *C10  O12       1.2501  114.32 -179.68  117.37  1.2618
DONO H2'  O2'
DONO H3   N3
DONO H81  N8
DONO H82  N8
ACCE O4   C4
ACCE S2   C2
ACCE O11  C10
ACCE O12  C10
ACCE O1P  P
ACCE O2P  P
ACCE O2'
ACCE O3'
ACCE O4'
ACCE O5'

RESI IAU            0.00 ! 5-(isopentenylaminomethyl)uridine
GROUP
ATOM N1     NG2R61 -0.34 !      H132 H133
ATOM C2     CG2R63  0.51 !         \ /
ATOM O2     OG2D4  -0.41 !   H131-C13  H121 H122
ATOM N3     NG2R61 -0.46 !          |    | /
ATOM H3     HGP1    0.36 !          C11-C12-H123
ATOM C4     CG2R63  0.50 !         //
ATOM O4     OG2D4  -0.45 !   H10-C10
ATOM C5     CG2R62 -0.05 !       |  H81  H72  O4
ATOM C6     CG2R62  0.17 !       |   |(+) |   ||
ATOM H6     HGR62   0.17 !   H92-C9 -N8 -C7   C4    H3
GROUP                    !       |   |   / \ /  \  /
ATOM C7     CG324   0.20 !      H91 H82 H71 C5   N3
ATOM H71    HGA2    0.09 !                  ||   |
ATOM H72    HGA2    0.09 !               H6-C6   C2
ATOM N8     NG3P2  -0.52 !                   \  / \\
ATOM H82    HGP2    0.38 !                    N1   O2
ATOM H81    HGP2    0.38 !                     \
ATOM C9     CG324   0.20 !                      \
```

```
ATOM H91    HGA2    0.09 !                       \
ATOM H92    HGA2    0.09 !   O1P    H5' H4'  O4'  \
GROUP                    !    |      |    \ /   \  \
ATOM C10    CG2D1  -0.15 !   -P-O5'-C5'---C4'    C1'
ATOM H10    HGA4    0.15 !    |      |     \     / \
GROUP                    !   O2P    H5''   C3'--C2' H1'
ATOM C11    CG2D1   0.00 !                / \   / \
ATOM C12    CG331  -0.27 !              O3' H3' O2' H2''
ATOM H121   HGA3    0.09 !               |       |
ATOM H122   HGA3    0.09 !                      H2'
ATOM H123   HGA3    0.09
GROUP                    !  !!!! PATCH 5UNI for the neutral amino form
ATOM C13    CG331  -0.27
ATOM H131   HGA3    0.09
ATOM H132   HGA3    0.09
ATOM H133   HGA3    0.09
GROUP
ATOM P      P       1.50
ATOM O1P    ON3    -0.78
ATOM O2P    ON3    -0.78
ATOM O5'    ON2    -0.57
ATOM C5'    CN8B   -0.08
ATOM H5'    HN8     0.09
ATOM H5''   HN8     0.09
GROUP
ATOM C4'    CN7     0.16
ATOM H4'    HN7     0.09
ATOM O4'    ON6B   -0.50
ATOM C1'    CN7B    0.16
ATOM H1'    HN7     0.09
GROUP
ATOM C2'    CN7B    0.14
ATOM H2''   HN7     0.09
ATOM O2'    ON5    -0.66
ATOM H2'    HN5     0.43
GROUP
ATOM C3'    CN7     0.01
ATOM H3'    HN7     0.09
ATOM O3'    ON2    -0.57
BOND N1   C2     N1   C6      C2   O2      C2   N3
BOND N3   C4     N3   H3      C4   O4      C4   C5
BOND C5   C6     C5   C7      C6   H6      C7   H71
BOND C7   H72    C7   N8      C9   C10     C9   H91
BOND C9   H92    C9   N8      C10  H10     C10  C11
BOND N8   H81    N8   H82     C11  C12     C11  C13
BOND C12  H121   C12  H122    C12  H123    C13  H131
BOND C13  H132   C13  H133
BOND P    O1P     P    O2P     P    O5'     O5'  C5'     C5'  H5''
BOND C5'  C4'     C4'  O4'     C4'  C3'     O4'  C1'
BOND C1'  N1      C1'  C2'     C2'  C3'     C3'  O3'     O3'  +P
BOND C2'  O2'     O2'  H2'
BOND C1'  H1'     C2'  H2''    C3'  H3'     C4'  H4'     C5'  H5'
IMPR C2     N1     N3     O2     C4     C5     N3     O4
!ribose
IC -O3' P    O5'   C5'    1.6001  101.45  -39.25  119.00  1.4401
IC -O3' O5'  *P    O1P    1.6001  101.45 -115.82  109.74  1.4802
IC -O3' O5'  *P    O2P    1.6001  101.45  115.90  109.80  1.4801
IC  P   O5'  C5'   C4'    1.5996  119.00 -151.39  110.04  1.5160
IC O5'  C5'  C4'   C3'    1.4401  108.83 -179.85  116.10  1.5284
IC C5'  C4'  C3'   O3'    1.5160  116.10   76.70  115.12  1.4212
IC C4'  C3'  O3'   +P     1.5284  111.92  159.13  119.05  1.6001
IC C3'  O3'  +P    +O5'   1.4212  119.05  -98.86  101.45  1.5996
IC O4'  C3'  *C4'  C5'    1.4572  104.06 -120.04  116.10  1.5160
```

```
IC C2'  C4'  *C3'  O3'    1.5284  100.16 -124.08  115.12  1.4212
IC C4'  C3'  C2'   C1'    1.5284  100.16   39.58  102.04  1.5251
IC C3'  C2'  C1'   N1     1.5284  101.97  144.39  113.71  1.4896
IC O4'  C1'  N1    C2     1.5251  113.71  -96.0   117.06  1.3746
IC C1'  C3'  *C2'  O2'    1.5284  102.04 -114.67  110.81  1.4212
IC H2'  O2'  C2'   C3'    0.9600  114.97  148.63  111.92  1.5284
IC O4'  C2'  *C1'  H1'    0.0       0.0  -115.0     0.0   0.0
IC C1'  C3'  *C2'  H2''   0.0       0.0   115.0     0.0   0.0
IC C2'  C4'  *C3'  H3'    0.0       0.0   115.0     0.0   0.0
IC C3'  O4'  *C4'  H4'    0.0       0.0  -115.0     0.0   0.0
IC C4'  O5'  *C5'  H5'    0.0       0.0  -115.0     0.0   0.0
IC C4'  O5'  *C5'  H5''   0.0       0.0   115.0     0.0   0.0
IC C2   C6   *N1   C1'       1.3966  121.57  179.97  122.49  1.4896
IC C3'  C2'  C1'   N1     1.5284  101.97  144.39  113.71  1.4896
IC O4'  C1'  N1    C2     1.5251  113.71  -96.0   117.06  1.3746
IC C1'  C2   *N1   C6     1.3966  121.57  179.97  122.49  1.4896
IC C6   N1   C2    N3        1.3643  120.51    0.01  116.07  1.3664
IC N3   N1   *C2   O2        1.3664  116.07  179.98  123.21  1.2260
IC N1   C2   N3    C4        1.4088  116.07   -0.07  125.99  1.3718
IC C4   C2   *N3   H3        1.3718  125.99 -179.74  116.12  1.0042
IC C2   N3   C4    C5        1.3664  125.99    0.22  116.98  1.4445
IC C5   N3   *C4   O4        1.4445  116.98  179.99  118.61  1.2265
IC C5   N1   *C6   H6        1.3842  123.10  179.82  114.24  1.0908
IC C6   C4   *C5   C7        1.3842  117.34 -179.58  111.49  1.4824
IC C4   C5   C7    N8        1.4445  111.49 -178.94  111.02  1.4877
IC N8   C5   *C7   H71       1.4877  111.02  118.04  110.50  1.1063
IC H71  C5   *C7   H72       1.1063  110.50  123.87  110.41  1.1071
IC C5   C7   N8    C9        1.4824  111.02 -179.30  115.04  1.5130
IC C9   C7   *N8   H81       1.5130  115.04  120.78  110.60  1.0103
IC C9   C7   *N8   H82       1.5130  115.04 -122.04  110.56  1.0093
IC C7   N8   C9    C10       1.4877  115.04  179.14  110.09  1.4994
IC C10  N8   *C9   H91       1.4994  110.09  120.72  106.01  1.1045
IC C10  N8   *C9   H92       1.4994  110.09 -122.82  105.82  1.1053
IC N8   C9   C10   C11       1.5130  110.09  -91.35  126.75  1.3475
IC C11  C9   *C10  H10       1.3475  126.75 -173.90  116.16  1.1024
IC C9   C10  C11   C12       1.4994  126.75 -178.89  120.81  1.5034
IC C12  C10  *C11  C13       1.5034  120.81 -179.48  125.27  1.5071
IC C10  C11  C12   H121      1.3475  120.81  120.68  110.59  1.1127
IC H121 C11  *C12  H122      1.1127  110.59  118.59  110.98  1.1120
IC H121 C11  *C12  H123      1.1127  110.59 -120.33  113.70  1.1135
IC C10  C11  C13   H131      1.3475  125.27 -120.28  109.97  1.1127
IC H131 C11  *C13  H132      1.1127  109.97  120.85  115.43  1.1099
IC H131 C11  *C13  H133      1.1127  109.97 -117.21  110.45  1.1124
DONO H2'  O2'
DONO H3   N3
DONO H81  N8
DONO H82  N8
ACCE O2   C2
ACCE O4   C4
ACCE O1P  P
ACCE O2P  P
ACCE O2'
ACCE O3'
ACCE O4'
ACCE O5'

RESI MIU            0.00 ! 5-(isopentenylaminomethyl)-2'-O-methyluridine
GROUP
ATOM N1     NG2R61 -0.34 !      H132 H133
ATOM C2     CG2R63  0.51 !         \ /
ATOM O2     OG2D4  -0.41 !   H131-C13  H121 H122
ATOM N3     NG2R61 -0.46 !         |    | /
ATOM H3     HGP1    0.36 !         C11-C12-H123
```

```
ATOM C4     CG2R63  0.50 !        //
ATOM O4     OG2D4  -0.45 !   H10-C10
ATOM C5     CG2R62 -0.05 !       |  H81  H72  O4
ATOM C6     CG2R62  0.17 !       |   |(+) |   ||
ATOM H6     HGR62   0.17 !   H92-C9 -N8 -C7   C4    H3
GROUP                    !       |   |   / \ /  \  /
ATOM C7     CG324   0.20 !      H91 H82 H71 C5   N3
ATOM H71    HGA2    0.09 !                  ||   |
ATOM H72    HGA2    0.09 !               H6-C6   C2
ATOM N8     NG3P2  -0.52 !                   \  / \\
ATOM H82    HGP2    0.38 !                    N1   O2
ATOM H81    HGP2    0.38 !                     \
ATOM C9     CG324   0.20 !                      \
ATOM H91    HGA2    0.09 !                       \
ATOM H92    HGA2    0.09 !   O1P    H5' H4'  O4'  \
GROUP                    !    |      |    \ /   \  \
ATOM C10    CG2D1  -0.15 !   -P-O5'-C5'---C4'    C1'
ATOM H10    HGA4    0.15 !    |      |     \     / \
GROUP                    !   O2P    H5''   C3'--C2' H1'
ATOM C11    CG2D1   0.00 !                / \   / \
ATOM C12    CG331  -0.27 !              O3' H3' O2' H2''
ATOM H121   HGA3    0.09 !               |       |
ATOM H122   HGA3    0.09 !                      CM2
ATOM H123   HGA3    0.09 !                    /  |  \
GROUP                    !                  HM1 HM2 HM3
ATOM C13    CG331  -0.27
ATOM H131   HGA3    0.09 !  !!!! PATCH 5UNI for the neutral amino form
ATOM H132   HGA3    0.09
ATOM H133   HGA3    0.09
GROUP
ATOM P      P       1.50
ATOM O1P    ON3    -0.78
ATOM O2P    ON3    -0.78
ATOM O5'    ON2    -0.57
ATOM C5'    CN8B   -0.08
ATOM H5'    HN8     0.09
ATOM H5''   HN8     0.09
GROUP
ATOM C4'    CN7     0.16
ATOM H4'    HN7     0.09
ATOM O4'    ON6B   -0.50
ATOM C1'    CN7B    0.16
ATOM H1'    HN7     0.09
GROUP
ATOM C2'    CN7B    0.08
ATOM H2''   HN7     0.09
ATOM O2'    OG301  -0.34
ATOM CM2    CG331  -0.10
ATOM HM1    HGA3    0.09
ATOM HM2    HGA3    0.09
ATOM HM3    HGA3    0.09
GROUP
ATOM C3'    CN7     0.01
ATOM H3'    HN7     0.09
ATOM O3'    ON2    -0.57
BOND N1   C2     N1   C6     C2   O2     C2   N3
BOND N3   C4     N3   H3     C4   O4     C4   C5
BOND C5   C6     C5   C7     C6   H6     C7   H71
BOND C7   H72    C7   N8     C9   C10    C9   H91
BOND C9   H92    C9   N8     C10  H10    C10  C11
BOND N8   H81    N8   H82    C11  C12    C11  C13
BOND C12  H121   C12  H122   C12  H123   C13  H131
BOND C13  H132   C13  H133
```

```
BOND P    O1P     P    O2P     P    O5'     O5'  C5'     C5'  H5''
BOND C5'  C4'     C4'  O4'     C4'  C3'     O4'  C1'
BOND C1'  N1      C1'  C2'     C2'  C3'     C3'  O3'     O3'  +P
BOND C2'  O2'     CM2  O2'     CM2  HM1     HM2  CM2     HM3  CM2
BOND C1'  H1'     C2'  H2''    C3'  H3'     C4'  H4'     C5'  H5'
IMPR C2     N1     N3     O2     C4     C5     N3     O4
!2OM-ribose
IC -O3' P    O5'  C5'    1.6001  101.45  -39.25  119.00  1.4401
IC -O3' O5'  *P   O1P    1.6001  101.45 -115.82  109.74  1.4802
IC -O3' O5'  *P   O2P    1.6001  101.45  115.90  109.80  1.4801
IC  P   O5'  C5'  C4'    1.5996  119.00 -151.39  110.04  1.5160
IC O5'  C5'  C4'  C3'    1.4401  108.83 -179.85  116.10  1.5284
IC C5'  C4'  C3'  O3'    1.5160  116.10   76.70  115.12  1.4212
IC C4'  C3'  O3'  +P     1.5284  111.92  159.13  119.05  1.6001
IC C3'  O3'  +P   +O5'   1.4212  119.05  -98.86  101.45  1.5996
IC O4'  C3'  *C4' C5'    1.4572  104.06 -120.04  116.10  1.5160
IC C2'  C4'  *C3' O3'    1.5284  100.16 -124.08  115.12  1.4212
IC C4'  C3'  C2'  C1'    1.5284  100.16   39.58  102.04  1.5251
IC C3'  C2'  C1'  N1     1.5284  101.97  144.39  113.71  1.4896
IC O4'  C1'  N1   C2     1.5251  113.71  -96.0   117.06  1.3746
IC C3'  C1'  *C2' O2'    1.5312  102.03  117.61  107.13  1.4206
IC C1'  C2'  O2'  CM2    1.5393  107.13   90.00  107.00  1.4150
IC C2'  O2'  CM2  HM2    1.4206  107.00  180.00    0.0   0.0
IC HM2  O2'  *CM2 HM3    0.0       0.0   120.00    0.0   0.0
IC HM2  O2'  *CM2 HM1    0.0       0.0  -120.00    0.0   0.0
IC O4'  C2'  *C1' H1'    0.0       0.0  -115.0     0.0   0.0
IC C1'  C3'  *C2' H2''   0.0       0.0   115.0     0.0   0.0
IC C2'  C4'  *C3' H3'    0.0       0.0   115.0     0.0   0.0
IC C3'  O4'  *C4' H4'    0.0       0.0  -115.0     0.0   0.0
IC C4'  O5'  *C5' H5'    0.0       0.0  -115.0     0.0   0.0
IC C4'  O5'  *C5' H5''   0.0       0.0   115.0     0.0   0.0
IC C2   C6   *N1  C1'      1.3966  121.57  179.97  122.49  1.4896
IC C3'  C2'  C1'  N1     1.5284  101.97  144.39  113.71  1.4896
IC O4'  C1'  N1   C2     1.5251  113.71  -96.0   117.06  1.3746
IC C1'  C2   *N1  C6     1.3966  121.57  179.97  122.49  1.4896
IC C6   N1   C2   N3       1.3643  120.51    0.01  116.07  1.3664
IC N3   N1   *C2  O2       1.3664  116.07  179.98  123.21  1.2260
IC N1   C2   N3   C4       1.4088  116.07   -0.07  125.99  1.3718
IC C4   C2   *N3  H3       1.3718  125.99 -179.74  116.12  1.0042
IC C2   N3   C4   C5       1.3664  125.99    0.22  116.98  1.4445
IC C5   N3   *C4  O4       1.4445  116.98  179.99  118.61  1.2265
IC C5   N1   *C6  H6       1.3842  123.10  179.82  114.24  1.0908
IC C6   C4   *C5  C7       1.3842  117.34 -179.58  111.49  1.4824
IC C4   C5   C7   N8       1.4445  111.49 -178.94  111.02  1.4877
IC N8   C5   *C7  H71      1.4877  111.02  118.04  110.50  1.1063
IC H71  C5   *C7  H72      1.1063  110.50  123.87  110.41  1.1071
IC C5   C7   N8   C9       1.4824  111.02 -179.30  115.04  1.5130
IC C9   C7   *N8  H81      1.5130  115.04  120.78  110.60  1.0103
IC C9   C7   *N8  H82      1.5130  115.04 -122.04  110.56  1.0093
IC C7   N8   C9   C10      1.4877  115.04  179.14  110.09  1.4994
IC C10  N8   *C9  H91      1.4994  110.09  120.72  106.01  1.1045
IC C10  N8   *C9  H92      1.4994  110.09 -122.82  105.82  1.1053
IC N8   C9   C10  C11      1.5130  110.09  -91.35  126.75  1.3475
IC C11  C9   *C10 H10      1.3475  126.75 -173.90  116.16  1.1024
IC C9   C10  C11  C12      1.4994  126.75 -178.89  120.81  1.5034
IC C12  C10  *C11 C13      1.5034  120.81 -179.48  125.27  1.5071
IC C10  C11  C12  H121     1.3475  120.81  120.68  110.59  1.1127
IC H121 C11  *C12 H122     1.1127  110.59  118.59  110.98  1.1120
IC H121 C11  *C12 H123     1.1127  110.59 -120.33  113.70  1.1135
IC C10  C11  C13  H131     1.3475  125.27 -120.28  109.97  1.1127
IC H131 C11  *C13 H132     1.1127  109.97  120.85  115.43  1.1099
IC H131 C11  *C13 H133     1.1127  109.97 -117.21  110.45  1.1124
DONO H3   N3
```

```
DONO H81  N8
DONO H82  N8
ACCE O2   C2
ACCE O4   C4
ACCE O1P  P
ACCE O2P  P
ACCE O2'
ACCE O3'
ACCE O4'
ACCE O5'

RESI ISU            0.00 ! 5-(isopentenylaminomethyl)-2-thiouridine
GROUP
ATOM N1     NG2R61 -0.26 !      H132 H133
ATOM C2     CG2R63  0.29 !         \ /
ATOM S2     SG2D1  -0.22 !   H131-C13  H121 H122
ATOM N3     NG2R61 -0.56 !          |    | /
ATOM H3     HGP1    0.40 !          C11-C12-H123
ATOM C4     CG2R63  0.39 !         //
ATOM O4     OG2D4  -0.41 !   H10-C10
ATOM C5     CG2R62 -0.03 !       |  H81  H72  O4
ATOM C6     CG2R62  0.18 !       |   |(+) |   ||
ATOM H6     HGR62   0.22 !   H92-C9 -N8 -C7   C4    H3
GROUP                    !       |   |   / \ /  \  /
ATOM C7     CG324   0.20 !      H91 H82 H71 C5   N3
ATOM H71    HGA2    0.09 !                  ||   |
ATOM H72    HGA2    0.09 !               H6-C6   C2
ATOM N8     NG3P2  -0.52 !                   \  / \\
ATOM H82    HGP2    0.38 !                    N1   S2
ATOM H81    HGP2    0.38 !                     \
ATOM C9     CG324   0.20 !                      \
ATOM H91    HGA2    0.09 !                       \
ATOM H92    HGA2    0.09 !   O1P    H5' H4'  O4'  \
GROUP                    !    |      |    \ /   \  \
ATOM C10    CG2D1  -0.15 !   -P-O5'-C5'---C4'    C1'
ATOM H10    HGA4    0.15 !    |      |     \     / \
GROUP                    !   O2P    H5''   C3'--C2' H1'
ATOM C11    CG2D1   0.00 !                / \   / \
ATOM C12    CG331  -0.27 !              O3' H3' O2' H2''
ATOM H121   HGA3    0.09 !               |       |
ATOM H122   HGA3    0.09 !                      H2'
ATOM H123   HGA3    0.09
GROUP                    !  !!!! PATCH 5UNI for the neutral amino form
ATOM C13    CG331  -0.27
ATOM H131   HGA3    0.09
ATOM H132   HGA3    0.09
ATOM H133   HGA3    0.09
GROUP
ATOM P      P       1.50
ATOM O1P    ON3    -0.78
ATOM O2P    ON3    -0.78
ATOM O5'    ON2    -0.57
ATOM C5'    CN8B   -0.08
ATOM H5'    HN8     0.09
ATOM H5''   HN8     0.09
GROUP
ATOM C4'    CN7     0.16
ATOM H4'    HN7     0.09
ATOM O4'    ON6B   -0.50
ATOM C1'    CN7B    0.16
ATOM H1'    HN7     0.09
GROUP
ATOM C2'    CN7B    0.14
```

```
ATOM H2''   HN7     0.09
ATOM O2'    ON5    -0.66
ATOM H2'    HN5     0.43
GROUP
ATOM C3'    CN7     0.01
ATOM H3'    HN7     0.09
ATOM O3'    ON2    -0.57
BOND N1   C2      N1   C6      C2   S2       C2   N3
BOND N3   C4      N3   H3      C4   O4       C4   C5
BOND C5   C6      C5   C7      C6   H6       C7   H71
BOND C7   H72     C7   N8      C9   C10      C9   H91
BOND C9   H92     C9   N8      C10  H10      C10  C11
BOND N8   H81     N8   H82     C11  C12      C11  C13
BOND C12  H121    C12  H122    C12  H123     C13  H131
BOND C13  H132    C13  H133
BOND P    O1P     P    O2P     P    O5'     O5'  C5'     C5'  H5''
BOND C5'  C4'     C4'  O4'     C4'  C3'     O4'  C1'
BOND C1'  N1      C1'  C2'     C2'  C3'     C3'  O3'     O3'  +P
BOND C2'  O2'     O2'  H2'
BOND C1'  H1'     C2'  H2''    C3'  H3'     C4'  H4'     C5'  H5'
IMPR C2     N1     N3     S2     C4     C5     N3     O4
!ribose
IC -O3' P    O5'  C5'    1.6001  101.45  -39.25  119.00  1.4401
IC -O3' O5'  *P   O1P    1.6001  101.45 -115.82  109.74  1.4802
IC -O3' O5'  *P   O2P    1.6001  101.45  115.90  109.80  1.4801
IC  P   O5'  C5'  C4'    1.5996  119.00 -151.39  110.04  1.5160
IC O5'  C5'  C4'  C3'    1.4401  108.83 -179.85  116.10  1.5284
IC C5'  C4'  C3'  O3'    1.5160  116.10   76.70  115.12  1.4212
IC C4'  C3'  O3'  +P     1.5284  111.92  159.13  119.05  1.6001
IC C3'  O3'  +P   +O5'   1.4212  119.05  -98.86  101.45  1.5996
IC O4'  C3'  *C4' C5'    1.4572  104.06 -120.04  116.10  1.5160
IC C2'  C4'  *C3' O3'    1.5284  100.16 -124.08  115.12  1.4212
IC C4'  C3'  C2'  C1'    1.5284  100.16   39.58  102.04  1.5251
IC C3'  C2'  C1'  N1     1.5284  101.97  144.39  113.71  1.4896
IC O4'  C1'  N1   C2     1.5251  113.71  -96.0   117.06  1.3746
IC C1'  C3'  *C2' O2'    1.5284  102.04 -114.67  110.81  1.4212
IC H2'  O2'  C2'  C3'    0.9600  114.97  148.63  111.92  1.5284
IC O4'  C2'  *C1' H1'    0.0       0.0  -115.0     0.0   0.0
IC C1'  C3'  *C2' H2''   0.0       0.0   115.0     0.0   0.0
IC C2'  C4'  *C3' H3'    0.0       0.0   115.0     0.0   0.0
IC C3'  O4'  *C4' H4'    0.0       0.0  -115.0     0.0   0.0
IC C4'  O5'  *C5' H5'    0.0       0.0  -115.0     0.0   0.0
IC C4'  O5'  *C5' H5''   0.0       0.0   115.0     0.0   0.0
IC C2   C6   *N1  C1'       1.3966  121.57  179.97  122.49  1.4896
IC C3'  C2'  C1'  N1     1.5284  101.97  144.39  113.71  1.4896
IC O4'  C1'  N1   C2     1.5251  113.71  -96.0   117.06  1.3746
IC C1'  C2   *N1  C6     1.3966  121.57  179.97  122.49  1.4896
IC C6   N1   C2   N3        1.3695  121.68   -0.03  113.66  1.3891
IC N3   N1   *C2  S2        1.3891  113.66  179.90  125.75  1.6487
IC N1   C2   N3   C4        1.4173  113.66   -0.30  127.37  1.3797
IC C4   C2   *N3  H3        1.3797  127.37  178.93  116.84  1.0092
IC C2   N3   C4   C5        1.3891  127.37   -0.42  116.39  1.4447
IC C5   N3   *C4  O4        1.4447  116.39 -177.06  118.92  1.2299
IC C5   N1   *C6  H6        1.3785  123.31 -178.15  114.97  1.0911
IC C6   C4   *C5  C7        1.3785  117.56  175.03  117.09  1.4824
IC C4   C5   C7   N8        1.4447  117.09   55.92  108.25  1.4982
IC N8   C5   *C7  H71       1.4982  108.25  117.66  111.82  1.1030
IC H71  C5   *C7  H72       1.1030  111.82  125.94  110.34  1.1049
IC C5   C7   N8   C9        1.4824  108.25 -175.48  117.08  1.5089
IC C9   C7   *N8  H81       1.5089  117.08  121.43  106.89  1.0189
IC C9   C7   *N8  H82       1.5089  117.08 -125.08  111.23  1.0091
IC C7   N8   C9   C10       1.4982  117.08 -179.36  110.12  1.4996
IC C10  N8   *C9  H91       1.4996  110.12  123.13  105.81  1.1037
```

```
IC C10  N8   *C9   H92       1.4996  110.12 -120.37  105.82  1.1046
IC N8   C9   C10   C11       1.5089  110.12   96.29  127.07  1.3480
IC C11  C9   *C10  H10       1.3480  127.07  176.37  115.99  1.1020
IC C9   C10  C11   C12       1.4996  127.07  179.14  120.71  1.5042
IC C12  C10  *C11  C13       1.5042  120.71  179.35  125.38  1.5061
IC C10  C11  C12   H121      1.3480  120.71 -120.83  110.58  1.1125
IC H121 C11  *C12  H122      1.1125  110.58  120.45  113.65  1.1123
IC H121 C11  *C12  H123      1.1125  110.58 -118.55  110.89  1.1121
IC C10  C11  C13   H131      1.3480  125.38  119.92  110.00  1.1135
IC H131 C11  *C13  H132      1.1135  110.00  117.20  110.36  1.1131
IC H131 C11  *C13  H133      1.1135  110.00 -121.08  115.39  1.1092
DONO H3   N3
DONO H81  N8
DONO H82  N8
ACCE O4   C4
ACCE S2   C2
DONO H2'  O2'
ACCE O1P  P
ACCE O2P  P
ACCE O2'
ACCE O3'
ACCE O4'
ACCE O5'

RESI 5CU            -2.00 ! 5-carboxymethyluridine
GROUP
ATOM N1     NG2R61 -0.34 !          O81  O82(-)
ATOM C2     CG2R63  0.51 !            \\ /
ATOM O2     OG2D4  -0.41 !             C8
ATOM N3     NG2R61 -0.46 !             |    O4
ATOM H3     HGP1    0.36 !             |    ||
ATOM C4     CG2R63  0.50 !         H72-C7   C4    H3
ATOM O4     OG2D4  -0.45 !            /  \ /  \  /
ATOM C5     CG2R62 -0.05 !          H71   C5   N3
ATOM C6     CG2R62  0.17 !                ||   |
ATOM H6     HGR62   0.17 !             H6-C6   C2
GROUP                    !                 \  / \\
ATOM C7     CG321  -0.18 !                  N1   O2
ATOM H71    HGA2    0.09 !                   \
ATOM H72    HGA2    0.09 !                    \
GROUP                    !                     \
ATOM C8     CG2O3   0.52 !                      \
ATOM O81    OG2D2  -0.76 !  O1P    H5' H4'  O4'  \
ATOM O82    OG2D2  -0.76 !   |      |    \ /   \  \
GROUP                    !  -P-O5'-C5'---C4'    C1'
ATOM P      P       1.50 !   |      |     \     / \
ATOM O1P    ON3    -0.78 !  O2P    H5''   C3'--C2' H1'
ATOM O2P    ON3    -0.78 !               / \   / \
ATOM O5'    ON2    -0.57 !             O3' H3' O2' H2''
ATOM C5'    CN8B   -0.08 !              |       |
ATOM H5'    HN8     0.09 !                     H2'
ATOM H5''   HN8     0.09
GROUP                    !  !!!! PATCH 5UHC for the carboxylic acid form
ATOM C4'    CN7     0.16
ATOM H4'    HN7     0.09
ATOM O4'    ON6B   -0.50
ATOM C1'    CN7B    0.16
ATOM H1'    HN7     0.09
GROUP
ATOM C2'    CN7B    0.14
ATOM H2''   HN7     0.09
ATOM O2'    ON5    -0.66
ATOM H2'    HN5     0.43
```

```
GROUP
ATOM C3'    CN7      0.01
ATOM H3'    HN7      0.09
ATOM O3'    ON2     -0.57
BOND N1   C2      N1   C6      C2   O2      C2   N3
BOND N3   C4      N3   H3      C4   O4      C4   C5
BOND C5   C6      C5   C7      C6   H6      C7   H71
BOND C7   H72     C7   C8      C8   O81     C8   O82
BOND P    O1P     P    O2P     P    O5'     O5'  C5'     C5'  H5''
BOND C5'  C4'     C4'  O4'     C4'  C3'     O4'  C1'
BOND C1'  N1      C1'  C2'     C2'  C3'     C3'  O3'     O3'  +P
BOND C2'  O2'     O2'  H2'
BOND C1'  H1'     C2'  H2''    C3'  H3'     C4'  H4'     C5'  H5'
IMPR C2     N1     N3     O2     C4     C5     N3     O4     C8     O82    O81    C7
!ribose
IC -O3' P    O5'  C5'    1.6001  101.45  -39.25  119.00  1.4401
IC -O3' O5'  *P   O1P    1.6001  101.45 -115.82  109.74  1.4802
IC -O3' O5'  *P   O2P    1.6001  101.45  115.90  109.80  1.4801
IC  P   O5'  C5'  C4'    1.5996  119.00 -151.39  110.04  1.5160
IC O5'  C5'  C4'  C3'    1.4401  108.83 -179.85  116.10  1.5284
IC C5'  C4'  C3'  O3'    1.5160  116.10   76.70  115.12  1.4212
IC C4'  C3'  O3'  +P     1.5284  111.92  159.13  119.05  1.6001
IC C3'  O3'  +P   +O5'   1.4212  119.05  -98.86  101.45  1.5996
IC O4'  C3'  *C4' C5'    1.4572  104.06 -120.04  116.10  1.5160
IC C2'  C4'  *C3' O3'    1.5284  100.16 -124.08  115.12  1.4212
IC C4'  C3'  C2'  C1'    1.5284  100.16   39.58  102.04  1.5251
IC C3'  C2'  C1'  N1     1.5284  101.97  144.39  113.71  1.4896
IC O4'  C1'  N1   C2     1.5251  113.71  -96.0   117.06  1.3746
IC C1'  C3'  *C2' O2'    1.5284  102.04 -114.67  110.81  1.4212
IC H2'  O2'  C2'  C3'    0.9600  114.97  148.63  111.92  1.5284
IC O4'  C2'  *C1' H1'    0.0       0.0  -115.0     0.0   0.0
IC C1'  C3'  *C2' H2''   0.0       0.0   115.0     0.0   0.0
IC C2'  C4'  *C3' H3'    0.0       0.0   115.0     0.0   0.0
IC C3'  O4'  *C4' H4'    0.0       0.0  -115.0     0.0   0.0
IC C4'  O5'  *C5' H5'    0.0       0.0  -115.0     0.0   0.0
IC C4'  O5'  *C5' H5''   0.0       0.0   115.0     0.0   0.0
IC C2   C6   *N1  C1'       1.3966  121.57  179.97  122.49  1.4896
IC C3'  C2'  C1'  N1     1.5284  101.97  144.39  113.71  1.4896
IC O4'  C1'  N1   C2     1.5251  113.71  -96.0   117.06  1.3746
IC C1'  C2   *N1  C6     1.3966  121.57  179.97  122.49  1.4896
IC C6   N1   C2   N3        1.3875  124.25   -4.37  112.29  1.3815
IC N3   N1   *C2  O2        1.3815  112.29 -178.88  124.09  1.2401
IC N1   C2   N3   C4        1.3730  112.29    6.31  128.24  1.4132
IC C4   C2   *N3  H3        1.4132  128.24  179.74  115.62  1.0182
IC C2   N3   C4   C5        1.3815  128.24   -6.65  114.72  1.4564
IC C5   N3   *C4  O4        1.4564  114.72 -179.19  118.51  1.2389
IC C5   N1   *C6  H6        1.3605  122.41  179.81  117.50  1.0879
IC C6   C4   *C5  C7        1.3605  117.70  177.13  119.14  1.5071
IC C4   C5   C7   C8        1.4564  119.14  165.62  113.88  1.5561
IC C8   C5   *C7  H71       1.5561  113.88  121.13  108.62  1.1001
IC H71  C5   *C7  H72       1.1001  108.62  116.48  108.86  1.0944
IC C5   C7   C8   O81       1.5071  113.88 -122.33  116.11  1.2619
IC O81  C7   *C8  O82       1.2619  116.11 -179.81  115.11  1.2775
DONO H2'  O2'
DONO H3   N3
ACCE O2   C2
ACCE O4   C4
ACCE O81
ACCE O82
ACCE O1P  P
ACCE O2P  P
ACCE O2'
ACCE O3'
```

```
ACCE O4'
ACCE O5'

RESI OCU           -1.00 ! 5-methoxycarbonylmethyluridine
GROUP
ATOM N1     NG2R61 -0.34 !          H101 H102
ATOM C2     CG2R63  0.51 !            \  /
ATOM O2     OG2D4  -0.41 !             C10-H103
ATOM N3     NG2R61 -0.46 !             |
ATOM H3     HGP1    0.36 !             O9
ATOM C4     CG2R63  0.50 !             |
ATOM O4     OG2D4  -0.45 !          O8=C8   O4
ATOM C5     CG2R62 -0.05 !             |    ||
ATOM C6     CG2R62  0.17 !         H72-C7   C4    H3
ATOM H6     HGR62   0.17 !            /  \ /  \  /
GROUP                    !          H71   C5   N3
ATOM C7     CG321  -0.22 !                ||   |
ATOM H71    HGA2    0.09 !             H6-C6   C2
ATOM H72    HGA2    0.09 !                 \  / \\
ATOM C8     CG2O2   0.90 !                  N1   O2
ATOM O9     OG302  -0.49 !                   \
ATOM O8     OG2D1  -0.63 !                    \
ATOM C10    CG331  -0.01 !                     \
ATOM H101   HGA3    0.09 ! O1P    H5' H4'  O4'  \
ATOM H102   HGA3    0.09 !  |      |    \ /   \  \
ATOM H103   HGA3    0.09 ! -P-O5'-C5'---C4'    C1'
GROUP                    !  |      |     \     / \
ATOM P      P       1.50 ! O2P    H5''   C3'--C2' H1'
ATOM O1P    ON3    -0.78 !              / \   / \
ATOM O2P    ON3    -0.78 !            O3' H3' O2' H2''
ATOM O5'    ON2    -0.57 !             |       |
ATOM C5'    CN8B   -0.08 !                    H2'
ATOM H5'    HN8     0.09
ATOM H5''   HN8     0.09
GROUP
ATOM C4'    CN7     0.16
ATOM H4'    HN7     0.09
ATOM O4'    ON6B   -0.50
ATOM C1'    CN7B    0.16
ATOM H1'    HN7     0.09
GROUP
ATOM C2'    CN7B    0.14
ATOM H2''   HN7     0.09
ATOM O2'    ON5    -0.66
ATOM H2'    HN5     0.43
GROUP
ATOM C3'    CN7     0.01
ATOM H3'    HN7     0.09
ATOM O3'    ON2    -0.57
BOND N1   C2      N1   C6      C2   O2      C2   N3
BOND N3   C4      N3   H3      C4   O4      C4   C5
BOND C5   C6      C5   C7      C6   H6      C7   H71
BOND C7   H72     C7   C8      C8   O9      C8   O8
BOND O9   C10     C10  H101    C10  H102    C10  H103
BOND P    O1P     P    O2P     P    O5'     O5'  C5'     C5'  H5''
BOND C5'  C4'     C4'  O4'     C4'  C3'     O4'  C1'
BOND C1'  N1      C1'  C2'     C2'  C3'     C3'  O3'     O3'  +P
BOND C2'  O2'     O2'  H2'
BOND C1'  H1'     C2'  H2''    C3'  H3'     C4'  H4'     C5'  H5'
IMPR C2     N1     N3     O2     C4     C5     N3     O4     C8     C7     O8     O9
!ribose
IC -O3' P    O5'   C5'    1.6001  101.45  -39.25  119.00  1.4401
IC -O3' O5'  *P    O1P    1.6001  101.45 -115.82  109.74  1.4802
```

```
IC -O3' O5'  *P    O2P    1.6001  101.45  115.90  109.80  1.4801
IC  P   O5'  C5'   C4'    1.5996  119.00 -151.39  110.04  1.5160
IC O5'  C5'  C4'   C3'    1.4401  108.83 -179.85  116.10  1.5284
IC C5'  C4'  C3'   O3'    1.5160  116.10   76.70  115.12  1.4212
IC C4'  C3'  O3'   +P     1.5284  111.92  159.13  119.05  1.6001
IC C3'  O3'  +P    +O5'   1.4212  119.05  -98.86  101.45  1.5996
IC O4'  C3'  *C4'  C5'    1.4572  104.06 -120.04  116.10  1.5160
IC C2'  C4'  *C3'  O3'    1.5284  100.16 -124.08  115.12  1.4212
IC C4'  C3'  C2'   C1'    1.5284  100.16   39.58  102.04  1.5251
IC C3'  C2'  C1'   N1     1.5284  101.97  144.39  113.71  1.4896
IC O4'  C1'  N1    C2     1.5251  113.71  -96.0   117.06  1.3746
IC C1'  C3'  *C2'  O2'    1.5284  102.04 -114.67  110.81  1.4212
IC H2'  O2'  C2'   C3'    0.9600  114.97  148.63  111.92  1.5284
IC O4'  C2'  *C1'  H1'    0.0       0.0  -115.0     0.0   0.0
IC C1'  C3'  *C2'  H2''   0.0       0.0   115.0     0.0   0.0
IC C2'  C4'  *C3'  H3'    0.0       0.0   115.0     0.0   0.0
IC C3'  O4'  *C4'  H4'    0.0       0.0  -115.0     0.0   0.0
IC C4'  O5'  *C5'  H5'    0.0       0.0  -115.0     0.0   0.0
IC C4'  O5'  *C5'  H5''   0.0       0.0   115.0     0.0   0.0
IC C2   C6   *N1   C1'       1.3966  121.57  179.97  122.49  1.4896
IC C3'  C2'  C1'   N1     1.5284  101.97  144.39  113.71  1.4896
IC O4'  C1'  N1    C2     1.5251  113.71  -96.0   117.06  1.3746
IC C1'  C2   *N1   C6     1.3966  121.57  179.97  122.49  1.4896
IC C6   N1   C2    N3        1.3672  121.55    0.31  116.57  1.3600
IC N3   N1   *C2   O2        1.3600  116.57  179.61  121.53  1.2238
IC N1   C2   N3    C4        1.3757  116.57   -0.42  125.65  1.3756
IC C4   C2   *N3   H3        1.3756  125.65 -178.07  117.25  1.0006
IC C2   N3   C4    C5        1.3600  125.65    1.40  116.43  1.4505
IC C5   N3   *C4   O4        1.4505  116.43  179.32  118.23  1.2275
IC C5   N1   *C6   H6        1.3766  122.21  179.56  117.58  1.0928
IC C6   C4   *C5   C7        1.3766  117.55 -176.55  118.94  1.4844
IC C4   C5   C7    C8        1.4505  118.94   73.63  111.87  1.5238
IC C8   C5   *C7   H71       1.5238  111.87  119.52  109.75  1.1104
IC H71  C5   *C7   H72       1.1104  109.75  119.38  109.01  1.1107
IC C5   C7   C8    O9        1.4844  111.87 -159.78  109.50  1.3302
IC O9   C7   *C8   O8        1.3302  109.50 -176.59  124.89  1.2170
IC C7   C8   O9    C10       1.5238  109.50 -180.00  112.43  1.4385
IC C8   O9   C10   H101      1.3302  112.43 -178.92  109.88  1.1126
IC H101 O9   *C10  H102      1.1126  109.88  119.48  110.83  1.1137
IC H101 O9   *C10  H103      1.1126  109.88 -119.62  110.90  1.1138
DONO H2'  O2'
DONO H3   N3
ACCE O2   C2
ACCE O4   C4
ACCE O8   C8
ACCE O9
ACCE O1P  P
ACCE O2P  P
ACCE O2'
ACCE O3'
ACCE O4'
ACCE O5'

RESI MEU           -1.00 ! 5-methoxycarbonylmethyl-2'-O-methyluridine
GROUP
ATOM N1     NG2R61 -0.34 !          H101 H102
ATOM C2     CG2R63  0.51 !             \  /
ATOM O2     OG2D4  -0.41 !              C10-H103
ATOM N3     NG2R61 -0.46 !              |
ATOM H3     HGP1    0.36 !              O9
ATOM C4     CG2R63  0.50 !              |
ATOM O4     OG2D4  -0.45 !          O8=C8   O4
ATOM C5     CG2R62 -0.05 !              |    ||
```

```
ATOM C6     CG2R62  0.17 !          H72-C7   C4    H3
ATOM H6     HGR62   0.17 !             /  \ /  \  /
GROUP                    !           H71   C5    N3
ATOM C7     CG321  -0.22 !                 ||    |
ATOM H71    HGA2    0.09 !              H6-C6   C2
ATOM H72    HGA2    0.09 !                  \  / \\
ATOM C8     CG2O2   0.90 !                   N1   O2
ATOM O9     OG302  -0.49 !                    \
ATOM O8     OG2D1  -0.63 !                     \
ATOM C10    CG331  -0.01 !                      \
ATOM H101   HGA3    0.09 !  O1P    H5' H4'  O4'  \
ATOM H102   HGA3    0.09 !   |      |    \ /   \  \
ATOM H103   HGA3    0.09 !  -P-O5'-C5'---C4'    C1'
GROUP                    !   |      |     \     / \
ATOM P      P       1.50 !  O2P    H5''   C3'--C2' H1'
ATOM O1P    ON3    -0.78 !               / \   / \
ATOM O2P    ON3    -0.78 !             O3' H3' O2' H2''
ATOM O5'    ON2    -0.57 !              |      |
ATOM C5'    CN8B   -0.08 !                    CM2
ATOM H5'    HN8     0.09 !                  /  |  \
ATOM H5''   HN8     0.09 !                HM1 HM2 HM3
GROUP
ATOM C4'    CN7     0.16
ATOM H4'    HN7     0.09
ATOM O4'    ON6B   -0.50
ATOM C1'    CN7B    0.16
ATOM H1'    HN7     0.09
GROUP
ATOM C2'    CN7B    0.08
ATOM H2''   HN7     0.09
ATOM O2'    OG301  -0.34
ATOM CM2    CG331  -0.10
ATOM HM1    HGA3    0.09
ATOM HM2    HGA3    0.09
ATOM HM3    HGA3    0.09
GROUP
ATOM C3'    CN7     0.01
ATOM H3'    HN7     0.09
ATOM O3'    ON2    -0.57
BOND N1   C2      N1   C6      C2   O2      C2   N3
BOND N3   C4      N3   H3      C4   O4      C4   C5
BOND C5   C6      C5   C7      C6   H6      C7   H71
BOND C7   H72     C7   C8      C8   O9      C8   O8
BOND O9   C10     C10  H101    C10  H102    C10  H103
BOND P    O1P     P    O2P     P    O5'     O5'  C5'     C5'  H5''
BOND C5'  C4'     C4'  O4'     C4'  C3'     O4'  C1'
BOND C1'  N1      C1'  C2'     C2'  C3'     C3'  O3'     O3'  +P
BOND C2'  O2'     CM2  O2'     CM2  HM1     HM2  CM2     HM3  CM2
BOND C1'  H1'     C2'  H2''    C3'  H3'     C4'  H4'     C5'  H5'
IMPR C2     N1     N3     O2     C4     C5     N3     O4     C8     C7     O8     O9
!2OM-ribose
IC -O3' P    O5'   C5'    1.6001  101.45  -39.25  119.00  1.4401
IC -O3' O5'   *P   O1P    1.6001  101.45 -115.82  109.74  1.4802
IC -O3' O5'   *P   O2P    1.6001  101.45  115.90  109.80  1.4801
IC  P   O5'  C5'   C4'    1.5996  119.00 -151.39  110.04  1.5160
IC O5'  C5'  C4'   C3'    1.4401  108.83 -179.85  116.10  1.5284
IC C5'  C4'  C3'   O3'    1.5160  116.10   76.70  115.12  1.4212
IC C4'  C3'  O3'   +P     1.5284  111.92  159.13  119.05  1.6001
IC C3'  O3'  +P    +O5'   1.4212  119.05  -98.86  101.45  1.5996
IC O4'  C3'  *C4'  C5'    1.4572  104.06 -120.04  116.10  1.5160
IC C2'  C4'  *C3'  O3'    1.5284  100.16 -124.08  115.12  1.4212
IC C4'  C3'  C2'   C1'    1.5284  100.16   39.58  102.04  1.5251
IC C3'  C2'  C1'   N1     1.5284  101.97  144.39  113.71  1.4896
```

```
IC O4'  C1'  N1    C2      1.5251  113.71  -96.0   117.06  1.3746
IC C3'  C1'  *C2'  O2'     1.5312  102.03  117.61  107.13  1.4206
IC C1'  C2'  O2'   CM2     1.5393  107.13   90.00  107.00  1.4150
IC C2'  O2'  CM2   HM2     1.4206  107.00  180.00    0.0   0.0
IC HM2  O2'  *CM2  HM3     0.0       0.0   120.00    0.0   0.0
IC HM2  O2'  *CM2  HM1     0.0       0.0  -120.00    0.0   0.0
IC O4'  C2'  *C1'  H1'     0.0       0.0  -115.0     0.0   0.0
IC C1'  C3'  *C2'  H2''    0.0       0.0   115.0     0.0   0.0
IC C2'  C4'  *C3'  H3'     0.0       0.0   115.0     0.0   0.0
IC C3'  O4'  *C4'  H4'     0.0       0.0  -115.0     0.0   0.0
IC C4'  O5'  *C5'  H5'     0.0       0.0  -115.0     0.0   0.0
IC C4'  O5'  *C5'  H5''    0.0       0.0   115.0     0.0   0.0
IC C2   C6   *N1   C1'        1.3966  121.57  179.97  122.49  1.4896
IC C3'  C2'  C1'   N1      1.5284  101.97  144.39  113.71  1.4896
IC O4'  C1'  N1    C2      1.5251  113.71  -96.0   117.06  1.3746
IC C1'  C2   *N1   C6      1.3966  121.57  179.97  122.49  1.4896
IC C6   N1   C2    N3         1.3672  121.55    0.31  116.57  1.3600
IC N3   N1   *C2   O2         1.3600  116.57  179.61  121.53  1.2238
IC N1   C2   N3    C4         1.3757  116.57   -0.42  125.65  1.3756
IC C4   C2   *N3   H3         1.3756  125.65 -178.07  117.25  1.0006
IC C2   N3   C4    C5         1.3600  125.65    1.40  116.43  1.4505
IC C5   N3   *C4   O4         1.4505  116.43  179.32  118.23  1.2275
IC C5   N1   *C6   H6         1.3766  122.21  179.56  117.58  1.0928
IC C6   C4   *C5   C7         1.3766  117.55 -176.55  118.94  1.4844
IC C4   C5   C7    C8         1.4505  118.94   73.63  111.87  1.5238
IC C8   C5   *C7   H71        1.5238  111.87  119.52  109.75  1.1104
IC H71  C5   *C7   H72        1.1104  109.75  119.38  109.01  1.1107
IC C5   C7   C8    O9         1.4844  111.87 -159.78  109.50  1.3302
IC O9   C7   *C8   O8         1.3302  109.50 -176.59  124.89  1.2170
IC C7   C8   O9    C10        1.5238  109.50 -180.00  112.43  1.4385
IC C8   O9   C10   H101       1.3302  112.43 -178.92  109.88  1.1126
IC H101 O9   *C10  H102       1.1126  109.88  119.48  110.83  1.1137
IC H101 O9   *C10  H103       1.1126  109.88 -119.62  110.90  1.1138
DONO H3   N3
ACCE O2   C2
ACCE O4   C4
ACCE O8   C8
ACCE O9
ACCE O1P  P
ACCE O2P  P
ACCE O2'
ACCE O3'
ACCE O4'
ACCE O5'

RESI 70U           -1.00 ! 5-methoxycarbonylmethyl-2-thiouridine, SMU
GROUP
ATOM N1     NG2R61 -0.26 !           H91 H92
ATOM C2     CG2R63  0.29 !             \  /
ATOM S2     SG2D1  -0.22 !              C9 -H93
ATOM N3     NG2R61 -0.56 !              |
ATOM H3     HGP1    0.40 !              O9
ATOM C4     CG2R63  0.39 !              |
ATOM O4     OG2D4  -0.41 !           O8=C8   O4
ATOM C5     CG2R62 -0.03 !              |    ||
ATOM C6     CG2R62  0.18 !          H52-C5M  C4    H3
ATOM H6     HGR62   0.22 !             /  \ /  \  /
GROUP                    !           H51   C5   N3
ATOM C5M    CG321  -0.22 !                 ||   |
ATOM H51    HGA2    0.09 !              H6-C6   C2
ATOM H52    HGA2    0.09 !                  \  / \\
ATOM C8     CG2O2   0.90 !                   N1   S2
ATOM O9     OG302  -0.49 !                    \
```

```
ATOM O8      OG2D1  -0.63 !                    \
ATOM C9      CG331  -0.01 !                     \
ATOM H91     HGA3    0.09 !  O1P    H5' H4'  O4'  \
ATOM H92     HGA3    0.09 !   |      |    \ /   \  \
ATOM H93     HGA3    0.09 !  -P-O5'-C5'---C4'    C1'
GROUP                     !   |      |     \     / \
ATOM P       P       1.50 !  O2P    H5''   C3'--C2' H1'
ATOM O1P     ON3    -0.78 !               / \   / \
ATOM O2P     ON3    -0.78 !            O3' H3' O2' H2''
ATOM O5'     ON2    -0.57 !             |       |
ATOM C5'     CN8B   -0.08 !                    H2'
ATOM H5'     HN8     0.09
ATOM H5''    HN8     0.09
GROUP
ATOM C4'     CN7     0.16
ATOM H4'     HN7     0.09
ATOM O4'     ON6B   -0.50
ATOM C1'     CN7B    0.16
ATOM H1'     HN7     0.09
GROUP
ATOM C2'     CN7B    0.14
ATOM H2''    HN7     0.09
ATOM O2'     ON5    -0.66
ATOM H2'     HN5     0.43
GROUP
ATOM C3'     CN7     0.01
ATOM H3'     HN7     0.09
ATOM O3'     ON2    -0.57
BOND N1   C2      N1   C6      C2   S2      C2   N3
BOND N3   C4      N3   H3      C4   O4      C4   C5
BOND C5   C6      C5   C5M     C6   H6      C5M  C8
BOND C5M  H51     C5M  H52     C8   O8      C8   O9
BOND O9   C9      C9   H91     C9   H92     C9   H93
BOND P    O1P     P    O2P     P    O5'     O5'  C5'     C5'  H5''
BOND C5'  C4'     C4'  O4'     C4'  C3'     O4'  C1'
BOND C1'  N1      C1'  C2'     C2'  C3'     C3'  O3'     O3'  +P
BOND C2'  O2'     O2'  H2'
BOND C1'  H1'     C2'  H2''    C3'  H3'     C4'  H4'     C5'  H5'
IMPR C2     N1     N3     S2     C4     C5     N3     O4     C8     C5M    O8     O9
!ribose
IC -O3' P    O5'   C5'    1.6001  101.45  -39.25  119.00  1.4401
IC -O3' O5'  *P    O1P    1.6001  101.45 -115.82  109.74  1.4802
IC -O3' O5'  *P    O2P    1.6001  101.45  115.90  109.80  1.4801
IC  P   O5'  C5'   C4'    1.5996  119.00 -151.39  110.04  1.5160
IC O5'  C5'  C4'   C3'    1.4401  108.83 -179.85  116.10  1.5284
IC C5'  C4'  C3'   O3'    1.5160  116.10   76.70  115.12  1.4212
IC C4'  C3'  O3'   +P     1.5284  111.92  159.13  119.05  1.6001
IC C3'  O3'  +P    +O5'   1.4212  119.05  -98.86  101.45  1.5996
IC O4'  C3'  *C4'  C5'    1.4572  104.06 -120.04  116.10  1.5160
IC C2'  C4'  *C3'  O3'    1.5284  100.16 -124.08  115.12  1.4212
IC C4'  C3'  C2'   C1'    1.5284  100.16   39.58  102.04  1.5251
IC C3'  C2'  C1'   N1     1.5284  101.97  144.39  113.71  1.4896
IC O4'  C1'  N1    C2     1.5251  113.71  -96.0   117.06  1.3746
IC C1'  C3'  *C2'  O2'    1.5284  102.04 -114.67  110.81  1.4212
IC H2'  O2'  C2'   C3'    0.9600  114.97  148.63  111.92  1.5284
IC O4'  C2'  *C1'  H1'    0.0       0.0  -115.0     0.0   0.0
IC C1'  C3'  *C2'  H2''   0.0       0.0   115.0     0.0   0.0
IC C2'  C4'  *C3'  H3'    0.0       0.0   115.0     0.0   0.0
IC C3'  O4'  *C4'  H4'    0.0       0.0  -115.0     0.0   0.0
IC C4'  O5'  *C5'  H5'    0.0       0.0  -115.0     0.0   0.0
IC C4'  O5'  *C5'  H5''   0.0       0.0   115.0     0.0   0.0
IC C2   C6   *N1   C1'       1.3966  121.57  179.97  122.49  1.4896
IC C3'  C2'  C1'   N1     1.5284  101.97  144.39  113.71  1.4896
```

```
IC O4'  C1'  N1    C2      1.5251  113.71  -96.0   117.06  1.3746
IC C1'  C2   *N1   C6      1.3966  121.57  179.97  122.49  1.4896
IC C6   N1   C2    N3        1.3753  121.60   -0.50  113.54  1.3874
IC N3   N1   *C2   S2        1.3874  113.54 -179.74  125.77  1.6511
IC N1   C2   N3    C4        1.4148  113.54   -0.17  127.72  1.3820
IC C4   C2   *N3   H3        1.3820  127.72 -178.93  117.96  1.0052
IC C2   N3   C4    C5        1.3874  127.72    1.52  116.48  1.4480
IC C5   N3   *C4   O4        1.4480  116.48 -179.88  117.97  1.2296
IC C5   N1   *C6   H6        1.3824  123.67  179.09  114.56  1.0881
IC C6   C4   *C5   C5M       1.3824  116.96 -176.75  115.69  1.5149
IC C4   C5   C5M   C8        1.4480  115.69 -170.48  117.09  1.5317
IC C8   C5   *C5M  H51       1.5317  117.09  120.36  106.03  1.1104
IC C8   C5   *C5M  H52       1.5317  117.09 -122.55  105.98  1.1083
IC C5   C5M  C8    O9        1.5149  117.09 -178.54  113.49  1.3450
IC O9   C5M  *C8   O8        1.3450  113.49  179.85  123.35  1.2173
IC C5M  C8   O9    C9        1.5317  113.49   -1.60  122.48  1.4328
IC C8   O9   C9    H91       1.3450  122.48   63.21  111.59  1.1130
IC H91  O9   *C9   H92       1.1130  111.59  118.16  109.01  1.1133
IC H91  O9   *C9   H93       1.1130  111.59 -123.59  111.60  1.1124
DONO H2'  O2'
DONO H3   N3
ACCE O4   C4
ACCE S2   C2
ACCE O8   C8
ACCE O9
ACCE O1P  P
ACCE O2P  P
ACCE O2'
ACCE O3'
ACCE O4'
ACCE O5'

RESI BCU           -1.00 ! 5-carbamoylmethyluridine
GROUP
ATOM N1     NG2R61 -0.34 !          H82    H81
ATOM C2     CG2R63  0.51 !             \  /
ATOM O2     OG2D4  -0.41 !              N8
ATOM N3     NG2R61 -0.46 !              |
ATOM H3     HGP1    0.36 !           O8=C8   O4
ATOM C4     CG2R63  0.50 !              |    ||
ATOM O4     OG2D4  -0.45 !          H72-C7   C4    H3
ATOM C5     CG2R62 -0.05 !             /  \ /  \  /
ATOM C6     CG2R62  0.17 !           H71   C5   N3
ATOM H6     HGR62   0.17 !                 ||   |
GROUP                    !              H6-C6   C2
ATOM C7     CG321  -0.18 !                  \  / \\
ATOM H71    HGA2    0.09 !                   N1   O2
ATOM H72    HGA2    0.09 !                    \
ATOM C8     CG2O1   0.55 !                     \
ATOM O8     OG2D1  -0.55 !                      \
ATOM N8     NG2S2  -0.62 ! O1P    H5' H4'  O4'  \
ATOM H81    HGP1    0.30 !  |      |    \ /   \  \
ATOM H82    HGP1    0.32 ! -P-O5'-C5'---C4'    C1'
GROUP                    !  |      |     \     / \
ATOM P      P       1.50 ! O2P    H5''   C3'--C2' H1'
ATOM O1P    ON3    -0.78 !              / \   / \
ATOM O2P    ON3    -0.78 !            O3' H3' O2' H2''
ATOM O5'    ON2    -0.57 !             |       |
ATOM C5'    CN8B   -0.08 !                    H2'
ATOM H5'    HN8     0.09
ATOM H5''   HN8     0.09
GROUP
ATOM C4'    CN7     0.16
```

```
ATOM H4'    HN7      0.09
ATOM O4'    ON6B   -0.50
ATOM C1'    CN7B    0.16
ATOM H1'    HN7      0.09
GROUP
ATOM C2'    CN7B    0.14
ATOM H2''   HN7      0.09
ATOM O2'    ON5    -0.66
ATOM H2'    HN5      0.43
GROUP
ATOM C3'    CN7      0.01
ATOM H3'    HN7      0.09
ATOM O3'    ON2    -0.57
BOND N1   C2      N1   C6      C2   O2      C2   N3
BOND N3   C4      N3   H3      C4   O4      C4   C5
BOND C5   C6      C5   C7      C6   H6      C7   H71
BOND C7   H72     C7   C8      C8   N8      C8   O8
BOND N8   H81     N8   H82
BOND P    O1P     P    O2P     P    O5'     O5'  C5'     C5'  H5''
BOND C5'  C4'     C4'  O4'     C4'  C3'     O4'  C1'
BOND C1'  N1      C1'  C2'     C2'  C3'     C3'  O3'     O3'  +P
BOND C2'  O2'     O2'  H2'
BOND C1'  H1'     C2'  H2''    C3'  H3'     C4'  H4'     C5'  H5'
IMPR C2     N1     N3     O2     C4     C5     N3     O4     C8     C7     N8     O8
!ribose
IC -O3' P    O5'  C5'    1.6001  101.45  -39.25  119.00  1.4401
IC -O3' O5'  *P   O1P    1.6001  101.45 -115.82  109.74  1.4802
IC -O3' O5'  *P   O2P    1.6001  101.45  115.90  109.80  1.4801
IC  P   O5'  C5'  C4'    1.5996  119.00 -151.39  110.04  1.5160
IC O5'  C5'  C4'  C3'    1.4401  108.83 -179.85  116.10  1.5284
IC C5'  C4'  C3'  O3'    1.5160  116.10   76.70  115.12  1.4212
IC C4'  C3'  O3'  +P     1.5284  111.92  159.13  119.05  1.6001
IC C3'  O3'  +P   +O5'   1.4212  119.05  -98.86  101.45  1.5996
IC O4'  C3'  *C4' C5'    1.4572  104.06 -120.04  116.10  1.5160
IC C2'  C4'  *C3' O3'    1.5284  100.16 -124.08  115.12  1.4212
IC C4'  C3'  C2'  C1'    1.5284  100.16   39.58  102.04  1.5251
IC C3'  C2'  C1'  N1     1.5284  101.97  144.39  113.71  1.4896
IC O4'  C1'  N1   C2     1.5251  113.71  -96.0   117.06  1.3746
IC C1'  C3'  *C2' O2'    1.5284  102.04 -114.67  110.81  1.4212
IC H2'  O2'  C2'  C3'    0.9600  114.97  148.63  111.92  1.5284
IC O4'  C2'  *C1' H1'    0.0       0.0  -115.0     0.0   0.0
IC C1'  C3'  *C2' H2''   0.0       0.0   115.0     0.0   0.0
IC C2'  C4'  *C3' H3'    0.0       0.0   115.0     0.0   0.0
IC C3'  O4'  *C4' H4'    0.0       0.0  -115.0     0.0   0.0
IC C4'  O5'  *C5' H5'    0.0       0.0  -115.0     0.0   0.0
IC C4'  O5'  *C5' H5''   0.0       0.0   115.0     0.0   0.0
IC C2   C6   *N1  C1'       1.3966  121.57  179.97  122.49  1.4896
IC C3'  C2'  C1'  N1     1.5284  101.97  144.39  113.71  1.4896
IC O4'  C1'  N1   C2     1.5251  113.71  -96.0   117.06  1.3746
IC C1'  C2   *N1  C6     1.3966  121.57  179.97  122.49  1.4896
IC C6   N1   C2   N3        1.3704  120.17   -0.36  116.35  1.3675
IC N3   N1   *C2  O2        1.3675  116.35 -179.88  123.15  1.2285
IC N1   C2   N3   C4        1.4088  116.35   -0.44  126.05  1.3742
IC C4   C2   *N3  H3        1.3742  126.05 -178.19  116.56  1.0003
IC C2   N3   C4   C5        1.3675  126.05    1.94  116.41  1.4478
IC C5   N3   *C4  O4        1.4478  116.41 -179.67  118.25  1.2310
IC C5   N1   *C6  H6        1.3769  123.03  178.78  116.53  1.0914
IC C6   C4   *C5  C7        1.3769  117.94 -177.21  119.77  1.5054
IC C4   C5   C7   C8        1.4478  119.77   81.52  110.45  1.4926
IC C8   C5   *C7  H71       1.4926  110.45 -123.67  109.60  1.1116
IC H71  C5   *C7  H72       1.1116  109.60 -118.32  108.77  1.1133
IC C5   C7   C8   N8        1.5054  110.45  -87.71  117.95  1.3541
IC N8   C7   *C8  O8        1.3541  117.95  177.43  119.91  1.2267
```

```
IC C7   C8   N8    H81       1.4926  117.95  175.64  116.77  0.9957
IC H81  C8   *N8   H82       0.9957  116.77 -164.96  118.86  0.9971
DONO H2'  O2'
DONO H3   N3
DONO H81  N8
DONO H82  N8
ACCE O2   C2
ACCE O4   C4
ACCE O8   C8
ACCE O1P  P
ACCE O2P  P
ACCE O2'
ACCE O3'
ACCE O4'
ACCE O5'

RESI MCU           -1.00 ! 5-carbamoylmethyl-2'-O-methyluridine
GROUP
ATOM N1     NG2R61 -0.34 !         H82    H81
ATOM C2     CG2R63  0.51 !            \  /
ATOM O2     OG2D4  -0.41 !             N8
ATOM N3     NG2R61 -0.46 !             |
ATOM H3     HGP1    0.36 !          O8=C8   O4
ATOM C4     CG2R63  0.50 !             |    ||
ATOM O4     OG2D4  -0.45 !         H72-C7   C4    H3
ATOM C5     CG2R62 -0.05 !            /  \ /  \  /
ATOM C6     CG2R62  0.17 !          H71   C5   N3
ATOM H6     HGR62   0.17 !                ||   |
GROUP                    !             H6-C6   C2
ATOM C7     CG321  -0.18 !                 \  / \\
ATOM H71    HGA2    0.09 !                  N1   O2
ATOM H72    HGA2    0.09 !                   \
ATOM C8     CG2O1   0.55 !                    \
ATOM O8     OG2D1  -0.55 !                     \
ATOM N8     NG2S2  -0.62 ! O1P    H5' H4'  O4'  \
ATOM H81    HGP1    0.30 !  |       |    \ /   \  \
ATOM H82    HGP1    0.32 ! -P-O5'-C5'---C4'    C1'
GROUP                    !  |       |     \     / \
ATOM P      P       1.50 ! O2P    H5''   C3'--C2' H1'
ATOM O1P    ON3    -0.78 !              / \   / \
ATOM O2P    ON3    -0.78 !            O3' H3' O2' H2''
ATOM O5'    ON2    -0.57 !             |       |
ATOM C5'    CN8B   -0.08 !                    CM2
ATOM H5'    HN8     0.09 !                   /  |  \
ATOM H5''   HN8     0.09 !                 HM1 HM2 HM3
GROUP
ATOM C4'    CN7     0.16
ATOM H4'    HN7     0.09
ATOM O4'    ON6B   -0.50
ATOM C1'    CN7B    0.16
ATOM H1'    HN7     0.09
GROUP
ATOM C2'    CN7B    0.08
ATOM H2''   HN7     0.09
ATOM O2'    OG301  -0.34
ATOM CM2    CG331  -0.10
ATOM HM1    HGA3    0.09
ATOM HM2    HGA3    0.09
ATOM HM3    HGA3    0.09
GROUP
ATOM C3'    CN7     0.01
ATOM H3'    HN7     0.09
ATOM O3'    ON2    -0.57
```

```
BOND N1   C2       N1   C6       C2   O2       C2   N3
BOND N3   C4       N3   H3       C4   O4       C4   C5
BOND C5   C6       C5   C7       C6   H6       C7   H71
BOND C7   H72      C7   C8       C8   N8       C8   O8
BOND N8   H81      N8   H82
BOND P    O1P      P    O2P      P    O5'      O5'  C5'      C5'  H5''
BOND C5'  C4'      C4'  O4'      C4'  C3'      O4'  C1'
BOND C1'  N1       C1'  C2'      C2'  C3'      C3'  O3'      O3'  +P
BOND C2'  O2'      CM2  O2'      CM2  HM1      HM2  CM2      HM3  CM2
BOND C1'  H1'      C2'  H2''     C3'  H3'      C4'  H4'      C5'  H5'
IMPR C2     N1     N3     O2     C4     C5     N3     O4     C8     C7     N8     O8
!2OM-ribose
IC -O3' P    O5'  C5'    1.6001  101.45  -39.25  119.00  1.4401
IC -O3' O5'  *P   O1P    1.6001  101.45 -115.82  109.74  1.4802
IC -O3' O5'  *P   O2P    1.6001  101.45  115.90  109.80  1.4801
IC  P   O5'  C5'  C4'    1.5996  119.00 -151.39  110.04  1.5160
IC O5'  C5'  C4'  C3'    1.4401  108.83 -179.85  116.10  1.5284
IC C5'  C4'  C3'  O3'    1.5160  116.10   76.70  115.12  1.4212
IC C4'  C3'  O3'  +P     1.5284  111.92  159.13  119.05  1.6001
IC C3'  O3'  +P   +O5'   1.4212  119.05  -98.86  101.45  1.5996
IC O4'  C3'  *C4' C5'    1.4572  104.06 -120.04  116.10  1.5160
IC C2'  C4'  *C3' O3'    1.5284  100.16 -124.08  115.12  1.4212
IC C4'  C3'  C2'  C1'    1.5284  100.16   39.58  102.04  1.5251
IC C3'  C2'  C1'  N1     1.5284  101.97  144.39  113.71  1.4896
IC O4'  C1'  N1   C2     1.5251  113.71  -96.0   117.06  1.3746
IC C3'  C1'  *C2' O2'    1.5312  102.03  117.61  107.13  1.4206
IC C1'  C2'  O2'  CM2    1.5393  107.13   90.00  107.00  1.4150
IC C2'  O2'  CM2  HM2    1.4206  107.00  180.00    0.0   0.0
IC HM2  O2'  *CM2 HM3    0.0       0.0   120.00    0.0   0.0
IC HM2  O2'  *CM2 HM1    0.0       0.0  -120.00    0.0   0.0
IC O4'  C2'  *C1' H1'    0.0       0.0  -115.0     0.0   0.0
IC C1'  C3'  *C2' H2''   0.0       0.0   115.0     0.0   0.0
IC C2'  C4'  *C3' H3'    0.0       0.0   115.0     0.0   0.0
IC C3'  O4'  *C4' H4'    0.0       0.0  -115.0     0.0   0.0
IC C4'  O5'  *C5' H5'    0.0       0.0  -115.0     0.0   0.0
IC C4'  O5'  *C5' H5''   0.0       0.0   115.0     0.0   0.0
IC C2   C6   *N1  C1'       1.3966  121.57  179.97  122.49  1.4896
IC C3'  C2'  C1'  N1     1.5284  101.97  144.39  113.71  1.4896
IC O4'  C1'  N1   C2     1.5251  113.71  -96.0   117.06  1.3746
IC C1'  C2   *N1  C6     1.3966  121.57  179.97  122.49  1.4896
IC C6   N1   C2   N3        1.3704  120.17   -0.36  116.35  1.3675
IC N3   N1   *C2  O2        1.3675  116.35 -179.88  123.15  1.2285
IC N1   C2   N3   C4        1.4088  116.35   -0.44  126.05  1.3742
IC C4   C2   *N3  H3        1.3742  126.05 -178.19  116.56  1.0003
IC C2   N3   C4   C5        1.3675  126.05    1.94  116.41  1.4478
IC C5   N3   *C4  O4        1.4478  116.41 -179.67  118.25  1.2310
IC C5   N1   *C6  H6        1.3769  123.03  178.78  116.53  1.0914
IC C6   C4   *C5  C7        1.3769  117.94 -177.21  119.77  1.5054
IC C4   C5   C7   C8        1.4478  119.77   81.52  110.45  1.4926
IC C8   C5   *C7  H71       1.4926  110.45 -123.67  109.60  1.1116
IC H71  C5   *C7  H72       1.1116  109.60 -118.32  108.77  1.1133
IC C5   C7   C8   N8        1.5054  110.45  -87.71  117.95  1.3541
IC N8   C7   *C8  O8        1.3541  117.95  177.43  119.91  1.2267
IC C7   C8   N8   H81       1.4926  117.95  175.64  116.77  0.9957
IC H81  C8   *N8  H82       0.9957  116.77 -164.96  118.86  0.9971
DONO H3   N3
DONO H81  N8
DONO H82  N8
ACCE O2   C2
ACCE O4   C4
ACCE O8   C8
ACCE O1P  P
ACCE O2P  P
```

```
ACCE O2'
ACCE O3'
ACCE O4'
ACCE O5'

RESI HCU           -2.00 ! 5-(carboxyhydroxymethyl)uridine
GROUP
ATOM N1     NG2R61 -0.34 !        O81  O82(-)
ATOM C2     CG2R63  0.51 !           \\ /
ATOM O2     OG2D4  -0.41 !            C8      O4
ATOM N3     NG2R61 -0.46 !              \     ||
ATOM H3     HGP1    0.36 !      H7O-O7-C7   C4    H3
ATOM C4     CG2R63  0.50 !             /  \ /  \  /
ATOM O4     OG2D4  -0.45 !           H7    C5   N3
ATOM C5     CG2R62 -0.05 !                 ||   |
ATOM C6     CG2R62  0.17 !              H6-C6   C2
ATOM H6     HGR62   0.17 !                  \  / \\
GROUP                    !                   N1   O2
ATOM C7     CG311   0.14 !                    \
ATOM H7     HGA1    0.09 !                     \
ATOM O7     OG311  -0.65 !                      \
ATOM H7O    HGP1    0.42 ! O1P    H5' H4'  O4'  \
GROUP                    !  |       |    \ /   \  \
ATOM C8     CG2O3   0.52 ! -P-O5'-C5'---C4'    C1'
ATOM O81    OG2D2  -0.76 !  |       |     \     / \
ATOM O82    OG2D2  -0.76 ! O2P    H5''   C3'--C2' H1'
GROUP                    !               / \   / \
ATOM P      P       1.50 !             O3' H3' O2' H2''
ATOM O1P    ON3    -0.78 !              |       |
ATOM O2P    ON3    -0.78 !                     H2'
ATOM O5'    ON2    -0.57
ATOM C5'    CN8B   -0.08 !  !!!! PATCH 5UHC for the carboxylic acid form
ATOM H5'    HN8     0.09
ATOM H5''   HN8     0.09
GROUP
ATOM C4'    CN7     0.16
ATOM H4'    HN7     0.09
ATOM O4'    ON6B   -0.50
ATOM C1'    CN7B    0.16
ATOM H1'    HN7     0.09
GROUP
ATOM C2'    CN7B    0.14
ATOM H2''   HN7     0.09
ATOM O2'    ON5    -0.66
ATOM H2'    HN5     0.43
GROUP
ATOM C3'    CN7     0.01
ATOM H3'    HN7     0.09
ATOM O3'    ON2    -0.57
BOND N1   C2      N1   C6      C2   O2      C2   N3
BOND N3   C4      N3   H3      C4   O4      C4   C5
BOND C5   C6      C5   C7      C6   H6      C7   H7
BOND C7   O7      C7   C8      O7   H7O     C8   O81
BOND C8   O82
BOND P    O1P     P    O2P     P    O5'     O5'  C5'     C5'  H5''
BOND C5'  C4'     C4'  O4'     C4'  C3'     O4'  C1'
BOND C1'  N1      C1'  C2'     C2'  C3'     C3'  O3'     O3'  +P
BOND C2'  O2'     O2'  H2'
BOND C1'  H1'     C2'  H2''    C3'  H3'     C4'  H4'     C5'  H5'
IMPR C2     N1     N3     O2     C4     C5     N3     O4     C8     O82    O81    C7
!ribose
IC -O3' P    O5'   C5'    1.6001  101.45  -39.25  119.00  1.4401
IC -O3' O5'  *P    O1P    1.6001  101.45 -115.82  109.74  1.4802
```

```
IC -O3' O5'  *P    O2P    1.6001  101.45  115.90  109.80  1.4801
IC  P   O5'  C5'   C4'    1.5996  119.00 -151.39  110.04  1.5160
IC O5'  C5'  C4'   C3'    1.4401  108.83 -179.85  116.10  1.5284
IC C5'  C4'  C3'   O3'    1.5160  116.10   76.70  115.12  1.4212
IC C4'  C3'  O3'   +P     1.5284  111.92  159.13  119.05  1.6001
IC C3'  O3'  +P    +O5'   1.4212  119.05  -98.86  101.45  1.5996
IC O4'  C3'  *C4'  C5'    1.4572  104.06 -120.04  116.10  1.5160
IC C2'  C4'  *C3'  O3'    1.5284  100.16 -124.08  115.12  1.4212
IC C4'  C3'  C2'   C1'    1.5284  100.16   39.58  102.04  1.5251
IC C3'  C2'  C1'   N1     1.5284  101.97  144.39  113.71  1.4896
IC O4'  C1'  N1    C2     1.5251  113.71  -96.0   117.06  1.3746
IC C1'  C3'  *C2'  O2'    1.5284  102.04 -114.67  110.81  1.4212
IC H2'  O2'  C2'   C3'    0.9600  114.97  148.63  111.92  1.5284
IC O4'  C2'  *C1'  H1'    0.0       0.0  -115.0     0.0   0.0
IC C1'  C3'  *C2'  H2''   0.0       0.0   115.0     0.0   0.0
IC C2'  C4'  *C3'  H3'    0.0       0.0   115.0     0.0   0.0
IC C3'  O4'  *C4'  H4'    0.0       0.0  -115.0     0.0   0.0
IC C4'  O5'  *C5'  H5'    0.0       0.0  -115.0     0.0   0.0
IC C4'  O5'  *C5'  H5''   0.0       0.0   115.0     0.0   0.0
IC C2   C6   *N1   C1'       1.3966  121.57  179.97  122.49  1.4896
IC C3'  C2'  C1'   N1     1.5284  101.97  144.39  113.71  1.4896
IC O4'  C1'  N1    C2     1.5251  113.71  -96.0   117.06  1.3746
IC C1'  C2   *N1   C6     1.3966  121.57  179.97  122.49  1.4896
IC C6   N1   C2    N3        1.3902  121.05   -8.63  113.53  1.3802
IC N3   N1   *C2   O2        1.3802  113.53 -177.70  123.40  1.2414
IC N1   C2   N3    C4        1.3800  113.53   13.22  128.58  1.4188
IC C4   C2   *N3   H3        1.4188  128.58  179.86  114.77  1.0178
IC C2   N3   C4    C5        1.3802  128.58  -11.82  113.18  1.4604
IC C5   N3   *C4   O4        1.4604  113.18 -178.63  119.02  1.2307
IC C5   N1   *C6   H6        1.3576  124.70  179.54  114.62  1.0866
IC C6   C4   *C5   C7        1.3576  117.74  173.28  120.96  1.4967
IC C4   C5   C7    C8        1.4604  120.96  -63.68  111.02  1.5726
IC C8   C5   *C7   O7        1.5726  111.02  123.97  111.35  1.4224
IC O7   C5   *C7   H7        1.4224  111.35  119.81  107.77  1.1031
IC C5   C7   O7    H7O       1.4967  111.35 -123.47  100.28  0.9936
IC C5   C7   C8    O81       1.4967  111.02  130.43  113.55  1.2684
IC O81  C7   *C8   O82       1.2684  113.55  178.25  115.25  1.2603
DONO H2'  O2'
DONO H3   N3
DONO H7O  O7
ACCE O2   C2
ACCE O4   C4
ACCE O7
ACCE O81  C8
ACCE O82  C8
ACCE O1P  P
ACCE O2P  P
ACCE O2'
ACCE O3'
ACCE O4'
ACCE O5'

RESI CMU           -1.00 ! 5-(carboxyhydroxymethyl)uridine methyl ester
GROUP
ATOM N1     NG2R61 -0.34 !              H102
ATOM C2     CG2R63  0.51 !               |
ATOM O2     OG2D4  -0.41 !         H103-C10-H101
ATOM N3     NG2R61 -0.46 !               |
ATOM H3     HGP1    0.36 !         O8   O9
ATOM C4     CG2R63  0.50 !          \\ /
ATOM O4     OG2D4  -0.45 !           C8      O4
ATOM C5     CG2R62 -0.05 !             \     ||
ATOM C6     CG2R62  0.17 !       H7O-O7-C7   C4    H3
```

```
ATOM H6     HGR62   0.17 !             /  \ /  \  /
GROUP                    !           H7    C5   N3
ATOM C7     CG311   0.14 !                 ||   |
ATOM H7     HGA1    0.09 !              H6-C6   C2
ATOM O7     OG311  -0.65 !                  \  / \\
ATOM H7O    HGP1    0.42 !                   N1   O2
GROUP                    !                    \
ATOM C8     CG2O2   0.86 !                     \
ATOM O8     OG2D1  -0.63 !                      \
ATOM O9     OG302  -0.49 !  O1P    H5' H4'  O4'  \
ATOM C10    CG331  -0.01 !   |      |    \ /   \  \
ATOM H101   HGA3    0.09 !  -P-O5'-C5'---C4'    C1'
ATOM H102   HGA3    0.09 !   |      |     \     / \
ATOM H103   HGA3    0.09 !  O2P    H5''   C3'--C2' H1'
GROUP                    !               / \   / \
ATOM P      P       1.50 !             O3' H3' O2' H2''
ATOM O1P    ON3    -0.78 !              |       |
ATOM O2P    ON3    -0.78 !                      H2'
ATOM O5'    ON2    -0.57
ATOM C5'    CN8B   -0.08
ATOM H5'    HN8     0.09
ATOM H5''   HN8     0.09
GROUP
ATOM C4'    CN7     0.16
ATOM H4'    HN7     0.09
ATOM O4'    ON6B   -0.50
ATOM C1'    CN7B    0.16
ATOM H1'    HN7     0.09
GROUP
ATOM C2'    CN7B    0.14
ATOM H2''   HN7     0.09
ATOM O2'    ON5    -0.66
ATOM H2'    HN5     0.43
GROUP
ATOM C3'    CN7     0.01
ATOM H3'    HN7     0.09
ATOM O3'    ON2    -0.57
BOND N1   C2      N1   C6      C2   O2      C2   N3
BOND N3   C4      N3   H3      C4   O4      C4   C5
BOND C5   C6      C5   C7      C6   H6      C7   H7
BOND C7   C8      C7   O7      C8   O8      C8   O9
BOND O7   H7O     O9   C10     C10  H101    C10  H102
BOND C10  H103
BOND P    O1P     P    O2P     P    O5'     O5'  C5'     C5'  H5''
BOND C5'  C4'     C4'  O4'     C4'  C3'     O4'  C1'
BOND C1'  N1      C1'  C2'     C2'  C3'     C3'  O3'     O3'  +P
BOND C2'  O2'     O2'  H2'
BOND C1'  H1'     C2'  H2''    C3'  H3'     C4'  H4'     C5'  H5'
IMPR C2     N1     N3     O2     C4     C5     N3     O4     C8     C7     O8     O9
!ribose
IC -O3' P    O5'   C5'    1.6001  101.45  -39.25  119.00  1.4401
IC -O3' O5'  *P    O1P    1.6001  101.45 -115.82  109.74  1.4802
IC -O3' O5'  *P    O2P    1.6001  101.45  115.90  109.80  1.4801
IC  P   O5'  C5'   C4'    1.5996  119.00 -151.39  110.04  1.5160
IC O5'  C5'  C4'   C3'    1.4401  108.83 -179.85  116.10  1.5284
IC C5'  C4'  C3'   O3'    1.5160  116.10   76.70  115.12  1.4212
IC C4'  C3'  O3'   +P     1.5284  111.92  159.13  119.05  1.6001
IC C3'  O3'  +P    +O5'   1.4212  119.05  -98.86  101.45  1.5996
IC O4'  C3'  *C4'  C5'    1.4572  104.06 -120.04  116.10  1.5160
IC C2'  C4'  *C3'  O3'    1.5284  100.16 -124.08  115.12  1.4212
IC C4'  C3'  C2'   C1'    1.5284  100.16   39.58  102.04  1.5251
IC C3'  C2'  C1'   N1     1.5284  101.97  144.39  113.71  1.4896
IC O4'  C1'  N1    C2     1.5251  113.71  -96.0   117.06  1.3746
```

```
IC C1'  C3'  *C2'  O2'    1.5284  102.04 -114.67  110.81  1.4212
IC H2'  O2'  C2'   C3'    0.9600  114.97  148.63  111.92  1.5284
IC O4'  C2'  *C1'  H1'    0.0       0.0  -115.0     0.0   0.0
IC C1'  C3'  *C2'  H2''   0.0       0.0   115.0     0.0   0.0
IC C2'  C4'  *C3'  H3'    0.0       0.0   115.0     0.0   0.0
IC C3'  O4'  *C4'  H4'    0.0       0.0  -115.0     0.0   0.0
IC C4'  O5'  *C5'  H5'    0.0       0.0  -115.0     0.0   0.0
IC C4'  O5'  *C5'  H5''   0.0       0.0   115.0     0.0   0.0
IC C2   C6   *N1   C1'       1.3966  121.57  179.97  122.49  1.4896
IC C3'  C2'  C1'   N1     1.5284  101.97  144.39  113.71  1.4896
IC O4'  C1'  N1    C2     1.5251  113.71  -96.0   117.06  1.3746
IC C1'  C2   *N1   C6     1.3966  121.57  179.97  122.49  1.4896
IC C6   N1   C2    N3        1.3762  121.50    5.27  113.95  1.3862
IC N3   N1   *C2   O2        1.3862  113.95  178.81  122.91  1.2317
IC N1   C2   N3    C4        1.3910  113.95   -8.54  128.20  1.4015
IC C4   C2   *N3   H3        1.4015  128.20 -179.39  114.87  1.0197
IC C2   N3   C4    C5        1.3862  128.20    9.31  113.13  1.4548
IC C5   N3   *C4   O4        1.4548  113.13  179.64  120.89  1.2350
IC C5   N1   *C6   H6        1.3583  123.18 -179.46  116.12  1.0856
IC C6   C4   *C5   C7        1.3583  119.33 -175.56  119.04  1.5018
IC C4   C5   C7    C8        1.4548  119.04  -87.27  112.02  1.5282
IC C8   C5   *C7   O7        1.5282  112.02 -120.85  109.77  1.4214
IC C8   C5   *C7   H7        1.5282  112.02  117.89  108.41  1.1012
IC C5   C7   O7    H7O       1.5018  109.77  143.91  106.25  0.9805
IC C5   C7   C8    O9        1.5018  112.02   44.07  112.46  1.3360
IC O9   C7   *C8   O8        1.3360  112.46  178.65  122.29  1.2280
IC C7   C8   O9    C10       1.5282  112.46  176.53  115.09  1.4506
IC C8   O9   C10   H101      1.3360  115.09 -177.66  104.62  1.0877
IC H101 O9   *C10  H102      1.0877  104.62  119.43  109.85  1.0899
IC H101 O9   *C10  H103      1.0877  104.62 -119.47  109.89  1.0915
DONO H2'  O2'
DONO H3   N3
DONO H7O  O7
ACCE O2   C2
ACCE O4   C4
ACCE O7
ACCE O8   C8
ACCE O9
ACCE O1P  P
ACCE O2P  P
ACCE O2'
ACCE O3'
ACCE O4'
ACCE O5'

RESI OAU           -2.00 ! uridine 5-oxyacetic acid   ! adjusted    5-
(CARBOXYMETHOXY) URIDINE
GROUP
ATOM N1     NG2R61 -0.30 !           O91   O92(-)
ATOM C2     CG2R63  0.57 !             \\ /
ATOM O2     OG2D4  -0.49 !          H82 C9
ATOM N3     NG2R61 -0.47 !            \ |
ATOM H3     HGP1    0.33 !         H81--C8  O4
ATOM C4     CG2R63  0.49 !              |   ||
ATOM O4     OG2D4  -0.49 !              O7  C4    H3
ATOM C5     CG2R62  0.17 !               \ / \   /
ATOM C6     CG2R62  0.21 !                C5   N3
ATOM H6     HGR62   0.16 !                ||   |
ATOM O7     OG301  -0.43 !             H6-C6   C2
ATOM C8     CG321   0.07 !                 \ /  \\
ATOM H81    HGA2    0.09 !                  N1   O2
ATOM H82    HGA2    0.09 !                   \
GROUP                    !                    \
```

```
ATOM C9      CG2O3   0.52 !                      \
ATOM O91     OG2D2  -0.76 ! O1P    H5' H4'  O4'  \
ATOM O92     OG2D2  -0.76 !  |      |    \ /   \  \
GROUP                     ! -P-O5'-C5'---C4'    C1'
ATOM P       P       1.50 !  |      |     \     / \
ATOM O1P     ON3    -0.78 ! O2P    H5''   C3'--C2' H1'
ATOM O2P     ON3    -0.78 !              / \   / \
ATOM O5'     ON2    -0.57 !            O3' H3' O2' H2''
ATOM C5'     CN8B   -0.08 !             |       |
ATOM H5'     HN8     0.09 !                     H2'
ATOM H5''    HN8     0.09
GROUP                     !  !!!! PATCH 5UHO for the carboxylic acid form
ATOM C4'     CN7     0.16 !  !!!! PATCH ENOU for the enol tautomer
ATOM H4'     HN7     0.09
ATOM O4'     ON6B   -0.50
ATOM C1'     CN7B    0.16
ATOM H1'     HN7     0.09
GROUP
ATOM C2'     CN7B    0.14
ATOM H2''    HN7     0.09
ATOM O2'     ON5    -0.66
ATOM H2'     HN5     0.43
GROUP
ATOM C3'     CN7     0.01
ATOM H3'     HN7     0.09
ATOM O3'     ON2    -0.57
BOND N1   C2      N1   C6      C2   O2      C2   N3
BOND N3   C4      N3   H3      C4   O4      C4   C5
BOND C5   C6      C5   O7      C6   H6      O7   C8
BOND C8   H81     C8   H82     C8   C9      C9   O91
BOND C9   O92
BOND P    O1P     P    O2P     P    O5'     O5'  C5'     C5'  H5''
BOND C5'  C4'     C4'  O4'     C4'  C3'     O4'  C1'
BOND C1'  N1      C1'  C2'     C2'  C3'     C3'  O3'     O3'  +P
BOND C2'  O2'     O2'  H2'
BOND C1'  H1'     C2'  H2''    C3'  H3'     C4'  H4'     C5'  H5'
IMPR C2     N1     N3     O2     C4     C5     N3     O4     C9     O92    O91    C8
!ribose
IC -O3' P    O5'  C5'    1.6001  101.45  -39.25  119.00  1.4401
IC -O3' O5'  *P   O1P    1.6001  101.45 -115.82  109.74  1.4802
IC -O3' O5'  *P   O2P    1.6001  101.45  115.90  109.80  1.4801
IC  P   O5'  C5'  C4'    1.5996  119.00 -151.39  110.04  1.5160
IC O5'  C5'  C4'  C3'    1.4401  108.83 -179.85  116.10  1.5284
IC C5'  C4'  C3'  O3'    1.5160  116.10   76.70  115.12  1.4212
IC C4'  C3'  O3'  +P     1.5284  111.92  159.13  119.05  1.6001
IC C3'  O3'  +P   +O5'   1.4212  119.05  -98.86  101.45  1.5996
IC O4'  C3'  *C4' C5'    1.4572  104.06 -120.04  116.10  1.5160
IC C2'  C4'  *C3' O3'    1.5284  100.16 -124.08  115.12  1.4212
IC C4'  C3'  C2'  C1'    1.5284  100.16   39.58  102.04  1.5251
IC C3'  C2'  C1'  N1     1.5284  101.97  144.39  113.71  1.4896
IC O4'  C1'  N1   C2     1.5251  113.71  -96.0   117.06  1.3746
IC C1'  C3'  *C2' O2'    1.5284  102.04 -114.67  110.81  1.4212
IC H2'  O2'  C2'  C3'    0.9600  114.97  148.63  111.92  1.5284
IC O4'  C2'  *C1' H1'    0.0       0.0  -115.0     0.0   0.0
IC C1'  C3'  *C2' H2''   0.0       0.0   115.0     0.0   0.0
IC C2'  C4'  *C3' H3'    0.0       0.0   115.0     0.0   0.0
IC C3'  O4'  *C4' H4'    0.0       0.0  -115.0     0.0   0.0
IC C4'  O5'  *C5' H5'    0.0       0.0  -115.0     0.0   0.0
IC C4'  O5'  *C5' H5''   0.0       0.0   115.0     0.0   0.0
IC C2   C6   *N1  C1'       1.3966  121.57  179.97  122.49  1.4896
IC C3'  C2'  C1'  N1     1.5284  101.97  144.39  113.71  1.4896
IC O4'  C1'  N1   C2     1.5251  113.71  -96.0   117.06  1.3746
IC C1'  C2   *N1  C6     1.3966  121.57  179.97  122.49  1.4896
```

```
IC C6   N1   C2    N3        1.3738  119.76    0.44  116.60  1.3706
IC N3   N1    *C2  O2        1.3706  116.60 -179.52  122.95  1.2315
IC N1   C2   N3    C4        1.4084  116.60   -0.35  125.93  1.3832
IC C4   C2    *N3  H3        1.3832  125.93  178.73  117.11  1.0017
IC C2   N3   C4    C5        1.3706  125.93   -0.72  116.28  1.4551
IC C5   N3    *C4  O4        1.4551  116.28 -178.76  117.25  1.2332
IC C5   N1    *C6  H6        1.3737  123.81 -177.10  117.37  1.0919
IC C6   C4    *C5  O7        1.3737  117.59  179.41  123.73  1.3629
IC C4   C5   O7    C8        1.4551  123.73  -81.97  112.33  1.4456
IC C5   O7   C8    C9        1.3629  112.33  -71.01  117.76  1.5464
IC C9   O7    *C8  H81       1.5464  117.76 -119.31  107.75  1.1107
IC H81  O7    *C8  H82       1.1107  107.75 -116.95  110.67  1.1112
IC O7   C8   C9    O91       1.4456  117.76   -1.66  118.47  1.2579
IC O91  C8    *C9  O92       1.2579  118.47  179.71  114.15  1.2588
DONO H2'  O2'
DONO H3   N3
ACCE O2   C2
ACCE O4   C4
ACCE O7
ACCE O91  C9
ACCE O92  C9
ACCE O1P  P
ACCE O2P  P
ACCE O2'
ACCE O3'
ACCE O4'
ACCE O5'

RESI OEU           -1.00 ! uridine 5-oxyacetic acid methyl ester    ! adjusted
GROUP
ATOM N1     NG2R61 -0.30 !             H111 H112
ATOM C2     CG2R63  0.57 !               \  /
ATOM O2     OG2D4  -0.49 !                C11-H113
ATOM N3     NG2R61 -0.47 !                |
ATOM H3     HGP1    0.33 !           O9   O10
ATOM C4     CG2R63  0.49 !            \\ /
ATOM O4     OG2D4  -0.49 !         H82 C9
ATOM C5     CG2R62  0.17 !            \ |
ATOM C6     CG2R62  0.21 !         H81-C8   O4
ATOM H6     HGR62   0.16 !              |   ||
ATOM O7     OG301  -0.43 !             O7   C4    H3
ATOM C8     CG321   0.07 !               \ /  \  /
ATOM H81    HGA2    0.09 !                C5   N3
ATOM H82    HGA2    0.09 !                ||   |
GROUP                    !             H6-C6   C2
ATOM C9     CG2O2   0.86 !                 \  / \\
ATOM O9     OG2D1  -0.63 !                  N1   O2
ATOM O10    OG302  -0.49 !                   \
ATOM C11    CG331  -0.01 !                    \
ATOM H111   HGA3    0.09 !                     \
ATOM H112   HGA3    0.09 ! O1P    H5' H4'  O4'  \
ATOM H113   HGA3    0.09 !  |      |    \ /   \  \
GROUP                    ! -P-O5'-C5'---C4'    C1'
ATOM P      P       1.50 !  |      |     \     / \
ATOM O1P    ON3    -0.78 ! O2P    H5''   C3'--C2' H1'
ATOM O2P    ON3    -0.78 !              / \   / \
ATOM O5'    ON2    -0.57 !            O3' H3' O2' H2''
ATOM C5'    CN8B   -0.08 !             |       |
ATOM H5'    HN8     0.09 !                    H2'
ATOM H5''   HN8     0.09
GROUP                    !!! PATCH ENOU for the enol tautomer
ATOM C4'    CN7     0.16
ATOM H4'    HN7     0.09
```

```
ATOM O4'    ON6B   -0.50
ATOM C1'    CN7B    0.16
ATOM H1'    HN7     0.09
GROUP
ATOM C2'    CN7B    0.14
ATOM H2''   HN7     0.09
ATOM O2'    ON5    -0.66
ATOM H2'    HN5     0.43
GROUP
ATOM C3'    CN7     0.01
ATOM H3'    HN7     0.09
ATOM O3'    ON2    -0.57
BOND N1   C2      N1   C6      C2   O2      C2   N3
BOND N3   C4      N3   H3      C4   O4      C4   C5
BOND C5   C6      C5   O7      C6   H6      O7   C8
BOND C8   H81     C8   H82     C8   C9      C9   O9
BOND C9   O10     O10  C11     C11  H111    C11  H112
BOND C11  H113
BOND P    O1P     P    O2P     P    O5'     O5'  C5'     C5'  H5''
BOND C5'  C4'     C4'  O4'     C4'  C3'     O4'  C1'
BOND C1'  N1      C1'  C2'     C2'  C3'     C3'  O3'     O3'  +P
BOND C2'  O2'     O2'  H2'
BOND C1'  H1'     C2'  H2''    C3'  H3'     C4'  H4'     C5'  H5'
IMPR C2     N1     N3     O2     C4     C5     N3     O4     C9     C8     O9     O10
!ribose
IC -O3' P    O5'  C5'    1.6001  101.45  -39.25  119.00  1.4401
IC -O3' O5'  *P   O1P    1.6001  101.45 -115.82  109.74  1.4802
IC -O3' O5'  *P   O2P    1.6001  101.45  115.90  109.80  1.4801
IC  P   O5'  C5'  C4'    1.5996  119.00 -151.39  110.04  1.5160
IC O5'  C5'  C4'  C3'    1.4401  108.83 -179.85  116.10  1.5284
IC C5'  C4'  C3'  O3'    1.5160  116.10   76.70  115.12  1.4212
IC C4'  C3'  O3'  +P     1.5284  111.92  159.13  119.05  1.6001
IC C3'  O3'  +P   +O5'   1.4212  119.05  -98.86  101.45  1.5996
IC O4'  C3'  *C4' C5'    1.4572  104.06 -120.04  116.10  1.5160
IC C2'  C4'  *C3' O3'    1.5284  100.16 -124.08  115.12  1.4212
IC C4'  C3'  C2'  C1'    1.5284  100.16   39.58  102.04  1.5251
IC C3'  C2'  C1'  N1     1.5284  101.97  144.39  113.71  1.4896
IC O4'  C1'  N1   C2     1.5251  113.71  -96.0   117.06  1.3746
IC C1'  C3'  *C2' O2'    1.5284  102.04 -114.67  110.81  1.4212
IC H2'  O2'  C2'  C3'    0.9600  114.97  148.63  111.92  1.5284
IC O4'  C2'  *C1' H1'    0.0       0.0  -115.0     0.0   0.0
IC C1'  C3'  *C2' H2''   0.0       0.0   115.0     0.0   0.0
IC C2'  C4'  *C3' H3'    0.0       0.0   115.0     0.0   0.0
IC C3'  O4'  *C4' H4'    0.0       0.0  -115.0     0.0   0.0
IC C4'  O5'  *C5' H5'    0.0       0.0  -115.0     0.0   0.0
IC C4'  O5'  *C5' H5''   0.0       0.0   115.0     0.0   0.0
IC C2   C6   *N1  C1'      1.3966  121.57  179.97  122.49  1.4896
IC C3'  C2'  C1'  N1     1.5284  101.97  144.39  113.71  1.4896
IC O4'  C1'  N1   C2     1.5251  113.71  -96.0   117.06  1.3746
IC C1'  C2   *N1  C6     1.3966  121.57  179.97  122.49  1.4896
IC C6   N1   C2   N3       1.3710  119.94   -0.46  116.46  1.3700
IC N3   N1   *C2  O2       1.3700  116.46 -179.84  122.91  1.2283
IC N1   C2   N3   C4       1.4088  116.46   -0.66  126.16  1.3821
IC C4   C2   *N3  H3       1.3821  126.16 -177.75  116.63  1.0036
IC C2   N3   C4   C5       1.3700  126.16    2.38  116.13  1.4683
IC C5   N3   *C4  O4       1.4683  116.13  179.92  116.95  1.2314
IC C5   N1   *C6  H6       1.3730  124.12  179.13  116.74  1.0892
IC C6   C4   *C5  O7       1.3730  117.12 -174.45  125.89  1.3723
IC C4   C5   O7   C8       1.4683  125.89  -23.68  117.27  1.4409
IC C5   O7   C8   C9       1.3723  117.27 -161.60  113.24  1.5411
IC C9   O7   *C8  H81      1.5411  113.24 -119.77  109.68  1.1111
IC H81  O7   *C8  H82      1.1111  109.68 -120.74  110.99  1.1118
IC O7   C8   C9   O10      1.4409  113.24 -177.34  108.89  1.3398
```

```
IC O10  C8   *C9   O9        1.3398  108.89 -179.46  126.60  1.2193
IC C8   C9   O10   C11       1.5411  108.89 -179.91  112.37  1.4387
IC C9   O10  C11   H111      1.3398  112.37 -179.80  109.85  1.1115
IC H111 O10  *C11  H112      1.1115  109.85  119.49  110.78  1.1140
IC H111 O10  *C11  H113      1.1115  109.85 -119.68  110.89  1.1133
DONO H2'  O2'
DONO H3   N3
ACCE O2   C2
ACCE O4   C4
ACCE O7
ACCE O9   C9
ACCE O9
ACCE O1P  P
ACCE O2P  P
ACCE O2'
ACCE O3'
ACCE O4'
ACCE O5'

RESI 3AU            -1.00 ! 3-(3-amino-3-carboxypropyl)uridine
GROUP
ATOM N1     NG2R61 -0.21 !                   O4    H101  H111 H12    O30 (-)
ATOM C2     CG2R63  0.52 !                   ||    |     |    |     /
ATOM O2     OG2D4  -0.44 !                   C4    C10---C11--C12--C13
ATOM N3     NG2R61 -0.32 !                  /  \  / \    |    |     \\
ATOM C4     CG2R63  0.50 !              H5-C5   N3  H102 H112 |(+)   O31
ATOM O4     OG2D4  -0.48 !                 ||   |             N14
ATOM C5     CG2R62 -0.22 !              H6-C6   C2          / |  \
ATOM H5     HGR62   0.09 !                  \  / \\     H141 H142 H143
ATOM C6     CG2R62  0.31 !                   N1   O2
ATOM H6     HGR62   0.11 !                    \
ATOM C10    CG321  -0.04 !                     \
ATOM H101   HGA2    0.09 !                      \
ATOM H102   HGA2    0.09 !  O1P    H5' H4'  O4'  \
GROUP                    !   |      |    \ /   \  \
ATOM C11    CG321  -0.18 !  -P-O5'-C5'---C4'    C1'
ATOM H111   HGA2    0.09 !   |      |     \     / \
ATOM H112   HGA2    0.09 !  O2P    H5''   C3'--C2' H1'
GROUP                    !               / \   / \
ATOM C12    CG314   0.17 !             O3' H3' O2' H2''
ATOM H12    HGA1    0.11 !              |       |
ATOM N14    NG3P3  -0.34 !                     H2'
ATOM H141   HGP2    0.30
ATOM H142   HGP2    0.30 !  !!!! PATCH 5UHA for the non-ionic tautomer
ATOM H143   HGP2    0.30
ATOM C13    CG2O3   0.32
ATOM O30    OG2D2  -0.58
ATOM O31    OG2D2  -0.58
GROUP
ATOM P      P       1.50
ATOM O1P    ON3    -0.78
ATOM O2P    ON3    -0.78
ATOM O5'    ON2    -0.57
ATOM C5'    CN8B   -0.08
ATOM H5'    HN8     0.09
ATOM H5''   HN8     0.09
GROUP
ATOM C4'    CN7     0.16
ATOM H4'    HN7     0.09
ATOM O4'    ON6B   -0.50
ATOM C1'    CN7B    0.16
ATOM H1'    HN7     0.09
GROUP
```

```
ATOM C2'    CN7B    0.14
ATOM H2''   HN7     0.09
ATOM O2'    ON5    -0.66
ATOM H2'    HN5     0.43
GROUP
ATOM C3'    CN7     0.01
ATOM H3'    HN7     0.09
ATOM O3'    ON2    -0.57
BOND N1   C2      N1   C6      C2   O2      C2   N3
BOND N3   C4      N3   C10     C4   O4      C4   C5
BOND C5   C6      C5   H5      C6   H6      C10  C11
BOND C10  H101    C10  H102    C11  C12     C11  H111
BOND C11  H112    C12  H12     C12  N14     C12  C13
BOND N14  H141    N14  H142    N14  H143    C13  O30
BOND C13  O31
BOND P    O1P     P    O2P     P    O5'     O5'  C5'     C5'  H5''
BOND C5'  C4'     C4'  O4'     C4'  C3'     O4'  C1'
BOND C1'  N1      C1'  C2'     C2'  C3'     C3'  O3'     O3'  +P
BOND C2'  O2'     O2'  H2'
BOND C1'  H1'     C2'  H2''    C3'  H3'     C4'  H4'     C5'  H5'
IMPR C2     N1     N3     O2     C4     C5     N3     O4     C13    O30    O31    C12
!ribose
IC -O3' P    O5'  C5'    1.6001  101.45  -39.25  119.00  1.4401
IC -O3' O5'  *P   O1P    1.6001  101.45 -115.82  109.74  1.4802
IC -O3' O5'  *P   O2P    1.6001  101.45  115.90  109.80  1.4801
IC  P   O5'  C5'  C4'    1.5996  119.00 -151.39  110.04  1.5160
IC O5'  C5'  C4'  C3'    1.4401  108.83 -179.85  116.10  1.5284
IC C5'  C4'  C3'  O3'    1.5160  116.10   76.70  115.12  1.4212
IC C4'  C3'  O3'  +P     1.5284  111.92  159.13  119.05  1.6001
IC C3'  O3'  +P   +O5'   1.4212  119.05  -98.86  101.45  1.5996
IC O4'  C3'  *C4' C5'    1.4572  104.06 -120.04  116.10  1.5160
IC C2'  C4'  *C3' O3'    1.5284  100.16 -124.08  115.12  1.4212
IC C4'  C3'  C2'  C1'    1.5284  100.16   39.58  102.04  1.5251
IC C3'  C2'  C1'  N1     1.5284  101.97  144.39  113.71  1.4896
IC O4'  C1'  N1   C2     1.5251  113.71  -96.0   117.06  1.3746
IC C1'  C3'  *C2' O2'    1.5284  102.04 -114.67  110.81  1.4212
IC H2'  O2'  C2'  C3'    0.9600  114.97  148.63  111.92  1.5284
IC O4'  C2'  *C1' H1'    0.0       0.0  -115.0     0.0   0.0
IC C1'  C3'  *C2' H2''   0.0       0.0   115.0     0.0   0.0
IC C2'  C4'  *C3' H3'    0.0       0.0   115.0     0.0   0.0
IC C3'  O4'  *C4' H4'    0.0       0.0  -115.0     0.0   0.0
IC C4'  O5'  *C5' H5'    0.0       0.0  -115.0     0.0   0.0
IC C4'  O5'  *C5' H5''   0.0       0.0   115.0     0.0   0.0
IC C2   C6   *N1  C1'       1.3966  121.57  179.97  122.49  1.4896
IC C3'  C2'  C1'  N1     1.5284  101.97  144.39  113.71  1.4896
IC O4'  C1'  N1   C2     1.5251  113.71  -96.0   117.06  1.3746
IC C1'  C2   *N1  C6     1.3966  121.57  179.97  122.49  1.4896
IC C6   N1   C2   N3        1.3608  119.99    0.36  117.13  1.4018
IC N3   N1   *C2  O2        1.4018  117.13  179.69  121.26  1.2317
IC N1   C2   N3   C10       1.4077  117.13 -178.20  118.56  1.4766
IC C10  C2   *N3  C4        1.4766  118.56  176.26  123.32  1.4021
IC C2   N3   C4   O4        1.4018  123.32 -176.30  119.66  1.2336
IC O4   N3   *C4  C5        1.2336  119.66  179.26  116.79  1.4253
IC C6   C4   *C5  H5        1.3664  119.35 -177.64  120.50  1.0882
IC C5   N1   *C6  H6        1.3664  123.36  179.62  116.15  1.0914
IC C2   N3   C10  C11       1.4018  118.56   95.55  112.96  1.5431
IC C11  N3   *C10 H101      1.5431  112.96  123.69  107.79  1.1118
IC C11  N3   *C10 H102      1.5431  112.96 -122.28  109.29  1.1155
IC N3   C10  C11  C12       1.4766  112.96  126.55  114.79  1.5531
IC C12  C10  *C11 H111      1.5531  114.79  120.49  107.80  1.1151
IC C12  C10  *C11 H112      1.5531  114.79 -124.14  111.37  1.1137
IC C10  C11  C12  C13       1.5431  114.79  177.96  110.39  1.5422
IC C13  C11  *C12 N14       1.5422  110.39  119.42  110.07  1.4898
```

```
IC N14  C11  *C12  H12       1.4898  110.07  121.15  110.27  1.1102
IC C11  C12  N14   H141      1.5531  110.07 -107.61   99.55  1.0568
IC H141 C12  *N14  H142      1.0568   99.55  117.42  111.79  1.0461
IC H141 C12  *N14  H143      1.0568   99.55 -114.93  111.81  1.0324
IC C11  C12  C13   O30       1.5531  110.39  116.32  116.39  1.2628
IC O30  C12  *C13  O31       1.2628  116.39  179.86  115.21  1.2507
DONO H2'  O2'
DONO H141 N14
DONO H142 N14
DONO H143 N14
ACCE O2   C2
ACCE O4   C4
ACCE O30  C13
ACCE O31  C13
ACCE O1P  P
ACCE O2P  P
ACCE O2'
ACCE O3'
ACCE O4'
ACCE O5'

RESI 13P           -1.00 ! 1-methyl-3-(3-amino-3-carboxypropyl)pseudouridine
GROUP
ATOM C5     CG2R62 -0.20 !          H1M1    O2     H101 H111 H12   O30 (-)
ATOM C4     CG2R63  0.74 !             \    ||     /    |    |    /
ATOM O4     OG2D4  -0.52 !        H1M2-C1M  C2    C10---C11--C12-C13
ATOM N3     NG2R61 -0.44 !             / \ /  \  / \    |    |    \\
ATOM C2     CG2R63  0.86 !          H1M3  N1   N3  H102 H112 |(+)  O31
ATOM O2     OG2D4  -0.54 !                |    |             N14
ATOM N1     NG2R61 -0.33 !             H6-C6   C4          / |  \
ATOM C6     CG2R62  0.03 !                 \  / \\     H141 H142 H143
ATOM H6     HGR62   0.18 !                  C5   O4
ATOM C1M    CG331  -0.15 !                   \
ATOM H1M1   HGA3    0.09 !                    \
ATOM H1M2   HGA3    0.09 !                     \
ATOM H1M3   HGA3    0.09 ! O1P    H5' H4'  O4'  \
ATOM C10    CG321  -0.08 !  |       |    \ /   \  \
ATOM H101   HGA2    0.09 ! -P-O5'-C5'---C4'    C1'
ATOM H102   HGA2    0.09 !  |       |     \     / \
GROUP                    ! O2P    H5''   C3'--C2' H1'
ATOM C11    CG321  -0.18 !              / \   / \
ATOM H111   HGA2    0.09 !            O3' H3' O2' H2''
ATOM H112   HGA2    0.09 !             |       |
GROUP                    !                     H2'
ATOM C12    CG314   0.17
ATOM H12    HGA1    0.11 !  !!!! PATCH 5UHA for the non-ionic tautomer
ATOM N14    NG3P3  -0.34
ATOM H141   HGP2    0.30
ATOM H142   HGP2    0.30
ATOM H143   HGP2    0.30
ATOM C13    CG2O3   0.32
ATOM O30    OG2D2  -0.58
ATOM O31    OG2D2  -0.58
GROUP
ATOM P      P       1.50
ATOM O1P    ON3    -0.78
ATOM O2P    ON3    -0.78
ATOM O5'    ON2    -0.57
ATOM C5'    CN8B   -0.08
ATOM H5'    HN8     0.09
ATOM H5''   HN8     0.09
GROUP
ATOM C1'    CN7B    0.12
```

```
ATOM H1'    HN7     0.09
ATOM C4'    CN7     0.16
ATOM O4'    ON6B   -0.46
ATOM H4'    HN7     0.09
GROUP
ATOM C2'    CN7B    0.14
ATOM H2''   HN7     0.09
ATOM O2'    ON5    -0.66
ATOM H2'    HN5     0.43
GROUP
ATOM C3'    CN7     0.01
ATOM H3'    HN7     0.09
ATOM O3'    ON2    -0.57
BOND C5   C4   C5   C6   C4   O4   C4   N3
BOND N3   C2   N3   C10  C2   O2   C2   N1
BOND N1   C6   N1   C1M  C6   H6   C1M  H1M1
BOND C1M  H1M2 C1M  H1M3 C10  H101 C10  H102
BOND C10  C11  C11  H111 C11  H112 C11  C12
BOND C12  H12  C12  N14  C12  C13  N14  H141
BOND N14 H142 N14 H143 C13  O30  C13  O31
BOND P    O1P     P    O2P     P    O5'     O5'  C5'     C5'  H5''
BOND C5'  C4'     C4'  O4'     C4'  C3'     O4'  C1'
BOND C1'  C5      C1'  C2'     C2'  C3'     C3'  O3'     O3'  +P
BOND C2'  O2'     O2'  H2'
BOND C1'  H1'     C2'  H2''    C3'  H3'     C4'  H4'     C5'  H5'
IMPR C4     C5     N3     O4     C2     N3     N1     O2     C13    O30    O31    C12
!ribose
IC -O3' P    O5'   C5'    1.6001  101.45  -39.25  119.00  1.4401
IC -O3' O5'  *P    O1P    1.6001  101.45 -115.82  109.74  1.4802
IC -O3' O5'  *P    O2P    1.6001  101.45  115.90  109.80  1.4801
IC  P   O5'  C5'   C4'    1.5996  119.00 -151.39  110.04  1.5160
IC O5'  C5'  C4'   C3'    1.4401  108.83 -179.85  116.10  1.5284
IC C5'  C4'  C3'   O3'    1.5160  116.10   76.70  115.12  1.4212
IC C4'  C3'  O3'   +P     1.5284  111.92  159.13  119.05  1.6001
IC C3'  O3'  +P    +O5'   1.4212  119.05  -98.86  101.45  1.5996
IC O4'  C3'  *C4'  C5'    1.4572  104.06 -120.04  116.10  1.5160
IC C2'  C4'  *C3'  O3'    1.5284  100.16 -124.08  115.12  1.4212
IC C4'  C3'  C2'   C1'    1.5284  100.16   39.58  102.04  1.5251
IC C5   C1'  O4'   C4'    1.4022  102.36 -123.09  109.06  1.4572
IC C6   C5   C1'   C2'    1.3750  121.43  -77.12  118.79  1.5251
IC C1'  C3'  *C2'  O2'    1.5284  102.04 -114.67  110.81  1.4212
IC H2'  O2'  C2'   C3'    0.9600  114.97  148.63  111.92  1.5284
IC O4'  C2'  *C1'  H1'    0.0       0.0  -115.0     0.0   0.0
IC C1'  C3'  *C2'  H2''   0.0       0.0   115.0     0.0   0.0
IC C2'  C4'  *C3'  H3'    0.0       0.0   115.0     0.0   0.0
IC C3'  O4'  *C4'  H4'    0.0       0.0  -115.0     0.0   0.0
IC C4'  O5'  *C5'  H5'    0.0       0.0  -115.0     0.0   0.0
IC C4'  O5'  *C5'  H5''   0.0       0.0   115.0     0.0   0.0
IC C4   C1'  *C5   C6     1.4540  120.46 -172.70  121.43  1.3750
IC C6   C5   C4    N3        1.3660  118.49   -7.00  117.65  1.3984
IC N3   C5   *C4   O4        1.3984  117.65  178.25  123.75  1.2370
IC C5   C4   N3    C10       1.4497  117.65 -177.28  119.62  1.4810
IC C10  C4   *N3   C2        1.4810  119.62 -177.91  122.21  1.3935
IC C4   N3   C2    N1        1.3984  122.21   -0.50  117.86  1.4093
IC N1   N3   *C2   O2        1.4093  117.86 -179.83  121.22  1.2313
IC N1   C5   *C6   H6        1.3585  122.78 -178.75  120.26  1.0945
IC C6   C2   *N1   C1M       1.3585  120.68 -179.18  118.19  1.4748
IC C2   N1   C1M   H1M1      1.4093  118.19  -59.56  111.19  1.1159
IC H1M1 N1   *C1M  H1M2      1.1159  111.19  119.30  111.36  1.1148
IC H1M1 N1   *C1M  H1M3      1.1159  111.19 -120.19  112.64  1.1135
IC C4   N3   C10   C11       1.3984  119.62  -94.09  113.57  1.5438
IC C11  N3   *C10  H101      1.5438  113.57 -120.33  108.16  1.1187
IC H101 N3   *C10  H102      1.1187  108.16 -114.38  109.35  1.1132
```

```
IC N3   C10  C11   C12       1.4810  113.57   59.92  116.24  1.5542
IC C12  C10  *C11  H111      1.5542  116.24 -121.64  108.51  1.1161
IC H111 C10  *C11  H112      1.1161  108.51 -114.91  108.76  1.1132
IC C10  C11  C12   C13       1.5438  116.24  178.42  112.06  1.5385
IC C13  C11  *C12  N14       1.5385  112.06 -122.74  109.94  1.4895
IC N14  C11  *C12  H12       1.4895  109.94 -119.42  109.80  1.1103
IC C11  C12  N14   H141      1.5542  109.94   43.09  107.87  1.0356
IC H141 C12  *N14  H142      1.0356  107.87  114.02  103.55  1.0468
IC H141 C12  *N14  H143      1.0356  107.87 -123.63  110.48  1.0442
IC C11  C12  C13   O30       1.5542  112.06 -123.89  116.34  1.2624
IC O30  C12  *C13  O31       1.2624  116.34 -178.92  115.32  1.2509
DONO H2'  O2'
DONO H141 N14
DONO H142 N14
DONO H143 N14
ACCE O2   C2
ACCE O4   C4
ACCE O30  C13
ACCE O31  C13
ACCE O1P  P
ACCE O2P  P
ACCE O2'
ACCE O3'
ACCE O4'
ACCE O5'

RESI 5TU           -1.00 ! 5-taurinomethyluridine, 5-{[(2-
sulfoethyl)amino]methyl}uridine, TM2
GROUP
ATOM N1     NG2R61 -0.34 !      O12 (-)
ATOM C2     CG2R63  0.51 !       |
ATOM O2     OG2D4  -0.41 ! O13 =S11=O11
ATOM N3     NG2R61 -0.46 !       |
ATOM H3     HGP1    0.36 ! H101-C10-H102
ATOM C4     CG2R63  0.50 !       |
ATOM O4     OG2D4  -0.45 !       |   H81 H72  O4
ATOM C5     CG2R62 -0.05 !       |   |(+) |   ||
ATOM C6     CG2R62  0.17 !  H91-C9 -N8 -C7    C4    H3
ATOM H6     HGR62   0.17 !       |   |   / \ /  \  /
GROUP                    !     H92 H82 H71 C5    N3
ATOM C7     CG324   0.14 !                 ||    |
ATOM H71    HGA2    0.09 !              H6-C6    C2
ATOM H72    HGA2    0.09 !                  \  / \\
ATOM N8     NG3P2  -0.30 !                   N1    O2
ATOM H81    HGP2    0.33 !                    \
ATOM H82    HGP2    0.33 !                     \
ATOM C9     CG324   0.14 !                      \
ATOM H91    HGA2    0.09 !  O1P    H5' H4'  O4'  \
ATOM H92    HGA2    0.09 !   |      |    \ /   \  \
GROUP                    !  -P-O5'-C5'---C4'    C1'
ATOM C10    CG321  -0.26 !   |      |     \     / \
ATOM H101   HGA2    0.09 !  O2P    H5''   C3'--C2' H1'
ATOM H102   HGA2    0.09 !               / \   / \
ATOM S11    SG3O1   0.73 !             O3' H3' O2' H2''
ATOM O11    OG2P1  -0.55 !              |       |
ATOM O12    OG2P1  -0.55 !                     H2'
ATOM O13    OG2P1  -0.55
GROUP
ATOM P      P       1.50
ATOM O1P    ON3    -0.78
ATOM O2P    ON3    -0.78
ATOM O5'    ON2    -0.57
ATOM C5'    CN8B   -0.08
```

```
ATOM H5'    HN8      0.09
ATOM H5''   HN8      0.09
GROUP
ATOM C4'    CN7      0.16
ATOM H4'    HN7      0.09
ATOM O4'    ON6B    -0.50
ATOM C1'    CN7B     0.16
ATOM H1'    HN7      0.09
GROUP
ATOM C2'    CN7B     0.14
ATOM H2''   HN7      0.09
ATOM O2'    ON5     -0.66
ATOM H2'    HN5      0.43
GROUP
ATOM C3'    CN7      0.01
ATOM H3'    HN7      0.09
ATOM O3'    ON2     -0.57
BOND N1   C2      N1   C6       C2   O2       C2   N3
BOND N3   C4      N3   H3       C4   O4       C4   C5
BOND C5   C6      C5   C7       C6   H6       C7   H71
BOND C7   H72     C7   N8       C9   C10      C9   H91
BOND C9   H92     C9   N8       C10  H101     C10  H102
BOND C10  S11     N8   H81      N8   H82      S11  O11
BOND S11  O12     S11  O13
BOND P    O1P     P    O2P     P    O5'     O5'  C5'     C5'  H5''
BOND C5'  C4'     C4'  O4'     C4'  C3'     O4'  C1'
BOND C1'  N1      C1'  C2'     C2'  C3'     C3'  O3'     O3'  +P
BOND C2'  O2'     O2'  H2'
BOND C1'  H1'     C2'  H2''    C3'  H3'     C4'  H4'     C5'  H5'
IMPR C2     N1     N3     O2     C4     C5     N3     O4
!ribose
IC -O3' P    O5'   C5'    1.6001  101.45  -39.25  119.00  1.4401
IC -O3' O5'  *P    O1P    1.6001  101.45 -115.82  109.74  1.4802
IC -O3' O5'  *P    O2P    1.6001  101.45  115.90  109.80  1.4801
IC  P   O5'  C5'   C4'    1.5996  119.00 -151.39  110.04  1.5160
IC O5'  C5'  C4'   C3'    1.4401  108.83 -179.85  116.10  1.5284
IC C5'  C4'  C3'   O3'    1.5160  116.10   76.70  115.12  1.4212
IC C4'  C3'  O3'   +P     1.5284  111.92  159.13  119.05  1.6001
IC C3'  O3'  +P    +O5'   1.4212  119.05  -98.86  101.45  1.5996
IC O4'  C3'  *C4'  C5'    1.4572  104.06 -120.04  116.10  1.5160
IC C2'  C4'  *C3'  O3'    1.5284  100.16 -124.08  115.12  1.4212
IC C4'  C3'  C2'   C1'    1.5284  100.16   39.58  102.04  1.5251
IC C3'  C2'  C1'   N1     1.5284  101.97  144.39  113.71  1.4896
IC O4'  C1'  N1    C2     1.5251  113.71  -96.0   117.06  1.3746
IC C1'  C3'  *C2'  O2'    1.5284  102.04 -114.67  110.81  1.4212
IC H2'  O2'  C2'   C3'    0.9600  114.97  148.63  111.92  1.5284
IC O4'  C2'  *C1'  H1'    0.0       0.0  -115.0     0.0   0.0
IC C1'  C3'  *C2'  H2''   0.0       0.0   115.0     0.0   0.0
IC C2'  C4'  *C3'  H3'    0.0       0.0   115.0     0.0   0.0
IC C3'  O4'  *C4'  H4'    0.0       0.0  -115.0     0.0   0.0
IC C4'  O5'  *C5'  H5'    0.0       0.0  -115.0     0.0   0.0
IC C4'  O5'  *C5'  H5''   0.0       0.0   115.0     0.0   0.0
IC C2   C6   *N1   C1'       1.3966  121.57  179.97  122.49  1.4896
IC C3'  C2'  C1'   N1     1.5284  101.97  144.39  113.71  1.4896
IC O4'  C1'  N1    C2     1.5251  113.71  -96.0   117.06  1.3746
IC C1'  C2   *N1   C6     1.3966  121.57  179.97  122.49  1.4896
IC C6   N1   C2    N3        1.3658  120.44    0.07  116.12  1.3654
IC N3   N1   *C2   O2        1.3654  116.12  179.91  123.16  1.2275
IC N1   C2   N3    C4        1.4082  116.12   -0.03  126.06  1.3735
IC C4   C2   *N3   H3        1.3735  126.06  179.95  116.44  1.0027
IC C2   N3   C4    C5        1.3654  126.06   -0.03  116.89  1.4442
IC C5   N3   *C4   O4        1.4442  116.89 -179.94  118.30  1.2265
IC C5   N1   *C6   H6        1.3847  123.13 -179.96  114.60  1.0916
```

```
IC C6   C4   *C5   C7        1.3847  117.36  179.95  111.96  1.4821
IC C4   C5   C7    N8        1.4442  111.96 -179.98  110.67  1.4834
IC N8   C5   *C7   H71       1.4834  110.67  117.93  110.72  1.1070
IC H71  C5   *C7   H72       1.1070  110.72  124.17  110.74  1.1071
IC C5   C7   N8    C9        1.4821  110.67  179.99  114.96  1.5016
IC C9   C7   *N8   H81       1.5016  114.96  120.35  111.64  1.0091
IC C9   C7   *N8   H82       1.5016  114.96 -120.31  111.62  1.0089
IC C7   N8   C9    C10       1.4834  114.96  179.98  105.11  1.5156
IC C10  N8   *C9   H91       1.5156  105.11  121.87  105.31  1.1061
IC C10  N8   *C9   H92       1.5156  105.11 -121.76  105.28  1.1066
IC N8   C9   C10   S11       1.5016  105.11 -179.97  103.34  1.7701
IC S11  C9   *C10  H101      1.7701  103.34  115.51  114.22  1.1072
IC H101 C9   *C10  H102      1.1072  114.22  128.97  114.16  1.1069
IC C9   C10  S11   O11       1.5156  103.34 -179.99  102.44  1.4409
IC O11  C10  *S11  O12       1.4409  102.44  120.38  101.21  1.4438
IC O11  C10  *S11  O13       1.4409  102.44 -120.41  101.20  1.4432
DONO H2'  O2'
DONO H3   N3
DONO H81  N8
DONO H82  N8
ACCE O2   C2
ACCE O4   C4
ACCE O11  S11
ACCE O12  S11
ACCE O13  S11
ACCE O1P  P
ACCE O2P  P
ACCE O2'
ACCE O3'
ACCE O4'
ACCE O5'

RESI STU           -1.00 ! 5-taurinomethyl-2-thiouridine
GROUP
ATOM N1     NG2R61 -0.26 !       O12 (-)
ATOM C2     CG2R63  0.29 !        |
ATOM S2     SG2D1  -0.22 !  O13 =S11=O11
ATOM N3     NG2R61 -0.56 !        |
ATOM H3     HGP1    0.40 !  H101-C10-H102
ATOM C4     CG2R63  0.39 !        |
ATOM O4     OG2D4  -0.41 !        |   H81 H72  O4
ATOM C5     CG2R62 -0.03 !        |   |(+) |   ||
ATOM C6     CG2R62  0.18 !   H91-C9 -N8 -C7   C4    H3
ATOM H6     HGR62   0.22 !        |   |   / \ /  \  /
GROUP                    !      H92 H82 H71 C5   N3
ATOM C7     CG324   0.14 !                  ||   |
ATOM H71    HGA2    0.09 !               H6-C6   C2
ATOM H72    HGA2    0.09 !                   \  / \\
ATOM N8     NG3P2  -0.30 !                    N1   S2
ATOM H81    HGP2    0.33 !                     \
ATOM H82    HGP2    0.33 !                      \
ATOM C9     CG324   0.14 !                       \
ATOM H91    HGA2    0.09 !   O1P    H5' H4'  O4'  \
ATOM H92    HGA2    0.09 !    |      |    \ /   \  \
GROUP                    !   -P-O5'-C5'---C4'    C1'
ATOM C10    CG321  -0.26 !    |      |     \     / \
ATOM H101   HGA2    0.09 !   O2P    H5''   C3'--C2' H1'
ATOM H102   HGA2    0.09 !                 / \   / \
ATOM S11    SG3O1   0.73 !               O3' H3' O2' H2''
ATOM O11    OG2P1  -0.55 !                |       |
ATOM O12    OG2P1  -0.55 !                       H2'
ATOM O13    OG2P1  -0.55
GROUP
```

```
ATOM P       P        1.50
ATOM O1P     ON3     -0.78
ATOM O2P     ON3     -0.78
ATOM O5'     ON2     -0.57
ATOM C5'     CN8B    -0.08
ATOM H5'     HN8      0.09
ATOM H5''    HN8      0.09
GROUP
ATOM C4'     CN7      0.16
ATOM H4'     HN7      0.09
ATOM O4'     ON6B    -0.50
ATOM C1'     CN7B     0.16
ATOM H1'     HN7      0.09
GROUP
ATOM C2'     CN7B     0.14
ATOM H2''    HN7      0.09
ATOM O2'     ON5     -0.66
ATOM H2'     HN5      0.43
GROUP
ATOM C3'     CN7      0.01
ATOM H3'     HN7      0.09
ATOM O3'     ON2     -0.57
BOND N1   C2      N1   C6      C2   S2      C2   N3
BOND N3   C4      N3   H3      C4   O4      C4   C5
BOND C5   C6      C5   C7      C6   H6      C7   H71
BOND C7   H72     C7   N8      C9   C10     C9   H91
BOND C9   H92     C9   N8      C10  H101    C10  H102
BOND C10  S11     N8   H81     N8   H82     S11  O11
BOND S11  O12     S11  O13
BOND P    O1P     P    O2P     P    O5'     O5'  C5'     C5'  H5''
BOND C5'  C4'     C4'  O4'     C4'  C3'     O4'  C1'
BOND C1'  N1      C1'  C2'     C2'  C3'     C3'  O3'     O3'  +P
BOND C2'  O2'     O2'  H2'
BOND C1'  H1'     C2'  H2''    C3'  H3'     C4'  H4'     C5'  H5'
IMPR C2     N1     N3     S2     C4     C5     N3     O4
!ribose
IC -O3' P    O5'   C5'    1.6001  101.45  -39.25  119.00  1.4401
IC -O3' O5'  *P    O1P    1.6001  101.45 -115.82  109.74  1.4802
IC -O3' O5'  *P    O2P    1.6001  101.45  115.90  109.80  1.4801
IC  P   O5'  C5'   C4'    1.5996  119.00 -151.39  110.04  1.5160
IC O5'  C5'  C4'   C3'    1.4401  108.83 -179.85  116.10  1.5284
IC C5'  C4'  C3'   O3'    1.5160  116.10   76.70  115.12  1.4212
IC C4'  C3'  O3'   +P     1.5284  111.92  159.13  119.05  1.6001
IC C3'  O3'  +P    +O5'   1.4212  119.05  -98.86  101.45  1.5996
IC O4'  C3'  *C4'  C5'    1.4572  104.06 -120.04  116.10  1.5160
IC C2'  C4'  *C3'  O3'    1.5284  100.16 -124.08  115.12  1.4212
IC C4'  C3'  C2'   C1'    1.5284  100.16   39.58  102.04  1.5251
IC C3'  C2'  C1'   N1     1.5284  101.97  144.39  113.71  1.4896
IC O4'  C1'  N1    C2     1.5251  113.71  -96.0   117.06  1.3746
IC C1'  C3'  *C2'  O2'    1.5284  102.04 -114.67  110.81  1.4212
IC H2'  O2'  C2'   C3'    0.9600  114.97  148.63  111.92  1.5284
IC O4'  C2'  *C1'  H1'    0.0       0.0  -115.0     0.0   0.0
IC C1'  C3'  *C2'  H2''   0.0       0.0   115.0     0.0   0.0
IC C2'  C4'  *C3'  H3'    0.0       0.0   115.0     0.0   0.0
IC C3'  O4'  *C4'  H4'    0.0       0.0  -115.0     0.0   0.0
IC C4'  O5'  *C5'  H5'    0.0       0.0  -115.0     0.0   0.0
IC C4'  O5'  *C5'  H5''   0.0       0.0   115.0     0.0   0.0
IC C2   C6   *N1   C1'       1.3966  121.57  179.97  122.49  1.4896
IC C3'  C2'  C1'   N1     1.5284  101.97  144.39  113.71  1.4896
IC O4'  C1'  N1    C2     1.5251  113.71  -96.0   117.06  1.3746
IC C1'  C2   *N1   C6     1.3966  121.57  179.97  122.49  1.4896
IC C6   N1   C2    N3        1.3706  121.55    0.03  113.53  1.3884
IC N3   N1   *C2   S2        1.3884  113.53  179.99  125.73  1.6497
```

```
IC N1   C2   N3   C4        1.4161  113.53   -0.04  127.62  1.3802
IC C4   C2   *N3  H3        1.3802  127.62 -179.95  117.62  1.0086
IC C2   N3   C4   C5        1.3884  127.62    0.05  116.47  1.4391
IC C5   N3   *C4  O4        1.4391  116.47  179.95  118.45  1.2272
IC C5   N1   *C6  H6        1.3837  123.48 -179.98  114.16  1.0886
IC C6   C4   *C5  C7        1.3837  117.33 -179.94  111.66  1.4820
IC C4   C5   C7   N8        1.4391  111.66  179.98  111.16  1.4843
IC N8   C5   *C7  H71       1.4843  111.16  118.10  110.47  1.1070
IC H71  C5   *C7  H72       1.1070  110.47  123.76  110.54  1.1062
IC C5   C7   N8   C9        1.4820  111.16  179.97  114.96  1.5020
IC C9   C7   *N8  H81       1.5020  114.96  120.14  111.80  1.0087
IC C9   C7   *N8  H82       1.5020  114.96 -120.11  111.81  1.0089
IC C7   N8   C9   C10       1.4843  114.96 -179.98  104.84  1.5164
IC C10  N8   *C9  H91       1.5164  104.84  121.67  105.35  1.1056
IC C10  N8   *C9  H92       1.5164  104.84 -121.73  105.35  1.1059
IC N8   C9   C10  S11       1.5020  104.84 -179.97  103.43  1.7693
IC S11  C9   *C10 H101      1.7693  103.43  115.51  114.17  1.1077
IC H101 C9   *C10 H102      1.1077  114.17  128.88  114.20  1.1068
IC C9   C10  S11  O11       1.5164  103.43 -180.00  102.37  1.4401
IC O11  C10  *S11 O12       1.4401  102.37  120.37  101.10  1.4443
IC O11  C10  *S11 O13       1.4401  102.37 -120.43  101.16  1.4433
DONO H2'  O2'
DONO H3   N3
DONO H81  N8
DONO H82  N8
ACCE O4   C4
ACCE S2   C2
ACCE O11  S11
ACCE O12  S11
ACCE O13  S11
ACCE O1P  P
ACCE O2P  P
ACCE O2'
ACCE O3'
ACCE O4'
ACCE O5'

RESI GAU            0.00 ! geranylated 5-methylaminomethyl-2-thiouridine
GROUP
ATOM N1     NG2R61 -0.25 !     H93  H82 H72  O4
ATOM C2     CG2R64  0.41 !      |   |(+) |   ||
ATOM S2     SG311  -0.14 !  H92-C9 -N8 -C7   C4
ATOM N3     NG2R62 -0.56 !      |   |   / \ /  \
ATOM C4     CG2R63  0.53 !     H91 H81 H71 C5   N3          C23            C29
ATOM O4     OG2D4  -0.53 !                 ||   ||           |              |
ATOM C5     CG2R62 -0.04 !              H6-C6   C2   C20    C22     C25    C27
ATOM C6     CG2R62  0.24 !                   \  / \  /   \  //  \   /   \  //  \
ATOM H6     HGR62   0.14 !                   N1   S2     C21    C24     C26    C28
ATOM C20    CG321   0.02 !                    \
ATOM H201   HGA2    0.09 !                     \
ATOM H202   HGA2    0.09 !                      \
GROUP                    !  O1P    H5' H4'  O4'  \
ATOM C7     CG324   0.20 !   |      |    \ /   \  \
ATOM H71    HGA2    0.09 !  -P-O5'-C5'---C4'    C1'
ATOM H72    HGA2    0.09 !   |      |     \     / \
ATOM N8     NG3P2  -0.52 !  O2P    H5''   C3'--C2' H1'
ATOM H81    HGP2    0.38 !               / \   / \
ATOM H82    HGP2    0.38 !             O3' H3' O2' H2''
ATOM C9     CG334   0.11 !              |       |
ATOM H91    HGA3    0.09 !                      H2'
ATOM H92    HGA3    0.09
ATOM H93    HGA3    0.09
GROUP
```

```
ATOM C21    CG2D1  -0.15
ATOM H21    HGA4    0.15
ATOM C22    CG2D1  -0.00
GROUP
ATOM C23    CG331  -0.27
ATOM H231   HGA3    0.09
ATOM H232   HGA3    0.09
ATOM H233   HGA3    0.09
GROUP
ATOM C24    CG321  -0.18
ATOM H241   HGA2    0.09
ATOM H242   HGA2    0.09
GROUP
ATOM C25    CG321  -0.18
ATOM H251   HGA2    0.09
ATOM H252   HGA2    0.09
GROUP
ATOM C26    CG2D1  -0.15
ATOM H26    HGA4    0.15
ATOM C27    CG2D1  -0.00
GROUP
ATOM C28    CG331  -0.27
ATOM H281   HGA3    0.09
ATOM H282   HGA3    0.09
ATOM H283   HGA3    0.09
GROUP
ATOM C29    CG331  -0.27
ATOM H291   HGA3    0.09
ATOM H292   HGA3    0.09
ATOM H293   HGA3    0.09
GROUP
ATOM P      P       1.50
ATOM O1P    ON3    -0.78
ATOM O2P    ON3    -0.78
ATOM O5'    ON2    -0.57
ATOM C5'    CN8B   -0.08
ATOM H5'    HN8     0.09
ATOM H5''   HN8     0.09
GROUP
ATOM C4'    CN7     0.16
ATOM H4'    HN7     0.09
ATOM O4'    ON6B   -0.50
ATOM C1'    CN7B    0.16
ATOM H1'    HN7     0.09
GROUP
ATOM C2'    CN7B    0.14
ATOM H2''   HN7     0.09
ATOM O2'    ON5    -0.66
ATOM H2'    HN5     0.43
GROUP
ATOM C3'    CN7     0.01
ATOM H3'    HN7     0.09
ATOM O3'    ON2    -0.57
BOND N1   C2      N1   C6      C2   S2      C2   N3      S2   C20
BOND N3   C4      C4   O4      C4   C5      C5   C6      C5   C7
BOND C6   H6      C7   H71     C7   H72     C7   N8      N8   H81
BOND N8   H82     N8   C9      C9   H91     C9   H92     C9   H93
BOND C20  H201    C20  H202    C20  C21     C21  H21     C21  C22
BOND C22  C23     C22  C24     C23  H231    C23  H232    C23  H233
BOND C24  H241    C24  H242    C24  C25     C25  H251    C25  H252
BOND C25  C26     C26  H26     C26  C27     C27  C28     C27  C29
BOND C28  H281    C28  H282    C28  H283    C29  H291    C29  H292
BOND C29  H293
```

```
BOND P    O1P     P    O2P     P    O5'     O5'  C5'     C5'  H5''
BOND C5'  C4'     C4'  O4'     C4'  C3'     O4'  C1'
BOND C1'  N1      C1'  C2'     C2'  C3'     C3'  O3'     O3'  +P
BOND C2'  O2'     O2'  H2'
BOND C1'  H1'     C2'  H2''    C3'  H3'     C4'  H4'     C5'  H5'
IMPR C4     C5     N3     O4
!ribose
IC -O3' P    O5'  C5'    1.6001  101.45  -39.25  119.00  1.4401
IC -O3' O5'  *P   O1P    1.6001  101.45 -115.82  109.74  1.4802
IC -O3' O5'  *P   O2P    1.6001  101.45  115.90  109.80  1.4801
IC  P   O5'  C5'  C4'    1.5996  119.00 -151.39  110.04  1.5160
IC O5'  C5'  C4'  C3'    1.4401  108.83 -179.85  116.10  1.5284
IC C5'  C4'  C3'  O3'    1.5160  116.10   76.70  115.12  1.4212
IC C4'  C3'  O3'  +P     1.5284  111.92  159.13  119.05  1.6001
IC C3'  O3'  +P   +O5'   1.4212  119.05  -98.86  101.45  1.5996
IC O4'  C3'  *C4' C5'    1.4572  104.06 -120.04  116.10  1.5160
IC C2'  C4'  *C3' O3'    1.5284  100.16 -124.08  115.12  1.4212
IC C4'  C3'  C2'  C1'    1.5284  100.16   39.58  102.04  1.5251
IC C3'  C2'  C1'  N1     1.5284  101.97  144.39  113.71  1.4896
IC O4'  C1'  N1   C2     1.5251  113.71  -96.0   117.06  1.3746
IC C1'  C3'  *C2' O2'    1.5284  102.04 -114.67  110.81  1.4212
IC H2'  O2'  C2'  C3'    0.9600  114.97  148.63  111.92  1.5284
IC O4'  C2'  *C1' H1'    0.0       0.0  -115.0     0.0   0.0
IC C1'  C3'  *C2' H2''   0.0       0.0   115.0     0.0   0.0
IC C2'  C4'  *C3' H3'    0.0       0.0   115.0     0.0   0.0
IC C3'  O4'  *C4' H4'    0.0       0.0  -115.0     0.0   0.0
IC C4'  O5'  *C5' H5'    0.0       0.0  -115.0     0.0   0.0
IC C4'  O5'  *C5' H5''   0.0       0.0   115.0     0.0   0.0
IC C6   C2   *N1  C1'    1.3430  122.00  180.00  115.40  1.4560
IC C6   N1   C2   S2     1.3430  122.00  180.00  110.00  1.7680
IC S2   N1   *C2  N3     1.7680  110.00  180.00  122.20  1.3420
IC N1   C2   N3   C4     1.3920  122.20  180.00  119.10  1.3350
IC C2   N3   C4   C5     1.3420  119.10  180.00  117.80  1.4030
IC C5   N3   *C4  O4     1.4030  117.80  180.00  123.80  1.2340
IC C5   N1   *C6  H6     1.3500  122.90  180.00  115.00  1.0900
IC N1   C2   S2   C20    1.3920  110.00  180.00   97.00  1.8180
IC C2   S2   C20  C21    1.7680   97.00  180.00  109.80  1.5020
IC C21  S2   *C20 H201   1.5020  109.80  120.00  111.30  1.1110
IC H201 S2   *C20 H202   1.1110  111.30 -120.00  111.30  1.1110
IC C6   C4   *C5  C7     1.3500  116.70  180.00  117.50  1.4800
IC C4   C5   C7   N8     1.4030  117.50  180.00  107.00  1.4900
IC N8   C5   *C7  H71    1.4900  107.00  120.00  107.50  1.1000
IC H71  C5   *C7  H72    1.1000  107.50 -120.00  107.50  1.1000
IC C5   C7   N8   C9     1.4800  107.00  180.00  115.20  1.4900
IC C9   C7   *N8  H81    1.4900  115.20  120.00  110.80  1.0060
IC H81  C7   *N8  H82    1.0060  110.80 -120.00  110.80  1.0060
IC C7   N8   C9   H91    1.4900  115.20  180.00  103.90  1.1110
IC H91  N8   *C9  H92    1.1110  103.90  120.00  103.90  1.1110
IC H91  N8   *C9  H93    1.1110  103.90 -120.00  103.90  1.1110
IC S2   C20  C21  C22    1.8180  109.80  180.00  123.50  1.3400
IC C22  C20  *C21 H21    1.3400  123.50  180.00  116.00  1.1000
IC C20  C21  C22  C24    1.5020  123.50  180.00  123.50  1.5020
IC C24  C21  *C22 C23    1.5020  123.50  180.00  123.50  1.5040
IC C21  C22  C23  H231   1.3400  123.50  180.00  111.50  1.1110
IC H231 C22  *C23 H232   1.1110  111.50  120.00  111.50  1.1110
IC H231 C22  *C23 H233   1.1110  111.50 -120.00  111.50  1.1110
IC C21  C22  C24  C25    1.3400  123.50  180.00  112.20  1.5300
IC C25  C22  *C24 H241   1.5300  112.20  120.00  111.50  1.1110
IC H241 C22  *C24 H242   1.1110  111.50 -120.00  111.50  1.1110
IC C22  C24  C25  C26    1.5020  112.20  180.00  112.20  1.5020
IC C26  C24  *C25 H251   1.5020  112.20  120.00  110.10  1.1110
IC H251 C24  *C25 H252   1.1110  110.10 -120.00  110.10  1.1110
IC C24  C25  C26  C27    1.5300  112.20  180.00  123.50  1.3400
```

```
IC C27  C25  *C26  H26    1.3400  123.50  180.00  116.00  1.1000
IC C25  C26  C27   C28    1.5020  123.50  180.00  123.50  1.5040
IC C28  C26  *C27  C29    1.5040  123.50  180.00  123.50  1.5040
IC C26  C27  C28   H281   1.3400  123.50  180.00  111.50  1.1110
IC H281 C27  *C28  H282   1.1110  111.50  120.00  111.50  1.1110
IC H281 C27  *C28  H283   1.1110  111.50 -120.00  111.50  1.1110
IC C26  C27  C29   H291   1.3400  123.50  180.00  111.50  1.1110
IC H291 C27  *C29  H292   1.1110  111.50  120.00  111.50  1.1110
IC H291 C27  *C29  H293   1.1110  111.50 -120.00  111.50  1.1110
DONO H2'  O2'
DONO H81  N8
DONO H82  N8
ACCE N3
ACCE O4   C4
ACCE O1P  P
ACCE O2P  P
ACCE O2'
ACCE O3'
ACCE O4'
ACCE O5'

RESI GCU           -1.00 ! geranylated 5-carboxymethylaminomethyl-2-thiouridine
GROUP
ATOM N1     NG2R61 -0.25 !    O11  O12 (-)
ATOM C2     CG2R64  0.41 !      \\ /
ATOM S2     SG311  -0.14 !       C10
ATOM N3     NG2R62 -0.56 !        |   H82 H72  O4
ATOM C4     CG2R63  0.53 !        |   |(+) |   ||
ATOM O4     OG2D4  -0.53 !   H92-C9 -N8 -C7   C4
ATOM C5     CG2R62 -0.04 !        |   |   / \ /  \
ATOM C6     CG2R62  0.24 !      H91 H81 H71 C5   N3          C23            C29
ATOM H6     HGR62   0.14 !                  ||   ||           |              |
ATOM C20    CG321   0.02 !               H6-C6   C2   C20    C22     C25    C27
ATOM H201   HGA2    0.09 !                    \  / \  /   \  //  \   /   \  //  \
ATOM H202   HGA2    0.09 !                     N1   S2     C21    C24     C26    C28
GROUP                    !                      \
ATOM C7     CG324   0.04 !                       \
ATOM H71    HGA2    0.09 !                        \
ATOM H72    HGA2    0.09 !   O1P    H5' H4'  O4'  \
ATOM N8     NG3P2  -0.34 !    |      |    \ /   \  \
ATOM H81    HGP2    0.34 !   -P-O5'-C5'---C4'    C1'
ATOM H82    HGP2    0.34 !    |      |     \     / \
ATOM C9     CG324  -0.01 !   O2P    H5''   C3'--C2' H1'
ATOM H91    HGA2    0.09 !                 / \   / \
ATOM H92    HGA2    0.09 !              O3' H3' O2' H2''
ATOM C10    CG2O3   0.59 !               |       |
ATOM O11    OG2D2  -0.66 !                       H2'
ATOM O12    OG2D2  -0.66
GROUP                    ! !! PATCH 5UHG for the non-ionic tautomer
ATOM C21    CG2D1  -0.15
ATOM H21    HGA4    0.15
ATOM C22    CG2D1  -0.00
GROUP
ATOM C23    CG331  -0.27
ATOM H231   HGA3    0.09
ATOM H232   HGA3    0.09
ATOM H233   HGA3    0.09
GROUP
ATOM C24    CG321  -0.18
ATOM H241   HGA2    0.09
ATOM H242   HGA2    0.09
GROUP
ATOM C25    CG321  -0.18
```

```
ATOM H251   HGA2    0.09
ATOM H252   HGA2    0.09
GROUP
ATOM C26    CG2D1  -0.15
ATOM H26    HGA4    0.15
ATOM C27    CG2D1  -0.00
GROUP
ATOM C28    CG331  -0.27
ATOM H281   HGA3    0.09
ATOM H282   HGA3    0.09
ATOM H283   HGA3    0.09
GROUP
ATOM C29    CG331  -0.27
ATOM H291   HGA3    0.09
ATOM H292   HGA3    0.09
ATOM H293   HGA3    0.09
GROUP
ATOM P      P       1.50
ATOM O1P    ON3    -0.78
ATOM O2P    ON3    -0.78
ATOM O5'    ON2    -0.57
ATOM C5'    CN8B   -0.08
ATOM H5'    HN8     0.09
ATOM H5''   HN8     0.09
GROUP
ATOM C4'    CN7     0.16
ATOM H4'    HN7     0.09
ATOM O4'    ON6B   -0.50
ATOM C1'    CN7B    0.16
ATOM H1'    HN7     0.09
GROUP
ATOM C2'    CN7B    0.14
ATOM H2''   HN7     0.09
ATOM O2'    ON5    -0.66
ATOM H2'    HN5     0.43
GROUP
ATOM C3'    CN7     0.01
ATOM H3'    HN7     0.09
ATOM O3'    ON2    -0.57
BOND N1   C2      N1   C6      C2   S2      C2   N3      S2   C20
BOND N3   C4      C4   O4      C4   C5      C5   C6      C5   C7
BOND C6   H6      C7   H71     C7   H72     C7   N8      N8   H81
BOND N8   H82     N8   C9      C9   H91     C9   H92     C9   C10
BOND C10  O11     C10  O12
BOND C20  H201    C20  H202    C20  C21     C21  H21     C21  C22
BOND C22  C23     C22  C24     C23  H231    C23  H232    C23  H233
BOND C24  H241    C24  H242    C24  C25     C25  H251    C25  H252
BOND C25  C26     C26  H26     C26  C27     C27  C28     C27  C29
BOND C28  H281    C28  H282    C28  H283    C29  H291    C29  H292
BOND C29  H293
BOND P    O1P     P    O2P     P    O5'     O5'  C5'     C5'  H5''
BOND C5'  C4'     C4'  O4'     C4'  C3'     O4'  C1'
BOND C1'  N1      C1'  C2'     C2'  C3'     C3'  O3'     O3'  +P
BOND C2'  O2'     O2'  H2'
BOND C1'  H1'     C2'  H2''    C3'  H3'     C4'  H4'     C5'  H5'
IMPR C4      C5      N3      O4      C10     O11     O12     C9
!ribose
IC -O3' P    O5'   C5'    1.6001  101.45  -39.25  119.00  1.4401
IC -O3' O5'  *P    O1P    1.6001  101.45 -115.82  109.74  1.4802
IC -O3' O5'  *P    O2P    1.6001  101.45  115.90  109.80  1.4801
IC  P   O5'  C5'   C4'    1.5996  119.00 -151.39  110.04  1.5160
IC O5'  C5'  C4'   C3'    1.4401  108.83 -179.85  116.10  1.5284
IC C5'  C4'  C3'   O3'    1.5160  116.10   76.70  115.12  1.4212
```

```
IC C4'  C3'  O3'   +P      1.5284  111.92  159.13  119.05  1.6001
IC C3'  O3'  +P    +O5'    1.4212  119.05  -98.86  101.45  1.5996
IC O4'  C3'  *C4'  C5'     1.4572  104.06 -120.04  116.10  1.5160
IC C2'  C4'  *C3'  O3'     1.5284  100.16 -124.08  115.12  1.4212
IC C4'  C3'  C2'   C1'     1.5284  100.16   39.58  102.04  1.5251
IC C3'  C2'  C1'   N1      1.5284  101.97  144.39  113.71  1.4896
IC O4'  C1'  N1    C2      1.5251  113.71  -96.0   117.06  1.3746
IC C1'  C3'  *C2'  O2'     1.5284  102.04 -114.67  110.81  1.4212
IC H2'  O2'  C2'   C3'     0.9600  114.97  148.63  111.92  1.5284
IC O4'  C2'  *C1'  H1'     0.0       0.0  -115.0     0.0   0.0
IC C1'  C3'  *C2'  H2''    0.0       0.0   115.0     0.0   0.0
IC C2'  C4'  *C3'  H3'     0.0       0.0   115.0     0.0   0.0
IC C3'  O4'  *C4'  H4'     0.0       0.0  -115.0     0.0   0.0
IC C4'  O5'  *C5'  H5'     0.0       0.0  -115.0     0.0   0.0
IC C4'  O5'  *C5'  H5''    0.0       0.0   115.0     0.0   0.0
IC C6   C2   *N1   C1'     1.3430  122.00  180.00  115.40  1.4560
IC C6   N1   C2    S2      1.3430  122.00  180.00  110.00  1.7680
IC S2   N1   *C2   N3      1.7680  110.00  180.00  122.20  1.3420
IC N1   C2   N3    C4      1.3920  122.20  180.00  119.10  1.3350
IC C2   N3   C4    C5      1.3420  119.10  180.00  117.80  1.4030
IC C5   N3   *C4   O4      1.4030  117.80  180.00  123.80  1.2340
IC C5   N1   *C6   H6      1.3500  122.90  180.00  115.00  1.0900
IC N1   C2   S2    C20     1.3920  110.00  180.00   97.00  1.8180
IC C2   S2   C20   C21     1.7680   97.00  180.00  109.80  1.5020
IC C21  S2   *C20  H201    1.5020  109.80  120.00  111.30  1.1110
IC H201 S2   *C20  H202    1.1110  111.30 -120.00  111.30  1.1110
IC C6   C4   *C5   C7      1.3500  116.70  180.00  117.50  1.4800
IC C4   C5   C7    N8      1.4030  117.50  180.00  107.00  1.4900
IC N8   C5   *C7   H71     1.4900  107.00  120.00  107.50  1.1000
IC H71  C5   *C7   H72     1.1000  107.50 -120.00  107.50  1.1000
IC C5   C7   N8    C9      1.4800  107.00  180.00  115.20  1.4900
IC C9   C7   *N8   H81     1.4900  115.20  120.00  110.80  1.0060
IC H81  C7   *N8   H82     1.0060  110.80 -120.00  110.80  1.0060
IC C7   N8   C9    C10     1.4900  115.20  180.00  104.00  1.5220
IC C10  N8   *C9   H91     1.5220  104.00  120.00  102.30  1.1000
IC H91  N8   *C9   H92     1.1000  102.30 -120.00  102.30  1.1000
IC N8   C9   C10   O11     1.4900  104.00  180.00  116.00  1.2600
IC O11  C9   *C10  O12     1.2600  116.00  180.00  116.00  1.2600
IC S2   C20  C21   C22     1.8180  109.80  180.00  123.50  1.3400
IC C22  C20  *C21  H21     1.3400  123.50  180.00  116.00  1.1000
IC C20  C21  C22   C24     1.5020  123.50  180.00  123.50  1.5020
IC C24  C21  *C22  C23     1.5020  123.50  180.00  123.50  1.5040
IC C21  C22  C23   H231    1.3400  123.50  180.00  111.50  1.1110
IC H231 C22  *C23  H232    1.1110  111.50  120.00  111.50  1.1110
IC H231 C22  *C23  H233    1.1110  111.50 -120.00  111.50  1.1110
IC C21  C22  C24   C25     1.3400  123.50  180.00  112.20  1.5300
IC C25  C22  *C24  H241    1.5300  112.20  120.00  111.50  1.1110
IC H241 C22  *C24  H242    1.1110  111.50 -120.00  111.50  1.1110
IC C22  C24  C25   C26     1.5020  112.20  180.00  112.20  1.5020
IC C26  C24  *C25  H251    1.5020  112.20  120.00  110.10  1.1110
IC H251 C24  *C25  H252    1.1110  110.10 -120.00  110.10  1.1110
IC C24  C25  C26   C27     1.5300  112.20  180.00  123.50  1.3400
IC C27  C25  *C26  H26     1.3400  123.50  180.00  116.00  1.1000
IC C25  C26  C27   C28     1.5020  123.50  180.00  123.50  1.5040
IC C28  C26  *C27  C29     1.5040  123.50  180.00  123.50  1.5040
IC C26  C27  C28   H281    1.3400  123.50  180.00  111.50  1.1110
IC H281 C27  *C28  H282    1.1110  111.50  120.00  111.50  1.1110
IC H281 C27  *C28  H283    1.1110  111.50 -120.00  111.50  1.1110
IC C26  C27  C29   H291    1.3400  123.50  180.00  111.50  1.1110
IC H291 C27  *C29  H292    1.1110  111.50  120.00  111.50  1.1110
IC H291 C27  *C29  H293    1.1110  111.50 -120.00  111.50  1.1110
DONO H2'  O2'
DONO H81  N8
```

```
DONO H82  N8
ACCE O4   C4
ACCE N3
ACCE O11  C10
ACCE O12  C10
ACCE O1P  P
ACCE O2P  P
ACCE O2'
ACCE O3'
ACCE O4'
ACCE O5'

!!***Cytidines***
RESI OMC           -1.00 ! 2'-O-methylcytidine, MRC
GROUP
ATOM N1     NN2    -0.13 !                H42  H41
ATOM C2     CN1     0.52 !                  \  /
ATOM O2     ON1C   -0.49 !                   N4
ATOM N3     NN3    -0.66 !                   |
ATOM C4     CN2     0.65 !                   C4
ATOM N4     NN1    -0.75 !                  /  \\
ATOM H41    HN1     0.37 !              H5-C5   N3
ATOM H42    HN1     0.33 !                 ||   |
ATOM C5     CN3    -0.13 !              H6-C6   C2
ATOM H5     HN3     0.07 !                  \  / \\
ATOM C6     CN3     0.05 !                   N1   O2
ATOM H6     HN3     0.17 !                    \
GROUP                    !                     \
ATOM P      P       1.50 !                      \
ATOM O1P    ON3    -0.78 !  O1P    H5' H4'  O4'  \
ATOM O2P    ON3    -0.78 !   |       |    \ /   \  \
ATOM O5'    ON2    -0.57 !  -P-O5'-C5'---C4'    C1'
ATOM C5'    CN8B   -0.08 !   |       |     \     / \
ATOM H5'    HN8     0.09 !  O2P    H5''   C3'--C2' H1'
ATOM H5''   HN8     0.09 !               / \   / \
GROUP                    !             O3' H3' O2' H2''
ATOM C4'    CN7     0.16 !             |       |
ATOM H4'    HN7     0.09 !                     CM2
ATOM O4'    ON6B   -0.50 !                   /  |  \
ATOM C1'    CN7B    0.16 !                HM1 HM2 HM3
ATOM H1'    HN7     0.09
GROUP                    !  !!!! PATCH CYTP for the protonated base
ATOM C2'    CN7B    0.08
ATOM H2''   HN7     0.09
ATOM O2'    OG301  -0.34
ATOM CM2    CG331  -0.10
ATOM HM1    HGA3    0.09
ATOM HM2    HGA3    0.09
ATOM HM3    HGA3    0.09
GROUP
ATOM C3'    CN7     0.01
ATOM H3'    HN7     0.09
ATOM O3'    ON2    -0.57
BOND N1   C2      N1   C6      C2   O2      C2   N3
BOND N3   C4      C4   C5      C4   N4      N4   H41
BOND N4   H42     C5   H5      C5   C6      C6   H6
BOND P    O1P     P    O2P     P    O5'     O5'  C5'     C5'  H5''
BOND C5'  C4'     C4'  O4'     C4'  C3'     O4'  C1'
BOND C1'  N1      C1'  C2'     C2'  C3'     C3'  O3'     O3'  +P
BOND C2'  O2'     CM2  O2'     CM2  HM1     HM2  CM2     HM3  CM2
BOND C1'  H1'     C2'  H2''    C3'  H3'     C4'  H4'     C5'  H5'
IMPR C2   N1   N3   O2        C4   N3   C5   N4        N4   C4   H41  H42
!2OM-ribose
```

```
IC -O3' P    O5'   C5'    1.6001  101.45  -39.25  119.00  1.4401
IC -O3' O5'  *P    O1P    1.6001  101.45 -115.82  109.74  1.4802
IC -O3' O5'  *P    O2P    1.6001  101.45  115.90  109.80  1.4801
IC  P   O5'  C5'   C4'    1.5996  119.00 -151.39  110.04  1.5160
IC O5'  C5'  C4'   C3'    1.4401  108.83 -179.85  116.10  1.5284
IC C5'  C4'  C3'   O3'    1.5160  116.10   76.70  115.12  1.4212
IC C4'  C3'  O3'   +P     1.5284  111.92  159.13  119.05  1.6001
IC C3'  O3'  +P    +O5'   1.4212  119.05  -98.86  101.45  1.5996
IC O4'  C3'  *C4'  C5'    1.4572  104.06 -120.04  116.10  1.5160
IC C2'  C4'  *C3'  O3'    1.5284  100.16 -124.08  115.12  1.4212
IC C4'  C3'  C2'   C1'    1.5284  100.16   39.58  102.04  1.5251
IC C3'  C2'  C1'   N1     1.5284  101.97  144.39  113.71  1.4896
IC O4'  C1'  N1    C2     1.5251  113.71  -96.0   117.06  1.3746
IC C3'  C1'  *C2'  O2'    1.5312  102.03  117.61  107.13  1.4206
IC C1'  C2'  O2'   CM2    1.5393  107.13   90.00  107.00  1.4150
IC C2'  O2'  CM2   HM2    1.4206  107.00  180.00    0.0   0.0
IC HM2  O2'  *CM2  HM3    0.0       0.0   120.00    0.0   0.0
IC HM2  O2'  *CM2  HM1    0.0       0.0  -120.00    0.0   0.0
IC O4'  C2'  *C1'  H1'    0.0       0.0  -115.0     0.0   0.0
IC C1'  C3'  *C2'  H2''   0.0       0.0   115.0     0.0   0.0
IC C2'  C4'  *C3'  H3'    0.0       0.0   115.0     0.0   0.0
IC C3'  O4'  *C4'  H4'    0.0       0.0  -115.0     0.0   0.0
IC C4'  O5'  *C5'  H5'    0.0       0.0  -115.0     0.0   0.0
IC C4'  O5'  *C5'  H5''   0.0       0.0   115.0     0.0   0.0
IC C2   C6   *N1   C1'       1.3966  121.57  179.97  122.49  1.4896
IC C3'  C2'  C1'   N1     1.5284  101.97  144.39  113.71  1.4896
IC O4'  C1'  N1    C2     1.5251  113.71  -96.0   117.06  1.3746
IC C1'  C2   *N1   C6     1.3966  121.57  179.97  122.49  1.4896
IC C2   N1   C6    C5        1.4065  120.45   -0.04  121.12  1.3422
IC C5   N1   *C6   H6        1.3422  121.12 -179.91  115.46  1.0913
IC N1   C6   C5    C4        1.3658  121.12    0.07  117.63  1.4291
IC C4   C6   *C5   H5        1.4291  117.63  179.98  122.45  1.0874
IC C6   N1   C2    O2        1.3658  120.45 -179.93  119.24  1.2406
IC O2   N1   *C2   N3        1.2406  119.24  179.93  118.85  1.3573
IC N3   C5   *C4   N4        1.3378  121.63 -179.95  120.30  1.3319
IC C5   C4   N4    H41       1.4291  120.30 -179.98  115.57  0.9959
IC H41  C4   *N4   H42       0.9959  115.57  179.95  123.51  0.9931
DONO H41  N4
DONO H42  N4
ACCE O2   C2
ACCE N3
ACCE O1P  P
ACCE O2P  P
ACCE O2'
ACCE O3'
ACCE O4'
ACCE O5'

RESI 2SC           -1.00 ! 2-thiocytidine
GROUP
ATOM N1     NG2R61 -0.21 !             H42  H41
ATOM C2     CG2R63  0.31 !               \  /
ATOM S2     SG2D1  -0.18 !                N4
ATOM N3     NG2R62 -0.77 !                |
ATOM C4     CG2R64  0.48 !                C4
ATOM N4     NG2S3  -0.69 !               /  \\
ATOM H41    HGP4    0.36 !           H5-C5   N3
ATOM H42    HGP4    0.36 !              ||   |
ATOM C5     CG2R61 -0.13 !           H6-C6   C2
ATOM H5     HGR62   0.14 !               \  / \\
ATOM C6     CG2R61  0.11 !                N1   S2
ATOM H6     HGR62   0.22 !                 \
GROUP                    !                  \
```

```
ATOM P       P       1.50 !                     \
ATOM O1P     ON3    -0.78 ! O1P    H5' H4'  O4'  \
ATOM O2P     ON3    -0.78 !  |       |    \ /   \  \
ATOM O5'     ON2    -0.57 ! -P-O5'-C5'---C4'    C1'
ATOM C5'     CN8B   -0.08 !  |       |     \     / \
ATOM H5'     HN8     0.09 ! O2P    H5''   C3'--C2' H1'
ATOM H5''    HN8     0.09 !               / \   / \
GROUP                     !             O3' H3' O2' H2''
ATOM C4'     CN7     0.16 !              |       |
ATOM H4'     HN7     0.09 !                     H2'
ATOM O4'     ON6B   -0.50
ATOM C1'     CN7B    0.16
ATOM H1'     HN7     0.09
GROUP
ATOM C2'     CN7B    0.14
ATOM H2''    HN7     0.09
ATOM O2'     ON5    -0.66
ATOM H2'     HN5     0.43
GROUP
ATOM C3'     CN7     0.01
ATOM H3'     HN7     0.09
ATOM O3'     ON2    -0.57
BOND N1   C2   N1   C6   C2   S2   C2   N3
BOND N3   C4   C4   C5   C4   N4   N4   H41
BOND N4   H42  C5   H5   C5   C6   C6   H6
BOND P    O1P     P    O2P     P    O5'     O5'  C5'     C5'  H5''
BOND C5'  C4'     C4'  O4'     C4'  C3'     O4'  C1'
BOND C1'  N1      C1'  C2'     C2'  C3'     C3'  O3'     O3'  +P
BOND C2'  O2'     O2'  H2'
BOND C1'  H1'     C2'  H2''    C3'  H3'     C4'  H4'     C5'  H5'
IMPR C2   N1   N3   S2        C4   C5   N3   N4        N4   H41  H42  C4
!ribose
IC -O3' P    O5'   C5'    1.6001  101.45  -39.25  119.00  1.4401
IC -O3' O5'  *P    O1P    1.6001  101.45 -115.82  109.74  1.4802
IC -O3' O5'  *P    O2P    1.6001  101.45  115.90  109.80  1.4801
IC  P   O5'  C5'   C4'    1.5996  119.00 -151.39  110.04  1.5160
IC O5'  C5'  C4'   C3'    1.4401  108.83 -179.85  116.10  1.5284
IC C5'  C4'  C3'   O3'    1.5160  116.10   76.70  115.12  1.4212
IC C4'  C3'  O3'   +P     1.5284  111.92  159.13  119.05  1.6001
IC C3'  O3'  +P    +O5'   1.4212  119.05  -98.86  101.45  1.5996
IC O4'  C3'  *C4'  C5'    1.4572  104.06 -120.04  116.10  1.5160
IC C2'  C4'  *C3'  O3'    1.5284  100.16 -124.08  115.12  1.4212
IC C4'  C3'  C2'   C1'    1.5284  100.16   39.58  102.04  1.5251
IC C3'  C2'  C1'   N1     1.5284  101.97  144.39  113.71  1.4896
IC O4'  C1'  N1    C2     1.5251  113.71  -96.0   117.06  1.3746
IC C1'  C3'  *C2'  O2'    1.5284  102.04 -114.67  110.81  1.4212
IC H2'  O2'  C2'   C3'    0.9600  114.97  148.63  111.92  1.5284
IC O4'  C2'  *C1'  H1'    0.0       0.0  -115.0     0.0   0.0
IC C1'  C3'  *C2'  H2''   0.0       0.0   115.0     0.0   0.0
IC C2'  C4'  *C3'  H3'    0.0       0.0   115.0     0.0   0.0
IC C3'  O4'  *C4'  H4'    0.0       0.0  -115.0     0.0   0.0
IC C4'  O5'  *C5'  H5'    0.0       0.0  -115.0     0.0   0.0
IC C4'  O5'  *C5'  H5''   0.0       0.0   115.0     0.0   0.0
IC C2   C6   *N1   C1'       1.3966  121.57  179.97  122.49  1.4896
IC C3'  C2'  C1'   N1     1.5284  101.97  144.39  113.71  1.4896
IC O4'  C1'  N1    C2     1.5251  113.71  -96.0   117.06  1.3746
IC C1'  C2   *N1   C6     1.3966  121.57  179.97  122.49  1.4896
IC C6   N1   C2    N3     1.3735  122.75    0.00  113.44  1.3851
IC N3   N1   *C2   S2        1.3603  117.82  180.00  119.83  1.6441
IC N1   C2   N3    C4        1.4017  117.82   -0.03  120.81  1.3421
IC C2   N3   C4    N4        1.3603  120.81  180.00  116.63  1.3319
IC N4   N3   *C4   C5        1.3319  116.63 -179.94  123.67  1.3901
IC N3   C4   N4    H41       1.3421  116.63    0.01  113.56  0.9960
```

```
IC H41  C4   *N4   H42       0.9960  113.56 -179.98  124.68  0.9919
IC C6   C4   *C5   H5        1.4028  116.00 -179.98  121.68  1.0739
IC C5   N1   *C6   H6        1.4028  120.23 -180.00  117.77  1.0824
DONO H2'  O2'
DONO H41  N4
DONO H42  N4
ACCE N3
ACCE S2   C2
ACCE O1P  P
ACCE O2P  P
ACCE O2'
ACCE O3'
ACCE O4'
ACCE O5'

RESI 5MC            -1.00 ! 5-Methylcytidine
GROUP
ATOM N1      NG2R61 -0.12 !                 H42  H41
ATOM C2      CG2R63  0.50 !                   \  /
ATOM O2      OG2D4  -0.45 !                    N4
ATOM N3      NG2R62 -0.79 !                    |
ATOM C4      CG2R64  0.62 !          H51       C4
ATOM N4      NG2S3  -0.65 !             \     /  \\
ATOM H41     HGP4    0.32 !         H52--CM5-C5   N3
ATOM H42     HGP4    0.32 !             /    ||   |
ATOM C5      CG2R62  0.04 !          H53  H6-C6   C2
ATOM C6      CG2R62  0.02 !                   \  / \\
ATOM H6      HGR62   0.19 !                    N1   O2
ATOM CM5     CG331  -0.21 !                     \
ATOM H51     HGA3    0.07 !                      \
ATOM H52     HGA3    0.07 !                       \
ATOM H53     HGA3    0.07 !   O1P    H5' H4'  O4'  \
GROUP                     !    |      |    \ /   \  \
ATOM P       P       1.50 !   -P-O5'-C5'---C4'    C1'
ATOM O1P     ON3    -0.78 !    |      |     \     / \
ATOM O2P     ON3    -0.78 !   O2P    H5''   C3'--C2' H1'
ATOM O5'     ON2    -0.57 !                / \   / \
ATOM C5'     CN8B   -0.08 !              O3' H3' O2' H2''
ATOM H5'     HN8     0.09 !               |       |
ATOM H5''    HN8     0.09 !                       H2'
GROUP
ATOM C4'     CN7     0.16 !  !!!! PATCH 5MCP for the protonated base
ATOM H4'     HN7     0.09
ATOM O4'     ON6B   -0.50
ATOM C1'     CN7B    0.16
ATOM H1'     HN7     0.09
GROUP
ATOM C2'     CN7B    0.14
ATOM H2''    HN7     0.09
ATOM O2'     ON5    -0.66
ATOM H2'     HN5     0.43
GROUP
ATOM C3'     CN7     0.01
ATOM H3'     HN7     0.09
ATOM O3'     ON2    -0.57
BOND N1   C2      N1   C6      C2   O2      C2   N3
BOND N3   C4      C4   C5      C4   N4      N4   H41
BOND N4   H42     C5   C6      C5   CM5     CM5  H51
BOND CM5  H52     CM5  H53     C6   H6
BOND P    O1P     P    O2P     P    O5'     O5'  C5'     C5'  H5''
BOND C5'  C4'     C4'  O4'     C4'  C3'     O4'  C1'
BOND C1'  N1      C1'  C2'     C2'  C3'     C3'  O3'     O3'  +P
BOND C2'  O2'     O2'  H2'
```

```
BOND C1'  H1'      C2'  H2''     C3'  H3'      C4'  H4'      C5'  H5'
IMPR C2   N1   N3   O2         C4   C5   N3   N4         N4   H41  H42  C4
!ribose
IC -O3' P    O5'  C5'    1.6001  101.45  -39.25  119.00  1.4401
IC -O3' O5'  *P   O1P    1.6001  101.45 -115.82  109.74  1.4802
IC -O3' O5'  *P   O2P    1.6001  101.45  115.90  109.80  1.4801
IC  P   O5'  C5'  C4'    1.5996  119.00 -151.39  110.04  1.5160
IC O5'  C5'  C4'  C3'    1.4401  108.83 -179.85  116.10  1.5284
IC C5'  C4'  C3'  O3'    1.5160  116.10   76.70  115.12  1.4212
IC C4'  C3'  O3'  +P     1.5284  111.92  159.13  119.05  1.6001
IC C3'  O3'  +P   +O5'   1.4212  119.05  -98.86  101.45  1.5996
IC O4'  C3'  *C4' C5'    1.4572  104.06 -120.04  116.10  1.5160
IC C2'  C4'  *C3' O3'    1.5284  100.16 -124.08  115.12  1.4212
IC C4'  C3'  C2'  C1'    1.5284  100.16   39.58  102.04  1.5251
IC C3'  C2'  C1'  N1     1.5284  101.97  144.39  113.71  1.4896
IC O4'  C1'  N1   C2     1.5251  113.71  -96.0   117.06  1.3746
IC C1'  C3'  *C2' O2'    1.5284  102.04 -114.67  110.81  1.4212
IC H2'  O2'  C2'  C3'    0.9600  114.97  148.63  111.92  1.5284
IC O4'  C2'  *C1' H1'    0.0       0.0  -115.0     0.0   0.0
IC C1'  C3'  *C2' H2''   0.0       0.0   115.0     0.0   0.0
IC C2'  C4'  *C3' H3'    0.0       0.0   115.0     0.0   0.0
IC C3'  O4'  *C4' H4'    0.0       0.0  -115.0     0.0   0.0
IC C4'  O5'  *C5' H5'    0.0       0.0  -115.0     0.0   0.0
IC C4'  O5'  *C5' H5''   0.0       0.0   115.0     0.0   0.0
IC C2   C6   *N1  C1'       1.3966  121.57  179.97  122.49  1.4896
IC C3'  C2'  C1'  N1     1.5284  101.97  144.39  113.71  1.4896
IC O4'  C1'  N1   C2     1.5251  113.71  -96.0   117.06  1.3746
IC C1'  C2   *N1  C6     1.3966  121.57  179.97  122.49  1.4896
IC C6   N1   C2   N3        1.3605  121.64   -1.57  116.97  1.3774
IC N3   N1   *C2  O2        1.3774  116.97  179.95  118.63  1.2364
IC N1   C2   N3   C4        1.4201  116.97    0.93  120.30  1.3208
IC C2   N3   C4   C5        1.3774  120.30    0.02  124.49  1.4364
IC C5   N3   *C4  N4        1.4364  124.49 -175.99  116.51  1.3773
IC N3   C4   N4   H41       1.3208  116.51 -153.70  117.99  1.0128
IC H41  C4   *N4  H42       1.0128  117.99  138.48  113.70  1.0165
IC C5   N1   *C6  H6        1.3677  121.92  179.26  116.09  1.0879
IC C6   C4   *C5  CM5       1.3677  114.66 -179.01  122.62  1.5010
IC C4   C5   CM5  H51       1.4364  122.62  176.16  110.49  1.0940
IC H51  C5   *CM5 H52       1.0940  110.49  119.57  111.94  1.0979
IC H51  C5   *CM5 H53       1.0940  110.49 -119.73  111.10  1.0970
DONO H41  N4
DONO H42  N4
DONO H2'  O2'
ACCE O2   C2
ACCE N3
ACCE O1P  P
ACCE O2P  P
ACCE O2'
ACCE O3'
ACCE O4'
ACCE O5'

RESI MMC           -1.00 ! 5,2'-O-dimethylcytidine
GROUP
ATOM N1     NG2R61 -0.12 !                H42  H41
ATOM C2     CG2R63  0.50 !                  \  /
ATOM O2     OG2D4  -0.45 !                   N4
ATOM N3     NG2R62 -0.79 !                   |
ATOM C4     CG2R64  0.62 !         H51       C4
ATOM N4     NG2S3  -0.65 !            \     /  \\
ATOM H41    HGP4    0.32 !         H52-CM5-C5   N3
ATOM H42    HGP4    0.32 !            /    ||   |
ATOM C5     CG2R62  0.04 !         H53  H6-C6   C2
```

```
ATOM C6     CG2R62  0.02 !                    \  / \\
ATOM H6     HGR62   0.19 !                    N1   O2
ATOM CM5    CG331  -0.21 !                     \
ATOM H51    HGA3    0.07 !                      \
ATOM H52    HGA3    0.07 !                       \
ATOM H53    HGA3    0.07 !   O1P    H5' H4'  O4'  \
GROUP                    !    |      |    \ /   \  \
ATOM P      P       1.50 !   -P-O5'-C5'---C4'    C1'
ATOM O1P    ON3    -0.78 !    |      |     \     / \
ATOM O2P    ON3    -0.78 !   O2P    H5''   C3'--C2' H1'
ATOM O5'    ON2    -0.57 !                / \   / \
ATOM C5'    CN8B   -0.08 !              O3' H3' O2' H2''
ATOM H5'    HN8     0.09 !               |       |
ATOM H5''   HN8     0.09 !                     CM2
GROUP                    !                    /  |  \
ATOM C4'    CN7     0.16 !                 HM1 HM2 HM3
ATOM H4'    HN7     0.09
ATOM O4'    ON6B   -0.50 !  !!!! PATCH 5MCP for the protonated base
ATOM C1'    CN7B    0.16
ATOM H1'    HN7     0.09
GROUP
ATOM C2'    CN7B    0.08
ATOM H2''   HN7     0.09
ATOM O2'    OG301  -0.34
ATOM CM2    CG331  -0.10
ATOM HM1    HGA3    0.09
ATOM HM2    HGA3    0.09
ATOM HM3    HGA3    0.09
GROUP
ATOM C3'    CN7     0.01
ATOM H3'    HN7     0.09
ATOM O3'    ON2    -0.57
BOND N1   C2      N1   C6      C2   O2      C2   N3
BOND N3   C4      C4   C5      C4   N4      N4   H41
BOND N4   H42     C5   C6      C5   CM5     CM5  H51
BOND CM5  H52     CM5  H53     C6   H6
BOND P    O1P     P    O2P     P    O5'     O5'  C5'     C5'  H5''
BOND C5'  C4'     C4'  O4'     C4'  C3'     O4'  C1'
BOND C1'  N1      C1'  C2'     C2'  C3'     C3'  O3'     O3'  +P
BOND C2'  O2'     CM2  O2'     CM2  HM1     HM2  CM2     HM3  CM2
BOND C1'  H1'     C2'  H2''    C3'  H3'     C4'  H4'     C5'  H5'
IMPR C2   N1   N3   O2        C4   C5   N3   N4        N4   H41  H42  C4
!2OM-ribose
IC -O3' P    O5'   C5'    1.6001  101.45  -39.25  119.00  1.4401
IC -O3' O5'  *P    O1P    1.6001  101.45 -115.82  109.74  1.4802
IC -O3' O5'  *P    O2P    1.6001  101.45  115.90  109.80  1.4801
IC  P   O5'  C5'   C4'    1.5996  119.00 -151.39  110.04  1.5160
IC O5'  C5'  C4'   C3'    1.4401  108.83 -179.85  116.10  1.5284
IC C5'  C4'  C3'   O3'    1.5160  116.10   76.70  115.12  1.4212
IC C4'  C3'  O3'   +P     1.5284  111.92  159.13  119.05  1.6001
IC C3'  O3'  +P    +O5'   1.4212  119.05  -98.86  101.45  1.5996
IC O4'  C3'  *C4'  C5'    1.4572  104.06 -120.04  116.10  1.5160
IC C2'  C4'  *C3'  O3'    1.5284  100.16 -124.08  115.12  1.4212
IC C4'  C3'  C2'   C1'    1.5284  100.16   39.58  102.04  1.5251
IC C3'  C2'  C1'   N1     1.5284  101.97  144.39  113.71  1.4896
IC O4'  C1'  N1    C2     1.5251  113.71  -96.0   117.06  1.3746
IC C3'  C1'  *C2'  O2'    1.5312  102.03  117.61  107.13  1.4206
IC C1'  C2'  O2'   CM2    1.5393  107.13   90.00  107.00  1.4150
IC C2'  O2'  CM2   HM2    1.4206  107.00  180.00    0.0   0.0
IC HM2  O2'  *CM2  HM3    0.0       0.0   120.00    0.0   0.0
IC HM2  O2'  *CM2  HM1    0.0       0.0  -120.00    0.0   0.0
IC O4'  C2'  *C1'  H1'    0.0       0.0  -115.0     0.0   0.0
IC C1'  C3'  *C2'  H2''   0.0       0.0   115.0     0.0   0.0
```

```
IC C2'  C4'  *C3'  H3'    0.0       0.0   115.0     0.0   0.0
IC C3'  O4'  *C4'  H4'    0.0       0.0  -115.0     0.0   0.0
IC C4'  O5'  *C5'  H5'    0.0       0.0  -115.0     0.0   0.0
IC C4'  O5'  *C5'  H5''   0.0       0.0   115.0     0.0   0.0
IC C2   C6   *N1   C1'       1.3966  121.57  179.97  122.49  1.4896
IC C3'  C2'  C1'   N1     1.5284  101.97  144.39  113.71  1.4896
IC O4'  C1'  N1    C2     1.5251  113.71  -96.0   117.06  1.3746
IC C1'  C2   *N1   C6     1.3966  121.57  179.97  122.49  1.4896
IC C6   N1   C2    N3        1.3605  121.64   -1.57  116.97  1.3774
IC N3   N1   *C2   O2        1.3774  116.97  179.95  118.63  1.2364
IC N1   C2   N3    C4        1.4201  116.97    0.93  120.30  1.3208
IC C2   N3   C4    C5        1.3774  120.30    0.02  124.49  1.4364
IC C5   N3   *C4   N4        1.4364  124.49 -175.99  116.51  1.3773
IC N3   C4   N4    H41       1.3208  116.51 -153.70  117.99  1.0128
IC H41  C4   *N4   H42       1.0128  117.99  138.48  113.70  1.0165
IC C5   N1   *C6   H6        1.3677  121.92  179.26  116.09  1.0879
IC C6   C4   *C5   CM5       1.3677  114.66 -179.01  122.62  1.5010
IC C4   C5   CM5   H51       1.4364  122.62  176.16  110.49  1.0940
IC H51  C5   *CM5  H52       1.0940  110.49  119.57  111.94  1.0979
IC H51  C5   *CM5  H53       1.0940  110.49 -119.73  111.10  1.0970
DONO H41  N4
DONO H42  N4
ACCE O2   C2
ACCE N3
ACCE O1P  P
ACCE O2P  P
ACCE O2'
ACCE O3'
ACCE O4'
ACCE O5'

RESI HMC           -1.00 ! 5-hydroxymethylcytidine, 5-(hydroxymethyl)cytidine, 5HM
GROUP
ATOM N1     NG2R61 -0.12 !         H7O   H42  H41
ATOM C2     CG2R63  0.50 !           \      \  /
ATOM O2     OG2D4  -0.45 !            O7    N4
ATOM N3     NG2R62 -0.79 !             \     |
ATOM C4     CG2R64  0.62 !         H71-C7   C4
ATOM N4     NG2S3  -0.65 !             / \ /  \\
ATOM H41    HGP4    0.32 !           H72  C5   N3
ATOM H42    HGP4    0.32 !                ||   |
ATOM C5     CG2R62  0.04 !             H6-C6   C2
ATOM C6     CG2R62  0.02 !                 \  / \\
ATOM H6     HGR62   0.19 !                  N1   O2
GROUP                    !                   \
ATOM C7     CG321   0.05 !                    \
ATOM H71    HGA2    0.09 !                     \
ATOM H72    HGA2    0.09 ! O1P    H5' H4'  O4'  \
ATOM O7     OG311  -0.65 !  |       |    \ /   \  \
ATOM H7O    HGP1    0.42 ! -P-O5'-C5'---C4'    C1'
GROUP                    !  |       |     \     / \
ATOM P      P       1.50 ! O2P    H5''   C3'--C2' H1'
ATOM O1P    ON3    -0.78 !              / \   / \
ATOM O2P    ON3    -0.78 !            O3' H3' O2' H2''
ATOM O5'    ON2    -0.57 !             |       |
ATOM C5'    CN8B   -0.08 !                    H2'
ATOM H5'    HN8     0.09
ATOM H5''   HN8     0.09
GROUP
ATOM C4'    CN7     0.16
ATOM H4'    HN7     0.09
ATOM O4'    ON6B   -0.50
ATOM C1'    CN7B    0.16
```

```
ATOM H1'    HN7      0.09
GROUP
ATOM C2'    CN7B     0.14
ATOM H2''   HN7      0.09
ATOM O2'    ON5     -0.66
ATOM H2'    HN5      0.43
GROUP
ATOM C3'    CN7      0.01
ATOM H3'    HN7      0.09
ATOM O3'    ON2     -0.57
BOND N1   C2   N1   C6   C2   O2   C2   N3
BOND N3   C4   C4   C5   C4   N4   N4   H41
BOND N4   H42  C5   C7   C5   C6   C6   H6
BOND C7   H71  C7   H72  C7   O7   O7   H7O
BOND P    O1P     P    O2P     P    O5'     O5'  C5'     C5'  H5''
BOND C5'  C4'     C4'  O4'     C4'  C3'     O4'  C1'
BOND C1'  N1      C1'  C2'     C2'  C3'     C3'  O3'     O3'  +P
BOND C2'  O2'     O2'  H2'
BOND C1'  H1'     C2'  H2''    C3'  H3'     C4'  H4'     C5'  H5'
IMPR C2   N1   N3   O2        C4   C5   N3   N4        N4   H41  H42  C4
!ribose
IC -O3' P    O5'  C5'    1.6001  101.45  -39.25  119.00  1.4401
IC -O3' O5'  *P   O1P    1.6001  101.45 -115.82  109.74  1.4802
IC -O3' O5'  *P   O2P    1.6001  101.45  115.90  109.80  1.4801
IC  P   O5'  C5'  C4'    1.5996  119.00 -151.39  110.04  1.5160
IC O5'  C5'  C4'  C3'    1.4401  108.83 -179.85  116.10  1.5284
IC C5'  C4'  C3'  O3'    1.5160  116.10   76.70  115.12  1.4212
IC C4'  C3'  O3'  +P     1.5284  111.92  159.13  119.05  1.6001
IC C3'  O3'  +P   +O5'   1.4212  119.05  -98.86  101.45  1.5996
IC O4'  C3'  *C4' C5'    1.4572  104.06 -120.04  116.10  1.5160
IC C2'  C4'  *C3' O3'    1.5284  100.16 -124.08  115.12  1.4212
IC C4'  C3'  C2'  C1'    1.5284  100.16   39.58  102.04  1.5251
IC C3'  C2'  C1'  N1     1.5284  101.97  144.39  113.71  1.4896
IC O4'  C1'  N1   C2     1.5251  113.71  -96.0   117.06  1.3746
IC C1'  C3'  *C2' O2'    1.5284  102.04 -114.67  110.81  1.4212
IC H2'  O2'  C2'  C3'    0.9600  114.97  148.63  111.92  1.5284
IC O4'  C2'  *C1' H1'    0.0       0.0  -115.0     0.0   0.0
IC C1'  C3'  *C2' H2''   0.0       0.0   115.0     0.0   0.0
IC C2'  C4'  *C3' H3'    0.0       0.0   115.0     0.0   0.0
IC C3'  O4'  *C4' H4'    0.0       0.0  -115.0     0.0   0.0
IC C4'  O5'  *C5' H5'    0.0       0.0  -115.0     0.0   0.0
IC C4'  O5'  *C5' H5''   0.0       0.0   115.0     0.0   0.0
IC C2   C6   *N1  C1'       1.3966  121.57  179.97  122.49  1.4896
IC C3'  C2'  C1'  N1     1.5284  101.97  144.39  113.71  1.4896
IC O4'  C1'  N1   C2     1.5251  113.71  -96.0   117.06  1.3746
IC C1'  C2   *N1  C6     1.3966  121.57  179.97  122.49  1.4896
IC C6   N1   C2   N3        1.3564  121.41   -8.08  117.13  1.3768
IC N3   N1   *C2  O2        1.3768  117.13 -178.16  118.34  1.2350
IC N1   C2   N3   C4        1.4243  117.13    5.96  120.22  1.3216
IC C2   N3   C4   C5        1.3768  120.22    0.28  123.82  1.4381
IC C5   N3   *C4  N4        1.4381  123.82  177.24  117.83  1.3733
IC N3   C4   N4   H41       1.3216  117.83   12.03  113.47  1.0164
IC H41  C4   *N4  H42       1.0164  113.47  136.44  115.37  1.0161
IC C5   N1   *C6  H6        1.3675  121.52 -179.71  116.38  1.0887
IC C6   C4   *C5  C7        1.3675  115.38  178.82  122.27  1.4906
IC C4   C5   C7   O7        1.4381  122.27   60.58  108.13  1.4469
IC O7   C5   *C7  H71       1.4469  108.13  120.60  109.72  1.0981
IC H71  C5   *C7  H72       1.0981  109.72  119.75  110.31  1.0992
IC C5   C7   O7   H7O       1.4906  108.13 -177.68  108.45  0.9756
DONO H2'  O2'
DONO H41  N4
DONO H42  N4
DONO H7O  O7
```

```
ACCE O2   C2
ACCE N3
ACCE O7
ACCE O1P  P
ACCE O2P  P
ACCE O2'
ACCE O3'
ACCE O4'
ACCE O5'

RESI 5FC           -1.00 ! 5-formylcytidine, RSQ
GROUP
ATOM N1     NG2R61 -0.08 !              H42  H41
ATOM C2     CG2R63  0.62 !                \  /
ATOM O2     OG2D4  -0.47 !                 N4
ATOM N3     NG2R62 -0.83 !                 |
ATOM C4     CG2R64  0.61 !           O7    C4
ATOM N4     NG2S3  -0.75 !           ||   /  \\
ATOM H41    HGP4    0.37 !        H7-C7 -C5   N3
ATOM H42    HGP4    0.37 !               ||   |
ATOM C5     CG2R62  0.04 !            H6-C6   C2
ATOM C6     CG2R62  0.15 !                \  / \\
ATOM H6     HGR62   0.17 !                 N1   O2
ATOM C7     CG2O4   0.17 !                  \
ATOM H7     HGR52   0.08 !                   \
ATOM O7     OG2D1  -0.45 !                    \
GROUP                    ! O1P    H5' H4'  O4'  \
ATOM P      P       1.50 !  |       |    \ /   \  \
ATOM O1P    ON3    -0.78 ! -P-O5'-C5'---C4'    C1'
ATOM O2P    ON3    -0.78 !  |       |     \     / \
ATOM O5'    ON2    -0.57 ! O2P    H5''   C3'--C2' H1'
ATOM C5'    CN8B   -0.08 !              / \   / \
ATOM H5'    HN8     0.09 !           O3' H3' O2' H2''
ATOM H5''   HN8     0.09 !            |       |
GROUP                    !                   H2'
ATOM C4'    CN7     0.16
ATOM H4'    HN7     0.09
ATOM O4'    ON6B   -0.50
ATOM C1'    CN7B    0.16
ATOM H1'    HN7     0.09
GROUP
ATOM C2'    CN7B    0.14
ATOM H2''   HN7     0.09
ATOM O2'    ON5    -0.66
ATOM H2'    HN5     0.43
GROUP
ATOM C3'    CN7     0.01
ATOM H3'    HN7     0.09
ATOM O3'    ON2    -0.57
BOND N1   C2   N1   C6   C2   O2   C2   N3
BOND N3   C4   C4   C5   C4   N4   N4   H41
BOND N4   H42  C5   C7   C5   C6   C6   H6
BOND C7   H7   C7   O7
BOND P    O1P     P    O2P     P    O5'     O5'  C5'     C5'  H5''
BOND C5'  C4'     C4'  O4'     C4'  C3'     O4'  C1'
BOND C1'  N1      C1'  C2'     C2'  C3'     C3'  O3'     O3'  +P
BOND C2'  O2'     O2'  H2'
BOND C1'  H1'     C2'  H2''    C3'  H3'     C4'  H4'     C5'  H5'
IMPR C2   N1   N3   O2        C4   C5   N3   N4        N4   H41  H42  C4
IMPR C7   C5   O7   H7
!ribose
IC -O3' P    O5'   C5'    1.6001  101.45  -39.25  119.00  1.4401
IC -O3' O5'  *P    O1P    1.6001  101.45 -115.82  109.74  1.4802
```

```
IC -O3' O5'  *P    O2P    1.6001  101.45  115.90  109.80  1.4801
IC  P   O5'  C5'   C4'    1.5996  119.00 -151.39  110.04  1.5160
IC O5'  C5'  C4'   C3'    1.4401  108.83 -179.85  116.10  1.5284
IC C5'  C4'  C3'   O3'    1.5160  116.10   76.70  115.12  1.4212
IC C4'  C3'  O3'   +P     1.5284  111.92  159.13  119.05  1.6001
IC C3'  O3'  +P    +O5'   1.4212  119.05  -98.86  101.45  1.5996
IC O4'  C3'  *C4'  C5'    1.4572  104.06 -120.04  116.10  1.5160
IC C2'  C4'  *C3'  O3'    1.5284  100.16 -124.08  115.12  1.4212
IC C4'  C3'  C2'   C1'    1.5284  100.16   39.58  102.04  1.5251
IC C3'  C2'  C1'   N1     1.5284  101.97  144.39  113.71  1.4896
IC O4'  C1'  N1    C2     1.5251  113.71  -96.0   117.06  1.3746
IC C1'  C3'  *C2'  O2'    1.5284  102.04 -114.67  110.81  1.4212
IC H2'  O2'  C2'   C3'    0.9600  114.97  148.63  111.92  1.5284
IC O4'  C2'  *C1'  H1'    0.0       0.0  -115.0     0.0   0.0
IC C1'  C3'  *C2'  H2''   0.0       0.0   115.0     0.0   0.0
IC C2'  C4'  *C3'  H3'    0.0       0.0   115.0     0.0   0.0
IC C3'  O4'  *C4'  H4'    0.0       0.0  -115.0     0.0   0.0
IC C4'  O5'  *C5'  H5'    0.0       0.0  -115.0     0.0   0.0
IC C4'  O5'  *C5'  H5''   0.0       0.0   115.0     0.0   0.0
IC C2   C6   *N1   C1'       1.3966  121.57  179.97  122.49  1.4896
IC C3'  C2'  C1'   N1     1.5284  101.97  144.39  113.71  1.4896
IC O4'  C1'  N1    C2     1.5251  113.71  -96.0   117.06  1.3746
IC C1'  C2   *N1   C6     1.3966  121.57  179.97  122.49  1.4896
IC C6   N1   C2    N3        1.3395  121.06    0.00  117.50  1.3670
IC N3   N1   *C2   O2        1.3670  117.50  180.00  117.03  1.2302
IC N1   C2   N3    C4        1.4487  117.50    0.00  120.86  1.3260
IC C2   N3   C4    C5        1.3670  120.86    0.00  122.90  1.4499
IC C5   N3   *C4   N4        1.4499  122.90  180.00  118.02  1.3460
IC N3   C4   N4    H41       1.3260  118.02    0.00  118.10  1.0132
IC H41  C4   *N4   H42       1.0132  118.10  180.00  120.17  1.0159
IC C5   N1   *C6   H6        1.3767  121.89  180.00  116.83  1.0901
IC C6   C4   *C5   C7        1.3767  115.78  180.00  125.23  1.4529
IC C4   C5   C7    O7        1.4499  125.23    0.00  125.29  1.2398
IC O7   C5   *C7   H7        1.2398  125.29  180.00  115.22  1.1083
DONO H2'  O2'
DONO H41  N4
DONO H42  N4
ACCE O2   C2
ACCE N3
ACCE O7   C7
ACCE O1P  P
ACCE O2P  P
ACCE O2'
ACCE O3'
ACCE O4'
ACCE O5'

RESI MFC           -1.00 ! 5-formyl-2'-O-methylcytidine
GROUP
ATOM N1     NG2R61 -0.08 !               H42  H41
ATOM C2     CG2R63  0.62 !                 \  /
ATOM O2     OG2D4  -0.47 !                  N4
ATOM N3     NG2R62 -0.83 !                  |
ATOM C4     CG2R64  0.61 !            O7    C4
ATOM N4     NG2S3  -0.75 !            ||   /  \\
ATOM H41    HGP4    0.37 !        H7 -C7 -C5   N3
ATOM H42    HGP4    0.37 !                ||   |
ATOM C5     CG2R62  0.04 !             H6-C6   C2
ATOM C6     CG2R62  0.15 !                 \  / \\
ATOM H6     HGR62   0.17 !                  N1   O2
ATOM C7     CG2O4   0.17 !                   \
ATOM H7     HGR52   0.08 !                    \
ATOM O7     OG2D1  -0.45 !                     \
```

```
GROUP                    !  O1P    H5' H4'  O4'  \
ATOM P      P       1.50 !   |      |    \ /   \  \
ATOM O1P    ON3    -0.78 !  -P-O5'-C5'---C4'    C1'
ATOM O2P    ON3    -0.78 !   |      |     \     / \
ATOM O5'    ON2    -0.57 !  O2P    H5''   C3'--C2' H1'
ATOM C5'    CN8B   -0.08 !               / \   / \
ATOM H5'    HN8     0.09 !             O3' H3' O2' H2''
ATOM H5''   HN8     0.09 !              |       |
GROUP                    !                     CM2
ATOM C4'    CN7     0.16 !                    /  |  \
ATOM H4'    HN7     0.09 !                  HM1 HM2 HM3
ATOM O4'    ON6B   -0.50
ATOM C1'    CN7B    0.16
ATOM H1'    HN7     0.09
GROUP
ATOM C2'    CN7B    0.08
ATOM H2''   HN7     0.09
ATOM O2'    OG301  -0.34
ATOM CM2    CG331  -0.10
ATOM HM1    HGA3    0.09
ATOM HM2    HGA3    0.09
ATOM HM3    HGA3    0.09
GROUP
ATOM C3'    CN7     0.01
ATOM H3'    HN7     0.09
ATOM O3'    ON2    -0.57
BOND N1   C2   N1   C6   C2   O2   C2   N3
BOND N3   C4   C4   C5   C4   N4   N4   H41
BOND N4   H42  C5   C7   C5   C6   C6   H6
BOND C7   H7   C7   O7
BOND P    O1P     P    O2P     P    O5'     O5'  C5'     C5'  H5''
BOND C5'  C4'     C4'  O4'     C4'  C3'     O4'  C1'
BOND C1'  N1      C1'  C2'     C2'  C3'     C3'  O3'     O3'  +P
BOND C2'  O2'     CM2  O2'     CM2  HM1     HM2  CM2     HM3  CM2
BOND C1'  H1'     C2'  H2''    C3'  H3'     C4'  H4'     C5'  H5'
IMPR C2   N1   N3   O2        C4   C5   N3   N4        N4   H41  H42  C4
IMPR C7   C5   O7   H7
!2OM-ribose
IC -O3' P    O5'  C5'    1.6001  101.45  -39.25  119.00  1.4401
IC -O3' O5'  *P   O1P    1.6001  101.45 -115.82  109.74  1.4802
IC -O3' O5'  *P   O2P    1.6001  101.45  115.90  109.80  1.4801
IC  P   O5'  C5'  C4'    1.5996  119.00 -151.39  110.04  1.5160
IC O5'  C5'  C4'  C3'    1.4401  108.83 -179.85  116.10  1.5284
IC C5'  C4'  C3'  O3'    1.5160  116.10   76.70  115.12  1.4212
IC C4'  C3'  O3'  +P     1.5284  111.92  159.13  119.05  1.6001
IC C3'  O3'  +P   +O5'   1.4212  119.05  -98.86  101.45  1.5996
IC O4'  C3'  *C4' C5'    1.4572  104.06 -120.04  116.10  1.5160
IC C2'  C4'  *C3' O3'    1.5284  100.16 -124.08  115.12  1.4212
IC C4'  C3'  C2'  C1'    1.5284  100.16   39.58  102.04  1.5251
IC C3'  C2'  C1'  N1     1.5284  101.97  144.39  113.71  1.4896
IC O4'  C1'  N1   C2     1.5251  113.71  -96.0   117.06  1.3746
IC C3'  C1'  *C2' O2'    1.5312  102.03  117.61  107.13  1.4206
IC C1'  C2'  O2'  CM2    1.5393  107.13   90.00  107.00  1.4150
IC C2'  O2'  CM2  HM2    1.4206  107.00  180.00    0.0   0.0
IC HM2  O2'  *CM2 HM3    0.0       0.0   120.00    0.0   0.0
IC HM2  O2'  *CM2 HM1    0.0       0.0  -120.00    0.0   0.0
IC O4'  C2'  *C1' H1'    0.0       0.0  -115.0     0.0   0.0
IC C1'  C3'  *C2' H2''   0.0       0.0   115.0     0.0   0.0
IC C2'  C4'  *C3' H3'    0.0       0.0   115.0     0.0   0.0
IC C3'  O4'  *C4' H4'    0.0       0.0  -115.0     0.0   0.0
IC C4'  O5'  *C5' H5'    0.0       0.0  -115.0     0.0   0.0
IC C4'  O5'  *C5' H5''   0.0       0.0   115.0     0.0   0.0
IC C2   C6   *N1  C1'       1.3966  121.57  179.97  122.49  1.4896
```

```
IC C3'  C2'  C1'   N1      1.5284  101.97  144.39  113.71  1.4896
IC O4'  C1'  N1    C2      1.5251  113.71  -96.0   117.06  1.3746
IC C1'  C2   *N1   C6      1.3966  121.57  179.97  122.49  1.4896
IC C6   N1   C2    N3        1.3395  121.06    0.00  117.50  1.3670
IC N3   N1   *C2   O2        1.3670  117.50  180.00  117.03  1.2302
IC N1   C2   N3    C4        1.4487  117.50    0.00  120.86  1.3260
IC C2   N3   C4    C5        1.3670  120.86    0.00  122.90  1.4499
IC C5   N3   *C4   N4        1.4499  122.90  180.00  118.02  1.3460
IC N3   C4   N4    H41       1.3260  118.02    0.00  118.10  1.0132
IC H41  C4   *N4   H42       1.0132  118.10  180.00  120.17  1.0159
IC C5   N1   *C6   H6        1.3767  121.89  180.00  116.83  1.0901
IC C6   C4   *C5   C7        1.3767  115.78  180.00  125.23  1.4529
IC C4   C5   C7    O7        1.4499  125.23    0.00  125.29  1.2398
IC O7   C5   *C7   H7        1.2398  125.29  180.00  115.22  1.1083
DONO H41  N4
DONO H42  N4
ACCE O2   C2
ACCE N3
ACCE O7   C7
ACCE O1P  P
ACCE O2P  P
ACCE O2'
ACCE O3'
ACCE O4'
ACCE O5'

RESI 4MC           -1.00 ! N4-methylcytidine
GROUP
ATOM N1     NG2R61 -0.12 !            H41 H42 H43
ATOM C2     CG2R63  0.52 !               \ | /
ATOM O2     OG2D4  -0.49 !                CM4   H4
ATOM N3     NG2R62 -0.67 !                  \  /
ATOM C4     CG2R64  0.64 !                   N4
ATOM N4     NG311  -0.52 !                   |
ATOM H4     HGPAM1  0.32 !                   C4
ATOM C5     CG2R62 -0.05 !                  /  \\
ATOM H5     HGR62   0.02 !              H5-C5   N3
ATOM C6     CG2R62  0.06 !                 ||   |
ATOM H6     HGR62   0.16 !              H6-C6   C2
ATOM CM4    CG331  -0.14 !                  \  / \\
ATOM H41    HGA3    0.09 !                   N1   O2
ATOM H42    HGA3    0.09 !                    \
ATOM H43    HGA3    0.09 !                     \
GROUP                    !                      \
ATOM P      P       1.50 !  O1P    H5' H4'  O4'  \
ATOM O1P    ON3    -0.78 !   |      |    \ /   \  \
ATOM O2P    ON3    -0.78 !  -P-O5'-C5'---C4'    C1'
ATOM O5'    ON2    -0.57 !   |      |     \     / \
ATOM C5'    CN8B   -0.08 !  O2P    H5''   C3'--C2' H1'
ATOM H5'    HN8     0.09 !               / \   / \
ATOM H5''   HN8     0.09 !             O3' H3' O2' H2''
GROUP                    !              |       |
ATOM C4'    CN7     0.16 !                     H2'
ATOM H4'    HN7     0.09
ATOM O4'    ON6B   -0.50
ATOM C1'    CN7B    0.16
ATOM H1'    HN7     0.09
GROUP
ATOM C2'    CN7B    0.14
ATOM H2''   HN7     0.09
ATOM O2'    ON5    -0.66
ATOM H2'    HN5     0.43
GROUP
```

```
ATOM C3'    CN7     0.01
ATOM H3'    HN7     0.09
ATOM O3'    ON2    -0.57
BOND N1   C2      N1   C6      C2   O2      C2   N3
BOND N3   C4      C4   C5      C4   N4      N4   H4
BOND N4   CM4     CM4  H41     CM4  H42     CM4  H43
BOND C5   H5      C5   C6      C6   H6
BOND P    O1P     P    O2P     P    O5'     O5'  C5'     C5'  H5''
BOND C5'  C4'     C4'  O4'     C4'  C3'     O4'  C1'
BOND C1'  N1      C1'  C2'     C2'  C3'     C3'  O3'     O3'  +P
BOND C2'  O2'     O2'  H2'
BOND C1'  H1'     C2'  H2''    C3'  H3'     C4'  H4'     C5'  H5'
IMPR C2   N1   N3   O2        C4   C5   N3   N4       ! N4   C4   CM4   H4
!ribose
IC -O3' P    O5'   C5'    1.6001  101.45  -39.25  119.00  1.4401
IC -O3' O5'  *P    O1P    1.6001  101.45 -115.82  109.74  1.4802
IC -O3' O5'  *P    O2P    1.6001  101.45  115.90  109.80  1.4801
IC  P   O5'  C5'   C4'    1.5996  119.00 -151.39  110.04  1.5160
IC O5'  C5'  C4'   C3'    1.4401  108.83 -179.85  116.10  1.5284
IC C5'  C4'  C3'   O3'    1.5160  116.10   76.70  115.12  1.4212
IC C4'  C3'  O3'   +P     1.5284  111.92  159.13  119.05  1.6001
IC C3'  O3'  +P    +O5'   1.4212  119.05  -98.86  101.45  1.5996
IC O4'  C3'  *C4'  C5'    1.4572  104.06 -120.04  116.10  1.5160
IC C2'  C4'  *C3'  O3'    1.5284  100.16 -124.08  115.12  1.4212
IC C4'  C3'  C2'   C1'    1.5284  100.16   39.58  102.04  1.5251
IC C3'  C2'  C1'   N1     1.5284  101.97  144.39  113.71  1.4896
IC O4'  C1'  N1    C2     1.5251  113.71  -96.0   117.06  1.3746
IC C1'  C3'  *C2'  O2'    1.5284  102.04 -114.67  110.81  1.4212
IC H2'  O2'  C2'   C3'    0.9600  114.97  148.63  111.92  1.5284
IC O4'  C2'  *C1'  H1'    0.0       0.0  -115.0     0.0   0.0
IC C1'  C3'  *C2'  H2''   0.0       0.0   115.0     0.0   0.0
IC C2'  C4'  *C3'  H3'    0.0       0.0   115.0     0.0   0.0
IC C3'  O4'  *C4'  H4'    0.0       0.0  -115.0     0.0   0.0
IC C4'  O5'  *C5'  H5'    0.0       0.0  -115.0     0.0   0.0
IC C4'  O5'  *C5'  H5''   0.0       0.0   115.0     0.0   0.0
IC C2   C6   *N1   C1'       1.3966  121.57  179.97  122.49  1.4896
IC C3'  C2'  C1'   N1     1.5284  101.97  144.39  113.71  1.4896
IC O4'  C1'  N1    C2     1.5251  113.71  -96.0   117.06  1.3746
IC C1'  C2   *N1   C6     1.3966  121.57  179.97  122.49  1.4896
IC C6   N1   C2    N3        1.3556  123.94    0.08  115.87  1.3763
IC N3   N1   *C2   O2        1.3763  115.87  179.76  118.56  1.2272
IC N1   C2   N3    C4        1.4208  115.87   -0.41  120.17  1.3253
IC C2   N3   C4    N4        1.3763  120.17 -178.16  115.73  1.3614
IC N4   N3   *C4   C5        1.3614  115.73  178.63  124.28  1.4372
IC N3   C4   N4    CM4       1.3253  115.73 -167.94  124.86  1.4481
IC CM4  C4   *N4   H4        1.4481  124.86  160.11  113.05  1.0138
IC C6   C4   *C5   H5        1.3606  115.88  179.98  123.17  1.0818
IC C5   N1   *C6   H6        1.3606  119.86  179.89  116.87  1.0858
IC C4   N4   CM4   H41       1.3614  124.86  177.76  108.04  1.0911
IC H41  N4   *CM4  H42       1.0911  108.04  117.87  110.97  1.0940
IC H41  N4   *CM4  H43       1.0911  108.04 -119.76  112.67  1.0970
DONO H2'  O2'
DONO H4   N4
ACCE O2   C2
ACCE N3
ACCE O1P  P
ACCE O2P  P
ACCE O2'
ACCE O3'
ACCE O4'
ACCE O5'

RESI 4OC          -1.00 ! N4,2'-O-dimethylcytidine, M4C
```

```
GROUP
ATOM N1     NG2R61 -0.12 !            H41 H42 H43
ATOM C2     CG2R63  0.52 !               \ | /
ATOM O2     OG2D4  -0.49 !                CM4   H4
ATOM N3     NG2R62 -0.67 !                  \  /
ATOM C4     CG2R64  0.64 !                   N4
ATOM N4     NG311  -0.52 !                   |
ATOM H4     HGPAM1  0.32 !                   C4
ATOM C5     CG2R62 -0.05 !                  /  \\
ATOM H5     HGR62   0.02 !             H5-C5   N3
ATOM C6     CG2R62  0.06 !                 ||   |
ATOM H6     HGR62   0.16 !             H6-C6   C2
ATOM CM4    CG331  -0.14 !                  \  / \\
ATOM H41    HGA3    0.09 !                   N1   O2
ATOM H42    HGA3    0.09 !                    \
ATOM H43    HGA3    0.09 !                     \
GROUP                    !                      \
ATOM P      P       1.50 !  O1P    H5' H4'  O4'  \
ATOM O1P    ON3    -0.78 !   |      |    \ /   \  \
ATOM O2P    ON3    -0.78 !  -P-O5'-C5'---C4'    C1'
ATOM O5'    ON2    -0.57 !   |      |     \     / \
ATOM C5'    CN8B   -0.08 !  O2P    H5''   C3'--C2' H1'
ATOM H5'    HN8     0.09 !               / \   / \
ATOM H5''   HN8     0.09 !             O3' H3' O2' H2''
GROUP                    !              |       |
ATOM C4'    CN7     0.16 !                     CM2
ATOM H4'    HN7     0.09 !                    /  |  \
ATOM O4'    ON6B   -0.50 !                HM1 HM2 HM3
ATOM C1'    CN7B    0.16
ATOM H1'    HN7     0.09
GROUP
ATOM C2'    CN7B    0.08
ATOM H2''   HN7     0.09
ATOM O2'    OG301  -0.34
ATOM CM2    CG331  -0.10
ATOM HM1    HGA3    0.09
ATOM HM2    HGA3    0.09
ATOM HM3    HGA3    0.09
GROUP
ATOM C3'    CN7     0.01
ATOM H3'    HN7     0.09
ATOM O3'    ON2    -0.57
BOND N1   C2      N1   C6      C2   O2      C2   N3
BOND N3   C4      C4   C5      C4   N4      N4   H4
BOND N4   CM4     CM4  H41     CM4  H42     CM4  H43
BOND C5   H5      C5   C6      C6   H6
BOND P    O1P     P    O2P     P    O5'     O5'  C5'     C5'  H5''
BOND C5'  C4'     C4'  O4'     C4'  C3'     O4'  C1'
BOND C1'  N1      C1'  C2'     C2'  C3'     C3'  O3'     O3'  +P
BOND C2'  O2'     CM2  O2'     CM2  HM1     HM2  CM2     HM3  CM2
BOND C1'  H1'     C2'  H2''    C3'  H3'     C4'  H4'     C5'  H5'
IMPR C2   N1   N3   O2        C4   C5   N3   N4       ! N4   C4   CM4   H4
!2OM-ribose
IC -O3' P    O5'   C5'    1.6001  101.45  -39.25  119.00  1.4401
IC -O3' O5'  *P    O1P    1.6001  101.45 -115.82  109.74  1.4802
IC -O3' O5'  *P    O2P    1.6001  101.45  115.90  109.80  1.4801
IC  P   O5'  C5'   C4'    1.5996  119.00 -151.39  110.04  1.5160
IC O5'  C5'  C4'   C3'    1.4401  108.83 -179.85  116.10  1.5284
IC C5'  C4'  C3'   O3'    1.5160  116.10   76.70  115.12  1.4212
IC C4'  C3'  O3'   +P     1.5284  111.92  159.13  119.05  1.6001
IC C3'  O3'  +P    +O5'   1.4212  119.05  -98.86  101.45  1.5996
IC O4'  C3'  *C4'  C5'    1.4572  104.06 -120.04  116.10  1.5160
IC C2'  C4'  *C3'  O3'    1.5284  100.16 -124.08  115.12  1.4212
```

```
IC C4'  C3'  C2'   C1'    1.5284  100.16   39.58  102.04  1.5251
IC C3'  C2'  C1'   N1     1.5284  101.97  144.39  113.71  1.4896
IC O4'  C1'  N1    C2     1.5251  113.71  -96.0   117.06  1.3746
IC C3'  C1'  *C2'  O2'    1.5312  102.03  117.61  107.13  1.4206
IC C1'  C2'  O2'   CM2    1.5393  107.13   90.00  107.00  1.4150
IC C2'  O2'  CM2   HM2    1.4206  107.00  180.00    0.0   0.0
IC HM2  O2'  *CM2  HM3    0.0       0.0   120.00    0.0   0.0
IC HM2  O2'  *CM2  HM1    0.0       0.0  -120.00    0.0   0.0
IC O4'  C2'  *C1'  H1'    0.0       0.0  -115.0     0.0   0.0
IC C1'  C3'  *C2'  H2''   0.0       0.0   115.0     0.0   0.0
IC C2'  C4'  *C3'  H3'    0.0       0.0   115.0     0.0   0.0
IC C3'  O4'  *C4'  H4'    0.0       0.0  -115.0     0.0   0.0
IC C4'  O5'  *C5'  H5'    0.0       0.0  -115.0     0.0   0.0
IC C4'  O5'  *C5'  H5''   0.0       0.0   115.0     0.0   0.0
IC C2   C6   *N1   C1'       1.3966  121.57  179.97  122.49  1.4896
IC C3'  C2'  C1'   N1     1.5284  101.97  144.39  113.71  1.4896
IC O4'  C1'  N1    C2     1.5251  113.71  -96.0   117.06  1.3746
IC C1'  C2   *N1   C6     1.3966  121.57  179.97  122.49  1.4896
IC C6   N1   C2    N3        1.3556  123.94    0.08  115.87  1.3763
IC N3   N1   *C2   O2        1.3763  115.87  179.76  118.56  1.2272
IC N1   C2   N3    C4        1.4208  115.87   -0.41  120.17  1.3253
IC C2   N3   C4    N4        1.3763  120.17 -178.16  115.73  1.3614
IC N4   N3   *C4   C5        1.3614  115.73  178.63  124.28  1.4372
IC N3   C4   N4    CM4       1.3253  115.73 -167.94  124.86  1.4481
IC CM4  C4   *N4   H4        1.4481  124.86  160.11  113.05  1.0138
IC C6   C4   *C5   H5        1.3606  115.88  179.98  123.17  1.0818
IC C5   N1   *C6   H6        1.3606  119.86  179.89  116.87  1.0858
IC C4   N4   CM4   H41       1.3614  124.86  177.76  108.04  1.0911
IC H41  N4   *CM4  H42       1.0911  108.04  117.87  110.97  1.0940
IC H41  N4   *CM4  H43       1.0911  108.04 -119.76  112.67  1.0970
DONO H4   N4
ACCE O2   C2
ACCE N3
ACCE O1P  P
ACCE O2P  P
ACCE O2'
ACCE O3'
ACCE O4'
ACCE O5'

RESI 4AC            -1.00 ! N4-acetylcytidine    ! adjusted
GROUP
ATOM N1     NG2R61 -0.11 !              H82
ATOM C2     CG2R63  0.52 !               |
ATOM O2     OG2D4  -0.54 !          H81-C8 -H83
ATOM N3     NG2R62 -0.79 !               |
ATOM C4     CG2R64  0.65 !              C7   H4
ATOM N4     NG2S1  -0.63 !             // \  /
ATOM H4     HGP1    0.37 !            O7   N4
ATOM C5     CG2R62 -0.08 !                 |
ATOM H5     HGR62   0.12 !                 C4
ATOM C6     CG2R62  0.14 !                /  \\
ATOM H6     HGR62   0.20 !            H5-C5   N3
ATOM C7     CG2O1   0.59 !               ||   |
ATOM O7     OG2D1  -0.50 !            H6-C6   C2
ATOM C8     CG331  -0.21 !                \  / \\
ATOM H81    HGA3    0.09 !                 N1   O2
ATOM H82    HGA3    0.09 !                  \
ATOM H83    HGA3    0.09 !                   \
GROUP                    !                    \
ATOM P      P       1.50 ! O1P    H5' H4'  O4'  \
ATOM O1P    ON3    -0.78 !  |      |    \ /   \  \
ATOM O2P    ON3    -0.78 ! -P-O5'-C5'---C4'    C1'
```

```
ATOM O5'    ON2     -0.57 !  |       |      \     / \
ATOM C5'    CN8B    -0.08 ! O2P     H5''   C3'--C2' H1'
ATOM H5'    HN8      0.09 !               / \   / \
ATOM H5''   HN8      0.09 !             O3' H3' O2' H2''
GROUP                     !              |       |
ATOM C4'    CN7      0.16 !                     H2'
ATOM H4'    HN7      0.09
ATOM O4'    ON6B    -0.50
ATOM C1'    CN7B     0.16
ATOM H1'    HN7      0.09
GROUP
ATOM C2'    CN7B     0.14
ATOM H2''   HN7      0.09
ATOM O2'    ON5     -0.66
ATOM H2'    HN5      0.43
GROUP
ATOM C3'    CN7      0.01
ATOM H3'    HN7      0.09
ATOM O3'    ON2     -0.57
BOND N1   C2   N1   C6   C2   O2   C2   N3
BOND N3   C4   C4   C5   C4   N4   N4   H4
BOND N4   C7   C7   C8   C7   O7   C8   H81
BOND C8   H82  C8   H83  C5   C6   C5   H5
BOND C6   H6
BOND P    O1P     P    O2P     P    O5'     O5'  C5'     C5'  H5''
BOND C5'  C4'     C4'  O4'     C4'  C3'     O4'  C1'
BOND C1'  N1      C1'  C2'     C2'  C3'     C3'  O3'     O3'  +P
BOND C2'  O2'     O2'  H2'
BOND C1'  H1'     C2'  H2''    C3'  H3'     C4'  H4'     C5'  H5'
IMPR C2   N1   N3   O2        C4   C5   N3   N4        C7   C8   N4   O7
!ribose
IC -O3' P    O5'  C5'    1.6001  101.45  -39.25  119.00  1.4401
IC -O3' O5'  *P   O1P    1.6001  101.45 -115.82  109.74  1.4802
IC -O3' O5'  *P   O2P    1.6001  101.45  115.90  109.80  1.4801
IC  P   O5'  C5'  C4'    1.5996  119.00 -151.39  110.04  1.5160
IC O5'  C5'  C4'  C3'    1.4401  108.83 -179.85  116.10  1.5284
IC C5'  C4'  C3'  O3'    1.5160  116.10   76.70  115.12  1.4212
IC C4'  C3'  O3'  +P     1.5284  111.92  159.13  119.05  1.6001
IC C3'  O3'  +P   +O5'   1.4212  119.05  -98.86  101.45  1.5996
IC O4'  C3'  *C4' C5'    1.4572  104.06 -120.04  116.10  1.5160
IC C2'  C4'  *C3' O3'    1.5284  100.16 -124.08  115.12  1.4212
IC C4'  C3'  C2'  C1'    1.5284  100.16   39.58  102.04  1.5251
IC C3'  C2'  C1'  N1     1.5284  101.97  144.39  113.71  1.4896
IC O4'  C1'  N1   C2     1.5251  113.71  -96.0   117.06  1.3746
IC C1'  C3'  *C2' O2'    1.5284  102.04 -114.67  110.81  1.4212
IC H2'  O2'  C2'  C3'    0.9600  114.97  148.63  111.92  1.5284
IC O4'  C2'  *C1' H1'    0.0       0.0  -115.0     0.0   0.0
IC C1'  C3'  *C2' H2''   0.0       0.0   115.0     0.0   0.0
IC C2'  C4'  *C3' H3'    0.0       0.0   115.0     0.0   0.0
IC C3'  O4'  *C4' H4'    0.0       0.0  -115.0     0.0   0.0
IC C4'  O5'  *C5' H5'    0.0       0.0  -115.0     0.0   0.0
IC C4'  O5'  *C5' H5''   0.0       0.0   115.0     0.0   0.0
IC C2   C6   *N1  C1'       1.3966  121.57  179.97  122.49  1.4896
IC C3'  C2'  C1'  N1     1.5284  101.97  144.39  113.71  1.4896
IC O4'  C1'  N1   C2     1.5251  113.71  -96.0   117.06  1.3746
IC C1'  C2   *N1  C6     1.3966  121.57  179.97  122.49  1.4896
IC C6   N1   C2   N3        1.3584  123.86   -0.78  115.63  1.3840
IC N3   N1   *C2  O2        1.3840  115.63 -179.77  119.60  1.2308
IC N1   C2   N3   C4        1.4126  115.63    0.58  120.07  1.3214
IC C2   N3   C4   N4        1.3840  120.07  179.26  112.92  1.3951
IC N4   N3   *C4  C5        1.3951  112.92 -179.12  125.01  1.4277
IC N3   C4   N4   C7        1.3214  112.92  173.17  129.44  1.3876
IC C7   C4   *N4  H4        1.3876  129.44 -173.25  112.00  1.0190
```

```
IC C6   C4   *C5   H5        1.3657  115.16 -179.24  122.45  1.0800
IC C5   N1   *C6   H6        1.3657  120.27 -179.85  116.65  1.0861
IC C4   N4   C7    O7        1.3951  129.44    4.10  123.54  1.2319
IC O7   N4   *C7   C8        1.2319  123.54 -179.26  113.46  1.5121
IC N4   C7   C8    H81       1.3876  113.46 -166.55  108.30  1.0903
IC H81  C7   *C8   H82       1.0903  108.30  120.91  111.62  1.0940
IC H81  C7   *C8   H83       1.0903  108.30 -118.93  109.83  1.0956
DONO H2'  O2'
DONO H4   N4
ACCE O2   C2
ACCE N3
ACCE O7
ACCE O1P  P
ACCE O2P  P
ACCE O2'
ACCE O3'
ACCE O4'
ACCE O5'

RESI MAC           -1.00 ! N4-acetyl-2'-O-methylcytidine
GROUP
ATOM N1     NG2R61 -0.11 !               H82
ATOM C2     CG2R63  0.52 !                |
ATOM O2     OG2D4  -0.54 !           H81-C8 -H83
ATOM N3     NG2R62 -0.79 !                |
ATOM C4     CG2R64  0.65 !               C7   H4
ATOM N4     NG2S1  -0.63 !              // \  /
ATOM H4     HGP1    0.37 !             O7   N4
ATOM C5     CG2R62 -0.08 !                   |
ATOM H5     HGR62   0.12 !                  C4
ATOM C6     CG2R62  0.14 !                 /  \\
ATOM H6     HGR62   0.20 !             H5-C5   N3
ATOM C7     CG2O1   0.59 !                ||   |
ATOM O7     OG2D1  -0.50 !             H6-C6   C2
ATOM C8     CG331  -0.21 !                 \  / \\
ATOM H81    HGA3    0.09 !                  N1   O2
ATOM H82    HGA3    0.09 !                   \
ATOM H83    HGA3    0.09 !                    \
GROUP                    !                     \
ATOM P      P        1.50 ! O1P    H5' H4'  O4'   \
ATOM O1P    ON3     -0.78 !  |       |    \ /   \  \
ATOM O2P    ON3     -0.78 ! -P-O5'-C5'---C4'    C1'
ATOM O5'    ON2     -0.57 !  |       |     \     / \
ATOM C5'    CN8B    -0.08 ! O2P    H5''   C3'--C2' H1'
ATOM H5'    HN8      0.09 !              / \   / \
ATOM H5''   HN8      0.09 !            O3' H3' O2' H2''
GROUP                    !             |      |
ATOM C4'    CN7      0.16 !                   CM2
ATOM H4'    HN7      0.09 !                 /  |  \
ATOM O4'    ON6B    -0.50 !               HM1 HM2 HM3
ATOM C1'    CN7B     0.16
ATOM H1'    HN7      0.09
GROUP
ATOM C2'    CN7B     0.08
ATOM H2''   HN7      0.09
ATOM O2'    OG301   -0.34
ATOM CM2    CG331   -0.10
ATOM HM1    HGA3     0.09
ATOM HM2    HGA3     0.09
ATOM HM3    HGA3     0.09
GROUP
ATOM C3'    CN7      0.01
ATOM H3'    HN7      0.09
```

```
ATOM O3'    ON2    -0.57
BOND N1   C2   N1   C6   C2   O2   C2   N3
BOND N3   C4   C4   C5   C4   N4   N4   H4
BOND N4   C7   C7   C8   C7   O7   C8   H81
BOND C8   H82  C8   H83  C5   C6   C5   H5
BOND C6   H6
BOND P    O1P     P    O2P     P    O5'     O5'  C5'     C5'  H5''
BOND C5'  C4'     C4'  O4'     C4'  C3'     O4'  C1'
BOND C1'  N1      C1'  C2'     C2'  C3'     C3'  O3'     O3'  +P
BOND C2'  O2'     CM2  O2'     CM2  HM1     HM2  CM2     HM3  CM2
BOND C1'  H1'     C2'  H2''    C3'  H3'     C4'  H4'     C5'  H5'
IMPR C2   N1   N3   O2        C4   C5   N3   N4       C7   C8   N4   O7
!2OM-ribose
IC -O3' P    O5'  C5'    1.6001  101.45  -39.25  119.00  1.4401
IC -O3' O5'  *P   O1P    1.6001  101.45 -115.82  109.74  1.4802
IC -O3' O5'  *P   O2P    1.6001  101.45  115.90  109.80  1.4801
IC  P   O5'  C5'  C4'    1.5996  119.00 -151.39  110.04  1.5160
IC O5'  C5'  C4'  C3'    1.4401  108.83 -179.85  116.10  1.5284
IC C5'  C4'  C3'  O3'    1.5160  116.10   76.70  115.12  1.4212
IC C4'  C3'  O3'  +P     1.5284  111.92  159.13  119.05  1.6001
IC C3'  O3'  +P   +O5'   1.4212  119.05  -98.86  101.45  1.5996
IC O4'  C3'  *C4' C5'    1.4572  104.06 -120.04  116.10  1.5160
IC C2'  C4'  *C3' O3'    1.5284  100.16 -124.08  115.12  1.4212
IC C4'  C3'  C2'  C1'    1.5284  100.16   39.58  102.04  1.5251
IC C3'  C2'  C1'  N1     1.5284  101.97  144.39  113.71  1.4896
IC O4'  C1'  N1   C2     1.5251  113.71  -96.0   117.06  1.3746
IC C3'  C1'  *C2' O2'    1.5312  102.03  117.61  107.13  1.4206
IC C1'  C2'  O2'  CM2    1.5393  107.13   90.00  107.00  1.4150
IC C2'  O2'  CM2  HM2    1.4206  107.00  180.00    0.0   0.0
IC HM2  O2'  *CM2 HM3    0.0       0.0   120.00    0.0   0.0
IC HM2  O2'  *CM2 HM1    0.0       0.0  -120.00    0.0   0.0
IC O4'  C2'  *C1' H1'    0.0       0.0  -115.0     0.0   0.0
IC C1'  C3'  *C2' H2''   0.0       0.0   115.0     0.0   0.0
IC C2'  C4'  *C3' H3'    0.0       0.0   115.0     0.0   0.0
IC C3'  O4'  *C4' H4'    0.0       0.0  -115.0     0.0   0.0
IC C4'  O5'  *C5' H5'    0.0       0.0  -115.0     0.0   0.0
IC C4'  O5'  *C5' H5''   0.0       0.0   115.0     0.0   0.0
IC C2   C6   *N1  C1'       1.3966  121.57  179.97  122.49  1.4896
IC C3'  C2'  C1'  N1     1.5284  101.97  144.39  113.71  1.4896
IC O4'  C1'  N1   C2     1.5251  113.71  -96.0   117.06  1.3746
IC C1'  C2   *N1  C6     1.3966  121.57  179.97  122.49  1.4896
IC C6   N1   C2   N3        1.3584  123.86   -0.78  115.63  1.3840
IC N3   N1   *C2  O2        1.3840  115.63 -179.77  119.60  1.2308
IC N1   C2   N3   C4        1.4126  115.63    0.58  120.07  1.3214
IC C2   N3   C4   N4        1.3840  120.07  179.26  112.92  1.3951
IC N4   N3   *C4  C5        1.3951  112.92 -179.12  125.01  1.4277
IC N3   C4   N4   C7        1.3214  112.92  173.17  129.44  1.3876
IC C7   C4   *N4  H4        1.3876  129.44 -173.25  112.00  1.0190
IC C6   C4   *C5  H5        1.3657  115.16 -179.24  122.45  1.0800
IC C5   N1   *C6  H6        1.3657  120.27 -179.85  116.65  1.0861
IC C4   N4   C7   O7        1.3951  129.44    4.10  123.54  1.2319
IC O7   N4   *C7  C8        1.2319  123.54 -179.26  113.46  1.5121
IC N4   C7   C8   H81       1.3876  113.46 -166.55  108.30  1.0903
IC H81  C7   *C8  H82       1.0903  108.30  120.91  111.62  1.0940
IC H81  C7   *C8  H83       1.0903  108.30 -118.93  109.83  1.0956
DONO H4   N4
ACCE O2   C2
ACCE N3
ACCE O7
ACCE O1P  P
ACCE O2P  P
ACCE O2'
ACCE O3'
```

```
ACCE O4'
ACCE O5'

RESI TMC           -1.00 ! N4,N4,2'-O-trimethylcytidine
GROUP
ATOM N1     NG2R61 -0.03 !            H73      H83
ATOM C2     CG2R63  0.57 !              \      /
ATOM O2     OG2D4  -0.52 !           H72-C7  C8-H82
ATOM N3     NG2R62 -0.74 !              / \  / \
ATOM C4     CG2R64  0.60 !            H71  N4  H81
ATOM N4     NG301  -0.37 !                 |
ATOM C5     CG2R62 -0.18 !                 C4
ATOM H5     HGR62   0.03 !                /  \\
ATOM C6     CG2R62  0.11 !            H5-C5   N3
ATOM H6     HGR62   0.13 !               ||   |
ATOM C7     CG331  -0.07 !            H6-C6   C2
ATOM H71    HGA3    0.09 !                \  / \\
ATOM H72    HGA3    0.09 !                 N1   O2
ATOM H73    HGA3    0.09 !                  \
ATOM C8     CG331  -0.07 !                   \
ATOM H81    HGA3    0.09 !                    \
ATOM H82    HGA3    0.09 ! O1P    H5' H4'  O4'  \
ATOM H83    HGA3    0.09 !  |      |    \ /   \  \
GROUP                    ! -P-O5'-C5'---C4'    C1'
ATOM P      P       1.50 !  |      |     \     / \
ATOM O1P    ON3    -0.78 ! O2P    H5''   C3'--C2' H1'
ATOM O2P    ON3    -0.78 !              / \   / \
ATOM O5'    ON2    -0.57 !           O3' H3' O2' H2''
ATOM C5'    CN8B   -0.08 !            |       |
ATOM H5'    HN8     0.09 !                   CM2
ATOM H5''   HN8     0.09 !                 /  |  \
GROUP                    !               HM1 HM2 HM3
ATOM C4'    CN7     0.16
ATOM H4'    HN7     0.09
ATOM O4'    ON6B   -0.50
ATOM C1'    CN7B    0.16
ATOM H1'    HN7     0.09
GROUP
ATOM C2'    CN7B    0.08
ATOM H2''   HN7     0.09
ATOM O2'    OG301  -0.34
ATOM CM2    CG331  -0.10
ATOM HM1    HGA3    0.09
ATOM HM2    HGA3    0.09
ATOM HM3    HGA3    0.09
GROUP
ATOM C3'    CN7     0.01
ATOM H3'    HN7     0.09
ATOM O3'    ON2    -0.57
BOND N1   C2   N1   C6   C2   O2   C2   N3
BOND N3   C4   C4   C5   C4   N4   N4   C7
BOND N4   C8   C7   H71  C7   H72  C7   H73
BOND C8   H81  C8   H82  C8   H83  C5   C6
BOND C5   H5   C6   H6
BOND P    O1P     P    O2P     P    O5'     O5'  C5'     C5'  H5''
BOND C5'  C4'     C4'  O4'     C4'  C3'     O4'  C1'
BOND C1'  N1      C1'  C2'     C2'  C3'     C3'  O3'     O3'  +P
BOND C2'  O2'     CM2  O2'     CM2  HM1     HM2  CM2     HM3  CM2
BOND C1'  H1'     C2'  H2''    C3'  H3'     C4'  H4'     C5'  H5'
IMPR C2   N1   N3   O2        C4   C5   N3   N4
!ribose
IC -O3' P    O5'   C5'    1.6001  101.45  -39.25  119.00  1.4401
IC -O3' O5'  *P    O1P    1.6001  101.45 -115.82  109.74  1.4802
```

```
IC -O3' O5'  *P   O2P    1.6001  101.45  115.90  109.80  1.4801
IC  P   O5'  C5'  C4'    1.5996  119.00 -151.39  110.04  1.5160
IC O5'  C5'  C4'  C3'    1.4401  108.83 -179.85  116.10  1.5284
IC C5'  C4'  C3'  O3'    1.5160  116.10   76.70  115.12  1.4212
IC C4'  C3'  O3'  +P     1.5284  111.92  159.13  119.05  1.6001
IC C3'  O3'  +P   +O5'   1.4212  119.05  -98.86  101.45  1.5996
IC O4'  C3'  *C4' C5'    1.4572  104.06 -120.04  116.10  1.5160
IC C2'  C4'  *C3' O3'    1.5284  100.16 -124.08  115.12  1.4212
IC C4'  C3'  C2'  C1'    1.5284  100.16   39.58  102.04  1.5251
IC C3'  C1'  *C2' O2'    1.5312  102.03  117.61  107.13  1.4206
IC C1'  C2'  O2'  CM2    1.5393  107.13   90.00  107.00  1.4150
IC C2'  O2'  CM2  HM2    1.4206  107.00  180.00    0.0   0.0
IC HM2  O2'  *CM2 HM3    0.0       0.0   120.00    0.0   0.0
IC HM2  O2'  *CM2 HM1    0.0       0.0  -120.00    0.0   0.0
IC O4'  C2'  *C1' H1'    0.0       0.0  -115.0     0.0   0.0
IC C1'  C3'  *C2' H2''   0.0       0.0   115.0     0.0   0.0
IC C2'  C4'  *C3' H3'    0.0       0.0   115.0     0.0   0.0
IC C3'  O4'  *C4' H4'    0.0       0.0  -115.0     0.0   0.0
IC C4'  O5'  *C5' H5'    0.0       0.0  -115.0     0.0   0.0
IC C4'  O5'  *C5' H5''   0.0       0.0   115.0     0.0   0.0
IC C3'  C2'  C1'  N1     1.5284  101.97  144.39  113.71  1.4896
IC O4'  C1'  N1   C2     1.5251  113.71  -96.0   117.06  1.3746
IC C1'  C2   *N1  C6     1.3966  121.57  179.97  122.49  1.4896
IC C6   N1   C2   N3       1.3584  123.86   -0.78  115.63  1.3840
IC N3   N1   *C2  O2       1.3840  115.63 -179.77  119.60  1.2308
IC N1   C2   N3   C4       1.4126  115.63    0.58  120.07  1.3214
IC C2   N3   C4   N4       1.3840  120.07  179.26  112.92  1.3951
IC N4   N3   *C4  C5       1.3951  112.92 -179.12  125.01  1.4277
IC N3   C4   N4   C7       1.3214  112.92  173.17  129.44  1.3876
IC C7   C4   *N4  C8       1.3876  129.44 -173.25  112.00  1.0190
IC C6   C4   *C5  H5       1.3657  115.16 -179.24  122.45  1.0800
IC C5   N1   *C6  H6       1.3657  120.27 -179.85  116.65  1.0861
IC C4   N4   C7   O7       1.3951  129.44    4.10  123.54  1.2319
IC C8   N4   C7   H71      1.3876  113.46 -166.55  108.30  1.0903
IC H71  N4   *C7  H72      1.0903  108.30  120.91  111.62  1.0940
IC H71  N4   *C7  H73      1.0903  108.30 -118.93  109.83  1.0956
IC C7   N4   C8   H81      1.3876  113.46 -166.55  108.30  1.0903
IC H81  N4   *C8  H82      1.0903  108.30  120.91  111.62  1.0940
IC H81  N4   *C8  H83      1.0903  108.30 -118.93  109.83  1.0956
ACCE O2   C2
ACCE N3
ACCE O1P  P
ACCE O2P  P
ACCE O2'
ACCE O3'
ACCE O4'
ACCE O5'

RESI 3MC           0.00 ! protonated N3-methylcytidine
GROUP
ATOM N1     NG2R61 -0.11 !             H42  H41
ATOM C2     CG2R63  0.55 !               \  /
ATOM O2     OG2D4  -0.33 !            (+) N4      H31
ATOM N3     NG2P1  -0.35 !                ||     /
ATOM C4     CG2R64  0.68 !                C4   CN3-H32
ATOM N4     NG2P1  -0.76 !               /  \ /  \
ATOM H41    HGP2    0.39 !           H5-C5   N3   H33
ATOM H42    HGP2    0.39 !              ||   |
ATOM C5     CG2R62 -0.18 !           H6-C6   C2
ATOM H5     HGR62   0.09 !               \  / \\
ATOM C6     CG2R62  0.16 !                N1   O2
ATOM H6     HGR62   0.20 !                  \
ATOM CN3    CG334   0.00 !                   \
```

```
ATOM H31    HGA3    0.09 !                      \
ATOM H32    HGA3    0.09 ! O1P    H5' H4'  O4'  \
ATOM H33    HGA3    0.09 !  |      |    \ /   \  \
GROUP                    ! -P-O5'-C5'---C4'    C1'
ATOM P      P       1.50 !  |      |     \     / \
ATOM O1P    ON3    -0.78 ! O2P    H5''   C3'--C2' H1'
ATOM O2P    ON3    -0.78 !              / \   / \
ATOM O5'    ON2    -0.57 !           O3' H3' O2' H2''
ATOM C5'    CN8B   -0.08 !            |       |
ATOM H5'    HN8     0.09 !                    H2'
ATOM H5''   HN8     0.09
GROUP                    !  !!!! PATCH 3MCN for the neutral base
ATOM C4'    CN7     0.16
ATOM H4'    HN7     0.09
ATOM O4'    ON6B   -0.50
ATOM C1'    CN7B    0.16
ATOM H1'    HN7     0.09
GROUP
ATOM C2'    CN7B    0.14
ATOM H2''   HN7     0.09
ATOM O2'    ON5    -0.66
ATOM H2'    HN5     0.43
GROUP
ATOM C3'    CN7     0.01
ATOM H3'    HN7     0.09
ATOM O3'    ON2    -0.57
BOND N1   C2   N1   C6   C2   O2   C2   N3
BOND N3   C4   N3   CN3  CN3  H31  CN3  H32
BOND CN3  H33  C4   N4   C4   C5   N4   H41
BOND N4   H42  C5   C6   C5   H5   C6   H6
BOND P    O1P     P    O2P     P    O5'     O5'  C5'     C5'  H5''
BOND C5'  C4'     C4'  O4'     C4'  C3'     O4'  C1'
BOND C1'  N1      C1'  C2'     C2'  C3'     C3'  O3'     O3'  +P
BOND C2'  O2'     O2'  H2'
BOND C1'  H1'     C2'  H2''    C3'  H3'     C4'  H4'     C5'  H5'
IMPR C2   N1   N3   O2        C4   C5   N3   N4        N4   H41  H42  C4
!ribose
IC -O3' P    O5'   C5'    1.6001  101.45  -39.25  119.00  1.4401
IC -O3' O5'  *P    O1P    1.6001  101.45 -115.82  109.74  1.4802
IC -O3' O5'  *P    O2P    1.6001  101.45  115.90  109.80  1.4801
IC  P   O5'  C5'   C4'    1.5996  119.00 -151.39  110.04  1.5160
IC O5'  C5'  C4'   C3'    1.4401  108.83 -179.85  116.10  1.5284
IC C5'  C4'  C3'   O3'    1.5160  116.10   76.70  115.12  1.4212
IC C4'  C3'  O3'   +P     1.5284  111.92  159.13  119.05  1.6001
IC C3'  O3'  +P    +O5'   1.4212  119.05  -98.86  101.45  1.5996
IC O4'  C3'  *C4'  C5'    1.4572  104.06 -120.04  116.10  1.5160
IC C2'  C4'  *C3'  O3'    1.5284  100.16 -124.08  115.12  1.4212
IC C4'  C3'  C2'   C1'    1.5284  100.16   39.58  102.04  1.5251
IC C3'  C2'  C1'   N1     1.5284  101.97  144.39  113.71  1.4896
IC O4'  C1'  N1    C2     1.5251  113.71  -96.0   117.06  1.3746
IC C1'  C3'  *C2'  O2'    1.5284  102.04 -114.67  110.81  1.4212
IC H2'  O2'  C2'   C3'    0.9600  114.97  148.63  111.92  1.5284
IC O4'  C2'  *C1'  H1'    0.0       0.0  -115.0     0.0   0.0
IC C1'  C3'  *C2'  H2''   0.0       0.0   115.0     0.0   0.0
IC C2'  C4'  *C3'  H3'    0.0       0.0   115.0     0.0   0.0
IC C3'  O4'  *C4'  H4'    0.0       0.0  -115.0     0.0   0.0
IC C4'  O5'  *C5'  H5'    0.0       0.0  -115.0     0.0   0.0
IC C4'  O5'  *C5'  H5''   0.0       0.0   115.0     0.0   0.0
IC C2   C6   *N1   C1'       1.3966  121.57  179.97  122.49  1.4896
IC C3'  C2'  C1'   N1     1.5284  101.97  144.39  113.71  1.4896
IC O4'  C1'  N1    C2     1.5251  113.71  -96.0   117.06  1.3746
IC C1'  C2   *N1   C6     1.3966  121.57  179.97  122.49  1.4896
IC C6   N1   C2    N3        1.3579  121.42    0.02  119.32  1.3485
```

```
IC N3   N1   *C2   O2        1.3485  119.32  179.99  117.06  1.2237
IC N1   C2   N3    C4        1.3785  119.32   -0.02  121.55  1.3603
IC C2   N3   C4    N4        1.3485  121.55  179.99  119.22  1.3772
IC N4   N3   *C4   C5        1.3772  119.22 -179.98  119.05  1.4401
IC C6   C4   *C5   H5        1.3653  118.25 -179.98  122.23  1.0882
IC C5   N1   *C6   H6        1.3653  120.40 -179.97  118.26  1.0924
IC N3   C4   N4    C7        1.3603  119.22  179.98  120.97  1.4598
IC C7   C4   *N4   C8        1.4598  120.97 -179.96  119.83  1.4631
IC C4   N4   C7    H71       1.3772  120.97  179.99  111.33  1.1118
IC H71  N4   *C7   H72       1.1118  111.33  119.65  111.27  1.1140
IC H71  N4   *C7   H73       1.1118  111.33 -119.54  111.21  1.1147
IC C4   N4   C8    H81       1.3772  119.83  179.94  111.72  1.1110
IC H81  N4   *C8   H82       1.1110  111.72  120.21  111.11  1.1142
IC H81  N4   *C8   H83       1.1110  111.72 -120.10  111.12  1.1142
IC C2   C6   *N1   C1'       1.3966  121.57  179.97  122.49  1.4896
IC H1   N1   C2    N3        1.0191  114.23  179.97  113.29  1.4259
IC N3   N1   *C2   O2        1.4259  113.29 -179.98  123.65  1.2129
IC N1   C2   N3    C4        1.3880  113.29    0.02  123.33  1.3567
IC C4   C2   *N3   CN3       1.3567  123.33 -179.99  116.07  1.4738
IC C2   N3   C4    N4        1.4259  123.33 -179.97  119.40  1.3326
IC N4   N3   *C4   C5        1.3326  119.40  179.95  119.73  1.4220
IC N3   C4   N4    H41       1.3567  119.40  180.00  119.78  1.0139
IC H41  C4   *N4   H42       1.0139  119.78  179.97  123.33  1.0123
IC C6   C4   *C5   H5        1.3597  118.06  179.99  120.70  1.0826
IC C5   N1   *C6   H6        1.3597  120.74 -179.98  116.41  1.0853
IC C2   N3   CN3   H31       1.4259  116.07 -118.73  109.65  1.0926
IC H31  N3   *CN3  H32       1.0926  109.65  118.70  107.25  1.0874
IC H31  N3   *CN3  H33       1.0926  109.65 -122.57  109.72  1.0923
DONO H2'  O2'
DONO H41  N4
DONO H42  N4
ACCE O2   C2
ACCE O1P  P
ACCE O2P  P
ACCE O2'
ACCE O3'
ACCE O4'
ACCE O5'

RESI K2C            0.00 ! lysidine
GROUP
ATOM N1     NG2P1  -0.24 !             H4
ATOM C2     CG2R64  0.77 !               \
ATOM N2     NG2P1  -0.60 !                N4
ATOM H2     HGP2    0.49 !                ||
ATOM N3     NG2P1  -0.81 !                C4    H3
ATOM H3     HGP2    0.44 !               /  \  /
ATOM C4     CG2R64  0.57 !           H5-C5   N3
ATOM N4     NG2D1  -0.68 !              ||   |
ATOM H4     HGP1    0.34 !           H6-C6   C2      H71 H81  H91  H101 H12    O30 (-
)
ATOM C5     CG2R61 -0.24 !               \  /  \\(+) |   |    |    |    |     /
ATOM H5     HGR61   0.21 !                N1    N2 --C7--C8 --C9 --C10--C12--C13
ATOM C6     CG2R61  0.13 !                 \    |    |   |    |    |    |     \\
ATOM H6     HGR62   0.20 !                  \   H2   H72 H82  H92  H102 | (+)  O31
ATOM C7     CG324   0.24 !                   \                         N14
ATOM H71    HGA2    0.09 !                    \                       / |  \
ATOM H72    HGA2    0.09 !                     \                  H141 H142 H143
GROUP                    ! O1P    H5' H4'  O4'  \
ATOM C8     CG321  -0.18 !  |      |    \ /   \  \
ATOM H81    HGA2    0.09 ! -P-O5'-C5'---C4'    C1'
ATOM H82    HGA2    0.09 !  |      |     \     / \
GROUP                    ! O2P    H5''   C3'--C2' H1'
```

```
ATOM C9     CG321  -0.18 !              / \   / \
ATOM H91    HGA2    0.09 !            O3' H3' O2' H2''
ATOM H92    HGA2    0.09 !             |       |
GROUP                    !                    H2'
ATOM C10    CG321  -0.18
ATOM H101   HGA2    0.09 !  !!!! PATCH 5UHA for the non-ionic tautomer of side chain
ATOM H102   HGA2    0.09 !  !!!! PATCH K2CN for the neutral base
GROUP                    !  !!!! PATCH 34HC for the tautomer of charged base
ATOM C12    CG314   0.17
ATOM H12    HGA1    0.11
ATOM N14    NG3P3  -0.34
ATOM H141   HGP2    0.30
ATOM H142   HGP2    0.30
ATOM H143   HGP2    0.30
ATOM C13    CG2O3   0.32
ATOM O30    OG2D2  -0.58
ATOM O31    OG2D2  -0.58
GROUP
ATOM P      P       1.50
ATOM O1P    ON3    -0.78
ATOM O2P    ON3    -0.78
ATOM O5'    ON2    -0.57
ATOM C5'    CN8B   -0.08
ATOM H5'    HN8     0.09
ATOM H5''   HN8     0.09
GROUP
ATOM C4'    CN7     0.16
ATOM H4'    HN7     0.09
ATOM O4'    ON6B   -0.50
ATOM C1'    CN7B    0.16
ATOM H1'    HN7     0.09
GROUP
ATOM C2'    CN7B    0.14
ATOM H2''   HN7     0.09
ATOM O2'    ON5    -0.66
ATOM H2'    HN5     0.43
GROUP
ATOM C3'    CN7     0.01
ATOM H3'    HN7     0.09
ATOM O3'    ON2    -0.57
BOND N1   C2   N1   C6   C2   N3   C2   N2
BOND N3   C4   C4   N4   C4   C5   N3   H3
BOND N4   H4   C5   H5   C5   C6   C6   H6
BOND N2   H2   N2   C7   C7   H71  C7   H72
BOND C7   C8   C8   H81  C8   H82  C8   C9
BOND C9   H91  C9   H92  C9   C10  C10  H101
BOND C10  H102 C10  C12  C12  H12  C12  C13
BOND C12  N14  C13  O30  C13  O31  N14  H141
BOND N14  H142 N14  H143
BOND P    O1P     P    O2P     P    O5'     O5'  C5'     C5'  H5''
BOND C5'  C4'     C4'  O4'     C4'  C3'     O4'  C1'
BOND C1'  N1      C1'  C2'     C2'  C3'     C3'  O3'     O3'  +P
BOND C2'  O2'     O2'  H2'
BOND C1'  H1'     C2'  H2''    C3'  H3'     C4'  H4'     C5'  H5'
IMPR C2   N1   N3   N2        C4   C5   N4   N3        C13  O30  O31  C12
!ribose
IC -O3' P    O5'   C5'    1.6001  101.45  -39.25  119.00  1.4401
IC -O3' O5'  *P    O1P    1.6001  101.45 -115.82  109.74  1.4802
IC -O3' O5'  *P    O2P    1.6001  101.45  115.90  109.80  1.4801
IC  P   O5'  C5'   C4'    1.5996  119.00 -151.39  110.04  1.5160
IC O5'  C5'  C4'   C3'    1.4401  108.83 -179.85  116.10  1.5284
IC C5'  C4'  C3'   O3'    1.5160  116.10   76.70  115.12  1.4212
IC C4'  C3'  O3'   +P     1.5284  111.92  159.13  119.05  1.6001
```

```
IC C3'  O3'  +P    +O5'   1.4212  119.05  -98.86  101.45  1.5996
IC O4'  C3'  *C4'  C5'    1.4572  104.06 -120.04  116.10  1.5160
IC C2'  C4'  *C3'  O3'    1.5284  100.16 -124.08  115.12  1.4212
IC C4'  C3'  C2'   C1'    1.5284  100.16   39.58  102.04  1.5251
IC N1   C1'  C2'   C3'    1.4699  112.37   91.35  100.52  1.5284
IC C2   N1   C1'   C2'    1.4281  121.60  141.79  112.37  1.5251
IC C1'  C3'  *C2'  O2'    1.5284  102.04 -114.67  110.81  1.4212
IC H2'  O2'  C2'   C3'    0.9600  114.97  148.63  111.92  1.5284
IC O4'  C2'  *C1'  H1'    0.0       0.0  -115.0     0.0   0.0
IC C1'  C3'  *C2'  H2''   0.0       0.0   115.0     0.0   0.0
IC C2'  C4'  *C3'  H3'    0.0       0.0   115.0     0.0   0.0
IC C3'  O4'  *C4'  H4'    0.0       0.0  -115.0     0.0   0.0
IC C4'  O5'  *C5'  H5'    0.0       0.0  -115.0     0.0   0.0
IC C4'  O5'  *C5'  H5''   0.0       0.0   115.0     0.0   0.0
IC C6   C2   *N1   C1'       1.4026  119.19 -179.92  120.52  1.4695
IC C6   N1   C2    N2        1.3751  118.04  176.32  119.27  1.3699
IC N2   N1   *C2   N3        1.3699  119.27 -179.78  121.03  1.3472
IC N1   C2   N2    C7        1.4281  119.27 -166.76  122.57  1.5023
IC C7   C2   *N2   H2        1.5023  122.57  134.97  111.40  1.0106
IC N1   C2   N3    C4        1.4281  121.03   15.57  117.79  1.3483
IC C4   C2   *N3   H3        1.3483  117.79  180.00  122.70  1.0000
IC C2   N3   C4    N4        1.3472  117.79  156.31  118.26  1.2967
IC N4   N3   *C4   C5        1.2967  118.26 -178.11  122.91  1.3959
IC N3   C4   N4    H4        1.3483  118.26  178.17  106.15  0.9981
IC C6   C4   *C5   H5        1.4055  116.26  171.79  121.47  1.0752
IC C5   N1   *C6   H6        1.4055  120.96 -176.28  116.78  1.0824
IC C2   N2   C7    C8        1.3699  122.57   72.89  117.61  1.5545
IC C8   N2   *C7   H71       1.5545  117.61  120.96  107.47  1.1162
IC H71  N2   *C7   H72       1.1162  107.47  114.47  109.54  1.1135
IC N2   C7   C8    C9        1.5023  117.61  -68.85  112.87  1.5333
IC C9   C7   *C8   H81       1.5333  112.87 -118.63  107.27  1.1151
IC H81  C7   *C8   H82       1.1151  107.27 -117.09  110.75  1.1101
IC C7   C8   C9    C10       1.5545  112.87 -160.53  119.34  1.5307
IC C10  C8   *C9   H91       1.5307  119.34 -124.15  108.88  1.1141
IC H91  C8   *C9   H92       1.1141  108.88 -117.17  105.14  1.1164
IC C8   C9   C10   C12       1.5333  119.34  178.10  144.77  2.5600
IC C12  C9   *C10  H101      2.5600  144.77  124.97  110.17  1.1132
IC H101 C9   *C10  H102      1.1132  110.17  118.23  110.16  1.1124
IC C9   C10  C12   C13       1.5307  144.77  180.00  108.00  1.5220
IC C13  C10  *C12  N14       1.5220  108.00  120.00  110.00  1.4800
IC C13  C10  *C12  H12       1.5220  108.00 -120.00  110.10  1.1110
IC C10  C12  C13   O30       2.5600  108.00  180.00  116.00  1.2600
IC O30  C12  *C13  O31       1.2600  116.00  180.00  116.00  1.2600
IC C10  C12  N14   H141      2.5600  110.00  180.00  109.50  1.0400
IC H141 C12  *N14  H142      1.0400  109.50  120.00  109.50  1.0400
IC H141 C12  *N14  H143      1.0400  109.50 -120.00  109.50  1.0400
DONO H2'  O2'
DONO H4   N4
DONO H3   N3
DONO H2   N2
DONO H141 N14
DONO H142 N14
DONO H143 N14
ACCE O30  C13
ACCE O31  C13
ACCE O1P  P
ACCE O2P  P
ACCE O2'
ACCE O3'
ACCE O4'
ACCE O5'

RESI R2C           1.00 ! agmatidine, AG9
```

```
GROUP
ATOM N1      NG2P1  -0.24 !               H4
ATOM C2      CG2R64  0.77 !                 \
ATOM N2      NG2P1  -0.60 !                  N4
ATOM H2      HGP2    0.49 !                  ||
ATOM N3      NG2P1  -0.81 !                  C4    H3
ATOM H3      HGP2    0.44 !                 /  \  /
ATOM C4      CG2R64  0.57 !             H5-C5   N3                              H21
ATOM N4      NG2D1  -0.68 !                ||   |                               |
ATOM H4      HGP1    0.34 !             H6-C6   C2      H71  HB1  HG1  HD1  HE
NH2--H22
ATOM C5      CG2R61 -0.24 !                 \  /  \\(+) |    |    |    |    |     /
ATOM H5      HGR61   0.21 !                  N1    N2 --C7 --CB --CG --CD --NE --CZ
ATOM C6      CG2R61  0.13 !                   \    |    |    |    |    |       (+)\\
ATOM H6      HGR62   0.20 !                    \   H2   H72  HB2  HG2  HD2
NH1--H11
ATOM C7      CG324   0.24 !                     \                                   |
ATOM H71     HGA2    0.09 !                      \                                  H12
ATOM H72     HGA2    0.09 !  O1P    H5' H4'  O4'  \
GROUP                     !   |      |    \ /   \  \
ATOM CB      CG321  -0.18 !  -P-O5'-C5'---C4'    C1'
ATOM HB1     HGA2    0.09 !   |      |     \     / \
ATOM HB2     HGA2    0.09 !  O2P    H5''   C3'--C2' H1'
GROUP                     !               / \   / \
ATOM CG      CG321  -0.18 !             O3' H3' O2' H2''
ATOM HG1     HGA2    0.09 !              |       |
ATOM HG2     HGA2    0.09 !                     H2'
GROUP
ATOM CD      CG324   0.21 ! !! PATCH K2CN for the neutral base
ATOM HD1     HGA2    0.09 ! !! PATCH 34HC for the tautomer of charged base
ATOM HD2     HGA2    0.09
ATOM NE      NG2P1  -0.71
ATOM HE      HGP2    0.44
ATOM CZ      CG2N1   0.64
ATOM NH1     NG2P1  -0.80
ATOM H11     HGP2    0.46
ATOM H12     HGP2    0.46
ATOM NH2     NG2P1  -0.80
ATOM H21     HGP2    0.46
ATOM H22     HGP2    0.46
GROUP
ATOM P       P       1.50
ATOM O1P     ON3    -0.78
ATOM O2P     ON3    -0.78
ATOM O5'     ON2    -0.57
ATOM C5'     CN8B   -0.08
ATOM H5'     HN8     0.09
ATOM H5''    HN8     0.09
GROUP
ATOM C4'     CN7     0.16
ATOM H4'     HN7     0.09
ATOM O4'     ON6B   -0.50
ATOM C1'     CN7B    0.16
ATOM H1'     HN7     0.09
GROUP
ATOM C2'     CN7B    0.14
ATOM H2''    HN7     0.09
ATOM O2'     ON5    -0.66
ATOM H2'     HN5     0.43
GROUP
ATOM C3'     CN7     0.01
ATOM H3'     HN7     0.09
ATOM O3'     ON2    -0.57
```

```
BOND N1   C2   N1   C6   C2   N3   C2   N2
BOND N3   C4   C4   N4   C4   C5   N4   H4
BOND C5   H5   C5   C6   C6   H6   N3   H3
BOND N2   H2   N2   C7   C7   H71  C7   H72
BOND C7   CB   CB   HB1  CB   HB2  CB   CG
BOND CG   HG1  CG   HG2  CG   CD   CD   HD1
BOND CD   HD2  CD   NE   NE   HE   NE   CZ
BOND CZ   NH1  CZ   NH2  NH1  H11  NH1  H12
BOND NH2  H21  NH2  H22
BOND P    O1P     P    O2P     P    O5'     O5'  C5'     C5'  H5''
BOND C5'  C4'     C4'  O4'     C4'  C3'     O4'  C1'
BOND C1'  N1      C1'  C2'     C2'  C3'     C3'  O3'     O3'  +P
BOND C2'  O2'     O2'  H2'
BOND C1'  H1'     C2'  H2''    C3'  H3'     C4'  H4'     C5'  H5'
IMPR C2   N1   N3   N2        C4   C5   N4   N3        CZ   NE   NH1  NH2
!ribose
IC -O3' P    O5'  C5'   1.6001  101.45  -39.25  119.00  1.4401
IC -O3' O5'  *P   O1P   1.6001  101.45 -115.82  109.74  1.4802
IC -O3' O5'  *P   O2P   1.6001  101.45  115.90  109.80  1.4801
IC  P   O5'  C5'  C4'   1.5996  119.00 -151.39  110.04  1.5160
IC O5'  C5'  C4'  C3'   1.4401  108.83 -179.85  116.10  1.5284
IC C5'  C4'  C3'  O3'   1.5160  116.10   76.70  115.12  1.4212
IC C4'  C3'  O3'  +P    1.5284  111.92  159.13  119.05  1.6001
IC C3'  O3'  +P   +O5'  1.4212  119.05  -98.86  101.45  1.5996
IC O4'  C3'  *C4' C5'   1.4572  104.06 -120.04  116.10  1.5160
IC C2'  C4'  *C3' O3'   1.5284  100.16 -124.08  115.12  1.4212
IC C4'  C3'  C2'  C1'   1.5284  100.16   39.58  102.04  1.5251
IC N1   C1'  C2'  C3'   1.4699  112.37   91.35  100.52  1.5284
IC C2   N1   C1'  C2'   1.4281  121.60  141.79  112.37  1.5251
IC C1'  C3'  *C2' O2'   1.5284  102.04 -114.67  110.81  1.4212
IC H2'  O2'  C2'  C3'   0.9600  114.97  148.63  111.92  1.5284
IC O4'  C2'  *C1' H1'   0.0       0.0  -115.0     0.0   0.0
IC C1'  C3'  *C2' H2''  0.0       0.0   115.0     0.0   0.0
IC C2'  C4'  *C3' H3'   0.0       0.0   115.0     0.0   0.0
IC C3'  O4'  *C4' H4'   0.0       0.0  -115.0     0.0   0.0
IC C4'  O5'  *C5' H5'   0.0       0.0  -115.0     0.0   0.0
IC C4'  O5'  *C5' H5''  0.0       0.0   115.0     0.0   0.0
IC C6   C2   *N1  C1'   1.4026  119.19 -179.92  120.52  1.4695
IC C6   N1   C2   N3    0.0       0.0    0.0     0.0   0.0
IC N1   C2   N3   C4       1.4281  121.03   15.57  117.79  1.3483
IC C2   N3   C4   N4       1.3472  117.79  156.31  118.26  1.2967
IC N4   N3   *C4  C5       1.2967  118.26 -178.11  122.91  1.3959
IC C4   C2   *N3  H3       1.3483  117.79  180.00  122.70  1.0000
IC N3   C4   N4   H4       1.3483  118.26  178.17  106.15  0.9981
IC C6   C4   *C5  H5       1.4055  116.26  171.79  121.47  1.0752
IC C5   N1   *C6  H6       1.4055  120.96 -176.28  116.78  1.0824
IC N1   N3   *C2  N2       1.3699  119.27 -179.78  121.03  1.3472
IC N1   C2   N2   C7       1.4281  119.27 -166.76  122.57  1.5023
IC C7   C2   *N2  H2       1.5023  122.57  134.97  111.40  1.0106
IC C2   N2   C7   CB       1.3699  122.57  180.00  110.00  1.5300
IC CB   N2   *C7  H71      1.5300  110.00  120.00  107.47  1.1162
IC H71  N2   *C7  H72      1.1162  107.47  114.47  109.54  1.1135
IC N2   C7   CB   CG       1.5023  110.00  180.00  110.50  1.5300
IC CG   C7   *CB  HB1      1.5300  110.50  120.00  110.10  1.1110
IC HB1  C7   *CB  HB2      1.1110  110.10 -120.00  110.10  1.1110
IC C7   CB   CG   CD       1.5300  110.50  180.00  110.50  1.5300
IC CD   CB   *CG  HG1      1.5300  110.50  120.00  110.10  1.1110
IC HG1  CB   *CG  HG2      1.1110  110.10 -120.00  110.10  1.1110
IC CB   CG   CD   NE       1.5300  110.50  180.00  110.00  1.4530
IC NE   CG   *CD  HD1      1.4530  110.00  120.00  111.80  1.1000
IC HD1  CG   *CD  HD2      1.1000  111.80 -120.00  111.80  1.1000
IC CG   CD   NE   CZ       1.5300  110.00  180.00  120.00  1.3650
IC CZ   CD   *NE  HE       1.3650  120.00  180.00  120.00  1.0000
```

```
IC CD   NE   CZ    NH1       1.4530  120.00  180.00  120.00  1.3650
IC NH1  NE   *CZ   NH2       1.3650  120.00  180.00  120.00  1.3650
IC NE   CZ   NH1   H11       1.3650  120.00  180.00  120.00  1.0000
IC H11  CZ   *NH1  H12       1.0000  120.00  180.00  120.00  1.0000
IC NE   CZ   NH2   H21       1.3650  120.00  180.00  120.00  1.0000
IC H21  CZ   *NH2  H22       1.0000  120.00  180.00  120.00  1.0000
DONO H2'  O2'
DONO H4   N4
DONO H3   N3
DONO H2   N2
DONO H11  NH1
DONO H12  NH1
DONO H21  NH2
DONO H22  NH2
DONO HE   NE
ACCE O1P  P
ACCE O2P  P
ACCE O2'
ACCE O3'
ACCE O4'
ACCE O5'

RESI 1PC           -1.00 !  1H-pseudoisocytositidine
GROUP
ATOM N1     NG2R61 -0.36 !              H21  H22
ATOM H1     HGP1    0.30 !                \  /
ATOM C2     CG2R64  0.64 !                 N2
ATOM N2     NG2S3  -0.66 !                 |
ATOM H21    HGP4    0.31 !                 C2
ATOM H22    HGP4    0.31 !                /  \\
ATOM N3     NG2R62 -0.77 !           H1-N1   N3
ATOM C4     CG2R63  0.53 !               |    |
ATOM O4     OG2D4  -0.47 !           H6-C6   C4
ATOM C5     CG2R62 -0.13 !                \\ / \\
ATOM C6     CG2R62  0.17 !                 C5   O4
ATOM H6     HGR62   0.13 !                  \
GROUP                    !                   \
ATOM P      P       1.50 !                    \
ATOM O1P    ON3    -0.78 !  O1P    H5' H4'  O4'  \
ATOM O2P    ON3    -0.78 !   |      |    \ /   \  \
ATOM O5'    ON2    -0.57 !  -P-O5'-C5'---C4'    C1'
ATOM C5'    CN8B   -0.08 !   |      |     \     / \
ATOM H5'    HN8     0.09 !  O2P    H5''   C3'--C2' H1'
ATOM H5''   HN8     0.09 !               / \   / \
GROUP                    !             O3' H3' O2' H2''
ATOM C1'    CN7B    0.12 !              |       |
ATOM H1'    HN7     0.09 !                     H2'
ATOM C4'    CN7     0.16
ATOM O4'    ON6B   -0.46
ATOM H4'    HN7     0.09
GROUP
ATOM C2'    CN7B    0.14
ATOM H2''   HN7     0.09
ATOM O2'    ON5    -0.66
ATOM H2'    HN5     0.43
GROUP
ATOM C3'    CN7     0.01
ATOM H3'    HN7     0.09
ATOM O3'    ON2    -0.57
BOND N1   C2   N1   H1   N1   C6   C2   N3
BOND C2   N2   N2  H21   N2  H22   N3   C4
BOND C4   O4   C4   C5   C5   C6   C6   H6
BOND P    O1P     P    O2P     P    O5'     O5'  C5'     C5'  H5''
```

```
BOND C5'  C4'      C4'  O4'      C4'  C3'      O4'  C1'
BOND C1'  C5       C1'  C2'      C2'  C3'      C3'  O3'      O3'  +P
BOND C2'  O2'      O2'  H2'
BOND C1'  H1'      C2'  H2''     C3'  H3'      C4'  H4'      C5'  H5'
IMPR C2   N1   N3   N2         N2   H22  H21  C2         C4   C5   N3   O4
!ribose
IC -O3' P    O5'   C5'    1.6001  101.45  -39.25  119.00  1.4401
IC -O3' O5'  *P    O1P    1.6001  101.45 -115.82  109.74  1.4802
IC -O3' O5'  *P    O2P    1.6001  101.45  115.90  109.80  1.4801
IC  P   O5'  C5'   C4'    1.5996  119.00 -151.39  110.04  1.5160
IC O5'  C5'  C4'   C3'    1.4401  108.83 -179.85  116.10  1.5284
IC C5'  C4'  C3'   O3'    1.5160  116.10   76.70  115.12  1.4212
IC C4'  C3'  O3'   +P     1.5284  111.92  159.13  119.05  1.6001
IC C3'  O3'  +P    +O5'   1.4212  119.05  -98.86  101.45  1.5996
IC O4'  C3'  *C4'  C5'    1.4572  104.06 -120.04  116.10  1.5160
IC C2'  C4'  *C3'  O3'    1.5284  100.16 -124.08  115.12  1.4212
IC C4'  C3'  C2'   C1'    1.5284  100.16   39.58  102.04  1.5251
IC C5   C1'  O4'   C4'    1.4022  102.36 -123.09  109.06  1.4572
IC C6   C5   C1'   C2'    1.3750  121.43  -77.12  118.79  1.5251
IC C1'  C3'  *C2'  O2'    1.5284  102.04 -114.67  110.81  1.4212
IC H2'  O2'  C2'   C3'    0.9600  114.97  148.63  111.92  1.5284
IC O4'  C2'  *C1'  H1'    0.0       0.0  -115.0     0.0   0.0
IC C1'  C3'  *C2'  H2''   0.0       0.0   115.0     0.0   0.0
IC C2'  C4'  *C3'  H3'    0.0       0.0   115.0     0.0   0.0
IC C3'  O4'  *C4'  H4'    0.0       0.0  -115.0     0.0   0.0
IC C4'  O5'  *C5'  H5'    0.0       0.0  -115.0     0.0   0.0
IC C4'  O5'  *C5'  H5''   0.0       0.0   115.0     0.0   0.0
IC C3'  C2'  C1'   C5     1.5284  101.97  144.39  113.71  1.4896
IC O4'  C1'  C5    C4     1.5251  113.71  -97.2   125.59  1.3783
IC C1'  C4   *C5   C6     0.0       0.0   180.0     0.0   0.0
IC C6   C5   C4    N3       1.3764  114.90    0.71  122.46  1.3695
IC C5   C4   N3    C2     0.0       0.0     0.0     0.0   0.0
IC C4   C5   C6    N1     0.0       0.0     0.0     0.0   0.0
IC N3   N1   *C2   N2       1.3695  122.46  179.37  120.39  1.3211
IC N1   C2   N2    H21      1.3381  120.39  179.95  123.72  0.9917
IC H21  C2   *N2   H22      0.9917  123.72 -179.64  114.92  0.9946
IC C4   C2   *N3   H3       1.3738  124.14  179.34  118.67  0.9958
IC C5   N3   *C4   O4       1.4446  114.99 -179.12  118.26  1.2288
IC C5   N1   *C6   H6       1.3805  126.24  178.28  115.02  1.0919
IC C2   C6   *N1   H1       1.3746  119.25 -179.82  124.25  1.0008
DONO H22  N2
DONO H21  N2
DONO H1   N1
ACCE N3
ACCE O4
ACCE O1P  P
ACCE O2P  P
ACCE O2'
ACCE O3'
ACCE O4'
ACCE O5'

RESI 3PC           -1.00 !  3H-pseudoisocytositidine
GROUP
ATOM N1     NG2R62 -0.78 !                 H21  H22
ATOM C2     CG2R64  0.63 !                   \  /
ATOM N2     NG2S3  -0.56 !                    N2
ATOM H21    HGP4    0.31 !                    |
ATOM H22    HGP4    0.31 !                    C2    H3
ATOM N3     NG2R61 -0.51 !                  //  \  /
ATOM H3     HGP1    0.35 !                  N1   N3
ATOM C4     CG2R63  0.46 !                  |     |
ATOM O4     OG2D4  -0.48 !               H6-C6   C4
```

```
ATOM C5      CG2R62 -0.16 !                    \\ / \\
ATOM C6      CG2R62  0.28 !                     C5   O4
ATOM H6      HGR62   0.15 !                      \
GROUP                     !                       \
ATOM P       P       1.50 !                        \
ATOM O1P     ON3    -0.78 !   O1P    H5' H4'  O4'  \
ATOM O2P     ON3    -0.78 !    |      |    \ /   \  \
ATOM O5'     ON2    -0.57 !   -P-O5'-C5'---C4'    C1'
ATOM C5'     CN8B   -0.08 !    |      |     \     / \
ATOM H5'     HN8     0.09 !   O2P    H5''   C3'--C2' H1'
ATOM H5''    HN8     0.09 !                 / \   / \
GROUP                     !              O3' H3' O2' H2''
ATOM C1'     CN7B    0.12 !               |       |
ATOM H1'     HN7     0.09 !                      H2'
ATOM C4'     CN7     0.16
ATOM O4'     ON6B   -0.46
ATOM H4'     HN7     0.09
GROUP
ATOM C2'     CN7B    0.14
ATOM H2''    HN7     0.09
ATOM O2'     ON5    -0.66
ATOM H2'     HN5     0.43
GROUP
ATOM C3'     CN7     0.01
ATOM H3'     HN7     0.09
ATOM O3'     ON2    -0.57
BOND N1   C2   N1   C6   C2   N2   C2   N3
BOND N2  H21   N2  H22   N3   H3   N3   C4
BOND C4   O4   C4   C5   C5   C6   C6   H6
BOND P    O1P     P    O2P     P    O5'     O5'  C5'     C5'  H5''
BOND C5'  C4'     C4'  O4'     C4'  C3'     O4'  C1'
BOND C1'  C5      C1'  C2'     C2'  C3'     C3'  O3'     O3'  +P
BOND C2'  O2'     O2'  H2'
BOND C1'  H1'     C2'  H2''    C3'  H3'     C4'  H4'     C5'  H5'
IMPR C2   N3   N1   N2        N2   H22  H21  C2        C4   C5   N3   O4
!ribose
IC -O3' P    O5'   C5'    1.6001  101.45  -39.25  119.00  1.4401
IC -O3' O5'  *P    O1P    1.6001  101.45 -115.82  109.74  1.4802
IC -O3' O5'  *P    O2P    1.6001  101.45  115.90  109.80  1.4801
IC  P   O5'  C5'   C4'    1.5996  119.00 -151.39  110.04  1.5160
IC O5'  C5'  C4'   C3'    1.4401  108.83 -179.85  116.10  1.5284
IC C5'  C4'  C3'   O3'    1.5160  116.10   76.70  115.12  1.4212
IC C4'  C3'  O3'   +P     1.5284  111.92  159.13  119.05  1.6001
IC C3'  O3'  +P    +O5'   1.4212  119.05  -98.86  101.45  1.5996
IC O4'  C3'  *C4'  C5'    1.4572  104.06 -120.04  116.10  1.5160
IC C2'  C4'  *C3'  O3'    1.5284  100.16 -124.08  115.12  1.4212
IC C4'  C3'  C2'   C1'    1.5284  100.16   39.58  102.04  1.5251
IC C5   C1'  O4'   C4'    1.4022  102.36 -123.09  109.06  1.4572
IC C6   C5   C1'   C2'    1.3750  121.43  -77.12  118.79  1.5251
IC C1'  C3'  *C2'  O2'    1.5284  102.04 -114.67  110.81  1.4212
IC H2'  O2'  C2'   C3'    0.9600  114.97  148.63  111.92  1.5284
IC O4'  C2'  *C1'  H1'    0.0       0.0  -115.0     0.0   0.0
IC C1'  C3'  *C2'  H2''   0.0       0.0   115.0     0.0   0.0
IC C2'  C4'  *C3'  H3'    0.0       0.0   115.0     0.0   0.0
IC C3'  O4'  *C4'  H4'    0.0       0.0  -115.0     0.0   0.0
IC C4'  O5'  *C5'  H5'    0.0       0.0  -115.0     0.0   0.0
IC C4'  O5'  *C5'  H5''   0.0       0.0   115.0     0.0   0.0
IC C3'  C2'  C1'   C5     1.5284  101.97  144.39  113.71  1.4896
IC O4'  C1'  C5    C4     1.5251  113.71  -97.2   125.59  1.3783
IC C1'  C4   *C5   C6     0.0       0.0   180.0     0.0   0.0
IC C6   C5   C4    N3       1.3764  114.90    0.71  122.46  1.3695
IC C5   C4   N3    C2     0.0       0.0     0.0     0.0   0.0
IC C4   C5   C6    N1     0.0       0.0     0.0     0.0   0.0
```

```
IC N3   N1   *C2   N2        1.3695  122.46  179.37  120.39  1.3211
IC N1   C2   N2    H21       1.3381  120.39  179.95  123.72  0.9917
IC H21  C2   *N2   H22       0.9917  123.72 -179.64  114.92  0.9946
IC C4   C2   *N3   H3        1.3738  124.14  179.34  118.67  0.9958
IC C5   N3   *C4   O4        1.4446  114.99 -179.12  118.26  1.2288
IC C5   N1   *C6   H6        1.3805  126.24  178.28  115.02  1.0919
DONO H22  N2
DONO H21  N2
DONO H3   N3
ACCE N1
ACCE O4
ACCE O1P  P
ACCE O2P  P
ACCE O2'
ACCE O3'
ACCE O4'
ACCE O5'

!!***Adenosine***
RESI OMA           -1.00 ! 2'-O-methyladenosine, MRA
GROUP
ATOM N9     NN2    -0.05 !       H61  H62
ATOM C8     CN4     0.34 !         \  /
ATOM H8     HN3     0.12 !          N6
ATOM N7     NN4    -0.71 !          |
ATOM C5     CN5     0.28 !          C6
ATOM C6     CN2     0.46 !        //  \
ATOM N6     NN1    -0.77 !        N1   C5--N7\\
ATOM H61    HN1     0.38 !        |    ||     C8-H8
ATOM H62    HN1     0.38 !        C2   C4--N9/
ATOM N1     NN3A   -0.74 !       / \\ /     \
ATOM C2     CN4     0.50 !     H2   N3       \
ATOM H2     HN3     0.13 !                    \
ATOM N3     NN3A   -0.75 !                     \
ATOM C4     CN5     0.43 !                      \
GROUP                    !  O1P    H5' H4'  O4'  \
ATOM P      P       1.50 !   |      |    \ /   \  \
ATOM O1P    ON3    -0.78 !  -P-O5'-C5'---C4'    C1'
ATOM O2P    ON3    -0.78 !   |      |     \     / \
ATOM O5'    ON2    -0.57 !  O2P    H5''   C3'--C2' H1'
ATOM C5'    CN8B   -0.08 !               / \   / \
ATOM H5'    HN8     0.09 !            O3' H3' O2' H2''
ATOM H5''   HN8     0.09 !             |       |
GROUP                    !                    CM2
ATOM C4'    CN7     0.16 !                  /  |  \
ATOM H4'    HN7     0.09 !                HM1 HM2 HM3
ATOM O4'    ON6B   -0.50
ATOM C1'    CN7B    0.16 !  !!!! PATCH ADEP for the protonated base
ATOM H1'    HN7     0.09 !  !!!! PATCH ADEI for the imine tautomer
GROUP
ATOM C2'    CN7B    0.08
ATOM H2''   HN7     0.09
ATOM O2'    OG301  -0.34
ATOM CM2    CG331  -0.10
ATOM HM1    HGA3    0.09
ATOM HM2    HGA3    0.09
ATOM HM3    HGA3    0.09
GROUP
ATOM C3'    CN7     0.01
ATOM H3'    HN7     0.09
ATOM O3'    ON2    -0.57
BOND N9   C8      N9   C4      C4   N3      C4   C5      N1   C6
BOND C2   N1      C6   N6      C8   H8      C2   H2      C2   N3
```

```
BOND N6   H61     N6   H62     C6   C5      C5   N7      N7   C8
BOND P    O1P     P    O2P     P    O5'     O5'  C5'     C5'  H5''
BOND C5'  C4'     C4'  O4'     C4'  C3'     O4'  C1'
BOND C1'  N9      C1'  C2'     C2'  C3'     C3'  O3'     O3'  +P
BOND C2'  O2'     CM2  O2'     CM2  HM1     HM2  CM2     HM3  CM2
BOND C1'  H1'     C2'  H2''    C3'  H3'     C4'  H4'     C5'  H5'
IMPR N6   C6   H61  H62        C6   N1   C5   N6
!2OM-ribose
IC -O3' P    O5'  C5'    1.6001  101.45  -39.25  119.00  1.4401
IC -O3' O5'  *P   O1P    1.6001  101.45 -115.82  109.74  1.4802
IC -O3' O5'  *P   O2P    1.6001  101.45  115.90  109.80  1.4801
IC  P   O5'  C5'  C4'    1.5996  119.00 -151.39  110.04  1.5160
IC O5'  C5'  C4'  C3'    1.4401  108.83 -179.85  116.10  1.5284
IC C5'  C4'  C3'  O3'    1.5160  116.10   76.70  115.12  1.4212
IC C4'  C3'  O3'  +P     1.5284  111.92  159.13  119.05  1.6001
IC C3'  O3'  +P   +O5'   1.4212  119.05  -98.86  101.45  1.5996
IC O4'  C3'  *C4' C5'    1.4572  104.06 -120.04  116.10  1.5160
IC C2'  C4'  *C3' O3'    1.5284  100.16 -124.08  115.12  1.4212
IC C4'  C3'  C2'  C1'    1.5284  100.16   39.58  102.04  1.5251
IC C3'  C2'  C1'  N9     1.5284  101.97  144.39  113.71  1.4896
IC O4'  C1'  N9   C4     1.5251  113.71  -97.2   125.59  1.3783
IC C3'  C1'  *C2' O2'    1.5312  102.03  117.61  107.13  1.4206
IC C1'  C2'  O2'  CM2    1.5393  107.13   90.00  107.00  1.4150
IC C2'  O2'  CM2  HM2    1.4206  107.00  180.00    0.0   0.0
IC HM2  O2'  *CM2 HM3    0.0       0.0   120.00    0.0   0.0
IC HM2  O2'  *CM2 HM1    0.0       0.0  -120.00    0.0   0.0
IC O4'  C2'  *C1' H1'    0.0       0.0  -115.0     0.0   0.0
IC C1'  C3'  *C2' H2''   0.0       0.0   115.0     0.0   0.0
IC C2'  C4'  *C3' H3'    0.0       0.0   115.0     0.0   0.0
IC C3'  O4'  *C4' H4'    0.0       0.0  -115.0     0.0   0.0
IC C4'  O5'  *C5' H5'    0.0       0.0  -115.0     0.0   0.0
IC C4'  O5'  *C5' H5''   0.0       0.0   115.0     0.0   0.0
IC C8   C4   *N9  C1'       1.3791  105.54 -179.95  126.56  1.4896
IC C4   N9   C8   N7        1.3827  105.54    0.01  113.96  1.3083
IC N7   N9   *C8  H8        1.3083  113.96 -179.96  121.22  1.0920
IC C8   N9   C4   N3        1.3791  105.54 -179.95  127.55  1.3434
IC N3   N9   *C4  C5        1.3434  127.55  179.93  105.67  1.3846
IC N7   C4   *C5  C6        1.3900  110.87 -179.98  116.88  1.4099
IC C4   C5   C6   N1        1.3846  116.88   -0.04  117.83  1.3540
IC N1   C5   *C6  N6        1.3540  117.83 -179.93  123.47  1.3450
IC C5   C6   N6   H61       1.4099  123.47  179.96  117.54  0.9954
IC H61  C6   *N6  H62       0.9954  117.54  179.98  121.22  0.9971
IC C5   C6   N1   C2        1.4099  117.83    0.08  118.36  1.3351
IC N3   N1   *C2  H2        1.3357  129.49 -179.97  115.25  1.0944
DONO H61  N6
DONO H62  N6
ACCE N3
ACCE N7
ACCE N1
ACCE O1P  P
ACCE O2P  P
ACCE O2'
ACCE O3'
ACCE O4'
ACCE O5'

RESI RIA          -2.00 ! 2'-O-ribosyladenosine(phosphate), 2RA
GROUP
ATOM N9     NN2    -0.05 !           H61  H62
ATOM C8     CN4     0.34 !             \  /
ATOM H8     HN3     0.12 !              N6
ATOM N7     NN4    -0.71 !              |
ATOM C5     CN5     0.28 !              C6
```

```
ATOM C6     CN2      0.46 !             //  \
ATOM N6     NN1     -0.77 !             N1   C5--N7\\
ATOM H61    HN1      0.38 !             |    ||      C8-H8
ATOM H62    HN1      0.38 !             C2   C4--N9/
ATOM N1     NN3A    -0.74 !            / \\ /     \
ATOM C2     CN4      0.50 !          H2   N3        \
ATOM H2     HN3      0.13 !                          \
ATOM N3     NN3A    -0.75 !                           \
ATOM C4     CN5      0.43 !                            \
GROUP                     !       O1P    H5' H4'  O4'   \
ATOM P      P        1.50 !        |      |    \ /   \   \
ATOM O1P    ON3     -0.78 !       -P-O5' C5' --C4'    C1'
ATOM O2P    ON3     -0.78 !        |      |     \     / \
ATOM O5'    ON2     -0.57 !       O2P    H5''   C3' -C2' H1'
ATOM C5'    CN8B    -0.08 !                     / \   / \
ATOM H5'    HN8      0.09 !                  O3' H3' O2' H2'
ATOM H5''   HN8      0.09 !                   |       \
GROUP                     !                            \
ATOM C4'    CN7      0.16 !                             \
ATOM H4'    HN7      0.09 !         O3X    H5A H4A  O4A  \
ATOM O4'    ON6B    -0.50 !         ||      |    \ /   \  \
ATOM C1'    CN7B     0.16 ! HX -O1X-PA-O5A-C5A---C4A    C1A
ATOM H1'    HN7      0.09 !          |      |     \     / \
GROUP                     !      (-)O2X    H5AA   C3A--C2A H1A
ATOM C3'    CN7      0.01 !                      / \   / \
ATOM H3'    HN7      0.09 !                   O3A H3A O2A H2A
ATOM O3'    ON2     -0.57 !                    |       |
GROUP                     !                  H3OA    H2OA
ATOM C2'    CN7B     0.09
ATOM H2'    HN7      0.09
ATOM O2'    OC301   -0.36
ATOM C1A    CC3152   0.29
ATOM H1A    HCA1     0.09
ATOM O4A    OC3C51  -0.40
ATOM C4A    CC3153   0.11
ATOM H4A    HCA1     0.09
GROUP
ATOM C2A    CC3151   0.14
ATOM H2A    HCA1     0.09
ATOM O2A    OC311   -0.65
ATOM H2OA   HCP1     0.42
GROUP
ATOM C3A    CC3151   0.14
ATOM H3A    HCA1     0.09
ATOM O3A    OC311   -0.65
ATOM H3OA   HCP1     0.42
GROUP
ATOM C5A    CC321   -0.08
ATOM H51A   HCA2     0.09
ATOM H52A   HCA2     0.09
ATOM O5A    OC30P   -0.62
ATOM PA     PC       1.50
ATOM O1X    OC312   -0.68
ATOM HX     HCP1     0.34
ATOM O2X    OC2DP   -0.82
ATOM O3X    OC2DP   -0.82
BOND N1   C6      C2   N3      C4   C5      N7   C8      N9   C4
BOND C2   N1      C6   N6      N9   C8      C4   N3      N6   H61
BOND N6   H62     C6   C5      C5   N7      C8   H8      C2   H2
BOND P    O1P     P    O2P     P    O5'     O5'  C5'     C5'  H5''
BOND C5'  C4'     C4'  O4'     C4'  C3'     O4'  C1'
BOND C1'  N9      C1'  C2'     C2'  C3'     C3'  O3'     O3'  +P
BOND C2'  O2'     O2'  C1A
```

```
BOND C1'  H1'     C2'  H2'     C3'  H3'     C4'  H4'     C5'  H5'
BOND HX   O1X     O1X  PA      H51A C5A     H52A C5A     C5A  O5A
BOND C5A  C4A     PA   O5A     PA   O3X     PA   O2X     H4A  C4A
BOND H3A  C3A     C4A  C3A     C4A  O4A     O3A  C3A     O3A  H3OA
BOND C3A  C2A     H2OA O2A     O4A  C1A     C2A  O2A     C2A  C1A
BOND C2A  H2A     C1A  H1A
IMPR N6   C6   H61  H62        C6   N1   C5   N6
!ribose
IC -O3' P    O5'   C5'    1.6001  101.45  -39.25  119.00  1.4401
IC -O3' O5'  *P    O1P    1.6001  101.45 -115.82  109.74  1.4802
IC -O3' O5'  *P    O2P    1.6001  101.45  115.90  109.80  1.4801
IC  P   O5'  C5'   C4'    1.5996  119.00 -151.39  110.04  1.5160
IC O5'  C5'  C4'   C3'    1.4401  108.83 -179.85  116.10  1.5284
IC C5'  C4'  C3'   O3'    1.5160  116.10   76.70  115.12  1.4212
IC C4'  C3'  O3'   +P     1.5284  111.92  159.13  119.05  1.6001
IC C3'  O3'  +P    +O5'   1.4212  119.05  -98.86  101.45  1.5996
IC O4'  C3'  *C4'  C5'    1.4572  104.06 -120.04  116.10  1.5160
IC C2'  C4'  *C3'  O3'    1.5284  100.16 -124.08  115.12  1.4212
IC C4'  C3'  C2'   C1'    1.5284  100.16   39.58  102.04  1.5251
IC C3'  C2'  C1'   N9     1.5284  101.97  144.39  113.71  1.4896
IC O4'  C1'  N9    C4     1.5251  113.71  -97.2   125.59  1.3783
IC C1'  C3'  *C2'  O2'    1.5284  102.04 -114.67  110.81  1.4212
IC O4'  C2'  *C1'  H1'    0.0       0.0  -115.0     0.0   0.0
IC C1'  C3'  *C2'  H2'    0.0       0.0   115.0     0.0   0.0
IC C2'  C4'  *C3'  H3'    0.0       0.0   115.0     0.0   0.0
IC C3'  O4'  *C4'  H4'    0.0       0.0  -115.0     0.0   0.0
IC C4'  O5'  *C5'  H5'    0.0       0.0  -115.0     0.0   0.0
IC C4'  O5'  *C5'  H5''   0.0       0.0   115.0     0.0   0.0
IC C1'  C2'  O2'   C1A       1.5063  107.56  106.98  111.64  1.4216
IC C2'  O2'  C1A   O4A       1.4298  111.64  -68.42  110.78  1.4403
IC O4A  O2'  *C1A  C2A       1.4403  110.78 -113.45  103.46  1.5457
IC O4A  O2'  *C1A  H1A       1.4403  110.78 -120.00  108.50  1.1000
IC O2'  C1A  O4A   C4A       1.4216  110.78  -95.22  109.95  1.4369
IC C1A  O4A  C4A   C5A       1.4403  109.95  136.29  115.40  1.5401
IC C5A  O4A  *C4A  C3A       1.5401  115.40 -127.06  105.45  1.5322
IC C5A  O4A  *C4A  H4A       1.5401  115.40 -120.00  107.30  1.1000
IC C3A  C1A  *C2A  O2A       1.5383   99.33  118.90  113.47  1.4361
IC C3A  C1A  *C2A  H2A       1.5383   99.33 -120.00  111.40  1.1000
IC C1A  C2A  O2A   H2OA      1.5457  113.47  180.00  109.00  0.9600
IC C2A  C4A  *C3A  O3A       1.5383  102.85 -125.22  112.10  1.4320
IC O3A  C4A  *C3A  H3A       1.4320  112.10 -120.00  111.40  1.1000
IC C4A  C3A  O3A   H3OA      1.5322  112.10  180.00  109.00  0.9600
IC O4A  C4A  C5A   O5A       1.4369  115.40 -149.51  108.85  1.4355
IC O5A  C4A  *C5A  H51A      1.4355  108.85  120.00  110.10  1.1110
IC H51A C4A  *C5A  H52A      1.1110  110.10 -120.00  110.10  1.1110
IC C4A  C5A  O5A   PA        1.5401  108.85 -167.20  121.22  1.6095
IC C5A  O5A  PA    O1X       1.4355  121.22   75.05  103.55  1.5871
IC O1X  O5A  *PA   O3X       1.5871  103.55  116.19  105.43  1.4997
IC O1X  O5A  *PA   O2X       1.5871  103.55 -113.52  107.57  1.5078
IC O5A  PA   O1X   HX        1.6095  103.55  180.00  115.00  0.9600
IC C8   C4   *N9   C1'       1.3791  105.54 -179.95  126.56  1.4896
IC C4   N9   C8    N7        1.3827  105.54    0.01  113.96  1.3083
IC N7   N9   *C8   H8        1.3083  113.96 -179.96  121.22  1.0920
IC C8   N9   C4    N3        1.3791  105.54 -179.95  127.55  1.3434
IC N3   N9   *C4   C5        1.3434  127.55  179.93  105.67  1.3846
IC N7   C4   *C5   C6        1.3900  110.87 -179.98  116.88  1.4099
IC C4   C5   C6    N1        1.3846  116.88   -0.04  117.83  1.3540
IC N1   C5   *C6   N6        1.3540  117.83 -179.93  123.47  1.3450
IC C5   C6   N6    H61       1.4099  123.47  179.96  117.54  0.9954
IC H61  C6   *N6   H62       0.9954  117.54  179.98  121.22  0.9971
IC C5   C6   N1    C2        1.4099  117.83    0.08  118.36  1.3351
IC N3   N1   *C2   H2        1.3357  129.49 -179.97  115.25  1.0944
DONO H2OA  O2A
```

```
DONO H3OA  O3A
DONO HX    O1X
DONO H61  N6
DONO H62  N6
ACCE N3
ACCE N7
ACCE N1
ACCE O1P  P
ACCE O2P  P
ACCE O2'
ACCE O3'
ACCE O4'
ACCE O5'
ACCE O4A
ACCE O2A
ACCE O3A
ACCE O1X
ACCE O2X
ACCE O3X  PA
ACCE O5A

RESI 6MA           -1.00 ! N6-methyladenosine
GROUP
ATOM N9     NG2R51 -0.01 !        H61
ATOM C8     CG2R53  0.43 !          \
ATOM H8     HGR52   0.08 !      H62-CM6   H6
ATOM N7     NG2R50 -0.85 !           / \  /
ATOM C5     CG2RC0  0.32 !        H63   N6
ATOM C6     CG2R64  0.45 !              |
ATOM N6     NG311  -0.45 !              C6
ATOM H6     HGPAM1  0.33 !             // \
ATOM N1     NG2R62 -0.78 !            N1   C5--N7\\
ATOM C2     CG2R64  0.49 !            |    ||     C8-H8
ATOM H2     HGR62   0.14 !            C2   C4--N9/
ATOM N3     NG2R62 -0.86 !           / \\ /     \
ATOM C4     CG2RC0  0.52 !         H2   N3       \
ATOM CM6    CG331  -0.08 !                        \
ATOM H61    HGA3    0.09 !                         \
ATOM H62    HGA3    0.09 !     O1P    H5' H4'  O4'  \
ATOM H63    HGA3    0.09 !      |      |    \ /   \  \
GROUP                    !     -P-O5'-C5'---C4'    C1'
ATOM P      P       1.50 !      |      |     \     / \
ATOM O1P    ON3    -0.78 !     O2P    H5''   C3'--C2' H1'
ATOM O2P    ON3    -0.78 !                  / \   / \
ATOM O5'    ON2    -0.57 !                O3' H3' O2' H2''
ATOM C5'    CN8B   -0.08 !                 |       |
ATOM H5'    HN8     0.09 !                         H2'
ATOM H5''   HN8     0.09
GROUP
ATOM C4'    CN7     0.16
ATOM H4'    HN7     0.09
ATOM O4'    ON6B   -0.50
ATOM C1'    CN7B    0.16
ATOM H1'    HN7     0.09
GROUP
ATOM C2'    CN7B    0.14
ATOM H2''   HN7     0.09
ATOM O2'    ON5    -0.66
ATOM H2'    HN5     0.43
GROUP
ATOM C3'    CN7     0.01
ATOM H3'    HN7     0.09
ATOM O3'    ON2    -0.57
```

```
BOND N9   C8   N9   C4   C8   N7   C8   H8
BOND N7   C5   C5   C6   C5   C4   C6   N6
BOND C6   N1   N6   CM6  N6   H6   N1   C2
BOND C2   N3   C2   H2   N3   C4   CM6  H61
BOND CM6  H62  CM6  H63
BOND P    O1P     P    O2P     P    O5'     O5'  C5'     C5'  H5''
BOND C5'  C4'     C4'  O4'     C4'  C3'     O4'  C1'
BOND C1'  N9      C1'  C2'     C2'  C3'     C3'  O3'     O3'  +P
BOND C2'  O2'     O2'  H2'
BOND C1'  H1'     C2'  H2''    C3'  H3'     C4'  H4'     C5'  H5'
IMPR C6   C5   N1   N6       ! N6   C6   CM6   H6
!ribose
IC -O3' P    O5'   C5'    1.6001  101.45  -39.25  119.00  1.4401
IC -O3' O5'  *P    O1P    1.6001  101.45 -115.82  109.74  1.4802
IC -O3' O5'  *P    O2P    1.6001  101.45  115.90  109.80  1.4801
IC  P   O5'  C5'   C4'    1.5996  119.00 -151.39  110.04  1.5160
IC O5'  C5'  C4'   C3'    1.4401  108.83 -179.85  116.10  1.5284
IC C5'  C4'  C3'   O3'    1.5160  116.10   76.70  115.12  1.4212
IC C4'  C3'  O3'   +P     1.5284  111.92  159.13  119.05  1.6001
IC C3'  O3'  +P    +O5'   1.4212  119.05  -98.86  101.45  1.5996
IC O4'  C3'  *C4'  C5'    1.4572  104.06 -120.04  116.10  1.5160
IC C2'  C4'  *C3'  O3'    1.5284  100.16 -124.08  115.12  1.4212
IC C4'  C3'  C2'   C1'    1.5284  100.16   39.58  102.04  1.5251
IC C3'  C2'  C1'   N9     1.5284  101.97  144.39  113.71  1.4896
IC O4'  C1'  N9    C4     1.5251  113.71  -97.2   125.59  1.3783
IC C1'  C3'  *C2'  O2'    1.5284  102.04 -114.67  110.81  1.4212
IC H2'  O2'  C2'   C3'    0.9600  114.97  148.63  111.92  1.5284
IC O4'  C2'  *C1'  H1'    0.0       0.0  -115.0     0.0   0.0
IC C1'  C3'  *C2'  H2''   0.0       0.0   115.0     0.0   0.0
IC C2'  C4'  *C3'  H3'    0.0       0.0   115.0     0.0   0.0
IC C3'  O4'  *C4'  H4'    0.0       0.0  -115.0     0.0   0.0
IC C4'  O5'  *C5'  H5'    0.0       0.0  -115.0     0.0   0.0
IC C4'  O5'  *C5'  H5''   0.0       0.0   115.0     0.0   0.0
IC C8   C4   *N9   C1'       1.3791  105.54 -179.95  126.56  1.4896
IC C4   N9   C8    N7        1.3497  107.34    0.02  112.90  1.3249
IC N7   N9   *C8   H8        1.3249  112.90 -179.86  122.61  1.0947
IC N9   C8   N7    C5        1.3736  112.90    0.40  103.62  1.3989
IC C4   N7   *C5   C6        1.3954  110.25 -178.17  133.05  1.4051
IC N7   C5   C6    N1        1.3989  133.05  177.05  117.60  1.3793
IC N1   C5   *C6   N6        1.3793  117.60  179.99  122.23  1.3815
IC C5   C6   N6    CM6       1.4051  122.23  167.61  118.33  1.4832
IC CM6  C6   *N6   H6        1.4832  118.33  180.00  112.50  1.0190
IC C5   C6   N1    C2        1.4051  117.60    1.54  120.04  1.3647
IC C6   N1   C2    N3        1.3793  120.04   -1.14  125.07  1.3600
IC N3   N1   *C2   H2        1.3600  125.07 -179.44  117.43  1.0938
IC C6   N6   CM6   H61       1.3815  118.33  174.59  110.48  1.1123
IC H61  N6   *CM6  H62       1.1123  110.48  120.34  111.22  1.1126
IC H61  N6   *CM6  H63       1.1123  110.48 -119.06  111.26  1.1131
DONO H2'  O2'
DONO H6   N6
ACCE N3
ACCE N7
ACCE N1
ACCE O1P  P
ACCE O2P  P
ACCE O2'
ACCE O3'
ACCE O4'
ACCE O5'

RESI MMA           -1.00 ! N6,2'-O-dimethyladenosine
GROUP
ATOM N9     NG2R51 -0.01 !        H61
```

```
ATOM C8      CG2R53  0.43 !          \
ATOM H8      HGR52   0.08 !      H62-CM6   H6
ATOM N7      NG2R50 -0.85 !           / \  /
ATOM C5      CG2RC0  0.32 !        H63   N6
ATOM C6      CG2R64  0.45 !              |
ATOM N6      NG311  -0.45 !              C6
ATOM H6      HGPAM1  0.33 !             // \
ATOM N1      NG2R62 -0.78 !            N1   C5--N7\\
ATOM C2      CG2R64  0.49 !            |    ||      C8-H8
ATOM H2      HGR62   0.14 !            C2   C4--N9/
ATOM N3      NG2R62 -0.86 !           / \\ /     \
ATOM C4      CG2RC0  0.52 !         H2   N3        \
ATOM CM6     CG331  -0.08 !                         \
ATOM H61     HGA3    0.09 !                          \
ATOM H62     HGA3    0.09 !     O1P    H5' H4'  O4'  \
ATOM H63     HGA3    0.09 !      |      |    \ /   \  \
GROUP                     !     -P-O5'-C5'---C4'    C1'
ATOM P       P       1.50 !      |      |     \     / \
ATOM O1P     ON3    -0.78 !     O2P    H5''   C3'--C2' H1'
ATOM O2P     ON3    -0.78 !                  / \   / \
ATOM O5'     ON2    -0.57 !                O3' H3' O2' H2''
ATOM C5'     CN8B   -0.08 !                 |       |
ATOM H5'     HN8     0.09 !                        CM2
ATOM H5''    HN8     0.09 !                      /  |  \
GROUP                     !                   HM1 HM2 HM3
ATOM C4'     CN7     0.16
ATOM H4'     HN7     0.09
ATOM O4'     ON6B   -0.50
ATOM C1'     CN7B    0.16
ATOM H1'     HN7     0.09
GROUP
ATOM C2'     CN7B    0.08
ATOM H2''    HN7     0.09
ATOM O2'     OG301  -0.34
ATOM CM2     CG331  -0.10
ATOM HM1     HGA3    0.09
ATOM HM2     HGA3    0.09
ATOM HM3     HGA3    0.09
GROUP
ATOM C3'     CN7     0.01
ATOM H3'     HN7     0.09
ATOM O3'     ON2    -0.57
BOND N9   C8   N9   C4   C8   N7   C8   H8
BOND N7   C5   C5   C6   C5   C4   C6   N6
BOND C6   N1   N6   CM6  N6   H6   N1   C2
BOND C2   N3   C2   H2   N3   C4   CM6  H61
BOND CM6  H62  CM6  H63
BOND P    O1P     P    O2P     P    O5'     O5'  C5'     C5'  H5''
BOND C5'  C4'     C4'  O4'     C4'  C3'     O4'  C1'
BOND C1'  N9      C1'  C2'     C2'  C3'     C3'  O3'     O3'  +P
BOND C2'  O2'     CM2  O2'     CM2  HM1     HM2  CM2     HM3  CM2
BOND C1'  H1'     C2'  H2''    C3'  H3'     C4'  H4'     C5'  H5'
IMPR C6   C5   N1   N6     ! N6   C6   CM6   H6
IC C8   C4   *N9   C1'       1.3791  105.54 -179.95  126.56  1.4896
IC C4   N9   C8    N7        1.3497  107.34    0.02  112.90  1.3249
IC N7   N9   *C8   H8        1.3249  112.90 -179.86  122.61  1.0947
IC N9   C8   N7    C5        1.3736  112.90    0.40  103.62  1.3989
IC C4   N7   *C5   C6        1.3954  110.25 -178.17  133.05  1.4051
IC N7   C5   C6    N1        1.3989  133.05  177.05  117.60  1.3793
IC N1   C5   *C6   N6        1.3793  117.60  179.99  122.23  1.3815
IC C5   C6   N6    CM6       1.4051  122.23  167.61  118.33  1.4832
IC CM6  C6   *N6   H6        1.4832  118.33  180.00  112.50  1.0190
IC C5   C6   N1    C2        1.4051  117.60    1.54  120.04  1.3647
```

```
IC C6   N1   C2    N3        1.3793  120.04   -1.14  125.07  1.3600
IC N3   N1   *C2   H2        1.3600  125.07 -179.44  117.43  1.0938
IC C6   N6   CM6   H61       1.3815  118.33  174.59  110.48  1.1123
IC H61  N6   *CM6  H62       1.1123  110.48  120.34  111.22  1.1126
IC H61  N6   *CM6  H63       1.1123  110.48 -119.06  111.26  1.1131
!2OM-ribose
IC -O3' P    O5'   C5'    1.6001  101.45  -39.25  119.00  1.4401
IC -O3' O5'  *P    O1P    1.6001  101.45 -115.82  109.74  1.4802
IC -O3' O5'  *P    O2P    1.6001  101.45  115.90  109.80  1.4801
IC  P   O5'  C5'   C4'    1.5996  119.00 -151.39  110.04  1.5160
IC O5'  C5'  C4'   C3'    1.4401  108.83 -179.85  116.10  1.5284
IC C5'  C4'  C3'   O3'    1.5160  116.10   76.70  115.12  1.4212
IC C4'  C3'  O3'   +P     1.5284  111.92  159.13  119.05  1.6001
IC C3'  O3'  +P    +O5'   1.4212  119.05  -98.86  101.45  1.5996
IC O4'  C3'  *C4'  C5'    1.4572  104.06 -120.04  116.10  1.5160
IC C2'  C4'  *C3'  O3'    1.5284  100.16 -124.08  115.12  1.4212
IC C4'  C3'  C2'   C1'    1.5284  100.16   39.58  102.04  1.5251
IC C3'  C2'  C1'   N9     1.5284  101.97  144.39  113.71  1.4896
IC O4'  C1'  N9    C4     1.5251  113.71  -97.2   125.59  1.3783
IC C3'  C1'  *C2'  O2'    1.5312  102.03  117.61  107.13  1.4206
IC C1'  C2'  O2'   CM2    1.5393  107.13   90.00  107.00  1.4150
IC C2'  O2'  CM2   HM2    1.4206  107.00  180.00    0.0   0.0
IC HM2  O2'  *CM2  HM3    0.0       0.0   120.00    0.0   0.0
IC HM2  O2'  *CM2  HM1    0.0       0.0  -120.00    0.0   0.0
IC O4'  C2'  *C1'  H1'    0.0       0.0  -115.0     0.0   0.0
IC C1'  C3'  *C2'  H2''   0.0       0.0   115.0     0.0   0.0
IC C2'  C4'  *C3'  H3'    0.0       0.0   115.0     0.0   0.0
IC C3'  O4'  *C4'  H4'    0.0       0.0  -115.0     0.0   0.0
IC C4'  O5'  *C5'  H5'    0.0       0.0  -115.0     0.0   0.0
IC C4'  O5'  *C5'  H5''   0.0       0.0   115.0     0.0   0.0
DONO H6   N6
ACCE N3
ACCE N7
ACCE N1
ACCE O1P  P
ACCE O2P  P
ACCE O2'
ACCE O3'
ACCE O4'
ACCE O5'

RESI M6A           -1.00 ! N6,N6-dimethyladenosine, MA6, DMA
GROUP
ATOM N9     NG2R51 -0.01 !     H91        H101
ATOM C8     CG2R53  0.31 !        \       /
ATOM H8     HGR52   0.09 !    H92 -C9   C10-H102
ATOM N7     NG2R50 -0.67 !          / \  / \
ATOM C5     CG2RC0  0.41 !      H93   N6  H103
ATOM C6     CG2R64  0.52 !            |
ATOM N6     NG301  -0.53 !            C6
ATOM N1     NG2R62 -0.69 !           // \
ATOM C2     CG2R64  0.57 !          N1   C5--N7\\
ATOM H2     HGR62   0.08 !          |    ||     C8-H8
ATOM N3     NG2R62 -0.87 !          C2   C4--N9/
ATOM C4     CG2RC0  0.43 !         / \\ /     \
ATOM C9     CG331  -0.09 !       H2   N3       \
ATOM H91    HGA3    0.09 !                      \
ATOM H92    HGA3    0.09 !                       \
ATOM H93    HGA3    0.09 !   O1P    H5' H4'  O4'  \
ATOM C10    CG331  -0.09 !    |      |    \ /   \  \
ATOM H101   HGA3    0.09 !   -P-O5'-C5'---C4'    C1'
ATOM H102   HGA3    0.09 !    |      |     \     / \
ATOM H103   HGA3    0.09 !   O2P    H5''   C3'--C2' H1'
```

```
GROUP                    !                / \   / \
ATOM P      P       1.50 !              O3' H3' O2' H2''
ATOM O1P    ON3    -0.78 !               |       |
ATOM O2P    ON3    -0.78 !                      H2'
ATOM O5'    ON2    -0.57
ATOM C5'    CN8B   -0.08
ATOM H5'    HN8     0.09
ATOM H5''   HN8     0.09
GROUP
ATOM C4'    CN7     0.16
ATOM H4'    HN7     0.09
ATOM O4'    ON6B   -0.50
ATOM C1'    CN7B    0.16
ATOM H1'    HN7     0.09
GROUP
ATOM C2'    CN7B    0.14
ATOM H2''   HN7     0.09
ATOM O2'    ON5    -0.66
ATOM H2'    HN5     0.43
GROUP
ATOM C3'    CN7     0.01
ATOM H3'    HN7     0.09
ATOM O3'    ON2    -0.57
BOND N9   C8   N9   C4   C8   N7   C8   H8
BOND N7   C5   C5   C6   C5   C4   C6   N6
BOND C6   N1   N6   C10  N6   C9   N1   C2
BOND C2   N3   C2   H2   N3   C4   C10  H101
BOND C10  H102 C10  H103 C9   H91  C9   H92
BOND C9   H93
BOND P    O1P     P    O2P     P    O5'     O5'  C5'     C5'  H5''
BOND C5'  C4'     C4'  O4'     C4'  C3'     O4'  C1'
BOND C1'  N9      C1'  C2'     C2'  C3'     C3'  O3'     O3'  +P
BOND C2'  O2'     O2'  H2'
BOND C1'  H1'     C2'  H2''    C3'  H3'     C4'  H4'     C5'  H5'
IMPR C6   C5   N1   N6
IC C8   C4   *N9   C1'       1.3791  105.54 -179.95  126.56  1.4896
IC C4   N9   C8    N7        1.3481  107.42    0.06  112.86  1.3228
IC N7   N9   *C8   H8        1.3228  112.86 -179.98  122.61  1.0942
IC N9   C8   N7    C5        1.3690  112.86   -0.02  104.04  1.4051
IC C4   N7   *C5   C6        1.3980  109.37 -179.79  134.11  1.4169
IC N7   C5   C6    N6        1.4051  134.11    1.61  126.00  1.3858
IC N6   C5   *C6   N1        1.3858  126.00  176.22  116.71  1.3854
IC C5   C6   N1    C2        1.4169  116.71    2.37  120.71  1.3602
IC C6   N1   C2    N3        1.3854  120.71   -1.40  125.03  1.3553
IC N3   N1   *C2   H2        1.3553  125.03 -179.35  117.49  1.0936
IC C5   C6   N6    C10       1.4169  126.00  168.70  120.09  1.4623
IC C10  C6   *N6   C9        1.4623  120.09  172.97  121.21  1.4593
IC C6   N6   C10   H101      1.3858  120.09 -173.59  111.47  1.1119
IC H101 N6   *C10  H102      1.1119  111.47  119.69  111.25  1.1136
IC H101 N6   *C10  H103      1.1119  111.47 -120.14  111.01  1.1134
IC C6   N6   C9    H91       1.3858  121.21 -166.44  111.62  1.1123
IC H91  N6   *C9   H92       1.1123  111.62  119.92  111.52  1.1145
IC H91  N6   *C9   H93       1.1123  111.62 -119.99  110.95  1.1141
!ribose
IC -O3' P    O5'   C5'    1.6001  101.45  -39.25  119.00  1.4401
IC -O3' O5'  *P    O1P    1.6001  101.45 -115.82  109.74  1.4802
IC -O3' O5'  *P    O2P    1.6001  101.45  115.90  109.80  1.4801
IC  P   O5'  C5'   C4'    1.5996  119.00 -151.39  110.04  1.5160
IC O5'  C5'  C4'   C3'    1.4401  108.83 -179.85  116.10  1.5284
IC C5'  C4'  C3'   O3'    1.5160  116.10   76.70  115.12  1.4212
IC C4'  C3'  O3'   +P     1.5284  111.92  159.13  119.05  1.6001
IC C3'  O3'  +P    +O5'   1.4212  119.05  -98.86  101.45  1.5996
IC O4'  C3'  *C4'  C5'    1.4572  104.06 -120.04  116.10  1.5160
```

```
IC C2'  C4'  *C3'  O3'    1.5284  100.16 -124.08  115.12  1.4212
IC C4'  C3'  C2'   C1'    1.5284  100.16   39.58  102.04  1.5251
IC C3'  C2'  C1'   N9     1.5284  101.97  144.39  113.71  1.4896
IC O4'  C1'  N9    C4     1.5251  113.71  -97.2   125.59  1.3783
IC C1'  C3'  *C2'  O2'    1.5284  102.04 -114.67  110.81  1.4212
IC H2'  O2'  C2'   C3'    0.9600  114.97  148.63  111.92  1.5284
IC O4'  C2'  *C1'  H1'    0.0       0.0  -115.0     0.0   0.0
IC C1'  C3'  *C2'  H2''   0.0       0.0   115.0     0.0   0.0
IC C2'  C4'  *C3'  H3'    0.0       0.0   115.0     0.0   0.0
IC C3'  O4'  *C4'  H4'    0.0       0.0  -115.0     0.0   0.0
IC C4'  O5'  *C5'  H5'    0.0       0.0  -115.0     0.0   0.0
IC C4'  O5'  *C5'  H5''   0.0       0.0   115.0     0.0   0.0
DONO H2'  O2'
ACCE N3
ACCE N7
ACCE N1
ACCE O1P  P
ACCE O2P  P
ACCE O2'
ACCE O3'
ACCE O4'
ACCE O5'

RESI MTA           -1.00 ! N6,N6,2'-O-trimethyladenosine
GROUP
ATOM N9     NG2R51 -0.01 !    H91        H101
ATOM C8     CG2R53  0.31 !       \       /
ATOM H8     HGR52   0.09 !   H92 -C9   C10-H102
ATOM N7     NG2R50 -0.67 !        / \  / \
ATOM C5     CG2RC0  0.41 !     H93   N6  H103
ATOM C6     CG2R64  0.52 !           |
ATOM N6     NG301  -0.53 !           C6
ATOM N1     NG2R62 -0.69 !          // \
ATOM C2     CG2R64  0.57 !         N1   C5--N7\\
ATOM H2     HGR62   0.08 !         |    ||     C8-H8
ATOM N3     NG2R62 -0.87 !         C2   C4--N9/
ATOM C4     CG2RC0  0.43 !        / \\ /     \
ATOM C9     CG331  -0.09 !      H2   N3       \
ATOM H91    HGA3    0.09 !                     \
ATOM H92    HGA3    0.09 !                      \
ATOM H93    HGA3    0.09 !  O1P    H5' H4'  O4'  \
ATOM C10    CG331  -0.09 !   |      |    \ /   \  \
ATOM H101   HGA3    0.09 !  -P-O5'-C5'---C4'    C1'
ATOM H102   HGA3    0.09 !   |      |     \     / \
ATOM H103   HGA3    0.09 !  O2P    H5''   C3'--C2' H1'
GROUP                    !               / \   / \
ATOM P      P       1.50 !             O3' H3' O2' H2''
ATOM O1P    ON3    -0.78 !              |       |
ATOM O2P    ON3    -0.78 !                     CM2
ATOM O5'    ON2    -0.57 !                    /  |  \
ATOM C5'    CN8B   -0.08 !                  HM1 HM2 HM3
ATOM H5'    HN8     0.09
ATOM H5''   HN8     0.09
GROUP
ATOM C4'    CN7     0.16
ATOM H4'    HN7     0.09
ATOM O4'    ON6B   -0.50
ATOM C1'    CN7B    0.16
ATOM H1'    HN7     0.09
GROUP
ATOM C2'    CN7B    0.08
ATOM H2''   HN7     0.09
ATOM O2'    OG301  -0.34
```

```
ATOM CM2    CG331  -0.10
ATOM HM1    HGA3    0.09
ATOM HM2    HGA3    0.09
ATOM HM3    HGA3    0.09
GROUP
ATOM C3'    CN7     0.01
ATOM H3'    HN7     0.09
ATOM O3'    ON2    -0.57
BOND N9   C8   N9   C4   C8   N7   C8   H8
BOND N7   C5   C5   C6   C5   C4   C6   N6
BOND C6   N1   N6   C10  N6   C9   N1   C2
BOND C2   N3   C2   H2   N3   C4   C10  H101
BOND C10  H102 C10  H103 C9   H91  C9   H92
BOND C9   H93
BOND P    O1P     P    O2P     P    O5'     O5'  C5'     C5'  H5''
BOND C5'  C4'     C4'  O4'     C4'  C3'     O4'  C1'
BOND C1'  N9      C1'  C2'     C2'  C3'     C3'  O3'     O3'  +P
BOND C2'  O2'     CM2  O2'     CM2  HM1     HM2  CM2     HM3  CM2
BOND C1'  H1'     C2'  H2''    C3'  H3'     C4'  H4'     C5'  H5'
IMPR C6   C5   N1   N6
IC C8   C4   *N9   C1'       1.3791  105.54 -179.95  126.56  1.4896
IC C4   N9   C8    N7        1.3481  107.42    0.06  112.86  1.3228
IC N7   N9   *C8   H8        1.3228  112.86 -179.98  122.61  1.0942
IC N9   C8   N7    C5        1.3690  112.86   -0.02  104.04  1.4051
IC C4   N7   *C5   C6        1.3980  109.37 -179.79  134.11  1.4169
IC N7   C5   C6    N6        1.4051  134.11    1.61  126.00  1.3858
IC N6   C5   *C6   N1        1.3858  126.00  176.22  116.71  1.3854
IC C5   C6   N1    C2        1.4169  116.71    2.37  120.71  1.3602
IC C6   N1   C2    N3        1.3854  120.71   -1.40  125.03  1.3553
IC N3   N1   *C2   H2        1.3553  125.03 -179.35  117.49  1.0936
IC C5   C6   N6    C10       1.4169  126.00  168.70  120.09  1.4623
IC C10  C6   *N6   C9        1.4623  120.09  172.97  121.21  1.4593
IC C6   N6   C10   H101      1.3858  120.09 -173.59  111.47  1.1119
IC H101 N6   *C10  H102      1.1119  111.47  119.69  111.25  1.1136
IC H101 N6   *C10  H103      1.1119  111.47 -120.14  111.01  1.1134
IC C6   N6   C9    H91       1.3858  121.21 -166.44  111.62  1.1123
IC H91  N6   *C9   H92       1.1123  111.62  119.92  111.52  1.1145
IC H91  N6   *C9   H93       1.1123  111.62 -119.99  110.95  1.1141
!2OM-ribose
IC -O3' P    O5'   C5'    1.6001  101.45  -39.25  119.00  1.4401
IC -O3' O5'  *P    O1P    1.6001  101.45 -115.82  109.74  1.4802
IC -O3' O5'  *P    O2P    1.6001  101.45  115.90  109.80  1.4801
IC  P   O5'  C5'   C4'    1.5996  119.00 -151.39  110.04  1.5160
IC O5'  C5'  C4'   C3'    1.4401  108.83 -179.85  116.10  1.5284
IC C5'  C4'  C3'   O3'    1.5160  116.10   76.70  115.12  1.4212
IC C4'  C3'  O3'   +P     1.5284  111.92  159.13  119.05  1.6001
IC C3'  O3'  +P    +O5'   1.4212  119.05  -98.86  101.45  1.5996
IC O4'  C3'  *C4'  C5'    1.4572  104.06 -120.04  116.10  1.5160
IC C2'  C4'  *C3'  O3'    1.5284  100.16 -124.08  115.12  1.4212
IC C4'  C3'  C2'   C1'    1.5284  100.16   39.58  102.04  1.5251
IC C3'  C2'  C1'   N9     1.5284  101.97  144.39  113.71  1.4896
IC O4'  C1'  N9    C4     1.5251  113.71  -97.2   125.59  1.3783
IC C3'  C1'  *C2'  O2'    1.5312  102.03  117.61  107.13  1.4206
IC C1'  C2'  O2'   CM2    1.5393  107.13   90.00  107.00  1.4150
IC C2'  O2'  CM2   HM2    1.4206  107.00  180.00    0.0   0.0
IC HM2  O2'  *CM2  HM3    0.0       0.0   120.00    0.0   0.0
IC HM2  O2'  *CM2  HM1    0.0       0.0  -120.00    0.0   0.0
IC O4'  C2'  *C1'  H1'    0.0       0.0  -115.0     0.0   0.0
IC C1'  C3'  *C2'  H2''   0.0       0.0   115.0     0.0   0.0
IC C2'  C4'  *C3'  H3'    0.0       0.0   115.0     0.0   0.0
IC C3'  O4'  *C4'  H4'    0.0       0.0  -115.0     0.0   0.0
IC C4'  O5'  *C5'  H5'    0.0       0.0  -115.0     0.0   0.0
IC C4'  O5'  *C5'  H5''   0.0       0.0   115.0     0.0   0.0
```

```
ACCE N3
ACCE N7
ACCE N1
ACCE O1P  P
ACCE O2P  P
ACCE O2'
ACCE O3'
ACCE O4'
ACCE O5'

RESI 2MA           -1.00 ! 2-methyladenosine
GROUP
ATOM N9     NG2R51  0.04 !        H61  H62
ATOM C8     CG2R53  0.43 !          \  /
ATOM H8     HGR52   0.06 !           N6
ATOM N7     NG2R50 -0.84 !           |
ATOM C5     CG2RC0  0.29 !           C6
ATOM C6     CG2R64  0.57 !          // \
ATOM N6     NG2S3  -0.76 !         N1   C5--N7\\
ATOM H61    HGP4    0.36 !         |    ||     C8-H8
ATOM H62    HGP4    0.36 !   H21   C2   C4--N9/
ATOM N1     NG2R62 -0.78 !      \  / \\ /     \
ATOM C2     CG2R64  0.38 ! H22-CM2   N3        \
ATOM N3     NG2R62 -0.83 !       |              \
ATOM C4     CG2RC0  0.54 !      H23              \
ATOM CM2    CG331  -0.09 !                        \
ATOM H21    HGA3    0.09 !                         \
ATOM H22    HGA3    0.09 !                          \
ATOM H23    HGA3    0.09 !      O1P    H5' H4'  O4'  \
GROUP                    !       |      |    \ /   \  \
ATOM P      P       1.50 !      -P-O5'-C5'---C4'    C1'
ATOM O1P    ON3    -0.78 !       |      |     \     / \
ATOM O2P    ON3    -0.78 !      O2P    H5''   C3'--C2' H1'
ATOM O5'    ON2    -0.57 !                   / \   / \
ATOM C5'    CN8B   -0.08 !                O3' H3' O2' H2''
ATOM H5'    HN8     0.09 !                 |       |
ATOM H5''   HN8     0.09 !                         H2'
GROUP
ATOM C4'    CN7     0.16
ATOM H4'    HN7     0.09
ATOM O4'    ON6B   -0.50
ATOM C1'    CN7B    0.16
ATOM H1'    HN7     0.09
GROUP
ATOM C2'    CN7B    0.14
ATOM H2''   HN7     0.09
ATOM O2'    ON5    -0.66
ATOM H2'    HN5     0.43
GROUP
ATOM C3'    CN7     0.01
ATOM H3'    HN7     0.09
ATOM O3'    ON2    -0.57
BOND N9   C8   N9   C4   C8   N7   C8   H8
BOND N7   C5   C5   C6   C5   C4   C6   N6
BOND C6   N1   N6   H61  N6   H62  N1   C2
BOND C2   N3   C2   CM2  N3   C4   CM2  H21
BOND CM2  H22  CM2  H23
BOND P    O1P     P    O2P     P    O5'     O5'  C5'     C5'  H5''
BOND C5'  C4'     C4'  O4'     C4'  C3'     O4'  C1'
BOND C1'  N9      C1'  C2'     C2'  C3'     C3'  O3'     O3'  +P
BOND C2'  O2'     O2'  H2'
BOND C1'  H1'     C2'  H2''    C3'  H3'     C4'  H4'     C5'  H5'
IMPR N6   H61  H62  C6        C6   C5   N1   N6
```

```
IC C8   C4   *N9   C1'       1.3791  105.54 -179.95  126.56  1.4896
IC C4   N9   C8    N7        1.3478  107.62   -0.02  113.03  1.3234
IC N7   N9   *C8   H8        1.3234  113.03 -179.92  122.45  1.0926
IC N9   C8   N7    C5        1.3704  113.03    0.02  103.27  1.4087
IC C4   N7   *C5   C6        1.3914  110.24  179.96  134.34  1.4102
IC N7   C5   C6    N1        1.4087  134.34 -179.98  118.65  1.3634
IC N1   C5   *C6   N6        1.3634  118.65 -179.97  124.28  1.3467
IC C5   C6   N6    H61       1.4102  124.28   -0.00  121.03  0.9961
IC H61  C6   *N6   H62       0.9961  121.03  179.96  117.38  0.9946
IC C5   C6   N1    C2        1.4102  118.65    0.03  119.61  1.3560
IC C6   N1   C2    CM2       1.3634  119.61  179.98  116.66  1.4976
IC CM2  N1   *C2   N3        1.4976  116.66  179.99  125.62  1.3527
IC N1   C2   CM2   H21       1.3560  116.66   59.69  109.88  1.1111
IC H21  C2   *CM2  H22       1.1111  109.88  120.30  111.57  1.1105
IC H21  C2   *CM2  H23       1.1111  109.88 -119.37  109.94  1.1102
!ribose
IC -O3' P    O5'   C5'    1.6001  101.45  -39.25  119.00  1.4401
IC -O3' O5'  *P    O1P    1.6001  101.45 -115.82  109.74  1.4802
IC -O3' O5'  *P    O2P    1.6001  101.45  115.90  109.80  1.4801
IC  P   O5'  C5'   C4'    1.5996  119.00 -151.39  110.04  1.5160
IC O5'  C5'  C4'   C3'    1.4401  108.83 -179.85  116.10  1.5284
IC C5'  C4'  C3'   O3'    1.5160  116.10   76.70  115.12  1.4212
IC C4'  C3'  O3'   +P     1.5284  111.92  159.13  119.05  1.6001
IC C3'  O3'  +P    +O5'   1.4212  119.05  -98.86  101.45  1.5996
IC O4'  C3'  *C4'  C5'    1.4572  104.06 -120.04  116.10  1.5160
IC C2'  C4'  *C3'  O3'    1.5284  100.16 -124.08  115.12  1.4212
IC C4'  C3'  C2'   C1'    1.5284  100.16   39.58  102.04  1.5251
IC C3'  C2'  C1'   N9     1.5284  101.97  144.39  113.71  1.4896
IC O4'  C1'  N9    C4     1.5251  113.71  -97.2   125.59  1.3783
IC C1'  C3'  *C2'  O2'    1.5284  102.04 -114.67  110.81  1.4212
IC H2'  O2'  C2'   C3'    0.9600  114.97  148.63  111.92  1.5284
IC O4'  C2'  *C1'  H1'    0.0       0.0  -115.0     0.0   0.0
IC C1'  C3'  *C2'  H2''   0.0       0.0   115.0     0.0   0.0
IC C2'  C4'  *C3'  H3'    0.0       0.0   115.0     0.0   0.0
IC C3'  O4'  *C4'  H4'    0.0       0.0  -115.0     0.0   0.0
IC C4'  O5'  *C5'  H5'    0.0       0.0  -115.0     0.0   0.0
IC C4'  O5'  *C5'  H5''   0.0       0.0   115.0     0.0   0.0
DONO H2'  O2'
DONO H61  N6
DONO H62  N6
ACCE N3
ACCE N7
ACCE N1
ACCE O1P  P
ACCE O2P  P
ACCE O2'
ACCE O3'
ACCE O4'
ACCE O5'

RESI 8MA           -1.00 ! 8-methyladenosine
GROUP
ATOM N9     NG2R51 -0.05 !      H61  H62
ATOM C8     CG2R53  0.40 !         \  /
ATOM N7     NG2R50 -0.71 !          N6
ATOM C5     CG2RC0  0.28 !          |
ATOM C6     CG2R64  0.46 !          C6
ATOM N6     NG2S3  -0.77 !        // \              H81
ATOM H61    HGP4    0.38 !       N1   C5--N7\\     /
ATOM H62    HGP4    0.38 !       |    ||     C8-C8M--H82
ATOM N1     NG2R62 -0.74 !       C2   C4--N9/      \
ATOM C2     CG2R64  0.50 !      / \\ /     \        H83
ATOM H2     HGR62   0.13 !    H2   N3       \
```

```
ATOM N3     NG2R62 -0.75 !                  \
ATOM C4     CG2RC0  0.43 !                   \
GROUP                    !                    \
ATOM C8M    CG331  -0.21 ! O1P    H5' H4'  O4'  \
ATOM H81    HGA3    0.09 !  |      |     \ /   \  \
ATOM H82    HGA3    0.09 ! -P-O5'-C5'---C4'    C1'
ATOM H83    HGA3    0.09 !  |      |     \     / \
GROUP                    ! O2P    H5''   C3'--C2' H1'
ATOM P      P       1.50 !               / \   / \
ATOM O1P    ON3    -0.78 !            O3' H3' O2' H2''
ATOM O2P    ON3    -0.78 !             |       |
ATOM O5'    ON2    -0.57 !                    H2'
ATOM C5'    CN8B   -0.08
ATOM H5'    HN8     0.09
ATOM H5''   HN8     0.09
GROUP
ATOM C4'    CN7     0.16
ATOM H4'    HN7     0.09
ATOM O4'    ON6B   -0.50
ATOM C1'    CN7B    0.16
ATOM H1'    HN7     0.09
GROUP
ATOM C2'    CN7B    0.14
ATOM H2''   HN7     0.09
ATOM O2'    ON5    -0.66
ATOM H2'    HN5     0.43
GROUP
ATOM C3'    CN7     0.01
ATOM H3'    HN7     0.09
ATOM O3'    ON2    -0.57
BOND N9   C8   N9   C4   C8   N7   C8   C8M
BOND N7   C5   C5   C6   C5   C4   C6   N6
BOND C6   N1   N6   H61  N6   H62  N1   C2
BOND C2   N3   C2   H2   N3   C4
BOND C8M  H81  C8M  H82  C8M  H83
BOND P    O1P     P    O2P     P    O5'     O5'  C5'     C5'  H5''
BOND C5'  C4'     C4'  O4'     C4'  C3'     O4'  C1'
BOND C1'  N9      C1'  C2'     C2'  C3'     C3'  O3'     O3'  +P
BOND C2'  O2'     O2'  H2'
BOND C1'  H1'     C2'  H2''    C3'  H3'     C4'  H4'     C5'  H5'
IMPR N6   H61  H62  C6        C6   C5   N1   N6
IC C8   C4   *N9   C1'      1.3791  105.54 -179.95  126.56  1.4896
IC C4   N9   C8    N7       1.3810  105.64    0.00  114.14  1.3222
IC N7   N9   *C8   C8M      1.3222  114.14 -179.97  122.54  1.4902
IC N9   C8   N7    C5       1.3872  114.14    0.02  103.47  1.3964
IC C4   N7   *C5   C6       1.3989  110.99 -179.96  132.58  1.4111
IC N7   C5   C6    N1       1.3964  132.58  179.98  118.68  1.3587
IC N1   C5   *C6   N6       1.3587  118.68 -179.99  124.58  1.3449
IC C5   C6   N6    H61      1.4111  124.58 -180.00  117.11  0.9955
IC H61  C6   *N6   H62      0.9955  117.11 -179.95  121.42  0.9956
IC C5   C6   N1    C2       1.4111  118.68    0.04  119.99  1.3616
IC C6   N1   C2    N3       1.3587  119.99   -0.10  125.10  1.3580
IC N3   N1   *C2   H2       1.3580  125.10 -179.88  117.46  1.0936
IC N9   C8   C8M   H81      1.3872  122.54  -60.35  109.80  1.1089
IC H81  C8   *C8M  H82      1.1089  109.80  120.67  109.81  1.1093
IC H81  C8   *C8M  H83      1.1089  109.80 -119.69  109.85  1.1104
!ribose
IC -O3' P    O5'   C5'    1.6001  101.45  -39.25  119.00  1.4401
IC -O3' O5'  *P    O1P    1.6001  101.45 -115.82  109.74  1.4802
IC -O3' O5'  *P    O2P    1.6001  101.45  115.90  109.80  1.4801
IC  P   O5'  C5'   C4'    1.5996  119.00 -151.39  110.04  1.5160
IC O5'  C5'  C4'   C3'    1.4401  108.83 -179.85  116.10  1.5284
IC C5'  C4'  C3'   O3'    1.5160  116.10   76.70  115.12  1.4212
```

```
IC C4'  C3'  O3'   +P     1.5284  111.92  159.13  119.05  1.6001
IC C3'  O3'  +P    +O5'   1.4212  119.05  -98.86  101.45  1.5996
IC O4'  C3'  *C4'  C5'    1.4572  104.06 -120.04  116.10  1.5160
IC C2'  C4'  *C3'  O3'    1.5284  100.16 -124.08  115.12  1.4212
IC C4'  C3'  C2'   C1'    1.5284  100.16   39.58  102.04  1.5251
IC C3'  C2'  C1'   N9     1.5284  101.97  144.39  113.71  1.4896
IC O4'  C1'  N9    C4     1.5251  113.71  -97.2   125.59  1.3783
IC C1'  C3'  *C2'  O2'    1.5284  102.04 -114.67  110.81  1.4212
IC H2'  O2'  C2'   C3'    0.9600  114.97  148.63  111.92  1.5284
IC O4'  C2'  *C1'  H1'    0.0       0.0  -115.0     0.0   0.0
IC C1'  C3'  *C2'  H2''   0.0       0.0   115.0     0.0   0.0
IC C2'  C4'  *C3'  H3'    0.0       0.0   115.0     0.0   0.0
IC C3'  O4'  *C4'  H4'    0.0       0.0  -115.0     0.0   0.0
IC C4'  O5'  *C5'  H5'    0.0       0.0  -115.0     0.0   0.0
IC C4'  O5'  *C5'  H5''   0.0       0.0   115.0     0.0   0.0
DONO H2'  O2'
DONO H61  N6
DONO H62  N6
ACCE N3
ACCE N7
ACCE N1
ACCE O1P  P
ACCE O2P  P
ACCE O2'
ACCE O3'
ACCE O4'
ACCE O5'

RESI INO           -1.00 ! inosine
GROUP
ATOM N9     NG2R51 -0.01 !        O6
ATOM C8     CG2R53  0.26 !        ||
ATOM H8     HGR52   0.15 !        C6
ATOM N7     NG2R50 -0.61 !       /  \
ATOM C5     CG2RC0  0.01 !   H1-N1   C5--N7\\
ATOM C6     CG2R63  0.55 !      |    ||     C8-H8
ATOM O6     OG2D4  -0.51 !   H2-C2   C4--N9/
ATOM N1     NG2R61 -0.32 !       \\ /     \
ATOM H1     HGP1    0.25 !        N3       \
ATOM C2     CG2R64  0.51 !                  \
ATOM H2     HGR62   0.10 !                   \
ATOM N3     NG2R62 -0.64 !                    \
ATOM C4     CG2RC0  0.26 ! O1P    H5' H4'  O4'  \
GROUP                    !  |      |    \ /   \  \
ATOM P      P       1.50 ! -P-O5'-C5'---C4'    C1'
ATOM O1P    ON3    -0.78 !  |      |     \     / \
ATOM O2P    ON3    -0.78 ! O2P    H5''   C3'--C2' H1'
ATOM O5'    ON2    -0.57 !              / \   / \
ATOM C5'    CN8B   -0.08 !            O3' H3' O2' H2''
ATOM H5'    HN8     0.09 !             |       |
ATOM H5''   HN8     0.09 !                    H2'
GROUP
ATOM C4'    CN7     0.16
ATOM H4'    HN7     0.09
ATOM O4'    ON6B   -0.50
ATOM C1'    CN7B    0.16
ATOM H1'    HN7     0.09
GROUP
ATOM C2'    CN7B    0.14
ATOM H2''   HN7     0.09
ATOM O2'    ON5    -0.66
ATOM H2'    HN5     0.43
GROUP
```

```
ATOM C3'    CN7      0.01
ATOM H3'    HN7      0.09
ATOM O3'    ON2     -0.57
BOND N9   C8   N9   C4   C8   N7   C8   H8
BOND N7   C5   C5   C6   C5   C4   C6   O6
BOND C6   N1   N1   C2   N1   H1   C2   N3
BOND C2   H2   N3   C4
BOND P    O1P     P    O2P     P    O5'     O5'  C5'     C5'  H5''
BOND C5'  C4'     C4'  O4'     C4'  C3'     O4'  C1'
BOND C1'  N9      C1'  C2'     C2'  C3'     C3'  O3'     O3'  +P
BOND C2'  O2'     O2'  H2'
BOND C1'  H1'     C2'  H2''    C3'  H3'     C4'  H4'     C5'  H5'
IMPR C6     C5     N1     O6
IC C8   C4   *N9   C1'       1.3791  105.54 -179.95  126.56  1.4896
IC C4   N9   C8    N7        1.3808  105.97   -0.01  113.61  1.3256
IC N7   N9   *C8   H8        1.3256  113.61 -179.98  121.80  1.0906
IC N9   C8   N7    C5        1.3789  113.61    0.00  104.54  1.3887
IC C4   N7   *C5   C6        1.4102  109.97 -180.00  130.78  1.4189
IC N7   C5   C6    N1        1.3887  130.78 -179.98  112.64  1.3862
IC N1   C5   *C6   O6        1.3862  112.64  179.99  130.12  1.2327
IC C5   C6   N1    C2        1.4189  112.64   -0.02  126.30  1.3907
IC C2   C6   *N1   H1        1.3907  126.30 -179.96  114.29  1.0034
IC C6   N1   C2    N3        1.3862  126.30    0.05  120.33  1.3574
IC N3   N1   *C2   H2        1.3574  120.33  179.94  117.90  1.0925
!ribose
IC -O3' P    O5'   C5'    1.6001  101.45  -39.25  119.00  1.4401
IC -O3' O5'  *P    O1P    1.6001  101.45 -115.82  109.74  1.4802
IC -O3' O5'  *P    O2P    1.6001  101.45  115.90  109.80  1.4801
IC  P   O5'  C5'   C4'    1.5996  119.00 -151.39  110.04  1.5160
IC O5'  C5'  C4'   C3'    1.4401  108.83 -179.85  116.10  1.5284
IC C5'  C4'  C3'   O3'    1.5160  116.10   76.70  115.12  1.4212
IC C4'  C3'  O3'   +P     1.5284  111.92  159.13  119.05  1.6001
IC C3'  O3'  +P    +O5'   1.4212  119.05  -98.86  101.45  1.5996
IC O4'  C3'  *C4'  C5'    1.4572  104.06 -120.04  116.10  1.5160
IC C2'  C4'  *C3'  O3'    1.5284  100.16 -124.08  115.12  1.4212
IC C4'  C3'  C2'   C1'    1.5284  100.16   39.58  102.04  1.5251
IC C3'  C2'  C1'   N9     1.5284  101.97  144.39  113.71  1.4896
IC O4'  C1'  N9    C4     1.5251  113.71  -97.2   125.59  1.3783
IC C1'  C3'  *C2'  O2'    1.5284  102.04 -114.67  110.81  1.4212
IC H2'  O2'  C2'   C3'    0.9600  114.97  148.63  111.92  1.5284
IC O4'  C2'  *C1'  H1'    0.0       0.0  -115.0     0.0   0.0
IC C1'  C3'  *C2'  H2''   0.0       0.0   115.0     0.0   0.0
IC C2'  C4'  *C3'  H3'    0.0       0.0   115.0     0.0   0.0
IC C3'  O4'  *C4'  H4'    0.0       0.0  -115.0     0.0   0.0
IC C4'  O5'  *C5'  H5'    0.0       0.0  -115.0     0.0   0.0
IC C4'  O5'  *C5'  H5''   0.0       0.0   115.0     0.0   0.0
DONO H2'  O2'
DONO H1   N1
ACCE O6   C6
ACCE N3
ACCE N7
ACCE O1P  P
ACCE O2P  P
ACCE O2'
ACCE O3'
ACCE O4'
ACCE O5'

RESI OMI           -1.00 ! 2'-O-methylinosine, MRI
GROUP
ATOM N9     NG2R51 -0.01 !         O6
ATOM C8     CG2R53  0.26 !         ||
ATOM H8     HGR52   0.15 !         C6
```

```
ATOM N7     NG2R50 -0.61 !        /  \
ATOM C5     CG2RC0  0.01 !    H1-N1   C5--N7\\
ATOM C6     CG2R63  0.55 !       |    ||     C8-H8
ATOM O6     OG2D4  -0.51 !    H2-C2   C4--N9/
ATOM N1     NG2R61 -0.32 !        \\ /     \
ATOM H1     HGP1    0.25 !         N3       \
ATOM C2     CG2R64  0.51 !                   \
ATOM H2     HGR62   0.10 !                    \
ATOM N3     NG2R62 -0.64 !                     \
ATOM C4     CG2RC0  0.26 ! O1P    H5' H4'  O4'  \
GROUP                    !  |      |    \ /   \  \
ATOM P      P       1.50 ! -P-O5'-C5'---C4'    C1'
ATOM O1P    ON3    -0.78 !  |      |     \     / \
ATOM O2P    ON3    -0.78 ! O2P    H5''   C3'--C2' H1'
ATOM O5'    ON2    -0.57 !              / \   / \
ATOM C5'    CN8B   -0.08 !            O3' H3' O2' H2''
ATOM H5'    HN8     0.09 !             |       |
ATOM H5''   HN8     0.09 !                    CM2
GROUP                    !                  /  |  \
ATOM C4'    CN7     0.16 !                HM1 HM2 HM3
ATOM H4'    HN7     0.09
ATOM O4'    ON6B   -0.50
ATOM C1'    CN7B    0.16
ATOM H1'    HN7     0.09
GROUP
ATOM C2'    CN7B    0.08
ATOM H2''   HN7     0.09
ATOM O2'    OG301  -0.34
ATOM CM2    CG331  -0.10
ATOM HM1    HGA3    0.09
ATOM HM2    HGA3    0.09
ATOM HM3    HGA3    0.09
GROUP
ATOM C3'    CN7     0.01
ATOM H3'    HN7     0.09
ATOM O3'    ON2    -0.57
BOND N9   C8   N9   C4   C8   N7   C8   H8
BOND N7   C5   C5   C6   C5   C4   C6   O6
BOND C6   N1   N1   C2   N1   H1   C2   N3
BOND C2   H2   N3   C4
BOND P    O1P     P    O2P     P    O5'     O5'  C5'     C5'  H5''
BOND C5'  C4'     C4'  O4'     C4'  C3'     O4'  C1'
BOND C1'  N9      C1'  C2'     C2'  C3'     C3'  O3'     O3'  +P
BOND C2'  O2'     CM2  O2'     CM2  HM1     HM2  CM2     HM3  CM2
BOND C1'  H1'     C2'  H2''    C3'  H3'     C4'  H4'     C5'  H5'
IMPR C6     C5     N1     O6
IC C8   C4   *N9   C1'      1.3791  105.54 -179.95  126.56  1.4896
IC C4   N9   C8    N7       1.3808  105.97   -0.01  113.61  1.3256
IC N7   N9   *C8   H8       1.3256  113.61 -179.98  121.80  1.0906
IC N9   C8   N7    C5       1.3789  113.61    0.00  104.54  1.3887
IC C4   N7   *C5   C6       1.4102  109.97 -180.00  130.78  1.4189
IC N7   C5   C6    N1       1.3887  130.78 -179.98  112.64  1.3862
IC N1   C5   *C6   O6       1.3862  112.64  179.99  130.12  1.2327
IC C5   C6   N1    C2       1.4189  112.64   -0.02  126.30  1.3907
IC C2   C6   *N1   H1       1.3907  126.30 -179.96  114.29  1.0034
IC C6   N1   C2    N3       1.3862  126.30    0.05  120.33  1.3574
IC N3   N1   *C2   H2       1.3574  120.33  179.94  117.90  1.0925
!2OM-ribose
IC -O3' P    O5'   C5'    1.6001  101.45  -39.25  119.00  1.4401
IC -O3' O5'  *P    O1P    1.6001  101.45 -115.82  109.74  1.4802
IC -O3' O5'  *P    O2P    1.6001  101.45  115.90  109.80  1.4801
IC  P   O5'  C5'   C4'    1.5996  119.00 -151.39  110.04  1.5160
IC O5'  C5'  C4'   C3'    1.4401  108.83 -179.85  116.10  1.5284
```

```
IC C5'  C4'  C3'   O3'    1.5160  116.10   76.70  115.12  1.4212
IC C4'  C3'  O3'   +P     1.5284  111.92  159.13  119.05  1.6001
IC C3'  O3'  +P    +O5'   1.4212  119.05  -98.86  101.45  1.5996
IC O4'  C3'  *C4'  C5'    1.4572  104.06 -120.04  116.10  1.5160
IC C2'  C4'  *C3'  O3'    1.5284  100.16 -124.08  115.12  1.4212
IC C4'  C3'  C2'   C1'    1.5284  100.16   39.58  102.04  1.5251
IC C3'  C2'  C1'   N9     1.5284  101.97  144.39  113.71  1.4896
IC O4'  C1'  N9    C4     1.5251  113.71  -97.2   125.59  1.3783
IC C3'  C1'  *C2'  O2'    1.5312  102.03  117.61  107.13  1.4206
IC C1'  C2'  O2'   CM2    1.5393  107.13   90.00  107.00  1.4150
IC C2'  O2'  CM2   HM2    1.4206  107.00  180.00    0.0   0.0
IC HM2  O2'  *CM2  HM3    0.0       0.0   120.00    0.0   0.0
IC HM2  O2'  *CM2  HM1    0.0       0.0  -120.00    0.0   0.0
IC O4'  C2'  *C1'  H1'    0.0       0.0  -115.0     0.0   0.0
IC C1'  C3'  *C2'  H2''   0.0       0.0   115.0     0.0   0.0
IC C2'  C4'  *C3'  H3'    0.0       0.0   115.0     0.0   0.0
IC C3'  O4'  *C4'  H4'    0.0       0.0  -115.0     0.0   0.0
IC C4'  O5'  *C5'  H5'    0.0       0.0  -115.0     0.0   0.0
IC C4'  O5'  *C5'  H5''   0.0       0.0   115.0     0.0   0.0
DONO H1   N1
ACCE O6   C6
ACCE N3
ACCE N7
ACCE O1P  P
ACCE O2P  P
ACCE O2'
ACCE O3'
ACCE O4'
ACCE O5'

RESI 1MI           -1.00 ! 1-methylinosine
GROUP
ATOM N9     NG2R51  0.00 !          O6
ATOM C8     CG2R53  0.36 !          ||
ATOM H8     HGR52   0.10 !  H11      C6
ATOM N7     NG2R50 -0.70 !     \    /  \
ATOM C5     CG2RC0  0.08 ! H12-C1M-N1   C5--N7\\
ATOM C6     CG2R63  0.66 !     /   |    ||     C8-H8
ATOM O6     OG2D4  -0.53 !  H13    C2   C4--N9/
ATOM N1     NG2R61 -0.36 !        / \\ /     \
ATOM C2     CG2R64  0.68 !      H2   N3       \
ATOM H2     HGR62   0.05 !                     \
ATOM N3     NG2R62 -0.81 !                      \
ATOM C4     CG2RC0  0.34 !                       \
ATOM C1M    CG331  -0.14 !   O1P    H5' H4'  O4'  \
ATOM H11    HGA3    0.09 !    |      |    \ /   \  \
ATOM H12    HGA3    0.09 !   -P-O5'-C5'---C4'    C1'
ATOM H13    HGA3    0.09 !    |      |     \     / \
GROUP                    !   O2P    H5''   C3'--C2' H1'
ATOM P      P       1.50 !                 / \   / \
ATOM O1P    ON3    -0.78 !              O3' H3' O2' H2''
ATOM O2P    ON3    -0.78 !               |       |
ATOM O5'    ON2    -0.57 !                      H2'
ATOM C5'    CN8B   -0.08
ATOM H5'    HN8     0.09
ATOM H5''   HN8     0.09
GROUP
ATOM C4'    CN7     0.16
ATOM H4'    HN7     0.09
ATOM O4'    ON6B   -0.50
ATOM C1'    CN7B    0.16
ATOM H1'    HN7     0.09
GROUP
```

```
ATOM C2'    CN7B    0.14
ATOM H2''   HN7     0.09
ATOM O2'    ON5    -0.66
ATOM H2'    HN5     0.43
GROUP
ATOM C3'    CN7     0.01
ATOM H3'    HN7     0.09
ATOM O3'    ON2    -0.57
BOND N9   C8   N9   C4   C8   N7   C8   H8
BOND N7   C5   C5   C6   C5   C4   C6   O6
BOND C6   N1   N1   C2   N1   C1M  C2   N3
BOND C2   H2   N3   C4   C1M  H11  C1M  H12
BOND C1M  H13
BOND P    O1P     P    O2P     P    O5'     O5'  C5'     C5'  H5''
BOND C5'  C4'     C4'  O4'     C4'  C3'     O4'  C1'
BOND C1'  N9      C1'  C2'     C2'  C3'     C3'  O3'     O3'  +P
BOND C2'  O2'     O2'  H2'
BOND C1'  H1'     C2'  H2''    C3'  H3'     C4'  H4'     C5'  H5'
IMPR C6     C5     N1     O6
IC C8   C4   *N9   C1'       1.3791  105.54 -179.95  126.56  1.4896
IC C4   N9   C8    N7        1.3456  107.54    0.02  112.52  1.3210
IC N7   N9   *C8   H8        1.3210  112.52  179.96  123.01  1.0928
IC N9   C8   N7    C5        1.3690  112.52   -0.00  104.40  1.3965
IC C4   N7   *C5   C6        1.3990  109.39  179.99  132.01  1.4186
IC N7   C5   C6    N1        1.3965  132.01 -179.98  113.25  1.4137
IC N1   C5   *C6   O6        1.4137  113.25 -179.99  128.21  1.2361
IC C5   C6   N1    C2        1.4186  113.25   -0.04  123.91  1.4000
IC C2   C6   *N1   C1M       1.4000  123.91 -179.93  117.61  1.4804
IC C6   N1   C2    N3        1.4137  123.91    0.10  121.49  1.3574
IC N3   N1   *C2   H2        1.3574  121.49  179.90  117.13  1.0917
IC C6   N1   C1M   H11       1.4137  117.61   59.12  110.93  1.1158
IC H11  N1   *C1M  H12       1.1158  110.93  120.89  113.83  1.1141
IC H11  N1   *C1M  H13       1.1158  110.93 -118.26  110.89  1.1166
!ribose
IC -O3' P    O5'   C5'    1.6001  101.45  -39.25  119.00  1.4401
IC -O3' O5'  *P    O1P    1.6001  101.45 -115.82  109.74  1.4802
IC -O3' O5'  *P    O2P    1.6001  101.45  115.90  109.80  1.4801
IC  P   O5'  C5'   C4'    1.5996  119.00 -151.39  110.04  1.5160
IC O5'  C5'  C4'   C3'    1.4401  108.83 -179.85  116.10  1.5284
IC C5'  C4'  C3'   O3'    1.5160  116.10   76.70  115.12  1.4212
IC C4'  C3'  O3'   +P     1.5284  111.92  159.13  119.05  1.6001
IC C3'  O3'  +P    +O5'   1.4212  119.05  -98.86  101.45  1.5996
IC O4'  C3'  *C4'  C5'    1.4572  104.06 -120.04  116.10  1.5160
IC C2'  C4'  *C3'  O3'    1.5284  100.16 -124.08  115.12  1.4212
IC C4'  C3'  C2'   C1'    1.5284  100.16   39.58  102.04  1.5251
IC C3'  C2'  C1'   N9     1.5284  101.97  144.39  113.71  1.4896
IC O4'  C1'  N9    C4     1.5251  113.71  -97.2   125.59  1.3783
IC C1'  C3'  *C2'  O2'    1.5284  102.04 -114.67  110.81  1.4212
IC H2'  O2'  C2'   C3'    0.9600  114.97  148.63  111.92  1.5284
IC O4'  C2'  *C1'  H1'    0.0       0.0  -115.0     0.0   0.0
IC C1'  C3'  *C2'  H2''   0.0       0.0   115.0     0.0   0.0
IC C2'  C4'  *C3'  H3'    0.0       0.0   115.0     0.0   0.0
IC C3'  O4'  *C4'  H4'    0.0       0.0  -115.0     0.0   0.0
IC C4'  O5'  *C5'  H5'    0.0       0.0  -115.0     0.0   0.0
IC C4'  O5'  *C5'  H5''   0.0       0.0   115.0     0.0   0.0
DONO H2'  O2'
ACCE O6   C6
ACCE N3
ACCE N7
ACCE O1P  P
ACCE O2P  P
ACCE O2'
ACCE O3'
```

```
ACCE O4'
ACCE O5'

RESI MMI            -1.00 ! 1,2'-O-dimethylinosine
GROUP
ATOM N9     NG2R51  0.01 !            O6
ATOM C8     CG2R53  0.36 !            ||
ATOM H8     HGR52   0.10 !   H11      C6
ATOM N7     NG2R50 -0.70 !      \    /  \
ATOM C5     CG2RC0  0.08 !  H12-C10-N1   C5--N7\\
ATOM C6     CG2R63  0.66 !      /   |    ||     C8-H8
ATOM O6     OG2D4  -0.53 !   H13    C2   C4--N9/
ATOM N1     NG2R61 -0.36 !         / \\ /     \
ATOM C2     CG2R64  0.68 !       H2   N3       \
ATOM H2     HGR62   0.05 !                      \
ATOM N3     NG2R62 -0.80 !                       \
ATOM C4     CG2RC0  0.33 !                        \
ATOM C10    CG331  -0.15 !    O1P    H5' H4'  O4'  \
ATOM H11    HGA3    0.09 !     |      |    \ /   \  \
ATOM H12    HGA3    0.09 !    -P-O5'-C5'---C4'    C1'
ATOM H13    HGA3    0.09 !     |      |     \     / \
GROUP                    !    O2P    H5''   C3'--C2' H1'
ATOM P      P       1.50 !                 / \   / \
ATOM O1P    ON3    -0.78 !               O3' H3' O2' H2''
ATOM O2P    ON3    -0.78 !                |       |
ATOM O5'    ON2    -0.57 !                       CM2
ATOM C5'    CN8B   -0.08 !                      /  |  \
ATOM H5'    HN8     0.09 !                   HM1 HM2 HM3
ATOM H5''   HN8     0.09
GROUP
ATOM C4'    CN7     0.16
ATOM H4'    HN7     0.09
ATOM O4'    ON6B   -0.50
ATOM C1'    CN7B    0.16
ATOM H1'    HN7     0.09
GROUP
ATOM C2'    CN7B    0.08
ATOM H2''   HN7     0.09
ATOM O2'    OG301  -0.34
ATOM CM2    CG331  -0.10
ATOM HM1    HGA3    0.09
ATOM HM2    HGA3    0.09
ATOM HM3    HGA3    0.09
GROUP
ATOM C3'    CN7     0.01
ATOM H3'    HN7     0.09
ATOM O3'    ON2    -0.57
BOND N9   C8   N9   C4   C8   N7   C8   H8
BOND N7   C5   C5   C6   C5   C4   C6   O6
BOND C6   N1   N1   C2   N1   C10  C2   N3
BOND C2   H2   N3   C4   C10  H11  C10  H12
BOND C10  H13
BOND P    O1P     P    O2P     P    O5'     O5'  C5'     C5'  H5''
BOND C5'  C4'     C4'  O4'     C4'  C3'     O4'  C1'
BOND C1'  N9      C1'  C2'     C2'  C3'     C3'  O3'     O3'  +P
BOND C2'  O2'     CM2  O2'     CM2  HM1     HM2  CM2     HM3  CM2
BOND C1'  H1'     C2'  H2''    C3'  H3'     C4'  H4'     C5'  H5'
IMPR C6     C5     N1     O6
!2OM-ribose
IC -O3' P    O5'   C5'    1.6001  101.45  -39.25  119.00  1.4401
IC -O3' O5'  *P    O1P    1.6001  101.45 -115.82  109.74  1.4802
IC -O3' O5'  *P    O2P    1.6001  101.45  115.90  109.80  1.4801
IC  P   O5'  C5'   C4'    1.5996  119.00 -151.39  110.04  1.5160
```

```
IC O5'  C5'  C4'   C3'    1.4401  108.83 -179.85  116.10  1.5284
IC C5'  C4'  C3'   O3'    1.5160  116.10   76.70  115.12  1.4212
IC C4'  C3'  O3'   +P     1.5284  111.92  159.13  119.05  1.6001
IC C3'  O3'  +P    +O5'   1.4212  119.05  -98.86  101.45  1.5996
IC O4'  C3'  *C4'  C5'    1.4572  104.06 -120.04  116.10  1.5160
IC C2'  C4'  *C3'  O3'    1.5284  100.16 -124.08  115.12  1.4212
IC C4'  C3'  C2'   C1'    1.5284  100.16   39.58  102.04  1.5251
IC C3'  C1'  *C2'  O2'    1.5312  102.03  117.61  107.13  1.4206
IC C1'  C2'  O2'   CM2    1.5393  107.13   90.00  107.00  1.4150
IC C2'  O2'  CM2   HM2    1.4206  107.00  180.00    0.0   0.0
IC HM2  O2'  *CM2  HM3    0.0       0.0   120.00    0.0   0.0
IC HM2  O2'  *CM2  HM1    0.0       0.0  -120.00    0.0   0.0
IC O4'  C2'  *C1'  H1'    0.0       0.0  -115.0     0.0   0.0
IC C1'  C3'  *C2'  H2''   0.0       0.0   115.0     0.0   0.0
IC C2'  C4'  *C3'  H3'    0.0       0.0   115.0     0.0   0.0
IC C3'  O4'  *C4'  H4'    0.0       0.0  -115.0     0.0   0.0
IC C4'  O5'  *C5'  H5'    0.0       0.0  -115.0     0.0   0.0
IC C4'  O5'  *C5'  H5''   0.0       0.0   115.0     0.0   0.0
IC C3'  C2'  C1'   N9     1.5284  101.97  144.39  113.71  1.4896
IC O4'  C1'  N9    C4     1.5251  113.71  -97.2   125.59  1.3783
IC C1'  C4   *N9   C8     0.0       0.0   180.0     0.0   0.0
IC C4   N9   C8    N7     1.3456  107.54    0.02  112.52  1.3210
IC N7   N9   *C8   H8     1.3210  112.52  179.96  123.01  1.0928
IC N9   C8   N7    C5     1.3690  112.52   -0.00  104.40  1.3965
IC C4   N7   *C5   C6     1.3990  109.39  179.99  132.01  1.4186
IC N7   C5   C6    N1     1.3965  132.01 -179.98  113.25  1.4137
IC N1   C5   *C6   O6     1.4137  113.25 -179.99  128.21  1.2361
IC C5   C6   N1    C2     1.4186  113.25   -0.04  123.91  1.4000
IC C6   N1   C2    N3     1.4137  123.91    0.10  121.49  1.3574
IC N3   N1   *C2   H2     1.3574  121.49  179.90  117.13  1.0917
IC C2   C6   *N1   C10    1.4000  123.91 -179.93  117.61  1.4804
IC C6   N1   C10   H11    1.4137  117.61   59.12  110.93  1.1158
IC H11  N1   *C10  H12    1.1158  110.93  120.89  113.83  1.1141
IC H11  N1   *C10  H13    1.1158  110.93 -118.26  110.89  1.1166
ACCE O6   C6
ACCE N3
ACCE N7
ACCE O1P  P
ACCE O2P  P
ACCE O2'
ACCE O3'
ACCE O4'
ACCE O5'

RESI SMA           -1.00 ! 2-methylthio-N6-methyladenosine
GROUP
ATOM N9     NG2R51 -0.02 !        H11
ATOM C8     CG2R53  0.37 !           \
ATOM H8     HGR52   0.10 !      H12--C10   H6
ATOM N7     NG2R50 -0.84 !           / \  /
ATOM C5     CG2RC0  0.28 !        H13   N6
ATOM C6     CG2R64  0.38 !              |
ATOM N6     NG311  -0.48 !              C6
ATOM H6     HGPAM1  0.36 !             // \
ATOM N1     NG2R62 -0.58 !    H21     N1   C5--N7\\
ATOM C2     CG2R64  0.63 !       \     |    ||     C8-H8
ATOM N3     NG2R62 -0.76 ! H22 -C20   C2   C4--N9/
ATOM C4     CG2RC0  0.48 !       / \  / \\ /      \
ATOM C10    CG331  -0.09 !   H23   S2   N3         \
ATOM H11    HGA3    0.09 !                          \
ATOM H12    HGA3    0.09 !                           \
ATOM H13    HGA3    0.09 !      O1P    H5' H4'  O4'   \
ATOM S2     SG311  -0.28 !       |      |    \ /   \  \
```

```
ATOM C20    CG331  -0.09 !      -P-O5'-C5'---C4'    C1'
ATOM H21    HGA3    0.09 !        |      |     \     / \
ATOM H22    HGA3    0.09 !      O2P    H5''   C3'--C2' H1'
ATOM H23    HGA3    0.09 !                   / \   / \
GROUP                      !               O3' H3' O2' H2''
ATOM P      P       1.50 !                  |       |
ATOM O1P    ON3    -0.78 !                         H2'
ATOM O2P    ON3    -0.78
ATOM O5'    ON2    -0.57
ATOM C5'    CN8B   -0.08
ATOM H5'    HN8     0.09
ATOM H5''   HN8     0.09
GROUP
ATOM C4'    CN7     0.16
ATOM H4'    HN7     0.09
ATOM O4'    ON6B   -0.50
ATOM C1'    CN7B    0.16
ATOM H1'    HN7     0.09
GROUP
ATOM C2'    CN7B    0.14
ATOM H2''   HN7     0.09
ATOM O2'    ON5    -0.66
ATOM H2'    HN5     0.43
GROUP
ATOM C3'    CN7     0.01
ATOM H3'    HN7     0.09
ATOM O3'    ON2    -0.57
BOND N9   C8   N9   C4   C8   N7   C8   H8
BOND N7   C5   C5   C6   C5   C4   C6   N6
BOND C6   N1   N6   C10  N6   H6   N1   C2
BOND C2   N3   C2   S2   N3   C4   C10  H11
BOND C10  H12  C10  H13  S2   C20  C20  H21
BOND C20  H22  C20  H23
BOND P    O1P     P    O2P     P    O5'     O5'  C5'     C5'  H5''
BOND C5'  C4'     C4'  O4'     C4'  C3'     O4'  C1'
BOND C1'  N9      C1'  C2'     C2'  C3'     C3'  O3'     O3'  +P
BOND C2'  O2'     O2'  H2'
BOND C1'  H1'     C2'  H2''    C3'  H3'     C4'  H4'     C5'  H5'
IMPR C6   C5   N1   N6     ! N6   C6   C10   H6
!ribose
IC -O3' P    O5'   C5'    1.6001  101.45  -39.25  119.00  1.4401
IC -O3' O5'  *P    O1P    1.6001  101.45 -115.82  109.74  1.4802
IC -O3' O5'  *P    O2P    1.6001  101.45  115.90  109.80  1.4801
IC  P   O5'  C5'   C4'    1.5996  119.00 -151.39  110.04  1.5160
IC O5'  C5'  C4'   C3'    1.4401  108.83 -179.85  116.10  1.5284
IC C5'  C4'  C3'   O3'    1.5160  116.10   76.70  115.12  1.4212
IC C4'  C3'  O3'   +P     1.5284  111.92  159.13  119.05  1.6001
IC C3'  O3'  +P    +O5'   1.4212  119.05  -98.86  101.45  1.5996
IC O4'  C3'  *C4'  C5'    1.4572  104.06 -120.04  116.10  1.5160
IC C2'  C4'  *C3'  O3'    1.5284  100.16 -124.08  115.12  1.4212
IC C4'  C3'  C2'   C1'    1.5284  100.16   39.58  102.04  1.5251
IC C3'  C2'  C1'   N9     1.5284  101.97  144.39  113.71  1.4896
IC O4'  C1'  N9    C4     1.5251  113.71  -97.2   125.59  1.3783
IC C1'  C3'  *C2'  O2'    1.5284  102.04 -114.67  110.81  1.4212
IC H2'  O2'  C2'   C3'    0.9600  114.97  148.63  111.92  1.5284
IC O4'  C2'  *C1'  H1'    0.0       0.0  -115.0     0.0   0.0
IC C1'  C3'  *C2'  H2''   0.0       0.0   115.0     0.0   0.0
IC C2'  C4'  *C3'  H3'    0.0       0.0   115.0     0.0   0.0
IC C3'  O4'  *C4'  H4'    0.0       0.0  -115.0     0.0   0.0
IC C4'  O5'  *C5'  H5'    0.0       0.0  -115.0     0.0   0.0
IC C4'  O5'  *C5'  H5''   0.0       0.0   115.0     0.0   0.0
IC C8   C4   *N9   C1'       1.3791  105.54 -179.95  126.56  1.4896
IC C4   N9   C8    N7        1.3822  106.04    0.04  114.04  1.3295
```

```
IC N7   N9  *C8   H8        1.3295  114.04 -179.99  121.53  1.0912
IC N9   C8   N7    C5        1.3836  114.04    0.18  103.32  1.3991
IC C4   N7  *C5   C6        1.4029  111.06 -179.46  131.88  1.4071
IC N7   C5   C6    N1        1.3991  131.88  178.29  117.85  1.3727
IC N1   C5  *C6   N6        1.3727  117.85 -178.34  120.77  1.3757
IC C5   C6   N6    C10       1.4071  120.77  168.55  125.04  1.4799
IC C10  C6  *N6   H6        1.4799  125.04 -155.51  116.33  1.0220
IC C5   C6   N1    C2        1.4071  117.85    1.48  119.79  1.3613
IC C6   N1   C2    S2        1.3727  119.79  179.54  118.24  1.7692
IC S2   N1  *C2   N3        1.7692  118.24  179.54  125.82  1.3545
IC C6   N6   C10   H11       1.3757  125.04  149.69  110.46  1.1133
IC H11  N6  *C10  H12       1.1133  110.46  119.87  110.71  1.1122
IC H11  N6  *C10  H13       1.1133  110.46 -119.60  111.82  1.1114
IC N1   C2   S2    C20       1.3613  118.24   -0.71  103.85  1.8245
IC C2   S2   C20   H21       1.7692  103.85 -179.86  110.75  1.1117
IC H21  S2  *C20  H22       1.1117  110.75  119.73  111.29  1.1113
IC H21  S2  *C20  H23       1.1117  110.75 -119.76  111.32  1.1114
DONO H2'  O2'
DONO H6   N6
ACCE N3
ACCE N7
ACCE N1
ACCE O1P  P
ACCE O2P  P
ACCE O2'
ACCE O3'
ACCE O4'
ACCE O5'

RESI 6AA           -1.00 ! N6-acetyladenosine
GROUP
ATOM N9     NG2R51 -0.01 !   H11    O10
ATOM C8     CG2R53  0.39 !      \   ||
ATOM H8     HGR52   0.09 ! H12--C11-C10   H6
ATOM N7     NG2R50 -0.81 !      /     \  /
ATOM C5     CG2RC0  0.35 !   H13       N6
ATOM C6     CG2R64  0.70 !              |
ATOM N6     NG2S1  -0.68 !             C6
ATOM H6     HGP1    0.32 !            // \
ATOM N1     NG2R62 -0.68 !           N1   C5--N7\\
ATOM C2     CG2R64  0.39 !           |    ||     C8-H8
ATOM H2     HGR62   0.14 !           C2   C4--N9/
ATOM N3     NG2R62 -0.82 !          / \\ /     \
ATOM C4     CG2RC0  0.58 !        H2   N3       \
ATOM C10    CG2O1   0.47 !                       \
ATOM O10    OG2D1  -0.44 !                        \
ATOM C11    CG331  -0.26 !                         \
ATOM H11    HGA3    0.09 !     O1P    H5' H4'  O4'  \
ATOM H12    HGA3    0.09 !      |      |    \ /   \  \
ATOM H13    HGA3    0.09 !     -P-O5'-C5'---C4'    C1'
GROUP                    !      |      |     \     / \
ATOM P      P       1.50 !     O2P    H5''   C3'--C2' H1'
ATOM O1P    ON3    -0.78 !                  / \   / \
ATOM O2P    ON3    -0.78 !                O3' H3' O2' H2''
ATOM O5'    ON2    -0.57 !                 |       |
ATOM C5'    CN8B   -0.08 !                        H2'
ATOM H5'    HN8     0.09
ATOM H5''   HN8     0.09
GROUP
ATOM C4'    CN7     0.16
ATOM H4'    HN7     0.09
ATOM O4'    ON6B   -0.50
ATOM C1'    CN7B    0.16
```

```
ATOM H1'    HN7     0.09
GROUP
ATOM C2'    CN7B    0.14
ATOM H2''   HN7     0.09
ATOM O2'    ON5    -0.66
ATOM H2'    HN5     0.43
GROUP
ATOM C3'    CN7     0.01
ATOM H3'    HN7     0.09
ATOM O3'    ON2    -0.57
BOND N9   C8   N9   C4   C8   N7   C8   H8
BOND N7   C5   C5   C6   C5   C4   C6   N6
BOND C6   N1   N6   C10  N6   H6   N1   C2
BOND C2   N3   C2   H2   N3   C4   C10  C11
BOND C10  O10  C11  H11  C11  H12  C11  H13
BOND P    O1P     P    O2P     P    O5'     O5'  C5'     C5'  H5''
BOND C5'  C4'     C4'  O4'     C4'  C3'     O4'  C1'
BOND C1'  N9      C1'  C2'     C2'  C3'     C3'  O3'     O3'  +P
BOND C2'  O2'     O2'  H2'
BOND C1'  H1'     C2'  H2''    C3'  H3'     C4'  H4'     C5'  H5'
IMPR C10    C11    N6     O10   C6   C5   N1   N6
IC C8   C4   *N9   C1'      1.3791  105.54 -179.95  126.56  1.4896
IC C4   N9   C8    N7       1.3502  107.42    0.03  112.64  1.3257
IC N7   N9   *C8   H8       1.3257  112.64  179.96  122.80  1.0941
IC N9   C8   N7    C5       1.3736  112.64   -0.02  103.68  1.4006
IC C4   N7   *C5   C6       1.3892  110.28 -179.94  133.00  1.4037
IC N7   C5   C6    N6       1.4006  133.00   -0.05  118.67  1.4073
IC N6   C5   *C6   N1       1.4073  118.67 -180.00  117.76  1.3746
IC C5   C6   N6    C10      1.4037  118.67 -179.95  133.09  1.3309
IC C10  C6   *N6   H6       1.3309  133.09  179.95  111.79  0.9912
IC C5   C6   N1    C2       1.4037  117.76   -0.03  120.02  1.3597
IC C6   N1   C2    N3       1.3746  120.02    0.05  124.76  1.3569
IC N3   N1   *C2   H2       1.3569  124.76  179.95  117.68  1.0949
IC C6   N6   C10   O10      1.4073  133.09  179.98  117.98  1.2198
IC O10  N6   *C10  C11      1.2198  117.98  179.97  122.31  1.4842
IC N6   C10  C11   H11      1.3309  122.31  -60.51  110.29  1.1095
IC H11  C10  *C11  H12      1.1095  110.29 -119.50  109.23  1.1106
IC H11  C10  *C11  H13      1.1095  110.29  121.05  110.28  1.1105
!ribose
IC -O3' P    O5'   C5'    1.6001  101.45  -39.25  119.00  1.4401
IC -O3' O5'  *P    O1P    1.6001  101.45 -115.82  109.74  1.4802
IC -O3' O5'  *P    O2P    1.6001  101.45  115.90  109.80  1.4801
IC  P   O5'  C5'   C4'    1.5996  119.00 -151.39  110.04  1.5160
IC O5'  C5'  C4'   C3'    1.4401  108.83 -179.85  116.10  1.5284
IC C5'  C4'  C3'   O3'    1.5160  116.10   76.70  115.12  1.4212
IC C4'  C3'  O3'   +P     1.5284  111.92  159.13  119.05  1.6001
IC C3'  O3'  +P    +O5'   1.4212  119.05  -98.86  101.45  1.5996
IC O4'  C3'  *C4'  C5'    1.4572  104.06 -120.04  116.10  1.5160
IC C2'  C4'  *C3'  O3'    1.5284  100.16 -124.08  115.12  1.4212
IC C4'  C3'  C2'   C1'    1.5284  100.16   39.58  102.04  1.5251
IC C3'  C2'  C1'   N9     1.5284  101.97  144.39  113.71  1.4896
IC O4'  C1'  N9    C4     1.5251  113.71  -97.2   125.59  1.3783
IC C1'  C3'  *C2'  O2'    1.5284  102.04 -114.67  110.81  1.4212
IC H2'  O2'  C2'   C3'    0.9600  114.97  148.63  111.92  1.5284
IC O4'  C2'  *C1'  H1'    0.0       0.0  -115.0     0.0   0.0
IC C1'  C3'  *C2'  H2''   0.0       0.0   115.0     0.0   0.0
IC C2'  C4'  *C3'  H3'    0.0       0.0   115.0     0.0   0.0
IC C3'  O4'  *C4'  H4'    0.0       0.0  -115.0     0.0   0.0
IC C4'  O5'  *C5'  H5'    0.0       0.0  -115.0     0.0   0.0
IC C4'  O5'  *C5'  H5''   0.0       0.0   115.0     0.0   0.0
DONO H2'  O2'
DONO H6   N6
ACCE N3
```

```
ACCE N7
ACCE N1
ACCE O10  C10
ACCE O1P  P
ACCE O2P  P
ACCE O2'
ACCE O3'
ACCE O4'
ACCE O5'

RESI 1MA            0.00 ! protonated 1-methyladenosine
GROUP
ATOM N9     NG2R51  0.00 !        H61  H62
ATOM C8     CG2R53  0.35 !          \  /
ATOM H8     HGR52   0.14 !           N6(+)
ATOM N7     NG2R50 -0.66 !           ||
ATOM C5     CG2RC0  0.10 !  H11      C6
ATOM C6     CG2R64  0.62 !     \    /  \
ATOM N6     NG2P1  -0.81 ! H12-CM1-N1   C5--N7\\
ATOM H61    HGP2    0.40 !     /   |    ||     C8-H8
ATOM H62    HGP2    0.40 !  H13    C2   C4--N9/
ATOM N1     NG2P1  -0.14 !        / \\ /     \
ATOM C2     CG2R64  0.28 !      H2   N3       \
ATOM H2     HGR62   0.17 !                     \
ATOM N3     NG2R62 -0.60 !                      \
ATOM C4     CG2RC0  0.55 !  O1P    H5' H4'  O4'  \
ATOM CM1    CG334  -0.07 !   |      |    \ /   \  \
ATOM H11    HGA3    0.09 !  -P-O5'-C5'---C4'    C1'
ATOM H12    HGA3    0.09 !   |      |     \     / \
ATOM H13    HGA3    0.09 !  O2P    H5''   C3'--C2' H1'
GROUP                    !               / \   / \
ATOM P      P       1.50 !             O3' H3' O2' H2''
ATOM O1P    ON3    -0.78 !              |       |
ATOM O2P    ON3    -0.78 !                     H2'
ATOM O5'    ON2    -0.57
ATOM C5'    CN8B   -0.08 !  !!!! PATCH 1MAN for the neutral bases
ATOM H5'    HN8     0.09
ATOM H5''   HN8     0.09
GROUP
ATOM C4'    CN7     0.16
ATOM H4'    HN7     0.09
ATOM O4'    ON6B   -0.50
ATOM C1'    CN7B    0.16
ATOM H1'    HN7     0.09
GROUP
ATOM C2'    CN7B    0.14
ATOM H2''   HN7     0.09
ATOM O2'    ON5    -0.66
ATOM H2'    HN5     0.43
GROUP
ATOM C3'    CN7     0.01
ATOM H3'    HN7     0.09
ATOM O3'    ON2    -0.57
BOND N9   C8   N9   C4   C8   H8   C8   N7
BOND N7   C5   C5   C6   C5   C4   C6   N6
BOND C6   N1   N6   H61  N6   H62  N1   C2
BOND N1   CM1  C2   H2   C2   N3   N3   C4
BOND CM1  H11  CM1  H12  CM1  H13
BOND P    O1P     P    O2P     P    O5'     O5'  C5'     C5'  H5''
BOND C5'  C4'     C4'  O4'     C4'  C3'     O4'  C1'
BOND C1'  N9      C1'  C2'     C2'  C3'     C3'  O3'     O3'  +P
BOND C2'  O2'     O2'  H2'
BOND C1'  H1'     C2'  H2''    C3'  H3'     C4'  H4'     C5'  H5'
```

```
IMPR C6   C5   N1   N6     N6    H62    H61    C6
!ribose
IC -O3' P    O5'   C5'    1.6001  101.45  -39.25  119.00  1.4401
IC -O3' O5'  *P    O1P    1.6001  101.45 -115.82  109.74  1.4802
IC -O3' O5'  *P    O2P    1.6001  101.45  115.90  109.80  1.4801
IC  P   O5'  C5'   C4'    1.5996  119.00 -151.39  110.04  1.5160
IC O5'  C5'  C4'   C3'    1.4401  108.83 -179.85  116.10  1.5284
IC C5'  C4'  C3'   O3'    1.5160  116.10   76.70  115.12  1.4212
IC C4'  C3'  O3'   +P     1.5284  111.92  159.13  119.05  1.6001
IC C3'  O3'  +P    +O5'   1.4212  119.05  -98.86  101.45  1.5996
IC O4'  C3'  *C4'  C5'    1.4572  104.06 -120.04  116.10  1.5160
IC C2'  C4'  *C3'  O3'    1.5284  100.16 -124.08  115.12  1.4212
IC C4'  C3'  C2'   C1'    1.5284  100.16   39.58  102.04  1.5251
IC C3'  C2'  C1'   N9     1.5284  101.97  144.39  113.71  1.4896
IC O4'  C1'  N9    C4     1.5251  113.71  -97.2   125.59  1.3783
IC C1'  C3'  *C2'  O2'    1.5284  102.04 -114.67  110.81  1.4212
IC H2'  O2'  C2'   C3'    0.9600  114.97  148.63  111.92  1.5284
IC O4'  C2'  *C1'  H1'    0.0       0.0  -115.0     0.0   0.0
IC C1'  C3'  *C2'  H2''   0.0       0.0   115.0     0.0   0.0
IC C2'  C4'  *C3'  H3'    0.0       0.0   115.0     0.0   0.0
IC C3'  O4'  *C4'  H4'    0.0       0.0  -115.0     0.0   0.0
IC C4'  O5'  *C5'  H5'    0.0       0.0  -115.0     0.0   0.0
IC C4'  O5'  *C5'  H5''   0.0       0.0   115.0     0.0   0.0
IC C8   C4   *N9   C1'       1.3791  105.54 -179.95  126.56  1.4896
IC C4   N9   C8    N7        1.3872  105.90    0.03  113.38  1.3302
IC N7   N9   *C8   H8        1.3302  113.38  179.97  121.71  1.0945
IC N9   C8   N7    C5        1.3821  113.38   -0.03  104.21  1.3867
IC C4   N7   *C5   C6        1.3984  110.82 -179.97  132.12  1.3843
IC N7   C5   C6    N1        1.3867  132.12  179.99  120.17  1.3977
IC N1   C5   *C6   N6        1.3977  120.17  179.99  122.15  1.3707
IC C5   C6   N6    H61       1.3843  122.15  179.99  124.05  0.9969
IC H61  C6   *N6   H62       0.9969  124.05  180.00  118.16  1.0028
IC C5   C6   N1    C2        1.3843  120.17    0.01  118.32  1.4122
IC C2   C6   *N1   CM1       1.4122  118.32  179.95  122.34  1.4780
IC C6   N1   C2    N3        1.3977  118.32   -0.03  122.77  1.3632
IC N3   N1   *C2   H2        1.3632  122.77 -179.97  118.51  1.0975
IC C6   N1   CM1   H11       1.3977  122.34   60.36  111.18  1.1146
IC H11  N1   *CM1  H12       1.1146  111.18  119.63  112.55  1.1146
IC H11  N1   *CM1  H13       1.1146  111.18 -120.65  111.23  1.1136
DONO H61  N6
DONO H62  N6
DONO H2'  O2'
ACCE N3
ACCE N7
ACCE O1P  P
ACCE O2P  P
ACCE O2'
ACCE O3'
ACCE O4'
ACCE O5'

RESI M2A            0.00 ! protonated 1,2'-O-dimethyladenosine
GROUP
ATOM N9     NG2R51  0.00 !         H61  H62
ATOM C8     CG2R53  0.35 !           \  /
ATOM H8     HGR52   0.14 !            N6(+)
ATOM N7     NG2R50 -0.66 !            ||
ATOM C5     CG2RC0  0.10 !   H11      C6
ATOM C6     CG2R64  0.62 !      \    /  \
ATOM N6     NG2P1  -0.81 !  H12-CM1-N1   C5--N7\\
ATOM H61    HGP2    0.40 !      /   |    ||     C8-H8
ATOM H62    HGP2    0.40 !   H13    C2   C4--N9/
ATOM N1     NG2P1  -0.14 !         / \\ /     \
```

```
ATOM C2     CG2R64  0.28 !       H2   N3       \
ATOM H2     HGR62   0.17 !                      \
ATOM N3     NG2R62 -0.60 !                       \
ATOM C4     CG2RC0  0.55 !   O1P    H5' H4'  O4'  \
ATOM CM1    CG334  -0.07 !    |      |    \ /   \  \
ATOM H11    HGA3    0.09 !   -P-O5'-C5'---C4'    C1'
ATOM H12    HGA3    0.09 !    |      |     \     / \
ATOM H13    HGA3    0.09 !   O2P    H5''   C3'--C2' H1'
GROUP                    !                / \   / \
ATOM P      P       1.50 !              O3' H3' O2' H2''
ATOM O1P    ON3    -0.78 !               |       |
ATOM O2P    ON3    -0.78 !                      CM2
ATOM O5'    ON2    -0.57 !                     /  |  \
ATOM C5'    CN8B   -0.08 !                   HM1 HM2 HM3
ATOM H5'    HN8     0.09
ATOM H5''   HN8     0.09 !  !!!! PATCH 1MAN for the neutral bases
GROUP
ATOM C4'    CN7     0.16
ATOM H4'    HN7     0.09
ATOM O4'    ON6B   -0.50
ATOM C1'    CN7B    0.16
ATOM H1'    HN7     0.09
GROUP
ATOM C2'    CN7B    0.08
ATOM H2''   HN7     0.09
ATOM O2'    OG301  -0.34
ATOM CM2    CG331  -0.10
ATOM HM1    HGA3    0.09
ATOM HM2    HGA3    0.09
ATOM HM3    HGA3    0.09
GROUP
ATOM C3'    CN7     0.01
ATOM H3'    HN7     0.09
ATOM O3'    ON2    -0.57
BOND N9   C8   N9   C4   C8   H8   C8   N7
BOND N7   C5   C5   C6   C5   C4   C6   N6
BOND C6   N1   N6   H61  N6   H62  N1   C2
BOND N1   CM1  C2   H2   C2   N3   N3   C4
BOND CM1  H11  CM1  H12  CM1  H13
BOND P    O1P     P    O2P     P    O5'     O5'  C5'     C5'  H5''
BOND C5'  C4'     C4'  O4'     C4'  C3'     O4'  C1'
BOND C1'  N9      C1'  C2'     C2'  C3'     C3'  O3'     O3'  +P
BOND C2'  O2'     CM2  O2'     CM2  HM1     HM2  CM2     HM3  CM2
BOND C1'  H1'     C2'  H2''    C3'  H3'     C4'  H4'     C5'  H5'
IMPR C6   C5   N1   N6    N6    H62    H61    C6
!2OM-ribose
IC -O3' P    O5'   C5'    1.6001  101.45  -39.25  119.00  1.4401
IC -O3' O5'  *P    O1P    1.6001  101.45 -115.82  109.74  1.4802
IC -O3' O5'  *P    O2P    1.6001  101.45  115.90  109.80  1.4801
IC  P   O5'  C5'   C4'    1.5996  119.00 -151.39  110.04  1.5160
IC O5'  C5'  C4'   C3'    1.4401  108.83 -179.85  116.10  1.5284
IC C5'  C4'  C3'   O3'    1.5160  116.10   76.70  115.12  1.4212
IC C4'  C3'  O3'   +P     1.5284  111.92  159.13  119.05  1.6001
IC C3'  O3'  +P    +O5'   1.4212  119.05  -98.86  101.45  1.5996
IC O4'  C3'  *C4'  C5'    1.4572  104.06 -120.04  116.10  1.5160
IC C2'  C4'  *C3'  O3'    1.5284  100.16 -124.08  115.12  1.4212
IC C4'  C3'  C2'   C1'    1.5284  100.16   39.58  102.04  1.5251
IC C3'  C2'  C1'   N9     1.5284  101.97  144.39  113.71  1.4896
IC O4'  C1'  N9    C4     1.5251  113.71  -97.2   125.59  1.3783
IC C3'  C1'  *C2'  O2'    1.5312  102.03  117.61  107.13  1.4206
IC C1'  C2'  O2'   CM2    1.5393  107.13   90.00  107.00  1.4150
IC C2'  O2'  CM2   HM2    1.4206  107.00  180.00    0.0   0.0
IC HM2  O2'  *CM2  HM3    0.0       0.0   120.00    0.0   0.0
```

```
IC HM2  O2'  *CM2  HM1    0.0       0.0  -120.00    0.0   0.0
IC O4'  C2'  *C1'  H1'    0.0       0.0  -115.0     0.0   0.0
IC C1'  C3'  *C2'  H2''   0.0       0.0   115.0     0.0   0.0
IC C2'  C4'  *C3'  H3'    0.0       0.0   115.0     0.0   0.0
IC C3'  O4'  *C4'  H4'    0.0       0.0  -115.0     0.0   0.0
IC C4'  O5'  *C5'  H5'    0.0       0.0  -115.0     0.0   0.0
IC C4'  O5'  *C5'  H5''   0.0       0.0   115.0     0.0   0.0
IC C8   C4   *N9   C1'       1.3791  105.54 -179.95  126.56  1.4896
IC C4   N9   C8    N7        1.3872  105.90    0.03  113.38  1.3302
IC N7   N9   *C8   H8        1.3302  113.38  179.97  121.71  1.0945
IC N9   C8   N7    C5        1.3821  113.38   -0.03  104.21  1.3867
IC C4   N7   *C5   C6        1.3984  110.82 -179.97  132.12  1.3843
IC N7   C5   C6    N1        1.3867  132.12  179.99  120.17  1.3977
IC N1   C5   *C6   N6        1.3977  120.17  179.99  122.15  1.3707
IC C5   C6   N6    H61       1.3843  122.15  179.99  124.05  0.9969
IC H61  C6   *N6   H62       0.9969  124.05  180.00  118.16  1.0028
IC C5   C6   N1    C2        1.3843  120.17    0.01  118.32  1.4122
IC C2   C6   *N1   CM1       1.4122  118.32  179.95  122.34  1.4780
IC C6   N1   C2    N3        1.3977  118.32   -0.03  122.77  1.3632
IC N3   N1   *C2   H2        1.3632  122.77 -179.97  118.51  1.0975
IC C6   N1   CM1   H11       1.3977  122.34   60.36  111.18  1.1146
IC H11  N1   *CM1  H12       1.1146  111.18  119.63  112.55  1.1146
IC H11  N1   *CM1  H13       1.1146  111.18 -120.65  111.23  1.1136
DONO H61  N6
DONO H62  N6
ACCE N3
ACCE N7
ACCE O1P  P
ACCE O2P  P
ACCE O2'
ACCE O3'
ACCE O4'
ACCE O5'

RESI 6IA           -1.00 ! N6-isopentenyladenosine
GROUP
ATOM N9     NG2R51 -0.01 !    H153      H161
ATOM C8     CG2R53  0.43 !      \      /
ATOM H8     HGR52   0.08 !  H152-C15  C16-H162
ATOM N7     NG2R50 -0.85 !       / \ / \
ATOM C5     CG2RC0  0.32 !    H151  C14 H163
ATOM C6     CG2R64  0.45 !          //
ATOM N6     NG311  -0.45 !    H13-C13
ATOM H6     HGPAM1  0.33 !          \
ATOM N1     NG2R62 -0.78 !      H121-C12   H6
ATOM C2     CG2R64  0.49 !           / \  /
ATOM H2     HGR62   0.14 !         H122 N6
ATOM N3     NG2R62 -0.86 !              |
ATOM C4     CG2RC0  0.52 !              C6
ATOM C12    CG321   0.01 !             // \
ATOM H121   HGA2    0.09 !           N1   C5--N7\\
ATOM H122   HGA2    0.09 !           |    ||     C8-H8
GROUP                    !           C2   C4--N9/
ATOM C13    CG2D1  -0.15 !          / \\ /      \
ATOM H13    HGA4    0.15 !         H2   N3        \
GROUP                    !                         \
ATOM C14    CG2D1   0.00 !                          \
ATOM C15    CG331  -0.27 !                           \
ATOM H151   HGA3    0.09 !       O1P    H5' H4'  O4'  \
ATOM H152   HGA3    0.09 !        |      |    \ /   \  \
ATOM H153   HGA3    0.09 !       -P-O5'-C5'---C4'    C1'
GROUP                    !        |      |     \     / \
ATOM C16    CG331  -0.27 !       O2P    H5''   C3'--C2' H1'
```

```
ATOM H161   HGA3    0.09 !                      / \   / \
ATOM H162   HGA3    0.09 !                    O3' H3' O2' H2''
ATOM H163   HGA3    0.09 !                     |       |
GROUP                    !                            H2'
ATOM P      P       1.50
ATOM O1P    ON3    -0.78
ATOM O2P    ON3    -0.78
ATOM O5'    ON2    -0.57
ATOM C5'    CN8B   -0.08
ATOM H5'    HN8     0.09
ATOM H5''   HN8     0.09
GROUP
ATOM C4'    CN7     0.16
ATOM H4'    HN7     0.09
ATOM O4'    ON6B   -0.50
ATOM C1'    CN7B    0.16
ATOM H1'    HN7     0.09
GROUP
ATOM C2'    CN7B    0.14
ATOM H2''   HN7     0.09
ATOM O2'    ON5    -0.66
ATOM H2'    HN5     0.43
GROUP
ATOM C3'    CN7     0.01
ATOM H3'    HN7     0.09
ATOM O3'    ON2    -0.57
BOND N9   C8     N9   C4      C8   N7      C8   H8
BOND N7   C5     C5   C6      C5   C4      C6   N6
BOND C6   N1     N6   C12     N6   H6      N1   C2
BOND C2   N3     C2   H2      N3   C4      C12  C13
BOND C12  H121   C12  H122    C13  C14     C13  H13
BOND C14  C15    C14  C16     C15  H151    C15  H152
BOND C15  H153   C16  H161    C16  H162    C16  H163
BOND P    O1P     P    O2P     P    O5'     O5'  C5'     C5'  H5''
BOND C5'  C4'     C4'  O4'     C4'  C3'     O4'  C1'
BOND C1'  N9      C1'  C2'     C2'  C3'     C3'  O3'     O3'  +P
BOND C2'  O2'     O2'  H2'
BOND C1'  H1'     C2'  H2''    C3'  H3'     C4'  H4'     C5'  H5'
IMPR C6   C5   N1   N6    ! N6   C6   C12   H6
!ribose
IC -O3' P    O5'   C5'    1.6001  101.45  -39.25  119.00  1.4401
IC -O3' O5'  *P    O1P    1.6001  101.45 -115.82  109.74  1.4802
IC -O3' O5'  *P    O2P    1.6001  101.45  115.90  109.80  1.4801
IC  P   O5'  C5'   C4'    1.5996  119.00 -151.39  110.04  1.5160
IC O5'  C5'  C4'   C3'    1.4401  108.83 -179.85  116.10  1.5284
IC C5'  C4'  C3'   O3'    1.5160  116.10   76.70  115.12  1.4212
IC C4'  C3'  O3'   +P     1.5284  111.92  159.13  119.05  1.6001
IC C3'  O3'  +P    +O5'   1.4212  119.05  -98.86  101.45  1.5996
IC O4'  C3'  *C4'  C5'    1.4572  104.06 -120.04  116.10  1.5160
IC C2'  C4'  *C3'  O3'    1.5284  100.16 -124.08  115.12  1.4212
IC C4'  C3'  C2'   C1'    1.5284  100.16   39.58  102.04  1.5251
IC C3'  C2'  C1'   N9     1.5284  101.97  144.39  113.71  1.4896
IC O4'  C1'  N9    C4     1.5251  113.71  -97.2   125.59  1.3783
IC C1'  C3'  *C2'  O2'    1.5284  102.04 -114.67  110.81  1.4212
IC H2'  O2'  C2'   C3'    0.9600  114.97  148.63  111.92  1.5284
IC O4'  C2'  *C1'  H1'    0.0       0.0  -115.0     0.0   0.0
IC C1'  C3'  *C2'  H2''   0.0       0.0   115.0     0.0   0.0
IC C2'  C4'  *C3'  H3'    0.0       0.0   115.0     0.0   0.0
IC C3'  O4'  *C4'  H4'    0.0       0.0  -115.0     0.0   0.0
IC C4'  O5'  *C5'  H5'    0.0       0.0  -115.0     0.0   0.0
IC C4'  O5'  *C5'  H5''   0.0       0.0   115.0     0.0   0.0
IC C8   C4   *N9   C1'       1.3791  105.54 -179.95  126.56  1.4896
IC C4   N9   C8    N7        1.3488  107.41    0.12  112.88  1.3252
```

```
IC N7   N9   *C8   H8        1.3252  112.88  179.79  122.68  1.0945
IC N9   C8   N7    C5        1.3734  112.88   -0.30  103.57  1.3989
IC C4   N7   *C5   C6        1.3955  110.29  179.29  133.05  1.4037
IC N7   C5   C6    N6        1.3989  133.05   -0.10  122.45  1.3768
IC N6   C5   *C6   N1        1.3768  122.45 -177.29  117.73  1.3785
IC C5   C6   N6    C12       1.4037  122.45  162.61  163.00  3.3322
IC C12  C6   *N6   H6        3.3322  163.00  176.69  115.17  1.0168
IC C5   C6   N1    C2        1.4037  117.73   -2.38  119.95  1.3644
IC C6   N1   C2    N3        1.3785  119.95    1.68  125.05  1.3610
IC N3   N1   *C2   H2        1.3610  125.05  179.00  117.44  1.0941
IC C6   N6   C12   C13       1.3768  163.00  166.36  143.14  1.5047
IC C13  N6   *C12  H121      1.5047  143.14  120.00  109.50  1.1110
IC C13  N6   *C12  H122      1.5047  143.14 -120.00  109.50  1.1110
IC N6   C12  C13   C14       3.3322  143.14  134.88   32.82  2.5294
IC C14  C12  *C13  H13       2.5294   32.82  180.00  116.00  1.1000
IC C12  C13  C14   C15       1.5047   32.82  180.00  123.50  1.5040
IC C15  C13  *C14  C16       1.5040  123.50  180.00  123.50  1.5040
IC C13  C14  C15   H151      2.5294  123.50  180.00  111.50  1.1110
IC H151 C14  *C15  H152      1.1110  111.50  120.00  111.50  1.1110
IC H151 C14  *C15  H153      1.1110  111.50 -120.00  111.50  1.1110
IC C13  C14  C16   H161      2.5294  123.50  180.00  111.50  1.1110
IC H161 C14  *C16  H162      1.1110  111.50  120.00  111.50  1.1110
IC H161 C14  *C16  H163      1.1110  111.50 -120.00  111.50  1.1110
DONO H2'  O2'
DONO H6   N6
ACCE N3
ACCE N7
ACCE N1
ACCE O1P  P
ACCE O2P  P
ACCE O2'
ACCE O3'
ACCE O4'
ACCE O5'

RESI HIA            -1.00 ! N6-(cis-hydroxyisopentenyl)adenosine
GROUP
ATOM N9     NG2R51 -0.01 !    H153      H161
ATOM C8     CG2R53  0.43 !       \      /
ATOM H8     HGR52   0.08 !  H152-C15  C16-H162
ATOM N7     NG2R50 -0.85 !       /  \ / \
ATOM C5     CG2RC0  0.32 !      H151 C14 O16-H16O
ATOM C6     CG2R64  0.45 !          //
ATOM N6     NG311  -0.45 !    H13-C13
ATOM H6     HGPAM1  0.33 !           \
ATOM N1     NG2R62 -0.78 !      H121-C12  H6
ATOM C2     CG2R64  0.49 !            / \  /
ATOM H2     HGR62   0.14 !         H122 N6
ATOM N3     NG2R62 -0.86 !               |
ATOM C4     CG2RC0  0.52 !               C6
ATOM C12    CG321   0.01 !              // \
ATOM H121   HGA2    0.09 !             N1   C5--N7\\
ATOM H122   HGA2    0.09 !             |    ||     C8-H8
GROUP                    !             C2   C4--N9/
ATOM C13    CG2D1  -0.15 !            / \\ /      \
ATOM H13    HGA4    0.15 !         H2   N3         \
GROUP                    !                          \
ATOM C14    CG2D1   0.00 !                           \
ATOM C15    CG331  -0.27 !                            \
ATOM H151   HGA3    0.09 !        O1P    H5' H4'  O4'  \
ATOM H152   HGA3    0.09 !         |      |    \ /   \  \
ATOM H153   HGA3    0.09 !        -P-O5'-C5'---C4'    C1'
GROUP                    !         |      |     \     / \
```

```
ATOM C16    CG321   0.05 !       O2P    H5''   C3'--C2' H1'
ATOM H161   HGA2    0.09 !                      / \   / \
ATOM H162   HGA2    0.09 !                    O3' H3' O2' H2''
ATOM O16    OG311  -0.65 !                     |       |
ATOM H16O   HGP1    0.42 !                            H2'
GROUP
ATOM P      P       1.50
ATOM O1P    ON3    -0.78
ATOM O2P    ON3    -0.78
ATOM O5'    ON2    -0.57
ATOM C5'    CN8B   -0.08
ATOM H5'    HN8     0.09
ATOM H5''   HN8     0.09
GROUP
ATOM C4'    CN7     0.16
ATOM H4'    HN7     0.09
ATOM O4'    ON6B   -0.50
ATOM C1'    CN7B    0.16
ATOM H1'    HN7     0.09
GROUP
ATOM C2'    CN7B    0.14
ATOM H2''   HN7     0.09
ATOM O2'    ON5    -0.66
ATOM H2'    HN5     0.43
GROUP
ATOM C3'    CN7     0.01
ATOM H3'    HN7     0.09
ATOM O3'    ON2    -0.57
BOND N9   C8     N9   C4      C8   N7      C8   H8
BOND N7   C5     C5   C6      C5   C4      C6   N6
BOND C6   N1     N6   C12     N6   H6      N1   C2
BOND C2   N3     C2   H2      N3   C4      C12  C13
BOND C12  H121   C12  H122    C13  C14     C13  H13
BOND C14  C15    C14  C16     C15  H151    C15  H152
BOND C15  H153   C16  O16     C16  H161    C16  H162
BOND O16  H16O
BOND P    O1P     P    O2P     P    O5'     O5'  C5'     C5'  H5''
BOND C5'  C4'     C4'  O4'     C4'  C3'     O4'  C1'
BOND C1'  N9      C1'  C2'     C2'  C3'     C3'  O3'     O3'  +P
BOND C2'  O2'     O2'  H2'
BOND C1'  H1'     C2'  H2''    C3'  H3'     C4'  H4'     C5'  H5'
IMPR C6   C5   N1   N6     ! N6   C6   C12   H6
!ribose
IC -O3' P    O5'   C5'    1.6001  101.45  -39.25  119.00  1.4401
IC -O3' O5'  *P    O1P    1.6001  101.45 -115.82  109.74  1.4802
IC -O3' O5'  *P    O2P    1.6001  101.45  115.90  109.80  1.4801
IC  P   O5'  C5'   C4'    1.5996  119.00 -151.39  110.04  1.5160
IC O5'  C5'  C4'   C3'    1.4401  108.83 -179.85  116.10  1.5284
IC C5'  C4'  C3'   O3'    1.5160  116.10   76.70  115.12  1.4212
IC C4'  C3'  O3'   +P     1.5284  111.92  159.13  119.05  1.6001
IC C3'  O3'  +P    +O5'   1.4212  119.05  -98.86  101.45  1.5996
IC O4'  C3'  *C4'  C5'    1.4572  104.06 -120.04  116.10  1.5160
IC C2'  C4'  *C3'  O3'    1.5284  100.16 -124.08  115.12  1.4212
IC C4'  C3'  C2'   C1'    1.5284  100.16   39.58  102.04  1.5251
IC C3'  C2'  C1'   N9     1.5284  101.97  144.39  113.71  1.4896
IC O4'  C1'  N9    C4     1.5251  113.71  -97.2   125.59  1.3783
IC C1'  C3'  *C2'  O2'    1.5284  102.04 -114.67  110.81  1.4212
IC H2'  O2'  C2'   C3'    0.9600  114.97  148.63  111.92  1.5284
IC O4'  C2'  *C1'  H1'    0.0       0.0  -115.0     0.0   0.0
IC C1'  C3'  *C2'  H2''   0.0       0.0   115.0     0.0   0.0
IC C2'  C4'  *C3'  H3'    0.0       0.0   115.0     0.0   0.0
IC C3'  O4'  *C4'  H4'    0.0       0.0  -115.0     0.0   0.0
IC C4'  O5'  *C5'  H5'    0.0       0.0  -115.0     0.0   0.0
```

```
IC C4'  O5'  *C5'  H5''   0.0       0.0   115.0     0.0   0.0
IC C8   C4   *N9  C1'      1.3791  105.54 -179.95  126.56  1.4896
IC C4   N9   C8   N7       1.3840  105.95    0.04  114.27  1.3284
IC N7   N9   *C8  H8       1.3284  114.27 -180.00  121.40  1.0910
IC N9   C8   N7   C5       1.3841  114.27    0.05  103.02  1.4043
IC C4   N7   *C5  C6       1.3995  111.22 -179.75  132.21  1.4055
IC N7   C5   C6   N6       1.4043  132.21   -0.27  120.44  1.3769
IC N6   C5   *C6  N1       1.3769  120.44  179.85  117.93  1.3739
IC C5   C6   N6   C12      1.4055  120.44  170.84  125.43  1.4875
IC C12  C6   *N6  H6       1.4875  125.43 -166.59  114.06  1.0188
IC C5   C6   N1   C2       1.4055  117.93    0.33  120.60  1.3594
IC C6   N1   C2   N3       1.3739  120.60   -0.41  124.63  1.3552
IC N3   N1   *C2  H2       1.3552  124.63 -179.72  117.61  1.0953
IC C6   N6   C12  C13      1.3769  125.43 -113.26  109.09  1.5101
IC C13  N6   *C12 H121     1.5101  109.09  124.02  111.43  1.1110
IC C13  N6   *C12 H122     1.5101  109.09 -120.07  108.81  1.1134
IC N6   C12  C13  C14      1.4875  109.09   91.95  128.35  1.3533
IC C14  C12  *C13 H13      1.3533  128.35 -179.11  113.80  1.0993
IC C12  C13  C14  C16      1.5101  128.35 -179.55  120.03  1.5125
IC C16  C13  *C14 C15      1.5125  120.03  179.56  122.63  1.5115
IC C13  C14  C15  H151     1.3533  122.63  116.44  110.82  1.1109
IC H151 C14  *C15 H152     1.1109  110.82  119.40  110.61  1.1109
IC H151 C14  *C15 H153     1.1109  110.82 -120.60  113.84  1.1108
IC C13  C14  C16  O16      1.3533  120.03 -111.71  110.18  1.4250
IC O16  C14  *C16 H161     1.4250  110.18  119.77  112.54  1.1152
IC O16  C14  *C16 H162     1.4250  110.18 -120.91  110.50  1.1138
IC C14  C16  O16  H16O     1.5125  110.18  -67.87  106.83  0.9618
DONO H2'  O2'
DONO H6   N6
DONO H16O O16
ACCE N3
ACCE N7
ACCE N1
ACCE O16
ACCE O1P  P
ACCE O2P  P
ACCE O2'
ACCE O3'
ACCE O4'
ACCE O5'

RESI MIA           -1.00 ! 2-methylthio-N6-isopentenyladenosine, SPA
GROUP
ATOM N9     NG2R51 -0.01 !    H151       H161
ATOM C8     CG2R53  0.37 !      \        /
ATOM H8     HGR52   0.10 !  H152-C15   C16-H162
ATOM N7     NG2R50 -0.84 !       / \   / \
ATOM C5     CG2RC0  0.28 !     H153 C14  H163
ATOM C6     CG2R64  0.38 !          //
ATOM N6     NG311  -0.48 !    H130-C13
ATOM H6     HGPAM1  0.36 !           \
ATOM N1     NG2R62 -0.58 !       H101-C12  H6
ATOM C2     CG2R64  0.63 !            / \  /
ATOM N3     NG2R62 -0.76 !         H102  N6
ATOM C4     CG2RC0  0.48 !               |
ATOM C12    CG321  -0.00 !               C6
ATOM H101   HGA2    0.09 !              // \
ATOM H102   HGA2    0.09 !     H11     N1   C5--N7\\
ATOM S10    SG311  -0.28 !      \      |    ||     C8-H8
ATOM C11    CG331  -0.10 !  H12 -C11   C2   C4--N9/
ATOM H11    HGA3    0.09 !       / \  / \\ /      \
ATOM H12    HGA3    0.09 !    H13   S10  N3        \
ATOM H13    HGA3    0.09 !                          \
```

```
GROUP                       !                            \
ATOM C13    CG2D1  -0.15 !       O1P    H5' H4'  O4'  \
ATOM H130   HGA4    0.15 !        |      |    \ /   \  \
GROUP                       !       -P-O5'-C5'---C4'    C1'
ATOM C14    CG2D1   0.00 !        |      |     \     / \
ATOM C15    CG331  -0.27 !       O2P    H5''   C3'--C2' H1'
ATOM H151   HGA3    0.09 !                      / \   / \
ATOM H152   HGA3    0.09 !                   O3' H3' O2' H2''
ATOM H153   HGA3    0.09 !                    |       |
GROUP                       !                             H2'
ATOM C16    CG331  -0.27
ATOM H161   HGA3    0.09
ATOM H162   HGA3    0.09
ATOM H163   HGA3    0.09
GROUP
ATOM P      P       1.50
ATOM O1P    ON3    -0.78
ATOM O2P    ON3    -0.78
ATOM O5'    ON2    -0.57
ATOM C5'    CN8B   -0.08
ATOM H5'    HN8     0.09
ATOM H5''   HN8     0.09
GROUP
ATOM C4'    CN7     0.16
ATOM H4'    HN7     0.09
ATOM O4'    ON6B   -0.50
ATOM C1'    CN7B    0.16
ATOM H1'    HN7     0.09
GROUP
ATOM C2'    CN7B    0.14
ATOM H2''   HN7     0.09
ATOM O2'    ON5    -0.66
ATOM H2'    HN5     0.43
GROUP
ATOM C3'    CN7     0.01
ATOM H3'    HN7     0.09
ATOM O3'    ON2    -0.57
BOND N9   C8     N9   C4      C8   N7      C8   H8
BOND N7   C5     C5   C6      C5   C4      C6   N6
BOND C6   N1     N6   H6      N6   C12     N1   C2
BOND C2   N3     C2   S10     N3   C4      S10  C11
BOND C11  H11    C11  H12     C11  H13     C12  C13
BOND C12  H101   C12  H102    C13  C14     C13  H130
BOND C14  C15    C14  C16     C15  H151    C15  H152
BOND C15  H153   C16  H161    C16  H162    C16  H163
BOND P    O1P     P    O2P     P    O5'     O5'  C5'     C5'  H5''
BOND C5'  C4'     C4'  O4'     C4'  C3'     O4'  C1'
BOND C1'  N9      C1'  C2'     C2'  C3'     C3'  O3'     O3'  +P
BOND C2'  O2'     O2'  H2'
BOND C1'  H1'     C2'  H2''    C3'  H3'     C4'  H4'     C5'  H5'
IMPR C6   C5   N1   N6    ! N6   C6   C12   H6
!ribose
IC -O3' P    O5'   C5'    1.6001  101.45  -39.25  119.00  1.4401
IC -O3' O5'  *P    O1P    1.6001  101.45 -115.82  109.74  1.4802
IC -O3' O5'  *P    O2P    1.6001  101.45  115.90  109.80  1.4801
IC  P   O5'  C5'   C4'    1.5996  119.00 -151.39  110.04  1.5160
IC O5'  C5'  C4'   C3'    1.4401  108.83 -179.85  116.10  1.5284
IC C5'  C4'  C3'   O3'    1.5160  116.10   76.70  115.12  1.4212
IC C4'  C3'  O3'   +P     1.5284  111.92  159.13  119.05  1.6001
IC C3'  O3'  +P    +O5'   1.4212  119.05  -98.86  101.45  1.5996
IC O4'  C3'  *C4'  C5'    1.4572  104.06 -120.04  116.10  1.5160
IC C2'  C4'  *C3'  O3'    1.5284  100.16 -124.08  115.12  1.4212
IC C4'  C3'  C2'   C1'    1.5284  100.16   39.58  102.04  1.5251
```

```
IC C3'  C2'  C1'   N9     1.5284  101.97  144.39  113.71  1.4896
IC O4'  C1'  N9    C4     1.5251  113.71  -97.2   125.59  1.3783
IC C1'  C3'  *C2'  O2'    1.5284  102.04 -114.67  110.81  1.4212
IC H2'  O2'  C2'   C3'    0.9600  114.97  148.63  111.92  1.5284
IC O4'  C2'  *C1'  H1'    0.0       0.0  -115.0     0.0   0.0
IC C1'  C3'  *C2'  H2''   0.0       0.0   115.0     0.0   0.0
IC C2'  C4'  *C3'  H3'    0.0       0.0   115.0     0.0   0.0
IC C3'  O4'  *C4'  H4'    0.0       0.0  -115.0     0.0   0.0
IC C4'  O5'  *C5'  H5'    0.0       0.0  -115.0     0.0   0.0
IC C4'  O5'  *C5'  H5''   0.0       0.0   115.0     0.0   0.0
IC C8   C4   *N9   C1'       1.3791  105.54 -179.95  126.56  1.4896
IC C4   N9   C8    N7        1.3836  105.94    0.03  114.07  1.3294
IC N7   N9   *C8   H8        1.3294  114.07  179.98  121.45  1.0917
IC N9   C8   N7    C5        1.3842  114.07    0.05  103.34  1.3988
IC C4   N7   *C5   C6        1.4023  111.06 -179.60  131.79  1.4058
IC N7   C5   C6    N1        1.3988  131.79  179.65  117.74  1.3733
IC N1   C5   *C6   N6        1.3733  117.74 -179.47  120.18  1.3781
IC C5   C6   N6    C12       1.4058  120.18  168.21  125.18  1.4886
IC C12  C6   *N6   H6        1.4886  125.18 -162.39  113.44  1.0199
IC C5   C6   N1    C2        1.4058  117.74   -0.07  119.87  1.3615
IC C6   N1   C2    S10       1.3733  119.87 -179.88  118.15  1.7691
IC S10  N1   *C2   N3        1.7691  118.15  179.95  125.77  1.3536
IC C6   N6   C12   C13       1.3781  125.18 -108.88  109.07  1.5095
IC C13  N6   *C12  H101      1.5095  109.07  123.91  111.37  1.1110
IC C13  N6   *C12  H102      1.5095  109.07 -120.02  108.74  1.1141
IC N1   C2   S10   C11       1.3615  118.15   -0.10  103.91  1.8250
IC C2   S10  C11   H11       1.7691  103.91 -179.44  110.73  1.1114
IC H11  S10  *C11  H12       1.1114  110.73  119.72  111.33  1.1117
IC H11  S10  *C11  H13       1.1114  110.73 -119.75  111.29  1.1114
IC N6   C12  C13   C14       1.4886  109.07   92.82  128.04  1.3484
IC C14  C12  *C13  H130      1.3484  128.04 -175.41  114.16  1.0990
IC C12  C13  C14   C15       1.5095  128.04 -179.59  121.05  1.5044
IC C15  C13  *C14  C16       1.5044  121.05  179.62  124.75  1.5050
IC C13  C14  C15   H151      1.3484  121.05 -120.92  110.55  1.1110
IC H151 C14  *C15  H152      1.1110  110.55  120.65  113.27  1.1123
IC H151 C14  *C15  H153      1.1110  110.55 -118.82  110.53  1.1111
IC C13  C14  C16   H161      1.3484  124.75  120.24  110.66  1.1108
IC H161 C14  *C16  H162      1.1108  110.66  118.99  110.48  1.1113
IC H161 C14  *C16  H163      1.1108  110.66 -120.87  113.54  1.1097
DONO H2'  O2'
DONO H6   N6
ACCE N3
ACCE N7
ACCE N1
ACCE O1P  P
ACCE O2P  P
ACCE O2'
ACCE O3'
ACCE O4'
ACCE O5'

RESI SIA           -1.00 ! 2-methylthio-N6-(cis-hydroxyisopentenyl) adenosine
GROUP
ATOM N9     NG2R51 -0.01 !     H153      H161
ATOM C8     CG2R53  0.37 !        \        /
ATOM H8     HGR52   0.10 !   H152-C15  C16-H162
ATOM N7     NG2R50 -0.84 !        / \  / \
ATOM C5     CG2RC0  0.28 !      H151 C14  O16-H16O
ATOM C6     CG2R64  0.38 !           //
ATOM N6     NG311  -0.48 !     H130-C13
ATOM H6     HGPAM1  0.36 !            \
ATOM N1     NG2R62 -0.58 !        H121-C12  H6
ATOM C2     CG2R64  0.63 !             / \  /
```

```
ATOM N3     NG2R62 -0.76 !           H122 N6
ATOM C4     CG2RC0  0.48 !                |
ATOM C12    CG321  -0.00 !                C6
ATOM H121   HGA2    0.09 !               // \
ATOM H122   HGA2    0.09 !     H11      N1   C5--N7\\
ATOM S2     SG311  -0.28 !        \      |    ||      C8-H8
ATOM C11    CG331  -0.10 !   H12 -C11   C2   C4--N9/
ATOM H11    HGA3    0.09 !         / \  / \\ /      \
ATOM H12    HGA3    0.09 !      H13  S2   N3         \
ATOM H13    HGA3    0.09 !                            \
GROUP                    !                             \
ATOM C13    CG2D1  -0.15 !                              \
ATOM H130   HGA4    0.15 !          O1P    H5' H4'  O4'  \
GROUP                    !           |      |    \ /   \  \
ATOM C14    CG2D1   0.00 !          -P-O5'-C5'---C4'    C1'
ATOM C15    CG331  -0.27 !           |      |     \     / \
ATOM H151   HGA3    0.09 !          O2P    H5''   C3'--C2' H1'
ATOM H152   HGA3    0.09 !                       / \   / \
ATOM H153   HGA3    0.09 !                     O3' H3' O2' H2''
GROUP                    !                      |       |
ATOM C16    CG321   0.05 !                             H2'
ATOM H161   HGA2    0.09
ATOM H162   HGA2    0.09
ATOM O16    OG311  -0.65
ATOM H16O   HGP1    0.42
GROUP
ATOM P      P       1.50
ATOM O1P    ON3    -0.78
ATOM O2P    ON3    -0.78
ATOM O5'    ON2    -0.57
ATOM C5'    CN8B   -0.08
ATOM H5'    HN8     0.09
ATOM H5''   HN8     0.09
GROUP
ATOM C4'    CN7     0.16
ATOM H4'    HN7     0.09
ATOM O4'    ON6B   -0.50
ATOM C1'    CN7B    0.16
ATOM H1'    HN7     0.09
GROUP
ATOM C2'    CN7B    0.14
ATOM H2''   HN7     0.09
ATOM O2'    ON5    -0.66
ATOM H2'    HN5     0.43
GROUP
ATOM C3'    CN7     0.01
ATOM H3'    HN7     0.09
ATOM O3'    ON2    -0.57
BOND N9   C8     N9   C4      C8   N7      C8   H8
BOND N7   C5     C5   C6      C5   C4      C6   N6
BOND C6   N1     N6   C12     N6   H6      N1   C2
BOND C2   N3     C2   S2      N3   C4      C12  H121
BOND C12  C13    C12  H122    C13  H130    C13  C14
BOND C14  C15    C14  C16     C15  H151    C15  H152
BOND C15  H153   C16  O16     C16  H162    C16  H161
BOND O16  H16O   S2   C11     C11  H13     C11  H12
BOND C11  H11
BOND P    O1P     P    O2P     P    O5'     O5'  C5'     C5'  H5''
BOND C5'  C4'     C4'  O4'     C4'  C3'     O4'  C1'
BOND C1'  N9      C1'  C2'     C2'  C3'     C3'  O3'     O3'  +P
BOND C2'  O2'     O2'  H2'
BOND C1'  H1'     C2'  H2''    C3'  H3'     C4'  H4'     C5'  H5'
IMPR C6   C5   N1   N6     ! N6   C6   C12   H6
```

```
!ribose
IC -O3' P    O5'   C5'    1.6001  101.45  -39.25  119.00  1.4401
IC -O3' O5'  *P    O1P    1.6001  101.45 -115.82  109.74  1.4802
IC -O3' O5'  *P    O2P    1.6001  101.45  115.90  109.80  1.4801
IC  P   O5'  C5'   C4'    1.5996  119.00 -151.39  110.04  1.5160
IC O5'  C5'  C4'   C3'    1.4401  108.83 -179.85  116.10  1.5284
IC C5'  C4'  C3'   O3'    1.5160  116.10   76.70  115.12  1.4212
IC C4'  C3'  O3'   +P     1.5284  111.92  159.13  119.05  1.6001
IC C3'  O3'  +P    +O5'   1.4212  119.05  -98.86  101.45  1.5996
IC O4'  C3'  *C4'  C5'    1.4572  104.06 -120.04  116.10  1.5160
IC C2'  C4'  *C3'  O3'    1.5284  100.16 -124.08  115.12  1.4212
IC C4'  C3'  C2'   C1'    1.5284  100.16   39.58  102.04  1.5251
IC C3'  C2'  C1'   N9     1.5284  101.97  144.39  113.71  1.4896
IC O4'  C1'  N9    C4     1.5251  113.71  -97.2   125.59  1.3783
IC C1'  C3'  *C2'  O2'    1.5284  102.04 -114.67  110.81  1.4212
IC H2'  O2'  C2'   C3'    0.9600  114.97  148.63  111.92  1.5284
IC O4'  C2'  *C1'  H1'    0.0       0.0  -115.0     0.0   0.0
IC C1'  C3'  *C2'  H2''   0.0       0.0   115.0     0.0   0.0
IC C2'  C4'  *C3'  H3'    0.0       0.0   115.0     0.0   0.0
IC C3'  O4'  *C4'  H4'    0.0       0.0  -115.0     0.0   0.0
IC C4'  O5'  *C5'  H5'    0.0       0.0  -115.0     0.0   0.0
IC C4'  O5'  *C5'  H5''   0.0       0.0   115.0     0.0   0.0
IC C8   C4   *N9   C1'       1.3791  105.54 -179.95  126.56  1.4896
IC C4   N9   C8    N7        1.3834  105.92   -0.00  114.15  1.3291
IC N7   N9   *C8   H8        1.3291  114.15 -180.00  121.40  1.0925
IC N9   C8   N7    C5        1.3837  114.15   -0.09  103.27  1.3996
IC C4   N7   *C5   C6        1.4021  111.07  179.68  131.76  1.4057
IC N7   C5   C6    N6        1.3996  131.76    0.01  120.19  1.3779
IC N6   C5   *C6   N1        1.3779  120.19 -179.48  117.70  1.3724
IC C5   C6   N6    C12       1.4057  120.19 -169.04  125.33  1.4880
IC C12  C6   *N6   H6        1.4880  125.33  164.08  113.56  1.0202
IC C5   C6   N1    C2        1.4057  117.70   -0.18  119.94  1.3603
IC C6   N1   C2    S2        1.3724  119.94  180.00  118.23  1.7695
IC S2   N1   *C2   N3        1.7695  118.23 -179.93  125.78  1.3535
IC C6   N6   C12   C13       1.3779  125.33  108.98  109.05  1.5096
IC C13  N6   *C12  H121      1.5096  109.05  119.93  108.74  1.1139
IC H121 N6   *C12  H122      1.1139  108.74  115.76  111.41  1.1104
IC N1   C2   S2    C11       1.3603  118.23   -0.18  103.94  1.8251
IC C2   S2   C11   H13       1.7695  103.94  179.31  110.78  1.1111
IC H13  S2   *C11  H12       1.1111  110.78  119.72  111.34  1.1118
IC H13  S2   *C11  H11       1.1111  110.78 -119.75  111.32  1.1119
IC N6   C12  C13   C14       1.4880  109.05  -94.07  128.53  1.3531
IC C14  C12  *C13  H130      1.3531  128.53  179.15  113.69  1.0998
IC C12  C13  C14   C16       1.5096  128.53  179.69  120.07  1.5138
IC C16  C13  *C14  C15       1.5138  120.07 -179.85  122.81  1.5105
IC C13  C14  C15   H151      1.3531  122.81 -119.83  110.11  1.1117
IC H151 C14  *C15  H152      1.1117  110.11  120.71  114.11  1.1088
IC H151 C14  *C15  H153      1.1117  110.11 -118.59  110.62  1.1105
IC C13  C14  C16   O16       1.3531  120.07  111.88  110.15  1.4272
IC O16  C14  *C16  H162      1.4272  110.15  119.79  109.35  1.1135
IC O16  C14  *C16  H161      1.4272  110.15 -121.03  112.62  1.1141
IC C14  C16  O16   H16O      1.5138  110.15  170.65  107.97  0.9613
DONO H2'  O2'
DONO H6   N6
DONO H16O O16
ACCE N3
ACCE N7
ACCE N1
ACCE O16
ACCE O1P  P
ACCE O2P  P
ACCE O2'
ACCE O3'
```

```
ACCE O4'
ACCE O5'

RESI HNA           -2.00 ! N6-hydroxynorvalylcarbamoyladenosine
GROUP
ATOM N9     NG2R51 -0.03 !                     H161 H162
ATOM C8     CG2R53  0.34 !                       |  /
ATOM H8     HGR52   0.12 !                H151 C16-H163
ATOM N7     NG2R50 -0.75 !                    \ /
ATOM C5     CG2RC0  0.28 !             H152-C15   O14-H14O
ATOM C6     CG2R64  0.70 !                      \  /
ATOM N6     NG2S1  -0.58 !            O10      C14-H14
ATOM H6     HGP1    0.33 !            ||       /
ATOM N1     NG2R62 -0.74 !       H6   C10  C12-H12
ATOM C2     CG2R64  0.49 !        \  /  \  /  \
ATOM H2     HGR62   0.12 !          N6    N11   C13-ODA (-)
ATOM N3     NG2R62 -0.78 !          |      |     ||
ATOM C4     CG2RC0  0.41 !          |     H11   ODB
ATOM C10    CG2O6   0.60 !          C6
ATOM O10    OG2D1  -0.50 !        // \
ATOM N11    NG2S1  -0.48 !       N1   C5--N7\\
ATOM H11    HGP1    0.29 !       |    ||     C8-H8
ATOM C12    CG311   0.09 !       C2   C4--N9/
ATOM H12    HGA1    0.09 !      / \\ /      \
GROUP                    !    H2   N3         \
ATOM C13    CG2O3   0.52 !                     \
ATOM ODA    OG2D2  -0.76 !                      \
ATOM ODB    OG2D2  -0.76 !                       \
GROUP                    !  O1P    H5' H4'  O4'  \
ATOM C14    CG311   0.14 !   |      |    \ /   \  \
ATOM H14    HGA1    0.09 !  -P-O5'-C5'---C4'    C1'
ATOM O14    OG311  -0.65 !   |      |     \     / \
ATOM H14O   HGP1    0.42 !  O2P    H5''   C3'--C2' H1'
ATOM C15    CG321  -0.18 !               / \   / \
ATOM H151   HGA2    0.09 !             O3' H3' O2' H2''
ATOM H152   HGA2    0.09 !              |       |
ATOM C16    CG331  -0.27 !                     H2'
ATOM H161   HGA3    0.09
ATOM H162   HGA3    0.09
ATOM H163   HGA3    0.09
GROUP
ATOM P      P       1.50
ATOM O1P    ON3    -0.78
ATOM O2P    ON3    -0.78
ATOM O5'    ON2    -0.57
ATOM C5'    CN8B   -0.08
ATOM H5'    HN8     0.09
ATOM H5''   HN8     0.09
GROUP
ATOM C4'    CN7     0.16
ATOM H4'    HN7     0.09
ATOM O4'    ON6B   -0.50
ATOM C1'    CN7B    0.16
ATOM H1'    HN7     0.09
GROUP
ATOM C2'    CN7B    0.14
ATOM H2''   HN7     0.09
ATOM O2'    ON5    -0.66
ATOM H2'    HN5     0.43
GROUP
ATOM C3'    CN7     0.01
ATOM H3'    HN7     0.09
ATOM O3'    ON2    -0.57
```

```
BOND N9   C8      N9   C4      C8   N7      C8   H8
BOND N7   C5      C5   C6      C5   C4      C6   N6
BOND C6   N1      N6   C10     N6   H6      N1   C2
BOND C2   N3      C2   H2      N3   C4      C10  N11
BOND C10  O10     N11  C12     N11  H11     C12  C14
BOND C12  C13     C12  H12     C14  C15     C14  O14
BOND C14  H14     C15  C16     C15  H151    C15  H152
BOND C16  H161    C16  H162    C16  H163    C13  ODA
BOND C13  ODB     O14  H14O
BOND P    O1P      P    O2P      P    O5'      O5'  C5'      C5'  H5''
BOND C5'  C4'      C4'  O4'      C4'  C3'      O4'  C1'
BOND C1'  N9       C1'  C2'      C2'  C3'      C3'  O3'      O3'  +P
BOND C2'  O2'      O2'  H2'
BOND C1'  H1'      C2'  H2''     C3'  H3'      C4'  H4'      C5'  H5'
IMPR C10    N6     N11    O10    C13    ODB    ODA    C12     C6   C5   N1   N6
!ribose
IC -O3' P    O5'   C5'    1.6001  101.45  -39.25  119.00  1.4401
IC -O3' O5'  *P    O1P    1.6001  101.45 -115.82  109.74  1.4802
IC -O3' O5'  *P    O2P    1.6001  101.45  115.90  109.80  1.4801
IC  P   O5'  C5'   C4'    1.5996  119.00 -151.39  110.04  1.5160
IC O5'  C5'  C4'   C3'    1.4401  108.83 -179.85  116.10  1.5284
IC C5'  C4'  C3'   O3'    1.5160  116.10   76.70  115.12  1.4212
IC C4'  C3'  O3'   +P     1.5284  111.92  159.13  119.05  1.6001
IC C3'  O3'  +P    +O5'   1.4212  119.05  -98.86  101.45  1.5996
IC O4'  C3'  *C4'  C5'    1.4572  104.06 -120.04  116.10  1.5160
IC C2'  C4'  *C3'  O3'    1.5284  100.16 -124.08  115.12  1.4212
IC C4'  C3'  C2'   C1'    1.5284  100.16   39.58  102.04  1.5251
IC C3'  C2'  C1'   N9     1.5284  101.97  144.39  113.71  1.4896
IC O4'  C1'  N9    C4     1.5251  113.71  -97.2   125.59  1.3783
IC C1'  C3'  *C2'  O2'    1.5284  102.04 -114.67  110.81  1.4212
IC H2'  O2'  C2'   C3'    0.9600  114.97  148.63  111.92  1.5284
IC O4'  C2'  *C1'  H1'    0.0       0.0  -115.0     0.0   0.0
IC C1'  C3'  *C2'  H2''   0.0       0.0   115.0     0.0   0.0
IC C2'  C4'  *C3'  H3'    0.0       0.0   115.0     0.0   0.0
IC C3'  O4'  *C4'  H4'    0.0       0.0  -115.0     0.0   0.0
IC C4'  O5'  *C5'  H5'    0.0       0.0  -115.0     0.0   0.0
IC C4'  O5'  *C5'  H5''   0.0       0.0   115.0     0.0   0.0
IC C8   C4   *N9   C1'       1.3791  105.54 -179.95  126.56  1.4896
IC C4   N9   C8    N7        1.3504  107.36    0.10  112.83  1.3241
IC N7   N9   *C8   H8        1.3241  112.83 -179.99  122.89  1.0943
IC N9   C8   N7    C5        1.3727  112.83   -0.24  103.70  1.3977
IC C4   N7   *C5   C6        1.3941  110.25  179.92  132.71  1.4020
IC N7   C5   C6    N6        1.3977  132.71    0.85  118.28  1.4043
IC N6   C5   *C6   N1        1.4043  118.28  179.74  117.11  1.3761
IC C5   C6   N6    C10       1.4020  118.28  179.02  128.67  1.3572
IC C10  C6   *N6   H6        1.3572  128.67 -174.14  113.59  0.9877
IC C5   C6   N1    C2        1.4020  117.11   -0.67  120.55  1.3612
IC C6   N1   C2    N3        1.3761  120.55    0.63  124.92  1.3606
IC N3   N1   *C2   H2        1.3606  124.92  179.52  117.21  1.0929
IC C6   N6   C10   N11       1.4043  128.67 -179.50  108.76  1.3596
IC N11  N6   *C10  O10       1.3596  108.76  178.51  126.84  1.2247
IC N6   C10  N11   C12       1.3572  108.76  158.41  123.65  1.4557
IC C12  C10  *N11  H11       1.4557  123.65 -137.39  118.40  0.9997
IC C10  N11  C12   C14       1.3596  123.65  125.29  113.74  1.5292
IC C14  N11  *C12  C13       1.5292  113.74  117.08  105.45  1.5439
IC C14  N11  *C12  H12       1.5292  113.74 -125.19  111.57  1.1114
IC N11  C12  C13   ODA       1.4557  105.45  180.00  116.00  1.2600
IC ODA  C12  *C13  ODB       1.2600  116.00  180.00  116.00  1.2600
IC N11  C12  C14   C15       1.4557  113.74  178.67  113.37  1.5506
IC C15  C12  *C14  O14       1.5506  113.37  120.61  111.37  1.4235
IC C15  C12  *C14  H14       1.5506  113.37 -121.01  108.51  1.1157
IC C12  C14  O14   H14O      1.5292  111.37  180.00  106.00  0.9600
IC C12  C14  C15   C16       1.5292  113.37  174.40  115.16  1.5396
```

```
IC C16  C14  *C15  H151      1.5396  115.16  120.94  108.13  1.1141
IC C16  C14  *C15  H152      1.5396  115.16 -123.75  108.54  1.1121
IC C14  C15  C16   H161      1.5506  115.16  172.87  109.69  1.1115
IC H161 C15  *C16  H162      1.1115  109.69  119.43  111.32  1.1092
IC H161 C15  *C16  H163      1.1115  109.69 -119.11  110.28  1.1118
DONO H2'  O2'
DONO H6   N6
DONO H11  N11
DONO H14O O14
ACCE N3
ACCE N7
ACCE N1
ACCE O10  C10
ACCE O14
ACCE ODA  C13
ACCE ODB  C13
ACCE O1P  P
ACCE O2P  P
ACCE O2'
ACCE O3'
ACCE O4'
ACCE O5'

RESI 26A           -2.00 ! 2-methylthio-N6-hydroxynorvalyl carbamoyladenosine
GROUP
ATOM N9     NG2R51 -0.01 !                         H161 H162
ATOM C8     CG2R53  0.37 !                          |  /
ATOM H8     HGR52   0.10 !                     H151 C16-H163
ATOM N7     NG2R50 -0.84 !                       \ /
ATOM C5     CG2RC0  0.28 !                  H152-C15   O14-H14O
ATOM C6     CG2R64  0.43 !                         \  /
ATOM N1     NG2R62 -0.58 !                 O10      C14-H14
ATOM C2     CG2R64  0.63 !                 ||      /
ATOM N3     NG2R62 -0.76 !            H6   C10  C12-H12
ATOM C4     CG2RC0  0.48 !             \  /  \  /  \
ATOM S2     SG311  -0.28 !              N6    N11   C13-ODA (-)
ATOM C2M    CG331  -0.09 !              |      |    ||
ATOM H21    HGA3    0.09 !              |     H11   ODB
ATOM H22    HGA3    0.09 !              C6
ATOM H23    HGA3    0.09 !             // \
ATOM N6     NG2S1  -0.43 !    H21     N1   C5--N7\\
ATOM H6     HGP1    0.32 !      \      |    ||     C8-H8
ATOM C10    CG2O6   0.60 !  H22 -C2M  C2   C4--N9/
ATOM O10    OG2D1  -0.50 !      / \  / \\ /      \
ATOM N11    NG2S1  -0.48 !    H23  S2   N3        \
ATOM H11    HGP1    0.31 !                         \
ATOM C12    CG311   0.09 !                          \
ATOM H12    HGA1    0.09 !                           \
GROUP                    !       O1P    H5' H4'  O4'  \
ATOM C13    CG2O3   0.52 !        |      |    \ /   \  \
ATOM ODA    OG2D2  -0.76 !       -P-O5'-C5'---C4'    C1'
ATOM ODB    OG2D2  -0.76 !        |      |     \     / \
GROUP                    !       O2P    H5''   C3'--C2' H1'
ATOM C14    CG311   0.14 !                    / \   / \
ATOM H14    HGA1    0.09 !                  O3' H3' O2' H2''
ATOM O14    OG311  -0.65 !                   |       |
ATOM H14O   HGP1    0.42 !                          H2'
ATOM C15    CG321  -0.18
ATOM H151   HGA2    0.09 !  !!!! PATCH 6AH for the carboxylic acid form
ATOM H152   HGA2    0.09
ATOM C16    CG331  -0.27
ATOM H161   HGA3    0.09
ATOM H162   HGA3    0.09
```

```
ATOM H163   HGA3    0.09
GROUP
ATOM P      P       1.50
ATOM O1P    ON3    -0.78
ATOM O2P    ON3    -0.78
ATOM O5'    ON2    -0.57
ATOM C5'    CN8B   -0.08
ATOM H5'    HN8     0.09
ATOM H5''   HN8     0.09
GROUP
ATOM C4'    CN7     0.16
ATOM H4'    HN7     0.09
ATOM O4'    ON6B   -0.50
ATOM C1'    CN7B    0.16
ATOM H1'    HN7     0.09
GROUP
ATOM C2'    CN7B    0.14
ATOM H2''   HN7     0.09
ATOM O2'    ON5    -0.66
ATOM H2'    HN5     0.43
GROUP
ATOM C3'    CN7     0.01
ATOM H3'    HN7     0.09
ATOM O3'    ON2    -0.57
BOND N9   C8     N9   C4      C8   N7      C8   H8
BOND N7   C5     C5   C6      C5   C4      C6   N6
BOND C6   N1     N6   C10     N6   H6      N1   C2
BOND C2   N3     C2   S2      N3   C4      C10  N11
BOND C10  O10    N11  C12     N11  H11     C12  C14
BOND C12  C13    C12  H12     C14  C15     C14  O14
BOND C14  H14    C15  C16     C15  H151    C15  H152
BOND C16  H161   C16  H162    C16  H163    C13  ODA
BOND C13  ODB    O14  H14O    S2   C2M     C2M  H21
BOND C2M  H22    C2M  H23
BOND P    O1P     P    O2P     P    O5'     O5'  C5'     C5'  H5''
BOND C5'  C4'     C4'  O4'     C4'  C3'     O4'  C1'
BOND C1'  N9      C1'  C2'     C2'  C3'     C3'  O3'     O3'  +P
BOND C2'  O2'     O2'  H2'
BOND C1'  H1'     C2'  H2''    C3'  H3'     C4'  H4'     C5'  H5'
IMPR C10    N6     N11    O10    C13    ODB    ODA    C12     C6   C5   N1   N6
!ribose
IC -O3' P    O5'   C5'    1.6001  101.45  -39.25  119.00  1.4401
IC -O3' O5'  *P    O1P    1.6001  101.45 -115.82  109.74  1.4802
IC -O3' O5'  *P    O2P    1.6001  101.45  115.90  109.80  1.4801
IC  P   O5'  C5'   C4'    1.5996  119.00 -151.39  110.04  1.5160
IC O5'  C5'  C4'   C3'    1.4401  108.83 -179.85  116.10  1.5284
IC C5'  C4'  C3'   O3'    1.5160  116.10   76.70  115.12  1.4212
IC C4'  C3'  O3'   +P     1.5284  111.92  159.13  119.05  1.6001
IC C3'  O3'  +P    +O5'   1.4212  119.05  -98.86  101.45  1.5996
IC O4'  C3'  *C4'  C5'    1.4572  104.06 -120.04  116.10  1.5160
IC C2'  C4'  *C3'  O3'    1.5284  100.16 -124.08  115.12  1.4212
IC C4'  C3'  C2'   C1'    1.5284  100.16   39.58  102.04  1.5251
IC C3'  C2'  C1'   N9     1.5284  101.97  144.39  113.71  1.4896
IC O4'  C1'  N9    C4     1.5251  113.71  -97.2   125.59  1.3783
IC C1'  C3'  *C2'  O2'    1.5284  102.04 -114.67  110.81  1.4212
IC H2'  O2'  C2'   C3'    0.9600  114.97  148.63  111.92  1.5284
IC O4'  C2'  *C1'  H1'    0.0       0.0  -115.0     0.0   0.0
IC C1'  C3'  *C2'  H2''   0.0       0.0   115.0     0.0   0.0
IC C2'  C4'  *C3'  H3'    0.0       0.0   115.0     0.0   0.0
IC C3'  O4'  *C4'  H4'    0.0       0.0  -115.0     0.0   0.0
IC C4'  O5'  *C5'  H5'    0.0       0.0  -115.0     0.0   0.0
IC C4'  O5'  *C5'  H5''   0.0       0.0   115.0     0.0   0.0
IC C8   C4   *N9   C1'       1.3791  105.54 -179.95  126.56  1.4896
```

```
IC C4   N9   C8    N7        1.3834  105.90    0.05  114.08  1.3272
IC N7   N9   *C8   H8        1.3272  114.08  179.94  121.51  1.0917
IC N9   C8   N7    C5        1.3842  114.08   -0.27  103.43  1.3964
IC C4   N7   *C5   C6        1.4026  111.06  179.94  131.69  1.3946
IC N7   C5   C6    N6        1.3964  131.69    0.62  115.13  1.3949
IC N6   C5   *C6   N1        1.3949  115.13 -179.75  117.75  1.3690
IC C5   C6   N1    C2        1.3946  117.75   -1.02  120.22  1.3566
IC C6   N1   C2    S2        1.3690  120.22 -179.75  117.72  1.7700
IC S2   N1   *C2   N3        1.7700  117.72 -179.36  125.62  1.3555
IC N1   C2   S2    C2M       1.3566  117.72    2.06  103.01  1.8278
IC C2   S2   C2M   H21       1.7700  103.01  179.65  111.06  1.1109
IC H21  S2   *C2M  H22       1.1109  111.06  120.07  111.42  1.1132
IC H21  S2   *C2M  H23       1.1109  111.06 -120.12  111.39  1.1118
IC C5   C6   N6    C10       1.3946  115.13  179.52  127.04  1.3586
IC C10  C6   *N6   H6        1.3586  127.04 -173.42  114.02  0.9907
IC C6   N6   C10   N11       1.3949  127.04 -177.40  109.23  1.3587
IC N11  N6   *C10  O10       1.3587  109.23  176.67  126.20  1.2243
IC N6   C10  N11   C12       1.3586  109.23  149.41  123.13  1.4558
IC C12  C10  *N11  H11       1.4558  123.13 -134.31  117.77  0.9999
IC C10  N11  C12   C14       1.3587  123.13  128.46  113.79  1.5287
IC C14  N11  *C12  C13       1.5287  113.79  117.11  104.98  1.5430
IC C14  N11  *C12  H12       1.5287  113.79 -125.26  111.63  1.1121
IC N11  C12  C13   ODA       1.4558  104.98  146.98  117.44  1.2559
IC ODA  C12  *C13  ODB       1.2559  117.44 -179.83  114.71  1.2649
IC N11  C12  C14   C15       1.4558  113.79  178.20  113.49  1.5499
IC C15  C12  *C14  O14       1.5499  113.49  120.73  111.29  1.4250
IC C15  C12  *C14  H14       1.5499  113.49 -121.01  108.47  1.1161
IC C12  C14  O14   H14O      1.5287  111.29 -167.51  103.47  0.9572
IC C12  C14  C15   C16       1.5287  113.49  174.70  115.13  1.5403
IC C16  C14  *C15  H151      1.5403  115.13  120.90  108.18  1.1146
IC C16  C14  *C15  H152      1.5403  115.13 -123.71  108.55  1.1117
IC C14  C15  C16   H161      1.5499  115.13  173.23  109.70  1.1114
IC H161 C15  *C16  H162      1.1114  109.70  119.41  111.20  1.1096
IC H161 C15  *C16  H163      1.1114  109.70 -119.17  110.33  1.1109
DONO H2'  O2'
DONO H6   N6
DONO H11  N11
DONO H14O O14
ACCE N3
ACCE N7
ACCE N1
ACCE O10  C10
ACCE O14
ACCE ODA  C13
ACCE ODB  C13
ACCE O1P  P
ACCE O2P  P
ACCE O2'
ACCE O3'
ACCE O4'
ACCE O5'

RESI 66A           -2.00 ! N6-methyl-N6-threonylcarbamoyladenosine, AET
GROUP
ATOM N9     NG2R51 -0.01 !                 H152 H153
ATOM C8     CG2R53  0.39 !                    \ /
ATOM H8     HGR52   0.09 !              H151-C15   O14-H14O
ATOM N7     NG2R50 -0.81 !                      \  /
ATOM C5     CG2RC0  0.35 !    H61      O10      C14-H14
ATOM C6     CG2R64  0.60 !       \      ||      /
ATOM N1     NG2R62 -0.68 !   H62-CM6   C10   C12-H12
ATOM C2     CG2R64  0.39 !       / \  /  \  /  \
ATOM H2     HGR62   0.14 !    H63   N6    N11   C13-ODA (-)
```

```
ATOM N3     NG2R62 -0.82 !           |       |     ||
ATOM C4     CG2RC0  0.57 !           |      H11   ODB
ATOM N6     NG2S0  -0.49 !           C6
ATOM CM6    CG331  -0.08 !          // \
ATOM H61     HGA3   0.09 !        N1   C5--N7\\
ATOM H62     HGA3   0.09 !        |    ||      C8-H8
ATOM H63     HGA3   0.09 !        C2   C4--N9/
ATOM C10    CG2O6   0.49 !       / \\ /      \
ATOM O10    OG2D1  -0.48 !     H2   N3         \
ATOM N11    NG2S1  -0.44 !                      \
ATOM H11     HGP1   0.30 !                       \
ATOM C12    CG311   0.13 !                        \
ATOM H12    HGA1    0.09 !   O1P    H5' H4'  O4'  \
GROUP                    !    |      |    \ /   \  \
ATOM C13    CG2O3   0.52 !   -P-O5'-C5'---C4'    C1'
ATOM ODA    OG2D2  -0.76 !    |      |     \     / \
ATOM ODB    OG2D2  -0.76 !   O2P    H5''   C3'--C2' H1'
GROUP                    !                 / \   / \
ATOM C14    CG311   0.14 !               O3' H3' O2' H2''
ATOM H14    HGA1    0.09 !                |       |
ATOM O14    OG311  -0.65 !                       H2'
ATOM H14O   HGP1    0.42
ATOM C15    CG331  -0.27 !  !!!! PATCH 6AH for the carboxylic acid form
ATOM H151   HGA3    0.09
ATOM H152   HGA3    0.09
ATOM H153   HGA3    0.09
GROUP
ATOM P      P       1.50
ATOM O1P    ON3    -0.78
ATOM O2P    ON3    -0.78
ATOM O5'    ON2    -0.57
ATOM C5'    CN8B   -0.08
ATOM H5'    HN8     0.09
ATOM H5''   HN8     0.09
GROUP
ATOM C4'    CN7     0.16
ATOM H4'    HN7     0.09
ATOM O4'    ON6B   -0.50
ATOM C1'    CN7B    0.16
ATOM H1'    HN7     0.09
GROUP
ATOM C2'    CN7B    0.14
ATOM H2''   HN7     0.09
ATOM O2'    ON5    -0.66
ATOM H2'    HN5     0.43
GROUP
ATOM C3'    CN7     0.01
ATOM H3'    HN7     0.09
ATOM O3'    ON2    -0.57
BOND N9   C8     N9   C4      C8   N7      C8   H8
BOND N7   C5     C5   C6      C5   C4      C6   N6
BOND C6   N1     N6   C10     N6   CM6     N1   C2
BOND C2   N3     C2   H2      N3   C4      C10  O10
BOND C10  N11    N11  C12     N11  H11     C12  C13
BOND C12  C14    C12  H12     C13  ODA     C13  ODB
BOND C14  O14    C14  C15     C14  H14     O14  H14O
BOND C15  H151   C15  H152    C15  H153    CM6  H61
BOND CM6  H62    CM6  H63
BOND P    O1P     P    O2P     P    O5'     O5'  C5'     C5'  H5''
BOND C5'  C4'     C4'  O4'     C4'  C3'     O4'  C1'
BOND C1'  N9      C1'  C2'     C2'  C3'     C3'  O3'     O3'  +P
BOND C2'  O2'     O2'  H2'
BOND C1'  H1'     C2'  H2''    C3'  H3'     C4'  H4'     C5'  H5'
```

```
IMPR C10    N6     N11    O10    C13    ODB    ODA    C12     C6   C5   N1   N6
!ribose
IC -O3' P    O5'   C5'    1.6001  101.45  -39.25  119.00  1.4401
IC -O3' O5'  *P    O1P    1.6001  101.45 -115.82  109.74  1.4802
IC -O3' O5'  *P    O2P    1.6001  101.45  115.90  109.80  1.4801
IC  P   O5'  C5'   C4'    1.5996  119.00 -151.39  110.04  1.5160
IC O5'  C5'  C4'   C3'    1.4401  108.83 -179.85  116.10  1.5284
IC C5'  C4'  C3'   O3'    1.5160  116.10   76.70  115.12  1.4212
IC C4'  C3'  O3'   +P     1.5284  111.92  159.13  119.05  1.6001
IC C3'  O3'  +P    +O5'   1.4212  119.05  -98.86  101.45  1.5996
IC O4'  C3'  *C4'  C5'    1.4572  104.06 -120.04  116.10  1.5160
IC C2'  C4'  *C3'  O3'    1.5284  100.16 -124.08  115.12  1.4212
IC C4'  C3'  C2'   C1'    1.5284  100.16   39.58  102.04  1.5251
IC C3'  C2'  C1'   N9     1.5284  101.97  144.39  113.71  1.4896
IC O4'  C1'  N9    C4     1.5251  113.71  -97.2   125.59  1.3783
IC C1'  C3'  *C2'  O2'    1.5284  102.04 -114.67  110.81  1.4212
IC H2'  O2'  C2'   C3'    0.9600  114.97  148.63  111.92  1.5284
IC O4'  C2'  *C1'  H1'    0.0       0.0  -115.0     0.0   0.0
IC C1'  C3'  *C2'  H2''   0.0       0.0   115.0     0.0   0.0
IC C2'  C4'  *C3'  H3'    0.0       0.0   115.0     0.0   0.0
IC C3'  O4'  *C4'  H4'    0.0       0.0  -115.0     0.0   0.0
IC C4'  O5'  *C5'  H5'    0.0       0.0  -115.0     0.0   0.0
IC C4'  O5'  *C5'  H5''   0.0       0.0   115.0     0.0   0.0
IC C8   C4   *N9   C1'       1.3791  105.54 -179.95  126.56  1.4896
IC C4   N9   C8    N7        1.3836  106.06   -0.40  113.87  1.3245
IC N7   N9   *C8   H8        1.3245  113.87 -179.99  121.60  1.0915
IC N9   C8   N7    C5        1.3788  113.87   -0.25  104.00  1.4062
IC C4   N7   *C5   C6        1.4020  110.03  175.13  132.63  1.4092
IC N7   C5   C6    N6        1.4062  132.63   13.05  123.23  1.4214
IC N6   C5   *C6   N1        1.4214  123.23  176.37  116.33  1.3871
IC C5   C6   N1    C2        1.4092  116.33    6.52  121.28  1.3523
IC C6   N1   C2    N3        1.3871  121.28   -4.78  124.69  1.3536
IC N3   N1   *C2   H2        1.3536  124.69 -176.81  117.31  1.0937
IC C5   C6   N6    C10       1.4092  123.23 -140.01  116.98  1.3654
IC C10  C6   *N6   CM6       1.3654  116.98  180.00  115.50  1.4340
IC C6   N6   CM6   H61       1.4214  115.50  180.00  105.00  1.1110
IC H61  N6   *CM6  H62       1.1110  105.00  120.00  105.00  1.1110
IC H61  N6   *CM6  H63       1.1110  105.00 -120.00  105.00  1.1110
IC C6   N6   C10   N11       1.4214  116.98 -178.49  111.99  1.3606
IC N11  N6   *C10  O10       1.3606  111.99 -177.53  126.32  1.2247
IC N6   C10  N11   C12       1.3654  111.99  155.31  123.90  1.4496
IC C12  C10  *N11  H11       1.4496  123.90 -133.68  116.66  1.0025
IC C10  N11  C12   C14       1.3606  123.90  108.11  114.88  1.5320
IC C14  N11  *C12  C13       1.5320  114.88  117.88  104.84  1.5503
IC C13  N11  *C12  H12       1.5503  104.84  116.34  110.88  1.1116
IC N11  C12  C13   ODA       1.4496  104.84  180.00  116.00  1.2600
IC ODA  C12  *C13  ODB       1.2600  116.00  180.00  116.00  1.2600
IC N11  C12  C14   O14       1.4496  114.88  179.16  111.73  1.4244
IC O14  C12  *C14  C15       1.4244  111.73 -119.07  110.63  1.5367
IC O14  C12  *C14  H14       1.4244  111.73  120.70  109.52  1.1160
IC C12  C14  O14   H14O      1.5320  111.73  180.00  106.00  0.9600
IC C12  C14  C15   H151      1.5320  110.63 -177.78  110.28  1.1103
IC H151 C14  *C15  H152      1.1103  110.28  119.80  109.85  1.1105
IC H151 C14  *C15  H153      1.1103  110.28 -120.59  110.72  1.1092
DONO H2'  O2'
DONO H11  N11
DONO H14O O14
ACCE N3
ACCE N7
ACCE N1
ACCE O10  C10
ACCE O14
ACCE ODA  C13
```

```
ACCE ODB  C13
ACCE O1P  P
ACCE O2P  P
ACCE O2'
ACCE O3'
ACCE O4'
ACCE O5'

RESI T6A           -2.00 ! N6-threonylcarbamoyladenosine, 6TA
GROUP
ATOM N9     NG2R51 -0.01 !                H152 H153
ATOM C8     CG2R53  0.39 !                  \ /
ATOM H8     HGR52   0.09 !             H151-C15   O14-H14O
ATOM N7     NG2R50 -0.81 !                    \  /
ATOM C5     CG2RC0  0.35 !            O10      C14-H14
ATOM C6     CG2R64  0.66 !            ||      /
ATOM N6     NG2S1  -0.68 !       H6   C10   C12-H12
ATOM H6     HGP1    0.32 !         \  /  \  /  \
ATOM N1     NG2R62 -0.68 !          N6    N11   C13-ODA (-)
ATOM C2     CG2R64  0.39 !          |      |    ||
ATOM H2     HGR62   0.14 !          |     H11   ODB
ATOM N3     NG2R62 -0.82 !          C6
ATOM C4     CG2RC0  0.57 !         // \
ATOM C10    CG2O6   0.60 !       N1   C5--N7\\
ATOM O10    OG2D1  -0.50 !       |    ||     C8-H8
ATOM N11    NG2S1  -0.48 !       C2   C4--N9/
ATOM H11    HGP1    0.29 !      / \\ /      \
ATOM C12    CG311   0.09 !    H2   N3        \
ATOM H12    HGA1    0.09 !                    \
GROUP                    !                     \
ATOM C13    CG2O3   0.52 !                      \
ATOM ODA    OG2D2  -0.76 !  O1P    H5' H4'  O4'  \
ATOM ODB    OG2D2  -0.76 !   |      |    \ /   \  \
GROUP                    !  -P-O5'-C5'---C4'    C1'
ATOM C14    CG311   0.14 !   |      |     \     / \
ATOM H14    HGA1    0.09 !  O2P    H5''   C3'--C2' H1'
ATOM O14    OG311  -0.65 !               / \   / \
ATOM H14O   HGP1    0.42 !             O3' H3' O2' H2''
ATOM C15    CG331  -0.27 !              |       |
ATOM H151   HGA3    0.09 !                      H2'
ATOM H152   HGA3    0.09
ATOM H153   HGA3    0.09 !  !!!! PATCH 6AH for the carboxylic acid form
GROUP
ATOM P      P       1.50
ATOM O1P    ON3    -0.78
ATOM O2P    ON3    -0.78
ATOM O5'    ON2    -0.57
ATOM C5'    CN8B   -0.08
ATOM H5'    HN8     0.09
ATOM H5''   HN8     0.09
GROUP
ATOM C4'    CN7     0.16
ATOM H4'    HN7     0.09
ATOM O4'    ON6B   -0.50
ATOM C1'    CN7B    0.16
ATOM H1'    HN7     0.09
GROUP
ATOM C2'    CN7B    0.14
ATOM H2''   HN7     0.09
ATOM O2'    ON5    -0.66
ATOM H2'    HN5     0.43
GROUP
ATOM C3'    CN7     0.01
```

```
ATOM H3'    HN7     0.09
ATOM O3'    ON2    -0.57
BOND N9   C8     N9   C4      C8   N7      C8   H8
BOND N7   C5     C5   C6      C5   C4      C6   N6
BOND C6   N1     N6   C10     N6   H6      N1   C2
BOND C2   N3     C2   H2      N3   C4      C10  O10
BOND C10  N11    N11  C12     N11  H11     C12  C13
BOND C12  C14    C12  H12     C13  ODA     C13  ODB
BOND C14  O14    C14  C15     C14  H14     O14  H14O
BOND C15  H151   C15  H152    C15  H153
BOND P    O1P     P    O2P     P    O5'     O5'  C5'     C5'  H5''
BOND C5'  C4'     C4'  O4'     C4'  C3'     O4'  C1'
BOND C1'  N9      C1'  C2'     C2'  C3'     C3'  O3'     O3'  +P
BOND C2'  O2'     O2'  H2'
BOND C1'  H1'     C2'  H2''    C3'  H3'     C4'  H4'     C5'  H5'
IMPR C10    N6     N11    O10    C13    ODB    ODA    C12     C6   C5   N1   N6
!ribose
IC -O3' P    O5'  C5'    1.6001  101.45  -39.25  119.00  1.4401
IC -O3' O5'  *P   O1P    1.6001  101.45 -115.82  109.74  1.4802
IC -O3' O5'  *P   O2P    1.6001  101.45  115.90  109.80  1.4801
IC  P   O5'  C5'  C4'    1.5996  119.00 -151.39  110.04  1.5160
IC O5'  C5'  C4'  C3'    1.4401  108.83 -179.85  116.10  1.5284
IC C5'  C4'  C3'  O3'    1.5160  116.10   76.70  115.12  1.4212
IC C4'  C3'  O3'  +P     1.5284  111.92  159.13  119.05  1.6001
IC C3'  O3'  +P   +O5'   1.4212  119.05  -98.86  101.45  1.5996
IC O4'  C3'  *C4' C5'    1.4572  104.06 -120.04  116.10  1.5160
IC C2'  C4'  *C3' O3'    1.5284  100.16 -124.08  115.12  1.4212
IC C4'  C3'  C2'  C1'    1.5284  100.16   39.58  102.04  1.5251
IC C3'  C2'  C1'  N9     1.5284  101.97  144.39  113.71  1.4896
IC O4'  C1'  N9   C4     1.5251  113.71  -97.2   125.59  1.3783
IC C1'  C3'  *C2' O2'    1.5284  102.04 -114.67  110.81  1.4212
IC H2'  O2'  C2'  C3'    0.9600  114.97  148.63  111.92  1.5284
IC O4'  C2'  *C1' H1'    0.0       0.0  -115.0     0.0   0.0
IC C1'  C3'  *C2' H2''   0.0       0.0   115.0     0.0   0.0
IC C2'  C4'  *C3' H3'    0.0       0.0   115.0     0.0   0.0
IC C3'  O4'  *C4' H4'    0.0       0.0  -115.0     0.0   0.0
IC C4'  O5'  *C5' H5'    0.0       0.0  -115.0     0.0   0.0
IC C4'  O5'  *C5' H5''   0.0       0.0   115.0     0.0   0.0
IC C8   C4   *N9  C1'      1.3791  105.54 -179.95  126.56  1.4896
IC C4   N9   C8   N7       1.3858  106.08    0.08  113.98  1.3243
IC N7   N9   *C8  H8       1.3243  113.98  179.91  121.69  1.0921
IC N9   C8   N7   C5       1.3819  113.98   -0.31  103.51  1.4083
IC C4   N7   *C5  C6       1.3959  110.71 -179.84  132.82  1.3995
IC N7   C5   C6   N6       1.4083  132.82    0.47  115.76  1.3912
IC N6   C5   *C6  N1       1.3912  115.76 -179.25  117.90  1.3719
IC C5   C6   N6   C10      1.3995  115.76  176.73  126.82  1.3525
IC C10  C6   *N6  H6       1.3525  126.82 -167.68  114.51  0.9903
IC C5   C6   N1   C2       1.3995  117.90   -1.64  120.90  1.3522
IC C6   N1   C2   N3       1.3719  120.90    1.14  124.45  1.3550
IC N3   N1   *C2  H2       1.3550  124.45  179.38  117.32  1.0960
IC C6   N6   C10  N11      1.3912  126.82 -173.91  107.81  1.3536
IC N11  N6   *C10 O10      1.3536  107.81  175.19  127.13  1.2207
IC N6   C10  N11  C12      1.3525  107.81  144.35  122.96  1.4558
IC C12  C10  *N11 H11      1.4558  122.96 -132.35  116.20  1.0012
IC C10  N11  C12  C14      1.3536  122.96  113.22  114.40  1.5314
IC C14  N11  *C12 C13      1.5314  114.40  118.82  106.39  1.5516
IC C13  N11  *C12 H12      1.5516  106.39  116.56  110.73  1.1120
IC N11  C12  C13  ODA      1.4558  106.39 -179.76  116.16  1.2598
IC ODA  C12  *C13 ODB      1.2598  116.16  178.91  116.11  1.2630
IC N11  C12  C14  O14      1.4558  114.40 -178.67  111.48  1.4230
IC O14  C12  *C14 C15      1.4230  111.48 -118.76  110.39  1.5370
IC O14  C12  *C14 H14      1.4230  111.48  120.98  109.60  1.1150
IC C12  C14  O14  H14O     1.5314  111.48  -42.06  101.01  0.9854
```

```
IC C12  C14  C15   H151      1.5314  110.39 -177.66  110.19  1.1096
IC H151 C14  *C15  H152      1.1096  110.19  119.85  109.84  1.1103
IC H151 C14  *C15  H153      1.1096  110.19 -120.46  110.68  1.1087
DONO H2'  O2'
DONO H6   N6
DONO H11  N11
DONO H14O O14
ACCE N3
ACCE N7
ACCE N1
ACCE O10  C10
ACCE O14
ACCE ODA  C13
ACCE ODB  C13
ACCE O1P  P
ACCE O2P  P
ACCE O2'
ACCE O3'
ACCE O4'
ACCE O5'

RESI 12A           -2.00 ! 2-methylthio-N6-threonyl carbamoyladenosine, STA
GROUP
ATOM N9     NG2R51 -0.01 !                     H152  H153
ATOM C8     CG2R53  0.37 !                       \ /
ATOM H8     HGR52   0.10 !                  H151-C15  O14-H14O
ATOM N7     NG2R50 -0.84 !                        \  /
ATOM C5     CG2RC0  0.28 !                 O10     C14-H14
ATOM C6     CG2R64  0.43 !                 ||     /
ATOM N1     NG2R62 -0.58 !            H6   C10  C12-H12
ATOM C2     CG2R64  0.63 !             \  /  \ /  \
ATOM N3     NG2R62 -0.76 !              N6    N11  C13-ODA (-)
ATOM C4     CG2RC0  0.48 !              |     |    ||
ATOM S2     SG311  -0.28 !              |     H11  ODB
ATOM C2M    CG331  -0.09 !              C6
ATOM H21    HGA3    0.09 !             // \
ATOM H22    HGA3    0.09 !    H21     N1   C5--N7\\
ATOM H23    HGA3    0.09 !      \     |    ||     C8-H8
ATOM N6     NG2S1  -0.43 !  H22 -C2M  C2   C4--N9/
ATOM H6     HGP1    0.32 !      / \  / \\ /      \
ATOM C10    CG2O6   0.60 !    H23  S2   N3        \
ATOM O10    OG2D1  -0.50 !                         \
ATOM N11    NG2S1  -0.48 !                          \
ATOM H11    HGP1    0.31 !                           \
ATOM C12    CG311   0.09 !       O1P    H5' H4'  O4'  \
ATOM H12    HGA1    0.09 !        |      |    \ /   \  \
GROUP                    !       -P-O5'-C5'---C4'    C1'
ATOM C13    CG2O3   0.52 !        |      |     \     / \
ATOM ODA    OG2D2  -0.76 !       O2P    H5''   C3'--C2' H1'
ATOM ODB    OG2D2  -0.76 !                    / \   / \
GROUP                    !                  O3' H3' O2' H2''
ATOM C14    CG311   0.14 !                   |       |
ATOM H14    HGA1    0.09 !                          H2'
ATOM O14    OG311  -0.65
ATOM H14O   HGP1    0.42 !  !!!! PATCH 6AH for the carboxylic acid form
ATOM C15    CG331  -0.27
ATOM H151   HGA3    0.09
ATOM H152   HGA3    0.09
ATOM H153   HGA3    0.09
GROUP
ATOM P      P       1.50
ATOM O1P    ON3    -0.78
ATOM O2P    ON3    -0.78
```

```
ATOM O5'    ON2    -0.57
ATOM C5'    CN8B   -0.08
ATOM H5'    HN8     0.09
ATOM H5''   HN8     0.09
GROUP
ATOM C4'    CN7     0.16
ATOM H4'    HN7     0.09
ATOM O4'    ON6B   -0.50
ATOM C1'    CN7B    0.16
ATOM H1'    HN7     0.09
GROUP
ATOM C2'    CN7B    0.14
ATOM H2''   HN7     0.09
ATOM O2'    ON5    -0.66
ATOM H2'    HN5     0.43
GROUP
ATOM C3'    CN7     0.01
ATOM H3'    HN7     0.09
ATOM O3'    ON2    -0.57
BOND N9   C8     N9   C4      C8   N7      C8   H8
BOND N7   C5     C5   C6      C5   C4      C6   N6
BOND C6   N1     N6   H6      N6   C10     N1   C2
BOND C2   N3     C2   S2      N3   C4      S2   C2M
BOND C2M  H21    C2M  H22     C2M  H23     C10  O10
BOND C10  N11    N11  C12     N11  H11     C12  C14
BOND C12  C13    C12  H12     C13   ODA    C13   ODB
BOND C14  O14    C14  C15     C14  H14     O14  H14O
BOND C15  H151   C15  H152    C15  H153
BOND P    O1P     P    O2P     P    O5'     O5'  C5'     C5'  H5''
BOND C5'  C4'     C4'  O4'     C4'  C3'     O4'  C1'
BOND C1'  N9      C1'  C2'     C2'  C3'     C3'  O3'     O3'  +P
BOND C2'  O2'     O2'  H2'
BOND C1'  H1'     C2'  H2''    C3'  H3'     C4'  H4'     C5'  H5'
IMPR C10    N6     N11    O10    C13     ODB     ODA    C12     C6   C5   N1   N6
!ribose
IC -O3' P    O5'  C5'    1.6001  101.45  -39.25  119.00  1.4401
IC -O3' O5'  *P   O1P    1.6001  101.45 -115.82  109.74  1.4802
IC -O3' O5'  *P   O2P    1.6001  101.45  115.90  109.80  1.4801
IC  P   O5'  C5'  C4'    1.5996  119.00 -151.39  110.04  1.5160
IC O5'  C5'  C4'  C3'    1.4401  108.83 -179.85  116.10  1.5284
IC C5'  C4'  C3'  O3'    1.5160  116.10   76.70  115.12  1.4212
IC C4'  C3'  O3'  +P     1.5284  111.92  159.13  119.05  1.6001
IC C3'  O3'  +P   +O5'   1.4212  119.05  -98.86  101.45  1.5996
IC O4'  C3'  *C4' C5'    1.4572  104.06 -120.04  116.10  1.5160
IC C2'  C4'  *C3' O3'    1.5284  100.16 -124.08  115.12  1.4212
IC C4'  C3'  C2'  C1'    1.5284  100.16   39.58  102.04  1.5251
IC C3'  C2'  C1'  N9     1.5284  101.97  144.39  113.71  1.4896
IC O4'  C1'  N9   C4     1.5251  113.71  -97.2   125.59  1.3783
IC C1'  C3'  *C2' O2'    1.5284  102.04 -114.67  110.81  1.4212
IC H2'  O2'  C2'  C3'    0.9600  114.97  148.63  111.92  1.5284
IC O4'  C2'  *C1' H1'    0.0       0.0  -115.0     0.0   0.0
IC C1'  C3'  *C2' H2''   0.0       0.0   115.0     0.0   0.0
IC C2'  C4'  *C3' H3'    0.0       0.0   115.0     0.0   0.0
IC C3'  O4'  *C4' H4'    0.0       0.0  -115.0     0.0   0.0
IC C4'  O5'  *C5' H5'    0.0       0.0  -115.0     0.0   0.0
IC C4'  O5'  *C5' H5''   0.0       0.0   115.0     0.0   0.0
IC C8   C4   *N9  C1'       1.3791  105.54 -179.95  126.56  1.4896
IC C4   N9   C8   N7        1.3837  105.91    0.10  114.04  1.3273
IC N7   N9   *C8  H8        1.3273  114.04  179.92  121.57  1.0916
IC N9   C8   N7   C5        1.3840  114.04   -0.30  103.46  1.3961
IC C4   N7   *C5  C6        1.4023  111.06  179.86  131.63  1.3944
IC N7   C5   C6   N6        1.3961  131.63    0.68  115.23  1.3946
IC N6   C5   *C6  N1        1.3946  115.23 -179.80  117.71  1.3699
```

```
IC C5   C6   N1    C2        1.3944  117.71   -0.95  120.21  1.3571
IC C6   N1   C2    S2        1.3699  120.21 -179.81  117.77  1.7687
IC S2   N1   *C2   N3        1.7687  117.77 -179.35  125.58  1.3553
IC N1   C2   S2    C2M       1.3571  117.77    1.93  103.13  1.8278
IC C2   S2   C2M   H21       1.7687  103.13  179.59  111.08  1.1102
IC H21  S2   *C2M  H22       1.1102  111.08  120.11  111.40  1.1124
IC H21  S2   *C2M  H23       1.1102  111.08 -120.11  111.40  1.1127
IC C5   C6   N6    C10       1.3944  115.23  179.54  126.84  1.3581
IC C10  C6   *N6   H6        1.3581  126.84 -173.03  114.06  0.9910
IC C6   N6   C10   N11       1.3946  126.84 -177.87  109.58  1.3582
IC N11  N6   *C10  O10       1.3582  109.58  176.46  126.65  1.2242
IC N6   C10  N11   C12       1.3581  109.58  146.57  122.91  1.4543
IC C12  C10  *N11  H11       1.4543  122.91 -131.03  117.15  1.0060
IC C10  N11  C12   C14       1.3582  122.91  113.30  114.67  1.5301
IC C14  N11  *C12  C13       1.5301  114.67  118.37  105.40  1.5499
IC C14  N11  *C12  H12       1.5301  114.67 -125.34  111.08  1.1130
IC N11  C12  C13   ODA       1.4543  105.40 -179.17  116.40  1.2591
IC ODA  C12  *C13  ODB       1.2591  116.40  178.94  115.63  1.2635
IC N11  C12  C14   O14       1.4543  114.67 -179.20  111.35  1.4232
IC O14  C12  *C14  C15       1.4232  111.35 -118.77  110.40  1.5366
IC O14  C12  *C14  H14       1.4232  111.35  120.90  109.68  1.1153
IC C12  C14  O14   H14O      1.5301  111.35  -42.90  101.20  0.9852
IC C12  C14  C15   H151      1.5301  110.40 -177.59  110.16  1.1098
IC H151 C14  *C15  H152      1.1098  110.16  119.68  109.78  1.1109
IC H151 C14  *C15  H153      1.1098  110.16 -120.54  110.73  1.1085
DONO H2'  O2'
DONO H6   N6
DONO H11  N11
DONO H14  O14
ACCE N3
ACCE N7
ACCE N1
ACCE O10 C10
ACCE O14
ACCE ODA  C13
ACCE ODB  C13
ACCE O1P  P
ACCE O2P  P
ACCE O2'
ACCE O3'
ACCE O4'
ACCE O5'

RESI 6GA           -2.00 ! N6-glycinylcarbamoyladenosine
GROUP
ATOM N9     NG2R51 -0.01 !           O10      H122
ATOM C8     CG2R53  0.39 !           ||      /
ATOM H8     HGR52   0.09 !      H6   C10  C12-H121
ATOM N7     NG2R50 -0.81 !       \  /  \  /  \
ATOM C5     CG2RC0  0.35 !        N6    N11   C13-ODA (-)
ATOM C6     CG2R64  0.66 !        |      |     ||
ATOM N6     NG2S1  -0.68 !        |     H11   ODB
ATOM H6     HGP1    0.32 !        C6
ATOM N1     NG2R62 -0.68 !       // \
ATOM C2     CG2R64  0.39 !      N1   C5--N7\\
ATOM H2     HGR62   0.14 !      |    ||     C8-H8
ATOM N3     NG2R62 -0.82 !      C2   C4--N9/
ATOM C4     CG2RC0  0.57 !     / \\ /      \
ATOM C10    CG2O6   0.60 !   H2   N3        \
ATOM O10    OG2D1  -0.50 !                   \
ATOM N11    NG2S1  -0.48 !                    \
ATOM H11    HGP1    0.29 !                     \
ATOM C12    CG321   0.00 ! O1P    H5' H4'  O4'  \
```

```
ATOM H121   HGA2    0.09 !  |       |     \ /   \  \
ATOM H122   HGA2    0.09 ! -P-O5'-C5'---C4'    C1'
GROUP                    !  |       |     \     / \
ATOM C13    CG2O3   0.52 ! O2P    H5''   C3'--C2' H1'
ATOM ODA    OG2D2  -0.76 !               / \   / \
ATOM ODB    OG2D2  -0.76 !            O3' H3' O2' H2''
GROUP                    !             |       |
ATOM P      P       1.50 !                    H2'
ATOM O1P    ON3    -0.78
ATOM O2P    ON3    -0.78 !  !!!! PATCH 6AH for the carboxylic acid form
ATOM O5'    ON2    -0.57
ATOM C5'    CN8B   -0.08
ATOM H5'    HN8     0.09
ATOM H5''   HN8     0.09
GROUP
ATOM C4'    CN7     0.16
ATOM H4'    HN7     0.09
ATOM O4'    ON6B   -0.50
ATOM C1'    CN7B    0.16
ATOM H1'    HN7     0.09
GROUP
ATOM C2'    CN7B    0.14
ATOM H2''   HN7     0.09
ATOM O2'    ON5    -0.66
ATOM H2'    HN5     0.43
GROUP
ATOM C3'    CN7     0.01
ATOM H3'    HN7     0.09
ATOM O3'    ON2    -0.57
BOND N9   C8     N9   C4      C8   N7      C8   H8
BOND N7   C5     C5   C6      C5   C4      C6   N6
BOND C6   N1     N6   C10     N6   H6      N1   C2
BOND C2   N3     C2   H2      N3   C4      C10  N11
BOND C10  O10    N11  C12     N11  H11     C12  C13
BOND C12  H121   C12  H122    C13  ODA     C13  ODB
BOND P    O1P     P    O2P     P    O5'     O5'  C5'     C5'  H5''
BOND C5'  C4'     C4'  O4'     C4'  C3'     O4'  C1'
BOND C1'  N9      C1'  C2'     C2'  C3'     C3'  O3'     O3'  +P
BOND C2'  O2'     O2'  H2'
BOND C1'  H1'     C2'  H2''    C3'  H3'     C4'  H4'     C5'  H5'
IMPR C10    N6     N11    O10    C13    ODB    ODA    C12    C6   C5   N1   N6
!ribose
IC -O3' P    O5'   C5'    1.6001  101.45  -39.25  119.00  1.4401
IC -O3' O5'  *P    O1P    1.6001  101.45 -115.82  109.74  1.4802
IC -O3' O5'  *P    O2P    1.6001  101.45  115.90  109.80  1.4801
IC  P   O5'  C5'   C4'    1.5996  119.00 -151.39  110.04  1.5160
IC O5'  C5'  C4'   C3'    1.4401  108.83 -179.85  116.10  1.5284
IC C5'  C4'  C3'   O3'    1.5160  116.10   76.70  115.12  1.4212
IC C4'  C3'  O3'   +P     1.5284  111.92  159.13  119.05  1.6001
IC C3'  O3'  +P    +O5'   1.4212  119.05  -98.86  101.45  1.5996
IC O4'  C3'  *C4'  C5'    1.4572  104.06 -120.04  116.10  1.5160
IC C2'  C4'  *C3'  O3'    1.5284  100.16 -124.08  115.12  1.4212
IC C4'  C3'  C2'   C1'    1.5284  100.16   39.58  102.04  1.5251
IC C3'  C2'  C1'   N9     1.5284  101.97  144.39  113.71  1.4896
IC O4'  C1'  N9    C4     1.5251  113.71  -97.2   125.59  1.3783
IC C1'  C3'  *C2'  O2'    1.5284  102.04 -114.67  110.81  1.4212
IC H2'  O2'  C2'   C3'    0.9600  114.97  148.63  111.92  1.5284
IC O4'  C2'  *C1'  H1'    0.0       0.0  -115.0     0.0   0.0
IC C1'  C3'  *C2'  H2''   0.0       0.0   115.0     0.0   0.0
IC C2'  C4'  *C3'  H3'    0.0       0.0   115.0     0.0   0.0
IC C3'  O4'  *C4'  H4'    0.0       0.0  -115.0     0.0   0.0
IC C4'  O5'  *C5'  H5'    0.0       0.0  -115.0     0.0   0.0
IC C4'  O5'  *C5'  H5''   0.0       0.0   115.0     0.0   0.0
```

```
IC C8   C4   *N9   C1'       1.3791  105.54 -179.95  126.56  1.4896
IC C4   N9   C8    N7        1.3838  106.01   -0.18  113.82  1.3262
IC N7   N9   *C8   H8        1.3262  113.82 -179.87  121.65  1.0916
IC N9   C8   N7    C5        1.3828  113.82    0.22  103.61  1.4034
IC C4   N7   *C5   C6        1.3958  110.73 -179.86  132.34  1.4076
IC N7   C5   C6    N6        1.4034  132.34   -3.02  118.94  1.4019
IC N6   C5   *C6   N1        1.4019  118.94 -175.12  117.58  1.3732
IC C5   C6   N6    C10       1.4076  118.94  176.19  127.17  1.3514
IC C10  C6   *N6   H6        1.3514  127.17  156.99  113.05  0.9866
IC C5   C6   N1    C2        1.4076  117.58   -2.46  120.65  1.3530
IC C6   N1   C2    N3        1.3732  120.65    1.29  124.73  1.3558
IC N3   N1   *C2   H2        1.3558  124.73  179.53  117.38  1.0932
IC C6   N6   C10   N11       1.4019  127.17  160.09  108.36  1.3555
IC N11  N6   *C10  O10       1.3555  108.36 -177.64  126.98  1.2220
IC N6   C10  N11   C12       1.3514  108.36 -149.68  123.43  1.4493
IC C12  C10  *N11  H11       1.4493  123.43  141.23  119.84  1.0009
IC C10  N11  C12   C13       1.3555  123.43  142.88  108.40  1.5366
IC C13  N11  *C12  H121      1.5366  108.40  118.35  110.82  1.1085
IC C13  N11  *C12  H122      1.5366  108.40 -118.83  112.29  1.1111
IC N11  C12  C13   ODA       1.4493  108.40  180.00  116.00  1.2600
IC ODA  C12  *C13  ODB       1.2600  116.00  180.00  116.00  1.2600
DONO H2'  O2'
DONO H11  N11
ACCE N3
ACCE N7
ACCE N1
ACCE O10  C10
ACCE ODA  C13
ACCE ODB  C13
ACCE O1P  P
ACCE O2P  P
ACCE O2'
ACCE O3'
ACCE O4'
ACCE O5'

!!***Guanosine***
RESI OMG           -1.00 ! 2'-O-methylguanosine, MRG
GROUP
ATOM N9     NN2B   -0.02 !            O6
ATOM C8     CN4     0.25 !            ||
ATOM H8     HN3     0.16 !            C6
ATOM N7     NN4    -0.60 !           /  \
ATOM C5     CN5G    0.00 !       H1-N1   C5--N7\\
ATOM C6     CN1     0.54 !          |    ||     C8-H8
ATOM O6     ON1    -0.51 !          C2   C4--N9/
ATOM N1     NN2G   -0.34 !         / \\ /      \
ATOM H1     HN2     0.26 !   H21-N2   N3        \
ATOM C2     CN2     0.75 !       |               \
ATOM N2     NN1    -0.68 !      H22               \
ATOM H21    HN1     0.32 !                         \
ATOM H22    HN1     0.35 !     O1P    H5' H4'  O4'  \
ATOM N3     NN3G   -0.74 !      |      |    \ /   \  \
ATOM C4     CN5     0.26 !     -P-O5'-C5'---C4'    C1'
GROUP                    !      |      |     \     / \
ATOM P      P       1.50 !     O2P    H5''   C3'--C2' H1'
ATOM O1P    ON3    -0.78 !                   / \   / \
ATOM O2P    ON3    -0.78 !                 O3' H3' O2' H2''
ATOM O5'    ON2    -0.57 !                 |        |
ATOM C5'    CN8B   -0.08 !                         CM2
ATOM H5'    HN8     0.09 !                        /  |  \
ATOM H5''   HN8     0.09 !                     HM1 HM2 HM3
GROUP                    !
```

```
ATOM C4'    CN7     0.16 !   !!! PATCH GENO for the enol form
ATOM H4'    HN7     0.09
ATOM O4'    ON6B   -0.50
ATOM C1'    CN7B    0.16
ATOM H1'    HN7     0.09
GROUP
ATOM C2'    CN7B    0.08
ATOM H2''   HN7     0.09
ATOM O2'    OG301  -0.34
ATOM CM2    CG331  -0.10
ATOM HM1    HGA3    0.09
ATOM HM2    HGA3    0.09
ATOM HM3    HGA3    0.09
GROUP
ATOM C3'    CN7     0.01
ATOM H3'    HN7     0.09
ATOM O3'    ON2    -0.57
BOND N9   C8      N9   C4      C4   N3      C4   C5      C5   N7
BOND C2   N2      C2   N1      C2   N3      N2   H21     N2   H22
BOND N1   H1      N1   C6      C6   C5      C6   O6      N7   C8
BOND C8   H8
BOND P    O1P     P    O2P     P    O5'     O5'  C5'     C5'  H5''
BOND C5'  C4'     C4'  O4'     C4'  C3'     O4'  C1'
BOND C1'  N9      C1'  C2'     C2'  C3'     C3'  O3'     O3'  +P
BOND C2'  O2'     CM2  O2'     CM2  HM1     HM2  CM2     HM3  CM2
BOND C1'  H1'     C2'  H2''    C3'  H3'     C4'  H4'     C5'  H5'
IMPR C2   N3   N1   N2        C6   N1   C5   O6        N2   H21  C2   H22
!2OM-ribose
IC -O3' P    O5'   C5'    1.6001  101.45  -39.25  119.00  1.4401
IC -O3' O5'  *P    O1P    1.6001  101.45 -115.82  109.74  1.4802
IC -O3' O5'  *P    O2P    1.6001  101.45  115.90  109.80  1.4801
IC  P   O5'  C5'   C4'    1.5996  119.00 -151.39  110.04  1.5160
IC O5'  C5'  C4'   C3'    1.4401  108.83 -179.85  116.10  1.5284
IC C5'  C4'  C3'   O3'    1.5160  116.10   76.70  115.12  1.4212
IC C4'  C3'  O3'   +P     1.5284  111.92  159.13  119.05  1.6001
IC C3'  O3'  +P    +O5'   1.4212  119.05  -98.86  101.45  1.5996
IC O4'  C3'  *C4'  C5'    1.4572  104.06 -120.04  116.10  1.5160
IC C2'  C4'  *C3'  O3'    1.5284  100.16 -124.08  115.12  1.4212
IC C4'  C3'  C2'   C1'    1.5284  100.16   39.58  102.04  1.5251
IC C3'  C2'  C1'   N9     1.5284  101.97  144.39  113.71  1.4896
IC O4'  C1'  N9    C4     1.5251  113.71  -97.2   125.59  1.3783
IC C3'  C1'  *C2'  O2'    1.5312  102.03  117.61  107.13  1.4206
IC C1'  C2'  O2'   CM2    1.5393  107.13   90.00  107.00  1.4150
IC C2'  O2'  CM2   HM2    1.4206  107.00  180.00    0.0   0.0
IC HM2  O2'  *CM2  HM3    0.0       0.0   120.00    0.0   0.0
IC HM2  O2'  *CM2  HM1    0.0       0.0  -120.00    0.0   0.0
IC O4'  C2'  *C1'  H1'    0.0       0.0  -115.0     0.0   0.0
IC C1'  C3'  *C2'  H2''   0.0       0.0   115.0     0.0   0.0
IC C2'  C4'  *C3'  H3'    0.0       0.0   115.0     0.0   0.0
IC C3'  O4'  *C4'  H4'    0.0       0.0  -115.0     0.0   0.0
IC C4'  O5'  *C5'  H5'    0.0       0.0  -115.0     0.0   0.0
IC C4'  O5'  *C5'  H5''   0.0       0.0   115.0     0.0   0.0
IC C8   C4   *N9   C1'       1.3791  105.54 -179.95  126.56  1.4896
IC C4   N9   C8    N7        1.3800  106.12    0.01  113.35  1.3074
IC N7   N9   *C8   H8        1.3074  113.35 -179.99  121.94  1.0910
IC C8   N9   C4    C5        1.3792  106.12    0.00  105.34  1.3833
IC C5   N9   *C4   N3        1.3833  105.34 -179.95  126.31  1.3506
IC N7   C4   *C5   C6        1.3889  110.94 -179.99  118.81  1.4195
IC C4   C5   C6    N1        1.3833  118.81    0.04  111.61  1.3907
IC N1   C5   *C6   O6        1.3907  111.61  179.96  128.55  1.2318
IC C5   C6   N1    C2        1.4195  111.61   -0.06  125.26  1.3725
IC C2   C6   *N1   H1        1.3725  125.26 -179.91  114.71  0.9985
IC N3   N1   *C2   N2        1.3238  123.75  179.95  116.18  1.3244
```

```
IC N1   C2   N2    H21       1.3725  116.18 -179.99  116.06  0.9953
IC H21  C2  *N2    H22       0.9953  116.06 -179.99  123.20  0.9912
DONO H21   N2
DONO H22   N2
DONO H1   N1
ACCE O6   C6
ACCE N3
ACCE N7
ACCE O1P  P
ACCE O2P  P
ACCE O2'
ACCE O3'
ACCE O4'
ACCE O5'

RESI RIG           -2.00 ! 2'-O-ribosylguanosine(phosphate), 2RG
GROUP
ATOM N9     NN2B   -0.02 !              O6
ATOM C8     CN4     0.25 !              ||
ATOM H8     HN3     0.16 !              C6
ATOM N7     NN4    -0.60 !             /  \
ATOM C5     CN5G    0.00 !         H1-N1   C5--N7\\
ATOM C6     CN1     0.54 !            |    ||     C8-H8
ATOM O6     ON1    -0.51 !            C2   C4--N9/
ATOM N1     NN2G   -0.34 !           / \\ /      \
ATOM H1     HN2     0.26 !     H21-N2   N3         \
ATOM C2     CN2     0.75 !         |               \
ATOM N2     NN1    -0.68 !        H22               \
ATOM H21    HN1     0.32 !                           \
ATOM H22    HN1     0.35 !                            \
ATOM N3     NN3G   -0.74 !        O1P    H5' H4'  O4'  \
ATOM C4     CN5     0.26 !         |      |    \ /   \  \
GROUP                    !        -P-O5' C5' --C4'    C1'
ATOM P      P       1.50 !         |      |     \     / \
ATOM O1P    ON3    -0.78 !        O2P    H5''   C3' -C2' H1'
ATOM O2P    ON3    -0.78 !                      / \   / \
ATOM O5'    ON2    -0.57 !                   O3' H3' O2' H2'
ATOM C5'    CN8B   -0.08 !                    |       \
ATOM H5'    HN8     0.09 !                             \
ATOM H5''   HN8     0.09 !                              \
GROUP                    !          O3X    H5A H4A  O4A  \
ATOM C4'    CN7     0.16 !          ||      |    \ /   \  \
ATOM H4'    HN7     0.09 !  HX -O1X-PA-O5A-C5A---C4A    C1A
ATOM O4'    ON6B   -0.50 !          |       |     \     / \
ATOM C1'    CN7B    0.16 !       (-)O2X    H5AA   C3A--C2A H1A
ATOM H1'    HN7     0.09 !                       / \   / \
GROUP                    !                    O3A H3A O2A H2A
ATOM C3'    CN7     0.01 !                    |       |
ATOM H3'    HN7     0.09 !                   H3OA    H2OA
ATOM O3'    ON2    -0.57 !
GROUP                    !   !!! PATCH GENO for the enol form
ATOM C2'    CN7B    0.09
ATOM H2'    HN7     0.09
ATOM O2'    OC301  -0.36
ATOM C1A    CC3152  0.29
ATOM H1A    HCA1    0.09
ATOM O4A    OC3C51 -0.40
ATOM C4A    CC3153  0.11
ATOM H4A    HCA1    0.09
GROUP
ATOM C2A    CC3151  0.14
ATOM H2A    HCA1    0.09
ATOM O2A    OC311  -0.65
```

```
ATOM H2OA   HCP1    0.42
GROUP
ATOM C3A    CC3151  0.14
ATOM H3A    HCA1    0.09
ATOM O3A    OC311  -0.65
ATOM H3OA   HCP1    0.42
GROUP
ATOM C5A    CC321  -0.08
ATOM H51A   HCA2    0.09
ATOM H52A   HCA2    0.09
ATOM O5A    OC30P  -0.62
ATOM PA     PC      1.50
ATOM O1X    OC312  -0.68
ATOM HX     HCP1    0.34
ATOM O2X    OC2DP  -0.82
ATOM O3X    OC2DP  -0.82
BOND N9   C8   N9   C4   C8   N7   C8   H8
BOND N7   C5   C5   C6   C5   C4   C6   O6
BOND C6   N1   N1   C2   N1   H1   C2   N2
BOND C2   N3   N2   H21  N2   H22  N3   C4
BOND P    O1P     P    O2P     P    O5'     O5'  C5'     C5'  H5''
BOND C5'  C4'     C4'  O4'     C4'  C3'     O4'  C1'
BOND C1'  N9      C1'  C2'     C2'  C3'     C3'  O3'     O3'  +P
BOND C2'  O2'     O2'  C1A
BOND C1'  H1'     C2'  H2'     C3'  H3'     C4'  H4'     C5'  H5'
BOND HX   O1X     O1X  PA      H51A C5A     H52A C5A     C5A  O5A
BOND C5A  C4A     PA   O5A     PA   O3X     PA   O2X     H4A  C4A
BOND H3A  C3A     C4A  C3A     C4A  O4A     O3A  C3A     O3A  H3OA
BOND C3A  C2A     H2OA O2A     O4A  C1A     C2A  O2A     C2A  C1A
BOND C2A  H2A     C1A  H1A
IMPR C2   N3   N1   N2        C6   N1   C5   O6        N2   H21  C2   H22
!ribose
IC -O3' P    O5'  C5'    1.6001  101.45  -39.25  119.00  1.4401
IC -O3' O5'  *P   O1P    1.6001  101.45 -115.82  109.74  1.4802
IC -O3' O5'  *P   O2P    1.6001  101.45  115.90  109.80  1.4801
IC  P   O5'  C5'  C4'    1.5996  119.00 -151.39  110.04  1.5160
IC O5'  C5'  C4'  C3'    1.4401  108.83 -179.85  116.10  1.5284
IC C5'  C4'  C3'  O3'    1.5160  116.10   76.70  115.12  1.4212
IC C4'  C3'  O3'  +P     1.5284  111.92  159.13  119.05  1.6001
IC C3'  O3'  +P   +O5'   1.4212  119.05  -98.86  101.45  1.5996
IC O4'  C3'  *C4' C5'    1.4572  104.06 -120.04  116.10  1.5160
IC C2'  C4'  *C3' O3'    1.5284  100.16 -124.08  115.12  1.4212
IC C4'  C3'  C2'  C1'    1.5284  100.16   39.58  102.04  1.5251
IC C3'  C2'  C1'  N9     1.5284  101.97  144.39  113.71  1.4896
IC O4'  C1'  N9   C4     1.5251  113.71  -97.2   125.59  1.3783
IC C1'  C3'  *C2' O2'    1.5284  102.04 -114.67  110.81  1.4212
IC O4'  C2'  *C1' H1'    0.0       0.0  -115.0     0.0   0.0
IC C1'  C3'  *C2' H2'    0.0       0.0   115.0     0.0   0.0
IC C2'  C4'  *C3' H3'    0.0       0.0   115.0     0.0   0.0
IC C3'  O4'  *C4' H4'    0.0       0.0  -115.0     0.0   0.0
IC C4'  O5'  *C5' H5'    0.0       0.0  -115.0     0.0   0.0
IC C4'  O5'  *C5' H5''   0.0       0.0   115.0     0.0   0.0
IC C1'  C2'  O2'  C1A       1.5063  107.56  106.98  111.64  1.4216
IC C2'  O2'  C1A  O4A       1.4298  111.64  -68.42  110.78  1.4403
IC O4A  O2'  *C1A C2A       1.4403  110.78 -113.45  103.46  1.5457
IC O4A  O2'  *C1A H1A       1.4403  110.78 -120.00  108.50  1.1000
IC O2'  C1A  O4A  C4A       1.4216  110.78  -95.22  109.95  1.4369
IC C1A  O4A  C4A  C5A       1.4403  109.95  136.29  115.40  1.5401
IC C5A  O4A  *C4A C3A       1.5401  115.40 -127.06  105.45  1.5322
IC C5A  O4A  *C4A H4A       1.5401  115.40 -120.00  107.30  1.1000
IC C3A  C1A  *C2A O2A       1.5383   99.33  118.90  113.47  1.4361
IC C3A  C1A  *C2A H2A       1.5383   99.33 -120.00  111.40  1.1000
IC C1A  C2A  O2A  H2OA      1.5457  113.47  180.00  109.00  0.9600
```

```
IC C2A  C4A  *C3A  O3A      1.5383  102.85 -125.22  112.10  1.4320
IC O3A  C4A  *C3A  H3A      1.4320  112.10 -120.00  111.40  1.1000
IC C4A  C3A  O3A   H3OA     1.5322  112.10  180.00  109.00  0.9600
IC O4A  C4A  C5A   O5A      1.4369  115.40 -149.51  108.85  1.4355
IC O5A  C4A  *C5A  H51A     1.4355  108.85  120.00  110.10  1.1110
IC H51A C4A  *C5A  H52A     1.1110  110.10 -120.00  110.10  1.1110
IC C4A  C5A  O5A   PA       1.5401  108.85 -167.20  121.22  1.6095
IC C5A  O5A  PA    O1X      1.4355  121.22   75.05  103.55  1.5871
IC O1X  O5A  *PA   O3X      1.5871  103.55  116.19  105.43  1.4997
IC O1X  O5A  *PA   O2X      1.5871  103.55 -113.52  107.57  1.5078
IC O5A  PA   O1X   HX       1.6095  103.55  180.00  115.00  0.9600
IC C8   C4   *N9   C1'      1.3791  105.54 -179.95  126.56  1.4896
IC C4   N9   C8    N7       1.3800  106.12    0.01  113.35  1.3074
IC N7   N9   *C8   H8       1.3074  113.35 -179.99  121.94  1.0910
IC C8   N9   C4    C5       1.3792  106.12    0.00  105.34  1.3833
IC C5   N9   *C4   N3       1.3833  105.34 -179.95  126.31  1.3506
IC N7   C4   *C5   C6       1.3889  110.94 -179.99  118.81  1.4195
IC C4   C5   C6    N1       1.3833  118.81    0.04  111.61  1.3907
IC N1   C5   *C6   O6       1.3907  111.61  179.96  128.55  1.2318
IC C5   C6   N1    C2       1.4195  111.61   -0.06  125.26  1.3725
IC C2   C6   *N1   H1       1.3725  125.26 -179.91  114.71  0.9985
IC N3   N1   *C2   N2       1.3238  123.75  179.95  116.18  1.3244
IC N1   C2   N2    H21      1.3725  116.18 -179.99  116.06  0.9953
IC H21  C2   *N2   H22      0.9953  116.06 -179.99  123.20  0.9912
DONO H2OA  O2A
DONO H3OA  O3A
DONO HX    O1X
DONO H21   N2
DONO H22   N2
DONO H1   N1
ACCE O6   C6
ACCE N3
ACCE N7
ACCE O1P  P
ACCE O2P  P
ACCE O2'
ACCE O3'
ACCE O4'
ACCE O5'
ACCE O4A
ACCE O2A
ACCE O3A
ACCE O1X
ACCE O2X
ACCE O3X  PA
ACCE O5A

RESI 1MG           -1.00 ! 1-methylguanosine
GROUP
ATOM N9     NG2R51  0.05 !    H11    O6
ATOM C8     CG2R53  0.25 !     |     ||
ATOM H8     HGR52   0.13 ! H12-CM1   C6
ATOM N7     NG2R50 -0.62 !     |  \ /  \
ATOM C5     CG2RC0 -0.01 !    H13  N1   C5--N7\\
ATOM C6     CG2R63  0.62 !         |    ||     C8-H8
ATOM O6     OG2D4  -0.50 !        C2   C4--N9/
ATOM N1     NG2R61 -0.21 !       / \\ /      \
ATOM C2     CG2R64  0.73 !  H21-N2   N3        \
ATOM N2     NG2S3  -0.64 !      |                \
ATOM H21    HGP4    0.32 !     H22                \
ATOM H22    HGP4    0.32 !                         \
ATOM N3     NG2R62 -0.77 !    O1P    H5' H4'  O4'   \
ATOM C4     CG2RC0  0.29 !     |      |    \ /   \  \
```

```
ATOM CM1    CG331  -0.23 !    -P-O5'-C5'---C4'    C1'
ATOM H11    HGA3    0.09 !     |      |     \     / \
ATOM H12    HGA3    0.09 !    O2P    H5''   C3'--C2' H1'
ATOM H13    HGA3    0.09 !                  / \   / \
GROUP                    !                O3' H3' O2' H2''
ATOM P      P       1.50 !                |       |
ATOM O1P    ON3    -0.78 !                        H2'
ATOM O2P    ON3    -0.78
ATOM O5'    ON2    -0.57
ATOM C5'    CN8B   -0.08
ATOM H5'    HN8     0.09
ATOM H5''   HN8     0.09
GROUP
ATOM C4'    CN7     0.16
ATOM H4'    HN7     0.09
ATOM O4'    ON6B   -0.50
ATOM C1'    CN7B    0.16
ATOM H1'    HN7     0.09
GROUP
ATOM C2'    CN7B    0.14
ATOM H2''   HN7     0.09
ATOM O2'    ON5    -0.66
ATOM H2'    HN5     0.43
GROUP
ATOM C3'    CN7     0.01
ATOM H3'    HN7     0.09
ATOM O3'    ON2    -0.57
BOND N9   C8   N9   C4   C8   N7   C8   H8
BOND N7   C5   C5   C6   C5   C4   C6   O6
BOND C6   N1   N1   C2   N1   CM1  C2   N2
BOND C2   N3   N2   H21  N2   H22  N3   C4
BOND CM1  H11  CM1  H12  CM1  H13
BOND P    O1P     P    O2P     P    O5'     O5'  C5'     C5'  H5''
BOND C5'  C4'     C4'  O4'     C4'  C3'     O4'  C1'
BOND C1'  N9      C1'  C2'     C2'  C3'     C3'  O3'     O3'  +P
BOND C2'  O2'     O2'  H2'
BOND C1'  H1'     C2'  H2''    C3'  H3'     C4'  H4'     C5'  H5'
IMPR C6     C5     N1     O6     C2     N1     N3     N2     N2     H22    H21    C2
!ribose
IC -O3' P    O5'   C5'    1.6001  101.45  -39.25  119.00  1.4401
IC -O3' O5'  *P    O1P    1.6001  101.45 -115.82  109.74  1.4802
IC -O3' O5'  *P    O2P    1.6001  101.45  115.90  109.80  1.4801
IC  P   O5'  C5'   C4'    1.5996  119.00 -151.39  110.04  1.5160
IC O5'  C5'  C4'   C3'    1.4401  108.83 -179.85  116.10  1.5284
IC C5'  C4'  C3'   O3'    1.5160  116.10   76.70  115.12  1.4212
IC C4'  C3'  O3'   +P     1.5284  111.92  159.13  119.05  1.6001
IC C3'  O3'  +P    +O5'   1.4212  119.05  -98.86  101.45  1.5996
IC O4'  C3'  *C4'  C5'    1.4572  104.06 -120.04  116.10  1.5160
IC C2'  C4'  *C3'  O3'    1.5284  100.16 -124.08  115.12  1.4212
IC C4'  C3'  C2'   C1'    1.5284  100.16   39.58  102.04  1.5251
IC C3'  C2'  C1'   N9     1.5284  101.97  144.39  113.71  1.4896
IC O4'  C1'  N9    C4     1.5251  113.71  -97.2   125.59  1.3783
IC C1'  C3'  *C2'  O2'    1.5284  102.04 -114.67  110.81  1.4212
IC H2'  O2'  C2'   C3'    0.9600  114.97  148.63  111.92  1.5284
IC O4'  C2'  *C1'  H1'    0.0       0.0  -115.0     0.0   0.0
IC C1'  C3'  *C2'  H2''   0.0       0.0   115.0     0.0   0.0
IC C2'  C4'  *C3'  H3'    0.0       0.0   115.0     0.0   0.0
IC C3'  O4'  *C4'  H4'    0.0       0.0  -115.0     0.0   0.0
IC C4'  O5'  *C5'  H5'    0.0       0.0  -115.0     0.0   0.0
IC C4'  O5'  *C5'  H5''   0.0       0.0   115.0     0.0   0.0
IC C8   C4   *N9   C1'       1.3791  105.54 -179.95  126.56  1.4896
IC C4   N9   C8    N7        1.3706  106.80   -0.25  113.01  1.3236
IC C8   N9   C4    C5        1.3756  106.80    0.28  104.92  1.3908
```

```
IC N9   C5   *C4   N3        1.3706  104.92 -178.38  128.79  1.3597
IC C5   C4   N3    C2        1.3908  128.79   -2.21  111.93  1.3155
IC C4   N3   C2    N1        1.3597  111.93    0.23  125.33  1.3775
IC N1   N3   *C2   N2        1.3775  125.33 -175.47  118.02  1.3874
IC N3   C2   N2    H21       1.3155  118.02  -11.87  109.98  1.0162
IC H21  C2   *N2   H22       1.0162  109.98 -128.49  115.91  1.0139
IC N3   C2   N1    C6        1.3155  125.33    2.33  124.21  1.4462
IC C6   C2   *N1   CM1       1.4462  124.21 -178.86  119.74  1.4639
IC C5   N1   *C6   O6        1.4356  110.25 -179.31  120.64  1.2270
IC N9   N7   *C8   H8        1.3756  113.01 -179.97  125.08  1.0827
IC C6   N1   CM1   H11       1.4462  116.04 -129.76  109.62  1.0911
IC H11  N1   *CM1  H12       1.0911  109.62 -121.75  111.06  1.0957
IC H11  N1   *CM1  H13       1.0911  109.62  120.02  106.67  1.0879
DONO H21   N2
DONO H22   N2
DONO H2'  O2'
ACCE O6   C6
ACCE N3
ACCE N7
ACCE O1P  P
ACCE O2P  P
ACCE O2'
ACCE O3'
ACCE O4'
ACCE O5'

RESI M1G           -1.00 ! 1,2'-O-dimethylguanosine
GROUP
ATOM N9     NG2R51  0.05 !    H11    O6
ATOM C8     CG2R53  0.25 !     |     ||
ATOM H8     HGR52   0.13 ! H12-CM1   C6
ATOM N7     NG2R50 -0.62 !     |  \ /  \
ATOM C5     CG2RC0 -0.01 !    H13  N1   C5--N7\\
ATOM C6     CG2R63  0.62 !         |    ||     C8-H8
ATOM O6     OG2D4  -0.50 !         C2   C4--N9/
ATOM N1     NG2R61 -0.21 !        / \\ /      \
ATOM C2     CG2R64  0.73 !  H21-N2   N3        \
ATOM N2     NG2S3  -0.64 !      |               \
ATOM H21    HGP4    0.32 !     H22               \
ATOM H22    HGP4    0.32 !                        \
ATOM N3     NG2R62 -0.77 !    O1P    H5' H4'  O4'  \
ATOM C4     CG2RC0  0.29 !     |      |    \ /   \  \
ATOM CM1    CG331  -0.23 !    -P-O5'-C5'---C4'    C1'
ATOM H11    HGA3    0.09 !     |      |     \     / \
ATOM H12    HGA3    0.09 !    O2P    H5''   C3'--C2' H1'
ATOM H13    HGA3    0.09 !                 / \   / \
GROUP                    !               O3' H3' O2' H2''
ATOM P      P       1.50 !                |       |
ATOM O1P    ON3    -0.78 !                        CM2
ATOM O2P    ON3    -0.78 !                       /  |  \
ATOM O5'    ON2    -0.57 !                     HM1 HM2 HM3
ATOM C5'    CN8B   -0.08
ATOM H5'    HN8     0.09
ATOM H5''   HN8     0.09
GROUP
ATOM C4'    CN7     0.16
ATOM H4'    HN7     0.09
ATOM O4'    ON6B   -0.50
ATOM C1'    CN7B    0.16
ATOM H1'    HN7     0.09
GROUP
ATOM C2'    CN7B    0.08
ATOM H2''   HN7     0.09
```

```
ATOM O2'    OG301  -0.34
ATOM CM2    CG331  -0.10
ATOM HM1    HGA3    0.09
ATOM HM2    HGA3    0.09
ATOM HM3    HGA3    0.09
GROUP
ATOM C3'    CN7     0.01
ATOM H3'    HN7     0.09
ATOM O3'    ON2    -0.57
BOND N9   C8   N9   C4   C8   N7   C8   H8
BOND N7   C5   C5   C6   C5   C4   C6   O6
BOND C6   N1   N1   C2   N1   CM1  C2   N2
BOND C2   N3   N2   H21  N2   H22  N3   C4
BOND CM1  H11  CM1  H12  CM1  H13
BOND P    O1P     P    O2P     P    O5'     O5'  C5'     C5'  H5''
BOND C5'  C4'     C4'  O4'     C4'  C3'     O4'  C1'
BOND C1'  N9      C1'  C2'     C2'  C3'     C3'  O3'     O3'  +P
BOND C2'  O2'     CM2  O2'     CM2  HM1     HM2  CM2     HM3  CM2
BOND C1'  H1'     C2'  H2''    C3'  H3'     C4'  H4'     C5'  H5'
IMPR C6     C5     N1     O6     C2     N1     N3     N2     N2     H22    H21    C2
!2OM-ribose
IC -O3' P    O5'  C5'    1.6001  101.45  -39.25  119.00  1.4401
IC -O3' O5'  *P   O1P    1.6001  101.45 -115.82  109.74  1.4802
IC -O3' O5'  *P   O2P    1.6001  101.45  115.90  109.80  1.4801
IC  P   O5'  C5'  C4'    1.5996  119.00 -151.39  110.04  1.5160
IC O5'  C5'  C4'  C3'    1.4401  108.83 -179.85  116.10  1.5284
IC C5'  C4'  C3'  O3'    1.5160  116.10   76.70  115.12  1.4212
IC C4'  C3'  O3'  +P     1.5284  111.92  159.13  119.05  1.6001
IC C3'  O3'  +P   +O5'   1.4212  119.05  -98.86  101.45  1.5996
IC O4'  C3'  *C4' C5'    1.4572  104.06 -120.04  116.10  1.5160
IC C2'  C4'  *C3' O3'    1.5284  100.16 -124.08  115.12  1.4212
IC C4'  C3'  C2'  C1'    1.5284  100.16   39.58  102.04  1.5251
IC C3'  C2'  C1'  N9     1.5284  101.97  144.39  113.71  1.4896
IC O4'  C1'  N9   C4     1.5251  113.71  -97.2   125.59  1.3783
IC C3'  C1'  *C2' O2'    1.5312  102.03  117.61  107.13  1.4206
IC C1'  C2'  O2'  CM2    1.5393  107.13   90.00  107.00  1.4150
IC C2'  O2'  CM2  HM2    1.4206  107.00  180.00    0.0   0.0
IC HM2  O2'  *CM2 HM3    0.0       0.0   120.00    0.0   0.0
IC HM2  O2'  *CM2 HM1    0.0       0.0  -120.00    0.0   0.0
IC O4'  C2'  *C1' H1'    0.0       0.0  -115.0     0.0   0.0
IC C1'  C3'  *C2' H2''   0.0       0.0   115.0     0.0   0.0
IC C2'  C4'  *C3' H3'    0.0       0.0   115.0     0.0   0.0
IC C3'  O4'  *C4' H4'    0.0       0.0  -115.0     0.0   0.0
IC C4'  O5'  *C5' H5'    0.0       0.0  -115.0     0.0   0.0
IC C4'  O5'  *C5' H5''   0.0       0.0   115.0     0.0   0.0
IC C8   C4   *N9  C1'       1.3791  105.54 -179.95  126.56  1.4896
IC C4   N9   C8   N7        1.3706  106.80   -0.25  113.01  1.3236
IC C8   N9   C4   C5        1.3756  106.80    0.28  104.92  1.3908
IC N9   C5   *C4  N3        1.3706  104.92 -178.38  128.79  1.3597
IC C5   C4   N3   C2        1.3908  128.79   -2.21  111.93  1.3155
IC C4   N3   C2   N1        1.3597  111.93    0.23  125.33  1.3775
IC N1   N3   *C2  N2        1.3775  125.33 -175.47  118.02  1.3874
IC N3   C2   N2   H21       1.3155  118.02  -11.87  109.98  1.0162
IC H21  C2   *N2  H22       1.0162  109.98 -128.49  115.91  1.0139
IC N3   C2   N1   C6        1.3155  125.33    2.33  124.21  1.4462
IC C6   C2   *N1  CM1       1.4462  124.21 -178.86  119.74  1.4639
IC C5   N1   *C6  O6        1.4356  110.25 -179.31  120.64  1.2270
IC N9   N7   *C8  H8        1.3756  113.01 -179.97  125.08  1.0827
IC C6   N1   CM1  H11       1.4462  116.04 -129.76  109.62  1.0911
IC H11  N1   *CM1 H12       1.0911  109.62 -121.75  111.06  1.0957
IC H11  N1   *CM1 H13       1.0911  109.62  120.02  106.67  1.0879
DONO H21   N2
DONO H22   N2
```

```
ACCE O6   C6
ACCE N3
ACCE N7
ACCE O1P  P
ACCE O2P  P
ACCE O2'
ACCE O3'
ACCE O4'
ACCE O5'

RESI 2MG           -1.00 ! N2-methylguanosine
GROUP
ATOM N9     NG2R51 -0.02 !             O6
ATOM C8     CG2R53  0.27 !             ||
ATOM H8     HGR52   0.14 !             C6
ATOM N7     NG2R50 -0.62 !            /  \
ATOM C5     CG2RC0  0.03 !        H1-N1   C5--N7\\
ATOM C6     CG2R63  0.52 !           |    ||     C8-H8
ATOM O6     OG2D4  -0.50 !           C2   C4--N9/
ATOM N1     NG2R61 -0.43 !          / \\ /      \
ATOM H1     HGP1    0.32 !    H2 -N2   N3        \
ATOM C4     CG2RC0  0.29 !        |               \
GROUP                    !       CM2               \
ATOM C2     CG2R64  0.68 !      / | \               \
ATOM N2     NG311  -0.47 !   H21 H22 H23             \
ATOM H2     HGPAM1  0.32 !                            \
ATOM N3     NG2R62 -0.77 !        O1P    H5' H4'  O4'  \
ATOM CM2    CG331  -0.03 !         |      |    \ /   \  \
ATOM H21    HGA3    0.09 !        -P-O5'-C5'---C4'    C1'
ATOM H22    HGA3    0.09 !         |      |     \     / \
ATOM H23    HGA3    0.09 !        O2P    H5''   C3'--C2' H1'
GROUP                    !                     / \   / \
ATOM P      P       1.50 !                   O3' H3' O2' H2''
ATOM O1P    ON3    -0.78 !                   |       |
ATOM O2P    ON3    -0.78 !                          H2'
ATOM O5'    ON2    -0.57
ATOM C5'    CN8B   -0.08
ATOM H5'    HN8     0.09
ATOM H5''   HN8     0.09
GROUP
ATOM C4'    CN7     0.16
ATOM H4'    HN7     0.09
ATOM O4'    ON6B   -0.50
ATOM C1'    CN7B    0.16
ATOM H1'    HN7     0.09
GROUP
ATOM C2'    CN7B    0.14
ATOM H2''   HN7     0.09
ATOM O2'    ON5    -0.66
ATOM H2'    HN5     0.43
GROUP
ATOM C3'    CN7     0.01
ATOM H3'    HN7     0.09
ATOM O3'    ON2    -0.57
BOND N9   C8   N9   C4   C8   N7   C8   H8
BOND N7   C5   C5   C6   C5   C4   C6   O6
BOND C6   N1   N1   C2   N1   H1   C2   N2
BOND C2   N3   N2   H2   N2   CM2  N3   C4
BOND CM2  H21  CM2  H22  CM2  H23
BOND P    O1P     P    O2P     P    O5'     O5'  C5'     C5'  H5''
BOND C5'  C4'     C4'  O4'     C4'  C3'     O4'  C1'
BOND C1'  N9      C1'  C2'     C2'  C3'     C3'  O3'     O3'  +P
BOND C2'  O2'     O2'  H2'
```

```
BOND C1'  H1'     C2'  H2''    C3'  H3'     C4'  H4'     C5'  H5'
IMPR C6     C5     N1     O6     C2     N1     N3     N2     ! N2   C2   CM2   H2
!ribose
IC -O3' P    O5'   C5'    1.6001  101.45  -39.25  119.00  1.4401
IC -O3' O5'  *P    O1P    1.6001  101.45 -115.82  109.74  1.4802
IC -O3' O5'  *P    O2P    1.6001  101.45  115.90  109.80  1.4801
IC  P   O5'  C5'   C4'    1.5996  119.00 -151.39  110.04  1.5160
IC O5'  C5'  C4'   C3'    1.4401  108.83 -179.85  116.10  1.5284
IC C5'  C4'  C3'   O3'    1.5160  116.10   76.70  115.12  1.4212
IC C4'  C3'  O3'   +P     1.5284  111.92  159.13  119.05  1.6001
IC C3'  O3'  +P    +O5'   1.4212  119.05  -98.86  101.45  1.5996
IC O4'  C3'  *C4'  C5'    1.4572  104.06 -120.04  116.10  1.5160
IC C2'  C4'  *C3'  O3'    1.5284  100.16 -124.08  115.12  1.4212
IC C4'  C3'  C2'   C1'    1.5284  100.16   39.58  102.04  1.5251
IC C3'  C2'  C1'   N9     1.5284  101.97  144.39  113.71  1.4896
IC O4'  C1'  N9    C4     1.5251  113.71  -97.2   125.59  1.3783
IC C1'  C3'  *C2'  O2'    1.5284  102.04 -114.67  110.81  1.4212
IC H2'  O2'  C2'   C3'    0.9600  114.97  148.63  111.92  1.5284
IC O4'  C2'  *C1'  H1'    0.0       0.0  -115.0     0.0   0.0
IC C1'  C3'  *C2'  H2''   0.0       0.0   115.0     0.0   0.0
IC C2'  C4'  *C3'  H3'    0.0       0.0   115.0     0.0   0.0
IC C3'  O4'  *C4'  H4'    0.0       0.0  -115.0     0.0   0.0
IC C4'  O5'  *C5'  H5'    0.0       0.0  -115.0     0.0   0.0
IC C4'  O5'  *C5'  H5''   0.0       0.0   115.0     0.0   0.0
IC C8   C4   *N9   C1'       1.3791  105.54 -179.95  126.56  1.4896
IC C4   N9   C8    N7        1.3452  107.51   -0.03  112.46  1.3213
IC N7   N9   *C8   H8        1.3213  112.46 -179.95  123.11  1.0926
IC N9   C8   N7    C5        1.3709  112.46    0.03  104.13  1.3957
IC C4   N7   *C5   C6        1.3922  109.82 -179.69  132.17  1.4128
IC N7   C5   C6    N1        1.3957  132.17 -179.93  113.01  1.3741
IC N1   C5   *C6   O6        1.3741  113.01  179.49  130.37  1.2307
IC C5   C6   N1    C2        1.4128  113.01   -0.65  126.11  1.3734
IC C2   C6   *N1   H1        1.3734  126.11 -177.56  114.59  0.9978
IC C6   N1   C2    N2        1.3741  126.11 -179.85  122.73  1.3539
IC N2   N1   *C2   N3        1.3539  122.73 -179.29  120.46  1.3394
IC N1   C2   N2    CM2       1.3734  122.73  -23.11  118.82  1.4827
IC CM2  C2   *N2   H21       1.4827  118.82 -130.64  109.14  1.0172
IC C2   N2   CM2   H21       1.3539  118.82  177.12  110.03  1.1136
IC H21  N2   *CM2  H22       1.1136  110.03  119.44  111.34  1.1123
IC H21  N2   *CM2  H23       1.1136  110.03 -118.71  111.74  1.1114
IC C2   CM2  *N2   H2     0.00      0.00  180.00    0.00  0.00
DONO H2   N2
DONO H1   N1
DONO H2'  O2'
ACCE O6   C6
ACCE N3
ACCE N7
ACCE O1P  P
ACCE O2P  P
ACCE O2'
ACCE O3'
ACCE O4'
ACCE O5'

RESI MMG           -1.00 ! N2,2'-O-dimethylguanosine
GROUP
ATOM N9     NG2R51 -0.02 !            O6
ATOM C8     CG2R53  0.27 !            ||
ATOM H8     HGR52   0.14 !            C6
ATOM N7     NG2R50 -0.62 !           /  \
ATOM C5     CG2RC0  0.03 !       H1-N1   C5--N7\\
ATOM C6     CG2R63  0.52 !           |    ||     C8-H8
ATOM O6     OG2D4  -0.50 !           C2   C4--N9/
```

```
ATOM N1     NG2R61 -0.43 !          / \\ /       \
ATOM H1     HGP1    0.32 !   H2 -N2   N3         \
ATOM C4     CG2RC0  0.29 !       |                \
GROUP                    !      CM2                \
ATOM C2     CG2R64  0.68 !     / | \                \
ATOM N2     NG311  -0.47 !  H21 H22 H23              \
ATOM H2     HGPAM1  0.32 !                            \
ATOM N3     NG2R62 -0.77 !       O1P    H5' H4'  O4'  \
ATOM CM2    CG331  -0.03 !        |      |    \ /   \  \
ATOM H21    HGA3    0.09 !       -P-O5'-C5'---C4'    C1'
ATOM H22    HGA3    0.09 !        |      |     \     / \
ATOM H23    HGA3    0.09
GROUP                    !       O2P    H5''   C3'--C2' H1'
ATOM P      P       1.50 !                    / \   / \
ATOM O1P    ON3    -0.78 !                 O3' H3' O2' H2''
ATOM O2P    ON3    -0.78 !                  |       |
ATOM O5'    ON2    -0.57 !                         CM2
ATOM C5'    CN8B   -0.08 !                        /  |  \
ATOM H5'    HN8     0.09 !                     HM1 HM2 HM3
ATOM H5''   HN8     0.09
GROUP
ATOM C4'    CN7     0.16
ATOM H4'    HN7     0.09
ATOM O4'    ON6B   -0.50
ATOM C1'    CN7B    0.16
ATOM H1'    HN7     0.09
GROUP
ATOM C2'    CN7B    0.08
ATOM H2''   HN7     0.09
ATOM O2'    OG301  -0.34
ATOM C2M    CG331  -0.10
ATOM HM1    HGA3    0.09
ATOM HM2    HGA3    0.09
ATOM HM3    HGA3    0.09
GROUP
ATOM C3'    CN7     0.01
ATOM H3'    HN7     0.09
ATOM O3'    ON2    -0.57
BOND N9   C8   N9   C4   C8   N7   C8   H8
BOND N7   C5   C5   C6   C5   C4   C6   O6
BOND C6   N1   N1   C2   N1   H1   C2   N2
BOND C2   N3   N2   H2   N2   CM2  N3   C4
BOND CM2  H21  CM2  H22  CM2  H23
BOND P    O1P     P    O2P     P    O5'     O5'  C5'     C5'  H5''
BOND C5'  C4'     C4'  O4'     C4'  C3'     O4'  C1'
BOND C1'  N9      C1'  C2'     C2'  C3'     C3'  O3'     O3'  +P
BOND C2'  O2'     C2M  O2'     C2M  HM1     HM2  C2M     HM3  C2M
BOND C1'  H1'     C2'  H2''    C3'  H3'     C4'  H4'     C5'  H5'
IMPR C6     C5     N1     O6     C2     N1     N3     N2     ! N2   C2   CM2   H2
!2OM-ribose
IC -O3' P    O5'   C5'    1.6001  101.45  -39.25  119.00  1.4401
IC -O3' O5'  *P    O1P    1.6001  101.45 -115.82  109.74  1.4802
IC -O3' O5'  *P    O2P    1.6001  101.45  115.90  109.80  1.4801
IC  P   O5'  C5'   C4'    1.5996  119.00 -151.39  110.04  1.5160
IC O5'  C5'  C4'   C3'    1.4401  108.83 -179.85  116.10  1.5284
IC C5'  C4'  C3'   O3'    1.5160  116.10   76.70  115.12  1.4212
IC C4'  C3'  O3'   +P     1.5284  111.92  159.13  119.05  1.6001
IC C3'  O3'  +P    +O5'   1.4212  119.05  -98.86  101.45  1.5996
IC O4'  C3'  *C4'  C5'    1.4572  104.06 -120.04  116.10  1.5160
IC C2'  C4'  *C3'  O3'    1.5284  100.16 -124.08  115.12  1.4212
IC C4'  C3'  C2'   C1'    1.5284  100.16   39.58  102.04  1.5251
IC C3'  C2'  C1'   N9     1.5284  101.97  144.39  113.71  1.4896
IC O4'  C1'  N9    C4     1.5251  113.71  -97.2   125.59  1.3783
```

```
IC C3'  C1'  *C2'  O2'    1.5312  102.03  117.61  107.13  1.4206
IC C1'  C2'  O2'   C2M    1.5393  107.13  180.00  107.00  1.4150
IC C2'  O2'  C2M   HM2    1.4206  107.00  180.00    0.0   0.0
IC HM2  O2'  *C2M  HM3    0.0       0.0   120.00    0.0   0.0
IC HM2  O2'  *C2M  HM1    0.0       0.0  -120.00    0.0   0.0
IC O4'  C2'  *C1'  H1'    0.0       0.0  -115.0     0.0   0.0
IC C1'  C3'  *C2'  H2''   0.0       0.0   115.0     0.0   0.0
IC C2'  C4'  *C3'  H3'    0.0       0.0   115.0     0.0   0.0
IC C3'  O4'  *C4'  H4'    0.0       0.0  -115.0     0.0   0.0
IC C4'  O5'  *C5'  H5'    0.0       0.0  -115.0     0.0   0.0
IC C4'  O5'  *C5'  H5''   0.0       0.0   115.0     0.0   0.0
IC C8   C4   *N9   C1'       1.3791  105.54 -179.95  126.56  1.4896
IC C4   N9   C8    N7        1.3452  107.51   -0.03  112.46  1.3213
IC N7   N9   *C8   H8        1.3213  112.46 -179.95  123.11  1.0926
IC N9   C8   N7    C5        1.3709  112.46    0.03  104.13  1.3957
IC C4   N7   *C5   C6        1.3922  109.82 -179.69  132.17  1.4128
IC N7   C5   C6    N1        1.3957  132.17 -179.93  113.01  1.3741
IC N1   C5   *C6   O6        1.3741  113.01  179.49  130.37  1.2307
IC C5   C6   N1    C2        1.4128  113.01   -0.65  126.11  1.3734
IC C2   C6   *N1   H1        1.3734  126.11 -177.56  114.59  0.9978
IC C6   N1   C2    N2        1.3741  126.11 -179.85  122.73  1.3539
IC N2   N1   *C2   N3        1.3539  122.73 -179.29  120.46  1.3394
IC N1   C2   N2    CM2       1.3734  122.73  -23.11  118.82  1.4827
IC CM2  C2   *N2   H21       1.4827  118.82 -130.64  109.14  1.0172
IC C2   N2   CM2   H21       1.3539  118.82  177.12  110.03  1.1136
IC H21  N2   *CM2  H22       1.1136  110.03  119.44  111.34  1.1123
IC H21  N2   *CM2  H23       1.1136  110.03 -118.71  111.74  1.1114
IC C2   CM2  *N2   H2     0.00      0.00  180.00    0.00  0.00
DONO H2   N2
DONO H1   N1
ACCE O6   C6
ACCE N3
ACCE N7
ACCE O1P  P
ACCE O2P  P
ACCE O2'
ACCE O3'
ACCE O4'
ACCE O5'

RESI 7MG            0.00 ! 7-methylguanosine
GROUP
ATOM N9     NG2R52 -0.10 !          O6     H71  H72
ATOM C8     CG2R53  0.40 !          ||       \ /
ATOM H8     HGR53   0.15 !          C6        CM7-H73
ATOM N7     NG2R52 -0.40 !         /  \   (+)/
ATOM C5     CG2RC0  0.13 !     H1-N1   C5--N7\\
ATOM C6     CG2R63  0.60 !         |    ||     C8-H8
ATOM O6     OG2D4  -0.45 !         C2   C4--N9/
ATOM N1     NG2R61 -0.29 !        / \\ /      \
ATOM H1     HGP1    0.25 !  H21-N2   N3        \
ATOM C2     CG2R64  0.73 !      |               \
ATOM N2     NG2S3  -0.69 !     H22               \
ATOM H21    HGP4    0.34 !                        \
ATOM H22    HGP4    0.34 !    O1P    H5' H4'  O4'  \
ATOM N3     NG2R62 -0.55 !     |      |    \ /   \  \
ATOM C4     CG2RC0  0.16 !    -P-O5'-C5'---C4'    C1'
ATOM CM7    CG334   0.11 !     |      |     \     / \
ATOM H71    HGA3    0.09 !    O2P    H5''   C3'--C2' H1'
ATOM H72    HGA3    0.09 !                 / \   / \
ATOM H73    HGA3    0.09 !               O3' H3' O2' H2''
GROUP                    !                |       |
ATOM P      P       1.50 !                        H2'
```

```
ATOM O1P    ON3    -0.78 !
ATOM O2P    ON3    -0.78 !   !!! PATCH 7MGE for the enol form
ATOM O5'    ON2    -0.57
ATOM C5'    CN8B   -0.08
ATOM H5'    HN8     0.09
ATOM H5''   HN8     0.09
GROUP
ATOM C4'    CN7     0.16
ATOM H4'    HN7     0.09
ATOM O4'    ON6B   -0.50
ATOM C1'    CN7B    0.16
ATOM H1'    HN7     0.09
GROUP
ATOM C2'    CN7B    0.14
ATOM H2''   HN7     0.09
ATOM O2'    ON5    -0.66
ATOM H2'    HN5     0.43
GROUP
ATOM C3'    CN7     0.01
ATOM H3'    HN7     0.09
ATOM O3'    ON2    -0.57
BOND N9   C8   N9   C4   C8   N7   C8   H8
BOND N7   C5   N7   CM7  C5   C6   C5   C4
BOND C6   O6   C6   N1   N1   C2   N1   H1
BOND C2   N2   C2   N3   N2   H21  N2   H22
BOND N3   C4   CM7  H71  CM7  H72  CM7  H73
BOND P    O1P     P    O2P     P    O5'     O5'  C5'     C5'  H5''
BOND C5'  C4'     C4'  O4'     C4'  C3'     O4'  C1'
BOND C1'  N9      C1'  C2'     C2'  C3'     C3'  O3'     O3'  +P
BOND C2'  O2'     O2'  H2'
BOND C1'  H1'     C2'  H2''    C3'  H3'     C4'  H4'     C5'  H5'
IMPR C6     C5     N1     O6     C2     N1     N3     N2     N2     H22    H21    C2
IC C8   C4   *N9   C1'      1.3511  107.49  180.00  125.45  1.4695
IC C4   N9   C8    N7       1.3797  109.30    0.23  108.85  1.3409
IC N7   N9   *C8   H8       1.3409  108.85  179.71  125.72  1.0820
IC N9   C8   N7    C5       1.3511  108.85    1.24  108.04  1.3782
IC C5   C8   *N7   CM7      1.3782  108.04  178.03  126.06  1.4751
IC C4   N7   *C5   C6       1.3883  108.18 -170.67  131.10  1.4435
IC N7   C5   C6    N1       1.3782  131.10  176.36  108.87  1.4146
IC N1   C5   *C6   O6       1.4146  108.87  179.20  128.38  1.2262
IC C5   C6   N1    C2       1.4435  108.87   -4.83  126.47  1.3822
IC C2   C6   *N1   H1       1.3822  126.47 -172.38  113.55  1.0211
IC C6   N1   C2    N2       1.4146  126.47 -175.33  117.26  1.3504
IC N2   N1   *C2   N3       1.3504  117.26  177.50  123.47  1.3271
IC N1   C2   N2    H21      1.3822  117.26 -172.78  116.15  1.0155
IC H21  C2   *N2   H22      1.0155  116.15  150.99  121.28  1.0131
IC C8   N7   CM7   H71      1.3409  126.06  125.37  108.67  1.0893
IC H71  N7   *CM7  H72      1.0893  108.67 -120.17  108.02  1.0902
IC H71  N7   *CM7  H73      1.0893  108.67  119.61  108.88  1.0896
!ribose
IC -O3' P    O5'   C5'    1.6001  101.45  -39.25  119.00  1.4401
IC -O3' O5'  *P    O1P    1.6001  101.45 -115.82  109.74  1.4802
IC -O3' O5'  *P    O2P    1.6001  101.45  115.90  109.80  1.4801
IC  P   O5'  C5'   C4'    1.5996  119.00 -151.39  110.04  1.5160
IC O5'  C5'  C4'   C3'    1.4401  108.83 -179.85  116.10  1.5284
IC C5'  C4'  C3'   O3'    1.5160  116.10   76.70  115.12  1.4212
IC C4'  C3'  O3'   +P     1.5284  111.92  159.13  119.05  1.6001
IC C3'  O3'  +P    +O5'   1.4212  119.05  -98.86  101.45  1.5996
IC O4'  C3'  *C4'  C5'    1.4572  104.06 -120.04  116.10  1.5160
IC C2'  C4'  *C3'  O3'    1.5284  100.16 -124.08  115.12  1.4212
IC C4'  C3'  C2'   C1'    1.5284  100.16   39.58  102.04  1.5251
IC C3'  C2'  C1'   N9     1.5284  101.97  144.39  113.71  1.4695
IC C4   N9   C1'   C2'    1.3797  125.45  124.52  112.36  1.5328
```

```
IC C1'  C3'  *C2'  O2'    1.5284  102.04 -114.67  110.81  1.4212
IC H2'  O2'  C2'   C3'    0.9600  114.97  148.63  111.92  1.5284
IC O4'  C2'  *C1'  H1'    0.0       0.0  -115.0     0.0   0.0
IC C1'  C3'  *C2'  H2''   0.0       0.0   115.0     0.0   0.0
IC C2'  C4'  *C3'  H3'    0.0       0.0   115.0     0.0   0.0
IC C3'  O4'  *C4'  H4'    0.0       0.0  -115.0     0.0   0.0
IC C4'  O5'  *C5'  H5'    0.0       0.0  -115.0     0.0   0.0
IC C4'  O5'  *C5'  H5''   0.0       0.0   115.0     0.0   0.0
DONO H21  N2
DONO H22  N2
DONO H1   N1
DONO H2'  O2'
ACCE O6   C6
ACCE N3
ACCE O1P  P
ACCE O2P  P
ACCE O2'
ACCE O3'
ACCE O4'
ACCE O5'

RESI 27G            0.00 ! N2,7-dimethylguanosine
GROUP
ATOM N9     NG2R52 -0.11 !              O6     H71  H72
ATOM C8     CG2R53  0.41 !              ||       \ /
ATOM H8     HGR53   0.15 !              C6        C7-H73
ATOM N7     NG2R52 -0.40 !             /  \    (+)/
ATOM C5     CG2RC0  0.13 !         H1-N1   C5--N7\\
ATOM C6     CG2R63  0.59 !            |    ||     C8-H8
ATOM O6     OG2D4  -0.46 !  H11       C2   C4--N9/
ATOM N1     NG2R61 -0.28 !     \     / \\ /      \
ATOM H1     HGP1    0.25 ! H12-C10-N2   N3        \
ATOM C2     CG2R64  0.73 !     /   |               \
ATOM N3     NG2R62 -0.57 !  H13    H2               \
ATOM C4     CG2RC0  0.14 !                           \
ATOM C7     CG334   0.15 !                            \
ATOM H71    HGA3    0.09 !        O1P    H5' H4'  O4'  \
ATOM H72    HGA3    0.09 !         |      |    \ /   \  \
ATOM H73    HGA3    0.09 !        -P-O5'-C5'---C4'    C1'
GROUP                    !         |      |     \     / \
ATOM N2     NG311  -0.52 !        O2P    H5''   C3'--C2' H1'
ATOM H2     HGPAM1  0.37 !                     / \   / \
ATOM C10    CG331  -0.12 !                   O3' H3' O2' H2''
ATOM H11    HGA3    0.09 !                    |       |
ATOM H12    HGA3    0.09 !                           H2'
ATOM H13    HGA3    0.09
GROUP
ATOM P      P       1.50
ATOM O1P    ON3    -0.78
ATOM O2P    ON3    -0.78
ATOM O5'    ON2    -0.57
ATOM C5'    CN8B   -0.08
ATOM H5'    HN8     0.09
ATOM H5''   HN8     0.09
GROUP
ATOM C4'    CN7     0.16
ATOM H4'    HN7     0.09
ATOM O4'    ON6B   -0.50
ATOM C1'    CN7B    0.16
ATOM H1'    HN7     0.09
GROUP
ATOM C2'    CN7B    0.14
ATOM H2''   HN7     0.09
```

```
ATOM O2'    ON5    -0.66
ATOM H2'    HN5     0.43
GROUP
ATOM C3'    CN7     0.01
ATOM H3'    HN7     0.09
ATOM O3'    ON2    -0.57
BOND N9   C8   N9   C4   C8   N7   C8   H8
BOND N7   C5   N7   C7   C5   C6   C5   C4
BOND C6   O6   C6   N1   N1   C2   N1   H1
BOND C2   N2   C2   N3   N2   H2   N2   C10
BOND N3   C4   C10  H11  C10  H12  C10  H13
BOND C7   H71  C7   H72  C7   H73
BOND P    O1P     P    O2P     P    O5'     O5'  C5'     C5'  H5''
BOND C5'  C4'     C4'  O4'     C4'  C3'     O4'  C1'
BOND C1'  N9      C1'  C2'     C2'  C3'     C3'  O3'     O3'  +P
BOND C2'  O2'     O2'  H2'
BOND C1'  H1'     C2'  H2''    C3'  H3'     C4'  H4'     C5'  H5'
IMPR C6     C5     N1     O6     C2     N1     N3     N2     ! N2   C2   C10   H2
IC C8   C4   *N9   C1'      1.3511  107.49  180.00  125.45  1.4695
IC C4   N9   C8    N7       1.3797  109.30    0.23  108.85  1.3409
IC N7   N9   *C8   H8       1.3409  108.85  179.71  125.72  1.0820
IC N9   C8   N7    C5       1.3511  108.85    1.24  108.04  1.3782
IC C5   C8   *N7   C7       1.4013  107.63 -179.95  126.60  1.4821
IC C4   N7   *C5   C6       1.3930  107.21 -179.87  133.17  1.4146
IC N7   C5   C6    N1       1.4013  133.17  179.86  112.75  1.3804
IC N1   C5   *C6   O6       1.3804  112.75  179.88  128.96  1.2295
IC C5   C6   N1    C2       1.4146  112.75   -0.11  126.42  1.3768
IC C2   C6   *N1   H1       1.3768  126.42 -179.36  116.59  1.0008
IC C6   N1   C2    N2       1.3804  126.42 -179.29  121.20  1.3532
IC N2   N1   *C2   N3       1.3532  121.20  179.61  119.59  1.3496
IC N1   C2   N2    C10      1.3768  121.20  -15.98  126.61  1.4860
IC C10  C2   *N2   H2       1.4860  126.61 -149.79  113.03  1.0180
IC C8   N7   C7    H71      1.3363  126.60    0.02  111.09  1.1138
IC H71  N7   *C7   H72      1.1138  111.09  120.39  109.21  1.1140
IC H71  N7   *C7   H73      1.1138  111.09 -120.50  109.25  1.1123
IC C2   N2   C10   H11      1.3532  126.61  -87.46  110.97  1.1130
IC H11  N2   *C10  H12      1.1130  110.97  121.68  112.71  1.1107
IC H11  N2   *C10  H13      1.1130  110.97 -118.99  110.15  1.1139
!ribose
IC -O3' P    O5'   C5'   1.6001  101.45  -39.25  119.00  1.4401
IC -O3' O5'  *P    O1P   1.6001  101.45 -115.82  109.74  1.4802
IC -O3' O5'  *P    O2P   1.6001  101.45  115.90  109.80  1.4801
IC  P   O5'  C5'   C4'   1.5996  119.00 -151.39  110.04  1.5160
IC O5'  C5'  C4'   C3'   1.4401  108.83 -179.85  116.10  1.5284
IC C5'  C4'  C3'   O3'   1.5160  116.10   76.70  115.12  1.4212
IC C4'  C3'  O3'   +P    1.5284  111.92  159.13  119.05  1.6001
IC C3'  O3'  +P    +O5'  1.4212  119.05  -98.86  101.45  1.5996
IC O4'  C3'  *C4'  C5'   1.4572  104.06 -120.04  116.10  1.5160
IC C2'  C4'  *C3'  O3'   1.5284  100.16 -124.08  115.12  1.4212
IC C4'  C3'  C2'   C1'   1.5284  100.16   39.58  102.04  1.5251
IC C3'  C2'  C1'   N9    1.5284  101.97  144.39  113.71  1.4695
IC C4   N9   C1'   C2'   1.3797  125.45  124.52  112.36  1.5328
IC C1'  C3'  *C2'  O2'   1.5284  102.04 -114.67  110.81  1.4212
IC H2'  O2'  C2'   C3'   0.9600  114.97  148.63  111.92  1.5284
IC O4'  C2'  *C1'  H1'   0.0       0.0  -115.0     0.0   0.0
IC C1'  C3'  *C2'  H2''  0.0       0.0   115.0     0.0   0.0
IC C2'  C4'  *C3'  H3'   0.0       0.0   115.0     0.0   0.0
IC C3'  O4'  *C4'  H4'   0.0       0.0  -115.0     0.0   0.0
IC C4'  O5'  *C5'  H5'   0.0       0.0  -115.0     0.0   0.0
IC C4'  O5'  *C5'  H5''  0.0       0.0   115.0     0.0   0.0
DONO H2'  O2'
DONO H2    N2
DONO H1   N1
```

```
ACCE O6   C6
ACCE N3
ACCE O1P  P
ACCE O2P  P
ACCE O2'
ACCE O3'
ACCE O4'
ACCE O5'

RESI M7G            0.00 ! N2,7,2'-O-trimethylguanosine
GROUP
ATOM N9     NG2R52 -0.11 !               O6     H71  H72
ATOM C8     CG2R53  0.41 !               ||       \ /
ATOM H8     HGR53   0.15 !               C6        C7-H73
ATOM N7     NG2R52 -0.40 !              /  \   (+)/
ATOM C5     CG2RC0  0.13 !          H1-N1   C5--N7\\
ATOM C6     CG2R63  0.59 !             |    ||     C8-H8
ATOM O6     OG2D4  -0.46 !   H11       C2   C4--N9/
ATOM N1     NG2R61 -0.28 !      \     / \\ /      \
ATOM H1     HGP1    0.25 !  H12-C10-N2   N3        \
ATOM C2     CG2R64  0.73 !      /   |               \
ATOM N3     NG2R62 -0.57 !   H13    H2               \
ATOM C4     CG2RC0  0.14 !                            \
ATOM C7     CG334   0.15 !                             \
ATOM H71    HGA3    0.09 !         O1P    H5' H4'  O4'  \
ATOM H72    HGA3    0.09 !          |      |    \ /   \  \
ATOM H73    HGA3    0.09 !         -P-O5'-C5'---C4'    C1'
GROUP                    !          |      |     \     / \
ATOM N2     NG311  -0.52 !         O2P    H5''   C3'--C2' H1'
ATOM H2     HGPAM1  0.37 !                      / \   / \
ATOM C10    CG331  -0.12 !                    O3' H3' O2' H2'
ATOM H11    HGA3    0.09 !                     |      | '
ATOM H12    HGA3    0.09 !                           CM2
ATOM H13    HGA3    0.09 !                         /  |  \
GROUP                    !                       HM1 HM2 HM3
ATOM P      P       1.50
ATOM O1P    ON3    -0.78
ATOM O2P    ON3    -0.78
ATOM O5'    ON2    -0.57
ATOM C5'    CN8B   -0.08
ATOM H5'    HN8     0.09
ATOM H5''   HN8     0.09
GROUP
ATOM C4'    CN7     0.16
ATOM H4'    HN7     0.09
ATOM O4'    ON6B   -0.50
ATOM C1'    CN7B    0.16
ATOM H1'    HN7     0.09
GROUP
ATOM C2'    CN7B    0.08
ATOM H2''   HN7     0.09
ATOM O2'    OG301  -0.34
ATOM CM2    CG331  -0.10
ATOM HM1    HGA3    0.09
ATOM HM2    HGA3    0.09
ATOM HM3    HGA3    0.09
GROUP
ATOM C3'    CN7     0.01
ATOM H3'    HN7     0.09
ATOM O3'    ON2    -0.57
BOND N9   C8   N9   C4   C8   N7   C8   H8
BOND N7   C5   N7   C7   C5   C6   C5   C4
BOND C6   O6   C6   N1   N1   C2   N1   H1
```

```
BOND C2   N2   C2   N3   N2   H2   N2   C10
BOND N3   C4   C10  H11  C10  H12  C10  H13
BOND C7   H71  C7   H72  C7   H73
BOND P    O1P     P    O2P     P    O5'     O5'  C5'     C5'  H5''
BOND C5'  C4'     C4'  O4'     C4'  C3'     O4'  C1'
BOND C1'  N9      C1'  C2'     C2'  C3'     C3'  O3'     O3'  +P
BOND C2'  O2'     CM2  O2'     CM2  HM1     HM2  CM2     HM3  CM2
BOND C1'  H1'     C2'  H2''    C3'  H3'     C4'  H4'     C5'  H5'
IMPR C6     C5     N1     O6     C2     N1     N3     N2     ! N2   C2   C10   H2
IC C8   C4   *N9   C1'      1.3511  107.49  180.00  125.45  1.4695
IC C4   N9   C8    N7       1.3797  109.30    0.23  108.85  1.3409
IC N7   N9   *C8   H8       1.3409  108.85  179.71  125.72  1.0820
IC N9   C8   N7    C5       1.3511  108.85    1.24  108.04  1.3782
IC C5   C8   *N7   C7       1.4013  107.63 -179.95  126.60  1.4821
IC C4   N7   *C5   C6       1.3930  107.21 -179.87  133.17  1.4146
IC N7   C5   C6    N1       1.4013  133.17  179.86  112.75  1.3804
IC N1   C5   *C6   O6       1.3804  112.75  179.88  128.96  1.2295
IC C5   C6   N1    C2       1.4146  112.75   -0.11  126.42  1.3768
IC C2   C6   *N1   H1       1.3768  126.42 -179.36  116.59  1.0008
IC C6   N1   C2    N2       1.3804  126.42 -179.29  121.20  1.3532
IC N2   N1   *C2   N3       1.3532  121.20  179.61  119.59  1.3496
IC N1   C2   N2    C10      1.3768  121.20  -15.98  126.61  1.4860
IC C10  C2   *N2   H2       1.4860  126.61 -149.79  113.03  1.0180
IC C8   N7   C7    H71      1.3363  126.60    0.02  111.09  1.1138
IC H71  N7   *C7   H72      1.1138  111.09  120.39  109.21  1.1140
IC H71  N7   *C7   H73      1.1138  111.09 -120.50  109.25  1.1123
IC C2   N2   C10   H11      1.3532  126.61  -87.46  110.97  1.1130
IC H11  N2   *C10  H12      1.1130  110.97  121.68  112.71  1.1107
IC H11  N2   *C10  H13      1.1130  110.97 -118.99  110.15  1.1139
!2OM-ribose
IC -O3' P    O5'   C5'    1.6001  101.45  -39.25  119.00  1.4401
IC -O3' O5'  *P    O1P    1.6001  101.45 -115.82  109.74  1.4802
IC -O3' O5'  *P    O2P    1.6001  101.45  115.90  109.80  1.4801
IC  P   O5'  C5'   C4'    1.5996  119.00 -151.39  110.04  1.5160
IC O5'  C5'  C4'   C3'    1.4401  108.83 -179.85  116.10  1.5284
IC C5'  C4'  C3'   O3'    1.5160  116.10   76.70  115.12  1.4212
IC C4'  C3'  O3'   +P     1.5284  111.92  159.13  119.05  1.6001
IC C3'  O3'  +P    +O5'   1.4212  119.05  -98.86  101.45  1.5996
IC O4'  C3'  *C4'  C5'    1.4572  104.06 -120.04  116.10  1.5160
IC C2'  C4'  *C3'  O3'    1.5284  100.16 -124.08  115.12  1.4212
IC C4'  C3'  C2'   C1'    1.5284  100.16   39.58  102.04  1.5251
IC C3'  C2'  C1'   N9     1.5284  101.97  144.39  113.71  1.4695
IC C4   N9   C1'   C2'    1.3797  125.45  124.52  112.36  1.5328
IC C3'  C1'  *C2'  O2'    1.5312  102.03  117.61  107.13  1.4206
IC C1'  C2'  O2'   CM2    1.5393  107.13   90.00  107.00  1.4150
IC C2'  O2'  CM2   HM2    1.4206  107.00  180.00    0.0   0.0
IC HM2  O2'  *CM2  HM3    0.0       0.0   120.00    0.0   0.0
IC HM2  O2'  *CM2  HM1    0.0       0.0  -120.00    0.0   0.0
IC O4'  C2'  *C1'  H1'    0.0       0.0  -115.0     0.0   0.0
IC C1'  C3'  *C2'  H2''   0.0       0.0   115.0     0.0   0.0
IC C2'  C4'  *C3'  H3'    0.0       0.0   115.0     0.0   0.0
IC C3'  O4'  *C4'  H4'    0.0       0.0  -115.0     0.0   0.0
IC C4'  O5'  *C5'  H5'    0.0       0.0  -115.0     0.0   0.0
IC C4'  O5'  *C5'  H5''   0.0       0.0   115.0     0.0   0.0
DONO H2    N2
DONO H1   N1
ACCE O6   C6
ACCE N3
ACCE O1P  P
ACCE O2P  P
ACCE O2'
ACCE O3'
ACCE O4'
```

```
ACCE O5'

RESI M2G           -1.00 ! N2,N2-dimethylguanosine, DMG      12/19
GROUP
ATOM N9     NG2R51 -0.01 !               O6
ATOM C8     CG2R53  0.33 !               ||
ATOM H8     HGR52   0.10 !               C6
ATOM N7     NG2R50 -0.65 !              /  \
ATOM C5     CG2RC0  0.01 !          H1-N1   C5--N7\\
ATOM C6     CG2R63  0.49 !              |    ||     C8-H8
ATOM O6     OG2D4  -0.50 !  H21        C2   C4--N9/
ATOM N1     NG2R61 -0.45 !     \      / \\ /      \
ATOM H1     HGP1    0.33 !  H22-CM2-N2   N3        \
ATOM C4     CG2RC0  0.34 !     /    |               \
GROUP                    !  H23    CM1               \
ATOM C2     CG2R64  0.71 !        / | \               \
ATOM N2     NG301  -0.36 !     H11 H12 H13             \
ATOM N3     NG2R62 -0.62 !                              \
ATOM CM1    CG331  -0.13 !          O1P    H5' H4'  O4'  \
ATOM H11    HGA3    0.09 !           |      |    \ /   \  \
ATOM H12    HGA3    0.09 !          -P-O5'-C5'---C4'    C1'
ATOM H13    HGA3    0.09 !           |      |     \     / \
ATOM CM2    CG331  -0.13 !          O2P    H5''   C3'--C2' H1'
ATOM H21    HGA3    0.09 !                       / \   / \
ATOM H22    HGA3    0.09 !                     O3' H3' O2' H2''
ATOM H23    HGA3    0.09 !                      |       |
GROUP                    !                             H2'
ATOM P      P       1.50
ATOM O1P    ON3    -0.78
ATOM O2P    ON3    -0.78
ATOM O5'    ON2    -0.57
ATOM C5'    CN8B   -0.08
ATOM H5'    HN8     0.09
ATOM H5''   HN8     0.09
GROUP
ATOM C4'    CN7     0.16
ATOM H4'    HN7     0.09
ATOM O4'    ON6B   -0.50
ATOM C1'    CN7B    0.16
ATOM H1'    HN7     0.09
GROUP
ATOM C2'    CN7B    0.14
ATOM H2''   HN7     0.09
ATOM O2'    ON5    -0.66
ATOM H2'    HN5     0.43
GROUP
ATOM C3'    CN7     0.01
ATOM H3'    HN7     0.09
ATOM O3'    ON2    -0.57
BOND N9   C8   N9   C4   C8   N7   C8   H8
BOND N7   C5   C5   C6   C5   C4   C6   O6
BOND C6   N1   N1   C2   N1   H1   C2   N2
BOND C2   N3   N2   CM2  N2   CM1  N3   C4
BOND CM2  H21  CM2  H22  CM2  H23  CM1  H11
BOND CM1  H12  CM1  H13
BOND P    O1P     P    O2P     P    O5'     O5'  C5'     C5'  H5''
BOND C5'  C4'     C4'  O4'     C4'  C3'     O4'  C1'
BOND C1'  N9      C1'  C2'     C2'  C3'     C3'  O3'     O3'  +P
BOND C2'  O2'     O2'  H2'
BOND C1'  H1'     C2'  H2''    C3'  H3'     C4'  H4'     C5'  H5'
IMPR C6     C5     N1     O6     C2     N1     N3     N2
IC C8   C4   *N9   C1'      1.3791  105.54 -179.95  126.56  1.4896
IC C4   N9   C8    N7       1.3718  107.05    0.15  112.92  1.3239
```

```
IC N7   N9   *C8   H8        1.3239  112.92 -179.94  121.93  1.0821
IC N9   C8   N7    C5        1.3749  112.92   -0.01  103.74  1.3787
IC C4   N7   *C5   C6        1.3936  111.71 -179.99  129.83  1.4385
IC N7   C5   C6    N1        1.3787  129.83 -179.76  109.33  1.4303
IC N1   C5   *C6   O6        1.4303  109.33  179.92  131.19  1.2270
IC C5   C6   N1    C2        1.4385  109.33   -0.43  127.35  1.3777
IC C2   C6   *N1   H1        1.3777  127.35 -178.10  112.07  1.0140
IC C6   N1   C2    N2        1.4303  127.35 -176.85  117.34  1.3783
IC N2   N1   *C2   N3        1.3783  117.34  177.94  122.89  1.3186
IC N1   C2   N2    CM2       1.3777  117.34  -23.49  119.27  1.4568
IC CM2  C2   *N2   CM1       1.4568  119.27 -146.65  115.92  1.4600
IC C2   N2   CM1   H11       1.3783  115.92 -165.35  108.06  1.0921
IC H11  N2   *CM1  H12       1.0921  108.06  119.04  108.98  1.0872
IC H11  N2   *CM1  H13       1.0921  108.06 -120.74  112.21  1.0983
IC C2   N2   CM2   H21       1.3783  119.27 -177.38  107.91  1.0903
IC H21  N2   *CM2  H22       1.0903  107.91  120.05  112.93  1.0983
IC H21  N2   *CM2  H23       1.0903  107.91 -116.75  111.13  1.0946
!ribose
IC -O3' P    O5'   C5'    1.6001  101.45  -39.25  119.00  1.4401
IC -O3' O5'  *P    O1P    1.6001  101.45 -115.82  109.74  1.4802
IC -O3' O5'  *P    O2P    1.6001  101.45  115.90  109.80  1.4801
IC  P   O5'  C5'   C4'    1.5996  119.00 -151.39  110.04  1.5160
IC O5'  C5'  C4'   C3'    1.4401  108.83 -179.85  116.10  1.5284
IC C5'  C4'  C3'   O3'    1.5160  116.10   76.70  115.12  1.4212
IC C4'  C3'  O3'   +P     1.5284  111.92  159.13  119.05  1.6001
IC C3'  O3'  +P    +O5'   1.4212  119.05  -98.86  101.45  1.5996
IC O4'  C3'  *C4'  C5'    1.4572  104.06 -120.04  116.10  1.5160
IC C2'  C4'  *C3'  O3'    1.5284  100.16 -124.08  115.12  1.4212
IC C4'  C3'  C2'   C1'    1.5284  100.16   39.58  102.04  1.5251
IC C3'  C2'  C1'   N9     1.5284  101.97  144.39  113.71  1.4896
IC O4'  C1'  N9    C4     1.5251  113.71  -97.2   125.59  1.3783
IC C1'  C3'  *C2'  O2'    1.5284  102.04 -114.67  110.81  1.4212
IC H2'  O2'  C2'   C3'    0.9600  114.97  148.63  111.92  1.5284
IC O4'  C2'  *C1'  H1'    0.0       0.0  -115.0     0.0   0.0
IC C1'  C3'  *C2'  H2''   0.0       0.0   115.0     0.0   0.0
IC C2'  C4'  *C3'  H3'    0.0       0.0   115.0     0.0   0.0
IC C3'  O4'  *C4'  H4'    0.0       0.0  -115.0     0.0   0.0
IC C4'  O5'  *C5'  H5'    0.0       0.0  -115.0     0.0   0.0
IC C4'  O5'  *C5'  H5''   0.0       0.0   115.0     0.0   0.0
DONO H1   N1
DONO H2'  O2'
ACCE O6   C6
ACCE N3
ACCE N7
ACCE O1P  P
ACCE O2P  P
ACCE O2'
ACCE O3'
ACCE O4'
ACCE O5'

RESI MTG           -1.00 ! N2,N2,2'-O-trimethylguanosine
GROUP
ATOM N9     NG2R51 -0.01 !                O6
ATOM C8     CG2R53  0.33 !                ||
ATOM H8     HGR52   0.10 !                C6
ATOM N7     NG2R50 -0.65 !               /  \
ATOM C5     CG2RC0  0.01 !           H1-N1   C5--N7\\
ATOM C6     CG2R63  0.49 !              |    ||     C8-H8
ATOM O6     OG2D4  -0.50 !   H21        C2   C4--N9/
ATOM N1     NG2R61 -0.45 !      \      / \\ /      \
ATOM H1     HGP1    0.33 !   H22-CM2-N2   N3        \
ATOM C4     CG2RC0  0.34 !      /    |               \
```

```
GROUP                    !   H23    CM1                 \
ATOM C2      CG2R64  0.71 !         / | \                \
ATOM N2      NG301  -0.36 !      H11 H12 H13              \
ATOM N3      NG2R62 -0.62 !                                \
ATOM CM1     CG331  -0.13 !           O1P    H5' H4'  O4'  \
ATOM H11     HGA3    0.09 !            |      |    \ /   \  \
ATOM H12     HGA3    0.09 !           -P-O5'-C5'---C4'    C1'
ATOM H13     HGA3    0.09 !            |      |     \     / \
ATOM CM2     CG331  -0.13 !           O2P    H5''   C3'--C2' H1'
ATOM H21     HGA3    0.09 !                        / \   / \
ATOM H22     HGA3    0.09 !                     O3' H3' O2' H2''
ATOM H23     HGA3    0.09 !                       |       |
GROUP                     !                              CM2
ATOM P       P       1.50 !                            /  |  \
ATOM O1P     ON3    -0.78 !                         HM1 HM2 HM3
ATOM O2P     ON3    -0.78
ATOM O5'     ON2    -0.57
ATOM C5'     CN8B   -0.08
ATOM H5'     HN8     0.09
ATOM H5''    HN8     0.09
GROUP
ATOM C4'     CN7     0.16
ATOM H4'     HN7     0.09
ATOM O4'     ON6B   -0.50
ATOM C1'     CN7B    0.16
ATOM H1'     HN7     0.09
GROUP
ATOM C2'     CN7B    0.08
ATOM H2''    HN7     0.09
ATOM O2'     OG301  -0.34
ATOM C2M     CG331  -0.10
ATOM HM1     HGA3    0.09
ATOM HM2     HGA3    0.09
ATOM HM3     HGA3    0.09
GROUP
ATOM C3'     CN7     0.01
ATOM H3'     HN7     0.09
ATOM O3'     ON2    -0.57
BOND N9   C8   N9   C4   C8   N7   C8   H8
BOND N7   C5   C5   C6   C5   C4   C6   O6
BOND C6   N1   N1   C2   N1   H1   C2   N2
BOND C2   N3   N2   CM2  N2   CM1  N3   C4
BOND CM2  H21  CM2  H22  CM2  H23  CM1  H11
BOND CM1  H12  CM1  H13
BOND P    O1P     P    O2P     P    O5'     O5'  C5'     C5'  H5''
BOND C5'  C4'     C4'  O4'     C4'  C3'     O4'  C1'
BOND C1'  N9      C1'  C2'     C2'  C3'     C3'  O3'     O3'  +P
BOND C2'  O2'     C2M  O2'     C2M  HM1     HM2  C2M     HM3  C2M
BOND C1'  H1'     C2'  H2''    C3'  H3'     C4'  H4'     C5'  H5'
IMPR C6     C5     N1     O6     C2     N1     N3     N2
IC C8   C4   *N9   C1'      1.3791  105.54 -179.95  126.56  1.4896
IC C4   N9   C8    N7       1.3718  107.05    0.15  112.92  1.3239
IC N7   N9   *C8   H8       1.3239  112.92 -179.94  121.93  1.0821
IC N9   C8   N7    C5       1.3749  112.92   -0.01  103.74  1.3787
IC C4   N7   *C5   C6       1.3936  111.71 -179.99  129.83  1.4385
IC N7   C5   C6    N1       1.3787  129.83 -179.76  109.33  1.4303
IC N1   C5   *C6   O6       1.4303  109.33  179.92  131.19  1.2270
IC C5   C6   N1    C2       1.4385  109.33   -0.43  127.35  1.3777
IC C2   C6   *N1   H1       1.3777  127.35 -178.10  112.07  1.0140
IC C6   N1   C2    N2       1.4303  127.35 -176.85  117.34  1.3783
IC N2   N1   *C2   N3       1.3783  117.34  177.94  122.89  1.3186
IC N1   C2   N2    CM2      1.3777  117.34  -23.49  119.27  1.4568
IC CM2  C2   *N2   CM1      1.4568  119.27 -146.65  115.92  1.4600
```

```
IC C2   N2   CM1  H11      1.3783  115.92 -165.35  108.06  1.0921
IC H11  N2   *CM1 H12      1.0921  108.06  119.04  108.98  1.0872
IC H11  N2   *CM1 H13      1.0921  108.06 -120.74  112.21  1.0983
IC C2   N2   CM2  H21      1.3783  119.27 -177.38  107.91  1.0903
IC H21  N2   *CM2 H22      1.0903  107.91  120.05  112.93  1.0983
IC H21  N2   *CM2 H23      1.0903  107.91 -116.75  111.13  1.0946
!2OM-ribose
IC -O3' P    O5'  C5'   1.6001  101.45  -39.25  119.00  1.4401
IC -O3' O5'  *P   O1P   1.6001  101.45 -115.82  109.74  1.4802
IC -O3' O5'  *P   O2P   1.6001  101.45  115.90  109.80  1.4801
IC  P   O5'  C5'  C4'   1.5996  119.00 -151.39  110.04  1.5160
IC O5'  C5'  C4'  C3'   1.4401  108.83 -179.85  116.10  1.5284
IC C5'  C4'  C3'  O3'   1.5160  116.10   76.70  115.12  1.4212
IC C4'  C3'  O3'  +P    1.5284  111.92  159.13  119.05  1.6001
IC C3'  O3'  +P   +O5'  1.4212  119.05  -98.86  101.45  1.5996
IC O4'  C3'  *C4' C5'   1.4572  104.06 -120.04  116.10  1.5160
IC C2'  C4'  *C3' O3'   1.5284  100.16 -124.08  115.12  1.4212
IC C4'  C3'  C2'  C1'   1.5284  100.16   39.58  102.04  1.5251
IC C3'  C2'  C1'  N9    1.5284  101.97  144.39  113.71  1.4896
IC O4'  C1'  N9   C4    1.5251  113.71  -97.2   125.59  1.3783
IC C3'  C1'  *C2' O2'   1.5312  102.03  117.61  107.13  1.4206
IC C1'  C2'  O2'  C2M   1.5393  107.13  180.00  107.00  1.4150
IC C2'  O2'  C2M  HM2   1.4206  107.00  180.00    0.0   0.0
IC HM2  O2'  *C2M HM3   0.0       0.0   120.00    0.0   0.0
IC HM2  O2'  *C2M HM1   0.0       0.0  -120.00    0.0   0.0
IC O4'  C2'  *C1' H1'   0.0       0.0  -115.0     0.0   0.0
IC C1'  C3'  *C2' H2''  0.0       0.0   115.0     0.0   0.0
IC C2'  C4'  *C3' H3'   0.0       0.0   115.0     0.0   0.0
IC C3'  O4'  *C4' H4'   0.0       0.0  -115.0     0.0   0.0
IC C4'  O5'  *C5' H5'   0.0       0.0  -115.0     0.0   0.0
IC C4'  O5'  *C5' H5''  0.0       0.0   115.0     0.0   0.0
DONO H1   N1
ACCE O6   C6
ACCE N3
ACCE N7
ACCE O1P  P
ACCE O2P  P
ACCE O2'
ACCE O3'
ACCE O4'
ACCE O5'

RESI N2G            0.00 ! N2,N2,7-trimethylguanosine
GROUP
ATOM N9     NG2R52 -0.09 !               O6     H71  H72
ATOM C8     CG2R53  0.41 !               ||       \ /
ATOM H8     HGR53   0.13 !               C6        C7-H73
ATOM N7     NG2R52 -0.39 !              /  \   (+)/
ATOM C5     CG2RC0  0.13 !          H1-N1   C5--N7\\
ATOM C6     CG2R63  0.58 !             |    ||     C8-H8
ATOM O6     OG2D4  -0.46 !   H21       C2   C4--N9/
ATOM N1     NG2R61 -0.31 !      \     / \\ /      \
ATOM H1     HGP1    0.23 !  H22-C20-N2   N3        \
ATOM C2     CG2R64  0.73 !      /   |               \
ATOM N3     NG2R62 -0.53 !   H23   C10               \
ATOM C4     CG2RC0  0.20 !        / | \               \
ATOM C7     CG334   0.10 !     H11 H12 H13             \
ATOM H71    HGA3    0.09 !                              \
ATOM H72    HGA3    0.09 !          O1P    H5' H4'  O4'  \
ATOM H73    HGA3    0.09 !           |      |    \ /   \  \
GROUP                    !          -P-O5'-C5'---C4'    C1'
ATOM N2     NG301  -0.30 !           |      |     \     / \
ATOM C10    CG331  -0.12 !          O2P    H5''   C3'--C2' H1'
```

```
ATOM H11    HGA3    0.09 !                        / \   / \
ATOM H12    HGA3    0.09 !                      O3' H3' O2' H2''
ATOM H13    HGA3    0.09 !                       |       |
ATOM C20    CG331  -0.12 !                              H2'
ATOM H21    HGA3    0.09
ATOM H22    HGA3    0.09
ATOM H23    HGA3    0.09
GROUP
ATOM P      P       1.50
ATOM O1P    ON3    -0.78
ATOM O2P    ON3    -0.78
ATOM O5'    ON2    -0.57
ATOM C5'    CN8B   -0.08
ATOM H5'    HN8     0.09
ATOM H5''   HN8     0.09
GROUP
ATOM C4'    CN7     0.16
ATOM H4'    HN7     0.09
ATOM O4'    ON6B   -0.50
ATOM C1'    CN7B    0.16
ATOM H1'    HN7     0.09
GROUP
ATOM C2'    CN7B    0.14
ATOM H2''   HN7     0.09
ATOM O2'    ON5    -0.66
ATOM H2'    HN5     0.43
GROUP
ATOM C3'    CN7     0.01
ATOM H3'    HN7     0.09
ATOM O3'    ON2    -0.57
BOND N9   C8   N9   C4   C8   N7   C8   H8
BOND N7   C5   N7   C7   C5   C6   C5   C4
BOND C6   O6   C6   N1   N1   C2   N1   H1
BOND C2   N2   C2   N3   N2   C20  N2   C10
BOND N3   C4   C20  H21  C20  H22  C20  H23
BOND C10  H11  C10  H12  C10  H13  C7   H71
BOND C7   H72  C7   H73
BOND P    O1P     P    O2P     P    O5'     O5'  C5'     C5'  H5''
BOND C5'  C4'     C4'  O4'     C4'  C3'     O4'  C1'
BOND C1'  N9      C1'  C2'     C2'  C3'     C3'  O3'     O3'  +P
BOND C2'  O2'     O2'  H2'
BOND C1'  H1'     C2'  H2''    C3'  H3'     C4'  H4'     C5'  H5'
IMPR C6     C5     N1     O6     C2     N1     N3     N2
IC C8   C4   *N9   C1'      1.3511  107.49  180.00  125.45  1.4695
IC C4   N9   C8    N7       1.3797  109.30    0.23  108.85  1.3409
IC N7   N9   *C8   H8       1.3409  108.85  179.71  125.72  1.0820
IC N9   C8   N7    C5       1.3511  108.85    1.24  108.04  1.3782
IC C5   C8   *N7   C7       1.4008  107.65 -179.88  126.50  1.4823
IC C4   N7   *C5   C6       1.3927  107.24 -179.95  132.99  1.4137
IC N7   C5   C6    N1       1.4008  132.99 -179.86  112.68  1.3815
IC N1   C5   *C6   O6       1.3815  112.68 -179.90  129.01  1.2287
IC C5   C6   N1    C2       1.4137  112.68    0.69  126.67  1.3825
IC C2   C6   *N1   H1       1.3825  126.67  179.85  116.27  1.0015
IC C6   N1   C2    N2       1.3815  126.67 -177.15  118.24  1.3725
IC N2   N1   *C2   N3       1.3725  118.24  175.08  118.91  1.3620
IC C8   N7   C7    H71      1.3369  126.50 -120.48  109.21  1.1137
IC H71  N7   *C7   H72      1.1137  109.21  120.45  111.17  1.1139
IC H71  N7   *C7   H73      1.1137  109.21 -119.03  109.22  1.1133
IC N1   C2   N2    C20      1.3825  118.24  -31.55  120.74  1.4627
IC C20  C2   *N2   C10      1.4627  120.74 -161.18  119.95  1.4637
IC C2   N2   C10   H11      1.3725  119.95 -169.26  111.15  1.1133
IC H11  N2   *C10  H12      1.1133  111.15  119.24  111.93  1.1140
IC H11  N2   *C10  H13      1.1133  111.15 -119.78  111.32  1.1141
```

```
IC C2   N2   C20   H21       1.3725  120.74 -161.60  110.96  1.1148
IC H21  N2   *C20  H22       1.1148  110.96  119.18  112.32  1.1134
IC H21  N2   *C20  H23       1.1148  110.96 -118.71  111.41  1.1142
!ribose
IC -O3' P    O5'   C5'    1.6001  101.45  -39.25  119.00  1.4401
IC -O3' O5'  *P    O1P    1.6001  101.45 -115.82  109.74  1.4802
IC -O3' O5'  *P    O2P    1.6001  101.45  115.90  109.80  1.4801
IC  P   O5'  C5'   C4'    1.5996  119.00 -151.39  110.04  1.5160
IC O5'  C5'  C4'   C3'    1.4401  108.83 -179.85  116.10  1.5284
IC C5'  C4'  C3'   O3'    1.5160  116.10   76.70  115.12  1.4212
IC C4'  C3'  O3'   +P     1.5284  111.92  159.13  119.05  1.6001
IC C3'  O3'  +P    +O5'   1.4212  119.05  -98.86  101.45  1.5996
IC O4'  C3'  *C4'  C5'    1.4572  104.06 -120.04  116.10  1.5160
IC C2'  C4'  *C3'  O3'    1.5284  100.16 -124.08  115.12  1.4212
IC C4'  C3'  C2'   C1'    1.5284  100.16   39.58  102.04  1.5251
IC C3'  C2'  C1'   N9     1.5284  101.97  144.39  113.71  1.4695
IC C4   N9   C1'   C2'    1.3797  125.45  124.52  112.36  1.5328
IC C1'  C3'  *C2'  O2'    1.5284  102.04 -114.67  110.81  1.4212
IC H2'  O2'  C2'   C3'    0.9600  114.97  148.63  111.92  1.5284
IC O4'  C2'  *C1'  H1'    0.0       0.0  -115.0     0.0   0.0
IC C1'  C3'  *C2'  H2''   0.0       0.0   115.0     0.0   0.0
IC C2'  C4'  *C3'  H3'    0.0       0.0   115.0     0.0   0.0
IC C3'  O4'  *C4'  H4'    0.0       0.0  -115.0     0.0   0.0
IC C4'  O5'  *C5'  H5'    0.0       0.0  -115.0     0.0   0.0
IC C4'  O5'  *C5'  H5''   0.0       0.0   115.0     0.0   0.0
DONO H2'  O2'
DONO H1   N1
ACCE O6   C6
ACCE N3
ACCE O1P  P
ACCE O2P  P
ACCE O2'
ACCE O3'
ACCE O4'
ACCE O5'

RESI DCG           -1.00 ! 7-cyano-7-deazaguanosine
GROUP
ATOM N9     NG2R51  0.18 !          O6         N10
ATOM C8     CG2R51 -0.09 !          ||        ///
ATOM H8     HGR52   0.19 !          C6       C10
ATOM C7     CG2R51 -0.10 !         /  \     /
ATOM C5     CG2RC0 -0.11 !     H1-N1   C5--C7\\
ATOM C6     CG2R63  0.57 !        |    ||     C8-H8
ATOM O6     OG2D4  -0.51 !        C2   C4--N9/
ATOM N1     NG2R61 -0.35 !       / \\ /      \
ATOM H1     HGP1    0.26 !  H21-N2   N3        \
ATOM C2     CG2R64  0.77 !      |               \
ATOM N2     NG2S3  -0.60 !     H22               \
ATOM H21    HGP4    0.29 !                        \
ATOM H22    HGP4    0.29 !    O1P    H5' H4'  O4'  \
ATOM N3     NG2R62 -0.73 !     |      |    \ /   \  \
ATOM C4     CG2RC0  0.14 !    -P-O5'-C5'---C4'    C1'
ATOM C10    CG1N1   0.24 !     |      |     \     / \
ATOM N10    NG1T1  -0.44 !    O2P    H5''   C3'--C2' H1'
GROUP                    !                 / \   / \
ATOM P      P       1.50 !               O3' H3' O2' H2''
ATOM O1P    ON3    -0.78 !                |       |
ATOM O2P    ON3    -0.78 !                        H2'
ATOM O5'    ON2    -0.57
ATOM C5'    CN8B   -0.08
ATOM H5'    HN8     0.09
ATOM H5''   HN8     0.09
```

```
GROUP
ATOM C4'    CN7      0.16
ATOM H4'    HN7      0.09
ATOM O4'    ON6B   -0.50
ATOM C1'    CN7B     0.16
ATOM H1'    HN7      0.09
GROUP
ATOM C2'    CN7B     0.14
ATOM H2''   HN7      0.09
ATOM O2'    ON5    -0.66
ATOM H2'    HN5      0.43
GROUP
ATOM C3'    CN7      0.01
ATOM H3'    HN7      0.09
ATOM O3'    ON2    -0.57
BOND N9   C8   N9   C4   C8   C7   C8   H8
BOND C7   C5   C7   C10  C5   C6   C5   C4
BOND C6   O6   C6   N1   N1   C2   N1   H1
BOND C2   N2   C2   N3   N2   H21  N2   H22
BOND N3   C4   C10  N10
BOND P    O1P     P    O2P     P    O5'     O5'  C5'     C5'  H5''
BOND C5'  C4'     C4'  O4'     C4'  C3'     O4'  C1'
BOND C1'  N9      C1'  C2'     C2'  C3'     C3'  O3'     O3'  +P
BOND C2'  O2'     O2'  H2'
BOND C1'  H1'     C2'  H2''    C3'  H3'     C4'  H4'     C5'  H5'
IMPR C6     C5     N1     O6     C2     N1     N3     N2     N2     H22    H21    C2
IC C8   C4   *N9   C1'      1.3791  105.54 -179.95  126.56  1.4896
IC C4   N9   C8    C7       1.3499  111.09   -0.02  107.80  1.3687
IC C7   N9   *C8   H8       1.3687  107.80 -179.93  124.15  1.0835
IC N9   C8   C7    C5       1.3866  107.80   -0.01  106.78  1.4375
IC C5   C8   *C7   C10      1.4375  106.78 -179.99  126.09  1.4271
IC C4   C7   *C5   C6       1.4016  107.60  179.89  133.01  1.4042
IC C7   C5   C6    N1       1.4375  133.01 -179.97  112.73  1.3808
IC N1   C5   *C6   O6       1.3808  112.73 -179.90  129.18  1.2288
IC C5   C6   N1    C2       1.4042  112.73    0.08  125.26  1.3756
IC C2   C6   *N1   H1       1.3756  125.26  179.93  116.15  0.9976
IC C6   N1   C2    N2       1.3808  125.26  179.97  116.78  1.3281
IC N2   N1   *C2   N3       1.3281  116.78  179.99  121.67  1.3434
IC N1   C2   N2    H21      1.3756  116.78   -0.00  123.36  0.9926
IC H21  C2   *N2   H22      0.9926  123.36 -179.99  116.11  0.9948
IC C8   C7   C10   N10      1.3687  126.09   -0.16  178.56  1.1807
!ribose
IC -O3' P    O5'   C5'    1.6001  101.45  -39.25  119.00  1.4401
IC -O3' O5'  *P    O1P    1.6001  101.45 -115.82  109.74  1.4802
IC -O3' O5'  *P    O2P    1.6001  101.45  115.90  109.80  1.4801
IC  P   O5'  C5'   C4'    1.5996  119.00 -151.39  110.04  1.5160
IC O5'  C5'  C4'   C3'    1.4401  108.83 -179.85  116.10  1.5284
IC C5'  C4'  C3'   O3'    1.5160  116.10   76.70  115.12  1.4212
IC C4'  C3'  O3'   +P     1.5284  111.92  159.13  119.05  1.6001
IC C3'  O3'  +P    +O5'   1.4212  119.05  -98.86  101.45  1.5996
IC O4'  C3'  *C4'  C5'    1.4572  104.06 -120.04  116.10  1.5160
IC C2'  C4'  *C3'  O3'    1.5284  100.16 -124.08  115.12  1.4212
IC C4'  C3'  C2'   C1'    1.5284  100.16   39.58  102.04  1.5251
IC C3'  C2'  C1'   N9     1.5284  101.97  144.39  113.71  1.4896
IC O4'  C1'  N9    C4     1.5251  113.71  -97.2   125.59  1.3783
IC C1'  C3'  *C2'  O2'    1.5284  102.04 -114.67  110.81  1.4212
IC H2'  O2'  C2'   C3'    0.9600  114.97  148.63  111.92  1.5284
IC O4'  C2'  *C1'  H1'    0.0       0.0  -115.0     0.0   0.0
IC C1'  C3'  *C2'  H2''   0.0       0.0   115.0     0.0   0.0
IC C2'  C4'  *C3'  H3'    0.0       0.0   115.0     0.0   0.0
IC C3'  O4'  *C4'  H4'    0.0       0.0  -115.0     0.0   0.0
IC C4'  O5'  *C5'  H5'    0.0       0.0  -115.0     0.0   0.0
IC C4'  O5'  *C5'  H5''   0.0       0.0   115.0     0.0   0.0
```

```
DONO H2'  O2'
DONO H21   N2
DONO H22   N2
DONO H1   N1
ACCE O6   C6
ACCE N3
ACCE N10  C10
ACCE O1P  P
ACCE O2P  P
ACCE O2'
ACCE O3'
ACCE O4'
ACCE O5'

RESI RCG            0.00 ! archaeosine
GROUP
ATOM N9     NG2R51  0.04 !                H112      H122
ATOM C4     CG2RC0  0.32 !                   \  (+) /
ATOM C5     CG2RC0 -0.06 !           O6  H111-N11  N12-H121
ATOM C6     CG2R63  0.42 !           ||        \  //
ATOM O6     OG2D4  -0.52 !           C6         C10
ATOM N1     NG2R61 -0.38 !          /  \       /
ATOM H1     HGP1    0.30 !      H1-N1   C5--C7\\
ATOM C2     CG2R64  0.65 !         |    ||     C8-H8
ATOM N2     NG2S3  -0.60 !         C2   C4--N9/
ATOM H21    HGP4    0.30 !        / \\ /      \
ATOM H22    HGP4    0.30 !  H21-N2   N3        \
ATOM N3     NG2R62 -0.77 !      |               \
GROUP                    !     H22               \
ATOM C7     CG2R51  0.06 !                        \
ATOM C8     CG2R51 -0.21 !    O1P    H5' H4'  O4'  \
ATOM H8     HGR52   0.24 !      |     |    \ /   \  \
ATOM C10    CG2N2   0.61 !    -P-O5'-C5'---C4'    C1'
ATOM N11    NG2P1  -0.43 !      |     |     \     / \
ATOM H111   HGP2    0.29 !    O2P    H5''   C3'--C2' H1'
ATOM H112   HGP2    0.29 !                 / \   / \
ATOM N12    NG2P1  -0.43 !               O3' H3' O2' H2''
ATOM H121   HGP2    0.29 !                |       |
ATOM H122   HGP2    0.29 !                        H2'
GROUP
ATOM P      P       1.50
ATOM O1P    ON3    -0.78
ATOM O2P    ON3    -0.78
ATOM O5'    ON2    -0.57
ATOM C5'    CN8B   -0.08
ATOM H5'    HN8     0.09
ATOM H5''   HN8     0.09
GROUP
ATOM C4'    CN7     0.16
ATOM H4'    HN7     0.09
ATOM O4'    ON6B   -0.50
ATOM C1'    CN7B    0.16
ATOM H1'    HN7     0.09
GROUP
ATOM C2'    CN7B    0.14
ATOM H2''   HN7     0.09
ATOM O2'    ON5    -0.66
ATOM H2'    HN5     0.43
GROUP
ATOM C3'    CN7     0.01
ATOM H3'    HN7     0.09
ATOM O3'    ON2    -0.57
BOND N9   C8   N9   C4   C8   C7   C8   H8
```

```
BOND C7   C5   C7   C10  C5   C6   C5   C4
BOND C6   O6   C6   N1   N1   C2   N1   H1
BOND C2   N2   C2   N3   N2   H21  N2   H22
BOND N3   C4   C10  N11  C10  N12  N11  H111
BOND N11  H112 N12  H121 N12  H122
BOND P    O1P     P    O2P     P    O5'     O5'  C5'     C5'  H5''
BOND C5'  C4'     C4'  O4'     C4'  C3'     O4'  C1'
BOND C1'  N9      C1'  C2'     C2'  C3'     C3'  O3'     O3'  +P
BOND C2'  O2'     O2'  H2'
BOND C1'  H1'     C2'  H2''    C3'  H3'     C4'  H4'     C5'  H5'
IMPR C6     C5     N1     O6     C2     N1     N3     N2
IMPR N2     H22    H21    C2     C10    N12    N11    C7
IC C8   C4   *N9   C1'       1.3791  105.54 -179.95  126.56  1.4896
IC C4   N9   C8    C7        1.3445  111.30    0.16  108.31  1.3833
IC C7   N9   *C8   H8        1.3833  108.31  179.88  122.42  1.0831
IC N9   C8   C7    C5        1.3863  108.31   -0.21  104.90  1.4531
IC C5   C8   *C7   C10       1.4531  104.90 -179.91  124.57  1.4787
IC C4   C7   *C5   C6        1.3891  108.72  179.97  133.50  1.3926
IC C7   C5   C6    N1        1.4531  133.50 -179.94  114.66  1.3675
IC N1   C5   *C6   O6        1.3675  114.66 -179.95  126.71  1.2264
IC C5   C6   N1    C2        1.3926  114.66    0.02  124.28  1.3572
IC C2   C6   *N1   H1        1.3572  124.28  179.93  117.11  0.9983
IC C6   N1   C2    N2        1.3675  124.28  179.97  116.62  1.3079
IC N2   N1   *C2   N3        1.3079  116.62  179.96  121.56  1.3307
IC N1   C2   N2    H21       1.3572  116.62   -0.03  123.58  0.9926
IC H21  C2   *N2   H22       0.9926  123.58 -179.97  116.43  0.9952
IC C8   C7   C10   N11       1.3833  124.57  178.62  120.94  1.3213
IC N11  C7   *C10  N12       1.3213  120.94  179.97  122.17  1.3255
IC C7   C10  N11   H111      1.4787  120.94  179.91  120.30  0.9964
IC H111 C10  *N11  H112      0.9964  120.30 -179.23  120.50  1.0097
IC C7   C10  N12   H121      1.4787  122.17 -179.97  119.27  0.9966
IC H121 C10  *N12  H122      0.9966  119.27  179.89  120.89  0.9977
!ribose
IC -O3' P    O5'   C5'    1.6001  101.45  -39.25  119.00  1.4401
IC -O3' O5'  *P    O1P    1.6001  101.45 -115.82  109.74  1.4802
IC -O3' O5'  *P    O2P    1.6001  101.45  115.90  109.80  1.4801
IC  P   O5'  C5'   C4'    1.5996  119.00 -151.39  110.04  1.5160
IC O5'  C5'  C4'   C3'    1.4401  108.83 -179.85  116.10  1.5284
IC C5'  C4'  C3'   O3'    1.5160  116.10   76.70  115.12  1.4212
IC C4'  C3'  O3'   +P     1.5284  111.92  159.13  119.05  1.6001
IC C3'  O3'  +P    +O5'   1.4212  119.05  -98.86  101.45  1.5996
IC O4'  C3'  *C4'  C5'    1.4572  104.06 -120.04  116.10  1.5160
IC C2'  C4'  *C3'  O3'    1.5284  100.16 -124.08  115.12  1.4212
IC C4'  C3'  C2'   C1'    1.5284  100.16   39.58  102.04  1.5251
IC C3'  C2'  C1'   N9     1.5284  101.97  144.39  113.71  1.4896
IC O4'  C1'  N9    C4     1.5251  113.71  -97.2   125.59  1.3783
IC C1'  C3'  *C2'  O2'    1.5284  102.04 -114.67  110.81  1.4212
IC H2'  O2'  C2'   C3'    0.9600  114.97  148.63  111.92  1.5284
IC O4'  C2'  *C1'  H1'    0.0       0.0  -115.0     0.0   0.0
IC C1'  C3'  *C2'  H2''   0.0       0.0   115.0     0.0   0.0
IC C2'  C4'  *C3'  H3'    0.0       0.0   115.0     0.0   0.0
IC C3'  O4'  *C4'  H4'    0.0       0.0  -115.0     0.0   0.0
IC C4'  O5'  *C5'  H5'    0.0       0.0  -115.0     0.0   0.0
IC C4'  O5'  *C5'  H5''   0.0       0.0   115.0     0.0   0.0
DONO H2'  O2'
DONO H21   N2
DONO H22   N2
DONO H111  N11
DONO H112  N11
DONO H121  N12
DONO H122  N12
DONO H1   N1
ACCE O6   C6
```

```
ACCE N3
ACCE O1P  P
ACCE O2P  P
ACCE O2'
ACCE O3'
ACCE O4'
ACCE O5'

RESI DAG            0.00 ! 7-aminomethyl-7-deazaguanosine, PQ1
GROUP
ATOM N9     NG2R51  0.04 !           O6    H101 H102  H111
ATOM C8     CG2R51  0.06 !           ||      \ /     /   (+)
ATOM H8     HGR52   0.08 !           C6       C10--N11-H112
ATOM C7     CG2R51 -0.14 !          /  \     /       \
ATOM C5     CG2RC0 -0.06 !      H1-N1   C5--C7\\      H113
ATOM C6     CG2R63  0.42 !         |     ||     C8-H8
ATOM O6     OG2D4  -0.52 !         C2   C4--N9/
ATOM N1     NG2R61 -0.38 !        / \\ /      \
ATOM H1     HGP1    0.30 !  H21-N2   N3        \
ATOM C2     CG2R64  0.65 !      |               \
ATOM N2     NG2S3  -0.60 !     H22               \
ATOM H21    HGP4    0.30 !                        \
ATOM H22    HGP4    0.30 !    O1P    H5' H4'  O4'  \
ATOM N3     NG2R62 -0.77 !     |      |    \ /   \  \
ATOM C4     CG2RC0  0.32 !    -P-O5'-C5'---C4'    C1'
GROUP                    !     |      |     \     / \
ATOM C10    CG324   0.21 !    O2P    H5''   C3'--C2' H1'
ATOM H101   HGA2    0.05 !                 / \   / \
ATOM H102   HGA2    0.05 !               O3' H3' O2' H2''
ATOM N11    NG3P3  -0.30 !                |       |
ATOM H111   HGP2    0.33 !                       H2'
ATOM H112   HGP2    0.33
ATOM H113   HGP2    0.33 !  !!!! PATCH 7GNA for the neutral amino form
GROUP
ATOM P      P       1.50
ATOM O1P    ON3    -0.78
ATOM O2P    ON3    -0.78
ATOM O5'    ON2    -0.57
ATOM C5'    CN8B   -0.08
ATOM H5'    HN8     0.09
ATOM H5''   HN8     0.09
GROUP
ATOM C4'    CN7     0.16
ATOM H4'    HN7     0.09
ATOM O4'    ON6B   -0.50
ATOM C1'    CN7B    0.16
ATOM H1'    HN7     0.09
GROUP
ATOM C2'    CN7B    0.14
ATOM H2''   HN7     0.09
ATOM O2'    ON5    -0.66
ATOM H2'    HN5     0.43
GROUP
ATOM C3'    CN7     0.01
ATOM H3'    HN7     0.09
ATOM O3'    ON2    -0.57
BOND N9   C8   N9   C4   C8   C7   C8   H8
BOND C7   C5   C7   C10  C5   C6   C5   C4
BOND C6   O6   C6   N1   N1   C2   N1   H1
BOND C2   N2   C2   N3   N2   H21  N2   H22
BOND N3   C4   C10  N11  C10  H101 C10  H102
BOND N11  H111 N11  H112 N11  H113
BOND P    O1P     P    O2P     P    O5'     O5'  C5'     C5'  H5''
```

```
BOND C5'  C4'     C4'  O4'     C4'  C3'     O4'  C1'
BOND C1'  N9      C1'  C2'     C2'  C3'     C3'  O3'     O3'  +P
BOND C2'  O2'     O2'  H2'
BOND C1'  H1'     C2'  H2''    C3'  H3'     C4'  H4'     C5'  H5'
IMPR C6     C5     N1     O6     C2     N1     N3     N2     N2     H22    H21    C2
IC C8   C4   *N9   C1'       1.3791  105.54 -179.95  126.56  1.4896
IC C4   N9   C8    C7        1.3456  111.66   -0.00  107.58  1.3722
IC C7   N9   *C8   H8        1.3722  107.58 -178.35  122.69  1.0842
IC N9   C8   C7    C5        1.3854  107.58    0.76  106.19  1.4427
IC C5   C8   *C7   C10       1.4427  106.19 -173.79  127.04  1.4961
IC C4   C7   *C5   C6        1.3938  108.15 -179.50  133.12  1.3990
IC C7   C5   C6    N1        1.4427  133.12  178.66  113.76  1.3820
IC N1   C5   *C6   O6        1.3820  113.76  178.80  126.34  1.2334
IC C5   C6   N1    C2        1.3990  113.76   -0.06  124.39  1.3787
IC C2   C6   *N1   H1        1.3787  124.39 -178.51  117.72  0.9994
IC C6   N1   C2    N2        1.3820  124.39 -179.53  117.30  1.3221
IC N2   N1   *C2   N3        1.3221  117.30 -179.13  121.37  1.3398
IC N1   C2   N2    H21       1.3787  117.30    0.85  124.05  0.9933
IC H21  C2   *N2   H22       0.9933  124.05  179.48  116.08  0.9959
IC C8   C7   C10   N11       1.3722  127.04 -138.65  110.52  1.4916
IC N11  C7   *C10  H101      1.4916  110.52  120.00  109.50  1.1000
IC N11  C7   *C10  H102      1.4916  110.52 -120.00  109.50  1.1000
IC C7   C10  N11   H111      1.4961  110.52 -175.58  111.12  1.0382
IC H111 C10  *N11  H112      1.0382  111.12  118.98  107.09  1.0545
IC H111 C10  *N11  H113      1.0382  111.12 -122.82  110.43  1.0377
!ribose
IC -O3' P    O5'   C5'    1.6001  101.45  -39.25  119.00  1.4401
IC -O3' O5'  *P    O1P    1.6001  101.45 -115.82  109.74  1.4802
IC -O3' O5'  *P    O2P    1.6001  101.45  115.90  109.80  1.4801
IC  P   O5'  C5'   C4'    1.5996  119.00 -151.39  110.04  1.5160
IC O5'  C5'  C4'   C3'    1.4401  108.83 -179.85  116.10  1.5284
IC C5'  C4'  C3'   O3'    1.5160  116.10   76.70  115.12  1.4212
IC C4'  C3'  O3'   +P     1.5284  111.92  159.13  119.05  1.6001
IC C3'  O3'  +P    +O5'   1.4212  119.05  -98.86  101.45  1.5996
IC O4'  C3'  *C4'  C5'    1.4572  104.06 -120.04  116.10  1.5160
IC C2'  C4'  *C3'  O3'    1.5284  100.16 -124.08  115.12  1.4212
IC C4'  C3'  C2'   C1'    1.5284  100.16   39.58  102.04  1.5251
IC C3'  C2'  C1'   N9     1.5284  101.97  144.39  113.71  1.4896
IC O4'  C1'  N9    C4     1.5251  113.71  -97.2   125.59  1.3783
IC C1'  C3'  *C2'  O2'    1.5284  102.04 -114.67  110.81  1.4212
IC H2'  O2'  C2'   C3'    0.9600  114.97  148.63  111.92  1.5284
IC O4'  C2'  *C1'  H1'    0.0       0.0  -115.0     0.0   0.0
IC C1'  C3'  *C2'  H2''   0.0       0.0   115.0     0.0   0.0
IC C2'  C4'  *C3'  H3'    0.0       0.0   115.0     0.0   0.0
IC C3'  O4'  *C4'  H4'    0.0       0.0  -115.0     0.0   0.0
IC C4'  O5'  *C5'  H5'    0.0       0.0  -115.0     0.0   0.0
IC C4'  O5'  *C5'  H5''   0.0       0.0   115.0     0.0   0.0
DONO H2'  O2'
DONO H21   N2
DONO H22   N2
DONO H1   N1
DONO H111  N11
DONO H112  N11
DONO H113  N11
ACCE O6   C6
ACCE N3
ACCE O1P  P
ACCE O2P  P
ACCE O2'
ACCE O3'
ACCE O4'
ACCE O5'
```

```
RESI QUG             0.00 ! queuosine, QUO
GROUP
ATOM N9      NG2R51  0.04 !                 H13  O13-H13O
ATOM C8      CG2R51  0.06 !                     \  /
ATOM H8      HGR52   0.08 !   H101 H111 H12   C13(R)  H14
ATOM C7      CG2R51 -0.14 !      |   |   \   /    \  /
ATOM C5      CG2RC0 -0.06 ! *---C10--N11--C12(S)   C14(S)
ATOM C6      CG2R63  0.42 !      |   |(+)   \      / \
ATOM O6      OG2D4  -0.52 !   H102 H112      C16==C15 O14-H14O
ATOM N1      NG2R61 -0.38 !                 /        \
ATOM H1      HGP1    0.30 !               H16      H15
ATOM C2      CG2R64  0.65
ATOM N2      NG2S3  -0.60 !           O6
ATOM H21     HGP4    0.30 !           ||         *
ATOM H22     HGP4    0.30 !           C6        /
ATOM N3      NG2R62 -0.77 !          /  \      /
ATOM C4      CG2RC0  0.32 !      H1-N1   C5--C7\\
GROUP                     !         |    ||     C8-H8
ATOM C10     CG324   0.18 !         C2   C4--N9/
ATOM H101    HGA2    0.09 !        / \\ /      \
ATOM H102    HGA2    0.09 !  H21-N2   N3        \
ATOM N11     NG3P2  -0.35 !      |               \
ATOM H111    HGP2    0.28 !     H22               \
ATOM H112    HGP2    0.28 !                        \
ATOM C12     CG3C53  0.34 !    O1P    H5' H4'  O4'  \
ATOM H12     HGA1    0.09 !     |      |    \ /   \  \
GROUP                     !    -P-O5'-C5'---C4'    C1'
ATOM C15     CG2R51 -0.20 !     |      |     \     / \
ATOM H15     HGR51   0.26 !    O2P    H5''   C3'--C2' H1'
ATOM C16     CG2R51 -0.24 !                 / \   / \
ATOM H16     HGR51   0.18 !               O3' H3' O2' H2''
GROUP                     !                |       |
ATOM C13     CG3C51  0.14 !                       H2'
ATOM H13     HGA1    0.09
ATOM O13     OG311  -0.65 !  !!!! PATCH 7GNM for the neutral amino form
ATOM H13O    HGP1    0.42
GROUP
ATOM C14     CG3C51  0.14
ATOM H14     HGA1    0.09
ATOM O14     OG311  -0.65
ATOM H14O    HGP1    0.42
GROUP
ATOM P       P       1.50
ATOM O1P     ON3    -0.78
ATOM O2P     ON3    -0.78
ATOM O5'     ON2    -0.57
ATOM C5'     CN8B   -0.08
ATOM H5'     HN8     0.09
ATOM H5''    HN8     0.09
GROUP
ATOM C4'     CN7     0.16
ATOM H4'     HN7     0.09
ATOM O4'     ON6B   -0.50
ATOM C1'     CN7B    0.16
ATOM H1'     HN7     0.09
GROUP
ATOM C2'     CN7B    0.14
ATOM H2''    HN7     0.09
ATOM O2'     ON5    -0.66
ATOM H2'     HN5     0.43
GROUP
ATOM C3'     CN7     0.01
ATOM H3'     HN7     0.09
```

```
ATOM O3'    ON2    -0.57
BOND N9   C8   N9   C4   C8   H8   C8   C7
BOND C7   C5   C7   C10  C5   C6   C5   C4
BOND C6   O6   C6   N1   N1   H1   N1   C2
BOND C2   N2   C2   N3   N2   H21  N2   H22
BOND N3   C4   C10  H101 C10  H102 C10  N11
BOND N11  H111 N11  H112 N11  C12  C12  H12
BOND C12  C13  C12  C16  C13  O13  C13  H13
BOND C13  C14  O13  H13O  C14  O14  C14  H14
BOND C14  C15  O14  H14O  C15  H15  C15  C16
BOND C16  H16
BOND P    O1P     P    O2P     P    O5'     O5'  C5'     C5'  H5''
BOND C5'  C4'     C4'  O4'     C4'  C3'     O4'  C1'
BOND C1'  N9      C1'  C2'     C2'  C3'     C3'  O3'     O3'  +P
BOND C2'  O2'     O2'  H2'
BOND C1'  H1'     C2'  H2''    C3'  H3'     C4'  H4'     C5'  H5'
IMPR C6     C5     N1     O6     C2     N1     N3     N2     N2     H22    H21    C2
IC C8   C4   *N9   C1'      1.3791  105.54 -179.95  126.56  1.4896
IC C4   N9   C8    C7       1.3818  110.12    0.38  108.60  1.3701
IC C7   N9   *C8   H8       1.3701  108.60  180.00  124.00  1.0830
IC N9   C8   C7    C10      1.3880  108.60  174.46  126.75  1.4904
IC C10  C8   *C7   C5       1.4904  126.75 -175.57  106.66  1.4405
IC C4   C7   *C5   C6       1.3991  108.20  179.62  132.66  1.4030
IC C7   C5   C6    N1       1.4405  132.66 -178.93  113.58  1.3799
IC N1   C5   *C6   O6       1.3799  113.58 -179.16  127.12  1.2314
IC C5   C6   N1    C2       1.4030  113.58   -0.01  124.94  1.3727
IC C2   C6   *N1   H1       1.3727  124.94  180.00  115.40  1.0100
IC C6   N1   C2    N2       1.3799  124.94  179.67  117.41  1.3224
IC N2   N1   *C2   N3       1.3224  117.41  179.36  121.37  1.3364
IC N1   C2   N2    H21      1.3727  117.41  180.00  121.50  1.0000
IC H21  C2   *N2   H22      1.0000  121.50  180.00  121.50  1.0000
IC C8   C7   C10   N11      1.3701  126.75   13.24  110.50  1.5058
IC N11  C7   *C10  H101     1.5058  110.50  120.00  109.50  1.1000
IC H101 C7   *C10  H102     1.1000  109.50 -120.00  109.50  1.1000
IC C7   C10  N11   C12      1.4904  110.50 -146.66  120.39  1.4978
IC C12  C10  *N11  H111     1.4978  120.39  120.00  110.80  1.0060
IC H111 C10  *N11  H112     1.0060  110.80 -120.00  110.80  1.0060
IC C10  N11  C12   C13      1.5058  120.39  -50.31  105.54  1.5274
IC C13  N11  *C12  C16      1.5274  105.54 -111.95  113.63  1.5041
IC C13  N11  *C12  H12      1.5274  105.54 -120.00  107.50  1.0800
IC N11  C12  C16   C15      1.4978  113.63  135.20  109.51  1.3789
IC C15  C12  *C16  H16      1.3789  109.51  180.00  124.60  1.0800
IC C12  C16  C15   C14      1.5041  109.51    0.87  109.87  1.5183
IC C14  C16  *C15  H15      1.5183  109.87  180.00  126.40  1.0800
IC C14  C12  *C13  O13      1.5419  102.73 -126.51  115.78  1.4400
IC O13  C12  *C13  H13      1.4400  115.78 -120.00  110.10  1.1000
IC C12  C13  O13   H13O     1.5274  115.78  180.00  109.00  0.9600
IC C13  C15  *C14  O14      1.5419  101.54  120.86  109.59  1.4250
IC C13  C15  *C14  H14      1.5419  101.54 -120.00  110.10  1.1000
IC C15  C14  O14   H14O     1.5183  109.59  180.00  109.00  0.9600
!ribose
IC -O3' P    O5'   C5'    1.6001  101.45  -39.25  119.00  1.4401
IC -O3' O5'  *P    O1P    1.6001  101.45 -115.82  109.74  1.4802
IC -O3' O5'  *P    O2P    1.6001  101.45  115.90  109.80  1.4801
IC  P   O5'  C5'   C4'    1.5996  119.00 -151.39  110.04  1.5160
IC O5'  C5'  C4'   C3'    1.4401  108.83 -179.85  116.10  1.5284
IC C5'  C4'  C3'   O3'    1.5160  116.10   76.70  115.12  1.4212
IC C4'  C3'  O3'   +P     1.5284  111.92  159.13  119.05  1.6001
IC C3'  O3'  +P    +O5'   1.4212  119.05  -98.86  101.45  1.5996
IC O4'  C3'  *C4'  C5'    1.4572  104.06 -120.04  116.10  1.5160
IC C2'  C4'  *C3'  O3'    1.5284  100.16 -124.08  115.12  1.4212
IC C4'  C3'  C2'   C1'    1.5284  100.16   39.58  102.04  1.5251
IC C3'  C2'  C1'   N9     1.5284  101.97  144.39  113.71  1.4896
```

```
IC O4'  C1'  N9    C4      1.5251  113.71  -97.2   125.59  1.3783
IC C1'  C3'  *C2'  O2'     1.5284  102.04 -114.67  110.81  1.4212
IC H2'  O2'  C2'   C3'     0.9600  114.97  148.63  111.92  1.5284
IC O4'  C2'  *C1'  H1'     0.0       0.0  -115.0     0.0   0.0
IC C1'  C3'  *C2'  H2''    0.0       0.0   115.0     0.0   0.0
IC C2'  C4'  *C3'  H3'     0.0       0.0   115.0     0.0   0.0
IC C3'  O4'  *C4'  H4'     0.0       0.0  -115.0     0.0   0.0
IC C4'  O5'  *C5'  H5'     0.0       0.0  -115.0     0.0   0.0
IC C4'  O5'  *C5'  H5''    0.0       0.0   115.0     0.0   0.0
DONO H2'  O2'
DONO H21   N2
DONO H22   N2
DONO H1   N1
DONO H111  N11
DONO H112  N11
DONO H13O  O13
DONO H14O  O14
ACCE O6   C6
ACCE N3
ACCE O13
ACCE O14
ACCE O1P  P
ACCE O2P  P
ACCE O2'
ACCE O3'
ACCE O4'
ACCE O5'

RESI EQG             0.00 ! epoxyqueuosine
GROUP
ATOM N9     NG2R51  0.04 !                H13  O13-H13O
ATOM C8     CG2R51  0.06 !                  \  /(R)
ATOM H8     HGR52   0.08 !   H101 H111 H12   C13    H14
ATOM C7     CG2R51 -0.14 !      |   |   \   /    \  /
ATOM C5     CG2RC0 -0.06 ! *---C10--N11--C12(R)   C14(R)
ATOM C6     CG2R63  0.42 !      |   |(+)   \      /  \
ATOM O6     OG2D4  -0.52 !   H102 H112  (S)C16--C15   O14-H14O
ATOM N1     NG2R61 -0.38 !                 / \ / \ (R)
ATOM H1     HGP1    0.30 !              H16  O15  H15
ATOM C2     CG2R64  0.65
ATOM N2     NG2S3  -0.60 !          O6
ATOM H21    HGP4    0.30 !          ||         *
ATOM H22    HGP4    0.30 !          C6        /
ATOM N3     NG2R62 -0.77 !         /  \      /
ATOM C4     CG2RC0  0.32 !     H1-N1   C5--C7\\
GROUP                    !        |    ||     C8-H8
ATOM C10    CG324   0.17 !        C2   C4--N9/
ATOM H101   HGA2    0.09 !       / \\ /      \
ATOM H102   HGA2    0.09 ! H21-N2   N3        \
ATOM N11    NG3P2  -0.36 !     |               \
ATOM H111   HGP2    0.29 !    H22               \
ATOM H112   HGP2    0.29 !                       \
ATOM C12    CG3C53  0.34 !   O1P    H5' H4'  O4'  \
ATOM H12    HGA1    0.09 !    |      |    \ /   \  \
GROUP                    !   -P-O5'-C5'---C4'    C1'
ATOM C15    CG3RC1  0.15 !    |      |     \     / \
ATOM H15    HGA1    0.09 !   O2P    H5''   C3'--C2' H1'
ATOM O15    OG3C31 -0.25 !                / \   / \
ATOM C16    CG3RC1 -0.08 !              O3' H3' O2' H2''
ATOM H16    HGA1    0.09 !               |       |
GROUP                    !                      H2'
ATOM C13    CG3C51  0.14
ATOM H13    HGA1    0.09 !  !!!! PATCH 7GNM for the neutral amino form
```

```
ATOM O13    OG311  -0.65
ATOM H13O   HGP1    0.42
GROUP
ATOM C14    CG3C51  0.14
ATOM H14    HGA1    0.09
ATOM O14    OG311  -0.65
ATOM H14O   HGP1    0.42
GROUP
ATOM P      P       1.50
ATOM O1P    ON3    -0.78
ATOM O2P    ON3    -0.78
ATOM O5'    ON2    -0.57
ATOM C5'    CN8B   -0.08
ATOM H5'    HN8     0.09
ATOM H5''   HN8     0.09
GROUP
ATOM C4'    CN7     0.16
ATOM H4'    HN7     0.09
ATOM O4'    ON6B   -0.50
ATOM C1'    CN7B    0.16
ATOM H1'    HN7     0.09
GROUP
ATOM C2'    CN7B    0.14
ATOM H2''   HN7     0.09
ATOM O2'    ON5    -0.66
ATOM H2'    HN5     0.43
GROUP
ATOM C3'    CN7     0.01
ATOM H3'    HN7     0.09
ATOM O3'    ON2    -0.57
BOND N9   C8   N9   C4   C8   H8   C8   C7
BOND C7   C5   C7   C10  C5   C6   C5   C4
BOND C6   O6   C6   N1   N1   H1   N1   C2
BOND C2   N2   C2   N3   N2   H21  N2   H22
BOND N3   C4   C10  H101 C10  H102 C10  N11
BOND N11  H111 N11  H112 N11  C12  C12  H12
BOND C12  C13  C12  C16  C13  O13  C13  H13
BOND C13  C14  O13  H13O C14  O14  C14  H14
BOND C14  C15  O14  H14O O15  C15  O15  C16
BOND C15  H15  C15  C16  C16  H16
BOND P    O1P      P    O2P      P    O5'      O5'  C5'      C5'  H5''
BOND C5'  C4'      C4'  O4'      C4'  C3'      O4'  C1'
BOND C1'  N9       C1'  C2'      C2'  C3'      C3'  O3'      O3'  +P
BOND C2'  O2'      O2'  H2'
BOND C1'  H1'      C2'  H2''     C3'  H3'      C4'  H4'      C5'  H5'
IMPR C6     C5     N1     O6     C2     N1     N3     N2     N2     H22    H21    C2
IC C8   C4   *N9   C1'      1.3791  105.54 -179.95  126.56  1.4896
IC C4   N9   C8    C7       1.3806  110.20   -0.44  108.62  1.3700
IC C7   N9   *C8   H8       1.3700  108.62  180.00  124.00  1.0830
IC N9   C8   C7    C10      1.3876  108.62 -173.38  126.54  1.4895
IC C10  C8   *C7   C5       1.4895  126.54  174.60  106.59  1.4424
IC C4   C7   *C5   C6       1.3994  108.17 -179.55  132.72  1.4024
IC C7   C5   C6    N1       1.4424  132.72  178.64  113.70  1.3796
IC N1   C5   *C6   O6       1.3796  113.70  179.23  127.07  1.2334
IC C5   C6   N1    C2       1.4024  113.70    0.11  124.85  1.3747
IC C2   C6   *N1   H1       1.3747  124.85  180.00  115.40  1.0100
IC C6   N1   C2    N2       1.3796  124.85 -179.62  117.36  1.3215
IC N2   N1   *C2   N3       1.3215  117.36 -179.27  121.27  1.3359
IC N1   C2   N2    H21      1.3747  117.36  180.00  121.50  1.0000
IC H21  C2   *N2   H22      1.0000  121.50  180.00  121.50  1.0000
IC C8   C7   C10   N11      1.3700  126.54  -31.51  110.58  1.5065
IC N11  C7   *C10  H101     1.5065  110.58  120.00  109.50  1.1000
IC H101 C7   *C10  H102     1.1000  109.50 -120.00  109.50  1.1000
```

```
IC C7   C10  N11   C12       1.4895  110.58 -115.16  118.55  1.5005
IC C12  C10  *N11  H111      1.5005  118.55  120.00  110.80  1.0060
IC H111 C10  *N11  H112      1.0060  110.80 -120.00  110.80  1.0060
IC C10  N11  C12   C13       1.5065  118.55  -25.32  110.91  1.5374
IC C13  N11  *C12  C16       1.5374  110.91 -112.83  109.92  1.5065
IC C13  N11  *C12  H12       1.5374  110.91 -120.00  107.50  1.0800
IC N11  C12  C16   C15       1.5005  109.92  142.84  105.54  1.4980
IC C15  C12  *C16  O15       1.4980  105.54   62.00  114.13  1.4444
IC O15  C12  *C16  H16       1.4444  114.13 -120.00  105.70  1.1110
IC O15  C16  *C15  C14       1.4387   58.88 -110.21  110.93  1.5364
IC C14  C16  *C15  H15       1.5364  110.93 -120.00  110.10  1.1110
IC C14  C12  *C13  O13       1.5393  105.61 -118.13  109.95  1.4342
IC O13  C12  *C13  H13       1.4342  109.95 -120.00  110.10  1.1000
IC C12  C13  O13   H13O      1.5374  109.95  180.00  109.00  0.9600
IC C13  C15  *C14  O14       1.5393   98.64  116.43  112.45  1.4338
IC C13  C15  *C14  H14       1.5393   98.64 -120.00  110.10  1.1000
IC C15  C14  O14   H14O      1.5364  112.45  180.00  109.00  0.9600
!ribose
IC -O3' P    O5'   C5'    1.6001  101.45  -39.25  119.00  1.4401
IC -O3' O5'  *P    O1P    1.6001  101.45 -115.82  109.74  1.4802
IC -O3' O5'  *P    O2P    1.6001  101.45  115.90  109.80  1.4801
IC  P   O5'  C5'   C4'    1.5996  119.00 -151.39  110.04  1.5160
IC O5'  C5'  C4'   C3'    1.4401  108.83 -179.85  116.10  1.5284
IC C5'  C4'  C3'   O3'    1.5160  116.10   76.70  115.12  1.4212
IC C4'  C3'  O3'   +P     1.5284  111.92  159.13  119.05  1.6001
IC C3'  O3'  +P    +O5'   1.4212  119.05  -98.86  101.45  1.5996
IC O4'  C3'  *C4'  C5'    1.4572  104.06 -120.04  116.10  1.5160
IC C2'  C4'  *C3'  O3'    1.5284  100.16 -124.08  115.12  1.4212
IC C4'  C3'  C2'   C1'    1.5284  100.16   39.58  102.04  1.5251
IC C3'  C2'  C1'   N9     1.5284  101.97  144.39  113.71  1.4896
IC O4'  C1'  N9    C4     1.5251  113.71  -97.2   125.59  1.3783
IC C1'  C3'  *C2'  O2'    1.5284  102.04 -114.67  110.81  1.4212
IC H2'  O2'  C2'   C3'    0.9600  114.97  148.63  111.92  1.5284
IC O4'  C2'  *C1'  H1'    0.0       0.0  -115.0     0.0   0.0
IC C1'  C3'  *C2'  H2''   0.0       0.0   115.0     0.0   0.0
IC C2'  C4'  *C3'  H3'    0.0       0.0   115.0     0.0   0.0
IC C3'  O4'  *C4'  H4'    0.0       0.0  -115.0     0.0   0.0
IC C4'  O5'  *C5'  H5'    0.0       0.0  -115.0     0.0   0.0
IC C4'  O5'  *C5'  H5''   0.0       0.0   115.0     0.0   0.0
DONO H2'  O2'
DONO H21   N2
DONO H22   N2
DONO H1   N1
DONO H111  N11
DONO H112  N11
DONO H13O  O13
DONO H14O  O14
ACCE O6   C6
ACCE N3
ACCE O13
ACCE O14
ACCE O15
ACCE O1P  P
ACCE O2P  P
ACCE O2'
ACCE O3'
ACCE O4'
ACCE O5'

RESI MQG            0.00 ! b-mannosyl-queuosine
GROUP
ATOM N9     NG2R51  0.04 !              O24-H24O
ATOM C8     CG2R51  0.06 !              |
```

```
ATOM H8     HGR52   0.08 !         H231-C23-H232
ATOM C7     CG2R51 -0.14 !              |
ATOM C5     CG2RC0 -0.06 !         H211-C21--O22
ATOM C6     CG2R63  0.42 !       H20   /       \    O17---*
ATOM O6     OG2D4  -0.52 !          \ /H19O H18O\  /
ATOM N1     NG2R61 -0.38 !          C20 |    |   C17
ATOM H1     HGP1    0.30 !          / \ O19 O18 /  \
ATOM C2     CG2R64  0.65 !  H20O-O20   \|    | /    H17
ATOM N2     NG2S3  -0.60 !              C19--C18
ATOM H21    HGP4    0.30 !              |    |
ATOM H22    HGP4    0.30 !              H19  H18
ATOM N3     NG2R62 -0.77
ATOM C4     CG2RC0  0.32 !                       *
GROUP                    !                H13   /
ATOM C10    CG324   0.18 !                  \  /
ATOM H101   HGA2    0.09 !    H101 H111 H12  C13      H14
ATOM H102   HGA2    0.09 !       |   |    \ /    \  /
ATOM N11    NG3P2  -0.35 !  >---C10--N11--C12     C14
ATOM H111   HGP2    0.28 !       |   |(+)  \      / \
ATOM H112   HGP2    0.28 !    H102 H112    C16==C15  O14-H14O
ATOM C12    CG3C53  0.34 !                 /      \
ATOM H12    HGA1    0.09 !                H16     H15
GROUP
ATOM C15    CG2R51 -0.20 !            O6
ATOM H15    HGR51   0.26 !            ||         >
ATOM C16    CG2R51 -0.24 !            C6        /
ATOM H16    HGR51   0.18 !           /  \      /
GROUP                    !       H1-N1   C5--C7\\
ATOM C14    CG3C51  0.14 !          |    ||     C8-H8
ATOM H14    HGA1    0.09 !          C2   C4--N9/
ATOM O14    OG311  -0.65 !         / \\ /      \
ATOM H14O   HGP1    0.42 !   H21-N2   N3         \
GROUP                    !       |               \
ATOM C13    CG3C51  0.14 !      H22               \
ATOM H13    HGA1    0.09 !                         \
ATOM O17    OG301  -0.41 !     O1P    H5' H4'  O4'  \
ATOM C17    CC3162  0.29 !      |      |    \ /   \  \
ATOM H17    HCA1    0.09 !     -P-O5'-C5'---C4'    C1'
ATOM C21    CC3163  0.11 !      |      |     \     / \
ATOM H211   HCA1    0.09 !     O2P    H5''   C3'--C2' H1'
ATOM O22    OC3C61 -0.40 !                  / \   / \
GROUP                    !                O3' H3' O2' H2''
ATOM C18    CC3161  0.14 !                 |       |
ATOM H18    HCA1    0.09 !                        H2'
ATOM O18    OC311  -0.65
ATOM H18O   HCP1    0.42 !  !!!! PATCH 7GNM for the neutral amino form
GROUP
ATOM C19    CC3161  0.14
ATOM H19    HCA1    0.09
ATOM O19    OC311  -0.65
ATOM H19O   HCP1    0.42
GROUP
ATOM C20    CC3161  0.14
ATOM H20    HCA1    0.09
ATOM O20    OC311  -0.65
ATOM H20O   HCP1    0.42
GROUP
ATOM C23    CC321   0.05
ATOM H231   HCA2    0.09
ATOM H232   HCA2    0.09
ATOM O24    OC311  -0.65
ATOM H24O   HCP1    0.42
GROUP
```

```
ATOM P       P        1.50
ATOM O1P     ON3     -0.78
ATOM O2P     ON3     -0.78
ATOM O5'     ON2     -0.57
ATOM C5'     CN8B    -0.08
ATOM H5'     HN8      0.09
ATOM H5''    HN8      0.09
GROUP
ATOM C4'     CN7      0.16
ATOM H4'     HN7      0.09
ATOM O4'     ON6B    -0.50
ATOM C1'     CN7B     0.16
ATOM H1'     HN7      0.09
GROUP
ATOM C2'     CN7B     0.14
ATOM H2''    HN7      0.09
ATOM O2'     ON5     -0.66
ATOM H2'     HN5      0.43
GROUP
ATOM C3'     CN7      0.01
ATOM H3'     HN7      0.09
ATOM O3'     ON2     -0.57
BOND N9   C8   N9   C4   C8   H8   C8   C7
BOND C7   C5   C7   C10  C5   C6   C5   C4
BOND C6   O6   C6   N1   N1   H1   N1   C2
BOND C2   N2   C2   N3   N2   H21  N2   H22
BOND N3   C4   C10  H101 C10  H102 C10  N11
BOND N11  H111 N11  H112 N11  C12  C12  H12
BOND C12  C16  C12  C13  C14  O14  C14  H14
BOND C14  C15  C14  C13  O14  H14O C15  H15
BOND C15  C16  C16  H16  O22  C17  O22  C21
BOND C17  H17  C17  O17  C17  C18  O17  C13
BOND C13  H13  C21  H211 C21  C20  C21  C23
BOND C18  O18  C18  H18  C18  C19  O18  H18O
BOND C19  O19  C19  H19  C19  C20  O19  H19O
BOND C20  O20  C20  H20  O20  H20O C23  O24
BOND C23  H231 C23  H232 O24  H24O
BOND P    O1P     P    O2P     P    O5'     O5'  C5'     C5'  H5''
BOND C5'  C4'     C4'  O4'     C4'  C3'     O4'  C1'
BOND C1'  N9      C1'  C2'     C2'  C3'     C3'  O3'     O3'  +P
BOND C2'  O2'     O2'  H2'
BOND C1'  H1'     C2'  H2''    C3'  H3'     C4'  H4'     C5'  H5'
IMPR C6     C5     N1     O6     C2     N1     N3     N2     N2     H22    H21    C2
IC C8   C4   *N9   C1'      1.3791  105.54 -179.95  126.56  1.4896
IC C4   N9   C8    C7       1.3804  110.22    0.33  108.83  1.3705
IC C7   N9   *C8   H8       1.3705  108.83  177.88  121.20  1.0827
IC N9   C8   C7    C10      1.3857  108.83  171.91  124.51  1.4918
IC C10  C8   *C7   C5       1.4918  124.51 -173.06  106.47  1.4471
IC C4   C7   *C5   C6       1.4024  107.98  179.52  133.30  1.4095
IC C7   C5   C6    N1       1.4471  133.30 -178.59  113.57  1.3792
IC N1   C5   *C6   O6       1.3792  113.57 -178.96  127.85  1.2328
IC C5   C6   N1    C2       1.4095  113.57   -0.15  125.13  1.3724
IC C2   C6   *N1   H1       1.3724  125.13  179.13  117.24  0.9994
IC C6   N1   C2    N2       1.3792  125.13  179.67  117.42  1.3220
IC N2   N1   *C2   N3       1.3220  117.42  179.26  121.27  1.3341
IC N1   C2   N2    H21      1.3724  117.42   -0.63  124.15  0.9931
IC H21  C2   *N2   H22      0.9931  124.15 -179.66  115.88  0.9962
IC C8   C7   C10   N11      1.3705  124.51  146.46  111.32  1.5028
IC N11  C7   *C10  H101     1.5028  111.32 -118.54  112.04  1.1040
IC H101 C7   *C10  H102     1.1040  112.04 -124.45  110.74  1.1048
IC C7   C10  N11   C12      1.4918  111.32 -177.34  118.72  1.4938
IC C12  C10  *N11  H111     1.4938  118.72 -120.20  106.30  1.0186
IC H111 C10  *N11  H112     1.0186  106.30 -114.13  110.59  1.0049
```

```
IC C10  N11  C12   C13       1.5028  118.72 -150.63  110.11  1.5129
IC C13  N11  *C12  C16       1.5129  110.11 -114.84  109.73  1.5008
IC C16  N11  *C12  H12       1.5008  109.73 -125.00  108.86  1.0869
IC N11  C12  C16   C15       1.4938  109.73  133.12  109.91  1.3762
IC C15  C12  *C16  H16       1.3762  109.91  173.38  124.56  1.0836
IC C12  C16  C15   C14       1.5008  109.91    0.44  111.04  1.5145
IC C14  C16  *C15  H15       1.5145  111.04  169.67  127.30  1.0840
IC C13  C15  *C14  O14       1.5431  102.73  122.98  110.44  1.4180
IC O14  C15  *C14  H14       1.4180  110.44  119.01  110.49  1.1046
IC C15  C14  O14   H14O      1.5145  110.44  171.33  108.79  0.9758
IC C14  C12  *C13  O17       1.5431  105.41 -121.55  109.82  1.4353
IC C14  C12  *C13  H13       1.5431  105.41  114.66  110.72  1.1058
IC C12  C13  O17   C17       1.5129  109.82 -115.13  115.38  1.4320
IC C13  O17  C17   C18       1.4353  115.38  118.62  111.92  1.5323
IC C18  O17  *C17  O22       1.5323  111.92  121.39  109.82  1.4278
IC O22  O17  *C17  H17       1.4278  109.82  118.49  111.61  1.1127
IC O17  C17  O22   C21       1.4320  109.82  -54.40  113.28  1.4458
IC C17  O22  C21   C20       1.4278  113.28  -39.38  111.25  1.5288
IC C20  O22  *C21  C23       1.5288  111.25  128.43  114.44  1.5269
IC C20  O22  *C21  H211      1.5288  111.25 -116.03  103.72  1.1174
IC O17  C17  C18   C19       1.4320  111.92   95.02  111.19  1.5304
IC C19  C17  *C18  O18       1.5304  111.19 -126.69  113.05  1.4337
IC O18  C17  *C18  H18       1.4337  113.05 -117.58  107.56  1.1169
IC C17  C18  O18   H18O      1.5323  113.05  151.63  103.45  0.9725
IC C20  C18  *C19  O19       1.5159  109.18 -125.55  112.37  1.4318
IC O19  C18  *C19  H19       1.4318  112.37 -118.31  107.98  1.1179
IC C18  C19  O19   H19O      1.5304  112.37 -171.81  107.95  0.9671
IC C19  C21  *C20  O20       1.5159  109.32 -125.61  110.26  1.4264
IC C19  C21  *C20  H20       1.5159  109.32  116.47  108.75  1.1177
IC C21  C20  O20   H20O      1.5288  110.26 -128.58  110.57  0.9639
IC O22  C21  C23   O24       1.4458  114.44   30.27  113.91  1.4307
IC O24  C21  *C23  H231      1.4307  113.91 -120.11  109.09  1.1124
IC O24  C21  *C23  H232      1.4307  113.91  123.39  109.63  1.1152
IC C21  C23  O24   H24O      1.5269  113.91   63.06  109.95  0.9655
!ribose
IC -O3' P    O5'   C5'    1.6001  101.45  -39.25  119.00  1.4401
IC -O3' O5'  *P    O1P    1.6001  101.45 -115.82  109.74  1.4802
IC -O3' O5'  *P    O2P    1.6001  101.45  115.90  109.80  1.4801
IC  P   O5'  C5'   C4'    1.5996  119.00 -151.39  110.04  1.5160
IC O5'  C5'  C4'   C3'    1.4401  108.83 -179.85  116.10  1.5284
IC C5'  C4'  C3'   O3'    1.5160  116.10   76.70  115.12  1.4212
IC C4'  C3'  O3'   +P     1.5284  111.92  159.13  119.05  1.6001
IC C3'  O3'  +P    +O5'   1.4212  119.05  -98.86  101.45  1.5996
IC O4'  C3'  *C4'  C5'    1.4572  104.06 -120.04  116.10  1.5160
IC C2'  C4'  *C3'  O3'    1.5284  100.16 -124.08  115.12  1.4212
IC C4'  C3'  C2'   C1'    1.5284  100.16   39.58  102.04  1.5251
IC C3'  C2'  C1'   N9     1.5284  101.97  144.39  113.71  1.4896
IC O4'  C1'  N9    C4     1.5251  113.71  -97.2   125.59  1.3783
IC C1'  C3'  *C2'  O2'    1.5284  102.04 -114.67  110.81  1.4212
IC H2'  O2'  C2'   C3'    0.9600  114.97  148.63  111.92  1.5284
IC O4'  C2'  *C1'  H1'    0.0        0.0  -115.0     0.0   0.0
IC C1'  C3'  *C2'  H2''   0.0        0.0   115.0     0.0   0.0
IC C2'  C4'  *C3'  H3'    0.0        0.0   115.0     0.0   0.0
IC C3'  O4'  *C4'  H4'    0.0        0.0  -115.0     0.0   0.0
IC C4'  O5'  *C5'  H5'    0.0        0.0  -115.0     0.0   0.0
IC C4'  O5'  *C5'  H5''   0.0        0.0   115.0     0.0   0.0
DONO H2'  O2'
DONO H21   N2
DONO H22   N2
DONO H1   N1
DONO H111  N11
DONO H112  N11
DONO H14O  O14
```

```
DONO H18O  O18
DONO H19O  O19
DONO H20O  O20
DONO H24O  O24
ACCE O6   C6
ACCE N3
ACCE O14
ACCE O17
ACCE O18
ACCE O19
ACCE O20
ACCE O22
ACCE O24
ACCE O1P  P
ACCE O2P  P
ACCE O2'
ACCE O3'
ACCE O4'
ACCE O5'

RESI GQG            0.00 ! b-galactosyl-queuosine
GROUP
ATOM N9     NG2R51  0.04 !              O24-H24O
ATOM C8     CG2R51  0.06 !              |
ATOM H8     HGR52   0.08 !         H231-C23-H232
ATOM C7     CG2R51 -0.14 !              |
ATOM C5     CG2RC0 -0.06 !         H211-C21--O22
ATOM C6     CG2R63  0.42 !  H20O-O20   /       \    O17---*
ATOM O6     OG2D4  -0.52 !          \ /H19O     \  /
ATOM N1     NG2R61 -0.38 !          C20 |       C17
ATOM H1     HGP1    0.30 !          / \ O19 H18 /  \
ATOM C2     CG2R64  0.65 !       H20   \|    | /    H17
ATOM N2     NG2S3  -0.60 !              C19--C18
ATOM H21    HGP4    0.30 !              |    |
ATOM H22    HGP4    0.30 !              H19  O18-H18O
ATOM N3     NG2R62 -0.77
ATOM C4     CG2RC0  0.32 !                        *
GROUP                    !                 H13   /
ATOM C10    CG324   0.18 !                   \  /
ATOM H101   HGA2    0.09 !     H101 H111 H12  C13     H14
ATOM H102   HGA2    0.09 !        |   |    \ /    \  /
ATOM N11    NG3P2  -0.35 !   >---C10--N11--C12     C14
ATOM H111   HGP2    0.28 !        |   |(+)  \      / \
ATOM H112   HGP2    0.28 !     H102 H112    C16==C15  O14-H14O
ATOM C12    CG3C53  0.34 !                  /      \
ATOM H12    HGA1    0.09 !                 H16     H15
GROUP
ATOM C15    CG2R51 -0.20 !             O6
ATOM H15    HGR51   0.26 !             ||         >
ATOM C16    CG2R51 -0.24 !             C6        /
ATOM H16    HGR51   0.18 !            /  \      /
GROUP                    !        H1-N1   C5--C7\\
ATOM C14    CG3C51  0.14 !           |    ||     C8-H8
ATOM H14    HGA1    0.09 !           C2   C4--N9/
ATOM O14    OG311  -0.65 !          / \\ /      \
ATOM H14O   HGP1    0.42 !    H21-N2   N3        \
GROUP                    !        |               \
ATOM C13    CG3C51  0.14 !       H22               \
ATOM H13    HGA1    0.09 !                          \
ATOM O17    OG301  -0.41 !      O1P    H5' H4'  O4'  \
ATOM C17    CC3162  0.29 !       |      |    \ /  \  \
ATOM H17    HCA1    0.09 !      -P-O5'-C5'---C4'    C1'
ATOM C21    CC3163  0.11 !       |      |     \     / \
```

```
ATOM H211   HCA1    0.09 !      O2P    H5''   C3'--C2' H1'
ATOM O22    OC3C61 -0.40 !                   / \   / \
GROUP                    !                 O3' H3' O2' H2''
ATOM C18    CC3161  0.14 !                  |       |
ATOM H18    HCA1    0.09 !                         H2'
ATOM O18    OC311  -0.65
ATOM H18O   HCP1    0.42 !  !!!! PATCH 7GNM for the neutral amino form
GROUP
ATOM C19    CC3161  0.14
ATOM H19    HCA1    0.09
ATOM O19    OC311  -0.65
ATOM H19O   HCP1    0.42
GROUP
ATOM C20    CC3161  0.14
ATOM H20    HCA1    0.09
ATOM O20    OC311  -0.65
ATOM H20O   HCP1    0.42
GROUP
ATOM C23    CC321   0.05
ATOM H231   HCA2    0.09
ATOM H232   HCA2    0.09
ATOM O24    OC311  -0.65
ATOM H24O   HCP1    0.42
GROUP
ATOM P      P       1.50
ATOM O1P    ON3    -0.78
ATOM O2P    ON3    -0.78
ATOM O5'    ON2    -0.57
ATOM C5'    CN8B   -0.08
ATOM H5'    HN8     0.09
ATOM H5''   HN8     0.09
GROUP
ATOM C4'    CN7     0.16
ATOM H4'    HN7     0.09
ATOM O4'    ON6B   -0.50
ATOM C1'    CN7B    0.16
ATOM H1'    HN7     0.09
GROUP
ATOM C2'    CN7B    0.14
ATOM H2''   HN7     0.09
ATOM O2'    ON5    -0.66
ATOM H2'    HN5     0.43
GROUP
ATOM C3'    CN7     0.01
ATOM H3'    HN7     0.09
ATOM O3'    ON2    -0.57
BOND N9   C8   N9   C4   C8   H8   C8   C7
BOND C7   C5   C7   C10  C5   C6   C5   C4
BOND C6   O6   C6   N1   N1   H1   N1   C2
BOND C2   N2   C2   N3   N2   H21  N2   H22
BOND N3   C4   C10  H101 C10  H102 C10  N11
BOND N11  H111 N11  H112 N11  C12  C12  H12
BOND C12  C16  C12  C13  C14  O14  C14  H14
BOND C14  C15  C14  C13  O14  H14O C15  H15
BOND C15  C16  C16  H16  O22  C17  O22  C21
BOND C17  H17  C17  O17  C17  C18  O17  C13
BOND C13  H13  C21  H211 C21  C20  C21  C23
BOND C18  O18  C18  H18  C18  C19  O18  H18O
BOND C19  O19  C19  H19  C19  C20  O19  H19O
BOND C20  O20  C20  H20  O20  H20O C23  O24
BOND C23  H231 C23  H232 O24  H24O
BOND P    O1P     P    O2P     P    O5'     O5'  C5'     C5'  H5''
BOND C5'  C4'     C4'  O4'     C4'  C3'     O4'  C1'
```

```
BOND C1'  N9       C1'  C2'      C2'  C3'      C3'  O3'      O3'  +P
BOND C2'  O2'      O2'  H2'
BOND C1'  H1'      C2'  H2''     C3'  H3'      C4'  H4'      C5'  H5'
IMPR C6      C5      N1      O6      C2      N1      N3      N2      N2      H22     H21     C2
IC C8   C4   *N9   C1'       1.3791  105.54 -179.95  126.56  1.4896
IC C4   N9   C8    C7        1.3804  110.22    0.33  108.83  1.3705
IC C7   N9   *C8   H8        1.3705  108.83  177.88  121.20  1.0827
IC N9   C8   C7    C10       1.3857  108.83  171.91  124.51  1.4918
IC C10  C8   *C7   C5        1.4918  124.51 -173.06  106.47  1.4471
IC C4   C7   *C5   C6        1.4024  107.98  179.52  133.30  1.4095
IC C7   C5   C6    N1        1.4471  133.30 -178.59  113.57  1.3792
IC N1   C5   *C6   O6        1.3792  113.57 -178.96  127.85  1.2328
IC C5   C6   N1    C2        1.4095  113.57   -0.15  125.13  1.3724
IC C2   C6   *N1   H1        1.3724  125.13  179.13  117.24  0.9994
IC C6   N1   C2    N2        1.3792  125.13  179.67  117.42  1.3220
IC N2   N1   *C2   N3        1.3220  117.42  179.26  121.27  1.3341
IC N1   C2   N2    H21       1.3724  117.42   -0.63  124.15  0.9931
IC H21  C2   *N2   H22       0.9931  124.15 -179.66  115.88  0.9962
IC C8   C7   C10   N11       1.3705  124.51  146.46  111.32  1.5028
IC N11  C7   *C10  H101      1.5028  111.32 -118.53  112.06  1.1049
IC H101 C7   *C10  H102      1.1049  112.06 -124.46  110.74  1.1048
IC C7   C10  N11   C12       1.4918  111.32 -177.34  118.72  1.4938
IC C12  C10  *N11  H111      1.4938  118.72 -120.20  106.30  1.0186
IC H111 C10  *N11  H112      1.0186  106.30 -114.13  110.59  1.0049
IC C10  N11  C12   C13       1.5028  118.72 -150.63  110.11  1.5129
IC C13  N11  *C12  C16       1.5129  110.11 -114.84  109.73  1.5008
IC C16  N11  *C12  H12       1.5008  109.73 -125.00  108.86  1.0869
IC N11  C12  C16   C15       1.4938  109.73  133.12  109.91  1.3762
IC C15  C12  *C16  H16       1.3762  109.91  173.33  124.53  1.0834
IC C12  C16  C15   C14       1.5008  109.91    0.44  111.04  1.5145
IC C14  C16  *C15  H15       1.5145  111.04  169.67  127.30  1.0840
IC C13  C15  *C14  O14       1.5431  102.73  122.98  110.44  1.4180
IC O14  C15  *C14  H14       1.4180  110.44  119.00  110.52  1.1038
IC C15  C14  O14   H14O      1.5145  110.44  171.33  108.79  0.9758
IC C14  C12  *C13  O17       1.5431  105.41 -121.55  109.82  1.4353
IC C14  C12  *C13  H13       1.5431  105.41  114.66  110.72  1.1058
IC C12  C13  O17   C17       1.5129  109.82 -115.13  115.38  1.4320
IC C13  O17  C17   C18       1.4353  115.38  118.62  111.92  1.5323
IC C18  O17  *C17  O22       1.5323  111.92  121.39  109.82  1.4278
IC O22  O17  *C17  H17       1.4278  109.82  118.49  111.61  1.1127
IC O17  C17  O22   C21       1.4320  109.82  -54.40  113.28  1.4458
IC C17  O22  C21   C20       1.4278  113.28  -39.38  111.25  1.5288
IC C20  O22  *C21  C23       1.5288  111.25  128.42  114.48  1.5268
IC C20  O22  *C21  H211      1.5288  111.25 -116.03  103.72  1.1174
IC O17  C17  C18   C19       1.4320  111.92   95.02  111.19  1.5304
IC C19  C17  *C18  O18       1.5304  111.19  127.40  111.78  1.4342
IC O18  C17  *C18  H18       1.4342  111.78  115.99  106.08  1.1166
IC C17  C18  O18   H18O      1.5323  111.78   25.23  103.46  0.9724
IC C20  C18  *C19  O19       1.5159  109.18 -125.55  112.37  1.4318
IC O19  C18  *C19  H19       1.4318  112.37 -118.31  107.98  1.1179
IC C18  C19  O19   H19O      1.5304  112.37 -171.81  107.95  0.9671
IC C19  C21  *C20  O20       1.5159  109.32  123.65  113.64  1.4266
IC C19  C21  *C20  H20       1.5159  109.32 -117.60  106.96  1.1179
IC C21  C20  O20   H20O      1.5288  113.64  108.27  110.59  0.9637
IC O22  C21  C23   O24       1.4458  114.48   30.26  113.86  1.4316
IC O24  C21  *C23  H231      1.4316  113.86 -120.03  109.10  1.1122
IC O24  C21  *C23  H232      1.4316  113.86  123.37  109.68  1.1144
IC C21  C23  O24   H24O      1.5268  113.86   63.08  109.95  0.9655
!ribose
IC -O3' P    O5'   C5'    1.6001  101.45  -39.25  119.00  1.4401
IC -O3' O5'  *P    O1P    1.6001  101.45 -115.82  109.74  1.4802
IC -O3' O5'  *P    O2P    1.6001  101.45  115.90  109.80  1.4801
IC  P   O5'  C5'   C4'    1.5996  119.00 -151.39  110.04  1.5160
```

```
IC O5'  C5'  C4'   C3'    1.4401  108.83 -179.85  116.10  1.5284
IC C5'  C4'  C3'   O3'    1.5160  116.10   76.70  115.12  1.4212
IC C4'  C3'  O3'   +P     1.5284  111.92  159.13  119.05  1.6001
IC C3'  O3'  +P    +O5'   1.4212  119.05  -98.86  101.45  1.5996
IC O4'  C3'  *C4'  C5'    1.4572  104.06 -120.04  116.10  1.5160
IC C2'  C4'  *C3'  O3'    1.5284  100.16 -124.08  115.12  1.4212
IC C4'  C3'  C2'   C1'    1.5284  100.16   39.58  102.04  1.5251
IC C3'  C2'  C1'   N9     1.5284  101.97  144.39  113.71  1.4896
IC O4'  C1'  N9    C4     1.5251  113.71  -97.2   125.59  1.3783
IC C1'  C3'  *C2'  O2'    1.5284  102.04 -114.67  110.81  1.4212
IC H2'  O2'  C2'   C3'    0.9600  114.97  148.63  111.92  1.5284
IC O4'  C2'  *C1'  H1'    0.0       0.0  -115.0     0.0   0.0
IC C1'  C3'  *C2'  H2''   0.0       0.0   115.0     0.0   0.0
IC C2'  C4'  *C3'  H3'    0.0       0.0   115.0     0.0   0.0
IC C3'  O4'  *C4'  H4'    0.0       0.0  -115.0     0.0   0.0
IC C4'  O5'  *C5'  H5'    0.0       0.0  -115.0     0.0   0.0
IC C4'  O5'  *C5'  H5''   0.0       0.0   115.0     0.0   0.0
DONO H2'  O2'
DONO H21   N2
DONO H22   N2
DONO H1   N1
DONO H111  N11
DONO H112  N11
DONO H14O  O14
DONO H18O  O18
DONO H19O  O19
DONO H20O  O20
DONO H24O  O24
ACCE O6   C6
ACCE N3
ACCE O14
ACCE O17
ACCE O18
ACCE O19
ACCE O20
ACCE O22
ACCE O24
ACCE O1P  P
ACCE O2P  P
ACCE O2'
ACCE O3'
ACCE O4'
ACCE O5'

RESI DWG           -1.00 ! 4-demethylwyosine             12/19
GROUP
ATOM N9     NG2R51  0.02 !                     O6
ATOM C8     CG2R53  0.37 !                     ||
ATOM H8     HGR52   0.09 !              H12    C6
ATOM N7     NG2R50 -0.67 !   H101        |    /  \
ATOM C5     CG2RC0  0.00 !      \      //C12-N1   C5--N7\\
ATOM C6     CG2R63  0.69 !  H102-C10-C11    |    ||     C8-H8
ATOM O6     OG2D4  -0.50 !      /       \N2==C2   C4--N9/
ATOM N1     NG2RC0 -0.11 !   H103            \  /      \
ATOM C2     CG2RC0  0.44 !                     N3        \
ATOM N2     NG2R50 -0.70 !                     |          \
ATOM N3     NG2R61 -0.42 !                     H3          \
ATOM H3     HGP1    0.34 !                                  \
ATOM C4     CG2RC0  0.29 !              O1P    H5' H4'  O4'  \
ATOM C12    CG2R51 -0.42 !               |      |    \ /   \  \
ATOM H12    HGR52   0.21 !              -P-O5'-C5'---C4'    C1'
ATOM C11    CG2R51  0.40 !               |      |     \     / \
GROUP                    !              O2P    H5''   C3'--C2' H1'
```

```
ATOM C10    CG331  -0.30 !                          / \   / \
ATOM H101   HGA3    0.09 !                        O3' H3' O2' H2''
ATOM H102   HGA3    0.09 !                         |       |
ATOM H103   HGA3    0.09 !                                H2'
GROUP
ATOM P      P       1.50
ATOM O1P    ON3    -0.78
ATOM O2P    ON3    -0.78
ATOM O5'    ON2    -0.57
ATOM C5'    CN8B   -0.08
ATOM H5'    HN8     0.09
ATOM H5''   HN8     0.09
GROUP
ATOM C4'    CN7     0.16
ATOM H4'    HN7     0.09
ATOM O4'    ON6B   -0.50
ATOM C1'    CN7B    0.16
ATOM H1'    HN7     0.09
GROUP
ATOM C2'    CN7B    0.14
ATOM H2''   HN7     0.09
ATOM O2'    ON5    -0.66
ATOM H2'    HN5     0.43
GROUP
ATOM C3'    CN7     0.01
ATOM H3'    HN7     0.09
ATOM O3'    ON2    -0.57
BOND N9   C8   N9   C4   C8   N7   C8   H8
BOND N7   C5   C5   C6   C5   C4   C6   O6
BOND C6   N1   N1   C2   N1   C12  C2   N2
BOND C2   N3   N2   C11  N3   C4   N3   H3
BOND C10  C11  C10  H101 C10  H102 C10  H103
BOND C11  C12  C12  H12
BOND P    O1P     P    O2P     P    O5'     O5'  C5'     C5'  H5''
BOND C5'  C4'     C4'  O4'     C4'  C3'     O4'  C1'
BOND C1'  N9      C1'  C2'     C2'  C3'     C3'  O3'     O3'  +P
BOND C2'  O2'     O2'  H2'
BOND C1'  H1'     C2'  H2''    C3'  H3'     C4'  H4'     C5'  H5'
IMPR C6     C5     N1     O6
IC C8   C4   *N9   C1'       1.3791  105.54 -179.95  126.56  1.4896
IC C4   N9   C8    N7        1.3668  106.36   -0.74  112.66  1.3161
IC N7   N9   *C8   H8        1.3161  112.66  179.52  121.39  1.0813
IC N9   C8   N7    C5        1.3859  112.66   -0.05  104.67  1.3822
IC C4   N7   *C5   C6        1.3927  110.50  178.33  127.67  1.4475
IC N7   C5   C6    N1        1.3822  127.67  179.82  109.42  1.4356
IC N1   C5   *C6   O6        1.4356  109.42 -179.70  129.66  1.2219
IC C5   C6   N1    C2        1.4475  109.42    2.29  127.87  1.3834
IC C2   C6   *N1   C12       1.3834  127.87  175.44  126.56  1.3882
IC C6   N1   C2    N2        1.4356  127.87  176.40  113.52  1.3138
IC N2   N1   *C2   N3        1.3138  113.52  178.07  119.44  1.3866
IC C4   C2   *N3   H3        1.3677  115.68  176.96  116.56  1.0146
IC C6   N1   C12   C11       1.4356  126.56 -176.39  105.94  1.3748
IC C11  N1   *C12  H12       1.3748  105.94  179.83  120.66  1.0795
IC N2   C12  *C11  C10       1.3931  111.03  179.97  128.62  1.4912
IC C12  C11  C10   H101      1.3748  128.62 -120.53  110.77  1.0938
IC H101 C11  *C10  H102      1.0938  110.77  120.33  110.57  1.0940
IC H101 C11  *C10  H103      1.0938  110.77 -119.36  110.76  1.0942
!ribose
IC -O3' P    O5'   C5'    1.6001  101.45  -39.25  119.00  1.4401
IC -O3' O5'  *P    O1P    1.6001  101.45 -115.82  109.74  1.4802
IC -O3' O5'  *P    O2P    1.6001  101.45  115.90  109.80  1.4801
IC  P   O5'  C5'   C4'    1.5996  119.00 -151.39  110.04  1.5160
IC O5'  C5'  C4'   C3'    1.4401  108.83 -179.85  116.10  1.5284
```

```
IC C5'  C4'  C3'   O3'    1.5160  116.10   76.70  115.12  1.4212
IC C4'  C3'  O3'   +P     1.5284  111.92  159.13  119.05  1.6001
IC C3'  O3'  +P    +O5'   1.4212  119.05  -98.86  101.45  1.5996
IC O4'  C3'  *C4'  C5'    1.4572  104.06 -120.04  116.10  1.5160
IC C2'  C4'  *C3'  O3'    1.5284  100.16 -124.08  115.12  1.4212
IC C4'  C3'  C2'   C1'    1.5284  100.16   39.58  102.04  1.5251
IC C3'  C2'  C1'   N9     1.5284  101.97  144.39  113.71  1.4896
IC O4'  C1'  N9    C4     1.5251  113.71  -97.2   125.59  1.3783
IC C1'  C3'  *C2'  O2'    1.5284  102.04 -114.67  110.81  1.4212
IC H2'  O2'  C2'   C3'    0.9600  114.97  148.63  111.92  1.5284
IC O4'  C2'  *C1'  H1'    0.0       0.0  -115.0     0.0   0.0
IC C1'  C3'  *C2'  H2''   0.0       0.0   115.0     0.0   0.0
IC C2'  C4'  *C3'  H3'    0.0       0.0   115.0     0.0   0.0
IC C3'  O4'  *C4'  H4'    0.0       0.0  -115.0     0.0   0.0
IC C4'  O5'  *C5'  H5'    0.0       0.0  -115.0     0.0   0.0
IC C4'  O5'  *C5'  H5''   0.0       0.0   115.0     0.0   0.0
DONO H2'  O2'
DONO H3   N3
ACCE O6   C6
ACCE N2
ACCE N7
ACCE O1P  P
ACCE O2P  P
ACCE O2'
ACCE O3'
ACCE O4'
ACCE O5'

RESI IMG           -1.00 ! wyosine               12/19
GROUP
ATOM N9     NG2R51  0.02 !                   O6
ATOM C8     CG2R53  0.38 !                   ||
ATOM H8     HGR52   0.08 !            H12    C6
ATOM N7     NG2R50 -0.68 !  H101       |    /  \
ATOM C5     CG2RC0  0.01 !     \     //C12-N1   C5--N7\\
ATOM C6     CG2R63  0.69 ! H102-C10-C11    |    ||     C8-H8
ATOM O6     OG2D4  -0.51 !     /      \N2==C2   C4--N9/
ATOM N1     NG2RC0 -0.10 !  H103            \  /       \
ATOM C2     CG2RC0  0.45 !                   N3         \
ATOM N2     NG2R50 -0.69 !                   |           \
ATOM N3     NG2R61 -0.34 !                  C3            \
ATOM C4     CG2RC0  0.30 !                /  |  \          \
ATOM C12    CG2R51 -0.43 !            H31  H32  H33         \
ATOM H12    HGR52   0.21 !                                   \
ATOM C11    CG2R51  0.41 !              O1P    H5' H4'  O4'   \
GROUP                    !               |      |    \ /    \  \
ATOM C3     CG331  -0.04 !              -P-O5'-C5'---C4'    C1'
ATOM H31    HGA3    0.09 !               |      |      \     / \
ATOM H32    HGA3    0.09 !              O2P    H5''   C3'--C2' H1'
ATOM H33    HGA3    0.09 !                           / \   / \
GROUP                    !                         O3' H3' O2' H2''
ATOM C10    CG331  -0.30 !                          |       |
ATOM H101   HGA3    0.09 !                                 H2'
ATOM H102   HGA3    0.09
ATOM H103   HGA3    0.09
GROUP
ATOM P      P       1.50
ATOM O1P    ON3    -0.78
ATOM O2P    ON3    -0.78
ATOM O5'    ON2    -0.57
ATOM C5'    CN8B   -0.08
ATOM H5'    HN8     0.09
ATOM H5''   HN8     0.09
```

```
GROUP
ATOM C4'    CN7     0.16
ATOM H4'    HN7     0.09
ATOM O4'    ON6B   -0.50
ATOM C1'    CN7B    0.16
ATOM H1'    HN7     0.09
GROUP
ATOM C2'    CN7B    0.14
ATOM H2''   HN7     0.09
ATOM O2'    ON5    -0.66
ATOM H2'    HN5     0.43
GROUP
ATOM C3'    CN7     0.01
ATOM H3'    HN7     0.09
ATOM O3'    ON2    -0.57
BOND N9   C8   N9   C4   C8   N7   C8   H8
BOND N7   C5   C5   C6   C5   C4   C6   O6
BOND C6   N1   N1   C2   N1   C12  C2   N2
BOND C2   N3   N2   C11  N3   C4   N3   C3
BOND C12  C11  C12  H12  C11  C10  C10  H101
BOND C10  H102 C10  H103 C3   H31  C3   H32
BOND C3   H33
BOND P    O1P     P    O2P     P    O5'     O5'  C5'     C5'  H5''
BOND C5'  C4'     C4'  O4'     C4'  C3'     O4'  C1'
BOND C1'  N9      C1'  C2'     C2'  C3'     C3'  O3'     O3'  +P
BOND C2'  O2'     O2'  H2'
BOND C1'  H1'     C2'  H2''    C3'  H3'     C4'  H4'     C5'  H5'
IMPR C6     C5     N1     O6
IC C8   C4   *N9   C1'       1.3791  105.54 -179.95  126.56  1.4896
IC C4   N9   C8    N7        1.3930  105.72    0.39  113.47  1.3197
IC N7   N9   *C8   H8        1.3197  113.47  179.93  121.94  1.0906
IC N9   C8   N7    C5        1.3773  113.47    0.24  105.21  1.3948
IC C4   N7   *C5   C6        1.4034  109.42  178.06  130.09  1.4206
IC N7   C5   C6    N1        1.3948  130.09 -172.32  113.05  1.4249
IC N1   C5   *C6   O6        1.4249  113.05  176.99  123.46  1.2393
IC C5   C6   N1    C2        1.4206  113.05   -4.41  125.84  1.4037
IC C2   C6   *N1   C12       1.4037  125.84  175.64  128.26  1.3809
IC C6   N1   C2    N2        1.4249  125.84  177.07  110.78  1.3515
IC N2   N1   *C2   N3        1.3515  110.78  177.75  119.13  1.3938
IC C6   N1   C12   C11       1.4249  128.26 -176.83  107.34  1.3599
IC C11  N1   *C12  H12       1.3599  107.34 -179.91  123.22  1.0821
IC C4   C2   *N3   C3        1.4001  115.49 -177.38  117.37  1.4744
IC C2   N3   C3    H31       1.3938  117.37  129.93  111.66  1.1130
IC H31  N3   *C3   H32       1.1130  111.66  120.63  110.94  1.1140
IC H31  N3   *C3   H33       1.1130  111.66 -119.78  112.54  1.1151
IC N2   C12  *C11  C10       1.3817  111.05  179.93  130.40  1.4822
IC C12  C11  C10   H101      1.3599  130.40  120.37  109.37  1.1097
IC H101 C11  *C10  H102      1.1097  109.37  119.39  109.36  1.1093
IC H101 C11  *C10  H103      1.1097  109.37 -120.31  110.31  1.1092
!ribose
IC -O3' P    O5'   C5'    1.6001  101.45  -39.25  119.00  1.4401
IC -O3' O5'  *P    O1P    1.6001  101.45 -115.82  109.74  1.4802
IC -O3' O5'  *P    O2P    1.6001  101.45  115.90  109.80  1.4801
IC  P   O5'  C5'   C4'    1.5996  119.00 -151.39  110.04  1.5160
IC O5'  C5'  C4'   C3'    1.4401  108.83 -179.85  116.10  1.5284
IC C5'  C4'  C3'   O3'    1.5160  116.10   76.70  115.12  1.4212
IC C4'  C3'  O3'   +P     1.5284  111.92  159.13  119.05  1.6001
IC C3'  O3'  +P    +O5'   1.4212  119.05  -98.86  101.45  1.5996
IC O4'  C3'  *C4'  C5'    1.4572  104.06 -120.04  116.10  1.5160
IC C2'  C4'  *C3'  O3'    1.5284  100.16 -124.08  115.12  1.4212
IC C4'  C3'  C2'   C1'    1.5284  100.16   39.58  102.04  1.5251
IC C3'  C2'  C1'   N9     1.5284  101.97  144.39  113.71  1.4896
IC O4'  C1'  N9    C4     1.5251  113.71  -97.2   125.59  1.3783
```

```
IC C1'  C3'  *C2'  O2'    1.5284  102.04 -114.67  110.81  1.4212
IC H2'  O2'  C2'   C3'    0.9600  114.97  148.63  111.92  1.5284
IC O4'  C2'  *C1'  H1'    0.0       0.0  -115.0     0.0   0.0
IC C1'  C3'  *C2'  H2''   0.0       0.0   115.0     0.0   0.0
IC C2'  C4'  *C3'  H3'    0.0       0.0   115.0     0.0   0.0
IC C3'  O4'  *C4'  H4'    0.0       0.0  -115.0     0.0   0.0
IC C4'  O5'  *C5'  H5'    0.0       0.0  -115.0     0.0   0.0
IC C4'  O5'  *C5'  H5''   0.0       0.0   115.0     0.0   0.0
DONO H2'  O2'
ACCE O6   C6
ACCE N2
ACCE N7
ACCE O1P  P
ACCE O2P  P
ACCE O2'
ACCE O3'
ACCE O4'
ACCE O5'

RESI IWG           -1.00 ! isowyosine
GROUP
ATOM N9     NG2R51  0.02 !         H132 H133 O6
ATOM C8     CG2R53  0.37 !           \   /   ||
ATOM H8     HGR52   0.09 !       H131-C13    C6
ATOM N7     NG2R50 -0.67 !  H101       |    /  \
ATOM C5     CG2RC0  0.00 !     \     //C12-N1   C5--N7\\
ATOM C6     CG2R63  0.69 ! H102-C10-C11    |    ||     C8-H8
ATOM O6     OG2D4  -0.50 !     /      \N2==C2   C4--N9/
ATOM N1     NG2RC0 -0.11 !  H103            \  /      \
ATOM C2     CG2RC0  0.44 !                   N3        \
ATOM N2     NG2R50 -0.70 !                   |          \
ATOM N3     NG2R61 -0.42 !                   H3          \
ATOM H3     HGP1    0.34 !                                \
ATOM C4     CG2RC0  0.29 !            O1P    H5' H4'  O4'  \
ATOM C12    CG2R51 -0.25 !             |      |    \ /   \  \
ATOM C11    CG2R51  0.40 !            -P-O5'-C5'---C4'    C1'
GROUP                    !             |      |     \     / \
ATOM C10    CG331  -0.30 !            O2P    H5''   C3'--C2' H1'
ATOM H101   HGA3    0.09 !                         / \   / \
ATOM H102   HGA3    0.09 !                       O3' H3' O2' H2''
ATOM H103   HGA3    0.09 !                        |       |
GROUP                    !                               H2'
ATOM C13    CG331  -0.23
ATOM H131   HGA3    0.09
ATOM H132   HGA3    0.09
ATOM H133   HGA3    0.09
GROUP
ATOM P      P       1.50
ATOM O1P    ON3    -0.78
ATOM O2P    ON3    -0.78
ATOM O5'    ON2    -0.57
ATOM C5'    CN8B   -0.08
ATOM H5'    HN8     0.09
ATOM H5''   HN8     0.09
GROUP
ATOM C4'    CN7     0.16
ATOM H4'    HN7     0.09
ATOM O4'    ON6B   -0.50
ATOM C1'    CN7B    0.16
ATOM H1'    HN7     0.09
GROUP
ATOM C2'    CN7B    0.14
ATOM H2''   HN7     0.09
```

```
ATOM O2'    ON5    -0.66
ATOM H2'    HN5     0.43
GROUP
ATOM C3'    CN7     0.01
ATOM H3'    HN7     0.09
ATOM O3'    ON2    -0.57
BOND N9   C8   N9   C4   C8   N7   C8   H8
BOND N7   C5   C5   C6   C5   C4   C6   O6
BOND C6   N1   N1   C2   N1   C12  C2   N2
BOND C2   N3   N2   C11  N3   C4   N3   H3
BOND C10  C11  C10  H101 C10  H102 C10  H103
BOND C11  C12  C12  C13  C13  H131 C13  H132
BOND C13  H133
BOND P    O1P     P    O2P     P    O5'     O5'  C5'     C5'  H5''
BOND C5'  C4'     C4'  O4'     C4'  C3'     O4'  C1'
BOND C1'  N9      C1'  C2'     C2'  C3'     C3'  O3'     O3'  +P
BOND C2'  O2'     O2'  H2'
BOND C1'  H1'     C2'  H2''    C3'  H3'     C4'  H4'     C5'  H5'
IMPR C6     C5     N1     O6
IC C8   C4   *N9   C1'       1.3791  105.54 -179.95  126.56  1.4896
IC C4   N9   C8    N7        1.3785  105.65    0.02  113.14  1.3241
IC N7   N9   *C8   H8        1.3241  113.14  179.98  122.11  1.0906
IC N9   C8   N7    C5        1.3784  113.14    0.03  105.17  1.3955
IC C4   N7   *C5   C6        1.3976  108.97  179.46  130.78  1.4271
IC N7   C5   C6    N1        1.3955  130.78 -177.10  113.18  1.4342
IC N1   C5   *C6   O6        1.4342  113.18  178.81  122.25  1.2392
IC C5   C6   N1    C12       1.4271  113.18  175.36  129.53  1.3811
IC C12  C6   *N1   C2        1.3811  129.53 -177.53  124.90  1.4016
IC C6   N1   C2    N2        1.4342  124.90  177.60  112.45  1.3350
IC N2   N1   *C2   N3        1.3350  112.45 -179.12  119.60  1.3670
IC C4   C2   *N3   H3        1.3808  117.48  176.77  116.13  1.0058
IC N1   C2   N2    C11       1.4016  112.45    0.48  104.07  1.3856
IC C12  N2   *C11  C10       1.3762  111.55 -179.78  116.91  1.4877
IC N2   C11  C10   H101      1.3856  116.91  -59.56  109.40  1.1099
IC H101 C11  *C10  H102      1.1099  109.40  119.31  109.39  1.1097
IC H101 C11  *C10  H103      1.1099  109.40 -120.33  110.70  1.1082
IC C11  N1   *C12  C13       1.3762  106.38  179.80  124.90  1.5149
IC N1   C12  C13   H131      1.3811  124.90  179.72  111.42  1.1117
IC H131 C12  *C13  H132      1.1117  111.42  120.46  110.10  1.1121
IC H131 C12  *C13  H133      1.1117  111.42 -120.36  110.02  1.1120
!ribose
IC -O3' P    O5'   C5'    1.6001  101.45  -39.25  119.00  1.4401
IC -O3' O5'  *P    O1P    1.6001  101.45 -115.82  109.74  1.4802
IC -O3' O5'  *P    O2P    1.6001  101.45  115.90  109.80  1.4801
IC  P   O5'  C5'   C4'    1.5996  119.00 -151.39  110.04  1.5160
IC O5'  C5'  C4'   C3'    1.4401  108.83 -179.85  116.10  1.5284
IC C5'  C4'  C3'   O3'    1.5160  116.10   76.70  115.12  1.4212
IC C4'  C3'  O3'   +P     1.5284  111.92  159.13  119.05  1.6001
IC C3'  O3'  +P    +O5'   1.4212  119.05  -98.86  101.45  1.5996
IC O4'  C3'  *C4'  C5'    1.4572  104.06 -120.04  116.10  1.5160
IC C2'  C4'  *C3'  O3'    1.5284  100.16 -124.08  115.12  1.4212
IC C4'  C3'  C2'   C1'    1.5284  100.16   39.58  102.04  1.5251
IC C3'  C2'  C1'   N9     1.5284  101.97  144.39  113.71  1.4896
IC O4'  C1'  N9    C4     1.5251  113.71  -97.2   125.59  1.3783
IC C1'  C3'  *C2'  O2'    1.5284  102.04 -114.67  110.81  1.4212
IC H2'  O2'  C2'   C3'    0.9600  114.97  148.63  111.92  1.5284
IC O4'  C2'  *C1'  H1'    0.0       0.0  -115.0     0.0   0.0
IC C1'  C3'  *C2'  H2''   0.0       0.0   115.0     0.0   0.0
IC C2'  C4'  *C3'  H3'    0.0       0.0   115.0     0.0   0.0
IC C3'  O4'  *C4'  H4'    0.0       0.0  -115.0     0.0   0.0
IC C4'  O5'  *C5'  H5'    0.0       0.0  -115.0     0.0   0.0
IC C4'  O5'  *C5'  H5''   0.0       0.0   115.0     0.0   0.0
DONO H2'  O2'
```

```
DONO H3   N3
ACCE O6   C6
ACCE N2
ACCE N7
ACCE O1P  P
ACCE O2P  P
ACCE O2'
ACCE O3'
ACCE O4'
ACCE O5'

RESI MWG            -1.00 ! methylwyosine
GROUP
ATOM N9     NG2R51  0.02 !         H132 H133 O6
ATOM C8     CG2R53  0.38 !            \   /   ||
ATOM H8     HGR52   0.08 !       H131-C13    C6
ATOM N7     NG2R50 -0.68 !  H101       |    /  \
ATOM C5     CG2RC0  0.01 !     \     //C12-N1   C5--N7\\
ATOM C6     CG2R63  0.69 ! H102-C10-C11    |    ||     C8-H8
ATOM O6     OG2D4  -0.51 !     /      \N2==C2   C4--N9/
ATOM N1     NG2RC0 -0.10 !  H103             \  /      \
ATOM C2     CG2RC0  0.45 !                    N3        \
ATOM N2     NG2R50 -0.69 !                    |          \
ATOM N3     NG2R61 -0.34 !                   C3           \
ATOM C4     CG2RC0  0.30 !                 /  |  \          \
ATOM C12    CG2R51 -0.26 !            H31  H32  H33         \
ATOM C11    CG2R51  0.41 !                                   \
GROUP                    !              O1P    H5' H4'  O4'  \
ATOM C3     CG331  -0.04 !               |      |    \ /   \  \
ATOM H31    HGA3    0.09 !              -P-O5'-C5'---C4'    C1'
ATOM H32    HGA3    0.09 !               |      |     \     / \
ATOM H33    HGA3    0.09 !              O2P    H5''   C3'--C2' H1'
GROUP                    !                           / \   / \
ATOM C10    CG331  -0.30 !                        O3' H3' O2' H2''
ATOM H101   HGA3    0.09 !                         |       |
ATOM H102   HGA3    0.09 !                                H2'
ATOM H103   HGA3    0.09
GROUP
ATOM C13    CG331  -0.23
ATOM H131   HGA3    0.09
ATOM H132   HGA3    0.09
ATOM H133   HGA3    0.09
GROUP
ATOM P      P       1.50
ATOM O1P    ON3    -0.78
ATOM O2P    ON3    -0.78
ATOM O5'    ON2    -0.57
ATOM C5'    CN8B   -0.08
ATOM H5'    HN8     0.09
ATOM H5''   HN8     0.09
GROUP
ATOM C4'    CN7     0.16
ATOM H4'    HN7     0.09
ATOM O4'    ON6B   -0.50
ATOM C1'    CN7B    0.16
ATOM H1'    HN7     0.09
GROUP
ATOM C2'    CN7B    0.14
ATOM H2''   HN7     0.09
ATOM O2'    ON5    -0.66
ATOM H2'    HN5     0.43
GROUP
ATOM C3'    CN7     0.01
```

```
ATOM H3'    HN7     0.09
ATOM O3'    ON2    -0.57
BOND N9   C8   N9   C4   C8   N7   C8   H8
BOND N7   C5   C5   C6   C5   C4   C6   O6
BOND C6   N1   N1   C2   N1   C12  C2   N2
BOND C2   N3   N2   C11  N3   C4   N3   C3
BOND C12  C11  C12  C13  C11  C10  C10  H101
BOND C10  H102 C10  H103 C3   H31  C3   H32
BOND C3   H33  C13  H131 C13  H132 C13  H133
BOND P    O1P      P    O2P      P    O5'      O5'  C5'      C5'  H5''
BOND C5'  C4'      C4'  O4'      C4'  C3'      O4'  C1'
BOND C1'  N9       C1'  C2'      C2'  C3'      C3'  O3'      O3'  +P
BOND C2'  O2'      O2'  H2'
BOND C1'  H1'      C2'  H2''     C3'  H3'      C4'  H4'      C5'  H5'
IMPR C6     C5     N1     O6
IC C8   C4   *N9   C1'      1.3791  105.54 -179.95  126.56  1.4896
IC C4   N9   C8    N7       1.3690  106.19   -0.49  112.74  1.3162
IC N7   N9   *C8   H8       1.3162  112.74  179.95  121.40  1.0820
IC N9   C8   N7    C5       1.3863  112.74    0.05  104.66  1.3829
IC C4   N7   *C5   C6       1.3907  110.50  178.64  127.52  1.4444
IC N7   C5   C6    N1       1.3829  127.52 -179.54  109.69  1.4384
IC N1   C5   *C6   O6       1.4384  109.69 -179.78  128.49  1.2235
IC C5   C6   N1    C2       1.4444  109.69    1.32  126.73  1.3896
IC C2   C6   *N1   C12      1.3896  126.73  177.81  127.68  1.4037
IC C6   N1   C2    N2       1.4384  126.73  178.31  113.42  1.3131
IC N2   N1   *C2   N3       1.3131  113.42  178.34  120.52  1.3867
IC N1   C2   N2    C11      1.3896  113.42   -0.01  104.24  1.3900
IC C12  N2   *C11  C10      1.3797  111.75 -179.72  119.17  1.4930
IC N2   C11  C10   H101     1.3900  119.17  -59.12  110.55  1.0945
IC H101 C11  *C10  H102     1.0945  110.55  118.96  110.53  1.0946
IC H101 C11  *C10  H103     1.0945  110.55 -120.55  111.46  1.0925
IC C11  N1   *C12  C13      1.3797  105.03 -179.89  123.57  1.4920
IC N1   C12  C13   H131     1.4037  123.57  179.40  108.54  1.0935
IC H131 C12  *C13  H132     1.0935  108.54  119.86  111.89  1.0919
IC H131 C12  *C13  H133     1.0935  108.54 -119.87  111.87  1.0920
IC C4   C2   *N3   C3       1.3606  115.08  170.67  120.26  1.4541
IC C2   N3   C3    H31      1.3867  120.26  133.74  109.76  1.0943
IC H31  N3   *C3   H32      1.0943  109.76  121.89  111.11  1.0954
IC H31  N3   *C3   H33      1.0943  109.76 -118.72  107.03  1.0890
!ribose
IC -O3' P    O5'   C5'   1.6001  101.45  -39.25  119.00  1.4401
IC -O3' O5'  *P    O1P   1.6001  101.45 -115.82  109.74  1.4802
IC -O3' O5'  *P    O2P   1.6001  101.45  115.90  109.80  1.4801
IC  P   O5'  C5'   C4'   1.5996  119.00 -151.39  110.04  1.5160
IC O5'  C5'  C4'   C3'   1.4401  108.83 -179.85  116.10  1.5284
IC C5'  C4'  C3'   O3'   1.5160  116.10   76.70  115.12  1.4212
IC C4'  C3'  O3'   +P    1.5284  111.92  159.13  119.05  1.6001
IC C3'  O3'  +P    +O5'  1.4212  119.05  -98.86  101.45  1.5996
IC O4'  C3'  *C4'  C5'   1.4572  104.06 -120.04  116.10  1.5160
IC C2'  C4'  *C3'  O3'   1.5284  100.16 -124.08  115.12  1.4212
IC C4'  C3'  C2'   C1'   1.5284  100.16   39.58  102.04  1.5251
IC C3'  C2'  C1'   N9    1.5284  101.97  144.39  113.71  1.4896
IC O4'  C1'  N9    C4    1.5251  113.71  -97.2   125.59  1.3783
IC C1'  C3'  *C2'  O2'   1.5284  102.04 -114.67  110.81  1.4212
IC H2'  O2'  C2'   C3'   0.9600  114.97  148.63  111.92  1.5284
IC O4'  C2'  *C1'  H1'   0.0       0.0  -115.0     0.0   0.0
IC C1'  C3'  *C2'  H2''  0.0       0.0   115.0     0.0   0.0
IC C2'  C4'  *C3'  H3'   0.0       0.0   115.0     0.0   0.0
IC C3'  O4'  *C4'  H4'   0.0       0.0  -115.0     0.0   0.0
IC C4'  O5'  *C5'  H5'   0.0       0.0  -115.0     0.0   0.0
IC C4'  O5'  *C5'  H5''  0.0       0.0   115.0     0.0   0.0
DONO H2'  O2'
ACCE O6   C6
```

```
ACCE N2
ACCE N7
ACCE O1P  P
ACCE O2P  P
ACCE O2'
ACCE O3'
ACCE O4'
ACCE O5'

RESI YYG            -1.00 ! wybutosine, WBG, YG
GROUP
ATOM N9     NG2R51  0.02 !                 H192
ATOM C8     CG2R53  0.38 !                  |
ATOM H8     HGR52   0.08 !            H191-C19-H193
ATOM N7     NG2R50 -0.68 !                    \
ATOM C5     CG2RC0  0.01 !                    O18
ATOM C6     CG2R63  0.69 !                   /
ATOM O6     OG2D4  -0.51 !    H131 H141 O17=C16  H20  O22 (cis)
ATOM N1     NG2RC0 -0.10 !      |    |      |    |    ||
ATOM C2     CG2RC0  0.45 !  *---C13--C14----C15--N20--C21       H241
ATOM N2     NG2R50 -0.69 !      |    |      |             \     /
ATOM N3     NG2R61 -0.34 !    H132  H142    H15           O23-C24-H242
ATOM C4     CG2RC0  0.30 !                                      \
ATOM C12    CG2R51 -0.26 !                                       H243
ATOM C11    CG2R51  0.41
GROUP                    !                    O6
ATOM C3     CG331  -0.04 !             *      ||
ATOM H31    HGA3    0.09 !             |      C6
ATOM H32    HGA3    0.09 !  H101       |    /  \
ATOM H33    HGA3    0.09 !     \     //C12-N1   C5--N7\\
GROUP                    ! H102-C10-C11    |    ||     C8-H8
ATOM C10    CG331  -0.30 !     /      \N2==C2   C4--N9/
ATOM H101   HGA3    0.09 !  H103            \  /      \
ATOM H102   HGA3    0.09 !                   N3        \
ATOM H103   HGA3    0.09 !                   |          \
GROUP                    !                   C3          \
ATOM C13    CG321  -0.14 !                 / |  \         \
ATOM H131   HGA2    0.09 !             H31  H32  H33       \
ATOM H132   HGA2    0.09 !                                  \
GROUP                    !              O1P    H5' H4'  O4'  \
ATOM C14    CG321  -0.18 !               |      |    \ /   \  \
ATOM H141   HGA2    0.09 !              -P-O5'-C5'---C4'     C1'
ATOM H142   HGA2    0.09 !               |      |     \     / \
GROUP                    !              O2P    H5''   C3'--C2' H1'
ATOM C15    CG311   0.10 !                           / \   / \
ATOM H15    HGA1    0.09 !                         O3' H3' O2' H2''
ATOM N20    NG2S1  -0.38 !                          |       |
ATOM H20    HGP1    0.32 !                                 H2'
ATOM C21    CG2O6   0.20
ATOM O22    OG2D1  -0.39
ATOM O23    OG302  -0.32
ATOM C24    CG331   0.07
ATOM H241   HGA3    0.09
ATOM H242   HGA3    0.09
ATOM H243   HGA3    0.09
GROUP
ATOM C16    CG2O2   0.90
ATOM O17    OG2D1  -0.63
ATOM O18    OG302  -0.49
ATOM C19    CG331  -0.01
ATOM H191   HGA3    0.09
ATOM H192   HGA3    0.09
ATOM H193   HGA3    0.09
```

```
GROUP
ATOM P       P       1.50
ATOM O1P     ON3    -0.78
ATOM O2P     ON3    -0.78
ATOM O5'     ON2    -0.57
ATOM C5'     CN8B   -0.08
ATOM H5'     HN8     0.09
ATOM H5''    HN8     0.09
GROUP
ATOM C4'     CN7     0.16
ATOM H4'     HN7     0.09
ATOM O4'     ON6B   -0.50
ATOM C1'     CN7B    0.16
ATOM H1'     HN7     0.09
GROUP
ATOM C2'     CN7B    0.14
ATOM H2''    HN7     0.09
ATOM O2'     ON5    -0.66
ATOM H2'     HN5     0.43
GROUP
ATOM C3'     CN7     0.01
ATOM H3'     HN7     0.09
ATOM O3'     ON2    -0.57
BOND N9   C8   N9   C4   C8   H8   C8   N7
BOND N7   C5   C5   C6   C5   C4   C6   O6
BOND C6   N1   N1   C2   N1   C12  C2   N2
BOND C2   N3   N2   C11  N3   C4   N3   C3
BOND C12  C11  C12  C13  C11  C10  C3   H31
BOND C3   H32  C3   H33  C10  H101 C10  H102
BOND C10  H103 C13  H131 C13  H132 C13  C14
BOND C14  H141 C14  H142 C14  C15  C15  H15
BOND C15  N20  C15  C16  N20  H20  N20  C21
BOND C21  O22  C21  O23  O23  C24  C24  H241
BOND C24  H242 C24  H243 C16  O17  C16  O18
BOND O18  C19  C19  H191 C19  H192 C19  H193
BOND P    O1P     P    O2P     P    O5'     O5'  C5'     C5'  H5''
BOND C5'  C4'     C4'  O4'     C4'  C3'     O4'  C1'
BOND C1'  N9      C1'  C2'     C2'  C3'     C3'  O3'     O3'  +P
BOND C2'  O2'     O2'  H2'
BOND C1'  H1'     C2'  H2''    C3'  H3'     C4'  H4'     C5'  H5'
IMPR C6     C5     N1     O6     C16    C15    O17    O18    C21    N20    O22    O23
IC C8   C4   *N9   C1'       1.3791  105.54 -179.95  126.56  1.4896
IC C4   N9   C8    N7        1.3989  105.75   -0.09  113.39  1.3178
IC N7   N9   *C8   H8        1.3178  113.39 -179.93  122.02  1.0918
IC N9   C8   N7    C5        1.3728  113.39   -0.02  105.30  1.3903
IC C4   N7   *C5   C6        1.3977  109.72 -179.74  129.59  1.4135
IC N7   C5   C6    N1        1.3903  129.59  179.04  116.17  1.4317
IC N1   C5   *C6   O6        1.4317  116.17 -179.78  124.40  1.2392
IC C5   C6   N1    C12       1.4135  116.17  180.00  122.50  1.3710
IC C12  C6   *N1   C2        1.3710  122.50  180.00  121.22  1.4137
IC C6   N1   C2    N2        1.4317  121.22  179.73  111.33  1.3439
IC N2   N1   *C2   N3        1.3439  111.33  180.00  122.04  1.3925
IC N1   C2   N2    C11       1.4137  111.33  180.00  103.00  1.3800
IC C4   C2   *N3   C3        1.3974  116.18 -179.94    9.38  3.5257
IC C2   N3   C3    H31       1.3925    9.38  180.00  110.10  1.1110
IC H31  N3   *C3   H32       1.1110  110.10  120.00  110.10  1.1110
IC H31  N3   *C3   H33       1.1110  110.10 -120.00  110.10  1.1110
IC C12  N2   *C11  C10       1.3600  110.00  180.00  120.00  1.5000
IC N2   C11  C10   H101      1.3800  120.00  180.00  109.50  1.1091
IC H101 C11  *C10  H102      1.1091  109.50  120.00  109.50  1.1112
IC H101 C11  *C10  H103      1.1091  109.50 -120.00  109.50  1.1047
IC C11  N1   *C12  C13       1.3600  108.20  180.00  126.70  1.5000
IC N1   C12  C13   C14       1.3710  126.70  180.00  114.00  1.5000
```

```
IC C14  C12  *C13  H131      1.5000  114.00  120.00  109.50  1.1135
IC H131 C12  *C13  H132      1.1135  109.50 -120.00  109.50  1.1135
IC C12  C13  C14   C15       1.5000  114.00  180.00  130.28  1.5410
IC C15  C13  *C14  H141      1.5410  130.28 -124.85  101.18  1.1162
IC H141 C13  *C14  H142      1.1162  101.18 -109.55  101.31  1.1147
IC C13  C14  C15   N20       1.5000  130.28  180.00  113.50  1.4300
IC N20  C14  *C15  C16       1.4300  113.50  120.00  113.09  1.5520
IC N20  C14  *C15  H15       1.4300  113.50 -120.00  110.10  1.1110
IC C14  C15  N20   C21       1.5410  113.50  180.00  120.00  1.3700
IC C21  C15  *N20  H20       1.3700  120.00  180.00  117.00  0.9970
IC C15  N20  C21   O23       1.4300  120.00  180.00  110.30  1.3500
IC O23  N20  *C21  O22       1.3500  110.30  180.00  125.70  1.2300
IC N20  C21  O23   C24       1.3700  110.30  180.00  111.00  1.4300
IC C21  O23  C24   H241      1.3500  111.00  180.00  109.50  1.1110
IC H241 O23  *C24  H242      1.1110  109.50  120.00  109.50  1.1110
IC H241 O23  *C24  H243      1.1110  109.50 -120.00  109.50  1.1110
IC C14  C15  C16   O18       1.5410  113.09  -22.55  116.93  2.3853
IC O18  C15  *C16  O17       2.3853  116.93  -55.88   98.98  2.4723
IC C15  C16  O18   C19       1.5520  116.93 -167.73   39.48  3.7474
IC C16  O18  C19   H191      2.3853   39.48  180.00  109.50  1.1110
IC H191 O18  *C19  H192      1.1110  109.50  120.00  109.50  1.1110
IC H191 O18  *C19  H193      1.1110  109.50 -120.00  109.50  1.1110
!ribose
IC -O3' P    O5'   C5'    1.6001  101.45  -39.25  119.00  1.4401
IC -O3' O5'  *P    O1P    1.6001  101.45 -115.82  109.74  1.4802
IC -O3' O5'  *P    O2P    1.6001  101.45  115.90  109.80  1.4801
IC  P   O5'  C5'   C4'    1.5996  119.00 -151.39  110.04  1.5160
IC O5'  C5'  C4'   C3'    1.4401  108.83 -179.85  116.10  1.5284
IC C5'  C4'  C3'   O3'    1.5160  116.10   76.70  115.12  1.4212
IC C4'  C3'  O3'   +P     1.5284  111.92  159.13  119.05  1.6001
IC C3'  O3'  +P    +O5'   1.4212  119.05  -98.86  101.45  1.5996
IC O4'  C3'  *C4'  C5'    1.4572  104.06 -120.04  116.10  1.5160
IC C2'  C4'  *C3'  O3'    1.5284  100.16 -124.08  115.12  1.4212
IC C4'  C3'  C2'   C1'    1.5284  100.16   39.58  102.04  1.5251
IC C3'  C2'  C1'   N9     1.5284  101.97  144.39  113.71  1.4896
IC O4'  C1'  N9    C4     1.5251  113.71  -97.2   125.59  1.3783
IC C1'  C3'  *C2'  O2'    1.5284  102.04 -114.67  110.81  1.4212
IC H2'  O2'  C2'   C3'    0.9600  114.97  148.63  111.92  1.5284
IC O4'  C2'  *C1'  H1'    0.0       0.0  -115.0     0.0   0.0
IC C1'  C3'  *C2'  H2''   0.0       0.0   115.0     0.0   0.0
IC C2'  C4'  *C3'  H3'    0.0       0.0   115.0     0.0   0.0
IC C3'  O4'  *C4'  H4'    0.0       0.0  -115.0     0.0   0.0
IC C4'  O5'  *C5'  H5'    0.0       0.0  -115.0     0.0   0.0
IC C4'  O5'  *C5'  H5''   0.0       0.0   115.0     0.0   0.0
DONO H20  N20
DONO H2'  O2'
ACCE O6   C6
ACCE N2
ACCE N7
ACCE O17  C16
ACCE O18
ACCE O22  C21
ACCE O23
ACCE O1P  P
ACCE O2P  P
ACCE O2'
ACCE O3'
ACCE O4'
ACCE O5'

RESI HWG           -1.00 ! hydroxywybutosine
GROUP
ATOM N9     NG2R51  0.02 !                 H192
```

```
ATOM C8     CG2R53  0.38 !                  |
ATOM H8     HGR52   0.08 !             H191-C19-H193
ATOM N7     NG2R50 -0.68 !                    \
ATOM C5     CG2RC0  0.01 !                    O18
ATOM C6     CG2R63  0.69 !                   /
ATOM O6     OG2D4  -0.51 !    H131  H14 O17=C16  H20  O22 (cis)
ATOM N1     NG2RC0 -0.10 !      |    |      |    |    ||
ATOM C2     CG2RC0  0.45 !  *---C13--C14----C15--N20--C21       H241
ATOM N2     NG2R50 -0.69 !      |    |      |            \     /
ATOM N3     NG2R61 -0.34 !    H132  O14     H15           O23-C24-H242
ATOM C4     CG2RC0  0.30 !            \                        \
ATOM C12    CG2R51 -0.26 !             H14O                     H243
ATOM C11    CG2R51  0.41
GROUP
ATOM C3     CG331  -0.04 !                    O6
ATOM H31    HGA3    0.09 !              *     ||
ATOM H32    HGA3    0.09 !              |     C6
ATOM H33    HGA3    0.09 !   H101       |    /  \
GROUP                    !      \     //C12-N1   C5--N7\\
ATOM C10    CG331  -0.30 !  H102-C10-C11    |    ||     C8-H8
ATOM H101   HGA3    0.09 !      /      \N2==C2   C4--N9/
ATOM H102   HGA3    0.09 !   H103            \  /      \
ATOM H103   HGA3    0.09 !                    N3        \
GROUP                    !                    |          \
ATOM C13    CG321  -0.14 !                    C3          \
ATOM H131   HGA2    0.09 !                  / |  \         \
ATOM H132   HGA2    0.09 !              H31  H32  H33       \
GROUP                    !                                   \
ATOM C14    CG311   0.14 !               O1P    H5' H4'  O4'  \
ATOM H14    HGA1    0.09 !                |      |    \ /   \  \
ATOM O14    OG311  -0.65 !               -P-O5'-C5'---C4'    C1'
ATOM H14O   HGP1    0.42 !                |      |     \     / \
GROUP                    !               O2P    H5''   C3'--C2' H1'
ATOM C15    CG311   0.10 !                            / \   / \
ATOM H15    HGA1    0.09 !                          O3' H3' O2' H2''
ATOM N20    NG2S1  -0.38 !                           |       |
ATOM H20    HGP1    0.32 !                                  H2'
ATOM C21    CG2O6   0.20
ATOM O22    OG2D1  -0.39
ATOM O23    OG302  -0.32
ATOM C24    CG331   0.07
ATOM H241   HGA3    0.09
ATOM H242   HGA3    0.09
ATOM H243   HGA3    0.09
GROUP
ATOM C16    CG2O2   0.90
ATOM O17    OG2D1  -0.63
ATOM O18    OG302  -0.49
ATOM C19    CG331  -0.01
ATOM H191   HGA3    0.09
ATOM H192   HGA3    0.09
ATOM H193   HGA3    0.09
GROUP
ATOM P      P       1.50
ATOM O1P    ON3    -0.78
ATOM O2P    ON3    -0.78
ATOM O5'    ON2    -0.57
ATOM C5'    CN8B   -0.08
ATOM H5'    HN8     0.09
ATOM H5''   HN8     0.09
GROUP
ATOM C4'    CN7     0.16
ATOM H4'    HN7     0.09
```

```
ATOM O4'    ON6B   -0.50
ATOM C1'    CN7B    0.16
ATOM H1'    HN7     0.09
GROUP
ATOM C2'    CN7B    0.14
ATOM H2''   HN7     0.09
ATOM O2'    ON5    -0.66
ATOM H2'    HN5     0.43
GROUP
ATOM C3'    CN7     0.01
ATOM H3'    HN7     0.09
ATOM O3'    ON2    -0.57
BOND N9   C8   N9   C4   C8   H8
BOND C8   N7   N7   C5   C5   C6   C5   C4
BOND C6   O6   C6   N1   N1   C2   N1   C12
BOND C2   N2   C2   N3   N2   C11  N3   C4
BOND N3   C3   C12  C11  C12  C13  C11  C10
BOND C3   H31  C3   H32  C3   H33  C10  H101
BOND C10  H102 C10  H103 C13  H131 C13  H132
BOND C13  C14  C14  H14  C14  O14  C14  C15
BOND O14  H14O C15  H15  C15  N20  C15  C16
BOND N20  H20  N20  C21  C21  O22  C21  O23
BOND O23  C24  C24  H241 C24  H242 C24  H243
BOND C16  O17  C16  O18  O18  C19  C19  H191
BOND C19  H192 C19  H193
BOND P    O1P     P    O2P     P    O5'     O5'  C5'     C5'  H5''
BOND C5'  C4'     C4'  O4'     C4'  C3'     O4'  C1'
BOND C1'  N9      C1'  C2'     C2'  C3'     C3'  O3'     O3'  +P
BOND C2'  O2'     O2'  H2'
BOND C1'  H1'     C2'  H2''    C3'  H3'     C4'  H4'     C5'  H5'
IMPR C6     C5     N1     O6     C16    C15    O17    O18    C21    N20    O22    O23
IC C8   C4   *N9   C1'       1.3791  105.54 -179.95  126.56  1.4896
IC C4   N9   C8    N7        1.3649  106.94    0.04  112.16  1.3171
IC N7   N9   *C8   H8        1.3171  112.16 -179.99  123.37  1.0930
IC N9   C8   N7    C5        1.3688  112.16   -0.00  105.34  1.3924
IC C4   N7   *C5   C6        1.3865  109.17 -179.96  130.72  1.4130
IC N7   C5   C6    N1        1.3924  130.72  179.64  115.87  1.4377
IC N1   C5   *C6   O6        1.4377  115.87  179.47  124.67  1.2399
IC C5   C6   N1    C12       1.4130  115.87  180.00  122.50  1.3710
IC C12  C6   *N1   C2        1.3710  122.50  180.00  121.04  1.4186
IC C6   N1   C2    N2        1.4377  121.04  177.77  111.49  1.3424
IC N2   N1   *C2   N3        1.3424  111.49 -179.84  121.87  1.3901
IC N1   C2   N2    C11       1.4186  111.49  180.00  103.00  1.3800
IC C4   C2   *N3   C3        1.3779  115.73  179.95    9.27  3.5219
IC C2   N3   C3    H31       1.3901    9.27  180.00  110.10  1.1110
IC H31  N3   *C3   H32       1.1110  110.10  120.00  110.10  1.1110
IC H31  N3   *C3   H33       1.1110  110.10 -120.00  110.10  1.1110
IC C12  N2   *C11  C10       1.3600  110.00  180.00  120.00  1.5000
IC N2   C11  C10   H101      1.3800  120.00  180.00  109.50  1.1095
IC H101 C11  *C10  H102      1.1095  109.50  120.00  109.50  1.1101
IC H101 C11  *C10  H103      1.1095  109.50 -120.00  109.50  1.1034
IC C11  N1   *C12  C13       1.3600  108.20  180.00  126.70  1.5000
IC N1   C12  C13   C14       1.3710  126.70  180.00  114.00  1.5000
IC C14  C12  *C13  H131      1.5000  114.00  120.00  109.50  1.1129
IC H131 C12  *C13  H132      1.1129  109.50 -120.00  109.50  1.1142
IC C12  C13  C14   C15       1.5000  114.00  180.00  133.23  1.5596
IC C15  C13  *C14  O14       1.5596  133.23  120.00  110.00  1.4200
IC O14  C13  *C14  H14       1.4200  110.00 -120.00  110.10  1.1110
IC C13  C14  O14   H14O      1.5000  110.00  180.00  106.00  0.9600
IC C13  C14  C15   N20       1.5000  133.23  180.00  113.50  1.4300
IC N20  C14  *C15  C16       1.4300  113.50  120.00  111.89  1.5186
IC N20  C14  *C15  H15       1.4300  113.50 -120.00  108.70  1.1150
IC C14  C15  N20   C21       1.5596  113.50  180.00  120.00  1.3700
```

```
IC C21  C15  *N20  H20       1.3700  120.00  180.00  117.00  0.9970
IC C15  N20  C21   O23       1.4300  120.00  180.00  110.30  1.3500
IC O23  N20  *C21  O22       1.3500  110.30  180.00  125.70  1.2300
IC N20  C21  O23   C24       1.3700  110.30  180.00  111.00  1.4300
IC C21  O23  C24   H241      1.3500  111.00  180.00  109.50  1.1110
IC H241 O23  *C24  H242      1.1110  109.50  120.00  109.50  1.1110
IC H241 O23  *C24  H243      1.1110  109.50 -120.00  109.50  1.1110
IC C14  C15  C16   O18       1.5596  111.89  -21.83  115.41  2.3726
IC O18  C15  *C16  O17       2.3726  115.41  -54.76   95.09  2.4421
IC C15  C16  O18   C19       1.5186  115.41 -169.80   39.27  3.7592
IC C16  O18  C19   H191      2.3726   39.27  180.00  109.50  1.1110
IC H191 O18  *C19  H192      1.1110  109.50  120.00  109.50  1.1110
IC H191 O18  *C19  H193      1.1110  109.50 -120.00  109.50  1.1110
!ribose
IC -O3' P    O5'   C5'    1.6001  101.45  -39.25  119.00  1.4401
IC -O3' O5'  *P    O1P    1.6001  101.45 -115.82  109.74  1.4802
IC -O3' O5'  *P    O2P    1.6001  101.45  115.90  109.80  1.4801
IC  P   O5'  C5'   C4'    1.5996  119.00 -151.39  110.04  1.5160
IC O5'  C5'  C4'   C3'    1.4401  108.83 -179.85  116.10  1.5284
IC C5'  C4'  C3'   O3'    1.5160  116.10   76.70  115.12  1.4212
IC C4'  C3'  O3'   +P     1.5284  111.92  159.13  119.05  1.6001
IC C3'  O3'  +P    +O5'   1.4212  119.05  -98.86  101.45  1.5996
IC O4'  C3'  *C4'  C5'    1.4572  104.06 -120.04  116.10  1.5160
IC C2'  C4'  *C3'  O3'    1.5284  100.16 -124.08  115.12  1.4212
IC C4'  C3'  C2'   C1'    1.5284  100.16   39.58  102.04  1.5251
IC C3'  C2'  C1'   N9     1.5284  101.97  144.39  113.71  1.4896
IC O4'  C1'  N9    C4     1.5251  113.71  -97.2   125.59  1.3783
IC C1'  C3'  *C2'  O2'    1.5284  102.04 -114.67  110.81  1.4212
IC H2'  O2'  C2'   C3'    0.9600  114.97  148.63  111.92  1.5284
IC O4'  C2'  *C1'  H1'    0.0       0.0  -115.0     0.0   0.0
IC C1'  C3'  *C2'  H2''   0.0       0.0   115.0     0.0   0.0
IC C2'  C4'  *C3'  H3'    0.0       0.0   115.0     0.0   0.0
IC C3'  O4'  *C4'  H4'    0.0       0.0  -115.0     0.0   0.0
IC C4'  O5'  *C5'  H5'    0.0       0.0  -115.0     0.0   0.0
IC C4'  O5'  *C5'  H5''   0.0       0.0   115.0     0.0   0.0
DONO H2'  O2'
DONO H20  N20
DONO H14O O14
ACCE O6   C6
ACCE N2
ACCE N7
ACCE O14
ACCE O17  C16
ACCE O18
ACCE O22  C21
ACCE O23
ACCE O1P  P
ACCE O2P  P
ACCE O2'
ACCE O3'
ACCE O4'
ACCE O5'

RESI PBG           -1.00 ! peroxywybutosine
GROUP
ATOM N9     NG2R51  0.02 !                H192
ATOM C8     CG2R53  0.38 !                 |
ATOM H8     HGR52   0.08 !            H191-C19-H193
ATOM N7     NG2R50 -0.68 !                   \
ATOM C5     CG2RC0  0.01 !                   O18
ATOM C6     CG2R63  0.69 !                  /
ATOM O6     OG2D4  -0.51 !   H131  H14 O17=C17  H20  O22 (cis)
ATOM N1     NG2RC0 -0.10 !     |    |      |    |    ||
```

```
ATOM C2     CG2RC0  0.45 ! *---C13--C14----C16--N20--C21       H241
ATOM N2     NG2R50 -0.69 !     |    |      |            \     /
ATOM N3     NG2R61 -0.34 !   H132  O14     H16           O23-C24-H242
ATOM C4     CG2RC0  0.30 !           \                        \
ATOM C12    CG2R51 -0.26 !            O15-H15O                 H243
ATOM C11    CG2R51  0.41
GROUP
ATOM C3     CG331  -0.04 !                   O6
ATOM H31    HGA3    0.09 !             *     ||
ATOM H32    HGA3    0.09 !             |     C6
ATOM H33    HGA3    0.09 !  H101       |    /  \
GROUP                    !     \     //C12-N1   C5--N7\\
ATOM C10    CG331  -0.30 ! H102-C10-C11    |    ||     C8-H8
ATOM H101   HGA3    0.09 !     /      \N2==C2   C4--N9/
ATOM H102   HGA3    0.09 !  H103            \  /      \
ATOM H103   HGA3    0.09 !                   N3        \
GROUP                    !                   |          \
ATOM C13    CG321  -0.14 !                   C3          \
ATOM H131   HGA2    0.09 !                 / |  \         \
ATOM H132   HGA2    0.09 !             H31  H32  H33       \
GROUP                    !                                  \
ATOM C14    CG311   0.17 !              O1P    H5' H4'  O4'  \
ATOM H14    HGA1    0.09 !               |      |    \ /   \  \
ATOM O14    OG301  -0.25 !              -P-O5'-C5'---C4'    C1'
ATOM O15    OG311  -0.44 !               |      |     \     / \
ATOM H15O   HGP1    0.43 !              O2P    H5''   C3'--C2' H1'
GROUP                    !                           / \   / \
ATOM C16    CG311   0.10 !                         O3' H3' O2' H2''
ATOM H16    HGA1    0.09 !                          |       |
ATOM N20    NG2S1  -0.38 !                                 H2'
ATOM H20    HGP1    0.32
ATOM C21    CG2O6   0.20
ATOM O22    OG2D1  -0.39
ATOM O23    OG302  -0.32
ATOM C24    CG331   0.07
ATOM H241   HGA3    0.09
ATOM H242   HGA3    0.09
ATOM H243   HGA3    0.09
GROUP
ATOM C17    CG2O2   0.90
ATOM O17    OG2D1  -0.63
ATOM O18    OG302  -0.49
ATOM C19    CG331  -0.01
ATOM H191   HGA3    0.09
ATOM H192   HGA3    0.09
ATOM H193   HGA3    0.09
GROUP
ATOM P      P       1.50
ATOM O1P    ON3    -0.78
ATOM O2P    ON3    -0.78
ATOM O5'    ON2    -0.57
ATOM C5'    CN8B   -0.08
ATOM H5'    HN8     0.09
ATOM H5''   HN8     0.09
GROUP
ATOM C4'    CN7     0.16
ATOM H4'    HN7     0.09
ATOM O4'    ON6B   -0.50
ATOM C1'    CN7B    0.16
ATOM H1'    HN7     0.09
GROUP
ATOM C2'    CN7B    0.14
ATOM H2''   HN7     0.09
```

```
ATOM O2'    ON5    -0.66
ATOM H2'    HN5     0.43
GROUP
ATOM C3'    CN7     0.01
ATOM H3'    HN7     0.09
ATOM O3'    ON2    -0.57
BOND N9   C8   N9   C4   C8   H8   C8   N7
BOND N7   C5   C5   C6   C5   C4   C6   O6
BOND C6   N1   N1   C2   N1   C12  C2   N2
BOND C2   N3   N2   C11  N3   C4   N3   C3
BOND C12  C11  C12  C13  C11  C10  C3   H31
BOND C3   H32  C3   H33  C10  H101 C10  H102
BOND C10  H103 C13  H131 C13  H132 C13  C14
BOND O15  H15O O15  O14  O14  C14  C14  H14
BOND C14  C16  C16  H16  C16  N20  C16  C17
BOND N20  H20  N20  C21  C21  O22  C21  O23
BOND O23  C24  C24  H241 C24  H242 C24  H243
BOND C17  O17  C17  O18  O18  C19  C19  H191
BOND C19  H192 C19  H193
BOND P    O1P     P    O2P     P    O5'     O5'  C5'     C5'  H5''
BOND C5'  C4'     C4'  O4'     C4'  C3'     O4'  C1'
BOND C1'  N9      C1'  C2'     C2'  C3'     C3'  O3'     O3'  +P
BOND C2'  O2'     O2'  H2'
BOND C1'  H1'     C2'  H2''    C3'  H3'     C4'  H4'     C5'  H5'
IMPR C6     C5     N1     O6     C17    C16    O17    O18    C21    N20    O22    O23
!ribose
IC -O3' P    O5'   C5'    1.6001  101.45  -39.25  119.00  1.4401
IC -O3' O5'  *P    O1P    1.6001  101.45 -115.82  109.74  1.4802
IC -O3' O5'  *P    O2P    1.6001  101.45  115.90  109.80  1.4801
IC  P   O5'  C5'   C4'    1.5996  119.00 -151.39  110.04  1.5160
IC O5'  C5'  C4'   C3'    1.4401  108.83 -179.85  116.10  1.5284
IC C5'  C4'  C3'   O3'    1.5160  116.10   76.70  115.12  1.4212
IC C4'  C3'  O3'   +P     1.5284  111.92  159.13  119.05  1.6001
IC C3'  O3'  +P    +O5'   1.4212  119.05  -98.86  101.45  1.5996
IC O4'  C3'  *C4'  C5'    1.4572  104.06 -120.04  116.10  1.5160
IC C2'  C4'  *C3'  O3'    1.5284  100.16 -124.08  115.12  1.4212
IC C4'  C3'  C2'   C1'    1.5284  100.16   39.58  102.04  1.5251
IC C3'  C2'  C1'   N9     1.5284  101.97  144.39  113.71  1.4896
IC O4'  C1'  N9    C4     1.5251  113.71  -97.2   125.59  1.3783
IC C1'  C3'  *C2'  O2'    1.5284  102.04 -114.67  110.81  1.4212
IC H2'  O2'  C2'   C3'    0.9600  114.97  148.63  111.92  1.5284
IC O4'  C2'  *C1'  H1'    0.0       0.0  -115.0     0.0   0.0
IC C1'  C3'  *C2'  H2''   0.0       0.0   115.0     0.0   0.0
IC C2'  C4'  *C3'  H3'    0.0       0.0   115.0     0.0   0.0
IC C3'  O4'  *C4'  H4'    0.0       0.0  -115.0     0.0   0.0
IC C4'  O5'  *C5'  H5'    0.0       0.0  -115.0     0.0   0.0
IC C4'  O5'  *C5'  H5''   0.0       0.0   115.0     0.0   0.0
IC C8   C4   *N9   C1'       1.3791  105.54 -179.95  126.56  1.4896
IC C4   N9   C8    N7        1.3992  105.68   -0.15  113.39  1.3174
IC N7   N9   *C8   H8        1.3174  113.39 -179.96  121.98  1.0919
IC N9   C8   N7    C5        1.3736  113.39   -0.07  105.30  1.3905
IC C4   N7   *C5   C6        1.3961  109.73 -179.35  129.57  1.4143
IC N7   C5   C6    N1        1.3905  129.57  177.36  116.16  1.4313
IC N1   C5   *C6   O6        1.4313  116.16 -179.62  124.45  1.2393
IC C5   C6   N1    C12       1.4143  116.16  180.00  122.50  1.3710
IC C12  C6   *N1   C2        1.3710  122.50  180.00  121.11  1.4141
IC C6   N1   C2    N2        1.4313  121.11  178.12  111.30  1.3439
IC N2   N1   *C2   N3        1.3439  111.30 -179.80  122.12  1.3923
IC N1   C2   N2    C11       1.4141  111.30  180.00  103.00  1.3800
IC C4   C2   *N3   C3        1.3975  116.09 -179.15    9.41  3.5248
IC C2   N3   C3    H31       1.3923    9.41  180.00  110.10  1.1110
IC H31  N3   *C3   H32       1.1110  110.10  120.00  110.10  1.1110
IC H31  N3   *C3   H33       1.1110  110.10 -120.00  110.10  1.1110
```

```
IC C12  N2   *C11  C10       1.3600  110.00  180.00  120.00  1.5000
IC N2   C11  C10   H101      1.3800  120.00  180.00  109.50  1.1094
IC H101 C11  *C10  H102      1.1094  109.50  120.00  109.50  1.1103
IC H101 C11  *C10  H103      1.1094  109.50 -120.00  109.50  1.1042
IC C11  N1   *C12  C13       1.3600  108.20  180.00  126.70  1.5000
IC N1   C12  C13   C14       1.3710  126.70  180.00  114.00  1.5000
IC C14  C12  *C13  H131      6.1865  114.00  120.00  109.50  1.1144
IC H131 C12  *C13  H132      1.1144  109.50 -120.00  109.50  1.1144
IC C12  C13  C14   C16       1.5000  114.00  180.00  165.19  2.5406
IC C16  C13  *C14  O14       2.5406  165.19  120.00  109.70  1.4150
IC O14  C13  *C14  H14       1.4150  109.70 -120.00  110.10  1.1110
IC C13  C14  O14   O15       6.1865  109.70  180.00  104.00  1.4610
IC C14  O14  O15   H15O      1.4150  104.00  180.00   98.30  0.9600
IC C13  C14  C16   N20       6.1865  165.19  180.00  113.50  1.4300
IC N20  C14  *C16  C17       1.4300  113.50  120.00   85.18  1.5422
IC N20  C14  *C16  H16       1.4300  113.50 -120.00   88.83  1.1119
IC C14  C16  N20   C21       2.5406  113.50  180.00  120.00  1.3700
IC C21  C16  *N20  H20       1.3700  120.00  180.00  117.00  0.9970
IC C16  N20  C21   O23       1.4300  120.00  180.00  110.30  1.3500
IC O23  N20  *C21  O22       1.3500  110.30  180.00  125.70  1.2300
IC N20  C21  O23   C24       1.3700  110.30  180.00  111.00  1.4300
IC C21  O23  C24   H241      1.3500  111.00  180.00  109.50  1.1110
IC H241 O23  *C24  H242      1.1110  109.50  120.00  109.50  1.1110
IC H241 O23  *C24  H243      1.1110  109.50 -120.00  109.50  1.1110
IC C14  C16  C17   O18       2.5406   85.18   87.36  110.56  1.3399
IC O18  C16  *C17  O17       1.3399  110.56 -175.19  124.88  1.2220
IC C16  C17  O18   C19       1.5422  110.56   56.29   53.62  3.6305
IC C17  O18  C19   H191      1.3399   53.62  180.00  109.50  1.1110
IC H191 O18  *C19  H192      1.1110  109.50  120.00  109.50  1.1110
IC H191 O18  *C19  H193      1.1110  109.50 -120.00  109.50  1.1110
DONO H2'  O2'
DONO H20  N20
DONO H15O O15
ACCE O6   C6
ACCE N2
ACCE N7
ACCE O14
ACCE O15
ACCE O17  C16
ACCE O18
ACCE O22  C21
ACCE O23
ACCE O1P  P
ACCE O2P  P
ACCE O2'
ACCE O3'
ACCE O4'
ACCE O5'

RESI BUG           -1.00 ! undermodified hydroxywybutosine
GROUP
ATOM N9     NG2R51  0.02 !   H131  H14   H15    O18 (-)
ATOM C8     CG2R53  0.38 !     |    |    |     /
ATOM H8     HGR52   0.08 ! *---C13--C14--C15--C17
ATOM N7     NG2R50 -0.68 !     |    |    |     \\
ATOM C5     CG2RC0  0.01 !   H132  O14   | (+)  O17
ATOM C6     CG2R63  0.69 !         /    N16
ATOM O6     OG2D4  -0.51 !      H14O   / | \
ATOM N1     NG2RC0 -0.10 !         H161 H162 H163
ATOM C2     CG2RC0  0.45
ATOM N2     NG2R50 -0.69 !                   O6
ATOM N3     NG2R61 -0.34 !              *     ||
ATOM C4     CG2RC0  0.30 !              |     C6
```

```
ATOM C12    CG2R51 -0.26 !  H101        |    /  \
ATOM C11    CG2R51  0.41 !      \      //C12-N1   C5--N7\\
GROUP                    ! H102-C10-C11    |    ||      C8-H8
ATOM C3     CG331  -0.04 !      /      \N2==C2   C4--N9/
ATOM H31    HGA3    0.09 !  H103            \\ /       \
ATOM H32    HGA3    0.09 !                   N3         \
ATOM H33    HGA3    0.09 !                   |           \
GROUP                    !                   C3           \
ATOM C10    CG331  -0.30 !                 / |  \          \
ATOM H101   HGA3    0.09 !             H31  H32  H33        \
ATOM H102   HGA3    0.09 !                                   \
ATOM H103   HGA3    0.09 !              O1P    H5' H4'  O4'  \
GROUP                    !               |      |    \ /   \  \
ATOM C13    CG321  -0.14 !              -P-O5'-C5'---C4'    C1'
ATOM H131   HGA2    0.09 !               |      |     \     / \
ATOM H132   HGA2    0.09 !              O2P    H5''   C3'--C2' H1'
GROUP                    !                           / \   / \
ATOM C14    CG311   0.14 !                         O3' H3' O2' H2''
ATOM H14    HGA1    0.09 !                          |       |
ATOM O14    OG311  -0.65 !                                 H2'
ATOM H14O   HGP1    0.42
GROUP
ATOM C15    CG314   0.17
ATOM H15    HGA1    0.11
ATOM N16    NG3P3  -0.34
ATOM H161   HGP2    0.30
ATOM H162   HGP2    0.30
ATOM H163   HGP2    0.30
ATOM C17    CG2O3   0.32
ATOM O18    OG2D2  -0.58
ATOM O17    OG2D2  -0.58
GROUP
ATOM P      P       1.50
ATOM O1P    ON3    -0.78
ATOM O2P    ON3    -0.78
ATOM O5'    ON2    -0.57
ATOM C5'    CN8B   -0.08
ATOM H5'    HN8     0.09
ATOM H5''   HN8     0.09
GROUP
ATOM C4'    CN7     0.16
ATOM H4'    HN7     0.09
ATOM O4'    ON6B   -0.50
ATOM C1'    CN7B    0.16
ATOM H1'    HN7     0.09
GROUP
ATOM C2'    CN7B    0.14
ATOM H2''   HN7     0.09
ATOM O2'    ON5    -0.66
ATOM H2'    HN5     0.43
GROUP
ATOM C3'    CN7     0.01
ATOM H3'    HN7     0.09
ATOM O3'    ON2    -0.57
BOND N9   C8   N9   C4   C8   H8   C8   N7
BOND N7   C5   C5   C6   C5   C4   C6   O6
BOND C6   N1   N1   C2   N1   C12  C2   N2
BOND C2   N3   N2   C11  N3   C4   N3   C3
BOND C12  C11  C12  C13  C11  C10  C3   H31
BOND C3   H32  C3   H33  C10  H101 C10  H102
BOND C10  H103 C13  H131 C13  H132 C13  C14
BOND C14  H14  C14  O14  C14  C15  O14  H14O
BOND C15  H15  C15  C17  C15  N16  C17  O18
```

```
BOND C17  O17  N16  H161 N16  H162 N16  H163
BOND P    O1P     P    O2P     P    O5'     O5'  C5'     C5'  H5''
BOND C5'  C4'     C4'  O4'     C4'  C3'     O4'  C1'
BOND C1'  N9      C1'  C2'     C2'  C3'     C3'  O3'     O3'  +P
BOND C2'  O2'     O2'  H2'
BOND C1'  H1'     C2'  H2''    C3'  H3'     C4'  H4'     C5'  H5'
IMPR C6     C5     N1     O6      C17    O17    O18    C15
IC C8   C4   *N9   C1'       1.3791  105.54 -179.95  126.56  1.4896
IC C4   N9   C8    N7        1.3988  105.71    0.07  113.41  1.3182
IC N7   N9   *C8   H8        1.3182  113.41  179.97  121.89  1.0915
IC N9   C8   N7    C5        1.3744  113.41    0.02  105.25  1.3902
IC C4   N7   *C5   C6        1.3983  109.79 -179.97  129.52  1.4121
IC N7   C5   C6    N1        1.3902  129.52  179.08  116.02  1.4306
IC N1   C5   *C6   O6        1.4306  116.02  179.50  124.69  1.2422
IC C5   C6   N1    C12       1.4121  116.02  180.00  122.50  1.3710
IC C12  C6   *N1   C2        1.3710  122.50  180.00  121.54  1.4116
IC C6   N1   C2    N2        1.4306  121.54  177.03  111.22  1.3430
IC N2   N1   *C2   N3        1.3430  111.22  179.50  121.78  1.3931
IC N1   C2   N2    C11       1.4116  111.22  180.00  103.00  1.3800
IC C4   C2   *N3   C3        1.3981  116.22 -177.37    9.18  3.5295
IC C2   N3   C3    H31       1.3931    9.18  180.00  110.10  1.1110
IC H31  N3   *C3   H32       1.1110  110.10  120.00  110.10  1.1110
IC H31  N3   *C3   H33       1.1110  110.10 -120.00  110.10  1.1110
IC C12  N2   *C11  C10       1.3600  110.00  180.00  120.00  1.5000
IC N2   C11  C10   H101      1.3800  120.00  180.00  109.50  1.1094
IC H101 C11  *C10  H102      1.1094  109.50  120.00  109.50  1.1093
IC H101 C11  *C10  H103      1.1094  109.50 -120.00  109.50  1.1055
IC C11  N1   *C12  C13       1.3600  108.20  180.00  126.70  1.5000
IC N1   C12  C13   C14       1.3710  126.70  180.00  114.00  1.5000
IC C14  C12  *C13  H131      1.5000  114.00  120.00  109.50  1.1130
IC H131 C12  *C13  H132      1.1130  109.50 -120.00  109.50  1.1137
IC C12  C13  C14   C15       1.5000  114.00  180.00  133.64  1.5617
IC C15  C13  *C14  O14       1.5617  133.64  120.00  110.00  1.4200
IC O14  C13  *C14  H14       1.4200  110.00 -120.00  110.10  1.1110
IC C13  C14  O14   H14O      6.1749  110.00  180.00  106.00  0.9600
IC C13  C14  C15   C17       6.1749  133.64  177.61  146.03  2.5046
IC C17  C14  *C15  N16       2.5046  146.03  120.00  110.00  1.4800
IC C17  C14  *C15  H15       2.5046  146.03 -109.64  108.35  1.1156
IC C14  C15  C17   O18       1.5617  146.03  180.00  116.00  1.2600
IC O18  C15  *C17  O17       1.2600  116.00  180.00  116.00  1.2600
IC C14  C15  N16   H161      1.5617  110.00  180.00  109.50  1.0400
IC H161 C15  *N16  H162      1.0400  109.50  120.00  109.50  1.0400
IC H161 C15  *N16  H163      1.0400  109.50 -120.00  109.50  1.0400
!ribose
IC -O3' P    O5'   C5'    1.6001  101.45  -39.25  119.00  1.4401
IC -O3' O5'  *P    O1P    1.6001  101.45 -115.82  109.74  1.4802
IC -O3' O5'  *P    O2P    1.6001  101.45  115.90  109.80  1.4801
IC  P   O5'  C5'   C4'    1.5996  119.00 -151.39  110.04  1.5160
IC O5'  C5'  C4'   C3'    1.4401  108.83 -179.85  116.10  1.5284
IC C5'  C4'  C3'   O3'    1.5160  116.10   76.70  115.12  1.4212
IC C4'  C3'  O3'   +P     1.5284  111.92  159.13  119.05  1.6001
IC C3'  O3'  +P    +O5'   1.4212  119.05  -98.86  101.45  1.5996
IC O4'  C3'  *C4'  C5'    1.4572  104.06 -120.04  116.10  1.5160
IC C2'  C4'  *C3'  O3'    1.5284  100.16 -124.08  115.12  1.4212
IC C4'  C3'  C2'   C1'    1.5284  100.16   39.58  102.04  1.5251
IC C3'  C2'  C1'   N9     1.5284  101.97  144.39  113.71  1.4896
IC O4'  C1'  N9    C4     1.5251  113.71  -97.2   125.59  1.3783
IC C1'  C3'  *C2'  O2'    1.5284  102.04 -114.67  110.81  1.4212
IC H2'  O2'  C2'   C3'    0.9600  114.97  148.63  111.92  1.5284
IC O4'  C2'  *C1'  H1'    0.0       0.0  -115.0     0.0   0.0
IC C1'  C3'  *C2'  H2''   0.0       0.0   115.0     0.0   0.0
IC C2'  C4'  *C3'  H3'    0.0       0.0   115.0     0.0   0.0
IC C3'  O4'  *C4'  H4'    0.0       0.0  -115.0     0.0   0.0
```

```
IC C4'  O5'  *C5'  H5'    0.0       0.0  -115.0     0.0   0.0
IC C4'  O5'  *C5'  H5''   0.0       0.0   115.0     0.0   0.0
DONO H2'  O2'
DONO H14O O14
DONO H161 N16
DONO H162 N16
DONO H162 N16
ACCE O14
ACCE O17 C17
ACCE O18 C17
ACCE O6   C6
ACCE N2
ACCE N7
ACCE O1P  P
ACCE O2P  P
ACCE O2'
ACCE O3'
ACCE O4'
ACCE O5'

RESI BMQG           1.00 ! b-mannosyl-queuosine
GROUP
ATOM N9     NG2R51  0.04 !             O24-H24O
ATOM C8     CG2R51  0.06 !              |
ATOM H8     HGR52   0.08 !         H231-C23-H232
ATOM C7     CG2R51 -0.14 !              |
ATOM C5     CG2RC0 -0.06 !         H211-C21--O22
ATOM C6     CG2R63  0.42 !       H20   /       \    O17---*
ATOM O6     OG2D4  -0.52 !          \ /H19O H18O\  /
ATOM N1     NG2R61 -0.38 !          C20 |    |   C17
ATOM H1     HGP1    0.30 !          / \ O19 O18 /  \
ATOM C2     CG2R64  0.65 !  H20O-O20   \|    | /    H17
ATOM N2     NG2S3  -0.60 !              C19--C18
ATOM H21    HGP4    0.30 !              |    |
ATOM H22    HGP4    0.30 !              H19  H18
ATOM N3     NG2R62 -0.77
ATOM C4     CG2RC0  0.32 !                        *
GROUP                    !                H13   /
ATOM C10    CG324   0.18 !                  \  /
ATOM H101   HGA2    0.09 !    H101 H111 H12  C13     H14
ATOM H102   HGA2    0.09 !       |   |    \ /    \  /
ATOM N11    NG3P2  -0.35 !  >---C10--N11--C12     C14
ATOM H111   HGP2    0.28 !       |   |(+)  \      / \
ATOM H112   HGP2    0.28 !    H102 H112    C16==C15  O14-H14O
ATOM C12    CG3C53  0.34 !                 /      \
ATOM H12    HGA1    0.09 !                H16     H15
GROUP
ATOM C15    CG2R51 -0.20 !            O6
ATOM H15    HGR51   0.26 !            ||         >
ATOM C16    CG2R51 -0.24 !            C6        /
ATOM H16    HGR51   0.18 !           /  \      /
GROUP                    !       H1-N1   C5--C7\\
ATOM C14    CG3C51  0.14 !          |    ||     C8-H8
ATOM H14    HGA1    0.09 !          C2   C4--N9/
ATOM O14    OG311  -0.65 !         / \\ /      \
ATOM H14O   HGP1    0.42 !   H21-N2   N3        \
GROUP                    !       |               \
ATOM C13    CG3C51  0.14 !      H22               \
ATOM H13    HGA1    0.09 !                         \
ATOM O17    OG301  -0.41
ATOM C17    CC3162  0.29
ATOM H17    HCA1    0.09
ATOM C21    CC3163  0.11
```

```
ATOM H211   HCA1    0.09
ATOM O22    OC3C61 -0.40
GROUP
ATOM C18    CC3161  0.14
ATOM H18    HCA1    0.09
ATOM O18    OC311  -0.65
ATOM H18O   HCP1    0.42 !  !!!! PATCH 7GNM for the neutral amino form
GROUP
ATOM C19    CC3161  0.14
ATOM H19    HCA1    0.09
ATOM O19    OC311  -0.65
ATOM H19O   HCP1    0.42
GROUP
ATOM C20    CC3161  0.14
ATOM H20    HCA1    0.09
ATOM O20    OC311  -0.65
ATOM H20O   HCP1    0.42
GROUP
ATOM C23    CC321   0.05
ATOM H231   HCA2    0.09
ATOM H232   HCA2    0.09
ATOM O24    OC311  -0.65
ATOM H24O   HCP1    0.42
GROUP
ATOM CM     CG331  -0.27
ATOM HM1    HGA3    0.09
ATOM HM2    HGA3    0.09
ATOM HM3    HGA3    0.09
BOND CM   HM1    CM   HM2   CM   HM3
BOND N9   C8   N9   C4   C8   H8   C8   C7
BOND C7   C5   C7   C10  C5   C6   C5   C4
BOND C6   O6   C6   N1   N1   H1   N1   C2
BOND C2   N2   C2   N3   N2   H21  N2   H22
BOND N3   C4   C10  H101 C10  H102 C10  N11
BOND N11  H111 N11  H112 N11  C12  C12  H12
BOND C12  C16  C12  C13  C14  O14  C14  H14
BOND C14  C15  C14  C13  O14  H14O C15  H15
BOND C15  C16  C16  H16  O22  C17  O22  C21
BOND C17  H17  C17  O17  C17  C18  O17  C13
BOND C13  H13  C21  H211 C21  C20  C21  C23
BOND C18  O18  C18  H18  C18  C19  O18  H18O
BOND C19  O19  C19  H19  C19  C20  O19  H19O
BOND C20  O20  C20  H20  O20  H20O C23  O24
BOND C23  H231 C23  H232 O24  H24O
BOND CM   N9
IMPR C6     C5     N1     O6     C2     N1     N3     N2     N2     H22    H21    C2
DONO H21   N2
DONO H22   N2
DONO H1   N1
DONO H111  N11
DONO H112  N11
DONO H14O  O14
DONO H18O  O18
DONO H19O  O19
DONO H20O  O20
DONO H24O  O24
ACCE O6   C6
ACCE N3
ACCE O14
ACCE O17
ACCE O18
ACCE O19
ACCE O20
```

```
ACCE O22
ACCE O24
IC C4   C8   *N9   CM        1.3819  110.09 -178.94  125.36  1.4668
IC C4   N9   C8    C7        1.3804  110.22    0.33  108.83  1.3705
IC C7   N9   *C8   H8        1.3705  108.83  177.88  121.20  1.0827
IC N9   C8   C7    C10       1.3857  108.83  171.91  124.51  1.4918
IC C10  C8   *C7   C5        1.4918  124.51 -173.06  106.47  1.4471
IC C4   C7   *C5   C6        1.4024  107.98  179.52  133.30  1.4095
IC C7   C5   C6    N1        1.4471  133.30 -178.59  113.57  1.3792
IC N1   C5   *C6   O6        1.3792  113.57 -178.96  127.85  1.2328
IC C5   C6   N1    C2        1.4095  113.57   -0.15  125.13  1.3724
IC C2   C6   *N1   H1        1.3724  125.13  179.13  117.24  0.9994
IC C6   N1   C2    N2        1.3792  125.13  179.67  117.42  1.3220
IC N2   N1   *C2   N3        1.3220  117.42  179.26  121.27  1.3341
IC N1   C2   N2    H21       1.3724  117.42   -0.63  124.15  0.9931
IC H21  C2   *N2   H22       0.9931  124.15 -179.66  115.88  0.9962
IC C8   C7   C10   N11       1.3705  124.51  146.46  111.32  1.5028
IC N11  C7   *C10  H101      1.5028  111.32 -118.54  112.04  1.1040
IC H101 C7   *C10  H102      1.1040  112.04 -124.45  110.74  1.1048
IC C7   C10  N11   C12       1.4918  111.32 -177.34  118.72  1.4938
IC C12  C10  *N11  H111      1.4938  118.72 -120.20  106.30  1.0186
IC H111 C10  *N11  H112      1.0186  106.30 -114.13  110.59  1.0049
IC C10  N11  C12   C13       1.5028  118.72 -150.63  110.11  1.5129
IC C13  N11  *C12  C16       1.5129  110.11 -114.84  109.73  1.5008
IC C16  N11  *C12  H12       1.5008  109.73 -125.00  108.86  1.0869
IC N11  C12  C16   C15       1.4938  109.73  133.12  109.91  1.3762
IC C15  C12  *C16  H16       1.3762  109.91  173.38  124.56  1.0836
IC C12  C16  C15   C14       1.5008  109.91    0.44  111.04  1.5145
IC C14  C16  *C15  H15       1.5145  111.04  169.67  127.30  1.0840
IC C13  C15  *C14  O14       1.5431  102.73  122.98  110.44  1.4180
IC O14  C15  *C14  H14       1.4180  110.44  119.01  110.49  1.1046
IC C15  C14  O14   H14O      1.5145  110.44  171.33  108.79  0.9758
IC C14  C12  *C13  O17       1.5431  105.41 -121.55  109.82  1.4353
IC C14  C12  *C13  H13       1.5431  105.41  114.66  110.72  1.1058
IC C12  C13  O17   C17       1.5129  109.82 -115.13  115.38  1.4320
IC C13  O17  C17   C18       1.4353  115.38  118.62  111.92  1.5323
IC C18  O17  *C17  O22       1.5323  111.92  121.39  109.82  1.4278
IC O22  O17  *C17  H17       1.4278  109.82  118.49  111.61  1.1127
IC O17  C17  O22   C21       1.4320  109.82  -54.40  113.28  1.4458
IC C17  O22  C21   C20       1.4278  113.28  -39.38  111.25  1.5288
IC C20  O22  *C21  C23       1.5288  111.25  128.43  114.44  1.5269
IC C20  O22  *C21  H211      1.5288  111.25 -116.03  103.72  1.1174
IC O17  C17  C18   C19       1.4320  111.92   95.02  111.19  1.5304
IC C19  C17  *C18  O18       1.5304  111.19 -126.69  113.05  1.4337
IC O18  C17  *C18  H18       1.4337  113.05 -117.58  107.56  1.1169
IC C17  C18  O18   H18O      1.5323  113.05  151.63  103.45  0.9725
IC C20  C18  *C19  O19       1.5159  109.18 -125.55  112.37  1.4318
IC O19  C18  *C19  H19       1.4318  112.37 -118.31  107.98  1.1179
IC C18  C19  O19   H19O      1.5304  112.37 -171.81  107.95  0.9671
IC C19  C21  *C20  O20       1.5159  109.32 -125.61  110.26  1.4264
IC C19  C21  *C20  H20       1.5159  109.32  116.47  108.75  1.1177
IC C21  C20  O20   H20O      1.5288  110.26 -128.58  110.57  0.9639
IC O22  C21  C23   O24       1.4458  114.44   30.27  113.91  1.4307
IC O24  C21  *C23  H231      1.4307  113.91 -120.11  109.09  1.1124
IC O24  C21  *C23  H232      1.4307  113.91  123.39  109.63  1.1152
IC C21  C23  O24   H24O      1.5269  113.91   63.06  109.95  0.9655
IC C4   N9   CM    HM1       1.3819  124.54  -62.74  110.14  1.1124
IC HM1  N9   *CM   HM2       1.1124  110.14  118.30  110.09  1.1122
IC HM1  N9   *CM   HM3       1.1124  110.14 -120.89  112.72  1.1123

RESI BGQG           1.00 ! b-galactosyl-queuosine
GROUP
ATOM N9     NG2R51  0.04 !              O24-H24O
```

```
ATOM C8     CG2R51  0.06 !              |
ATOM H8     HGR52   0.08 !         H231-C23-H232
ATOM C7     CG2R51 -0.14 !              |
ATOM C5     CG2RC0 -0.06 !         H211-C21--O22
ATOM C6     CG2R63  0.42 !  H20O-O20   /       \    O17---*
ATOM O6     OG2D4  -0.52 !          \ /H19O     \  /
ATOM N1     NG2R61 -0.38 !          C20 |       C17
ATOM H1     HGP1    0.30 !          / \ O19 H18 /  \
ATOM C2     CG2R64  0.65 !       H20   \|    | /    H17
ATOM N2     NG2S3  -0.60 !              C19--C18
ATOM H21    HGP4    0.30 !              |    |
ATOM H22    HGP4    0.30 !              H19  O18-H18O
ATOM N3     NG2R62 -0.77
ATOM C4     CG2RC0  0.32 !                        *
GROUP                    !                 H13   /
ATOM C10    CG324   0.18 !                   \  /
ATOM H101   HGA2    0.09 !     H101 H111 H12  C13     H14
ATOM H102   HGA2    0.09 !        |   |    \ /    \  /
ATOM N11    NG3P2  -0.35 !   >---C10--N11--C12     C14
ATOM H111   HGP2    0.28 !        |   |(+)  \      / \
ATOM H112   HGP2    0.28 !     H102 H112    C16==C15  O14-H14O
ATOM C12    CG3C53  0.34 !                  /      \
ATOM H12    HGA1    0.09 !                 H16     H15
GROUP
ATOM C15    CG2R51 -0.20 !             O6
ATOM H15    HGR51   0.26 !             ||         >
ATOM C16    CG2R51 -0.24 !             C6        /
ATOM H16    HGR51   0.18 !            /  \      /
GROUP                    !        H1-N1   C5--C7\\
ATOM C14    CG3C51  0.14 !           |    ||     C8-H8
ATOM H14    HGA1    0.09 !           C2   C4--N9/
ATOM O14    OG311  -0.65 !          / \\ /      \
ATOM H14O   HGP1    0.42 !    H21-N2   N3        \
GROUP                    !        |               \
ATOM C13    CG3C51  0.14 !       H22               \
ATOM H13    HGA1    0.09 !                          \
ATOM O17    OG301  -0.41
ATOM C17    CC3162  0.29
ATOM H17    HCA1    0.09
ATOM C21    CC3163  0.11
ATOM H211   HCA1    0.09
ATOM O22    OC3C61 -0.40
GROUP
ATOM C18    CC3161  0.14
ATOM H18    HCA1    0.09
ATOM O18    OC311  -0.65
ATOM H18O   HCP1    0.42 !  !!!! PATCH 7GNM for the neutral amino form
GROUP
ATOM C19    CC3161  0.14
ATOM H19    HCA1    0.09
ATOM O19    OC311  -0.65
ATOM H19O   HCP1    0.42
GROUP
ATOM C20    CC3161  0.14
ATOM H20    HCA1    0.09
ATOM O20    OC311  -0.65
ATOM H20O   HCP1    0.42
GROUP
ATOM C23    CC321   0.05
ATOM H231   HCA2    0.09
ATOM H232   HCA2    0.09
ATOM O24    OC311  -0.65
ATOM H24O   HCP1    0.42
```

```
GROUP
ATOM CM     CG331  -0.27
ATOM HM1    HGA3    0.09
ATOM HM2    HGA3    0.09
ATOM HM3    HGA3    0.09
BOND CM   HM1    CM   HM2   CM   HM3
BOND N9   C8   N9   C4   C8   H8   C8   C7
BOND C7   C5   C7   C10  C5   C6   C5   C4
BOND C6   O6   C6   N1   N1   H1   N1   C2
BOND C2   N2   C2   N3   N2   H21  N2   H22
BOND N3   C4   C10  H101 C10  H102 C10  N11
BOND N11  H111 N11  H112 N11  C12  C12  H12
BOND C12  C16  C12  C13  C14  O14  C14  H14
BOND C14  C15  C14  C13  O14  H14O C15  H15
BOND C15  C16  C16  H16  O22  C17  O22  C21
BOND C17  H17  C17  O17  C17  C18  O17  C13
BOND C13  H13  C21  H211 C21  C20  C21  C23
BOND C18  O18  C18  H18  C18  C19  O18  H18O
BOND C19  O19  C19  H19  C19  C20  O19  H19O
BOND C20  O20  C20  H20  O20  H20O C23  O24
BOND C23  H231 C23  H232 O24  H24O
BOND CM   N9
IMPR C6     C5     N1     O6     C2     N1     N3     N2     N2     H22    H21    C2
DONO H21   N2
DONO H22   N2
DONO H1   N1
DONO H111  N11
DONO H112  N11
DONO H14O  O14
DONO H18O  O18
DONO H19O  O19
DONO H20O  O20
DONO H24O  O24
ACCE O6   C6
ACCE N3
ACCE O14
ACCE O17
ACCE O18
ACCE O19
ACCE O20
ACCE O22
ACCE O24
IC C4   C8   *N9   CM       1.3819  110.09 -178.94  125.36  1.4668
IC C4   N9   C8    C7       1.3804  110.22    0.33  108.83  1.3705
IC C7   N9   *C8   H8       1.3705  108.83  177.88  121.20  1.0827
IC N9   C8   C7    C10      1.3857  108.83  171.91  124.51  1.4918
IC C10  C8   *C7   C5       1.4918  124.51 -173.06  106.47  1.4471
IC C4   C7   *C5   C6       1.4024  107.98  179.52  133.30  1.4095
IC C7   C5   C6    N1       1.4471  133.30 -178.59  113.57  1.3792
IC N1   C5   *C6   O6       1.3792  113.57 -178.96  127.85  1.2328
IC C5   C6   N1    C2       1.4095  113.57   -0.15  125.13  1.3724
IC C2   C6   *N1   H1       1.3724  125.13  179.13  117.24  0.9994
IC C6   N1   C2    N2       1.3792  125.13  179.67  117.42  1.3220
IC N2   N1   *C2   N3       1.3220  117.42  179.26  121.27  1.3341
IC N1   C2   N2    H21      1.3724  117.42   -0.63  124.15  0.9931
IC H21  C2   *N2   H22      0.9931  124.15 -179.66  115.88  0.9962
IC C8   C7   C10   N11      1.3705  124.51  146.46  111.32  1.5028
IC N11  C7   *C10  H101     1.5028  111.32 -118.53  112.06  1.1049
IC H101 C7   *C10  H102     1.1049  112.06 -124.46  110.74  1.1048
IC C7   C10  N11   C12      1.4918  111.32 -177.34  118.72  1.4938
IC C12  C10  *N11  H111     1.4938  118.72 -120.20  106.30  1.0186
IC H111 C10  *N11  H112     1.0186  106.30 -114.13  110.59  1.0049
IC C10  N11  C12   C13      1.5028  118.72 -150.63  110.11  1.5129
```

```
IC C13  N11  *C12  C16       1.5129  110.11 -114.84  109.73  1.5008
IC C16  N11  *C12  H12       1.5008  109.73 -125.00  108.86  1.0869
IC N11  C12  C16   C15       1.4938  109.73  133.12  109.91  1.3762
IC C15  C12  *C16  H16       1.3762  109.91  173.33  124.53  1.0834
IC C12  C16  C15   C14       1.5008  109.91    0.44  111.04  1.5145
IC C14  C16  *C15  H15       1.5145  111.04  169.67  127.30  1.0840
IC C13  C15  *C14  O14       1.5431  102.73  122.98  110.44  1.4180
IC O14  C15  *C14  H14       1.4180  110.44  119.00  110.52  1.1038
IC C15  C14  O14   H14O      1.5145  110.44  171.33  108.79  0.9758
IC C14  C12  *C13  O17       1.5431  105.41 -121.55  109.82  1.4353
IC C14  C12  *C13  H13       1.5431  105.41  114.66  110.72  1.1058
IC C12  C13  O17   C17       1.5129  109.82 -115.13  115.38  1.4320
IC C13  O17  C17   C18       1.4353  115.38  118.62  111.92  1.5323
IC C18  O17  *C17  O22       1.5323  111.92  121.39  109.82  1.4278
IC O22  O17  *C17  H17       1.4278  109.82  118.49  111.61  1.1127
IC O17  C17  O22   C21       1.4320  109.82  -54.40  113.28  1.4458
IC C17  O22  C21   C20       1.4278  113.28  -39.38  111.25  1.5288
IC C20  O22  *C21  C23       1.5288  111.25  128.42  114.48  1.5268
IC C20  O22  *C21  H211      1.5288  111.25 -116.03  103.72  1.1174
IC O17  C17  C18   C19       1.4320  111.92   95.02  111.19  1.5304
IC C19  C17  *C18  O18       1.5304  111.19  127.40  111.78  1.4342
IC O18  C17  *C18  H18       1.4342  111.78  115.99  106.08  1.1166
IC C17  C18  O18   H18O      1.5323  111.78   25.23  103.46  0.9724
IC C20  C18  *C19  O19       1.5159  109.18 -125.55  112.37  1.4318
IC O19  C18  *C19  H19       1.4318  112.37 -118.31  107.98  1.1179
IC C18  C19  O19   H19O      1.5304  112.37 -171.81  107.95  0.9671
IC C19  C21  *C20  O20       1.5159  109.32  123.65  113.64  1.4266
IC C19  C21  *C20  H20       1.5159  109.32 -117.60  106.96  1.1179
IC C21  C20  O20   H20O      1.5288  113.64  108.27  110.59  0.9637
IC O22  C21  C23   O24       1.4458  114.48   30.26  113.86  1.4316
IC O24  C21  *C23  H231      1.4316  113.86 -120.03  109.10  1.1122
IC O24  C21  *C23  H232      1.4316  113.86  123.37  109.68  1.1144
IC C21  C23  O24   H24O      1.5268  113.86   63.08  109.95  0.9655
IC C4   N9   CM    HM1       1.3819  124.54  -62.74  110.14  1.1124
IC HM1  N9   *CM   HM2       1.1124  110.14  118.30  110.09  1.1122
IC HM1  N9   *CM   HM3       1.1124  110.14 -120.89  112.72  1.1123

!!!!***PATCH FOR PROTONATED STATES AND TAUTOMERS***
PRES ENOU           0.00 ! patch for enol form of 5-sub uracils:
!RING 6 N1 C2 N3 C4 C5 C6! OAU and OEU
DELE ATOM H3             ! Optimized but not validated!!
ATOM N1    NG2R61  -0.15 !
ATOM C2    CG2R63   0.43 !          H4
ATOM O2    OG2D4   -0.42 !         /
ATOM N3    NG2R62  -0.69 !  C8    O4
ATOM C4    CG2R62   0.34 !   \    |
ATOM O4    OG311   -0.46 !    O7  C4
ATOM H4    HGP1     0.44 !     \ /  \\
ATOM C5    CG2R62   0.39 !      C5   N3
ATOM C6    CG2R62   0.20 !      ||   |
ATOM H6    HGR62    0.12 !   H6-C6   C2
ATOM O7    OG301   -0.48 !       \  / \\
ATOM C8    CG321    0.10 !        N1   O2
ATOM H81   HGA2     0.09
ATOM H82   HGA2     0.09
BOND O4   H4
DELE IMPR C4   C5   N3   O4
IC N3   C4   O4    H4        1.3061  118.59   -0.53  104.79  0.9812
DONO H4 O4
ACCE N3

PRES UENO           0.00 ! patch for enol form of uracil:
!RING 6 N1 C2 N3 C4 C5 C6
```

```
DELE ATOM H3              ! Optimized but not validated!!
ATOM N1    NG2R61  -0.10 !          H4
ATOM C2    CG2R63   0.53 !         /
ATOM O2    OG2D4   -0.45 !        O4
ATOM N3    NG2R62  -0.69 !        |
ATOM C4    CG2R62   0.42 !        C4
ATOM O4    OG311   -0.54 !       /  \\
ATOM H4    HGP1     0.47 !   H5-C5   N3
ATOM C5    CG2R62  -0.07 !      ||   |
ATOM H5    HGR62    0.10 !   H6-C6   C2
ATOM C6    CG2R62   0.19 !       \  / \\
ATOM H6    HGR62    0.14 !        N1   O2
BOND O4   H4
DELE IMPR C4   N3   C5   O4
IC N3   C4   O4    H4        1.3061  118.59   -0.53  104.79  0.9812
DONO H4 O4
ACCE N3

PRES 5UNA           0.00 ! patch for neutral aminomethyl in 5-sub uracil:
DELE ATOM H83             ! SAU
GROUP
ATOM C7     CG321   0.02
ATOM H71    HGA2    0.09
ATOM H72    HGA2    0.09
ATOM N8     NG321  -0.90
ATOM H81    HGPAM2  0.35
ATOM H82    HGPAM2  0.35

PRES 5UNM           0.00 ! patch for neutral methylaminomethyl in 5-sub uracils:
DELE ATOM HN1             ! 5AU and U8U
DELE ATOM HN2
GROUP
ATOM C      CG321   0.03
ATOM HC1    HGA2    0.09
ATOM HC2    HGA2    0.09
ATOM N      NG311  -0.78
ATOM HN     HGPAM1  0.36
ATOM CA     CG331  -0.06
ATOM HA1    HGA3    0.09
ATOM HA2    HGA3    0.09
ATOM HA3    HGA3    0.09
BOND N  HN
IC CA   C    *N    HN     1.4630  112.20  180.00  111.00  1.0190

PRES 5UNI           0.00 ! patch for neutral isopentenylaminomethyl in 5-sub uracils:
DELE ATOM H81             ! IAU, MIU and ISU
DELE ATOM H82
GROUP
ATOM C7     CG321   0.03
ATOM H71    HGA2    0.09
ATOM H72    HGA2    0.09
ATOM N8     NG311  -0.78
ATOM H8     HGPAM1  0.36
ATOM C9     CG321   0.03
ATOM H91    HGA2    0.09
ATOM H92    HGA2    0.09
BOND N8  H8
IC C9   C7   *N8   H8     1.4740  109.60  180.00  111.00  1.0190

PRES 5UHG           0.00 ! patch for non-ionized glysyl in 5-sub uracils:
DELE ATOM H81             ! 5DU, MAU, GCU and SCU
DELE ATOM H82
GROUP
```

```
ATOM C7     CG321   0.03
ATOM H71    HGA2    0.09
ATOM H72    HGA2    0.09
ATOM N8     NG311  -0.78
ATOM H8     HGPAM1  0.36
ATOM C9     CG321   0.03
ATOM H91    HGA2    0.09
ATOM H92    HGA2    0.09
GROUP
ATOM C10    CG2O2   0.72
ATOM O11    OG2D1  -0.55
ATOM O12    OG311  -0.60
ATOM H12O   HGP1    0.43
BOND N8  H8   O12  H12O
DELE IMPR C10  O11  O12  C9
IMPR C10   C9   O11   O12
IC C9   C7   *N8   H8     1.4740  109.60  180.00  111.00  1.0190
IC C9   C10  O12   H12O   1.5220  110.50  180.00  115.00  0.9600

PRES 5UHC           0.00 ! patch for acetic acid in 5-sub uracils:
DELE ATOM O81            ! 5CU and HCU
DELE ATOM O82
GROUP
ATOM C8     CG2O2   0.72
ATOM O8     OG2D1  -0.55
ATOM O9     OG311  -0.60
ATOM H9O    HGP1    0.43
BOND C8 O8  C8 O9  O9 H9O
IMPR C8 C7  O8 O9
IC C5   C7   C8    O9     1.4800  107.50  180.00  110.50  1.4000
IC O9   C7   *C8   O8     1.4000  110.50  180.00  125.00  1.2200
IC C7   C8   O9    H9O    1.5220  110.50  180.00  115.00  0.9600
DONO H9O  O9
ACCE O8
ACCE O9

PRES 5UHO           0.00 ! patch for acetic acid in 5-sub uracil:
DELE ATOM O91            ! OAU
DELE ATOM O92
GROUP
ATOM C9     CG2O2   0.72
ATOM O9     OG2D1  -0.55
ATOM O10    OG311  -0.60
ATOM H10O   HGP1    0.43
BOND C9 O9  C9 O10  O10 H10O
IMPR C9 C8  O9 O10
IC O7   C8   C9    O10    1.4150  109.00  180.00  110.50  1.4000
IC O10  C8   *C9   O9     1.4000  110.50  180.00  125.00  1.2200
IC C8   C9   O10   H10O   1.5220  110.50  180.00  115.00  0.9600
DONO H10O  O10
ACCE O9
ACCE O10

PRES 5UHA           0.00 ! patch for non-ionized alanyl in sub-pyrimidines:
DELE ATOM H143           ! 3AU, 13P and K2C
GROUP
ATOM C12    CG311   0.11 !     H12    O31--H31O
ATOM H12    HGA1    0.09 !      |     /
ATOM N14    NG321  -0.92 !  *-C12--C13
ATOM H141   HGPAM2  0.36 !      |     \\
ATOM H142   HGPAM2  0.36 !      |      O30
GROUP                    !     N14
ATOM C13    CG2O2   0.72 !    /  \
```

```
ATOM O30    OG2D1  -0.55 !  H141 H142
ATOM O31    OG311  -0.60
ATOM H31O   HGP1    0.43
BOND O31   H31O
DELE IMPR C13    O30    O31    C12
IMPR C13    C12    O30    O31
IC C12  C13  O31   H31O   1.5220  110.50  180.00  115.00  0.9600

PRES CYTP           1.00 ! patch for protonated cytosines:     ! RCP
!RING 6 N1 C2 N3 C4 C5 C6! CYT and OMC
GROUP                    !
ATOM N1     NG2R61 -0.11 !    H42  H41
ATOM C2     CG2R63  0.78 !      \  /
ATOM O2     OG2D4  -0.40 !       N4(+)
ATOM N3     NG2P1  -0.71 !       ||
ATOM H3     HGP2    0.40 !       C4   H3
ATOM C4     CG2R64  0.75 !      /  \ /
ATOM N4     NG2P1  -0.76 !  H5-C5   N3
ATOM H41    HGP2    0.40 !     ||    |
ATOM H42    HGP2    0.40 !  H6-C6   C2
ATOM C5     CG2R62 -0.15 !      \  / \\
ATOM H5     HGR62   0.06 !       N1   O2
ATOM C6     CG2R62  0.09 !        \
ATOM H6     HGR62   0.25 !
BOND N3   H3             !
DELE IMPR N4   C4   H41  H42
IMPR N4   H41  H42  C4
DONO H3   N3
IC C4   C2   *N3   H3        1.3462  122.41 -179.95  110.98  0.9898

PRES 5MCP           1.00 ! patch for protonated 5-methylcytosines:
!RING 6 N1 C2 N3 C4 C5 C6! 5MC and MMC
GROUP                    !
ATOM N1     NG2R61 -0.11 !          H42  H41
ATOM C2     CG2R63  0.73 !            \  /
ATOM O2     OG2D4  -0.39 !             N4(+)
ATOM N3     NG2P1  -0.71 !             ||
ATOM H3     HGP2    0.40 !   H51       C4   H3
ATOM C4     CG2R64  0.72 !      \     /  \ /
ATOM N4     NG2P1  -0.73 !  H52--CM5-C5   N3
ATOM H41    HGP2    0.40 !      /    ||    |
ATOM H42    HGP2    0.40 !   H53  H6-C6   C2
ATOM C5     CG2R62 -0.04 !            \  / \\
ATOM C6     CG2R62  0.06 !             N1   O2
ATOM H6     HGR62   0.27 !              \
ATOM CM5    CG331  -0.21 !
ATOM H51    HGA3    0.07 !
ATOM H52    HGA3    0.07 !
ATOM H53    HGA3    0.07 !
BOND N3   H3             !
IC C4   C2   *N3   H3        1.3462  122.41 -179.95  110.98  0.9898

PRES 3MCN           0.00 ! patch for neutral 3N-methyl cytosine:
!RING 6 N1 C2 N3 C4 C5 C6! 3MC
DELE ATOM  H41
DELE ATOM  H42
GROUP                    !    H4
ATOM N1     NG2R61 -0.08 !      \
ATOM C2     CG2R63  0.41 !       N4      H31
ATOM O2     OG2D4  -0.44 !       ||     /
ATOM N3     NG2R61 -0.34 !       C4   CN3-H32
ATOM C4     CG2R64  0.49 !      /  \ /  \
ATOM N4     NG2D1  -0.86 !  H5-C5   N3   H33
```

```
ATOM H4      HGP1    0.32 !     ||   |
ATOM C5      CG2R62  0.04 !  H6-C6   C2
ATOM H5      HGR62   0.02 !      \  / \\
ATOM C6      CG2R62  0.13 !       N1   O2
ATOM H6      HGR62   0.10 !         \
ATOM CN3     CG331  -0.06 !          \
ATOM H31     HGA3    0.09
ATOM H32     HGA3    0.09
ATOM H33     HGA3    0.09
BOND N4   H4
DELE IMPR C4   C5   N3   N4
IMPR C4   C5   N4   N3
IC N3   C4   N4    H4        1.4065  118.52  179.98  113.45  0.9989

PRES K2CN           0.00 ! patch for neutral 2-aminocytosines:
!RING 6 N1 C2 N3 C4 C5 C6! K2C and R2C
DELE ATOM  H3
GROUP
ATOM N1      NG2R61 -0.31 !            H4
ATOM C2      CG2R64  0.85 !           /
ATOM N2      NG311  -0.59 !         N4
ATOM H2      HGPAM1  0.37 !         ||
ATOM N3      NG2R62 -0.73 !         C4
ATOM C4      CG2R64  0.63 !        /  \
ATOM N4      NG2D1  -0.90 !    H5-C5   N3
ATOM H4      HGP1    0.32 !       ||   ||
ATOM C5      CG2R61 -0.21 !    H6-C6   C2      H71
ATOM H5      HGR61   0.14 !        \  /  \      |
ATOM C6      CG2R61  0.10 !         N1    N2 --C7-
ATOM H6      HGR62   0.16 !          \    |     |
ATOM C7      CG321  -0.01 !           \   H2   H72
ATOM H71     HGA2    0.09 !            \
ATOM H72     HGA2    0.09

PRES 34HC           1.00 ! patch for tautomers of charged 2-aminocytosines:
!RING 6 N1 C2 N3 C4 C5 C6! K2C and R2C
DELE ATOM  H3             !
DELE ATOM  H4             !
GROUP                     !
ATOM N1      NG2R61 -0.40 !      H41   H42
ATOM C2      CG2R64  0.81 !        \  /
ATOM N2      NG311  -0.57 !         N4
ATOM H2      HGPAM1  0.46 !         ||
ATOM N3      NG2R62 -0.64 !         C4
ATOM C4      CG2R64  0.61 !        /  \
ATOM N4      NG2P1  -0.79 !    H5-C5   N3
ATOM H41     HGP2    0.37 !       ||   ||
ATOM H42     HGP2    0.37 !    H6-C6   C2      H71
ATOM C5      CG2R61  0.08 !        \  /  \      |
ATOM H5      HGR61   0.07 !         N1    N2 --C7-
ATOM C6      CG2R61  0.18 !          \    |     |
ATOM H6      HGR62   0.20 !           \   H2   H72
ATOM C7      CG321   0.07 !            \
ATOM H71     HGA2    0.09 !
ATOM H72     HGA2    0.09
BOND N4   H41   N4   H42
DONO H41  N4
DONO H42  N4
IC N3   C4   N4    H41    1.3420  122.00  180.00  120.70  1.0000
IC H41  C4   *N4   H42    1.0000  120.70  180.00  120.70  1.0000

PRES ADEP           1.00 ! patch for protonated adenines:    ! RAP
!RING 5 C4 C5 N7 C8 N9   ! ADE and OMA
```

```
!RING 6 N1 C2 N3 C4 C5 C6!
GROUP                   !
ATOM N9     NG2R51  0.01 !
ATOM C8     CG2R53  0.37 !      H61  H62
ATOM H8     HGR52   0.14 !        \  /
ATOM N7     NG2R50 -0.69 !      (+)N6
ATOM C5     CG2RC0  0.21 !         ||
ATOM C6     CG2R64  0.59 !    H1   C6
ATOM N6     NG2P1  -0.67 !      \ /  \
ATOM H61    HGP2    0.38 !       N1   C5--N7\\
ATOM H62    HGP2    0.38 !       |    ||      C8-H8
ATOM N1     NG2P1  -0.65 !       C2   C4--N9/
ATOM H1     HGP2    0.40 !      / \\ /     \
ATOM C2     CG2R64  0.49 !    H2   N3       \
ATOM H2     HGR62   0.18 !                   \
ATOM N3     NG2R62 -0.63 !
ATOM C4     CG2RC0  0.49 !
BOND N1   H1
DELE IMPR N6   C6   H61  H62
DELE IMPR C6    N1    C5    N6
IMPR C6   C5   N1   N6
IMPR N6   H61  H62   C6
IC C2   C6   *N1   H1        1.4172  121.71  179.96  117.58  0.9963
DONO H1 N1

PRES ADEI           0.00 ! patch for imine adenines:
!RING 5 C4 C5 N7 C8 N9   ! ADE and OMA
!RING 6 N1 C2 N3 C4 C5 C6
DELE ATOM  H61
DELE ATOM  H62
GROUP                   !              H6
ATOM N9     NG2R51  0.00 !             /
ATOM C8     CG2R53  0.42 !           N6
ATOM H8     HGR52   0.07 !           ||
ATOM N7     NG2R50 -0.79 !           C6
ATOM C5     CG2RC0  0.27 !          /  \
ATOM C6     CG2R64  0.28 !      H1-N1   C5--N7\\
ATOM N6     NG2D1  -0.82 !         |    ||      C8-H8
ATOM H6     HGP1    0.37 !         C2   C4--N9/
ATOM N1     NG2R61 -0.43 !        / \\ /     \
ATOM H1     HGP1    0.28 !      H2   N3       \
ATOM C2     CG2R64  0.56 !                     \
ATOM H2     HGR62   0.09
ATOM N3     NG2R62 -0.82
ATOM C4     CG2RC0  0.52
BOND N1   H1    N6   H6
DELE IMPR C6   N1   C5   N6
IMPR C6   C5   N6   N1
DONO H6  N6
DONO H1  N1
ACCE N6
IC C5   C6   N6    H6        1.4093  127.86  179.97  107.01  0.9968

PRES 1MAN           0.00 ! patch for neutral 1N-methyl adenines:
!RING 5 C4 C5 N7 C8 N9   ! 1MA and M2A
!RING 6 N1 C2 N3 C4 C5 C6
DELE ATOM  H61
DELE ATOM  H62
GROUP                   !              H6
ATOM N9     NG2R51  0.00 !             /
ATOM C8     CG2R53  0.42 !           N6
ATOM H8     HGR52   0.07 !           ||
ATOM N7     NG2R50 -0.79 !  H11      C6
```

```
ATOM C5     CG2RC0  0.27 !     \    /  \
ATOM C6     CG2R64  0.27 ! H12-CM1-N1   C5--N7\\
ATOM N6     NG2D1  -0.82 !     /   |    ||     C8-H8
ATOM H6     HGP1    0.37 !  H13    C2   C4--N9/
ATOM N1     NG2R61 -0.38 !        / \\ /     \
ATOM C2     CG2R64  0.56 !      H2   N3       \
ATOM H2     HGR62   0.09 !                     \
ATOM N3     NG2R62 -0.82
ATOM C4     CG2RC0  0.52
ATOM CM1    CG331  -0.03
ATOM H11    HGA3    0.09
ATOM H12    HGA3    0.09
ATOM H13    HGA3    0.09
BOND N6   H6
DELE IMPR C6   C5   N1   N6
IMPR C6   C5   N6   N1
DONO H6  N6
ACCE N6
IC C5   C6   N6    H6        1.4093  127.86  179.97  107.01  0.9968

PRES 6AH            0.00 ! patch for neutral carboxylic acid in N6-
carbamoyladenosines:
                         ! HNA, 26A, 66A, T6A, 12A and 6GA
GROUP
ATOM C13    CG2O2   0.72 !     C12
ATOM ODA    OG2D1  -0.55 !       \
ATOM ODB    OG311  -0.60 !        C13-ODB-HOB
ATOM HOB    HGP1    0.43 !        ||
BOND ODB HOB             !        ODA
DELE IMPR C13  ODB  ODA  C12
IMPR C13  C12  ODA  ODB
IC C12  C13  ODB   HOB    1.5220  110.50  180.00  115.00  0.9600

!PRES 6AH            0.00 ! patch for neutral carboxylic acid in N6-
carbamoyladenosines:
!                         ! HNA, 26A, 66A, T6A, 12A and 6GA
!GROUP                    !                              (different PDB atom names!)
!ATOM C      CG2O2   0.72 !      CA
!ATOM O      OG2D1  -0.55 !       \
!ATOM OXT    OG311  -0.60 !        C--OXT-HOX
!ATOM HOX    HGP1    0.43 !        ||
!BOND OXT  HOX             !        O
!IMPR C    O    OXT    CA

PRES GENO           0.00 ! patch for enol form of guanines:
!RING 5 C4 C5 N7 C8 N9   ! GUA, OMG, RIG, etc.
!RING 6 N1 C2 N3 C4 C5 C6 ! Optimized but not validated!!
DELE ATOM  H1            !
GROUP                    !
ATOM  N9    NG2R51  0.06 !         H6
ATOM  C8    CG2R53  0.33 !          \
ATOM  H8    HGR52   0.09 !           O6
ATOM  N7    NG2R50 -0.64 !           |
ATOM  C5    CG2RC0  0.06 !           C6
ATOM  C6    CG2R61  0.51 !         //  \
ATOM  O6    OG311  -0.49 !         N1   C5--N7\\
ATOM  H6    HGP1    0.45 !         |    ||     C8-H8
ATOM  N1    NG2R62 -0.77 !         C2   C4--N9/
ATOM  C2    CG2R64  0.66 !        / \\ /      \
ATOM  N2    NG2S3  -0.66 !  H21-N2   N3        \
ATOM  H21   HGP4    0.34 !      |               \
ATOM  H22   HGP4    0.34 !     H22               \
ATOM  N3    NG2R62 -0.76 !
```

```
ATOM  C4    CG2RC0  0.48 !
BOND O6   H6
DELE IMPR C6   N1   C5   O6
DELE IMPR N2   H21  C2   H22
IMPR N2   H21  H22  C2
IC C5   C6   O6   H6    1.3600  120.00  180.00  108.00   0.9600
DONO H6  O6
ACCE N1

PRES 7MGE           0.00 ! patch for enol form of 7-methyl guanine:
!RING 5 C4 C5 N7 C8 N9   ! 7MG
!RING 6 N1 C2 N3 C4 C5 C6 ! Optimized but not validated!!
DELE ATOM  H1             !
GROUP                     !
ATOM  N9    NG2R52 -0.02 !          H6
ATOM  C8    CG2R53  0.35 !           \
ATOM  H8    HGR53   0.16 !            O6     H71  H72
ATOM  N7    NG2R52 -0.55 !            |        \ /
ATOM  C5    CG2RC0  0.46 !            C6        CM7-H73
ATOM  C6    CG2R61  0.39 !          //  \   (+)/
ATOM  O6    OG311  -0.54 !          N1   C5--N7\\
ATOM  H6    HGP1    0.47 !          |    ||     C8-H8
ATOM  N1    NG2R62 -0.64 !          C2   C4--N9/
ATOM  C2    CG2R64  0.69 !         / \\ /      \
ATOM  N2    NG2S3  -0.77 !   H21-N2   N3        \
ATOM  H21   HGP4    0.39 !       |               \
ATOM  H22   HGP4    0.39 !      H22               \
ATOM  N3    NG2R62 -0.64 !
ATOM  C4    CG2RC0  0.46 !
ATOM  CM7   CG334   0.13
ATOM  H71   HGA3    0.09
ATOM  H72   HGA3    0.09
ATOM  H73   HGA3    0.09
BOND O6   H6
DELE IMPR C6   C5   N1   O6
IC C5   C6   O6   H6    1.3600  120.00  180.00  108.00   0.9600
DONO H6  O6
ACCE N1

PRES 7GNA           0.00 ! patch for neutral aminomethyl in 7-sub deazaguanines:
DELE ATOM H113           ! DAG
GROUP
ATOM C10    CG321   0.02
ATOM H101   HGA2    0.09
ATOM H102   HGA2    0.09
ATOM N11    NG321  -0.90
ATOM H111   HGPAM2  0.35
ATOM H112   HGPAM2  0.35

PRES 7GNM           0.00 ! patch for neutral aminomethyl in 7-sub deazaguanines:
DELE ATOM H111           ! QUG, EQG, MQG and GQG
DELE ATOM H112
GROUP
ATOM C10    CG321   0.03
ATOM H101   HGA2    0.09
ATOM H102   HGA2    0.09
ATOM N11    NG311  -0.78
ATOM H11    HGPAM1  0.36
ATOM C12    CG3C51  0.12
ATOM H12    HGA1    0.09
BOND N11  H11
IC C12  C10  *N11  H11    1.4560  111.00  180.00  111.00  1.0190
```

```
PRES BUGN           0.00 ! patch for non-ionized alanyl in undermodified
hydroxywybutosine:
DELE ATOM H163           ! BUG
GROUP
ATOM C15    CG311   0.11 !     H15    O18--H18O
ATOM H15    HGA1    0.09 !     |     /
ATOM N16    NG321  -0.92 !  *-C15--C17
ATOM H161   HGPAM2  0.36 !     |     \\
ATOM H162   HGPAM2  0.36 !     |      O17
GROUP                    !     N16
ATOM C17    CG2O2   0.72 !    /  \
ATOM O17    OG2D1  -0.55 ! H161  H162
ATOM O18    OG311  -0.60
ATOM H18O   HGP1    0.43
BOND O18  H18O
DELE IMPR C17    O17    O18    C15
IMPR C17    C15    O17    O18
IC C15  C17  O18   H18O   1.5220  110.50  180.00  115.00  0.9600

!!!!!!!! ######################################## !!!!!!!!
!!!!!!!! ######## CGenFF model compounds ######## !!!!!!!!
!!!!!!!! ######################################## !!!!!!!!

!!***uracils***

RESI B2SU           0.00 ! 2-thiouracil, yxu
GROUP
ATOM N1     NG2R61 -0.26
ATOM C2     CG2R63  0.31
ATOM S2     SG2D1  -0.22 !      O4
ATOM N3     NG2R61 -0.56 !      ||
ATOM H3     HGP1    0.40 !      C4    H3
ATOM C4     CG2R63  0.41 !     /  \  /
ATOM O4     OG2D4  -0.43 ! H5-C5   N3
ATOM C5     CG2R62 -0.25 !    ||   |
ATOM H5     HGR62   0.20 ! H6-C6   C2
ATOM C6     CG2R62  0.20 !     \  / \\
ATOM H6     HGR62   0.20 !      N1   S2
GROUP                    !       \
ATOM CM     CG331  -0.27 !        \
ATOM HM1    HGA3    0.09 !         \
ATOM HM2    HGA3    0.09
ATOM HM3    HGA3    0.09

BOND CM   HM1    CM   HM2   CM   HM3
BOND N1   C2      N1   C6      C2   S2      C2   N3
BOND N3   C4      N3   H3      C4   O4      C4   C5
BOND C5   C6      C5   H5      C6   H6
BOND CM   N1
IMPR C2     N1     N3     S2     C4     C5     N3     O4
DONO H3   N3
ACCE O4   C4
ACCE S2   C2
IC C6   C2   *N1   CM       1.3715  121.09 -179.97  118.42  1.4745
IC C6   N1   C2    N3       1.3715  121.09   -0.04  113.56  1.3854
IC N3   N1   *C2   S2       1.3854  113.56 -179.98  126.36  1.6538
IC N1   C2   N3    C4       1.4182  113.56    0.04  127.86  1.3727
IC C4   C2   *N3   H3       1.3727  127.86 -180.00  117.69  1.0026
IC C2   N3   C4    O4       1.3854  127.86  179.99  118.80  1.2256
IC O4   N3   *C4   C5       1.2256  118.80  179.94  115.74  1.4190
IC C6   C4   *C5   H5       1.3718  119.08  179.92  119.11  1.0852
IC C5   N1   *C6   H6       1.3718  122.67  179.95  116.10  1.0912
IC C6   N1   CM    HM1      1.3715  120.48 -120.20  111.15  1.1150
```

```
IC HM1  N1   *CM   HM2       1.1150  111.15  120.23  112.77  1.1125
IC HM1  N1   *CM   HM3       1.1150  111.15 -119.56  111.15  1.1154

RESI B4SU           0.00 ! 4-thiouracil, yxu
GROUP
ATOM N1     NG2R61 -0.20
ATOM C2     CG2R63  0.44
ATOM O2     OG2D4  -0.39 !      S4
ATOM N3     NG2R61 -0.64 !      ||
ATOM H3     HGP1    0.42 !      C4    H3
ATOM C4     CG2R63  0.33 !     /  \  /
ATOM S4     SG2D1  -0.25 ! H5-C5   N3
ATOM C5     CG2R62 -0.20 !    ||   |
ATOM H5     HGR62   0.13 ! H6-C6   C2
ATOM C6     CG2R62  0.15 !     \  / \\
ATOM H6     HGR62   0.21 !      N1   O2
GROUP                    !       \
ATOM CM     CG331  -0.27 !        \
ATOM HM1    HGA3    0.09 !         \
ATOM HM2    HGA3    0.09
ATOM HM3    HGA3    0.09

BOND CM   HM1    CM   HM2   CM   HM3
BOND N1   C2      N1   C6      C2   O2      C2   N3
BOND N3   C4      N3   H3      C4   S4      C4   C5
BOND C5   C6      C5   H5      C6   H6
BOND CM   N1
IMPR C2     N1     N3     O2         C4     C5     N3     S4
DONO H3   N3
ACCE O2   C2
ACCE S4   C4
IC C2   C6   *N1   CM        1.4193  107.70 -179.45  107.87  1.4850
IC C6   N1   C2    N3        1.3755  107.70  -59.67  111.98  1.3862
IC N3   N1   *C2   O2        1.3862  111.98  179.26  122.83  1.2268
IC N1   C2   N3    C4        1.4193  111.98   41.23  120.65  1.4004
IC C4   C2   *N3   H3        1.4004  120.65  179.39  116.01  1.0021
IC C2   N3   C4    S4        1.3862  120.65  168.63  121.48  1.6473
IC S4   N3   *C4   C5        1.6473  121.48 -179.22  114.43  1.4522
IC C6   C4   *C5   H5        1.3865  118.20  179.04  120.79  1.0875
IC C5   N1   *C6   H6        1.3865  118.66  179.61  114.47  1.0903
IC C6   N1   CM    HM1       1.3755  107.87   54.64  115.11  1.1129
IC HM1  N1   *CM   HM2       1.1129  115.11  120.73  110.33  1.1148
IC HM1  N1   *CM   HM3       1.1129  115.11 -121.85  111.01  1.1149

RESI B52U           0.00 ! 5-methyl-2-thiouracil, yxu
GROUP
ATOM N1     NG2R61 -0.26
ATOM C2     CG2R63  0.29
ATOM S2     SG2D1  -0.22 !    H51   O4
ATOM N3     NG2R61 -0.56 !     |    ||
ATOM H3     HGP1    0.40 ! H52-C5M  C4    H3
ATOM C4     CG2R63  0.39 !    /  \ /  \  /
ATOM O4     OG2D4  -0.41 !  H53   C5   N3
ATOM C5     CG2R62 -0.13 !        ||   |
ATOM C6     CG2R62  0.18 !     H6-C6   C2
ATOM H6     HGR62   0.22 !         \  / \\
ATOM C5M    CG331  -0.17 !          N1   S2
ATOM H51    HGA3    0.09 !           \
ATOM H52    HGA3    0.09 !            \
ATOM H53    HGA3    0.09 !             \
GROUP
ATOM CM     CG331  -0.27
ATOM HM1    HGA3    0.09
```

```
ATOM HM2    HGA3    0.09
ATOM HM3    HGA3    0.09

BOND CM   HM1    CM   HM2   CM   HM3
BOND N1   C2   N1   C6   C2   S2   C2   N3
BOND N3   C4   N3   H3   C4   O4   C4   C5
BOND C5   C6   C5   C5M  C6   H6   C5M  H51
BOND C5M  H52  C5M  H53
BOND CM   N1
IMPR C2     N1     N3     S2     C4     C5     N3     O4
DONO H3   N3
ACCE O4   C4
ACCE S2   C2
IC C2   C6   *N1   CM        1.4167  121.25  179.71  120.44  1.4743
IC C6   N1   C2    N3        1.3725  121.25    0.58  113.53  1.3846
IC N3   N1   *C2   S2        1.3846  113.53  179.65  126.45  1.6536
IC N1   C2   N3    C4        1.4167  113.53    0.78  127.95  1.3785
IC C4   C2   *N3   H3        1.3785  127.95  177.08  117.51  1.0036
IC C2   N3   C4    C5        1.3846  127.95   -3.82  115.84  1.4400
IC C5   N3   *C4   O4        1.4400  115.84 -179.69  118.16  1.2282
IC C5   N1   *C6   H6        1.3737  123.46 -179.24  116.10  1.0886
IC C6   C4   *C5   C5M       1.3737  117.76  171.34  118.86  1.4980
IC C4   C5   C5M   H51       1.4400  118.86  156.01  111.71  1.1126
IC H51  C5   *C5M  H52       1.1126  111.71  120.77  110.71  1.1130
IC H51  C5   *C5M  H53       1.1126  111.71 -119.90  110.96  1.1142
IC C6   N1   CM    HM1       1.3725  120.44    0.20  112.76  1.1136
IC HM1  N1   *CM   HM2       1.1136  112.76  120.25  111.20  1.1145
IC HM1  N1   *CM   HM3       1.1136  112.76 -120.16  111.16  1.1150

RESI BH2U           0.00 ! Dihydrouracil, DHU, D, yxu
GROUP
ATOM N1     NG2S0  -0.19
ATOM C2     CG2O6   0.32 !       O4
ATOM O2     OG2D1  -0.42 !       ||
ATOM N3     NG2S1  -0.40 !  H51  C4    H3
ATOM H3     HGP1    0.32 !    \ /  \  /
ATOM C4     CG2O1   0.55 ! H52-C5   N3
ATOM O4     OG2D1  -0.49 !      |    |
ATOM C5     CG321  -0.14 ! H61-C6   C2
ATOM H51    HGA2    0.09 !    / \  / \\
ATOM H52    HGA2    0.09 !  H62  N1   O2
ATOM C6     CG321   0.09 !         \
ATOM H61    HGA2    0.09 !          \
ATOM H62    HGA2    0.09 !           \
GROUP
ATOM CM     CG331  -0.27
ATOM HM1    HGA3    0.09
ATOM HM2    HGA3    0.09
ATOM HM3    HGA3    0.09

BOND CM   HM1    CM   HM2   CM   HM3
BOND N1   C2      N1   C6      C2   O2      C2   N3
BOND N3   C4      N3   H3      C4   O4      C4   C5
BOND C5   C6      C5   H51     C5   H52     C6   H61     C6   H62
BOND CM   N1
IMPR C2     N1     N3     O2      C4     C5     N3     O4
DONO H3   N3
ACCE O2   C2
ACCE O4   C4
IC C2   C6   *N1   CM        1.3740  119.36 -162.57  118.38  1.4519
IC C6   N1   C2    N3        1.4546  119.36   14.59  115.04  1.3596
IC N3   N1   *C2   O2        1.3596  115.04  179.16  125.38  1.2238
IC N1   C2   N3    C4        1.3740  115.04    9.32  127.08  1.3235
```

```
IC C4   C2   *N3   H3        1.3235  127.08  179.70  116.41  0.9898
IC C2   N3   C4    O4        1.3596  127.08 -177.13  120.84  1.2226
IC O4   N3   *C4   C5        1.2226  120.84  179.99  118.30  1.4822
IC C6   C4   *C5   H51       1.5245  106.91  119.22  108.89  1.1121
IC C6   C4   *C5   H52       1.5245  106.91 -121.19  109.89  1.1102
IC C5   N1   *C6   H61       1.5245  109.99  123.40  109.42  1.1138
IC C5   N1   *C6   H62       1.5245  109.99 -119.30  109.10  1.1141
IC C6   N1   CM    HM1       1.4546  118.38   65.07  107.60  1.1106
IC HM1  N1   *CM   HM2       1.1106  107.60  120.51  109.87  1.1133
IC HM1  N1   *CM   HM3       1.1106  107.60 -119.90  107.62  1.1101

RESI BMDU           0.00 ! 5-methyldihydrouracil, DMU, yxu
GROUP
ATOM N1     NG2S0  -0.19
ATOM C2     CG2O6   0.32
ATOM O2     OG2D1  -0.42
ATOM N3     NG2S1  -0.40
ATOM H3     HGP1    0.32 ! H52 H53  O4
ATOM C4     CG2O1   0.55 !    \ |   ||
ATOM O4     OG2D1  -0.49 ! H51-C5M  C4    H3
ATOM C5     CG311  -0.05 !       \ /  \  /
ATOM H5     HGA1    0.09 !     H5-C5   N3
ATOM C6     CG321   0.09 !         |    |
ATOM H61    HGA2    0.09 !    H61-C6   C2
ATOM H62    HGA2    0.09 !       / \  / \\
GROUP                    !     H62  N1   O2
ATOM C5M    CG331  -0.27 !           \
ATOM H51    HGA3    0.09 !            \
ATOM H52    HGA3    0.09 !             \
ATOM H53    HGA3    0.09 !              \
GROUP
ATOM CM     CG331  -0.27
ATOM HM1    HGA3    0.09
ATOM HM2    HGA3    0.09
ATOM HM3    HGA3    0.09

BOND CM   HM1    CM   HM2   CM   HM3
BOND N1   C2   N1   C6   C2   O2   C2   N3
BOND N3   C4   N3   H3   C4   O4   C4   C5
BOND C5   C6   C5   H5   C5   C5M  C6   H61
BOND C6   H62  C5M  H51  C5M  H52  C5M  H53
BOND CM   N1
IMPR C2     N1     N3     O2     C4     C5     N3     O4
DONO H3   N3
ACCE O2   C2
ACCE O4   C4
IC C2   C6   *N1   CM        1.3753  119.40 -163.43  118.45  1.4520
IC C6   N1   C2    N3        1.4557  119.40   13.69  115.09  1.3598
IC N3   N1   *C2   O2        1.3598  115.09  179.32  125.28  1.2241
IC N1   C2   N3    C4        1.3753  115.09    7.83  127.35  1.3258
IC C4   C2   *N3   H3        1.3258  127.35 -177.37  116.20  0.9888
IC C2   N3   C4    C5        1.3598  127.35    6.03  118.57  1.4952
IC C5   N3   *C4   O4        1.4952  118.57 -179.53  120.36  1.2233
IC C6   C4   *C5   C5M       1.5354  105.49  123.19  111.05  1.5534
IC C6   C4   *C5   H5        1.5354  105.49 -117.32  108.03  1.1100
IC C5   N1   *C6   H61       1.5354  111.05  123.33  109.30  1.1121
IC C5   N1   *C6   H62       1.5354  111.05 -119.52  109.05  1.1129
IC C4   C5   C5M   H51       1.4952  111.05 -179.97  110.29  1.1111
IC H51  C5   *C5M  H52       1.1111  110.29  119.91  111.28  1.1115
IC H51  C5   *C5M  H53       1.1111  110.29 -119.53  110.70  1.1120
IC C6   N1   CM    HM1       1.4557  118.45  -55.08  107.51  1.1106
IC HM1  N1   *CM   HM2       1.1106  107.51  119.82  107.58  1.1104
IC HM1  N1   *CM   HM3       1.1106  107.51 -119.61  109.91  1.1128
```

```
RESI B5HU           0.00 ! 5-hydroxyuracil, yxu
GROUP
ATOM N1     NG2R61 -0.25 ! HO5  O4
ATOM C2     CG2R63  0.52 !  |   ||
ATOM O2     OG2D4  -0.43 !  O5  C4    H3
ATOM N3     NG2R61 -0.48 !   \ /  \  /
ATOM H3     HGP1    0.30 !    C5   N3
ATOM C4     CG2R63  0.55 !    ||   |
ATOM O4     OG2D4  -0.46 ! H6-C6   C2
ATOM C5     CG2R62  0.14 !      \  / \\
ATOM O5     OG311  -0.60 !       N1   O2
ATOM HO5    HGP1    0.39 !        \
ATOM C6     CG2R62  0.17 !         \
ATOM H6     HGR62   0.15 !          \
GROUP
ATOM CM     CG331  -0.27
ATOM HM1    HGA3    0.09
ATOM HM2    HGA3    0.09
ATOM HM3    HGA3    0.09

BOND CM   HM1    CM   HM2   CM   HM3
BOND N1   C2   N1   C6   C2   O2   C2   N3
BOND N3   C4   N3   H3   C4   O4   C4   C5
BOND C5   C6   C5   O5   O5   HO5  C6   H6
BOND CM   N1
IMPR C2     N1     N3     O2        C4     C5     N3     O4
DONO H3   N3
DONO HO5  O5
ACCE O5   C5
ACCE O2   C2
ACCE O4   C4
IC C2   C6   *N1   CM        1.4094  120.13  179.95  121.34  1.4733
IC C6   N1   C2    N3        1.3715  120.13   -0.05  116.45  1.3693
IC N3   N1   *C2   O2        1.3693  116.45 -179.93  122.91  1.2283
IC N1   C2   N3    C4        1.4094  116.45    0.05  126.27  1.3812
IC C4   C2   *N3   H3        1.3812  126.27  179.94  117.02  1.0035
IC C2   N3   C4    C5        1.3693  126.27   -0.04  116.08  1.4697
IC C5   N3   *C4   O4        1.4697  116.08 -179.97  117.13  1.2315
IC C6   C4   *C5   O5        1.3764  117.28  179.97  121.16  1.3729
IC C4   C5   O5    HO5       1.4697  121.16 -179.99  110.82  0.9607
IC C5   N1   *C6   H6        1.3764  123.80 -179.99  115.99  1.0891
IC C6   N1   CM    HM1       1.3715  121.34   -0.02  112.96  1.1130
IC HM1  N1   *CM   HM2       1.1130  112.96  120.64  110.99  1.1153
IC HM1  N1   *CM   HM3       1.1130  112.96 -120.54  111.00  1.1161

RESI BMOU           0.00 ! 5-methoxyuracil, yxu
GROUP
ATOM N1     NG2R61 -0.29
ATOM C2     CG2R63  0.53 ! H81 H82 H83
ATOM O2     OG2D4  -0.44 !    \ | /
ATOM N3     NG2R61 -0.47 !     C8   O4
ATOM H3     HGP1    0.33 !      |   ||
ATOM C4     CG2R63  0.46 !     O7   C4    H3
ATOM O4     OG2D4  -0.46 !       \ /  \  /
ATOM C5     CG2R62  0.19 !        C5   N3
ATOM C6     CG2R62  0.17 !        ||   |
ATOM H6     HGR62   0.16 !     H6-C6   C2
ATOM O7     OG301  -0.43 !         \  / \\
ATOM C8     CG331  -0.02 !          N1   O2
ATOM H81    HGA3    0.09 !           \
ATOM H82    HGA3    0.09 !            \
ATOM H83    HGA3    0.09 !             \
```

```
GROUP
ATOM CM      CG331  -0.27
ATOM HM1     HGA3    0.09
ATOM HM2     HGA3    0.09
ATOM HM3     HGA3    0.09

BOND CM   HM1    CM   HM2   CM   HM3
BOND N1   C2   N1   C6   C2   O2   C2   N3
BOND N3   C4   N3   H3   C4   O4   C4   C5
BOND C5   C6   C5   O7   C6   H6   O7   C8
BOND C8   H81  C8   H82  C8   H83
BOND CM   N1
IMPR C2     N1     N3     O2     C4     C5     N3     O4
DONO H3   N3
ACCE O2   C2
ACCE O4   C4
ACCE O7
IC C2   C6   *N1   CM        1.4081  120.26 -179.99  121.30  1.4745
IC C6   N1   C2    N3        1.3735  120.26   -0.00  116.16  1.3668
IC N3   N1   *C2   O2        1.3668  116.16 -179.97  123.11  1.2275
IC N1   C2   N3    C4        1.4081  116.16    0.02  126.35  1.3811
IC C4   C2   *N3   H3        1.3811  126.35  179.98  116.72  1.0029
IC C2   N3   C4    C5        1.3668  126.35   -0.01  116.71  1.4614
IC C5   N3   *C4   O4        1.4614  116.71 -179.98  117.07  1.2305
IC C5   N1   *C6   H6        1.3845  123.87 -179.99  114.30  1.0865
IC C6   C4   *C5   O7        1.3845  116.65 -179.95  121.43  1.3819
IC C4   C5   O7    C8        1.4614  121.43 -179.99  116.02  1.4278
IC C5   O7   C8    H81       1.3819  116.02  179.97  108.24  1.1126
IC H81  O7   *C8   H82       1.1126  108.24  118.07  111.36  1.1125
IC H81  O7   *C8   H83       1.1126  108.24 -118.06  111.36  1.1130
IC C6   N1   CM    HM1       1.3735  121.30    0.07  112.93  1.1140
IC HM1  N1   *CM   HM2       1.1140  112.93  120.59  111.00  1.1155
IC HM1  N1   *CM   HM3       1.1140  112.93 -120.59  110.97  1.1153

RESI B3MU           0.00 ! 3-methyluracil, UR3, yxu
GROUP
ATOM N1      NG2R61 -0.20
ATOM C2      CG2R63  0.52
ATOM O2      OG2D4  -0.44 !      O4      H31
ATOM N3      NG2R61 -0.32 !      ||     /
ATOM C4      CG2R63  0.50 !      C4    C3U-H32
ATOM O4      OG2D4  -0.48 !     /  \  / \
ATOM C5      CG2R62 -0.22 ! H5-C5   N3   H33
ATOM H5      HGR62   0.09 !    ||   |
ATOM C6      CG2R62  0.31 ! H6-C6   C2
ATOM H6      HGR62   0.11 !     \  / \\
ATOM C3U     CG331  -0.14 !      N1   O2
ATOM H31     HGA3    0.09 !       \
ATOM H32     HGA3    0.09 !        \
ATOM H33     HGA3    0.09 !         \
GROUP
ATOM CM      CG331  -0.27
ATOM HM1     HGA3    0.09
ATOM HM2     HGA3    0.09
ATOM HM3     HGA3    0.09

BOND CM   HM1    CM   HM2   CM   HM3
BOND N1   C2      N1   C6      C2   O2      C2   N3
BOND N3   C4      N3   C3U     C4   O4      C4   C5
BOND C5   C6      C5   H5      C6   H6      C3U  H31
BOND C3U  H32     C3U  H33
BOND CM   N1
IMPR C2     N1     N3     O2      C4     C5     N3     O4
```

```
ACCE O2   C2
ACCE O4   C4
IC C6   C2   *N1   CM        1.3620  119.86  178.92  118.50  1.4763
IC C6   N1   C2    N3        1.3620  119.86    1.43  117.06  1.3999
IC N3   N1   *C2   O2        1.3999  117.06 -179.75  121.38  1.2332
IC N1   C2   N3    C4        1.4089  117.06   -3.27  123.55  1.4034
IC C4   C2   *N3   C3U       1.4034  123.55 -175.78  118.23  1.4798
IC C2   N3   C4    O4        1.3999  123.55 -176.25  120.07  1.2302
IC O4   N3   *C4   C5        1.2302  120.07  179.92  116.55  1.4258
IC C6   C4   *C5   H5        1.3654  119.41 -178.35  120.19  1.0879
IC C5   N1   *C6   H6        1.3654  123.47  179.86  116.13  1.0909
IC C2   N3   C3U   H31       1.3999  118.23 -148.89  112.14  1.1141
IC H31  N3   *C3U  H32       1.1141  112.14  119.69  112.52  1.1143
IC H31  N3   *C3U  H33       1.1141  112.14 -120.01  110.87  1.1131
IC C6   N1   CM    HM1       1.3620  121.62  119.23  110.97  1.1151
IC HM1  N1   *CM   HM2       1.1151  110.97  118.87  110.96  1.1160
IC HM1  N1   *CM   HM3       1.1151  110.97 -120.63  112.82  1.1133

RESI BCYU           0.00 ! 5-cyanomethyl-uracil, yxu
GROUP
ATOM N1     NG2R61 -0.34
ATOM C2     CG2R63  0.51
ATOM O2     OG2D4  -0.41
ATOM N3     NG2R61 -0.46
ATOM H3     HGP1    0.36 !        H71    O4
ATOM C4     CG2R63  0.50 !   _       |      ||
ATOM O4     OG2D4  -0.45 ! N9= C8--C7\   C4    H3
ATOM C5     CG2R62 -0.05 !         |  \ /  \  /
ATOM C6     CG2R62  0.17 !        H73  C5   N3
ATOM H6     HGR62   0.17 !             ||   |
GROUP                    !          H6-C6   C2
ATOM C7     CG321  -0.08 !               \  / \\
ATOM H71    HGA2    0.09 !                N1   O2
ATOM H72    HGA2    0.09 !                 \
ATOM C8     CG1N1   0.36 !                  \
ATOM N9     NG1T1  -0.46 !                   \
GROUP
ATOM CM     CG331  -0.27
ATOM HM1    HGA3    0.09
ATOM HM2    HGA3    0.09
ATOM HM3    HGA3    0.09

BOND CM   HM1    CM   HM2   CM   HM3
BOND N1   C2      N1   C6      C2   O2      C2   N3
BOND N3   H3      N3   C4      C4   O4      C4   C5
BOND C5   C6      C5   C7      C6   H6      C7   C8
BOND C7   H71     C7   H72     C8   N9
BOND CM   N1
IMPR C2   N1   N3   O2        C4   C5   N3   O4
DONO H3   N3
ACCE O2   C2
ACCE O4   C4
ACCE N9   C8
IC C2   C6   *N1   CM        1.4083  120.19  179.96  121.32  1.4736
IC C6   N1   C2    N3        1.3698  120.19   -0.03  116.26  1.3659
IC N3   N1   *C2   O2        1.3659  116.26 -179.97  123.11  1.2288
IC N1   C2   N3    C4        1.4083  116.26    0.04  126.15  1.3741
IC C4   C2   *N3   H3        1.3741  126.15  179.96  116.64  1.0012
IC C2   N3   C4    C5        1.3659  126.15   -0.03  116.65  1.4461
IC C5   N3   *C4   O4        1.4461  116.65  179.98  118.16  1.2283
IC C5   N1   *C6   H6        1.3817  123.18 -179.94  115.89  1.0928
IC C6   C4   *C5   C7        1.3817  117.57  179.96  117.55  1.5067
IC C4   C5   C7    C8        1.4461  117.55  179.90  116.25  1.4702
```

```
IC C8   C5   *C7   H71       1.4702  116.25  121.60  107.96  1.1118
IC C8   C5   *C7   H72       1.4702  116.25 -121.61  107.95  1.1116
IC C5   C7   C8    N9        1.5067  116.25   -0.30  175.85  1.1783
IC C6   N1   CM    HM1       1.3698  121.32   -0.03  112.82  1.1139
IC HM1  N1   *CM   HM2       1.1139  112.82  120.49  111.08  1.1150
IC HM1  N1   *CM   HM3       1.1139  112.82 -120.48  111.11  1.1156

RESI BPSU           0.00 ! Pseudouracil, yxu
GROUP
ATOM C5     CG2R62 -0.17
ATOM C4     CG2R63  0.53
ATOM O4     OG2D4  -0.46 !      O2
ATOM N3     NG2R61 -0.51 !      ||
ATOM H3     HGP1    0.37 !      C2
ATOM C2     CG2R63  0.45 !     /  \
ATOM O2     OG2D4  -0.46 ! H1-N1   N3-H3
ATOM N1     NG2R61 -0.36 !    |     |
ATOM H1     HGP1    0.33 ! H6-C6   C4
ATOM C6     CG2R62  0.10 !     \\ / \\
ATOM H6     HGR62   0.18 !      C5   O4
GROUP                    !        \
ATOM CM     CG331  -0.27 !         \
ATOM HM1    HGA3    0.09 !          \
ATOM HM2    HGA3    0.09
ATOM HM3    HGA3    0.09

BOND CM   HM1    CM   HM2   CM   HM3
BOND C5   C4      C5   C6      C4   O4      C4   N3
BOND N3   C2      N3   H3      C2   O2      C2   N1
BOND N1   C6      N1   H1      C6   H6
BOND CM   C5
IMPR C2   N1   N3   O2        C4   C5   N3   O4
DONO H3   N3
DONO H1   N1
ACCE O2   C2
ACCE O4   C4
IC C4   C6   *C5   CM        1.4537  117.94 -171.05  121.41  1.5007
IC C6   C5   C4    N3        1.3757  117.94    5.61  115.92  1.3767
IC N3   C5   *C4   O4        1.3767  115.92  179.61  126.11  1.2282
IC C5   C4   N3    C2        1.4537  115.92   -3.54  125.83  1.3628
IC C2   C4   *N3   H3        1.3628  125.83 -177.07  116.66  0.9992
IC C4   N3   C2    O2        1.3767  125.83 -179.15  121.69  1.2236
IC O2   N3   *C2   N1        1.2236  121.69  179.72  116.67  1.3788
IC C6   C2   *N1   H1        1.3706  121.29 -179.39  113.63  1.0050
IC N1   C5   *C6   H6        1.3706  122.11 -179.94  119.97  1.0924
IC C6   C5   CM    HM1       1.3757  121.41   86.06  110.75  1.1128
IC HM1  C5   *CM   HM2       1.1128  110.75  119.35  110.91  1.1138
IC HM1  C5   *CM   HM3       1.1128  110.75 -120.77  111.70  1.1128

RESI B1MP           0.00 ! 1-methylpseudouracil, yxu
GROUP
ATOM C5     CG2R62 -0.19
ATOM C4     CG2R63  0.48
ATOM O4     OG2D4  -0.46 !  H11     O2
ATOM N3     NG2R61 -0.49 !     \    ||
ATOM H3     HGP1    0.35 ! H12-C1   C2    H3
ATOM C2     CG2R63  0.52 !     / \ /  \  /
ATOM O2     OG2D4  -0.47 !  H13   N1   N3
ATOM N1     NG2R61 -0.27 !        |     |
ATOM C6     CG2R62  0.17 !     H6-C6   C4
ATOM H6     HGR62   0.16 !         \\ / \\
ATOM C1     CG331  -0.07 !          C5   O4
ATOM H11    HGA3    0.09 !            \
```

```
ATOM H12    HGA3    0.09 !            \
ATOM H13    HGA3    0.09 !             \
GROUP
ATOM CM     CG331  -0.27
ATOM HM1    HGA3    0.09
ATOM HM2    HGA3    0.09
ATOM HM3    HGA3    0.09

BOND CM   HM1    CM   HM2   CM   HM3
BOND C5   C4   C5   C6   C4   O4   C4   N3
BOND N3   C2   N3   H3   C2   O2   C2   N1
BOND N1   C6   N1   C1   C6   H6   C1   H11
BOND C1   H12  C1   H13
BOND CM   C5
IMPR C4     C5     N3     O4     C2     N3     N1     O2
DONO H3   N3
ACCE O2   C2
ACCE O4   C4
IC C4   C6   *C5   CM        1.4487  117.97 -171.17  121.54  1.5008
IC C6   C5   C4    N3        1.3758  117.97    5.57  116.02  1.3739
IC N3   C5   *C4   O4        1.3739  116.02  179.59  126.00  1.2284
IC C5   C4   N3    C2        1.4487  116.02   -4.18  126.12  1.3650
IC C2   C4   *N3   H3        1.3650  126.12 -176.32  116.77  1.0013
IC C4   N3   C2    N1        1.3739  126.12    1.17  116.63  1.4029
IC N1   N3   *C2   O2        1.4029  116.63 -179.55  120.77  1.2277
IC N1   C5   *C6   H6        1.3703  123.06  179.16  119.90  1.0902
IC C6   C2   *N1   C1        1.3703  119.97 -179.64  117.52  1.4721
IC C2   N1   C1    H11       1.4029  117.52  179.60  112.53  1.1131
IC H11  N1   *C1   H12       1.1131  112.53  120.50  111.03  1.1145
IC H11  N1   *C1   H13       1.1131  112.53 -120.51  110.98  1.1154
IC C6   C5   CM    HM1       1.3758  121.54  -36.19  111.72  1.1129
IC HM1  C5   *CM   HM2       1.1129  111.72  120.86  110.81  1.1129
IC HM1  C5   *CM   HM3       1.1129  111.72 -119.83  110.91  1.1135

RESI B3MP           0.00 ! 3-methylpseudouracil, yxu
GROUP
ATOM C5     CG2R62 -0.20
ATOM C4     CG2R63  0.63
ATOM O4     OG2D4  -0.47 !      O2    H31
ATOM N3     NG2R61 -0.40 !      ||     |
ATOM C2     CG2R63  0.53 !      C2    C3 -H32
ATOM O2     OG2D4  -0.47 !     /  \  /  \
ATOM N1     NG2R61 -0.32 ! H1-N1   N3   H33
ATOM H1     HGP1    0.31 !    |     |
ATOM C6     CG2R62  0.03 ! H6-C6   C4
ATOM H6     HGR62   0.19 !     \\ / \\
ATOM C3     CG331  -0.10 !      C5   O4
ATOM H31    HGA3    0.09 !        \
ATOM H32    HGA3    0.09 !         \
ATOM H33    HGA3    0.09 !          \
GROUP
ATOM CM     CG331  -0.27
ATOM HM1    HGA3    0.09
ATOM HM2    HGA3    0.09
ATOM HM3    HGA3    0.09

BOND CM   HM1    CM   HM2   CM   HM3
BOND C5   C4      C5   C6      C4   O4      C4   N3
BOND N3   C2      N3   C3      C2   O2      C2   N1
BOND N1   C6      N1   H1      C6   H6      C3   H31
BOND C3   H32     C3   H33
BOND CM   C5
IMPR C4     C5     N3     O4       C2     N3     N1     O2
```

```
DONO H1   N1
ACCE O2   C2
ACCE O4   C4
IC C4   C6   *C5   CM        1.4487  117.97 -171.17  121.54  1.5008
IC C6   C5   C4    N3        1.3758  117.97    5.57  116.02  1.3739
IC N3   C5   *C4   O4        1.3739  116.02  179.59  126.00  1.2284
IC C5   C4   N3    C2        1.4487  116.02   -4.18  126.12  1.3650
IC C2   C4   *N3   C3        1.3650  126.12 -176.32  116.77  1.4913
IC C4   N3   C2    N1        1.3739  126.12    1.17  116.63  1.4029
IC N1   N3   *C2   O2        1.4029  116.63 -179.55  120.77  1.2277
IC C6   C2   *N1   H1        1.3876  115.89  180.00  110.87  1.0071
IC N1   C5   *C6   H6        1.3876  120.75 -179.86  120.66  1.0940
IC C4   N3   C3    H31       1.4173  107.85  178.46  109.78  1.1168
IC H31  N3   *C3   H32       1.1168  109.78  115.65  109.98  1.1167
IC H31  N3   *C3   H33       1.1168  109.78 -122.11  118.22  1.1093
IC C6   C5   CM    HM1       1.3880  119.56  163.48  110.88  1.1142
IC HM1  C5   *CM   HM2       1.1142  110.88  119.19  110.77  1.1130
IC HM1  C5   *CM   HM3       1.1142  110.88 -120.00  111.70  1.1127

RESI BSAU           1.00 ! 5-aminomethyl-2-thiouracil, yxu
GROUP
ATOM N1     NG2R61 -0.26
ATOM C2     CG2R63  0.29
ATOM S2     SG2D1  -0.22
ATOM N3     NG2R61 -0.56
ATOM H3     HGP1    0.40
ATOM C4     CG2R63  0.39
ATOM O4     OG2D4  -0.41 !     H81  H71  O4
ATOM C5     CG2R62 -0.03 !  (+) |     |   ||
ATOM C6     CG2R62  0.18 ! H82-N8 --C7   C4    H3
ATOM H6     HGR62   0.22 !      |   / \ /  \  /
GROUP                    !    H83 H72  C5   N3
ATOM C7     CG324   0.21 !             ||   |
ATOM H71    HGA2    0.05 !          H6-C6   C2
ATOM H72    HGA2    0.05 !               \  / \\
ATOM N8     NG3P3  -0.30 !                N1   S2
ATOM H81    HGP2    0.33 !                 \
ATOM H82    HGP2    0.33 !                  \
ATOM H83    HGP2    0.33 !                   \
GROUP
ATOM CM     CG331  -0.27
ATOM HM1    HGA3    0.09
ATOM HM2    HGA3    0.09
ATOM HM3    HGA3    0.09

BOND CM   HM1    CM   HM2   CM   HM3
BOND N1   C2   N1   C6   C2   S2   C2   N3
BOND N3   C4   N3   H3   C4   O4   C4   C5
BOND C5   C6   C5   C7   C6   H6   C7   N8
BOND C7   H71  C7   H72  N8   H81  N8   H82
BOND N8   H83
BOND CM   N1
IMPR C2     N1     N3     S2     C4     C5     N3     O4
DONO H3   N3
DONO H81  N8
DONO H82  N8
DONO H83  N8
ACCE O4   C4
ACCE S2   C2
IC C2   C6   *N1   CM        1.4173  121.68  179.99  120.23  1.4740
IC C6   N1   C2    N3        1.3689  121.68    0.20  113.27  1.3840
IC N3   N1   *C2   S2        1.3840  113.27  179.95  126.61  1.6510
IC N1   C2   N3    C4        1.4173  113.27    0.09  127.91  1.3755
```

```
IC C4   C2   *N3   H3        1.3755  127.91  179.53  116.52  1.0073
IC C2   N3   C4    C5        1.3840  127.91   -0.59  116.46  1.4367
IC C5   N3   *C4   O4        1.4367  116.46 -179.89  118.60  1.2260
IC C5   N1   *C6   H6        1.3824  123.30 -179.67  113.69  1.0893
IC C6   C4   *C5   C7        1.3824  117.37  179.29  111.51  1.4938
IC C4   C5   C7    N8        1.4367  111.51  177.18  114.82  1.4899
IC N8   C5   *C7   H71       1.4899  114.82  120.93  108.57  1.1039
IC N8   C5   *C7   H72       1.4899  114.82 -120.28  108.53  1.1047
IC C5   C7   N8    H81       1.4938  114.82 -178.68  108.22  1.0431
IC H81  C7   *N8   H82       1.0431  108.22  118.15  110.77  1.0407
IC H81  C7   *N8   H83       1.0431  108.22 -118.44  111.02  1.0400
IC C6   N1   CM    HM1       1.3689  120.23   -0.39  113.48  1.1123
IC HM1  N1   *CM   HM2       1.1123  113.48  120.59  111.12  1.1164
IC HM1  N1   *CM   HM3       1.1123  113.48 -120.53  111.12  1.1164

RESI B5AU           1.00 ! 5-methylaminomethyluracil, yxu
GROUP
ATOM N1     NG2R61 -0.34
ATOM C2     CG2R63  0.51
ATOM O2     OG2D4  -0.41
ATOM N3     NG2R61 -0.46
ATOM H3     HGP1    0.36
ATOM C4     CG2R63  0.50
ATOM O4     OG2D4  -0.45
ATOM C5     CG2R62 -0.05 !    HA3 HN1  HC2  O4
ATOM C6     CG2R62  0.17 !      |   |(+) |    ||
ATOM H6     HGR62   0.17 ! HA2-CA -N --C    C4    H3
GROUP                      !      |   |   / \ /  \  /
ATOM C      CG324   0.20 !    HA1 HN2 HC1 C5   N3
ATOM HC1    HGA2    0.09 !                ||    |
ATOM HC2    HGA2    0.09 !             H6-C6   C2
ATOM N      NG3P2  -0.52 !                 \  / \\
ATOM HN1    HGP2    0.38 !                  N1   O2
ATOM HN2    HGP2    0.38 !                   \
ATOM CA     CG334   0.11 !                    \
ATOM HA1    HGA3    0.09 !                     \
ATOM HA2    HGA3    0.09
ATOM HA3    HGA3    0.09
GROUP
ATOM CM     CG331  -0.27
ATOM HM1    HGA3    0.09
ATOM HM2    HGA3    0.09
ATOM HM3    HGA3    0.09

BOND CM   HM1    CM   HM2   CM   HM3
BOND N1   C2    N1   C6     C2   O2     C2   N3
BOND N3   C4    N3   H3     C4   O4     C4   C5
BOND C5   C6    C5   C      C6   H6     C    N
BOND C    HC1   C    HC2    N    CA     N    HN2
BOND N    HN1   CA   HA1    CA   HA2    CA   HA3
BOND CM   N1
IMPR C2     N1     N3     O2      C4     C5     N3     O4
DONO H3   N3
DONO HN1  N
DONO HN2  N
ACCE O2   C2
ACCE O4   C4
IC C2   C6   *N1   CM        1.4088  120.45  180.00  121.27  1.4731
IC C6   N1   C2    N3        1.3645  120.45    0.00  116.11  1.3663
IC N3   N1   *C2   O2        1.3663  116.11  180.00  123.18  1.2264
IC N1   C2   N3    C4        1.4088  116.11    0.00  125.97  1.3727
IC C4   C2   *N3   H3        1.3727  125.97  180.00  116.16  1.0040
IC C2   N3   C4    C5        1.3663  125.97    0.00  116.93  1.4424
```

```
IC C5   N3   *C4   O4        1.4424  116.93  180.00  118.59  1.2259
IC C5   N1   *C6   H6        1.3837  123.08  180.00  114.33  1.0912
IC C6   C4   *C5   C         1.3837  117.45  180.00  111.19  1.4800
IC C4   C5   C     N         1.4424  111.19  180.00  110.83  1.4922
IC N    C5   *C    HC1       1.4922  110.83  118.08  110.47  1.1070
IC N    C5   *C    HC2       1.4922  110.83 -118.08  110.47  1.1070
IC C5   C    N     CA        1.4800  110.83  180.00  113.93  1.5096
IC CA   C    *N    HN2       1.5096  113.93  122.06  109.44  1.0123
IC CA   C    *N    HN1       1.5096  113.93 -122.06  109.44  1.0123
IC C    N    CA    HA1       1.4922  113.93  180.00  107.40  1.1114
IC HA1  N    *CA   HA2       1.1114  107.40  119.95  107.42  1.1104
IC HA1  N    *CA   HA3       1.1114  107.40 -119.95  107.42  1.1104
IC C6   N1   CM    HM1       1.3645  121.27    0.00  113.52  1.1131
IC HM1  N1   *CM   HM2       1.1131  113.52  120.78  111.06  1.1164
IC HM1  N1   *CM   HM3       1.1131  113.52 -120.78  111.06  1.1164

RESI BU8U           1.00 ! 5-methylaminomethyl-2-thiouracil, ESU, yxu
GROUP
ATOM N1     NG2R61 -0.26
ATOM C2     CG2R63  0.29
ATOM S2     SG2D1  -0.22
ATOM N3     NG2R61 -0.56
ATOM H3     HGP1    0.40
ATOM C4     CG2R63  0.39
ATOM O4     OG2D4  -0.41
ATOM C5     CG2R62 -0.03 !    HA3 HN1  HC2  O4
ATOM C6     CG2R62  0.18 !      |   |(+) |    ||
ATOM H6     HGR62   0.22 ! HA2-CA -N --C    C4    H3
GROUP                    !      |   |   / \ /  \  /
ATOM C      CG324   0.20 !    HA1 HN2 HC1 C5   N3
ATOM HC1    HGA2    0.09 !                ||    |
ATOM HC2    HGA2    0.09 !             H6-C6   C2
ATOM N      NG3P2  -0.52 !                 \  / \\
ATOM HN1    HGP2    0.38 !                  N1   S2
ATOM HN2    HGP2    0.38 !                   \
ATOM CA     CG334   0.11 !                    \
ATOM HA1    HGA3    0.09 !                     \
ATOM HA2    HGA3    0.09
ATOM HA3    HGA3    0.09
GROUP
ATOM CM     CG331  -0.27
ATOM HM1    HGA3    0.09
ATOM HM2    HGA3    0.09
ATOM HM3    HGA3    0.09

BOND CM   HM1    CM   HM2   CM   HM3
BOND N1   C2     N1   C6     C2   S2     C2   N3
BOND N3   C4     N3   H3     C4   O4     C4   C5
BOND C5   C6     C5   C      C6   H6     C    N
BOND C    HC1    C    HC2    N    CA     N    HN2
BOND N    HN1    CA   HA1    CA   HA2    CA   HA3
BOND CM   N1
IMPR C2     N1     N3     S2     C4     C5     N3     O4
DONO H3   N3
DONO HN1  N
DONO HN2  N
ACCE O4   C4
ACCE S2   C2
IC C2   C6   *N1   CM        1.4181  121.74 -178.37  118.85  1.4745
IC C6   N1   C2    N3        1.3565  121.74    3.29  113.31  1.3904
IC N3   N1   *C2   S2        1.3904  113.31  178.81  126.63  1.6499
IC N1   C2   N3    C4        1.4181  113.31    4.00  127.12  1.3734
IC C4   C2   *N3   H3        1.3734  127.12  174.49  116.22  1.0060
```

```
IC C2   N3   C4    C5        1.3904  127.12   -7.92  116.22  1.4286
IC C5   N3   *C4   O4        1.4286  116.22  175.46  119.23  1.2349
IC C5   N1   *C6   H6        1.3752  122.95 -175.60  115.15  1.0911
IC C6   C4   *C5   C         1.3752  118.11  170.44  116.94  1.4668
IC C4   C5   C     N         1.4286  116.94  -68.03  104.70  1.4945
IC N    C5   *C    HC1       1.4945  104.70  116.56  113.18  1.1039
IC N    C5   *C    HC2       1.4945  104.70 -115.87  110.70  1.0994
IC C5   C    N     CA        1.4668  104.70 -175.38  114.02  1.4965
IC CA   C    *N    HN2       1.4965  114.02  125.08  109.53  1.0089
IC CA   C    *N    HN1       1.4965  114.02 -122.57  106.66  1.0182
IC C    N    CA    HA1       1.4945  114.02  169.97  106.93  1.1116
IC HA1  N    *CA   HA2       1.1116  106.93  120.38  106.54  1.1118
IC HA1  N    *CA   HA3       1.1116  106.93 -119.92  106.49  1.1127
IC C6   N1   CM    HM1       1.3565  118.85  -12.37  112.79  1.1162
IC HM1  N1   *CM   HM2       1.1162  112.79  120.74  111.48  1.1138
IC HM1  N1   *CM   HM3       1.1162  112.79 -118.84  110.97  1.1155

RESI BSEU           1.00 ! 5-methylaminomethyl-2-selenouracil, yxu
GROUP
ATOM N1     NG2R61 -0.26
ATOM C2     CG2R63  0.25
ATOM SE2    SEGD1  -0.16
ATOM N3     NG2R61 -0.56
ATOM H3     HGP1    0.40
ATOM C4     CG2R63  0.39
ATOM O4     OG2D4  -0.41
ATOM C5     CG2R62 -0.05 !    HA3 HN1  HC2  O4
ATOM C6     CG2R62  0.18 !      |   |(+) |    ||
ATOM H6     HGR62   0.22 ! HA2-CA -N --C    C4    H3
GROUP                      !      |   |   / \ /  \  /
ATOM C      CG324   0.20 !    HA1 HN2 HC1 C5   N3
ATOM HC1    HGA2    0.09 !                ||    |
ATOM HC2    HGA2    0.09 !             H6-C6   C2
ATOM N      NG3P2  -0.52 !                   \  / \\
ATOM HN1    HGP2    0.38 !                    N1   SE2
ATOM HN2    HGP2    0.38 !                     \
ATOM CA     CG334   0.11 !                      \
ATOM HA1    HGA3    0.09 !                       \
ATOM HA2    HGA3    0.09
ATOM HA3    HGA3    0.09
GROUP
ATOM CM     CG331  -0.27
ATOM HM1    HGA3    0.09
ATOM HM2    HGA3    0.09
ATOM HM3    HGA3    0.09

BOND CM   HM1    CM   HM2   CM   HM3
BOND N1   C2     N1   C6     C2   SE2    C2   N3
BOND N3   C4     N3   H3     C4   O4     C4   C5
BOND C5   C6     C5   C      C6   H6     C    N
BOND C    HC1    C    HC2    N    CA     N    HN2
BOND N    HN1    CA   HA1    CA   HA2    CA   HA3
BOND CM   N1
IMPR C2     N1     N3     SE2     C4     C5     N3     O4
DONO H3   N3
DONO HN1  N
DONO HN2  N
ACCE O4   C4
IC C6   C2   *N1   CM        1.3708  121.62  176.61  118.35  1.4758
IC C6   N1   C2    N3        1.3708  121.62    4.00  113.33  1.3871
IC N3   N1   *C2   SE2       1.3871  113.33  179.26  126.61  1.8506
IC N1   C2   N3    C4        1.4180  113.33    1.56  127.74  1.3746
IC C4   C2   *N3   H3        1.3746  127.74  176.56  116.54  1.0097
```

```
IC C2   N3   C4    C5        1.3871  127.74   -4.73  116.30  1.4394
IC C5   N3   *C4   O4        1.4394  116.30  175.87  118.96  1.2279
IC C5   N1   *C6   H6        1.3799  123.02 -175.89  114.65  1.0920
IC C6   C4   *C5   C         1.3799  117.66  175.37  116.17  1.4810
IC C4   C5   C     N         1.4394  116.17  -63.01  105.66  1.5102
IC N    C5   *C    HC1       1.5102  105.66  116.77  111.73  1.1061
IC N    C5   *C    HC2       1.5102  105.66 -116.88  112.01  1.1028
IC C5   C    N     CA        1.4810  105.66 -173.07  117.38  1.5074
IC CA   C    *N    HN2       1.5074  117.38  125.72  109.32  1.0096
IC CA   C    *N    HN1       1.5074  117.38 -122.87  105.99  1.0227
IC C    N    CA    HA1       1.5102  117.38  169.59  106.80  1.1147
IC HA1  N    *CA   HA2       1.1147  106.80  119.89  107.38  1.1112
IC HA1  N    *CA   HA3       1.1147  106.80 -119.40  107.92  1.1129
IC C6   N1   CM    HM1       1.3708  119.95  -19.97  113.44  1.1151
IC HM1  N1   *CM   HM2       1.1151  113.44  121.22  111.08  1.1168
IC HM1  N1   *CM   HM3       1.1151  113.44 -118.98  111.52  1.1161

RESI B5DU           0.00 ! 5-carboxymethylaminomethyluracil, yxu
GROUP
ATOM N1     NG2R61 -0.34
ATOM C2     CG2R63  0.51
ATOM O2     OG2D4  -0.41
ATOM N3     NG2R61 -0.46
ATOM H3     HGP1    0.36
ATOM C4     CG2R63  0.50
ATOM O4     OG2D4  -0.45
ATOM C5     CG2R62 -0.05 !  O11  O12 (-)
ATOM C6     CG2R62  0.17 !    \\ /
ATOM H6     HGR62   0.17 !     C10
GROUP                    !      |   H81 H72  O4
ATOM C7     CG324   0.04 !      |   |(+) |   ||
ATOM H71    HGA2    0.09 ! H92-C9 -N8 -C7   C4    H3
ATOM H72    HGA2    0.09 !      |   |   / \ /  \  /
ATOM N8     NG3P2  -0.34 !    H91 H82 H71 C5   N3
ATOM H81    HGP2    0.34 !                ||   |
ATOM H82    HGP2    0.34 !             H6-C6   C2
ATOM C9     CG324  -0.01 !                  \  / \\
ATOM H91    HGA2    0.09 !                   N1   O2
ATOM H92    HGA2    0.09 !                    \
ATOM C10    CG2O3   0.59 !                     \
ATOM O11    OG2D2  -0.66 !                      \
ATOM O12    OG2D2  -0.66
GROUP
ATOM CM     CG331  -0.27
ATOM HM1    HGA3    0.09
ATOM HM2    HGA3    0.09
ATOM HM3    HGA3    0.09

BOND CM   HM1    CM   HM2   CM   HM3
BOND N1   C2    N1   C6     C2   O2     C2   N3
BOND N3   C4    N3   H3     C4   O4     C4   C5
BOND C5   C6    C5   C7     C6   H6     C7   H71
BOND C7   H72   C7   N8     N8   H82    N8   C9
BOND N8   H81   C9   H91    C9   H92    C9   C10
BOND C10  O11   C10  O12
BOND CM   N1
IMPR C2     N1     N3     O2     C4     C5     N3     O4     C10    O11    O12    C9
DONO H3   N3
DONO H81  N8
DONO H82  N8
ACCE O2   C2
ACCE O4   C4
ACCE O11  C10
```

```
ACCE O12  C10
IC C2   C6   *N1   CM        1.4088  120.32  179.58  121.21  1.4740
IC C6   N1   C2    N3        1.3698  120.32   -0.71  116.25  1.3673
IC N3   N1   *C2   O2        1.3673  116.25 -179.71  123.19  1.2273
IC N1   C2   N3    C4        1.4088  116.25   -0.42  125.93  1.3732
IC C4   C2   *N3   H3        1.3732  125.93 -178.48  116.18  1.0023
IC C2   N3   C4    C5        1.3673  125.93    2.02  116.67  1.4458
IC C5   N3   *C4   O4        1.4458  116.67 -177.78  118.43  1.2294
IC C5   N1   *C6   H6        1.3770  122.95  179.91  116.16  1.0927
IC C6   C4   *C5   C7        1.3770  117.83 -179.51  118.22  1.4786
IC C4   C5   C7    N8        1.4458  118.22   64.18  107.17  1.4906
IC N8   C5   *C7   H71       1.4906  107.17 -117.25  111.73  1.1034
IC H71  C5   *C7   H72       1.1034  111.73 -126.68  111.47  1.1067
IC C5   C7   N8    C9        1.4786  107.17  167.84  114.67  1.5272
IC C9   C7   *N8   H82       1.5272  114.67 -114.37  107.93  1.0202
IC H82  C7   *N8   H81       1.0202  107.93 -117.20  111.39  1.0082
IC C7   N8   C9    C10       1.4906  114.67  -86.99  110.07  1.5367
IC C10  N8   *C9   H91       1.5367  110.07  120.98  106.99  1.0960
IC H91  N8   *C9   H92       1.0960  106.99  120.09  106.41  1.0969
IC N8   C9   C10   O11       1.5272  110.07  176.53  114.59  1.2514
IC O11  C9   *C10  O12       1.2514  114.59  179.85  116.80  1.2615
IC C6   N1   CM    HM1       1.3698  121.21    0.80  112.91  1.1132
IC HM1  N1   *CM   HM2       1.1132  112.91  120.43  111.06  1.1165
IC HM1  N1   *CM   HM3       1.1132  112.91 -120.64  111.13  1.1155

RESI BSCU            0.00 ! 5-carboxymethylaminomethyl-2-thiouracil, yxu
!RING 6 N1 C2 N3 C4 C5 C6
GROUP
ATOM N1     NG2R61 -0.26
ATOM C2     CG2R63  0.29
ATOM S2     SG2D1  -0.22
ATOM N3     NG2R61 -0.56
ATOM H3     HGP1    0.40
ATOM C4     CG2R63  0.39
ATOM O4     OG2D4  -0.41
ATOM C5     CG2R62 -0.03 !  O11  O12 (-)
ATOM C6     CG2R62  0.18 !    \\ /
ATOM H6     HGR62   0.22 !     C10
GROUP                    !     |   H82 H72  O4
ATOM C7     CG324   0.04 !     |   |(+) |    ||
ATOM H71    HGA2    0.09 ! H92-C9 -N8 -C7   C4    H3
ATOM H72    HGA2    0.09 !     |   |   / \ /  \  /
ATOM N8     NG3P2  -0.34 !    H91 H81 H71 C5   N3
ATOM H81    HGP2    0.34 !                ||   |
ATOM H82    HGP2    0.34 !             H6-C6   C2
ATOM C9     CG324  -0.01 !                 \  / \\
ATOM H91    HGA2    0.09 !                  N1   S2
ATOM H92    HGA2    0.09 !                   \
ATOM C10    CG2O3   0.59 !                    \
ATOM O11    OG2D2  -0.66 !                     \
ATOM O12    OG2D2  -0.66
GROUP
ATOM CM     CG331  -0.27
ATOM HM1    HGA3    0.09
ATOM HM2    HGA3    0.09
ATOM HM3    HGA3    0.09

BOND CM   HM1    CM   HM2   CM   HM3
BOND N1   C2     N1   C6      C2   S2      C2   N3
BOND N3   C4     N3   H3      C4   O4      C4   C5
BOND C5   C6     C5   C7      C6   H6      C7   H71
BOND C7   H72    C7   N8      N8   H82     N8   C9
BOND N8   H81    C9   H91     C9   H92     C9   C10
```

```
BOND C10  O11    C10  O12
BOND CM   N1
IMPR C2     N1     N3     S2      C4     C5     N3     O4     C10    O11    O12    C9
DONO H3   N3
DONO H81  N8
DONO H82  N8
ACCE O4   C4
ACCE S2   C2
ACCE O11  C10
ACCE O12  C10
IC C2   C6   *N1   CM        1.4172  121.49  179.25  120.31  1.4736
IC C6   N1   C2    N3        1.3730  121.49   -1.35  113.52  1.3850
IC N3   N1   *C2   S2        1.3850  113.52 -179.64  126.65  1.6515
IC N1   C2   N3    C4        1.4172  113.52   -0.46  127.80  1.3766
IC C4   C2   *N3   H3        1.3766  127.80 -178.26  116.61  1.0061
IC C2   N3   C4    C5        1.3850  127.80    3.14  116.12  1.4403
IC C5   N3   *C4   O4        1.4403  116.12 -177.88  118.62  1.2294
IC C5   N1   *C6   H6        1.3764  123.19  178.07  116.05  1.0936
IC C6   C4   *C5   C7        1.3764  117.77 -176.62  117.66  1.4763
IC C4   C5   C7    N8        1.4403  117.66   69.47  106.21  1.4923
IC N8   C5   *C7   H71       1.4923  106.21 -117.36  112.17  1.1038
IC H71  C5   *C7   H72       1.1038  112.17 -126.92  111.14  1.1055
IC C5   C7   N8    C9        1.4763  106.21  158.35  114.62  1.5273
IC C9   C7   *N8   H82       1.5273  114.62 -114.59  107.51  1.0177
IC H82  C7   *N8   H81       1.0177  107.51 -116.51  111.12  1.0069
IC C7   N8   C9    C10       1.4923  114.62  -81.70  110.21  1.5386
IC C10  N8   *C9   H91       1.5386  110.21  121.16  106.98  1.0953
IC H91  N8   *C9   H92       1.0953  106.98  120.02  106.32  1.0971
IC N8   C9   C10   O11       1.5273  110.21  177.21  114.51  1.2501
IC O11  C9   *C10  O12       1.2501  114.51  179.99  117.00  1.2620
IC C6   N1   CM    HM1       1.3730  120.31 -118.47  111.30  1.1151
IC HM1  N1   *CM   HM2       1.1151  111.30  120.48  112.85  1.1137
IC HM1  N1   *CM   HM3       1.1151  111.30 -119.56  111.13  1.1161

RESI BIAU           1.00 ! 5-(isopentenylaminomethyl)uracil, yxu
GROUP
ATOM N1     NG2R61 -0.34
ATOM C2     CG2R63  0.51
ATOM O2     OG2D4  -0.41
ATOM N3     NG2R61 -0.46
ATOM H3     HGP1    0.36
ATOM C4     CG2R63  0.50
ATOM O4     OG2D4  -0.45
ATOM C5     CG2R62 -0.05
ATOM C6     CG2R62  0.17
ATOM H6     HGR62   0.17 !    H132 H133
GROUP                    !      \ /
ATOM C7     CG324   0.20 ! H131-C13  H121 H122
ATOM H71    HGA2    0.09 !       |    | /
ATOM H72    HGA2    0.09 !       C11-C12-H123
ATOM N8     NG3P2  -0.52 !      //
ATOM H82    HGP2    0.38 ! H10-C10
ATOM H81    HGP2    0.38 !     |  H81  H72  O4
ATOM C9     CG324   0.20 !     |   |(+) |   ||
ATOM H91    HGA2    0.09 ! H92-C9 -N8 -C7   C4    H3
ATOM H92    HGA2    0.09 !     |   |   / \ /   \  /
GROUP                    !    H91 H82 H71 C5   N3
ATOM C10    CG2D1  -0.15 !                ||   |
ATOM H10    HGA4    0.15 !             H6-C6   C2
GROUP                    !                 \  / \\
ATOM C11    CG2D1   0.00 !                  N1   O2
ATOM C12    CG331  -0.27 !                   \
ATOM H121   HGA3    0.09 !                    \
```

```
ATOM H122   HGA3    0.09 !                      \
ATOM H123   HGA3    0.09
GROUP                    !  !!!! PATCH 5UNI for the neutral amino form
ATOM C13    CG331  -0.27
ATOM H131   HGA3    0.09
ATOM H132   HGA3    0.09
ATOM H133   HGA3    0.09
GROUP
ATOM CM     CG331  -0.27
ATOM HM1    HGA3    0.09
ATOM HM2    HGA3    0.09
ATOM HM3    HGA3    0.09

BOND CM   HM1    CM   HM2   CM   HM3
BOND N1   C2     N1   C6      C2   O2      C2   N3
BOND N3   C4     N3   H3      C4   O4      C4   C5
BOND C5   C6     C5   C7      C6   H6      C7   H71
BOND C7   H72    C7   N8      C9   C10     C9   H91
BOND C9   H92    C9   N8      C10  H10     C10  C11
BOND N8   H81    N8   H82     C11  C12     C11  C13
BOND C12  H121   C12  H122    C12  H123    C13  H131
BOND C13  H132   C13  H133
BOND CM   N1
IMPR C2     N1     N3     O2     C4     C5     N3     O4
DONO H3   N3
DONO H81  N8
DONO H82  N8
ACCE O2   C2
ACCE O4   C4
IC C2   C6   *N1   CM        1.4112  120.54 -178.74  120.81  1.4754
IC C6   N1   C2    N3        1.3664  120.54    1.24  116.04  1.3691
IC N3   N1   *C2   O2        1.3691  116.04 -179.75  123.40  1.2232
IC N1   C2   N3    C4        1.4112  116.04   -0.94  125.87  1.3731
IC C4   C2   *N3   H3        1.3731  125.87  178.45  115.68  1.0026
IC C2   N3   C4    C5        1.3691  125.87   -0.61  116.81  1.4457
IC C5   N3   *C4   O4        1.4457  116.81 -177.17  118.77  1.2278
IC C5   N1   *C6   H6        1.3774  122.93 -178.86  115.32  1.0922
IC C6   C4   *C5   C7        1.3774  117.78  177.56  117.39  1.4779
IC C4   C5   C7    N8        1.4457  117.39   56.25  109.55  1.5056
IC N8   C5   *C7   H71       1.5056  109.55  118.28  112.04  1.1052
IC H71  C5   *C7   H72       1.1052  112.04  124.85  109.99  1.1055
IC C5   C7   N8    C9        1.4779  109.55   69.89  120.90  1.5195
IC C9   C7   *N8   H81       1.5195  120.90  126.55  109.20  1.0095
IC C9   C7   *N8   H82       1.5195  120.90 -121.38  106.25  1.0194
IC C7   N8   C9    C10       1.5056  120.90  -76.77  111.91  1.4996
IC C10  N8   *C9   H91       1.4996  111.91  121.70  105.82  1.1038
IC C10  N8   *C9   H92       1.4996  111.91 -122.99  105.03  1.1046
IC N8   C9   C10   C11       1.5195  111.91 -105.12  127.59  1.3483
IC C11  C9   *C10  H10       1.3483  127.59 -174.52  115.90  1.0999
IC C9   C10  C11   C12       1.4996  127.59  178.63  120.29  1.5040
IC C12  C10  *C11  C13       1.5040  120.29 -179.61  125.90  1.5060
IC C10  C11  C12   H121      1.3483  120.29  113.94  110.69  1.1115
IC H121 C11  *C12  H122      1.1115  110.69  118.22  110.87  1.1127
IC H121 C11  *C12  H123      1.1115  110.69 -120.91  113.78  1.1119
IC C10  C11  C13   H131      1.3483  125.90 -123.82  110.03  1.1134
IC H131 C11  *C13  H132      1.1134  110.03  120.73  115.49  1.1092
IC H131 C11  *C13  H133      1.1134  110.03 -117.15  110.54  1.1118
IC C6   N1   CM    HM1       1.3664  120.81  -21.08  113.02  1.1134
IC HM1  N1   *CM   HM2       1.1134  113.02  121.01  111.16  1.1160
IC HM1  N1   *CM   HM3       1.1134  113.02 -119.81  111.64  1.1167

RESI BISU           1.00 ! 5-(isopentenylaminomethyl)-2-thiouracil, yxu
GROUP
```

```
ATOM N1      NG2R61 -0.26
ATOM C2      CG2R63  0.29
ATOM S2      SG2D1  -0.22
ATOM N3      NG2R61 -0.56
ATOM H3      HGP1    0.40
ATOM C4      CG2R63  0.39
ATOM O4      OG2D4  -0.41
ATOM C5      CG2R62 -0.03
ATOM C6      CG2R62  0.18
ATOM H6      HGR62   0.22 !    H132 H133
GROUP                     !      \ /
ATOM C7      CG324   0.20 ! H131-C13  H121 H122
ATOM H71     HGA2    0.09 !       |     | /
ATOM H72     HGA2    0.09 !       C11-C12-H123
ATOM N8      NG3P2  -0.52 !      //
ATOM H82     HGP2    0.38 ! H10-C10
ATOM H81     HGP2    0.38 !     |  H81  H72  O4
ATOM C9      CG324   0.20 !     |   |(+) |   ||
ATOM H91     HGA2    0.09 ! H92-C9 -N8 -C7   C4    H3
ATOM H92     HGA2    0.09 !     |   |   / \ /  \  /
GROUP                     !    H91 H82 H71 C5   N3
ATOM C10     CG2D1  -0.15 !                ||   |
ATOM H10     HGA4    0.15 !             H6-C6   C2
GROUP                     !                  \  / \\
ATOM C11     CG2D1   0.00 !                  N1   S2
ATOM C12     CG331  -0.27 !                   \
ATOM H121    HGA3    0.09 !                    \
ATOM H122    HGA3    0.09 !                     \
ATOM H123    HGA3    0.09
GROUP                     !  !!!! PATCH 5UNI for the neutral amino form
ATOM C13     CG331  -0.27
ATOM H131    HGA3    0.09
ATOM H132    HGA3    0.09
ATOM H133    HGA3    0.09
GROUP
ATOM CM      CG331  -0.27
ATOM HM1     HGA3    0.09
ATOM HM2     HGA3    0.09
ATOM HM3     HGA3    0.09

BOND CM   HM1    CM   HM2   CM   HM3
BOND N1   C2      N1   C6      C2   S2       C2   N3
BOND N3   C4      N3   H3      C4   O4       C4   C5
BOND C5   C6      C5   C7      C6   H6       C7   H71
BOND C7   H72     C7   N8      C9   C10      C9   H91
BOND C9   H92     C9   N8      C10  H10      C10  C11
BOND N8   H81     N8   H82     C11  C12      C11  C13
BOND C12  H121    C12  H122    C12  H123     C13  H131
BOND C13  H132    C13  H133
BOND CM   N1
IMPR C2     N1     N3     S2     C4     C5     N3     O4
DONO H3   N3
DONO H81  N8
DONO H82  N8
ACCE O4   C4
ACCE S2   C2
IC C2   C6   *N1   CM        1.4169  121.62  179.66  120.26  1.4722
IC C6   N1   C2    N3        1.3694  121.62    0.38  113.45  1.3828
IC N3   N1   *C2   S2        1.3828  113.45  179.80  126.68  1.6505
IC N1   C2   N3    C4        1.4169  113.45    0.66  127.81  1.3740
IC C4   C2   *N3   H3        1.3740  127.81  177.82  116.10  1.0083
IC C2   N3   C4    C5        1.3828  127.81   -1.87  116.34  1.4446
IC C5   N3   *C4   O4        1.4446  116.34 -177.45  118.64  1.2288
```

```
IC C5   N1   *C6   H6        1.3764  123.38 -177.84  114.65  1.0924
IC C6   C4   *C5   C7        1.3764  117.36  175.23  117.78  1.4823
IC C4   C5   C7    N8        1.4446  117.78   52.43  108.08  1.4961
IC N8   C5   *C7   H71       1.4961  108.08  117.97  112.13  1.1054
IC H71  C5   *C7   H72       1.1054  112.13  125.76  110.01  1.1063
IC C5   C7   N8    C9        1.4823  108.08 -170.97  117.62  1.5086
IC C9   C7   *N8   H81       1.5086  117.62  120.39  106.36  1.0213
IC C9   C7   *N8   H82       1.5086  117.62 -126.43  111.59  1.0091
IC C7   N8   C9    C10       1.4961  117.62  171.81  109.50  1.4991
IC C10  N8   *C9   H91       1.4991  109.50  123.35  105.73  1.1025
IC C10  N8   *C9   H92       1.4991  109.50 -119.95  106.18  1.1048
IC N8   C9   C10   C11       1.5086  109.50  107.68  127.66  1.3476
IC C11  C9   *C10  H10       1.3476  127.66  176.73  115.53  1.1019
IC C9   C10  C11   C12       1.4991  127.66  179.89  120.27  1.5051
IC C12  C10  *C11  C13       1.5051  120.27  179.67  125.70  1.5072
IC C10  C11  C12   H121      1.3476  120.27 -119.34  110.70  1.1127
IC H121 C11  *C12  H122      1.1127  110.70  120.52  113.93  1.1135
IC H121 C11  *C12  H123      1.1127  110.70 -118.56  110.91  1.1118
IC C10  C11  C13   H131      1.3476  125.70  121.38  110.26  1.1135
IC H131 C11  *C13  H132      1.1135  110.26  117.64  110.45  1.1126
IC H131 C11  *C13  H133      1.1135  110.26 -120.73  115.49  1.1091
IC C6   N1   CM    HM1       1.3694  120.26   -0.00  113.39  1.1135
IC HM1  N1   *CM   HM2       1.1135  113.39  120.58  111.37  1.1155
IC HM1  N1   *CM   HM3       1.1135  113.39 -120.11  111.12  1.1158

RESI B5CU          -1.00 ! 5-carboxymethyluracil, yxu
!RING 6 N1 C2 N3 C4 C5 C6
GROUP
ATOM N1     NG2R61 -0.34
ATOM C2     CG2R63  0.51
ATOM O2     OG2D4  -0.41 !  O81  O82(-)
ATOM N3     NG2R61 -0.46 !    \\ /
ATOM H3     HGP1    0.36 !     C8
ATOM C4     CG2R63  0.50 !     |    O4
ATOM O4     OG2D4  -0.45 !     |    ||
ATOM C5     CG2R62 -0.05 ! H72-C7   C4    H3
ATOM C6     CG2R62  0.17 !    /  \ /  \  /
ATOM H6     HGR62   0.17 !  H71   C5   N3
GROUP                    !        ||   |
ATOM C7     CG321  -0.18 !     H6-C6   C2
ATOM H71    HGA2    0.09 !         \  / \\
ATOM H72    HGA2    0.09 !          N1   O2
GROUP                    !           \
ATOM C8     CG2O3   0.52 !            \
ATOM O81    OG2D2  -0.76 !             \
ATOM O82    OG2D2  -0.76 !              \
GROUP
ATOM CM     CG331  -0.27
ATOM HM1    HGA3    0.09
ATOM HM2    HGA3    0.09
ATOM HM3    HGA3    0.09

BOND CM   HM1    CM   HM2   CM   HM3
BOND N1   C2      N1   C6      C2   O2      C2   N3
BOND N3   C4      N3   H3      C4   O4      C4   C5
BOND C5   C6      C5   C7      C6   H6      C7   H71
BOND C7   H72     C7   C8      C8   O81     C8   O82
BOND CM   N1
IMPR C2     N1     N3     O2     C4     C5     N3     O4     C8     O82    O81    C7
DONO H3   N3
ACCE O2   C2
ACCE O4   C4
ACCE O81
```

```
ACCE O82
IC C2   C6   *N1   CM        1.4048  119.99  180.00  121.27  1.4737
IC C6   N1   C2    N3        1.3769  119.99   -0.08  116.39  1.3657
IC N3   N1   *C2   O2        1.3657  116.39 -179.99  123.19  1.2312
IC N1   C2   N3    C4        1.4048  116.39   -0.09  126.26  1.3766
IC C4   C2   *N3   H3        1.3766  126.26 -179.60  117.13  0.9977
IC C2   N3   C4    C5        1.3657  126.26    0.46  116.55  1.4470
IC C5   N3   *C4   O4        1.4470  116.55 -179.98  117.25  1.2316
IC C5   N1   *C6   H6        1.3807  123.30  179.55  116.72  1.0926
IC C6   C4   *C5   C7        1.3807  117.52 -179.70  118.38  1.5141
IC C4   C5   C7    C8        1.4470  118.38 -175.71  115.31  1.5258
IC C8   C5   *C7   H71       1.5258  115.31  122.15  108.48  1.1103
IC H71  C5   *C7   H72       1.1103  108.48  117.72  107.93  1.1093
IC C5   C7   C8    O81       1.5141  115.31  125.41  115.76  1.2540
IC O81  C7   *C8   O82       1.2540  115.76 -179.69  116.01  1.2578
IC C6   N1   CM    HM1       1.3769  121.27    0.29  112.17  1.1148
IC HM1  N1   *CM   HM2       1.1148  112.17  120.14  111.08  1.1145
IC HM1  N1   *CM   HM3       1.1148  112.17 -120.20  111.12  1.1154

RESI BOCU           0.00 ! 5-methoxycarbonylmethyluracil, yxu
!RING 6 N1 C2 N3 C4 C5 C6
GROUP
ATOM N1     NG2R61 -0.34
ATOM C2     CG2R63  0.51
ATOM O2     OG2D4  -0.41
ATOM N3     NG2R61 -0.46 !  H101 H102
ATOM H3     HGP1    0.36 !    \  /
ATOM C4     CG2R63  0.50 !     C10-H103
ATOM O4     OG2D4  -0.45 !      |
ATOM C5     CG2R62 -0.05 !     O9
ATOM C6     CG2R62  0.17 !      |
ATOM H6     HGR62   0.17 !  O8=C8   O4
GROUP                    !      |    ||
ATOM C7     CG321  -0.22 ! H72-C7   C4    H3
ATOM H71    HGA2    0.09 !    /  \ /  \  /
ATOM H72    HGA2    0.09 !  H71   C5   N3
ATOM C8     CG2O2   0.90 !        ||    |
ATOM O9     OG302  -0.49 !     H6-C6   C2
ATOM O8     OG2D1  -0.63 !         \  / \\
ATOM C10    CG331  -0.01 !          N1   O2
ATOM H101   HGA3    0.09 !           \
ATOM H102   HGA3    0.09 !            \
ATOM H103   HGA3    0.09 !             \
GROUP
ATOM CM     CG331  -0.27
ATOM HM1    HGA3    0.09
ATOM HM2    HGA3    0.09
ATOM HM3    HGA3    0.09

BOND CM   HM1    CM   HM2   CM   HM3
BOND N1   C2      N1   C6      C2   O2      C2   N3
BOND N3   C4      N3   H3      C4   O4      C4   C5
BOND C5   C6      C5   C7      C6   H6      C7   H71
BOND C7   H72     C7   C8      C8   O9      C8   O8
BOND O9   C10     C10  H101    C10  H102    C10  H103
BOND CM   N1
IMPR C2     N1     N3     O2     C4     C5     N3     O4     C8     C7     O8     O9
DONO H3   N3
ACCE O2   C2
ACCE O4   C4
ACCE O8   C8
ACCE O9
IC C2   C6   *N1   CM        1.4084  120.13 -179.74  121.41  1.4739
```

```
IC C6   N1   C2    N3        1.3698  120.13    0.03  116.34  1.3676
IC N3   N1   *C2   O2        1.3676  116.34  179.85  123.14  1.2285
IC N1   C2   N3    C4        1.4084  116.34   -0.27  126.09  1.3753
IC C4   C2   *N3   H3        1.3753  126.09 -178.39  116.76  1.0002
IC C2   N3   C4    C5        1.3676  126.09    1.10  116.39  1.4494
IC C5   N3   *C4   O4        1.4494  116.39  179.80  117.99  1.2286
IC C5   N1   *C6   H6        1.3764  123.23  179.03  116.28  1.0913
IC C6   C4   *C5   C7        1.3764  117.80 -178.18  120.06  1.5059
IC C4   C5   C7    C8        1.4494  120.06   94.02  110.40  1.5190
IC C8   C5   *C7   H71       1.5190  110.40 -120.51  109.59  1.1111
IC H71  C5   *C7   H72       1.1111  109.59 -119.53  109.45  1.1094
IC C5   C7   C8    O9        1.5059  110.40 -163.39  109.53  1.3287
IC O9   C7   *C8   O8        1.3287  109.53 -177.53  124.79  1.2169
IC C7   C8   O9    C10       1.5190  109.53  179.97  112.53  1.4392
IC C8   O9   C10   H101      1.3287  112.53  -60.09  110.82  1.1137
IC H101 O9   *C10  H102      1.1137  110.82  120.96  110.92  1.1136
IC H101 O9   *C10  H103      1.1137  110.82 -119.52  109.84  1.1125
IC C6   N1   CM    HM1       1.3698  121.41    1.32  112.77  1.1135
IC HM1  N1   *CM   HM2       1.1135  112.77  120.33  111.08  1.1148
IC HM1  N1   *CM   HM3       1.1135  112.77 -120.56  111.08  1.1161

RESI B70U           0.00 ! 5-methoxycarbonylmethyl-2-thiouracil, SMU, yxu
GROUP
ATOM N1     NG2R61 -0.26
ATOM C2     CG2R63  0.29
ATOM S2     SG2D1  -0.22
ATOM N3     NG2R61 -0.56 !  H91 H92
ATOM H3     HGP1    0.40 !    \  /
ATOM C4     CG2R63  0.39 !     C9 -H93
ATOM O4     OG2D4  -0.41 !      |
ATOM C5     CG2R62 -0.03 !     O9
ATOM C6     CG2R62  0.18 !      |
ATOM H6     HGR62   0.22 !  O8=C8   O4
GROUP                    !      |    ||
ATOM C5M    CG321  -0.22 ! H52-C5M  C4    H3
ATOM H51    HGA2    0.09 !    /  \ /  \  /
ATOM H52    HGA2    0.09 !  H51   C5   N3
ATOM C8     CG2O2   0.90 !        ||    |
ATOM O9     OG302  -0.49 !     H6-C6   C2
ATOM O8     OG2D1  -0.63 !         \  / \\
ATOM C9     CG331  -0.01 !          N1   S2
ATOM H91    HGA3    0.09 !            \
ATOM H92    HGA3    0.09 !             \
ATOM H93    HGA3    0.09 !              \
GROUP
ATOM CM     CG331  -0.27
ATOM HM1    HGA3    0.09
ATOM HM2    HGA3    0.09
ATOM HM3    HGA3    0.09

BOND CM   HM1    CM   HM2   CM   HM3
BOND N1   C2      N1   C6      C2   S2      C2   N3
BOND N3   C4      N3   H3      C4   O4      C4   C5
BOND C5   C6      C5   C5M     C6   H6      C5M  C8
BOND C5M  H51     C5M  H52     C8   O8      C8   O9
BOND O9   C9      C9   H91     C9   H92     C9   H93
BOND CM   N1
IMPR C2     N1     N3     S2     C4     C5     N3     O4     C8     C5M    O8     O9
DONO H3   N3
ACCE O4   C4
ACCE S2   C2
ACCE O8   C8
ACCE O9
```

```
IC C2   C6   *N1   CM        1.4152  121.38  179.08  120.28  1.4727
IC C6   N1   C2    N3        1.3747  121.38   -0.45  113.54  1.3844
IC N3   N1   *C2   S2        1.3844  113.54  179.57  126.57  1.6535
IC N1   C2   N3    C4        1.4152  113.54    0.90  127.98  1.3789
IC C4   C2   *N3   H3        1.3789  127.98  178.15  117.24  1.0034
IC C2   N3   C4    C5        1.3844  127.98   -1.83  115.91  1.4424
IC C5   N3   *C4   O4        1.4424  115.91  179.42  118.05  1.2277
IC C5   N1   *C6   H6        1.3747  123.38 -176.53  116.46  1.0906
IC C6   C4   *C5   C5M       1.3747  117.76  174.91  120.15  1.5027
IC C4   C5   C5M   C8        1.4424  120.15 -103.49  107.77  1.5154
IC C8   C5   *C5M  H51       1.5154  107.77 -118.61  109.25  1.1092
IC C8   C5   *C5M  H52       1.5154  107.77  121.05  110.17  1.1100
IC C5   C5M  C8    O9        1.5027  107.77  119.25  110.54  1.3260
IC O9   C5M  *C8   O8        1.3260  110.54 -179.81  123.54  1.2176
IC C5M  C8   O9    C9        1.5154  110.54 -174.78  113.08  1.4378
IC C8   O9   C9    H91       1.3260  113.08  179.67  109.61  1.1127
IC H91  O9   *C9   H92       1.1127  109.61  119.86  111.23  1.1135
IC H91  O9   *C9   H93       1.1127  109.61 -118.82  110.53  1.1132
IC C6   N1   CM    HM1       1.3747  120.28    2.82  112.54  1.1122
IC HM1  N1   *CM   HM2       1.1122  112.54  120.13  111.45  1.1157
IC HM1  N1   *CM   HM3       1.1122  112.54 -120.06  111.18  1.1155

RESI BBCU            0.00 ! 5-carbamoylmethyluracil, yxu
!RING 6 N1 C2 N3 C4 C5 C6
GROUP
ATOM N1      NG2R61 -0.34
ATOM C2      CG2R63  0.51
ATOM O2      OG2D4  -0.41
ATOM N3      NG2R61 -0.46 ! H82    H81
ATOM H3      HGP1    0.36 !    \  /
ATOM C4      CG2R63  0.50 !     N8
ATOM O4      OG2D4  -0.45 !     |
ATOM C5      CG2R62 -0.05 !  O8=C8   O4
ATOM C6      CG2R62  0.17 !     |    ||
ATOM H6      HGR62   0.17 ! H72-C7   C4    H3
GROUP                     !    /  \ /  \  /
ATOM C7      CG321  -0.18 !  H71   C5   N3
ATOM H71     HGA2    0.09 !        ||    |
ATOM H72     HGA2    0.09 !     H6-C6   C2
ATOM C8      CG2O1   0.55 !         \  / \\
ATOM O8      OG2D1  -0.55 !          N1   O2
ATOM N8      NG2S2  -0.62 !           \
ATOM H81     HGP1    0.30 !            \
ATOM H82     HGP1    0.32 !             \
GROUP
ATOM CM      CG331  -0.27
ATOM HM1     HGA3    0.09
ATOM HM2     HGA3    0.09
ATOM HM3     HGA3    0.09

BOND CM   HM1    CM   HM2   CM   HM3
BOND N1   C2      N1   C6      C2   O2      C2   N3
BOND N3   C4      N3   H3      C4   O4      C4   C5
BOND C5   C6      C5   C7      C6   H6      C7   H71
BOND C7   H72     C7   C8      C8   N8      C8   O8
BOND N8   H81     N8   H82
BOND CM   N1
IMPR C2     N1     N3     O2     C4     C5     N3     O4     C8     C7     N8     O8
DONO H3   N3
DONO H81  N8
DONO H82  N8
ACCE O2   C2
ACCE O4   C4
```

```
ACCE O8   C8
IC C2   C6   *N1   CM        1.4090  120.13  179.88  121.33  1.4732
IC C6   N1   C2    N3        1.3707  120.13   -0.52  116.36  1.3679
IC N3   N1   *C2   O2        1.3679  116.36 -179.84  123.10  1.2279
IC N1   C2   N3    C4        1.4090  116.36   -0.38  126.04  1.3747
IC C4   C2   *N3   H3        1.3747  126.04 -178.16  116.56  1.0000
IC C2   N3   C4    C5        1.3679  126.04    1.93  116.41  1.4474
IC C5   N3   *C4   O4        1.4474  116.41 -179.28  118.15  1.2298
IC C5   N1   *C6   H6        1.3770  123.07  178.46  116.69  1.0916
IC C6   C4   *C5   C7        1.3770  117.94 -177.76  119.77  1.5041
IC C4   C5   C7    C8        1.4474  119.77   92.95  109.67  1.4913
IC C8   C5   *C7   H71       1.4913  109.67 -123.29  109.77  1.1120
IC H71  C5   *C7   H72       1.1120  109.77 -118.02  108.63  1.1132
IC C5   C7   C8    N8        1.5041  109.67  -96.21  118.06  1.3527
IC N8   C7   *C8   O8        1.3527  118.06  177.22  119.76  1.2268
IC C7   C8   N8    H81       1.4913  118.06  176.06  116.84  0.9966
IC H81  C8   *N8   H82       0.9966  116.84 -168.59  119.42  0.9966
IC C6   N1   CM    HM1       1.3707  121.33    0.34  112.79  1.1136
IC HM1  N1   *CM   HM2       1.1136  112.79  120.38  111.04  1.1156
IC HM1  N1   *CM   HM3       1.1136  112.79 -120.53  111.14  1.1155

RESI BHCU          -1.00 ! 5-(carboxyhydroxymethyl)uracil, yxu
!RING 6 N1 C2 N3 C4 C5 C6
GROUP
ATOM N1      NG2R61 -0.34
ATOM C2      CG2R63  0.51
ATOM O2      OG2D4  -0.41
ATOM N3      NG2R61 -0.46
ATOM H3      HGP1    0.36
ATOM C4      CG2R63  0.50 !   O81  O82(-)
ATOM O4      OG2D4  -0.45 !     \\ /
ATOM C5      CG2R62 -0.05 !      C8      O4
ATOM C6      CG2R62  0.17 !        \    ||
ATOM H6      HGR62   0.17 ! H7O-O7-C7   C4    H3
GROUP                     !       /  \ /  \  /
ATOM C7      CG311   0.14 !     H7    C5   N3
ATOM H7      HGA1    0.09 !           ||   |
ATOM O7      OG311  -0.65 !        H6-C6   C2
ATOM H7O     HGP1    0.42 !            \  / \\
GROUP                     !             N1   O2
ATOM C8      CG2O3   0.52 !              \
ATOM O81     OG2D2  -0.76 !               \
ATOM O82     OG2D2  -0.76 !                \
GROUP
ATOM CM      CG331  -0.27
ATOM HM1     HGA3    0.09
ATOM HM2     HGA3    0.09
ATOM HM3     HGA3    0.09

BOND CM   HM1    CM   HM2   CM   HM3
BOND N1   C2       N1   C6       C2   O2       C2   N3
BOND N3   C4       N3   H3       C4   O4       C4   C5
BOND C5   C6       C5   C7       C6   H6       C7   H7
BOND C7   O7       C7   C8       O7   H7O      C8   O81
BOND C8   O82
BOND CM   N1
IMPR C2     N1     N3     O2     C4     C5     N3     O4     C8     O82    O81    C7
DONO H3   N3
DONO H7O  O7
ACCE O2   C2
ACCE O4   C4
ACCE O7
ACCE O81  C8
```

```
ACCE O82  C8
IC C2   C6   *N1   CM        1.4028  119.87 -179.65  121.40  1.4745
IC C6   N1   C2    N3        1.3738  119.87    0.15  116.18  1.3638
IC N3   N1   *C2   O2        1.3638  116.18  179.66  123.21  1.2309
IC N1   C2   N3    C4        1.4028  116.18    0.73  126.81  1.3767
IC C4   C2   *N3   H3        1.3767  126.81  179.85  117.27  0.9975
IC C2   N3   C4    C5        1.3638  126.81   -1.07  116.24  1.4559
IC C5   N3   *C4   O4        1.4559  116.24  178.94  115.89  1.2298
IC C5   N1   *C6   H6        1.3806  124.01  177.62  116.39  1.0937
IC C6   C4   *C5   C7        1.3806  116.88  177.18  124.06  1.5199
IC C4   C5   C7    C8        1.4559  124.06  131.70  113.85  1.5709
IC C8   C5   *C7   O7        1.5709  113.85 -129.87  116.79  1.4344
IC O7   C5   *C7   H7        1.4344  116.79 -116.50  106.71  1.1100
IC C5   C7   O7    H7O       1.5199  116.79  150.87   99.36  0.9741
IC C5   C7   C8    O81       1.5199  113.85   13.00  118.69  1.2579
IC O81  C7   *C8   O82       1.2579  118.69  178.46  114.42  1.2631
IC C6   N1   CM    HM1       1.3738  121.40  120.53  110.88  1.1143
IC HM1  N1   *CM   HM2       1.1143  110.88  119.59  111.15  1.1151
IC HM1  N1   *CM   HM3       1.1143  110.88 -119.89  112.23  1.1138

RESI BCMU           0.00 ! 5-(carboxyhydroxymethyl)uracil methyl ester, yxu
GROUP
ATOM N1     NG2R61 -0.34
ATOM C2     CG2R63  0.51
ATOM O2     OG2D4  -0.41
ATOM N3     NG2R61 -0.46
ATOM H3     HGP1    0.36
ATOM C4     CG2R63  0.50 !          H102
ATOM O4     OG2D4  -0.45 !          |
ATOM C5     CG2R62 -0.05 !    H103-C10-H101
ATOM C6     CG2R62  0.17 !          |
ATOM H6     HGR62   0.17 !    O8   O9
GROUP                    !     \\ /
ATOM C7     CG311   0.14 !      C8      O4
ATOM H7     HGA1    0.09 !         \    ||
ATOM O7     OG311  -0.65 ! H7O-O7-C7   C4    H3
ATOM H7O    HGP1    0.42 !        /  \ /  \  /
GROUP                    !     H7    C5   N3
ATOM C8     CG2O2   0.86 !           ||   |
ATOM O8     OG2D1  -0.63 !        H6-C6   C2
ATOM O9     OG302  -0.49 !             \  / \\
ATOM C10    CG331  -0.01 !              N1   O2
ATOM H101   HGA3    0.09 !               \
ATOM H102   HGA3    0.09 !                \
ATOM H103   HGA3    0.09 !                 \
GROUP
ATOM CM     CG331  -0.27
ATOM HM1    HGA3    0.09
ATOM HM2    HGA3    0.09
ATOM HM3    HGA3    0.09

BOND CM   HM1    CM   HM2   CM   HM3
BOND N1   C2      N1   C6      C2   O2      C2   N3
BOND N3   C4      N3   H3      C4   O4      C4   C5
BOND C5   C6      C5   C7      C6   H6      C7   H7
BOND C7   C8      C7   O7      C8   O8      C8   O9
BOND O7   H7O     O9   C10     C10  H101    C10  H102
BOND C10  H103
BOND CM   N1
IMPR C2     N1     N3     O2     C4     C5     N3     O4     C8     C7     O8     O9
DONO H3   N3
DONO H7O  O7
ACCE O2   C2
```

```
ACCE O4   C4
ACCE O7
ACCE O8   C8
ACCE O9
IC C2   C6   *N1   CM        1.4088  120.07 -179.99  121.41  1.4735
IC C6   N1   C2    N3        1.3701  120.07    0.20  116.22  1.3670
IC N3   N1   *C2   O2        1.3670  116.22  179.99  123.15  1.2287
IC N1   C2   N3    C4        1.4088  116.22    0.21  126.05  1.3745
IC C4   C2   *N3   H3        1.3745  126.05  178.86  116.69  1.0006
IC C2   N3   C4    C5        1.3670  126.05   -1.26  116.64  1.4407
IC C5   N3   *C4   O4        1.4407  116.64  179.91  118.01  1.2289
IC C5   N1   *C6   H6        1.3772  123.20 -178.94  116.62  1.0926
IC C6   C4   *C5   C7        1.3772  117.79  178.84  119.35  1.4989
IC C4   C5   C7    C8        1.4407  119.35   95.20  111.19  1.5731
IC C8   C5   *C7   O7        1.5731  111.19  123.64  111.40  1.4442
IC C8   C5   *C7   H7        1.5731  111.19 -117.15  110.71  1.1122
IC C5   C7   O7    H7O       1.4989  111.40 -161.53  107.97  0.9689
IC C5   C7   C8    O9        1.4989  111.19  -42.70  111.32  1.3378
IC O9   C7   *C8   O8        1.3378  111.32 -179.50  124.11  1.2190
IC C7   C8   O9    C10       1.5731  111.32 -179.44  112.76  1.4377
IC C8   O9   C10   H101      1.3378  112.76 -179.84  109.81  1.1119
IC H101 O9   *C10  H102      1.1119  109.81  119.63  110.98  1.1132
IC H101 O9   *C10  H103      1.1119  109.81 -119.40  110.84  1.1139
IC C6   N1   CM    HM1       1.3701  121.41    0.15  112.76  1.1144
IC HM1  N1   *CM   HM2       1.1144  112.76  120.50  111.09  1.1153
IC HM1  N1   *CM   HM3       1.1144  112.76 -120.42  111.07  1.1155

RESI BOAU          -1.00 ! uracil 5-oxyacetic acid, yxu
GROUP
ATOM N1     NG2R61 -0.30
ATOM C2     CG2R63  0.57
ATOM O2     OG2D4  -0.49 !   O91   O92(-)
ATOM N3     NG2R61 -0.47 !      \\ /
ATOM H3     HGP1    0.33 !  H82 C9
ATOM C4     CG2R63  0.49 !    \ |
ATOM O4     OG2D4  -0.49 ! H81--C8  O4
ATOM C5     CG2R62  0.17 !      |   ||
ATOM C6     CG2R62  0.21 !      O7  C4    H3
ATOM H6     HGR62   0.16 !       \ /  \  /
ATOM O7     OG301  -0.43 !        C5   N3
ATOM C8     CG321   0.07 !        ||   |
ATOM H81    HGA2    0.09 !     H6-C6   C2
ATOM H82    HGA2    0.09 !          \  / \\
GROUP                    !           N1   O2
ATOM C9     CG2O3   0.52 !            \
ATOM O91    OG2D2  -0.76 !             \
ATOM O92    OG2D2  -0.76 !              \
GROUP
ATOM CM     CG331  -0.27
ATOM HM1    HGA3    0.09
ATOM HM2    HGA3    0.09
ATOM HM3    HGA3    0.09

BOND CM   HM1    CM   HM2   CM   HM3
BOND N1   C2      N1   C6      C2   O2      C2   N3
BOND N3   C4      N3   H3      C4   O4      C4   C5
BOND C5   C6      C5   O7      C6   H6      O7   C8
BOND C8   H81     C8   H82     C8   C9      C9   O91
BOND C9   O92
BOND CM   N1
IMPR C2     N1     N3     O2     C4     C5     N3     O4     C9     O92    O91    C8
DONO H3   N3
ACCE O2   C2
```

```
ACCE O4   C4
ACCE O7
ACCE O91  C9
ACCE O92  C9
IC C2   C6   *N1   CM        1.4080  119.71  179.73  121.44  1.4748
IC C6   N1   C2    N3        1.3774  119.71    0.22  116.53  1.3655
IC N3   N1   *C2   O2        1.3655  116.53 -179.62  123.16  1.2288
IC N1   C2   N3    C4        1.4080  116.53   -0.36  126.21  1.3803
IC C4   C2   *N3   H3        1.3803  126.21  178.45  116.79  0.9994
IC C2   N3   C4    C5        1.3655  126.21   -0.69  116.37  1.4573
IC C5   N3   *C4   O4        1.4573  116.37 -179.03  116.93  1.2319
IC C5   N1   *C6   H6        1.3738  123.85 -177.87  117.49  1.0912
IC C6   C4   *C5   O7        1.3738  117.30 -179.18  123.82  1.3655
IC C4   C5   O7    C8        1.4573  123.82  -82.59  112.30  1.4472
IC C5   O7   C8    C9        1.3655  112.30  -70.17  117.78  1.5472
IC C9   O7   *C8   H81       1.5472  117.78 -119.32  107.78  1.1101
IC H81  O7   *C8   H82       1.1101  107.78 -116.97  110.66  1.1108
IC O7   C8   C9    O91       1.4472  117.78   -0.99  118.35  1.2583
IC O91  C8   *C9   O92       1.2583  118.35  179.68  114.24  1.2586
IC C6   N1   CM    HM1       1.3774  121.44   -1.34  112.19  1.1144
IC HM1  N1   *CM   HM2       1.1144  112.19  120.74  111.27  1.1138
IC HM1  N1   *CM   HM3       1.1144  112.19 -119.70  110.83  1.1155

RESI BOEU           0.00 ! uracil 5-oxyacetic acid methyl ester, yxu
GROUP
ATOM N1     NG2R61 -0.30
ATOM C2     CG2R63  0.57
ATOM O2     OG2D4  -0.49 !     H111 H112
ATOM N3     NG2R61 -0.47 !       \  /
ATOM H3     HGP1    0.33 !        C11-H113
ATOM C4     CG2R63  0.49 !        |
ATOM O4     OG2D4  -0.49 !   O9   O10
ATOM C5     CG2R62  0.17 !    \\ /
ATOM C6     CG2R62  0.21 ! H82 C9
ATOM H6     HGR62   0.16 !    \ |
ATOM O7     OG301  -0.43 ! H81-C8   O4
ATOM C8     CG321   0.07 !      |   ||
ATOM H81    HGA2    0.09 !     O7   C4    H3
ATOM H82    HGA2    0.09 !       \ /  \  /
GROUP                    !        C5   N3
ATOM C9     CG2O2   0.86 !        ||   |
ATOM O9     OG2D1  -0.63 !     H6-C6   C2
ATOM O10    OG302  -0.49 !         \  / \\
ATOM C11    CG331  -0.01 !          N1   O2
ATOM H111   HGA3    0.09 !           \
ATOM H112   HGA3    0.09 !            \
ATOM H113   HGA3    0.09 !             \
GROUP
ATOM CM     CG331  -0.27
ATOM HM1    HGA3    0.09
ATOM HM2    HGA3    0.09
ATOM HM3    HGA3    0.09

BOND CM   HM1    CM   HM2   CM   HM3
BOND N1   C2      N1   C6      C2   O2      C2   N3
BOND N3   C4      N3   H3      C4   O4      C4   C5
BOND C5   C6      C5   O7      C6   H6      O7   C8
BOND C8   H81     C8   H82     C8   C9      C9   O9
BOND C9   O10     O10  C11     C11  H111    C11  H112
BOND C11  H113
BOND CM   N1
IMPR C2     N1     N3     O2     C4     C5     N3     O4     C9     C8     O9     O10
DONO H3   N3
```

```
ACCE O2   C2
ACCE O4   C4
ACCE O7
ACCE O9   C9
ACCE O9
IC C2   C6   *N1   CM        1.4076  119.95  179.98  121.38  1.4749
IC C6   N1   C2    N3        1.3725  119.95   -0.26  116.36  1.3645
IC N3   N1   *C2   O2        1.3645  116.36 -179.89  123.14  1.2269
IC N1   C2   N3    C4        1.4076  116.36   -0.52  126.45  1.3789
IC C4   C2   *N3   H3        1.3789  126.45 -178.30  116.19  1.0018
IC C2   N3   C4    C5        1.3645  126.45    1.63  116.24  1.4701
IC C5   N3   *C4   O4        1.4701  116.24  179.90  116.58  1.2314
IC C5   N1   *C6   H6        1.3731  124.21  179.19  116.72  1.0901
IC C6   C4   *C5   O7        1.3731  116.78 -175.95  126.38  1.3756
IC C4   C5   O7    C8        1.4701  126.38  -15.46  117.58  1.4423
IC C5   O7   C8    C9        1.3756  117.58 -168.70  113.55  1.5440
IC C9   O7   *C8   H81       1.5440  113.55 -119.70  109.94  1.1107
IC H81  O7   *C8   H82       1.1107  109.94 -120.78  110.67  1.1130
IC O7   C8   C9    O10       1.4423  113.55 -178.60  108.86  1.3403
IC O10  C8   *C9   O9        1.3403  108.86 -179.66  126.64  1.2185
IC C8   C9   O10   C11       1.5440  108.86  179.92  112.31  1.4387
IC C9   O10  C11   H111      1.3403  112.31  179.74  109.80  1.1124
IC H111 O10  *C11  H112      1.1124  109.80  119.62  110.90  1.1134
IC H111 O10  *C11  H113      1.1124  109.80 -119.48  110.90  1.1144
IC C6   N1   CM    HM1       1.3725  121.38   -0.27  112.87  1.1136
IC HM1  N1   *CM   HM2       1.1136  112.87  120.56  111.01  1.1153
IC HM1  N1   *CM   HM3       1.1136  112.87 -120.55  111.03  1.1159

RESI B3AU           0.00 ! 3-(3-amino-3-carboxypropyl)uracil, yxu
GROUP
ATOM N1     NG2R61 -0.21
ATOM C2     CG2R63  0.52 !      O4    H101  H111 H12    O30 (-)
ATOM O2     OG2D4  -0.44 !      ||     |     |    |     /
ATOM N3     NG2R61 -0.32 !      C4    C10---C11--C12--C13
ATOM C4     CG2R63  0.50 !     /  \  / \     |    |     \\
ATOM O4     OG2D4  -0.48 ! H5-C5   N3  H102 H112 |(+)   O31
ATOM C5     CG2R62 -0.22 !    ||   |              N14
ATOM H5     HGR62   0.09 ! H6-C6   C2           / |  \
ATOM C6     CG2R62  0.31 !     \  / \\     H141 H142 H143
ATOM H6     HGR62   0.11 !      N1   O2
ATOM C10    CG321  -0.04 !        \
ATOM H101   HGA2    0.09 !         \
ATOM H102   HGA2    0.09 !          \
GROUP
ATOM C11    CG321  -0.18
ATOM H111   HGA2    0.09
ATOM H112   HGA2    0.09
GROUP
ATOM C12    CG314   0.17
ATOM H12    HGA1    0.11
ATOM N14    NG3P3  -0.34
ATOM H141   HGP2    0.30
ATOM H142   HGP2    0.30 !  !!!! PATCH 5UHA for the non-ionic tautomer
ATOM H143   HGP2    0.30
ATOM C13    CG2O3   0.32
ATOM O30    OG2D2  -0.58
ATOM O31    OG2D2  -0.58
GROUP
ATOM CM     CG331  -0.27
ATOM HM1    HGA3    0.09
ATOM HM2    HGA3    0.09
ATOM HM3    HGA3    0.09
```

```
BOND CM   HM1    CM   HM2   CM   HM3
BOND N1   C2      N1   C6      C2   O2      C2   N3
BOND N3   C4      N3   C10     C4   O4      C4   C5
BOND C5   C6      C5   H5      C6   H6      C10  C11
BOND C10  H101    C10  H102    C11  C12     C11  H111
BOND C11  H112    C12  H12     C12  N14     C12  C13
BOND N14  H141    N14  H142    N14  H143    C13  O30
BOND C13  O31
BOND CM   N1
IMPR C2     N1     N3     O2     C4     C5     N3     O4     C13    O30    O31    C12
DONO H141 N14
DONO H142 N14
DONO H143 N14
ACCE O2   C2
ACCE O4   C4
ACCE O30  C13
ACCE O31  C13
IC C6   C2   *N1   CM        1.3605  119.97  179.94  118.54  1.4756
IC C6   N1   C2    N3        1.3605  119.97    0.26  117.12  1.4024
IC N3   N1   *C2   O2        1.4024  117.12  179.74  121.28  1.2322
IC N1   C2   N3    C10       1.4086  117.12 -179.26  118.46  1.4757
IC C10  C2   *N3   C4        1.4757  118.46  177.37  123.31  1.4020
IC C2   N3   C4    O4        1.4024  123.31 -176.34  119.70  1.2336
IC O4   N3   *C4   C5        1.2336  119.70  179.18  116.79  1.4252
IC C6   C4   *C5   H5        1.3662  119.38 -178.05  120.32  1.0872
IC C5   N1   *C6   H6        1.3662  123.38  179.47  116.14  1.0916
IC C2   N3   C10   C11       1.4024  118.46   93.92  113.30  1.5434
IC C11  N3   *C10  H101      1.5434  113.30  123.73  107.95  1.1135
IC C11  N3   *C10  H102      1.5434  113.30 -122.29  109.05  1.1157
IC N3   C10  C11   C12       1.4757  113.30  125.97  114.58  1.5545
IC C12  C10  *C11  H111      1.5545  114.58  120.22  107.24  1.1159
IC C12  C10  *C11  H112      1.5545  114.58 -124.41  111.51  1.1118
IC C10  C11  C12   C13       1.5434  114.58  173.41  110.16  1.5432
IC C13  C11  *C12  N14       1.5432  110.16  122.04  109.55  1.4942
IC N14  C11  *C12  H12       1.4942  109.55  119.74  109.57  1.1113
IC C11  C12  N14   H141      1.5545  109.55 -100.49  102.82  1.0487
IC H141 C12  *N14  H142      1.0487  102.82  118.64  111.48  1.0464
IC H141 C12  *N14  H143      1.0487  102.82 -115.86  110.95  1.0337
IC C11  C12  C13   O30       1.5545  110.16  116.92  116.88  1.2611
IC O30  C12  *C13  O31       1.2611  116.88  179.78  115.06  1.2542
IC C6   N1   CM    HM1       1.3605  121.48  119.99  110.93  1.1154
IC HM1  N1   *CM   HM2       1.1154  110.93  118.81  111.17  1.1156
IC HM1  N1   *CM   HM3       1.1154  110.93 -120.48  112.91  1.1137

RESI B13P           0.00 ! 1-methyl-3-(3-amino-3-carboxypropyl)pseudouracil, yxu
GROUP
ATOM C5     CG2R62 -0.20
ATOM C4     CG2R63  0.74
ATOM O4     OG2D4  -0.52
ATOM N3     NG2R61 -0.44
ATOM C2     CG2R63  0.86 !   H1M1    O2     H101 H111 H12   O30 (-)
ATOM O2     OG2D4  -0.54 !       \    ||      /    |    |    /
ATOM N1     NG2R61 -0.33 ! H1M2-C1M  C2    C10---C11--C12-C13
ATOM C6     CG2R62  0.03 !      / \ /  \  / \    |    |    \\
ATOM H6     HGR62   0.18 !   H1M3  N1   N3  H102 H112 |(+)  O31
ATOM C1M    CG331  -0.15 !         |    |              N14
ATOM H1M1   HGA3    0.09 !      H6-C6   C4           / |  \
ATOM H1M2   HGA3    0.09 !          \  / \\     H141 H142 H143
ATOM H1M3   HGA3    0.09 !           C5   O4
ATOM C10    CG321  -0.08 !            \
ATOM H101   HGA2    0.09 !             \
ATOM H102   HGA2    0.09 !              \
GROUP
```

```
ATOM C11    CG321  -0.18
ATOM H111   HGA2    0.09
ATOM H112   HGA2    0.09
GROUP
ATOM C12    CG314   0.17
ATOM H12    HGA1    0.11 !  !!!! PATCH 5UHA for the non-ionic tautomer
ATOM N14    NG3P3  -0.34
ATOM H141   HGP2    0.30
ATOM H142   HGP2    0.30
ATOM H143   HGP2    0.30
ATOM C13    CG2O3   0.32
ATOM O30    OG2D2  -0.58
ATOM O31    OG2D2  -0.58
GROUP
ATOM CM     CG331  -0.27
ATOM HM1    HGA3    0.09
ATOM HM2    HGA3    0.09
ATOM HM3    HGA3    0.09

BOND CM   HM1    CM   HM2   CM   HM3
BOND C5   C4   C5   C6   C4   O4   C4   N3
BOND N3   C2   N3   C10  C2   O2   C2   N1
BOND N1   C6   N1   C1M  C6   H6   C1M  H1M1
BOND C1M  H1M2 C1M  H1M3 C10  H101 C10  H102
BOND C10  C11  C11  H111 C11  H112 C11  C12
BOND C12  H12  C12  N14  C12  C13  N14  H141
BOND N14 H142 N14 H143 C13  O30  C13  O31
BOND CM   C5
IMPR C4     C5     N3     O4     C2     N3     N1     O2     C13    O31    O30    C12
DONO H141 N14
DONO H142 N14
DONO H143 N14
ACCE O2   C2
ACCE O4   C4
ACCE O30  C13
ACCE O31  C13
IC C6   C4   *C5   CM        1.3801  116.91  180.00  121.05  1.5015
IC C6   C5   C4    N3        1.3801  116.91    2.84  116.70  1.4130
IC N3   C5   *C4   O4        1.4130  116.70  176.41  124.45  1.2357
IC C5   C4   N3    C10       1.4708  116.70 -168.84  119.96  1.4772
IC C10  C4   *N3   C2        1.4772  119.96  155.94  116.40  1.3964
IC C4   N3   C2    N1        1.4130  116.40   44.31  112.72  1.4195
IC N1   N3   *C2   O2        1.4195  112.72 -154.24  120.03  1.2285
IC N1   C5   *C6   H6        1.3702  118.08 -179.36  117.21  1.0945
IC C6   C2   *N1   C1M       1.3702  106.90  180.00  111.97  1.4862
IC C2   N1   C1M   H1M1      1.4195  111.97   55.86  111.04  1.1168
IC H1M1 N1   *C1M  H1M2      1.1168  111.04  116.75  110.27  1.1162
IC H1M1 N1   *C1M  H1M3      1.1168  111.04 -122.20  116.09  1.1115
IC C4   N3   C10   C11       1.4130  119.96 -100.98  112.22  1.5393
IC C11  N3   *C10  H101      1.5393  112.22 -120.10  108.83  1.1177
IC H101 N3   *C10  H102      1.1177  108.83 -115.54  109.43  1.1138
IC N3   C10  C11   C12       1.4772  112.22   57.74  115.95  1.5548
IC C12  C10  *C11  H111      1.5548  115.95 -121.44  108.17  1.1155
IC H111 C10  *C11  H112      1.1155  108.17 -115.05  108.93  1.1139
IC C10  C11  C12   C13       1.5393  115.95 -177.99  110.81  1.5390
IC C13  C11  *C12  N14       1.5390  110.81 -124.88  109.60  1.4948
IC N14  C11  *C12  H12       1.4948  109.60 -118.47  109.64  1.1104
IC C11  C12  N14   H141      1.5548  109.60   48.25  108.13  1.0362
IC H141 C12  *N14  H142      1.0362  108.13  116.01  106.16  1.0423
IC H141 C12  *N14  H143      1.0362  108.13 -122.44  109.88  1.0462
IC C11  C12  C13   O30       1.5548  110.81 -122.16  116.83  1.2589
IC O30  C12  *C13  O31       1.2589  116.83 -179.19  114.90  1.2550
IC C6   C5   CM    HM1       1.3801  121.33  -35.38  111.51  1.1129
```

```
IC HM1  C5   *CM   HM2       1.1129  111.51  120.14  110.81  1.1134
IC HM1  C5   *CM   HM3       1.1129  111.51 -119.98  111.42  1.1137

RESI B5TU           0.00 ! 5-taurinomethyluracil (aka 5-{[(2-
sulfoethyl)amino]methyl}uridine), yxu
GROUP
ATOM N1     NG2R61 -0.34
ATOM C2     CG2R63  0.51
ATOM O2     OG2D4  -0.41 !      O12 (-)
ATOM N3     NG2R61 -0.46 !       |
ATOM H3     HGP1    0.36 ! O13 =S11=O11
ATOM C4     CG2R63  0.50 !       |
ATOM O4     OG2D4  -0.45 ! H101-C10-H102
ATOM C5     CG2R62 -0.05 !       |
ATOM C6     CG2R62  0.17 !       |   H81 H72  O4
ATOM H6     HGR62   0.17 !       |   |(+) |   ||
GROUP                    !  H91-C9 -N8 -C7   C4    H3
ATOM C7     CG324   0.14 !       |   |   / \ /  \  /
ATOM H71    HGA2    0.09 !     H92 H82 H71 C5   N3
ATOM H72    HGA2    0.09 !                 ||   |
ATOM N8     NG3P2  -0.30 !              H6-C6   C2
ATOM H81    HGP2    0.33 !                  \  / \\
ATOM H82    HGP2    0.33 !                   N1   O2
ATOM C9     CG324   0.14 !                    \
ATOM H91    HGA2    0.09 !                     \
ATOM H92    HGA2    0.09 !                      \
GROUP
ATOM C10    CG321  -0.26
ATOM H101   HGA2    0.09
ATOM H102   HGA2    0.09
ATOM S11    SG3O1   0.73
ATOM O11    OG2P1  -0.55
ATOM O12    OG2P1  -0.55
ATOM O13    OG2P1  -0.55
GROUP
ATOM CM     CG331  -0.27
ATOM HM1    HGA3    0.09
ATOM HM2    HGA3    0.09
ATOM HM3    HGA3    0.09

BOND CM   HM1    CM   HM2   CM   HM3
BOND N1   C2      N1   C6       C2   O2       C2   N3
BOND N3   C4      N3   H3       C4   O4       C4   C5
BOND C5   C6      C5   C7       C6   H6       C7   H71
BOND C7   H72     C7   N8       C9   C10      C9   H91
BOND C9   H92     C9   N8       C10  H101     C10  H102
BOND C10  S11     N8   H81      N8   H82      S11  O11
BOND S11  O12     S11  O13
BOND CM   N1
IMPR C2     N1     N3     O2     C4     C5     N3     O4
DONO H3   N3
DONO H81  N8
DONO H82  N8
ACCE O2   C2
ACCE O4   C4
ACCE O11  S11
ACCE O12  S11
ACCE O13  S11
IC C2   C6   *N1   CM        1.4092  120.25  179.27  121.24  1.4737
IC C6   N1   C2    N3        1.3693  120.25   -0.80  116.23  1.3679
IC N3   N1   *C2   O2        1.3679  116.23  179.82  123.16  1.2276
IC N1   C2   N3    C4        1.4092  116.23    0.64  125.99  1.3728
IC C4   C2   *N3   H3        1.3728  125.99  178.03  116.03  1.0032
```

```
IC C2   N3   C4    C5        1.3679  125.99   -0.23  116.70  1.4468
IC C5   N3   *C4   O4        1.4468  116.70 -176.68  118.55  1.2292
IC C5   N1   *C6   H6        1.3775  123.13 -176.95  115.81  1.0951
IC C6   C4   *C5   C7        1.3775  117.70  173.51  119.17  1.4753
IC C4   C5   C7    N8        1.4468  119.17   52.84  110.32  1.4923
IC N8   C5   *C7   H71       1.4923  110.32  117.05  109.87  1.1027
IC H71  C5   *C7   H72       1.1027  109.87  124.82  111.14  1.1061
IC C5   C7   N8    C9        1.4753  110.32 -172.08  117.99  1.5222
IC C9   C7   *N8   H81       1.5222  117.99  121.48  107.15  1.0143
IC C9   C7   *N8   H82       1.5222  117.99 -123.38  109.85  1.0070
IC C7   N8   C9    C10       1.4923  117.99  -68.84  115.06  1.5343
IC C10  N8   *C9   H91       1.5343  115.06  127.92  104.91  1.1036
IC C10  N8   *C9   H92       1.5343  115.06 -119.72  103.38  1.1061
IC N8   C9   C10   S11       1.5222  115.06   99.78  111.15  1.7917
IC S11  C9   *C10  H101      1.7917  111.15  114.84  109.22  1.1096
IC H101 C9   *C10  H102      1.1096  109.22  121.72  114.34  1.1058
IC C9   C10  S11   O11       1.5343  111.15  171.51  102.77  1.4448
IC O11  C10  *S11  O12       1.4448  102.77  120.38  104.37  1.4456
IC O11  C10  *S11  O13       1.4448  102.77 -120.04  101.76  1.4456
IC C6   N1   CM    HM1       1.3693  121.24   -8.71  112.94  1.1139
IC HM1  N1   *CM   HM2       1.1139  112.94  120.83  111.25  1.1153
IC HM1  N1   *CM   HM3       1.1139  112.94 -120.27  111.18  1.1169

RESI BSTU           0.00 ! 5-taurinomethyl-2-thiouracil, yxu
GROUP
ATOM N1     NG2R61 -0.26
ATOM C2     CG2R63  0.29
ATOM S2     SG2D1  -0.22 !      O12 (-)
ATOM N3     NG2R61 -0.56 !       |
ATOM H3     HGP1    0.40 ! O13 =S11=O11
ATOM C4     CG2R63  0.39 !       |
ATOM O4     OG2D4  -0.41 ! H101-C10-H102
ATOM C5     CG2R62 -0.03 !       |
ATOM C6     CG2R62  0.18 !       |   H81 H72  O4
ATOM H6     HGR62   0.22 !       |   |(+) |   ||
GROUP                    !  H91-C9 -N8 -C7   C4    H3
ATOM C7     CG324   0.14 !       |   |   / \ /  \  /
ATOM H71    HGA2    0.09 !     H92 H82 H71 C5   N3
ATOM H72    HGA2    0.09 !                 ||   |
ATOM N8     NG3P2  -0.30 !             H6-C6   C2
ATOM H81    HGP2    0.33 !                  \  / \\
ATOM H82    HGP2    0.33 !                   N1   S2
ATOM C9     CG324   0.14 !                    \
ATOM H91    HGA2    0.09 !                     \
ATOM H92    HGA2    0.09 !                      \
GROUP
ATOM C10    CG321  -0.26
ATOM H101   HGA2    0.09
ATOM H102   HGA2    0.09
ATOM S11    SG3O1   0.73
ATOM O11    OG2P1  -0.55
ATOM O12    OG2P1  -0.55
ATOM O13    OG2P1  -0.55
GROUP
ATOM CM     CG331  -0.27
ATOM HM1    HGA3    0.09
ATOM HM2    HGA3    0.09
ATOM HM3    HGA3    0.09
BOND CM   HM1    CM   HM2   CM   HM3
BOND N1   C2      N1   C6      C2   S2      C2   N3
BOND N3   C4      N3   H3      C4   O4      C4   C5
BOND C5   C6      C5   C7      C6   H6      C7   H71
BOND C7   H72     C7   N8      C9   C10     C9   H91
```

```
BOND C9   H92     C9   N8      C10  H101    C10  H102
BOND C10  S11     N8   H81     N8   H82     S11  O11
BOND S11  O12     S11  O13
BOND CM   N1
IMPR C2     N1     N3     S2     C4     C5     N3     O4
DONO H3   N3
DONO H81  N8
DONO H82  N8
ACCE O4   C4
ACCE S2   C2
ACCE O11  S11
ACCE O12  S11
ACCE O13  S11
IC C2   C6   *N1   CM        1.4147  121.69  179.95  120.03  1.4725
IC C6   N1   C2    N3        1.3732  121.69   -0.50  113.48  1.3852
IC N3   N1   *C2   S2        1.3852  113.48  179.68  126.60  1.6528
IC N1   C2   N3    C4        1.4147  113.48    0.41  127.62  1.3764
IC C4   C2   *N3   H3        1.3764  127.62 -179.69  116.27  1.0046
IC C2   N3   C4    C5        1.3852  127.62    0.63  116.54  1.4415
IC C5   N3   *C4   O4        1.4415  116.54 -178.09  118.47  1.2311
IC C5   N1   *C6   H6        1.3772  123.28 -178.56  115.24  1.0930
IC C6   C4   *C5   C7        1.3772  117.37  175.54  116.90  1.4825
IC C4   C5   C7    N8        1.4415  116.90   58.69  109.04  1.5004
IC N8   C5   *C7   H71       1.5004  109.04  119.44  113.13  1.1014
IC H71  C5   *C7   H72       1.1014  113.13  124.46  109.58  1.1030
IC C5   C7   N8    C9        1.4825  109.04   92.83  122.18  1.5230
IC C9   C7   *N8   H81       1.5230  122.18  124.01  107.69  1.0072
IC C9   C7   *N8   H82       1.5230  122.18 -123.97  106.39  1.0131
IC C7   N8   C9    C10       1.5004  122.18  -64.75  116.44  1.5309
IC C10  N8   *C9   H91       1.5309  116.44  128.78  104.45  1.1047
IC C10  N8   *C9   H92       1.5309  116.44 -119.43  103.12  1.1069
IC N8   C9   C10   S11       1.5230  116.44  103.09  109.88  1.7849
IC S11  C9   *C10  H101      1.7849  109.88  114.42  109.60  1.1100
IC H101 C9   *C10  H102      1.1100  109.60  122.64  114.64  1.1055
IC C9   C10  S11   O11       1.5309  109.88  176.32  103.14  1.4419
IC O11  C10  *S11  O12       1.4419  103.14  120.44  103.92  1.4426
IC O11  C10  *S11  O13       1.4419  103.14 -120.38  101.82  1.4426
IC C6   N1   CM    HM1       1.3732  120.03    0.93  112.47  1.1144
IC HM1  N1   *CM   HM2       1.1144  112.47  119.33  111.08  1.1156
IC HM1  N1   *CM   HM3       1.1144  112.47 -120.57  111.55  1.1150

RESI BGAU           1.00 ! geranylated 5-methylaminomethyl-2-thiouracil, yxu
GROUP
ATOM N1      NG2R61 -0.25 !     H93  H82 H72  O4
ATOM C2      CG2R64  0.41 !      |   |(+) |   ||
ATOM S2      SG311  -0.14 !  H92-C9 -N8 -C7   C4
ATOM N3      NG2R62 -0.56 !      |   |   / \ /  \
ATOM C4      CG2R63  0.53 !     H91 H81 H71 C5   N3          C23            C29
ATOM O4      OG2D4  -0.53 !                 ||   ||           |              |
ATOM C5      CG2R62 -0.04 !              H6-C6   C2   C20    C22     C25    C27
ATOM C6      CG2R62  0.24 !                   \ / \  /   \  //  \   /   \  //  \
ATOM H6      HGR62   0.14 !                   N1   S2     C21    C24     C26    C28
ATOM C20     CG321   0.02 !                    \
ATOM H201    HGA2    0.09 !                     \
ATOM H202    HGA2    0.09 !                      \
GROUP
ATOM C7      CG324   0.20
ATOM H71     HGA2    0.09
ATOM H72     HGA2    0.09
ATOM N8      NG3P2  -0.52
ATOM H81     HGP2    0.38
ATOM H82     HGP2    0.38
ATOM C9      CG334   0.11
```

```
ATOM H91    HGA3    0.09
ATOM H92    HGA3    0.09
ATOM H93    HGA3    0.09
GROUP
ATOM C21    CG2D1  -0.15
ATOM H21    HGA4    0.15
ATOM C22    CG2D1  -0.00
GROUP
ATOM C23    CG331  -0.27
ATOM H231   HGA3    0.09
ATOM H232   HGA3    0.09
ATOM H233   HGA3    0.09
GROUP
ATOM C24    CG321  -0.18
ATOM H241   HGA2    0.09
ATOM H242   HGA2    0.09
GROUP
ATOM C25    CG321  -0.18
ATOM H251   HGA2    0.09
ATOM H252   HGA2    0.09
GROUP
ATOM C26    CG2D1  -0.15
ATOM H26    HGA4    0.15
ATOM C27    CG2D1  -0.00
GROUP
ATOM C28    CG331  -0.27
ATOM H281   HGA3    0.09
ATOM H282   HGA3    0.09
ATOM H283   HGA3    0.09
GROUP
ATOM C29    CG331  -0.27
ATOM H291   HGA3    0.09
ATOM H292   HGA3    0.09
ATOM H293   HGA3    0.09
GROUP
ATOM CM     CG331  -0.27
ATOM HM1    HGA3    0.09
ATOM HM2    HGA3    0.09
ATOM HM3    HGA3    0.09
BOND CM   HM1    CM   HM2   CM   HM3
BOND N1   C2      N1   C6      C2   S2      C2   N3      S2   C20
BOND N3   C4      C4   O4      C4   C5      C5   C6      C5   C7
BOND C6   H6      C7   H71     C7   H72     C7   N8      N8   H81
BOND N8   H82     N8   C9      C9   H91     C9   H92     C9   H93
BOND C20  H201    C20  H202    C20  C21     C21  H21     C21  C22
BOND C22  C23     C22  C24     C23  H231    C23  H232    C23  H233
BOND C24  H241    C24  H242    C24  C25     C25  H251    C25  H252
BOND C25  C26     C26  H26     C26  C27     C27  C28     C27  C29
BOND C28  H281    C28  H282    C28  H283    C29  H291    C29  H292
BOND C29  H293
BOND CM   N1
IMPR C4     C5     N3     O4
DONO H81  N8
DONO H82  N8
ACCE N3
ACCE O4   C4
IC C6   C2   *N1   CM        1.3585  118.86  179.52  120.25  1.4759
IC C6   N1   C2    S2        1.3585  118.86 -179.07  123.21  1.7530
IC S2   N1   *C2   N3        1.7530  123.21  177.61  119.48  1.3547
IC N1   C2   N3    C4        1.4155  119.48    1.61  122.17  1.3523
IC C2   N3   C4    C5        1.3547  122.17   -1.30  119.36  1.4458
IC C5   N3   *C4   O4        1.4458  119.36 -176.18  120.88  1.2278
IC C5   N1   *C6   H6        1.3697  122.79  179.68  115.50  1.0911
```

```
IC N1   C2   S2    C20       1.4155  123.21  175.47  106.58  1.8348
IC C2   S2   C20   C21       1.7530  106.58   83.57  109.74  1.5139
IC C21  S2   *C20  H201      1.5139  109.74  118.82  108.76  1.1128
IC H201 S2   *C20  H202      1.1128  108.76  115.12  110.37  1.1098
IC C6   C4   *C5   C7        1.3697  117.33  179.36  117.70  1.4808
IC C4   C5   C7    N8        1.4458  117.70   56.50  107.48  1.5030
IC N8   C5   *C7   H71       1.5030  107.48 -116.62  110.50  1.1060
IC H71  C5   *C7   H72       1.1060  110.50 -125.55  112.02  1.1039
IC C5   C7   N8    C9        1.4808  107.48  178.84  116.65  1.5031
IC C9   C7   *N8   H81       1.5031  116.65 -127.34  110.42  1.0104
IC H81  C7   *N8   H82       1.0104  110.42 -110.98  104.42  1.0261
IC C7   N8   C9    H91       1.5030  116.65  -65.05  107.18  1.1115
IC H91  N8   *C9   H92       1.1115  107.18  120.41  107.19  1.1115
IC H91  N8   *C9   H93       1.1115  107.18 -119.70  107.47  1.1113
IC S2   C20  C21   C22       1.8348  109.74 -126.39  128.95  1.3507
IC C22  C20  *C21  H21       1.3507  128.95 -179.08  113.64  1.1001
IC C20  C21  C22   C24       1.5139  128.95  179.95  119.53  1.5137
IC C24  C21  *C22  C23       1.5137  119.53  178.95  123.81  1.5098
IC C21  C22  C23   H231      1.3507  123.81 -119.00  110.55  1.1109
IC H231 C22  *C23  H232      1.1109  110.55  121.00  114.77  1.1089
IC H231 C22  *C23  H233      1.1109  110.55 -118.53  110.53  1.1117
IC C21  C22  C24   C25       1.3507  119.53  105.76  113.64  1.5520
IC C25  C22  *C24  H241      1.5520  113.64 -121.76  111.70  1.1155
IC H241 C22  *C24  H242      1.1155  111.70 -116.60  108.82  1.1130
IC C22  C24  C25   C26       1.5137  113.64  176.65  112.00  1.5132
IC C26  C24  *C25  H251      1.5132  112.00 -123.62  109.51  1.1125
IC H251 C24  *C25  H252      1.1125  109.51 -115.31  108.89  1.1138
IC C24  C25  C26   C27       1.5520  112.00  -96.28  127.68  1.3490
IC C27  C25  *C26  H26       1.3490  127.68  178.01  114.72  1.1001
IC C25  C26  C27   C28       1.5132  127.68  179.63  120.94  1.5053
IC C28  C26  *C27  C29       1.5053  120.94 -178.12  124.91  1.5051
IC C26  C27  C28   H281      1.3490  120.94  121.58  110.72  1.1111
IC H281 C27  *C28  H282      1.1111  110.72  118.95  110.26  1.1105
IC H281 C27  *C28  H283      1.1111  110.72 -120.68  113.44  1.1122
IC C26  C27  C29   H291      1.3490  124.91 -115.90  110.65  1.1109
IC H291 C27  *C29  H292      1.1109  110.65  121.13  113.88  1.1115
IC H291 C27  *C29  H293      1.1109  110.65 -118.68  110.17  1.1107
IC C6   N1   CM    HM1       1.3585  120.89 -121.70  111.75  1.1148
IC HM1  N1   *CM   HM2       1.1148  111.75  120.26  113.05  1.1147
IC HM1  N1   *CM   HM3       1.1148  111.75 -119.94  111.13  1.1162

RESI BGCU            0.00 ! geranylated 5-carboxymethylaminomethyl-2-thiouracil, yxu
!RING 6 N1 C2 N3 C4 C5 C6
GROUP
ATOM N1     NG2R61 -0.25 !    O11  O12 (-)
ATOM C2     CG2R64  0.41 !      \\ /
ATOM S2     SG311  -0.14 !       C10
ATOM N3     NG2R62 -0.56 !       |   H82 H72  O4
ATOM C4     CG2R63  0.53 !       |   |(+) |   ||
ATOM O4     OG2D4  -0.53 !   H92-C9 -N8 -C7   C4
ATOM C5     CG2R62 -0.04 !       |   |   / \ /  \
ATOM C6     CG2R62  0.24 !      H91 H81 H71 C5   N3          C23            C29
ATOM H6     HGR62   0.14 !                  ||   ||           |              |
ATOM C20    CG321   0.02 !               H6-C6   C2   C20    C22     C25    C27
ATOM H201   HGA2    0.09 !                   \  / \  /   \  //  \   /   \  //  \
ATOM H202   HGA2    0.09 !                    N1   S2     C21    C24     C26    C28
GROUP                    !                     \
ATOM C7     CG324   0.04 !                      \
ATOM H71    HGA2    0.09 !                       \
ATOM H72    HGA2    0.09
ATOM N8     NG3P2  -0.34
ATOM H81    HGP2    0.34
ATOM H82    HGP2    0.34
```

```
ATOM C9     CG324  -0.01
ATOM H91    HGA2    0.09
ATOM H92    HGA2    0.09
ATOM C10    CG2O3   0.59
ATOM O11    OG2D2  -0.66
ATOM O12    OG2D2  -0.66
GROUP                    ! !! PATCH 5UHG for the non-ionic tautomer
ATOM C21    CG2D1  -0.15
ATOM H21    HGA4    0.15
ATOM C22    CG2D1  -0.00
GROUP
ATOM C23    CG331  -0.27
ATOM H231   HGA3    0.09
ATOM H232   HGA3    0.09
ATOM H233   HGA3    0.09
GROUP
ATOM C24    CG321  -0.18
ATOM H241   HGA2    0.09
ATOM H242   HGA2    0.09
GROUP
ATOM C25    CG321  -0.18
ATOM H251   HGA2    0.09
ATOM H252   HGA2    0.09
GROUP
ATOM C26    CG2D1  -0.15
ATOM H26    HGA4    0.15
ATOM C27    CG2D1  -0.00
GROUP
ATOM C28    CG331  -0.27
ATOM H281   HGA3    0.09
ATOM H282   HGA3    0.09
ATOM H283   HGA3    0.09
GROUP
ATOM C29    CG331  -0.27
ATOM H291   HGA3    0.09
ATOM H292   HGA3    0.09
ATOM H293   HGA3    0.09
GROUP
ATOM CM     CG331  -0.27
ATOM HM1    HGA3    0.09
ATOM HM2    HGA3    0.09
ATOM HM3    HGA3    0.09
BOND CM   HM1    CM   HM2   CM   HM3
BOND N1   C2      N1   C6      C2   S2      C2   N3      S2   C20
BOND N3   C4      C4   O4      C4   C5      C5   C6      C5   C7
BOND C6   H6      C7   H71     C7   H72     C7   N8      N8   H81
BOND N8   H82     N8   C9      C9   H91     C9   H92     C9   C10
BOND C10  O11     C10  O12
BOND C20  H201    C20  H202    C20  C21     C21  H21     C21  C22
BOND C22  C23     C22  C24     C23  H231    C23  H232    C23  H233
BOND C24  H241    C24  H242    C24  C25     C25  H251    C25  H252
BOND C25  C26     C26  H26     C26  C27     C27  C28     C27  C29
BOND C28  H281    C28  H282    C28  H283    C29  H291    C29  H292
BOND C29  H293
BOND CM   N1
IMPR C4     C5     N3     O4     C10    O11    O12    C9
DONO H81  N8
DONO H82  N8
ACCE O4   C4
ACCE N3
ACCE O11  C10
ACCE O12  C10
IC C6   C2   *N1   CM        1.3588  118.51  179.28  119.98  1.4734
```

```
IC C6   N1   C2    S2        1.3588  118.51 -177.38  123.69  1.7532
IC S2   N1   *C2   N3        1.7532  123.69  177.74  119.39  1.3526
IC N1   C2   N3    C4        1.4181  119.39    2.24  122.51  1.3566
IC C2   N3   C4    C5        1.3526  122.51   -5.17  119.10  1.4495
IC C5   N3   *C4   O4        1.4495  119.10  179.70  120.42  1.2264
IC C5   N1   *C6   H6        1.3712  123.46  178.12  115.93  1.0930
IC N1   C2   S2    C20       1.4181  123.69  145.98  104.90  1.8366
IC C2   S2   C20   C21       1.7532  104.90   98.82  110.66  1.5099
IC C21  S2   *C20  H201      1.5099  110.66  118.43  108.25  1.1130
IC H201 S2   *C20  H202      1.1130  108.25  114.54  110.72  1.1101
IC C6   C4   *C5   C7        1.3712  116.82 -179.66  119.43  1.4817
IC C4   C5   C7    N8        1.4495  119.43   76.65  107.28  1.4927
IC N8   C5   *C7   H71       1.4927  107.28 -118.70  112.37  1.1043
IC H71  C5   *C7   H72       1.1043  112.37 -126.03  111.11  1.1048
IC C5   C7   N8    C9        1.4817  107.28  -99.83  120.72  1.5396
IC C9   C7   *N8   H81       1.5396  120.72 -115.73  110.41  1.0245
IC H81  C7   *N8   H82       1.0245  110.41 -115.49  112.30  1.0035
IC C7   N8   C9    C10       1.4927  120.72 -134.44  109.01  1.5346
IC C10  N8   *C9   H91       1.5346  109.01  119.39  106.96  1.0951
IC H91  N8   *C9   H92       1.0951  106.96  120.02  106.59  1.0946
IC N8   C9   C10   O11       1.5396  109.01 -179.98  115.32  1.2543
IC O11  C9   *C10  O12       1.2543  115.32  179.91  116.27  1.2607
IC S2   C20  C21   C22       1.8366  110.66 -117.80  129.10  1.3597
IC C22  C20  *C21  H21       1.3597  129.10  179.33  113.90  1.0970
IC C20  C21  C22   C24       1.5099  129.10 -178.06  119.52  1.5138
IC C24  C21  *C22  C23       1.5138  119.52 -179.89  123.44  1.5043
IC C21  C22  C23   H231      1.3597  123.44 -127.52  111.34  1.1113
IC H231 C22  *C23  H232      1.1113  111.34  121.80  114.77  1.1103
IC H231 C22  *C23  H233      1.1113  111.34 -118.73  109.94  1.1133
IC C21  C22  C24   C25       1.3597  119.52  101.49  113.10  1.5525
IC C25  C22  *C24  H241      1.5525  113.10 -122.04  111.55  1.1140
IC H241 C22  *C24  H242      1.1140  111.55 -116.79  108.85  1.1127
IC C22  C24  C25   C26       1.5138  113.10  179.44  114.49  1.5177
IC C26  C24  *C25  H251      1.5177  114.49 -124.42  108.33  1.1118
IC H251 C24  *C25  H252      1.1118  108.33 -114.11  108.37  1.1127
IC C24  C25  C26   C27       1.5525  114.49 -105.53  128.09  1.3477
IC C27  C25  *C26  H26       1.3477  128.09  178.60  114.24  1.0999
IC C25  C26  C27   C28       1.5177  128.09 -176.34  121.27  1.5087
IC C28  C26  *C27  C29       1.5087  121.27  179.04  125.04  1.5055
IC C26  C27  C28   H281      1.3477  121.27  121.41  110.24  1.1121
IC H281 C27  *C28  H282      1.1121  110.24  118.68  110.79  1.1102
IC H281 C27  *C28  H283      1.1121  110.24 -120.19  113.22  1.1119
IC C26  C27  C29   H291      1.3477  125.04 -116.32  110.69  1.1111
IC H291 C27  *C29  H292      1.1111  110.69  121.25  113.64  1.1102
IC H291 C27  *C29  H293      1.1111  110.69 -118.66  110.30  1.1107
IC C6   N1   CM    HM1       1.3588  121.51 -121.12  111.50  1.1151
IC HM1  N1   *CM   HM2       1.1151  111.50  120.00  112.74  1.1142
IC HM1  N1   *CM   HM3       1.1151  111.50 -119.96  111.57  1.1147

!!***cytosines***

RESI B2SC           0.00 ! 2-thiocytosine, yxu
GROUP
ATOM N1     NG2R61 -0.21 !              H42  H41
ATOM C2     CG2R63  0.31 !                \  /
ATOM S2     SG2D1  -0.18 !                 N4
ATOM N3     NG2R62 -0.77 !                 |
ATOM C4     CG2R64  0.48 !                 C4
ATOM N4     NG2S3  -0.69 !                /  \\
ATOM H41    HGP4    0.36 !            H5-C5   N3
ATOM H42    HGP4    0.36 !               ||   |
ATOM C5     CG2R61 -0.13 !            H6-C6   C2
ATOM H5     HGR62   0.14 !                \  / \\
```

```
ATOM C6      CG2R61  0.11 !                   N1   S2
ATOM H6      HGR62   0.22 !                    \
GROUP                     !                     \
ATOM CM      CG331  -0.27
ATOM HM1     HGA3    0.09
ATOM HM2     HGA3    0.09
ATOM HM3     HGA3    0.09
BOND CM   HM1    CM   HM2   CM   HM3
BOND N1   C2   N1   C6   C2   S2   C2   N3
BOND N3   C4   C4   C5   C4   N4   N4   H41
BOND N4   H42  C5   H5   C5   C6   C6   H6
BOND CM   N1
IMPR C2   N1   N3   S2        C4   C5   N3   N4        N4   H41  H42  C4
DONO H41  N4
DONO H42  N4
ACCE N3
ACCE S2   C2
IC C6   C2   *N1   CM        1.4287   98.10  180.00  113.73  1.4813
IC C6   N1   C2    N3        1.4287   98.10  -68.59  116.10  1.3713
IC N3   N1   *C2   S2        1.3713  116.10  180.00  121.94  1.6586
IC N1   C2   N3    C4        1.4417  116.10   47.59  114.13  1.3478
IC C2   N3   C4    N4        1.3713  114.13  174.70  117.63  1.3293
IC N4   N3   *C4   C5        1.3293  117.63 -179.90  121.22  1.4075
IC N3   C4   N4    H41       1.3478  117.63 -178.93  124.01  0.9895
IC H41  C4   *N4   H42       0.9895  124.01  178.71  114.83  0.9963
IC C6   C4   *C5   H5        1.4351  114.09 -171.19  122.37  1.0715
IC C5   N1   *C6   H6        1.4351  119.29  180.00  111.74  1.0801
IC C6   N1   CM    HM1       1.4287  110.94   46.17  115.47  1.1114
IC HM1  N1   *CM   HM2       1.1114  115.47  120.21  109.86  1.1158
IC HM1  N1   *CM   HM3       1.1114  115.47 -122.39  110.79  1.1141

RESI B5MC           0.00 ! 5-Methylcytosine yxu
GROUP
ATOM N1      NG2R61 -0.12 !                H42  H41
ATOM C2      CG2R63  0.50 !                  \  /
ATOM O2      OG2D4  -0.45 !                   N4
ATOM N3      NG2R62 -0.79 !                   |
ATOM C4      CG2R64  0.62 !          H51       C4
ATOM N4      NG2S3  -0.65 !             \     /  \\
ATOM H41     HGP4    0.32 !         H52--CM5-C5   N3
ATOM H42     HGP4    0.32 !             /    ||   |
ATOM C5      CG2R62  0.04 !          H53  H6-C6   C2
ATOM C6      CG2R62  0.02 !                  \  / \\
ATOM H6      HGR62   0.19 !                   N1   O2
ATOM CM5     CG331  -0.21 !                    \
ATOM H51     HGA3    0.07 !                     \
ATOM H52     HGA3    0.07 !                      \
ATOM H53     HGA3    0.07
GROUP
ATOM CM      CG331  -0.27
ATOM HM1     HGA3    0.09
ATOM HM2     HGA3    0.09
ATOM HM3     HGA3    0.09
BOND CM   HM1    CM   HM2   CM   HM3
BOND N1   C2      N1   C6      C2   O2      C2   N3
BOND N3   C4      C4   C5      C4   N4      N4   H41
BOND N4   H42     C5   C6      C5   CM5     CM5  H51
BOND CM5  H52     CM5  H53     C6   H6
BOND CM   N1
IMPR C2   N1   N3   O2        C4   C5   N3   N4        N4   H41  H42  C4
DONO H41  N4
DONO H42  N4
ACCE O2   C2
```

```
ACCE N3
IC C2   C6   *N1   CM        1.4259  119.73  180.00  121.16  1.4732
IC C6   N1   C2    N3        1.3647  119.73    0.00  119.03  1.3604
IC N3   N1   *C2   O2        1.3604  119.03  180.00  119.10  1.2297
IC N1   C2   N3    C4        1.4259  119.03    0.00  121.21  1.3400
IC C2   N3   C4    C5        1.3604  121.21    0.00  121.30  1.4511
IC C5   N3   *C4   N4        1.4511  121.30  180.00  117.23  1.3380
IC N3   C4   N4    H41       1.3400  117.23  180.00  123.62  0.9926
IC H41  C4   *N4   H42       0.9926  123.62  180.00  115.47  0.9969
IC C5   N1   *C6   H6        1.3691  122.06  180.00  116.64  1.0891
IC C6   C4   *C5   CM5       1.3691  116.67  180.00  121.46  1.5026
IC C4   C5   CM5   H51       1.4511  121.46  180.00  111.45  1.1127
IC H51  C5   *CM5  H52       1.1127  111.45  119.83  110.93  1.1131
IC H51  C5   *CM5  H53       1.1127  111.45 -119.83  110.93  1.1131
IC C6   N1   CM    HM1       1.3647  121.16    0.00  112.91  1.1117
IC HM1  N1   *CM   HM2       1.1117  112.91  120.78  110.68  1.1160
IC HM1  N1   *CM   HM3       1.1117  112.91 -120.78  110.68  1.1160

RESI BHMC           0.00 ! 5-hydroxymethylcytosine     5-(hydroxymethyl)cytidine, yxu
GROUP
ATOM N1     NG2R61 -0.12 !         H7O   H42  H41
ATOM C2     CG2R63  0.50 !           \      \  /
ATOM O2     OG2D4  -0.45 !            O7    N4
ATOM N3     NG2R62 -0.79 !             \     |
ATOM C4     CG2R64  0.62 !         H71-C7   C4
ATOM N4     NG2S3  -0.65 !             / \ /  \\
ATOM H41    HGP4    0.32 !           H72  C5   N3
ATOM H42    HGP4    0.32 !                ||    |
ATOM C5     CG2R62  0.04 !             H6-C6   C2
ATOM C6     CG2R62  0.02 !                 \  / \\
ATOM H6     HGR62   0.19 !                  N1   O2
GROUP                    !                   \
ATOM C7     CG321   0.05 !                    \
ATOM H71    HGA2    0.09 !                     \
ATOM H72    HGA2    0.09
ATOM O7     OG311  -0.65
ATOM H7O    HGP1    0.42
GROUP
ATOM CM     CG331  -0.27
ATOM HM1    HGA3    0.09
ATOM HM2    HGA3    0.09
ATOM HM3    HGA3    0.09
BOND CM   HM1    CM   HM2   CM   HM3
BOND N1   C2   N1   C6   C2   O2   C2   N3
BOND N3   C4   C4   C5   C4   N4   N4   H41
BOND N4   H42  C5   C7   C5   C6   C6   H6
BOND C7   H71  C7   H72  C7   O7   O7   H7O
BOND CM   N1
IMPR C2   N1   N3   O2        C4   C5   N3   N4        N4   H41  H42  C4
DONO H41  N4
DONO H42  N4
DONO H7O  O7
ACCE O2   C2
ACCE N3
ACCE O7
IC C2   C6   *N1   CM        1.4229  119.42  178.85  121.34  1.4744
IC C6   N1   C2    N3        1.3634  119.42   -1.40  119.17  1.3572
IC N3   N1   *C2   O2        1.3572  119.17 -179.96  118.97  1.2299
IC N1   C2   N3    C4        1.4229  119.17   -0.04  121.15  1.3372
IC C2   N3   C4    C5        1.3572  121.15    2.10  121.45  1.4433
IC C5   N3   *C4   N4        1.4433  121.45 -179.64  116.98  1.3321
IC N3   C4   N4    H41       1.3372  116.98  165.97  121.08  0.9953
IC H41  C4   *N4   H42       0.9953  121.08 -164.57  115.30  0.9949
```

```
IC C5   N1   *C6   H6        1.3689  122.22 -179.73  116.41  1.0899
IC C6   C4   *C5   C7        1.3689  116.53  178.87  121.24  1.4975
IC C4   C5   C7    O7        1.4433  121.24   66.48  110.49  1.4253
IC O7   C5   *C7   H71       1.4253  110.49 -121.88  108.70  1.1147
IC H71  C5   *C7   H72       1.1147  108.70 -118.23  108.84  1.1149
IC C5   C7   O7    H7O       1.4975  110.49  175.67  108.59  0.9640
IC C6   N1   CM    HM1       1.3634  121.34    1.46  112.94  1.1126
IC HM1  N1   *CM   HM2       1.1126  112.94  120.72  110.67  1.1148
IC HM1  N1   *CM   HM3       1.1126  112.94 -120.84  110.72  1.1149

RESI B5FC           0.00 ! 5-formylcytosine, yxu
GROUP
ATOM N1     NG2R61 -0.08 !              H42  H41
ATOM C2     CG2R63  0.62 !                \  /
ATOM O2     OG2D4  -0.47 !                 N4
ATOM N3     NG2R62 -0.83 !                 |
ATOM C4     CG2R64  0.61 !           O7    C4
ATOM N4     NG2S3  -0.75 !           ||   /  \\
ATOM H41    HGP4    0.37 !        H7-C7 -C5   N3
ATOM H42    HGP4    0.37 !               ||   |
ATOM C5     CG2R62  0.04 !            H6-C6   C2
ATOM C6     CG2R62  0.15 !                \  / \\
ATOM H6     HGR62   0.17 !                 N1   O2
ATOM C7     CG2O4   0.17 !                  \
ATOM H7     HGR52   0.08 !                   \
ATOM O7     OG2D1  -0.45 !                    \
GROUP
ATOM CM     CG331  -0.27
ATOM HM1    HGA3    0.09
ATOM HM2    HGA3    0.09
ATOM HM3    HGA3    0.09
BOND CM   HM1    CM   HM2   CM   HM3
BOND N1   C2   N1   C6   C2   O2   C2   N3
BOND N3   C4   C4   C5   C4   N4   N4   H41
BOND N4   H42  C5   C7   C5   C6   C6   H6
BOND C7   H7   C7   O7
BOND CM   N1
IMPR C2   N1   N3   O2        C4   C5   N3   N4        N4   H41  H42  C4
IMPR C7   C5   O7   H7
DONO H41  N4
DONO H42  N4
ACCE O2   C2
ACCE N3
ACCE O7   C7
IC C2   C6   *N1   CM        1.4259  119.16  180.00  121.48  1.4752
IC C6   N1   C2    N3        1.3647  119.16    0.00  119.02  1.3623
IC N3   N1   *C2   O2        1.3623  119.02  180.00  119.08  1.2297
IC N1   C2   N3    C4        1.4259  119.02    0.00  121.26  1.3337
IC C2   N3   C4    C5        1.3623  121.26    0.00  121.23  1.4322
IC C5   N3   *C4   N4        1.4322  121.23  180.00  116.05  1.3323
IC N3   C4   N4    H41       1.3337  116.05  180.00  126.01  0.9938
IC H41  C4   *N4   H42       0.9938  126.01  180.00  113.23  0.9977
IC C5   N1   *C6   H6        1.3655  121.97  180.00  117.26  1.0926
IC C6   C4   *C5   C7        1.3655  117.34  180.00  123.77  1.4568
IC C4   C5   C7    O7        1.4322  123.77  180.00  124.76  1.2132
IC O7   C5   *C7   H7        1.2132  124.76  180.00  116.83  1.1107
IC C6   N1   CM    HM1       1.3647  121.48    0.00  112.77  1.1128
IC HM1  N1   *CM   HM2       1.1128  112.77  120.63  110.78  1.1150
IC HM1  N1   *CM   HM3       1.1128  112.77 -120.63  110.78  1.1150

RESI B4MC           0.00 ! N4-methylcytosine yxu
GROUP
ATOM N1     NG2R61 -0.12 !           H41 H42 H43
```

```
ATOM C2      CG2R63  0.52 !                \ | /
ATOM O2      OG2D4  -0.49 !                 CM4   H4
ATOM N3      NG2R62 -0.67 !                    \  /
ATOM C4      CG2R64  0.64 !                     N4
ATOM N4      NG311  -0.52 !                     |
ATOM H4      HGPAM1  0.32 !                     C4
ATOM C5      CG2R62 -0.05 !                    /  \\
ATOM H5      HGR62   0.02 !               H5-C5   N3
ATOM C6      CG2R62  0.06 !                   ||   |
ATOM H6      HGR62   0.16 !               H6-C6   C2
ATOM CM4     CG331  -0.14 !                    \  / \\
ATOM H41     HGA3    0.09 !                     N1   O2
ATOM H42     HGA3    0.09 !                      \
ATOM H43     HGA3    0.09 !                       \
GROUP                     !                        \
ATOM CM      CG331  -0.27
ATOM HM1     HGA3    0.09
ATOM HM2     HGA3    0.09
ATOM HM3     HGA3    0.09
BOND CM   HM1    CM   HM2   CM   HM3
BOND N1   C2      N1   C6      C2   O2      C2   N3
BOND N3   C4      C4   C5      C4   N4      N4   H4
BOND N4   CM4     CM4  H41     CM4  H42     CM4  H43
BOND C5   H5      C5   C6      C6   H6
BOND CM   N1
IMPR C2   N1   N3   O2        C4   C5   N3   N4       ! N4   C4   CM4   H4
DONO H4   N4
ACCE O2   C2
ACCE N3
IC C2   C6   *N1   CM       1.4259  119.54  179.90  121.33  1.4735
IC C6   N1   C2    N3       1.3631  119.54   -0.04  119.19  1.3606
IC N3   N1   *C2   O2       1.3606  119.19  179.91  119.01  1.2300
IC N1   C2   N3    C4       1.4259  119.19   -0.19  121.15  1.3481
IC C2   N3   C4    N4       1.3606  121.15  179.51  117.18  1.3622
IC N4   N3   *C4   C5       1.3622  117.18 -179.11  120.60  1.4355
IC N3   C4   N4    CM4      1.3481  117.18 -165.97  127.51  1.4815
IC CM4  C4   *N4   H4       1.4815  127.51  160.82  115.37  1.0184
IC C6   C4   *C5   H5       1.3664  117.91 -179.72  122.07  1.0893
IC C5   N1   *C6   H6       1.3664  121.61  179.94  116.87  1.0912
IC C4   N4   CM4   H41      1.3622  127.51 -157.50  110.39  1.1132
IC H41  N4   *CM4  H42      1.1132  110.39  119.55  111.76  1.1119
IC H41  N4   *CM4  H43      1.1132  110.39 -119.48  110.76  1.1127
IC C6   N1   CM    HM1      1.3631  121.33  120.82  110.72  1.1155
IC HM1  N1   *CM   HM2      1.1155  110.72  118.60  110.79  1.1151
IC HM1  N1   *CM   HM3      1.1155  110.72 -120.70  112.86  1.1128

RESI B4AC           0.00 ! N4-acetylcytosine    ! adjusted, yxu
GROUP
ATOM N1      NG2R61 -0.11 !               H82
ATOM C2      CG2R63  0.52 !                |
ATOM O2      OG2D4  -0.54 !           H81-C8 -H83
ATOM N3      NG2R62 -0.79 !                |
ATOM C4      CG2R64  0.65 !               C7   H4
ATOM N4      NG2S1  -0.63 !              // \  /
ATOM H4      HGP1    0.37 !             O7   N4
ATOM C5      CG2R62 -0.08 !                  |
ATOM H5      HGR62   0.12 !                  C4
ATOM C6      CG2R62  0.14 !                 /  \\
ATOM H6      HGR62   0.20 !             H5-C5   N3
ATOM C7      CG2O1   0.59 !                ||   |
ATOM O7      OG2D1  -0.50 !             H6-C6   C2
ATOM C8      CG331  -0.21 !                 \  / \\
ATOM H81     HGA3    0.09 !                  N1   O2
```

```
ATOM H82    HGA3    0.09 !                   \
ATOM H83    HGA3    0.09 !                    \
GROUP                    !                     \
ATOM CM     CG331  -0.27
ATOM HM1    HGA3    0.09
ATOM HM2    HGA3    0.09
ATOM HM3    HGA3    0.09
BOND CM   HM1    CM   HM2   CM   HM3
BOND N1   C2   N1   C6   C2   O2   C2   N3
BOND N3   C4   C4   C5   C4   N4   N4   H4
BOND N4   C7   C7   C8   C7   O7   C8   H81
BOND C8   H82  C8   H83  C5   C6   C5   H5
BOND C6   H6
BOND CM   N1
IMPR C2   N1   N3   O2        C4   C5   N3   N4       C7   C8   N4   O7
DONO H4   N4
ACCE O2   C2
ACCE N3
ACCE O7
IC C2   C6   *N1   CM        1.4233  119.16 -179.41  121.57  1.4755
IC C6   N1   C2    N3        1.3607  119.16    0.51  118.98  1.3582
IC N3   N1   *C2   O2        1.3582  118.98  180.00  119.06  1.2278
IC N1   C2   N3    C4        1.4233  118.98    1.15  121.36  1.3420
IC C2   N3   C4    N4        1.3582  121.36 -176.54  116.21  1.3675
IC N4   N3   *C4   C5        1.3675  116.21  173.95  120.81  1.4147
IC N3   C4   N4    C7        1.3420  116.21 -141.03  131.23  1.3252
IC C7   C4   *N4   H4        1.3252  131.23  164.66  108.87  0.9893
IC C6   C4   *C5   H5        1.3686  117.94 -178.25  121.19  1.0849
IC C5   N1   *C6   H6        1.3686  121.70  179.13  116.35  1.0915
IC C4   N4   C7    O7        1.3675  131.23  177.98  119.14  1.2178
IC O7   N4   *C7   C8        1.2178  119.14 -178.94  120.78  1.4854
IC N4   C7   C8    H81       1.3252  120.78  163.58  108.55  1.1117
IC H81  C7   *C8   H82       1.1117  108.55  118.63  110.45  1.1083
IC H81  C7   *C8   H83       1.1117  108.55 -118.68  110.77  1.1103
IC C6   N1   CM    HM1       1.3607  121.57  120.51  110.55  1.1153
IC HM1  N1   *CM   HM2       1.1153  110.55  117.99  110.57  1.1160
IC HM1  N1   *CM   HM3       1.1153  110.55 -121.03  113.21  1.1115

RESI B3MC           1.00 ! protonated N3-methylcytosine  yxu
GROUP
ATOM N1     NG2R61 -0.11 !              H42  H41
ATOM C2     CG2R63  0.55 !                \  /
ATOM O2     OG2D4  -0.33 !             (+) N4      H31
ATOM N3     NG2P1  -0.35 !                 ||     /
ATOM C4     CG2R64  0.68 !                 C4   CN3-H32
ATOM N4     NG2P1  -0.76 !                /  \ /  \
ATOM H41    HGP2    0.39 !            H5-C5   N3   H33
ATOM H42    HGP2    0.39 !               ||    |
ATOM C5     CG2R62 -0.18 !            H6-C6   C2
ATOM H5     HGR62   0.09 !                \  / \\
ATOM C6     CG2R62  0.16 !                 N1   O2
ATOM H6     HGR62   0.20 !                   \
ATOM CN3    CG334   0.00 !                    \
ATOM H31    HGA3    0.09 !                     \
ATOM H32    HGA3    0.09
ATOM H33    HGA3    0.09
GROUP
ATOM CM     CG331  -0.27
ATOM HM1    HGA3    0.09
ATOM HM2    HGA3    0.09
ATOM HM3    HGA3    0.09
BOND CM   HM1    CM   HM2   CM   HM3
BOND N1   C2   N1   C6   C2   O2   C2   N3
```

```
BOND N3   C4   N3   CN3  CN3  H31  CN3  H32
BOND CN3  H33  C4   N4   C4   C5   N4   H41
BOND N4   H42  C5   C6   C5   H5   C6   H6
BOND CM   N1
IMPR C2   N3   N1   O2        C4   C5   N3   N4        N4   H41  H42  C4
DONO H41  N4
DONO H42  N4
ACCE O2   C2
IC C6   C2   *N1   CM        1.3655  120.43  179.79  118.64  1.4739
IC C6   N1   C2    N3        1.3655  120.43    0.21  117.04  1.4345
IC N3   N1   *C2   O2        1.4345  117.04 -179.98  119.13  1.2319
IC N1   C2   N3    C4        1.4226  117.04   -0.03  120.84  1.3724
IC C4   C2   *N3   CN3       1.3724  120.84  179.93  117.22  1.4851
IC C2   N3   C4    N4        1.4345  120.84  179.79  119.04  1.3468
IC N4   N3   *C4   C5        1.3468  119.04 -179.97  120.34  1.4137
IC N3   C4   N4    H41       1.3724  119.04 -179.90  120.63  0.9989
IC H41  C4   *N4   H42       0.9989  120.63  179.75  119.67  0.9958
IC C6   C4   *C5   H5        1.3690  118.89  179.92  120.91  1.0887
IC C5   N1   *C6   H6        1.3690  122.46 -179.94  116.18  1.0955
IC C2   N3   CN3   H31       1.4345  117.22  120.30  111.55  1.1150
IC H31  N3   *CN3  H32       1.1150  111.55  121.24  111.44  1.1142
IC H31  N3   *CN3  H33       1.1150  111.55 -119.37  113.21  1.1153
IC C6   N1   CM    HM1       1.3655  120.93 -122.14  111.34  1.1161
IC HM1  N1   *CM   HM2       1.1161  111.34  120.36  113.00  1.1142
IC HM1  N1   *CM   HM3       1.1161  111.34 -119.24  111.35  1.1167

RESI B1PC          0.00 !  1H-pseudoisocytositidine, yxu
!RING 6 N1 C2 N3 C4 C5 C6
GROUP
ATOM N1     NG2R61 -0.36 !               H21  H22
ATOM H1     HGP1    0.30 !                 \  /
ATOM C2     CG2R64  0.64 !                  N2
ATOM N2     NG2S3  -0.66 !                  |
ATOM H21    HGP4    0.31 !                  C2
ATOM H22    HGP4    0.31 !                 /  \\
ATOM N3     NG2R62 -0.77 !             H1-N1   N3
ATOM C4     CG2R63  0.53 !                |     |
ATOM O4     OG2D4  -0.47 !             H6-C6   C4
ATOM C5     CG2R62 -0.13 !                 \\ / \\
ATOM C6     CG2R62  0.17 !                  C5   O4
ATOM H6     HGR62   0.13 !                   \
GROUP                    !                    \
ATOM CM     CG331  -0.27
ATOM HM1    HGA3    0.09
ATOM HM2    HGA3    0.09
ATOM HM3    HGA3    0.09
BOND CM   HM1    CM   HM2   CM   HM3
BOND N1   C2   N1   H1   N1   C6   C2   N3
BOND C2   N2   N2  H21   N2  H22   N3   C4
BOND C4   O4   C4   C5   C5   C6   C6   H6
BOND CM   C5
IMPR C2   N1   N3   N2        N2   H22  H21  C2        C4   C5   N3   O4
DONO H22  N2
DONO H21  N2
DONO H1   N1
ACCE N3
ACCE O4
IC C2   C6   *N1   H1        1.3808  118.77  180.00  124.43  1.0010
IC C6   N1   C2    N3        1.3582  118.77    0.00  122.59  1.3217
IC N3   N1   *C2   N2        1.3217  122.59  180.00  116.96  1.3228
IC N1   C2   N2    H21       1.3808  116.96  180.00  115.23  0.9967
IC H21  C2   *N2   H22       0.9967  115.23  180.00  124.28  0.9900
IC N1   C2   N3    C4        1.3808  122.59    0.00  120.60  1.3623
```

```
IC C2   N3   C4    C5        1.3217  120.60    0.00  118.96  1.4595
IC C5   N3   *C4   O4        1.4595  118.96  180.00  120.04  1.2303
IC C5   N1   *C6   H6        1.3704  121.80  180.00  117.89  1.0916
IC C6   C4   *C5   CM        1.3704  117.29  180.00  120.35  1.5023
IC C4   C5   CM    HM1       1.4595  120.35  180.00  112.30  1.1109
IC HM1  C5   *CM   HM2       1.1109  112.30  120.74  110.39  1.1134
IC HM1  C5   *CM   HM3       1.1109  112.30 -120.74  110.39  1.1134

RESI B3PC           0.00 !  3H-pseudoisocytositidine, yxu
!RING 6 N1 C2 N3 C4 C5 C6
GROUP
ATOM N1     NG2R62 -0.78 !                 H21  H22
ATOM C2     CG2R64  0.63 !                   \  /
ATOM N2     NG2S3  -0.56 !                    N2
ATOM H21    HGP4    0.31 !                    |
ATOM H22    HGP4    0.31 !                    C2     H3
ATOM N3     NG2R61 -0.51 !                  //  \  /
ATOM H3     HGP1    0.35 !                  N1    N3
ATOM C4     CG2R63  0.46 !                  |     |
ATOM O4     OG2D4  -0.48 !               H6-C6    C4
ATOM C5     CG2R62 -0.16 !                   \\ / \\
ATOM C6     CG2R62  0.28 !                    C5    O4
ATOM H6     HGR62   0.15 !                     \
GROUP                    !                      \
ATOM CM     CG331  -0.27
ATOM HM1    HGA3    0.09
ATOM HM2    HGA3    0.09
ATOM HM3    HGA3    0.09
BOND CM   HM1    CM   HM2   CM   HM3
BOND N1   C2   N1   C6   C2   N2   C2   N3
BOND N2  H21   N2  H22   N3   H3   N3   C4
BOND C4   O4   C4   C5   C5   C6   C6   H6
BOND CM   C5
IMPR C2   N3   N1   N2        N2   H22  H21  C2        C4   C5   N3   O4
DONO H22  N2
DONO H21  N2
DONO H3   N3
ACCE N1
ACCE O4
IC C6   N1   C2    N3        1.3976  117.00   -0.08  121.08  1.3625
IC N3   N1   *C2   N2        1.3625  121.08  179.58  121.75  1.3202
IC N1   C2   N2    H21       1.3308  121.75  179.74  123.11  0.9922
IC H21  C2   *N2   H22       0.9922  123.11 -179.53  115.81  0.9940
IC N1   C2   N3    C4        1.3308  121.08   -1.23  124.64  1.3670
IC C4   C2   *N3   H3        1.3670  124.64  178.85  119.73  0.9960
IC C2   N3   C4    C5        1.3625  124.64   -2.15  115.77  1.4461
IC C5   N3   *C4   O4        1.4461  115.77 -178.83  117.71  1.2277
IC C5   N1   *C6   H6        1.3792  124.05  176.74  117.92  1.0894
IC C6   C4   *C5   CM        1.3792  116.99  170.42  119.84  1.5032
IC C4   C5   CM    HM1       1.4461  119.84  155.14  111.54  1.1122
IC HM1  C5   *CM   HM2       1.1122  111.54  120.67  110.68  1.1126
IC HM1  C5   *CM   HM3       1.1122  111.54 -119.88  110.88  1.1133

!!***Adenines***

RESI B6MA           0.00 ! N6-methyladenosine
GROUP
ATOM N9     NG2R51 -0.01 !       H61
ATOM C8     CG2R53  0.43 !         \
ATOM H8     HGR52   0.08 !     H62-CM6   H6
ATOM N7     NG2R50 -0.85 !          / \  /
ATOM C5     CG2RC0  0.32 !       H63   N6
ATOM C6     CG2R64  0.45 !              |
```

```
ATOM N6     NG311  -0.45 !              C6
ATOM H6     HGPAM1  0.33 !             // \
ATOM N1     NG2R62 -0.78 !            N1   C5--N7\\
ATOM C2     CG2R64  0.49 !            |    ||      C8-H8
ATOM H2     HGR62   0.14 !            C2   C4--N9/
ATOM N3     NG2R62 -0.86 !           / \\ /      \
ATOM C4     CG2RC0  0.52 !         H2   N3        \
ATOM CM6    CG331  -0.08 !                         \
ATOM H61    HGA3    0.09 !                          \
ATOM H62    HGA3    0.09 !
ATOM H63    HGA3    0.09 !
GROUP                    !
ATOM CM     CG331  -0.27
ATOM HM1    HGA3    0.09
ATOM HM2    HGA3    0.09
ATOM HM3    HGA3    0.09
BOND CM   HM1    CM   HM2   CM   HM3
BOND N9   C8   N9   C4   C8   N7   C8   H8
BOND N7   C5   C5   C6   C5   C4   C6   N6
BOND C6   N1   N6   CM6  N6   H6   N1   C2
BOND C2   N3   C2   H2   N3   C4   CM6  H61
BOND CM6  H62  CM6  H63
BOND CM   N9
IMPR C6   C5   N1   N6       ! N6   C6   CM6   H6
DONO H6   N6
ACCE N3
ACCE N7
ACCE N1
IC C8   C4   *N9   CM        1.3830  106.03  179.68  126.91  1.4740
IC C4   N9   C8    N7        1.3497  107.34    0.02  112.90  1.3249
IC N7   N9   *C8   H8        1.3249  112.90 -179.86  122.61  1.0947
IC N9   C8   N7    C5        1.3736  112.90    0.40  103.62  1.3989
IC C4   N7   *C5   C6        1.3954  110.25 -178.17  133.05  1.4051
IC N7   C5   C6    N1        1.3989  133.05  177.05  117.60  1.3793
IC N1   C5   *C6   N6        1.3793  117.60  179.99  122.23  1.3815
IC C5   C6   N6    CM6       1.4051  122.23  167.61  118.33  1.4832
IC CM6  C6   *N6   H6        1.4832  118.33  180.00  112.50  1.0190
IC C5   C6   N1    C2        1.4051  117.60    1.54  120.04  1.3647
IC C6   N1   C2    N3        1.3793  120.04   -1.14  125.07  1.3600
IC N3   N1   *C2   H2        1.3600  125.07 -179.44  117.43  1.0938
IC C6   N6   CM6   H61       1.3815  118.33  174.59  110.48  1.1123
IC H61  N6   *CM6  H62       1.1123  110.48  120.34  111.22  1.1126
IC H61  N6   *CM6  H63       1.1123  110.48 -119.06  111.26  1.1131

RESI B2MA           0.00 ! 2-methyladenosine  yxu
GROUP
ATOM N9     NG2R51  0.04 !        H61  H62
ATOM C8     CG2R53  0.43 !          \  /
ATOM H8     HGR52   0.06 !           N6
ATOM N7     NG2R50 -0.84 !           |
ATOM C5     CG2RC0  0.29 !           C6
ATOM C6     CG2R64  0.57 !          // \
ATOM N6     NG2S3  -0.76 !         N1   C5--N7\\
ATOM H61    HGP4    0.36 !         |    ||      C8-H8
ATOM H62    HGP4    0.36 !   H21   C2   C4--N9/
ATOM N1     NG2R62 -0.78 !     \  / \\ /      \
ATOM C2     CG2R64  0.38 ! H22-CM2   N3        \
ATOM N3     NG2R62 -0.83 !      |               \
ATOM C4     CG2RC0  0.54 !     H23               \
ATOM CM2    CG331  -0.09 !                        \
ATOM H21    HGA3    0.09 !                         \
ATOM H22    HGA3    0.09 !                          \
ATOM H23    HGA3    0.09
```

```
GROUP
ATOM CM     CG331  -0.27
ATOM HM1    HGA3    0.09
ATOM HM2    HGA3    0.09
ATOM HM3    HGA3    0.09
BOND CM   HM1    CM   HM2   CM   HM3
BOND N9   C8   N9   C4   C8   N7   C8   H8
BOND N7   C5   C5   C6   C5   C4   C6   N6
BOND C6   N1   N6   H61  N6   H62  N1   C2
BOND C2   N3   C2   CM2  N3   C4   CM2  H21
BOND CM2  H22  CM2  H23
BOND CM   N9
IMPR N6   H61  H62  C6        C6   C5   N1   N6
DONO H61  N6
DONO H62  N6
ACCE N3
ACCE N7
ACCE N1
IC C8   C4   *N9   CM        1.3830  106.03  179.68  126.91  1.4740
IC C4   N9   C8    N7        1.3872  106.03   -0.02  114.22  1.3274
IC N7   N9   *C8   H8        1.3274  114.22 -179.92  121.60  1.0909
IC N9   C8   N7    C5        1.3830  114.22   -0.01  103.14  1.4083
IC C4   N7   *C5   C6        1.3981  111.07 -179.82  133.20  1.4122
IC N7   C5   C6    N1        1.4083  133.20  179.41  119.03  1.3585
IC N1   C5   *C6   N6        1.3585  119.03  179.98  124.43  1.3454
IC C5   C6   N6    H61       1.4122  124.43  179.81  116.82  0.9951
IC H61  C6   *N6   H62       0.9951  116.82  179.99  121.44  0.9958
IC C5   C6   N1    C2        1.4122  119.03   -0.13  119.78  1.3525
IC C6   N1   C2    CM2       1.3585  119.78  179.60  117.31  1.4975
IC CM2  N1   *C2   N3        1.4975  117.31 -178.46  125.47  1.3491
IC N1   C2   CM2   H21       1.3525  117.31   29.02  110.78  1.1109
IC H21  C2   *CM2  H22       1.1109  110.78  119.97  110.76  1.1109
IC H21  C2   *CM2  H23       1.1109  110.78 -120.03  109.89  1.1093
IC C4   N9   CM    HM1       1.3872  126.91  130.43  111.22  1.1122
IC HM1  N9   *CM   HM2       1.1122  111.22  120.67  110.74  1.1120
IC HM1  N9   *CM   HM3       1.1122  111.22 -119.78  110.90  1.1138

RESI B8MA           0.00 ! 8-methyladenosine, yxu
GROUP
ATOM N9     NG2R51 -0.05 !      H61  H62
ATOM C8     CG2R53  0.40 !        \  /
ATOM N7     NG2R50 -0.71 !         N6
ATOM C5     CG2RC0  0.28 !         |
ATOM C6     CG2R64  0.46 !         C6
ATOM N6     NG2S3  -0.77 !        // \              H81
ATOM H61    HGP4    0.38 !       N1   C5--N7\\     /
ATOM H62    HGP4    0.38 !       |    ||     C8-C8M--H82
ATOM N1     NG2R62 -0.74 !       C2   C4--N9/      \
ATOM C2     CG2R64  0.50 !      / \\ /     \        H83
ATOM H2     HGR62   0.13 !    H2   N3       \
ATOM N3     NG2R62 -0.75 !                   \
ATOM C4     CG2RC0  0.43 !                    \
GROUP                    !                     \
ATOM C8M    CG331  -0.21
ATOM H81    HGA3    0.09
ATOM H82    HGA3    0.09
ATOM H83    HGA3    0.09
GROUP
ATOM CM     CG331  -0.27
ATOM HM1    HGA3    0.09
ATOM HM2    HGA3    0.09
ATOM HM3    HGA3    0.09
BOND CM   HM1    CM   HM2   CM   HM3
```

```
BOND N9   C8   N9   C4   C8   N7   C8   C8M
BOND N7   C5   C5   C6   C5   C4   C6   N6
BOND C6   N1   N6   H61  N6   H62  N1   C2
BOND C2   N3   C2   H2   N3   C4
BOND C8M  H81  C8M  H82  C8M  H83
BOND CM   N9
IMPR N6   H61  H62  C6        C6   C5   N1   N6
DONO H61  N6
DONO H62  N6
ACCE N3
ACCE N7
ACCE N1
IC C8   C4   *N9   CM        1.3882  105.63  179.76  125.48  1.4741
IC C4   N9   C8    N7        1.3813  105.63   -0.66  114.11  1.3216
IC N7   N9   *C8   C8M       1.3216  114.11 -179.49  122.51  1.4902
IC N9   C8   N7    C5        1.3882  114.11    0.45  103.52  1.3978
IC C4   N7   *C5   C6        1.3985  110.93 -179.98  132.59  1.4111
IC N7   C5   C6    N1        1.3978  132.59 -179.38  118.64  1.3594
IC N1   C5   *C6   N6        1.3594  118.64 -179.96  124.71  1.3439
IC C5   C6   N6    H61       1.4111  124.71 -179.44  117.11  0.9949
IC H61  C6   *N6   H62       0.9949  117.11  179.94  121.54  0.9962
IC C5   C6   N1    C2        1.4111  118.64   -0.29  119.96  1.3610
IC C6   N1   C2    N3        1.3594  119.96   -0.37  125.13  1.3577
IC N3   N1   *C2   H2        1.3577  125.13 -179.63  117.48  1.0936
IC N9   C8   C8M   H81       1.3882  122.51   65.53  109.76  1.1092
IC H81  C8   *C8M  H82       1.1092  109.76  119.74  109.88  1.1105
IC H81  C8   *C8M  H83       1.1092  109.76 -120.67  109.85  1.1094
IC C4   N9   CM    HM1       1.3813  125.48  -58.53  110.15  1.1117
IC HM1  N9   *CM   HM2       1.1117  110.15  118.01  109.61  1.1123
IC HM1  N9   *CM   HM3       1.1117  110.15 -121.35  113.44  1.1116

RESI BINO           0.00 ! inosine, yxu
GROUP
ATOM N9     NG2R51 -0.01 !         O6
ATOM C8     CG2R53  0.26 !         ||
ATOM H8     HGR52   0.15 !         C6
ATOM N7     NG2R50 -0.61 !        /  \
ATOM C5     CG2RC0  0.01 !    H1-N1   C5--N7\\
ATOM C6     CG2R63  0.55 !       |    ||      C8-H8
ATOM O6     OG2D4  -0.51 !    H2-C2   C4--N9/
ATOM N1     NG2R61 -0.32 !        \\ /      \
ATOM H1     HGP1    0.25 !         N3        \
ATOM C2     CG2R64  0.51 !                    \
ATOM H2     HGR62   0.10 !                     \
ATOM N3     NG2R62 -0.64 !                      \
ATOM C4     CG2RC0  0.26
GROUP
ATOM CM     CG331  -0.27
ATOM HM1    HGA3    0.09
ATOM HM2    HGA3    0.09
ATOM HM3    HGA3    0.09
BOND CM   HM1    CM   HM2   CM   HM3
BOND N9   C8   N9   C4   C8   N7   C8   H8
BOND N7   C5   C5   C6   C5   C4   C6   O6
BOND C6   N1   N1   C2   N1   H1   C2   N3
BOND C2   H2   N3   C4
BOND CM   N9
IMPR C6     C5     N1     O6
DONO H1   N1
ACCE O6   C6
ACCE N3
ACCE N7
IC C8   C4   *N9   CM        1.3788  106.03 -179.97  126.24  1.4709
```

```
IC C4   N9   C8    N7        1.3824  106.03    0.02  113.65  1.3247
IC N7   N9   *C8   H8        1.3247  113.65 -179.96  121.82  1.0904
IC N9   C8   N7    C5        1.3788  113.65   -0.02  104.35  1.3965
IC C4   N7   *C5   C6        1.5275  106.25 -178.78  133.65  1.4203
IC N7   C5   C6    N1        1.3948  133.65  177.82  111.47  1.3825
IC N1   C5   *C6   O6        1.3825  111.47 -179.78  130.75  1.2323
IC C5   C6   N1    C2        1.4203  111.47    0.78  128.71  1.3956
IC C2   C6   *N1   H1        1.3956  128.71  179.30  112.80  1.0062
IC C6   N1   C2    N3        1.3825  128.71   -0.11  121.17  1.3609
IC N3   N1   *C2   H2        1.3609  121.17  178.76  120.82  1.0917
IC C4   N9   CM    HM1       1.5115  167.35    8.10  121.25  1.1187
IC HM1  N9   *CM   HM2       1.1187  121.25  120.98  107.49  1.1133
IC HM1  N9   *CM   HM3       1.1187  121.25 -124.61  108.15  1.1132

RESI B1MI           0.00 ! 1-methylinosine, yxu
GROUP
ATOM N9     NG2R51  0.00 !           O6
ATOM C8     CG2R53  0.36 !           ||
ATOM H8     HGR52   0.10 !  H11      C6
ATOM N7     NG2R50 -0.70 !     \    /  \
ATOM C5     CG2RC0  0.08 ! H12-C1M-N1   C5--N7\\
ATOM C6     CG2R63  0.66 !     /   |    ||      C8-H8
ATOM O6     OG2D4  -0.53 !  H13    C2   C4--N9/
ATOM N1     NG2R61 -0.36 !        / \\ /     \
ATOM C2     CG2R64  0.68 !      H2   N3       \
ATOM H2     HGR62   0.05 !                     \
ATOM N3     NG2R62 -0.81 !                      \
ATOM C4     CG2RC0  0.34 !                       \
ATOM C1M    CG331  -0.14
ATOM H11    HGA3    0.09
ATOM H12    HGA3    0.09
ATOM H13    HGA3    0.09
GROUP
ATOM CM     CG331  -0.27
ATOM HM1    HGA3    0.09
ATOM HM2    HGA3    0.09
ATOM HM3    HGA3    0.09
BOND CM   HM1    CM   HM2   CM   HM3
BOND N9   C8   N9   C4   C8   N7   C8   H8
BOND N7   C5   C5   C6   C5   C4   C6   O6
BOND C6   N1   N1   C2   N1   C1M  C2   N3
BOND C2   H2   N3   C4   C1M  H11  C1M  H12
BOND C1M  H13
BOND CM   N9
IMPR C6     C5     N1     O6
ACCE O6   C6
ACCE N3
ACCE N7
IC C8   C4   *N9   CM        1.3788  106.03 -179.97  126.24  1.4709
IC C4   N9   C8    N7        1.3824  106.03    0.02  113.65  1.3247
IC N7   N9   *C8   H8        1.3247  113.65 -179.96  121.82  1.0904
IC N9   C8   N7    C5        1.3788  113.65   -0.02  104.35  1.3965
IC C4   N7   *C5   C6        1.4052  110.00  179.93  131.27  1.4191
IC N7   C5   C6    N1        1.3965  131.27  179.94  113.61  1.4112
IC N1   C5   *C6   O6        1.4112  113.61 -179.85  127.97  1.2357
IC C5   C6   N1    C2        1.4191  113.61    0.27  124.46  1.3974
IC C2   C6   *N1   C1M       1.3974  124.46  179.65  117.44  1.4797
IC C6   N1   C2    N3        1.4112  124.46   -0.29  120.73  1.3557
IC N3   N1   *C2   H2        1.3557  120.73 -179.79  120.48  1.0911
IC C6   N1   C1M   H11       1.4112  117.44  -60.69  110.94  1.1167
IC H11  N1   *C1M  H12       1.1167  110.94  118.33  110.99  1.1162
IC H11  N1   *C1M  H13       1.1167  110.94 -120.89  113.89  1.1140
IC C4   N9   CM    HM1       1.3824  126.24  -59.00  110.07  1.1123
```

```
IC HM1  N9   *CM   HM2       1.1123  110.07  118.52  110.02  1.1128
IC HM1  N9   *CM   HM3       1.1123  110.07 -120.77  112.33  1.1113

RESI BSMA           0.00 ! 2-methylthio-N6-methyladenosine, yxu
GROUP
ATOM N9     NG2R51 -0.02 !        H11
ATOM C8     CG2R53  0.37 !            \
ATOM H8     HGR52   0.10 !      H12--C10   H6
ATOM N7     NG2R50 -0.84 !           / \  /
ATOM C5     CG2RC0  0.28 !        H13   N6
ATOM C6     CG2R64  0.38 !              |
ATOM N6     NG311  -0.48 !              C6
ATOM H6     HGPAM1  0.36 !             // \
ATOM N1     NG2R62 -0.58 !    H21     N1   C5--N7\\
ATOM C2     CG2R64  0.63 !      \     |    ||     C8-H8
ATOM N3     NG2R62 -0.76 ! H22 -C20   C2   C4--N9/
ATOM C4     CG2RC0  0.48 !      / \  / \\ /      \
ATOM C10    CG331  -0.09 !   H23   S2   N3        \
ATOM H11    HGA3    0.09 !                         \
ATOM H12    HGA3    0.09 !                          \
ATOM H13    HGA3    0.09
ATOM S2     SG311  -0.28
ATOM C20    CG331  -0.09
ATOM H21    HGA3    0.09
ATOM H22    HGA3    0.09
ATOM H23    HGA3    0.09
GROUP
ATOM CM     CG331  -0.27
ATOM HM1    HGA3    0.09
ATOM HM2    HGA3    0.09
ATOM HM3    HGA3    0.09
BOND CM   HM1    CM   HM2   CM   HM3
BOND N9   C8   N9   C4   C8   N7   C8   H8
BOND N7   C5   C5   C6   C5   C4   C6   N6
BOND C6   N1   N6   C10  N6   H6   N1   C2
BOND C2   N3   C2   S2   N3   C4   C10  H11
BOND C10  H12  C10  H13  S2   C20  C20  H21
BOND C20  H22  C20  H23
BOND CM   N9
IMPR C6   C5   N1   N6     ! N6   C6   C10   H6
DONO H6   N6
ACCE N3
ACCE N7
ACCE N1
IC C8   C4   *N9   CM        1.3846  105.95 -179.78  126.32  1.4722
IC C4   N9   C8    N7        1.3825  105.95    0.06  114.09  1.3283
IC N7   N9   *C8   H8        1.3283  114.09  179.95  121.43  1.0916
IC N9   C8   N7    C5        1.3846  114.09    0.10  103.30  1.3994
IC C4   N7   *C5   C6        1.4020  111.07 -179.79  131.87  1.4048
IC N7   C5   C6    N1        1.3994  131.87  179.46  117.93  1.3718
IC N1   C5   *C6   N6        1.3718  117.93 -179.71  120.87  1.3751
IC C5   C6   N6    C10       1.4048  120.87  166.46  125.65  1.4787
IC C10  C6   *N6   H6        1.4787  125.65 -160.60  117.33  1.0208
IC C5   C6   N1    C2        1.4048  117.93    0.48  119.95  1.3631
IC C6   N1   C2    S2        1.3718  119.95  179.76  120.09  1.7501
IC S2   N1   *C2   N3        1.7501  120.09  179.81  125.40  1.3550
IC C6   N6   C10   H11       1.3751  125.65  157.43  110.59  1.1120
IC H11  N6   *C10  H12       1.1120  110.59  120.20  110.71  1.1130
IC H11  N6   *C10  H13       1.1120  110.59 -119.67  111.42  1.1125
IC N1   C2   S2    C20       1.3631  120.09   -1.22   98.98  1.8306
IC C2   S2   C20   H21       1.7501   98.98 -179.58  110.43  1.1112
IC H21  S2   *C20  H22       1.1112  110.43  119.17  111.64  1.1125
IC H21  S2   *C20  H23       1.1112  110.43 -119.24  111.69  1.1113
```

```
IC C4   N9   CM    HM1      1.3825  126.32   60.84  110.21  1.1127
IC HM1  N9   *CM   HM2      1.1127  110.21  120.88  112.38  1.1113
IC HM1  N9   *CM   HM3      1.1127  110.21 -118.46  110.05  1.1133

RESI B6AA          0.00 ! N6-acetyladenosine, yxu
GROUP
ATOM N9     NG2R51 -0.01 !   H11    O10
ATOM C8     CG2R53  0.39 !      \   ||
ATOM H8     HGR52   0.09 ! H12--C11-C10   H6
ATOM N7     NG2R50 -0.81 !      /     \  /
ATOM C5     CG2RC0  0.35 !   H13       N6
ATOM C6     CG2R64  0.70 !             |
ATOM N6     NG2S1  -0.68 !             C6
ATOM H6     HGP1    0.32 !            // \
ATOM N1     NG2R62 -0.68 !           N1   C5--N7\\
ATOM C2     CG2R64  0.39 !           |    ||     C8-H8
ATOM H2     HGR62   0.14 !           C2   C4--N9/
ATOM N3     NG2R62 -0.82 !          / \\ /     \
ATOM C4     CG2RC0  0.58 !        H2   N3       \
ATOM C10    CG2O1   0.47 !                       \
ATOM O10    OG2D1  -0.44 !                        \
ATOM C11    CG331  -0.26 !                         \
ATOM H11    HGA3    0.09
ATOM H12    HGA3    0.09
ATOM H13    HGA3    0.09
GROUP
ATOM CM     CG331  -0.27
ATOM HM1    HGA3    0.09
ATOM HM2    HGA3    0.09
ATOM HM3    HGA3    0.09
BOND CM   HM1    CM   HM2   CM   HM3
BOND N9   C8   N9   C4   C8   N7   C8   H8
BOND N7   C5   C5   C6   C5   C4   C6   N6
BOND C6   N1   N6   C10  N6   H6   N1   C2
BOND C2   N3   C2   H2   N3   C4   C10  C11
BOND C10  O10  C11  H11  C11  H12  C11  H13
BOND CM   N9
IMPR C10    C11    N6     O10   C6   C5   N1   N6
DONO H6   N6
ACCE N3
ACCE N7
ACCE N1
ACCE O10  C10
IC C8   C4   *N9   CM       1.3827  106.04  179.95  126.40  1.4736
IC C4   N9   C8    N7       1.3866  106.04    0.02  113.99  1.3275
IC N7   N9   *C8   H8       1.3275  113.99  179.99  121.59  1.0928
IC N9   C8   N7    C5       1.3827  113.99   -0.06  103.32  1.4070
IC C4   N7   *C5   C6       1.3947  110.98  179.91  132.63  1.3997
IC N7   C5   C6    N6       1.4070  132.63    0.06  115.54  1.3985
IC N6   C5   *C6   N1       1.3985  115.54  179.99  118.30  1.3711
IC C5   C6   N6    C10      1.3997  115.54  179.82  132.54  1.3340
IC C10  C6   *N6   H6       1.3340  132.54 -180.00  112.06  0.9936
IC C5   C6   N1    C2       1.3997  118.30    0.00  120.57  1.3554
IC C6   N1   C2    N3       1.3711  120.57    0.04  124.26  1.3534
IC N3   N1   *C2   H2       1.3534  124.26 -179.98  117.78  1.0964
IC C6   N6   C10   O10      1.3985  132.54  179.99  118.50  1.2200
IC O10  N6   *C10  C11      1.2200  118.50 -180.00  121.57  1.4837
IC N6   C10  C11   H11      1.3340  121.57  -60.36  110.28  1.1096
IC H11  C10  *C11  H12      1.1096  110.28 -119.41  109.33  1.1107
IC H11  C10  *C11  H13      1.1096  110.28  121.04  110.30  1.1098
IC C4   N9   CM    HM1      1.3866  126.40  -59.84  110.31  1.1124
IC HM1  N9   *CM   HM2      1.1124  110.31  118.57  110.26  1.1134
IC HM1  N9   *CM   HM3      1.1124  110.31 -120.77  112.44  1.1119
```

```
RESI B1MA           1.00 ! protonated 1-methyladenosine yxu
GROUP
ATOM N9     NG2R51  0.00 !        H61  H62
ATOM C8     CG2R53  0.35 !          \  /
ATOM H8     HGR52   0.14 !           N6(+)
ATOM N7     NG2R50 -0.66 !           ||
ATOM C5     CG2RC0  0.10 !  H11      C6
ATOM C6     CG2R64  0.62 !     \    /  \
ATOM N6     NG2P1  -0.81 ! H12-CM1-N1   C5--N7\\
ATOM H61    HGP2    0.40 !     /   |    ||     C8-H8
ATOM H62    HGP2    0.40 !  H13    C2   C4--N9/
ATOM N1     NG2P1  -0.14 !        / \\ /     \
ATOM C2     CG2R64  0.28 !      H2   N3       \
ATOM H2     HGR62   0.17 !                     \
ATOM N3     NG2R62 -0.60 !                      \
ATOM C4     CG2RC0  0.55
ATOM CM1    CG334  -0.07
ATOM H11    HGA3    0.09
ATOM H12    HGA3    0.09
ATOM H13    HGA3    0.09
GROUP
ATOM CM     CG331  -0.27
ATOM HM1    HGA3    0.09
ATOM HM2    HGA3    0.09
ATOM HM3    HGA3    0.09
BOND CM   HM1    CM   HM2   CM   HM3
BOND N9   C8   N9   C4   C8   H8   C8   N7
BOND N7   C5   C5   C6   C5   C4   C6   N6
BOND C6   N1   N6   H61  N6   H62  N1   C2
BOND N1   CM1  C2   H2   C2   N3   N3   C4
BOND CM1  H11  CM1  H12  CM1  H13
BOND CM   N9
IMPR C6   C5   N1   N6     N6    H62    H61    C6
DONO H61  N6
DONO H62  N6
ACCE N3
ACCE N7
IC C8   C4   *N9   CM       1.3823  106.10 -179.41  126.58  1.4717
IC C4   N9   C8    N7       1.3868  106.10    0.45  113.45  1.3314
IC N7   N9   *C8   H8       1.3314  113.45 -179.98  121.65  1.0942
IC N9   C8   N7    C5       1.3823  113.45   -0.24  104.22  1.3906
IC C4   N7   *C5   C6       1.4058  110.62  179.89  131.83  1.3914
IC N7   C5   C6    N1       1.3906  131.83  179.23  117.59  1.4098
IC N1   C5   *C6   N6       1.4098  117.59  179.27  122.55  1.3588
IC C5   C6   N6    H61      1.3914  122.55 -176.76  122.98  0.9974
IC H61  C6   *N6   H62      0.9974  122.98  177.09  117.24  1.0034
IC C5   C6   N1    C2       1.3914  117.59    1.00  121.48  1.4178
IC C2   C6   *N1   CM1      1.4178  121.48 -179.43  120.65  1.4800
IC C6   N1   C2    N3       1.4098  121.48   -0.35  120.61  1.3649
IC N3   N1   *C2   H2       1.3649  120.61  179.38  117.95  1.0961
IC C6   N1   CM1   H11      1.4098  120.65   53.38  111.40  1.1140
IC H11  N1   *CM1  H12      1.1140  111.40  119.44  112.67  1.1146
IC H11  N1   *CM1  H13      1.1140  111.40 -120.66  111.10  1.1145
IC C4   N9   CM    HM1      1.3868  126.58   70.37  110.51  1.1130
IC HM1  N9   *CM   HM2      1.1130  110.51  120.38  112.33  1.1134
IC HM1  N9   *CM   HM3      1.1130  110.51 -119.34  110.78  1.1133

RESI B6IA           0.00 ! N6-isopentenyladenosine  yxu
GROUP
ATOM N9     NG2R51 -0.01 !    H153      H161
ATOM C8     CG2R53  0.43 !      \      /
ATOM H8     HGR52   0.08 !  H152-C15  C16-H162
```

```
ATOM N7     NG2R50 -0.85 !       / \ / \
ATOM C5     CG2RC0  0.32 !    H151  C14 H163
ATOM C6     CG2R64  0.45 !         //
ATOM N6     NG311  -0.45 !    H13-C13
ATOM H6     HGPAM1  0.33 !          \
ATOM N1     NG2R62 -0.78 !      H121-C12   H6
ATOM C2     CG2R64  0.49 !           / \  /
ATOM H2     HGR62   0.14 !         H122 N6
ATOM N3     NG2R62 -0.86 !              |
ATOM C4     CG2RC0  0.52 !              C6
ATOM C12    CG321   0.01 !             // \
ATOM H121   HGA2    0.09 !            N1   C5--N7\\
ATOM H122   HGA2    0.09 !            |    ||     C8-H8
GROUP                    !            C2   C4--N9/
ATOM C13    CG2D1  -0.15 !           / \\ /      \
ATOM H13    HGA4    0.15 !         H2   N3        \
GROUP                    !                         \
ATOM C14    CG2D1   0.00 !                          \
ATOM C15    CG331  -0.27 !                           \
ATOM H151   HGA3    0.09
ATOM H152   HGA3    0.09
ATOM H153   HGA3    0.09
GROUP
ATOM C16    CG331  -0.27
ATOM H161   HGA3    0.09
ATOM H162   HGA3    0.09
ATOM H163   HGA3    0.09
GROUP
ATOM CM     CG331  -0.27
ATOM HM1    HGA3    0.09
ATOM HM2    HGA3    0.09
ATOM HM3    HGA3    0.09
BOND CM   HM1    CM   HM2   CM   HM3
BOND N9   C8     N9   C4      C8   N7      C8   H8
BOND N7   C5     C5   C6      C5   C4      C6   N6
BOND C6   N1     N6   C12     N6   H6      N1   C2
BOND C2   N3     C2   H2      N3   C4      C12  C13
BOND C12  H121   C12  H122    C13  C14     C13  H13
BOND C14  C15    C14  C16     C15  H151    C15  H152
BOND C15  H153   C16  H161    C16  H162    C16  H163
BOND CM   N9
IMPR C6   C5   N1   N6    ! N6   C6   C12   H6
DONO H6   N6
ACCE N3
ACCE N7
ACCE N1
IC C8   C4   *N9   CM       1.3835  105.90 -179.77  126.22  1.4740
IC C4   N9   C8    N7       1.3838  105.90   -0.05  114.35  1.3293
IC N7   N9   *C8   H8       1.3293  114.35  179.83  121.44  1.0921
IC N9   C8   N7    C5       1.3835  114.35    0.12  102.92  1.4040
IC C4   N7   *C5   C6       1.3994  111.25 -179.86  132.33  1.4065
IC N7   C5   C6    N6       1.4040  132.33    1.45  121.06  1.3737
IC N6   C5   *C6   N1       1.3737  121.06  178.63  118.05  1.3734
IC C5   C6   N6    C12      1.4065  121.06 -179.40  124.85  1.4866
IC C12  C6   *N6   H6       1.4866  124.85 -176.92  115.64  1.0172
IC C5   C6   N1    C2       1.4065  118.05   -0.29  120.56  1.3593
IC C6   N1   C2    N3       1.3734  120.56   -0.11  124.63  1.3556
IC N3   N1   *C2   H2       1.3556  124.63 -179.66  117.81  1.0941
IC C6   N6   C12   C13      1.3737  124.85  150.19  109.28  1.5086
IC C13  N6   *C12  H121     1.5086  109.28  121.77  109.00  1.1143
IC C13  N6   *C12  H122     1.5086  109.28 -120.93  109.96  1.1147
IC N6   C12  C13   C14      1.4866  109.28 -177.21  125.26  1.3463
IC C14  C12  *C13  H13      1.3463  125.26 -176.65  116.57  1.0996
```

```
IC C12  C13  C14   C15       1.5086  125.26 -178.91  121.75  1.5043
IC C15  C13  *C14  C16       1.5043  121.75  179.46  123.38  1.5047
IC C13  C14  C15   H151      1.3463  121.75  117.56  110.41  1.1118
IC H151 C14  *C15  H152      1.1118  110.41  118.78  110.61  1.1112
IC H151 C14  *C15  H153      1.1118  110.41 -120.51  113.20  1.1124
IC C13  C14  C16   H161      1.3463  123.38 -118.00  110.68  1.1106
IC H161 C14  *C16  H162      1.1106  110.68  120.63  113.66  1.1114
IC H161 C14  *C16  H163      1.1106  110.68 -119.07  110.38  1.1112
IC C4   N9   CM    HM1       1.3838  126.22   58.11  109.99  1.1129
IC HM1  N9   *CM   HM2       1.1129  109.99  120.93  112.47  1.1126
IC HM1  N9   *CM   HM3       1.1129  109.99 -117.85  110.20  1.1126

RESI BHIA           0.00 ! N6-(cis-hydroxyisopentenyl)adenosine, yxu
GROUP
ATOM N9     NG2R51 -0.01 !    H153      H161
ATOM C8     CG2R53  0.43 !       \      /
ATOM H8     HGR52   0.08 !  H152-C15  C16-H162
ATOM N7     NG2R50 -0.85 !       /  \ / \
ATOM C5     CG2RC0  0.32 !     H151 C14 O16-H16O
ATOM C6     CG2R64  0.45 !         //
ATOM N6     NG311  -0.45 !    H13-C13
ATOM H6     HGPAM1  0.33 !          \
ATOM N1     NG2R62 -0.78 !      H121-C12  H6
ATOM C2     CG2R64  0.49 !           / \  /
ATOM H2     HGR62   0.14 !         H122 N6
ATOM N3     NG2R62 -0.86 !              |
ATOM C4     CG2RC0  0.52 !              C6
ATOM C12    CG321   0.01 !             // \
ATOM H121   HGA2    0.09 !            N1   C5--N7\\
ATOM H122   HGA2    0.09 !            |    ||     C8-H8
GROUP                    !            C2   C4--N9/
ATOM C13    CG2D1  -0.15 !           / \\ /      \
ATOM H13    HGA4    0.15 !         H2   N3        \
GROUP                    !                         \
ATOM C14    CG2D1   0.00 !                          \
ATOM C15    CG331  -0.27 !                           \
ATOM H151   HGA3    0.09
ATOM H152   HGA3    0.09
ATOM H153   HGA3    0.09
GROUP
ATOM C16    CG321   0.05
ATOM H161   HGA2    0.09
ATOM H162   HGA2    0.09
ATOM O16    OG311  -0.65
ATOM H16O   HGP1    0.42
GROUP
ATOM CM     CG331  -0.27
ATOM HM1    HGA3    0.09
ATOM HM2    HGA3    0.09
ATOM HM3    HGA3    0.09
BOND CM   HM1    CM   HM2   CM   HM3
BOND N9   C8     N9   C4      C8   N7      C8   H8
BOND N7   C5     C5   C6      C5   C4      C6   N6
BOND C6   N1     N6   C12     N6   H6      N1   C2
BOND C2   N3     C2   H2      N3   C4      C12  C13
BOND C12  H121   C12  H122    C13  C14     C13  H13
BOND C14  C15    C14  C16     C15  H151    C15  H152
BOND C15  H153   C16  O16     C16  H161    C16  H162
BOND O16  H16O
BOND CM   N9
IMPR C6   C5   N1   N6     ! N6   C6   C12   H6
DONO H6   N6
DONO H16O O16
```

```
ACCE N3
ACCE N7
ACCE N1
ACCE O16
IC C8   C4   *N9   CM        1.3840  106.02 -179.88  126.41  1.4736
IC C4   N9   C8    N7        1.3833  106.02   -0.13  114.25  1.3283
IC N7   N9   *C8   H8        1.3283  114.25  179.92  121.37  1.0908
IC N9   C8   N7    C5        1.3840  114.25   -0.03  103.10  1.4031
IC C4   N7   *C5   C6        1.4028  111.15 -179.83  132.43  1.4042
IC N7   C5   C6    N6        1.4031  132.43    2.55  120.79  1.3768
IC N6   C5   *C6   N1        1.3768  120.79  178.52  118.23  1.3721
IC C5   C6   N6    C12       1.4042  120.79  179.81  125.57  1.4873
IC C12  C6   *N6   H6        1.4873  125.57 -177.34  115.15  1.0188
IC C5   C6   N1    C2        1.4042  118.23   -0.96  120.46  1.3608
IC C6   N1   C2    N3        1.3721  120.46   -0.17  124.63  1.3552
IC N3   N1   *C2   H2        1.3552  124.63 -179.52  117.41  1.0945
IC C6   N6   C12   C13       1.3768  125.57  135.90  109.48  1.5086
IC C13  N6   *C12  H121      1.5086  109.48  120.88  108.68  1.1141
IC C13  N6   *C12  H122      1.5086  109.48 -121.67  110.71  1.1129
IC N6   C12  C13   C14       1.4873  109.48 -173.18  125.93  1.3502
IC C14  C12  *C13  H13       1.3502  125.93 -173.81  115.77  1.1006
IC C12  C13  C14   C16       1.5086  125.93 -178.84  120.57  1.5117
IC C16  C13  *C14  C15       1.5117  120.57  179.58  122.22  1.5106
IC C13  C14  C15   H151      1.3502  122.22 -121.08  109.83  1.1111
IC H151 C14  *C15  H152      1.1111  109.83  120.61  114.56  1.1111
IC H151 C14  *C15  H153      1.1111  109.83 -118.26  110.60  1.1118
IC C13  C14  C16   O16       1.3502  120.57  105.81  110.10  1.4280
IC O16  C14  *C16  H161      1.4280  110.10  119.76  109.43  1.1123
IC O16  C14  *C16  H162      1.4280  110.10 -120.90  112.38  1.1144
IC C14  C16  O16   H16O      1.5117  110.10  168.79  107.73  0.9614
IC C4   N9   CM    HM1       1.3833  126.41   65.02  110.08  1.1127
IC HM1  N9   *CM   HM2       1.1127  110.08  120.70  112.44  1.1131
IC HM1  N9   *CM   HM3       1.1127  110.08 -118.50  110.16  1.1126

RESI BMIA           0.00 ! 2-methylthio-N6-isopentenyladenosine, SPA  yxu
GROUP
ATOM N9     NG2R51 -0.01 !    H151       H161
ATOM C8     CG2R53  0.37 !      \        /
ATOM H8     HGR52   0.10 !  H152-C15   C16-H162
ATOM N7     NG2R50 -0.84 !       / \   / \
ATOM C5     CG2RC0  0.28 !     H153 C14  H163
ATOM C6     CG2R64  0.38 !          //
ATOM N6     NG311  -0.48 !    H130-C13
ATOM H6     HGPAM1  0.36 !           \
ATOM N1     NG2R62 -0.58 !       H101-C12  H6
ATOM C2     CG2R64  0.63 !            / \  /
ATOM N3     NG2R62 -0.76 !         H102  N6
ATOM C4     CG2RC0  0.48 !               |
ATOM C12    CG321  -0.00 !               C6
ATOM H101   HGA2    0.09 !              // \
ATOM H102   HGA2    0.09 !     H11     N1   C5--N7\\
ATOM S10    SG311  -0.28 !       \     |    ||      C8-H8
ATOM C11    CG331  -0.10 !  H12 -C11   C2   C4--N9/
ATOM H11    HGA3    0.09 !       / \  / \\ /      \
ATOM H12    HGA3    0.09 !    H13   S10  N3        \
ATOM H13    HGA3    0.09 !                          \
GROUP                    !                           \
ATOM C13    CG2D1  -0.15
ATOM H130   HGA4    0.15
GROUP
ATOM C14    CG2D1   0.00
ATOM C15    CG331  -0.27
ATOM H151   HGA3    0.09
```

```
ATOM H152   HGA3    0.09
ATOM H153   HGA3    0.09
GROUP
ATOM C16    CG331  -0.27
ATOM H161   HGA3    0.09
ATOM H162   HGA3    0.09
ATOM H163   HGA3    0.09
GROUP
ATOM CM     CG331  -0.27
ATOM HM1    HGA3    0.09
ATOM HM2    HGA3    0.09
ATOM HM3    HGA3    0.09
BOND CM   HM1    CM   HM2   CM   HM3
BOND N9   C8     N9   C4      C8   N7      C8   H8
BOND N7   C5     C5   C6      C5   C4      C6   N6
BOND C6   N1     N6   H6      N6   C12     N1   C2
BOND C2   N3     C2   S10     N3   C4      S10  C11
BOND C11  H11    C11  H12     C11  H13     C12  C13
BOND C12  H101   C12  H102    C13  C14     C13  H130
BOND C14  C15    C14  C16     C15  H151    C15  H152
BOND C15  H153   C16  H161    C16  H162    C16  H163
BOND CM   N9
IMPR C6   C5   N1   N6    ! N6   C6   C12   H6
DONO H6   N6
ACCE N3
ACCE N7
ACCE N1
IC C8   C4   *N9   CM        1.3833  105.92 -179.96  126.58  1.4727
IC C4   N9   C8    N7        1.3870  105.92    0.37  114.22  1.3283
IC N7   N9   *C8   H8        1.3283  114.22 -179.95  121.58  1.0924
IC N9   C8   N7    C5        1.3833  114.22   -0.04  103.26  1.3978
IC C4   N7   *C5   C6        1.4033  111.26  178.62  131.63  1.4036
IC N7   C5   C6    N1        1.3978  131.63  179.79  117.86  1.3705
IC N1   C5   *C6   N6        1.3705  117.86  179.40  120.27  1.3753
IC C5   C6   N6    C12       1.4036  120.27 -173.54  126.28  1.4889
IC C12  C6   *N6   H6        1.4889  126.28  175.58  114.41  1.0203
IC C5   C6   N1    C2        1.4036  117.86    0.92  120.09  1.3615
IC C6   N1   C2    S10       1.3705  120.09  179.82  119.66  1.7495
IC S10  N1   *C2   N3        1.7495  119.66 -179.50  125.34  1.3569
IC C6   N6   C12   C13       1.3753  126.28  120.78  108.58  1.5091
IC C13  N6   *C12  H101      1.5091  108.58  119.63  108.45  1.1131
IC C13  N6   *C12  H102      1.5091  108.58 -123.78  110.79  1.1107
IC N1   C2   S10   C11       1.3615  119.66    1.51   98.93  1.8314
IC C2   S10  C11   H11       1.7495   98.93 -178.40  110.36  1.1111
IC H11  S10  *C11  H12       1.1111  110.36  119.33  111.94  1.1125
IC H11  S10  *C11  H13       1.1111  110.36 -119.06  111.62  1.1116
IC N6   C12  C13   C14       1.4889  108.58 -140.03  128.50  1.3468
IC C14  C12  *C13  H130      1.3468  128.50 -177.95  114.45  1.0988
IC C12  C13  C14   C15       1.5091  128.50 -179.25  120.72  1.5045
IC C15  C13  *C14  C16       1.5045  120.72  178.85  125.37  1.5050
IC C13  C14  C15   H151      1.3468  120.72  120.37  110.67  1.1113
IC H151 C14  *C15  H152      1.1113  110.67  118.97  110.79  1.1113
IC H151 C14  *C15  H153      1.1113  110.67 -120.45  112.97  1.1122
IC C13  C14  C16   H161      1.3468  125.37 -119.86  110.22  1.1110
IC H161 C14  *C16  H162      1.1110  110.22  121.01  114.23  1.1095
IC H161 C14  *C16  H163      1.1110  110.22 -118.29  109.68  1.1108
IC C4   N9   CM    HM1       1.3870  126.58  -56.07  110.26  1.1129
IC HM1  N9   *CM   HM2       1.1129  110.26  119.07  110.70  1.1125
IC HM1  N9   *CM   HM3       1.1129  110.26 -120.28  112.09  1.1123

RESI BSIA           0.00 ! 2-methylthio-N6-(cis-hydroxyisopentenyl) adenosine  yxu
GROUP
ATOM N9     NG2R51 -0.01 !     H153      H161
```

```
ATOM C8     CG2R53  0.37 !       \       /
ATOM H8     HGR52   0.10 !   H152-C15  C16-H162
ATOM N7     NG2R50 -0.84 !        / \  / \
ATOM C5     CG2RC0  0.28 !      H151 C14  O16-H16O
ATOM C6     CG2R64  0.38 !           //
ATOM N6     NG311  -0.48 !     H130-C13
ATOM H6     HGPAM1  0.36 !            \
ATOM N1     NG2R62 -0.58 !        H121-C12  H6
ATOM C2     CG2R64  0.63 !             / \  /
ATOM N3     NG2R62 -0.76 !           H122 N6
ATOM C4     CG2RC0  0.48 !                |
ATOM C12    CG321  -0.00 !                C6
ATOM H121   HGA2    0.09 !               // \
ATOM H122   HGA2    0.09 !     H11      N1   C5--N7\\
ATOM S2     SG311  -0.28 !       \      |    ||     C8-H8
ATOM C11    CG331  -0.10 !   H12 -C11   C2   C4--N9/
ATOM H11    HGA3    0.09 !        / \  / \\ /      \
ATOM H12    HGA3    0.09 !      H13  S2   N3        \
ATOM H13    HGA3    0.09 !                           \
GROUP                    !                            \
ATOM C13    CG2D1  -0.15 !                             \
ATOM H130   HGA4    0.15
GROUP
ATOM C14    CG2D1   0.00
ATOM C15    CG331  -0.27
ATOM H151   HGA3    0.09
ATOM H152   HGA3    0.09
ATOM H153   HGA3    0.09
GROUP
ATOM C16    CG321   0.05
ATOM H161   HGA2    0.09
ATOM H162   HGA2    0.09
ATOM O16    OG311  -0.65
ATOM H16O   HGP1    0.42
GROUP
ATOM CM     CG331  -0.27
ATOM HM1    HGA3    0.09
ATOM HM2    HGA3    0.09
ATOM HM3    HGA3    0.09
BOND CM   HM1    CM   HM2   CM   HM3
BOND N9   C8     N9   C4      C8   N7      C8   H8
BOND N7   C5     C5   C6      C5   C4      C6   N6
BOND C6   N1     N6   C12     N6   H6      N1   C2
BOND C2   N3     C2   S2      N3   C4      C12  H121
BOND C12  C13    C12  H122    C13  H130    C13  C14
BOND C14  C15    C14  C16     C15  H151    C15  H152
BOND C15  H153   C16  O16     C16  H162    C16  H161
BOND O16  H16O   S2   C11     C11  H13     C11  H12
BOND C11  H11
BOND CM   N9
IMPR C6   C5   N1   N6     ! N6   C6   C12   H6
DONO H6   N6
DONO H16O O16
ACCE N3
ACCE N7
ACCE N1
ACCE O16
IC C8   C4   *N9   CM        1.3846  105.86  179.50  126.78  1.4703
IC C4   N9   C8    N7        1.3849  105.86    0.11  114.17  1.3274
IC N7   N9   *C8   H8        1.3274  114.17 -180.00  121.29  1.0922
IC N9   C8   N7    C5        1.3846  114.17   -0.04  103.30  1.3984
IC C4   N7   *C5   C6        1.4015  111.16  179.65  131.61  1.4035
IC N7   C5   C6    N6        1.3984  131.61    0.23  119.89  1.3770
```

```
IC N6   C5   *C6   N1        1.3770  119.89 -179.57  117.84  1.3705
IC C5   C6   N6    C12       1.4035  119.89 -176.64  126.86  1.4880
IC C12  C6   *N6   H6        1.4880  126.86  175.50  114.15  1.0202
IC C5   C6   N1    C2        1.4035  117.84   -0.60  119.97  1.3627
IC C6   N1   C2    S2        1.3705  119.97 -179.84  119.85  1.7502
IC S2   N1   *C2   N3        1.7502  119.85 -179.66  125.46  1.3553
IC C6   N6   C12   C13       1.3770  126.86  108.43  108.59  1.5101
IC C13  N6   *C12  H121      1.5101  108.59  119.44  108.35  1.1137
IC H121 N6   *C12  H122      1.1137  108.35  116.31  110.44  1.1116
IC N1   C2   S2    C11       1.3627  119.85   -2.21   98.85  1.8310
IC C2   S2   C11   H13       1.7502   98.85 -178.05  110.44  1.1119
IC H13  S2   *C11  H12       1.1119  110.44  119.06  111.63  1.1119
IC H13  S2   *C11  H11       1.1119  110.44 -119.41  111.76  1.1117
IC N6   C12  C13   C14       1.4880  108.59 -145.90  128.36  1.3524
IC C14  C12  *C13  H130      1.3524  128.36 -173.81  113.95  1.0994
IC C12  C13  C14   C16       1.5101  128.36  179.30  119.86  1.5148
IC C16  C13  *C14  C15       1.5148  119.86  178.68  122.65  1.5112
IC C13  C14  C15   H151      1.3524  122.65 -123.08  109.62  1.1118
IC H151 C14  *C15  H152      1.1118  109.62  119.85  114.47  1.1100
IC H151 C14  *C15  H153      1.1118  109.62 -118.25  110.84  1.1104
IC C13  C14  C16   O16       1.3524  119.86  114.21  110.17  1.4271
IC O16  C14  *C16  H162      1.4271  110.17  119.84  109.58  1.1140
IC O16  C14  *C16  H161      1.4271  110.17 -120.91  112.63  1.1158
IC C14  C16  O16   H16O      1.5148  110.17  175.43  108.12  0.9621
IC C4   N9   CM    HM1       1.3849  126.78   53.61  110.47  1.1116
IC HM1  N9   *CM   HM2       1.1116  110.47  120.63  112.18  1.1122
IC HM1  N9   *CM   HM3       1.1116  110.47 -118.92  110.22  1.1121

RESI BHNA           -1.00 ! N6-hydroxynorvalylcarbamoyladenosine   yxu
GROUP
ATOM N9     NG2R51 -0.03 !                   H161 H162
ATOM C8     CG2R53  0.34 !                     |  /
ATOM H8     HGR52   0.12 !              H151 C16-H163
ATOM N7     NG2R50 -0.75 !                  \ /
ATOM C5     CG2RC0  0.28 !             H152-C15   O14-H14O
ATOM C6     CG2R64  0.70 !                      \  /
ATOM N6     NG2S1  -0.58 !           O10      C14-H14
ATOM H6     HGP1    0.33 !           ||       /
ATOM N1     NG2R62 -0.74 !       H6   C10  C12-H12
ATOM C2     CG2R64  0.49 !        \  /  \  /  \
ATOM H2     HGR62   0.12 !         N6    N11   C13-ODA (-)
ATOM N3     NG2R62 -0.78 !         |      |     ||
ATOM C4     CG2RC0  0.41 !         |     H11   ODB
ATOM C10    CG2O6   0.60 !         C6
ATOM O10    OG2D1  -0.50 !        // \
ATOM N11    NG2S1  -0.48 !       N1   C5--N7\\
ATOM H11    HGP1    0.29 !       |    ||     C8-H8
ATOM C12    CG311   0.09 !       C2   C4--N9/
ATOM H12    HGA1    0.09 !      / \\ /      \
GROUP                    !    H2   N3        \
ATOM C13    CG2O3   0.52 !                    \
ATOM ODA    OG2D2  -0.76 !                     \
ATOM ODB    OG2D2  -0.76 !                      \
GROUP
ATOM C14    CG311   0.14
ATOM H14    HGA1    0.09
ATOM O14    OG311  -0.65
ATOM H14O   HGP1    0.42
ATOM C15    CG321  -0.18
ATOM H151   HGA2    0.09
ATOM H152   HGA2    0.09
ATOM C16    CG331  -0.27
ATOM H161   HGA3    0.09
```

```
ATOM H162   HGA3    0.09
ATOM H163   HGA3    0.09
GROUP
ATOM CM     CG331  -0.27
ATOM HM1    HGA3    0.09
ATOM HM2    HGA3    0.09
ATOM HM3    HGA3    0.09
BOND CM   HM1    CM   HM2   CM   HM3
BOND N9   C8     N9   C4      C8   N7      C8   H8
BOND N7   C5     C5   C6      C5   C4      C6   N6
BOND C6   N1     N6   C10     N6   H6      N1   C2
BOND C2   N3     C2   H2      N3   C4      C10  N11
BOND C10  O10    N11  C12     N11  H11     C12  C14
BOND C12  C13    C12  H12     C14  C15     C14  O14
BOND C14  H14    C15  C16     C15  H151    C15  H152
BOND C16  H161   C16  H162    C16  H163    C13  ODA
BOND C13  ODB    O14  H14O
BOND CM   N9
IMPR C10    N6     N11    O10    C13    ODB    ODA    C12     C6   C5   N1   N6
DONO H6   N6
DONO H11  N11
DONO H14O O14
ACCE N3
ACCE N7
ACCE N1
ACCE O10  C10
ACCE O14
ACCE ODA  C13
ACCE ODB  C13
IC C8   C4   *N9   CM        1.3819  106.06  179.90  126.27  1.4710
IC C4   N9   C8    N7        1.3842  106.06    0.17  113.86  1.3264
IC N7   N9   *C8   H8        1.3264  113.86  179.95  121.64  1.0918
IC N9   C8   N7    C5        1.3819  113.86   -0.35  103.64  1.4041
IC C4   N7   *C5   C6        1.3977  110.65  179.95  132.48  1.3996
IC N7   C5   C6    N6        1.4041  132.48    0.94  114.92  1.3884
IC N6   C5   *C6   N1        1.3884  114.92 -179.98  118.10  1.3707
IC C5   C6   N6    C10       1.3996  114.92  179.06  126.95  1.3521
IC C10  C6   *N6   H6        1.3521  126.95 -168.86  113.79  0.9877
IC C5   C6   N1    C2        1.3996  118.10   -1.31  120.37  1.3530
IC C6   N1   C2    N3        1.3707  120.37    1.22  124.77  1.3571
IC N3   N1   *C2   H2        1.3571  124.77  179.21  117.37  1.0937
IC C6   N6   C10   N11       1.3884  126.95 -172.26  108.07  1.3577
IC N11  N6   *C10  O10       1.3577  108.07  176.45  126.69  1.2214
IC N6   C10  N11   C12       1.3521  108.07  147.67  123.28  1.4563
IC C12  C10  *N11  H11       1.4563  123.28 -135.21  117.07  0.9965
IC C10  N11  C12   C14       1.3577  123.28  131.55  113.81  1.5304
IC C14  N11  *C12  C13       1.5304  113.81  116.82  105.55  1.5441
IC C14  N11  *C12  H12       1.5304  113.81 -125.18  111.57  1.1119
IC N11  C12  C13   ODA       1.4563  105.55  144.29  117.39  1.2566
IC ODA  C12  *C13  ODB       1.2566  117.39  179.78  114.93  1.2643
IC N11  C12  C14   C15       1.4563  113.81  178.58  113.56  1.5512
IC C15  C12  *C14  O14       1.5512  113.56  120.83  111.39  1.4242
IC C15  C12  *C14  H14       1.5512  113.56 -120.94  108.33  1.1156
IC C12  C14  O14   H14O      1.5304  111.39 -169.46  103.47  0.9572
IC C12  C14  C15   C16       1.5304  113.56  175.68  114.98  1.5399
IC C16  C14  *C15  H151      1.5399  114.98  120.91  108.23  1.1140
IC C16  C14  *C15  H152      1.5399  114.98 -123.60  108.54  1.1126
IC C14  C15  C16   H161      1.5512  114.98  173.23  109.77  1.1118
IC H161 C15  *C16  H162      1.1118  109.77  119.41  111.24  1.1093
IC H161 C15  *C16  H163      1.1118  109.77 -119.21  110.22  1.1110
IC C4   N9   CM    HM1       1.3842  126.27   59.71  109.97  1.1127
IC HM1  N9   *CM   HM2       1.1127  109.97  120.81  112.41  1.1111
IC HM1  N9   *CM   HM3       1.1127  109.97 -118.34  109.96  1.1124
```

```
RESI B26A          -1.00 ! 2-methylthio-N6-hydroxynorvalyl carbamoyladenosine   yxu
GROUP
ATOM N9     NG2R51 -0.01 !                         H161 H162
ATOM C8     CG2R53  0.37 !                          |  /
ATOM H8     HGR52   0.10 !                     H151 C16-H163
ATOM N7     NG2R50 -0.84 !                       \ /
ATOM C5     CG2RC0  0.28 !                  H152-C15   O14-H14O
ATOM C6     CG2R64  0.43 !                         \  /
ATOM N1     NG2R62 -0.58 !                 O10      C14-H14
ATOM C2     CG2R64  0.63 !                 ||      /
ATOM N3     NG2R62 -0.76 !            H6   C10  C12-H12
ATOM C4     CG2RC0  0.48 !             \  /  \  /  \
ATOM S2     SG311  -0.28 !              N6    N11   C13-ODA (-)
ATOM C2M    CG331  -0.09 !              |      |    ||
ATOM H21    HGA3    0.09 !              |     H11   ODB
ATOM H22    HGA3    0.09 !              C6
ATOM H23    HGA3    0.09 !             // \
ATOM N6     NG2S1  -0.43 !    H21     N1   C5--N7\\
ATOM H6     HGP1    0.32 !      \     |    ||     C8-H8
ATOM C10    CG2O6   0.60 !  H22 -C2M  C2   C4--N9/
ATOM O10    OG2D1  -0.50 !      / \  / \\ /      \
ATOM N11    NG2S1  -0.48 !    H23  S2   N3        \
ATOM H11    HGP1    0.31 !                         \
ATOM C12    CG311   0.09 !                          \
ATOM H12    HGA1    0.09 !                           \
GROUP
ATOM C13    CG2O3   0.52
ATOM ODA    OG2D2  -0.76
ATOM ODB    OG2D2  -0.76
GROUP
ATOM C14    CG311   0.14
ATOM H14    HGA1    0.09
ATOM O14    OG311  -0.65
ATOM H14O   HGP1    0.42
ATOM C15    CG321  -0.18
ATOM H151   HGA2    0.09 !  !!!! PATCH 6AH for the carboxylic acid form
ATOM H152   HGA2    0.09
ATOM C16    CG331  -0.27
ATOM H161   HGA3    0.09
ATOM H162   HGA3    0.09
ATOM H163   HGA3    0.09
GROUP
ATOM CM     CG331  -0.27
ATOM HM1    HGA3    0.09
ATOM HM2    HGA3    0.09
ATOM HM3    HGA3    0.09
BOND CM   HM1    CM   HM2   CM   HM3
BOND N9   C8     N9   C4      C8   N7      C8   H8
BOND N7   C5     C5   C6      C5   C4      C6   N6
BOND C6   N1     N6   C10     N6   H6      N1   C2
BOND C2   N3     C2   S2      N3   C4      C10  N11
BOND C10  O10    N11  C12     N11  H11     C12  C14
BOND C12  C13    C12  H12     C14  C15     C14  O14
BOND C14  H14    C15  C16     C15  H151    C15  H152
BOND C16  H161   C16  H162    C16  H163    C13  ODA
BOND C13  ODB    O14  H14O    S2   C2M     C2M  H21
BOND C2M  H22    C2M  H23
BOND CM   N9
IMPR C10    N6     N11    O10    C13    ODB    ODA    C12     C6   C5   N1   N6
DONO H6   N6
DONO H11  N11
DONO H14O O14
```

```
ACCE N3
ACCE N7
ACCE N1
ACCE O10  C10
ACCE O14
ACCE ODA  C13
ACCE ODB  C13
IC C8   C4   *N9   CM        1.3838  105.98  179.97  126.30  1.4719
IC C4   N9   C8    N7        1.3830  105.98    0.13  114.05  1.3277
IC N7   N9   *C8   H8        1.3277  114.05  179.75  121.57  1.0918
IC N9   C8   N7    C5        1.3838  114.05   -0.27  103.41  1.3975
IC C4   N7   *C5   C6        1.4028  111.05  179.95  131.81  1.3940
IC N7   C5   C6    N6        1.3975  131.81    1.79  115.31  1.3944
IC N6   C5   *C6   N1        1.3944  115.31  178.27  117.78  1.3690
IC C5   C6   N1    C2        1.3940  117.78   -0.34  120.44  1.3571
IC C6   N1   C2    S2        1.3690  120.44 -179.52  119.51  1.7505
IC S2   N1   *C2   N3        1.7505  119.51 -179.53  125.20  1.3565
IC N1   C2   S2    C2M       1.3571  119.51    3.15   98.46  1.8339
IC C2   S2   C2M   H21       1.7505   98.46  178.82  110.76  1.1111
IC H21  S2   *C2M  H22       1.1111  110.76  119.70  111.99  1.1123
IC H21  S2   *C2M  H23       1.1111  110.76 -119.37  111.90  1.1135
IC C5   C6   N6    C10       1.3940  115.31 -177.53  126.48  1.3587
IC C10  C6   *N6   H6        1.3587  126.48 -168.76  113.84  0.9890
IC C6   N6   C10   N11       1.3944  126.48 -161.67  109.34  1.3586
IC N11  N6   *C10  O10       1.3586  109.34  177.73  125.93  1.2241
IC N6   C10  N11   C12       1.3587  109.34  151.27  122.63  1.4559
IC C12  C10  *N11  H11       1.4559  122.63 -131.78  116.59  0.9997
IC C10  N11  C12   C14       1.3586  122.63  135.32  113.77  1.5285
IC C14  N11  *C12  C13       1.5285  113.77  116.83  105.22  1.5426
IC C14  N11  *C12  H12       1.5285  113.77 -125.16  111.73  1.1128
IC N11  C12  C13   ODA       1.4559  105.22  144.35  117.54  1.2556
IC ODA  C12  *C13  ODB       1.2556  117.54  179.85  114.59  1.2655
IC N11  C12  C14   C15       1.4559  113.77 -180.00  113.34  1.5496
IC C15  C12  *C14  O14       1.5496  113.34  120.54  111.21  1.4253
IC C15  C12  *C14  H14       1.5496  113.34 -121.12  108.51  1.1161
IC C12  C14  O14   H14O      1.5285  111.21 -169.34  103.51  0.9575
IC C12  C14  C15   C16       1.5285  113.34  177.64  114.88  1.5390
IC C16  C14  *C15  H151      1.5390  114.88  120.91  108.28  1.1146
IC C16  C14  *C15  H152      1.5390  114.88 -123.55  108.55  1.1120
IC C14  C15  C16   H161      1.5496  114.88  174.09  109.70  1.1115
IC H161 C15  *C16  H162      1.1115  109.70  119.59  111.37  1.1093
IC H161 C15  *C16  H163      1.1115  109.70 -118.96  110.24  1.1113
IC C4   N9   CM    HM1       1.3830  126.30   58.24  109.90  1.1120
IC HM1  N9   *CM   HM2       1.1120  109.90  120.71  112.31  1.1118
IC HM1  N9   *CM   HM3       1.1120  109.90 -118.42  110.04  1.1126

RESI B66A           -1.00 ! N6-methyl-N6-threonylcarbamoyladenosine, AET   yxu
GROUP
ATOM N9     NG2R51 -0.01 !                 H152 H153
ATOM C8     CG2R53  0.39 !                    \ /
ATOM H8     HGR52   0.09 !              H151-C15   O14-H14O
ATOM N7     NG2R50 -0.81 !                      \  /
ATOM C5     CG2RC0  0.35 !    H61      O10      C14-H14
ATOM C6     CG2R64  0.60 !       \      ||      /
ATOM N1     NG2R62 -0.68 !   H62-CM6   C10   C12-H12
ATOM C2     CG2R64  0.39 !       / \  /  \  /  \
ATOM H2     HGR62   0.14 !    H63   N6    N11   C13-ODA (-)
ATOM N3     NG2R62 -0.82 !          |       |    ||
ATOM C4     CG2RC0  0.57 !          |     H11   ODB
ATOM N6     NG2S0  -0.49 !          C6
ATOM CM6    CG331  -0.08 !         // \
ATOM H61     HGA3   0.09 !        N1   C5--N7\\
ATOM H62     HGA3   0.09 !        |    ||     C8-H8
```

```
ATOM H63     HGA3   0.09 !        C2   C4--N9/
ATOM C10    CG2O6   0.49 !       / \\ /      \
ATOM O10    OG2D1  -0.48 !     H2   N3        \
ATOM N11    NG2S1  -0.44 !                     \
ATOM H11     HGP1   0.30 !                      \
ATOM C12    CG311   0.13 !                       \
ATOM H12    HGA1    0.09
GROUP
ATOM C13    CG2O3   0.52
ATOM ODA    OG2D2  -0.76
ATOM ODB    OG2D2  -0.76
GROUP
ATOM C14    CG311   0.14
ATOM H14    HGA1    0.09
ATOM O14    OG311  -0.65
ATOM H14O   HGP1    0.42
ATOM C15    CG331  -0.27 !  !!!! PATCH 6AH for the carboxylic acid form
ATOM H151   HGA3    0.09
ATOM H152   HGA3    0.09
ATOM H153   HGA3    0.09
GROUP
ATOM CM     CG331  -0.27
ATOM HM1    HGA3    0.09
ATOM HM2    HGA3    0.09
ATOM HM3    HGA3    0.09
BOND CM   HM1    CM   HM2   CM   HM3
BOND N9   C8     N9   C4      C8   N7      C8   H8
BOND N7   C5     C5   C6      C5   C4      C6   N6
BOND C6   N1     N6   C10     N6   CM6     N1   C2
BOND C2   N3     C2   H2      N3   C4      C10  O10
BOND C10  N11    N11  C12     N11  H11     C12  C13
BOND C12  C14    C12  H12     C13  ODA     C13  ODB
BOND C14  O14    C14  C15     C14  H14     O14  H14O
BOND C15  H151   C15  H152    C15  H153    CM6  H61
BOND CM6  H62    CM6  H63
BOND CM   N9
IMPR C10    N6     N11    O10    C13    ODB    ODA    C12     C6   C5   N1   N6
DONO H11  N11
DONO H14O O14
ACCE N3
ACCE N7
ACCE N1
ACCE O10  C10
ACCE O14
ACCE ODA  C13
ACCE ODB  C13
IC C8   C4   *N9   CM        1.3760  106.03 -179.93  126.37  1.4731
IC C4   N9   C8    N7        1.3842  106.03    0.04  114.06  1.3226
IC N7   N9   *C8   H8        1.3226  114.06  179.87  121.53  1.0921
IC N9   C8   N7    C5        1.3760  114.06   -0.21  104.23  1.4146
IC C4   N7   *C5   C6        1.4062  109.29  180.00  133.54  1.4229
IC N7   C5   C6    N6        1.4146  133.54    1.28  126.39  1.4277
IC N6   C5   *C6   N1        1.4277  126.39  178.24  114.86  1.3870
IC C5   C6   N1    C2        1.4229  114.86    0.73  123.03  1.3498
IC C6   N1   C2    N3        1.3870  123.03   -0.22  124.41  1.3476
IC N3   N1   *C2   H2        1.3476  124.41  179.96  117.30  1.0961
IC C5   C6   N6    C10       1.4229  126.39 -179.15  121.99  1.3765
IC C10  C6   *N6   CM6       1.3765  121.99 -172.08  120.90  1.4710
IC C6   N6   CM6   H61       1.4277  120.90 -164.11  111.56  1.1151
IC H61  N6   *CM6  H62       1.1151  111.56  116.84  108.98  1.1094
IC H61  N6   *CM6  H63       1.1151  111.56 -122.23  108.26  1.1139
IC C6   N6   C10   N11       1.4277  121.99 -138.09  111.29  1.3622
IC N11  N6   *C10  O10       1.3622  111.29 -179.80  125.94  1.2261
```

```
IC N6   C10  N11   C12       1.3765  111.29  161.02  123.60  1.4535
IC C12  C10  *N11  H11       1.4535  123.60 -134.37  116.18  1.0018
IC C10  N11  C12   C14       1.3622  123.60  107.19  114.64  1.5331
IC C14  N11  *C12  C13       1.5331  114.64  118.14  106.26  1.5501
IC C13  N11  *C12  H12       1.5501  106.26  116.65  110.63  1.1111
IC N11  C12  C13   ODA       1.4535  106.26  178.70  116.17  1.2605
IC ODA  C12  *C13  ODB       1.2605  116.17  178.56  116.18  1.2637
IC N11  C12  C14   O14       1.4535  114.64 -178.54  111.57  1.4249
IC O14  C12  *C14  C15       1.4249  111.57  119.01  110.89  1.5375
IC O14  C12  *C14  H14       1.4249  111.57 -120.84  109.50  1.1151
IC C12  C14  O14   H14O      1.5331  111.57  -42.06  101.39  0.9846
IC C12  C14  C15   H151      1.5331  110.89 -178.38  110.18  1.1099
IC H151 C14  *C15  H152      1.1099  110.18  119.85  109.89  1.1103
IC H151 C14  *C15  H153      1.1099  110.18 -120.46  110.72  1.1094
IC C4   N9   CM    HM1       1.3842  126.37  -58.53  110.24  1.1128
IC HM1  N9   *CM   HM2       1.1128  110.24  118.36  110.05  1.1131
IC HM1  N9   *CM   HM3       1.1128  110.24 -120.94  112.56  1.1126

RESI BT6A          -1.00 ! N6-threonylcarbamoyladenosine, 6TA yxu
GROUP
ATOM N9     NG2R51 -0.01 !               H152 H153
ATOM C8     CG2R53  0.39 !                  \ /
ATOM H8     HGR52   0.09 !            H151-C15   O14-H14O
ATOM N7     NG2R50 -0.81 !                     \  /
ATOM C5     CG2RC0  0.35 !           O10      C14-H14
ATOM C6     CG2R64  0.66 !           ||      /
ATOM N6     NG2S1  -0.68 !       H6   C10   C12-H12
ATOM H6     HGP1    0.32 !        \  /  \  /  \
ATOM N1     NG2R62 -0.68 !         N6    N11   C13-ODA (-)
ATOM C2     CG2R64  0.39 !         |      |     ||
ATOM H2     HGR62   0.14 !         |     H11   ODB
ATOM N3     NG2R62 -0.82 !         C6
ATOM C4     CG2RC0  0.57 !        // \
ATOM C10    CG2O6   0.60 !       N1   C5--N7\\
ATOM O10    OG2D1  -0.50 !       |    ||     C8-H8
ATOM N11    NG2S1  -0.48 !       C2   C4--N9/
ATOM H11    HGP1    0.29 !      / \\ /      \
ATOM C12    CG311   0.09 !    H2   N3        \
ATOM H12    HGA1    0.09 !                    \
GROUP                    !                     \
ATOM C13    CG2O3   0.52 !                      \
ATOM ODA    OG2D2  -0.76
ATOM ODB    OG2D2  -0.76
GROUP
ATOM C14    CG311   0.14
ATOM H14    HGA1    0.09
ATOM O14    OG311  -0.65
ATOM H14O   HGP1    0.42
ATOM C15    CG331  -0.27
ATOM H151   HGA3    0.09
ATOM H152   HGA3    0.09
ATOM H153   HGA3    0.09 !  !!!! PATCH 6AH for the carboxylic acid form
GROUP
ATOM CM     CG331  -0.27
ATOM HM1    HGA3    0.09
ATOM HM2    HGA3    0.09
ATOM HM3    HGA3    0.09
BOND CM   HM1    CM   HM2   CM   HM3
BOND N9   C8     N9   C4      C8   N7      C8   H8
BOND N7   C5     C5   C6      C5   C4      C6   N6
BOND C6   N1     N6   C10     N6   H6      N1   C2
BOND C2   N3     C2   H2      N3   C4      C10  O10
BOND C10  N11    N11  C12     N11  H11     C12  C13
```

```
BOND C12  C14    C12  H12     C13  ODA     C13  ODB
BOND C14  O14    C14  C15     C14  H14     O14  H14O
BOND C15  H151   C15  H152    C15  H153
BOND CM   N9
IMPR C10    N6     N11    O10    C13    ODB    ODA    C12     C6   C5   N1   N6
DONO H6   N6
DONO H11  N11
DONO H14O O14
ACCE N3
ACCE N7
ACCE N1
ACCE O10  C10
ACCE O14
ACCE ODA  C13
ACCE ODB  C13
IC C8   C4   *N9   CM        1.3828  106.09  179.99  126.44  1.4731
IC C4   N9   C8    N7        1.3849  106.09    0.11  113.99  1.3240
IC N7   N9   *C8   H8        1.3240  113.99  179.92  121.59  1.0916
IC N9   C8   N7    C5        1.3828  113.99   -0.25  103.48  1.4083
IC C4   N7   *C5   C6        1.3970  110.73  179.73  132.83  1.4006
IC N7   C5   C6    N6        1.4083  132.83    1.51  115.74  1.3912
IC N6   C5   *C6   N1        1.3912  115.74  179.14  117.95  1.3716
IC C5   C6   N6    C10       1.4006  115.74 -179.30  126.67  1.3512
IC C10  C6   *N6   H6        1.3512  126.67 -166.81  114.36  0.9900
IC C5   C6   N1    C2        1.4006  117.95   -0.59  120.88  1.3530
IC C6   N1   C2    N3        1.3716  120.88    0.62  124.48  1.3554
IC N3   N1   *C2   H2        1.3554  124.48  179.69  117.26  1.0948
IC C6   N6   C10   N11       1.3912  126.67 -165.14  108.04  1.3549
IC N11  N6   *C10  O10       1.3549  108.04  177.28  126.98  1.2213
IC N6   C10  N11   C12       1.3512  108.04  148.36  123.11  1.4555
IC C12  C10  *N11  H11       1.4555  123.11 -132.28  115.99  1.0021
IC C10  N11  C12   C14       1.3549  123.11  113.44  114.35  1.5303
IC C14  N11  *C12  C13       1.5303  114.35  118.53  106.46  1.5501
IC C13  N11  *C12  H12       1.5501  106.46  116.72  110.75  1.1118
IC N11  C12  C13   ODA       1.4555  106.46  179.84  116.22  1.2604
IC ODA  C12  *C13  ODB       1.2604  116.22  178.93  116.06  1.2629
IC N11  C12  C14   O14       1.4555  114.35 -178.22  111.42  1.4232
IC O14  C12  *C14  C15       1.4232  111.42  118.82  110.51  1.5366
IC O14  C12  *C14  H14       1.4232  111.42 -120.88  109.63  1.1155
IC C12  C14  O14   H14O      1.5303  111.42  -42.18  100.98  0.9855
IC C12  C14  C15   H151      1.5303  110.51 -177.51  110.26  1.1098
IC H151 C14  *C15  H152      1.1098  110.26  119.93  109.79  1.1100
IC H151 C14  *C15  H153      1.1098  110.26 -120.38  110.57  1.1095
IC C4   N9   CM    HM1       1.3849  126.44   60.23  110.19  1.1136
IC HM1  N9   *CM   HM2       1.1136  110.19  120.70  112.45  1.1118
IC HM1  N9   *CM   HM3       1.1136  110.19 -118.52  110.16  1.1121

RESI B12A          -1.00 ! 2-methylthio-N6-threonyl carbamoyladenosine, STA yxu
GROUP
ATOM N9     NG2R51 -0.01 !                    H152  H153
ATOM C8     CG2R53  0.37 !                      \ /
ATOM H8     HGR52   0.10 !                 H151-C15  O14-H14O
ATOM N7     NG2R50 -0.84 !                       \  /
ATOM C5     CG2RC0  0.28 !                O10     C14-H14
ATOM C6     CG2R64  0.43 !                ||     /
ATOM N1     NG2R62 -0.58 !           H6   C10  C12-H12
ATOM C2     CG2R64  0.63 !            \  /  \ /  \
ATOM N3     NG2R62 -0.76 !             N6    N11  C13-ODA (-)
ATOM C4     CG2RC0  0.48 !             |      |    ||
ATOM S2     SG311  -0.28 !             |     H11  ODB
ATOM C2M    CG331  -0.09 !             C6
ATOM H21    HGA3    0.09 !            // \
ATOM H22    HGA3    0.09 !    H21      N1   C5--N7\\
```

```
ATOM H23    HGA3    0.09 !      \      |    ||     C8-H8
ATOM N6     NG2S1  -0.43 !  H22 -C2M  C2   C4--N9/
ATOM H6     HGP1    0.32 !      / \  / \\ /      \
ATOM C10    CG2O6   0.60 !    H23  S2   N3         \
ATOM O10    OG2D1  -0.50 !                          \
ATOM N11    NG2S1  -0.48 !                           \
ATOM H11    HGP1    0.31 !                            \
ATOM C12    CG311   0.09
ATOM H12    HGA1    0.09
GROUP
ATOM C13    CG2O3   0.52
ATOM ODA    OG2D2  -0.76
ATOM ODB    OG2D2  -0.76
GROUP
ATOM C14    CG311   0.14
ATOM H14    HGA1    0.09
ATOM O14    OG311  -0.65
ATOM H14O   HGP1    0.42 !  !!!! PATCH 6AH for the carboxylic acid form
ATOM C15    CG331  -0.27
ATOM H151   HGA3    0.09
ATOM H152   HGA3    0.09
ATOM H153   HGA3    0.09
GROUP
ATOM CM     CG331  -0.27
ATOM HM1    HGA3    0.09
ATOM HM2    HGA3    0.09
ATOM HM3    HGA3    0.09
BOND CM   HM1    CM   HM2   CM   HM3
BOND N9   C8     N9   C4      C8   N7      C8   H8
BOND N7   C5     C5   C6      C5   C4      C6   N6
BOND C6   N1     N6   H6      N6   C10     N1   C2
BOND C2   N3     C2   S2      N3   C4      S2   C2M
BOND C2M  H21    C2M  H22     C2M  H23     C10  O10
BOND C10  N11    N11  C12     N11  H11     C12  C14
BOND C12  C13    C12  H12     C13   ODA    C13   ODB
BOND C14  O14    C14  C15     C14  H14     O14  H14O
BOND C15  H151   C15  H152    C15  H153
BOND CM   N9
IMPR C10    N6     N11    O10    C13     ODB     ODA    C12     C6   C5   N1   N6
DONO H6   N6
DONO H11  N11
DONO H14  O14
ACCE N3
ACCE N7
ACCE N1
ACCE O10 C10
ACCE O14
ACCE ODA  C13
ACCE ODB  C13
IC C8   C4   *N9   CM        1.3833  105.89 -179.99  126.48  1.4711
IC C4   N9   C8    N7        1.3846  105.89   -0.09  114.12  1.3274
IC N7   N9   *C8   H8        1.3274  114.12 -179.99  121.46  1.0924
IC N9   C8   N7    C5        1.3833  114.12   -0.17  103.37  1.3974
IC C4   N7   *C5   C6        1.4012  111.10  179.71  131.64  1.3929
IC N7   C5   C6    N6        1.3974  131.64    2.16  115.54  1.3944
IC N6   C5   *C6   N1        1.3944  115.54  178.29  117.77  1.3691
IC C5   C6   N1    C2        1.3929  117.77   -0.19  120.44  1.3579
IC C6   N1   C2    S2        1.3691  120.44 -179.86  119.45  1.7512
IC S2   N1   *C2   N3        1.7512  119.45 -179.88  125.19  1.3571
IC N1   C2   S2    C2M       1.3579  119.45    2.95   98.34  1.8338
IC C2   S2   C2M   H21       1.7512   98.34  178.67  110.79  1.1109
IC H21  S2   *C2M  H22       1.1109  110.79  119.71  112.02  1.1120
IC H21  S2   *C2M  H23       1.1109  110.79 -119.27  111.83  1.1133
```

```
IC C5   C6   N6    C10       1.3929  115.54 -174.55  126.36  1.3557
IC C10  C6   *N6   H6        1.3557  126.36 -167.67  113.84  0.9908
IC C6   N6   C10   N11       1.3944  126.36 -163.27  109.52  1.3585
IC N11  N6   *C10  O10       1.3585  109.52  178.15  126.51  1.2243
IC N6   C10  N11   C12       1.3557  109.52  151.13  122.73  1.4547
IC C12  C10  *N11  H11       1.4547  122.73 -128.48  115.96  1.0052
IC C10  N11  C12   C14       1.3585  122.73  118.24  114.45  1.5293
IC C14  N11  *C12  C13       1.5293  114.45  117.90  105.41  1.5484
IC C14  N11  *C12  H12       1.5293  114.45 -125.45  111.20  1.1125
IC N11  C12  C13   ODA       1.4547  105.41  179.83  116.25  1.2601
IC ODA  C12  *C13  ODB       1.2601  116.25  178.73  115.70  1.2621
IC N11  C12  C14   O14       1.4547  114.45 -177.81  111.15  1.4233
IC O14  C12  *C14  C15       1.4233  111.15  118.90  110.44  1.5376
IC O14  C12  *C14  H14       1.4233  111.15 -120.85  109.87  1.1149
IC C12  C14  O14   H14O      1.5293  111.15  -43.61  101.27  0.9847
IC C12  C14  C15   H151      1.5293  110.44 -177.17  110.28  1.1094
IC H151 C14  *C15  H152      1.1094  110.28  119.82  109.96  1.1104
IC H151 C14  *C15  H153      1.1094  110.28 -120.17  110.58  1.1091
IC C4   N9   CM    HM1       1.3846  126.48   59.44  109.77  1.1129
IC HM1  N9   *CM   HM2       1.1129  109.77  120.54  112.49  1.1125
IC HM1  N9   *CM   HM3       1.1129  109.77 -118.49  110.17  1.1119

RESI B6GA          -1.00 ! N6-glycinylcarbamoyladenosine  yxu
!RING 5 C4 C5 N7 C8 N9
!RING 6 N1 C2 N3 C4 C5 C6
GROUP
ATOM N9     NG2R51 -0.01 !           O10      H122
ATOM C8     CG2R53  0.39 !           ||      /
ATOM H8     HGR52   0.09 !      H6   C10  C12-H121
ATOM N7     NG2R50 -0.81 !       \  /  \  /  \
ATOM C5     CG2RC0  0.35 !        N6    N11   C13-ODA (-)
ATOM C6     CG2R64  0.66 !        |      |     ||
ATOM N6     NG2S1  -0.68 !        |     H11   ODB
ATOM H6     HGP1    0.32 !        C6
ATOM N1     NG2R62 -0.68 !       // \
ATOM C2     CG2R64  0.39 !      N1   C5--N7\\
ATOM H2     HGR62   0.14 !      |    ||     C8-H8
ATOM N3     NG2R62 -0.82 !      C2   C4--N9/
ATOM C4     CG2RC0  0.57 !     / \\ /      \
ATOM C10    CG2O6   0.60 !   H2   N3        \
ATOM O10    OG2D1  -0.50 !                   \
ATOM N11    NG2S1  -0.48 !                    \
ATOM H11    HGP1    0.29 !                     \
ATOM C12    CG321   0.00
ATOM H121   HGA2    0.09
ATOM H122   HGA2    0.09
GROUP
ATOM C13    CG2O3   0.52
ATOM ODA    OG2D2  -0.76
ATOM ODB    OG2D2  -0.76
GROUP
ATOM CM     CG331  -0.27
ATOM HM1    HGA3    0.09
ATOM HM2    HGA3    0.09
ATOM HM3    HGA3    0.09
BOND CM   HM1    CM   HM2   CM   HM3
BOND N9   C8     N9   C4      C8   N7      C8   H8
BOND N7   C5     C5   C6      C5   C4      C6   N6
BOND C6   N1     N6   C10     N6   H6      N1   C2
BOND C2   N3     C2   H2      N3   C4      C10  N11
BOND C10  O10    N11  C12     N11  H11     C12  C13
BOND C12  H121   C12  H122    C13  ODA     C13  ODB
BOND CM   N9
```

```
IMPR C10    N6     N11    O10    C13    ODB    ODA    C12    C6   C5   N1   N6
DONO H11  N11
ACCE N3
ACCE N7
ACCE N1
ACCE O10  C10
ACCE ODA  C13
ACCE ODB  C13
IC C8   C4   *N9   CM        1.3827  106.04  179.98  126.26  1.4721
IC C4   N9   C8    N7        1.3855  106.04   -0.14  114.03  1.3246
IC N7   N9   *C8   H8        1.3246  114.03  179.96  121.59  1.0920
IC N9   C8   N7    C5        1.3827  114.03   -0.10  103.44  1.4079
IC C4   N7   *C5   C6        1.3963  110.79 -179.42  132.79  1.4007
IC N7   C5   C6    N6        1.4079  132.79    0.35  115.89  1.3916
IC N6   C5   *C6   N1        1.3916  115.89 -179.90  117.86  1.3720
IC C5   C6   N6    C10       1.4007  115.89  176.86  126.83  1.3532
IC C10  C6   *N6   H6        1.3532  126.83 -174.64  115.04  0.9894
IC C5   C6   N1    C2        1.4007  117.86   -0.89  120.98  1.3526
IC C6   N1   C2    N3        1.3720  120.98    0.41  124.40  1.3559
IC N3   N1   *C2   H2        1.3559  124.40  179.69  117.36  1.0945
IC C6   N6   C10   N11       1.3916  126.83 -178.01  108.66  1.3543
IC N11  N6   *C10  O10       1.3543  108.66  177.42  126.92  1.2226
IC N6   C10  N11   C12       1.3532  108.66  155.20  123.53  1.4507
IC C12  C10  *N11  H11       1.4507  123.53 -148.82  120.78  0.9991
IC C10  N11  C12   C13       1.3543  123.53 -154.16  109.75  1.5364
IC C13  N11  *C12  H121      1.5364  109.75  118.91  111.62  1.1113
IC C13  N11  *C12  H122      1.5364  109.75 -119.00  110.75  1.1087
IC N11  C12  C13   ODA       1.4507  109.75 -177.81  115.78  1.2570
IC ODA  C12  *C13  ODB       1.2570  115.78 -179.95  116.16  1.2630
IC C4   N9   CM    HM1       1.3855  126.26  -59.89  110.34  1.1125
IC HM1  N9   *CM   HM2       1.1125  110.34  118.51  110.05  1.1136
IC HM1  N9   *CM   HM3       1.1125  110.34 -120.93  112.46  1.1129

!!***guanines***

RESI B1MG           0.00 ! 1-methylguanine yxu
GROUP
ATOM N9     NG2R51  0.05 !    H11    O6
ATOM C8     CG2R53  0.25 !     |     ||
ATOM H8     HGR52   0.13 ! H12-CM1   C6
ATOM N7     NG2R50 -0.62 !     |  \ /  \
ATOM C5     CG2RC0 -0.01 !    H13  N1   C5--N7\\
ATOM C6     CG2R63  0.62 !         |    ||     C8-H8
ATOM O6     OG2D4  -0.50 !         C2   C4--N9/
ATOM N1     NG2R61 -0.21 !        / \\ /      \
ATOM C2     CG2R64  0.73 !  H21-N2   N3        \
ATOM N2     NG2S3  -0.64 !      |               \
ATOM H21    HGP4    0.32 !     H22               \
ATOM H22    HGP4    0.32 !                        \
ATOM N3     NG2R62 -0.77
ATOM C4     CG2RC0  0.29
ATOM CM1    CG331  -0.23
ATOM H11    HGA3    0.09
ATOM H12    HGA3    0.09
ATOM H13    HGA3    0.09
GROUP
ATOM CM     CG331  -0.27
ATOM HM1    HGA3    0.09
ATOM HM2    HGA3    0.09
ATOM HM3    HGA3    0.09
BOND CM   HM1    CM   HM2   CM   HM3
BOND N9   C8   N9   C4   C8   N7   C8   H8
BOND N7   C5   C5   C6   C5   C4   C6   O6
```

```
BOND C6   N1   N1   C2   N1   CM1  C2   N2
BOND C2   N3   N2   H21  N2   H22  N3   C4
BOND CM1  H11  CM1  H12  CM1  H13
BOND CM   N9
IMPR C6     C5     N1     O6     C2     N1     N3     N2     N2     H22    H21    C2
!ribose
IC C8   C4   *N9   CM        1.3791  105.54 -179.95  126.56  1.4896
IC C4   N9   C8    N7        1.3706  106.80   -0.25  113.01  1.3236
IC C8   N9   C4    C5        1.3756  106.80    0.28  104.92  1.3908
IC N9   C5   *C4   N3        1.3706  104.92 -178.38  128.79  1.3597
IC C5   C4   N3    C2        1.3908  128.79   -2.21  111.93  1.3155
IC C4   N3   C2    N1        1.3597  111.93    0.23  125.33  1.3775
IC N1   N3   *C2   N2        1.3775  125.33 -175.47  118.02  1.3874
IC N3   C2   N2    H21       1.3155  118.02  -11.87  109.98  1.0162
IC H21  C2   *N2   H22       1.0162  109.98 -128.49  115.91  1.0139
IC N3   C2   N1    C6        1.3155  125.33    2.33  124.21  1.4462
IC C6   C2   *N1   CM1       1.4462  124.21 -178.86  119.74  1.4639
IC C5   N1   *C6   O6        1.4356  110.25 -179.31  120.64  1.2270
IC N9   N7   *C8   H8        1.3756  113.01 -179.97  125.08  1.0827
IC C6   N1   CM1   H11       1.4462  116.04 -129.76  109.62  1.0911
IC H11  N1   *CM1  H12       1.0911  109.62 -121.75  111.06  1.0957
IC H11  N1   *CM1  H13       1.0911  109.62  120.02  106.67  1.0879
IC C4   N9   CM    HM1       1.3797  126.25   61.01  109.91  1.1125
IC HM1  N9   *CM   HM2       1.1125  109.91  120.71  112.26  1.1123
IC HM1  N9   *CM   HM3       1.1125  109.91 -118.56  109.94  1.1113
DONO H21   N2
DONO H22   N2
ACCE O6   C6
ACCE N3
ACCE N7

RESI B2MG           0.00 ! N2-methylguanine yxu
GROUP
ATOM N9     NG2R51 -0.02 !            O6
ATOM C8     CG2R53  0.27 !            ||
ATOM H8     HGR52   0.14 !            C6
ATOM N7     NG2R50 -0.62 !           /  \
ATOM C5     CG2RC0  0.03 !       H1-N1   C5--N7\\
ATOM C6     CG2R63  0.52 !          |    ||     C8-H8
ATOM O6     OG2D4  -0.50 !          C2   C4--N9/
ATOM N1     NG2R61 -0.43 !         / \\ /      \
ATOM H1     HGP1    0.32 !   H2 -N2   N3        \
ATOM C4     CG2RC0  0.29 !       |               \
GROUP                    !      CM2               \
ATOM C2     CG2R64  0.68 !     / | \               \
ATOM N2     NG311  -0.47 !  H21 H22 H23             \
ATOM H2     HGPAM1  0.32 !                           \
ATOM N3     NG2R62 -0.77
ATOM CM2    CG331  -0.03
ATOM H21    HGA3    0.09
ATOM H22    HGA3    0.09
ATOM H23    HGA3    0.09
GROUP
ATOM CM     CG331  -0.27
ATOM HM1    HGA3    0.09
ATOM HM2    HGA3    0.09
ATOM HM3    HGA3    0.09
BOND CM   HM1   CM   HM2   CM   HM3
BOND N9   C8   N9   C4   C8   N7   C8   H8
BOND N7   C5   C5   C6   C5   C4   C6   O6
BOND C6   N1   N1   C2   N1   H1   C2   N2
BOND C2   N3   N2   H2   N2   CM2  N3   C4
BOND CM2  H21  CM2  H22  CM2  H23
```

```
BOND CM   N9
IMPR C6     C5     N1     O6     C2     N1     N3     N2     ! N2   C2   CM2   H2
DONO H2   N2
DONO H1   N1
ACCE O6   C6
ACCE N3
ACCE N7
IC C8   C4   *N9   CM        1.3802  105.93  179.98  126.25  1.4689
IC C4   N9   C8    N7        1.3797  105.93    0.03  113.75  1.3249
IC N7   N9   *C8   H8        1.3249  113.75 -179.93  121.68  1.0907
IC N9   C8   N7    C5        1.3802  113.75    0.01  104.33  1.3903
IC C4   N7   *C5   C6        1.4100  110.10 -179.95  131.15  1.4172
IC N7   C5   C6    N1        1.3903  131.15 -179.67  112.61  1.3799
IC N1   C5   *C6   O6        1.3799  112.61  179.61  130.53  1.2316
IC C5   C6   N1    C2        1.4172  112.61   -0.56  126.72  1.3757
IC C2   C6   *N1   H1        1.3757  126.72 -178.01  114.74  0.9967
IC C6   N1   C2    N2        1.3799  126.72  179.58  121.68  1.3516
IC N2   N1   *C2   N3        1.3516  121.68 -179.14  120.51  1.3418
IC N1   C2   N2    CM2       1.3757  121.68  -28.67  125.35  1.4795
IC CM2  C2   *N2   H2        1.4795  125.35 -148.22  113.37  1.0148
IC C2   N2   CM2   H21       1.3516  125.35  -76.62  110.79  1.1144
IC H21  N2   *CM2  H22       1.1144  110.79  120.88  112.05  1.1116
IC H21  N2   *CM2  H23       1.1144  110.79 -119.44  110.41  1.1133
IC C4   N9   CM    HM1       1.3797  126.25   61.01  109.91  1.1125
IC HM1  N9   *CM   HM2       1.1125  109.91  120.71  112.26  1.1123
IC HM1  N9   *CM   HM3       1.1125  109.91 -118.56  109.94  1.1113

RESI B7MG           1.00 ! 7-methylguanine yxu
GROUP
ATOM N9     NG2R52 -0.10 !           O6     H71  H72
ATOM C8     CG2R53  0.40 !           ||       \ /
ATOM H8     HGR53   0.15 !           C6        CM7-H73
ATOM N7     NG2R52 -0.40 !          /  \   (+)/
ATOM C5     CG2RC0  0.13 !      H1-N1   C5--N7\\
ATOM C6     CG2R63  0.60 !         |    ||     C8-H8
ATOM O6     OG2D4  -0.45 !         C2   C4--N9/
ATOM N1     NG2R61 -0.29 !        / \\ /      \
ATOM H1     HGP1    0.25 !  H21-N2   N3        \
ATOM C2     CG2R64  0.73 !      |               \
ATOM N2     NG2S3  -0.69 !     H22               \
ATOM H21    HGP4    0.34 !                        \
ATOM H22    HGP4    0.34
ATOM N3     NG2R62 -0.55
ATOM C4     CG2RC0  0.16
ATOM CM7    CG334   0.11
ATOM H71    HGA3    0.09
ATOM H72    HGA3    0.09
ATOM H73    HGA3    0.09
GROUP                    !                |      |
ATOM CM     CG334  -0.27
ATOM HM1    HGA3    0.09
ATOM HM2    HGA3    0.09
ATOM HM3    HGA3    0.09
BOND CM   HM1    CM   HM2   CM   HM3
BOND N9   C8   N9   C4   C8   N7   C8   H8
BOND N7   C5   N7   CM7  C5   C6   C5   C4
BOND C6   O6   C6   N1   N1   C2   N1   H1
BOND C2   N2   C2   N3   N2   H21  N2   H22
BOND N3   C4   CM7  H71  CM7  H72  CM7  H73
BOND CM   N9
IMPR C6     C5     N1     O6     C2     N1     N3     N2     N2     H22    H21    C2
DONO H21  N2
DONO H22  N2
```

```
DONO H1   N1
ACCE O6   C6
ACCE N3
IC C8   C4   *N9   CM        1.3333  109.17 -179.99  124.96  1.4776
IC C4   N9   C8    N7        1.3833  109.17    0.00  109.98  1.3361
IC N7   N9   *C8   H8        1.3361  109.98 -179.98  124.19  1.0733
IC N9   C8   N7    C5        1.3333  109.98    0.03  107.79  1.3967
IC C5   C8   *N7   CM7       1.3967  107.79  179.96  126.34  1.4800
IC C4   N7   *C5   C6        1.3953  107.02 -179.92  132.78  1.4162
IC N7   C5   C6    N1        1.3967  132.78  179.97  112.51  1.3887
IC N1   C5   *C6   O6        1.3887  112.51  179.96  128.83  1.2306
IC C5   C6   N1    C2        1.4162  112.51    0.02  125.33  1.3815
IC C2   C6   *N1   H1        1.3815  125.33  179.93  116.65  1.0026
IC C6   N1   C2    N2        1.3887  125.33  179.99  116.89  1.3324
IC N2   N1   *C2   N3        1.3324  116.89  179.96  120.98  1.3483
IC N1   C2   N2    H21       1.3815  116.89   -0.07  123.87  0.9959
IC H21  C2   *N2   H22       0.9959  123.87 -179.94  117.66  0.9978
IC C8   N7   CM7   H71       1.3361  126.34 -120.51  109.18  1.1138
IC H71  N7   *CM7  H72       1.1138  109.18  120.49  111.19  1.1133
IC H71  N7   *CM7  H73       1.1138  109.18 -118.98  109.19  1.1133
IC C4   N9   CM    HM1       1.3833  124.96  179.96  111.38  1.1121
IC HM1  N9   *CM   HM2       1.1121  111.38  120.75  108.83  1.1131
IC HM1  N9   *CM   HM3       1.1121  111.38 -120.71  108.83  1.1131

RESI B27G           1.00 ! N2,7-dimethylguanine, yxu
GROUP
ATOM N9     NG2R52 -0.11 !             O6     H71  H72
ATOM C8     CG2R53  0.41 !             ||       \ /
ATOM H8     HGR53   0.15 !             C6        C7-H73
ATOM N7     NG2R52 -0.40 !            /  \   (+)/
ATOM C5     CG2RC0  0.13 !        H1-N1   C5--N7\\
ATOM C6     CG2R63  0.59 !            |    ||     C8-H8
ATOM O6     OG2D4  -0.46 !  H11        C2   C4--N9/
ATOM N1     NG2R61 -0.28 !     \     / \\ /      \
ATOM H1     HGP1    0.25 ! H12-C10-N2   N3         \
ATOM C2     CG2R64  0.73 !     /   |                \
ATOM N3     NG2R62 -0.57 !  H13    H2                \
ATOM C4     CG2RC0  0.14 !                            \
ATOM C7     CG334   0.15 !                             \
ATOM H71    HGA3    0.09
ATOM H72    HGA3    0.09
ATOM H73    HGA3    0.09
GROUP
ATOM N2     NG311  -0.52
ATOM H2     HGPAM1  0.37
ATOM C10    CG331  -0.12
ATOM H11    HGA3    0.09
ATOM H12    HGA3    0.09
ATOM H13    HGA3    0.09
GROUP
ATOM CM     CG334  -0.27
ATOM HM1    HGA3    0.09
ATOM HM2    HGA3    0.09
ATOM HM3    HGA3    0.09
BOND CM   HM1    CM   HM2   CM   HM3
BOND N9   C8   N9   C4   C8   N7   C8   H8
BOND N7   C5   N7   C7   C5   C6   C5   C4
BOND C6   O6   C6   N1   N1   C2   N1   H1
BOND C2   N2   C2   N3   N2   H2   N2   C10
BOND N3   C4   C10  H11  C10  H12  C10  H13
BOND C7   H71  C7   H72  C7   H73
BOND CM   N9
IMPR C6     C5     N1     O6     C2     N1     N3     N2     ! N2   C2   C10   H2
```

```
DONO H2    N2
DONO H1   N1
ACCE O6   C6
ACCE N3
IC C8   C4   *N9   CM        1.3332  109.17  179.99  124.87  1.4774
IC C4   N9   C8    N7        1.3837  109.17   -0.03  109.90  1.3360
IC N7   N9   *C8   H8        1.3360  109.90  179.95  124.09  1.0725
IC N9   C8   N7    C5        1.3332  109.90    0.02  107.85  1.3970
IC C5   C8   *N7   C7        1.3970  107.85  179.90  126.43  1.4816
IC C4   N7   *C5   C6        1.3937  106.99  179.88  132.77  1.4144
IC N7   C5   C6    N1        1.3970  132.77 -179.84  112.45  1.3869
IC N1   C5   *C6   O6        1.3869  112.45 -179.96  128.75  1.2304
IC C5   C6   N1    C2        1.4144  112.45    0.09  126.02  1.3857
IC C2   C6   *N1   H1        1.3857  126.02  179.44  116.69  1.0031
IC C6   N1   C2    N2        1.3869  126.02  179.87  121.20  1.3572
IC N2   N1   *C2   N3        1.3572  121.20  179.83  119.82  1.3547
IC C8   N7   C7    H71       1.3360  126.43 -122.99  109.17  1.1140
IC H71  N7   *C7   H72       1.1140  109.17  120.44  111.15  1.1134
IC H71  N7   *C7   H73       1.1140  109.17 -119.00  109.15  1.1124
IC N1   C2   N2    C10       1.3857  121.20   18.26  127.37  1.4827
IC C10  C2   *N2   H2        1.4827  127.37  157.15  115.45  1.0194
IC C2   N2   C10   H11       1.3572  127.37 -159.49  110.35  1.1141
IC H11  N2   *C10  H12       1.1141  110.35  119.38  112.31  1.1108
IC H11  N2   *C10  H13       1.1141  110.35 -119.14  111.04  1.1139
IC C4   N9   CM    HM1       1.3837  124.87 -178.72  111.35  1.1128
IC HM1  N9   *CM   HM2       1.1128  111.35  120.66  108.75  1.1127
IC HM1  N9   *CM   HM3       1.1128  111.35 -120.76  108.81  1.1123

RESI BDCG           0.00 ! 7-cyano-7-deazaguanine, yxu
!RING 6 N1 C2 N3 C4 C5 C6
GROUP
ATOM N9     NG2R51  0.18 !           O6         N10
ATOM C8     CG2R51 -0.09 !           ||        ///
ATOM H8     HGR52   0.19 !           C6       C10
ATOM C7     CG2R51 -0.10 !          /  \     /
ATOM C5     CG2RC0 -0.11 !      H1-N1   C5--C7\\
ATOM C6     CG2R63  0.57 !          |    ||     C8-H8
ATOM O6     OG2D4  -0.51 !          C2   C4--N9/
ATOM N1     NG2R61 -0.35 !         / \\ /      \
ATOM H1     HGP1    0.26 !  H21-N2   N3         \
ATOM C2     CG2R64  0.77 !      |                \
ATOM N2     NG2S3  -0.60 !     H22                \
ATOM H21    HGP4    0.29 !                         \
ATOM H22    HGP4    0.29
ATOM N3     NG2R62 -0.73
ATOM C4     CG2RC0  0.14
ATOM C10    CG1N1   0.24
ATOM N10    NG1T1  -0.44
GROUP
ATOM CM     CG331  -0.27
ATOM HM1    HGA3    0.09
ATOM HM2    HGA3    0.09
ATOM HM3    HGA3    0.09
BOND CM   HM1    CM   HM2   CM   HM3
BOND N9   C8   N9   C4   C8   C7   C8   H8
BOND C7   C5   C7   C10  C5   C6   C5   C4
BOND C6   O6   C6   N1   N1   C2   N1   H1
BOND C2   N2   C2   N3   N2   H21  N2   H22
BOND N3   C4   C10  N10
BOND CM   N9
IMPR C6     C5     N1     O6     C2     N1     N3     N2     N2     H22    H21    C2
DONO H21   N2
DONO H22   N2
```

```
DONO H1   N1
ACCE O6   C6
ACCE N3
ACCE N10  C10
IC C8   C4   *N9   CM        1.3844  110.07  179.98  124.48  1.4638
IC C4   N9   C8    C7        1.3805  110.07   -0.01  108.55  1.3681
IC C7   N9   *C8   H8        1.3681  108.55  179.97  122.19  1.0804
IC N9   C8   C7    C5        1.3844  108.55    0.00  107.15  1.4355
IC C5   C8   *C7   C10       1.4355  107.15  179.98  125.85  1.4271
IC C4   C7   *C5   C6        1.4050  107.69  179.97  132.57  1.4044
IC C7   C5   C6    N1        1.4355  132.57 -179.98  112.92  1.3791
IC N1   C5   *C6   O6        1.3791  112.92  179.96  128.98  1.2281
IC C5   C6   N1    C2        1.4044  112.92   -0.04  125.48  1.3728
IC C2   C6   *N1   H1        1.3728  125.48 -179.89  116.08  0.9974
IC C6   N1   C2    N2        1.3791  125.48 -179.95  116.89  1.3269
IC N2   N1   *C2   N3        1.3269  116.89 -179.93  121.48  1.3405
IC N1   C2   N2    H21       1.3728  116.89    0.03  123.45  0.9934
IC H21  C2   *N2   H22       0.9934  123.45 -179.98  116.10  0.9951
IC C8   C7   C10   N10       1.3681  125.85    0.30  178.17  1.1809
IC C4   N9   CM    HM1       1.3805  124.48  179.63  112.48  1.1103
IC HM1  N9   *CM   HM2       1.1103  112.48  120.88  109.72  1.1113
IC HM1  N9   *CM   HM3       1.1103  112.48 -120.73  109.71  1.1108

RESI BRCG           1.00 ! archaeosine, yxu
!RING 6 N1 C2 N3 C4 C5 C6
GROUP
ATOM N9     NG2R51  0.04 !                 H112      H122
ATOM C4     CG2RC0  0.32 !                    \  (+) /
ATOM C5     CG2RC0 -0.06 !            O6  H111-N11  N12-H121
ATOM C6     CG2R63  0.42 !            ||         \  //
ATOM O6     OG2D4  -0.52 !            C6          C10
ATOM N1     NG2R61 -0.38 !           /  \        /
ATOM H1     HGP1    0.30 !      H1-N1   C5--C7\\
ATOM C2     CG2R64  0.65 !          |    ||     C8-H8
ATOM N2     NG2S3  -0.60 !          C2   C4--N9/
ATOM H21    HGP4    0.30 !         / \\ /      \
ATOM H22    HGP4    0.30 !  H21-N2   N3         \
ATOM N3     NG2R62 -0.77 !      |                \
GROUP                    !     H22                \
ATOM C7     CG2R51  0.06 !                         \
ATOM C8     CG2R51 -0.21
ATOM H8     HGR52   0.24
ATOM C10    CG2N2   0.61
ATOM N11    NG2P1  -0.43
ATOM H111   HGP2    0.29
ATOM H112   HGP2    0.29
ATOM N12    NG2P1  -0.43
ATOM H121   HGP2    0.29
ATOM H122   HGP2    0.29
GROUP
ATOM CM     CG331  -0.27
ATOM HM1    HGA3    0.09
ATOM HM2    HGA3    0.09
ATOM HM3    HGA3    0.09
BOND CM   HM1    CM   HM2   CM   HM3
BOND N9   C8   N9   C4   C8   C7   C8   H8
BOND C7   C5   C7   C10  C5   C6   C5   C4
BOND C6   O6   C6   N1   N1   C2   N1   H1
BOND C2   N2   C2   N3   N2   H21  N2   H22
BOND N3   C4   C10  N11  C10  N12  N11  H111
BOND N11  H112 N12  H121 N12  H122
BOND CM   N9
IMPR C6     C5     N1     O6     C2     N1     N3     N2
```

```
IMPR N2     H22    H21    C2     C10    N12    N11    C7
DONO H21   N2
DONO H22   N2
DONO H111  N11
DONO H112  N11
DONO H121  N12
DONO H122  N12
DONO H1   N1
ACCE O6   C6
ACCE N3
IC C8   C4   *N9   CM        1.3851  110.54  179.56  124.70  1.4640
IC C8   N9   C4    C5        1.3851  110.54    0.04  106.26  1.4034
IC C5   N9   *C4   N3        1.4034  106.26 -179.59  128.22  1.3439
IC N9   C4   C5    C6        1.3790  106.26 -179.02  118.44  1.4040
IC C6   C4   *C5   C7        1.4040  118.44  179.92  108.57  1.4512
IC C4   C5   C6    N1        1.4034  118.44   -0.55  113.86  1.3808
IC N1   C5   *C6   O6        1.3808  113.86  179.69  127.24  1.2293
IC C5   C6   N1    C2        1.4040  113.86    0.11  125.27  1.3754
IC C2   C6   *N1   H1        1.3754  125.27 -179.64  116.80  1.0007
IC N3   N1   *C2   N2        1.3357  120.79  179.77  117.61  1.3239
IC N1   C2   N2    H21       1.3754  117.61   -0.19  123.86  0.9936
IC H21  C2   *N2   H22       0.9936  123.86 -179.61  116.35  0.9963
IC C7   N9   *C8   H8        1.3825  108.94  179.51  119.01  1.0850
IC C8   C5   *C7   C10       1.3825  105.67 -179.91  128.69  1.4649
IC C5   C7   C10   N11       1.4512  128.69 -157.57  120.42  1.3288
IC N11  C7   *C10  N12       1.3288  120.42 -179.79  120.01  1.3248
IC C7   C10  N11   H111      1.4649  120.42  179.79  120.08  0.9983
IC H111 C10  *N11  H112      0.9983  120.08 -177.45  120.30  0.9988
IC C7   C10  N12   H121      1.4649  120.01 -178.52  121.28  0.9973
IC H121 C10  *N12  H122      0.9973  121.28  168.29  118.81  1.0093
IC C8   N9   CM    HM1       1.3851  124.76  120.20  109.97  1.1125
IC HM1  N9   *CM   HM2       1.1125  109.97  118.04  110.01  1.1124
IC HM1  N9   *CM   HM3       1.1125  109.97 -120.92  113.10  1.1110

RESI BDAG           1.00 ! 7-aminomethyl-7-deazaguanine, PQ1   yxu
GROUP
ATOM N9     NG2R51  0.04 !          O6    H101 H102  H111
ATOM C8     CG2R51  0.06 !          ||      \ /     /   (+)
ATOM H8     HGR52   0.08 !          C6       C10--N11-H112
ATOM C7     CG2R51 -0.14 !         /  \     /       \
ATOM C5     CG2RC0 -0.06 !     H1-N1   C5--C7\\      H113
ATOM C6     CG2R63  0.42 !         |    ||     C8-H8
ATOM O6     OG2D4  -0.52 !        C2   C4--N9/
ATOM N1     NG2R61 -0.38 !       / \\ /      \
ATOM H1     HGP1    0.30 !  H21-N2   N3        \
ATOM C2     CG2R64  0.65 !      |               \
ATOM N2     NG2S3  -0.60 !     H22               \
ATOM H21    HGP4    0.30 !                        \
ATOM H22    HGP4    0.30
ATOM N3     NG2R62 -0.77
ATOM C4     CG2RC0  0.32
GROUP
ATOM C10    CG324   0.21
ATOM H101   HGA2    0.05
ATOM H102   HGA2    0.05
ATOM N11    NG3P3  -0.30
ATOM H111   HGP2    0.33
ATOM H112   HGP2    0.33
ATOM H113   HGP2    0.33 !  !!!! PATCH 7GNA for the neutral amino form
GROUP
ATOM CM     CG331  -0.27
ATOM HM1    HGA3    0.09
ATOM HM2    HGA3    0.09
```

```
ATOM HM3    HGA3    0.09
BOND CM   HM1    CM   HM2   CM   HM3
BOND N9   C8   N9   C4   C8   C7   C8   H8
BOND C7   C5   C7   C10  C5   C6   C5   C4
BOND C6   O6   C6   N1   N1   C2   N1   H1
BOND C2   N2   C2   N3   N2   H21  N2   H22
BOND N3   C4   C10  N11  C10  H101 C10  H102
BOND N11  H111 N11  H112 N11  H113
BOND CM   N9
IMPR C6     C5     N1     O6     C2     N1     N3     N2     N2     H22    H21    C2
DONO H21   N2
DONO H22   N2
DONO H1   N1
DONO H111  N11
DONO H112  N11
DONO H113  N11
ACCE O6   C6
ACCE N3
IC C8   C4   *N9   CM        1.3874  110.07 -179.74  124.51  1.4666
IC C4   N9   C8    C7        1.3835  110.07   -0.10  108.80  1.3718
IC C7   N9   *C8   H8        1.3718  108.80 -178.36  121.48  1.0825
IC N9   C8   C7    C5        1.3874  108.80    0.85  106.46  1.4412
IC C5   C8   *C7   C10       1.4412  106.46 -173.39  126.89  1.4936
IC C4   C7   *C5   C6        1.4008  108.33 -179.34  132.47  1.4004
IC C7   C5   C6    N1        1.4412  132.47  178.61  113.58  1.3810
IC N1   C5   *C6   O6        1.3810  113.58  178.90  126.65  1.2326
IC C5   C6   N1    C2        1.4004  113.58   -0.04  125.05  1.3768
IC C2   C6   *N1   H1        1.3768  125.05 -178.53  117.10  1.0008
IC C6   N1   C2    N2        1.3810  125.05 -179.52  117.56  1.3243
IC N2   N1   *C2   N3        1.3243  117.56 -179.24  121.01  1.3379
IC N1   C2   N2    H21       1.3768  117.56    0.95  123.97  0.9930
IC H21  C2   *N2   H22       0.9930  123.97  179.37  116.26  0.9968
IC C8   C7   C10   N11       1.3718  126.89 -138.03  110.63  1.4919
IC N11  C7   *C10  H101      1.4919  110.63  120.42  111.00  1.1026
IC N11  C7   *C10  H102      1.4919  110.63 -118.86  109.45  1.1025
IC C7   C10  N11   H111      1.4936  110.63 -175.33  111.22  1.0375
IC H111 C10  *N11  H112      1.0375  111.22  118.70  106.87  1.0555
IC H111 C10  *N11  H113      1.0375  111.22 -123.12  110.66  1.0371
IC C4   N9   CM    HM1       1.3835  124.51  179.80  112.81  1.1123
IC HM1  N9   *CM   HM2       1.1123  112.81  120.91  110.18  1.1124
IC HM1  N9   *CM   HM3       1.1123  112.81 -120.78  110.11  1.1130

RESI BQUG           1.00 ! queuosine, QUO   yxu
GROUP
ATOM N9     NG2R51  0.04 !                 H13  O13-H13O
ATOM C8     CG2R51  0.06 !                   \  /
ATOM H8     HGR52   0.08 !   H101 H111 H12   C13(R)  H14
ATOM C7     CG2R51 -0.14 !      |    |   \   /    \  /
ATOM C5     CG2RC0 -0.06 ! *---C10--N11--C12(S)   C14(S)
ATOM C6     CG2R63  0.42 !      |    |(+)   \       / \
ATOM O6     OG2D4  -0.52 !   H102 H112      C16==C15 O14-H14O
ATOM N1     NG2R61 -0.38 !                 /       \
ATOM H1     HGP1    0.30 !               H16      H15
ATOM C2     CG2R64  0.65
ATOM N2     NG2S3  -0.60 !           O6
ATOM H21    HGP4    0.30 !           ||         *
ATOM H22    HGP4    0.30 !           C6        /
ATOM N3     NG2R62 -0.77 !          /  \      /
ATOM C4     CG2RC0  0.32 !      H1-N1   C5--C7\\
GROUP                    !         |    ||     C8-H8
ATOM C10    CG324   0.18 !         C2   C4--N9/
ATOM H101   HGA2    0.09 !        / \\ /      \
ATOM H102   HGA2    0.09 !  H21-N2   N3        \
```

```
ATOM N11    NG3P2  -0.35 !      |               \
ATOM H111   HGP2    0.28 !     H22               \
ATOM H112   HGP2    0.28 !                        \
ATOM C12    CG3C53  0.34
ATOM H12    HGA1    0.09
GROUP
ATOM C15    CG2R51 -0.20
ATOM H15    HGR51   0.26
ATOM C16    CG2R51 -0.24
ATOM H16    HGR51   0.18
GROUP
ATOM C13    CG3C51  0.14
ATOM H13    HGA1    0.09
ATOM O13    OG311  -0.65 !  !!!! PATCH 7GNM for the neutral amino form
ATOM H13O   HGP1    0.42
GROUP
ATOM C14    CG3C51  0.14
ATOM H14    HGA1    0.09
ATOM O14    OG311  -0.65
ATOM H14O   HGP1    0.42
GROUP
ATOM CM     CG331  -0.27
ATOM HM1    HGA3    0.09
ATOM HM2    HGA3    0.09
ATOM HM3    HGA3    0.09
BOND CM   HM1    CM   HM2   CM   HM3
BOND N9   C8   N9   C4   C8   H8   C8   C7
BOND C7   C5   C7   C10  C5   C6   C5   C4
BOND C6   O6   C6   N1   N1   H1   N1   C2
BOND C2   N2   C2   N3   N2   H21  N2   H22
BOND N3   C4   C10  H101 C10  H102 C10  N11
BOND N11  H111 N11  H112 N11  C12  C12  H12
BOND C12  C13  C12  C16  C13  O13  C13  H13
BOND C13  C14  O13  H13O  C14  O14  C14  H14
BOND C14  C15  O14  H14O  C15  H15  C15  C16
BOND C16  H16
BOND CM   N9
IMPR C6     C5     N1     O6     C2     N1     N3     N2     N2     H22    H21    C2
DONO H21   N2
DONO H22   N2
DONO H1   N1
DONO H111  N11
DONO H112  N11
DONO H13O  O13
DONO H14O  O14
ACCE O6   C6
ACCE N3
ACCE O13
ACCE O14
IC C4   C8   *N9   CM        1.3829  110.11 -179.74  125.42  1.4682
IC C4   N9   C8    C7        1.3818  110.12    0.38  108.60  1.3701
IC C7   N9   *C8   H8        1.3701  108.60  180.00  124.00  1.0830
IC N9   C8   C7    C10       1.3880  108.60  174.46  126.75  1.4904
IC C10  C8   *C7   C5        1.4904  126.75 -175.57  106.66  1.4405
IC C4   C7   *C5   C6        1.3991  108.20  179.62  132.66  1.4030
IC C7   C5   C6    N1        1.4405  132.66 -178.93  113.58  1.3799
IC N1   C5   *C6   O6        1.3799  113.58 -179.16  127.12  1.2314
IC C5   C6   N1    C2        1.4030  113.58   -0.01  124.94  1.3727
IC C2   C6   *N1   H1        1.3727  124.94  180.00  115.40  1.0100
IC C6   N1   C2    N2        1.3799  124.94  179.67  117.41  1.3224
IC N2   N1   *C2   N3        1.3224  117.41  179.36  121.37  1.3364
IC N1   C2   N2    H21       1.3727  117.41  180.00  121.50  1.0000
IC H21  C2   *N2   H22       1.0000  121.50  180.00  121.50  1.0000
```

```
IC C8   C7   C10  N11       1.3701  126.75   13.24  110.50  1.5058
IC N11  C7   *C10 H101      1.5058  110.50  120.00  109.50  1.1000
IC N11  C7   *C10 H102      1.1000  109.50 -120.00  109.50  1.1000
IC C7   C10  N11  C12       1.4904  110.50 -146.66  120.39  1.4978
IC C12  C10  *N11 H111      1.4978  120.39  120.00  110.80  1.0060
IC C12  C10  *N11 H112      1.0060  110.80 -120.00  110.80  1.0060
IC C10  N11  C12  C13       1.5058  120.39  -50.31  105.54  1.5274
IC C13  N11  *C12 C16       1.5274  105.54 -111.95  113.63  1.5041
IC C13  N11  *C12 H12       1.5274  105.54  120.00  107.50  1.0800
IC N11  C12  C16  C15       1.4978  113.63  135.20  109.51  1.3789
IC C15  C12  *C16 H16       1.3789  109.51  180.00  124.60  1.0800
IC C12  C16  C15  C14       1.5041  109.51    0.87  109.87  1.5183
IC C14  C16  *C15 H15       1.5183  109.87  180.00  126.40  1.0800
IC C14  C12  *C13 O13       1.5419  102.73 -126.51  115.78  1.4400
IC O13  C12  *C13 H13       1.4400  115.78 -120.00  110.10  1.1000
IC C12  C13  O13  H13O      1.5274  115.78  180.00  109.00  0.9600
IC C13  C15  *C14 O14       1.5419  101.54  120.86  109.59  1.4250
IC C13  C15  *C14 H14       1.5419  101.54 -120.00  110.10  1.1000
IC C15  C14  O14  H14O      1.5183  109.59  180.00  109.00  0.9600
IC C4   N9   CM   HM1       1.3829  124.47  -59.78  109.99  1.1123
IC HM1  N9   *CM  HM2       1.1123  109.99  118.32  110.12  1.1126
IC HM1  N9   *CM  HM3       1.1123  109.99 -120.80  112.74  1.1120

RESI BEQG           1.00 ! epoxyqueuosine, yxu
GROUP
ATOM N9     NG2R51  0.04 !                H13  O13-H13O
ATOM C8     CG2R51  0.06 !                  \  /(R)
ATOM H8     HGR52   0.08 !   H101 H111 H12   C13    H14
ATOM C7     CG2R51 -0.14 !      |   |   \   /    \  /
ATOM C5     CG2RC0 -0.06 ! *---C10--N11--C12(R)   C14(R)
ATOM C6     CG2R63  0.42 !      |   |(+)   \      /  \
ATOM O6     OG2D4  -0.52 !   H102 H112  (S)C16--C15   O14-H14O
ATOM N1     NG2R61 -0.38 !                 / \ / \ (R)
ATOM H1     HGP1    0.30 !              H16  O15  H15
ATOM C2     CG2R64  0.65
ATOM N2     NG2S3  -0.60 !          O6
ATOM H21    HGP4    0.30 !          ||         *
ATOM H22    HGP4    0.30 !          C6        /
ATOM N3     NG2R62 -0.77 !         /  \      /
ATOM C4     CG2RC0  0.32 !     H1-N1   C5--C7\\
GROUP                    !        |    ||     C8-H8
ATOM C10    CG324   0.17 !        C2   C4--N9/
ATOM H101   HGA2    0.09 !       / \\ /      \
ATOM H102   HGA2    0.09 ! H21-N2   N3        \
ATOM N11    NG3P2  -0.36 !     |               \
ATOM H111   HGP2    0.29 !    H22               \
ATOM H112   HGP2    0.29 !                       \
ATOM C12    CG3C53  0.34
ATOM H12    HGA1    0.09
GROUP
ATOM C15    CG3RC1  0.15
ATOM H15    HGA1    0.09
ATOM O15    OG3C31 -0.25
ATOM C16    CG3RC1 -0.08
ATOM H16    HGA1    0.09
GROUP
ATOM C13    CG3C51  0.14
ATOM H13    HGA1    0.09 !  !!!! PATCH 7GNM for the neutral amino form
ATOM O13    OG311  -0.65
ATOM H13O   HGP1    0.42
GROUP
ATOM C14    CG3C51  0.14
ATOM H14    HGA1    0.09
```

```
ATOM O14    OG311  -0.65
ATOM H14O   HGP1    0.42
GROUP
ATOM CM     CG331  -0.27
ATOM HM1    HGA3    0.09
ATOM HM2    HGA3    0.09
ATOM HM3    HGA3    0.09
BOND CM   HM1    CM   HM2   CM   HM3
BOND N9   C8   N9   C4   C8   H8   C8   C7
BOND C7   C5   C7   C10  C5   C6   C5   C4
BOND C6   O6   C6   N1   N1   H1   N1   C2
BOND C2   N2   C2   N3   N2   H21  N2   H22
BOND N3   C4   C10  H101 C10  H102 C10  N11
BOND N11  H111 N11  H112 N11  C12  C12  H12
BOND C12  C13  C12  C16  C13  O13  C13  H13
BOND C13  C14  O13  H13O C14  O14  C14  H14
BOND C14  C15  O14  H14O O15  C15  O15  C16
BOND C15  H15  C15  C16  C16  H16
BOND CM   N9
IMPR C6     C5     N1     O6     C2     N1     N3     N2     N2     H22    H21    C2
DONO H21   N2
DONO H22   N2
DONO H1   N1
DONO H111  N11
DONO H112  N11
DONO H13O  O13
DONO H14O  O14
ACCE O6   C6
ACCE N3
ACCE O13
ACCE O14
ACCE O15
IC C4   C8   *N9   CM        1.3918  109.77 -179.79  123.85  1.4747
IC C4   N9   C8    C7        1.3806  110.20   -0.44  108.62  1.3700
IC C7   N9   *C8   H8        1.3700  108.62  180.00  124.00  1.0830
IC N9   C8   C7    C10       1.3876  108.62 -173.38  126.54  1.4895
IC C10  C8   *C7   C5        1.4895  126.54  174.60  106.59  1.4424
IC C4   C7   *C5   C6        1.3994  108.17 -179.55  132.72  1.4024
IC C7   C5   C6    N1        1.4424  132.72  178.64  113.70  1.3796
IC N1   C5   *C6   O6        1.3796  113.70  179.23  127.07  1.2334
IC C5   C6   N1    C2        1.4024  113.70    0.11  124.85  1.3747
IC C2   C6   *N1   H1        1.3747  124.85  180.00  115.40  1.0100
IC C6   N1   C2    N2        1.3796  124.85 -179.62  117.36  1.3215
IC N2   N1   *C2   N3        1.3215  117.36 -179.27  121.27  1.3359
IC N1   C2   N2    H21       1.3747  117.36  180.00  121.50  1.0000
IC H21  C2   *N2   H22       1.0000  121.50  180.00  121.50  1.0000
IC C8   C7   C10   N11       1.3700  126.54  -31.51  110.58  1.5065
IC N11  C7   *C10  H101      1.5065  110.58  120.00  109.50  1.1000
IC N11  C7   *C10  H102      1.1000  109.50 -120.00  109.50  1.1000
IC C7   C10  N11   C12       1.4895  110.58 -115.16  118.55  1.5005
IC C12  C10  *N11  H111      1.5005  118.55  120.00  110.80  1.0060
IC C12  C10  *N11  H112      1.0060  110.80 -120.00  110.80  1.0060
IC C10  N11  C12   C13       1.5065  118.55  -25.32  110.91  1.5374
IC C13  N11  *C12  C16       1.5374  110.91 -112.83  109.92  1.5065
IC C13  N11  *C12  H12       1.5374  110.91  120.00  107.50  1.0800
IC N11  C12  C16   C15       1.5005  109.92  142.84  105.54  1.4980
IC C15  C12  *C16  O15       1.4980  105.54   62.00  114.13  1.4444
IC C15  C12  *C16  H16       1.4444  114.13 -120.00  105.70  1.1110
IC O15  C16  *C15  C14       1.4387   58.88 -110.21  110.93  1.5364
IC C14  C16  *C15  H15       1.5364  110.93 -120.00  110.10  1.1110
IC C14  C12  *C13  O13       1.5393  105.61 -118.13  109.95  1.4342
IC O13  C12  *C13  H13       1.4342  109.95 -120.00  110.10  1.1000
IC C12  C13  O13   H13O      1.5374  109.95  180.00  109.00  0.9600
```

```
IC C13  C15  *C14  O14       1.5393   98.64  116.43  112.45  1.4338
IC C13  C15  *C14  H14       1.5393   98.64 -120.00  110.10  1.1000
IC C15  C14  O14   H14O      1.5364  112.45  180.00  109.00  0.9600
IC C4   N9   CM    HM1       1.3918  126.38   -0.66  136.37  1.1200
IC HM1  N9   *CM   HM2       1.1200  136.37  119.92   83.00  1.1145
IC HM1  N9   *CM   HM3       1.1200  136.37 -119.48   82.80  1.1132

RESI BDWG           0.00 ! 4-demethylwyosine             12/19, yxu
GROUP
ATOM N9     NG2R51  0.02 !                     O6
ATOM C8     CG2R53  0.37 !                     ||
ATOM H8     HGR52   0.09 !              H12    C6
ATOM N7     NG2R50 -0.67 !   H101        |    /  \
ATOM C5     CG2RC0  0.00 !       \      //C12-N1   C5--N7\\
ATOM C6     CG2R63  0.69 !  H102-C10-C11    |    ||      C8-H8
ATOM O6     OG2D4  -0.50 !       /      \N2==C2   C4--N9/
ATOM N1     NG2RC0 -0.11 !   H103             \  /       \
ATOM C2     CG2RC0  0.44 !                     N3         \
ATOM N2     NG2R50 -0.70 !                     |           \
ATOM N3     NG2R61 -0.42 !                     H3           \
ATOM H3     HGP1    0.34 !                                   \
ATOM C4     CG2RC0  0.29
ATOM C12    CG2R51 -0.42
ATOM H12    HGR52   0.21
ATOM C11    CG2R51  0.40
GROUP
ATOM C10    CG331  -0.30
ATOM H101   HGA3    0.09
ATOM H102   HGA3    0.09
ATOM H103   HGA3    0.09
GROUP
ATOM CM     CG331  -0.27
ATOM HM1    HGA3    0.09
ATOM HM2    HGA3    0.09
ATOM HM3    HGA3    0.09
BOND CM   HM1    CM   HM2   CM   HM3
BOND N9   C8   N9   C4   C8   N7   C8   H8
BOND N7   C5   C5   C6   C5   C4   C6   O6
BOND C6   N1   N1   C2   N1   C12  C2   N2
BOND C2   N3   N2   C11  N3   C4   N3   H3
BOND C10  C11  C10  H101 C10  H102 C10  H103
BOND C11  C12  C12  H12
BOND CM   N9
IMPR C6     C5     N1     O6
DONO H3   N3
ACCE O6   C6
ACCE N2
ACCE N7
IC C8   C4   *N9   CM        1.3784  105.67  179.97  126.10  1.4697
IC C4   N9   C8    N7        1.3790  105.67   -0.01  113.20  1.3239
IC N7   N9   *C8   H8        1.3239  113.20  180.00  122.08  1.0904
IC N9   C8   N7    C5        1.3784  113.20   -0.01  105.13  1.3944
IC C4   N7   *C5   C6        1.3989  109.06 -179.85  130.72  1.4252
IC N7   C5   C6    N1        1.3944  130.72  179.17  113.04  1.4333
IC N1   C5   *C6   O6        1.4333  113.04 -179.58  125.40  1.2389
IC C5   C6   N1    C2        1.4252  113.04    0.59  125.52  1.4017
IC C2   C6   *N1   C12       1.4017  125.52 -179.21  129.23  1.3810
IC C6   N1   C2    N2        1.4333  125.52 -179.30  111.88  1.3391
IC N2   N1   *C2   N3        1.3391  111.88  179.81  119.01  1.3679
IC C4   C2   *N3   H3        1.3821  117.80 -175.69  116.80  1.0050
IC C6   N1   C12   C11       1.4333  129.23  179.32  107.21  1.3613
IC C11  N1   *C12  H12       1.3613  107.21 -179.99  123.27  1.0828
IC N2   C12  *C11  C10       1.3845  111.20  179.98  130.33  1.4829
```

```
IC C12  C11  C10   H101      1.3613  130.33 -120.10  109.35  1.1094
IC H101 C11  *C10  H102      1.1094  109.35  120.31  110.31  1.1093
IC H101 C11  *C10  H103      1.1094  109.35 -119.36  109.36  1.1094
IC C4   N9   CM    HM1       1.3790  126.10  -60.06  110.61  1.1127
IC HM1  N9   *CM   HM2       1.1127  110.61  119.91  110.66  1.1120
IC HM1  N9   *CM   HM3       1.1127  110.61 -120.01  111.70  1.1129

RESI BIMG           0.00 ! wyosine               12/19, yxu
GROUP
ATOM N9     NG2R51  0.02 !                    O6
ATOM C8     CG2R53  0.38 !                    ||
ATOM H8     HGR52   0.08 !             H12    C6
ATOM N7     NG2R50 -0.68 !  H101        |    /  \
ATOM C5     CG2RC0  0.01 !      \     //C12-N1   C5--N7\\
ATOM C6     CG2R63  0.69 ! H102-C10-C11    |    ||     C8-H8
ATOM O6     OG2D4  -0.51 !      /      \N2==C2   C4--N9/
ATOM N1     NG2RC0 -0.10 !  H103             \  /      \
ATOM C2     CG2RC0  0.45 !                    N3        \
ATOM N2     NG2R50 -0.69 !                    |          \
ATOM N3     NG2R61 -0.34 !                   C3           \
ATOM C4     CG2RC0  0.30 !                 /  |  \          \
ATOM C12    CG2R51 -0.43 !             H31  H32  H33         \
ATOM H12    HGR52   0.21 !                                    \
ATOM C11    CG2R51  0.41
GROUP
ATOM C3     CG331  -0.04
ATOM H31    HGA3    0.09
ATOM H32    HGA3    0.09
ATOM H33    HGA3    0.09
GROUP
ATOM C10    CG331  -0.30
ATOM H101   HGA3    0.09
ATOM H102   HGA3    0.09
ATOM H103   HGA3    0.09
GROUP
ATOM CM     CG331  -0.27
ATOM HM1    HGA3    0.09
ATOM HM2    HGA3    0.09
ATOM HM3    HGA3    0.09
BOND CM   HM1    CM   HM2   CM   HM3
BOND N9   C8   N9   C4   C8   N7   C8   H8
BOND N7   C5   C5   C6   C5   C4   C6   O6
BOND C6   N1   N1   C2   N1   C12  C2   N2
BOND C2   N3   N2   C11  N3   C4   N3   C3
BOND C12  C11  C12  H12  C11  C10  C10  H101
BOND C10  H102 C10  H103 C3   H31  C3   H32
BOND C3   H33
BOND CM   N9
IMPR C6     C5     N1     O6
ACCE O6   C6
ACCE N2
ACCE N7
IC C8   C4   *N9   CM        1.3779  105.84 -179.89  127.40  1.4704
IC C4   N9   C8    N7        1.3904  105.84    0.54  113.34  1.3209
IC N7   N9   *C8   H8        1.3209  113.34  179.67  122.04  1.0915
IC N9   C8   N7    C5        1.3779  113.34    0.47  105.16  1.3934
IC C4   N7   *C5   C6        1.4031  109.53  177.41  130.14  1.4204
IC N7   C5   C6    N1        1.3934  130.14 -170.13  112.88  1.4260
IC N1   C5   *C6   O6        1.4260  112.88  175.72  125.44  1.2387
IC C5   C6   N1    C2        1.4204  112.88   -4.69  125.74  1.4031
IC C2   C6   *N1   C12       1.4031  125.74  173.93  128.37  1.3809
IC C6   N1   C2    N2        1.4260  125.74  176.06  110.94  1.3515
IC N2   N1   *C2   N3        1.3515  110.94  175.84  119.04  1.3922
```

```
IC C6   N1   C12   C11       1.4260  128.37 -175.44  107.39  1.3593
IC C11  N1   *C12  H12       1.3593  107.39 -178.87  123.14  1.0833
IC C4   C2   *N3   C3        1.3997  114.86 -159.30  114.01  1.4735
IC C2   N3   C3    H31       1.3922  114.01  172.63  113.42  1.1106
IC H31  N3   *C3   H32       1.1106  113.42  120.78  110.73  1.1166
IC H31  N3   *C3   H33       1.1106  113.42 -120.63  111.15  1.1156
IC N2   C12  *C11  C10       1.3823  111.08 -179.89  129.99  1.4811
IC C12  C11  C10   H101      1.3593  129.99  118.91  109.52  1.1100
IC H101 C11  *C10  H102      1.1100  109.52  119.72  109.49  1.1097
IC H101 C11  *C10  H103      1.1100  109.52 -120.40  110.02  1.1086
IC C4   N9   CM    HM1       1.3904  127.40   73.92  110.97  1.1117
IC HM1  N9   *CM   HM2       1.1117  110.97  120.18  111.84  1.1123
IC HM1  N9   *CM   HM3       1.1117  110.97 -120.10  110.24  1.1126

RESI BIWG           0.00 ! isowyosine, yxu
GROUP
ATOM N9     NG2R51  0.02 !         H132 H133 O6
ATOM C8     CG2R53  0.37 !            \   /   ||
ATOM H8     HGR52   0.09 !       H131-C13    C6
ATOM N7     NG2R50 -0.67 !  H101       |    /  \
ATOM C5     CG2RC0  0.00 !     \     //C12-N1   C5--N7\\
ATOM C6     CG2R63  0.69 ! H102-C10-C11    |    ||     C8-H8
ATOM O6     OG2D4  -0.50 !     /      \N2==C2   C4--N9/
ATOM N1     NG2RC0 -0.11 !  H103            \  /      \
ATOM C2     CG2RC0  0.44 !                   N3        \
ATOM N2     NG2R50 -0.70 !                   |          \
ATOM N3     NG2R61 -0.42 !                   H3          \
ATOM H3     HGP1    0.34 !                                \
ATOM C4     CG2RC0  0.29
ATOM C12    CG2R51 -0.25
ATOM C11    CG2R51  0.40
GROUP
ATOM C10    CG331  -0.30
ATOM H101   HGA3    0.09
ATOM H102   HGA3    0.09
ATOM H103   HGA3    0.09
GROUP
ATOM C13    CG331  -0.23
ATOM H131   HGA3    0.09
ATOM H132   HGA3    0.09
ATOM H133   HGA3    0.09
GROUP
ATOM CM     CG331  -0.27
ATOM HM1    HGA3    0.09
ATOM HM2    HGA3    0.09
ATOM HM3    HGA3    0.09
BOND CM   HM1    CM   HM2   CM   HM3
BOND N9   C8   N9   C4   C8   N7   C8   H8
BOND N7   C5   C5   C6   C5   C4   C6   O6
BOND C6   N1   N1   C2   N1   C12  C2   N2
BOND C2   N3   N2   C11  N3   C4   N3   H3
BOND C10  C11  C10  H101 C10  H102 C10  H103
BOND C11  C12  C12  C13  C13  H131 C13  H132
BOND C13  H133
BOND CM   N9
IMPR C6     C5     N1     O6
DONO H3   N3
ACCE O6   C6
ACCE N2
ACCE N7
IC C8   C4   *N9   CM        1.3785  105.70  179.49  126.15  1.4697
IC C4   N9   C8    N7        1.3796  105.70   -0.16  113.17  1.3234
IC N7   N9   *C8   H8        1.3234  113.17 -179.89  122.13  1.0911
```

```
IC N9   C8   N7    C5        1.3785  113.17    0.01  105.14  1.3948
IC C4   N7   *C5   C6        1.3984  109.09 -178.60  130.66  1.4256
IC N7   C5   C6    N1        1.3948  130.66  173.42  113.03  1.4353
IC N1   C5   *C6   O6        1.4353  113.03 -177.39  124.20  1.2389
IC C5   C6   N1    C12       1.4256  113.03 -169.48  129.66  1.3823
IC C12  C6   *N1   C2        1.3823  129.66  174.60  124.76  1.4028
IC C6   N1   C2    N2        1.4353  124.76 -175.18  112.56  1.3339
IC N2   N1   *C2   N3        1.3339  112.56  177.56  119.65  1.3682
IC C4   C2   *N3   H3        1.3806  116.94 -157.50  114.86  1.0060
IC N1   C2   N2    C11       1.4028  112.56   -0.67  104.01  1.3866
IC C12  N2   *C11  C10       1.3753  111.59  178.99  116.85  1.4892
IC N2   C11  C10   H101      1.3866  116.85  -59.98  109.37  1.1100
IC H101 C11  *C10  H102      1.1100  109.37  119.30  109.42  1.1099
IC H101 C11  *C10  H103      1.1100  109.37 -120.30  110.64  1.1076
IC C11  N1   *C12  C13       1.3753  106.42 -179.22  125.06  1.5153
IC N1   C12  C13   H131      1.3823  125.06  179.66  111.53  1.1111
IC H131 C12  *C13  H132      1.1111  111.53  120.53  110.02  1.1113
IC H131 C12  *C13  H133      1.1111  111.53 -120.34  109.98  1.1122
IC C4   N9   CM    HM1       1.3796  126.15  -61.05  110.62  1.1134
IC HM1  N9   *CM   HM2       1.1134  110.62  119.97  110.65  1.1124
IC HM1  N9   *CM   HM3       1.1134  110.62 -120.05  111.71  1.1124

RESI BMWG           0.00 ! methylwyosine, yxu
GROUP
ATOM N9     NG2R51  0.02 !         H132 H133 O6
ATOM C8     CG2R53  0.38 !            \   /   ||
ATOM H8     HGR52   0.08 !       H131-C13    C6
ATOM N7     NG2R50 -0.68 !  H101       |    /  \
ATOM C5     CG2RC0  0.01 !     \     //C12-N1   C5--N7\\
ATOM C6     CG2R63  0.69 ! H102-C10-C11    |    ||     C8-H8
ATOM O6     OG2D4  -0.51 !     /      \N2==C2   C4--N9/
ATOM N1     NG2RC0 -0.10 !  H103            \  /      \
ATOM C2     CG2RC0  0.45 !                   N3        \
ATOM N2     NG2R50 -0.69 !                   |          \
ATOM N3     NG2R61 -0.34 !                  C3           \
ATOM C4     CG2RC0  0.30 !                /  |  \          \
ATOM C12    CG2R51 -0.26 !            H31  H32  H33         \
ATOM C11    CG2R51  0.41 !                                   \
GROUP
ATOM C3     CG331  -0.04
ATOM H31    HGA3    0.09
ATOM H32    HGA3    0.09
ATOM H33    HGA3    0.09
GROUP
ATOM C10    CG331  -0.30
ATOM H101   HGA3    0.09
ATOM H102   HGA3    0.09
ATOM H103   HGA3    0.09
GROUP
ATOM C13    CG331  -0.23
ATOM H131   HGA3    0.09
ATOM H132   HGA3    0.09
ATOM H133   HGA3    0.09
GROUP
ATOM CM     CG331  -0.27
ATOM HM1    HGA3    0.09
ATOM HM2    HGA3    0.09
ATOM HM3    HGA3    0.09
BOND CM   HM1    CM   HM2   CM   HM3
BOND N9   C8   N9   C4   C8   N7   C8   H8
BOND N7   C5   C5   C6   C5   C4   C6   O6
BOND C6   N1   N1   C2   N1   C12  C2   N2
BOND C2   N3   N2   C11  N3   C4   N3   C3
```

```
BOND C12  C11  C12  C13  C11  C10  C10  H101
BOND C10  H102 C10  H103 C3   H31  C3   H32
BOND C3   H33  C13  H131 C13  H132 C13  H133
BOND CM   N9
IMPR C6     C5     N1     O6
ACCE O6   C6
ACCE N2
ACCE N7
IC C8   C4   *N9   CM        1.3771  105.76 -179.80  127.63  1.4717
IC C4   N9   C8    N7        1.3909  105.76    0.48  113.33  1.3210
IC N7   N9   *C8   H8        1.3210  113.33  179.80  121.98  1.0904
IC N9   C8   N7    C5        1.3771  113.33    0.21  105.20  1.3943
IC C4   N7   *C5   C6        1.4006  109.43  177.57  130.08  1.4204
IC N7   C5   C6    N1        1.3943  130.08 -169.59  113.10  1.4304
IC N1   C5   *C6   O6        1.4304  113.10  176.06  124.09  1.2397
IC C5   C6   N1    C2        1.4204  113.10   -6.83  124.89  1.4062
IC C2   C6   *N1   C12       1.4062  124.89  174.83  129.19  1.3840
IC C6   N1   C2    N2        1.4304  124.89  176.61  111.58  1.3468
IC N2   N1   *C2   N3        1.3468  111.58  177.86  119.52  1.3909
IC N1   C2   N2    C11       1.4062  111.58   -0.61  104.52  1.3827
IC C4   C2   *N3   C3        1.3960  115.10 -166.03  117.64  1.4743
IC C2   N3   C3    H31       1.3909  117.64  134.24  111.86  1.1128
IC H31  N3   *C3   H32       1.1128  111.86  120.78  110.83  1.1139
IC H31  N3   *C3   H33       1.1128  111.86 -119.79  112.29  1.1157
IC C12  N2   *C11  C10       1.3739  111.54 -179.59  116.74  1.4890
IC N2   C11  C10   H101      1.3827  116.74  -59.15  109.43  1.1097
IC H101 C11  *C10  H102      1.1097  109.43  119.32  109.36  1.1093
IC H101 C11  *C10  H103      1.1097  109.43 -120.34  110.63  1.1088
IC C11  N1   *C12  C13       1.3739  106.59  179.80  125.17  1.5158
IC N1   C12  C13   H131      1.3840  125.17  179.92  111.56  1.1106
IC H131 C12  *C13  H132      1.1106  111.56  120.45  109.97  1.1124
IC H131 C12  *C13  H133      1.1106  111.56 -120.48  110.01  1.1125
IC C4   N9   CM    HM1       1.3909  127.63  -52.24  110.38  1.1124
IC HM1  N9   *CM   HM2       1.1124  110.38  120.11  110.86  1.1111
IC HM1  N9   *CM   HM3       1.1124  110.38 -119.77  111.79  1.1133

RESI BYYG           0.00 ! wybutosine, WBG, YG yxu
GROUP
ATOM N9     NG2R51  0.02 !                 H192
ATOM C8     CG2R53  0.38 !                  |
ATOM H8     HGR52   0.08 !             H191-C19-H193
ATOM N7     NG2R50 -0.68 !                    \
ATOM C5     CG2RC0  0.01 !                    O18
ATOM C6     CG2R63  0.69 !                   /
ATOM O6     OG2D4  -0.51 !    H131 H141 O17=C16  H20  O22 (cis)
ATOM N1     NG2RC0 -0.10 !      |    |      |    |    ||
ATOM C2     CG2RC0  0.45 !  *---C13--C14----C15--N20--C21       H241
ATOM N2     NG2R50 -0.69 !      |    |      |            \     /
ATOM N3     NG2R61 -0.34 !    H132  H142    H15           O23-C24-H242
ATOM C4     CG2RC0  0.30 !                                     \
ATOM C12    CG2R51 -0.26 !                                      H243
ATOM C11    CG2R51  0.41
GROUP                    !                   O6
ATOM C3     CG331  -0.04 !             *     ||
ATOM H31    HGA3    0.09 !             |     C6
ATOM H32    HGA3    0.09 !  H101       |    /  \
ATOM H33    HGA3    0.09 !     \     //C12-N1   C5--N7\\
GROUP                    ! H102-C10-C11    |    ||     C8-H8
ATOM C10    CG331  -0.30 !     /      \N2==C2   C4--N9/
ATOM H101   HGA3    0.09 !  H103            \  /      \
ATOM H102   HGA3    0.09 !                   N3        \
ATOM H103   HGA3    0.09 !                   |          \
GROUP                    !                   C3          \
```

```
ATOM C13    CG321  -0.14 !                  / |  \          \
ATOM H131   HGA2    0.09 !              H31  H32  H33        \
ATOM H132   HGA2    0.09 !                                    \
GROUP
ATOM C14    CG321  -0.18
ATOM H141   HGA2    0.09
ATOM H142   HGA2    0.09
GROUP
ATOM C15    CG311   0.10
ATOM H15    HGA1    0.09
ATOM N20    NG2S1  -0.38
ATOM H20    HGP1    0.32
ATOM C21    CG2O6   0.20
ATOM O22    OG2D1  -0.39
ATOM O23    OG302  -0.32
ATOM C24    CG331   0.07
ATOM H241   HGA3    0.09
ATOM H242   HGA3    0.09
ATOM H243   HGA3    0.09
GROUP
ATOM C16    CG2O2   0.90
ATOM O17    OG2D1  -0.63
ATOM O18    OG302  -0.49
ATOM C19    CG331  -0.01
ATOM H191   HGA3    0.09
ATOM H192   HGA3    0.09
ATOM H193   HGA3    0.09
GROUP
ATOM CM     CG331  -0.27
ATOM HM1    HGA3    0.09
ATOM HM2    HGA3    0.09
ATOM HM3    HGA3    0.09
BOND CM   HM1    CM   HM2   CM   HM3
BOND N9   C8   N9   C4   C8   H8   C8   N7
BOND N7   C5   C5   C6   C5   C4   C6   O6
BOND C6   N1   N1   C2   N1   C12  C2   N2
BOND C2   N3   N2   C11  N3   C4   N3   C3
BOND C12  C11  C12  C13  C11  C10  C3   H31
BOND C3   H32  C3   H33  C10  H101 C10  H102
BOND C10  H103 C13  H131 C13  H132 C13  C14
BOND C14  H141 C14  H142 C14  C15  C15  H15
BOND C15  N20  C15  C16  N20  H20  N20  C21
BOND C21  O22  C21  O23  O23  C24  C24  H241
BOND C24  H242 C24  H243 C16  O17  C16  O18
BOND O18  C19  C19  H191 C19  H192 C19  H193
BOND CM   N9
IMPR C6     C5     N1     O6     C16    C15    O17    O18    C21    N20    O22    O23
DONO H20  N20
ACCE O6   C6
ACCE N2
ACCE N7
ACCE O17  C16
ACCE O18
ACCE O22  C21
ACCE O23
IC C8   C4   *N9   CM       1.3760  105.78  179.76  128.19  1.4725
IC C4   N9   C8    N7       1.3949  105.78   -0.34  113.38  1.3199
IC N7   N9   *C8   H8       1.3199  113.38 -179.96  122.05  1.0908
IC N9   C8   N7    C5       1.3760  113.38   -0.19  105.28  1.3935
IC C4   N7   *C5   C6       1.4021  109.49 -178.60  129.85  1.4189
IC N7   C5   C6    N1       1.3935  129.85  173.49  113.51  1.4301
IC N1   C5   *C6   O6       1.4301  113.51 -177.60  123.82  1.2388
IC C5   C6   N1    C12      1.4189  113.51 -172.75  128.93  1.3901
```

```
IC C12  C6   *N1   C2        1.3901  128.93  177.64  124.86  1.4029
IC C6   N1   C2    N2        1.4301  124.86 -178.85  111.31  1.3434
IC N2   N1   *C2   N3        1.3434  111.31 -178.56  120.01  1.3938
IC N1   C2   N2    C11       1.4029  111.31   -0.11  105.12  1.3818
IC C4   C2   *N3   C3        1.3994  115.60  171.93  117.90  1.4745
IC C2   N3   C3    H31       1.3938  117.90  110.16  110.94  1.1139
IC H31  N3   *C3   H32       1.1139  110.94  120.68  111.54  1.1127
IC H31  N3   *C3   H33       1.1139  110.94 -119.70  112.92  1.1142
IC C12  N2   *C11  C10       1.3800  111.29  178.86  114.34  1.4945
IC N2   C11  C10   H101      1.3818  114.34  -81.38  109.37  1.1092
IC H101 C11  *C10  H102      1.1092  109.37  119.39  109.82  1.1105
IC H101 C11  *C10  H103      1.1092  109.37 -121.32  111.09  1.1057
IC C11  N1   *C12  C13       1.3800  106.10 -177.01  122.76  1.5252
IC N1   C12  C13   C14       1.3901  122.76  178.03  118.48  1.5425
IC C14  C12  *C13  H131      1.5425  118.48 -122.07  107.88  1.1159
IC H131 C12  *C13  H132      1.1159  107.88 -114.90  107.90  1.1142
IC C12  C13  C14   C15       1.5252  118.48 -173.53  113.29  1.5510
IC C15  C13  *C14  H141      1.5510  113.29  120.21  109.55  1.1126
IC H141 C13  *C14  H142      1.1126  109.55  118.82  108.93  1.1125
IC C13  C14  C15   N20       1.5425  113.29 -179.76  110.49  1.4490
IC N20  C14  *C15  C16       1.4490  110.49  124.95  109.55  1.5607
IC N20  C14  *C15  H15       1.4490  110.49 -117.57  108.26  1.1137
IC C14  C15  N20   C21       1.5510  110.49 -174.85  120.63  1.3586
IC C21  C15  *N20  H20       1.3586  120.63 -156.01  117.94  0.9947
IC C15  N20  C21   O23       1.4490  120.63 -177.22  109.61  1.3462
IC O23  N20  *C21  O22       1.3462  109.61  179.32  125.56  1.2254
IC N20  C21  O23   C24       1.3586  109.61  178.84  113.41  1.4369
IC C21  O23  C24   H241      1.3462  113.41  179.41  109.60  1.1097
IC H241 O23  *C24  H242      1.1097  109.60  119.44  110.75  1.1130
IC H241 O23  *C24  H243      1.1097  109.60 -119.50  110.82  1.1139
IC C14  C15  C16   O18       1.5510  109.55  107.92  110.21  1.3400
IC O18  C15  *C16  O17       1.3400  110.21 -176.32  125.14  1.2208
IC C15  C16  O18   C19       1.5607  110.21 -175.97  112.70  1.4370
IC C16  O18  C19   H191      1.3400  112.70 -179.94  109.73  1.1126
IC H191 O18  *C19  H192      1.1126  109.73  119.47  110.83  1.1133
IC H191 O18  *C19  H193      1.1126  109.73 -119.32  110.83  1.1131
IC C4   N9   CM    HM1       1.3949  128.19  173.73  111.79  1.1132
IC HM1  N9   *CM   HM2       1.1132  111.79  119.85  110.83  1.1123
IC HM1  N9   *CM   HM3       1.1132  111.79 -119.78  110.61  1.1117

RESI BHWG           0.00 ! hydroxywybutosine, yxu
GROUP
ATOM N9     NG2R51  0.02 !                 H192
ATOM C8     CG2R53  0.38 !                  |
ATOM H8     HGR52   0.08 !             H191-C19-H193
ATOM N7     NG2R50 -0.68 !                    \
ATOM C5     CG2RC0  0.01 !                    O18
ATOM C6     CG2R63  0.69 !                   /
ATOM O6     OG2D4  -0.51 !    H131  H14 O17=C16  H20  O22 (cis)
ATOM N1     NG2RC0 -0.10 !      |    |       |    |    ||
ATOM C2     CG2RC0  0.45 !  *---C13--C14----C15--N20--C21       H241
ATOM N2     NG2R50 -0.69 !      |    |       |            \     /
ATOM N3     NG2R61 -0.34 !    H132  O14     H15           O23-C24-H242
ATOM C4     CG2RC0  0.30 !            \                         \
ATOM C12    CG2R51 -0.26 !             H14O                     H243
ATOM C11    CG2R51  0.41
GROUP
ATOM C3     CG331  -0.04 !                    O6
ATOM H31    HGA3    0.09 !              *     ||
ATOM H32    HGA3    0.09 !              |     C6
ATOM H33    HGA3    0.09 !   H101       |    /  \
GROUP                    !      \     //C12-N1   C5--N7\\
ATOM C10    CG331  -0.30 !  H102-C10-C11    |    ||     C8-H8
```

```
ATOM H101   HGA3    0.09 !      /       \N2==C2   C4--N9/
ATOM H102   HGA3    0.09 !   H103             \  /       \
ATOM H103   HGA3    0.09 !                     N3         \
GROUP                    !                     |           \
ATOM C13    CG321  -0.14 !                     C3            \
ATOM H131   HGA2    0.09 !                   / |  \            \
ATOM H132   HGA2    0.09 !               H31  H32  H33          \
GROUP                    !                                        \
ATOM C14    CG311   0.14
ATOM H14    HGA1    0.09
ATOM O14    OG311  -0.65
ATOM H14O   HGP1    0.42
GROUP
ATOM C15    CG311   0.10
ATOM H15    HGA1    0.09
ATOM N20    NG2S1  -0.38
ATOM H20    HGP1    0.32
ATOM C21    CG2O6   0.20
ATOM O22    OG2D1  -0.39
ATOM O23    OG302  -0.32
ATOM C24    CG331   0.07
ATOM H241   HGA3    0.09
ATOM H242   HGA3    0.09
ATOM H243   HGA3    0.09
GROUP
ATOM C16    CG2O2   0.90
ATOM O17    OG2D1  -0.63
ATOM O18    OG302  -0.49
ATOM C19    CG331  -0.01
ATOM H191   HGA3    0.09
ATOM H192   HGA3    0.09
ATOM H193   HGA3    0.09
GROUP
ATOM CM     CG331  -0.27
ATOM HM1    HGA3    0.09
ATOM HM2    HGA3    0.09
ATOM HM3    HGA3    0.09
BOND CM   HM1    CM   HM2   CM   HM3
BOND N9   C8   N9   C4   C8   H8
BOND C8   N7   N7   C5   C5   C6   C5   C4
BOND C6   O6   C6   N1   N1   C2   N1   C12
BOND C2   N2   C2   N3   N2   C11  N3   C4
BOND N3   C3   C12  C11  C12  C13  C11  C10
BOND C3   H31  C3   H32  C3   H33  C10  H101
BOND C10  H102 C10  H103 C13  H131 C13  H132
BOND C13  C14  C14  H14  C14  O14  C14  C15
BOND O14  H14O C15  H15  C15  N20  C15  C16
BOND N20  H20  N20  C21  C21  O22  C21  O23
BOND O23  C24  C24  H241 C24  H242 C24  H243
BOND C16  O17  C16  O18  O18  C19  C19  H191
BOND C19  H192 C19  H193
BOND CM   N9
IMPR C6     C5     N1     O6     C16    C15    O17    O18    C21    N20    O22    O23
DONO H20  N20
DONO H14O O14
ACCE O6   C6
ACCE N2
ACCE N7
ACCE O14
ACCE O17  C16
ACCE O18
ACCE O22  C21
ACCE O23
```

```
IC C8   C4   *N9   CM        1.3756  105.72  179.40  128.44  1.4724
IC C4   N9   C8    N7        1.3979  105.72   -0.55  113.47  1.3182
IC N7   N9   *C8   H8        1.3182  113.47 -179.98  121.97  1.0914
IC N9   C8   N7    C5        1.3756  113.47   -0.04  105.31  1.3928
IC C4   N7   *C5   C6        1.4020  109.56 -178.82  129.73  1.4188
IC N7   C5   C6    N1        1.3928  129.73  175.39  113.63  1.4287
IC N1   C5   *C6   O6        1.4287  113.63 -179.18  123.70  1.2401
IC C5   C6   N1    C12       1.4188  113.63 -175.16  128.77  1.3890
IC C12  C6   *N1   C2        1.3890  128.77 -179.73  124.94  1.4038
IC C6   N1   C2    N2        1.4287  124.94  178.16  111.14  1.3443
IC N2   N1   *C2   N3        1.3443  111.14 -179.21  119.98  1.3953
IC N1   C2   N2    C11       1.4038  111.14    0.77  105.14  1.3831
IC C4   C2   *N3   C3        1.3999  115.88  175.07  118.22  1.4750
IC C2   N3   C3    H31       1.3953  118.22  112.96  110.96  1.1142
IC H31  N3   *C3   H32       1.1142  110.96  120.48  111.36  1.1131
IC H31  N3   *C3   H33       1.1142  110.96 -119.77  113.12  1.1139
IC C12  N2   *C11  C10       1.3789  111.28 -179.23  114.46  1.4913
IC N2   C11  C10   H101      1.3831  114.46  -76.40  109.03  1.1099
IC H101 C11  *C10  H102      1.1099  109.03  119.61  109.98  1.1098
IC H101 C11  *C10  H103      1.1099  109.03 -120.31  110.92  1.1039
IC C11  N1   *C12  C13       1.3789  106.11 -178.51  122.38  1.5369
IC N1   C12  C13   C14       1.3890  122.38  171.88  122.00  1.5631
IC C14  C12  *C13  H131      1.5631  122.00 -122.07  107.39  1.1135
IC H131 C12  *C13  H132      1.1135  107.39 -113.60  106.03  1.1105
IC C12  C13  C14   C15       1.5369  122.00 -177.50  112.07  1.5208
IC C15  C13  *C14  O14       1.5208  112.07  120.31  109.55  1.4263
IC O14  C13  *C14  H14       1.4263  109.55  119.73  108.29  1.1140
IC C13  C14  O14   H14O      1.5631  109.55 -176.44  107.57  0.9622
IC C13  C14  C15   N20       1.5631  112.07 -173.26  112.63  1.4598
IC N20  C14  *C15  C16       1.4598  112.63  122.61  106.83  1.5385
IC N20  C14  *C15  H15       1.4598  112.63 -120.74  108.96  1.1157
IC C14  C15  N20   C21       1.5208  112.63 -168.91  119.95  1.3593
IC C21  C15  *N20  H20       1.3593  119.95 -148.40  117.36  0.9959
IC C15  N20  C21   O23       1.4598  119.95 -176.03  109.51  1.3461
IC O23  N20  *C21  O22       1.3461  109.51  178.91  125.67  1.2243
IC N20  C21  O23   C24       1.3593  109.51  179.60  113.41  1.4380
IC C21  O23  C24   H241      1.3461  113.41 -179.67  109.58  1.1113
IC H241 O23  *C24  H242      1.1113  109.58  119.46  110.89  1.1128
IC H241 O23  *C24  H243      1.1113  109.58 -119.37  110.79  1.1134
IC C14  C15  C16   O18       1.5208  106.83  111.40  110.82  1.3358
IC O18  C15  *C16  O17       1.3358  110.82 -176.76  124.30  1.2213
IC C15  C16  O18   C19       1.5385  110.82 -175.50  113.09  1.4365
IC C16  O18  C19   H191      1.3358  113.09  178.94  109.60  1.1125
IC H191 O18  *C19  H192      1.1125  109.60  119.59  110.77  1.1133
IC H191 O18  *C19  H193      1.1125  109.60 -119.26  110.76  1.1137
IC C4   N9   CM    HM1       1.3979  128.44   54.80  110.62  1.1120
IC HM1  N9   *CM   HM2       1.1120  110.62  119.57  111.64  1.1132
IC HM1  N9   *CM   HM3       1.1120  110.62 -120.53  110.88  1.1115

RESI BPBG           0.00 ! peroxywybutosine, yxu
GROUP
ATOM N9     NG2R51  0.02 !               H192
ATOM C8     CG2R53  0.38 !                |
ATOM H8     HGR52   0.08 !           H191-C19-H193
ATOM N7     NG2R50 -0.68 !                  \
ATOM C5     CG2RC0  0.01 !                  O18
ATOM C6     CG2R63  0.69 !                 /
ATOM O6     OG2D4  -0.51 !   H131  H14 O17=C17  H20  O22 (cis)
ATOM N1     NG2RC0 -0.10 !     |     |      |     |    ||
ATOM C2     CG2RC0  0.45 ! *---C13--C14----C16--N20--C21       H241
ATOM N2     NG2R50 -0.69 !     |     |      |            \      /
ATOM N3     NG2R61 -0.34 !   H132  O14     H16           O23-C24-H242
ATOM C4     CG2RC0  0.30 !            \                          \
```

```
ATOM C12    CG2R51 -0.26 !            O15-H15O                 H243
ATOM C11    CG2R51  0.41
GROUP
ATOM C3     CG331  -0.04 !                      O6
ATOM H31    HGA3    0.09 !              *       ||
ATOM H32    HGA3    0.09 !              |       C6
ATOM H33    HGA3    0.09 !  H101        |     /  \
GROUP                    !     \      //C12-N1    C5--N7\\
ATOM C10    CG331  -0.30 ! H102-C10-C11     |     ||      C8-H8
ATOM H101   HGA3    0.09 !     /       \N2==C2    C4--N9/
ATOM H102   HGA3    0.09 !  H103             \  /       \
ATOM H103   HGA3    0.09 !                     N3         \
GROUP                    !                     |           \
ATOM C13    CG321  -0.14 !                     C3           \
ATOM H131   HGA2    0.09 !                   / |  \           \
ATOM H132   HGA2    0.09 !               H31  H32  H33         \
GROUP                    !                                      \
ATOM C14    CG311   0.17
ATOM H14    HGA1    0.09
ATOM O14    OG301  -0.25
ATOM O15    OG311  -0.44
ATOM H15O   HGP1    0.43
GROUP
ATOM C16    CG311   0.10
ATOM H16    HGA1    0.09
ATOM N20    NG2S1  -0.38
ATOM H20    HGP1    0.32
ATOM C21    CG2O6   0.20
ATOM O22    OG2D1  -0.39
ATOM O23    OG302  -0.32
ATOM C24    CG331   0.07
ATOM H241   HGA3    0.09
ATOM H242   HGA3    0.09
ATOM H243   HGA3    0.09
GROUP
ATOM C17    CG2O2   0.90
ATOM O17    OG2D1  -0.63
ATOM O18    OG302  -0.49
ATOM C19    CG331  -0.01
ATOM H191   HGA3    0.09
ATOM H192   HGA3    0.09
ATOM H193   HGA3    0.09
GROUP
ATOM CM     CG331  -0.27
ATOM HM1    HGA3    0.09
ATOM HM2    HGA3    0.09
ATOM HM3    HGA3    0.09
BOND CM   HM1    CM   HM2   CM   HM3
BOND N9   C8   N9   C4   C8   H8   C8   N7
BOND N7   C5   C5   C6   C5   C4   C6   O6
BOND C6   N1   N1   C2   N1   C12  C2   N2
BOND C2   N3   N2   C11  N3   C4   N3   C3
BOND C12  C11  C12  C13  C11  C10  C3   H31
BOND C3   H32  C3   H33  C10  H101 C10  H102
BOND C10  H103 C13  H131 C13  H132 C13  C14
BOND O15  H15O O15  O14  O14  C14  C14  H14
BOND C14  C16  C16  H16  C16  N20  C16  C17
BOND N20  H20  N20  C21  C21  O22  C21  O23
BOND O23  C24  C24  H241 C24  H242 C24  H243
BOND C17  O17  C17  O18  O18  C19  C19  H191
BOND C19  H192 C19  H193
BOND CM   N9
IMPR C6     C5     N1     O6     C17    C16    O17    O18    C21    N20    O22    O23
```

```
DONO H20  N20
DONO H15O O15
ACCE O6   C6
ACCE N2
ACCE N7
ACCE O14
ACCE O15
ACCE O17  C16
ACCE O18
ACCE O22  C21
ACCE O23
IC C8   C4   *N9   CM        1.3752  105.79  179.92  128.54  1.4721
IC C4   N9   C8    N7        1.3981  105.79    0.07  113.43  1.3189
IC N7   N9   *C8   H8        1.3189  113.43  179.94  122.03  1.0901
IC N9   C8   N7    C5        1.3752  113.43    0.04  105.29  1.3929
IC C4   N7   *C5   C6        1.4021  109.62 -179.96  129.54  1.4187
IC N7   C5   C6    N1        1.3929  129.54 -179.57  113.63  1.4304
IC N1   C5   *C6   O6        1.4304  113.63  179.09  123.81  1.2383
IC C5   C6   N1    C12       1.4187  113.63  179.04  128.80  1.3900
IC C12  C6   *N1   C2        1.3900  128.80 -177.58  124.89  1.4042
IC C6   N1   C2    N2        1.4304  124.89  176.82  111.10  1.3443
IC N2   N1   *C2   N3        1.3443  111.10  179.33  120.12  1.3948
IC N1   C2   N2    C11       1.4042  111.10    0.21  105.24  1.3817
IC C4   C2   *N3   C3        1.4013  115.89 -176.36  118.00  1.4752
IC C2   N3   C3    H31       1.3948  118.00  119.87  111.22  1.1131
IC H31  N3   *C3   H32       1.1131  111.22  120.60  111.24  1.1134
IC H31  N3   *C3   H33       1.1131  111.22 -119.70  113.40  1.1150
IC C12  N2   *C11  C10       1.3793  111.27 -179.71  114.30  1.4915
IC N2   C11  C10   H101      1.3817  114.30  -76.21  109.21  1.1083
IC H101 C11  *C10  H102      1.1083  109.21  119.14  109.60  1.1106
IC H101 C11  *C10  H103      1.1083  109.21 -121.44  111.03  1.1050
IC C11  N1   *C12  C13       1.3793  106.09 -179.36  122.77  1.5344
IC N1   C12  C13   C14       1.3900  122.77  168.77  120.72  1.5646
IC C14  C12  *C13  H131      1.5646  120.72 -120.80  107.38  1.1142
IC H131 C12  *C13  H132      1.1142  107.38 -114.27  106.73  1.1105
IC C12  C13  C14   C16       1.5344  120.72 -174.85  111.02  1.5258
IC C16  C13  *C14  O14       1.5258  111.02  120.62  108.60  1.4337
IC O14  C13  *C14  H14       1.4337  108.60  120.37  108.18  1.1150
IC C13  C14  O14   O15       1.5646  108.60  158.14  109.85  1.4726
IC C14  O14  O15   H15O      1.4337  109.85 -125.60   99.21  0.9639
IC C13  C14  C16   N20       1.5646  111.02 -178.07  114.33  1.4543
IC N20  C14  *C16  C17       1.4543  114.33  124.72  105.70  1.5424
IC N20  C14  *C16  H16       1.4543  114.33 -119.32  108.37  1.1128
IC C14  C16  N20   C21       1.5258  114.33  177.09  121.34  1.3599
IC C21  C16  *N20  H20       1.3599  121.34  154.41  115.30  1.0009
IC C16  N20  C21   O23       1.4543  121.34  173.50  109.66  1.3457
IC O23  N20  *C21  O22       1.3457  109.66 -179.84  125.53  1.2245
IC N20  C21  O23   C24       1.3599  109.66  178.16  113.39  1.4371
IC C21  O23  C24   H241      1.3457  113.39 -179.53  109.57  1.1104
IC H241 O23  *C24  H242      1.1104  109.57  119.45  110.73  1.1135
IC H241 O23  *C24  H243      1.1104  109.57 -119.50  110.76  1.1132
IC C14  C16  C17   O18       1.5258  105.70  112.36  110.31  1.3405
IC O18  C16  *C17  O17       1.3405  110.31 -174.44  124.96  1.2214
IC C16  C17  O18   C19       1.5424  110.31 -175.76  112.76  1.4365
IC C17  O18  C19   H191      1.3405  112.76  178.78  109.65  1.1123
IC H191 O18  *C19  H192      1.1123  109.65  119.49  110.68  1.1134
IC H191 O18  *C19  H193      1.1123  109.65 -119.32  110.91  1.1137
IC C4   N9   CM    HM1       1.3981  128.54   60.49  110.84  1.1116
IC HM1  N9   *CM   HM2       1.1116  110.84  119.66  111.81  1.1128
IC HM1  N9   *CM   HM3       1.1116  110.84 -120.55  110.68  1.1122

RESI BBUG           0.00 ! undermodified hydroxywybutosine, yxu
GROUP
```

```
ATOM N9     NG2R51  0.02 !   H131  H14   H15    O18 (-)
ATOM C8     CG2R53  0.38 !     |    |    |      /
ATOM H8     HGR52   0.08 ! *---C13--C14--C15--C17
ATOM N7     NG2R50 -0.68 !     |    |    |      \\
ATOM C5     CG2RC0  0.01 !   H132  O14   | (+)  O17
ATOM C6     CG2R63  0.69 !         /    N16
ATOM O6     OG2D4  -0.51 !      H14O   / | \
ATOM N1     NG2RC0 -0.10 !         H161 H162 H163
ATOM C2     CG2RC0  0.45
ATOM N2     NG2R50 -0.69 !                    O6
ATOM N3     NG2R61 -0.34 !              *     ||
ATOM C4     CG2RC0  0.30 !              |     C6
ATOM C12    CG2R51 -0.26 !  H101        |    /  \
ATOM C11    CG2R51  0.41 !     \     //C12-N1   C5--N7\\
GROUP                    ! H102-C10-C11    |    ||      C8-H8
ATOM C3     CG331  -0.04 !     /      \N2==C2   C4--N9/
ATOM H31    HGA3    0.09 !  H103            \\ /      \
ATOM H32    HGA3    0.09 !                    N3        \
ATOM H33    HGA3    0.09 !                    |          \
GROUP                    !                    C3          \
ATOM C10    CG331  -0.30 !                  / |  \         \
ATOM H101   HGA3    0.09 !              H31  H32  H33        \
ATOM H102   HGA3    0.09 !                                    \
ATOM H103   HGA3    0.09
GROUP
ATOM C13    CG321  -0.14
ATOM H131   HGA2    0.09
ATOM H132   HGA2    0.09
GROUP
ATOM C14    CG311   0.14
ATOM H14    HGA1    0.09
ATOM O14    OG311  -0.65
ATOM H14O   HGP1    0.42
GROUP
ATOM C15    CG314   0.17
ATOM H15    HGA1    0.11
ATOM N16    NG3P3  -0.34
ATOM H161   HGP2    0.30
ATOM H162   HGP2    0.30
ATOM H163   HGP2    0.30
ATOM C17    CG2O3   0.32
ATOM O18    OG2D2  -0.58
ATOM O17    OG2D2  -0.58
GROUP
ATOM CM     CG331  -0.27
ATOM HM1    HGA3    0.09
ATOM HM2    HGA3    0.09
ATOM HM3    HGA3    0.09
BOND CM   HM1    CM   HM2   CM   HM3
BOND N9   C8   N9   C4   C8   H8   C8   N7
BOND N7   C5   C5   C6   C5   C4   C6   O6
BOND C6   N1   N1   C2   N1   C12  C2   N2
BOND C2   N3   N2   C11  N3   C4   N3   C3
BOND C12  C11  C12  C13  C11  C10  C3   H31
BOND C3   H32  C3   H33  C10  H101 C10  H102
BOND C10  H103 C13  H131 C13  H132 C13  C14
BOND C14  H14  C14  O14  C14  C15  O14  H14O
BOND C15  H15  C15  C17  C15  N16  C17  O18
BOND C17  O17  N16  H161 N16  H162 N16  H163
BOND CM   N9
IMPR C6     C5     N1     O6      C17    O17    O18    C15
DONO H14O O14
DONO H161 N16
```

```
DONO H162 N16
DONO H162 N16
ACCE O14
ACCE O17 C17
ACCE O18 C17
ACCE O6   C6
ACCE N2
ACCE N7
IC C8   C4   *N9   CM        1.3772  105.72  179.94  127.97  1.4713
IC C4   N9   C8    N7        1.3937  105.72    0.34  113.36  1.3204
IC N7   N9   *C8   H8        1.3204  113.36  179.99  121.98  1.0903
IC N9   C8   N7    C5        1.3772  113.36    0.17  105.24  1.3934
IC C4   N7   *C5   C6        1.4010  109.51  178.81  129.84  1.4182
IC N7   C5   C6    N1        1.3934  129.84 -173.73  113.45  1.4306
IC N1   C5   *C6   O6        1.4306  113.45  176.13  123.94  1.2425
IC C5   C6   N1    C12       1.4182  113.45  172.49  128.67  1.3883
IC C12  C6   *N1   C2        1.3883  128.67 -174.48  124.86  1.4027
IC C6   N1   C2    N2        1.4306  124.86  174.35  111.29  1.3415
IC N2   N1   *C2   N3        1.3415  111.29  178.10  119.73  1.3926
IC N1   C2   N2    C11       1.4027  111.29    0.78  105.13  1.3842
IC C4   C2   *N3   C3        1.3976  115.51 -168.83  117.48  1.4750
IC C2   N3   C3    H31       1.3926  117.48  129.74  111.69  1.1125
IC H31  N3   *C3   H32       1.1125  111.69  120.68  110.97  1.1137
IC H31  N3   *C3   H33       1.1125  111.69 -119.72  112.70  1.1151
IC C12  N2   *C11  C10       1.3804  111.18 -178.50  114.15  1.4911
IC N2   C11  C10   H101      1.3842  114.15  -69.22  109.12  1.1098
IC H101 C11  *C10  H102      1.1098  109.12  119.33  109.76  1.1095
IC H101 C11  *C10  H103      1.1098  109.12 -120.16  111.37  1.1046
IC C11  N1   *C12  C13       1.3804  106.08 -179.23  122.03  1.5322
IC N1   C12  C13   C14       1.3883  122.03  171.67  122.31  1.5636
IC C14  C12  *C13  H131      1.5636  122.31 -121.64  107.10  1.1146
IC H131 C12  *C13  H132      1.1146  107.10 -114.14  105.38  1.1086
IC C12  C13  C14   C15       1.5322  122.31 -179.25  111.41  1.5411
IC C15  C13  *C14  O14       1.5411  111.41  122.13  108.80  1.4260
IC O14  C13  *C14  H14       1.4260  108.80  118.20  107.93  1.1149
IC C13  C14  O14   H14O      1.5636  108.80 -174.21  104.32  0.9776
IC C13  C14  C15   C17       1.5636  111.41 -169.27  109.00  1.5432
IC C17  C14  *C15  N16       1.5432  109.00  124.44  113.29  1.5093
IC C17  C14  *C15  H15       1.5432  109.00 -116.09  108.65  1.1127
IC C14  C15  N16   H161      1.5411  113.29 -168.71  105.76  1.0438
IC H161 C15  *N16  H162      1.0438  105.76  114.45  107.83  1.0391
IC H161 C15  *N16  H163      1.0438  105.76 -122.14  111.98  1.0361
IC C14  C15  C17   O18       1.5411  109.00  125.90  116.75  1.2582
IC O18  C15  *C17  O17       1.2582  116.75 -179.86  115.22  1.2561
IC C4   N9   CM    HM1       1.3937  127.97 -173.65  111.79  1.1127
IC HM1  N9   *CM   HM2       1.1127  111.79  119.83  110.58  1.1123
IC HM1  N9   *CM   HM3       1.1127  111.79 -119.98  110.77  1.1119

!!The fragment compounds

RESI AMPU           1.00 ! 7-deazapurin-7-yl-methyl ammonium, yxu
GROUP
ATOM N9     NG2R51 -0.37
ATOM H9     HGP1    0.34
ATOM C8     CG2R51  0.01
ATOM H8     HGR52   0.11
ATOM C7     CG2R51 -0.10
ATOM C5     CG2RC0 -0.11
ATOM C6     CG2R61  0.24
ATOM H6     HGR62   0.10
ATOM N1     NG2R62 -0.60
ATOM C2     CG2R64  0.57
ATOM H2     HGR62   0.11
```

```
ATOM N3     NG2R62 -0.57
ATOM C4     CG2RC0  0.27
GROUP
ATOM C10    CG324   0.21
ATOM H11    HGA2    0.05
ATOM H12    HGA2    0.05
ATOM N11    NG3P3  -0.30
ATOM H111   HGP2    0.33
ATOM H112   HGP2    0.33
ATOM H113   HGP2    0.33
BOND H12  C10
BOND H6   C6     C10  H11    C10  N11    C10  C7
BOND H111 N11    H112 N11    C6   N1     C6   C5
BOND N1   C2     N11  H113   C7   C5     C7   C8
BOND C5   C4     C2   H2     C2   N3     C4   N3
BOND C4   N9     C8   H8     C8   N9     N9   H9
IC C8   C4   *N9   H9       1.3862  111.41  177.43  120.52  1.0101
IC C4   N9   C8    C7       1.3523  111.41   -0.68  107.67  1.3734
IC C7   N9   *C8   H8       1.3734  107.67  179.72  122.54  1.0844
IC N9   C8   C7    C5       1.3862  107.67    1.02  105.95  1.4368
IC C5   C8   *C7   C10      1.4368  105.95 -177.16  127.38  1.4875
IC C4   C7   *C5   C6       1.3873  108.84  178.92  134.84  1.3957
IC C7   C5   C6    N1       1.4368  134.84  178.99  121.33  1.3349
IC N1   C5   *C6   H6       1.3349  121.33 -179.22  122.11  1.0839
IC C5   C6   N1    C2       1.3957  121.33    1.95  116.86  1.3768
IC C6   N1   C2    N3       1.3349  116.86   -1.34  126.11  1.3664
IC N3   N1   *C2   H2       1.3664  126.11 -179.03  117.01  1.0927
IC C8   C7   C10   N11      1.3734  127.38   99.76  109.25  1.4922
IC N11  C7   *C10  H12      1.4922  109.25  119.52  110.18  1.1017
IC H12  C7   *C10  H11      1.1017  110.18  121.07  110.83  1.1020
IC C7   C10  N11   H111     1.4875  109.25  178.11  110.51  1.0396
IC H111 C10  *N11  H112     1.0396  110.51  120.52  109.04  1.0404
IC H111 C10  *N11  H113     1.0396  110.51 -120.93  109.31  1.0401

RESI 7MIP           1.00 ! 7-deazapurin-7-yl-methyl amidinium, yxu
GROUP
ATOM C5     CG2RC0 -0.21
ATOM C6     CG2R61  0.24
ATOM H6     HGR62   0.07
ATOM N1     NG2R62 -0.60
ATOM C2     CG2R64  0.57
ATOM H2     HGR62   0.11
ATOM N3     NG2R62 -0.58
ATOM C4     CG2RC0  0.41
ATOM N9     NG2R51 -0.43
ATOM H9     HGP1    0.42
GROUP
ATOM C8     CG2R51 -0.21
ATOM H8     HGR52   0.24
ATOM C7     CG2R51  0.06
ATOM C10    CG2N2   0.61
ATOM N11    NG2P1  -0.43
ATOM H111   HGP2    0.29
ATOM H112   HGP2    0.29
ATOM N12    NG2P1  -0.43
ATOM H121   HGP2    0.29
ATOM H122   HGP2    0.29
BOND H9   N9
BOND N9   C8     N9   C4     H8   C8     C8   C7
BOND N3   C4     N3   C2     C4   C5     H2   C2
BOND C2   N1     C7   C5     C7   C10    C5   C6
BOND N1   C6     H112 N11    C10  N11    C10  N12
BOND C6   H6     N11  H111   H122 N12    N12  H121
```

```
IMPR C10    N12    N11    C7
IC C4   C7   *C5   C6        1.3866  109.35 -177.96  134.75  1.3933
IC C7   C5   C6    N1        1.4461  134.75  179.71  121.37  1.3312
IC N1   C5   *C6   H6        1.3312  121.37  179.52  122.01  1.0850
IC C5   C6   N1    C2        1.3933  121.37   -1.10  117.08  1.3745
IC C6   N1   C2    N3        1.3312  117.08    0.11  126.03  1.3645
IC N3   N1   *C2   H2        1.3645  126.03 -179.96  116.96  1.0923
IC N3   C5   *C4   N9        1.3179  126.44  179.92  106.06  1.3460
IC C5   C4   N9    C8        1.3866  106.06   -0.01  111.82  1.3915
IC C8   C4   *N9   H9        1.3915  111.82  179.70  117.87  1.0115
IC C7   N9   *C8   H8        1.3832  107.62  178.26  122.26  1.0882
IC C8   C5   *C7   C10       1.3832  105.15 -178.74  127.70  1.4530
IC C5   C7   C10   N11       1.4461  127.70 -143.03  120.07  1.3263
IC N11  C7   *C10  N12       1.3263  120.07  179.83  118.89  1.3245
IC C7   C10  N11   H112      1.4530  120.07  179.23  120.54  0.9985
IC H112 C10  *N11  H111      0.9985  120.54 -176.80  119.65  0.9998
IC C7   C10  N12   H122      1.4530  118.89  179.89  120.80  0.9983
IC H122 C10  *N12  H121      0.9983  120.80 -178.00  119.43  1.0000

RESI 2MSA           0.00 ! 2-methylthio-adenine, yxu
GROUP
ATOM N9     NG2R51 -0.36
ATOM H9     HGP1    0.34
ATOM C8     CG2R53  0.37
ATOM H8     HGR52   0.10
ATOM N7     NG2R50 -0.83
ATOM C5     CG2RC0  0.28
ATOM C6     CG2R64  0.38
ATOM N6     NG2S3  -0.63
ATOM H61    HGP4    0.34
ATOM H62    HGP4    0.34
ATOM N1     NG2R62 -0.58
ATOM C2     CG2R64  0.63
ATOM N3     NG2R62 -0.76
ATOM C4     CG2RC0  0.48
ATOM S2     SG311  -0.28
ATOM C20    CG331  -0.09
ATOM H21    HGA3    0.09
ATOM H22    HGA3    0.09
ATOM H23    HGA3    0.09
BOND N9   C8     N9   C4     C8   N7     C8   H8
BOND N7   C5     C5   C6     C5   C4     C6   N6
BOND C6   N1     N6   H61    N6   H62    N1   C2
BOND C2   N3     C2   S2     N3   C4     H9   N9
BOND S2   C20    C20  H21    C20  H22    C20  H23
IC C8   C4   *N9   H9        1.3721  107.46 -179.94  122.15  1.0012
IC C4   N9   C8    N7        1.3500  107.46    0.08  112.87  1.3243
IC N7   N9   *C8   H8        1.3243  112.87  179.91  122.62  1.0931
IC N9   C8   N7    C5        1.3721  112.87   -0.07  103.65  1.3988
IC C4   N7   *C5   C6        1.3959  110.25  179.96  133.28  1.4070
IC N7   C5   C6    N1        1.3988  133.28 -179.95  118.02  1.3665
IC N1   C5   *C6   N6        1.3665  118.02 -179.97  123.15  1.3538
IC C5   C6   N6    H61       1.4070  123.15  179.97  119.69  0.9953
IC H61  C6   *N6   H62       0.9953  119.69  179.98  120.00  0.9993
IC C5   C6   N1    C2        1.4070  118.02   -0.04  119.65  1.3671
IC C6   N1   C2    S2        1.3665  119.65  179.99  120.08  1.7512
IC S2   N1   *C2   N3        1.7512  120.08 -179.91  125.57  1.3579
IC N1   C2   S2    C20       1.3671  120.08    0.06   99.02  1.8302
IC C2   S2   C20   H21       1.7512   99.02  180.00  110.33  1.1112
IC H21  S2   *C20  H22       1.1112  110.33  119.17  111.72  1.1122
IC H21  S2   *C20  H23       1.1112  110.33 -119.23  111.72  1.1121

RESI NMBA           1.00 ! 3-methylbuten-1-yl methyl ammonium, yxu
```

```
GROUP
ATOM C7     CG334   0.11
ATOM H71    HGA3    0.09
ATOM H72    HGA3    0.09
ATOM H73    HGA3    0.09
ATOM N8     NG3P2  -0.52
ATOM H81    HGP2    0.38
ATOM H82    HGP2    0.38
ATOM C9     CG324   0.20
ATOM H91    HGA2    0.09
ATOM H92    HGA2    0.09
GROUP
ATOM C10    CG2D1  -0.15
ATOM H10    HGA4    0.15
ATOM C11    CG2D1   0.00
GROUP
ATOM C12    CG331  -0.27
ATOM H121   HGA3    0.09
ATOM H122   HGA3    0.09
ATOM H123   HGA3    0.09
GROUP
ATOM C13    CG331  -0.27
ATOM H131   HGA3    0.09
ATOM H132   HGA3    0.09
ATOM H133   HGA3    0.09
BOND H121 C12    H82  N8     H71  C7     H81  N8
BOND H133 C13    N8   C7     N8   C9     H122 C12
BOND C12  C11    C12  H123   C7   H72    C7   H73
BOND C11  C13    C11  C10    C13  H132   C13  H131
BOND H10  C10    C10  C9     C9   H92    C9   H91

IC H71  N8   *C7   H72       1.1113  107.42  119.92  107.37  1.1116
IC H71  N8   *C7   H73       1.1113  107.42 -120.12  107.45  1.1105
IC H71  C7   N8    C9        1.1113  107.42   60.41  115.73  1.5132
IC C9   C7   *N8   H82       1.5132  115.73 -123.14  110.59  1.0109
IC H82  C7   *N8   H81       1.0109  110.59 -115.20  110.50  1.0121
IC C7   N8   C9    C10       1.5032  115.73  179.14  109.92  1.4987
IC C10  N8   *C9   H92       1.4987  109.92  120.52  106.00  1.1047
IC C10  N8   *C9   H91       1.4987  109.92 -123.01  105.84  1.1043
IC N8   C9   C10   C11       1.5132  109.92  -95.90  127.02  1.3473
IC C11  C9   *C10  H10       1.3473  127.02 -175.62  115.87  1.1015
IC C9   C10  C11   C12       1.4987  127.02 -178.90  120.71  1.5043
IC C12  C10  *C11  C13       1.5043  120.71 -179.45  125.40  1.5062
IC C10  C11  C12   H121      1.3473  120.71  120.84  110.63  1.1120
IC H121 C11  *C12  H122      1.1120  110.63  118.55  110.88  1.1121
IC H121 C11  *C12  H123      1.1120  110.63 -120.52  113.72  1.1133
IC C10  C11  C13   H133      1.3473  125.40 -119.55  110.06  1.1131
IC H133 C11  *C13  H132      1.1131  110.06  121.16  115.37  1.1096
IC H133 C11  *C13  H131      1.1131  110.06 -117.28  110.37  1.1126

RESI M6PA           0.00 ! N-(6-purinyl)-N-methyl acetamide, yxu
GROUP
ATOM N9     NG2R51 -0.33 !   H61      O10
ATOM H9     HGP1    0.32 !      \      ||
ATOM C8     CG2R53  0.39 !  H62-C16   C10
ATOM H8     HGR52   0.09 !      / \  /  \
ATOM N7     NG2R50 -0.81 !   H63   N6    C11
ATOM C5     CG2RC0  0.35 !         |
ATOM C6     CG2R64  0.60 !         |
ATOM N1     NG2R62 -0.68 !         C6
ATOM C2     CG2R64  0.41 !       // \
ATOM H2     HGR62   0.14 !      N1   C5--N7\\
ATOM N3     NG2R62 -0.82 !      |    ||     C8-H8
```

```
ATOM C4      CG2RC0  0.57 !       C2   C4--N9/
ATOM N6      NG2S0  -0.49 !        \\ /
ATOM C16     CG331  -0.08 !         N3
ATOM H161    HGA3    0.09
ATOM H162    HGA3    0.09
ATOM H163    HGA3    0.09
ATOM C10     CG2O1   0.49
ATOM O10     OG2D1  -0.44
ATOM C11     CG331  -0.25
ATOM H111    HGA3    0.09
ATOM H112    HGA3    0.09
ATOM H113    HGA3    0.09
BOND H161 C16    H163 C16    C8   H8     N9   H9
BOND C16  H162   C16  N6     H112 C11    N6   C6
BOND N6   C10    C11  H111   C11  C10    C11  H113
BOND N1   C6     N1   C2     C6   C5     H2   C2
BOND C5   N7     C5   C4     C2   N3     C10  O10
BOND N7   C8     C4   N3     C4   N9     C8   N9
IMPR C6     C5     N1     N6       C10    C11    N6     O10
IC C8   C4   *N9   H9        1.3638  107.73  177.04  121.92  1.0032
IC C4   N9   C8    N7        1.3497  107.73    0.64  112.62  1.3221
IC N7   N9   *C8   H8        1.3221  112.62  179.66  122.73  1.0942
IC N9   C8   N7    C5        1.3638  112.62   -0.06  104.60  1.4137
IC C4   N7   *C5   C6        1.4016  108.48  179.63  134.64  1.4273
IC N7   C5   C6    N6        1.4137  134.64   -1.70  126.16  1.4374
IC N6   C5   *C6   N1        1.4374  126.16  179.42  114.74  1.3831
IC C5   C6   N1    C2        1.4273  114.74    1.69  122.70  1.3576
IC C6   N1   C2    N3        1.3831  122.70    0.89  124.39  1.3487
IC N3   N1   *C2   H2        1.3487  124.39  178.77  117.54  1.0949
IC C5   C6   N6    C10       1.4273  126.16  172.04  127.66  1.3829
IC C10  C6   *N6   C16       1.3829  127.66 -175.99  117.54  1.4713
IC C6   N6   C16   H161      1.4374  117.54 -160.83  112.58  1.1148
IC H161 N6   *C16  H163      1.1148  112.58  117.48  109.16  1.1114
IC H161 N6   *C16  H162      1.1148  112.58 -121.87  107.96  1.1125
IC C6   N6   C10   O10       1.4374  127.66 -166.96  119.54  1.2292
IC O10  N6   *C10  C11       1.2292  119.54 -178.74  126.67  1.4902
IC N6   C10  C11   H112      1.3829  126.67  -57.81  111.67  1.1047
IC H112 C10  *C11  H111      1.1047  111.67 -118.19  108.32  1.1100
IC H112 C10  *C11  H113      1.1047  111.67  123.47  110.49  1.1070

RESI BEPA           0.00 ! 1-(2-pyridinyl)amino-3-methyl butene, yxu
GROUP
ATOM N1      NG2R60 -0.600 !     H133      H141
ATOM C2      CG2R61  0.180 !       \      /
ATOM H2      HGR62   0.120 !   H132-C13  C14-H142
ATOM C3      CG2R61 -0.115 !        / \ / \
ATOM H3      HGR61   0.115 !     H131  C12 H143
ATOM C4      CG2R61 -0.115 !          //
ATOM H4      HGR61   0.115 !     H11-C11
ATOM C5      CG2R61 -0.115 !           \
ATOM H5      HGR61   0.115 !       H101-C10   H6
ATOM C6      CG2R64  0.320 !            / \  /
ATOM N6      NG311  -0.450 !          H102 N6
ATOM H6      HGPAM1  0.320 !               |
ATOM C10     CG321  -0.070 !               C6
ATOM H101    HGA2    0.090 !              // \
ATOM H102    HGA2    0.090 !             N1   C5
GROUP                                    |    ||
ATOM C11     CG2D1  -0.15  !             C2   C4
ATOM H11     HGA4    0.15  !              \\ /
ATOM C12     CG2D1   0.00  !               C3
GROUP
ATOM C13     CG331  -0.27
```

```
ATOM H131   HGA3    0.09
ATOM H132   HGA3    0.09
ATOM H133   HGA3    0.09
GROUP
ATOM C14    CG331  -0.27
ATOM H141   HGA3    0.09
ATOM H142   HGA3    0.09
ATOM H143   HGA3    0.09
BOND H11  C11    H101 C10    H133 C13
BOND H2   C2     C11  C10    C11  C12    N1   C2
BOND N1   C6     C2   C3     C10  N6     C10  H102
BOND N6   C6     N6   H6     H131 C13    C6   C5
BOND C3   H3     C3   C4     C13  C12    C13  H132
BOND C12  C14    C5   C4     C5   H5     C4   H4
BOND H142 C14    C14  H143   C14  H141
IC C6   N1   C2   C3        1.3264  121.26   -0.61  121.60  1.4025
IC C3   N1   *C2  H2        1.4025  121.60 -179.51  117.78  1.0838
IC N1   C2   C3   C4        1.3299  121.60    0.50  117.99  1.4026
IC C4   C2   *C3  H3        1.4026  117.99  179.98  120.68  1.0775
IC C2   C3   C4   C5        1.4025  117.99   -0.13  119.46  1.4086
IC C5   C3   *C4  H4        1.4086  119.46 -179.89  120.34  1.0803
IC C6   C4   *C5  H5        1.3993  117.92 -179.97  120.84  1.0766
IC C5   N1   *C6  N6        1.3993  121.76  178.41  118.47  1.3718
IC N1   C6   N6   C10       1.3264  118.47   -5.39  123.30  1.4865
IC C10  C6   *N6  H6        1.4865  123.30 -159.33  116.35  1.0144
IC C6   N6   C10  C11       1.3718  123.30  161.81  109.35  1.5084
IC C11  N6   *C10 H101      1.5084  109.35 -122.03  109.85  1.1144
IC H101 N6   *C10 H102      1.1144  109.85 -116.42  109.39  1.1138
IC N6   C10  C11  C12       1.4865  109.35  -84.52  127.54  1.3478
IC C12  C10  *C11 H11       1.3478  127.54  175.86  114.37  1.0989
IC C10  C11  C12  C13       1.5084  127.54 -179.52  121.35  1.5054
IC C13  C11  *C12 C14       1.5054  121.35  179.87  124.06  1.5060
IC C11  C12  C13  H133      1.3478  121.35 -120.87  110.76  1.1105
IC H133 C12  *C13 H131      1.1105  110.76  120.66  113.06  1.1122
IC H133 C12  *C13 H132      1.1105  110.76 -119.04  110.48  1.1113
IC C11  C12  C14  H142      1.3478  124.06 -117.72  110.53  1.1107
IC H142 C12  *C14 H143      1.1107  110.53  120.74  113.28  1.1121
IC H142 C12  *C14 H141      1.1107  110.53 -119.00  110.57  1.1098

RESI ABOH           0.00 ! 2-hydroxyl-3-amino butane, yxu
GROUP
ATOM C1     CG331  -0.27
ATOM H11    HGA3    0.09
ATOM H12    HGA3    0.09
ATOM H13    HGA3    0.09
GROUP
ATOM C2     CG311   0.14
ATOM H2     HGA1    0.09
ATOM O2     OG311  -0.65
ATOM H2O    HGP1    0.42
GROUP
ATOM C3     CG311   0.12
ATOM H3     HGA1    0.09
ATOM N4     NG321  -0.99
ATOM H41    HGPAM2  0.39
ATOM H42    HGPAM2  0.39
GROUP
ATOM C5     CG331  -0.27
ATOM H51    HGA3    0.09
ATOM H52    HGA3    0.09
ATOM H53    HGA3    0.09
BOND H2O  O2     O2   C2     H2   C2     H13  C1
BOND H52  C5     C2   C1     C2   C3     H53  C5
```

```
BOND C5   C3      C5   H51     C1   H11     C1   H12
BOND H42  N4      C3   N4      C3   H3      N4   H41
IC H13  C2   *C1   H11       1.1096  110.29  118.93  109.77  1.1117
IC H13  C2   *C1   H12       1.1096  110.29 -121.43  111.54  1.1085
IC H13  C1   C2    C3        1.1096  110.29   56.71  111.93  1.5429
IC C3   C1   *C2   O2        1.5429  111.93 -120.42  106.49  1.4207
IC O2   C1   *C2   H2        1.4207  106.49 -117.52  109.84  1.1135
IC C1   C2   O2    H2O       1.5370  106.49  160.08  101.87  0.9662
IC C1   C2   C3    N4        1.5370  111.93 -173.98  112.36  1.4866
IC N4   C2   *C3   C5        1.4866  112.36 -119.63  110.60  1.5340
IC C5   C2   *C3   H3        1.5340  110.60 -119.65  108.10  1.1175
IC C2   C3   N4    H42       1.5429  112.36  -64.88  112.18  1.0154
IC H42  C3   *N4   H41       1.0154  112.18 -118.11  112.00  1.0167
IC C2   C3   C5    H52       1.5429  110.60   57.35  110.74  1.1102
IC H52  C3   *C5   H53       1.1102  110.74  120.29  110.50  1.1116
IC H52  C3   *C5   H51       1.1102  110.74 -120.65  111.04  1.1095

RESI MOAC           0.00 ! methoxyacetic acid, yxu
GROUP
ATOM C5     CG331  -0.10
ATOM H51    HGA3    0.09
ATOM H52    HGA3    0.09
ATOM H53    HGA3    0.09
ATOM O6     OG301  -0.34
ATOM C7     CG321  -0.01
ATOM H71    HGA2    0.09
ATOM H72    HGA2    0.09
GROUP
ATOM C8     CG2O2   0.72
ATOM O8     OG2D1  -0.55
ATOM O9     OG311  -0.60
ATOM H9     HGP1    0.43
BOND O8   C8      C5   H51     C5   H52
BOND C8   O9      C8   C7      H72  C7      O9   H9
BOND C7   O6      C7   H71     O6   C5      H53  C5
IMPR C8     C7     O8     O9
IC H51  O6   *C5   H52       1.1119  109.09  119.33  110.46  1.1117
IC H51  O6   *C5   H53       1.1119  109.09 -119.33  110.46  1.1117
IC H51  C5   O6    C7        1.1119  109.09  180.00  111.34  1.4286
IC C5   O6   C7    C8        1.4190  111.34  180.00  112.76  1.5355
IC C8   O6   *C7   H72       1.5355  112.76  120.18  109.47  1.1104
IC C8   O6   *C7   H71       1.5355  112.76 -120.18  109.47  1.1104
IC O6   C7   C8    O9        1.4286  112.76  180.00  112.76  1.3846
IC O9   C7   *C8   O8        1.3846  112.76  180.00  128.28  1.2185
IC C7   C8   O9    H9        1.5355  112.76  180.00  107.05  0.9566

RESI BZMA           1.00 ! benzyl methyl ammonium, yxu
GROUP
ATOM C1     CG2R61 -0.115
ATOM H1     HGR61   0.115
GROUP
ATOM C2     CG2R61 -0.115
ATOM H2     HGR61   0.115
GROUP
ATOM C3     CG2R61 -0.115
ATOM H3     HGR61   0.115
GROUP
ATOM C4     CG2R61 -0.115
ATOM H4     HGR61   0.115
ATOM C5     CG2R61  0.000
GROUP
ATOM C6     CG2R61 -0.115
ATOM H6     HGR61   0.115
```

```
GROUP
ATOM C7     CG324   0.20
ATOM H71    HGA2    0.09
ATOM H72    HGA2    0.09
ATOM N8     NG3P2  -0.52
ATOM H81    HGP2    0.38
ATOM H82    HGP2    0.38
ATOM C9     CG334   0.11
ATOM H91    HGA3    0.09
ATOM H92    HGA3    0.09
ATOM H93    HGA3    0.09
BOND H71  C7
BOND H93  C9     C7   C5     C7   H72    C7   N8
BOND H4   C4     C5   C4     C5   C6     C4   C3
BOND C3   H3     C3   C2     C1   C6     C1   C2
BOND C1   H1     C6   H6     C2   H2     H82  N8
BOND C9   H91    C9   N8     C9   H92    N8   H81
IC C2   C6   *C1   H1        1.4002  120.02  179.62  120.33  1.0823
IC C6   C1   C2    C3        1.4002  120.02    0.21  120.05  1.4003
IC C3   C1   *C2   H2        1.4003  120.05  179.49  119.99  1.0814
IC C1   C2   C3    C4        1.4002  120.05   -0.18  120.05  1.4004
IC C4   C2   *C3   H3        1.4004  120.05  179.54  119.67  1.0819
IC C2   C3   C4    C5        1.4003  120.05   -0.04  120.26  1.4072
IC C5   C3   *C4   H4        1.4072  120.26  179.40  118.97  1.0809
IC C5   C1   *C6   H6        1.4074  120.29 -179.34  118.93  1.0811
IC C6   C4   *C5   C7        1.4074  119.32 -179.05  120.35  1.5003
IC C4   C5   C7    N8        1.4072  120.35   89.49  109.59  1.5123
IC N8   C5   *C7   H71       1.5123  109.59 -117.68  110.96  1.1034
IC H71  C5   *C7   H72       1.1034  110.96 -124.53  111.01  1.1035
IC C5   C7   N8    C9        1.5003  109.59 -179.92  115.69  1.5040
IC C9   C7   *N8   H82       1.5040  115.69 -124.06  107.62  1.0123
IC H82  C7   *N8   H81       1.0123  107.62 -111.80  107.65  1.0119
IC C7   N8   C9    H93       1.5123  115.69  -60.04  107.38  1.1105
IC H93  N8   *C9   H91       1.1105  107.38  120.12  107.39  1.1106
IC H93  N8   *C9   H92       1.1105  107.38 -119.92  107.32  1.1119

RESI RBRB           0.00 ! tetrahydrofuran-1-oxy-cyclopentane, yxu
GROUP
ATOM C1     CG3C52 -0.18
ATOM H11    HGA2    0.09
ATOM H12    HGA2    0.09
GROUP
ATOM C2     CG3C51  0.13
ATOM H2     HGA1    0.09
ATOM O2     OG301  -0.41
ATOM C6     CG3C51  0.29
ATOM H6     HGA1    0.09
ATOM C9     CG3C52  0.03
ATOM H91    HGA2    0.09
ATOM H92    HGA2    0.09
ATOM O9     OG3C51 -0.40
GROUP
ATOM C3     CG3C52 -0.18
ATOM H31    HGA2    0.09
ATOM H32    HGA2    0.09
GROUP
ATOM C4     CG3C52 -0.18
ATOM H41    HGA2    0.09
ATOM H42    HGA2    0.09
GROUP
ATOM C5     CG3C52 -0.18
ATOM H51    HGA2    0.09
ATOM H52    HGA2    0.09
```

```
GROUP
ATOM C7     CG3C52 -0.18
ATOM H71    HGA2    0.09
ATOM H72    HGA2    0.09
GROUP
ATOM C8     CG3C52 -0.18
ATOM H81    HGA2    0.09
ATOM H82    HGA2    0.09
BOND H52  C5     H51  C5     C7   H71    C7   H72
BOND H12  C1     H11  C1     C5   C1     C5   C4
BOND C1   C2     H92  C9     H42  C4     C4   H41
BOND C4   C3     C9   O9     C9   H91    C9   C8
BOND H81  C8     O9   C6     C2   H2     C2   C3
BOND C2   O2     H31  C3     C3   H32    C8   H82
BOND C8   C7     O2   C6     C6   C7     C6   H6
IC C5   C2   *C1   H12       1.5390  106.67  118.15  108.75  1.1013
IC H12  C2   *C1   H11       1.1013  108.75  116.89  113.51  1.0961
IC H12  C1   C2    O2        1.1013  108.75 -132.09  112.62  1.4296
IC O2   C1   *C2   C3        1.4296  112.62 -121.34  106.58  1.5399
IC C3   C1   *C2   H2        1.5399  106.58 -119.70  110.00  1.1020
IC C1   C2   O2    C6        1.5404  112.62  -49.60  117.29  1.4228
IC C2   O2   C6    O9        1.4296  117.29   95.88  112.36  1.4274
IC O9   O2   *C6   C7        1.4274  112.36  113.97  109.21  1.5301
IC O9   O2   *C6   H6        1.4274  112.36 -121.50  113.20  1.0993
IC O2   C6   O9    C9        1.4228  112.36  155.29  108.22  1.4237
IC C6   O9   C9    C8        1.4274  108.22  -44.15  101.96  1.5242
IC C8   O9   *C9   H92       1.5242  101.96  119.61  108.08  1.1008
IC H92  O9   *C9   H91       1.1008  108.08  118.66  109.31  1.0992
IC C1   C2   C3    C4        1.5404  106.58   -9.35  106.39  1.5411
IC C4   C2   *C3   H31       1.5411  106.39  120.50  111.16  1.0990
IC C4   C2   *C3   H32       1.5411  106.39 -121.94  111.59  1.0989
IC C5   C3   *C4   H42       1.5335  105.03  124.00  113.19  1.0978
IC C5   C3   *C4   H41       1.5335  105.03 -117.35  109.50  1.1034
IC C4   C1   *C5   H52       1.5335  104.31  123.25  112.87  1.0988
IC H52  C1   *C5   H51       1.0988  112.87  118.56  110.09  1.1023
IC C8   C6   *C7   H71       1.5420  104.97  120.54  111.46  1.0996
IC H71  C6   *C7   H72       1.0996  111.46  118.55  110.53  1.0988
IC C7   C9   *C8   H81       1.5420  103.62  123.30  113.14  1.0977
IC H81  C9   *C8   H82       1.0977  113.14  119.50  109.84  1.1023

RESI 5FOP           0.00 ! 2-oxo-5-formylpyrimidine, yxu
GROUP
ATOM N1     NG2R61 -0.21
ATOM C2     CG2R63  0.22
ATOM O2     OG2D4  -0.52
ATOM N3     NG2R62 -0.58
ATOM C4     CG2R62  0.42
ATOM H4     HGR62   0.10
ATOM C5     CG2R62  0.01
ATOM C6     CG2R62  0.17
ATOM H6     HGR62   0.12
ATOM C7     CG2O4   0.28
ATOM H7     HGR52   0.08
ATOM O7     OG2D1  -0.41
ATOM H1     HGP1    0.32
BOND N1   C2     N1   C6     N1   H1     C2   O2
BOND C2   N3     N3   C4     C4   H4     C4   C5
BOND C5   C6     C5   C7     C6   H6     C7   H7
BOND C7   O7
IMPR C2     N1     N3     O2       C7     C5     O7     H7
IC C2   C6   *N1   H1        1.3838  120.92  179.97  126.16  1.0069
IC C6   N1   C2    N3        1.3593  120.92    0.03  119.69  1.3592
IC N3   N1   *C2   O2        1.3592  119.69  179.96  116.85  1.2226
```

```
IC N1   C2   N3    C4        1.3838  119.69   -0.05  118.37  1.3878
IC C2   N3   C4    C5        1.3592  118.37    0.06  122.31  1.3690
IC C5   N3   *C4   H4        1.3690  122.31  179.95  117.97  1.0883
IC C5   N1   *C6   H6        1.3670  120.57 -179.95  118.09  1.0914
IC C6   C4   *C5   C7        1.3670  118.14 -179.94  121.43  1.4584
IC C4   C5   C7    O7        1.3690  121.43 -180.00  124.95  1.2121
IC O7   C5   *C7   H7        1.2121  124.95  179.93  116.66  1.1104

RESI PENM           0.00 ! 3-methylamino-propene, yxu
GROUP
ATOM C5     CG331  -0.06
ATOM H51    HGA3    0.09
ATOM H52    HGA3    0.09
ATOM H53    HGA3    0.09
ATOM N6     NG311  -0.78
ATOM H6     HGPAM1  0.36
ATOM C7     CG321   0.03
ATOM H71    HGA2    0.09
ATOM H72    HGA2    0.09
GROUP
ATOM C8     CG2D1  -0.15
ATOM H8     HGA4    0.15
GROUP
ATOM C9     CG2D2  -0.42
ATOM H91    HGA5    0.21
ATOM H92    HGA5    0.21
BOND H72  C7     H71  C7     C7   C8
BOND C7   N6     H8   C8     H51  C5     C8   C9
BOND H53  C5     C9   H91    C9   H92    C5   N6
BOND C5   H52    N6   H6
IC H51  N6   *C5   H53       1.1131  110.85  119.23  110.78  1.1147
IC H51  N6   *C5   H52       1.1131  110.85 -120.49  111.46  1.1124
IC H51  C5   N6    C7        1.1131  110.85 -178.73  111.53  1.4729
IC C7   C5   *N6   H6        1.4729  111.53 -121.78  106.56  1.0231
IC C5   N6   C7    C8        1.4681  111.53 -178.74  108.33  1.5034
IC C8   N6   *C7   H72       1.5034  108.33 -120.52  109.91  1.1140
IC H72  N6   *C7   H71       1.1140  109.91 -118.13  109.70  1.1145
IC N6   C7   C8    C9        1.4729  108.33  152.49  126.78  1.3483
IC C9   C7   *C8   H8        1.3483  126.78  176.83  114.70  1.1034
IC C7   C8   C9    H91       1.5034  126.78  178.36  120.94  1.1012
IC H91  C8   *C9   H92       1.1012  120.94 -179.97  120.81  1.1010

RESI PESU           0.00 ! 2-(propene-3-yl)thiouracil, yxu
GROUP
ATOM N1     NG2R61 -0.56 !     O4
ATOM H1     HGP1    0.31 !     ||
ATOM C2     CG2R64  0.41 !     C4
ATOM S2     SG311  -0.14 !    /  \
ATOM N3     NG2R62 -0.56 !   C5   N3
ATOM C4     CG2R63  0.53 !   ||   ||
ATOM O4     OG2D4  -0.53 !   C6   C2   C20    C22
ATOM C5     CG2R62 -0.14 !    \  / \  /   \  //
ATOM H5     HGR62   0.10 !     N1   S2     C21
ATOM C6     CG2R62  0.24
ATOM H6     HGR62   0.14
ATOM C20    CG321   0.02
ATOM H201   HGA2    0.09
ATOM H202   HGA2    0.09
GROUP
ATOM C21    CG2D1  -0.15
ATOM H21    HGA4    0.15
GROUP
ATOM C22    CG2D2  -0.42
```

```
ATOM H221   HGA5    0.21
ATOM H222   HGA5    0.21
BOND N1   C2      N1   C6      C22  H222
BOND N1   H1      C2   S2      C2   N3      S2   C20
BOND N3   C4      C4   O4      C4   C5      C5   H5
BOND C5   C6      C6   H6      C20  H201    C20  H202
BOND C20  C21     C21  H21     C21  C22     C22  H221
IMPR C4     C5     N3     O4
IC C6   C2   *N1   H1        1.3576  119.80 -179.84  116.57  1.0069
IC C6   N1   C2    S2        1.3576  119.80  179.99  121.06  1.7437
IC S2   N1   *C2   N3        1.7437  121.06  179.95  119.83  1.3501
IC N1   C2   N3    C4        1.3951  119.83    0.07  121.50  1.3605
IC C2   N3   C4    O4        1.3501  121.50  179.95  120.71  1.2284
IC O4   N3   *C4   C5        1.2284  120.71  179.98  118.98  1.4321
IC C6   C4   *C5   H5        1.3631  118.49  179.92  120.42  1.0864
IC C5   N1   *C6   H6        1.3631  121.40  179.97  117.90  1.0936
IC N1   C2   S2    C20       1.3951  121.06 -179.18  102.40  1.8294
IC C2   S2   C20   C21       1.7437  102.40 -179.62  108.14  1.5067
IC C21  S2   *C20  H201      1.5067  108.14  120.23  110.49  1.1120
IC H201 S2   *C20  H202      1.1120  110.49  118.01  110.39  1.1134
IC S2   C20  C21   C22       1.8294  108.14 -118.32  126.75  1.3472
IC C22  C20  *C21  H21       1.3472  126.75  179.90  114.99  1.1030
IC C20  C21  C22   H222      1.5067  126.75  179.89  121.17  1.1015
IC H222 C21  *C22  H221      1.1015  121.17 -179.87  120.84  1.1014

RESI BZAM           1.00 ! phenylammonium, yxu
GROUP
ATOM C1     CG2R61 -0.115
ATOM H1     HGR61   0.115
GROUP
ATOM C2     CG2R61 -0.115
ATOM H2     HGR61   0.115
GROUP
ATOM C3     CG2R61 -0.115
ATOM H3     HGR61   0.115
GROUP
ATOM C4     CG2R61 -0.115
ATOM H4     HGR61   0.115
GROUP
ATOM C6     CG2R61 -0.115
ATOM H6     HGR61   0.115
GROUP
ATOM C5     CG2R61  0.090
ATOM C7     CG324   0.080
ATOM H71    HGA2    0.090
ATOM H72    HGA2    0.090
ATOM N8     NG3P3  -0.340
ATOM H81    HGP2    0.330
ATOM H82    HGP2    0.330
ATOM H83    HGP2    0.330
BOND H72  C7      H4   C4      H71  C7
BOND H3   C3      C4   C3      C4   C5      C3   C2
BOND C7   C5      C7   N8      C5   C6      C2   H2
BOND C2   C1      C6   C1      C6   H6      C1   H1
BOND N8   H81     N8   H83     N8   H82
IC C2   C6   *C1   H1        1.3998  119.93 -179.56  120.32  1.0822
IC C6   C1   C2    C3        1.4002  119.93   -0.17  120.19  1.3993
IC C3   C1   *C2   H2        1.3993  120.19 -179.52  119.88  1.0831
IC C1   C2   C3    C4        1.3998  120.19    0.14  119.97  1.4008
IC C4   C2   *C3   H3        1.4008  119.97 -179.51  119.76  1.0824
IC C2   C3   C4    C5        1.3993  119.97    0.08  120.30  1.4079
IC C5   C3   *C4   H4        1.4079  120.30 -179.41  119.04  1.0812
IC C5   C1   *C6   H6        1.4075  120.36  179.35  119.03  1.0808
```

```
IC C6   C4   *C5   C7        1.4075  119.24  179.01  120.34  1.5083
IC C4   C5   C7    N8        1.4079  120.34  -89.53  110.11  1.5011
IC N8   C5   *C7   H72       1.5011  110.11  120.19  109.41  1.1025
IC H72  C5   *C7   H71       1.1025  109.41  119.68  109.41  1.1023
IC C5   C7   N8    H81       1.5083  110.11 -179.98  110.71  1.0415
IC H81  C7   *N8   H83       1.0415  110.71  120.45  109.94  1.0416
IC H81  C7   *N8   H82       1.0415  110.71 -120.43  109.90  1.0421

RESI CMBZ           0.00 ! carboxyhydroxymethyl benzene, yxu
GROUP
ATOM C1     CG2R61 -0.115
ATOM H1     HGR61   0.115
GROUP
ATOM C2     CG2R61 -0.115
ATOM H2     HGR61   0.115
GROUP
ATOM C3     CG2R61 -0.115
ATOM H3     HGR61   0.115
GROUP
ATOM C4     CG2R61 -0.070
ATOM H4     HGR61   0.115
ATOM C5     CG2R61 -0.090
ATOM C6     CG2R61 -0.070
ATOM H6     HGR61   0.115
GROUP
ATOM C7     CG311   0.14
ATOM H7     HGA1    0.09
ATOM O7     OG311  -0.65
ATOM H7O    HGP1    0.42
GROUP
ATOM O8     OG2D1  -0.63
ATOM C8     CG2O2   0.86
ATOM O9     OG302  -0.49
ATOM C9     CG331  -0.01
ATOM H91    HGA3    0.09
ATOM H92    HGA3    0.09
ATOM H93    HGA3    0.09
BOND H93  C9     H91  C9     O8   C8
BOND C9   H92    C9   O9     H7O  O7     C8   O9
BOND C8   C7     O7   C7     H4   C4     C7   C5
BOND C7   H7     C4   C5     C4   C3     C5   C6
BOND H3   C3     C3   C2     C6   H6     C6   C1
BOND C2   C1     C2   H2     C1   H1
IMPR C8     C7     O8     O9
IC C2   C6   *C1   H1        1.3996  120.03 -179.98  119.86  1.0801
IC C6   C1   C2    C3        1.4008  120.03    0.00  119.95  1.3996
IC C3   C1   *C2   H2        1.3996  119.95 -179.81  119.99  1.0805
IC C1   C2   C3    C4        1.3996  119.95    0.09  120.03  1.4003
IC C4   C2   *C3   H3        1.4003  120.03 -179.72  120.00  1.0802
IC C2   C3   C4    C5        1.3996  120.03   -0.13  120.82  1.4097
IC C5   C3   *C4   H4        1.4097  120.82 -179.18  119.27  1.0803
IC C5   C1   *C6   H6        1.4116  120.76 -179.84  119.37  1.0801
IC C6   C4   *C5   C7        1.4116  118.42  179.70  118.91  1.5252
IC C4   C5   C7    C8        1.4097  118.91   66.59  110.96  1.5800
IC C8   C5   *C7   O7        1.5800  110.96  127.46  114.42  1.4517
IC C8   C5   *C7   H7        1.5800  110.96 -115.41  107.94  1.1105
IC C5   C7   O7    H7O       1.5252  114.42 -164.01  107.02  0.9668
IC C5   C7   C8    O9        1.5252  110.96  162.00  110.04  1.3525
IC O9   C7   *C8   O8        1.3525  110.04  176.91  126.33  1.2188
IC C7   C8   O9    C9        1.5800  110.04  179.05  112.07  1.4386
IC C8   O9   C9    H93       1.3525  112.07  179.20  109.88  1.1121
IC H93  O9   *C9   H91       1.1121  109.88  119.57  110.92  1.1144
IC H93  O9   *C9   H92       1.1121  109.88 -119.56  110.89  1.1132
```

```
RESI HMBT           0.00 ! 1-hydroxyl-2-methyl butene, yxu
GROUP
ATOM C10    CG331  -0.27
ATOM H101   HGA3    0.09
ATOM H102   HGA3    0.09
ATOM H103   HGA3    0.09
GROUP
ATOM C11    CG2D1  -0.15
ATOM H11    HGA4    0.15
ATOM C12    CG2D1   0.00
GROUP
ATOM C13    CG331  -0.27
ATOM H131   HGA3    0.09
ATOM H132   HGA3    0.09
ATOM H133   HGA3    0.09
GROUP
ATOM C14    CG321   0.05
ATOM H141   HGA2    0.09
ATOM H142   HGA2    0.09
ATOM O14    OG311  -0.65
ATOM HO14   HGP1    0.42
BOND H101 C10    C14  H141   O14  HO14
BOND H11  C11    H133 C13    C11  C10    C11  C12
BOND C10  H102   C10  H103   C13  H131   C13  C12
BOND C13  H132   C12  C14    C14  H142   C14  O14
IC H101 C11  *C10  H102      1.1113  110.80  118.77  110.49  1.1120
IC H101 C11  *C10  H103      1.1113  110.80 -121.07  114.01  1.1106
IC H101 C10  C11   C12       1.1113  110.80  115.60  128.83  1.3529
IC C12  C10  *C11  H11       1.3529  128.83  176.17  113.39  1.1016
IC C10  C11  C12   C14       1.5128  128.83  179.67  119.70  1.5137
IC C14  C11  *C12  C13       1.5137  119.70  179.55  123.28  1.5126
IC C11  C12  C13   H133      1.3529  123.28 -126.22  110.45  1.1117
IC H133 C12  *C13  H131      1.1117  110.45  120.25  114.23  1.1092
IC H133 C12  *C13  H132      1.1117  110.45 -119.00  110.58  1.1110
IC C11  C12  C14   O14       1.3529  119.70 -114.17  110.26  1.4262
IC O14  C12  *C14  H141      1.4262  110.26  119.86  112.56  1.1154
IC H141 C12  *C14  H142      1.1154  112.56  119.31  110.50  1.1126
IC C12  C14  O14   HO14      1.5137  110.26  -67.56  106.76  0.9614

RESI HPIZ           0.00 ! 3-(2-hydroxylpropyl)indolizine, yxu
GROUP
ATOM C5     CG2R61 -0.14 !     H141  H15   H161
ATOM H5     HGR61   0.17 !       |    |     |
ATOM C6     CG2R61 -0.10 !   *---C14--C15--C16-H162
ATOM H6     HGR62   0.24 !       |    |     |
ATOM N1     NG2RC0 -0.29 !     H142  O15   H162
ATOM C7     CG2R51  0.12 !             \
ATOM C8     CG2R51 -0.23 !              H15O
ATOM H8     HGR51   0.21 !    *
ATOM C9     CG2R51 -0.29 !    |      C6
ATOM H9     HGR51   0.23 !    |    /  \\
ATOM C2     CG2RC0 -0.07 !  //C7--N1   C5
ATOM C3     CG2R61 -0.22 ! C8     |    |
ATOM H3     HGR61   0.24 !   \C9==C2   C4
ATOM C4     CG2R61 -0.15 !         \  //
ATOM H4     HGR61   0.20 !          C3
ATOM C14    CG321  -0.10
ATOM H141   HGA2    0.09
ATOM H142   HGA2    0.09
GROUP
ATOM C15    CG311   0.14
ATOM H15    HGA1    0.09
```

```
ATOM O15    OG311  -0.65
ATOM H15O   HGP1    0.42
GROUP
ATOM C16    CG331  -0.27
ATOM H161   HGA3    0.09
ATOM H162   HGA3    0.09
ATOM H163   HGA3    0.09
BOND H142 C14    H15  C15    H163 C16
BOND H162 C16    H9   C9     H3   C3     C9   C2
BOND C9   C8     C3   C2     C3   C4     C2   N1
BOND H8   C8     C8   C7     C4   H4     C4   C5
BOND N1   C7     N1   C6     C7   C14    C6   C5
BOND C6   H6     C5   H5     C14  C15    C14  H141
BOND C16  C15    C16  H161   C15  O15    H15O O15
IC C4   C6   *C5   H5        1.4021  120.11 -179.96  119.87  1.0797
IC C4   C5   C6    N1        1.4021  120.11   -0.20  120.82  1.4015
IC N1   C5   *C6   H6        1.4015  120.82 -179.92  120.58  1.0799
IC C5   C6   N1    C7        1.4010  120.82 -179.72  133.55  1.3726
IC C7   C6   *N1   C2        1.3726  133.55 -179.89  118.43  1.4229
IC C6   N1   C7    C14       1.4015  133.55   -2.94  123.40  1.5100
IC C14  N1   *C7   C8        1.5100  123.40 -176.31  108.01  1.3742
IC N1   C7   C8    C9        1.3726  108.01   -0.82  110.64  1.3701
IC C9   C7   *C8   H8        1.3701  110.64  179.96  124.48  1.0779
IC C2   C8   *C9   H9        1.4211  106.62  179.38  127.08  1.0799
IC C9   N1   *C2   C3        1.4211  106.70  179.96  120.66  1.3878
IC C4   C2   *C3   H3        1.4028  120.36  179.89  118.89  1.0786
IC C3   C5   *C4   H4        1.4028  119.62 -179.92  120.13  1.0803
IC N1   C7   C14   C15       1.3726  123.40  167.25  117.07  1.5453
IC C15  C7   *C14  H142      1.5453  117.07 -121.35  108.07  1.1116
IC H142 C7   *C14  H141      1.1116  108.07 -115.58  107.69  1.1109
IC C7   C14  C15   O15       1.5100  117.07  173.33  108.25  1.4165
IC O15  C14  *C15  C16       1.4165  108.25  118.44  113.41  1.5323
IC C16  C14  *C15  H15       1.5323  113.41  123.60  109.53  1.1147
IC C14  C15  O15   H15O      1.5453  108.25 -176.54  106.16  0.9602
IC C14  C15  C16   H163      1.5453  113.41 -179.65  111.05  1.1102
IC H163 C15  *C16  H162      1.1102  111.05  120.23  110.10  1.1118
IC H163 C15  *C16  H161      1.1102  111.05 -120.44  110.64  1.1100

RESI PEPR           0.00 ! 1-(3-cyclopentenoxy) tetrahydropyran, yxu
GROUP
ATOM C1     CG3C52 -0.18
ATOM H11    HGA2    0.09
ATOM H12    HGA2    0.09
GROUP
ATOM C2     CG3C51  0.13
ATOM H2     HGA1    0.09
ATOM O2     OG301  -0.41
ATOM C6     CG311   0.29
ATOM H6     HGA1    0.09
ATOM O6     OG3C61 -0.40
ATOM C7     CG321   0.03
ATOM H71    HGA2    0.09
ATOM H72    HGA2    0.09
GROUP
ATOM C3     CG3C52 -0.18
ATOM H31    HGA2    0.09
ATOM H32    HGA2    0.09
GROUP
ATOM C4     CG2R51 -0.25
ATOM H4     HGR51   0.25
GROUP
ATOM C5     CG2R51 -0.25
ATOM H5     HGR51   0.25
```

```
GROUP
ATOM C8      CG321  -0.18
ATOM H81     HGA2    0.09
ATOM H82     HGA2    0.09
GROUP
ATOM C9      CG321  -0.18
ATOM H91     HGA2    0.09
ATOM H92     HGA2    0.09
GROUP
ATOM C10     CG321  -0.18
ATOM H101    HGA2    0.09
ATOM H102    HGA2    0.09
BOND H81  C8
BOND H91  C9      H82  C8      C8   C9      C8   C7
BOND C9   H92     C9   C10     H101 C10     H72  C7
BOND C10  H102    C10  C6      C7   O6      C7   H71
BOND O6   C6      C6   H6      C6   O2      O2   C2
BOND H31  C3      H32  C3      C3   C2      C3   C4
BOND C2   H2      C2   C1      H12  C1      C1   H11
BOND C1   C5      C4   H4      C4   C5      C5   H5
IC C5   C2   *C1   H12       1.5176  104.10  120.25  110.79  1.1023
IC H12  C2   *C1   H11       1.1023  110.79  117.67  111.13  1.1008
IC H12  C1   C2    O2        1.1023  110.79  -20.97  108.13  1.4281
IC O2   C1   *C2   C3        1.4281  108.13  115.58  104.68  1.5189
IC O2   C1   *C2   H2        1.4281  108.13 -124.69  110.74  1.1047
IC C1   C2   O2    C6        1.5185  108.13 -128.28  112.38  1.4200
IC C2   O2   C6    O6        1.4281  112.38  167.71  110.17  1.4190
IC O6   O2   *C6   C10       1.4190  110.17  120.47  109.61  1.5468
IC C10  O2   *C6   H6        1.5468  109.61  121.28  109.42  1.1130
IC O2   C6   O6    C7        1.4200  110.17 -170.41  112.65  1.4243
IC C6   O6   C7    C8        1.4190  112.65  -35.60  113.36  1.5406
IC C8   O6   *C7   H72       1.5406  113.36 -119.53  106.76  1.1133
IC C8   O6   *C7   H71       1.5406  113.36  124.05  110.14  1.1119
IC C1   C2   C3    C4        1.5185  104.68   25.62  104.13  1.5168
IC C4   C2   *C3   H31       1.5168  104.13 -120.31  110.73  1.1028
IC H31  C2   *C3   H32       1.1028  110.73 -117.60  111.09  1.1008
IC C5   C3   *C4   H4        1.3696  109.95 -173.06  123.72  1.0830
IC C4   C1   *C5   H5        1.3696  109.94  172.99  123.63  1.0833
IC O6   C7   C8    C9        1.4243  113.36  -28.05  111.02  1.5304
IC C9   C7   *C8   H81       1.5304  111.02  118.93  108.81  1.1132
IC H81  C7   *C8   H82       1.1132  108.81  117.79  109.89  1.1106
IC C10  C8   *C9   H91       1.5367  109.39  123.42  111.88  1.1110
IC H91  C8   *C9   H92       1.1110  111.88  118.67  107.71  1.1155
IC C9   C6   *C10  H101      1.5367  112.62  120.50  108.90  1.1119
IC C9   C6   *C10  H102      1.5367  112.62 -122.12  108.59  1.1100

RESI PNPA           1.00 ! 1-(3-pyrrolyl)-N-(3-cyclopentenyl) methylammonium, yxu
GROUP
ATOM N9      NG2R51 -0.38 !                        H13  O13-H13O
ATOM H9      HGP1    0.39 !                          \  /
ATOM C8      CG2R51 -0.04 !               H111 H12   C13(R)  H14
ATOM H8      HGR52   0.16 !                 |   \   /    \  /
ATOM C7      CG2R51 -0.11 !           /C10--N11--C12(S)   C14(S)
ATOM C5      CG2R51 -0.25 !          /      |(+)   \      / \
ATOM H5      HGR51   0.13 !         /     H112      C16==C15 O14-H14O
ATOM C4      CG2R51 -0.04 !        /               /       \
ATOM H4      HGR52   0.14 !       /              H16      H15
GROUP                     ! C5--C7\\
ATOM C10     CG324   0.14 ! ||     C8-H8
ATOM H101    HGA2    0.09 ! C4--N9/
ATOM H102    HGA2    0.09
ATOM N11     NG3P2  -0.31
ATOM H111    HGP2    0.28
```

```
ATOM H112   HGP2    0.28
ATOM C12    CG3C53  0.34
ATOM H12    HGA1    0.09
GROUP
ATOM C13    CG3C52 -0.18
ATOM H131   HGA2    0.09
ATOM H132   HGA2    0.09
GROUP
ATOM C14    CG3C52 -0.18
ATOM H141   HGA2    0.09
ATOM H142   HGA2    0.09
GROUP
ATOM C15    CG2R51 -0.27
ATOM H15    HGR51   0.29
ATOM C16    CG2R51 -0.21
ATOM H16    HGR51   0.19
BOND H12  C12    H132 C13    C8   H8     C15  H15
BOND H112 N11    H111 N11    C12  N11    C12  C13
BOND C12  C16    N11  C10    C13  H131   C13  C14
BOND H141 C14    H5   C5     H4   C4     C5   C4
BOND C5   C7     C4   N9     C14  H142   C14  C15
BOND C7   C10    C7   C8     C16  H16    C16  C15
BOND N9   C8     N9   H9     C10  H101   C10  H102
IC C4   C8   *N9   H9       1.3753  109.31 -177.45  125.98  1.0144
IC C4   N9   C8    C7       1.3753  109.31   -0.20  106.72  1.3650
IC C7   N9   *C8   H8       1.3650  106.72  178.39  123.33  1.0846
IC C8   N9   C4    C5       1.3773  109.31    0.03  106.28  1.3583
IC C5   N9   *C4   H4       1.3583  106.28 -178.95  124.13  1.0850
IC C7   C4   *C5   H5       1.3656  109.52 -178.56  124.37  1.0814
IC C5   C8   *C7   C10      1.3656  108.16  179.51  126.54  1.4867
IC C8   C7   C10   N11      1.3650  126.54   99.39  109.28  1.5091
IC N11  C7   *C10  H101     1.5091  109.28  117.55  111.26  1.1022
IC N11  C7   *C10  H102     1.5091  109.28 -117.37  112.03  1.1039
IC C7   C10  N11   C12      1.4867  109.28 -178.89  119.17  1.4910
IC C12  C10  *N11  H112     1.4910  119.17 -121.34  108.29  1.0074
IC H112 C10  *N11  H111     1.0074  108.29 -114.27  108.86  1.0054
IC C10  N11  C12   C13      1.5091  119.17  176.64  107.82  1.5242
IC C13  N11  *C12  C16      1.5242  107.82  112.43  109.79  1.5039
IC C13  N11  *C12  H12      1.5242  107.82 -124.92  107.60  1.0845
IC N11  C12  C13   C14      1.4910  107.82  142.13  104.87  1.5205
IC C14  C12  *C13  H132     1.5205  104.87 -118.38  112.74  1.1059
IC H132 C12  *C13  H131     1.1059  112.74 -119.65  111.90  1.1010
IC C12  C13  C14   C15      1.5242  104.87  -27.34  103.24  1.5136
IC C15  C13  *C14  H141     1.5136  103.24 -122.43  111.94  1.1043
IC H141 C13  *C14  H142     1.1043  111.94 -117.80  111.18  1.1051
IC C16  C14  *C15  H15      1.3725  109.91  172.54  123.37  1.0889
IC C15  C12  *C16  H16      1.3725  110.40 -178.03  124.85  1.0818

RESI PRNC           0.00 ! 2-propylamino-4-imino-pyrimidine, yxu
GROUP
ATOM N1     NG2R61 -0.65
ATOM H1     HGP1    0.31
ATOM C2     CG2R64  0.83
ATOM N2     NG311  -0.58
ATOM H2     HGPAM1  0.38
ATOM N3     NG2R62 -0.72
ATOM C4     CG2R64  0.60
ATOM N4     NG2D1  -0.94
ATOM H4     HGP1    0.32
ATOM C5     CG2R61 -0.23
ATOM H5     HGR61   0.17
ATOM C6     CG2R61  0.08
ATOM H6     HGR62   0.17
```

```
ATOM C7     CG321   0.08
ATOM H71    HGA2    0.09
ATOM H72    HGA2    0.09
GROUP
ATOM C8     CG321  -0.18
ATOM H81    HGA2    0.09
ATOM H82    HGA2    0.09
GROUP
ATOM C9     CG331  -0.27
ATOM H91    HGA3    0.09
ATOM H92    HGA3    0.09
ATOM H93    HGA3    0.09
BOND N1   C2     N1   C6     C9   H93
BOND N1   H1     C2   N2     C2   N3     N2   H2
BOND N2   C7     N3   C4     C4   N4     C4   C5
BOND N4   H4     C5   H5     C5   C6     C6   H6
BOND C7   H71    C7   H72    C7   C8     C8   H81
BOND C8   H82    C8   C9     C9   H91    C9   H92
IMPR C2     N1     N3     N2    C4   C5   N4   N3
IC C6   C2   *N1   H1        1.3943  120.22  175.07  115.08  1.0019
IC C6   N1   C2    N2        1.3943  120.22  179.74  116.21  1.3506
IC N2   N1   *C2   N3        1.3506  116.21 -179.29  122.00  1.3305
IC N1   C2   N2    C7        1.3898  116.21 -167.15  122.26  1.4872
IC C7   C2   *N2   H2        1.4872  122.26  145.73  111.63  1.0073
IC N1   C2   N3    C4        1.3898  122.00    0.45  119.82  1.3809
IC C2   N3   C4    N4        1.3305  119.82  178.98  121.52  1.3283
IC N4   N3   *C4   C5        1.3283  121.52 -179.86  120.27  1.4279
IC N3   C4   N4    H4        1.3809  121.52  179.95  110.16  0.9934
IC C6   C4   *C5   H5        1.3919  118.94 -179.91  119.32  1.0734
IC C5   N1   *C6   H6        1.3919  118.75  179.97  119.31  1.0844
IC C2   N2   C7    C8        1.3506  122.26   74.16  110.89  1.5372
IC C8   N2   *C7   H71       1.5372  110.89 -121.70  110.79  1.1146
IC H71  N2   *C7   H72       1.1146  110.79 -117.75  109.43  1.1136
IC N2   C7   C8    C9        1.4872  110.89  177.21  113.80  1.5358
IC C9   C7   *C8   H81       1.5358  113.80  120.88  108.98  1.1160
IC H81  C7   *C8   H82       1.1160  108.98  117.24  109.42  1.1125
IC C7   C8   C9    H93       1.5372  113.80  179.47  110.48  1.1112
IC H93  C8   *C9   H91       1.1112  110.48  120.30  110.87  1.1100
IC H93  C8   *C9   H92       1.1112  110.48 -119.40  110.30  1.1127

RESI CONA           0.00 ! 3-methylamino-1,2-epoxycyclopentene, yxu
GROUP
ATOM C1     CG331  -0.06
ATOM H11    HGA3    0.09
ATOM H12    HGA3    0.09
ATOM H13    HGA3    0.09
ATOM N2     NG311  -0.78
ATOM H2     HGPAM1  0.36
ATOM C3     CG3C51  0.12
ATOM H3     HGA1    0.09
ATOM C4     CG3RC1 -0.08
ATOM H4     HGA1    0.09
ATOM O4     OG3C31 -0.25
ATOM C5     CG3RC1  0.15
ATOM H5     HGA1    0.09
ATOM C6     CG3C52 -0.18
ATOM H61    HGA2    0.09
ATOM H62    HGA2    0.09
ATOM C7     CG3C52 -0.18
ATOM H71    HGA2    0.09
ATOM H72    HGA2    0.09
BOND H11  C1     H12  C1     C6   H62    C6   H61
BOND C1   H13    C1   N2     H2   N2     N2   C3
```

```
BOND H4   C4      C3   H3      C3   C4      C3   C7
BOND C4   O4      C4   C5      H71  C7      C7   H72
BOND C7   C6      O4   C5      C5   H5      C5   C6
IC H11  N2   *C1   H12       1.0701  109.47  119.97  109.49  1.0697
IC H11  N2   *C1   H13       1.0701  109.47 -120.01  109.49  1.0699
IC H11  C1   N2    C3        1.0701  109.47  166.33  118.80  1.4787
IC C3   C1   *N2   H2        1.4787  118.80  130.58  110.71  1.0200
IC C1   N2   C3    C4        1.4879  118.80  112.13  111.79  1.5209
IC C4   N2   *C3   C7        1.5209  111.79  108.50  111.48  1.5500
IC C4   N2   *C3   H3        1.5209  111.79 -125.67  114.15  1.1032
IC N2   C3   C4    C5        1.4787  111.79  146.53  110.46  1.4939
IC C5   C3   *C4   O4        1.4939  110.46   63.69  112.36  1.4358
IC O4   C3   *C4   H4        1.4358  112.36  144.27  122.75  1.0987
IC O4   C4   *C5   C6        1.4406   58.55 -112.90  107.49  1.5393
IC O4   C4   *C5   H5        1.4406   58.55  104.94  118.61  1.0993
IC C7   C5   *C6   H62       1.5564  100.59  117.70  111.14  1.0700
IC C7   C5   *C6   H61       1.5564  100.59 -117.73  111.13  1.0704
IC C6   C3   *C7   H71       1.5564  107.04  119.41  109.95  1.0698
IC H71  C3   *C7   H72       1.0698  109.95  121.18  109.94  1.0700

RESI BZHA          -1.00 ! 2-phenyl-2-hydroxylacetate, yxu
GROUP
ATOM C1     CG2R61 -0.115
ATOM H1     HGR61   0.115
GROUP
ATOM C2     CG2R61 -0.115
ATOM H2     HGR61   0.115
GROUP
ATOM C3     CG2R61 -0.115
ATOM H3     HGR61   0.115
GROUP
ATOM C4     CG2R61 -0.090
ATOM H4     HGR61   0.115
ATOM C5     CG2R61 -0.050
ATOM C6     CG2R61 -0.090
ATOM H6     HGR61   0.115
GROUP
ATOM C7     CG311   0.14
ATOM H7     HGA1    0.09
ATOM O7     OG311  -0.65
ATOM HO7    HGP1    0.42
ATOM C8     CG2O3   0.52
ATOM O81    OG2D2  -0.76
ATOM O82    OG2D2  -0.76
BOND O81  C8      H4   C4
BOND H7   C7      C8   C7      C8   O82     C7   C5
BOND C7   O7      C4   C3      C4   C5      H3   C3
BOND C3   C2      C5   C6      O7   HO7     C6   H6
BOND C6   C1      C2   H2      C2   C1      C1   H1
IMPR C8     O82    O81    C7
IC C2   C6   *C1   H1        1.4002  119.99  179.09  119.70  1.0798
IC C6   C1   C2    C3        1.3992  119.99    0.39  119.90  1.4003
IC C3   C1   *C2   H2        1.4003  119.90  178.96  120.05  1.0796
IC C1   C2   C3    C4        1.4002  119.90   -0.45  120.01  1.4004
IC C4   C2   *C3   H3        1.4004  120.01  179.21  120.20  1.0796
IC C2   C3   C4    C5        1.4003  120.01   -0.11  120.83  1.4114
IC C5   C3   *C4   H4        1.4114  120.83  178.18  119.84  1.0799
IC C5   C1   *C6   H6        1.4093  120.96 -178.57  120.20  1.0821
IC C6   C4   *C5   C7        1.4093  118.30 -179.43  122.89  1.5312
IC C4   C5   C7    C8        1.4114  122.89 -111.39  108.67  1.5691
IC C8   C5   *C7   O7        1.5691  108.67  128.89  113.79  1.4499
IC C8   C5   *C7   H7        1.5691  108.67 -114.27  108.40  1.1107
IC C5   C7   O7    HO7       1.5312  113.79   62.72  105.01  0.9557
```

```
IC C5   C7   C8    O81       1.5312  108.67  -90.70  113.96  1.2670
IC O81  C7   *C8   O82       1.2670  113.96  178.99  119.43  1.2594

RESI MOAT          -1.00 ! methoxy acetate, yxu
GROUP
ATOM C5     CG331  -0.07
ATOM H51    HGA3    0.09
ATOM H52    HGA3    0.09
ATOM H53    HGA3    0.09
ATOM O7     OG301  -0.42
ATOM C7     CG321   0.04
ATOM H71    HGA2    0.09
ATOM H72    HGA2    0.09
GROUP
ATOM C8     CG2O3   0.52
ATOM O81    OG2D2  -0.76
ATOM O82    OG2D2  -0.76
BOND O82  C8     O81  C8
BOND C8   C7     H72  C7     C7   O7     C7   H71
BOND O7   C5     H53  C5     C5   H51    C5   H52
IMPR C8     O82    O81    C7
IC H53  O7   *C5   H51       1.1105  109.07  119.50  110.05  1.1106
IC H53  O7   *C5   H52       1.1105  109.07 -119.55  110.05  1.1111
IC H53  C5   O7    C7        1.1105  109.07  180.00  111.17  1.4398
IC C5   O7   C7    C8        1.4171  111.17  180.00  117.85  1.5429
IC C8   O7   *C7   H72       1.5429  117.85  120.78  109.05  1.1095
IC C8   O7   *C7   H71       1.5429  117.85 -120.78  109.05  1.1095
IC O7   C7   C8    O82       1.4398  117.85  180.00  113.27  1.2643
IC O82  C7   *C8   O81       1.2643  113.27  180.00  119.51  1.2562

RESI MOAE           0.00 ! methoxyacetate methyl, yxu
GROUP
ATOM C5     CG331  -0.07
ATOM H51    HGA3    0.09
ATOM H52    HGA3    0.09
ATOM H53    HGA3    0.09
ATOM O7     OG301  -0.42
ATOM C7     CG321   0.04
ATOM H81    HGA2    0.09
ATOM H82    HGA2    0.09
GROUP
ATOM C8     CG2O2   0.86
ATOM O8     OG2D1  -0.63
ATOM O9     OG302  -0.49
ATOM C10    CG331  -0.01
ATOM H101   HGA3    0.09
ATOM H102   HGA3    0.09
ATOM H103   HGA3    0.09
BOND H53  C5     C10  H101
BOND H51  C5     C5   H52    C5   O7    H81  C7
BOND O7   C7     C7   H82    C7   C8    C8   O8
BOND C8   O9     O9   C10    H103 C10   C10  H102
IMPR C8     C7     O8     O9
IC H53  O7   *C5   H51       1.1122  109.08  119.24  110.54  1.1123
IC H53  O7   *C5   H52       1.1122  109.08 -119.30  110.60  1.1118
IC H53  C5   O7    C7        1.1122  109.08  179.99  111.55  1.4339
IC C5   O7   C7    C8        1.4204  111.55 -179.97  114.32  1.5422
IC C8   O7   *C7   H81       1.5422  114.32 -120.32  109.38  1.1093
IC H81  O7   *C7   H82       1.1093  109.38 -119.41  109.40  1.1100
IC O7   C7   C8    O9        1.4339  114.32  180.00  108.28  1.3435
IC O9   C7   *C8   O8        1.3435  108.28  179.94  127.41  1.2182
IC C7   C8   O9    C10       1.5422  108.28  179.98  112.08  1.4395
IC C8   O9   C10   H101      1.3435  112.08 -179.98  109.89  1.1119
```

```
IC H101 O9   *C10  H103      1.1119  109.89  119.55  110.84  1.1144
IC H101 O9   *C10  H102      1.1119  109.89 -119.65  110.85  1.1133

RESI MPYU           0.00 ! N1-(3-pyridinyl)-N2-methylurea, yxu
GROUP
ATOM C1     CG2R61 -0.115 !        O7
ATOM H1     HGR61   0.115 !        ||
GROUP                     !   H6   C7    C9
ATOM C2     CG2R61  0.180 !    \  /  \  /
ATOM H2     HGR62   0.120 !     N6    N8
ATOM N3     NG2R60 -0.600 !     |      |
ATOM C4     CG2R61  0.180 !     |     H8
ATOM H4     HGR62   0.120 !    C6
GROUP                     !   // \
ATOM C5     CG2R61 -0.115 !  C1   C5
ATOM H5     HGR61   0.115 !  |    ||
GROUP                     !  C2   C4
ATOM C6     CG2R61  0.09  !   \\ /
ATOM N6     NG2S1  -0.47  !    N3
ATOM H6     HGP1    0.30
ATOM C7     CG2O6   0.60
ATOM O7     OG2D1  -0.50
ATOM N8     NG2S1  -0.48
ATOM H8     HGP1    0.29
ATOM C9     CG331  -0.10
ATOM H91    HGA3    0.09
ATOM H92    HGA3    0.09
ATOM H93    HGA3    0.09
BOND H1   C1     H2   C2     C9   H93    C9   H91
BOND C9   N8     C1   C2     C1   C6     C2   N3
BOND H8   N8     O7   C7     N8   C7     C7   N6
BOND N6   C6     N6   H6     C6   C5     N3   C4
BOND C4   C5     C4   H4     C5   H5     H92  C9
IMPR C7     N8     N6     O7
IC C2   C6   *C1   H1        1.4023  118.55 -179.74  121.75  1.0762
IC C6   C1   C2    N3        1.4025  118.55   -0.72  121.71  1.3317
IC N3   C1   *C2   H2        1.3317  121.71 -179.48  120.34  1.0825
IC C1   C2   N3    C4        1.4023  121.71    0.46  120.73  1.3297
IC C2   N3   C4    C5        1.3317  120.73   -0.53  121.70  1.4016
IC C5   N3   *C4   H4        1.4016  121.70 -179.48  117.76  1.0840
IC C6   C4   *C5   H5        1.4041  118.60  179.33  120.42  1.0768
IC C5   C1   *C6   N6        1.4041  118.70  177.57  124.39  1.4188
IC C1   C6   N6    C7        1.4025  124.39   19.45  127.21  1.3650
IC C7   C6   *N6   H6        1.3650  127.21  175.93  115.18  0.9875
IC C6   N6   C7    N8        1.4188  127.21 -174.52  110.87  1.3629
IC N8   N6   *C7   O7        1.3629  110.87 -179.22  126.53  1.2233
IC N6   C7   N8    C9        1.3650  110.87  179.64  121.37  1.4444
IC C9   C7   *N8   H8        1.4444  121.37 -175.07  119.68  0.9905
IC C7   N8   C9    H93       1.3629  121.37 -176.13  110.73  1.1121
IC H93  N8   *C9   H91       1.1121  110.73  120.15  110.63  1.1142
IC H93  N8   *C9   H92       1.1121  110.73 -120.20  110.56  1.1135

RESI HPAT          -1.00 ! 2-hydroxyl propanoate, yxu
GROUP
ATOM C5     CG331  -0.27
ATOM H51    HGA3    0.09
ATOM H52    HGA3    0.09
ATOM H53    HGA3    0.09
GROUP
ATOM C7     CG311   0.14
ATOM H7     HGA1    0.09
ATOM O7     OG311  -0.65
ATOM HO7    HGP1    0.42
```

```
GROUP
ATOM C8      CG2O3   0.52
ATOM O81     OG2D2  -0.76
ATOM O82     OG2D2  -0.76
BOND C5   H51    C5   H52    C5   H53    C5   C7
BOND C7   H7     C7   O7     C7   C8     O7   HO7
BOND C8   O81    C8   O82
IMPR C8      O82    O81    C7
IC H51  C7   *C5   H52       1.1102  109.86  120.20  110.26  1.1080
IC H51  C7   *C5   H53       1.1102  109.86 -119.28  109.72  1.1112
IC H51  C5   C7    C8        1.1102  109.86   61.06  112.05  1.5662
IC C8   C5   *C7   O7        1.5662  112.05 -122.36  107.78  1.4330
IC O7   C5   *C7   H7        1.4330  107.78 -118.73  109.78  1.1094
IC C5   C7   O7    HO7       1.5433  107.78  122.45   97.38  0.9774
IC C5   C7   C8    O81       1.5433  112.05 -118.64  116.50  1.2646
IC O81  C7   *C8   O82       1.2646  116.50 -179.38  115.92  1.2591

RESI BZAC           -1.00 ! phenylacetate, yxu
GROUP
ATOM C1      CG2R61 -0.115
ATOM H1      HGR61   0.115
GROUP
ATOM C2      CG2R61 -0.115
ATOM H2      HGR61   0.115
GROUP
ATOM C3      CG2R61 -0.115
ATOM H3      HGR61   0.115
GROUP
ATOM C4      CG2R61 -0.115
ATOM H4      HGR61   0.115
GROUP
ATOM C6      CG2R61 -0.115
ATOM H6      HGR61   0.115
GROUP
ATOM C5      CG2R61  0.04
ATOM C7      CG321  -0.32
ATOM H71     HGA2    0.09
ATOM H72     HGA2    0.09
ATOM C8      CG2O3   0.62
ATOM O81     OG2D2  -0.76
ATOM O82     OG2D2  -0.76
BOND O81  C8     H4   C4     C8   O82    C8   C7
BOND H72  C7     C7   C5     C7   H71    C4   C3
BOND C4   C5     H3   C3     C3   C2     C5   C6
BOND C2   H2     C2   C1     C6   H6     C6   C1
BOND C1   H1
IMPR C8      O81    O82    C7
IC C2   C6   *C1   H1        1.4018  119.87  179.42  119.75  1.0793
IC C6   C1   C2    C3        1.4018  119.87    0.34  120.01  1.4017
IC C3   C1   *C2   H2        1.4017  120.01  179.34  119.97  1.0790
IC C1   C2   C3    C4        1.4018  120.01   -0.25  119.89  1.4025
IC C4   C2   *C3   H3        1.4025  119.89  179.64  120.27  1.0796
IC C2   C3   C4    C5        1.4017  119.89    0.17  120.47  1.4050
IC C5   C3   *C4   H4        1.4050  120.47  179.09  120.56  1.0791
IC C5   C1   *C6   H6        1.4039  120.55 -177.99  120.80  1.0799
IC C6   C4   *C5   C7        1.4039  119.19 -177.99  120.22  1.5091
IC C4   C5   C7    C8        1.4050  120.22 -107.51  112.60  1.5205
IC C8   C5   *C7   H72       1.5205  112.60  121.67  109.73  1.1092
IC C8   C5   *C7   H71       1.5205  112.60 -119.44  108.56  1.1095
IC C5   C7   C8    O81       1.5091  112.60  117.80  115.22  1.2586
IC O81  C7   *C8   O82       1.2586  115.22  178.63  116.64  1.2574

RESI 3PRU             0.00 ! N3-propyluracil, yxu
```

```
GROUP
ATOM N1     NG2R61 -0.57
ATOM H1     HGP1    0.32
ATOM C2     CG2R63  0.60
ATOM O2     OG2D4  -0.46
ATOM N3     NG2R61 -0.36
ATOM C4     CG2R63  0.58
ATOM O4     OG2D4  -0.51
ATOM C5     CG2R62 -0.17
ATOM H5     HGR62   0.07
ATOM C6     CG2R62  0.21
ATOM H6     HGR62   0.15
ATOM C7     CG321  -0.04
ATOM H71    HGA2    0.09
ATOM H72    HGA2    0.09
GROUP
ATOM C8     CG321  -0.18
ATOM H81    HGA2    0.09
ATOM H82    HGA2    0.09
GROUP
ATOM C9     CG331  -0.27
ATOM H91    HGA3    0.09
ATOM H92    HGA3    0.09
ATOM H93    HGA3    0.09
BOND H72  C7     H71  C7     C7   N3     C7   C8
BOND H91  C9     N3   C4     N3   C2     O4   C4
BOND C4   C5     C2   O2     C2   N1     C5   H5
BOND C5   C6     N1   C6     N1   H1     C6   H6
BOND C9   H93    C9   C8     C9   H92    C8   H82
BOND C8   H81
IMPR C2     N3     N1     O2       C4     C5     N3     O4
IC C6   C2   *N1   H1       1.3571  121.91  179.19  113.27  1.0057
IC C6   N1   C2    N3       1.3571  121.91    0.86  117.23  1.3906
IC N3   N1   *C2   O2       1.3906  117.23  180.00  119.75  1.2270
IC N1   C2   N3    C7       1.3768  117.23 -178.57  118.30  1.4742
IC C7   C2   *N3   C4       1.4742  118.30  176.44  122.88  1.4043
IC C2   N3   C4    O4       1.3906  122.88 -177.81  120.14  1.2308
IC O4   N3   *C4   C5       1.2308  120.14 -179.83  116.74  1.4318
IC C6   C4   *C5   H5       1.3639  119.29 -179.16  120.37  1.0881
IC C5   N1   *C6   H6       1.3639  121.91  179.67  117.67  1.0950
IC C2   N3   C7    C8       1.3906  118.30   88.50  112.29  1.5354
IC C8   N3   *C7   H72      1.5354  112.29  122.09  108.92  1.1147
IC H72  N3   *C7   H71      1.1147  108.92  115.97  108.85  1.1149
IC N3   C7   C8    C9       1.4742  112.29 -179.77  113.56  1.5326
IC C9   C7   *C8   H82      1.5326  113.56  121.59  108.89  1.1147
IC C9   C7   *C8   H81      1.5326  113.56 -121.44  108.78  1.1150
IC C7   C8   C9    H91      1.5354  113.56 -179.90  110.54  1.1111
IC H91  C8   *C9   H93      1.1111  110.54  119.99  110.48  1.1112
IC H91  C8   *C9   H92      1.1111  110.54 -119.99  110.47  1.1117

RESI PMMU           0.00 ! N1-(3-pyridinyl)-N1,N2-dimethylurea, yxu
GROUP
ATOM C1     CG2R61 -0.115 !   H61      O7
ATOM H1     HGR61   0.115 !      \     ||
GROUP                     !  H62-C60   C7    C9
ATOM C2     CG2R61  0.180 !      / \  /  \  /
ATOM H2     HGR62   0.120 !   H63   N6    N8
ATOM N3     NG2R60 -0.600 !         |     |
ATOM C4     CG2R61  0.180 !         |     H8
ATOM H4     HGR62   0.120 !         C6
GROUP                     !        // \
ATOM C5     CG2R61 -0.115 !       C1   C5
ATOM H5     HGR61   0.115 !       |    ||
```

```
GROUP                      !       C2   C4
ATOM C6      CG2R61  0.06  !         \\ /
ATOM N6      NG2S0  -0.34  !          N3
ATOM C60     CG331  -0.08
ATOM H61     HGA3    0.09
ATOM H62     HGA3    0.09
ATOM H63     HGA3    0.09
ATOM C7      CG2O6   0.59
ATOM O7      OG2D1  -0.54
ATOM N8      NG2S1  -0.44
ATOM H8      HGP1    0.29
ATOM C9      CG331  -0.08
ATOM H91     HGA3    0.09
ATOM H92     HGA3    0.09
ATOM H93     HGA3    0.09
BOND H62  C60    H63  C60    C2   H2
BOND H5   C5     C60  H61    C60  N6     H8   N8
BOND H92  C9     H4   C4     C5   C4     C5   C6
BOND N8   C9     N8   C7     H91  C9     N6   C6
BOND N6   C7     C4   N3     C9   H93    C6   C1
BOND C7   O7     N3   C2     C1   C2     C1   H1
IMPR C7     N6     N8     O7
IC C2   C6   *C1   H1       1.4008  119.75 -179.23  122.34  1.0750
IC C6   C1   C2    N3       1.4115  119.75   -1.09  121.92  1.3286
IC N3   C1   *C2   H2       1.3286  121.92 -178.90  120.34  1.0833
IC C1   C2   N3    C4       1.4008  121.92    0.93  120.09  1.3262
IC C2   N3   C4    C5       1.3286  120.09   -0.71  122.17  1.4020
IC C5   N3   *C4   H4       1.4020  122.17 -179.55  117.62  1.0843
IC C6   C4   *C5   H5       1.4129  119.48  179.59  117.27  1.0735
IC C5   C1   *C6   N6       1.4129  116.58  177.12  123.24  1.4507
IC C1   C6   N6    C7       1.4115  123.24    8.24  121.16  1.3828
IC C7   C6   *N6   C60      1.3828  121.16 -176.54  119.00  1.4675
IC C6   N6   C60   H62      1.4507  119.00 -171.36  112.17  1.1123
IC H62  N6   *C60  H63      1.1123  112.17  117.21  107.73  1.1122
IC H62  N6   *C60  H61      1.1123  112.17 -122.83  107.87  1.1113
IC C6   N6   C7    N8       1.4507  121.16 -144.03  112.73  1.3663
IC N8   N6   *C7   O7       1.3663  112.73  178.18  126.73  1.2258
IC N6   C7   N8    C9       1.3828  112.73  179.13  121.19  1.4438
IC C9   C7   *N8   H8       1.4438  121.19 -161.08  117.27  0.9912
IC C7   N8   C9    H92      1.3663  121.19 -177.79  110.74  1.1117
IC H92  N8   *C9   H91      1.1117  110.74  120.28  110.54  1.1146
IC H92  N8   *C9   H93      1.1117  110.74 -120.17  110.47  1.1139

RESI CENA           0.00 ! 1-(3-pyrrolyl)-N-(3-cyclopentenyl)-methyl amine, yxu
GROUP
ATOM N9      NG2R51 -0.38
ATOM H9      HGP1    0.39
ATOM C8      CG2R51 -0.04
ATOM H8      HGR52   0.16
ATOM C7      CG2R51 -0.11
ATOM C5      CG2R51 -0.25
ATOM H5      HGR51   0.13
ATOM C4      CG2R51 -0.04
ATOM H4      HGR52   0.14
ATOM C10     CG321   0.03
ATOM H101    HGA2    0.09
ATOM H102    HGA2    0.09
ATOM N11     NG311  -0.78
ATOM H11     HGPAM1  0.36
ATOM C12     CG3C51  0.12
ATOM H12     HGA1    0.09
GROUP
ATOM C13     CG3C52 -0.18
```

```
ATOM H131   HGA2    0.09
ATOM H132   HGA2    0.09
GROUP
ATOM C14    CG3C52 -0.18
ATOM H141   HGA2    0.09
ATOM H142   HGA2    0.09
GROUP
ATOM C15    CG2R51 -0.20
ATOM H15    HGR51   0.26
ATOM C16    CG2R51 -0.24
ATOM H16    HGR51   0.18
BOND H12  C12    H132 C13    H11  N11
BOND C12  N11    C12  C13    C12  C16    N11  C10
BOND C13  H131   C13  C14    H141 C14    H5   C5
BOND H4   C4     C5   C4     C5   C7     C4   N9
BOND C14  H142   C14  C15    C7   C10    C7   C8
BOND C16  H16    C16  C15    N9   C8     N9   H9
BOND C10  H101   C10  H102   C8   H8     C15  H15
IC C4   C8   *N9   H9       1.3735  109.09 -179.24  125.30  1.0113
IC C4   N9   C8    C7       1.3735  109.09   -0.28  107.18  1.3639
IC C7   N9   *C8   H8       1.3639  107.18  179.97  125.69  1.0828
IC C8   N9   C4    C5       1.3776  109.09   -0.01  106.27  1.3586
IC C5   N9   *C4   H4       1.3586  106.27 -179.74  124.36  1.0840
IC C7   C4   *C5   H5       1.3669  109.78 -180.00  125.15  1.0801
IC C5   C8   *C7   C10      1.3669  107.68  177.80  126.29  1.5171
IC C8   C7   C10   N11      1.3639  126.29   36.15  112.19  1.4799
IC N11  C7   *C10  H101     1.4799  112.19  122.86  108.41  1.1106
IC N11  C7   *C10  H102     1.4799  112.19 -120.90  108.33  1.1138
IC C7   C10  N11   C12      1.5171  112.19  175.50  112.83  1.4436
IC C12  C10  *N11  H11      1.4436  112.83 -124.87  111.36  1.0190
IC C10  N11  C12   C13      1.4799  112.83  170.04  113.20  1.5096
IC C13  N11  *C12  C16      1.5096  113.20  111.12  106.47  1.5173
IC C13  N11  *C12  H12      1.5096  113.20 -127.77  110.20  1.1039
IC N11  C12  C13   C14      1.4436  113.20  143.19  105.99  1.5242
IC C14  C12  *C13  H132     1.5242  105.99 -119.45  110.98  1.1052
IC H132 C12  *C13  H131     1.1052  110.98 -117.67  111.87  1.0997
IC C12  C13  C14   C15      1.5096  105.99  -28.50  102.47  1.5129
IC C15  C13  *C14  H141     1.5129  102.47 -122.35  111.85  1.1009
IC H141 C13  *C14  H142     1.1009  111.85 -118.58  110.76  1.1032
IC C16  C14  *C15  H15      1.3726  109.83  174.87  124.09  1.0821
IC C15  C12  *C16  H16      1.3726  110.70 -163.77  122.76  1.0794

RESI BZAA           0.00 ! phenylacetic acid, yxu
GROUP
ATOM C1     CG2R61 -0.115
ATOM H1     HGR61   0.115
GROUP
ATOM C2     CG2R61 -0.115
ATOM H2     HGR61   0.115
GROUP
ATOM C3     CG2R61 -0.115
ATOM H3     HGR61   0.115
GROUP
ATOM C4     CG2R61 -0.105
ATOM H4     HGR61   0.115
ATOM C5     CG2R61 -0.000
ATOM C6     CG2R61 -0.105
ATOM H6     HGR61   0.115
ATOM C7     CG321  -0.200
ATOM H71    HGA2    0.090
ATOM H72    HGA2    0.090
GROUP
ATOM C8     CG2O2   0.72
```

```
ATOM O8      OG2D1  -0.55
ATOM O9      OG311  -0.60
ATOM H9      HGP1    0.43
BOND O8   C8      H4   C4
BOND C8   O9      C8   C7      H72  C7      O9   H9
BOND C7   C5      C7   H71     C4   C3      C4   C5
BOND H3   C3      C3   C2      C5   C6      C2   H2
BOND C2   C1      C6   H6      C6   C1      C1   H1
IMPR C8      C7      O8      O9
IC C2   C6   *C1   H1        1.4007  120.00 -179.66  119.98  1.0805
IC C6   C1   C2    C3        1.4009  120.00   -0.10  119.98  1.4010
IC C3   C1   *C2   H2        1.4010  119.98 -179.69  120.00  1.0804
IC C1   C2   C3    C4        1.4007  119.98    0.13  119.99  1.4014
IC C4   C2   *C3   H3        1.4014  119.99 -179.77  119.98  1.0808
IC C2   C3   C4    C5        1.4010  119.99    0.03  120.32  1.4042
IC C5   C3   *C4   H4        1.4042  120.32 -179.77  119.84  1.0798
IC C5   C1   *C6   H6        1.4050  120.32  179.54  119.89  1.0802
IC C6   C4   *C5   C7        1.4050  119.39 -179.80  120.24  1.5032
IC C4   C5   C7    C8        1.4042  120.24   89.61  110.46  1.5161
IC C8   C5   *C7   H72       1.5161  110.46  120.21  109.28  1.1099
IC C8   C5   *C7   H71       1.5161  110.46 -120.76  109.05  1.1103
IC C5   C7   C8    O9        1.5032  110.46 -168.08  112.23  1.3761
IC O9   C7   *C8   O8        1.3761  112.23 -179.04  127.99  1.2169
IC C7   C8   O9    H9        1.5161  112.23 -179.71  107.20  0.9555

RESI 12MU           0.00 ! N1,N2-dimethylurea, yxu
GROUP
ATOM C1      CG331  -0.05
ATOM H11     HGA3    0.09
ATOM H12     HGA3    0.09
ATOM H13     HGA3    0.09
ATOM N1      NG2S1  -0.44
ATOM H1      HGP1    0.30
ATOM C       CG2O6   0.25
ATOM O       OG2D1  -0.41
ATOM N2      NG2S1  -0.44
ATOM H2      HGP1    0.30
ATOM C2      CG331  -0.05
ATOM H21     HGA3    0.09
ATOM H22     HGA3    0.09
ATOM H23     HGA3    0.09
BOND H13  C1      H12  C1      C1   H11
BOND C1   N1      H1   N1      N1   C       C    O
BOND C    N2      H2   N2      N2   C2      H23  C2
BOND C2   H22     C2   H21
IMPR C       N1      N2      O
IC H13  N1   *C1   H12       1.1116  110.68  120.25  110.37  1.1126
IC H13  N1   *C1   H11       1.1116  110.68 -120.25  110.37  1.1126
IC H13  C1   N1    C         1.1116  110.68  180.00  120.38  1.3611
IC C    C1   *N1   H1        1.3611  120.38  180.00  119.01  0.9925
IC C1   N1   C     N2        1.4395  120.38  180.00  112.54  1.3623
IC N2   N1   *C    O         1.3623  112.54  180.00  123.76  1.2241
IC N1   C    N2    C2        1.3611  112.54  180.00  120.41  1.4398
IC C2   C    *N2   H2        1.4398  120.41  180.00  120.54  0.9923
IC C    N2   C2    H23       1.3623  120.41  180.00  110.69  1.1112
IC H23  N2   *C2   H22       1.1112  110.69  120.28  110.37  1.1129
IC H23  N2   *C2   H21       1.1112  110.69 -120.28  110.37  1.1129

RESI PPOX           0.00 ! 1-(3-pyrrolyl)prop-2-yl hydrogen peroxide, yxu
GROUP
ATOM C1      CG2R51 -0.30 !                O9-H9
ATOM H1      HGR51   0.13 !               /
ATOM C2      CG2R51 -0.04 !     C6--C7--O8
```

```
ATOM H2      HGR52   0.14 !      |     |
ATOM N3      NG2R51 -0.38 !      |     C8
ATOM H3      HGP1    0.39 !      |
ATOM C4      CG2R51 -0.08 !      |
ATOM H4      HGR52   0.16 !   //C5--C1
ATOM C5      CG2R51 -0.02 !  C4      |
GROUP                     !    \N3==C2
ATOM C6      CG321  -0.18
ATOM H61     HGA2    0.09
ATOM H62     HGA2    0.09
GROUP
ATOM C7      CG311   0.17
ATOM H7      HGA1    0.09
ATOM O8      OG301  -0.25
ATOM O9      OG311  -0.43
ATOM H9      HGP1    0.42
GROUP
ATOM C8      CG331  -0.27
ATOM H81     HGA3    0.09
ATOM H82     HGA3    0.09
ATOM H83     HGA3    0.09
BOND H61  C6     H62  C6     H4   C4     C6   C5
BOND C6   C7     C4   C5     C4   N3     H81  C8
BOND C5   C1     N3   H3     N3   C2     C1   C2
BOND C1   H1     C2   H2     H83  C8     C8   C7
BOND C8   H82    C7   O8     C7   H7     O8   O9
BOND O9   H9
IC C2   C5   *C1   H1       1.3577  109.87 -179.95  124.88  1.0805
IC C5   C1   C2    N3       1.3632  109.87   -0.02  106.19  1.3749
IC N3   C1   *C2   H2       1.3749  106.19 -179.96  129.44  1.0842
IC C1   C2   N3    C4       1.3577  106.19   -0.02  109.03  1.3779
IC C4   C2   *N3   H3       1.3779  109.03 -179.95  125.22  1.0117
IC C5   N3   *C4   H4       1.3625  107.11  179.71  124.70  1.0820
IC C4   C1   *C5   C6       1.3625  107.81 -179.86  125.58  1.5089
IC C1   C5   C6    C7       1.3632  125.58   80.00  114.80  1.5504
IC C7   C5   *C6   H61      1.5504  114.80 -121.02  108.30  1.1120
IC H61  C5   *C6   H62      1.1120  108.30 -116.34  109.07  1.1103
IC C5   C6   C7    O8       1.5089  114.80 -168.71  110.02  1.4281
IC O8   C6   *C7   C8       1.4281  110.02 -125.02  113.26  1.5410
IC C8   C6   *C7   H7       1.5410  113.26 -119.07  107.95  1.1151
IC C6   C7   O8    O9       1.5504  110.02  -63.90  109.24  1.4713
IC C7   O8   O9    H9       1.4281  109.24  110.72   98.97  0.9616
IC C6   C7   C8    H81      1.5504  113.26   57.57  110.44  1.1088
IC H81  C7   *C8   H83      1.1088  110.44  119.54  109.81  1.1107
IC H81  C7   *C8   H82      1.1088  110.44 -120.41  110.46  1.1095

RESI HPME            0.00 ! 2-hydroxyl proponate methylester, yxu
GROUP
ATOM C5      CG331  -0.27
ATOM H51     HGA3    0.09
ATOM H52     HGA3    0.09
ATOM H53     HGA3    0.09
GROUP
ATOM C7      CG311   0.14
ATOM H7      HGA1    0.09
ATOM O7      OG311  -0.65
ATOM HO7     HGP1    0.42
GROUP
ATOM C8      CG2O2   0.86
ATOM O8      OG2D1  -0.63
ATOM O9      OG302  -0.49
ATOM C10     CG331  -0.01
ATOM H101    HGA3    0.09
```

```
ATOM H102   HGA3    0.09
ATOM H103   HGA3    0.09
BOND H102 C10    H103 C10    C10  H101
BOND C10  O9     O8   C8     O9   C8     C8   C7
BOND HO7  O7     C7   O7     C7   H7     C7   C5
BOND H51  C5     H53  C5     C5   H52
IMPR C8     C7     O8     O9
IC H51  C7   *C5   H53       1.1111  110.18  119.51  110.27  1.1108
IC H51  C7   *C5   H52       1.1111  110.18 -120.28  110.61  1.1096
IC H51  C5   C7    C8        1.1111  110.18  177.71  112.18  1.5830
IC C8   C5   *C7   O7        1.5830  112.18  124.55  107.46  1.4416
IC C8   C5   *C7   H7        1.5830  112.18 -118.02  108.85  1.1119
IC C5   C7   O7    HO7       1.5444  107.46 -158.16  107.11  0.9663
IC C5   C7   C8    O9        1.5444  112.18  149.77  110.79  1.3487
IC O9   C7   *C8   O8        1.3487  110.79  177.45  125.16  1.2194
IC C7   C8   O9    C10       1.5830  110.79  179.13  112.18  1.4385
IC C8   O9   C10   H102      1.3487  112.18  179.48  109.92  1.1127
IC H102 O9   *C10  H103      1.1127  109.92  119.58  110.87  1.1139
IC H102 O9   *C10  H101      1.1127  109.92 -119.55  110.90  1.1138

RESI OEBZ           0.00 ! phenoxyacetate methylester, yxu
GROUP
ATOM C1     CG2R61 -0.115
ATOM H1     HGR61   0.115
GROUP
ATOM C2     CG2R61 -0.115
ATOM H2     HGR61   0.115
GROUP
ATOM C3     CG2R61 -0.115
ATOM H3     HGR61   0.115
GROUP
ATOM C4     CG2R61 -0.115
ATOM H4     HGR61   0.115
GROUP
ATOM C6     CG2R61 -0.115
ATOM H6     HGR61   0.115
GROUP
ATOM C5     CG2R61  0.20
ATOM O7     OG301  -0.42
ATOM C7     CG321   0.04
ATOM H71    HGA2    0.09
ATOM H72    HGA2    0.09
GROUP
ATOM C8     CG2O2   0.86
ATOM O8     OG2D1  -0.63
ATOM O9     OG302  -0.49
ATOM C10    CG331  -0.01
ATOM H101   HGA3    0.09
ATOM H102   HGA3    0.09
ATOM H103   HGA3    0.09
BOND H2   C2     H3   C3     C2   C3
BOND C2   C1     C3   C4     H1   C1     C1   C6
BOND C4   H4     C4   C5     C6   C5     C6   H6
BOND C5   O7     H71  C7     O7   C7     C7   H72
BOND C7   C8     C8   O8     C8   O9     O9   C10
BOND H103 C10    C10  H102   C10  H101
IMPR C8     C7     O8     O9
IC C2   C6   *C1   H1        1.4002  119.87 -179.79  120.00  1.0808
IC C6   C1   C2    C3        1.3997  119.87    0.12  119.98  1.4007
IC C3   C1   *C2   H2        1.4007  119.98 -179.95  119.92  1.0800
IC C1   C2   C3    C4        1.4002  119.98   -0.29  120.04  1.4026
IC C4   C2   *C3   H3        1.4026  120.04 -179.91  119.88  1.0802
IC C2   C3   C4    C5        1.4007  120.04    0.11  120.29  1.4124
```

```
IC C5   C3   *C4   H4        1.4124  120.29  179.81  118.06  1.0776
IC C5   C1   *C6   H6        1.4003  120.89  179.36  120.34  1.0812
IC C6   C4   *C5   O7        1.4003  118.93 -179.72  123.05  1.4095
IC C4   C5   O7    C7        1.4124  123.05    6.97  117.52  1.4388
IC C5   O7   C7    C8        1.4095  117.52  172.97  113.95  1.5430
IC C8   O7   *C7   H71       1.5430  113.95 -119.09  109.96  1.1119
IC H71  O7   *C7   H72       1.1119  109.96 -120.87  110.02  1.1110
IC O7   C7   C8    O9        1.4388  113.95 -174.80  108.55  1.3421
IC O9   C7   *C8   O8        1.3421  108.55  179.42  127.21  1.2207
IC C7   C8   O9    C10       1.5430  108.55  176.70  112.23  1.4408
IC C8   O9   C10   H103      1.3421  112.23  -60.16  110.68  1.1132
IC H103 O9   *C10  H102      1.1132  110.68  120.86  110.92  1.1133
IC H103 O9   *C10  H101      1.1132  110.68 -119.45  109.90  1.1118

RESI ATBZ          -1.00 ! phenoxyacetate, yxu
GROUP
ATOM C1     CG2R61 -0.115
ATOM H1     HGR61   0.115
GROUP
ATOM C2     CG2R61 -0.115
ATOM H2     HGR61   0.115
GROUP
ATOM C3     CG2R61 -0.115
ATOM H3     HGR61   0.115
GROUP
ATOM C4     CG2R61 -0.115
ATOM H4     HGR61   0.115
GROUP
ATOM C6     CG2R61 -0.115
ATOM H6     HGR61   0.115
GROUP
ATOM C5     CG2R61  0.20
ATOM O7     OG301  -0.42
ATOM C7     CG321   0.04
ATOM H71    HGA2    0.09
ATOM H72    HGA2    0.09
GROUP
ATOM C8     CG2O3   0.52
ATOM O81    OG2D2  -0.76
ATOM O82    OG2D2  -0.76
BOND O82  C8     O81  C8     C8   C7
BOND H72  C7     C7   O7     C7   H71    O7   C5
BOND H4   C4     C5   C4     C5   C6     H6   C6
BOND C4   C3     C6   C1     C3   H3     C3   C2
BOND C1   C2     C1   H1     C2   H2
IMPR C8     O82    O81    C7
IC C2   C6   *C1   H1        1.4001  119.74  179.56  120.02  1.0800
IC C6   C1   C2    C3        1.4015  119.74    0.06  119.96  1.4010
IC C3   C1   *C2   H2        1.4010  119.96  179.39  120.12  1.0794
IC C1   C2   C3    C4        1.4001  119.96   -0.30  120.10  1.4028
IC C4   C2   *C3   H3        1.4028  120.10  179.32  120.29  1.0801
IC C2   C3   C4    C5        1.4010  120.10    0.46  120.39  1.4116
IC C5   C3   *C4   H4        1.4116  120.39  177.93  118.47  1.0770
IC C5   C1   *C6   H6        1.4018  121.08 -179.69  120.54  1.0790
IC C6   C4   *C5   O7        1.4018  118.73 -177.90  123.42  1.4131
IC C4   C5   O7    C7        1.4116  123.42    0.40  118.52  1.4481
IC C5   O7   C7    C8        1.4131  118.52  -70.39  119.56  1.5467
IC C8   O7   *C7   H72       1.5467  119.56  124.98  110.21  1.1100
IC C8   O7   *C7   H71       1.5467  119.56 -119.20  107.05  1.1100
IC O7   C7   C8    O82       1.4481  119.56  178.43  113.50  1.2625
IC O82  C7   *C8   O81       1.2625  113.50 -179.43  119.19  1.2550

RESI PYMU           0.00 ! N1-(4-pyrimidinyl)-N2-methylurea, yxu
```

```
GROUP
ATOM N1     NG2R62 -0.71 !          O7
ATOM C2     CG2R64  0.49 !          ||
ATOM H2     HGR62   0.12 !    H6    C7    C9
ATOM N3     NG2R62 -0.69 !      \  /  \  /
ATOM C4     CG2R61  0.17 !       N6    N8
ATOM H4     HGR62   0.17 !       |      |
ATOM C5     CG2R61 -0.10 !       |     H8
ATOM H5     HGR61   0.12 !       C6
ATOM C6     CG2R64  0.51 !      // \
ATOM N6     NG2S1  -0.47 !     N1   C5
ATOM H6     HGP1    0.30 !     |    ||
ATOM C7     CG2O6   0.60 !     C2   C4
ATOM O7     OG2D1  -0.50 !      \\ /
ATOM N8     NG2S1  -0.48 !       N3
ATOM H8     HGP1    0.29
ATOM C9     CG331  -0.09
ATOM H91    HGA3    0.09
ATOM H92    HGA3    0.09
ATOM H93    HGA3    0.09
BOND H92  C9     H2   C2     C4   H4
BOND N1   C2     N1   C6     H8   N8     C2   N3
BOND C9   H91    C9   N8     C9   H93    N8   C7
BOND N6   C7     N6   C6     N6   H6     C7   O7
BOND C6   C5     N3   C4     C5   C4     C5   H5
IMPR C7     N8     N6     O7
IC C6   N1   C2    N3       1.3560  117.73    0.00  124.86  1.3689
IC N3   N1   *C2   H2       1.3689  124.86  180.00  117.45  1.0922
IC N1   C2   N3    C4       1.3578  124.86    0.00  116.16  1.3323
IC C2   N3   C4    C5       1.3689  116.16    0.00  123.46  1.4063
IC C5   N3   *C4   H4       1.4063  123.46  180.00  116.72  1.0860
IC C6   C4   *C5   H5       1.4001  116.64  180.00  121.57  1.0735
IC C5   N1   *C6   N6       1.4001  121.15  180.00  123.80  1.4100
IC N1   C6   N6    C7       1.3560  123.80    0.00  127.21  1.3607
IC C7   C6   *N6   H6       1.3607  127.21  180.00  114.57  0.9815
IC C6   N6   C7    N8       1.4100  127.21  180.00  110.16  1.3632
IC N8   N6   *C7   O7       1.3632  110.16  180.00  128.14  1.2209
IC N6   C7   N8    C9       1.3607  110.16  180.00  121.14  1.4445
IC C9   C7   *N8   H8       1.4445  121.14  180.00  120.37  0.9914
IC C7   N8   C9    H92      1.3632  121.14  180.00  110.79  1.1116
IC H92  N8   *C9   H91      1.1116  110.79  120.22  110.54  1.1135
IC H92  N8   *C9   H93      1.1116  110.79 -120.22  110.54  1.1135

RESI ACBZ           0.00 ! carbomoylbenzene, yxu
GROUP
ATOM C1     CG2R61 -0.115
ATOM H1     HGR61   0.115
GROUP
ATOM C2     CG2R61 -0.115
ATOM H2     HGR61   0.115
GROUP
ATOM C3     CG2R61 -0.115
ATOM H3     HGR61   0.115
GROUP
ATOM C4     CG2R61 -0.120
ATOM H4     HGR61   0.115
ATOM C5     CG2R61 -0.000
ATOM C6     CG2R61 -0.120
ATOM H6     HGR61   0.115
ATOM C7     CG321  -0.170
ATOM H71    HGA2    0.090
ATOM H72    HGA2    0.090
GROUP
```

```
ATOM C8      CG2O1   0.55
ATOM O8      OG2D1  -0.55
ATOM N8      NG2S2  -0.62
ATOM H81     HGP1    0.30
ATOM H82     HGP1    0.32
BOND H82  N8     N8   H81    N8   C8
BOND O8   C8     H2   C2     H3   C3     C2   C3
BOND C2   C1     C8   C7     C3   C4     H1   C1
BOND C1   C6     C4   H4     C4   C5     C6   C5
BOND C6   H6     C5   C7     C7   H71    C7   H72
IMPR C8      C7      N8      O8
IC C2   C6   *C1   H1        1.4013  119.91 -179.80  119.99  1.0810
IC C6   C1   C2    C3        1.4008  119.91    0.14  120.07  1.4000
IC C3   C1   *C2   H2        1.4000  120.07  179.94  119.91  1.0808
IC C1   C2   C3    C4        1.4013  120.07   -0.19  119.96  1.4020
IC C4   C2   *C3   H3        1.4020  119.96 -179.98  119.98  1.0815
IC C2   C3   C4    C5        1.4000  119.96   -0.08  120.28  1.4043
IC C5   C3   *C4   H4        1.4043  120.28 -179.94  119.74  1.0801
IC C5   C1   *C6   H6        1.4044  120.35  179.19  119.98  1.0813
IC C6   C4   *C5   C7        1.4044  119.44 -178.19  120.55  1.5141
IC C4   C5   C7    C8        1.4043  120.55  101.27  112.93  1.4965
IC C8   C5   *C7   H71       1.4965  112.93  120.06  108.21  1.1129
IC C8   C5   *C7   H72       1.4965  112.93 -122.39  109.48  1.1117
IC C5   C7   C8    N8        1.5141  112.93  -53.73  118.77  1.3555
IC N8   C7   *C8   O8        1.3555  118.77 -178.27  120.20  1.2281
IC C7   C8   N8    H82       1.4965  118.77  176.33  116.06  0.9969
IC H82  C8   *N8   H81       0.9969  116.06 -166.20  119.08  0.9929

RESI AMBZ           0.00 ! N-methyl phenylethylamine, yxu
GROUP
ATOM C1      CG2R61 -0.115
ATOM H1      HGR61   0.115
GROUP
ATOM C2      CG2R61 -0.115
ATOM H2      HGR61   0.115
GROUP
ATOM C3      CG2R61 -0.115
ATOM H3      HGR61   0.115
GROUP
ATOM C4      CG2R61 -0.120
ATOM H4      HGR61   0.115
ATOM C5      CG2R61  0.010
ATOM C6      CG2R61 -0.120
ATOM H6      HGR61   0.115
GROUP
ATOM C7      CG321   0.00
ATOM H71     HGA2    0.09
ATOM H72     HGA2    0.09
ATOM N8      NG311  -0.66
ATOM H8      HGPAM1  0.37
ATOM C9      CG331  -0.16
ATOM H91     HGA3    0.09
ATOM H92     HGA3    0.09
ATOM H93     HGA3    0.09
BOND H1   C1     H92  C9     H6   C6     C1   C6
BOND C1   C2     N8   C9     N8   C7     C6   C5
BOND C9   H91    C9   H93    H2   C2     C2   C3
BOND C5   C7     C5   C4     C7   H71    C7   H72
BOND C3   C4     C3   H3     C4   H4     H8   N8
IC C2   C6   *C1   H1        1.4002  119.99  179.74  120.11  1.0807
IC C6   C1   C2    C3        1.4006  119.99    0.11  119.98  1.4002
IC C3   C1   *C2   H2        1.4002  119.98  179.85  120.02  1.0806
IC C1   C2   C3    C4        1.4002  119.98   -0.14  119.92  1.3997
```

```
IC C4   C2   *C3   H3        1.3997  119.92  179.86  120.02  1.0809
IC C2   C3   C4    C5        1.4002  119.92   -0.63  120.89  1.4080
IC C5   C3   *C4   H4        1.4080  120.89 -179.58  119.59  1.0798
IC C5   C1   *C6   H6        1.4107  120.71  179.79  119.09  1.0786
IC C6   C4   *C5   C7        1.4107  118.49  177.04  117.89  1.5226
IC C4   C5   C7    N8        1.4080  117.89  156.85  114.95  1.4832
IC N8   C5   *C7   H71       1.4832  114.95  121.19  106.51  1.1137
IC N8   C5   *C7   H72       1.4832  114.95 -122.79  108.75  1.1137
IC C5   C7   N8    C9        1.5226  114.95  -69.82  111.91  1.4679
IC C9   C7   *N8   H8        1.4679  111.91  120.22  112.51  1.0233
IC C7   N8   C9    H92       1.4832  111.91 -177.72  110.92  1.1135
IC H92  N8   *C9   H91       1.1135  110.92  119.21  110.76  1.1141
IC H92  N8   *C9   H93       1.1135  110.92 -120.54  111.85  1.1130

RESI NMGN           0.00 ! methylamino acetic acid, yxu
GROUP
ATOM C1     CG331  -0.06
ATOM H11    HGA3    0.09
ATOM H12    HGA3    0.09
ATOM H13    HGA3    0.09
ATOM N2     NG311  -0.78
ATOM H2     HGPAM1  0.36
ATOM C3     CG321   0.03
ATOM H31    HGA2    0.09
ATOM H32    HGA2    0.09
GROUP
ATOM C4     CG2O2   0.72
ATOM O4     OG2D1  -0.55
ATOM O5     OG311  -0.60
ATOM H5     HGP1    0.43
BOND H32  C3     H2   N2     H5   O5     N2   C3
BOND N2   C1     C3   C4     C3   H31    O5   C4
BOND C4   O4     H13  C1     C1   H12    C1   H11
IMPR C4     C3     O4     O5
IC H13  N2   *C1   H12       1.1143  110.81  119.13  110.91  1.1136
IC H13  N2   *C1   H11       1.1143  110.81 -120.34  111.58  1.1128
IC H13  C1   N2    C3        1.1143  110.81 -178.08  111.11  1.5031
IC C3   C1   *N2   H2        1.5031  111.11 -123.39  106.22  1.0269
IC C1   N2   C3    C4        1.4665  111.11 -177.97  118.56  1.5592
IC C4   N2   *C3   H32       1.5592  118.56 -122.93  108.54  1.1106
IC H32  N2   *C3   H31       1.1106  108.54 -116.60  108.79  1.1108
IC N2   C3   C4    O5        1.5031  118.56 -108.09  115.09  1.3873
IC O5   C3   *C4   O4        1.3873  115.09 -178.10  126.18  1.2196
IC C3   C4   O5    H5        1.5592  115.09  178.56  107.50  0.9560

RESI BZHE           0.00 ! 1-hydroxylethyl benzene, yxu
GROUP
ATOM C1     CG2R61 -0.115
ATOM H1     HGR61   0.115
GROUP
ATOM C2     CG2R61 -0.115
ATOM H2     HGR61   0.115
GROUP
ATOM C3     CG2R61 -0.115
ATOM H3     HGR61   0.115
GROUP
ATOM C4     CG2R61 -0.105
ATOM H4     HGR61   0.115
ATOM C5     CG2R61 -0.020
ATOM C6     CG2R61 -0.105
ATOM H6     HGR61   0.115
GROUP
ATOM C7     CG311   0.14
```

```
ATOM H7      HGA1    0.09
ATOM O7      OG311  -0.65
ATOM HO7     HGP1    0.42
GROUP
ATOM C8      CG331  -0.27
ATOM H81     HGA3    0.09
ATOM H82     HGA3    0.09
ATOM H83     HGA3    0.09
BOND H81  C8      H82  C8      C1   H1
BOND C8   H83     C8   C7      H4   C4      H7   C7
BOND C7   C5      C7   O7      C4   C5      C4   C3
BOND H3   C3      C5   C6      C3   C2      O7   HO7
BOND C6   H6      C6   C1      C2   C1      C2   H2
!IMPR C5  C4  C6  C7
IC C2   C6   *C1   H1        1.4003  120.02  179.84  120.02  1.0809
IC C6   C1   C2    C3        1.4012  120.02    0.14  119.99  1.4004
IC C3   C1   *C2   H2        1.4004  119.99  179.75  119.99  1.0803
IC C1   C2   C3    C4        1.4003  119.99   -0.15  119.96  1.4004
IC C4   C2   *C3   H3        1.4004  119.96  179.76  120.05  1.0801
IC C2   C3   C4    C5        1.4004  119.96   -0.32  120.61  1.4097
IC C5   C3   *C4   H4        1.4097  120.61  179.91  119.92  1.0802
IC C5   C1   *C6   H6        1.4069  120.57 -179.96  119.58  1.0800
IC C6   C4   *C5   C7        1.4069  118.84  178.34  121.60  1.5189
IC C4   C5   C7    O7        1.4097  121.60   39.29  114.14  1.4212
IC O7   C5   *C7   C8        1.4212  114.14 -118.54  107.53  1.5370
IC C8   C5   *C7   H7        1.5370  107.53 -118.37  111.25  1.1156
IC C5   C7   O7    HO7       1.5189  114.14   62.52  106.00  0.9596
IC C5   C7   C8    H81       1.5189  107.53 -178.34  109.95  1.1107
IC H81  C7   *C8   H82       1.1107  109.95  119.91  110.93  1.1105
IC H81  C7   *C8   H83       1.1107  109.95 -119.26  110.62  1.1105

RESI CPEA           1.00 ! 5-hydroxyl-cyclopenten-3-yl ammonium, yxu
GROUP
ATOM N6      NG3P3  -0.32
ATOM H61     HGP2    0.30
ATOM H62     HGP2    0.30
ATOM H63     HGP2    0.30
ATOM C1      CG3C53  0.33
ATOM H1      HGA1    0.09
GROUP
ATOM C2      CG3C52 -0.18
ATOM H21     HGA2    0.09
ATOM H22     HGA2    0.09
GROUP
ATOM C3      CG3C51  0.14
ATOM H3      HGA1    0.09
ATOM O3      OG311  -0.65
ATOM H3O     HGP1    0.42
GROUP
ATOM C4      CG2R51 -0.20
ATOM H4      HGR51   0.26
ATOM C5      CG2R51 -0.24
ATOM H5      HGR51   0.18
BOND H5   C5
BOND H4   C4      C5   C4      C5   C1      C4   C3
BOND H1   C1      C1   C2      C1   N6      H22  C2
BOND H3O  O3      C3   O3      C3   C2      C3   H3
BOND H63  N6      H62  N6      C2   H21     N6   H61

IC H63  C1   *N6   H62       1.0398  108.28  119.45  109.63  1.0388
IC H63  C1   *N6   H61       1.0398  108.28 -119.76  109.60  1.0392
IC H63  N6   C1    C2        1.0398  108.28   56.18  108.09  1.5178
IC C2   N6   *C1   C5        1.5178  108.09 -111.79  108.66  1.4968
```

```
IC C5   N6   *C1   H1        1.4968  108.66 -122.53  108.07  1.0857
IC N6   C1   C2    C3        1.4830  108.09 -145.04  104.54  1.5083
IC C3   C1   *C2   H22       1.5083  104.54 -121.69  111.11  1.1037
IC H22  C1   *C2   H21       1.1037  111.11 -119.32  113.63  1.1032
IC C1   C2   C3    O3        1.5178  104.54  -92.22  109.32  1.4139
IC O3   C2   *C3   C4        1.4139  109.32  120.79  102.87  1.5126
IC C4   C2   *C3   H3        1.5126  102.87  119.15  111.46  1.1056
IC C2   C3   O3    H3O       1.5083  109.32 -168.98  110.90  0.9633
IC C5   C3   *C4   H4        1.3735  110.34 -177.72  122.79  1.0829
IC C4   C1   *C5   H5        1.3735  109.26  173.51  125.46  1.0835

RESI CYBZ           0.00 ! cyanomethylbenzene, yxu
GROUP
ATOM C1     CG2R61 -0.115
ATOM H1     HGR61   0.115
GROUP
ATOM C2     CG2R61 -0.115
ATOM H2     HGR61   0.115
GROUP
ATOM C3     CG2R61 -0.115
ATOM H3     HGR61   0.115
GROUP
ATOM C4     CG2R61 -0.105
ATOM H4     HGR61   0.115
ATOM C5     CG2R61 -0.020
ATOM C6     CG2R61 -0.105
ATOM H6     HGR61   0.115
GROUP
ATOM C7     CG321  -0.08
ATOM H71    HGA2    0.09
ATOM H72    HGA2    0.09
ATOM C8     CG1N1   0.36
ATOM N9     NG1T1  -0.46
BOND H2   C2     C1   C2     C1   C6     C2   C3
BOND H6   C6     C6   C5     C3   H3     C3   C4
BOND C5   C4     C5   C7     C4   H4     H72  C7
BOND N9   C8     C8   C7     C7   H71    H1   C1
IC C2   C6   *C1   H1        1.3996  120.00  179.89  119.95  1.0807
IC C6   C1   C2    C3        1.4008  120.00    0.08  119.99  1.4003
IC C3   C1   *C2   H2        1.4003  119.99  179.89  120.02  1.0803
IC C1   C2   C3    C4        1.3996  119.99   -0.14  120.04  1.4010
IC C4   C2   *C3   H3        1.4010  120.04  179.98  119.97  1.0816
IC C2   C3   C4    C5        1.4003  120.04   -0.39  120.51  1.4064
IC C5   C3   *C4   H4        1.4064  120.51 -179.71  119.50  1.0798
IC C5   C1   *C6   H6        1.4089  120.51  179.61  119.48  1.0809
IC C6   C4   *C5   C7        1.4089  118.95  177.74  119.78  1.5113
IC C4   C5   C7    C8        1.4064  119.78  136.02  115.39  1.4705
IC C8   C5   *C7   H72       1.4705  115.39 -121.42  109.38  1.1103
IC H72  C5   *C7   H71       1.1103  109.38 -117.78  107.46  1.1100
IC C5   C7   C8    N9        1.5113  115.39  141.55  178.91  1.1787

RESI NMCY           0.00 ! 2-methylamino-4-imino-pyrimidine, yxu
GROUP
ATOM N1     NG2R61 -0.64
ATOM H1     HGP1    0.33
ATOM C2     CG2R64  0.85
ATOM N2     NG311  -0.59
ATOM H2     HGPAM1  0.37
ATOM N3     NG2R62 -0.73
ATOM C4     CG2R64  0.63
ATOM N4     NG2D1  -0.89
ATOM H4     HGP1    0.32
ATOM C5     CG2R61 -0.21
```

```
ATOM H5     HGR61   0.14
ATOM C6     CG2R61  0.10
ATOM H6     HGR62   0.15
ATOM C7     CG331  -0.10
ATOM H71    HGA3    0.09
ATOM H72    HGA3    0.09
ATOM H73    HGA3    0.09
BOND N1   C2     N1   C6     N1   H1     C2   N2
BOND C2   N3     N2   H2     N2   C7     N3   C4
BOND C4   N4     C4   C5     N4   H4     C5   H5
BOND C5   C6     C6   H6     C7   H71    C7   H72
BOND C7   H73
IMPR C2     N1     N3     N2     C4   C5   N4   N3
IC C6   C2   *N1   H1        1.3933  120.26  173.68  114.47  1.0016
IC C6   N1   C2    N3        1.3933  120.26    1.14  122.03  1.3317
IC N3   N1   *C2   N2        1.3317  122.03  178.55  116.35  1.3547
IC N1   C2   N2    C7        1.3873  116.35 -159.04  121.96  1.4842
IC C7   C2   *N2   H2        1.4842  121.96  132.64  111.56  1.0102
IC N1   C2   N3    C4        1.3873  122.03   -0.41  119.74  1.3820
IC C2   N3   C4    N4        1.3317  119.74  179.59  121.48  1.3288
IC N4   N3   *C4   C5        1.3288  121.48 -179.88  120.29  1.4294
IC N3   C4   N4    H4        1.3820  121.48  179.94  109.99  0.9934
IC C6   C4   *C5   H5        1.3915  118.79 -179.78  119.85  1.0750
IC C5   N1   *C6   H6        1.3915  118.88  179.84  119.16  1.0842
IC C2   N2   C7    H71       1.3547  121.96 -172.00  110.91  1.1120
IC H71  N2   *C7   H72       1.1120  110.91  119.35  111.08  1.1140
IC H71  N2   *C7   H73       1.1120  110.91 -120.76  111.26  1.1133

RESI MAES           0.00 ! 2-methylamino ethyl sulfate, yxu
GROUP
ATOM C7     CG334   0.05
ATOM H71    HGA3    0.09
ATOM H72    HGA3    0.09
ATOM H73    HGA3    0.09
ATOM N8     NG3P2  -0.30
ATOM H81    HGP2    0.33
ATOM H82    HGP2    0.33
ATOM C9     CG324   0.14
ATOM H91    HGA2    0.09
ATOM H92    HGA2    0.09
GROUP
ATOM C10    CG321  -0.26
ATOM H101   HGA2    0.09
ATOM H102   HGA2    0.09
ATOM S11    SG3O1   0.73
ATOM O111   OG2P1  -0.55
ATOM O112   OG2P1  -0.55
ATOM O113   OG2P1  -0.55
BOND O113 S11    H101 C10    H92  C9     C7   H72
BOND H71  C7     H81  N8     O111 S11    S11  C10
BOND S11  O112   C10  C9     C10  H102   C9   N8
BOND C9   H91    N8   C7     N8   H82    H73  C7
IC H72  N8   *C7   H71       1.1099  107.27  120.51  106.99  1.1104
IC H72  N8   *C7   H73       1.1099  107.27 -120.17  106.69  1.1117
IC H72  C7   N8    C9        1.1099  107.27   61.52  117.14  1.5190
IC C9   C7   *N8   H81       1.5190  117.14 -127.83  113.33  1.0057
IC H81  C7   *N8   H82       1.0057  113.33 -114.92  110.99  1.0278
IC C7   N8   C9    C10       1.4936  117.14  160.02  113.37  1.5364
IC C10  N8   *C9   H92       1.5364  113.37 -121.49  104.63  1.1058
IC H92  N8   *C9   H91       1.1058  104.63 -114.86  105.22  1.1011
IC N8   C9   C10   S11       1.5190  113.37  -52.49  110.29  1.7941
IC S11  C9   *C10  H101      1.7941  110.29  119.73  112.42  1.1074
IC H101 C9   *C10  H102      1.1074  112.42  121.99  110.53  1.1089
```

```
IC C9   C10  S11   O113      1.5364  110.29 -172.26  104.28  1.4387
IC O113 C10  *S11  O111      1.4387  104.28  121.39  101.17  1.4441
IC O113 C10  *S11  O112      1.4387  104.28 -121.02  101.61  1.4489

RESI PONM           1.00 ! 3-methylamino epoxypentene, yxu
GROUP
ATOM C1     CG3C53  0.34
ATOM H1     HGA1    0.09
ATOM N6     NG3P2  -0.36
ATOM H61    HGP2    0.29
ATOM H62    HGP2    0.29
ATOM C7     CG334   0.08
ATOM H71    HGA3    0.09
ATOM H72    HGA3    0.09
ATOM H73    HGA3    0.09
GROUP
ATOM C2     CG3C52 -0.18
ATOM H21    HGA2    0.09
ATOM H22    HGA2    0.09
GROUP
ATOM C3     CG3C52 -0.18
ATOM H31    HGA2    0.09
ATOM H32    HGA2    0.09
GROUP
ATOM C4     CG3RC1  0.15
ATOM H4     HGA1    0.09
ATOM O4     OG3C31 -0.25
ATOM C5     CG3RC1 -0.08
ATOM H5     HGA1    0.09
BOND H21  C2
BOND H32  C3     H31  C3     C3   C2     C3   C4
BOND H61  N6     C2   H22    C2   C1     N6   H62
BOND N6   C1     N6   C7     C1   H1     C1   C5
BOND H71  C7     C4   H4     C4   O4     C4   C5
BOND C7   H72    C7   H73    O4   C5     C5   H5

IC C2   C5   *C1   N6        1.5535  104.16  116.76  108.71  1.4992
IC C2   C5   *C1   H1        1.5535  104.16 -125.43  110.14  1.0857
IC C5   C1   N6    C7        1.5116  108.71  172.89  118.92  1.5018
IC C7   C1   *N6   H61       1.5018  118.92 -125.26  105.73  1.0088
IC H61  C1   *N6   H62       1.0088  105.73 -109.92  104.37  1.0096
IC C1   N6   C7    H71       1.4992  118.92 -177.43  106.90  1.1118
IC H71  N6   *C7   H72       1.1118  106.90  119.65  107.32  1.1100
IC H71  N6   *C7   H73       1.1118  106.90 -119.68  107.80  1.1088
IC C5   C1   C2    C3        1.5116  104.16   25.90  107.36  1.5347
IC C3   C1   *C2   H21       1.5347  107.36 -116.79  107.11  1.1054
IC H21  C1   *C2   H22       1.1054  107.11 -116.56  115.00  1.0966
IC C1   C2   C3    C4        1.5535  107.36  -25.37  102.77  1.5047
IC C4   C2   *C3   H32       1.5047  102.77  119.15  113.07  1.1022
IC H32  C2   *C3   H31       1.1022  113.07  120.70  111.98  1.1032
IC C5   C3   *C4   O4        1.4883  111.24   64.57  113.80  1.4283
IC O4   C3   *C4   H4        1.4283  113.80  143.61  121.69  1.1030
IC C4   C1   *C5   H5        1.4883  107.50  150.11  122.49  1.0767

RESI MMMU           0.00 ! N1,N1,N2-trimethylurea, yxu
GROUP
ATOM C1     CG331  -0.10
ATOM H11    HGA3    0.09
ATOM H12    HGA3    0.09
ATOM H13    HGA3    0.09
ATOM N1     NG2S1  -0.44
ATOM H1     HGP1    0.36
ATOM C      CG2O6   0.27
```

```
ATOM O      OG2D1  -0.42
ATOM N2     NG2S0  -0.30
ATOM C2     CG331  -0.09
ATOM H21    HGA3    0.09
ATOM H22    HGA3    0.09
ATOM H23    HGA3    0.09
ATOM C3     CG331  -0.09
ATOM H31    HGA3    0.09
ATOM H32    HGA3    0.09
ATOM H33    HGA3    0.09
BOND H13  C1     H12  C1     C1   H11    C1   N1
BOND H1   N1     N1   C      H32  C3     H31  C3
BOND C3   H33    C3   N2     C    O      C    N2
BOND N2   C2     H23  C2     C2   H22    C2   H21
IMPR C      N2     N1     O
IC H13  N1   *C1   H12      1.1110  110.94  120.29  110.28  1.1138
IC H13  N1   *C1   H11      1.1110  110.94 -120.49  110.41  1.1137
IC H13  C1   N1    C        1.1110  110.94 -177.72  120.98  1.3664
IC C    C1   *N1   H1       1.3664  120.98  179.46  119.92  0.9911
IC C1   N1   C     N2       1.4415  120.98 -178.56  113.96  1.3755
IC N2   N1   *C    O        1.3755  113.96  179.31  121.62  1.2250
IC N1   C    N2    C3       1.3664  113.96  168.25  116.62  1.4516
IC C3   C    *N2   C2       1.4516  116.62 -159.93  122.36  1.4498
IC C    N2   C2    H23      1.3755  122.36 -168.06  107.01  1.1108
IC H23  N2   *C2   H22      1.1108  107.01  118.10  110.10  1.1115
IC H23  N2   *C2   H21      1.1108  107.01 -118.71  107.81  1.1112
IC C    N2   C3    H32      1.3755  116.62  149.68  107.35  1.1089
IC H32  N2   *C3   H31      1.1089  107.35  119.38  107.34  1.1106
IC H32  N2   *C3   H33      1.1089  107.35 -119.43  110.64  1.1137

RESI CPOA           1.00 ! epoxycyclopenten-3-yl ammonium, yxu
GROUP
ATOM N6     NG3P3  -0.34
ATOM H61    HGP2    0.31
ATOM H62    HGP2    0.31
ATOM H63    HGP2    0.31
ATOM C1     CG3C53  0.32
ATOM H1     HGA1    0.09
GROUP
ATOM C2     CG3C52 -0.18
ATOM H21    HGA2    0.09
ATOM H22    HGA2    0.09
GROUP
ATOM C3     CG3C52 -0.18
ATOM H31    HGA2    0.09
ATOM H32    HGA2    0.09
GROUP
ATOM C4     CG3RC1  0.16
ATOM H4     HGA1    0.09
ATOM O4     OG3C31 -0.25
ATOM C5     CG3RC1 -0.09
ATOM H5     HGA1    0.09
BOND H21  C2     H32  C3     H31  C3     C3   C2
BOND C3   C4     H61  N6     C2   H22    C2   C1
BOND N6   H62    N6   C1     N6   H63    C1   H1
BOND C1   C5     C4   H4     C4   O4     C4   C5
BOND O4   C5     C5   H5
IC H61  C1   *N6   H62      1.0381  110.56  119.42  107.00  1.0426
IC H61  C1   *N6   H63      1.0381  110.56 -122.31  110.28  1.0385
IC H61  N6   C1    C5       1.0381  110.56 -171.20  107.55  1.5024
IC C5   N6   *C1   C2       1.5024  107.55  113.78  109.71  1.5531
IC C2   N6   *C1   H1       1.5531  109.71  128.00  108.34  1.0879
IC N6   C1   C2    C3       1.4860  109.71 -144.28  105.66  1.5361
```

```
IC C3   C1   *C2   H21       1.5361  105.66  117.82  111.29  1.1037
IC H21  C1   *C2   H22       1.1037  111.29  119.25  112.13  1.0990
IC C1   C2   C3    C4        1.5531  105.66   27.61  102.63  1.5078
IC C4   C2   *C3   H32       1.5078  102.63  120.66  112.75  1.1007
IC H32  C2   *C3   H31       1.1007  112.75  121.16  112.23  1.1026
IC C5   C3   *C4   O4        1.4812  111.43   64.43  113.38  1.4360
IC O4   C3   *C4   H4        1.4360  113.38  143.00  121.65  1.1037
IC C4   C1   *C5   H5        1.4812  106.60  148.98  123.18  1.0768

RESI NMGI           0.00 ! carboxymethyl methyl ammonium, yxu
GROUP
ATOM C7     CG334  -0.03 !      H72
ATOM H71    HGA3    0.09 !      |
ATOM H72    HGA3    0.09 !  H71-C7 -H73
ATOM H73    HGA3    0.09 !      |(+)
ATOM N8     NG3P2  -0.33 !  H81-N8 -H82
ATOM H81    HGP2    0.32 !      |
ATOM H82    HGP2    0.32 !  H91-C9 -H92
ATOM C9     CG324   0.02 !      |
ATOM H91    HGA2    0.09 !      O11=C10
ATOM H92    HGA2    0.09 !      |
ATOM C10    CG2O3   0.61 !      O12(-)
ATOM O101   OG2D2  -0.68
ATOM O102   OG2D2  -0.68
BOND H82  N8     H92  C9     N8   H81    N8   C9
BOND N8   C7     H73  C7     H72  C7     C9   C10
BOND C9   H91    O101 C10    C7   H71    C10  O102
IMPR C10    O102   O101   C9
IC H73  N8   *C7   H72       1.1112  106.64  119.57  107.10  1.1102
IC H73  N8   *C7   H71       1.1112  106.64 -119.94  107.14  1.1094
IC H73  C7   N8    C9        1.1112  106.64   55.60  115.62  1.5304
IC C9   C7   *N8   H82       1.5304  115.62 -113.53  109.08  1.0253
IC H82  C7   *N8   H81       1.0253  109.08 -117.52  113.45  1.0051
IC C7   N8   C9    C10       1.4969  115.62 -108.42  109.83  1.5345
IC C10  N8   *C9   H92       1.5345  109.83  120.15  106.96  1.0957
IC H92  N8   *C9   H91       1.0957  106.96  120.42  106.58  1.0952
IC N8   C9   C10   O101      1.5304  109.83  177.45  114.74  1.2517
IC O101 C9   *C10  O102      1.2517  114.74  179.58  116.53  1.2606

RESI 15HE           0.00 ! 1,5-hexadiene, yxu
GROUP
ATOM C1     CG2D2  -0.42
ATOM H11    HGA5    0.21
ATOM H12    HGA5    0.21
GROUP
ATOM C2     CG2D1  -0.15
ATOM H2     HGA4    0.15
GROUP
ATOM C3     CG321  -0.18
ATOM H31    HGA2    0.09
ATOM H32    HGA2    0.09
GROUP
ATOM C4     CG321  -0.18
ATOM H41    HGA2    0.09
ATOM H42    HGA2    0.09
GROUP
ATOM C5     CG2D1  -0.15
ATOM H5     HGA4    0.15
GROUP
ATOM C6     CG2D2  -0.42
ATOM H61    HGA5    0.21
ATOM H62    HGA5    0.21
BOND C1   H12    C1   C2     C2   H2     C2   C3
```

```
BOND C3   H31    C3   H32    C3   C4     C4   H41
BOND C4   H42    C4   C5     C5   H5     C5   C6
BOND C6   H61    C6   H62    C1   H11
IC H12  C2   *C1   H11       1.1009  121.13  179.71  120.48  1.1010
IC H12  C1   C2    C3        1.1009  121.13 -179.70  126.01  1.5067
IC C3   C1   *C2   H2        1.5067  126.01  179.54  118.59  1.1028
IC C1   C2   C3    C4        1.3435  126.01  117.81  111.95  1.5470
IC C4   C2   *C3   H31       1.5470  111.95 -121.89  110.87  1.1138
IC H31  C2   *C3   H32       1.1138  110.87 -117.47  109.33  1.1136
IC C2   C3   C4    C5        1.5067  111.95 -180.00  111.95  1.5067
IC C5   C3   *C4   H41       1.5067  111.95 -122.95  108.98  1.1143
IC H41  C3   *C4   H42       1.1143  108.98 -116.11  108.81  1.1136
IC C3   C4   C5    C6        1.5470  111.95 -117.82  126.01  1.3445
IC C6   C4   *C5   H5        1.3445  126.01  179.58  115.47  1.1033
IC C4   C5   C6    H61       1.5067  126.01  179.70  121.14  1.1009
IC H61  C5   *C6   H62       1.1009  121.14 -179.72  120.44  1.0997

RESI IDAM           0.00 ! 3-aminomethyl indole, yxu
GROUP
ATOM N9     NG2R51 -0.52 !                      H111
ATOM H9     HGP1    0.36 !                      /
ATOM C8     CG2R51 -0.15 !      C6       C10--N11
ATOM H8     HGR52   0.21 !     // \     /       \
ATOM C7     CG2R51 -0.10 !    C1   C5--C7\\      H113
ATOM C5     CG2RC0  0.16 !    |    ||     C8
ATOM C6     CG2R61 -0.26 !    C2   C4--N9/
ATOM H6     HGR61   0.20 !     \\ /
ATOM C1     CG2R61 -0.24 !      C3
ATOM H1     HGR61   0.20
ATOM C2     CG2R61 -0.20
ATOM H2     HGR61   0.20
ATOM C3     CG2R61 -0.28
ATOM H3     HGR61   0.19
ATOM C4     CG2RC0  0.23
GROUP
ATOM C10    CG321   0.04
ATOM H101   HGA2    0.09
ATOM H102   HGA2    0.09
ATOM N11    NG321  -0.90
ATOM H111   HGPAM2  0.34
ATOM H112   HGPAM2  0.34
BOND H9   N9     H8   C8     N9   C8     N9   C4
BOND C8   C7     H3   C3     H101 C10    C4   C3
BOND C4   C5     H102 C10    C7   C10    C7   C5
BOND C3   C2     C10  N11    C5   C6     C2   H2
BOND C2   C1     C6   C1     C6   H6     C1   H1
BOND N11  H111   N11  H112
IC C8   C4   *N9   H9        1.3878  110.89  179.87  122.58  1.0088
IC C4   N9   C8    C7        1.3717  110.89   -0.63  107.85  1.3711
IC C7   N9   *C8   H8        1.3711  107.85 -179.59  124.20  1.0849
IC H9   N9   C4    C5        1.0088  122.58 -179.77  106.28  1.4029
IC C5   N9   *C4   C3        1.4029  106.28 -179.68  132.65  1.3845
IC C7   C4   *C5   C6        1.4386  107.88 -179.97  119.73  1.3925
IC C4   C5   C6    C1        1.4029  119.73    0.16  119.67  1.4014
IC C1   C5   *C6   H6        1.4014  119.67 -179.00  118.89  1.0811
IC C5   C6   C1    C2        1.3925  119.67    0.04  120.15  1.4018
IC C2   C6   *C1   H1        1.4018  120.15 -179.77  119.88  1.0827
IC C3   C1   *C2   H2        1.4017  120.06 -179.70  119.81  1.0831
IC C2   C4   *C3   H3        1.4017  119.32 -179.90  119.70  1.0782
IC C5   C8   *C7   C10       1.4386  107.10  176.58  127.93  1.5092
IC C8   C7   C10   N11       1.3711  127.93  122.70  110.12  1.4841
IC N11  C7   *C10  H101      1.4841  110.12 -121.77  109.92  1.1140
IC H101 C7   *C10  H102      1.1140  109.92 -117.74  108.62  1.1147
```

```
IC C7   C10  N11   H111      1.5092  110.12  -54.10  112.42  1.0157
IC H111 C10  *N11  H112      1.0157  112.42 -120.69  113.84  1.0149

RESI MEBZ           0.00 ! phenylacetate methylester, yxu
GROUP
ATOM C1     CG2R61 -0.115
ATOM H1     HGR61   0.115
GROUP
ATOM C2     CG2R61 -0.115
ATOM H2     HGR61   0.115
GROUP
ATOM C3     CG2R61 -0.115
ATOM H3     HGR61   0.115
GROUP
ATOM C4     CG2R61 -0.115
ATOM H4     HGR61   0.115
GROUP
ATOM C6     CG2R61 -0.115
ATOM H6     HGR61   0.115
GROUP
ATOM C5     CG2R61 -0.00
ATOM C7     CG321  -0.22
ATOM H71    HGA2    0.09
ATOM H73    HGA2    0.09
ATOM C8     CG2O2   0.90
ATOM O8     OG2D1  -0.63
ATOM O9     OG302  -0.49
ATOM C9     CG331  -0.01
ATOM H91    HGA3    0.09
ATOM H92    HGA3    0.09
ATOM H93    HGA3    0.09
BOND O8   C8     H92  C9     H4   C4     H93  C9
BOND C8   C7     C8   O9     H73  C7     C9   O9
BOND C9   H91    C7   H71    C7   C5     C4   C3
BOND C4   C5     H3   C3     C3   C2     C5   C6
BOND C2   H2     C2   C1     C6   H6     C6   C1
BOND C1   H1
IMPR C8     C7     O8     O9
IC C2   C6   *C1   H1        1.4015  119.95 -179.79  119.98  1.0803
IC C6   C1   C2    C3        1.4018  119.95   -0.11  120.05  1.4009
IC C3   C1   *C2   H2        1.4009  120.05 -179.79  119.97  1.0803
IC C1   C2   C3    C4        1.4015  120.05    0.23  119.93  1.4017
IC C4   C2   *C3   H3        1.4017  119.93 -179.83  120.07  1.0803
IC C2   C3   C4    C5        1.4009  119.93    0.08  120.28  1.4035
IC C5   C3   *C4   H4        1.4035  120.28 -179.53  120.01  1.0798
IC C5   C1   *C6   H6        1.4040  120.23  179.86  119.89  1.0802
IC C6   C4   *C5   C7        1.4040  119.56 -178.72  120.13  1.5008
IC C4   C5   C7    C8        1.4035  120.13   84.50  109.30  1.5138
IC C8   C5   *C7   H73       1.5138  109.30  119.13  109.81  1.1092
IC C8   C5   *C7   H71       1.5138  109.30 -120.59  109.67  1.1094
IC C5   C7   C8    O9        1.5008  109.30  -82.42  110.04  1.3365
IC O9   C7   *C8   O8        1.3365  110.04 -178.16  124.81  1.2168
IC C7   C8   O9    C9        1.5138  110.04 -179.76  112.52  1.4380
IC C8   O9   C9    H92       1.3365  112.52 -179.24  109.71  1.1123
IC H92  O9   *C9   H93       1.1123  109.71  119.49  110.89  1.1133
IC H92  O9   *C9   H91       1.1123  109.71 -119.50  110.89  1.1134

RESI AMBA           0.00 ! 2-carboxamide-3-methoxy butyraldehyde, yxu
GROUP
ATOM C4     CG331  -0.27
ATOM H41    HGA3    0.09
ATOM H42    HGA3    0.09
ATOM H43    HGA3    0.09
```

```
GROUP
ATOM C5     CG311   0.08
ATOM H5     HGA1    0.09
ATOM O5     OG301  -0.34
ATOM C6     CG331  -0.10
ATOM H61    HGA3    0.09
ATOM H62    HGA3    0.09
ATOM H63    HGA3    0.09
GROUP
ATOM C7     CG311   0.18
ATOM C10    CG2O4   0.20
ATOM O10    OG2D1  -0.40
ATOM H10    HGR52   0.09
ATOM H7     HGA1    0.09
ATOM N8     NG2S1  -0.47
ATOM H8     HGP1    0.31
GROUP
ATOM C9     CG2O1   0.43
ATOM O9     OG2D1  -0.51
ATOM H9     HGR52   0.08
BOND H5   C5     O9   C9     C4   H43    C4   C5
BOND C4   H41    C7   C5     C7   N8     C7   H7
BOND C9   N8     C9   H9     C5   O5     N8   H8
BOND H61  C6     O5   C6     C6   H62    C6   H63
BOND C10  C7     O10  C10    H10  C10    C4   H42
IMPR C9     N8     O9     H9       C10    C7     O10    H10
IC H43  C5   *C4   H41       1.1084  111.00  120.17  110.48  1.1107
IC H43  C5   *C4   H42       1.1084  111.00 -119.67  110.53  1.1099
IC H43  C4   C5    C7        1.1084  111.00  173.87  108.76  1.5203
IC C7   C4   *C5   O5        1.5203  108.76  119.86  111.02  1.4245
IC C7   C4   *C5   H5        1.5203  108.76 -118.70  109.13  1.1158
IC C4   C5   O5    C6        1.5445  111.02   73.19  112.75  1.4228
IC C5   O5   C6    H61       1.4245  112.75  172.71  108.96  1.1117
IC H61  O5   *C6   H62       1.1117  108.96  118.85  111.11  1.1112
IC H61  O5   *C6   H63       1.1117  108.96 -119.10  110.77  1.1119
IC C4   C5   C7    N8        1.5445  108.76   69.16  113.27  1.4441
IC N8   C5   *C7   C10       1.4441  113.27  119.59  107.01  1.5063
IC N8   C5   *C7   H7        1.4441  113.27 -123.34  109.55  1.1139
IC C5   C7   C10   O10       1.5203  107.01  107.28  125.35  1.2150
IC O10  C7   *C10  H10       1.2150  125.35 -179.83  116.01  1.1098
IC C5   C7   N8    C9        1.5203  113.27 -168.46  124.32  1.3325
IC C9   C7   *N8   H8        1.3325  124.32 -177.54  116.94  0.9959
IC C7   N8   C9    O9        1.4441  124.32  179.97  121.00  1.2234
IC O9   N8   *C9   H9        1.2234  121.00  179.84  113.33  1.0959


RESI DMPU           0.00 ! N1,N2-dimethyl-N1-phenylurea, yxu
GROUP
ATOM C1     CG2R61 -0.115 !   H101      O7       H92
ATOM H1     HGR61   0.115 !       \     ||      /
GROUP                     !  H102-C10   C7    C9-H91
ATOM C2     CG2R61 -0.115 !       / \  /  \  /  \
ATOM H2     HGR61   0.115 !   H103   N6    N8    H93
GROUP                     !          |      |
ATOM C3     CG2R61 -0.115 !          |      H8
ATOM H3     HGR61   0.115 !          C6
GROUP                     !         // \
ATOM C4     CG2R61 -0.115 !        C1   C5
ATOM H4     HGR61   0.115 !        |    ||
GROUP                     !        C2   C4
ATOM C5     CG2R61 -0.115 !         \\ /
ATOM H5     HGR61   0.115 !          C3
GROUP
```

```
ATOM C6     CG2R61  0.220
ATOM N6     NG2S0  -0.49
ATOM C7     CG2O6   0.49
ATOM O7     OG2D1  -0.48
ATOM N8     NG2S1  -0.44
ATOM H8     HGP1    0.30
ATOM C9     CG331  -0.06
ATOM H91    HGA3    0.09
ATOM H92    HGA3    0.09
ATOM H93    HGA3    0.09
ATOM C10    CG331  -0.08
ATOM H101   HGA3    0.09
ATOM H102   HGA3    0.09
ATOM H103   HGA3    0.09
BOND C1   C2     C1   C6     C1   H1     C2   C3
BOND C2   H2     C3   C4     C3   H3     C6   N6
BOND C4   C5     C4   H4     C5   C6     C5   H5
BOND N6   C7     N6   C10    C7   O7     C7   N8
BOND N8   H8     N8   C9     C9   H91    C9   H92
BOND C10  H101   C10  H102   C10  H103   C9   H93
IC C2   C6   *C1   H1        1.4018  121.16  177.19  120.21  1.0772
IC C6   C1   C2    C3        1.4078  121.16   -0.79  119.90  1.3988
IC C3   C1   *C2   H2        1.3988  119.90  179.87  120.13  1.0808
IC C1   C2   C3    C4        1.4018  119.90    0.18  119.92  1.3991
IC C4   C2   *C3   H3        1.3991  119.92  179.93  120.11  1.0808
IC C2   C3   C4    C5        1.3988  119.92    0.08  119.79  1.4012
IC C5   C3   *C4   H4        1.4012  119.79 -179.89  120.24  1.0804
IC C6   C4   *C5   H5        1.4079  121.29 -178.70  118.92  1.0788
IC C5   C1   *C6   N6        1.4079  117.93  177.10  119.14  1.4415
IC C1   C6   N6    C7        1.4078  119.14  146.18  119.61  1.3786
IC C7   C6   *N6   C10       1.3786  119.61 -177.21  119.32  1.4667
IC C6   N6   C7    N8        1.4415  119.61 -170.34  111.78  1.3633
IC N8   N6   *C7   O7        1.3633  111.78  164.56  126.38  1.2253
IC N6   C7   N8    C9        1.3786  111.78  174.02  121.02  1.4412
IC C9   C7   *N8   H8        1.4412  121.02 -158.33  116.36  0.9897
IC C7   N8   C9    H91       1.3633  121.02  177.93  110.59  1.1121
IC H91  N8   *C9   H92       1.1121  110.59  120.33  110.52  1.1137
IC H91  N8   *C9   H93       1.1121  110.59 -120.05  110.46  1.1138
IC C6   N6   C10   H101      1.4415  119.32 -136.07  109.40  1.1134
IC H101 N6   *C10  H102      1.1134  109.40  117.17  109.54  1.1127
IC H101 N6   *C10  H103      1.1134  109.40 -122.41  108.35  1.1120

RESI MMAM           1.00 ! dimethylammonium, yxu
GROUP
ATOM C1     CG334   0.11 !       H11
ATOM H11    HGA3    0.09 !        |
ATOM H12    HGA3    0.09 !   H12-C1-H13
ATOM H13    HGA3    0.09 !        |(+)
ATOM N      NG3P2  -0.52 !   HN1-N-HN2
ATOM HN1    HGP2    0.38 !        |
ATOM HN2    HGP2    0.38 !   H21-C2-H22
ATOM C2     CG334   0.11 !        |
ATOM H21    HGA3    0.09 !       H23
ATOM H22    HGA3    0.09
ATOM H23    HGA3    0.09
BOND N    HN1    N    HN2    N    C1     N    C2
BOND C1   H11    C1   H12    C1   H13
BOND C2   H21    C2   H22    C2   H23
IC H11  N    *C1   H12       1.1104  107.35  120.00  107.37  1.1110
IC H11  N    *C1   H13       1.1104  107.35 -120.04  107.32  1.1107
IC H11  C1   N     C2        1.1104  107.35   60.02  114.57  1.5065
IC C2   C1   *N    HN1       1.5065  114.57 -123.15  109.32  1.0143
IC HN1  C1   *N    HN2       1.0143  109.32 -113.74  109.34  1.0142
```

```
IC C1   N    C2    H21       1.5070  114.57  -60.02  107.32  1.1110
IC H21  N    *C2   H22       1.1110  107.32  120.03  107.31  1.1106
IC H21  N    *C2   H23       1.1110  107.32 -119.98  107.36  1.1110

RESI ALAI           0.00 ! zwitterionic alanine, yxu
GROUP
ATOM C7     CG331  -0.27
ATOM H71    HGA3    0.09
ATOM H72    HGA3    0.09
ATOM H73    HGA3    0.09
ATOM C8     CG314   0.16
ATOM H8     HGA1    0.11
ATOM N9     NG3P3  -0.35
ATOM H91    HGP2    0.30
ATOM H92    HGP2    0.30
ATOM H93    HGP2    0.30
ATOM C10    CG2O3   0.32
ATOM O11    OG2D2  -0.57
ATOM O12    OG2D2  -0.57
BOND C7   H71    C7   H72    C7   H73    C7   C8
BOND C8   H8     C8   N9     C8   C10    N9   H91
BOND N9   H92    N9   H93    C10  O11    C10  O12
IC H71  C8   *C7   H72       1.1116  111.09  122.39  111.61  1.1097
IC H71  C8   *C7   H73       1.1116  111.09 -118.30  109.28  1.1136
IC H71  C7   C8    C10       1.1116  111.09   59.76  111.38  1.5452
IC C10  C7   *C8   N9        1.5452  111.38 -122.88  108.51  1.4916
IC N9   C7   *C8   H8        1.4916  108.51 -119.02  110.09  1.1131
IC C7   C8   N9    H91       1.5438  108.51  144.50  102.73  1.0502
IC H91  C8   *N9   H92       1.0502  102.73  120.24  112.44  1.0333
IC H91  C8   *N9   H93       1.0502  102.73 -114.64  108.70  1.0363
IC C7   C8   C10   O11       1.5438  111.38 -127.71  116.02  1.2627
IC O11  C8   *C10  O12       1.2627  116.02 -167.33  114.69  1.2541

RESI MHPO           0.00 ! methyl hydrogen peroxide, yxu
GROUP
ATOM C1     CG331  -0.01
ATOM H11    HGA3    0.09
ATOM H12    HGA3    0.09
ATOM H13    HGA3    0.09
ATOM O2     OG301  -0.25
ATOM O3     OG311  -0.44
ATOM H3     HGP1    0.43
BOND C1   H11    C1   H12    C1   H13    C1   O2
BOND O2   O3     O3   H3
IC H11  O2   *C1   H12       1.1106  109.10  119.48  110.42  1.1117
IC H11  O2   *C1   H13       1.1106  109.10 -119.48  110.42  1.1117
IC H11  C1   O2    O3        1.1106  109.10  180.00  105.01  1.4633
IC C1   O2   O3    H3        1.4190  105.01  180.00   98.86  0.9621

RESI 7DNG           0.00 ! 7-deazaguanine, yxu
GROUP
ATOM N9     NG2R51 -0.31 !           O6
ATOM H9     HGP1    0.35 !           ||
ATOM C8     CG2R51  0.06 !           C6       H7
ATOM H8     HGR52   0.08 !          /  \     /
ATOM C7     CG2R51 -0.27 !      H1-N1   C5--C7\\
ATOM H7     HGR51   0.13 !         |    ||     C8-H8
ATOM C5     CG2RC0 -0.06 !         C2   C4--N9/
ATOM C6     CG2R63  0.42 !        / \\ /
ATOM O6     OG2D4  -0.52 !  H21-N2   N3
ATOM N1     NG2R61 -0.38 !      |
ATOM H1     HGP1    0.30 !     H22
ATOM C2     CG2R64  0.65
```

```
ATOM N2      NG2S3  -0.60
ATOM H21     HGP4    0.30
ATOM H22     HGP4    0.30
ATOM N3      NG2R62 -0.77
ATOM C4      CG2RC0  0.32
BOND N9   C8      N9   C4      N9   H9      N3   C4
BOND C8   H8      C8   C7      C7   H7      C7   C5
BOND C5   C6      C5   C4      C6   O6      C6   N1
BOND N1   H1      N1   C2      C2   N2      C2   N3
BOND N2   H21     N2   H22
IC C8   C4   *N9   H9        1.3859  111.33  179.47  120.17  1.0048
IC C4   N9   C8    C7        1.3467  111.33   -3.99  107.67  1.3682
IC C7   N9   *C8   H8        1.3682  107.67 -179.93  123.16  1.0824
IC N9   C8   C7    C5        1.3859  107.67   -2.39  107.06  1.4387
IC C5   C8   *C7   H7        1.4387  107.06  178.52  126.17  1.0796
IC C4   C7   *C5   C6        1.4185  106.77  159.54  133.85  1.4209
IC C7   C5   C6    N1        1.4387  133.85  152.35   98.56  1.4018
IC N1   C5   *C6   O6        1.4018   98.56  180.00  109.95  1.2366
IC C5   C6   N1    C2        1.4209   98.56   68.92  116.54  1.3861
IC C2   C6   *N1   H1        1.3861  116.54 -180.00  101.07  1.0006
IC C6   N1   C2    N2        1.4018  116.54  162.49  114.30  1.3235
IC N2   N1   *C2   N3        1.3235  114.30  179.10  112.53  1.3623
IC N1   C2   N2    H21       1.3861  114.30  -33.99  120.90  0.9932
IC H21  C2   *N2   H22       0.9932  120.90 -165.83  116.51  0.9933

RESI NCYP           1.00 ! 2-methylamino-4-imino pyrimidine, protonated, yxu
GROUP
ATOM N1      NG2P1  -0.59
ATOM H1      HGP2    0.35
ATOM C2      CG2R64  0.77
ATOM N2      NG2P1  -0.60
ATOM H2      HGP2    0.49
ATOM N3      NG2P1  -0.81
ATOM H3      HGP2    0.44
ATOM C4      CG2R64  0.57
ATOM N4      NG2D1  -0.68
ATOM H4      HGP1    0.34
ATOM C5      CG2R61 -0.24
ATOM H5      HGR61   0.21
ATOM C6      CG2R61  0.13
ATOM H6      HGR62   0.20
ATOM C7      CG334   0.15
ATOM H71     HGA3    0.09
ATOM H72     HGA3    0.09
ATOM H73     HGA3    0.09
BOND N1   C2      N1   C6      C2   N2      C2   N3
BOND N2   H2      N2   C7      N3   H3      N3   C4
BOND C4   N4      C4   C5      N4   H4      C5   H5
BOND C6   H6      C5   C6      C7   H71     C7   H72
BOND C7   H73     N1   H1
IMPR C2      N1      N2      N3        C4   C5   N4   N3
IC C6   C2   *N1   H1        1.4047  122.78 -179.75  118.10  0.9935
IC C6   N1   C2    N3        1.4047  122.78   -0.03  116.91  1.3584
IC N3   N1   *C2   N2        1.3584  116.91 -180.00  121.40  1.3342
IC N1   C2   N2    C7        1.3584  121.40 -178.01  122.40  1.4741
IC C7   C2   *N2   H2        1.4741  122.40  178.38  116.45  0.9938
IC N1   C2   N3    C4        1.3584  116.91    0.16  125.75  1.4007
IC C4   C2   *N3   H3        1.4007  125.75 -179.72  121.83  0.9864
IC C2   N3   C4    N4        1.3584  125.75  180.00  116.81  1.2931
IC N4   N3   *C4   C5        1.2931  116.81  179.92  115.06  1.4300
IC N3   C4   N4    H4        1.4007  116.81  179.94  110.84  0.9976
IC C6   C4   *C5   H5        1.3856  120.79 -179.85  118.16  1.0771
IC C5   N1   *C6   H6        1.3856  118.71  179.84  118.49  1.0888
```

```
IC C2   N2   C7    H71       1.3342  122.40  178.70  111.00  1.1155
IC H71  N2   *C7   H72       1.1155  111.00  119.37  111.80  1.1149
IC H71  N2   *C7   H73       1.1155  111.00 -119.31  111.91  1.1160

RESI PNCP           1.00 ! 2-propylamino-4-imino-pyrimidine, protonated, yxu
GROUP
ATOM N1     NG2P1  -0.59
ATOM H1     HGP2    0.35
ATOM C2     CG2R64  0.77
ATOM N2     NG2P1  -0.60
ATOM H2     HGP2    0.49
ATOM N3     NG2P1  -0.81
ATOM H3     HGP2    0.44
ATOM C4     CG2R64  0.57
ATOM N4     NG2D1  -0.68
ATOM H4     HGP1    0.34
ATOM C5     CG2R61 -0.24
ATOM H5     HGR61   0.21
ATOM C6     CG2R61  0.13
ATOM H6     HGR62   0.20
ATOM C7     CG324   0.24
ATOM H71    HGA2    0.09
ATOM H72    HGA2    0.09
GROUP
ATOM C8     CG321  -0.18
ATOM H81    HGA2    0.09
ATOM H82    HGA2    0.09
GROUP
ATOM C9     CG331  -0.27
ATOM H91    HGA3    0.09
ATOM H92    HGA3    0.09
ATOM H93    HGA3    0.09
BOND N1   C2     C2   N2     C2   N3     N1   H1
BOND N1   C6     C9   H91    C9   H92    C9   H93
BOND N2   H2     N2   C7     N3   H3     N3   C4
BOND C4   N4     C4   C5     N4   H4     C5   H5
BOND C5   C6     C6   H6     C7   H71    C7   H72
BOND C7   C8     C8   H81    C8   H82    C8   C9
IMPR C2     N1     N2     N3     C4   C5   N4   N3
IC C6   C2   *N1   H1        1.4051  122.90  179.47  118.12  0.9939
IC H1   N1   C2    N2        0.9939  118.12   -0.38  120.96  1.3324
IC N2   N1   *C2   N3        1.3324  120.96  179.89  116.77  1.3590
IC N1   C2   N2    C7        1.3579  120.96 -178.00  123.14  1.4680
IC C7   C2   *N2   H2        1.4680  123.14  175.97  116.58  0.9913
IC N1   C2   N3    C4        1.3579  116.77    0.14  125.78  1.4001
IC C4   C2   *N3   H3        1.4001  125.78  178.94  121.60  0.9862
IC C2   N3   C4    N4        1.3590  125.78  179.71  116.83  1.2942
IC N4   N3   *C4   C5        1.2942  116.83 -179.91  115.13  1.4293
IC N3   C4   N4    H4        1.4001  116.83 -180.00  110.94  0.9975
IC C6   C4   *C5   H5        1.3857  120.75  179.99  118.26  1.0767
IC C5   N1   *C6   H6        1.3857  118.66 -179.84  118.54  1.0884
IC C2   N2   C7    C8        1.3324  123.14   80.75  109.66  1.5382
IC C8   N2   *C7   H71       1.5382  109.66 -123.39  112.32  1.1060
IC H71  N2   *C7   H72       1.1060  112.32 -117.19  109.55  1.1057
IC N2   C7   C8    C9        1.4680  109.66  179.22  110.99  1.5368
IC C9   C7   *C8   H81       1.5368  110.99  120.88  110.60  1.1133
IC H81  C7   *C8   H82       1.1133  110.60  118.65  109.86  1.1136
IC C7   C8   C9    H91       1.5382  110.99 -179.76  110.34  1.1128
IC H91  C8   *C9   H92       1.1128  110.34  119.55  111.20  1.1111
IC H91  C8   *C9   H93       1.1128  110.34 -119.45  111.19  1.1118

RESI C34H           1.00 ! 2-methylamino-4-amino-pyrimidine, protonated, yxu
GROUP
```

```
ATOM N1     NG2R61 -0.75 !    H41   H42
ATOM H1     HGP1    0.32 !      \  /
ATOM C2     CG2R64  0.81 !       N4
ATOM N2     NG311  -0.57 !       ||
ATOM H2     HGPAM1  0.46 !       C4
ATOM N3     NG2R62 -0.62 !      /  \
ATOM C4     CG2R64  0.61 !  H5-C5   N3
ATOM N4     NG2P1  -0.80 !     ||   ||
ATOM H41    HGP2    0.38 !  H6-C6   C2     H71
ATOM H42    HGP2    0.38 !      \  /  \     |
ATOM C5     CG2R61  0.08 !       N1    N2--C7--H73
ATOM H5     HGR61   0.07 !             |    |
ATOM C6     CG2R61  0.18 !             H2  H72
ATOM H6     HGR62   0.20
ATOM C7     CG331  -0.02
ATOM H71    HGA3    0.09
ATOM H72    HGA3    0.09
ATOM H73    HGA3    0.09
BOND N1   C2     N1   C6     N1   H1     N3   C4
BOND C2   N2     C2   N3     N2   H2     N2   C7
BOND C4   N4     C4   C5     N4   H41    N4   H42
BOND C5   H5     C5   C6     C6   H6     C7   H72
BOND C7   H71    C7   H73
IC C6   C2   *N1   H1       1.4103  120.22 -172.93  115.53  1.0076
IC C6   N1   C2    N3       1.4103  120.22   -2.64  121.47  1.3437
IC N3   N1   *C2   N2       1.3437  121.47 -176.71  116.68  1.3466
IC N1   C2   N2    C7       1.3945  116.68  156.69  122.55  1.4836
IC C7   C2   *N2   H2       1.4836  122.55 -134.62  111.99  1.0125
IC N1   C2   N3    C4       1.3945  121.47    3.26  117.91  1.3429
IC C2   N3   C4    N4       1.3437  117.91  178.97  115.17  1.3633
IC N4   N3   *C4   C5       1.3633  115.17  178.36  125.85  1.4236
IC N3   C4   N4    H41      1.3429  115.17 -178.33  123.38  0.9957
IC H41  C4   *N4   H42      0.9957  123.38  177.17  115.67  0.9995
IC C6   C4   *C5   H5       1.4049  114.86  179.31  122.64  1.0755
IC C5   N1   *C6   H6       1.4049  119.61 -179.70  119.39  1.0877
IC C2   N2   C7    H72      1.3466  122.55  170.91  110.78  1.1141
IC H72  N2   *C7   H71      1.1141  110.78  120.28  111.61  1.1128
IC H72  N2   *C7   H73      1.1141  110.78 -118.92  111.51  1.1140

RESI 2MSU          0.00 ! 2-methylthiouracil, yxu
GROUP
ATOM N1     NG2R61 -0.60 !      O4
ATOM H1     HGP1    0.35 !      ||
ATOM C2     CG2R64  0.41 !      C4
ATOM S2     SG311  -0.13 !     /  \
ATOM N3     NG2R62 -0.56 ! H5-C5   N3
ATOM C4     CG2R63  0.53 !    ||   ||
ATOM O4     OG2D4  -0.52 ! H6-C6   C2   C20
ATOM C5     CG2R62 -0.17 !     \  / \  /
ATOM H5     HGR62   0.10 !      N1   S2
ATOM C6     CG2R62  0.24 !      |
ATOM H6     HGR62   0.14 !      H1
ATOM C20    CG331  -0.06
ATOM H201   HGA3    0.09
ATOM H202   HGA3    0.09
ATOM H203   HGA3    0.09
BOND N1   C2     N1   C6     N1   H1     S2   C20
BOND C2   S2     C2   N3     S2   C20    N3   C4
BOND C4   O4     C4   C5     C5   H5     C5   C6
BOND C6   H6     C20 H201    C20  H202   C20  H203
IC C6   C2   *N1   H1       1.3574  120.00 -179.98  116.37  1.0065
IC C6   N1   C2    N3       1.3574  120.00   -0.05  119.65  1.3495
IC N3   N1   *C2   S2       1.3495  119.65  179.87  119.71  1.7518
```

```
IC N1   C2   N3    C4        1.3940  119.65   -0.04  121.64  1.3602
IC C2   N3   C4    O4        1.3495  121.64 -179.90  120.77  1.2291
IC O4   N3   *C4   C5        1.2291  120.77 -179.97  118.89  1.4323
IC C6   C4   *C5   H5        1.3624  118.54  179.97  120.38  1.0864
IC C5   N1   *C6   H6        1.3624  121.28 -179.98  118.09  1.0935
IC N1   C2   S2    C20       1.3940  119.71 -179.69   97.91  1.8290
IC C2   S2   C20   H201      1.7518   97.91  179.96  110.56  1.1114
IC H201 S2   *C20  H202      1.1114  110.56  119.40  111.76  1.1119
IC H201 S2   *C20  H203      1.1114  110.56 -119.40  111.73  1.1125


!! Withheld because of incorrect atom typing and improper definitions -- Kenno.

RESI BK2C           1.00 ! lysidine, yxu
GROUP
ATOM N1     NG2P1  -0.24 !             H4
ATOM C2     CG2R64  0.77 !               \
ATOM N2     NG2P1  -0.60 !                N4
ATOM H2     HGP2    0.49 !                ||
ATOM N3     NG2P1  -0.81 !                C4    H3
ATOM H3     HGP2    0.44 !               /  \  /
ATOM C4     CG2R64  0.57 !           H5-C5   N3
ATOM N4     NG2D1  -0.68 !              ||   |
ATOM H4     HGP1    0.34 !           H6-C6   C2      H71 H81  H91  H101 H12    O30 (-
)
ATOM C5     CG2R61 -0.24 !               \  /  \\(+) |   |    |    |    |     /
ATOM H5     HGR61   0.21 !                N1    N2 --C7--C8 --C9 --C10--C12--C13
ATOM C6     CG2R61  0.13 !                 \    |    |   |    |    |    |     \\
ATOM H6     HGR62   0.20 !                  \    H2   H72 H82  H92  H102 | (+)  O31
ATOM C7     CG324   0.24 !                   \                         N14
ATOM H71    HGA2    0.09 !                    \                       / |  \
ATOM H72    HGA2    0.09 !                     \                  H141 H142 H143
GROUP
ATOM C8     CG321  -0.18
ATOM H81    HGA2    0.09
ATOM H82    HGA2    0.09
GROUP
ATOM C9     CG321  -0.18
ATOM H91    HGA2    0.09
ATOM H92    HGA2    0.09
GROUP
ATOM C10    CG321  -0.18
ATOM H101   HGA2    0.09 !  !!!! PATCH 5UHA for the non-ionic tautomer of side chain
ATOM H102   HGA2    0.09 !  !!!! PATCH K2CN for the neutral base
GROUP                    !  !!!! PATCH 34HC for the tautomer of charged base
ATOM C12    CG314   0.17
ATOM H12    HGA1    0.11
ATOM N14    NG3P3  -0.34
ATOM H141   HGP2    0.30
ATOM H142   HGP2    0.30
ATOM H143   HGP2    0.30
ATOM C13    CG2O3   0.32
ATOM O30    OG2D2  -0.58
ATOM O31    OG2D2  -0.58
GROUP
ATOM CM     CG334  -0.27
ATOM HM1    HGA3    0.09
ATOM HM2    HGA3    0.09
ATOM HM3    HGA3    0.09
BOND CM   HM1    CM   HM2   CM   HM3
BOND N1   C2   N1   C6   C2   N3   C2   N2
BOND N3   C4   C4   N4   C4   C5   N3   H3
BOND N4   H4   C5   H5   C5   C6   C6   H6
```

```
BOND N2   H2   N2   C7   C7   H71  C7   H72
BOND C7   C8   C8   H81  C8   H82  C8   C9
BOND C9   H91  C9   H92  C9   C10  C10  H101
BOND C10  H102 C10  C12  C12  H12  C12  C13
BOND C12  N14  C13  O30  C13  O31  N14  H141
BOND N14  H142 N14  H143
BOND CM   N1
IMPR C2   N1   N3   N2        C4   C5   N4   N3        C13  O31  O30  C12
DONO H4   N4
DONO H3   N3
DONO H2   N2
DONO H141 N14
DONO H142 N14
DONO H143 N14
ACCE O30  C13
ACCE O31  C13
IC C6   C2   *N1   CM        1.4045  120.81 -178.87  120.92  1.4824
IC C6   N1   C2    N2        1.4045  120.81  178.78  122.01  1.3409
IC N2   N1   *C2   N3        1.3409  122.01 -179.66  117.55  1.3668
IC N1   C2   N2    C7        1.3832  122.01 -165.23  123.79  1.4680
IC C7   C2   *N2   H2        1.4680  123.79  164.10  113.28  0.9946
IC N1   C2   N3    C4        1.3832  117.55    0.68  125.57  1.3998
IC C4   C2   *N3   H3        1.3998  125.57 -179.50  122.27  0.9872
IC C2   N3   C4    N4        1.3668  125.57  179.88  116.52  1.2933
IC N4   N3   *C4   C5        1.2933  116.52 -179.98  115.22  1.4301
IC N3   C4   N4    H4        1.3998  116.52 -179.71  111.10  0.9979
IC C6   C4   *C5   H5        1.3868  120.67 -179.87  118.30  1.0772
IC C5   N1   *C6   H6        1.3868  120.18  179.90  116.36  1.0852
IC C2   N2   C7    C8        1.3409  123.79   77.02  109.03  1.5379
IC C8   N2   *C7   H71       1.5379  109.03 -123.07  112.26  1.1057
IC H71  N2   *C7   H72       1.1057  112.26 -117.30  109.56  1.1051
IC N2   C7   C8    C9        1.4680  109.03 -177.56  112.56  1.5427
IC C9   C7   *C8   H81       1.5427  112.56 -121.63  110.19  1.1134
IC H81  C7   *C8   H82       1.1134  110.19 -118.39  109.91  1.1141
IC C7   C8   C9    C10       1.5379  112.56  175.50  110.31  1.5404
IC C10  C8   *C9   H91       1.5404  110.31 -119.29  109.42  1.1147
IC H91  C8   *C9   H92       1.1147  109.42 -118.67  109.86  1.1119
IC C8   C9   C10   C12       1.5427  110.31 -175.49  116.53  1.5544
IC C12  C9   *C10  H101      1.5544  116.53  120.00  106.75  1.1175
IC H101 C9   *C10  H102      1.1175  106.75  114.76  110.08  1.1134
IC C9   C10  C12   C13       1.5404  116.53  171.10  109.14  1.5416
IC C13  C10  *C12  N14       1.5416  109.14  121.33  111.02  1.4953
IC C13  C10  *C12  H12       1.5416  109.14 -117.33  110.21  1.1119
IC C10  C12  N14   H141      1.5544  111.02 -121.55  100.94  1.0559
IC H141 C12  *N14  H142      1.0559  100.94  115.74  111.29  1.0334
IC H141 C12  *N14  H143      1.0559  100.94 -116.34  112.24  1.0350
IC C10  C12  C13   O30       1.5544  109.14  120.02  116.73  1.2601
IC O30  C12  *C13  O31       1.2601  116.73 -179.93  114.86  1.2541
IC C6   N1   CM    HM1       1.4045  118.27 -116.59  111.57  1.1145
IC HM1  N1   *CM   HM2       1.1145  111.57  118.91  112.09  1.1154
IC HM1  N1   *CM   HM3       1.1145  111.57 -122.07  111.78  1.1142

RESI BR2C           2.00 ! agmatidine, AG9  yxu
GROUP
ATOM N1     NG2P1  -0.24 !              H4
ATOM C2     CG2R64  0.77 !                \
ATOM N2     NG2P1  -0.60 !                 N4
ATOM H2     HGP2    0.49 !                 ||
ATOM N3     NG2P1  -0.81 !                 C4    H3
ATOM H3     HGP2    0.44 !                /  \  /
ATOM C4     CG2R64  0.57 !            H5-C5   N3                              H21
ATOM N4     NG2D1  -0.68 !               ||   |                               |
```

```
ATOM H4      HGP1     0.34 !              H6-C6    C2       H71  HB1  HG1  HD1  HE
NH2--H22
ATOM C5      CG2R61 -0.24 !                   \  /  \\(+) |    |    |    |    |     /
ATOM H5      HGR61    0.21 !                   N1    N2 --C7 --CB --CG --CD --NE --CZ
ATOM C6      CG2R61  0.13 !                    \     |    |    |    |    |       (+)\\
ATOM H6      HGR62    0.20 !                     \   H2   H72  HB2  HG2  HD2
NH1--H11
ATOM C7      CG324    0.24 !                      \                                  |
ATOM H71     HGA2     0.09 !                       \                                 H12
ATOM H72     HGA2     0.09
GROUP
ATOM CB      CG321  -0.18
ATOM HB1     HGA2     0.09
ATOM HB2     HGA2     0.09
GROUP
ATOM CG      CG321  -0.18
ATOM HG1     HGA2     0.09
ATOM HG2     HGA2     0.09
GROUP
ATOM CD      CG324    0.21 ! !! PATCH K2CN for the neutral base
ATOM HD1     HGA2     0.09 ! !! PATCH 34HC for the tautomer of charged base
ATOM HD2     HGA2     0.09
ATOM NE      NG2P1  -0.71
ATOM HE      HGP2     0.44
ATOM CZ      CG2N1    0.64
ATOM NH1     NG2P1  -0.80
ATOM H11     HGP2     0.46
ATOM H12     HGP2     0.46
ATOM NH2     NG2P1  -0.80
ATOM H21     HGP2     0.46
ATOM H22     HGP2     0.46
GROUP
ATOM CM      CG334  -0.27
ATOM HM1     HGA3     0.09
ATOM HM2     HGA3     0.09
ATOM HM3     HGA3     0.09
BOND CM   HM1    CM   HM2   CM   HM3
BOND N1   C2   N1   C6   C2   N3   C2   N2
BOND N3   C4   C4   N4   C4   C5   N4   H4
BOND C5   H5   C5   C6   C6   H6   N3   H3
BOND N2   H2   N2   C7   C7   H71  C7   H72
BOND C7   CB   CB   HB1  CB   HB2  CB   CG
BOND CG   HG1  CG   HG2  CG   CD   CD   HD1
BOND CD   HD2  CD   NE   NE   HE   NE   CZ
BOND CZ   NH1  CZ   NH2  NH1  H11  NH1  H12
BOND NH2  H21  NH2  H22
BOND CM   N1
IMPR C2   N1   N3   N2        C4   C5   N4   N3        CZ   NE   NH1  NH2
DONO H4   N4
DONO H3   N3
DONO H2   N2
DONO H11  NH1
DONO H12  NH1
DONO H21  NH2
DONO H22  NH2
DONO HE   NE
IC C6   C2   *N1   CM        1.4055  120.76  179.50  120.91  1.4822
IC C6   N1   C2    N2        1.4055  120.76 -179.16  122.11  1.3426
IC N2   N1   *C2   N3        1.3426  122.11 -179.95  117.65  1.3664
IC N1   C2   N2    C7        1.3825  122.11  175.60  124.50  1.4691
IC C7   C2   *N2   H2        1.4691  124.50 -173.87  113.38  0.9968
IC N1   C2   N3    C4        1.3825  117.65   -0.27  125.52  1.3995
IC C4   C2   *N3   H3        1.3995  125.52 -178.13  122.51  0.9855
```

```
IC C2   N3   C4    N4        1.3664  125.52  179.43  116.26  1.2930
IC N4   N3   *C4   C5        1.2930  116.26 -179.78  115.29  1.4288
IC N3   C4   N4    H4        1.3995  116.26  179.80  110.81  0.9993
IC C6   C4   *C5   H5        1.3883  120.65 -179.92  118.45  1.0772
IC C5   N1   *C6   H6        1.3883  120.13 -179.61  116.44  1.0850
IC C2   N2   C7    CB        1.3426  124.50   83.99  110.02  1.5462
IC CB   N2   *C7   H71       1.5462  110.02 -123.61  111.92  1.1054
IC H71  N2   *C7   H72       1.1054  111.92 -116.72  109.26  1.1057
IC N2   C7   CB    CG        1.4691  110.02 -179.84  111.78  1.5505
IC CG   C7   *CB   HB1       1.5505  111.78 -121.14  109.31  1.1126
IC HB1  C7   *CB   HB2       1.1126  109.31 -118.00  109.73  1.1131
IC C7   CB   CG    CD        1.5462  111.78  177.89  111.85  1.5433
IC CD   CB   *CG   HG1       1.5433  111.85 -120.55  108.94  1.1145
IC HG1  CB   *CG   HG2       1.1145  108.94 -117.90  109.75  1.1127
IC CB   CG   CD    NE        1.5505  111.85 -175.46  109.09  1.4699
IC NE   CG   *CD   HD1       1.4699  109.09 -125.31  110.78  1.1044
IC HD1  CG   *CD   HD2       1.1044  110.78 -115.94  109.15  1.1069
IC CG   CD   NE    CZ        1.5433  109.09 -100.67  126.32  1.3431
IC CZ   CD   *NE   HE        1.3431  126.32 -176.72  118.30  0.9955
IC CD   NE   CZ    NH1       1.4699  126.32 -177.99  119.15  1.3327
IC NH1  NE   *CZ   NH2       1.3327  119.15  179.78  122.41  1.3295
IC NE   CZ   NH1   H11       1.3431  119.15  179.17  119.77  0.9936
IC H11  CZ   *NH1  H12       0.9936  119.77 -177.97  120.37  0.9926
IC NE   CZ   NH2   H21       1.3431  122.41 -177.81  119.51  0.9931
IC H21  CZ   *NH2  H22       0.9931  119.51  175.40  119.17  0.9883
IC C6   N1   CM    HM1       1.4055  118.33 -120.52  111.83  1.1146
IC HM1  N1   *CM   HM2       1.1146  111.83  118.89  112.04  1.1166
IC HM1  N1   *CM   HM3       1.1146  111.83 -122.24  111.74  1.1144

RESI MDMP           0.00 ! 1,3,5-trimethyl-pseudouracil, yxu
GROUP
ATOM C5     CG2R62 -0.20 !    H11    O2     H31
ATOM C4     CG2R63  0.74 !       \    ||     /
ATOM O4     OG2D4  -0.52 !  H12-C1   C2    C3-H32
ATOM N3     NG2R61 -0.44 !       / \ /  \  / \
ATOM C2     CG2R63  0.86 !    H13  N1   N3   H33
ATOM O2     OG2D4  -0.53 !          |    |
ATOM N1     NG2R61 -0.33 !      H6-C6   C4
ATOM C6     CG2R62  0.02 !           \  / \\
ATOM H6     HGR62   0.19 !            C5   O4
ATOM C1     CG331  -0.15 !            |
ATOM H11    HGA3    0.09 !            CM
ATOM H12    HGA3    0.09
ATOM H13    HGA3    0.09
ATOM C3     CG331  -0.18
ATOM H31    HGA3    0.09
ATOM H32    HGA3    0.09
ATOM H33    HGA3    0.09
GROUP
ATOM CM     CG331  -0.27
ATOM HM1    HGA3    0.09
ATOM HM2    HGA3    0.09
ATOM HM3    HGA3    0.09
BOND C5   C4     C5   C6     C5   CM     C6   H6
BOND C4   O4     C4   N3     N3   C2     N3   C3
BOND C2   O2     C2   N1     N1   C6     N1   C1
BOND C1   H11    C1   H12    C1   H13    C3   H33
BOND C3   H31    C3   H32
BOND CM   HM1    CM   HM2    CM   HM3
IC C4   C6   *C5   CM     1.4674  115.05  179.48  118.21  1.5009
IC C6   C5   C4    N3     1.3809  115.05  -43.05  110.53  1.4072
IC N3   C5   *C4   O4     1.4072  110.53  179.41  119.55  1.2318
IC C5   C4   N3    C2     1.4674  110.53   62.95  107.48  1.4040
```

```
IC C2   C4   *N3   C3      1.4040  107.48 -179.09  107.99  1.4957
IC C4   N3   C2    N1      1.4072  107.48  -60.32  114.50  1.4270
IC N1   N3   *C2   O2      1.4270  114.50  179.13  121.48  1.2318
IC N1   C5   *C6   H6      1.3820  121.55  179.90  120.70  1.0934
IC C6   C2   *N1   C1      1.3820  115.53  179.40  118.28  1.4739
IC C2   N1   C1    H11     1.4270  118.28  174.05  112.18  1.1142
IC H11  N1   *C1   H12     1.1142  112.18  120.65  111.29  1.1154
IC H11  N1   *C1   H13     1.1142  112.18 -119.91  111.31  1.1154
IC C4   N3   C3    H33     1.4072  107.99 -175.67  109.89  1.1170
IC H33  N3   *C3   H31     1.1170  109.89  116.24  110.97  1.1161
IC H33  N3   *C3   H32     1.1170  109.89 -120.83  117.02  1.1128
IC C6   C5   CM    HM1     1.3809  118.21   45.38  111.63  1.1123
IC HM1  C5   *CM   HM2     1.1123  111.63  120.08  111.06  1.1135
IC HM1  C5   *CM   HM3     1.1123  111.63 -120.51  110.67  1.1143

RESI BM2G           0.00 ! N2,N2-dimethylguanine, DMG      12/19, yxu
GROUP
ATOM N9     NG2R51 -0.01 !               O6
ATOM C8     CG2R53  0.33 !               ||
ATOM H8     HGR52   0.10 !               C6
ATOM N7     NG2R50 -0.65 !              /  \
ATOM C5     CG2RC0  0.01 !          H1-N1   C5--N7\\
ATOM C6     CG2R63  0.49 !              |    ||     C8-H8
ATOM O6     OG2D4  -0.50 !  H21         C2   C4--N9/
ATOM N1     NG2R61 -0.45 !     \       / \\ /       \
ATOM H1     HGP1    0.33 !  H22-CM2-N2   N3          \
ATOM C4     CG2RC0  0.34 !     /    |                 \
GROUP                    !  H23    CM1                 \
ATOM C2     CG2R64  0.71 !        / | \                 \
ATOM N2     NG301  -0.36 !     H11 H12 H13               \
ATOM N3     NG2R62 -0.62 !                                \
ATOM CM1    CG331  -0.13
ATOM H11    HGA3    0.09
ATOM H12    HGA3    0.09
ATOM H13    HGA3    0.09
ATOM CM2    CG331  -0.13
ATOM H21    HGA3    0.09
ATOM H22    HGA3    0.09
ATOM H23    HGA3    0.09
GROUP
ATOM CM     CG331  -0.27
ATOM HM1    HGA3    0.09
ATOM HM2    HGA3    0.09
ATOM HM3    HGA3    0.09
BOND CM   HM1    CM   HM2   CM   HM3
BOND N9   C8   N9   C4   C8   N7   C8   H8
BOND N7   C5   C5   C6   C5   C4   C6   O6
BOND C6   N1   N1   C2   N1   H1   C2   N2
BOND C2   N3   N2   CM2  N2   CM1  N3   C4
BOND CM2  H21  CM2  H22  CM2  H23  CM1  H11
BOND CM1  H12  CM1  H13
BOND CM   N9
IMPR C6     C5     N1     O6     C2     N1     N3     N2
DONO H1   N1
ACCE O6   C6
ACCE N3
ACCE N7
IC C8   C4   *N9   CM        1.3801  105.90 -180.00  126.28  1.4709
IC C4   N9   C8    N7        1.3796  105.90    0.01  113.70  1.3245
IC N7   N9   *C8   H8        1.3245  113.70 -179.95  121.84  1.0912
IC N9   C8   N7    C5        1.3801  113.70   -0.03  104.44  1.3902
IC C4   N7   *C5   C6        1.4098  109.98  179.95  131.04  1.4172
IC N7   C5   C6    N1        1.3902  131.04 -179.59  112.46  1.3781
```

```
IC N1   C5   *C6   O6        1.3781  112.46 -179.90  130.61  1.2301
IC C5   C6   N1    C2        1.4172  112.46    0.78  127.16  1.3822
IC C2   C6   *N1   H1        1.3822  127.16  179.49  115.09  0.9975
IC C6   N1   C2    N2        1.3781  127.16 -177.68  117.92  1.3730
IC N2   N1   *C2   N3        1.3730  117.92  175.25  119.73  1.3567
IC N1   C2   N2    CM2       1.3822  117.92  -27.56  122.17  1.4625
IC CM2  C2   *N2   CM1       1.4625  122.17 -169.47  120.99  1.4619
IC C2   N2   CM1   H11       1.3730  120.99 -166.95  111.43  1.1128
IC H11  N2   *CM1  H12       1.1128  111.43  119.56  111.55  1.1146
IC H11  N2   *CM1  H13       1.1128  111.43 -120.19  111.04  1.1134
IC C2   N2   CM2   H21       1.3730  122.17 -158.71  111.05  1.1133
IC H21  N2   *CM2  H22       1.1133  111.05  119.20  111.96  1.1130
IC H21  N2   *CM2  H23       1.1133  111.05 -119.27  111.09  1.1138
IC C4   N9   CM    HM1       1.3796  126.28  -59.27  110.11  1.1114
IC HM1  N9   *CM   HM2       1.1114  110.11  118.88  110.14  1.1125
IC HM1  N9   *CM   HM3       1.1114  110.11 -120.62  112.16  1.1115

RESI BN2G           1.00 ! N2,N2,7-trimethylguanine, yxu
GROUP
ATOM N9     NG2R52 -0.09 !               O6     H71  H72
ATOM C8     CG2R53  0.41 !               ||       \ /
ATOM H8     HGR53   0.13 !               C6        C7-H73
ATOM N7     NG2R52 -0.39 !              /  \   (+)/
ATOM C5     CG2RC0  0.13 !          H1-N1   C5--N7\\
ATOM C6     CG2R63  0.58 !             |    ||     C8-H8
ATOM O6     OG2D4  -0.46 !   H21       C2   C4--N9/
ATOM N1     NG2R61 -0.31 !      \     / \\ /      \
ATOM H1     HGP1    0.23 !  H22-C20-N2   N3        \
ATOM C2     CG2R64  0.73 !      /   |               \
ATOM N3     NG2R62 -0.53 !   H23   C10               \
ATOM C4     CG2RC0  0.20 !        / | \               \
ATOM C7     CG334   0.10 !     H11 H12 H13             \
ATOM H71    HGA3    0.09 !                              \
ATOM H72    HGA3    0.09
ATOM H73    HGA3    0.09
GROUP
ATOM N2     NG301  -0.30
ATOM C10    CG331  -0.12
ATOM H11    HGA3    0.09
ATOM H12    HGA3    0.09
ATOM H13    HGA3    0.09
ATOM C20    CG331  -0.12
ATOM H21    HGA3    0.09
ATOM H22    HGA3    0.09
ATOM H23    HGA3    0.09
GROUP
ATOM CM     CG334  -0.27
ATOM HM1    HGA3    0.09
ATOM HM2    HGA3    0.09
ATOM HM3    HGA3    0.09
BOND CM   HM1    CM   HM2   CM   HM3
BOND N9   C8   N9   C4   C8   N7   C8   H8
BOND N7   C5   N7   C7   C5   C6   C5   C4
BOND C6   O6   C6   N1   N1   C2   N1   H1
BOND C2   N2   C2   N3   N2   C20  N2   C10
BOND N3   C4   C20  H21  C20  H22  C20  H23
BOND C10  H11  C10  H12  C10  H13  C7   H71
BOND C7   H72  C7   H73
BOND CM   N9
IMPR C6     C5     N1     O6     C2     N1     N3     N2
DONO H1   N1
ACCE O6   C6
ACCE N3
```

```
IC C8   C4   *N9   CM       1.3335  109.12 -179.70  125.02  1.4789
IC C4   N9   C8    N7       1.3842  109.12   -0.21  109.92  1.3358
IC N7   N9   *C8   H8       1.3358  109.92 -179.89  124.35  1.0721
IC N9   C8   N7    C5       1.3335  109.92    0.09  107.85  1.3967
IC C5   C8   *N7   C7       1.3967  107.85  179.83  126.37  1.4811
IC C4   N7   *C5   C6       1.3931  107.00 -179.93  132.55  1.4127
IC N7   C5   C6    N1       1.3967  132.55 -179.97  112.38  1.3875
IC N1   C5   *C6   O6       1.3875  112.38  179.78  128.93  1.2290
IC C5   C6   N1    C2       1.4127  112.38   -0.94  126.30  1.3927
IC C2   C6   *N1   H1       1.3927  126.30 -179.12  116.39  1.0041
IC C6   N1   C2    N2       1.3875  126.30  177.48  118.55  1.3741
IC N2   N1   *C2   N3       1.3741  118.55 -175.42  119.17  1.3649
IC C8   N7   C7    H71      1.3358  126.37 -123.05  109.21  1.1133
IC H71  N7   *C7   H72      1.1133  109.21  120.45  111.21  1.1131
IC H71  N7   *C7   H73      1.1133  109.21 -118.91  109.14  1.1134
IC N1   C2   N2    C20      1.3927  118.55   25.13  122.15  1.4625
IC C20  C2   *N2   C10      1.4625  122.15  169.57  121.26  1.4621
IC C2   N2   C10   H11      1.3741  121.26  165.74  111.39  1.1135
IC H11  N2   *C10  H12      1.1135  111.39  119.81  111.17  1.1137
IC H11  N2   *C10  H13      1.1135  111.39 -119.42  112.01  1.1138
IC C2   N2   C20   H21      1.3741  122.15  161.49  111.09  1.1146
IC H21  N2   *C20  H22      1.1146  111.09  118.98  111.38  1.1141
IC H21  N2   *C20  H23      1.1146  111.09 -119.04  112.19  1.1128
IC C4   N9   CM    HM1      1.3842  125.02  179.92  111.34  1.1124
IC HM1  N9   *CM   HM2      1.1124  111.34  120.77  108.90  1.1127
IC HM1  N9   *CM   HM3      1.1124  111.34 -120.58  108.79  1.1137

END

read param card flex append
* Additive CHARMM parameters for modified nucleic acids, BETA version
* You Xu, Karolinska Institutet, 2015.
*

ATOMS
MASS   413 SEGD1   78.96000  ! selenocarbonyl Se

BONDS
!!!!!!!! ############################### !!!!!!!!
!!!!!!!! ######## Nucleic Acids ######## !!!!!!!!
!!!!!!!! ############################### !!!!!!!!
!!***Guanine***
CC3162 OG301   360.00     1.4150 ! MQG, from CG36

!!***Model compounds***
CG331  NN2B    400.00     1.4560 ! U & G, from CG36
CG331  NN2     400.00     1.4560 ! A & C, from CG36

!!***2'-O-substituted ribose***
CN7B   OG301    334.30     1.4110 ! 2OM, from CG3C51 OG301, yxu
CN7B   OC301    334.30     1.4110 ! 2OR, from CG3C51 OG301, yxu

!!***glycosyl linkage***
CN7B   NG2R61   220.00     1.4570 ! U, from NA36
CN7B   NG2R51   220.00     1.4570 ! G, from NA36
CG2R62 CN7B     350.00     1.4860 ! PSU, yxu
CN7B   NG2S0    320.00     1.4340 ! H2U, from CG36
NG2R52 CN7B     320.00     1.4600 ! 7MG, from NG2R52 CG334, yxu
CN7B   NG2P1    243.00     1.4530 ! K2C, yxu

!!!!!!!! ######################################## !!!!!!!!
!!!!!!!! ######## CGenFF model compounds ######## !!!!!!!!
!!!!!!!! ######################################## !!!!!!!!
```

```
!!***Uracils***
CG2R63 SEGD1   350.00     1.8500 ! SEU, yxu, tmp
CG2O6  NG2S0   445.00     1.3600 ! H2U, yxu, 21/2/14
CG321  NG2S0   340.00     1.4510 ! H2U, yxu, 21/2/14
CG2R62 OG311   348.00     1.3490 ! 5HU, yxu
CG2R62 OG301   315.00     1.3420 ! MOU, yxu, 21/2/14
CG2R62 CG324   300.00     1.4800 ! SAU, yxu
CG334  NG3P2   220.00     1.4900 ! 5AU, yxu
CG2D1  CG324   370.00     1.4950 ! IAU, yxu
CG2R62 CG321   265.00     1.4800 ! 5CU, yxu
CG2R62 CG311   265.00     1.4800 ! CMU, from 5CU, yxu
CG321  NG2R61  375.00     1.4510 ! 3AU, cgenff_xyu, yxu
CG311  NG321   263.00     1.4740 ! 5UHA, from CG321  NG321, yxu
CG1N1  CG321   400.00     1.4700 ! CYU, from CG1N1 CG331, yxu

!!***Cytosines***
CG2O4  CG2R62  390.00     1.4640 ! 5FC, yxu
CG2R64 NG311   370.00     1.3700 ! 4MC, yxu
CG2R64 NG2D1   485.00     1.2950 ! 3MCn, yxu
CG2R63 NG2P1   410.00     1.4100 ! 3MC, yxu
CG2R64 NG2P1   440.00     1.3700 ! 3MC, yxu
CG2R61 NG2P1   300.00     1.3820 ! K2C, yxu

!!***Adenine***
CG2R64 CG331   252.00     1.5020 ! 2MA, yxu
CG2R64 NG2S0   300.00     1.3860 ! 66A, yxu
CG2R53 CG331   270.00     1.4950 ! 8MA, yxu

!!***Guanine***
CG2RC0 NG2R52  340.00     1.3810 ! 7MG, yxu
CG334  NG2R52  330.00     1.4680 ! 7MG, yxu
CG1N1  CG2R51  375.00     1.4220 ! DCG, yxu
CG2N2  CG2R51  325.00     1.4200 ! RCG, yxu
CG2R51 CG324   300.00     1.4800 ! DAG, yxu
CG2R51 CG3C53  355.00     1.4960 ! QUG, yxu
CG2R51 CG3C51  345.00     1.5100 ! QUG, yxu
CG3C53 CG3RC1  222.50     1.5240 ! EQG, from CG3C51 CG3RC1, yxu
CG3RC1 OG3C31  250.00     1.4250 ! EQG, yxu
CG2RC0 NG2R61  360.00     1.3790 ! DWG, yxu
CG2R63 NG2RC0  300.00     1.3910 ! DWG, yxu
OG301  OG311   300.00     1.4610 ! PBG, yxu

!!***Model compounds***
CG2O4  CG311   250.00     1.5000 ! amba, from CG2O4 CG321, not optimized, yxu
CG311  OG3C61  360.00     1.4150 ! pepr, from CG321 OG3C61, not optimized, yxu
CG3C53 NG3P3   320.00     1.4850 ! cpoa, not optimized, yxu
CG2R61 NG2S0   305.00     1.4140 ! dmpu, not optimized, yxu
CG3C51 NG311   220.00     1.4560 ! 7GNM, from CG3C51 NG301, not optimized, yxu

ANGLES
!!!!!!!! ################################ !!!!!!!!
!!!!!!!! ######## Nucleic Acids ######## !!!!!!!!
!!!!!!!! ################################ !!!!!!!!
!!***Adenine***

!!***Guanine***
CC3161 CC3162 OG301   115.00    109.70 ! MQG, from CG36
OG301  CC3162 HCA1     60.00    109.50 ! MQG, from CG36
CC3162 OG301  CG3C51   65.00    107.00 ! MQG, from CG36
OG301  CC3162 OC3C61   90.00    112.00 ! MQG, from Carb36

!!***Model compounds***
CN3    NN2B   CG331    70.00    120.50 ! U, from CG36
```

```
CN1T   NN2B   CG331    70.00    115.40 ! U, from CG36
NN2B   CG331  HGA3     33.43    110.10   22.53 2.179 ! U & G, from CG36
CN3    NN2    CG331    70.00    120.50 ! C, from CG36
CN1    NN2    CG331    70.00    115.40 ! C, from CG36
NN2    CG331  HGA3     33.43    110.10   22.53 2.179 ! A & C, from CG36
CN4    NN2    CG331    70.00    127.80 ! A, from CG36
CN5    NN2    CG331    70.00    125.90 ! A, from CG36
CN4    NN2B   CG331    70.00    127.80 ! G, from CG36
CN5    NN2B   CG331    70.00    125.90 ! G, from CG36

!!***2'-O-substituted ribose***
CN7B   CN7B   OG301    76.00    107.00 ! 2OM, yxu
CN7    CN7B   OG301    60.00    106.50 ! 2OM, yxu
OG301  CN7B   HN7      45.90    108.50 ! 2OM, from CG36
CG331  OG301  CN7B     65.00    107.00 ! 2OM, from CG36
CN7B   CN7B   OC301    76.00    107.00 ! 2OR, yxu
CN7    CN7B   OC301    60.00    106.50 ! 2OR, yxu
OC301  CN7B   HN7      45.90    108.50 ! 2OR, yxu
CN7B   OC301  CC3152   55.00    107.00 ! 2OR, from rbrb, yxu
CC3153 CC321  OC30P    75.70    110.10 ! 2OR, from CG3C51 CG321  OG303, yxu
CC321  OC30P  PC       20.00    120.00   35.00   2.33 ! 2OR, from CG321  OG303  PG1,
yxu

!!***glycosyl linkage***
CG2R62 NG2R61 CN7B     45.00    119.60 ! U, from NA36
CG2R63 NG2R61 CN7B     45.00    118.40 ! U, from NA36
NG2R61 CN7B   ON6B    110.00    112.00 ! U, from NA36
NG2R61 CN7B   HN7      43.00    111.00 ! U, from NA36
NG2R61 CN7B   CN7B    110.00    111.00 ! U, from NA36
CG2RC0 NG2R51 CN7B     45.00    126.30 ! G, from NA36
CG2R53 NG2R51 CN7B     45.00    126.90 ! G, from NA36
ON6B   CN7B   NG2R51  110.00    112.00 ! G, from NA36
CN7B   CN7B   NG2R51  110.00    111.00 ! G, from NA36
HN7    CN7B   NG2R51   43.00    111.00 ! G, from NA36
CG2R62 CG2R62 CN7B     36.00    121.00 ! PSU, yxu
CG2R63 CG2R62 CN7B     43.00    122.30 ! PSU, yxu
CG2R62 CN7B   CN7B     54.00    109.30 ! PSU, yxu
CG2R62 CN7B   ON6B     74.00    107.50 ! PSU, yxu
CG2R62 CN7B   HN7      48.00    111.00 ! PSU, yxu
NG2S0  CN7B   CN7B     45.00    110.80 ! H2U, yxu
NG2S0  CN7B   ON6B     85.00    108.00 ! H2U, yxu
CG2O6  NG2S0  CN7B     38.00    116.00 ! H2U, yxu, 1/12/14
CG321  NG2S0  CN7B     40.00    120.00 ! H2U, yxu, 1/12/14
NG2S0  CN7B   HN7      48.00    112.00 ! H2U, from CG36
CG2R61 NG2R61 CN7B     45.00    119.60 ! 2SC, from C
CG2R53 NG2R52 CN7B    100.00    112.00 ! 7MG, yxu
CG2RC0 NG2R52 CN7B     72.00    112.90 ! 7MG, from CG2RC0 NG2R52 CG334, yxu
NG2R52 CN7B   HN7      54.00    106.00 ! 7MG, yxu
NG2R52 CN7B   CN7B     90.00    105.50 ! 7MG, yxu
NG2R52 CN7B   ON6B     75.00    102.00 ! 7MG, yxu
NG2P1  CN7B   ON6B     85.00    108.00 ! K2C, yxu
NG2P1  CN7B   CN7B     55.00    109.30 ! K2C, yxu
NG2P1  CN7B   HN7      52.00    107.70 ! K2C, yxu
CG2R64 NG2P1  CN7B     60.00    118.10 ! K2C, yxu
CG2R61 NG2P1  CN7B     53.00    120.00 ! K2C, yxu
CG2R64 NG2R61 CN7B     50.00    115.40 ! K2Cn, yxu
CG2R51 NG2R51 CN7B     45.00    126.90 ! DCG, from NA36
!!@@Deoxyribose
CG2R62 CN7B   CN8      54.00    109.30 ! PSU, from CG2R62 CN7B CN7B, yxu
CG2R62 CN7B   ON6      74.00    107.50 ! PSU, from CG2R62 CN7B ON6B, yxu
NG2R61 CN7B   ON6     110.00    108.00 ! C/U, from NA36
NG2R61 CN7B   CN8     110.00    113.70 ! C/U, from NA36
NG2R51 CN7B   ON6     110.0     108.0  ! A/G, from NA36
```

```
NG2R51 CN7B   CN8     110.0     113.7  ! A/G, from NA36


!!!!!!!! ######################################## !!!!!!!!
!!!!!!!! ######## CGenFF model compounds ######## !!!!!!!!
!!!!!!!! ######################################## !!!!!!!!
!!***Uracils***
NG2R61 CG2R63 SEGD1    65.00    122.50 ! SEU, yxu, tmp
NG2S0  CG2O6  OG2D1    62.70    125.70 ! H2U, yxu
CG321  CG321  NG2S0    70.00    111.00 ! H2U, yxu, 21/2/14
NG2S0  CG321  HGA2     54.00    109.50 ! H2U, yxu
NG2S0  CG2O6  NG2S1    70.50    112.90 ! H2U, yxu, 21/2/14
CG321  NG2S0  CG331    40.00    121.00 ! H2U, yxu
CG2O6  NG2S0  CG321    39.50    117.00 ! H2U, yxu, 21/2/14
CG2O6  NG2S0  CG331    38.00    116.00 ! H2U, yxu, angsum 04/15
CG2O1  NG2S1  CG2O6    46.80    124.60 ! H2U, yxu, angsum 04/15
CG2R63 CG2R62 OG311    49.00    117.50 ! 5HU, yxu
CG2R62 CG2R62 OG311    51.00    123.80 ! 5HU, yxu
CG2R62 OG311  HGP1     66.00    108.00   40      1.9280  ! 5HU, yxu
CG2R62 CG2R62 OG301   120.00    120.30 ! MOU, yxu, 21/2/14
CG2R63 CG2R62 OG301   110.00    123.00 ! MOU, yxu, 21/2/14
CG2R62 OG301  CG331    65.00    104.00 ! MOU, yxu, 21/2/14
CG311  CG321  NG2S0    85.00    111.50 ! MDU, yxu
CG2R62 CG2R62 CG324    45.80    125.80 ! SAU, yxu
CG2R63 CG2R62 CG324    42.00    117.50 ! SAU, yxu
CG2R62 CG324  NG3P3    54.00    108.50   37.00   2.43000 ! SAU, yxu
CG2R62 CG324  HGA2     49.30    107.50 ! SAU, from CG2R61 CG324 HGA2, yxu
CG2R62 CG324  NG3P2    54.00    107.00   37.00   2.21000 ! 5AU, yxu, 21/2/14
NG3P2  CG334  HGA3     45.00    103.90   35.00   2.08000 ! 5AU, yxu
CG324  NG3P2  CG334    50.00    115.20 ! 5AU, yxu
CG334  NG3P2  HGP2     30.00    111.30   27.00   2.09000 ! 5AU, yxu
CG2O3  CG324  NG3P2    70.00    104.00 ! 5DU, yxu (too strong?)
CG2D1  CG2D1  CG324    39.00    122.50 ! IAU, yxu
CG324  CG2D1  HGA4     37.00    118.00 ! IAU, yxu
CG2D1  CG324  NG3P2    50.00    110.50   31.00   2.48500 ! IAU, yxu
CG331  CG2D1  CG331    55.00    119.00 ! IAU, yxu
CG2D1  CG324  HGA2     45.00    110.00 ! IAU, yxu
CG2R63 CG2R62 CG321    61.00    119.10 ! 5CU, yxu
CG2R62 CG2R62 CG321    40.00    123.00 ! 5CU, yxu
CG2R62 CG321  HGA2     49.30    107.50 ! 5CU, yxu
CG2O3  CG321  CG2R62   51.80    107.50 ! 5CU, from mebz, yxu
CG2O2  CG321  CG2R62   51.80    107.50 ! OCU, from mebz, yxu
CG2O1  CG321  CG2R62   51.80    107.50 ! BCU, from mebz, yxu
CG2O2  CG311  OG311   110.00    106.70 ! CMU, yxu
CG2R62 CG311  OG311    82.00    112.80 ! CMU, from cmbz, yxu
CG2O2  CG311  CG2R62   40.00    107.50 ! CMU, from cmbz, yxu
CG2R62 CG2R62 CG311    40.00    124.20 ! CMU, from CG2R62 CG2R62 CG331, yxu
CG2R62 CG311  HGA1     43.00    111.00 ! CMU, from CG2R61 CG311 HGA1, yxu
CG2R63 CG2R62 CG311    43.00    121.20 ! CMU, yxu
CG2O3  CG311  CG2R62   58.00    104.50 ! HCU, yxu
CG2O3  CG311  OG311    80.00    107.00 ! HCU, yxu
CG2R62 OG301  CG321    65.00    108.00 ! OAU, from CG2R61 OG301 CG321 yxu
CG2O3  CG321  OG301    63.00    109.00 ! OAU, yxu
CG2O2  CG321  OG301    70.00    109.00 ! OEU, yxu
CG2R63 NG2R61 CG321    70.00    115.40 ! 3AU, from CG2R63 NG2R61 CG331, yxu
CG321  CG321  NG2R61   67.00    112.00 ! 3AU, yxu
NG2R61 CG321  HGA2     51.50    106.00 ! 3AU, cgenff_xyu, yxu
CG324  CG321  SG3O1    35.00    104.50 ! 5TU, yxu
CG2O2  CG321  NG311    43.70    110.00 ! 5UHG, from CG2O2 CG321 NG321, yxu
CG2R62 CG321  NG311    78.00    107.00 ! 5UHG, yxu
CG2O2  CG311  NG321    43.70    110.00 ! 5UHA, from CG2O2  CG321  NG321, yxu
CG321  CG311  NG321    43.70    112.20 ! 5UHA, from CG331  CG321  NG311, yxu
```

```
NG321  CG311  HGA1     32.40    109.50   50.00   2.14000 ! 5UHA, from NG321  CG321
HGA2, cgenff_xyu, yxu
CG311  NG321  HGPAM2   41.00    112.10 ! 5UHA, from CG321  NG321  HGPAM2, cgenff_xyu,
yxu
CG2R62 CG321  NG321    78.00    107.00 ! 5UNA , from 5UHG, yxu
CG321  CG1N1  NG1T1    21.20    180.00 ! CYU, from CG331 CG1N1 NG1T1, yxu
CG1N1  CG321  CG2R62   57.00    112.00 ! CYU from cybz, yxu
CG1N1  CG321  HGA2     50.00    109.00 ! CYU, yxu

!!***Cytosines***
CG2R64 CG2R61 HGR62    38.00    120.10 ! 2SC, from CG2R62, NA36
!the following is not in CGenFF
CG2R61 CG2R64 NG2S3    60.00    118.50 ! 2SC, cgenff_compromise, xxwy, KEVO:
exocyclic N in 2SC planar, issue with CGenFF atom types?
NG2R62 CG2R63 SG2D1    70.00    123.70 ! 2SC, yxu
CG2R64 CG2R62 CG331    46.00    118.90 ! 5MC, yxu
CG2R64 CG2R62 CG321    46.00    118.50 ! HMC, from 5MC, yxu
CG2R62 CG321  OG311    75.70    109.70 ! HMC, yxu
CG2R62 CG2O4  OG2D1    75.00    126.00 ! 5FC, from CG2R61 CG2O4 OG2D1, yxu
CG2R62 CG2O4  HGR52    22.00    114.00 ! 5FC, yxu
CG2O4  CG2R62 CG2R62   50.00    120.00 ! 5FC, yxu
CG2O4  CG2R62 CG2R64   65.00    122.20 ! 5FC, yxu
NG2R62 CG2R64 NG311    66.00    121.70 ! 4MC, yxu
CG2R62 CG2R64 NG311    61.00    119.00 ! 4MC, yxu
CG2R64 NG311  HGPAM1   43.50    113.50 ! 4MC, yxu
CG2R64 NG311  CG331    46.00    114.00 ! 4MC, yxu  ###ang small, but N is not always
in planar geometry
NG2R62 CG2R64 NG2S1    44.00    122.70   35.00   2.4162 ! 4AC, yxu
CG2R62 CG2R64 NG2S1    40.00    118.00   35.00   2.2420 ! 4AC, yxu
CG2R62 CG2R64 NG2D1    48.00    124.50   43.00   2.5120 ! 3MCn, yxu
NG2D1  CG2R64 NG2R61   90.00    116.80 ! 3MCn, yxu
CG2R62 CG2R64 NG2R61   80.00    120.20 ! 3MCn, yxu
CG2R64 NG2R61 CG331    50.00    115.40 ! 3MCn, yxu
CG2R64 NG2D1  HGP1     58.00    111.00 ! 3MCn, yxu, angsum 04/15
NG2P1  CG2R63 NG2R61   61.00    115.60 ! 3MC, yxu
NG2P1  CG2R63 OG2D4    80.00    125.00 ! 3MC, yxu
CG2R63 NG2P1  CG2R64   50.00    126.40 ! 3MC, yxu
CG2R63 NG2P1  CG334    60.00    115.50 ! 3MC, yxu
CG2R64 NG2P1  CG334    60.00    118.10 ! 3MC, yxu
NG2P1  CG2R64 NG2P1    50.00    118.00   40.00   2.27500 ! 3MC, yxu
CG2R62 CG2R64 NG2P1    52.00    120.30   35.00   2.36000 ! 3MC, yxu
CG2R64 NG2P1  HGP2     40.00    120.70 ! 3MC, yxu, angsum 05/15
CG2R61 NG2P1  CG2R64   47.30    121.40 ! K2C (model: ncyp), yxu
CG2R61 NG2P1  CG334    53.00    120.00 ! K2C (model: ncyp), yxu
CG2R64 NG2P1  CG2R64   42.80    125.00   78.00   2.6700 ! K2C (model: ncyp), yxu,
angsum 05/15
CG2R61 CG2R64 NG2P1    60.00    110.00   51.00   2.5600 ! K2C (model: ncyp), yxu,
angsum 04/15
NG2D1  CG2R64 NG2P1    63.00    121.00   32.00   2.4830 ! K2C (model: ncyp), yxu,
angsum 04/15
CG2R61 CG2R61 NG2P1    39.00    121.20   43.00   2.1900 ! K2C (model: ncyp), yxu
NG2P1  CG2R61 HGR62    35.00    113.00 ! K2C, yxu
CG2R61 CG2R64 NG2D1    48.00    119.00   60.00   2.5830 ! K2Cn (model: nmcy), yxu,
angsum 04/15
NG2D1  CG2R64 NG2R62   41.00    124.00   85.00   2.5620 ! K2Cn (model: nmcy), yxu,
angsum 04/15
CG2R61 NG2R61 CG2R64   70.00    122.00 ! K2Cn, from CG2R62 NG2R61 CG2R63, yxu
CG2R64 NG311  CG321    46.00    114.00 ! K2Cn, from 4MC, yxu
CG2R64 NG2P1  CG324    60.00    118.10 ! pncp, from 3MC, yxu
CG2R63 NG2P1  HGP2     38.00    118.70 ! CYTp, yxu
CG2R62 CG2R63 NG2R62   80.00    117.80 ! 1PC, yxu
CG2R62 NG2R61 CG2R64   70.00    122.00 ! 1PC, from CG2R62 NG2R61 CG2R63, yxu
```

```
!!***Adenine***
CG331  CG2R64 NG2R62   63.50    116.00 ! 2MA, yxu
CG2R64 CG331  HGA3     33.43    110.10   22.53   2.17900 ! 2MA, from CG2R62 CG331
HGA3, yxu
CG2RC0 CG2R64 NG311    61.00    119.00 ! 6MA, from 4MC CG2R62 CG2R64 NG311, yxu
CG2RC0 CG2R64 NG2S1    40.00    118.00   35.00   2.2420 ! 6AA, from 4AC CG2R62 CG2R64
NG2S1, yxu
CG2RC0 CG2R64 NG2D1    48.00    124.50   43.00   2.5120 ! 1MAn, from 3MCn CG2R62
CG2R64 NG2D1, yxu
CG2RC0 CG2R64 NG2R61   80.00    120.20 ! 1MAn, from 3MCn CG2R62 CG2R64 NG2R61, yxu
CG2R64 NG2R61 CG2R64   70.00    131.10 ! 1MAn, from CG2R63 NG2R61 CG2R64, yxu
CG2RC0 CG2R64 NG2P1    52.00    119.00   35.00   2.36000 ! 1MA, from 3MC CG2R62
CG2R64 NG2P1, yxu, angsum
NG2P1  CG2R64 NG2R62   70.00    122.00 ! 1MA, from NG2R61 CG2R64 NG2R62, yxu, angsum
NG2P1  CG2R64 HGR62    48.00    112.60 ! 1MA, from un-angsumed NG2R61 CG2R64 HGR62,
yxu
CG2D1  CG321  NG311    78.00    110.00 ! 6IA, yxu
NG2S1  CG2O6  NG2S1    70.00    115.00 ! HNA, from NG2S2 CG2O6 NG2S2, cgenff_xyu, yxu
CG2O6  NG2S1  CG2R64   50.00    119.00 ! HNA, from mpyu CG2R61 NG2S1  CG2O6, yxu
CG2O6  NG2S1  CG311    60.00    120.00 ! HNA, from CG2O6 NG2S1 CG321, yxu
CG2O6  NG2S0  CG2R64   60.00    118.00 ! 66A, from m6pa CG2R64 NG2S0  CG2O1, yxu,
angsum 04/15
CG2R64 NG2S0  CG331    45.00    117.50 ! 66A (m6pa), yxu, angsum 04/15
CG2RC0 CG2R64 NG2S0    35.00    126.00   15.00   2.3800 ! 66A (m6pa), yxu
NG2R62 CG2R64 NG2S0    35.00    120.00   10.00   2.2500 ! 66A (m6pa), yxu
CG331  CG2R53 NG2R51   48.00    121.00 ! 8MA, yxu
CG331  CG2R53 NG2R50   45.80    126.00 ! 8MA, yxu
CG2R53 CG331  HGA3     55.00    109.50 ! 8MA, yxu

!!***Guanine***
NG2R61 CG2R64 NG311    73.00    120.00 ! MMG, from K2Cn, yxu
CG2R53 NG2R52 CG334    44.00    126.30   23.00   2.4300 ! 7MG, yxu
CG2R63 CG2RC0 NG2R52   85.00    126.50 ! 7MG, yxu
NG2R52 CG2RC0 NG2R62   72.00    126.90 ! 7MG, yxu
CG2RC0 CG2RC0 NG2R52   72.00    106.90 ! 7MG, yxu
NG2R52 CG334  HGA3     42.00    107.00 ! 7MG, yxu
CG2R53 NG2R52 CG2RC0  103.00    108.00 ! 7MG, yxu
CG2RC0 NG2R52 CG334    44.00    124.90 ! 7MG, yxu
CG2RC0 NG2R52 HGP2     39.00    126.90 ! 7MG, yxu
CG2R51 CG1N1  NG1T1    40.00    180.00 ! DCG, yxu
CG2R51 CG2RC0 CG2R63  130.00    132.00 ! DCG, from CG2R51 CG2RC0 CG2R61, yxu
CG1N1  CG2R51 CG2R51   45.00    128.70 ! DCG, yxu
CG1N1  CG2R51 CG2RC0   40.00    126.70 ! DCG, yxu
NG2R61 CG2R64 NG301    76.00    117.00 ! M2G, yxu
CG2N2  CG2R51 CG2R51   15.00    125.30 ! RCG, yxu
CG2N2  CG2R51 CG2RC0   15.00    128.00 ! RCG, yxu
CG2R51 CG2N2  NG2P1    63.00    118.50 ! RCG, yxu
CG2R51 CG324  HGA2     55.00    109.50 ! DAG, from CG2R51 CG321 HGA2, yxu
CG2R51 CG2R51 CG324    45.00    128.00 ! DAG, yxu
CG2RC0 CG2R51 CG324    40.00    127.20 ! DAG, yxu
CG2R51 CG324  NG3P3    52.00    109.00   38.00   2.45100 ! DAG, yxu
CG2R51 CG324  NG3P2    52.00    109.00   38.00   2.45100 ! QUG, from DAG, yxu
CG3C51 CG3C53 NG3P2    70.00    108.50 ! QUG, from CG3C52 CG3C53 NG3P2, yxu
CG2R51 CG2R51 CG3C51  115.00    109.00 ! QUG, from CG2R51 CG2R51 CG3C52, yxu
CG2R51 CG2R51 CG3C53  115.00    109.00 ! QUG, from CG2R51 CG2R51 CG3C54, yxu
CG3C51 CG2R51 HGR51    29.00    124.60 ! QUG, from CG3C52 CG2R51 HGR51, yxu
CG3C53 CG2R51 HGR51    13.00    124.60 ! QUG, from CG3C54 CG2R51 HGR51, yxu
CG2R51 CG3C53 HGA1     46.00    114.80 ! QUG, yxu
CG2R51 CG3C51 OG311    60.00    115.50 ! QUG, yxu
CG2R51 CG3C51 HGA1     48.00    110.10 ! QUG, yxu
CG2R51 CG3C51 CG3C51  112.00    102.70 ! QUG, from cpea, yxu
CG2R51 CG3C53 CG3C51  105.00    106.00 ! QUG, from cpea, yxu
CG2R51 CG3C53 NG3P2    77.00    109.50 ! QUG, from cpea, yxu
```

```
CG324  NG3P2  CG3C53   46.00    115.20 ! QUG, yxu
CG3C51 CG3C53 CG3RC1   53.35    103.70    8.00   2.56100 ! EQG, from CG3C51 CG3C51
CG3RC1, yxu
CG3RC1 CG3C53 NG3P2    70.00    108.50 ! EQG, from CG3C52 CG3C53 NG3P2, yxu
CG3RC1 CG3C53 HGA1     34.50    110.10   22.53   2.17900 ! EQG, from CG3RC1 CG3C51
HGA1, yxu
CG3C51 CG3RC1 OG3C31   95.00    112.00 ! EQG, from cpoa, yxu
CG3C53 CG3RC1 CG3RC1  103.00    107.00   45.00   2.38300 ! EQG, yxu
CG3C53 CG3RC1 OG3C31   93.00    105.80 ! EQG, yxu
CG3C53 CG3RC1 HGA1     17.50    105.70   44.00   2.01000 ! EQG, yxu
CG3RC1 CG3RC1 OG3C31   54.00     59.60    8.00   1.42500 ! EQG, yxu
OG3C31 CG3RC1 HGA1     73.00    112.50 ! EQG, yxu
CG3RC1 OG3C31 CG3RC1   90.00     60.80 ! EQG, yxu
CG331  CG2R51 NG2R50   45.80    120.00 ! DWG, from CG321  CG2R51 NG2R50, yxu
CG2R51 NG2R50 CG2RC0   60.00    103.00 ! DWG, from CG2R52 NG2R50 CG2RC0, yxu
CG2RC0 NG2R61 CG2RC0   30.00    120.00 ! DWG, from CG2R62 NG2R61 CG2R62, yxu
NG2R51 CG2RC0 NG2R61  100.00    126.90 ! DWG, from NG2R51 CG2RC0 NG2R62, yxu
NG2R50 CG2RC0 NG2R61   20.00    131.00 ! DWG, yxu
CG2RC0 CG2RC0 NG2R61   59.00    127.40 ! DWG, yxu
CG2R51 NG2RC0 CG2R63   55.00    122.50 ! DWG, yxu
CG2R63 NG2RC0 CG2RC0   60.00    127.80 ! DWG, yxu
CG2RC0 CG2R63 NG2RC0   70.00    108.00 ! DWG, yxu, angsum 04/15
NG2RC0 CG2R63 OG2D4    97.00    122.10 ! DWG, yxu, angsum 04/15
NG2R50 CG2RC0 NG2RC0  140.00    112.60 ! DWG, yxu
NG2R61 CG2RC0 NG2RC0   68.00    116.40 ! DWG, yxu
CG331  CG2R51 NG2RC0   60.00    126.70 ! MWG, yxu
CG2RC0 NG2R61 CG331    24.00    120.50 ! MWG, yxu
CG321  CG2R51 NG2RC0   60.00    126.70 ! HWG, from MWG, yxu
CG2O2  CG311  CG311    52.00    108.00 ! HWG, from CG2O2 CG311 CG321, yxu
CG2R51 CG321  CG321    58.35    114.00 ! WBG, from CG2R51 CG321 CG311, yxu
CG314  CG311  OG311    75.70    112.10 ! BUG, from CG324 CG311 OG311, yxu
CG3C51 CG3C51 OG301    58.00    106.50    8.00   2.56100 ! MQG, from CG36
CG3C53 CG3C51 OG301    75.70    110.10 ! MQG, from CG3C53 CG3C51 OG311, yxu
CG311  OG301  OG311    72.00    104.00 ! PBG, yxu
OG301  OG311  HGP1     61.00     98.30 ! PBG, yxu
CG311  CG311  OG301   115.00    109.70 ! PBG, from CG321 CG311 OG302, yxu
CG321  CG311  OG301   115.00    109.70 ! PBG, from CG321 CG311 OG302, yxu
CG311  CG311  NG321    73.00    111.20 ! BUGn, from CG331 CG321 NG311, yxu
CG2R51 CG321  NG321    70.00    110.00 ! 7GNA (idam), yxu
CG2R51 CG321  NG311    70.00    111.50 ! 7GNM, yxu
CG3C51 CG3C51 NG311   110.00    111.00 ! 7GNM, from CG3C51 CG3C51 NG301, yxu
NG311  CG3C51 HGA1     43.00    106.00 ! 7GNM, yxu
CG3RC1 CG3C51 NG311   110.00    111.00 ! 7GNM, from CG3C51 CG3C51 NG301, yxu
CG2R51 CG3C51 NG311    68.50    105.00 ! 7GNM (cena), yxu
CG321  NG311  CG3C51   70.00    111.00    40.00   2.3100 ! 7GNM (cena), yxu
CG3C51 NG311  HGPAM1   50.00    111.00 ! 7GNM (cena), yxu

CG2R61 CG2RC0 NG2R52  130.00    130.00 ! 7MGe, from CG2R61 CG2RC0 NG2R50, not
optimized, yxu
CG2RC0 CG2R61 OG311    45.20    120.00 ! 7MGe, from CG2R61 CG2R61 OG311, not
optimized, yxu
NG2R62 CG2R61 OG311    45.20    120.00 ! 7MGe, from CG2R61 CG2R61 OG311, not
optimized, yxu

!!***Model compounds***
CG2R51 NG2R51 CG331    70.00    127.80 ! RCG, from G, cgenff_xyu, yxu
CG2R61 CG324  NG3P2    54.00    108.50   37.00   2.43000 ! bzma, yxu
CG2O2  CG321  CG2R61   51.80    107.50 ! mebz, yxu
CG2O1  CG321  CG2R61   51.80    107.50 ! acbz, from mebz, yxu
CG2R61 CG311  OG311    82.00    112.80 ! cmbz, yxu
CG2O2  CG311  CG2R61   40.00    107.50 ! cmbz, yxu
CG321  NG311  CG331    48.00    112.20   38.00   2.35000 ! nmgn, yxu
CG2R61 CG321  NG311    73.00    109.00  ! ambz, yxu
```

```
CG2R61 NG2P1  HGP2     47.00    120.00 ! ncyp, yxu
CG2O6  NG2S1  CG2R61   50.00    119.00 ! mpyu, yxu
CG2O1  NG2S0  CG2R64   60.00    118.00 ! m6pa, yxu, angsum 04/15
CG3C51 CG3C52 CG3C53   70.00    108.50 ! cpea, from CG3C52 CG3C52 CG3C53, yxu
CG2R51 CG3C51 CG3C52  112.00    102.70 ! cpea, yxu
CG2R51 CG3C53 CG3C52  105.00    106.00 ! cpea, yxu
CG2R51 CG3C53 NG3P3    77.00    109.50 ! cpea, yxu
CG3C52 CG3RC1 OG3C31   95.00    112.00 ! cpoa, yxu
CG2RC0 NG2R61 HGP1     32.00    117.40 ! DWG, from CG2R62 NG2R61 HGP1, yxu
CG331  CG311  NG321    73.00    113.20 ! aboh, cgenff_xyu, yxu
CG3C51 OG301  CG3C51   55.00    107.00 ! rbrb, yxu
OG301  CG3C51 OG3C51  100.00    112.00 ! rbrb, yxu
CG331  OG301  OG311    72.00    104.00 ! mhp, from ppox, yxu
CG2R61 NG2R61 CG331    70.00    120.50 ! 34c, yxu
CG2R61 CG2R61 NG2S0    40.00    120.00   35.00   2.41620 ! dmpu, from CG2R61 CG2R61
NG2S1, yxu
CG2O6  NG2S0  CG2R61   60.00    115.00 ! dmpu, from m6pa, yxu
CG2R61 NG2S0  CG331    45.00    115.50 ! dmpu, from m6pa, yxu
CG3C52 CG3C53 NG3P3    70.00    108.50 ! cpoa, from CG3C52 CG3C53 NG3P2, yxu
NG3P3  CG3C53 HGA1     51.50    107.50 ! cpoa, from NG3P2 CG3C53 HGA1, yxu
CG3C53 NG3P3  HGP2     30.00    109.50   20.00   2.07400 ! cpoa, from CG314 NG3P3
HGP2, yxu
CG3C52 CG3C53 CG3RC1   53.35    103.70    8.00   2.56100 ! cpoa, from CG3C52 CG3C51
CG3RC1, yxu
CG3RC1 CG3C53 NG3P3    70.00    108.50 ! cpoa, from CG3C52 CG3C53 NG3P2, yxu
CG2O3  CG321  CG2R61   51.80    107.50 ! bzac, from mebz, yxu
CG3C52 CG3C51 NG311   110.00    113.00 ! cena, yxu
CG331  NG311  CG3C51   90.00    112.20    35.00   2.3600 ! cona, from cena, yxu
NG2R60 CG2R64 NG311    78.00    119.70 ! bepa, from 4MC, yxu
CG2R61 CG2R64 NG311    63.00    121.00 ! bepa, from 4MC, yxu
CG334  NG3P2  CG3C53   46.00    115.20 ! ponm, from pnpa, yxu
CG1N1  CG321  CG2R61   57.00    112.00 ! cybz, yxu
CG2R61 CG324  NG3P3    45.00    107.00 ! bzam, cgenff_compromise, pram
NG2R62 CG2R62 OG311    89.00    121.00   90.0    2.308  ! enoU, yxu
OG301  CG311  OG3C61   90.00    112.00 ! MQG, from Carb36
CG2R64 SG311  CG321    46.00     97.00 ! pesu, yxu
CG2D1  CG321  SG311    47.00    109.80 ! pesu, yxu
CG334  NG3P2  CG334    50.00    115.20 ! dma, not optimized, yxu
NG2S1  CG2O1  HGR52    44.00    111.00   50.00   1.98000 ! amba, from NG2S2 CG2O1
HGR52, not optimized, yxu
CG311  CG2O4  OG2D1    45.00    126.00 ! amba, from CG321 CG2O4 OG2D1, not optimized,
yxu
CG311  CG2O4  HGR52    65.00    116.00 ! amba, from CG321 CG2O4 HGR52, not optimized,
yxu
CG2O4  CG311  CG311    52.00    108.00 ! amba, from CG2O2 CG311 CG321, not optimized,
yxu
CG2O4  CG311  NG2S1    50.00    107.00 ! amba, from CG2O2 CG311 NG2S1, not optimized,
yxu
CG2O4  CG311  HGA1     50.00    109.50 ! amba, from CG2O5 CG311 HGA1, not optimized,
yxu
CG2R62 NG2R62 CG2R63   40.00    110.50 ! 5fop, from CG2R61 NG2R62 CG2R64, not
optimized, yxu
CG321  CG311  OG3C61   45.00    111.50 ! pepr, from CG321 CG321 OG3C61, not
optimized, yxu
OG3C61 CG311  HGA1     45.00    109.50 ! pepr, from OG3C61 CG321 HGA2, not optimized,
yxu
CG2R51 CG3C52 CG3C51   52.00    106.00 ! pepr, from CG2R51 CG3C52 CG3C52, not
optimized, yxu
CG311  OG301  CG3C51   65.00    107.00 ! pepr, from CG331 OG301 CG3C51, not
optimized, yxu
CG311  OG3C61 CG321    95.00    109.70 ! pepr, from CG321 OG3C61 CG321, not
optimized, yxu
```

```
CG2O2  CG311  CG331    52.00    108.00 ! hpme, from CG2O2 CG311 CG321, not optimized,
yxu

DIHEDRALS
!!!!!!!! ############################### !!!!!!!!
!!!!!!!! ######## Nucleic Acids ######## !!!!!!!!
!!!!!!!! ############################### !!!!!!!!
!!***Cytosines***
NG2P1  CG2R63 NG2R61 CN7B      11.000  2   180.0 ! CYTp, from NA36
CG2R61 CG2R61 NG2R61 CN7B      11.000  2   180.0 ! 2SC, from C
HGR62  CG2R61 NG2R61 CN7B       0.300  2   180.0 ! 2SC, from C

!!***Guanine***
OG301  CC3162 CC3161 CC3161     0.200  3     0.0 ! MQG, from Carb36
OG301  CC3162 CC3161 OC311      2.650  1   180.0 ! MQG, from Carb36
OG301  CC3162 CC3161 OC311      0.000  2     0.0 ! MQG, from Carb36
OG301  CC3162 CC3161 OC311      0.130  3   180.0 ! MQG, from Carb36
OG301  CC3162 CC3161 HCA1       0.200  3     0.0 ! MQG, from Carb36
OG301  CC3162 OC3C61 CC3163     0.200  3     0.0 ! MQG, from Carb36
CC3161 CC3162 OG301  CG3C51     0.450  3     0.0 ! MQG, from pepr, yxu
OC3C61 CC3162 OG301  CG3C51     0.820  1     0.0 ! MQG, from pepr, yxu
OC3C61 CC3162 OG301  CG3C51     0.810  2     0.0 ! MQG, from pepr, yxu
OC3C61 CC3162 OG301  CG3C51     0.350  3     0.0 ! MQG, from pepr, yxu
HCA1   CC3162 OG301  CG3C51     0.350  3     0.0 ! MQG, from pepr, yxu
CG3C51 CG3C51 OG301  CC3162     0.200  1   180.0 ! MQG, from pepr, yxu
CG3C51 CG3C51 OG301  CC3162     0.300  3     0.0 ! MQG, from pepr, yxu
CG3C53 CG3C51 OG301  CC3162     0.200  1   180.0 ! MQG, from pepr, yxu
CG3C53 CG3C51 OG301  CC3162     0.300  3     0.0 ! MQG, from pepr, yxu
HGA1   CG3C51 OG301  CC3162     0.500  1     0.0 ! MQG, from pepr, yxu
HGA1   CG3C51 OG301  CC3162     0.600  3   180.0 ! MQG, from pepr, yxu

!!***Model compounds***
CN3    NN2B   CG331  HGA3       0.000  3     0.0 ! U, from CG36
CN1T   NN2B   CG331  HGA3       0.190  3     0.0 ! U, from CG36
CN3    NN2    CG331  HGA3       0.000  3     0.0 ! C, from CG36
CN1    NN2    CG331  HGA3       0.190  3     0.0 ! C, from CG36
CN4    NN2    CG331  HGA3       0.000  3     0.0 ! A, from CG36A
CN5    NN2    CG331  HGA3       0.190  3     0.0 ! A, from CG36
CN4    NN2B   CG331  HGA3       0.000  3     0.0 ! G, from CG36A
CN5    NN2B   CG331  HGA3       0.190  3     0.0 ! G, from CG36

!!***2'-O-substituted ribose***
OG301  CN7B   CN7    ON2        0.000  3     0.0 ! 2OM, yxu
OG301  CN7B   CN7    ON5        0.000  3     0.0 ! 2OM, yxu
OG301  CN7B   CN7B   ON6B       0.000  3     0.0 ! 2OM, yxu
OG301  CN7B   CN7    CN7        0.000  3     0.0 ! 2OM, yxu
CG331  OG301  CN7B   CN7B       1.300  2   180.0 ! 2OM, yxu lower to increase base-
orientation
CG331  OG301  CN7B   CN7B       0.400  3     0.0 ! 2OM, yxu
CG331  OG301  CN7B   CN7        1.600  2   180.0 ! 2OM, yxu
CG331  OG301  CN7B   CN7        0.300  3     0.0 ! 2OM, yxu
CG331  OG301  CN7B   HN7        1.000  1     0.0 ! 2OM, yxu
CG331  OG301  CN7B   HN7        1.600  2   180.0 ! 2OM, yxu
CG331  OG301  CN7B   HN7        0.400  3     0.0 ! 2OM, yxu
OG301  CN7B   CN7B   HN7        0.195  3     0.0 ! 2OM, yxu
OG301  CN7B   CN7    HN7        0.195  3     0.0 ! 2OM, from NA36
CN9    CN7B   CN7B   OG301      0.000  3   180.0 ! 2OM, from NA36
HGA3   CG331  OG301  CN7B       0.200  1   180.0 ! 2OM, from CG36
HGA3   CG331  OG301  CN7B       1.200  2   180.0 ! 2OM, from CG36
NN2    CN7B   CN7B   OG301      0.000  3     0.0 ! 2OM(A/C), yxu 0.2 180 pyrimidine:
doesn't change pucker but enlarge 2OM torsion effect
NN2B   CN7B   CN7B   OG301      0.000  3     0.0 ! 2OM(G/U), yxu         purine:
increase C3'endo a little
```

```
NG2R61 CN7B   CN7B   OG301       0.000  3     0.0 ! 2OM(U), yxu
NG2R51 CN7B   CN7B   OG301       0.000  3     0.0 ! 2OM(G), yxu
CG2R62 CN7B   CN7B   OG301       0.000  3     0.0 ! 2OM(P), yxu
NG2R52 CN7B   CN7B   OG301       0.000  3     0.0 ! 2OM(G+), yxu
OC301  CN7B   CN7    ON2         0.000  3     0.0 ! 2OR, yxu
OC301  CN7B   CN7    ON5         0.000  3     0.0 ! 2OR, yxu
OC301  CN7B   CN7B   ON6B        0.000  3     0.0 ! 2OR, yxu
OC301  CN7B   CN7    CN7         0.000  3     0.0 ! 2OR, yxu
OC301  CN7B   CN7B   HN7         0.195  3     0.0 ! 2OR, yxu
OC301  CN7B   CN7    HN7         0.195  3     0.0 ! 2OR, from NA36
NN2    CN7B   CN7B   OC301       0.000  3     0.0 ! 2OR(A), yxu
NN2B   CN7B   CN7B   OC301       0.000  3     0.0 ! 2OR(G), yxu
CN7    CN7B   OC301  CC3152      0.300  1   180.0 ! 2OR, from rbrb, yxu
CN7    CN7B   OC301  CC3152      0.800  2   180.0 ! 2OR, from rbrb, yxu
CN7B   CN7B   OC301  CC3152      0.300  1   180.0 ! 2OR, from rbrb, yxu
CN7B   CN7B   OC301  CC3152      0.800  2   180.0 ! 2OR, from rbrb, yxu
HN7    CN7B   OC301  CC3152      0.300  1     0.0 ! 2OR, from rbrb, yxu
HN7    CN7B   OC301  CC3152      0.800  2   180.0 ! 2OR, from rbrb, yxu
CC3151 CC3152 OC301  CN7B        0.300  1   180.0 ! 2OR, from rbrb, yxu
CC3151 CC3152 OC301  CN7B        0.800  2   180.0 ! 2OR, from rbrb, yxu
OC3C51 CC3152 OC301  CN7B        0.600  2     0.0 ! 2OR, from rbrb, yxu
OC3C51 CC3152 OC301  CN7B        0.200  3     0.0 ! 2OR, from rbrb, yxu
HCA1   CC3152 OC301  CN7B        0.300  1     0.0 ! 2OR, from rbrb, yxu
HCA1   CC3152 OC301  CN7B        0.800  2   180.0 ! 2OR, from rbrb, yxu
CC3153 CC321  OC30P  PC          0.600  1   180.0 ! 2OR, from CG3C51 CG321  OG303
PG1, yxu
CC3153 CC321  OC30P  PC          0.650  2     0.0 ! 2OR, from CG3C51 CG321  OG303
PG1, yxu
CC3153 CC321  OC30P  PC          0.050  3     0.0 ! 2OR, from CG3C51 CG321  OG303
PG1, yxu
CC321  OC30P  PC     OC2DP       0.100  3     0.0 ! 2OR, from CG321  OG303  PG1
OG2P1, yxu
OC3C51 CC3153 CC321  OC30P       3.400  1   180.0 ! 2OR, from OG303  CG321  CG3C51
OG3C51 , yxu
HCA1   CC3153 CC321  OC30P       0.195  3     0.0 ! 2OR, from OG303  CG321  CG3C51
HGA1, yxu
CC3151 CC3153 CC321  OC30P       2.500  1   180.0 ! 2OR, from OG303  CG321  CG3C51
CG3C51, yxu
CC3151 CC3153 CC321  OC30P       0.400  2     0.0 ! 2OR, from OG303  CG321  CG3C51
CG3C51, yxu
CC3151 CC3153 CC321  OC30P       0.800  3   180.0 ! 2OR, from OG303  CG321  CG3C51
CG3C51, yxu
CC3151 CC3153 CC321  OC30P       0.200  4   180.0 ! 2OR, from OG303  CG321  CG3C51
CG3C51, yxu
CC321  OC30P  PC     OC312       0.950  2     0.0 ! 2OR, from CG321  OG303  PG1
OG311, yxu
CC321  OC30P  PC     OC312       0.500  3     0.0 ! 2OR, from CG321  OG303  PG1
OG311, yxu
HCA2   CC321  OC30P  PC          0.000  3     0.0 ! 2OR, from HGA2   CG321  OG303
PG1, yxu

!!***glycosyl linkage***
!!@@general
NG2R61 CN7B   CN7B   ON5         0.000  3     0.0 ! U, from NA36
NG2R61 CN7B   CN7B   HN7         0.000  3     0.0 ! U, from NA36
NG2R61 CN7B   ON6B   CN7         0.000  3     0.0 ! U, from NA36
NG2R61 CN7B   CN7B   CN7         0.000  3     0.0 ! U, from NA36
NG2R51 CN7B   CN7B   ON5         0.000  3     0.0 ! G, from NA36
NG2R51 CN7B   CN7B   HN7         0.000  3     0.0 ! G, from NA36
NG2R51 CN7B   ON6B   CN7         0.000  3     0.0 ! G, from NA36
NG2R51 CN7B   CN7B   CN7         0.000  3     0.0 ! G, from NA36
HGR62  CG2R62 NG2R61 CN7B        0.300  2   180.0 ! U, from NA36
OG2D4  CG2R63 NG2R61 CN7B       11.000  2   180.0 ! U, from NA36
```

```
CG2R62 CG2R62 NG2R61 CN7B      11.000  2   180.0 ! U, from NA36
NG2R61 CG2R63 NG2R61 CN7B      11.000  2   180.0 ! U, from NA36
NG2R62 CG2R63 NG2R61 CN7B      11.000  2   180.0 ! C, from NA36
HGR52  CG2R53 NG2R51 CN7B       0.300  2   180.0 ! G, from NA36
NG2R50 CG2R53 NG2R51 CN7B      11.000  2   180.0 ! G, from NA36
CG2RC0 CG2RC0 NG2R51 CN7B      11.000  2   180.0 ! G, from NA36
NG2R62 CG2RC0 NG2R51 CN7B      11.000  2   180.0 ! G, from NA36
CG2R62 NG2R61 CN7B   CN7B       0.000  3   180.0 ! U & C, chi, from CG36
CG2R63 NG2R61 CN7B   CN7B       1.000  3     0.0 ! U & C, chi, from CG36
CG2R62 NG2R61 CN7B   ON6B       1.000  1     0.0 ! U & C, chi, from CG36
CG2R63 NG2R61 CN7B   ON6B       0.000  3     0.0 ! U & C, chi, from CG36
CG2R62 NG2R61 CN7B   HN7        0.195  3     0.0 ! U & C, chi, from CG36
CG2R63 NG2R61 CN7B   HN7        0.195  3     0.0 ! U & C, chi, from CG36
CG2R53 NG2R51 CN7B   CN7B       0.000  3   180.0 ! A & G, chi, from CG36
CG2RC0 NG2R51 CN7B   CN7B       0.000  3     0.0 ! A & G, chi, from CG36
CG2R53 NG2R51 CN7B   ON6B       1.100  1     0.0 ! A & G, chi, from CG36
CG2RC0 NG2R51 CN7B   ON6B       1.100  1   180.0 ! A & G, chi, from CG36
CG2RC0 NG2R51 CN7B   ON6B       0.200  3     0.0 ! A & G, chi, from CG36
CG2R53 NG2R51 CN7B   HN7        0.250  2   180.0 ! A & G, chi, from CG36
CG2R53 NG2R51 CN7B   HN7        0.195  3     0.0 ! A & G, chi, from CG36
CG2RC0 NG2R51 CN7B   HN7        0.250  2   180.0 ! A & G, chi, from CG36
!!@@pseudouridine
CG2R62 CN7B   CN7B   CN7        0.000  3     0.0 ! PSU, yxu
CG2R62 CN7B   CN7B   ON5        0.000  3     0.0 ! PSU, yxu
CG2R62 CN7B   CN7B   HN7        0.000  3   180.0 ! PSU, yxu  enhance C3'endo
CG2R62 CN7B   ON6B   CN7        0.000  3     0.0 ! PSU, yxu
NG2R61 CG2R62 CG2R62 CN7B      11.000  2   180.0 ! PSU, from NA36
HGR62  CG2R62 CG2R62 CN7B       4.000  2   180.0 ! PSU, yxu
NG2R61 CG2R63 CG2R62 CN7B      11.000  2   180.0 ! PSU, from NA36
OG2D4  CG2R63 CG2R62 CN7B      11.000  2   180.0 ! PSU, from NA36
CG2R63 CG2R62 CN7B   ON6B       0.300  1   180.0 ! PSU, chi, yxu 1.1 higher, more syn
CG2R62 CG2R62 CN7B   ON6B       0.700  3   180.0 ! PSU, chi, yxu 0.7
CG2R63 CG2R62 CN7B   CN7B       1.300  1   180.0 ! PSU, chi, yxu 1.3
CG2R62 CG2R62 CN7B   CN7B       0.800  3   180.0 ! PSU, chi, yxu 0.8
CG2R63 CG2R62 CN7B   HN7        0.000  3     0.0 ! PSU, chi, yxu 0.0
CG2R62 CG2R62 CN7B   HN7        0.000  3   180.0 ! PSU, chi, yxu 0.0
!!@@dihydrouridine
NG2S0  CN7B   CN7B   CN7        0.200  3     0.0 ! H2U, enhance C2'endo, yxu
NG2S0  CN7B   CN7B   ON5        0.200  3     0.0 ! H2U, enhance C2'endo, yxu
NG2S0  CN7B   CN7B   HN7        0.200  3     0.0 ! H2U, enhance C2'endo, yxu
NG2S0  CN7B   ON6B   CN7        0.000  3     0.0 ! H2U, yxu
NG2S1  CG2O6  NG2S0  CN7B       3.200  2   180.0 ! H2U, yxu
OG2D1  CG2O6  NG2S0  CN7B       4.700  2   180.0 ! H2U, yxu
CG321  CG321  NG2S0  CN7B       0.100  1     0.0 ! H2U, yxu
CG311  CG321  NG2S0  CN7B       0.100  1     0.0 ! MDU, from H2U, yxu
HGA2   CG321  NG2S0  CN7B       0.320  3     0.0 ! H2U, yxu
CG2O6  NG2S0  CN7B   ON6B       2.000  1   180.0 ! H2U, chi, yxu
CG2O6  NG2S0  CN7B   ON6B       0.900  2     0.0 ! H2U, chi, yxu
CG321  NG2S0  CN7B   ON6B       1.700  1     0.0 ! H2U, chi, yxu
CG321  NG2S0  CN7B   ON6B       0.400  2     0.0 ! H2U, chi, yxu
CG2O6  NG2S0  CN7B   CN7B       1.000  1   180.0 ! H2U, chi, yxu
CG321  NG2S0  CN7B   CN7B       0.000  3     0.0 ! H2U, chi, yxu
CG2O6  NG2S0  CN7B    HN7       0.000  3     0.0 ! H2U, chi, yxu
CG321  NG2S0  CN7B    HN7       0.000  3     0.0 ! H2U, chi, yxu
!!@@2-thiocytidine
CG2R61 NG2R61 CN7B   CN7B       0.000  3   180.0 ! 2SC, from C
CG2R61 NG2R61 CN7B   ON6B       1.000  1     0.0 ! 2SC, from C
CG2R61 NG2R61 CN7B   HN7        0.195  3     0.0 ! 2SC, from C
!!@@2-thiouridine
SG2D1  CG2R63 NG2R61 CN7B      11.000  2   180.0 ! 2SU, yxu
CG2R64 NG2R61 CN7B   ON6B       0.800  1   180.0 ! GAU, from U, yxu
CG2R64 NG2R61 CN7B   ON6B       0.700  3     0.0 ! GAU, from U, yxu
CG2R64 NG2R61 CN7B   HN7        0.000  3     0.0 ! GAU, from U, yxu
```

```
CG2R64 NG2R61 CN7B   CN7B       0.200  3   180.0 ! GAU, from U, yxu
SG311  CG2R64 NG2R61 CN7B       6.000  2   180.0 ! GAU, yxu
NG2R62 CG2R64 NG2R61 CN7B      11.000  2   180.0 ! GAU, from u, yxu
!!@@7-methylguanosine
NG2R52 CN7B   CN7B   CN7        0.000  3     0.0 ! 7MG, yxu
NG2R52 CN7B   CN7B   ON5        0.000  3     0.0 ! 7MG, yxu
NG2R52 CN7B   CN7B   HN7        0.000  3     0.0 ! 7MG, yxu
NG2R52 CN7B   ON6B   CN7        0.000  3     0.0 ! 7MG, yxu
NG2R52 CG2R53 NG2R52 CN7B       7.700  2   180.0 ! 7MG, from NG2R52 CG2R53 NG2R52
C334 , yxu
HGR53  CG2R53 NG2R52 CN7B       6.300  2   180.0 ! 7MG, from HGR53  CG2R53 NG2R52
C334, yxu
CG2RC0 CG2RC0 NG2R52 CN7B       5.500  2   180.0 ! 7MG, yxu
NG2R62 CG2RC0 NG2R52 CN7B      11.000  2   180.0 ! 7MG, from NA36
CG2R53 NG2R52 CN7B   CN7B       0.850  3   180.0 ! 7MG, chi, yxu
CG2RC0 NG2R52 CN7B   CN7B       1.100  1   180.0 ! 7MG, chi, yxu
CG2R53 NG2R52 CN7B   ON6B       0.700  1     0.0 ! 7MG, chi, yxu
CG2RC0 NG2R52 CN7B   ON6B       0.400  1   180.0 ! 7MG, chi, yxu
CG2R53 NG2R52 CN7B   HN7        0.000  3   180.0 ! 7MG, chi, yxu
CG2RC0 NG2R52 CN7B   HN7        0.100  3     0.0 ! 7MG, chi, yxu
!!@@7-deazaguanosine
CG2R51 NG2R51 CN7B   CN7B       0.000  3   180.0 ! DCG, from G
CG2R51 NG2R51 CN7B   ON6B       1.100  1     0.0 ! DCG, from G
CG2R51 NG2R51 CN7B   HN7        0.250  2   180.0 ! DCG, from G
CG2R51 NG2R51 CN7B   HN7        0.195  3     0.0 ! DCG, from G
CG2R51 CG2R51 NG2R51 CN7B      11.000  2   180.0 ! DCG, from G
HGR52  CG2R51 NG2R51 CN7B       0.300  2   180.0 ! DCG, from G
!!@@lysidine
NG2P1  CN7B   ON6B   CN7        0.000  3     0.0 ! K2C, from NA36
NG2P1  CN7B   CN7B   CN7        0.000  3     0.0 ! K2C, from NA36
NG2P1  CN7B   CN7B   ON5        0.000  3     0.0 ! K2C, from NA36
NG2P1  CN7B   CN7B   HN7        0.000  3     0.0 ! K2C, from NA36
CG2R61 CG2R61 NG2P1  CN7B       0.750  2   180.0 ! K2C, yxu
HGR62  CG2R61 NG2P1  CN7B       2.700  2   180.0 ! K2C, yxu
NG2P1  CG2R64 NG2P1  CN7B       1.520  2   180.0 ! K2C, yxu
NG311  CG2R64 NG2R61 CN7B       3.500  2   180.0 ! 34HC, yxu
CG2R61 NG2P1  CN7B   CN7B       0.000  1     0.0 ! K2C, chi, yxu
CG2R64 NG2P1  CN7B   CN7B       0.000  1   180.0 ! K2C, chi, yxu
CG2R61 NG2P1  CN7B   ON6B       1.300  1   180.0 ! K2C, chi, yxu
CG2R61 NG2P1  CN7B   ON6B       0.000  2     0.0 ! K2C, chi, yxu
CG2R64 NG2P1  CN7B   ON6B       1.300  1     0.0 ! K2C, chi, yxu
CG2R64 NG2P1  CN7B   ON6B       0.000  2     0.0 ! K2C, chi, yxu
CG2R61 NG2P1  CN7B   HN7        0.000  3   180.0 ! K2C, chi, yxu
CG2R64 NG2P1  CN7B   HN7        0.000  3     0.0 ! K2C, chi, yxu
!!@@others
SEGD1  CG2R63 NG2R61 CN7B      11.000  2   180.0 ! SEU, yxu
NG2R61 CG2RC0 NG2R51 CN7B       7.500  2   180.0 ! DWG, yxu
CG331  CG2R53 NG2R51 CN7B       3.000  2   180.0 ! 8MA, yxu
NG2R62 CG2R63 CG2R62 CN7B       9.000  2   180.0 ! 1PC, yxu, 21/2/14
NG2R62 CG2R62 CG2R62 CN7B       9.000  2   180.0 ! 3PC, yxu, 21/2/14
!!@@Deoxyribose
CG2R62 CN7B   CN8    CN7        0.000  3     0.0 ! PSU, yxu
CG2R62 CN7B   CN8    HN8        0.000  3     0.0 ! PSU, yxu
CG2R62 CN7B   ON6    CN7        0.000  3     0.0 ! PSU, yxu
CG2R63 CG2R62 CN7B   ON6        0.300  1   180.0 ! PSU, yxu
CG2R62 CG2R62 CN7B   ON6        0.700  3   180.0 ! PSU, yxu
CG2R63 CG2R62 CN7B   CN8        1.000  1   180.0 ! PSU, yxu
CG2R62 CG2R62 CN7B   CN8        0.800  3   180.0 ! PSU, yxu
NG2R61 CN7B   ON6    CN7        0.000  3     0.0 ! C/U, from NA36
NG2R61 CN7B   CN8    HN8        0.000  3     0.0 ! C/U, from NA36
NG2R61 CN7B   CN8    CN7        0.000  3     0.0 ! C/U, from NA36
NG2R51 CN7B   ON6    CN7        0.000  3     0.0 ! A/G, from NA36
NG2R51 CN7B   CN8    CN7        0.000  3     0.0 ! A/G, from NA36
```

```
NG2R51 CN7B   CN8    HN8        0.000  3     0.0 ! A/G, from NA36
CG2R62 NG2R61 CN7B   CN8        0.000  3   180.0 ! U & C, chi, from CG36
CG2R63 NG2R61 CN7B   CN8        1.000  3     0.0 ! U & C, chi, from CG36
CG2R62 NG2R61 CN7B   ON6        1.000  1     0.0 ! U & C, chi, from CG36
CG2R63 NG2R61 CN7B   ON6        0.000  3     0.0 ! U & C, chi, from CG36
CG2R53 NG2R51 CN7B   CN8        0.000  3   180.0 ! A & G, chi, from CG36
CG2RC0 NG2R51 CN7B   CN8        0.000  3     0.0 ! A & G, chi, from CG36
CG2R53 NG2R51 CN7B   ON6        1.100  1     0.0 ! A & G, chi, from CG36
CG2RC0 NG2R51 CN7B   ON6        1.100  1   180.0 ! A & G, chi, from CG36
CG2RC0 NG2R51 CN7B   ON6        0.200  3     0.0 ! A & G, chi, from CG36

!!!!!!!! ######################################## !!!!!!!!
!!!!!!!! ######## CGenFF model compounds ######## !!!!!!!!
!!!!!!!! ######################################## !!!!!!!!
!***Uracils***
SEGD1  CG2R63 NG2R61 CG2R62     2.5000  2   180.00 ! SEU, yxu, tmp
SEGD1  CG2R63 NG2R61 CG2R63     2.5000  2   180.00 ! SEU, yxu, tmp
SEGD1  CG2R63 NG2R61 HGP1       0.4500  2   180.00 ! SEU, yxu, tmp
HGA3   CG331  NG2S0  CG2O6      0.0000  3     0.00 ! H2U, from HGA3 CG331 NG2S0
CG2O1, yxu
HGA3   CG331  NG2S0  CG321      0.4200  3     0.00 ! H2U, from HGA3 CG331 NG2S0
CG331, yxu
HGA2   CG321  NG2S0  CG2O6      0.0000  3     0.00 ! H2U, yxu
CG2O1  CG321  CG321  NG2S0      0.3800  3     0.00 ! H2U, yxu
NG2S0  CG321  CG321  HGA2       0.1950  3     0.00 ! H2U, yxu
NG2S1  CG2O6  NG2S0  CG321      0.2000  2   180.00 ! H2U, yxu
OG2D1  CG2O6  NG2S0  CG321      2.8000  2   180.00 ! H2U, yxu
NG2S0  CG2O6  NG2S1  CG2O1      1.3000  2   180.00 ! H2U, yxu
OG2D1  CG2O6  NG2S1  CG2O1      0.8000  2   180.00 ! H2U, yxu
NG2S0  CG2O6  NG2S1  HGP1       0.9000  2   180.00 ! H2U, yxu
CG321  CG2O1  NG2S1  CG2O6      1.6500  2   180.00 ! H2U, yxu
OG2D1  CG2O1  NG2S1  CG2O6      1.2000  2   180.00 ! H2U, yxu
CG321  CG321  NG2S0  CG2O6      0.0000  3     0.00 ! H2U, yxu
CG2O1  CG311  CG321  NG2S0      0.4500  3     0.00 ! MDU, yxu
HGA1   CG311  CG321  NG2S0      0.3000  3     0.00 ! MDU, yxu
CG331  CG311  CG321  NG2S0      0.3500  3     0.00 ! MDU, yxu
CG311  CG321  NG2S0  CG2O6      0.0000  3     0.00 ! MDU, yxu
CG311  CG321  NG2S0  CG331      0.1000  3   180.00 ! MDU, from H2U_m, yxu, 8/8,13
CG311  CG2O1  NG2S1  CG2O6      0.7400  2   180.00 ! MDU, from H2U, yxu
NG2R61 CG2R62 CG2R62 OG311      3.0000  2   180.00 ! 5HU, yxu
OG311  CG2R62 CG2R62 HGR62      2.7000  2   180.00 ! 5HU, yxu
OG311  CG2R62 CG2R63 NG2R61     2.7000  2   180.00 ! 5HU, yxu
OG311  CG2R62 CG2R63 OG2D4      0.0000  2   180.00 ! 5HU, yxu
CG2R63 CG2R62 OG311  HGP1       0.4100  1   180.00 ! 5HU, yxu lower if interaction
between HO5 and O4 is weakened in solution
CG2R63 CG2R62 OG311  HGP1       0.9900  2   180.00 ! 5HU, yxu
CG2R63 CG2R62 OG311  HGP1       0.1000  3     0.00 ! 5HU, yxu
CG2R62 CG2R62 OG311  HGP1       0.9900  2   180.00 ! 5HU, yxu
NG2R61 CG2R62 CG2R62 OG301      1.6000  2   180.00 ! MOU, yxu, 21/2/14
OG301  CG2R62 CG2R62 HGR62      3.7000  2   180.00 ! MOU, yxu, 21/2/14
OG301  CG2R62 CG2R63 NG2R61     2.5000  2   180.00 ! MOU, yxu, 21/2/14
OG301  CG2R62 CG2R63 OG2D4      0.0000  2   180.00 ! MOU, yxu, 21/2/14
CG2R63 CG2R62 OG301  CG331      1.4000  1     0.00 ! MOU, yxu
CG2R63 CG2R62 OG301  CG331      1.0000  2   180.00 ! MOU, yxu
CG2R63 CG2R62 OG301  CG331      1.3000  3     0.00 ! MOU, yxu
CG2R62 CG2R62 OG301  CG331      1.5800  2   180.00 ! MOU, from bzmo, yxu
CG2R62 CG2R62 OG301  CG331      0.2000  4   180.00 ! MOU, from bzmo, yxu
HGA3   CG331  OG301  CG2R62     0.0850  3     0.00 ! MOU, from HGA3 CG331 OG301
CG2R61, yxu
CG2R62 CG2R63 NG2R61 CG331      5.5000  2   180.00 ! 3MP, yxu
CG324  CG2R62 CG2R62 NG2R61     3.2000  2   180.00 ! SAU, yxu, 21/2/14
CG324  CG2R62 CG2R62 HGR62      5.4000  2   180.00 ! SAU, yxu, 21/2/14
CG324  CG2R62 CG2R63 NG2R61     4.5000  2   180.00 ! SAU, yxu, 21/2/14
```

```
CG324  CG2R62 CG2R63 OG2D4      0.0000  2   180.00 ! SAU, yxu, 21/2/14
CG2R62 CG324  NG3P3  HGP2       0.0400  3     0.00 ! SAU, from CG2R61 CG324 NG3P1
HGP2, yxu
CG2R62 CG2R62 CG324  NG3P3      0.5000  2   180.00 ! SAU, from bzam, yxu
CG2R62 CG2R62 CG324  HGA2       0.0500  2     0.00 ! SAU, yxu, 21/2/14
CG2R63 CG2R62 CG324  NG3P3      0.7500  2     0.00 ! SAU, yxu, 21/2/14
CG2R63 CG2R62 CG324  HGA2       0.5500  3     0.00 ! SAU, yxu, 21/2/14
CG2R62 CG324  NG3P2  CG334      0.6500  1   180.00 ! 5AU, from bzma, yxu
CG2R62 CG324  NG3P2  CG334      0.7800  3     0.00 ! 5AU, from bzma, yxu
CG2R62 CG324  NG3P2  HGP2       0.1000  3     0.00 ! 5AU, from bzma, yxu
CG2R63 CG2R62 CG324  NG3P2      1.9000  1   180.00 ! 5AU, (compensation for inner H-
bond N8-H..O6), yxu
CG2R63 CG2R62 CG324  NG3P2      0.2000  2     0.00 ! 5AU, yxu
CG2R62 CG2R62 CG324  NG3P2      0.2000  2     0.00 ! 5AU, from bzma, yxu
HGA2   CG324  NG3P2  CG334      0.1000  3     0.00 ! 5AU, from HGA2 CG324 NG3P2
CG324, yxu
HGA3   CG334  NG3P2  CG324      0.1000  3     0.00 ! 5AU, from HGA2 CG324 NG3P2
CG324, yxu
HGA3   CG334  NG3P2  HGP2       0.1000  3     0.00 ! 5AU, from HGA2 CG324 NG3P2 HGP2,
yxu
CG2R62 CG324  NG3P2  CG324      0.6500  1   180.00 ! 5DU, from 5AU, yxu
CG2R62 CG324  NG3P2  CG324      0.7800  3     0.00 ! 5DU, from 5AU, yxu
OG2D2  CG2O3  CG324  NG3P2      2.9000  2   180.00 ! 5DU, yxu
CG2O3  CG324  NG3P2  CG324      1.0000  1   180.00 ! 5DU, from nmgi, yxu
CG2O3  CG324  NG3P2  CG324      0.7000  2     0.00 ! 5DU, from nmgi, yxu
CG2O3  CG324  NG3P2  HGP2       0.0400  3   180.00 ! 5DU, yxu
CG324  CG2D1  CG2D1  CG331      0.4500  1   180.00 ! IAU, yxu
CG324  CG2D1  CG2D1  CG331      7.0000  2   180.00 ! IAU, yxu
HGA4   CG2D1  CG324  HGA2       0.0000  3     0.00 ! IAU, from HGA4 CG2D1 CG321 HGA2,
yxu
CG2D1  CG2D1  CG324  NG3P2      0.6000  1     0.00 ! IAU, yxu
CG2D1  CG2D1  CG324  NG3P2      0.8200  2     0.00 ! IAU, yxu
CG2D1  CG2D1  CG324  HGA2       0.3000  3   180.00 ! IAU, yxu
HGA4   CG2D1  CG324  NG3P2      0.2000  3     0.00 ! IAU, yxu
CG331  CG2D1  CG331  HGA3       0.1600  3     0.00 ! IAU, from CG321 CG2D1 CG331
HGA3, yxu
CG2D1  CG324  NG3P2  CG324      0.7000  1   180.00 ! IAU, from nmba, yxu
CG2D1  CG324  NG3P2  CG324      0.8000  3     0.00 ! IAU, from nmba, yxu
CG2D1  CG324  NG3P2  HGP2       0.0500  3     0.00 ! IAU, yxu
OG2D2  CG2O3  CG321  CG2R62     0.6000  2   180.00 ! 5CU, from bzac, yxu
OG2D2  CG2O3  CG321  CG2R62     0.1000  4     0.00 ! 5CU, from bzac, yxu
CG321  CG2R62 CG2R62 NG2R61     4.0000  2   180.00 ! 5CU, from CG331 CG2R62 CG2R62
NG2R61, yxu
CG321  CG2R62 CG2R62 HGR62      4.0000  2   180.00 ! 5CU, from CG331 CG2R62 CG2R62
HGR62, yxu
CG321  CG2R62 CG2R63 NG2R61     5.6000  2   180.00 ! 5CU, from CG331 CG2R62 CG2R63
NG2R61, yxu
CG321  CG2R62 CG2R63 OG2D4      1.0000  2   180.00 ! 5CU, from OG2D4 CG2R63 CG2R62
CG331, yxu
CG2R62 CG2R62 CG321  CG2O3      0.1000  4     0.00 ! 5CU, from bzac, yxu
CG2R62 CG2R62 CG321  HGA2       0.0600  4     0.00 ! 5CU, yxu
CG2R63 CG2R62 CG321  CG2O3      0.1500  3     0.00 ! 5CU, (compensation for inner
repulsion O8..O6), yxu
CG2R63 CG2R62 CG321  CG2O3      0.1000  4     0.00 ! 5CU, yxu
CG2R63 CG2R62 CG321  HGA2       0.0600  4     0.00 ! 5CU, yxu
OG2D1  CG2O2  CG321  CG2R62     0.8500  1     0.00 ! OCU, from mebz, yxu
OG302  CG2O2  CG321  CG2R62     0.3000  1     0.00 ! OCU, from mebz, yxu
OG302  CG2O2  CG321  CG2R62     1.0000  2   180.00 ! OCU, from mebz, yxu
CG2R62 CG2R62 CG321  CG2O2      0.1000  2     0.00 ! OCU, from mebz, yxu
CG2R63 CG2R62 CG321  CG2O2      0.1000  2     0.00 ! OCU, from mebz, yxu
NG2S2  CG2O1  CG321  CG2R62     0.2100  1   180.00 ! BCU, from acbz, yxu
NG2S2  CG2O1  CG321  CG2R62     0.5500  3     0.00 ! BCU, from acbz, yxu
OG2D1  CG2O1  CG321  CG2R62     0.6700  2   180.00 ! BCU, from acbz, yxu
```

```
OG2D1  CG2O1  CG321  CG2R62     0.9400  3     0.00 ! BCU, from acbz, yxu
CG2R62 CG2R62 CG321  CG2O1      0.1000  2     0.00 ! BCU, from mebz, yxu
CG2R63 CG2R62 CG321  CG2O1      0.1000  2     0.00 ! BCU, from mebz, yxu
CG311  CG2R62 CG2R62 NG2R61     4.0000  2   180.00 ! CMU, from CG331 CG2R62 CG2R62
NG2R61, yxu
CG311  CG2R62 CG2R62 HGR62      4.0000  2   180.00 ! CMU, from CG331 CG2R62 CG2R62
HGR62, yxu
CG311  CG2R62 CG2R63 NG2R61     5.6000  2   180.00 ! CMU, from CG331 CG2R62 CG2R63
NG2R61, yxu
CG311  CG2R62 CG2R63 OG2D4      0.0000  2   180.00 ! CMU, yxu
CG2R62 CG2R62 CG311  CG2O2      0.2000  2     0.00 ! CMU, from cmbz, yxu
CG2R62 CG2R62 CG311  OG311      0.7000  2   180.00 ! CMU, from cmbz, yxu
CG2R62 CG2R62 CG311  HGA1       0.1000  6   180.00 ! CMU, from CG2R61 CG2R61 CG311
HGA1 yxu
CG2R63 CG2R62 CG311  CG2O2      0.2000  2     0.00 ! CMU, from cmbz, yxu
CG2R63 CG2R62 CG311  OG311      0.8000  1   180.00 ! CMU, (compensation for inner
O8..O6 & H7O..O6), yxu
CG2R63 CG2R62 CG311  HGA1       0.1000  6   180.00 ! CMU, yxu
OG2D1  CG2O2  CG311  OG311      0.5500  2   180.00 ! CMU (hpme), yxu
OG302  CG2O2  CG311  OG311      0.4000  2   180.00 ! CMU (hpme), yxu
OG302  CG2O2  CG311  OG311      0.1000  6     0.00 ! CMU (hpme), yxu
OG2D1  CG2O2  CG311  CG2R62     0.7000  1     0.00 ! CMU, from cmbz, yxu
OG2D1  CG2O2  CG311  CG2R62     0.3000  3   180.00 ! CMU, from cmbz, yxu
OG302  CG2O2  CG311  CG2R62     1.0000  1   180.00 ! CMU, (compensation for inner
O8..O6), yxu
OG302  CG2O2  CG311  CG2R62     0.3000  3     0.00 ! CMU, cmbz, yxu
CG2O2  CG311  OG311  HGP1       0.8500  1   180.00 ! CMU (hpme), yxu
CG2O2  CG311  OG311  HGP1       0.3800  2   180.00 ! CMU (hpme), yxu
CG2O2  CG311  OG311  HGP1       0.3500  3     0.00 ! CMU (hpme), yxu
CG2R62 CG311  OG311  HGP1       1.0800  1     0.00 ! CMU, from bzhe, yxu
CG2R62 CG311  OG311  HGP1       0.5400  2     0.00 ! CMU, from bzhe, yxu
CG2R62 CG311  OG311  HGP1       0.7700  3     0.00 ! CMU, from bzhe, yxu
CG2R63 CG2R62 OG301  CG321      1.8000  1     0.00 ! OAU, from MOU, yxu
CG2R63 CG2R62 OG301  CG321      2.8000  2   180.00 ! OAU, from MOU, yxu
CG2R63 CG2R62 OG301  CG321      1.0000  3     0.00 ! OAU, from MOU, yxu
CG2R62 CG2R62 OG301  CG321      1.5800  2   180.00 ! OAU, from bzmo, yxu
CG2R62 CG2R62 OG301  CG321      0.2000  4   180.00 ! OAU, from bzmo, yxu
OG2D2  CG2O3  CG321  OG301      1.2800  2   180.00 ! OAU, yxu
CG2O3  CG321  OG301  CG2R62     1.3100  1   180.00 ! OAU, from atbz, yxu
HGA2   CG321  OG301  CG2R62     0.0950  3     0.00 ! OAU, from CG2R62 OG301 CG331
HGA3 yxu
OG2D1  CG2O2  CG321  OG301      0.1000  1     0.00 ! OEU, yxu
OG2D1  CG2O2  CG321  OG301      0.9800  2   180.00 ! OEU, yxu
OG302  CG2O2  CG321  OG301      0.1000  1   180.00 ! OEU, yxu
OG302  CG2O2  CG321  OG301      0.9800  2   180.00 ! OEU, yxu
CG2O2  CG321  OG301  CG2R62     0.9100  1   180.00 ! OEU, from oebz, yxu
CG2O2  CG321  OG301  CG2R62     0.5000  2     0.00 ! OEU, from oebz, yxu
OG2D2  CG2O3  CG311  CG2R62     3.1000  2   180.00 ! HCU, from OG2D2 CG2O3 CG311
CG2R61, yxu
OG2D2  CG2O3  CG311  OG311      0.8500  2   180.00 ! HCU, yxu, 17/4,13
CG2R62 CG2R62 CG311  CG2O3      0.2300  2   180.00 ! HCU, from CG2R61 CG2R61 CG311
CG2O3, yxu
CG2R63 CG2R62 CG311  CG2O3      0.2300  2   180.00 ! HCU, from CG2R61 CG2R61 CG311
CG2O3, yxu
CG2O3  CG311  OG311  HGP1       0.3000  1     0.00 ! HCU, yxu, 17/4,13
CG2O3  CG311  OG311  HGP1       0.3000  2   180.00 ! HCU, yxu, 17/4,13
CG2O3  CG311  OG311  HGP1       0.4000  3     0.00 ! HCU, yxu, 17/4,13
CG2R62 CG2R63 NG2R61 CG321      5.5000  2   180.00 ! 3AU, from CG2R62 CG2R62 NG2R61
CG331, yxu
NG2R61 CG2R63 NG2R61 CG321      5.5000  2   180.00 ! 3AU, from NG2R61 CG2R63 NG2R61
CG331, yxu
OG2D4  CG2R63 NG2R61 CG321      0.0000  2   180.00 ! 3AU, from OG2D4 CG2R63 NG2R61
CG331, yxu
```

```
CG314  CG321  CG321  NG2R61     0.5000  1   180.00 ! 3AU, from 3pru, yxu
NG2R61 CG321  CG321  HGA2       0.1000  3     0.00 ! 3AU, yxu
CG321  CG321  NG2R61 CG2R63     0.5000  2     0.00 ! 3AU, yxu
HGA2   CG321  NG2R61 CG2R63     0.0500  6     0.00 ! 3AU, yxu
SG3O1  CG321  CG324  NG3P2      4.8000  1   180.00 ! 5TU, yxu
SG3O1  CG321  CG324  NG3P2      0.4500  2   180.00 ! 5TU, yxu
SG3O1  CG321  CG324  HGA2       0.0500  1     0.00 ! 5TU, yxu
CG324  CG321  SG3O1  OG2P1      0.2200  3     0.00 ! 5TU, yxu
OG2D1  CG2O2  CG321  NG311      0.0000  6   180.00 ! 5UHG, from OG2D1 CG2O2 CG321
NG321, yxu
OG311  CG2O2  CG321  NG311      0.0000  6   180.00 ! 5UHG, from OG311 CG2O2 CG321
NG321, yxu
CG2R62 CG2R62 CG321  NG311      1.0000  2   180.00 ! 5UHG, from ambz, yxu
CG2R63 CG2R62 CG321  NG311      1.0000  2   180.00 ! 5UHG, from ambz, yxu
CG2R62 CG321  NG311  CG321      0.8000  1   180.00 ! 5UHG, from ambz, yxu
CG2R62 CG321  NG311  CG321      0.5000  2     0.00 ! 5UHG, from ambz, yxu
CG2R62 CG321  NG311  CG321      0.7000  3     0.00 ! 5UHG, from ambz, yxu
CG2R62 CG321  NG311  HGPAM1     0.3000  3     0.00 ! 5UHG, from ambz, yxu
CG2O2  CG321  NG311  CG321      2.0000  1     0.00 ! 5UHG, from nmgn, yxu
CG2O2  CG321  NG311  CG321      1.8000  2     0.00 ! 5UHG, from nmgn, yxu
CG2O2  CG321  NG311  CG321      0.5000  3     0.00 ! 5UHG, from nmgn, yxu
CG2O2  CG321  NG311  HGPAM1     0.6000  1   180.00 ! 5UHG, yxu
CG2O2  CG321  NG311  HGPAM1     0.3000  2     0.00 ! 5UHG, yxu
CG2O2  CG321  NG311  HGPAM1     0.5000  3     0.00 ! 5UHG, from nmgn, yxu
CG2R62 CG321  NG311  CG331      0.8000  1   180.00 ! 5UNM, from ambz, yxu
CG2R62 CG321  NG311  CG331      0.5000  2     0.00 ! 5UNM, from ambz, yxu
CG2R62 CG321  NG311  CG331      0.7000  3     0.00 ! 5UNM, from ambz, yxu
HGA3   CG331  NG311  CG321      0.5000  3     0.00 ! 5UNM, from HGA3 CG331 NG311
CG2N1, yxu
OG2D1  CG2O2  CG311  NG321      0.0000  6   180.00 ! 5UHA, from OG2D1  CG2O2  CG321
NG321, yxu
OG311  CG2O2  CG311  NG321      0.0000  6   180.00 ! 5UHA, from OG311  CG2O2  CG321
NG321, yxu
NG321  CG311  CG321  CG321      0.1950  3     0.00 ! 5UHA, from NG3P2 CG314 CG321
CG321, cgenff_xyu, yxu
NG321  CG311  CG321  HGA2       0.1600  3     0.00 ! 5UHA, from NG311 CG321 CG331
HGA3, yxu
CG2O2  CG311  NG321  HGPAM2     0.1600  3     0.00 ! 5UHA, from CG2O2 CG321 NG321
HGPAM2, yxu
CG321  CG311  NG321  HGPAM2     0.4000  3     0.00 ! 5UHA, from aboh, yxu
HGA1   CG311  NG321  HGPAM2     0.0100  3     0.00 ! 5UHA, from HGA2   CG321  NG321
HGPAM2, cgenff_xyu, yxu
CG311  CG321  CG321  NG2R61     0.2000  3     0.00 ! 5UHA, from CG321  CG321  CG321
NG2S1, yxu
CG2R62 CG2R62 CG321  NG321      1.0000  2   180.00 ! 5UNA, from ambz, yxu
CG2R63 CG2R62 CG321  NG321      1.0000  2   180.00 ! 5UNA, from ambz, yxu
CG2R62 CG321  NG321  HGPAM2     0.3000  3     0.00 ! 5UNA, from ambz, yxu
OG311  CG2O2  CG321  CG2R62     0.9300  2   180.00 ! 5UHC, from bzaa, yxu
OG311  CG2O2  CG321  CG2R62     0.1200  3   180.00 ! 5UHC, from bzaa, yxu
OG311  CG2O2  CG311  CG2R62     0.9300  2   180.00 ! 5UHC, from bzaa, yxu
OG311  CG2O2  CG311  CG2R62     0.1200  3   180.00 ! 5UHC, from bzaa, yxu
OG311  CG2O2  CG321  OG301      0.1700  1   180.00 ! 5UHC (moac), yxu
OG311  CG2O2  CG321  OG301      0.4600  2   180.00 ! 5UHC (moac), yxu
OG311  CG2O2  CG311  OG311      0.4600  2   180.00 ! 5UHC, from moac, yxu
CG2D1  CG321  NG311  CG321      1.1000  1   180.00 ! 5UNI, from penm, yxu
CG2D1  CG321  NG311  CG321      0.6000  2   180.00 ! 5UNI, from penm, yxu
CG2D1  CG321  NG311  CG321      0.5000  3     0.00 ! 5UNI, from penm, yxu
CG2R62 CG2R62 CG321  CG1N1      0.3900  2   180.00 ! CYU, from cybz, yxu
CG2R62 CG2R62 CG321  CG1N1      0.0400  4     0.00 ! CYU, from cybz, yxu
CG2R63 CG2R62 CG321  CG1N1      0.3900  2   180.00 ! CYU, from cybz, yxu
CG2R63 CG2R62 CG321  CG1N1      0.0400  4     0.00 ! CYU, from cybz, yxu
CG324  CG2R62 CG2R63 NG2R62     4.5000  2   180.00 ! SAU, yxu
SG311  CG2R64 NG2R61 CG2R62     6.0000  2   180.00 ! GAU, from  GAU, yxu
```

```
SG311  CG2R64 NG2R62 CG2R63    10.0000  2   180.00 ! GAU, from SMA, yxu
CG321  CG2R62 CG2R63 NG2R62     4.5000  2   180.00 ! GAU, from NG2R62 CG2R63 CG2R62
CG324, yxu
HGR62  CG2R62 CG2R63 NG2R62     1.0000  2   180.00 ! GAU, yxu
CG2D1  CG321  SG311  CG2R64     1.3000  1   180.00 ! GAU, yxu
CG2D1  CG321  SG311  CG2R64     1.0000  2     0.00 ! GAU, yxu
CG2D1  CG321  SG311  CG2R64     0.2000  3     0.00 ! GAU, yxu
HGA2   CG321  SG311  CG2R64     0.2000  3     0.00 ! GAU, yxu
CG2D1  CG2D1  CG321  SG311      0.7000  1   180.00 ! GAU, from pesu
CG2D1  CG2D1  CG321  SG311      1.4000  3   180.00 ! GAU, from pesu
HGA4   CG2D1  CG321  SG311      0.2000  3     0.00 ! GAU, yxu
CG2D1  CG321  CG321  CG2D1      0.9000  1   180.00 ! GAU, (15he), yxu
CG2D1  CG321  CG321  CG2D1      0.3000  3   180.00 ! GAU, (15he), yxu

!***Cytosines***
CG2R64 CG2R61 CG2R61 NG2R61     6.0000  2   180.00 ! 2SC, from NA36
CG2R61 CG2R61 CG2R64 NG2S3      2.0000  2   180.00 ! yxu KEVO: exocyclic N in 2SC
planar, issue with CGenFF atom types?
HGR62  CG2R61 CG2R64 NG2R62     3.4000  2   180.00 ! 2SC, from NA36
HGR62  CG2R61 CG2R64 NG2S3      2.0000  2   180.00 ! 2SC, from NA36
NG2R62 CG2R63 NG2R61 CG2R61     0.6000  2   180.00 ! 2SC, from NA36
CG2R61 CG2R64 NG2R62 CG2R63     6.0000  2   180.00 ! 2SC, from NA36
CG2R61 CG2R64 NG2S3  HGP4       2.0000  2   180.00 ! yxu KEVO: exocyclic N in 2SC
planar, issue with CGenFF atom types?
NG2R62 CG2R63 NG2R61 HGP1       3.6000  2   180.00 ! 2SC, from NG2R62 CG2R64 NG2R61
HGP1, NA36
SG2D1  CG2R63 NG2R62 CG2R64     1.6000  2   180.00 ! 2SC, from OG2D4 CG2R63 NG2R62
CG2R64, yxu
CG331  CG2R62 CG2R64 NG2R62     1.0000  2   180.00 ! 5MC, from HMC, yxu
CG331  CG2R62 CG2R64 NG2P1      1.0000  2   180.00 ! 5MCp, from HMC, yxu
CG331  CG2R62 CG2R64 NG2S3      2.4000  2   180.00 ! 5MC, from CG331 CG2R61 CG2R61
NG2S1, yxu
CG2R64 CG2R62 CG331  HGA3       0.4600  3     0.00 ! 5MC, from CG2R63 CG2R62 CG331
HGA3, yxu
CG321  CG2R62 CG2R64 NG2R62     1.0000  2   180.00 ! HMC, yxu
CG321  CG2R62 CG2R64 NG2S3      2.4000  2   180.00 ! HMC, yxu
CG2R62 CG321  OG311  HGP1       2.1000  1     0.00 ! HMC, from CG2R61 CG321 OG311
HGP1, yxu
CG2R62 CG321  OG311  HGP1       1.4000  2     0.00 ! HMC, from CG2R61 CG321 OG311
HGP1, yxu
CG2R62 CG321  OG311  HGP1       1.1000  3     0.00 ! HMC, from CG2R61 CG321 OG311
HGP1, yxu
CG2R62 CG2R62 CG321  OG311      0.0000  2     0.00 ! HMC, yxu
CG2R64 CG2R62 CG321  OG311      1.0000  1   180.00 ! HMC, yxu
CG2R64 CG2R62 CG321  OG311      1.0000  2     0.00 ! HMC, yxu
CG2R64 CG2R62 CG321  HGA2       0.8100  3     0.00 ! HMC, yxu
CG2O4  CG2R62 CG2R62 NG2R61     2.5000  2   180.00 ! 5FC, yxu
CG2O4  CG2R62 CG2R62 HGR62      2.8000  2   180.00 ! 5FC, yxu
CG2O4  CG2R62 CG2R64 NG2R62     3.6000  2   180.00 ! 5FC, yxu
CG2O4  CG2R62 CG2R64 NG2S3      1.5000  2   180.00 ! 5FC, yxu
OG2D1  CG2O4  CG2R62 CG2R62     1.0800  2   180.00 ! 5FC, yxu
HGR52  CG2O4  CG2R62 CG2R62     1.0800  2   180.00 ! 5FC, yxu
OG2D1  CG2O4  CG2R62 CG2R64     2.6000  1   180.00 ! 5FC, yxu
OG2D1  CG2O4  CG2R62 CG2R64     1.8000  2   180.00 ! 5FC, yxu
HGR52  CG2O4  CG2R62 CG2R64     1.5000  2   180.00 ! 5FC, yxu
CG2R62 CG2R62 CG2R64 NG311      3.1000  2   180.00 ! 4MC, yxu
HGR62  CG2R62 CG2R64 NG311      0.0000  2   180.00 ! 4MC, yxu
NG311  CG2R64 NG2R62 CG2R63     3.1000  2   180.00 ! 4MC, yxu
CG2R62 CG2R64 NG311  CG331      1.4000  1     0.00 ! 4MC, yxu
CG2R62 CG2R64 NG311  CG331      2.7000  2   180.00 ! 4MC, yxu, 1/2015
CG2R62 CG2R64 NG311  HGPAM1     3.0000  2   180.00 ! 4MC, yxu
NG2R62 CG2R64 NG311  CG331      1.4000  1   180.00 ! 4MC, yxu
NG2R62 CG2R64 NG311  CG331      1.3000  2   180.00 ! 4MC, yxu, 1/2015
```

```
NG2R62 CG2R64 NG311  HGPAM1     2.4000  2   180.00 ! 4MC, yxu
HGA3   CG331  NG311  CG2R64     0.4000  3     0.00 ! 4MC, yxu     0.24
HGA3   CG331  NG311  CG2R64     0.0800  6     0.00 ! 4MC, yxu     0.12
CG2R62 CG2R62 CG2R64 NG2S1      3.1000  2   180.00 ! 4AC, from CG2R61 CG2R61 CG2R64
NG2S1, yxu
HGR62  CG2R62 CG2R64 NG2S1      2.4000  2   180.00 ! 4AC, from HGR61 CG2R61 CG2R64
NG2S1, yxu
NG2S1  CG2R64 NG2R62 CG2R63     2.0000  2   180.00 ! 4AC, from NG2S3 CG2R64 NG2R62
CG2R63, yxu
CG2R62 CG2R64 NG2S1  CG2O1      0.8000  1     0.00 ! 4AC, lower to enhance trans, yxu
CG2R62 CG2R64 NG2S1  CG2O1      2.2000  2   180.00 ! 4AC, yxu
CG2R62 CG2R64 NG2S1  CG2O1      0.4000  3     0.00 ! 4AC, yxu
CG2R62 CG2R64 NG2S1  HGP1       0.5000  2   180.00 ! 4AC, yxu
NG2R62 CG2R64 NG2S1  CG2O1      0.5000  1   180.00 ! 4AC, lower to enhance trans, yxu
NG2R62 CG2R64 NG2S1  CG2O1      1.8000  2   180.00 ! 4AC, yxu
NG2R62 CG2R64 NG2S1  CG2O1      0.4000  3   180.00 ! 4AC, yxu
NG2R62 CG2R64 NG2S1  HGP1       0.5000  2   180.00 ! 4AC, yxu
NG2R61 CG2R63 NG2R61 CG2R64     3.0000  2   180.00 ! 3MCn, from NG2R61 CG2R63 NG2R61
CG2R63, yxu
CG2R62 CG2R62 CG2R64 NG2R61     7.0000  2   180.00 ! 3MCn, from CG2R62 CG2R62 CG2R62
NG2R61, yxu
HGR62  CG2R62 CG2R64 NG2R61     3.4000  2   180.00 ! 3MCn, from NG2R61 CG2R62 CG2R62
HGR62, yxu
CG2R62 CG2R62 CG2R64 NG2D1      3.0000  2   180.00 ! 3MCn, yxu
HGR62  CG2R62 CG2R64 NG2D1      1.0000  2   180.00 ! 3MCn, yxu
CG2R62 CG2R64 NG2R61 CG2R63     0.6000  2   180.00 ! 3MCn, from CG2R62 CG2R62 NG2R61
CG2R63, yxu
CG2R62 CG2R64 NG2R61 CG331      0.0000  2   180.00 ! 3MCn, yxu
NG2D1  CG2R64 NG2R61 CG2R63     3.0000  2   180.00 ! 3MCn, yxu
NG2D1  CG2R64 NG2R61 CG331      0.0000  2   180.00 ! 3MCn, yxu
CG2R62 CG2R64 NG2D1  HGP1       6.8500  2   180.00 ! 3MCn, yxu
NG2R61 CG2R64 NG2D1  HGP1       1.6000  1     0.00 ! 3MCn, yxu
NG2R61 CG2R64 NG2D1  HGP1       6.8500  2   180.00 ! 3MCn, yxu
HGA3   CG331  NG2R61 CG2R64     0.1900  3     0.00 ! 3MCn, from HGA3 CG331 NG2R61
CG2R63, yxu
HGR62  CG2R62 CG2R64 NG2P1      1.0000  2   180.00 ! 3MC, yxu
CG2R62 CG2R64 NG2P1  CG334      3.3500  2   180.00 ! 3MC, yxu
NG2R61 CG2R63 NG2P1  CG334      3.6500  2   180.00 ! 3MC, yxu
NG2P1  CG2R64 NG2P1  CG334      0.6000  2   180.00 ! 3MC, yxu
NG2P1  CG2R64 NG2P1  CG2R63     2.3000  2   180.00 ! 3MC, yxu
OG2D4  CG2R63 NG2P1  CG2R64     2.6000  2   180.00 ! 3MC, yxu
OG2D4  CG2R63 NG2P1  CG334      1.7000  2   180.00 ! 3MC, yxu
NG2P1  CG2R63 NG2R61 CG2R62     0.6000  2   180.00 ! 3MC, yxu
NG2R61 CG2R63 NG2P1  CG2R64     0.6000  2   180.00 ! 3MC, yxu
CG2R62 CG2R64 NG2P1  CG2R63     0.6000  2   180.00 ! 3MC, yxu
CG2R62 CG2R62 CG2R64 NG2P1      0.6000  2   180.00 ! 3MC, yxu
CG2R62 CG2R64 NG2P1  HGP2       2.4000  2   180.00 ! 3MC, yxu
CG2R62 CG2R64 NG2P1  HGP2       0.7000  3   180.00 ! 3MC, yxu
NG2P1  CG2R64 NG2P1  HGP2       2.4000  2   180.00 ! 3MC, yxu
NG2P1  CG2R63 NG2R61 HGP1       0.0000  2   180.00 ! 3MC, from OG2D4 CG2R63 NG2R61
HGP1, yxu
HGA3   CG334  NG2P1  CG2R63     0.0000  6   180.00 ! 3MC, from HGA3 CG334 NG2P1
CG2N1, yxu
HGA3   CG334  NG2P1  CG2R64     0.0000  6   180.00 ! 3MC, from HGA3 CG334 NG2P1
CG2N1, yxu
NG2P1  CG2R64 NG2P1  CG2R61     0.4000  2   180.00 ! K2C, yxu, 16/7,13
NG2P1  CG2R64 NG2P1  CG2R64     1.5000  2   180.00 ! K2C, yxu, 16/7,13
CG2R61 CG2R64 NG2P1  CG2R64     1.3000  2   180.00 ! K2C, yxu, 16/7,13
CG2R61 CG2R61 CG2R64 NG2P1      2.8000  2   180.00 ! K2C, yxu, 16/7,13
CG2R64 CG2R61 CG2R61 NG2P1      3.1000  2   180.00 ! K2C, yxu, 16/7,13
CG2R61 CG2R61 NG2P1  CG2R64     0.6000  2   180.00 ! K2C, yxu, 16/7,13
NG2P1  CG2R61 CG2R61 HGR61      3.4000  2   180.00 ! K2C, yxu, 16/7,13
HGR61  CG2R61 CG2R64 NG2P1      2.6000  2   180.00 ! K2C, yxu, 16/7,13
```

```
HGR62  CG2R61 NG2P1  CG2R64     4.0000  2   180.00 ! K2C, yxu
NG2P1  CG2R64 NG2D1  HGP1       1.6000  1     0.00 ! K2C, from 3MCn, yxu
NG2P1  CG2R64 NG2D1  HGP1       6.8500  2   180.00 ! K2C, from 3MCn, yxu
CG2R61 CG2R64 NG2P1  HGP2       0.1900  2   180.00 ! K2C, yxu, 16/7,13
NG2D1  CG2R64 NG2P1  HGP2       0.5000  2   180.00 ! K2C, yxu, 16/7,13
NG2D1  CG2R64 NG2P1  CG2R64     1.0000  2   180.00 ! K2C, yxu, 16/7,13
NG2P1  CG2R64 NG2P1  CG324      1.4600  2   180.00 ! K2C, yxu
CG321  CG324  NG2P1  CG2R64     0.1000  3     0.00 ! K2C, yxu
HGA2   CG324  NG2P1  CG2R64     0.0000  3     0.00 ! K2C, yxu
HGR61  CG2R61 CG2R64 NG2R62     3.4000  2   180.00 ! K2Cn, from NA36, yxu
CG2R61 CG2R61 NG2R61 CG2R64     0.6000  2   180.00 ! K2Cn, from CG2R62 CG2R62 NG2R61
CG2R63, yxu
HGR62  CG2R61 NG2R61 CG2R64     4.6000  2   180.00 ! K2Cn, from HGR62 CG2R62 NG2R61
CG2R63, yxu
NG2R62 CG2R64 NG2R61 CG2R61     0.2000  2   180.00 ! K2Cn, from NG2R62 CG2R64 NG2R61
CG2R63, yxu
NG2R61 CG2R64 NG2R62 CG2R64     2.0000  2   180.00 ! K2Cn, from NG2R61 CG2R64 NG2R62
CG2RC0, yxu
CG2R61 CG2R61 CG2R64 NG2D1      3.0000  2   180.00 ! K2Cn, from 3MCn, yxu
HGR61  CG2R61 CG2R64 NG2D1      1.0000  2   180.00 ! K2Cn, from 3MCn, yxu
CG2R61 CG2R64 NG2D1  HGP1       6.8500  2   180.00 ! K2Cn, from 3MCn, yxu
NG2R62 CG2R64 NG2D1  HGP1       1.6000  1     0.00 ! K2Cn, from 3MCn, yxu
NG2R62 CG2R64 NG2D1  HGP1       6.8500  2   180.00 ! K2Cn, from 3MCn, yxu
NG2D1  CG2R64 NG2R62 CG2R64     3.0000  2   180.00 ! K2Cn, from 3MCn, yxu
NG311  CG2R64 NG2R61 CG2R61     4.0000  2   180.00 ! K2Cn, from NG2S3 CG2R64 NG2R61
CG2R63, yxu
NG311  CG2R64 NG2R62 CG2R64     3.1000  2   180.00 ! K2Cn, from CG331 CG2R61 NG2R60
CG2R64, yxu
NG2R61 CG2R64 NG311  CG321      2.8000  2   180.00 ! K2Cn, yxu
NG2R62 CG2R64 NG311  CG321      2.4000  1   180.00 ! K2Cn, yxu
NG2R62 CG2R64 NG311  CG321      3.0000  2   180.00 ! K2Cn, yxu
CG321  CG321  NG311  CG2R64     0.8000  1   180.00 ! K2Cn, yxu
CG321  CG321  NG311  CG2R64     1.1000  3     0.00 ! K2Cn, yxu
HGA2   CG321  NG311  CG2R64     0.0500  3   180.00 ! K2Cn, from HGA2 CG321 NG311
HGP1, yxu
CG321  CG321  CG321  NG311      0.3000  3     0.00 ! K2Cn, from prnc, yxu
CG324  CG321  CG321  CG324      0.1950  3     0.00 ! R2C, from CG36, yxu
NG2R61 CG2R63 NG2P1  HGP2       2.4500  2   180.00 ! CYTp, yxu
OG2D4  CG2R63 NG2P1  HGP2       1.6000  2   180.00 ! CYTp, yxu
CG2R62 CG2R62 NG2R61 CG2R64     0.8000  2   180.00 ! 1PC, yxu
HGR62  CG2R62 NG2R61 CG2R64     4.8000  2   180.00 ! 1PC, from HGR62 CG2R62 NG2R61
CG2R63, yxu
NG2R62 CG2R64 NG2R61 CG2R62     0.6000  2   180.00 ! 1PC, yxu
NG2S3  CG2R64 NG2R61 CG2R62     4.0000  2   180.00 ! 1PC, from NG2S3 CG2R64 NG2R61
CG2R63, yxu
NG2R61 CG2R64 NG2R62 CG2R63     3.2000  2   180.00 ! 1PC, yxu
CG2R62 CG2R62 CG2R63 NG2R62     0.8000  2   180.00 ! 1PC, yxu
CG331  CG2R62 CG2R63 NG2R62     9.0000  2   180.00 ! 1PC, yxu
CG2R62 CG2R63 NG2R62 CG2R64     0.4000  2   180.00 ! 1PC, yxu
NG2S3  CG2R64 NG2R62 CG2R62     1.0000  2   180.00 ! 3PC, yxu
CG331  CG2R62 CG2R62 NG2R62     9.0000  2   180.00 ! 3PC, yxu

!***Adenine***
NG2R62 CG2R64 NG2P1  HGP2       1.9000  2   180.00 ! ADEp, yxu
HGR62  CG2R64 NG2P1  HGP2       0.5500  2   180.00 ! ADEp, yxu
NG2R62 CG2R64 CG331  HGA3       0.0030  6   180.00 ! 2MA, from NG2R60 CG2R61 CG331
HGA3, yxu
CG331  CG2R64 NG2R62 CG2R64     5.5000  2   180.00 ! 2MA, yxu
CG331  CG2R64 NG2R62 CG2RC0    11.0000  2   180.00 ! 2MA, yxu
NG2R62 CG2R64 NG2R61 CG331      5.5000  2   180.00 ! 1MI, yxu
CG2RC0 CG2R63 NG2R61 CG331      5.5000  2   180.00 ! 1MI, yxu
HGR62  CG2R64 NG2R61 CG331      0.0000  2   180.00 ! 1MI, yxu
SG311  CG2R64 NG2R62 CG2R64    10.0000  2   180.00 ! SMA, yxu
```

```
SG311  CG2R64 NG2R62 CG2RC0    10.0000  2   180.00 ! SMA, yxu
SG311  CG331  HGA3   CG2R64     0.3300  3     0.00 ! SMA, yxu
CG2RC0 CG2R64 NG311  CG331      0.2000  1     0.00 ! 6MA , yxu
CG2RC0 CG2R64 NG311  CG331      1.4000  2   180.00 ! 6MA , yxu, 1/2015
CG2RC0 CG2R64 NG311  HGPAM1     2.8000  2   180.00 ! 6MA , yxu
NG311  CG2R64 CG2RC0 CG2RC0     3.1000  2   180.00 ! 6MA, from 4MC, yxu
NG311  CG2R64 CG2RC0 NG2R50     0.0000  2   180.00 ! 6MA, from NG2S3 CG2R64 CG2RC0
NG2R50, yxu
NG2S1  CG2R64 CG2RC0 CG2RC0     3.1000  2   180.00 ! 6AA, from 4AC, yxu
NG2S1  CG2R64 NG2R62 CG2R64     2.0000  2   180.00 ! 6AA, from 4AC, yxu
NG2S1  CG2R64 CG2RC0 NG2R50     0.0000  2   180.00 ! 6AA, from NG2S3 CG2R64 CG2RC0
NG2R50, yxu
CG2RC0 CG2R64 NG2S1  CG2O1      1.8000  1     0.00 ! 6AA, from CG2R62 CG2R64 NG2S1
CG2O1, without the scale of elec, yxu
CG2RC0 CG2R64 NG2S1  CG2O1      1.5000  2   180.00 ! 6AA, yxu
CG2RC0 CG2R64 NG2S1  HGP1       0.5000  2   180.00 ! 6AA, yxu
NG2D1  CG2R64 CG2RC0 CG2RC0     3.0000  2   180.00 ! 1MAn, from 3MCn, yxu
NG2D1  CG2R64 CG2RC0 NG2R50     0.0000  2   180.00 ! 1MAn, from NG2S3 CG2R64 CG2RC0
NG2R50, yxu
NG2D1  CG2R64 NG2R61 CG2R64     3.0000  2   180.00 ! 1MAn, from 3MCn, yxu
NG2R61 CG2R64 CG2RC0 CG2RC0     1.8000  2   180.00 ! 1MAn, from NA36
NG2R61 CG2R64 CG2RC0 NG2R50     2.0000  2   180.00 ! 1MAn, from NA36
CG2RC0 CG2R64 NG2D1  HGP1       6.8500  2   180.00 ! 1MAn, from 3MCn, yxu
CG2RC0 CG2R64 NG2R61 CG2R64     0.6000  2   180.00 ! 1MAn, from 3MCn, yxu
CG2RC0 CG2R64 NG2R61 CG331      0.0000  2   180.00 ! 1MAn, from 3MCn, yxu
NG2R62 CG2R64 NG2R61 CG2R64     0.2000  2   180.00 ! 1MAn, from NG2R62 CG2R64 NG2R61
CG2R63, yxu
HGR62  CG2R64 NG2R61 CG2R64     4.6000  2   180.00 ! 1MAn, from HGR62 CG2R62 NG2R61
CG2R63, yxu
NG2P1  CG2R64 CG2RC0 NG2R50     2.0000  2   180.00 ! 1MA, from 1MAn, yxu
NG2P1  CG2R64 CG2RC0 CG2RC0     1.8000  2   180.00 ! 1MA, from 1MAn, yxu
CG2RC0 CG2R64 NG2P1  CG2R64     1.8000  2   180.00 ! 1MA, from NA36, yxu
NG2R62 CG2R64 NG2P1  CG2R64     1.8000  2   180.00 ! 1MA, from NA36, yxu
NG2P1  CG2R64 NG2R62 CG2RC0     2.0000  2   180.00 ! 1MA, from NA36, yxu
CG2RC0 CG2R64 NG2P1  CG334      5.5000  2   180.00 ! 1MA, from 1MI, yxu
HGR62  CG2R64 NG2P1  CG334      0.0000  2   180.00 ! 1MA, from 1MI, yxu
HGR62  CG2R64 NG2P1  CG2R64     4.9000  2   180.00 ! 1MA, from 1MI, yxu
NG2R62 CG2R64 NG2P1  CG334      5.5000  2   180.00 ! 1MA, from 1MI, yxu
CG2RC0 CG2R64 NG2P1  HGP2       2.4000  2   180.00 ! 1MA, from 3MC, yxu
CG2RC0 CG2R64 NG2P1  HGP2       0.7000  3   180.00 ! 1MA, from 3MC, yxu
CG2RC0 CG2R64 NG311  CG321      2.0000  2   180.00 ! 6IA, from bepa, yxu
CG2D1  CG2D1  CG321  NG311      1.6000  1   180.00 ! 6IA, yxu
CG2D1  CG2D1  CG321  NG311      0.3000  3     0.00 ! 6IA, yxu
HGA4   CG2D1  CG321  NG311      0.2600  3     0.00 ! 6IA, yxu
CG2D1  CG321  NG311  CG2R64     2.0000  1   180.00 ! 6IA, yxu
CG2D1  CG321  NG311  CG2R64     0.3000  3   180.00 ! 6IA, yxu
CG2D1  CG321  NG311  HGPAM1     0.4800  3     0.00 ! 6IA, yxu
CG2D1  CG2D1  CG321  OG311      1.9000  1   180.00 ! HIA (hbt), yxu
CG2D1  CG2D1  CG321  OG311      0.4000  2   180.00 ! HIA, yxu
CG2D1  CG2D1  CG321  OG311      0.6000  3   180.00 ! HIA, yxu
CG331  CG2D1  CG321  OG311      0.2500  1   180.00 ! HIA, yxu
CG331  CG2D1  CG321  OG311      0.5200  2   180.00 ! HIA, yxu
CG331  CG2D1  CG321  OG311      0.2300  3   180.00 ! HIA, yxu
NG2S1  CG2O6  NG2S1  CG2R64     1.6000  2   180.00 ! HNA, from mpyu, yxu
NG2S1  CG2O6  NG2S1  CG2R64     0.2800  4     0.00 ! HNA, from mpyu, yxu
OG2D1  CG2O6  NG2S1  CG2R64     2.0000  2   180.00 ! HNA, from mpyu, yxu
NG2S1  CG2O6  NG2S1  HGP1       2.0000  2   180.00 ! HNA, from mpyu, cgenff_xyu, yxu
CG2RC0 CG2R64 NG2S1  CG2O6      1.5000  1     0.00 ! HNA, from pymu, yxu
CG2RC0 CG2R64 NG2S1  CG2O6      1.9000  2   180.00 ! HNA, from pymu, yxu
CG2RC0 CG2R64 NG2S1  CG2O6      0.7200  3     0.00 ! HNA, from pymu, yxu
NG2R62 CG2R64 NG2S1  CG2O6      2.6000  2   180.00 ! HNA, from pymu, yxu
NG2S1  CG2O6  NG2S1  CG311      0.8000  1     0.00 ! HNA, from 12mu, yxu
```

```
OG2D1  CG2O6  NG2S1  CG311      4.0000  2   180.00 ! HNA, from OG2D1 CG2O6 NG2S1
CG321, yxu
OG2D1  CG2O6  NG2S1  CG311      0.9500  4     0.00 ! HNA, from OG2D1 CG2O6 NG2S1
CG321, yxu
CG2O3  CG311  NG2S1  CG2O6      0.2000  1   180.00 ! HNA, from CG2O3 CG311 NG2S1
CG2O1, yxu
CG311  CG311  NG2S1  CG2O6      1.8000  1     0.00 ! HNA, from CG311 CG311 NG2S1
CG2O1, yxu
HGA1   CG311  NG2S1  CG2O6      0.0000  1     0.00 ! HNA, from HGA1 CG311 NG2S1
CG2O1, yxu
HGA3   CG331  NG2S0  CG2R64     0.0000  3     0.00 ! 66A, from HGA3 CG331 NG2S0
CG2O1, yxu
NG2S0  CG2R64 CG2RC0 CG2RC0     4.0000  2   180.00 ! 66A, yxu
NG2S0  CG2R64 CG2RC0 NG2R50     4.6000  2   180.00 ! 66A, yxu
NG2S0  CG2R64 NG2R62 CG2R64     4.8000  2   180.00 ! 66A, yxu
CG2RC0 CG2R64 NG2S0  CG2O6      1.0000  1     0.00 ! 66A, from m6pa, yxu
CG2RC0 CG2R64 NG2S0  CG2O6      2.2000  2   180.00 ! 66A, from m6pa, yxu
CG2RC0 CG2R64 NG2S0  CG2O6      0.2000  3     0.00 ! 66A, from m6pa, yxu
CG2RC0 CG2R64 NG2S0  CG331      2.2000  2   180.00 ! 66A, yxu
NG2R62 CG2R64 NG2S0  CG2O6      2.6000  2   180.00 ! 66A, from m6pa, yxu
NG2R62 CG2R64 NG2S0  CG331      2.6000  2   180.00 ! 66A, yxu
NG2S1  CG2O6  NG2S0  CG2R64     1.3000  2   180.00 ! 66A, from pmmu, yxu
OG2D1  CG2O6  NG2S0  CG2R64     1.3000  2   180.00 ! 66A, from pmmu, yxu
NG2S0  CG2O6  NG2S1  CG311      1.2000  1   180.00 ! 66A, from mmmu, yxu
NG2S0  CG2O6  NG2S1  CG311      1.5000  2   180.00 ! 66A, from mmmu, yxu
CG2O3  CG321  NG2S1  CG2O6      0.2000  1   180.00 ! 6GA, from CG2O3 CG321 NG2S1
CG2O1, yxu
CG2O2  CG321  NG2S1  CG2O6      0.2000  1   180.00 ! 6GA, from CG2O3 CG321 NG2S1
CG2O1, yxu
NG2S1  CG2O6  NG2S1  CG321      0.8000  1     0.00 ! 6GA, from 12mu, yxu
NG2R50 CG2R53 CG331  HGA3       0.1900  3     0.00 ! 8MA, from NG2R50 CG2R51 CG321
HGA2, yxu
NG2R51 CG2R53 CG331  HGA3       0.1900  3     0.00 ! 8MA, from NG2R51 CG2R51 CG331
HGA3, yxu
CG331  CG2R53 NG2R50 CG2RC0     5.5000  2   180.00 ! 8MA, yxu
CG331  CG2R53 NG2R51 CG2RC0     5.5000  2   180.00 ! 8MA, yxu
CG331  CG2R53 NG2R51 CG331      3.0000  2   180.00 ! 8MA, yxu
CG2O2  CG311  CG311  CG331      0.2000  3     0.00 ! 6AH, from CG2O1  CG311  CG311
CG331, yxu
OG311  CG2O2  CG311  NG2S1      0.0000  1     0.00 ! 6AH, from OG302  CG2O2  CG311
NG2S1, yxu
OG2D1  CG2O2  CG321  NG2S1      0.0000  1     0.00 ! 6AH, from OG2D1  CG2O2  CG311
NG2S1, yxu
OG311  CG2O2  CG321  NG2S1      0.0000  1     0.00 ! 6AH, from OG302  CG2O2  CG311
NG2S1, yxu
CG2RC0 CG2R64 NG2R61 HGP1       0.0000  2   180.00 ! IMIA
NG2D1  CG2R64 NG2R61 HGP1       0.0000  2   180.00 ! IMIA

!***Guanine***
NG2S3  CG2R64 NG2R61 CG331      2.5000  2   180.00 ! 1MG, yxu
NG2R52 CG2R53 NG2R52 CG2RC0    14.0000  2   180.00 ! 7MG, NA36
HGR53  CG2R53 NG2R52 CG2RC0     2.7000  2   180.00 ! 7MG, yxu
NG2R61 CG2R63 CG2RC0 NG2R52     2.0000  2   180.00 ! 7MG, from NG2R61 CG2R63 CG2RC0
NG2R50, yxu
OG2D4  CG2R63 CG2RC0 NG2R52     0.0000  2   180.00 ! 7MG, from OG2D4 CG2R63 CG2RC0
NG2R50, yxu
CG2R63 CG2RC0 CG2RC0 NG2R52     5.0000  2   180.00 ! 7MG, yxu
NG2R52 CG2RC0 CG2RC0 NG2R52     5.0000  2   180.00 ! 7MG, yxu
NG2R52 CG2RC0 CG2RC0 NG2R62     5.5000  2   180.00 ! 7MG, yxu
CG2R63 CG2RC0 NG2R52 CG2R53     2.0000  2   180.00 ! 7MG, from CG2R63 CG2RC0 NG2R50
CG2R53, yxu
CG2RC0 CG2RC0 NG2R52 CG2R53     3.0000  2   180.00 ! 7MG, yxu
```

```
NG2R62 CG2RC0 NG2R52 CG2R53     2.0000  2   180.00 ! 7MG, from NG2R62 CG2RC0 NG2R50
CG2R53, yxu
NG2R52 CG2RC0 NG2R62 CG2R64     2.0000  2   180.00 ! 7MG, from NG2R51 CG2RC0 NG2R62
CG2R64, yxu
NG2R52 CG2R53 NG2R52 CG334      3.7000  2   180.00 ! 7MG, yxu
HGR53  CG2R53 NG2R52 CG334      1.0000  2   180.00 ! 7MG, yxu
CG2R63 CG2RC0 NG2R52 CG334      3.0000  2   180.00 ! 7MG, yxu
CG2RC0 CG2RC0 NG2R52 CG334      5.5000  2   180.00 ! 7MG, yxu
HGA3   CG334  NG2R52 CG2R53     0.1500  3   180.00 ! 7MG, from HGA3 CG334 NG2P1
CG2D1, yxu
HGA3   CG334  NG2R52 CG2RC0     0.0000  6   180.00 ! 7MG, from HGA3 CG334 NG2P1
CG2N1, yxu
NG311  CG2R64 NG2R61 CG2R63     3.1000  2   180.00 ! 2MG, from 4MC, yxu
NG311  CG2R64 NG2R62 CG2RC0     3.1000  2   180.00 ! 2MG, from 4MC, yxu
NG311  CG2R64 NG2R61 HGP1       0.5000  2   180.00 ! 2MG, yxu
NG2R61 CG2R64 NG311  CG331      1.2000  1     0.00 ! 2MG, yxu
NG2R61 CG2R64 NG311  CG331      1.3000  2   180.00 ! 2MG, yxu, 1/2015
NG2R61 CG2R64 NG311  HGPAM1     2.0000  2   180.00 ! 2MG, from K2Cn, yxu
CG1N1  CG2R51 CG2R51 NG2R51     2.2000  2   180.00 ! DCG, yxu
CG1N1  CG2R51 CG2R51 HGR52      0.0000  2   180.00 ! DCG, yxu
CG1N1  CG2R51 CG2RC0 CG2R63     1.6000  2   180.00 ! DCG, yxu
CG1N1  CG2R51 CG2RC0 CG2RC0     2.2000  2   180.00 ! DCG, yxu
CG2R51 CG2R51 CG2RC0 CG2R63     3.0000  2   180.00 ! DCG, from CG2R51 CG2R51 CG2RC0
CG2R61, yxu
NG2R62 CG2RC0 NG2R51 CG2R51     2.0000  2   180.00 ! DCG, from NG2R62 CG2RC0 NG2R51
CG2R53, yxu
NG2R61 CG2R63 CG2RC0 CG2R51     2.0000  2   180.00 ! DCG, from NA36, yxu
OG2D4  CG2R63 CG2RC0 CG2R51     0.0000  2   180.00 ! DCG, from NA36, yxu
CG2R51 CG2RC0 CG2RC0 NG2R62     7.0000  2   180.00 ! DCG, from NA36, yxu
HGA3   CG331  NG2R51 CG2R51     0.0000  3     0.00 ! DCG, from HGA3 CG331 NG2R51
CG2R53, yxu
CG2R51 CG2R51 NG2R51 CG331     11.0000  2   180.00 ! DCG, yxu
HGR52  CG2R51 NG2R51 CG331      0.0000  2   180.00 ! DCG, from HGR52 CG2R51 NG2R51
CG321, yxu
NG301  CG2R64 NG2R61 CG2R63     3.1000  2   180.00 ! M2G, from 4MC, yxu
NG301  CG2R64 NG2R62 CG2RC0     3.6000  2   180.00 ! M2G, yxu
NG301  CG2R64 NG2R61 HGP1       0.5000  2   180.00 ! M2G, from 2MC, yxu
NG2P1  CG2N2  CG2R51 CG2R51     1.1500  2   180.00 ! RCG, yxu
NG2P1  CG2N2  CG2R51 CG2R51     0.3100  4     0.00 ! RCG, yxu
NG2P1  CG2N2  CG2R51 CG2RC0     1.1500  2   180.00 ! RCG, yxu
NG2P1  CG2N2  CG2R51 CG2RC0     0.3100  4     0.00 ! RCG, yxu
CG2R51 CG2N2  NG2P1  HGP2       2.0000  2   180.00 ! RCG, from CG2R61 CG2N2 NG2P1
HGP2, yxu
CG2N2  CG2R51 CG2R51 NG2R51     2.4000  2   180.00 ! RCG, yxu
CG2N2  CG2R51 CG2R51 HGR52      1.1000  2   180.00 ! RCG, yxu
CG2N2  CG2R51 CG2RC0 CG2R63     0.0000  2   180.00 ! RCG, from 7mip, yxu
CG2N2  CG2R51 CG2RC0 CG2RC0     2.4000  2   180.00 ! RCG, yxu
CG324  CG2R51 CG2R51 NG2R51     1.8000  2   180.00 ! DAG, yxu
CG324  CG2R51 CG2R51 HGR52      1.0000  2   180.00 ! DAG, yxu
CG324  CG2R51 CG2RC0 CG2R63     2.2000  2   180.00 ! DAG, from ampu, yxu
CG324  CG2R51 CG2RC0 CG2RC0     1.6000  2   180.00 ! DAG, yxu
CG2R51 CG2R51 CG324  HGA2       0.0000  3     0.00 ! DAG, from CG2R51 CG2R51 CG321
HGA2, yxu
CG2RC0 CG2R51 CG324  HGA2       0.2000  3     0.00 ! DAG, from CG2RC0 CG2R51 CG321
HGA2, yxu
CG2R51 CG2R51 CG324  NG3P3      0.6000  2     0.00 ! DAG, yxu
CG2RC0 CG2R51 CG324  NG3P3      0.7000  1   180.00 ! DAG, yxu
CG2RC0 CG2R51 CG324  NG3P3      0.6000  2     0.00 ! DAG, yxu
CG2RC0 CG2R51 CG324  NG3P3      0.1500  3     0.00 ! DAG, yxu
CG2R51 CG324  NG3P3  HGP2       0.0400  3     0.00 ! DAG, from CG2R61 CG324 NG3P1
HGP2, yxu
CG2R51 CG2R51 CG324  NG3P2      0.6000  2     0.00 ! QUG, from DAG, yxu
CG2RC0 CG2R51 CG324  NG3P2      0.7000  1   180.00 ! QUG, from DAG, yxu
```

```
CG2RC0 CG2R51 CG324  NG3P2       0.6000  2     0.00 ! QUG, from DAG, yxu
CG2RC0 CG2R51 CG324  NG3P2       0.1500  3     0.00 ! QUG, from DAG, yxu
CG3C51 CG2R51 CG2R51 CG3C53     12.0000  2   180.00 ! QUG, from CG3C52 CG2R51 CG2R51
CG3C52, yxu
CG3C51 CG2R51 CG2R51 HGR51       2.9000  2   180.00 ! QUG, from CG3C52 CG2R51 CG2R51
HGR51, yxu
CG3C53 CG2R51 CG2R51 HGR51       4.2500  2   180.00 ! QUG, from CG3C54 CG2R51 CG2R51
HGR51, yxu
CG2R51 CG2R51 CG3C51 CG3C51      0.3500  3   180.00 ! QUG, from cpea, yxu
HGR51  CG2R51 CG3C51 CG3C51      0.5000  2     0.00 ! QUG, from cpea, yxu
CG2R51 CG2R51 CG3C51 OG311       2.1000  3   180.00 ! QUG, yxu
HGR51  CG2R51 CG3C51 OG311       0.5000  2   180.00 ! QUG, yxu
CG2R51 CG2R51 CG3C51 HGA1        0.0000  3     0.00 ! QUG, yxu
HGR51  CG2R51 CG3C51 HGA1        0.0000  3     0.00 ! QUG, yxu
CG2R51 CG2R51 CG3C53 CG3C51      0.3500  3   180.00 ! QUG, from cpea, yxu
CG2R51 CG2R51 CG3C53 NG3P2       2.1000  3   180.00 ! QUG, from cpea, yxu
HGR51  CG2R51 CG3C53 CG3C51      0.5000  2     0.00 ! QUG, from cpea, yxu
HGR51  CG2R51 CG3C53 NG3P2       0.0000  2   180.00 ! QUG, from cpea, yxu
CG2R51 CG2R51 CG3C53 HGA1        0.0000  3     0.00 ! QUG, yxu
HGR51  CG2R51 CG3C53 HGA1        0.0000  3     0.00 ! QUG, yxu
CG2R51 CG3C51 CG3C51 CG3C53      0.3400  3   180.00 ! QUG, from cpea, yxu
CG2R51 CG3C51 CG3C51 OG311       0.1400  3     0.00 ! QUG, from cpea, yxu
CG2R51 CG3C51 CG3C51 HGA1        0.1900  3     0.00 ! QUG, from cpea, yxu
CG3C51 CG3C51 CG3C53 CG2R51      0.3400  3   180.00 ! QUG, from cpea, yxu
OG311  CG3C51 CG3C53 CG2R51      0.1400  3     0.00 ! QUG, from cpea, yxu
HGA1   CG3C51 CG3C53 CG2R51      0.1400  3     0.00 ! QUG, from cpea, yxu
CG3C51 CG3C51 CG3C53 NG3P2       0.1400  3     0.00 ! QUG, from cpea, yxu
OG311  CG3C51 CG3C53 NG3P2       0.0000  3     0.00 ! QUG, from NG301 CG3C51 CG3C51
OG311, yxu
HGA1   CG3C51 CG3C53 NG3P2       0.1400  3     0.00 ! QUG, from cpea, yxu
CG2R51 CG3C51 OG311  HGP1        0.1500  1     0.00 ! QUG, yxu
CG2R51 CG3C51 OG311  HGP1        1.1000  2     0.00 ! QUG, yxu
CG2R51 CG3C51 OG311  HGP1        0.6500  3     0.00 ! QUG, yxu
CG2R51 CG324  NG3P2  CG3C53      1.8000  1   180.00 ! QUG, yxu
CG2R51 CG324  NG3P2  CG3C53      0.5000  2   180.00 ! QUG, yxu
CG2R51 CG324  NG3P2  CG3C53      0.1000  3     0.00 ! QUG, yxu
CG2R51 CG324  NG3P2  HGP2        0.1000  3     0.00 ! QUG, yxu
HGA2   CG324  NG3P2  CG3C53      0.1000  3     0.00 ! QUG, yxu
CG2R51 CG3C53 NG3P2  CG324       1.2000  1   180.00 ! QUG, yxu
CG2R51 CG3C53 NG3P2  CG324       0.6500  3     0.00 ! QUG, yxu
CG2R51 CG3C53 NG3P2  HGP2        0.1000  3     0.00 ! QUG, yxu
CG3C51 CG3C53 NG3P2  CG324       0.8000  1   180.00 ! QUG, from pnpa, yxu
CG3C51 CG3C53 NG3P2  CG324       0.0800  3     0.00 ! QUG, from pnpa, yxu
CG3C51 CG3C53 NG3P2  HGP2        0.0800  3     0.00 ! QUG, from CG3C52 CG3C53 NG3P2
HGP2, yxu
HGA1   CG3C53 NG3P2  CG324       0.0500  3     0.00 ! QUG, yxu
CG3C53 CG3C51 CG3C51 CG3RC1      0.4000  6     0.00 ! EQG, from CG3C51 CG3C51 CG3C51
CG3C53, yxu
CG3C51 CG3C51 CG3C53 CG3RC1      0.1500  3     0.00 ! EQG, from CG3C52 CG3C51 CG3C51
CG3RC1, yxu
OG311  CG3C51 CG3C53 CG3RC1      0.6000  1     0.00 ! EQG, from CG3RC1 CG3C51 CG3C51
OG311, yxu
OG311  CG3C51 CG3C53 CG3RC1      0.7000  3     0.00 ! EQG, from CG3RC1 CG3C51 CG3C51
OG311, yxu
HGA1   CG3C51 CG3C53 CG3RC1      0.1500  3     0.00 ! EQG, from CG3RC1 CG3C51 CG3C51
HGA1, yxu
CG3C51 CG3C51 CG3RC1 OG3C31      0.6000  3     0.00 ! EQG, from cpoa, yxu
CG3C51 CG3C51 CG3RC1 OG3C31      0.5000  4     0.00 ! EQG, from cpoa, yxu
HGA1   CG3C51 CG3RC1 OG3C31      0.3600  3     0.00 ! EQG, from cpoa, yxu
OG311  CG3C51 CG3RC1 OG3C31      0.0000  4     0.00 ! EQG, yxu
CG3C51 CG3C53 CG3RC1 OG3C31      0.6000  3     0.00 ! EQG, from cpoa, yxu
CG3C51 CG3C53 CG3RC1 OG3C31      0.5000  4     0.00 ! EQG, from cpoa, yxu
CG3C51 CG3C53 CG3RC1 CG3RC1      0.1500  3     0.00 ! EQG, from cpoa, yxu
```

```
CG3C51 CG3C53 CG3RC1 HGA1       0.1500  3     0.00 ! EQG, from cpoa, yxu
NG3P2  CG3C53 CG3RC1 CG3RC1     0.9000  2     0.00 ! EQG, from cpoa, yxu
NG3P2  CG3C53 CG3RC1 CG3RC1     0.7000  3     0.00 ! EQG, from cpoa, yxu
NG3P2  CG3C53 CG3RC1 OG3C31     0.5000  4   180.00 ! EQG, from cpoa, yxu
NG3P2  CG3C53 CG3RC1 HGA1       0.2500  3     0.00 ! EQG, from cpoa, yxu
HGA1   CG3C53 CG3RC1 CG3RC1     0.1500  3     0.00 ! EQG, yxu
HGA1   CG3C53 CG3RC1 HGA1       0.1500  3     0.00 ! EQG, yxu
HGA1   CG3C53 CG3RC1 OG3C31     0.4600  3     0.00 ! EQG, yxu
CG3C51 CG3RC1 CG3RC1 CG3C53     0.6000  3     0.00 ! EQG, from cpoa, yxu
CG3C51 CG3RC1 CG3RC1 OG3C31     0.5000  4     0.00 ! EQG, from cpoa, yxu
CG3C53 CG3RC1 CG3RC1 OG3C31     0.5000  4     0.00 ! EQG, yxu
CG3C53 CG3RC1 CG3RC1 HGA1       0.3000  3     0.00 ! EQG, yxu
OG3C31 CG3RC1 CG3RC1 HGA1       0.3000  3     0.00 ! EQG, yxu
CG3C51 CG3RC1 OG3C31 CG3RC1     2.4500  3   180.00 ! EQG, from cpoa, yxu
CG3C51 CG3RC1 OG3C31 CG3RC1     1.0000  4     0.00 ! EQG, from cpoa, yxu
CG3C53 CG3RC1 OG3C31 CG3RC1     2.4500  3     0.00 ! EQG, yxu
CG3C53 CG3RC1 OG3C31 CG3RC1     1.0000  4     0.00 ! EQG, yxu
HGA1   CG3RC1 OG3C31 CG3RC1     1.2000  3   180.00 ! EQG, yxu
CG3RC1 CG3C53 NG3P2  CG324      0.4000  1   180.00 ! EQG, from ponm, yxu
CG3RC1 CG3C53 NG3P2  CG324      0.2500  2     0.00 ! EQG, from ponm, yxu
CG3RC1 CG3C53 NG3P2  CG324      0.6400  3     0.00 ! EQG, from ponm, yxu
CG3RC1 CG3C53 NG3P2  HGP2       0.0800  3     0.00 ! EQG, yxu
CG2R51 CG3C51 CG3C51 OG301      0.1400  3     0.00 ! MQG, from QUG, yxu
OG301  CG3C51 CG3C53 CG2R51     0.1400  3     0.00 ! MQG, from QUG, yxu
OG301  CG3C51 CG3C53 NG3P2      0.0000  3     0.00 ! MQG, from QUG, yxu
OG301  CG3C51 CG3C51 OG311      0.0000  3     0.00 ! MQG, from OG303 CG3C51 CG3C51
OG311, yxu
OG301  CG3C51 CG3C51 HGA1       0.1950  3     0.00 ! MQG, from OG303 CG3C51 CG3C51
HGA1, yxu
OG301  CG3C51 CG3C53 HGA1       0.1950  3     0.00 ! MQG, from OG303 CG3C51 CG3C51
HGA1, yxu
NG2R50 CG2R51 CG331  HGA3       0.1900  3     0.00 ! DWG, from NG2R50 CG2R51 CG321
HGA2, yxu
CG2R63 CG2RC0 CG2RC0 NG2R61     2.0000  2   180.00 ! DWG, from CG2R63 CG2RC0 CG2RC0
NG2R62, yxu
NG2R61 CG2RC0 NG2R51 CG2R53     2.0000  2   180.00 ! DWG, from NG2R62 CG2RC0 NG2R51
CG2R53, yxu
NG2R50 CG2RC0 NG2R61 CG2RC0     0.0000  2   180.00 ! DWG, yxu
NG2R51 CG2RC0 NG2R61 CG2RC0     0.0000  2   180.00 ! DWG, yxu
NG2R61 CG2RC0 NG2R50 CG2R51     2.0000  2   180.00 ! DWG, yxu
NG2R61 CG2RC0 NG2R51 HGP1       0.7000  2   180.00 ! DWG, yxu
CG331  CG2R51 NG2R50 CG2RC0     4.1000  2   180.00 ! DWG, yxu
NG2R50 CG2RC0 NG2R61 HGP1       0.8000  2   180.00 ! DWG, yxu
CG2RC0 CG2RC0 NG2R61 HGP1       0.4500  2   180.00 ! DWG, yxu
NG2R51 CG2RC0 NG2R61 HGP1       0.4500  2   180.00 ! DWG, yxu
NG2R50 CG2RC0 CG2RC0 NG2R61     5.3000  2   180.00 ! DWG, yxu
CG2R51 CG2R51 NG2R50 CG2RC0    11.5000  2   180.00 ! DWG, yxu
CG2RC0 CG2RC0 NG2R61 CG2RC0     0.0000  2   180.00 ! DWG, yxu
CG2R51 CG2R51 NG2RC0 CG2R63     3.0000  2   180.00 ! DWG, from CG2R51 CG2R51 NG2RC0
CG2R61, yxu
NG2RC0 CG2R63 CG2RC0 CG2RC0     0.2000  2   180.00 ! DWG, from NG2R61 CG2R63 CG2RC0
CG2RC0, yxu
NG2RC0 CG2R63 CG2RC0 NG2R50     2.0000  2   180.00 ! DWG, from NG2R61 CG2R63 CG2RC0
NG2R50, yxu
CG2RC0 CG2R63 NG2RC0 CG2R51     0.0000  2   180.00 ! DWG, from CG2R61 CG2R61 NG2RC0
CG2R51, yxu
OG2D4  CG2R63 NG2RC0 CG2R51     0.5000  2   180.00 ! DWG, yxu
OG2D4  CG2R63 NG2RC0 CG2RC0     0.5000  2   180.00 ! DWG, yxu
HGR52  CG2R51 NG2RC0 CG2R63     0.3500  2   180.00 ! DWG, yxu
CG331  CG2R51 CG2R51 NG2RC0     4.3000  2   180.00 ! DWG, yxu
NG2RC0 CG2RC0 NG2R61 HGP1       0.6000  2   180.00 ! DWG, yxu
NG2R50 CG2RC0 NG2RC0 CG2R63     3.0000  2   180.00 ! DWG, yxu
NG2R61 CG2RC0 NG2RC0 CG2R51     7.3000  2   180.00 ! DWG, yxu
```

```
NG2R50 CG2RC0 NG2RC0 CG2R51      7.0000  2   180.00 ! DWG, yxu
NG2RC0 CG2RC0 NG2R50 CG2R51      5.0000  2   180.00 ! DWG, yxu
NG2R50 CG2R51 CG2R51 NG2RC0     10.0000  2   180.00 ! DWG, yxu
CG2RC0 CG2R63 NG2RC0 CG2RC0      1.0000  2   180.00 ! DWG, yxu
NG2R61 CG2RC0 NG2RC0 CG2R63      0.6000  2   180.00 ! DWG, yxu
NG2RC0 CG2RC0 NG2R61 CG2RC0      0.0000  2   180.00 ! DWG, yxu
NG2RC0 CG2R51 CG331  HGA3        0.1900  3     0.00 ! MWG, from NG2R51 CG2R51 CG331
HGA3, yxu
NG2RC0 CG2RC0 NG2R61 CG331       0.2000  2   180.00 ! MWG, yxu
CG331  CG2R51 NG2RC0 CG2R63      0.7000  2   180.00 ! MWG, yxu
CG331  CG2R51 NG2RC0 CG2RC0      0.7000  2   180.00 ! MWG, yxu
HGA3   CG331  NG2R61 CG2RC0      0.0000  3     0.00 ! MWG, from HGA3 CG331 NG2R61
CG2R62, yxu
CG2RC0 CG2RC0 NG2R61 CG331       0.3200  2   180.00 ! MWG, yxu
NG2R50 CG2RC0 NG2R61 CG331       0.2000  2   180.00 ! MWG, yxu
NG2R51 CG2RC0 NG2R61 CG331       0.2000  2   180.00 ! MWG, yxu
CG331  CG2R51 CG2R51 CG331       0.5000  2   180.00 ! MWG, yxu
OG2D1  CG2O2  CG311  CG311       0.0500  6   180.00 ! HWG, from OG2D1 CG2O2 CG311
CG321, yxu
OG302  CG2O2  CG311  CG311       0.0500  6   180.00 ! HWG, from OG302 CG2O2 CG311
CG321, yxu
OG302  CG2O6  NG2S1  CG311       4.0000  2   180.00 ! HWG, from OG302 CG2O6 NG2S1
CG321, yxu
OG302  CG2O6  NG2S1  CG311       0.9500  4     0.00 ! HWG, from OG302 CG2O6 NG2S1
CG321, yxu
CG321  CG2R51 CG2R51 CG331       0.5000  2   180.00 ! HWG, from MWG, yxu
CG2O2  CG311  CG311  CG321       0.2000  3     0.00 ! HWG, from CG2O1 CG311 CG311
CG321, yxu
CG2O2  CG311  CG311  OG311       0.2000  3     0.00 ! HWG, from CG2O1 CG311 CG311
OG311, yxu
CG2O2  CG311  CG311  HGA1        0.2000  3     0.00 ! HWG, from CG2O1 CG311 CG311
HGA1, yxu
CG2O2  CG311  NG2S1  CG2O6       0.2000  1   180.00 ! HWG, from CG2O2 CG311 NG2S1
CG2O1, yxu
CG311  CG311  CG321  CG2R51      0.3500  3     0.00 ! HWG, from hpiz, yxu
OG311  CG311  CG321  CG2R51      1.8000  1   180.00 ! HWG, yxu
OG311  CG311  CG321  CG2R51      0.3500  3     0.00 ! HWG, yxu
CG321  CG2R51 NG2RC0 CG2R63      0.7000  2   180.00 ! HWG, from MWG, yxu
CG321  CG2R51 NG2RC0 CG2RC0      0.7000  2   180.00 ! HWG, from MWG, yxu
NG2RC0 CG2R51 CG321  CG311       0.3200  3     0.00 ! HWG, yxu
NG2RC0 CG2R51 CG321  HGA2        0.3200  3     0.00 ! HWG, yxu
NG2RC0 CG2R51 CG321  CG321       0.3200  3     0.00 ! WBG, from HWG, yxu
CG2R51 CG2R51 CG321  CG321       0.2000  1     0.00 ! WBG, from CG2R51 CG2R51 CG321
CG311, yxu
CG321  CG311  NG2S1  CG2O6       1.8000  1     0.00 ! WBG, from CG321 CG311 NG2S1
CG2O1, yxu
CG2R51 CG321  CG321  HGA2        0.2000  3     0.00 ! WBG, from HGA1 CG311 CG321
CG2R51, yxu
CG2R51 CG321  CG321  CG311       0.0400  3     0.00 ! WBG, from CG2R61 CG321 CG321
CG321, yxu
CG2O2  CG311  CG311  OG301       2.0000  1   180.00 ! PBG, from amba, yxu
CG2O2  CG311  CG311  OG301       0.8000  2     0.00 ! PBG, from amba, yxu
NG2S1  CG311  CG311  OG301       1.8000  1   180.00 ! PBG, yxu
OG301  CG311  CG311  HGA1        0.1950  3     0.00 ! PBG, yxu
OG301  CG311  CG321  CG2R51      0.8000  1   180.00 ! PBG, yxu
OG301  CG311  CG321  CG2R51      0.2000  3   180.00 ! PBG, yxu
OG301  CG311  CG321  HGA2        0.1950  3     0.00 ! PBG, from OG302 CG311 CG321
HGA2, yxu
CG321  CG311  OG301  OG311       1.1000  1   180.00 ! PBG, yxu
CG321  CG311  OG301  OG311       0.5000  3     0.00 ! PBG, yxu
CG311  CG311  OG301  OG311       1.1000  1   180.00 ! PBG, from ppox, yxu
CG311  CG311  OG301  OG311       0.5000  3     0.00 ! PBG, from ppox, yxu
HGA1   CG311  OG301  OG311       0.2000  3     0.00 ! PBG, yxu
```

```
CG311  OG301  OG311  HGP1       0.4000  1     0.00 ! PBG, yxu
CG311  OG301  OG311  HGP1       1.1500  2     0.00 ! PBG, yxu
CG311  OG301  OG311  HGP1       0.1200  3     0.00 ! PBG, yxu
OG311  CG311  CG314  CG2O3      0.2000  3     0.00 ! BUG, from CG2O3 CG311 CG311
OG311, yxu
OG311  CG311  CG314  NG3P3      0.2000  3     0.00 ! BUG, from NG3P3 CG314 CG321
OG311, yxu
OG311  CG311  CG314  HGA1       0.1950  3     0.00 ! BUG, from OG311 CG311 CG311
HGA1, yxu
CG314  CG311  CG321  CG2R51     0.3500  3     0.00 ! BUG, from HWG, yxu
CG314  CG311  OG311  HGP1       0.5000  1     0.00 ! BUG, from CG324 CG311 OG311
HGP1,, yxu
CG314  CG311  OG311  HGP1       0.7000  2     0.00 ! BUG, from CG324 CG311 OG311
HGP1,, yxu
OG311  CG2O2  CG311  CG311      0.0500  6   180.00 ! BUGn, from OG311 CG2O2 CG311
CG321, yxu
CG321  CG311  CG311  NG321      0.4000  1     0.00 ! BUGn, from aboh, yxu
CG321  CG311  CG311  NG321      0.8000  3     0.00 ! BUGn, from aboh, yxu
NG321  CG311  CG311  OG311      0.4000  1   180.00 ! BUGn, from aboh, yxu
NG321  CG311  CG311  OG311      0.8000  3     0.00 ! BUGn, from aboh, yxu
NG321  CG311  CG311  HGA1       0.5000  3     0.00 ! BUGn, from aboh, yxu
CG311  CG311  NG321  HGPAM2     0.5000  1   180.00 ! BUGn, yxu
CG311  CG311  NG321  HGPAM2     0.4000  3     0.00 ! BUGn, yxu
CG321  CG2R51 CG2RC0 CG2R63     2.5000  2   180.00 ! 7GNA, from CG321 CG2R51 CG2RC0
CG2R61, yxu
CG2R51 CG2R51 CG321  NG321      0.4100  2   180.00 ! 7GNA (idam), yxu
CG2RC0 CG2R51 CG321  NG321      0.5400  1     0.00 ! 7GNA (idam), yxu
CG2RC0 CG2R51 CG321  NG321      0.4600  3     0.00 ! 7GNA (idam), yxu
CG2R51 CG321  NG321  HGPAM2     0.5800  1     0.00 ! 7GNA (idam), yxu
CG2R51 CG321  NG321  HGPAM2     0.7100  3     0.00 ! 7GNA (idam), yxu
CG3C51 CG2R51 CG2R51 CG3C51    12.0000  2   180.00 ! 7GNM, from CG3C52 CG2R51 CG2R51
CG3C52, yxu
CG3C51 CG3C51 CG3C51 NG311      0.0000  3     0.00 ! 7GNM, from CG3C51 CG3C51 CG3C51
NG301, yxu
NG311  CG3C51 CG3C51 OG311      0.0000  3     0.00 ! 7GNM, from NG301 CG3C51 CG3C51
OG311, yxu
NG311  CG3C51 CG3C51 HGA1       0.1950  3     0.00 ! 7GNM, from NG301 CG3C51 CG3C51
HGA1, yxu
CG3C51 CG3RC1 CG3RC1 CG3C51     4.0000  3     0.00 ! 7GNM, from CG3C51 CG3RC1 CG3RC1
CG3C52, yxu
CG3C51 CG3C51 CG3C51 CG3RC1     0.1500  3     0.00 ! 7GNM, from CG3C52 CG3C51 CG3C51
CG3RC1, yxu
NG311  CG3C51 CG3C51 OG301      0.0000  3     0.00 ! 7GNM, from NG301 CG3C51 CG3C51
OG311, yxu
CG2R51 CG3C51 CG3C51 CG3C51     0.3400  3   180.00 ! 7GNM, from cpea, yxu
CG2R51 CG2R51 CG321  NG311      0.4100  2   180.00 ! 7GNM, from idam, yxu
CG2RC0 CG2R51 CG321  NG311      0.5400  1     0.00 ! 7GNM, from idam, yxu
CG2RC0 CG2R51 CG321  NG311      0.4600  3     0.00 ! 7GNM, from idam, yxu
CG2R51 CG321  NG311  HGPAM1     1.0000  2   180.00 ! 7GNM, yxu
CG2R51 CG321  NG311  HGPAM1     0.8000  3     0.00 ! 7GNM, yxu
CG2R51 CG321  NG311  CG3C51     3.0000  1   180.00 ! 7GNM, yxu
HGA2   CG321  NG311  CG3C51     0.8000  3     0.00 ! 7GNM, yxu
CG3C51 CG3C51 NG311  CG321      2.0000  1   180.00 ! 7GNM, from cena, yxu
CG3C51 CG3C51 NG311  CG321      0.7000  3     0.00 ! 7GNM, from cena, yxu
CG3C51 CG3C51 NG311  HGPAM1     0.2000  2     0.00 ! 7GNM, from cena, yxu
CG2R51 CG3C51 NG311  CG321      2.0000  1   180.00 ! 7GNM, yxu
CG2R51 CG3C51 NG311  CG321      0.7000  3     0.00 ! 7GNM, yxu
CG2R51 CG3C51 NG311  HGPAM1     0.2000  2     0.00 ! 7GNM, yxu
HGA1   CG3C51 NG311  CG321      0.1950  3     0.00 ! 7GNM, yxu
HGA1   CG3C51 NG311  HGPAM1     0.0100  3     0.00 ! 7GNM, yxu
CG2R51 CG2R51 CG3C51 NG311      1.7000  2     0.00 ! 7GNM, yxu
CG2R51 CG2R51 CG3C51 NG311      3.1000  3     0.00 ! 7GNM, yxu
HGR51  CG2R51 CG3C51 NG311      1.7000  2     0.00 ! 7GNM, yxu
```

```
HGR51  CG2R51 CG3C51 NG311      3.1000  3     0.00 ! 7GNM, yxu
CG3RC1 CG3C51 NG311  CG321      0.0000  3     0.00 ! 7GNM, from cona, yxu
CG3RC1 CG3C51 NG311  HGPAM1     0.2000  2     0.00 ! 7GNM, yxu
NG311  CG3C51 CG3RC1 CG3RC1     0.8000  4   180.00 ! 7GNM, yxu
NG311  CG3C51 CG3RC1 CG3RC1     0.5000  6     0.00 ! 7GNM, yxu
NG311  CG3C51 CG3RC1 HGA1       0.1950  3     0.00 ! 7GNM, yxu
NG311  CG3C51 CG3RC1 OG3C31     0.8000  4   180.00 ! 7GNM, yxu
NG311  CG3C51 CG3RC1 OG3C31     0.5000  6     0.00 ! 7GNM, yxu

NG2R62 CG2R61 CG2RC0 NG2R52     0.0000  2   180.00 ! 7MGe, from NG2R62 CG2R61 CG2RC0
NG2R50, not optimized, yxu
OG311  CG2R61 CG2RC0 CG2RC0     3.1000  2   180.00 ! 7MGe, from CG2R61 CG2R61 CG2R61
OG311, not optimized, yxu
OG311  CG2R61 CG2RC0 NG2R52     2.4000  2   180.00 ! 7MGe, from NG2S1 CG2R61 CG2R61
OG311, not optimized, yxu
OG311  CG2R61 NG2R62 CG2R64     3.1000  2   180.00 ! 7MGe, from CG331 CG2R61 NG2R60
CG2R64, not optimized, yxu
CG2RC0 CG2R61 OG311  HGP1       0.9900  2   180.00 ! 7MGe, from CG2R61 CG2R61 OG311
HGP1, not optimized, yxu
NG2R62 CG2R61 OG311  HGP1       0.9900  2   180.00 ! 7MGe, from CG2R61 CG2R61 OG311
HGP1, not optimized, yxu
NG2S3  CG2R64 NG2R62 CG2R61     2.0000  2   180.00 ! 7MGe, from NG2S3 CG2R64 NG2R62
CG2R63, not optimized, yxu
CG2R61 CG2RC0 CG2RC0 NG2R52     1.5000  2   180.00 ! 7MGe, from CG2R61 CG2RC0 CG2RC0
NG2R50, not optimized, yxu
CG2R61 CG2RC0 NG2R52 CG2R53    12.0000  2   180.00 ! 7MGe, from CG2R51 CG2R51 NG2R52
CG2R53, not optimized, yxu
CG2R61 CG2RC0 NG2R52 CG334      6.0000  2   180.00 ! 7MGe, from CG2R51 CG2R52 NG2R52
CG3C54, not optimized, yxu
OG311  CG2R61 CG2RC0 NG2R50     1.0000  2   180.00 ! GENO, from NG2R60 CG2R61 CG2R61
OG311, not optimized, yxu

!***Model compounds***
SG2D1  CG2R63 NG2R61 CG331      7.0000  2   180.00 ! 2SU, yxu
SEGD1  CG2R63 NG2R61 CG331      7.0000  2   180.00 ! SEU, yxu
CG2R61 CG2R61 CG324  NG3P2      0.2000  2     0.00 ! bzma, yxu 4/16,14
CG2R61 CG324  NG3P2  CG334      0.6500  1   180.00 ! bzma, yxu
CG2R61 CG324  NG3P2  CG334      0.7800  3     0.00 ! bzma, yxu
CG2R61 CG324  NG3P2  HGP2       0.1000  3     0.00 ! bzma, yxu
CG2O3  CG324  NG3P2  CG334      1.0000  1   180.00 ! nmgi, yxu
CG2O3  CG324  NG3P2  CG334      0.7000  2     0.00 ! nmgi, yxu
CG2D1  CG324  NG3P2  CG334      0.7000  1   180.00 ! nmba, yxu
CG2D1  CG324  NG3P2  CG334      0.8000  3     0.00 ! nmba, yxu
OG2D1  CG2O2  CG321  CG2R61     0.8500  1     0.00 ! mebz, yxu
OG302  CG2O2  CG321  CG2R61     0.3000  1     0.00 ! mebz, yxu
OG302  CG2O2  CG321  CG2R61     1.0000  2   180.00 ! mebz, yxu
CG2R61 CG2R61 CG321  CG2O2      0.1000  2     0.00 ! mebz, yxu
CG2R61 CG2R61 CG321  CG2O1      0.1000  2     0.00 ! acbz, from mebz, yxu
NG2S2  CG2O1  CG321  CG2R61     0.2100  1   180.00 ! acbz, yxu
NG2S2  CG2O1  CG321  CG2R61     0.5500  3     0.00 ! acbz, yxu
OG2D1  CG2O1  CG321  CG2R61     0.6700  2   180.00 ! acbz, yxu
OG2D1  CG2O1  CG321  CG2R61     0.9400  3     0.00 ! acbz, yxu
CG2R61 CG2R61 CG311  CG2O2      0.2000  2     0.00 ! cmbz, yxu,  corrected 1/2015
CG2R61 CG2R61 CG311  OG311      0.7000  2   180.00 ! bzha & cmbz, yxu,  corrected
1/2015
OG2D1  CG2O2  CG311  CG2R61     0.7000  1     0.00 ! cmbz, yxu
OG2D1  CG2O2  CG311  CG2R61     0.3000  3   180.00 ! cmbz, yxu
OG302  CG2O2  CG311  CG2R61     0.3000  3     0.00 ! cmbz, yxu
CG2R61 CG2R61 CG321  CG2O3      0.1000  4     0.00 ! bzac, yxu
OG2D2  CG2O3  CG321  CG2R61     0.6000  2   180.00 ! bzac, yxu
OG2D2  CG2O3  CG321  CG2R61     0.1000  4     0.00 ! bzac, yxu
CG2R62 CG2R62 CG311  CG331      0.4000  2     0.00 ! qhe, yxu, 7/5,13
CG2R61 CG311  OG311  HGP1       1.0800  1     0.00 ! bzhe, yxu
```

```
CG2R61 CG311  OG311  HGP1       0.5400  2     0.00 ! bzhe, yxu
CG2R61 CG311  OG311  HGP1       0.7700  3     0.00 ! bzhe, yxu
CG2O3  CG321  OG301  CG2R61     1.3100  1   180.00 ! atbz, yxu
CG2O2  CG321  OG301  CG2R61     0.9100  1   180.00 ! oebz, yxu
CG2O2  CG321  OG301  CG2R61     0.5000  2     0.00 ! oebz, yxu
CG331  CG321  CG321  NG2R61     0.5000  1   180.00 ! 3pru, yxu
CG2O2  CG321  NG311  CG331      2.0000  1     0.00 ! nmgn, yxu
CG2O2  CG321  NG311  CG331      1.8000  2     0.00 ! nmgn, yxu
CG2O2  CG321  NG311  CG331      0.5000  3     0.00 ! nmgn, yxu
HGA2   CG321  NG311  CG331      0.4000  3     0.00 ! nmgn, yxu
CG2R61 CG2R61 CG321  NG311      1.0000  2   180.00 ! ambz, yxu
CG2R61 CG321  NG311  CG331      0.8000  1   180.00 ! ambz, yxu
CG2R61 CG321  NG311  CG331      0.5000  2     0.00 ! ambz, yxu
CG2R61 CG321  NG311  CG331      0.7000  3     0.00 ! ambz, yxu
CG2R61 CG321  NG311  HGPAM1     0.3000  3     0.00 ! ambz, yxu
HGR62  CG2R61 NG2P1  CG334      2.7000  2   180.00 ! ncyp, yxu
CG2R61 CG2R61 NG2P1  CG334      0.4000  2   180.00 ! ncyp, yxu
HGR62  CG2R61 NG2P1  HGP2       0.0000  2   180.00 ! ncyp, yxu
CG2R61 CG2R61 NG2P1  HGP2       0.0000  2   180.00 ! ncyp, yxu
CG331  CG321  CG324  NG2P1      0.1950  3     0.00 ! pncp, yxu
CG331  CG321  CG321  NG311      0.3000  3     0.00 ! prnc, yxu
CG2R61 CG2R64 NG311  CG321      1.1000  1     0.00 ! bepa, yxu
CG2R61 CG2R64 NG311  CG321      2.0000  2   180.00 ! bepa, yxu
CG2R61 CG2R64 NG311  HGPAM1     1.6000  2   180.00 ! bepa, yxu
NG2R60 CG2R64 NG311  CG321      1.1000  1   180.00 ! bepa, yxu
NG2R60 CG2R64 NG311  CG321      2.0000  2   180.00 ! bepa, yxu
NG2R60 CG2R64 NG311  HGPAM1     1.6000  2   180.00 ! bepa, yxu
CG2R61 CG2R64 NG2S1  CG2O6      1.5000  1     0.00 ! pymu, yxu
CG2R61 CG2R64 NG2S1  CG2O6      1.9000  2   180.00 ! pymu, yxu
CG2R61 CG2R64 NG2S1  CG2O6      0.7200  3     0.00 ! pymu, yxu
CG2R61 CG2R61 NG2S1  CG2O6      1.2000  2   180.00 ! mpyu, yxu
NG2S1  CG2O6  NG2S1  CG2R61     1.6000  2   180.00 ! mpyu, yxu
NG2S1  CG2O6  NG2S1  CG2R61     0.2800  4     0.00 ! mpyu, yxu
OG2D1  CG2O6  NG2S1  CG2R61     2.0000  2   180.00 ! mpyu, yxu
NG2S0  CG2O6  NG2S1  CG331      1.2000  1   180.00 ! mmmu, yxu
NG2S0  CG2O6  NG2S1  CG331      1.5000  2   180.00 ! mmmu, yxu
NG2S1  CG2O6  NG2S0  CG331      2.0000  2   180.00 ! mmmu, yxu
OG2D1  CG2O6  NG2S0  CG331      2.3000  2   180.00 ! mmmu, yxu
CG2RC0 CG2R64 NG2S0  CG2O1      1.0000  1     0.00 ! m6pa, yxu
CG2RC0 CG2R64 NG2S0  CG2O1      2.2000  2   180.00 ! m6pa, yxu
CG2RC0 CG2R64 NG2S0  CG2O1      0.2000  3     0.00 ! m6pa, yxu
NG2R62 CG2R64 NG2S0  CG2O1      2.6000  2   180.00 ! m6pa, yxu
NG2S1  CG2O6  NG2S0  CG2R61     1.3000  2   180.00 ! pmmu, yxu
OG2D1  CG2O6  NG2S0  CG2R61     1.3000  2   180.00 ! pmmu, yxu
NG2R62 CG2RC0 NG2R52 CG334      3.0000  2   180.00 ! 7MG, yxu
CG2RC0 CG2RC0 NG2R52 HGP2       1.6000  2   180.00 ! 7MG, yxu
NG2R62 CG2RC0 NG2R52 HGP2       1.6000  2   180.00 ! 7MG, yxu
CG2N2  CG2R51 CG2RC0 CG2R61     0.0000  2   180.00 ! 7mip, yxu
CG324  CG2R51 CG2RC0 CG2R61     2.2000  2   180.00 ! ampu, yxu
HGR62  CG2R61 CG2RC0 CG2R51     4.0000  2   180.00 ! ampu, from HGR61 CG2R61 CG2RC0
CG2R51, yxu
CG321  CG321  NG2S0  CG331      0.1000  3   180.00 ! H2U_m, yxu, 8/8,13
HGA2   CG321  NG2S0  CG331      0.3200  3     0.00 ! H2U_m, yxu, 8/8,13
CG2R51 CG2R51 CG3C51 CG3C52     0.3500  3   180.00 ! cpea, yxu
HGR51  CG2R51 CG3C51 CG3C52     0.5000  2     0.00 ! cpea, yxu
CG2R51 CG2R51 CG3C53 CG3C52     0.3500  3   180.00 ! cpea, yxu
CG2R51 CG2R51 CG3C53 NG3P3      2.1000  3   180.00 ! cpea, yxu
HGR51  CG2R51 CG3C53 CG3C52     0.5000  2     0.00 ! cpea, yxu
HGR51  CG2R51 CG3C53 NG3P3      0.0000  2   180.00 ! cpea, yxu
CG2R51 CG3C51 CG3C52 HGA2       0.1400  3     0.00 ! cpea, from CG2R51 CG3C52 CG3C52
HGA2, yxu
CG2R51 CG3C51 CG3C52 CG3C53     0.3400  3   180.00 ! cpea, yxu
OG311  CG3C51 CG3C52 CG3C53     0.1400  3     0.00 ! cpea, yxu
```

```
HGA1   CG3C51 CG3C52 CG3C53     0.1900  3     0.00 ! cpea, from CG3C53 CG3C51 CG3C51
HGA1, yxu
CG3C51 CG3C52 CG3C53 CG2R51     0.3400  3   180.00 ! cpea, yxu
CG3C51 CG3C52 CG3C53 NG3P3      0.1400  3     0.00 ! cpea, yxu
CG3C51 CG3C52 CG3C53 HGA1       0.1400  3     0.00 ! cpea, from CG3C52 CG3C52 CG3C53
HGA1, yxu
HGA2   CG3C52 CG3C53 CG2R51     0.1400  3     0.00 ! cpea, from from CG2R51 CG3C52
CG3C52 HGA2, yxu
HGA2   CG3C52 CG3C53 NG3P3      0.1400  3     0.00 ! cpea, from from HGA2 CG3C52
CG3C53 NG3P2, yxu
CG3C52 CG3C53 NG3P2  CG324      0.8000  1   180.00 ! pnpa, yxu
CG3C52 CG3C53 NG3P2  CG324      0.0800  3     0.00 ! pnpa, yxu
CG324  CG2R51 CG2R51 HGR51      1.0000  2   180.00 ! pnpa, from DAG, yxu
CG2R51 CG2R51 CG2R51 CG324      4.3000  2   180.00 ! pnpa, from DWG, yxu
CG3C52 CG3C53 CG3RC1 OG3C31     0.6000  3     0.00 ! cpoa, yxu
CG3C52 CG3C53 CG3RC1 OG3C31     0.5000  4     0.00 ! cpoa, yxu
CG3C52 CG3C53 CG3RC1 CG3RC1     0.1500  3     0.00 ! cpoa, yxu
CG3C52 CG3C53 CG3RC1 HGA1       0.1500  3     0.00 ! cpoa, yxu
NG3P3  CG3C53 CG3RC1 CG3RC1     0.9000  2     0.00 ! cpoa, yxu
NG3P3  CG3C53 CG3RC1 CG3RC1     0.7000  3     0.00 ! cpoa, yxu
NG3P3  CG3C53 CG3RC1 OG3C31     0.5000  4   180.00 ! cpoa, yxu
NG3P3  CG3C53 CG3RC1 HGA1       0.2500  3     0.00 ! cpoa, yxu
CG3C52 CG3C52 CG3RC1 OG3C31     0.6000  3     0.00 ! cpoa, yxu
CG3C52 CG3C52 CG3RC1 OG3C31     0.5000  4     0.00 ! cpoa, yxu
HGA2   CG3C52 CG3RC1 OG3C31     0.3600  3     0.00 ! cpoa, yxu
CG3C52 CG3RC1 CG3RC1 CG3C53     0.6000  3     0.00 ! cpoa, yxu
CG3C52 CG3RC1 CG3RC1 OG3C31     0.5000  4     0.00 ! cpoa, yxu
CG3C52 CG3RC1 OG3C31 CG3RC1     2.4500  3   180.00 ! cpoa, yxu
CG3C52 CG3RC1 OG3C31 CG3RC1     1.0000  4     0.00 ! cpoa, yxu
CG3C53 CG3C52 CG3C52 CG3RC1     0.4000  6     0.00 ! cpoa, from CG3C51 CG3C51 CG3C51
CG3C53, yxu
CG3C52 CG3C52 CG3C53 CG3RC1     0.1500  3     0.00 ! cpoa, from CG3RC1 CG3C51 CG3C52
CG3C52, yxu
CG3C52 CG3C52 CG3C53 NG3P3      0.1400  3     0.00 ! cpoa, from CG3C52 CG3C52 CG3C53
NG3P2, yxu
HGA2   CG3C52 CG3C53 CG3RC1     0.1500  3     0.00 ! cpoa, from CG3RC1 CG3C51 CG3C52
HGA2, yxu
CG3C52 CG3C53 NG3P3  HGP2       0.0800  3     0.00 ! cpoa, from CG3C52 CG3C53 NG3P2
HGP2, yxu
CG3RC1 CG3C53 NG3P3  HGP2       0.0800  3     0.00 ! cpoa, from CG3C52 CG3C53 NG3P2
HGP2, yxu
HGA1   CG3C53 NG3P3  HGP2       0.0800  3     0.00 ! cpoa, from HGA1 CG3C53 NG3P2
HGP2, yxu
CG3C52 CG2R51 CG2R51 CG3C53    12.0000  2   180.00 ! cpea, from CG3C52 CG2R51 CG2R51
CG3C52, yxu
CG2R51 CG3C52 CG3C52 CG3C53     0.3400  3   180.00 ! cpea, from CG2R53 CG3C52 CG3C52
CG3C52, yxu
CG3C52 CG3C52 CG3C53 CG2R51     0.3400  3   180.00 ! cpea, from CG2R53 CG3C52 CG3C52
CG3C52, yxu
CG2R51 CG3C53 NG3P3  HGP2       0.3000  3     0.00 ! cpea, from CG2R51 CG3C54 NG3P2
HGP2, yxu
CG3RC1 CG3C53 NG3P2  CG334      0.4000  1   180.00 ! ponm, yxu
CG3RC1 CG3C53 NG3P2  CG334      0.2500  2     0.00 ! ponm, yxu
CG3RC1 CG3C53 NG3P2  CG334      0.6400  3     0.00 ! ponm, yxu
HGA1   CG3C53 NG3P2  CG334      0.0500  3     0.00 ! ponm, yxu
CG3C52 CG3C53 NG3P2  CG334      1.2000  1   180.00 ! ponm, from pnpa, yxu
CG3C52 CG3C53 NG3P2  CG334      0.2500  3     0.00 ! ponm, from pnpa, yxu
CG3C52 CG3C51 OG301  CG311      0.2000  1   180.00 ! pepr, yxu
CG3C52 CG3C51 OG301  CG311      0.3000  3     0.00 ! pepr, yxu
HGA1   CG3C51 OG301  CG311      0.5000  1     0.00 ! pepr, yxu
HGA1   CG3C51 OG301  CG311      0.6000  3   180.00 ! pepr, yxu
CG321  CG311  OG301  CG3C51     0.4500  3     0.00 ! pepr, yxu
OG3C61 CG311  OG301  CG3C51     0.8200  1     0.00 ! pepr, yxu
```

```
OG3C61 CG311  OG301  CG3C51     0.8100  2     0.00 ! pepr, yxu
OG3C61 CG311  OG301  CG3C51     0.3500  3     0.00 ! pepr, yxu
HGA1   CG311  OG301  CG3C51     0.3500  3     0.00 ! pepr, yxu
OG301  CG311  OG3C61 CG321      0.2000  3     0.00 ! pepr, from Carb36
OG301  CG3C51 CG3C52 CG2R51     0.1400  3     0.00 ! pepr, from QUG, yxu
CG2R51 CG2R51 CG2R51 CG321      4.3000  2   180.00 ! hpiz, from DWG, yxu
CG331  CG311  CG321  CG2R51     0.3500  3     0.00 ! hpiz, yxu
CG321  CG2R51 NG2RC0 CG2R61     0.7000  2   180.00 ! hpiz, from MWG, yxu
CG331  CG311  OG301  OG311      1.1000  1   180.00 ! ppox, yxu
CG331  CG311  OG301  OG311      0.5000  3     0.00 ! ppox, yxu
CG2O4  CG311  CG311  OG301      2.0000  1   180.00 ! amba, yxu
CG2O4  CG311  CG311  OG301      0.8000  2     0.00 ! amba, yxu
CG331  CG311  NG321  HGPAM2     0.5000  1     0.00 ! aboh, cgenff_xyu, yxu
CG331  CG311  NG321  HGPAM2     0.4000  3     0.00 ! aboh, cgenff_xyu, yxu
CG331  CG311  CG311  NG321      0.4000  1     0.00 ! aboh, yxu
CG331  CG311  CG311  NG321      0.8000  3     0.00 ! aboh, yxu
CG3C52 CG3C51 OG301  CG3C51     0.3000  1   180.00 ! rbrb, yxu
CG3C52 CG3C51 OG301  CG3C51     0.8000  2   180.00 ! rbrb, yxu
HGA1   CG3C51 OG301  CG3C51     0.3000  1     0.00 ! rbrb, yxu
HGA1   CG3C51 OG301  CG3C51     0.8000  2   180.00 ! rbrb, yxu
OG3C51 CG3C51 OG301  CG3C51     0.6000  2     0.00 ! rbrb, yxu
OG3C51 CG3C51 OG301  CG3C51     0.2000  3     0.00 ! rbrb, yxu
OG301  CG3C51 OG3C51 CG3C52     3.2000  2     0.00 ! rbrb, yxu
OG301  CG3C51 OG3C51 CG3C52     1.9000  3     0.00 ! rbrb, yxu
OG301  CG3C51 OG3C51 CG3C52     1.0000  4   180.00 ! rbrb, yxu
OG301  CG3C51 OG3C51 CG3C51     3.2000  2     0.00 ! rbrb, yxu
OG301  CG3C51 OG3C51 CG3C51     1.9000  3     0.00 ! rbrb, yxu
OG301  CG3C51 OG3C51 CG3C51     1.0000  4   180.00 ! rbrb, yxu
HGA3   CG331  OG301  OG311      0.2000  3     0.00 ! mhp from ppox, yxu
CG331  OG301  OG311  HGP1       0.4000  1     0.00 ! mhp from ppox, yxu
CG331  OG301  OG311  HGP1       1.1500  2     0.00 ! mhp from ppox, yxu
CG331  OG301  OG311  HGP1       0.1200  3     0.00 ! mhp from ppox, yxu
CG321  CG324  NG3P2  CG334      0.4000  1     0.00 ! maes, from CG321 CG324 NG3P2
CG324, yxu
CG321  CG324  NG3P2  CG334      0.2500  2     0.00 ! maes, from CG321 CG324 NG3P2
CG324, yxu
CG321  CG324  NG3P2  CG334      0.6000  3     0.00 ! maes, from CG321 CG324 NG3P2
CG324, yxu
OG311  CG2O2  CG321  CG2R61     0.9300  2   180.00 ! bzaa, yxu
OG311  CG2O2  CG321  CG2R61     0.1200  3   180.00 ! bzaa, yxu
CG2D2  CG2D1  CG321  NG311      1.6000  1   180.00 ! penm, from bepa, yxu
CG2D2  CG2D1  CG321  NG311      0.3000  3     0.00 ! penm, from bepa, yxu
CG2D1  CG321  NG311  CG331      1.1000  1   180.00 ! penm, yxu
CG2D1  CG321  NG311  CG331      0.6000  2   180.00 ! penm, yxu
CG2D1  CG321  NG311  CG331      0.5000  3     0.00 ! penm, yxu
CG2O2  CG321  OG301  CG331      0.1000  1   180.00 ! moac, yxu
CG2O2  CG321  OG301  CG331      0.8000  2     0.00 ! moac, yxu
CG2O2  CG321  OG301  CG331      0.5900  3     0.00 ! moac, yxu
CG321  CG2R51 CG2R51 HGR51      1.0000  2   180.00 ! cena, from CG321 CG2R51 CG2R51
HGR52, yxu
NG311  CG3C51 CG3C52 HGA2       0.1500  3   180.00 ! cena, from NG321 CG3C51 CG3C52
HGA2, yxu
NG311  CG3C51 CG3C52 CG3C52     0.3000  3   180.00 ! cena, from NG321 CG3C51 CG3C52
CG3C51, yxu
CG2R51 CG3C52 CG3C52 CG3C51     0.3400  3   180.00 ! cena, from CG2R53 CG3C52 CG3C52
CG3C52, yxu
CG3C51 CG2R51 CG2R51 CG3C52    12.0000  2   180.00 ! cena, from CG3C52 CG2R51 CG2R51
CG3C52, yxu
CG2R51 CG3C51 CG3C52 CG3C52     0.3400  3   180.00 ! cena, from CG2R53 CG3C52 CG3C52
CG3C52, yxu
CG3C52 CG3C51 NG311  CG321      2.0000  1   180.00 ! cena, yxu
CG3C52 CG3C51 NG311  CG321      0.7000  3     0.00 ! cena, yxu
CG3C52 CG3C51 NG311  HGPAM1     0.2000  2     0.00 ! cena, yxu
```

```
HGA3   CG331  NG311  CG3C51     0.0000  3   180.00 ! cona, from HGA2 CG321 NG311
CG2R61, yxu
CG3C52 CG3C51 CG3RC1 OG3C31     0.6000  3     0.00 ! cona, from cpoa, yxu
CG3C52 CG3C51 CG3RC1 OG3C31     0.5000  4     0.00 ! cona, from cpoa, yxu
HGA1   CG3C51 NG311  CG331      0.1950  3     0.00 ! cona, yxu
CG3C52 CG3C51 NG311  CG331      2.0000  1   180.00 ! cona, yxu
CG3C52 CG3C51 NG311  CG331      0.7000  3     0.00 ! cona, yxu
CG3RC1 CG3C51 NG311  CG331      0.5000  1   180.00 ! cona, yxu
CG3RC1 CG3C51 NG311  CG331      0.7000  3     0.00 ! cona, yxu
CG2R61 CG2R61 CG2R61 NG2S0      3.1000  2   180.00 ! dmpu, from CG2R61 CG2R61 CG2R61
NG2S1, yxu
NG2S0  CG2R61 CG2R61 HGR61      2.4000  2   180.00 ! DMPU, from NG2S1 CG2R61 CG2R61
HGR61, yxu
CG2R61 CG2R61 NG2S0  CG2O6      2.2000  2   180.00 ! dmpu, from m6pa, yxu
CG2R61 CG2R61 NG2S0  CG331      2.2000  2   180.00 ! dmpu, from m6pa, yxu
HGA3   CG331  NG2S0  CG2R61     0.0000  3     0.00 ! dmpu, from HGA3 CG331 NG2S0
CG2O1, yxu
CG2R61 CG2R61 NG2R61 CG331     11.0000  2   180.00 ! cyt, yxu
HGA3   CG331  NG2R61 CG2R61     0.0000  3     0.00 ! 34c, yxu
HGR62  CG2R61 NG2R61 CG331      0.3000  2   180.00 ! 34c, yxu
NG311  CG2R64 NG2R61 CG331      3.5000  2   180.00 ! 34c, yxu
NG2P1  CG2R64 NG2R62 CG2R64     3.0000  2   180.00 ! 34c, from K2Cn
CG2R61 CG2R61 CG321  CG1N1      0.3900  2   180.00 ! cybz, yxu
CG2R61 CG2R61 CG321  CG1N1      0.0400  4     0.00 ! cybz, yxu
CG2D2  CG2D1  CG321  SG311      0.7000  1   180.00 ! pesu
CG2D2  CG2D1  CG321  SG311      1.4000  3   180.00 ! pesu
SG311  CG2R64 NG2R61 CG331      6.0000  2   180.00 ! msu
NG321  CG311  CG331  HGA3       0.1600  3     0.00 ! aboh, from NG311 CG321 CG331
HGA3, cgenff_xyu, yxu
NG2R62 CG2R62 CG2R62 OG301      1.6000  2   180.00 ! enoU, from NG2R61 CG2R62 CG2R62
OG301, yxu
CG2R62 CG2R62 CG2R62 OG311      3.0000  2   180.00 ! enoU, from NG2R61 CG2R62 CG2R62
OG311, yxu
OG311  CG2R62 NG2R62 CG2R63     3.0000  2   180.00 ! enoU, from NG2R61 CG2R62 CG2R62
OG311, yxu
OG301  CG2R62 CG2R62 OG311      0.0000  2   180.00 ! enoU, from OG2D4  CG2R63 CG2R62
OG301, yxu
NG2R62 CG2R62 OG311  HGP1       2.5000  1     0.00 ! enoU, yxu  might be lower in
water
NG2R62 CG2R62 OG311  HGP1       2.7000  2   180.00 ! enoU, yxu
NG2R62 CG2R62 OG311  HGP1       0.8500  3     0.00 ! enoU, yxu
NG2P1  CG2R63 NG2R61 CG331     11.0000  2   180.00 ! 3MC, not optimized, yxu
HGA3   CG334  NG2P1  CG2R61     0.0000  3     0.00 ! ncyp, not optimized, yxu
HGA3   CG334  NG3P2  CG334      0.1000  3     0.00 ! dma, not optimized, yxu
NG2R61 CG2RC0 NG2R51 CG331     11.0000  2   180.00 ! DWG, not optimized, yxu
HGR51  CG2R51 CG2RC0 CG2R63     2.8000  2   180.00 ! dng, not optimized, yxu
NG2R62 CG2R61 CG2RC0 CG2R51     2.2000  2   180.00 ! ampu, not optimized, yxu
CG331  CG2O1  NG2S0  CG2R64     1.6000  1     0.00 ! m6pa, not optimized, yxu
CG331  CG2O1  NG2S0  CG2R64     2.5000  2   180.00 ! m6pa, not optimized, yxu
OG2D1  CG2O1  NG2S0  CG2R64     2.5000  2   180.00 ! m6pa, not optimized, yxu
CG2O4  CG2R62 CG2R62 NG2R62     2.5000  2   180.00 ! 5fop, not optimized, yxu
CG2O4  CG311  CG311  CG331      0.2000  3     0.00 ! amba, from CG2O1 CG311 CG311
CG331, not optimized, yxu
CG2O4  CG311  CG311  HGA1       0.2000  3     0.00 ! amba, from CG2O1 CG311 CG311
HGA1, not optimized, yxu
OG2D1  CG2O4  CG311  CG311      1.0500  1   180.00 ! amba, from OG2D1 CG2O4 CG321
CG331, not optimized, yxu
OG2D1  CG2O4  CG311  CG311      0.4000  2   180.00 ! amba, from OG2D1 CG2O4 CG321
CG331, not optimized, yxu
OG2D1  CG2O4  CG311  CG311      0.6000  3   180.00 ! amba, from OG2D1 CG2O4 CG321
CG331, not optimized, yxu
OG2D1  CG2O4  CG311  CG311      0.1000  4   180.00 ! amba, from OG2D1 CG2O4 CG321
CG331, not optimized, yxu
```

```
OG2D1  CG2O4  CG311  NG2S1      0.0000  1     0.00 ! amba, from OG2D1 CG2O2 CG311
NG2S1, not optimized, yxu
OG2D1  CG2O4  CG311  HGA1       0.0000  3   180.00 ! amba, from OG2D1 CG2O4 CG321
HGA2, not optimized, yxu
HGR52  CG2O4  CG311  CG311      0.0000  3   180.00 ! amba, from HGR52 CG2O4 CG321
CG331, not optimized, yxu
CG311  CG311  OG301  CG331      0.4000  1     0.00 ! amba, from CG331 CG311 OG301
CG331, not optimized, yxu
CG311  CG311  OG301  CG331      0.4900  3     0.00 ! amba, from CG331 CG311 OG301
CG331, not optimized, yxu
HGR52  CG2O1  NG2S1  CG311      2.6000  2   180.00 ! amba, from HGR52 CG2O1 NG2S0
CG331, not optimized, yxu
HGR52  CG2O1  NG2S1  HGP1       1.4000  2   180.00 ! amba, from HGR52 CG2O1 NG2S2
HGP1, not optimized, yxu
HGR52  CG2O4  CG311  NG2S1      0.0000  3   180.00 ! amba, from HGR52 CG2O4 CG321
CG331, not optimized, yxu
CG2O4  CG311  NG2S1  CG2O1      0.2000  1   180.00 ! amba, from CG2O2 CG311 NG2S1
CG2O1, not optimized, yxu
CG2O4  CG311  NG2S1  HGP1       0.0000  1     0.00 ! amba, from CG2O2 CG311 NG2S1
HGP1, not optimized, yxu
HGR52  CG2O4  CG311  HGA1       0.0000  3   180.00 ! amba, from HGR52 CG2O4 CG321
HGA2, not optimized, yxu
CG331  CG311  CG311  CG331      0.5000  4   180.00 ! aboh, from CG321 CG311 CG311
CG321, not optimized, yxu
CG331  CG311  CG311  OG311      0.1400  3     0.00 ! aboh, from CG311 CG311 CG311
OG311, not optimized, yxu
CG2R61 CG324  NG3P3  HGP2       0.0400  3     0.00 ! 4FBA, bzam; from CG2R61 CG324
NG3P1 HGP2; pram & yxu
CG2R61 CG2R61 CG2R64 NG311      3.1000  2   180.00 ! bepa, from CG2R61 CG2R61 CG2R61
NG311, not optimized, yxu
NG311  CG2R64 NG2R60 CG2R61     3.1000  2   180.00 ! bepa, from CG321 CG2R61 NG2R60
CG2R61, not optimized, yxu
HGR61  CG2R61 CG2R64 NG311      2.4000  2   180.00 ! bepa, from NG311 CG2R61 CG2R61
HGR61, not optimized, yxu
CG2R62 CG2R62 NG2R62 CG2R63     2.0000  2   180.00 ! 5fop, from CG2R61 CG2R61 NG2R62
CG2R64, not optimized, yxu
HGR62  CG2R62 NG2R62 CG2R63     7.3000  2   180.00 ! 5fop, from HGR62 CG2R61 NG2R62
CG2R64, not optimized, yxu
NG2R61 CG2R63 NG2R62 CG2R62     0.6000  2   180.00 ! 5fop, from NG2R61 CG2R63 NG2R62
CG2R64, not optimized, yxu
OG2D4  CG2R63 NG2R62 CG2R62     1.6000  2   180.00 ! 5fop, from OG2D4 CG2R63 NG2R62
CG2R64, not optimized, yxu
CG2R51 CG2R51 CG3C52 CG3C51     0.0500  3   180.00 ! pepr, from CG2R51 CG2R51 CG3C52
CG3C52, not optimized, yxu
HGR51  CG2R51 CG3C52 CG3C51     2.0000  2   180.00 ! pepr, from HGR51 CG2R51 CG3C52
CG3C52, not optimized, yxu
OG301  CG311  CG321  CG321      0.1600  1   180.00 ! pepr, from CG321 CG321 CG321
OG301, not optimized, yxu
OG301  CG311  CG321  CG321      0.3900  2     0.00 ! pepr, from CG321 CG321 CG321
OG301, not optimized, yxu
OG3C61 CG311  CG321  CG321      0.1900  1   180.00 ! pepr, from CG321 CG321 CG321
OG3C61, not optimized, yxu
OG3C61 CG311  CG321  CG321      1.0000  2   180.00 ! pepr, from CG321 CG321 CG321
OG3C61, not optimized, yxu
OG3C61 CG311  CG321  CG321      0.6000  3     0.00 ! pepr, from CG321 CG321 CG321
OG3C61, not optimized, yxu
OG3C61 CG311  CG321  CG321      0.0800  4   180.00 ! pepr, from CG321 CG321 CG321
OG3C61, not optimized, yxu
OG3C61 CG311  CG321  HGA2       0.1950  3     0.00 ! pepr, from OG3C61 CG321 CG321
HGA2, not optimized, yxu
CG321  CG311  OG3C61 CG321      0.5300  1   180.00 ! pepr, from CG321 CG321 OG3C61
CG321, not optimized, yxu
```

```
CG321  CG311  OG3C61 CG321      0.6800  2     0.00 ! pepr, from CG321 CG321 OG3C61
CG321, not optimized, yxu
CG321  CG311  OG3C61 CG321      0.2100  3   180.00 ! pepr, from CG321 CG321 OG3C61
CG321, not optimized, yxu
CG321  CG311  OG3C61 CG321      0.1500  4     0.00 ! pepr, from CG321 CG321 OG3C61
CG321, not optimized, yxu
HGA1   CG311  OG3C61 CG321      0.1950  3     0.00 ! pepr, from HGA2 CG321 OG3C61
CG321, not optimized, yxu
CG321  CG321  OG3C61 CG311      0.5300  1   180.00 ! pepr, from CG321 CG321 OG3C61
CG321, not optimized, yxu
CG321  CG321  OG3C61 CG311      0.6800  2     0.00 ! pepr, from CG321 CG321 OG3C61
CG321, not optimized, yxu
CG321  CG321  OG3C61 CG311      0.2100  3   180.00 ! pepr, from CG321 CG321 OG3C61
CG321, not optimized, yxu
CG321  CG321  OG3C61 CG311      0.1500  4     0.00 ! pepr, from CG321 CG321 OG3C61
CG321, not optimized, yxu
HGA2   CG321  OG3C61 CG311      0.1950  3     0.00 ! pepr, from HGA2 CG321 OG3C61
CG321, not optimized, yxu
CG3C52 CG3C51 CG3C52 CG2R51     0.3400  3   180.00 ! pepr, from CG2R53 CG3C52 CG3C52
CG3C52, not optimized, yxu
HGA1   CG3C51 CG3C52 CG2R51     0.1400  3     0.00 ! pepr, from CG2R51 CG3C52 CG3C52
HGA2, not optimized, yxu
OG2D1  CG2O2  CG311  CG331      0.0500  6   180.00 ! hpme, from OG2D1 CG2O2 CG311
CG321, not optimized, yxu
OG302  CG2O2  CG311  CG331      0.0500  6   180.00 ! hpme, from OG302 CG2O2 CG311
CG321, not optimized, yxu
CG2O2  CG311  CG331  HGA3       0.2000  3     0.00 ! hpme, from CG2O1 CG311 CG331
HGA3, not optimized, yxu
CG2O3  CG321  OG301  CG331      0.2000  3     0.00 ! moat, from CG2O3 CG311 OG301
CG331, not optimized, yxu
HGA3   CG334  NG3P2  CG3C53     0.1000  3     0.00 ! ponm, from HGA2 CG324 NG3P2
CG314, not optimized, yxu
CG2D2  CG2D1  CG321  CG321      0.5000  1   180.00 ! 15he, from CG2D2 CG2D1 CG321
CG331, not optimized, yxu
CG2D2  CG2D1  CG321  CG321      1.3000  3   180.00 ! 15he, from CG2D2 CG2D1 CG321
CG331, not optimized, yxu

!***Misc parameters that might lead to changes in the existing CGenFF***
NG2R61 CG2R64 NG301  CG331      2.8000  1     0.000 ! M2G, yxu.
!Can only occur at both sides in cases such as the minor tautomer of protonated
isocytosine, which yields
!a warning in all current versions of cgenffprog (<=1.1). However, may cause trouble
if transfered to
!other nitrogen types ==> FIXME! -- kevo
!
NG2R61 CG2R64 NG301  CG331      1.0000  2   180.00 ! M2G, yxu
NG2R61 CG2R64 NG301  CG331      0.5500  4     0.00 ! M2G, yxu
NG2R61 CG2R64 SG311  CG321      1.4500  1     0.00 ! BGAU BGCU GAU GCU; from NG2R61
CG2R64 SG311 CG331, 4O2SM; yxu & kevo
NG2R62 CG2R64 SG311  CG321      2.1200  2   180.00 ! BGAU BGCU GAU GCU; from NG2R62
CG2R64 SG311 CG331, 2SMPYR; yxu & kevo
NG2R62 CG2R64 SG311  CG321      0.1900  4   180.00 ! BGAU BGCU GAU GCU; from NG2R62
CG2R64 SG311 CG331, 2SMPYR; yxu & kevo

IMPROPER
!!!!!!!! ######################################## !!!!!!!!
!!!!!!!! ######## CGenFF model compounds ######## !!!!!!!!
!!!!!!!! ######################################## !!!!!!!!
!!***Uracils***
CG2R63 NG2R61 NG2R61 SEGD1     58.0000  0     0.00 ! SEU, yxu, tmp
CG2O6  NG2S0  NG2S1  OG2D1     86.5000  0     0.00 ! H2U, aa
CG2O3  OG2D2  OG2D2  CG324     96.0000  0     0.00 ! 5DU, yxu
CG2R62 CG2R62 NG2R62 OG311      0.0000  0     0.00 ! enoU, yxu
```

```
!!***Cytosines***
CG2R63 NG2P1  NG2R61 OG2D4     78.0000  0     0.00 ! CYTp, yxu, TO BE OPTIMIZED
CG2R63 NG2R61 NG2P1  OG2D4     78.0000  0     0.00 ! 3MC, yxu, slack TO BE OPTIMIZED
CG2R64 NG2P1  NG2P1  CG2R62    52.0000  0     0.00 ! CYTp, yxu, TO BE OPTIMIZED
CG2R64 NG2P1  CG2R62 NG2P1     52.0000  0     0.00 ! CYTp, yxu, SLACK to be removed
CG2R64 CG2R62 NG2P1  NG2P1     52.0000  0     0.00 ! 3MC, yxu, slack TO BE OPTIMIZED
CG2R64 HGP2   HGP2   NG2P1     -1.0000  0     0.00 ! 3MC, yxu, slack TO BE OPTIMIZED
CG2R64 CG2R62 NG2D1  NG2R61    42.0000  0     0.00 ! 3MCn, slack TO BE OPTIMIZED
CG2R64 CG2R61 NG2D1  NG2P1     28.0000  0     0.00 ! K2C, yxu, TO BE OPTIMIZED
CG2R64 NG2P1  NG2P1  NG2P1     47.0000  0     0.00 ! K2C, yxu, TO BE OPTIMIZED
CG2R64 CG2R61 NG2D1  NG2R62    42.0000  0     0.00 ! K2Cn, from 3MCn, yxu
CG2R64 CG2R61 NG2P1  NG2R62    42.0000  0     0.00 ! 34HC, from 3MCn, yxu, TO BE
OPTIMIZED
CG2R64 CG2R61 NG2R62 NG2S3     60.0000  0     0.00 ! 2SC, from CG2R64 CG2R62 NG2R62
NG2S3, from NA36
CG2R63 NG2R61 NG2R62 SG2D1     58.0000  0     0.00 ! 2SC, from 2SU, yxu
CG2O4  CG2R62 OG2D1  HGR52     53.0000  0     0.00 ! 5FC, yxu
CG2R64 CG2R62 NG2R62 NG311     48.0000  0     0.00 ! 4MC, yxu
CG2R64 CG2R62 NG2R62 NG2S1     19.0000  0     0.00 ! 4AC, from CG2R64 CG2R61 NG2R60
NG2S1, yxu
CG2R63 CG2R62 NG2R62 OG2D4     60.0000  0     0.00 ! 1PC, yxu

!!***Adenine***
CG2R64 CG2RC0 NG2P1  NG2P1     52.0000  0     0.00 ! ADEp, from 3MC, yxu, TO BE
OPTIMIZED
CG2R64 CG2RC0 NG2R62 NG311     50.0000  0     0.00 ! 6MA, yxu manually added
CG2R64 CG2RC0 NG2R62 NG2S1     40.0000  0     0.00 ! 26A, yxu manually added
CG2R64 CG2RC0 NG2D1  NG2R61    42.0000  0     0.00 ! 1MAn, from 3MCn, yxu, slack TO
BE OPTIMIZED

!!***Guanine***
CG2R64 NG2R61 NG2R62 NG311     40.0000  0     0.00 ! 2MG, from K2Cn, yxu
CG2R64 NG2R61 NG2R62 NG301     40.0000  0     0.00 ! M2G, from TMC, yxu
CG2N2  NG2P1  NG2P1  CG2R51    26.0000  0     0.00 ! RCG, yxu
CG2R63 CG2RC0 NG2RC0 OG2D4     52.0000  0     0.00 ! DWG, from CG2R63 CG2RC0 NG2R61
OG2D4, yxu
CG2R64 NG2R62 NG2R62 NG2S3     40.0000  0     0.00 ! 7MGe , from CG2R64 NG2R61 NG2R62
NG2S3, not optimized, yxu

!!***Model compounds***
CG2O6  NG2S1  NG2S1  OG2D1     80.0000  0     0.00 ! H2U, yxu
CG2R64 CG2RC0 NG2R62 NG2S0     22.0000  0     0.00 ! m6pa, yxu  ##check!!
CG2O1  NG2S1  OG2D1  HGR52     66.0000  0     0.00 ! amba, from CG2O1 NG2S2 OG2D1
HGR52, yxu
CG2O4  CG311  OG2D1  HGR52     50.0000  0     0.0 ! amba, from CG2O4 CG321 OG2D1
HGR52, yxu

NONBONDED  NBXMOD 5  ATOM CDIEL FSHIFT VATOM VDISTANCE VFSWITCH -
     CUTNB 14.0  CTOFNB 12.0  CTONNB 10.0  EPS 1.0  E14FAC 1.0  WMIN 1.5
SEGD1    0.0       -0.6230     2.1750 ! Se, starting from SG2D1 -0.5650  2.0500, TO
BE OPTIMIZED, yxu

END
RETURN
```
